# Pedigree-based genome re-sequencing reveals genetic variation patterns of elite backbone varieties during modern rice improvement

**DOI:** 10.1038/s41598-017-00415-1

**Published:** 2017-03-22

**Authors:** Xingfei Zheng, Lanzhi Li, Fan Liang, Changjun Tan, Shuzhu Tang, Sibin Yu, Ying Diao, Shuangcheng Li, Zhongli Hu

**Affiliations:** 1 State Key Laboratory of Hybrid Rice, Lotus Engineering Research Center of Hubei Province, College of Life Sciences, Wuhan University, Wuhan, 430072 P.R. China; 2 Hunan Provincial Key Laboratory for Biology and Control of Plant Disease and Insect Pests, College of Plant Protection, Hunan Agricultural University, Changsha, 410128 P.R. China; 3 Nextomics Biosciences, Wuhan, 430075 P.R. China; 4 Agricultural College of Yangzhou University, Yangzhou, 225009 P.R. China; 5 College of Plant Science and Technology, Huazhong Agricultural University, Wuhan, 430070 China; 6 Rice Institute, Sichuan Agricultural University, Chengdou, 611130 China

## Abstract

Rice breeding has achieved great productivity improvements by semi-dwarf varieties and hybrid vigour. Due to poor understanding of genetic basis of elite backbone varieties, the continuous increasing in rice yield still faces great challenges. Here, 52 elite rice varieties from three historical representative pedigrees were re-sequenced with 10.1× depth on average, and ~6.5 million single nucleotide polymorphisms (SNPs) were obtained. We identified thousands of low-diversity genomic regions and 0-diversity genes during breeding. Using pedigree information, we also traced SNP transmission patterns and observed breeding signatures in pedigree. These regions included the larger number of key well-known functional genes. Besides, 35 regions spanning 0.16% of the rice gnome had been differentially selected between conventional and restorer pedigrees. These genes identified here will be useful to the further pedigree breeding. Our study provides insights into the genetic basis of backbone varieties and will have immediate implications for performing genome-wide breeding by design.

## Introduction

Rice is an important food source for humankind, and breeders are always trying to increase its productivity to meet society’s growing food needs. Roughly 10,000 years ago, humans began to domesticate rice from wild populations^1^. During this process, valuable traits for domestication, such as rice shattering, and awn and tiller angles, have undergone significant changes^2–4^. In the past seven decades, rice crop experienced intensive breeding selection. Subsequently, grain yield has been greatly improved due to the exploitation of semi-dwarfing^5, 6^ and heterosis^7, 8^.

Genomics research in recent years has identified a large number of loci that were under selection and improvement during rice breeding^9, 10^. However, few studies have assessed genome-wide variation patterns in the breeding of backbone parents. Next-generation sequencing technologies now allow genome sequencing at relatively low costs, providing opportunities to inspect transmission patterns in pedigrees. High-yield was considered a fundamental trait for almost all elite rice varieties, and other agronomic traits (for example, disease resistance and grain quality) were subsequently acquired. Correspondingly, DNA recombination and directional selection by breeders caused some ancestral genomic regions to be substituted by excellent genes of donor parents. Importantly, domestication-related genes/loci, such as *sh4*, *prog1* and *phr1*, should be located on low- or even zero-diversity chromosome regions. Obviously, these chromosome regions provide valuable information for further crop improvement.

Thus, we examined genome-wide variations in three historical and representative pedigrees of backbone parents, covering the semi-dwarfing, heterosis and Green Super Rice (GSR) periods of rice modern breeding. The large number of selection genes identified here reveals the genetic basis and evolutionary laws of backbone varieties and provides new insight for elite varieties breeding in pedigrees. In addition, some well-known genes controlling disease resistance, grain quality and plant architecture were also observed. These results may have significant implications for further pedigree breeding, genetic improvement and will also be useful for the cloning of important genes in rice.

## Results

### Sequencing and mapping

We selected the Guichao2hao pedigree (GC2H, 11 varieties), Huanghuazhan pedigree (HHZ pedigree, 22 varieties) and Shuhui527 pedigree (SH527, 23 varieties) (Supplementary Fig. S1), which covered three key breeding stages of rice (Supplementary Fig. S2) to perform genome re-sequencing. The GC2H (1946~1976) and HHZ pedigrees (1946~2002), which belonged to inbred varieties from conventional rice, are outstanding representatives of semi-dwarfism and GSR breeding, respectively; The SH527 pedigree (1976~2011) belongs to restorers of hybrid cultivars, which represents hybrid breeding (Supplementary Fig. S2).

A total of 2.14 billion paired-end reads (~243 Gb) was generated on the Illumina HiSeq 2500 platform, with an average read depth of 10.1× (Supplementary Table S1). The sequencing quality of raw reads was generally high (Q30 ≥ 83.9%). Using Burrows-Wheeler Aligner (BWA) tools^11^, all raw reads were mapped against the reference genome^12^. The mapping rates ranged from 93.38% to 95.68%, and the final unique depth was >12× for most varieties (Supplementary Table S1). The consistent mapping rates indicate similar levels of genetic variation between sequenced varieties and the reference genome. However, unmapped reads may be caused by library contamination, sequencing errors or subspecies-specific sequences. We also mapped reads back to the 9311 reference genome (http://rice.genomics.org.cn), and the final mapping rates ranged from 94.74% to 95.07%. We further assembled unmapped reads for each pedigree into contigs, and further uni-contigs were obtained (Supplementary Table S2, Supplementary Data S1). A total of 37 genes from ≥4 kb uni-contigs were predicted, of which 32 (86.5%) had homologues in the *japonica* genome (Supplementary Table S3). This result may indicate that either the genomic regions/genes from *japonica* were introgressed into the *indica* genome during breeding, or the 93–11 reference genome is incomplete^13^. Protein function annotations were completed by searching against the NR and SWISS-PROT databases (Supplementary Table S3). BLAST results were essentially in agreement, and 33 and 17 genes were successfully annotated in the NR and SWISS-PROT databases, respectively. Of 17 annotated genes in the SWISS-PROT database, ten (30.3%) were observed to code for disease resistance proteins, including five NB-ARC-containing and two NB-LRR-containing proteins. Most of the predicted genes (63.6%) were common among three pedigrees, and 16 (48.5%) were observed in all three pedigrees (Supplementary Table S3). These novel genes, especially plant resistance genes, tend to be common across the three pedigrees, indicating that these genes may play an important role in breeding. Their functional roles will require further investigation at the transcript and protein levels.

### Variation detection and large-effect SNPs

Using stringent quality control criteria, 6,464,341 SNPs were obtained in all 52 varieties. Of these SNPs, 5,610,003 (86.78%) were located in inter-genic regions, and only 3.86% (249,646) were located in the coding sequencing regions (Table 1). Compared with SNPs identified from the 3000 rice genome project^14^, high concordance rates ranging from 95.19% to 99.31% were observed, except for S30 (85.40%) and S50 (82.20%) (Supplementary Table S4). To further evaluate the accuracy of SNPs, we randomly selected three genomic regions containing 66 SNP loci to perform PCR amplification for Sanger sequencing (Supplementary Tables S5 and S6). We found that 373 (98.68%) of 378 SNP genotypes were correct (Supplementary Table S7). These results confirmed high quality SNP calling in this study.

**Table 1:** The distribution of SNPs located in different genomic regions for three rice pedigrees.

Pedigree	Varieties number	Inter-genic	Intron	5′UTR	CDS	3′UTR	Non coding exon	Total	dn/ds
**GC2H**	11	4,406,715	304,538	67,004	201,979	109,810	9,414	5,099,460	1.29
**HHZ**	22	4,738,401	319,460	70,466	210,384	114,793	10,289	5,463,793	1.30
**SH527**	23	4,417,701	301,779	68,383	208,342	109,909	9,478	5,115,592	1.30
**Total**	52	5,610,003	373,999	83,554	249,646	134,991	12,148	6,464,341	1.30

Note: GC2H means GC2H pedigree; HHZ means HHZ pedigree; SH527 means SH527 pedigree.

**Figure 1:**
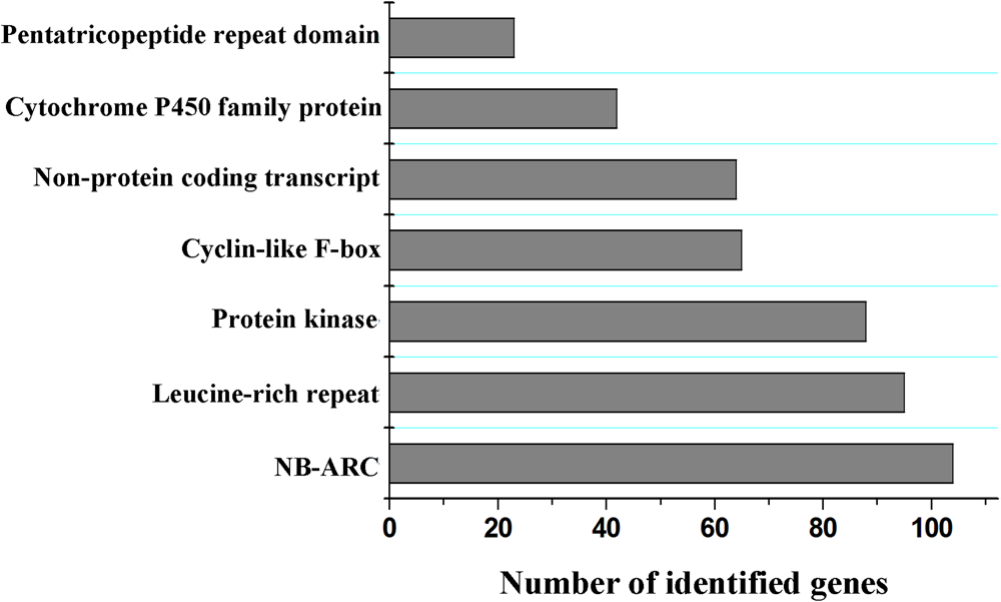
Functional annotations of large-effect SNPs. The numbers (shown by bar lengths) of genes from the most protein families in large-effect SNPs are displayed.

We further calculated that the ratio of non-synonymous to synonymous substitutions (dn/ds) was 1.30, which is basically consistent with the findings of previous work^3, 15^. and is higher than that of *A. thaliana* ^16^, but lower than that of soybean^17^. Interestingly, this ratio is also lower than that from the 3000 rice genome protect (1.45)^18^, which may be due to higher genetic variations. We also identified large-effect SNPs that were located at potentially disabling positions of gene, leading to severe coding sequence variations. Finally, 5,851 large-effect SNPs were identified in 3,650 genes, of which 3,653 (62.4%, 2,427 genes) stop-gained SNPs that caused shortened transcripts, and 808 (13.8%, 689 genes) stop-lost SNPs that caused elongated transcripts (Supplementary Table S8). After functional annotation, we found that the largest proportion of plant disease-related proteins, such as those containing NB-ARCs and Leucine-rich repeat domains, was obtained from large-effect SNPs annotation (Fig. 1). Furthermore, 4,464 large-effect SNPs (76.4%) were common between two pedigrees, and 3,430 (58.6%) were common in all three pedigrees. These large-effect variants are likely to play an important role in breeding, thus representing potential gene resources for rice improvement. In addition to SNPs, we also identified 1,299,868 INDELs (shorter than 50 bp) in the GATK pipeline. Only 24,656 (0.019%) INDELs were located in coding regions, of which 18,944 (76.8%) caused frame shifts (Supplementary Table S9).

### Polymorphisms analysis among three pedigrees

In total, 5,099,460 (78.9%), 5,463,793 (84.5%) and 5,115,592 (79.1%) SNPs were obtained for GC2H, HHZ and SH527 pedigrees, respectively (Table 1). The presence of more unique SNPs (542,175) in SH527 pedigree and the large number of common SNPs (4,625,136) between two conventional pedigrees (GC2H and HHZ) reflect genetic distances (Supplementary Fig. S3). The unique SNPs for the SH527 pedigree may explain why restorers possess such a high level of combining ability.

We further identified pedigree-specific SNP genotypes. For GC2H and SH527 pedigrees, 16 and 14,691 SNP genotypes were obtained, of which two (one gene) and 1,340 unique SNPs (631 genes) were located in coding regions, respectively. For these SNP genotypes, eleven large-effect SNPs were observed and their gene functions were further annotated. Finally, four were found to be plant disease-related genes. Eight SNPs (72.7%) were located on chromosome 6 (Supplementary Table S10). Subsequently, we considered regional distribution of all large-effect SNPs for each pedigree. The number of large-effect SNPs from chromosome 6 in SH527 pedigree (366, 7.73%) is lower than that in GC2H (386, 8.49%) and HHZ (393, 8.07%) pedigrees. Meanwhile, of 1,340 unique SNPs in SH527 pedigree, 692 (51.6%) were found to be located on chromosome 6. These results imply that chromosome 6 may have undergone stronger differential selection than other chromosomes during restorer breeding process.

For each pedigree, we also calculated π values, which can be used to evaluate the level of genetic diversity in a population^19^. π values were 0.0027, 0.0025 and 0.0019 for GC2H, HHZ and SH527 pedigrees, respectively. Obviously, the π value for SH527 pedigree was lower than those for conventional pedigrees, although more restorer lines were sampled in this study, indicating that all restorer varieties of SH527 pedigree have a high level of genetic similarity. Furthermore, more unique SNPs in SH527 pedigree have been identified in this study. These results revealed that the SH527 pedigree has undergone a stronger bottleneck than conventional rice pedigrees, and genetic differentiation may have been occurred during breeding, as combining ability needs to be first considered for restorer breeding. This result demonstrated that the division of rice heterosis groups maybe possible, as it is for maize and wheat^20, 21^.

### Population structure

**Figure 2:**
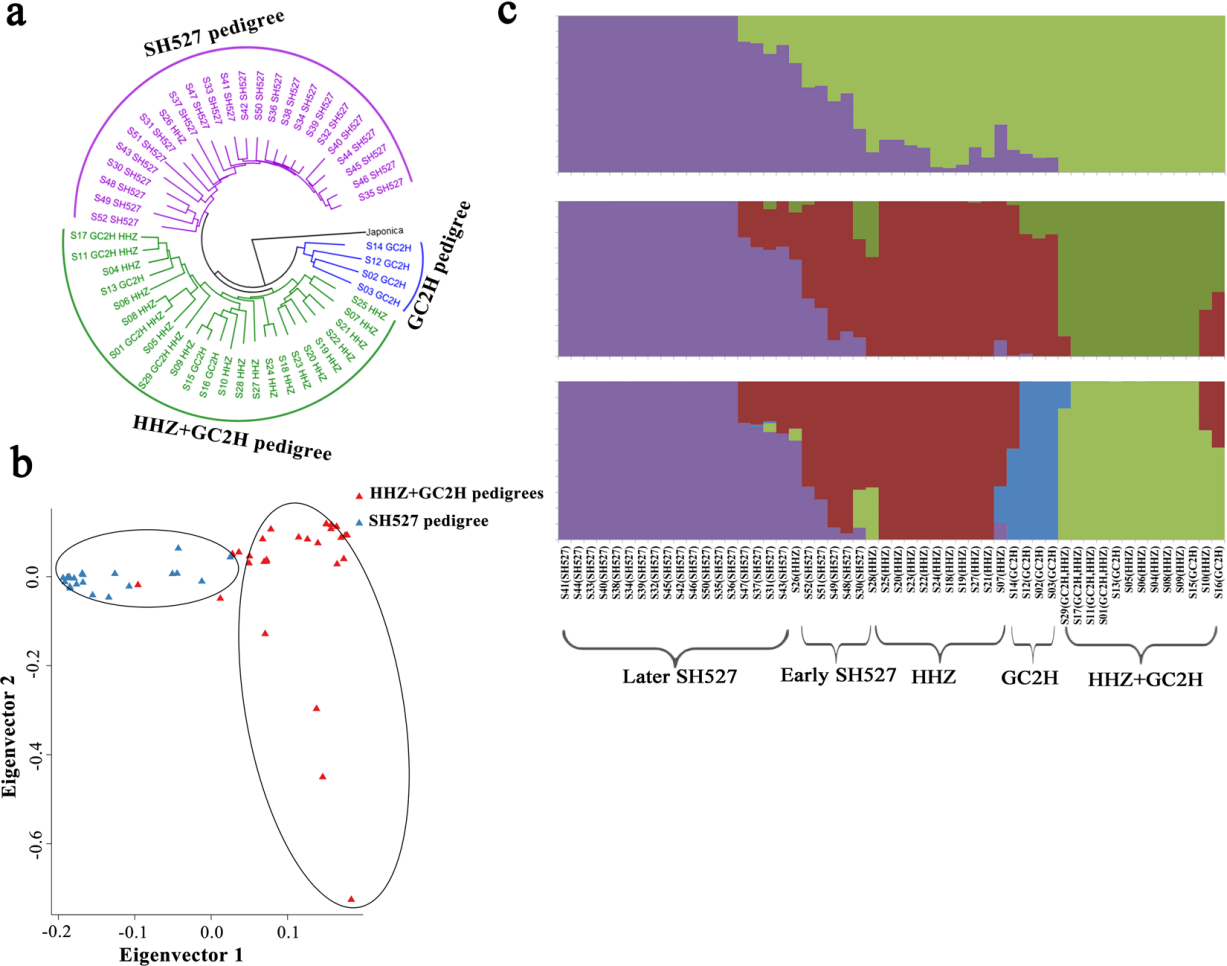
Population structure of pedigree varieties. (**a**) Neighbour-joining phylogenetic trees of pedigree varieties. Japonica was used as a reference genome for phylogenetic analysis. (**b**) Two-D PCA plot of pedigree varieties. (**c**) Population structure analysis of pedigree varieties. Each color represents one population. Each variety is represented by a vertical bar, and the length of each colored segment represents the proportion contributed by ancestral populations.

The neighbour-joining tree constructed by MEGA tools^22^ contained three major groups, corresponding to GC2H+HHZ pedigrees, SH527 pedigree and 4 varieties from GC2H pedigree. S26, which belonged to HHZ pedigree, was grouped into the SH525 pedigree group (Fig. 2a). In the principle component analysis (PCA) (Fig. 2b), all samples were divided into two groups using 2-d eigenvectors, corresponding to GC2H+HHZ pedigrees and SH527 pedigree. The conventional rice pedigrees show much more dispersion than the restorer pedigree. Furthermore, we used PLINK tools to investigate the population structure with a maximum likelihood method^23^. The pedigree populations were analysed by increasing *K* (the number of assuming populations) from 2 to 4 (Fig. 2c). For *K* = 2, a rough division between conventional rice and restorer pedigrees was obtained. For *K* = 4, early restorer varieties from SH527 pedigree were separated and grouped with the HHZ pedigree. This grouping may have occurred because early restorers were directly selected from conventional rice, only based on their level of combining ability. However, later restorers were further created by hybrid breeding methods, while early restorer lines were used as breeding parents, simultaneously considering their yield and combining ability performances. For conventional rice breeding, yield traits were only considered (Supplementary Fig. S4). Thus, different breeding objectives may lead to genetic differentiation between restorer and conventional rice.

### Linkage disequilibrium (LD) analysis

**Figure 3:**
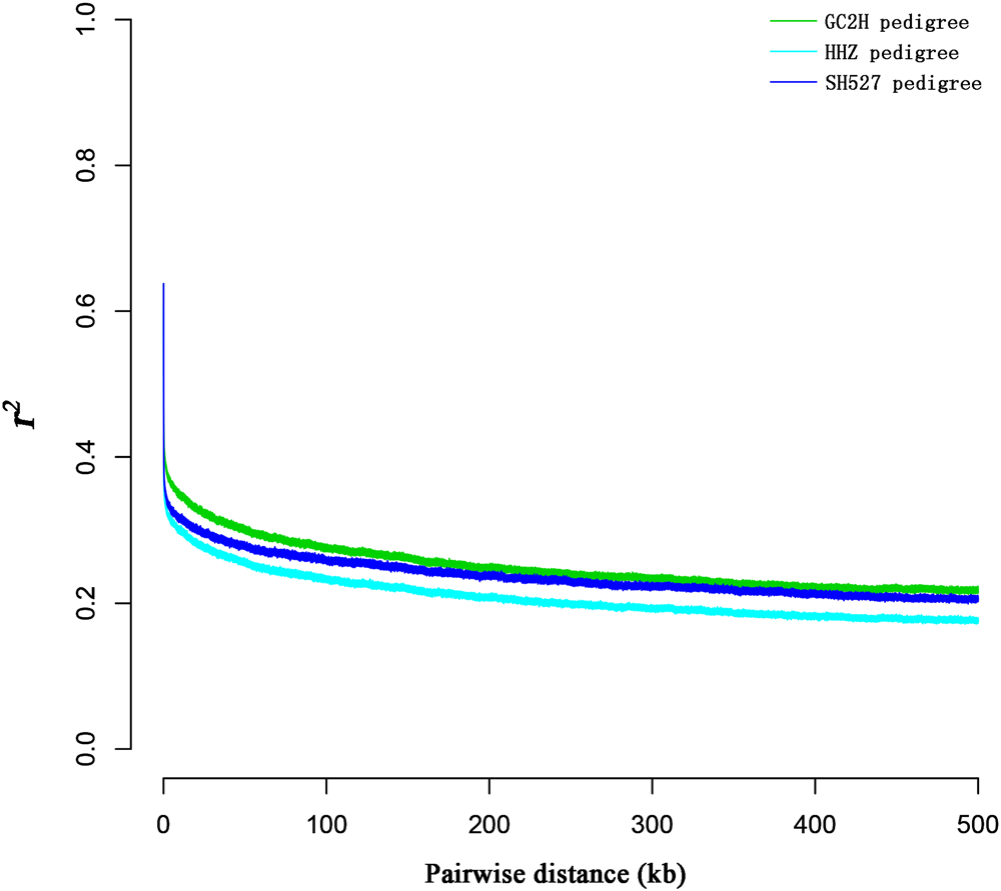
LD decays for GC2H, HHZ, and SH527 pedigrees, as measured by *r* ^*2*^.

To estimate LD patterns in each pedigree, we calculated the average pairwise correlation coefficient (*r* ^*2*^) between pairs of SNPs using Haploview tools^24^. LD decayed to its half-maximum within ~2.5 kb for HHZ and SH527 pedigrees and ~9 kb for GC2H pedigree, whereas *r* ^*2*^ dropped to 0.30, 0.31 and 0.32, respectively (Fig. 3). These LD values were significantly lower than those found in previous studies, which were 65 kb, ~75 kb and 123 kb for *indica*, respectively^25–27^. Compared with other self-compatible plants, *A. thaliana* (~3–4 kb)^28^, wild soybean (~75 kb)^29^, edible peach (~14 kb)^30^, pedigree LD values remained at low levels, which revealed that large scale recombination had occurred, and genomic regions for pedigree varieties were reset frequently during breeding. Moreover, high frequency artificial hybridization was carried out, and varieties with far genetic distance were used as hybrid parents, supported by high *π* values for each pedigrees. All of these factors caused low levels of LD in pedigrees. Breeding practices have shown that elite genes are often linked to deleterious genes, and breaking such negative linkage patterns is the key to the cultivation of elite rice lines.

### Low-diversity genomic regions and zero-diversity genes

Because genomic regions from collateral varieties will be selected, only when they contribute to breeding, these regions can be reserved in offspring varieties (*i.e.*, immediate varieties). Thus, only using immediate varieties for each pedigree (Supplementary Fig. S1), we identified low-diversity genomic regions and zero-diversity genes. We scanned genomic regions using the nucleotide diversity for each pedigree (*π*_*GC2H*_, *π*_*HHZ*_ and *π*_*SH527*_) with 100 kb/10 kb sliding windows. Chromosome segments were empirically selected as low-diversity regions, for which *π* values were less than 0.00011956, 0.00012435 and 0.00009726 for GC2H, HHZ and SH527 pedigrees, respectively. Finally, we identified 93, 102 and 96 genomic regions covering 3.52% (13.15 MB), 3.63% (13.55 MB) and 3.61% (13.47 MB) of the reference genome (Supplementary Table S11). The average length ranged from 127.8 kb to 141.4 kb, and seven longest DNA blocks with lengths of ≧300 kb were observed on chromosome 4 (containing 3 blocks), chromosome 6 (containing 1 blocks) and chromosome 12 (containing 3 blocks) (Supplementary Table S11). Hitting these low-diversity regions, we identified a total of 2,977 genes, and 1,455, 1,478 and 1,594 genes were observed for GC2H, HHZ and SH527 pedigrees, respectively (Supplementary Table S12). Of those genes, 459 were shared across three pedigrees (Supplementary Fig. S5a), indicating that these putative genes underwent strong selection pressure during modern breeding. we also observed that 24 genes were known to have been under selection during rice domestication and improvement, including the domestication gene *sh4* ^31^, the resistance gene *dsm*2^32^ and the yield gene *Ossut2* ^33^, and 10% (123 out of 1,242) of putative artificial selection genes^25^ (Supplementary Table S13).

In addition, we further identified 3,595 zero-diversity genes, including 3,008, 2,633 and 2,488 in GC2H, HHZ and SH527 pedigrees, respectively (Supplementary Table S14). 1,848 (51.4%) genes were common among all pedigrees, and 331 genes existed only in SH527 pedigree (Supplementary Fig. S5b). We also observed 21 well-known genes associated with important domestication and agronomic trait improvements, such as *prog1* ^4^, *sh4* ^31^ and *lc2* ^34^. Besides, 20.4% (253 of 1242) of a set of selected candidate genes^25^ and 23.2% (690 of 2,977) of genes identified from low-diversity genomic regions were also found. Moreover, we observed that 67 of 331 SH527 pedigree-specific zero-diversity genes represented low-diversity genes (Supplementary Table S15). These genes could play an important role for combining ability in restorers.

### Identification of pedigree breeding signature

The immediate varieties from pedigree provide an opportunity to detect important genes that were selected for rice improvement by breeders in the process of breeding. Thus, if one SNP without recurrent selection across continuous pedigree generations was preserved in the end, we empirically consider preserved SNP genotype as pedigree breeding signature, which should be linked to elite alleles. We observed 138,545 (2.72% of GC2H pedigree SNPs) and 58,715 (1.08% of HHZ pedigree SNPs) signature SNPs for GC2H and HHZ pedigrees, respectively (Supplementary Data S2), and further 3,925 and 2,412 genes were obtained, covering 10.4% and 6.4% of annotated japonica genes, respectively. In total, 111,458 (80.5%) and 46,134 (78.6%) inter-genic signature SNPs were observed for GC2H and HHZ pedigrees, respectively (Supplementary Table S16); however, more total SNPs (4,406,715, 86.4% and 4,738,401, 86.7%) were located in the inter-genic region (Table 1), confirming that these signature SNPs have been affected by artificial selections.

To reduce opportunism, we only considered signature SNPs within CDS regions as candidate improvement targets. In total, 2,938 putative genes were identified, corresponding to 2,176 (7,497 signature SNPs, 3.46 SNPs/gene) and 1,236 (3,391 signature SNPs, 2.75 SNPs/gene) genes for GC2H and HHZ pedigrees, respectively (Supplementary Tables S17 and S18). Only 474 (16.1% of the 2,938) genes were shared by both pedigrees, which reflects that pedigree breeding objectives were not perfectly consistent. GC2H pedigree represents semi-dwarfism, while HHZ pedigree covered the semi-dwarfism and GSR breeding stages. We further counted the number of signature SNPs for each putative gene (Supplementary Tables S17 and S18). GO analysis indicated all putative genes from two pedigrees in candidate improvement targets enriched in binding molecular function (Supplementary Table 19). Besides, For HHZ pedigree, two genes (Os11g0615700 and Os08g0254500) with more than 500 signature SNPs were observed, Os11g0615700 (*PAE1*, 524 signature SNPs) belongs to the peptidase T1A family, and Os08g0254500 (*SECY*, 514 signature SNPs) belongs to the SecY/SEC61-alpha family. For GC2H pedigree, we observed two genes with the highest numbers of signature SNPs (more than 136), and these genes belonged to the amidohydrolase (Os12g0468600) and NB-ARC (Os11g0481150) families. Although their biological functions in rice are not clear, transcript levels have been shown in the Uni-prot database. As expected, we observed 46 well-known genes from pedigree breeding signature (Supplementary Table 20), such as grain quality gene *Osppdkb* ^35^ and *SSIIa* ^36^, yield gene *TGW6* ^37^ and disease resistance gene *NLS1*. These genes provide a valuable index for further rice improvements and gene cloning.

### Selective sweeps in the restorer pedigree

**Figure 4:**
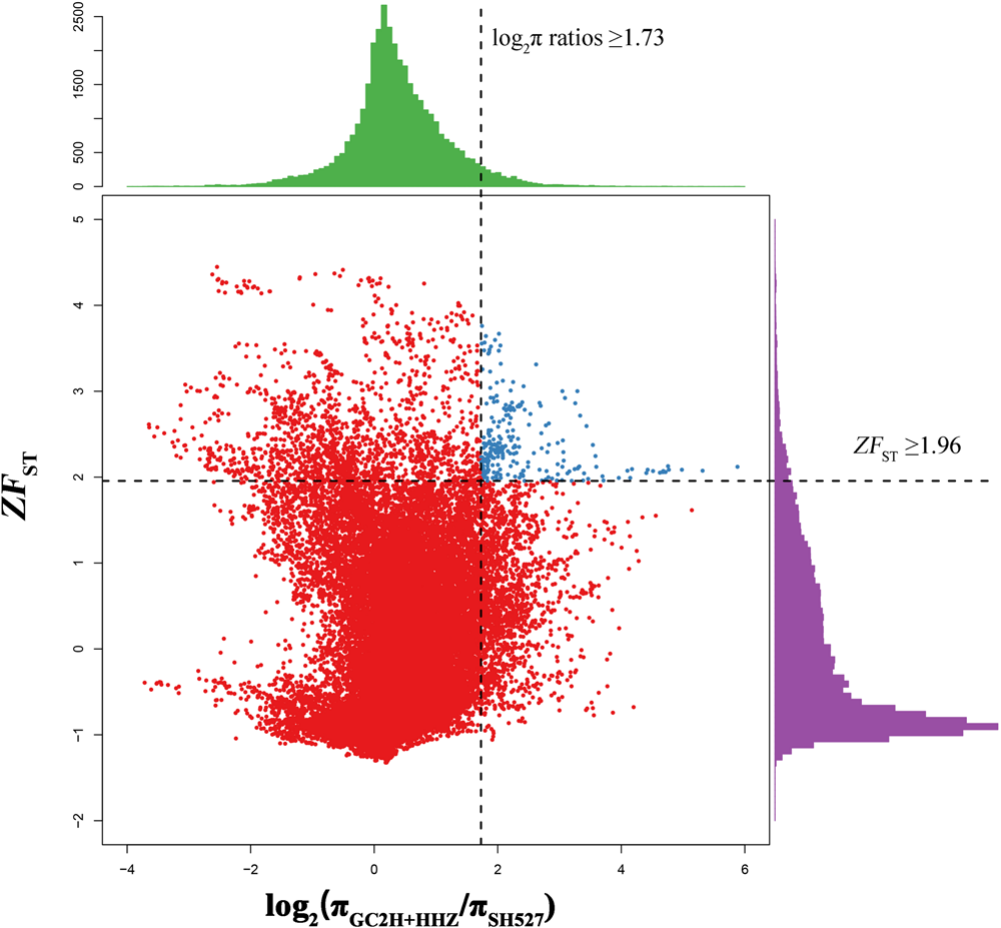
Distribution of π ratios (π_GC2H+HHZ_/π_SH527_) and *ZF*_ST_ values, which are calculated in 100-kb windows sliding in 10-kb steps. Sweep signals are located to the right of the vertical dashed lines (the 5% right tails of the empirical π ratio distribution, log_2_ *π*_*GC2H+HHZ*_/*π*_*SH*527_), and above the horizontal dashed line (the 5% right tail of the empirical *ZF*_ST_ distribution, where *ZF*_ST_ is 1.96).

The previous results have shown a significant genetic differentiation between conventional rice and restorer pedigrees (Fig. 2). This difference has allowed us to identify selective sweeps during restorer breeding. We empirically selected genomic regions with the high right tail of log_2_ *π*_*GC*2+*HHZ*_/*π*_*SH527*_ (top 5%) and Z*F*_ST_ values (top 5%) as candidate sweeps (Fig. 4), where log_2_ *π* ratio was 1.73 and Z*F*_ST_ was 1.96. Finally, we identified 35 potential selective sweeps ranging from 100 kb to 430 kb in length (165 kb on average) (Supplementary Table S21). More regions on chromosomes 6 and 7 were found, suggesting that underwent stronger differential selection than those on other chromosomes during restorer breeding. A total of 5.78 MB occupied 0.16% of the reference genome, corresponding to 749 genes (1.98%) (Supplementary Table S22). GO analysis showed these genes in the selective sweeps enriched in oxidoreductase and dioxygenase molecular function (Supplementary Table 19). Of these genes, 30 genes on chromosomes 6 and 10 also belonged to unique zero-diversity genes and low-diversity region genes in SH527 pedigree (Supplementary Table S15), confirming that they underwent strong differential selection between conventional rice and the restorer pedigrees. Besides, five well-known genes were involved, including *Ghd7*, *wx*, *OsNRAMP5* and *d-6*. This result further confirmed that genes controlling agronomic traits may play important roles in the combining ability effects. Other uncharacterized selected genes identified here provide useful guidance for identifying genes with a high combining ability in rice.

## Discussion

Artificial selection has driven genomic adaptation for traits improvement in crop. Population genetic analysis has been used to identify artificial selection genes in rice^3, 10^. Pedigree analysis^38, 39^ was also be used as effective strategy to reveal the dynamic change of genome during crop breeding. However, genome-wide genetic character of pedigree remained unclear during modern rice breeding. In this study, we found π values for conventional rice pedigrees were slightly higher than those reported in a previous study^26^. Collateral varieties were the sole source of pedigree diversity during pedigree breeding. High pedigree π values revealed that rice lines with far genetic distance were most likely selected as crossing parents for creating new elite varieties, which is also consistent with that distant hybridization is an easy method to produce obviously hybrid vigour^40^. We further calculated π values of differential stages varieties in the pedigree, and low genetic diversity were observed in modern varieties. The π values deceased from 0.00210163 to 0.00181801 in HHZ pedigree. The reason that modern varieties were bred from breeding parents with genetic similarity. Therefore, it is important that the breeders used rice lines with broad genetic background as hybrid parents in further pedigree breeding.

Compared with 0-diversity genes, only 23.2% was overlapped with that from low-diversity genomic regions. During breeding, high frequency recombination has occurred by high frequency artificial hybridization, but we still observed many 0-diveristy genes that located in high diversity genomic regions. Some low diversity regions depends partly on chromosomal location^41^. Thus, we deduced that molecular design breeding with coding region SNPs maybe more effective. Besides, these genes also included well-known important genes, such as *prog1* ^4^, *sh4* ^31^ and *lc2* ^34^, further revealing these genes selected by breeders. From pedigree breeding signatures, we observed some key plant-type and Grain quality genes such as *Osppdkb* ^35^, *SSIIa* ^36^, *TGW6* ^37^ (Supplementary Table 20). And Os07g0421300 (110 signature SNPs) and Os04g0164900 (80 signature SNPs) with starch biosynthesis related quantitative trait loci (QTLs) were also found, suggesting that elite allele of important genes had been selected by breeder in modern rice breeding. In addition, several pollen development-related genes were also found, such as *Rf4*, *Rf6* and *MADS63*, suggesting that these genes also performed important functions for rice yield^10^. However, these selection genes still need more evidence to analysis traits performance by QTL mapping or association test.

Besides, we observed more pedigree-specific SNP genotypes in restorer pedigree. And 692 (51.6%) were found to locate on chromosome 6. Meanwhile, more differentially selection regions were also found in chromosomes 6 and 7 in restores pedigree, indicating that chromosome 6 may have undergone stronger differential selection than other chromosomes between conventional and restore pedigrees. Thus, chromosome 6 might partly explains the high level of combining ability in restores. Interesting, one big DNA blocks (320 kb) were observed on chromosomes 10. This is consistent with the most important gene for the fertility restoration of 3-line CMS system that locates on chromosome 10. Besides, we also find that some well-known genes for important agronomic traits such as *Ghd7*, *wx*, *OsNRAMP5* and *d-6* included selection genes in restorer pedigree. At present, the rice heterosis gene *Ghd7.1* (Os07g0695100) have been identified^42^. In our analysis, *Ghd7* (Os07g0261200)^43^ was also identified. Although they are located in different regions, two pleiotropic genes control rice height, flowering and grain number per panicle. One explanation for this difference is that different populations were used in the two studies. These results further confirmed that genes controlling agronomic traits may play important roles in combining ability effects.

In this study, a larger number of low-diversity regions, 0-diveristy and pedigree signatures genes were identified from three breeding pedigree and potentially included elite alleles of genes for important agronomic traits. Therefore, our study provides insights into the genetic basis of backbone varieties and should be helpful to high-through assessment of allele performance in rice populations, and will have immediate implications for performing genome-wide breeding by design.

## Methods

### Plant material and sequencing

A total of 52 elite rice varieties derived from three breeding pedigrees (Huanghuazhan, HHZ; Guichao2hao, GC2H; Shuhui527, SH527) were selected for Illumina sequencing (Supplementary Table S1, Supplementary Fig. S1). The HHZ and GC2H pedigrees belong to inbred varieties, while the SH527 pedigree is designed as important CMS (Cytoplasmic Male Sterile) restorers of hybrid cultivars.

All samples were planted in Wuhan, China. Young leaves were dried with silica gel and genomic DNA was extracted using the CTAB method^44^. At least 5 μg of genomic DNA for each variety was sheared into 200–5000 bp using the Covaris system (Life Technologies, American). After end-repairing and A-tailing, the DNA fragments were ligated to paired-end adaptors, and PCR amplified with ~500 bp inserts for library construction according to the manufacturer’s instructions (Illumina). We generated approximately 3–5 Gb of sequence data with 125-bp paired-end reads for each variety using the Illumina HiSeq 2500 platform (Supplementary Table S1). All sequence data has been deposited in NCBI’s Sequence Read Archive (SRA) database with accession number SRP080763.

### Mapping and variation calling

First, adaptor sequences, reads with an N ratio > 10%, reads with more than 50% of the Q value (<5) ratio, and reads with average values <15 were removed from the raw reads. For variant calling, we selected Nipponbare as the reference genome (IRGSP-1.0, http://rapdb.dna.affrc.go.jp), and BWA software^11^ was used to map all reads from each sample to the reference genome. SAMtools^45^ was used to convert the mapping results to a bam format and to further sort the reads. The reads caused by PCR duplication were removed by the SAMtools rmdup function. Variation detection was performed with the Genome Analysis Toolkit (GATK, version 3.1)^46^. Using multi-sample analysis, we aligned all reads to the reference genome with a coverage of no less than 2 and a total coverage of <4000. After adding headers processing for the reads, realignment around the INDELs was performed with the Realigner TargetCreator package to identify regions that needing to be re-aligned. The indelRealigner was used to perform re-alignment within these regions. Index files were generated using SAMtools. We used HaplotypeCaller to identify variations (SNPs and INDELs) for each variety. The threshold of SNP calling was set to 20 for both the base quality and mapping quality. All variations were joined together by GenotypeGVCFs.

### Identification of novel genes

To identify novel genes, we used 93–11 as a mapping reference genome (http://rice.genomics.org.cn). The unmapped reads from each pedigree were pooled, and three pools of unmapped reads were assembled separately into contigs. Finally uni-contigs for three contigs pools were further obtained by SOAP*denovo* ^47^ tools with default parameters. Contigs shorter than 1 kb were discarded. Only ≥4 kb uni-contigs were selected for novel genes prediction by GENSCAN^48^, and gene annotations were accomplished by running local BLAST searches to query the non-redundant and SWISS-PROT protein sequence databases.

### Validation of SNP calling

To evaluate the accuracy of the SNPs calling in this study, we compared our SNP data with that reported by previous studies^14^. Raw data on the same varieties as our studies were loaded and used to call SNPs according to the previous flow and criteria (Supplementary Table S4). The concordances in both SNP sets were defined as validated SNPs. Furthermore, we randomly selected 3 genomic regions containing 66 SNPs for PCR amplification and Sanger sequencing (Supplementary Tables S5 and S6).

### Identification of large-effect variants

The identified SNPs were further categorized based on their locations and effects using SNPEff version 3.1b^49^. We considered variants annotated as large-effect variants if they sorted into one of the following effect variants: splice site acceptor; splice site donor; start lost; stop gained and stop lost for proteins.

### Phylogenetic analysis and population structure

Using SNPs in all 52 varieties, a neighbour-joining tree was built by MEGA6.0 with 1000 bootstrap replicates^50^. The population structure was investigated using STRUCTURE^51^. In addition, we performed PCA^52^. Two-dimensional coordinates were plotted for the 52 rice accessions.

### LD analysis

To evaluate the LD level in each pedigree, the correlation coefficients (*r* ^*2*^) of SNPs were calculated using Haploview software^24^. The parameters were as follows: -n pedfile -info -log -maxdistance 500000 -minMAF 0.005 -hwcutoff 0.001 -dprime -memory 5120. Then, R scripts were used to plot the values.

### Detection of low-diversity regions and zero-diversity genes

To identify genomic regions with low sequence diversity, we calculated π (the average pairwise nucleotide diversity) to measure the level of sequence variability for each pedigree^19^, with a 100-kb window and a step size of 10 kb across the genome. The regions with low *π* values (the top 1% of the left tail) were chosen as potential low-diversity sweeps. Based on the genome annotation, we also identified zero-diversity genes for which no SNPs could be identified (containing 1 kb upstream of transcriptional start sites) among the re-sequenced varieties in one pedigree.

### Detection of pedigree breeding signature

To identify genes that experienced the pedigree breeding selection, we investigated SNPs transmission patterns in immediate varieties of two conventional pedigrees. If one SNP without recurrent selection across continuous pedigree generations were observed, such as AATTTT corresponding to S29, S17, S13, S03, S14 and S16 from the GC2H pedigree (Supplementary Fig. S1), rather than AATTAT, AATTTA or other patterns, this SNP was considered as pedigree breeding signature, and the linked genes can be further identified. Based on the locus position and sequences of cloned gene, elite alleles of each gene were also be observed.

### Identification of selective sweeps

A sliding-windows method (100-kb sliding windows with a step of 10-kb) was used to calculate the *π* ratios (*π*_*GC*2*H*+*HHZ*_/_*SH*527_) and genetic differentiation (*ZF*_ST_)^53, 54^ between the two populations. To identify potential sweeps affected by artificial selection, we considered the distribution of the log_2_ *π* ratios and *ZF*_ST_ values. We empirically selected the genomic regions with simultaneous high log_2_ *π* ratios (5% right tails of *π*_*GC*2*H*+*HHZ*_/*π*_*SH*527_ and high *ZF*_ST_ values (5% right tails) as selective regions signals across the genome, which are predicted to harbour genes that underwent a selective sweeps (Fig. 4).

## Electronic supplementary material

Supplementary Data file

Additional information file
