# Supplementary material for: Pedigree-based genome re-sequencing reveals genetic variation patterns of elite backbone varieties during modern rice improvement
Source: Sci Rep. 2017 Mar 22;7:292. doi: 10.1038/s41598-017-00415-1 (PMC5428501; doi:10.1038/s41598-017-00415-1)
Supplement: Supplementary file 2 — Additional information file [file 41598_2017_415_MOESM2_ESM.pdf]

Pedigree-based genome re-sequencing reveals genetic variation patterns of elite backbone varieties during modern rice improvement

Xingfei Zheng, Lanzhi Li, Fan Liang, Changjun Tan, Shuzhu Tang, Sibin Yu, Ying Diao, Shuangcheng Li,and Zhongli Hu

Supplementary Table S1 The detailed information of pedigree varieties and sequencing data statistics

Individual code	Accession name	Pedigree group[a]	Yield High-quality Base(Mb)	Reads (M)	Reads length (bases)	% of >= Q30 Bases	Mapping Rate (%)	Duplication Rate(%)	Uniq Depth (X)	Uniq Coverage at least 1 × (%)
S01	Aizizhan	GC2H, HHZ	3,486	27.89	125	86.57	94.74	0.9	8.85	85.98
S02	Dongqiubo	GC2H	4,941	39.53	125	88.2	94.81	1.07	12.56	89.16
S03	Guangqiuai	GC2H	4,501	36.01	125	89.05	94.91	1.07	11.45	88.66
S04	Guangchangai3784	HHZ	4,822	38.58	125	88.2	95.08	0.92	12.29	88.82
S05	Qingfengai	HHZ	4,863	38.91	125	87.63	95	1.23	12.38	87.77
S06	Jiangerai	HHZ	1,918	15.35	125	87.5	95.68	0.61	4.92	80.63
S07	Qingerai	HHZ	6,012	48.09	125	87.63	95	0.92	15.31	90.1
S08	Jiduilun	HHZ	5,544	44.35	125	88.72	94.95	1.14	14.11	89.56
S09	Kuoyedao	HHZ	5,657	45.25	125	88.77	95.02	0.91	14.41	89.59
S10	Yeqinglun	HHZ	5,711	45.69	125	89.37	95.07	0.89	14.55	90.25
S11	Lucaihao	GC2H, HHZ	6,044	48.35	125	89.77	94.95	1.2	15.38	90.22
S12	Huanan15	GC2H	6,083	48.66	125	89.67	94.52	1.55	15.41	90.05
S13	Guangchangai4182	GC2H	4,902	39.22	125	86.85	95.3	0.78	12.52	89.44
S14	Guiyangai49	GC2H	5,381	43.05	125	85.51	94.96	0.9	13.69	89.83
S15	Chaoyangzao18	GC2H	5,286	42.29	125	87.32	94.91	0.98	13.45	89.62
S16	Guichao2hao	GC2H	5,901	47.21	125	87.14	94.86	0.97	15	90.37
S17	Guangchang13	GC2H, HHZ	5,932	47.46	125	87.37	95.15	0.84	15.13	90.34
S18	Fengqingai	HHZ	5,361	42.89	125	87.94	95.01	0.87	13.65	89.74
S19	Fengaizhan1hao	HHZ	5,274	42.19	125	87.02	95.12	0.79	13.45	89.62
S20	Huanghuazhan	HHZ	4,444	35.55	125	84.71	95.04	0.81	11.32	88.09
S21	Huangxinzhan	HHZ	4,966	39.73	125	86.42	94.91	0.79	12.63	89.19
S22	28zhan	HHZ	4,870	38.96	125	86.07	94.98	0.73	12.4	89.76
S23	Fenghuazhan	HHZ	5,065	40.52	125	86.95	94.93	0.78	12.89	90.04
S24	Qingliuai	HHZ	5,302	42.41	125	85.22	94.73	1.25	13.46	89.36
S25	Fengbazhan	HHZ	5,102	40.82	125	83.9	94.6	1.13	12.93	88.23
S26	Changsizhan	HHZ	5,069	40.55	125	84.88	94.41	1.48	12.83	87.67
S27	Huasizhan	HHZ	5,351	42.81	125	85.18	94.64	1.48	13.57	88.97
S28	Teqing	HHZ	5,475	43.8	125	86.94	93.38	1.09	13.7	89.84
S29	Nantehao	GC2H, HHZ	4,524	36.2	125	86.7	95.11	0.69	11.53	89.53
S30	Gu154	SH527	4,936	39.49	125	84.9	94.73	0.99	12.53	87.86
S31	Minghui63	SH527	5,758	46.07	125	85.75	94.4	0.96	14.57	89.61
S32	Shuhui527	SH527	5,208	41.67	125	86.84	95.04	0.78	13.27	89.31
S33	Shuhui288	SH527	5,279	42.23	125	87.07	94.9	1.12	13.43	87.69
S34	Shuhui707	SH527	5,762	46.09	125	85.85	94.65	0.9	14.62	89.72
S35	Longhui11	SH527	5,648	45.19	125	85.57	94.55	1.03	14.31	87.74
S36	Nanhui511	SH527	6,008	48.06	125	85.84	94.53	1.03	15.22	88.7
S37	Xianghui529	SH527	7,605	60.84	125	85.62	94.34	1.25	19.23	91.1
S38	Jinhui275	SH527	5,972	47.78	125	86.18	94.71	0.94	15.16	89.28
S39	Huhui5240	SH527	5,793	46.34	125	86.05	94.82	0.86	14.72	87.51
S40	Minghui2088	SH527	4,744	37.95	125	86.16	95.22	0.56	12.11	88.58
S41	Shanhui287	SH527	4,412	35.3	125	84.97	95.11	0.68	11.25	87.41
S42	Shanhui8281	SH527	5,106	40.85	125	86.3	95	0.84	13	88.09
S43	R238	SH527	4,425	35.4	125	88.21	95.25	0.67	11.3	87.93
S44	R727	SH527	3,788	30.3	125	88.3	95.13	0.78	9.66	87.67
S45	205R	SH527	4,388	35.11	125	88.43	94.89	0.8	11.16	86.87
S46	781R	SH527	4,794	38.35	125	87.47	95.11	0.81	12.22	88.44
S47	3301R	SH527	5,346	42.77	125	88.69	95.11	0.95	13.63	87.45
S48	IR24	SH527	5,736	45.89	125	87.92	95.13	0.72	14.62	88.42
S49	IR1544	SH527	4,967	39.74	125	88.83	95.46	0.87	12.71	85.78
S50	Gui630	SH527	4,676	37.41	125	88.58	95.2	0.87	11.93	82.54
S51	Fu36-2	SH527	4,730	37.84	125	88.23	95.29	0.67	12.08	88.11
S52	R1318	SH527	4,922	39.38	125	87.77	95.07	0.79	12.54	86.08

a GC2H: Guichao 2hao pedigree; HHZ: Huanghuazhan pedigree; SH527: Shuhui527 pedigree

Pedigree-based genome re-sequencing reveals genetic variation patterns of elite backbone varieties during modern rice improvement

Xingfei Zheng, Lanzhi Li, Fan Liang, Changjun Tan, Shuzhu Tang, Sibin Yu, Ying Diao, Shuangcheng Li,and Zhongli Hu

Supplementary Table S2 Detailed statistics for contigs assembly from the unmapped reads in each breeding pedigree

Pedigree[a]	No. contigs	No. contigs (⩾1kb)	No. contigs (⩾4kb)	Predicited/functional annotation genes[b]	Total length (bp)	Average length(bp)	Longest(bp)	GC content(%)
GC2H	17,193	1040	15	22/17	7,815,383	455	5630	45.5
HHZ	19,590	1208	19	25/18	8,859,365	452	9607	45.1
SH527	15,906	914	9	12/9	7,015,597	441	6526	47.4
Total	23,001	1823	28	37/26	10,981,947	478	9607	45.9

Note: (a) GC2H represents GC2H pedigree, HHZ represents HHZ pedigree, and SH527 represents SH527 pedigree;

(b) only ⩾4kb contigs were used for *de novo* gene prediction in three rice pedigrees

Pedigree-based genome re-sequencing reveals genetic variation patterns of elite backbone varieties during modern rice improvement

Xingfei Zheng, Lanzhi Li, Fan Liang, Changjun Tan, Shuzhu Tang, Sibin Yu, Ying Diao, Shuangcheng Li,and Zhongli Hu

Supplementary Table S3 Annotation information of novel genes identified from the unmapped uni-contigs(⩾4kb)

Code	Contig name	Pedigree[a]			Number of hits in japonica	Lowest E-value	Homologous genes in *japonica*	Function	Nr accession	Swiss-Prot accession	Homolog Protein name
		GC2H	HHZ	SH527							
Contig1	ConsensusfromContig1	+	+	+	2	1.72E-11	Os11g0588932	-	CAE03136.1	-	-
Contig2	ConsensusfromContig2	+	+	+	446	0	Os07g0521600	Similar to NBS-LRR protein (Fragment).	BAC57648.1	Q9LRR5(DRL21_ARATH)	Putative disease resistance protein At3g14460
Contig3	ConsensusfromContig3	+	+	+	7	1.89E-138	Os11g0513000	Hypothetical conserved gene.	ABA93872.1	-	-
Contig4	ConsensusfromContig4	+	+	+	294	0	Os03g0207000	-	CAH66365.1	Q8L3R3(RFL1_ARATH)	Disease resistance protein RFL1
Contig5	ConsensusfromContig5	+	+	+	20	3.79E-79	Os06g0484800	Reverse transcriptase domain containing protein.	EEC75227.1	P14381(YTX2_XENLA)	protein
Contig6	ConsensusfromContig7	+	+	+	3	0	Os02g0115600	S1, RNA binding domain containing protein.	NP_001045672.1	Q6GGT5(RS1_STAAR)	30S ribosomal protein S1
Contig7	ConsensusfromContig9	+	+	+	394	0	Os01g0547000	Similar to NBS-LRR19.	EEE54376.1	Q39214(RPM1_ARATH)	Disease resistance protein RPM1
Contig8	ConsensusfromContig14	+	+	+	30	7.56E-150	Os07g0539200	Conserved hypothetical protein.	EAZ40147.1	P14381(YTX2_XENLA)	protein
Contig9	ConsensusfromContig19	-	+	+	12	2.07E-75	Os10g0195100	Hypothetical conserved gene.	XP_008664019.1	-	-
Contig10	ConsensusfromContig28	-	+	+	22	2.63E-42	Os06g0580100	sequence.	XP_003563711.1	-	-
Contig11	ConsensusfromContig31	+	+	+	214	2.30E-35	Os11g0606800	Similar to NB-ARC domain containing protein.	EAY88487.1	Q9LQ54(DRL12_ARATH)	Putative disease resistance protein At3g14460
Contig12	ConsensusfromContig36	+	+	-	25	1.56E-81	Os11g0146200	Ankyrin repeat domain containing protein.	CBX25488.1	Q9QW30(NOTC2_RAT)	Neurogenic locus notch homolog protein 2
Contig13	ConsensusfromContig39_1	+	+	+	10	9.79E-26	Os06g0484800	Reverse transcriptase domain containing protein.	CAE02720.2	P14381(YTX2_XENLA)	protein
Contig14	ConsensusfromContig52	+	+	+	124	4.30E-21	Os02g0301800	NB-ARC domain containing protein.	BAD16427.1	Q39214(RPM1_ARATH)	Disease resistance protein RPM1
Contig15	ConsensusfromContig56	+	+	+	1	1.80E-07	Os09g0495500	Conserved hypothetical protein.	EAZ45233.1	-	-
Contig16	ConsensusfromContig124	+	+	-	-	1E-22	-	retrotransposon protein	AAO66546.1	-	-
Contig17	ConsensusfromContig339	-	+	+	13	2.90E-146	Os08g0208400	Transposon, En/Spm-like domain containing protein.	XP_002458085.1	-	-
Contig18	ConsensusfromContig298_1	+	+	+	157	1.06E-80	Os11g0462500	Similar to NB-ARC domain containing protein.	AAY34259.1	Q9FJB5(RP8L3_ARATH)	Putative disease resistance protein At3g14460
Contig19	ConsensusfromContig298_2	+	+	+	4	1.97E-24	Os07g0250900	Harpin-induced 1 domain containing protein.	EMT18859.1	-	-
Contig20	ConsensusfromContig474	+	+	+	2	5.74E-43	Os08g0422000	Similar to CM0545.530.nc protein (Fragment).	EAZ42775.1	Q8H329(MTP8_ORYSJ)	Metal tolerance protein 8
Contig21	ConsensusfromContig3073	+	+	+	3	1.99E-13	Os06g0360600	Similar to OSIGBa0104J13.3 protein.	AAT01348.1	P0CT43(TF28_SCHPO)	Transposon Tf2-8 polyprotein
Contig22	GC2H_51129_1	+	-	-	322	2.03E-164	Os11g0689100	NB-ARC domain containing protein.	XP_010237386.1	Q39214(RPM1_ARATH)	Disease resistance protein RPM1
Contig23	GC2H_51139_2	+	-	-	20	9.03E-87	Os04g0220501	Hypothetical conserved gene.	CAE02720.2	-	-
Contig24	HHZ_64168	-	+	-	12	0	Os06g0271400	Transposase, Ptta/En/Spm	BAD68428.1	-	-
Contig25	HHZ_64180	-	+	-	377	5.20E-111	Os12g0564800	NB-ARC domain containing protein.	EMT30310.1	Q39214(RPM1_ARATH)	Disease resistance protein RPM1
Contig26	HHZ_64194	-	+	-	9	0	Os08g0176300	-	BAD03229.1	-	-
Contig27	HHZ_64198	-	+	-	34	4.10E-164	Os08g0208400	Transposon, En/Spm-like domain containing protein.	ABA97584.2	-	-
Contig28	HHZ_64172_1	-	+	-	1	6.96E-11	Os01g0744850	-	ABA95871.1	-	-
Contig29	HHZ_64172_2	-	+	-	64	4.18E-90	Os01g0524100	-	EMT11823.1	-	-
Contig30	SH527_53621	-	-	+	43	8.41E-124	Os12g0568100	Similar to Zinc knuckle family protein, expressed.	ABA99635.2	-	-
Contig31	SH527_53625	-	-	+	4	4.78E-29	Os11g0467632	Similar to Aminotransferase-like.	ABA95103.1	-	-
Contig32	SH527_53633	-	-	+	72	2.87E-81	Os11g0681300	Cyclin-like F-box; FAR1; Zinc finger, SWIM-type.	XP_010238888.1	Q9SZL8(FRS5_ARATH)	Protein FAR1-RELATED SEQUENCE 5
Contig33	SH527_53637	-	-	+	462	0	Os11g0429100	Hypothetical conserved gene.	XP_010236423.1	Q7XBQ9(RGA2_SOLBU)	Disease resistance protein RGA2

Note: (a) "+" means present , and "-" means absent and GC2H represents GC2H pedigree, HHZ represents HHZ pedigree, and SH527 represents SH527 pedigree.

Pedigree-based genome re-sequencing reveals genetic variation patterns of elite backbone varieties during modern rice improvement

Xingfei Zheng, Lanzhi Li, Fan Liang, Changjun Tan, Shuzhu Tang, Sibin Yu, Ying Diao, Shuangcheng Li,and Zhongli Hu

Supplementary Table S4 Concordance rates of calling SNPs between the Rice 3000 Genomes Project and our re-sequencing data

Individual code	Variety name	SRA Number (3,000 rice genomes project)	Number of compared SNPs	Consistent	Inconsistent	Consistent/inconsistent rates
S11	Lucaihao	ERS470411	2594487	2576234	18253	0.9930/0.0070
S16	Guichao2hao	ERS470277	4138347	4109719	28628	0.9931/0.0069
S29	Nantehao	ERS468110	4166869	4138220	28649	0.9931/0.0069
S30	Gu154	ERS470348	3356133	2866252	489881	0.8540/0.1460
S31	Minghui63	ERS470509	3659172	3483016	176156	0.9519/0.0481
S50	Gui630	ERS470440	3348307	2752127	596180	0.8220/0.1781

Pedigree-based genome re-sequencing reveals genetic variation patterns of elite backbone varieties during modern rice improvement

Xingfei Zheng, Lanzhi Li, Fan Liang, Changjun Tan, Shuzhu Tang, Sibin Yu, Ying Diao, Shuangcheng Li,and Zhongli Hu

Supplementary Table S5 Primer sequences for SNP validation by PCR amplification and Sanger sequencing

Primers	Chromosome	Primer Seqences (5'-3')	Tm(℃)	Products(bp)
Rm1	chr01	Forward:TTGATTCACCTGGAGGAAGC	60.24	746
		Reverse:CGTGGAGCCATCTGCTTATT		
Rm2	chr04	Forward:ATGGTATGCATGCTTCTTGG	58.6	654
		Reverse:ATGGTATGCATGCTTCTTGG		
Rm3	chr04	Forward:TCACAGTGCTGCTGTTGAAG	59.45	659
		Reverse:GCGCTCAATATTCGTGTGAG		

Pedigree-based genome re-sequencing reveals genetic variation patterns of elite backbone varieties during modern rice improvement

Xingfei Zheng, Lanzhi Li, Fan Liang, Changjun Tan, Shuzhu Tang, Sibin Yu, Ying Diao, Shuangcheng Li,and Zhongli Hu

Supplementary Table S6 The information of SNPs loci for validation by PCR amplification and Sanger sequencing

Chromosome	SNPs position	SNPs type
Rm1		
chr01	20284560	A/G
chr01	20284575	C/G
chr01	20284596	A/G
chr01	20284601	A/G
chr01	20284642	A/G
chr01	20284647	A/G
chr01	20284686	C/G
chr01	20284692	A/G
chr01	20284707	A/G
chr01	20284743	A/G
chr01	20284753	G/T
chr01	20284781	C/G
chr01	20284788	C/T
chr01	20284790	C/G
chr01	20284799	A/C
chr01	20284806	G/T
chr01	20284808	C/T
chr01	20284809	A/G
chr01	20284816	A/T
chr01	20284818	C/T
chr01	20284869	G/T
chr01	20284871	A/T
chr01	20284874	C/T
chr01	20284876	C/G
chr01	20284877	A/G
chr01	20284878	A/C
chr01	20284880	A/G
chr01	20284914	A/G
chr01	20284933	A/C
chr01	20285179	A/C
Rm2		
chr04	17669717	C/T
chr04	17669735	C/T
chr04	17669779	A/T
chr04	17669785	A/C
chr04	17669798	A/G
chr04	17669805	A/G
chr04	17669827	A/G
chr04	17669861	C/T
chr04	17669891	C/T
chr04	17669903	A/G
chr04	17669921	A/G
chr04	17669940	A/G
chr04	17669967	C/T
chr04	17670049	C/T
chr04	17670051	A/G
Rm3		
chr04	21518845	A/G
chr04	21518871	C/T
chr04	21518900	A/G
chr04	21518902	A/G
chr04	21518913	C/T
chr04	21518958	A/G
chr04	21519017	C/T
chr04	21519019	A/G
chr04	21519059	A/G
chr04	21519131	C/T
chr04	21519158	C/T
chr04	21519185	A/C
chr04	21519187	A/C
chr04	21519234	A/G
chr04	21519246	A/T
chr04	21519303	C/T
chr04	21519386	A/G
chr04	21519407	C/T
chr04	21519426	A/C
chr04	21519439	A/G
chr04	21519446	G/T

Pedigree-based genome re-sequencing reveals genetic variation patterns of elite backbone varieties during modern rice improvement

Xingfei Zheng, Lanzhi Li, Fan Liang, Changjun Tan, Shuzhu Tang, Sibin Yu, Ying Diao, Shuangcheng Li,and Zhongli Hu

Supplementary Table S7 The accuracy rates of identified SNPs by PCR amplification and Sanger sequencing

Indivadual code	Varieties	Number of SNPs checked	Percent of true SNPs(%)	Persent of false SNPs(%)
S01	Aizizhan	31	30(96.77%)	1(3.13%)
S07	Qingerai	43	42(100%)	0(0)
S09	Kuoyedao	15	15(100%)	0(0)
S11	Lucaihao	15	15(100%)	0(0)
S13	Guangchangai4182	44	44(100%)	0(0)
S20	Huanghuazhan	31	31(100%)	0(0)
S24	Qingliuai	15	15(100%)	0(0)
S26	Changsizhan	36	36(100%)	0(0)
S28	Teqing	15	15(100%)	0(0)
S29	Nantehao	32	31(96.88%)	1(3.12%)
S33	Shuhui288	35	35(100%)	0(0)
S43	R238	35	34(97.14%)	1(2.86%)
S49	IR1544	20	19(95%)	1(5.00%)
S51	Fu36-2	11	11(100%)	0(0)
Total		378	373(98.68%)	5(1.32%)

Pedigree-based genome re-sequencing reveals genetic variation patterns of elite backbone varieties during modern rice improvement

Xingfei Zheng, Lanzhi Li, Fan Liang, Changjun Tan, Shuzhu Tang, Sibin Yu, Ying Diao, Shuangcheng Li,and Zhongli Hu

Supplementary Table S8 Statistics of large-effect SNPs

Pedigree	Splice acceptor variant	Splice donor variant	Start lost	Stop gained	Stop lost	Total
GC2H	396	411	309	2740	693	4549
HHZ	420	435	319	2985	705	4864
SH527	396	421	331	2890	697	4735
Total	502	513	375	3653	808	5851

Note: GC2H represents GC2H pedigree, HHZ represents HHZ pedigree, and SH527 represents SH527 pedigree.

Pedigree-based genome re-sequencing reveals genetic variation patterns of elite backbone varieties during modern rice improvement

Xingfei Zheng, Lanzhi Li, Fan Liang, Changjun Tan, Shuzhu Tang, Sibin Yu, Ying Diao, Shuangcheng Li,and Zhongli Hu

Supplementary Table S9 The distribution of INDELs located in different genomic regions

Pedigree	No. varieties	Intergenic	Intron	5'UTR	CDS	3'UTR	Non coding exon	Total
GC2H	11	823,106	75,769	15,378	18,053	24,523	1,908	958,737
HHZ	22	922,901	81,498	16,315	19,705	26,028	2,183	1,068,630
SH527	23	838,865	75,934	15,705	18,784	24,351	1,904	975,543
Total	52	1,124,628	96,792	19,833	24,656	31,335	2,624	1,299,868

Pedigree-based genome re-sequencing reveals genetic variation patterns of elite backbone varieties during modern rice improvement

Xingfei Zheng, Lanzhi Li, Fan Liang, Changjun Tan, Shuzhu Tang, Sibin Yu, Ying Diao, Shuangcheng Li,and Zhongli Hu

Supplementary Table S10 Functional annotation of specific large-effect SNPs in restorer (SH527) pedigree

Chromosome	Position	SNPs	Effect types	Gene ID	Function	SWISS-PROT accession
chr02	986330	C/T	stop gained	Os02g0118800	Similar to NBS-LRR disease resistance protein homologue	Q5VPE6_ORYSJ
chr04	5688300	A/T	splice donor variant	Os04g0183401	Hypothetical protein.	A0A0P0W6Y7_ORYSJ
chr06	2036652	T/C	start lost	Os06g0138400	Hypothetical conserved gene.	Q5VPG5_ORYSJ
chr06	2112789	G/T	stop gained	Os06g0140300	Leucine-rich repeat(LRR) N-terminal domain containing protein.	Q5VPE6_ORYSJ
chr06	2114036	C/T	stop gained	Os06g0140300	Leucine-rich repeat(LRR) N-terminal domain containing protein.	Q5VPE6_ORYSJ
chr06	2187658	T/C	stop lost	Os06g0141950	Similar to MYB transcription factor.	A0A0P0WST9_ORYSJ
chr06	2442358	A/T	stop gained	Os06g0146650	Hypothetical protein.	A0A0P0WSX7_ORYSJ
chr06	3042529	C/T	stop gained	Os06g0159600	Tetratricopeptide-like helical domain containing protein.	Q5VMX_ORYSJ
chr06	3245705	A/T	stop lost	Os06g0163900	Conserved hypothetical protein.	Q5VRS9_ORYSJ;A0A0N7KLL0_ORYSJ
chr06	3300607	T/C	splice acceptor variant	Os06g0165300	Transposase	Q0DEA2_ORYSJ;A0A0P0WTC3_ORYSJ
chr10	20939649	A/G	splice donor variant	Os10g0537450	Hypothetical protein.	A0A0P0XWM8_ORYSJ

Pedigree-based genome re-sequencing reveals genetic variation patterns of elite backbone varieties during modern rice improvement
Xingfei Zheng, Lanzhi Li, Fan Liang, Changjun Tan, Shuzhu Tang, Sibin Yu, Ying Diao, Shuangcheng Li,and Zhongli Hu

Supplementary Table S11 Genomic regions with low diversity identified from three breeding pedigrees

**Chromosome**	**Position start**	**Positon end**	**Length(kb)**	$\pi_{min, 100kb\ windows}$	$\pi_{max, 100kb\ windows}$
**GC2H peidgree**					
chr01	4,170,001	4,280,000	110.0	0.000078	0.000080
chr01	7,400,001	7,520,000	120.0	0.000076	0.000081
chr01	8,330,001	8,450,000	120.0	0.000115	0.000116
chr01	12,990,001	13,090,000	100.0	0.000026	0.000026
chr01	16,750,001	16,920,000	170.0	0.000029	0.000094
chr01	21,130,001	21,330,000	200.0	0.000068	0.000114
chr01	21,490,001	21,590,000	100.0	0.000118	0.000118
chr01	26,490,001	26,620,000	130.0	0.000095	0.000104
chr01	43,260,001	43,360,000	100.0	0.000068	0.000068
chr02	9,280,001	9,390,000	110.0	0.000066	0.000093
chr02	13,610,001	13,760,000	150.0	0.000020	0.000100
chr02	16,170,001	16,270,000	100.0	0.000077	0.000077
chr02	23,280,001	23,390,000	110.0	0.000066	0.000098
chr03	570,001	780,000	210.0	0.000043	0.000118
chr03	1,390,001	1,580,000	190.0	0.000095	0.000117
chr03	2,820,001	2,940,000	120.0	0.000096	0.000100
chr03	3,820,001	3,930,000	110.0	0.000101	0.000109
chr03	4,240,001	4,410,000	170.0	0.000034	0.000093
chr03	4,910,001	5,010,000	100.0	0.000112	0.000112
chr03	5,420,001	5,590,000	170.0	0.000073	0.000113
chr03	9,670,001	9,770,000	100.0	0.000094	0.000094
chr03	10,800,001	10,930,000	130.0	0.000097	0.000113
chr03	27,050,001	27,160,000	110.0	0.000107	0.000109
chr03	28,420,001	28,570,000	150.0	0.000083	0.000118
chr03	28,650,001	28,850,000	200.0	0.000076	0.000107
chr03	29,070,001	29,190,000	120.0	0.000102	0.000106
chr03	30,790,001	30,920,000	130.0	0.000089	0.000107
chr03	31,100,001	31,260,000	160.0	0.000056	0.000082
chr03	31,570,001	31,770,000	200.0	0.000075	0.000117
chr03	34,970,001	35,070,000	100.0	0.000087	0.000087
chr03	36,160,001	36,300,000	140.0	0.000045	0.000101
chr04	9,080,001	9,230,000	150.0	0.000049	0.000100
chr04	15,550,001	15,850,000	300.0	0.000000	0.000059
chr04	26,520,001	26,660,000	140.0	0.000073	0.000119
chr04	28,520,001	28,700,000	180.0	0.000108	0.000116
chr04	34,150,001	34,380,000	230.0	0.000041	0.000106
chr04	34,410,001	34,540,000	130.0	0.000079	0.000101
chr04	34,850,001	34,950,000	100.0	0.000079	0.000079
chr04	35,360,001	35,460,000	100.0	0.000110	0.000110
chr05	2,820,001	2,950,000	130.0	0.000115	0.000120
chr05	3,180,001	3,320,000	140.0	0.000084	0.000116
chr05	15,330,001	15,450,000	120.0	0.000069	0.000078
chr05	25,560,001	25,670,000	110.0	0.000109	0.000115
chr05	28,180,001	28,320,000	140.0	0.000091	0.000094
chr06	2,110,001	2,390,000	280.0	0.000045	0.000112
chr06	18,250,001	18,380,000	130.0	0.000043	0.000085
chr06	18,600,001	18,730,000	130.0	0.000083	0.000114
chr06	20,970,001	21,070,000	100.0	0.000106	0.000106
chr06	24,090,001	24,200,000	110.0	0.000088	0.000090
chr06	25,770,001	25,900,000	130.0	0.000081	0.000111
chr06	27,670,001	27,770,000	100.0	0.000036	0.000036
chr06	29,170,001	29,470,000	300.0	0.000010	0.000117
chr06	29,900,001	30,020,000	120.0	0.000090	0.000094
chr06	30,090,001	30,310,000	220.0	0.000067	0.000120
chr07	1,270,001	1,440,000	170.0	0.000018	0.000110
chr07	2,980,001	3,110,000	130.0	0.000074	0.000098
chr07	12,120,001	12,280,000	160.0	0.000002	0.000004
chr07	22,590,001	22,700,000	110.0	0.000057	0.000059
chr07	22,790,001	22,900,000	110.0	0.000091	0.000099
chr07	23,050,001	23,180,000	130.0	0.000084	0.000097
chr07	29,120,001	29,330,000	210.0	0.000090	0.000119
chr07	29,670,001	29,780,000	110.0	0.000005	0.000025
chr08	1,320,001	1,450,000	130.0	0.000023	0.000052
chr08	3,610,001	3,710,000	100.0	0.000090	0.000090
chr08	11,140,001	11,250,000	110.0	0.000087	0.000089
chr08	13,160,001	13,350,000	190.0	0.000039	0.000089
chr08	16,490,001	16,590,000	100.0	0.000097	0.000097
chr08	22,930,001	23,060,000	130.0	0.000101	0.000115
chr08	23,560,001	23,680,000	120.0	0.000107	0.000112

chr08	24,030,001	24,130,000	100.0	0.000107	0.000107
chr08	24,370,001	24,470,000	100.0	0.000102	0.000102
chr08	28,440,001	28,540,000	100.0	0.000011	0.000011
chr09	2,750,001	2,870,000	120.0	0.000011	0.000041
chr09	19,960,001	20,130,000	170.0	0.000094	0.000115
chr09	22,410,001	22,620,000	210.0	0.000041	0.000114
chr09	22,660,001	22,820,000	160.0	0.000054	0.000105
chr09	22,940,001	23,040,000	100.0	0.000071	0.000071
chr10	4,090,001	4,190,000	100.0	0.000116	0.000116
chr10	10,790,001	10,920,000	130.0	0.000000	0.000000
chr10	14,260,001	14,390,000	130.0	0.000040	0.000060
chr10	15,260,001	15,370,000	110.0	0.000104	0.000116
chr10	17,710,001	17,830,000	120.0	0.000102	0.000108
chr10	19,410,001	19,530,000	120.0	0.000057	0.000067
chr10	21,210,001	21,360,000	150.0	0.000100	0.000116
chr10	23,190,001	23,300,000	110.0	0.000017	0.000061
chr11	12,080,001	12,290,000	210.0	0.000021	0.000054
chr11	12,300,001	12,420,000	120.0	0.000006	0.000019
chr11	29,010,001	29,110,000	100.0	0.000067	0.000067
chr12	490,001	590,000	100.0	0.000101	0.000101
chr12	5,130,001	5,320,000	190.0	0.000044	0.000113
chr12	16,720,001	16,860,000	140.0	0.000047	0.000099
chr12	20,410,001	20,720,000	310.0	0.000003	0.000115
chr12	27,510,001	27,630,000	120.0	0.000027	0.000111
**HHZ pedigree**					
chr01	4,180,001	4,280,000	100.0	0.000098	0.000098
chr01	7,240,001	7,370,000	130.0	0.000047	0.000098
chr01	7,400,001	7,520,000	120.0	0.000091	0.000097
chr01	8,340,001	8,450,000	110.0	0.000106	0.000113
chr01	12,990,001	13,090,000	100.0	0.000031	0.000031
chr01	16,750,001	16,920,000	170.0	0.000040	0.000096
chr01	26,470,001	26,630,000	160.0	0.000082	0.000122
chr01	26,770,001	26,890,000	120.0	0.000103	0.000110
chr01	26,920,001	27,040,000	120.0	0.000104	0.000122
chr01	27,260,001	27,390,000	130.0	0.000106	0.000107
chr01	38,810,001	38,910,000	100.0	0.000124	0.000124
chr01	39,920,001	40,060,000	140.0	0.000069	0.000122
chr01	40,210,001	40,350,000	140.0	0.000110	0.000119
chr01	43,260,001	43,360,000	100.0	0.000062	0.000062
chr02	3,470,001	3,570,000	100.0	0.000105	0.000105
chr02	9,270,001	9,380,000	110.0	0.000081	0.000112
chr02	11,890,001	11,990,000	100.0	0.000115	0.000115
chr02	13,610,001	13,760,000	150.0	0.000014	0.000092
chr02	23,270,001	23,390,000	120.0	0.000072	0.000121
chr02	27,510,001	27,610,000	100.0	0.000121	0.000121
chr02	32,490,001	32,590,000	100.0	0.000111	0.000111
chr03	590,001	780,000	190.0	0.000056	0.000115
chr03	1,380,001	1,650,000	270.0	0.000052	0.000121
chr03	2,820,001	2,940,000	120.0	0.000102	0.000116
chr03	4,310,001	4,410,000	100.0	0.000116	0.000116
chr03	10,800,001	10,920,000	120.0	0.000117	0.000122
chr03	33,550,001	33,650,000	100.0	0.000082	0.000082
chr03	33,690,001	33,790,000	100.0	0.000081	0.000081
chr04	9,090,001	9,230,000	140.0	0.000001	0.000097
chr04	15,550,001	15,860,000	310.0	0.000002	0.000082
chr04	20,620,001	20,820,000	200.0	0.000122	0.000122
chr04	21,320,001	21,450,000	130.0	0.000102	0.000118
chr04	26,470,001	26,680,000	210.0	0.000049	0.000111
chr04	28,360,001	28,460,000	100.0	0.000056	0.000056
chr04	28,520,001	28,700,000	180.0	0.000080	0.000098
chr04	29,310,001	29,410,000	100.0	0.000091	0.000091
chr04	32,670,001	32,780,000	110.0	0.000114	0.000121
chr04	34,110,001	34,380,000	270.0	0.000035	0.000123
chr04	34,390,001	34,530,000	140.0	0.000088	0.000124
chr04	35,160,001	35,280,000	120.0	0.000102	0.000122
chr04	35,290,001	35,460,000	170.0	0.000073	0.000106
chr05	2,820,001	2,950,000	130.0	0.000113	0.000124
chr05	15,330,001	15,430,000	100.0	0.000124	0.000124
chr06	2,000,001	2,100,000	100.0	0.000099	0.000099
chr06	2,110,001	2,290,000	180.0	0.000077	0.000114
chr06	18,250,001	18,380,000	130.0	0.000053	0.000093
chr06	18,610,001	18,730,000	120.0	0.000084	0.000114
chr06	24,100,001	24,200,000	100.0	0.000119	0.000119
chr06	25,770,001	25,880,000	110.0	0.000082	0.000118
chr06	26,330,001	26,450,000	120.0	0.000099	0.000118
chr06	28,090,001	28,240,000	150.0	0.000072	0.000124

chr06	29,220,001	29,360,000	140.0	0.000013	0.000094
chr06	29,730,001	29,830,000	100.0	0.000109	0.000109
chr06	29,870,001	30,020,000	150.0	0.000071	0.000124
chr06	30,090,001	30,350,000	260.0	0.000042	0.000115
chr06	31,240,001	31,340,000	100.0	0.000000	0.000000
chr07	1,320,001	1,430,000	110.0	0.000057	0.000057
chr07	2,980,001	3,140,000	160.0	0.000035	0.000119
chr07	3,760,001	3,880,000	120.0	0.000108	0.000118
chr07	4,490,001	4,590,000	100.0	0.000114	0.000114
chr07	12,120,001	12,280,000	160.0	0.000001	0.000009
chr07	22,590,001	22,700,000	110.0	0.000044	0.000047
chr07	22,790,001	22,900,000	110.0	0.000104	0.000117
chr07	23,050,001	23,230,000	180.0	0.000006	0.000069
chr07	23,450,001	23,570,000	120.0	0.000092	0.000097
chr07	23,840,001	23,960,000	120.0	0.000093	0.000098
chr07	29,670,001	29,780,000	110.0	0.000008	0.000022
chr08	1,320,001	1,450,000	130.0	0.000068	0.000099
chr08	1,650,001	1,760,000	110.0	0.000118	0.000122
chr08	11,140,001	11,250,000	110.0	0.000069	0.000095
chr08	13,160,001	13,350,000	190.0	0.000042	0.000082
chr08	23,560,001	23,670,000	110.0	0.000122	0.000122
chr08	24,030,001	24,130,000	100.0	0.000123	0.000123
chr08	24,170,001	24,280,000	110.0	0.000092	0.000111
chr08	26,610,001	26,710,000	100.0	0.000069	0.000069
chr08	27,940,001	28,040,000	100.0	0.000100	0.000100
chr08	28,430,001	28,530,000	100.0	0.000101	0.000101
chr09	2,750,001	2,880,000	130.0	0.000003	0.000105
chr09	17,910,001	18,020,000	110.0	0.000084	0.000118
chr09	22,660,001	22,820,000	160.0	0.000058	0.000105
chr09	22,940,001	23,040,000	100.0	0.000041	0.000041
chr10	10,790,001	10,930,000	140.0	0.000001	0.000091
chr10	14,260,001	14,390,000	130.0	0.000058	0.000111
chr10	15,260,001	15,360,000	100.0	0.000117	0.000117
chr10	17,710,001	17,830,000	120.0	0.000075	0.000113
chr10	19,410,001	19,530,000	120.0	0.000078	0.000093
chr10	19,610,001	19,770,000	160.0	0.000100	0.000116
chr10	20,850,001	20,950,000	100.0	0.000120	0.000120
chr10	21,210,001	21,370,000	160.0	0.000098	0.000121
chr10	23,170,001	23,280,000	110.0	0.000064	0.000118
chr11	1,270,001	1,370,000	100.0	0.000100	0.000100
chr11	12,090,001	12,280,000	190.0	0.000118	0.000124
chr11	12,300,001	12,420,000	120.0	0.000002	0.000007
chr11	12,450,001	12,550,000	100.0	0.000001	0.000001
chr11	28,520,001	28,620,000	100.0	0.000094	0.000094
chr11	29,010,001	29,110,000	100.0	0.000080	0.000080
chr12	5,140,001	5,320,000	180.0	0.000048	0.000122
chr12	16,720,001	16,850,000	130.0	0.000030	0.000075
chr12	18,930,001	19,040,000	110.0	0.000035	0.000066
chr12	20,410,001	20,720,000	310.0	0.000013	0.000119
chr12	25,940,001	26,040,000	100.0	0.000105	0.000105
chr12	27,510,001	27,630,000	120.0	0.000012	0.000082
**SH527 pedigree**					
chr01	3,580,001	3,780,000	200.0	0.000043	0.000088
chr01	4,170,001	4,280,000	110.0	0.000042	0.000092
chr01	4,370,001	4,520,000	150.0	0.000064	0.000085
chr01	4,840,001	4,960,000	120.0	0.000094	0.000094
chr01	4,980,001	5,120,000	140.0	0.000069	0.000092
chr01	7,250,001	7,520,000	270.0	0.000045	0.000091
chr01	12,990,001	13,090,000	100.0	0.000051	0.000051
chr01	16,750,001	16,920,000	170.0	0.000014	0.000058
chr01	21,210,001	21,310,000	100.0	0.000084	0.000084
chr01	26,770,001	26,890,000	120.0	0.000067	0.000076
chr01	26,910,001	27,150,000	240.0	0.000062	0.000094
chr01	27,660,001	27,780,000	120.0	0.000063	0.000074
chr01	29,230,001	29,340,000	110.0	0.000091	0.000092
chr01	29,980,001	30,140,000	160.0	0.000047	0.000094
chr01	34,610,001	34,750,000	140.0	0.000035	0.000079
chr01	34,820,001	34,930,000	110.0	0.000033	0.000043
chr01	35,830,001	35,940,000	110.0	0.000082	0.000097
chr01	37,570,001	37,700,000	130.0	0.000059	0.000069
chr01	38,810,001	38,930,000	120.0	0.000054	0.000088
chr01	39,830,001	40,020,000	190.0	0.000086	0.000097
chr01	40,210,001	40,360,000	150.0	0.000053	0.000093
chr01	40,610,001	40,800,000	190.0	0.000034	0.000045
chr01	41,410,001	41,510,000	100.0	0.000077	0.000077
chr01	43,260,001	43,360,000	100.0	0.000046	0.000046

chr02	1,590,001	1,690,000	100.0	0.000095	0.000095
chr02	3,470,001	3,730,000	260.0	0.000070	0.000092
chr02	6,930,001	7,030,000	100.0	0.000076	0.000076
chr02	13,610,001	13,750,000	140.0	0.000038	0.000082
chr02	21,230,001	21,370,000	140.0	0.000083	0.000096
chr02	22,850,001	22,960,000	110.0	0.000040	0.000049
chr02	23,280,001	23,380,000	100.0	0.000090	0.000090
chr02	32,490,001	32,590,000	100.0	0.000076	0.000076
chr03	1,380,001	1,520,000	140.0	0.000058	0.000087
chr03	1,530,001	1,650,000	120.0	0.000047	0.000076
chr03	2,820,001	2,950,000	130.0	0.000054	0.000082
chr03	4,260,001	4,410,000	150.0	0.000044	0.000076
chr03	24,130,001	24,270,000	140.0	0.000091	0.000094
chr03	33,230,001	33,340,000	110.0	0.000064	0.000079
chr03	33,380,001	33,650,000	270.0	0.000023	0.000075
chr03	33,680,001	33,800,000	120.0	0.000034	0.000066
chr03	34,560,001	34,690,000	130.0	0.000077	0.000083
chr04	9,080,001	9,230,000	150.0	0.000079	0.000088
chr04	15,580,001	15,850,000	270.0	0.000009	0.000047
chr04	26,340,001	26,680,000	340.0	0.000020	0.000097
chr04	27,180,001	27,330,000	150.0	0.000060	0.000077
chr04	28,550,001	28,700,000	150.0	0.000061	0.000087
chr04	29,450,001	29,550,000	100.0	0.000068	0.000068
chr04	30,330,001	30,450,000	120.0	0.000031	0.000092
chr04	34,150,001	34,330,000	180.0	0.000035	0.000089
chr04	34,340,001	34,550,000	210.0	0.000039	0.000092
chr04	35,300,001	35,460,000	160.0	0.000058	0.000091
chr05	2,820,001	2,940,000	120.0	0.000076	0.000077
chr05	13,020,001	13,120,000	100.0	0.000085	0.000085
chr06	3,920,001	4,020,000	100.0	0.000095	0.000095
chr06	18,270,001	18,380,000	110.0	0.000051	0.000055
chr06	24,090,001	24,200,000	110.0	0.000072	0.000085
chr06	25,770,001	25,880,000	110.0	0.000095	0.000095
chr06	28,140,001	28,240,000	100.0	0.000061	0.000061
chr06	28,320,001	28,430,000	110.0	0.000058	0.000091
chr06	29,900,001	30,020,000	120.0	0.000043	0.000064
chr06	30,180,001	30,340,000	160.0	0.000041	0.000097
chr06	31,200,001	31,340,000	140.0	0.000015	0.000095
chr07	1,310,001	1,430,000	120.0	0.000080	0.000091
chr07	2,380,001	2,480,000	100.0	0.000096	0.000096
chr07	2,600,001	2,710,000	110.0	0.000081	0.000081
chr07	2,980,001	3,150,000	170.0	0.000055	0.000069
chr07	3,740,001	3,930,000	190.0	0.000042	0.000094
chr07	12,120,001	12,280,000	160.0	0.000000	0.000005
chr07	19,810,001	19,910,000	100.0	0.000091	0.000091
chr07	29,680,001	29,790,000	110.0	0.000000	0.000074
chr08	1,330,001	1,450,000	120.0	0.000072	0.000087
chr08	13,160,001	13,350,000	190.0	0.000058	0.000069
chr08	25,800,001	25,920,000	120.0	0.000048	0.000083
chr08	26,610,001	26,710,000	100.0	0.000073	0.000073
chr08	28,440,001	28,540,000	100.0	0.000007	0.000007
chr09	2,740,001	2,870,000	130.0	0.000003	0.000084
chr09	15,480,001	15,590,000	110.0	0.000080	0.000097
chr09	22,660,001	22,800,000	140.0	0.000063	0.000095
chr09	22,940,001	23,040,000	100.0	0.000053	0.000053
chr10	4,090,001	4,190,000	100.0	0.000079	0.000079
chr10	14,260,001	14,390,000	130.0	0.000045	0.000096
chr10	21,200,001	21,370,000	170.0	0.000057	0.000086
chr10	21,880,001	21,990,000	110.0	0.000083	0.000090
chr10	22,000,001	22,120,000	120.0	0.000054	0.000093
chr10	23,170,001	23,280,000	110.0	0.000037	0.000083
chr11	1,270,001	1,470,000	200.0	0.000033	0.000067
chr11	12,080,001	12,290,000	210.0	0.000075	0.000090
chr11	12,300,001	12,420,000	120.0	0.000001	0.000010
chr11	12,450,001	12,550,000	100.0	0.000001	0.000001
chr12	3,930,001	4,040,000	110.0	0.000071	0.000088
chr12	5,180,001	5,320,000	140.0	0.000030	0.000084
chr12	16,720,001	16,850,000	130.0	0.000033	0.000083
chr12	20,410,001	20,720,000	310.0	0.000009	0.000078
chr12	25,260,001	25,360,000	100.0	0.000084	0.000084
chr12	25,940,001	26,040,000	100.0	0.000077	0.000077
chr12	27,510,001	27,630,000	120.0	0.000003	0.000033

Pedigree-based genome re-sequencing reveals genetic variation patterns of elite backbone varieties during modern rice improvement

Xingfei Zheng, Lanzhi Li, Fan Liang, Changjun Tan, Shuzhu Tang, Sibin Yu, Ying Diao, Shuangcheng Li,and Zhongli Hu

Supplementary Table S12 Gene lists identified from low diversity chromosome segments in three pedigrees

Gene ID	Nr annotation	Transcript evidence	ORF evidence	InterPro annotaition
**GC2H pedigree**				
Os01g0179800	TMS membrane protein/tumour differentially expressed protein	AK069734	B6SVV5 (UniProt)	TMS membrane protein/tumour differentially expressed protein
Os01g0179901	Conserved hypothetical protein.	AK242152	NP_001172204.1 (RefSeq)	-
Os01g0180000	Pistil-specific extensin-like protein family protein.	AK103199	NP_001150408.1 (RefSeq)	Leucine-rich repeat
Os01g0180050	Hypothetical protein.	BT068200	longestORF	-
Os01g0180300	Lipoprotein, type 6 family protein.	AK120377	B9ETH2 (UniProt)	-
Os01g0180400	Protein of unknown function DUF581 domain containing protein.	AK108226	B9ETH3 (UniProt)	Protein of unknown function DUF581
Os01g0180500	Non-protein coding transcript.	EU973947	NONE	-
Os01g0180600	Similar to MutS homolog 7 (Fragment).	AK122068	Q8RVT1 (UniProt)	DNA mismatch repair protein MutS, C-terminal
Os01g0180700	Hypothetical conserved gene.	AK240913	Q9AQU6 (UniProt)	-
Os01g0180800	Heat shock protein Hsp70 family protein.	AK100676	B6U237 (UniProt)	Heat shock protein Hsp70
Os01g0180850	Hypothetical protein.	tplb0021a19 (Wheat FLcDNA)	longestORF	-
Os01g0180900	Conserved hypothetical protein.	AK357358	NP_001172205.1 (RefSeq)	-
Os01g0181000	Frigida-like domain containing protein.	EU948578	Q5VR39 (UniProt)	Frigida-like
Os01g0181033	Similar to NADH dehydrogenase subunit 1.	AJ010976	YP_024343.1 (RefSeq)	NADH:ubiquinone oxidoreductase, subunit 1/F420H2 oxidoreductase subunit H
Os01g0181166	Hypothetical gene.	CU405586	longestORF	-
Os01g0233800	Similar to Viroid symptom modulation protein.	AK069983	Q41297 (UniProt)	Protein kinase, catalytic domain
Os01g0233850	Hypothetical gene.	EU943630	longestORF	-
Os01g0233900	Similar to cDNA clone:J013003E06, full insert sequence.	AK100714	B7EAV1 (UniProt)	Ubiquitin-conjugating enzyme, E2
Os01g0234001	Hypothetical conserved gene.	AK241763	B9G6H6 (UniProt)	-
Os01g0234100	Transcriptional factor B3 family protein.	AK106332	Q0JP99 (UniProt)	Transcriptional factor B3
Os01g0234200	Similar to DNA topoisomerase 2.	ab initio prediction	A2WMH7 (UniProt)	DNA topoisomerase, type IIA, subunit B/N-terminal
Os01g0234300	Similar to Pectinesterase.	ab initio prediction	B9EUJ6 (UniProt)	Pectinesterase, catalytic
Os01g0234433	Hypothetical protein.	EU943103	longestORF	-
Os01g0234499	Similar to Pectinesterase.	ab initio prediction	B9EUJ6 (UniProt)	Pectinesterase, catalytic
Os01g0234566	Non-protein coding transcript.	X06283	NONE	-
Os01g0234700	Harpin-induced 1 domain containing protein.	AK108936	NP_001147348.1 (RefSeq)	-
Os01g0234800	Similar to 25.3 kDa vesicle transport protein.	AK103163	B6TMY9 (UniProt)	Longin
Os01g0234850	Hypothetical conserved gene.	AK242318	NP_001172247.1 (RefSeq)	Phosphatidylinositol 3-/4-kinase, catalytic
Os01g0234900	Similar to Ubiquitin ligase SINAT5 (EC 6.3.2.-) (Seven in absentia homolog 5). Splice isoform 2.	AK120816	B8Q8B9 (UniProt)	Zinc finger, RING-type
Os01g0235100	Similar to predicted protein.	AK287587	Q5NAX5 (UniProt)	-
Os01g0235200	Conserved hypothetical protein.	AK103537	B9EUK0 (UniProt)	-
Os01g0235300	SOUL haem-binding protein domain containing protein.	AK059862	B6SR01 (UniProt)	SOUL haem-binding protein
Os01g0235325	Hypothetical protein.	EU943029	longestORF	-
Os01g0235350	Conserved hypothetical protein.	ab initio prediction	B9EUK1 (UniProt)	-
Os01g0235400	Similar to predicted protein.	AK064696	Q9ZPY7 (UniProt)	Importin-beta, N-terminal
Os01g0235500	Conserved hypothetical protein.	AK121404	A2WMJ1 (UniProt)	-
Os01g0235632	Hypothetical protein.	tplb0028d05 (Wheat FLcDNA)	longestORF	-
Os01g0235700	Similar to BHLH transcription factor (Fragment).	AK064943	Q5IWM1 (UniProt)	Helix-loop-helix DNA-binding
Os01g0235800	K Homology domain containing protein.	AK058731	B9EUK2 (UniProt)	K Homology
Os01g0235850	Hypothetical protein.	EU974948	longestORF	-
Os01g0235900	Thioredoxin fold domain containing protein.	ab initio prediction	NP_001042526.1 (RefSeq)	Glutaredoxin
Os01g0236000	Conserved hypothetical protein.	AK065569	B8AB33 (UniProt)	-
Os01g0252100	Similar to Glycogen synthase kinase-3 homolog MSK-3 (EC [illegible]	AK099742	Q7GC12 (UniProt)	Protein kinase, catalytic domain
Os01g0252150	Hypothetical protein.	tplb0034j24 (Wheat FLcDNA)	longestORF	-
Os01g0252200	Zinc finger, CCCH-type domain containing protein.	AK071420	B8ABS8 (UniProt)	Zinc finger, CCCH-type
Os01g0252300	Conserved hypothetical protein.	AK242398	NP_001172259.1 (RefSeq)	-
Os01g0252400	Hypothetical gene.	AK063327	longestORF	-
Os01g0252600	Similar to predicted protein.	CT835001	B8ABT0 (UniProt)	-
Os01g0252900	Zinc finger, CCHC-type domain containing protein.	AK105250	XP_002878450.1 (RefSeq)	Zinc finger, CCHC-type
Os01g0253000	Similar to LpimPth3.	AK071644	Q5NBC2 (UniProt)	Protein kinase, catalytic domain
Os01g0253050	Conserved hypothetical protein.	EU957207	NP_001042608.1 (RefSeq)	-
Os01g0253100	Similar to Avr9/Cf-9 induced kinase 1.	AK067133	B9EV01 (UniProt)	Protein kinase, catalytic domain
Os01g0253200	Similar to pectinesterase inhibitor domain containing protein.	tplb0011f19 (Wheat FLcDNA)	NP_001148594.1 (RefSeq)	Pectinesterase inhibitor
Os01g0253300	Importin alpha-1a subunit.	AK068233	B6T451 (UniProt)	Armadillo
Os01g0253400	Protein of unknown function DUF1218 family protein.	AK108904	Q9S809 (UniProt)	Protein of unknown function DUF1218
Os01g0253500	Conserved hypothetical protein.	AK243648	NP_001172261.1 (RefSeq)	-
Os01g0253600	Replication factor A protein 3 domain containing protein.	AK058837	NP_001152271.1 (RefSeq)	Nucleic acid-binding, OB-fold
Os01g0253800	Conserved hypothetical protein.	ab initio prediction	NP_001172262.1 (RefSeq)	-
Os01g0253900	Similar to triacylglycerol lipase.	AK072113	NP_001148192.1 (RefSeq)	Lipase, class 3
Os01g0254000	Similar to NTGB2 (Fragment).	AK111904 ,AK111979 ,AK11954[illegible]	B4FPT6 (UniProt)	Small GTP-binding protein domain
Os01g0254100	Similar to CTV.2.	AK111762	Q2HW32 (UniProt)	WD40 repeat
Os01g0254200	Conserved hypothetical protein.	AK121256	A2WMZ3 (UniProt)	-
Os01g0254300	Pectinesterase (EC 3.1.1.11) (Fragment).	EU974973	B4F9U3 (UniProt)	Pectinesterase, catalytic
Os01g0254350	Hypothetical gene.	BT085732	longestORF	-
Os01g0558500	PWWP domain containing protein.	AK099982	A2WRD3 (UniProt)	PWWP
Os01g0558600	Ras-related protein RIC1.	AK243597	P40392 (UniProt)	Small GTPase superfamily
Os01g0558700	Conserved hypothetical protein.	AK287514	NP_001172419.1 (RefSeq)	-
Os01g0558800	Similar to oxidoreductase/ transition metal ion binding protein.	AK068120	XP_002869432.1 (RefSeq)	Protein of unknown function DUF3531
Os01g0558825	Hypothetical protein.	AF207545	longestORF	-
Os01g0558850	Similar to peptidase M16 family protein / insulinase family [illegible]	BT037467	XP_002866163.1 (RefSeq)	Peptidase M16, zinc-binding site
Os01g0558900	Conserved hypothetical protein.	AK058447	Q5JKR1 (UniProt)	-
Os01g0559000	Protein of unknown function DUF1000 family protein.	AK061456	B6T8X4 (UniProt)	Galactose-binding domain-like
Os01g0559100	Similar to Seryl-tRNA synthetase (EC 6.1.1.11) (Serine--tRNA ligase) (SerRS) (Fragment).	AK105730	B4FRC4 (UniProt)	Aminoacyl-tRNA synthetase, class II
Os01g0559150	Hypothetical protein.	tplb0033a05 (Wheat FLcDNA)	longestORF	-
Os01g0559200	Phosphorylated adapter RNA export protein, RNA-binding domain domain containing protein.	AK102611	B9EXJ7 (UniProt)	Phosphorylated adapter RNA export protein, RNA-binding domain
Os01g0559300	Conserved hypothetical protein.	AK064666	A2WRD9 (UniProt)	-
Os01g0559500	Similar to predicted protein.	AK288159	Q5JKQ7 (UniProt)	Pentatricopeptide repeat
Os01g0559525	Non-protein coding transcript.	tplb0046j15 (Wheat FLcDNA)	NONE	-
Os01g0559550	Non-protein coding transcript.	CT834992	NONE"	-
Os01g0559600	Similar to C13 endopeptidase NP1 precursor.	AK067597	Q9SSZ4 (UniProt)	Peptidase C13, legumain
Os01g0559750	Hypothetical gene.	AK241989	GeneMark	-
Os01g0559825	Hypothetical protein.	EU942443	longestORF	-
Os01g0559900	Non-protein coding transcript.	AK243203	NONE	-
Os01g0560000	Similar to Auxin amidohydrolase.	AK105974	Q8S9S4 (UniProt)	Peptidase M20
Os01g0560200	Similar to Vesicle transport v-SNARE 13 (AtVTI13) (Vesicle transport v-SNARE protein VTI13) (Vesicle soluble NSF attachment protein receptor 13).	AK102003	NP_001147199.1 (RefSeq)	Vesicle transport v-SNARE, N-terminal
Os01g0564300	Peptidyl-prolyl cis-trans isomerase, FKBP-type domain containing protein.	AK064825	B9EXK9 (UniProt)	Peptidyl-prolyl cis-trans isomerase, FKBP-type, domain
Os01g0564400	Conserved hypothetical protein.	AK068728	NP_001044210.1 (RefSeq)	-
Os01g0564532	Hypothetical gene.	AK241021	longestORF	-
Os01g0564600	Protein of unknown function DUF247, plant family protein.	ab initio prediction	NP_001172424.1 (RefSeq)	Protein of unknown function DUF247, plant
Os01g0654100	CTP synthase domain containing protein.	AK111300	Q6AUM6 (UniProt)	CTP synthase
Os01g0654150	Non-protein coding transcript.	tplb0056h06 (Wheat FLcDNA)	NONE	-
Os01g0654200	Peptidase M50 domain containing protein.	AK100750	B9EYD8 (UniProt)	Peptidase M50
Os01g0654300	Similar to ARP2/3 complex 34 kDa subunit.	AK058593	B6TA29 (UniProt)	Arp2/3 complex, 34kDa subunit p34-Arc
Os01g0654400	Similar to Salt tolerant correlative protein.	AK288103	E6Y2L2 (UniProt)	-
Os01g0654450	Hypothetical gene.	EU946002	longestORF	-
Os01g0654500	Similar to NADP-isocitrate dehydrogenase.	AK061752	Q9XGU8 (UniProt)	Isocitrate dehydrogenase NADP-dependent, eukaryotic-type
Os01g0654650	Non-protein coding transcript.	BT016882	NONE	-
Os01g0654800	Conserved hypothetical protein.	AK359861	B9EYE1 (UniProt)	-
Os01g0654900	Hypothetical conserved gene.	ab initio prediction	NP_001172487.1 (RefSeq)	-
Os01g0655250	PWWP domain containing protein.	ab initio prediction	B9EYE3 (UniProt)	PWWP
Os01g0655300	Similar to Trithorax 4 (Fragment).	AK062870	B3VSN3 (UniProt)	SET domain
Os01g0655400	Conserved hypothetical protein.	AK068171	B8A6V1 (UniProt)	-
Os01g0655500	Protein kinase, core domain containing protein.	AK120660	NP_001147834.1 (RefSeq)	Protein kinase, catalytic domain
Os02g0263432	Conserved hypothetical protein.	ab initio prediction	B9F4W3 (UniProt)	-
Os02g0433600	Helix-loop-helix DNA-binding domain containing protein.	AK069790 ,AK103986	B6UE48 (UniProt)	Helix-loop-helix DNA-binding
Os02g0474300	Similar to DNA topoisomerase.	AK122152	B9EZZ0 (UniProt)	DNA topoisomerase, type IA, central region, subdomain 3
Os02g0474700	Armadillo-like helical domain containing protein.	AK068629	Q01KY9 (UniProt)	Armadillo-like helical
Os02g0475101	Similar to RING-H2 finger protein ATL5A.	EU953643	NP_001151367.1 (RefSeq)	Zinc finger, RING-type
Os02g0475300	Membrane attack complex component/perforin/complement C9 family protein.	AK120516	NP_001148060.1 (RefSeq)	Membrane attack complex component/perforin
Os02g0475400	Bile acid:sodium symporter family protein.	AK121008	XP_002885704.1 (RefSeq)	Bile acid:sodium symporter
Os02g0598500	Protein phosphatase 2C family protein.	AK106903	NP_001047333.1 (RefSeq)	Protein phosphatase 2C, manganese/magnesium aspartate binding site

Os02g0598600	Cyclin-like F-box domain containing protein.	AK105564	Q6K1T5 (UniProt)	F-box domain, cyclin-like
Os02g0598800	Telomere length regulation protein, conserved domain domain containing protein.	AK372629	NP_001047336.2 (RefSeq)	Telomere length regulation protein, conserved domain
Os02g0598900	Cyclin-like F-box domain containing protein.	AK105564	Q6K1T5 (UniProt)	F-box domain, cyclin-like
Os02g0599100	Hypothetical conserved gene.	tplb0009a09 (Wheat FLcDNA)	Q6K1U1 (UniProt)	Telomere length regulation protein, conserved domain
Os02g0599150	Hypothetical conserved gene.	AK106903	Q6K1U4 (UniProt)	Protein phosphatase 2C, manganese/magnesium aspartate binding site
Os02g0599151	Probable protein phosphatase 2C 17.	Q6K1U0 (UniProt)	Q6K1U0 (UniProt)	Protein phosphatase 2C, manganese/magnesium aspartate binding site
Os03g0109700	Similar to predicted protein.	AK069551	Q94JY0 (UniProt)	-
Os03g0109800	Hypothetical conserved gene.	AK066015	Q8H7V3 (UniProt)	-
Os03g0109900	Peptidase, trypsin-like serine and cysteine domain containing	AK065011	Q8H7V4 (UniProt)	Peptidase cysteine/serine, trypsin-like
Os03g0110300	Conserved hypothetical protein.	AK106206 ,AK066190 ,AK06676	B9FAD8 (UniProt)	-
Os03g0110400	Similar to nuclear matrix protein-related.	AK100395	NP_568219.1 (RefSeq)	THO complex, subunit THOC1
Os03g0110500	KIP1-like domain containing protein.	AK101157	NP_001149784.1 (RefSeq)	KIP1-like
Os03g0110600	Similar to LysM domain containing protein, expressed.	AK356815	Q10SU7 (UniProt)"	-
Os03g0110700	Hypothetical protein.	tplb0029n18 (Wheat FLcDNA)	longestORF	-
Os03g0110800	Similar to DNA methyltransferase.	AK065147	B6UD67 (UniProt)	C-5 cytosine methyltransferase
Os03g0110900	Dimeric alpha-beta barrel domain containing protein.	AK070403	Q10SU2 (UniProt)	Dimeric alpha-beta barrel
Os03g0111000	Conserved hypothetical protein.	AK062759	B8ALG1 (UniProt)	-
Os03g0111100	Similar to Dihydrofolate synthetase /folylpolyglutamate	AK102025	B6SW32 (UniProt)	Folylpolyglutamate synthetase
Os03g0111200	Similar to Remorin.	AK060379	B6U3W6 (UniProt)	Remorin, C-terminal
Os03g0111300	Nonspecific lipid-transfer protein 2 (nsLTP2) (7 kDa lipid transfer protein).	AK062506	Q10ST8 (UniProt)	Plant lipid transfer protein/hydrophobic protein, helical domain
Os03g0111400	Heavy metal transport/detoxification protein domain containing protein.	AK243276	Q01II0 (UniProt)	Heavy metal-associated domain, HMA
Os03g0111500	Hypothetical conserved gene.	AK067241	Q10ST5 (UniProt)	-
Os03g0111600	Protein of unknown function DUF1618 domain containing	AK101020	A3ADD8 (UniProt)	Domain of unknown function DUF1618
Os03g0111700	Similar to TWN2 (TWIN 2)%3B ATP binding / aminoacyl-tRNA ligase/ nucleotide binding / valine-tRNA ligase.	AK066973	NP_172913.1 (RefSeq)	-
Os03g0111800	WD40/YVTN repeat-like domain containing protein.	AK121627	B9FAE6 (UniProt)	WD40 repeat
Os03g0112101	Similar to Adaptin N terminal region family protein, expressed.	CT835180	Q10SS6 (UniProt)	Clathrin/coatomer adaptor, adaptin-like, N-terminal
Os03g0112400	Clathrin/coatomer adaptor, adaptin-like, appendage, C-terminal subdomain domain containing protein.	AK063082	Q10SS6 (UniProt)	Clathrin adaptor, alpha-adaptin, appendage, C-terminal subdomain
Os03g0112600	Similar to Plus-3 domain containing protein, expressed.	EU942983	Q7XCX5 (UniProt)	SWIB/MDM2 domain
Os03g0112700	Zinc finger, CCCH-type domain containing protein.	AK112095	Q10SS3 (UniProt)	Zinc finger, CCCH-type
Os03g0112800	Protein of unknown function DUF726 family protein.	AK100572	A2XBP3 (UniProt)	Protein of unknown function DUF726
Os03g0112900	Similar to Aldehyde 5-hydroxylase.	AB207253	B6SWS3 (UniProt)	Cytochrome P450
Os03g0112950	Non-protein coding transcript.	AK242233	NONE	-
Os03g0113000	Serine/threonine protein kinase domain containing protein.	AK071309	NP_001151054.1 (RefSeq)	Protein kinase, catalytic domain
Os03g0113100	Similar to Thymidine kinase.	AK066124	O81263 (UniProt)	Thymidine kinase
Os03g0113200	Protein of unknown function DUF295 family protein.	AK109767	Q10SR6 (UniProt)	Protein of unknown function DUF295
Os03g0113500	Conserved hypothetical protein.	AK241800	B9FAE9 (UniProt)	-
Os03g0113700	Similar to Heat shock 70 kDa protein, mitochondrial precursor.	AK103835	NP_001151739.1 (RefSeq)	Heat shock protein Hsp70
Os03g0113750	Hypothetical protein.	tplb0023b01 (Wheat FLcDNA)	longestORF	-
Os03g0113800	Tetratricopeptide-like helical domain containing protein.	AK065925	XP_002870606.1 (RefSeq)	Tetratricopeptide-like helical
Os03g0124000	Homeodomain-related domain containing protein.	AK121207	B6SZ15 (UniProt)	Homeobox
Os03g0124033	Hypothetical protein.	tplb0025a22 (Wheat FLcDNA)	longestORF	-
Os03g0124100	Protein of unknown function DUF604 domain containing protein.	AK107007	Q01KI0 (UniProt)	Protein of unknown function DUF604
Os03g0124200	Similar to Pto-like protein kinase F.	AK102524	A2XBX8 (UniProt)	Protein kinase, catalytic domain
Os03g0124300	Similar to ATP binding protein.	AK106076	B6TWY5 (UniProt)	Malectin-like carbohydrate-binding domain
Os03g0124500	Conserved hypothetical protein.	AK119960	B8ALZ1 (UniProt)	-
Os03g0124800	Non-protein coding transcript.	AK121064	NONE	-
Os03g0124900	Pectin lyase fold/virulence factor domain containing protein.	AK070284	NP_001151102.1 (RefSeq)	Glycoside hydrolase, family 28
Os03g0125000	Similar to 50S ribosomal protein L5, chloroplast.	AK070779	Q9ZST0 (UniProt)	Ribosomal protein L5
Os03g0125100	Beta-carotene hydroxylase, Drought and oxidative stress	AK287823	Q10SE7 (UniProt)	-
Os03g0125300	D111/G-patch domain containing protein.	AK121790 ,AK065004	B6TBI8 (UniProt)	D111/G-patch
Os03g0125400	Similar to D-erythro-sphingosine kinase/ diacylglycerol kinase.	AK101342	B6TDW8 (UniProt)	Diacylglycerol kinase, catalytic domain
Os03g0125600	Ser Thr specific protein kinase-like protein.	AK243600	Q10SE3 (UniProt)	Protein kinase, catalytic domain
Os03g0125650	Hypothetical gene.	AK289045	longestORF	-
Os03g0125700	Ubiquitin ligase, Det1/DDB1-complexing domain containing	AK062913	B8ALZ5 (UniProt)	Ubiquitin ligase, Det1/DDB1-complexing
Os03g0125800	Cystathionine beta-synthase, core domain containing protein.	AK102496 ,AK100164	B6SVJ7 (UniProt)	Domain of unknown function DUF21
Os03g0125900	Conserved hypothetical protein.	AK060370	A2XBY9 (UniProt)	-
Os03g0126000	Similar to Phosphorybosyl anthranilate transferase 1.	AK121680	NP_001130955.1 (RefSeq)	Glycosyl transferase, family 3
Os03g0126100	Similar to Arabidopsis thaliana DNA, 40 kb surrounding ACS1	AK111921	NP_001151564.1 (RefSeq)	D-isomer specific 2-hydroxyacid dehydrogenase, NAD-binding
Os03g0126300	Similar to cDNA clone:001-042-G03, full insert sequence.	AK061256	Q6V9S3 (UniProt)	-
Os03g0126450	Domain of unknown function DUF623 domain containing protein.	EU952385	Q8S5W2 (UniProt)	Domain of unknown function DUF623
Os03g0126600	Conserved hypothetical protein.	AK243575	Q8S5W1 (UniProt)	-
Os03g0126700	Similar to Barley stem rust resistance protein.	AK099929	Q2QSQ6 (UniProt)	Heavy metal-associated domain, HMA
Os03g0126800	Similar to CBL-interacting protein kinase 9.	FJ901199	Q10SC8 (UniProt)	Protein kinase, catalytic domain
Os03g0126825	Hypothetical gene.	AK242859	longestORF	-
Os03g0126850	Conserved hypothetical protein.	AK241174	NP_001173247.1 (RefSeq)	-
Os03g0126900	Conserved hypothetical protein.	AK109217	A2XBZ9 (UniProt)	-
Os03g0127000	Similar to cell growth defect factor 2.	AK068479	NP_001148819.1 (RefSeq)	-
Os03g0127100	Hypothetical gene.	AK120780	longestORF	-
Os03g0127500	bZIP transcription factor, bZIP-1 domain containing protein.	EU971509	NP_001151643.1 (RefSeq)	Basic-leucine zipper
Os03g0127600	Forkhead-associated domain containing protein.	AK103695	Q8S5V4 (UniProt)	Forkhead-associated
Os03g0127650	Non-protein coding transcript.	BT017530	NONE	-
Os03g0127700	Serine/threonine protein kinase-related domain containing protein.	AK101081	Q9LQ11 (UniProt)	Protein kinase, catalytic domain
Os03g0127800	Non-protein coding transcript.	EU947022	NONE	-
Os03g0127900	Cation/H+ exchanger domain containing protein.	AK062977	NP_001147290.1 (RefSeq)	Cation/H+ exchanger
Os03g0127950	Similar to inner membrane protein ybaL.	CT835134	NP_001147290.1 (RefSeq)	-
Os03g0128000	FAS1 domain domain containing protein.	AK121246	Q06IA4 (UniProt)	FAS1 domain
Os03g0151000	Conserved hypothetical protein.	AF216531	Q5NAS3 (UniProt)"	-
Os03g0151100	Appr-1-p processing domain containing protein.	AK099979	NP_001152046.1 (RefSeq)	Appr-1-p processing
Os03g0151201	Non-protein coding transcript.	AK288979	NONE	-
Os03g0151300	Similar to JmjC domain containing protein, expressed.	AK110927	Q10RP5 (UniProt)	Transcription factor jumonji/aspartyl beta-hydroxylase
Os03g0151400	Similar to Zinc finger, C2H2 type family protein, expressed.	AK110807	Q10RP4 (UniProt)	-
Os03g0151500	Conserved hypothetical protein.	AK109181	A3AE69 (UniProt)	-
Os03g0151600	Acyl-CoA N-acyltransferase domain containing protein.	AK066845	B6T916 (UniProt)	GCN5-related N-acetyltransferase
Os03g0151700	Similar to WD repeat protein (Fragment).	AK101270	NP_188791.3 (RefSeq)	WD40 repeat
Os03g0151800	Similar to Cell division control protein 48 homolog A	AK121641 ,AK064955 ,AK09887	Q2HZ34 (UniProt)	-
Os03g0151850	Hypothetical gene.	AK373447	longestORF	-
Os03g0151900	Similar to Small GTP-binding protein.	AK121527	Q9FPK1 (UniProt)	Small GTPase superfamily
Os03g0152000	Heavy metal transport/detoxification protein domain containing protein.	AK102357	A2XCL8 (UniProt)	Heavy metal-associated domain, HMA
Os03g0152100	Similar to E2F-DP transcription factor.	AK242229	B4FHH8 (UniProt)	Transcription factor E2F/dimerisation partner
Os03g0152200	Non-protein coding transcript.	BT009415	NONE	-
Os03g0152300	Haem peroxidase family protein.	AK070875	A2XCM0 (UniProt)	Plant peroxidase
Os03g0152400	Similar to 4-coumarate--CoA ligase-like 1.	AK242087	Q0DV32 (UniProt)	AMP-dependent synthetase/ligase
Os03g0152500	Hypothetical protein.	BT062457	longestORF	-
Os03g0152600	Conserved hypothetical protein.	AK109525	B8AND6 (UniProt)	-
Os03g0152700	Pseudouridine synthase domain containing protein.	AK067387	B6U7U0 (UniProt)	RNA-binding S4
Os03g0152800	Conserved hypothetical protein.	AK066205	B9FBD7 (UniProt)	-
Os03g0152900	Similar to predicted protein.	AK288414	XP_002876276.1 (RefSeq)	Armadillo
Os03g0152950	Non-protein coding transcript.	BT085237	NONE	-
Os03g0153000	Similar to cDNA clone:001-019-C07, full insert sequence.	CT835172	Q337U3 (UniProt)	RNA recognition motif domain
Os03g0153100	Similar to FAD binding domain containing protein, expressed.	BT054188	Q10RM1 (UniProt)	Monooxygenase, FAD-binding
Os03g0171300	Similar to DNA-binding protein-like.	AK069569	NP_849712.1 (RefSeq)	Helix-loop-helix DNA-binding
Os03g0171600	Kelch-type beta propeller domain containing protein.	AK072716	NP_001148653.1 (RefSeq)	Kelch repeat type 1
Os03g0171700	Basic helix-loop-helix dimerisation region bHLH domain containing protein.	AK241674	NP_001149761.1 (RefSeq)	Helix-loop-helix DNA-binding
Os03g0171900	Similar to Alanine:glyoxylate aminotransferase-like protein	AK105642	D2KZ10 (UniProt)	Aminotransferase class-III
Os03g0171950	Non-protein coding transcript.	BT083955	NONE	-
Os03g0172000	Peptidase S59, nucleoporin family protein.	AK122024	NP_178183.2 (RefSeq)	Peptidase S59, nucleoporin
Os03g0172100	Similar to Leucine zipper protein.	AK121297	P93427 (UniProt)	-
Os03g0172200	Mo25-like domain containing protein.	AK069130	NP_001151860.1 (RefSeq)	Armadillo-like helical
Os03g0172400	Proteinase inhibitor I25, cystatin, conserved region domain containing protein.	ab initio prediction	NP_001049119.1 (RefSeq)	Proteinase inhibitor I25, cystatin, conserved region
Os03g0172700	Conserved hypothetical protein.	AK111307	A2XD26 (UniProt)	-
Os03g0172850	Proteinase inhibitor I25, cystatin, conserved region domain containing protein.	EU961152	B9FBK7 (UniProt)	Proteinase inhibitor I25, cystatin, conserved region
Os03g0173500	Similar to Kinase associated protein phosphatase.	AK099854	O81444 (UniProt)	-
Os03g0181100	Tify domain containing protein.	AK120087	NP_001149525.1 (RefSeq)	Tify
Os03g0181400	Similar to ubiquitin thiolesterase.	AK064006	NP_680185.1 (RefSeq)	Plant organelle RNA recognition domain
Os03g0181500	Similar to Fiddlehead protein.	AK061335 ,AK060840 ,AK09923	B9F5I5 (UniProt)	Very-long-chain 3-ketoacyl-CoA synthase

Os03g0181550	Hypothetical protein.	tplb0055l20 (Wheat FLcDNA)	longestORF	-
Os03g0181600	Similar to cDNA clone:J033068D01, full insert sequence.	AK101197	B8A2M6 (UniProt)	Zinc finger, GATA-type
Os03g0181675	ABC transporter, transmembrane domain domain containing	ab initio prediction	B8APN9 (UniProt)	ABC transporter, transmembrane domain
Os03g0181750	ABC transporter, transmembrane domain domain containing	BT066367	Q8H7L0 (UniProt)	ABC transporter, transmembrane domain
Os03g0181800	Protein of unknown function DUF936, plant family protein.	AK070399	A2XD85 (UniProt)	Protein of unknown function DUF936, plant
Os03g0182000	Similar to flavin-dependent monooxygenase 1.	BT040713	XP_002893036.1 (RefSeq)	Flavin monooxygenase-like
Os03g0182350	Non-protein coding transcript.	AK241509	NONE	-
Os03g0182400	Similar to SAC domain protein 1 (FIG4-like protein AtFIG4).	AK100037	Q10QV0 (UniProt)	Synaptojanin, N-terminal
Os03g0182600	Ribosomal protein S2 family protein.	AK061289	Q10QU9 (UniProt)	Ribosomal protein S2
Os03g0182700	Eukaryotic translation initiation factor 3 subunit 12 (eIF-3 p25) (eIF3k).	AK073293 ,AK120235	Q94HF1 (UniProt)	-
Os03g0182800	Similar to Ethylene responsive element binding factor3 [illegible]	AK073133	Q8VXC3 (UniProt)	Pathogenesis-related transcriptional factor/ERF, DNA-binding
Os03g0182900	Non-protein coding transcript.	AK103663	NONE	-
Os03g0183000	Similar to AP2 domain containing protein RAP2.6 (Fragment).	AK060527	Q94HF3 (UniProt)	Pathogenesis-related transcriptional factor/ERF, DNA-binding
Os03g0183050	Hypothetical gene.	EU941438	longestORF	-
Os03g0183100	SAP-like protein BP-73 (OsBP-73) (Riaa1).	AK065516	Q8L4E7 (UniProt)	Rho termination factor, N-terminal
Os03g0183200	Similar to AP2 domain containing protein, expressed.	AK106987	Q10QU3 (UniProt)	-
Os03g0183300	Pathogenesis-related transcriptional factor and ERF domain containing protein.	AK106163 ,AK105922 ,AK11955	Q5MFV0 (UniProt)	Pathogenesis-related transcriptional factor/ERF, DNA-binding
Os03g0183500	Protein of unknown function DUF581 family protein.	AK063042	NP_197570.1 (RefSeq)	Protein of unknown function DUF581
Os03g0183600	Similar to Alanine aminotransferase.	Z26322	Q9S768 (UniProt)	1-aminocyclopropane-1-carboxylate synthase
Os03g0183800	Similar to Leucine-rich repeat transmembrane protein kinase 1 (Fragment).	AK100621	O81105 (UniProt)	Protein kinase, catalytic domain
Os03g0183850	Hypothetical protein.	tplb0047d04 (Wheat FLcDNA)	longestORF	-
Os03g0183900	Similar to Plasma membrane H+-ATPase.	AK242894	Q8RW26 (UniProt)	ATPase, P-type, H+ transporting proton pump
Os03g0183950	Hypothetical protein.	tplb0039e05 (Wheat FLcDNA)	longestORF	-
Os03g0194450	Hypothetical gene.	BT019071	longestORF	-
Os03g0194500	Similar to Protein translocase/ protein transporter.	AK242198	B6T215 (UniProt)	Mitochondrial inner membrane translocase subunit Tim17/Tim22/Tim23/peroxisomal protein PMP24
Os03g0194600	Protein of unknown function DUF568, DOMON-like domain containing protein.	AK062839	NP_001152720.1 (RefSeq)	DOMON domain
Os03g0194900	Uncharacterised conserved protein UCP037471 domain containing protein.	AK104041 ,AK070149	NP_001151876.1 (RefSeq)	Cytochrome b561, eukaryote
Os03g0195100	Similar to AGD2 (ABERRANT GROWTH AND DEATH 2)%3B transaminase.	CT828078	NP_567934.1 (RefSeq)	Diaminopimelate aminotransferase, DapL, plant/Chlamydia-type
Os03g0195200	Protein of unknown function DUF367 domain containing protein.	AK068949	Q10QI5 (UniProt)	Domain of unknown function DUF367
Os03g0195300	Similar to Low affinity sulphate transporter 3.	AK067353	XP_002302276.1 (RefSeq)	Sulphate anion transporter
Os03g0195350	Non-protein coding transcript.	tplb0023b09 (Wheat FLcDNA)	NONE	-
Os03g0195400	Hypothetical gene.	AK064567	longestORF	-
Os03g0195450	Similar to sulfate/bicarbonate/oxalate exchanger and transporter	CT835280	D4IIA9 (UniProt)	Sulphate transporter/antisigma-factor antagonist STAS
Os03g0195475	Hypothetical protein.	tplb0023b09 (Wheat FLcDNA)	longestORF	-
Os03g0195500	Similar to Sulfate permease (Fragment).	AK111395	XP_002302276.1 (RefSeq)	Sulphate transporter
Os03g0195800	Similar to Sulfate transporter (Fragment).	AK103007	A7YF68 (UniProt)	Sulphate anion transporter
Os03g0195900	Hypothetical protein.	AK102458	longestORF	-
Os03g0196000	Similar to Sulfate transporter.	ab initio prediction	A7YF68 (UniProt)	Sulphate anion transporter
Os03g0196100	Hypothetical protein.	AK107004	longestORF	-
Os03g0203200	Alpha/beta hydrolase fold-1 domain containing protein.	AK070827	B6TRW7 (UniProt)	-
Os03g0203451	Non-protein coding transcript.	CT835161	NONE"	-
Os03g0203700	Similar to Calcium-transporting ATPase 2, plasma membrane-type (EC 3.6.3.8) (Ca(2+)-ATPase isoform 2).	AK100415	Q8L8A0 (UniProt)	ATPase, P-type, H+ transporting proton pump
Os03g0203750	Non-protein coding transcript.	EU971904	NONE	-
Os03g0203800	Cyclin D domain containing protein.	AK070370	Q10QA2 (UniProt)	Cyclin, C-terminal
Os03g0204100	Actin-binding FH2 domain containing protein.	AK121042	Q10Q99 (UniProt)	Actin-binding FH2/DRF autoregulatory
Os03g0204300	Conserved hypothetical protein.	AK243647	NP_001173306.1 (RefSeq)	-
Os03g0204366	Conserved hypothetical protein.	ab initio prediction	NP_001172855.1 (RefSeq)	-
Os03g0204432	Non-protein coding transcript.	AF190302	NONE	-
Os03g0204500	Conserved hypothetical protein.	ab initio prediction	NP_001173307.1 (RefSeq)	-
Os03g0204600	Conserved hypothetical protein.	AK369551	A2ZQ28 (UniProt)	-
Os03g0204900	Conserved hypothetical protein.	AK062601	A3AF92 (UniProt)	-
Os03g0205000	Ubiquitin system component Cue domain containing protein.	AK069442	B8AQF4 (UniProt)	Ubiquitin system component Cue
Os03g0205150	Hypothetical conserved gene.	AK241595 ,AK241577	B9F623 (UniProt)	-
Os03g0205300	Similar to Helix-loop-helix DNA-binding domain containing	ab initio prediction	Q10Q90 (UniProt)	Helix-loop-helix DNA-binding
Os03g0205400	Similar to XPG I-region family protein, expressed.	AK376826	Q10Q89 (UniProt)	Xeroderma pigmentosum group G protein
Os03g0205500	Similar to fiber protein Fb38.	AK287752	NP_001152719.1 (RefSeq)	Cytochrome b5
Os03g0205700	BTB domain containing protein.	AK103674	NP_001151545.1 (RefSeq)	BTB/POZ-like
Os03g0205800	Similar to Acetyltransferase, GNAT family protein, expressed.	ab initio prediction	Q10Q85 (UniProt)	GCN5-related N-acetyltransferase
Os03g0206100	Conserved hypothetical protein.	AK242567	NP_001049317.2 (RefSeq)	-
Os03g0206150	Hypothetical protein.	tplb0054f13 (Wheat FLcDNA)	longestORF	-
Os03g0206201	Similar to predicted protein.	EU953990	B9F630 (UniProt)	-
Os03g0206300	Similar to augmenter of liver regeneration.	AK102625	NP_001148317.1 (RefSeq)	Erv1/Alr
Os03g0206400	Conserved hypothetical protein.	AK107011	A2XDQ7 (UniProt)	-
Os03g0206600	Uncharacterised protein family UPF0497, trans-membrane plant domain containing protein.	AK058618	F2EIJ0 (UniProt)	Uncharacterised protein family UPF0497, trans-membrane plant
Os03g0206700	BTB/POZ fold domain containing protein.	BT053887	NP_001152299.1 (RefSeq)	BTB/POZ-like
Os03g0282232	Conserved hypothetical protein.	ab initio prediction	B9F7D7 (UniProt)	-
Os03g0282300	Hypothetical conserved gene.	BT038365	B9F7D7 (UniProt)	-
Os03g0282700	DEAD-like helicase, N-terminal domain containing protein.	ab initio prediction	Q8H8U9 (UniProt)	Helicase, C-terminal
Os03g0282800	Conserved hypothetical protein.	AK101658	B8ALI3 (UniProt)	-
Os03g0282900	Conserved hypothetical protein.	AK102161 ,AK061166	B8ALI4 (UniProt)	-
Os03g0283000	Similar to In2-1 protein.	AY332468	Q8H8U5 (UniProt)	Glutathione S-transferase, N-terminal
Os03g0283100	Similar to In2-1 protein.	AK065887	Q8H8U5 (UniProt)	Glutathione S-transferase, N-terminal
Os03g0283200	Similar to IN2-1 protein.	AF237487	NP_001105433.1 (RefSeq)	Glutathione S-transferase, N-terminal
Os03g0283300	Conserved hypothetical protein.	AK070169	Q10N42 (UniProt)	-
Os03g0283400	Conserved hypothetical protein.	AK064670	B9F7E1 (UniProt)	-
Os03g0283500	Pentatricopeptide repeat domain containing protein.	AK103244	B8ALI6 (UniProt)	Pentatricopeptide repeat
Os03g0283600	CDP-alcohol phosphatidyltransferase domain containing protein.	AK070093	B6SU69 (UniProt)	CDP-alcohol phosphatidyltransferase
Os03g0283650	Hypothetical protein.	ab initio prediction	NONE	-
Os03g0283750	Conserved hypothetical protein.	AK241622	NP_001173371.1 (RefSeq)	-
Os03g0283800	Similar to TBC domain containing protein, expressed.	AK072879	Q10N37 (UniProt)	-
Os03g0283850	Hypothetical protein.	tplb0061a18 (Wheat FLcDNA)	longestORF	-
Os03g0283900	Similar to Serine/threonine-protein kinase PBS1 (EC 2.7.1.37) (AvrPphB susceptible protein 1).	AK105569	Q32SF8 (UniProt)	Protein kinase, catalytic domain
Os03g0284000	Hypothetical conserved gene.	EU970944	B8ALI9 (UniProt)	Uncharacterised protein family UPF0405
Os03g0284100	Similar to Two-component response regulator-like PRR73.	AK121136	Q10N34 (UniProt)	Signal transduction response regulator, receiver domain
Os03g0284150	Hypothetical protein.	tplb0030a07 (Wheat FLcDNA)	longestORF	-
Os03g0284200	Non-protein coding transcript.	AK062789	NONE	-
Os03g0304800	Lg106-like family protein.	AK062656	NP_001148326.1 (RefSeq)	Endosulphine
Os03g0305000	AMP-binding protein.	AK243161	Q10ML0 (UniProt)	AMP-dependent synthetase/ligase
Os03g0305050	Hypothetical gene.	EU941562	longestORF	-
Os03g0305100	Similar to AMP-binding protein.	AK101765	B6SS27 (UniProt)	AMP-dependent synthetase/ligase
Os03g0305150	Hypothetical protein.	EU941562	longestORF	-
Os03g0305200	Conserved hypothetical protein.	AK108450	A2XFT0 (UniProt)	-
Os03g0305400	UspA domain containing protein.	AK071397	Q10MK6 (UniProt)	Universal stress protein A
Os03g0305500	Fumarate lyase domain containing protein.	AK070638	B4FAV4 (UniProt)	Fumarate lyase
Os03g0305550	Hypothetical gene.	EU949424	longestORF	-
Os03g0305600	Mitochondrial import inner membrane translocase, subunit Tim17/22 family protein.	AK063714	Q2HU23 (UniProt)	Mitochondrial inner membrane translocase subunit Tim17/Tim22/Tim23/peroxisomal protein PMP24
Os03g0305700	Similar to Peptide chain release factor 2 (Fragment).	AK073807	NP_001148129.1 (RefSeq)	Peptide chain release factor class I/class II
Os03g0305800	Galactosyl transferase family protein.	AK105101 ,AK105005	A2XFT5 (UniProt)	-
Os03g0305950	Similar to Xyloglucan 6-xylosyltransferase (EC 2.4.2.39)	AK241375	longestORF	-
Os03g0306100	Galactosyl transferase family protein.	AK335833	NP_001151451.1 (RefSeq)	Galactosyl transferase
Os03g0306200	Similar to transducin family protein / WD-40 repeat family	AK241828	XP_002865733.1 (RefSeq)	Bromodomain
Os03g0306302	Hypothetical conserved gene.	DQ245981	Q10MJ9 (UniProt)	-
Os03g0306400	Similar to Lon protease homolog, mitochondrial.	AK288546	A2YQ56 (UniProt)	-
Os03g0306700	Conserved hypothetical protein.	AK109929	A3AH67 (UniProt)	-
Os03g0306800	Similar to CP12 (Fragment).	AK103722	B7XB92 (UniProt)	Domain of unknown function CP12
Os03g0306900	Haem oxygenase-like, multi-helical domain containing protein.	AK073626	B6TPF2 (UniProt)	TENA/THI-4 protein/Coenzyme PQQ biosynthesis protein C domain
Os03g0307000	Conserved hypothetical protein.	AK243201	NP_001049892.2 (RefSeq)	-
Os03g0307100	Peptidase S9A, oligopeptidase, N-terminal beta-propeller domain containing protein.	AK102205	XP_002882118.1 (RefSeq)	Peptidase S9, prolyl oligopeptidase, catalytic domain
Os03g0307200	Similar to Nicotianamine synthase 2.	AK112011	Q10MI9 (UniProt)	Nicotianamine synthase
Os03g0307300	Nicotianamine synthase 1 (EC 2.5.1.43) (S-adenosyl-L-methionine:S- adenosyl-L-methionine:S-adenosyl-methionine 3-amino-3- carboxypropyltransferase 1) (OsNAS1).	AK112069	Q0DSH9 (UniProt)	Nicotianamine synthase

Os03g0680600	Ankyrin repeat domain containing protein.	ab initio prediction	B9FAL0 (UniProt)	Ankyrin repeat
Os03g0680700	Similar to Knotted1-interacting protein.	EU964245	NP_001105184.1 (RefSeq)	Homeobox
Os03g0680800	Similar to cDNA clone:J023038C04, full insert sequence.	AK070543	Q9AYD9 (UniProt)	Homeobox
Os03g0681000	Protein of unknown function DUF819 domain containing protein.	BT061349	B8API7 (UniProt)	Protein of unknown function DUF819
Os03g0681201	Similar to predicted protein.	CT835232	NP_176121.2 (RefSeq)"	-
Os03g0681300	Similar to SABRE.	AK062837	Q10F47 (UniProt)	-
Os03g0681400	Similar to Ubiquitin-conjugating enzyme E2-18 kDa (EC 6.3.2.19) (Ubiquitin- conjugating enzyme 15) (Ubiquitin-protein ligase) (Ubiquitin carrier protein) (PM42).	AK103809	B9HFA8 (UniProt)	Ubiquitin-conjugating enzyme, E2
Os03g0681700	Similar to Yarrowia lipolytica chromosome E of strain CLIB99 of Yarrowia lipolytica.	AK111767	B4F9U1 (UniProt)	WD40 repeat
Os03g0681750	Hypothetical gene.	CT835257	longestORF	-
Os03g0681800	Protein phosphatase 2A, regulatory B subunit, B56 domain containing protein.	EU957243	NP_001148001.1 (RefSeq)	Protein phosphatase 2A, regulatory B subunit, B56
Os03g0681900	Similar to RGP-3 (Fragment).	AK061750 ,AK062094	B8APJ2 (UniProt)	RNA recognition motif domain
Os03g0682100	Similar to nitrate and chloride transporter.	AK359238	NP_001152028.1 (RefSeq)	Nodulin-like
Os03g0682200	Similar to Protein argonaute 12.	BT069625	Q7Y001 (UniProt)	Stem cell self-renewal protein Piwi
Os03g0706500	TCP family transcription factor, Negative regulator for lateral branching	AK107083	Q8LN68 (UniProt)	Transcription factor, TCP
Os03g0706650	Hypothetical protein.	tplb0055g09 (Wheat FLcDNA)	longestORF	-
Os03g0706900	Zinc finger, RING-type domain containing protein.	AK065293	NP_001149034.1 (RefSeq)	Zinc finger, RING-type
Os03g0707200	Similar to Integral membrane protein DUF6 containing protein, expressed.	ab initio prediction	Q10E65 (UniProt)	Drug/metabolite transporter
Os03g0707250	Non-protein coding transcript.	AK242476	NONE	-
Os03g0707300	Conserved hypothetical protein.	AK066944	Q10E63 (UniProt)	-
Os03g0707600	OsGAI.	AK242577	Q18ND8 (UniProt)	Transcription factor GRAS
Os03g0707900	Similar to Toc64.	AK067052 ,AK098901	B6UCR6 (UniProt)	Amidase
Os03g0708000	Phospholipase A2 family protein.	AK105828	Q10E50 (UniProt)	Phospholipase A2, eukaryotic
Os03g0708100	Phytanoyl-CoA dioxygenase family protein.	AK103821	NP_565262.1 (RefSeq)	Phytanoyl-CoA dioxygenase
Os03g0708200	Cyclin-like F-box domain containing protein.	AK287707	NP_001173607.1 (RefSeq)	Protein of unknown function DUF295
Os03g0708250	Conserved hypothetical protein.	CT837770	NP_001173938.1 (RefSeq)"	-
Os03g0708400	Protein of unknown function DUF1295 family protein.	AK066216	NP_565068.1 (RefSeq)	3-oxo-5-alpha-steroid 4-dehydrogenase, C-terminal
Os03g0708500	Capsid/spike protein, ssDNA virus domain containing protein.	CT835099	NP_001150378.1 (RefSeq)	Peptidyl-prolyl cis-trans isomerase, FKBP-type, domain
Os03g0708600	DEAD-like helicase, N-terminal domain containing protein.	AK069658	B7EG14 (UniProt)	RNA helicase, ATP-dependent, DEAD-box, conserved site
Os03g0710550	Conserved hypothetical protein.	AK288133	NP_001173611.1 (RefSeq)	-
Os03g0710600	Conserved hypothetical protein.	AK068746	A2XL91 (UniProt)	-
Os03g0710700	Similar to predicted protein.	CT835290	NP_195677.2 (RefSeq)	Glycolipid transfer protein domain
Os03g0710800	14-3-3-like protein S94.	AK062077 ,AK103065	Q06967 (UniProt)	14-3-3 protein
Os03g0710850	Non-protein coding transcript.	BT017754	NONE	-
Os03g0710900	Similar to CCAAT displacement protein-related / CDP-related.	AK369948	NP_566611.1 (RefSeq)	CASP, C-terminal
Os03g0711100	Similar to CONSTANS-like protein.	AK120563	NP_001149548.1 (RefSeq)	Zinc finger, B-box
Os03g0711150	Hypothetical protein.	tplb0030k24 (Wheat FLcDNA)	longestORF	-
Os03g0711200	Similar to Phosphatidylinositol 4-kinase.	AK063479	Q8L5D9 (UniProt)	Phosphatidylinositol 3-/4-kinase, catalytic
Os03g0711250	Hypothetical gene.	AK289048	longestORF	-
Os03g0711300	Serine/threonine protein kinase domain containing protein.	AK102350	C3TX65 (UniProt)	Protein kinase, catalytic domain
Os03g0711400	Similar to Coatomer alpha subunit.	AK100286	Q6RYF4 (UniProt)	WD40 repeat
Os03g0711425	Hypothetical protein.	tplb0039i16 (Wheat FLcDNA)	longestORF	-
Os03g0711500	Similar to cDNA clone:J023075G08, full insert sequence.	AK058652	B7EQ85 (UniProt)	Coatomer, WD associated region
Os03g0711525	Hypothetical protein.	tplb0039i16 (Wheat FLcDNA)	longestORF	-
Os03g0711600	Similar to DNA binding protein (Fragment).	AK288161	Q10E13 (UniProt)	Histone H1/H5
Os03g0711650	Similar to AT hook motif family protein, expressed.	AK242375	Q10E13 (UniProt)	-
Os03g0711700	Similar to Transcription factor S-II family protein, expressed.	CT835090	Q10E12 (UniProt)	Zinc finger, TFIIS-type
Os03g0711800	Similar to IRE homolog 1 (Fragment).	AK122108	Q9LVI5 (UniProt)	Protein kinase, catalytic domain
Os03g0712000	Similar to Lipase family protein.	ab initio prediction	Q10E06 (UniProt)	Lipase, class 3
Os03g0712100	Cell division cycle-associated protein domain containing protein.	AK106258	Q9AUR0 (UniProt)	Zinc-finger domain of monoamine-oxidase A repressor R1
Os03g0712200	Zinc finger, RanBP2-type domain containing protein.	AK073205 ,AK098968	Q9AUQ9 (UniProt)	Zinc finger, RanBP2-type
Os03g0712300	Similar to CROC-1-like protein (Fragment).	AK120951	B6T4J0 (UniProt)	Ubiquitin-conjugating enzyme, E2
Os03g0712400	Similar to atypical receptor-like kinase MARK.	AK111063	NP_001105207.1 (RefSeq)	Protein kinase, catalytic domain
Os03g0712500	Hypothetical protein.	BT086967	longestORF	-
Os03g0712600	Similar to F-box domain containing protein, expressed.	AK103705	Q10DR5 (UniProt)	-
Os03g0712700	Similar to Phosphoglucomutase, cytoplasmic 2 (EC 5.4.2.2) (Glucose phosphomutase 2) (PGM 2).	AK099746 ,AK072313	A5HSI1 (UniProt)	Alpha-D-phosphohexomutase superfamily
Os03g0712733	Hypothetical gene.	BT016600	longestORF	-
Os03g0712800	Similar to Glutamine synthetase root isozyme 2 (EC 6.3.1.2) (Glutamate--ammonia ligase).	AK063913 ,AK099290	Q4W8D0 (UniProt)	-
Os03g0712900	Pentatricopeptide repeat domain containing protein.	AK103803	Q10DZ7 (UniProt)	Pentatricopeptide repeat
Os03g0713000	Similar to Chloroplast threonine deaminase 1.	tplb0062e09 (Wheat FLcDNA)	A0FKE6 (UniProt)	Serine/threonine dehydratase, pyridoxal-phosphate-binding site
Os03g0713100	Similar to dynamin-related protein 1C.	AK243639	NP_001151519.1 (RefSeq)	Dynamin central domain
Os03g0713150	Hypothetical protein.	tplb0034e16 (Wheat FLcDNA)	longestORF	-
Os03g0718000	Similar to Anthranilate synthase beta chain.	AK105178	Q8L9I8 (UniProt)	Carbamoyl-phosphate synthase, GATase domain
Os03g0718100	Actin 1.	AK100267 ,AK058421	Q10DV7 (UniProt)	Actin-like
Os03g0718150	Hypothetical protein.	BT018641	longestORF	-
Os03g0718200	Zinc finger, SWIM-type domain containing protein.	AK063808	Q10DV6 (UniProt)	Zinc finger, PMZ-type
Os03g0718300	Hypothetical protein.	AK108509	longestORF	-
Os03g0718500	Homeodomain-related domain containing protein.	EU971875	NP_001151696.1 (RefSeq)	Homeobox domain, ZF-HD class
Os03g0718600	Cytochrome c oxidase assembly protein CtaG/Cox11 family	EF431929	C6YXJ9 (UniProt)	Cytochrome c oxidase assembly protein CtaG/Cox11
Os03g0718650	Hypothetical protein.	EU955192	longestORF	-
Os03g0718800	Similar to Physical impedance induced protein.	AK288124	NP_001149478.1 (RefSeq)	Plant lipid transfer protein/seed storage/trypsin-alpha amylase inhibitor
Os03g0719000	MAP65/ASE1 family protein.	AK108923	XP_002875099.1 (RefSeq)	Microtubule-associated protein, MAP65/ASE1-type
Os03g0719100	Zinc finger, RING/FYVE/PHD-type domain containing protein.	AK065127	Q6ASW7 (UniProt)	Zinc finger, PHD-type
Os03g0719150	Non-protein coding transcript.	tplb0051k01 (Wheat FLcDNA)	NONE	-
Os03g0719300	Similar to Dihydroxyacetone/glycerone kinase-like protein.	AK119254	NP_001148575.1 (RefSeq)	Dak kinase
Os03g0719400	Alpha/beta hydrolase fold-1 domain containing protein.	AK120822	Q8LLN8 (UniProt)	Alpha/beta hydrolase, N-terminal
Os03g0719450	Hypothetical gene.	EU946282	longestORF	-
Os03g0719500	Hypothetical conserved gene.	AK106321	Q6ASW0 (UniProt)	-
Os03g0719700	Similar to Phytochrome A.	AK065707	longestORF	-
Os03g0719800	Phytochrome A, Photoreceptor	AB109891 ,AK072482	Q10DU0 (UniProt)	-
Os03g0719850	Similar to Protein kinase 2.	BT038499	Q709M0 (UniProt)	Protein kinase, catalytic domain
Os03g0719900	Similar to Peptide transporter 1.	AK072691	A6YJX4 (UniProt)	Oligopeptide transporter
Os03g0720033	Non-protein coding transcript.	tplb0038k08 (Wheat FLcDNA)	NONE	-
Os03g0748200	Similar to Inhibitor of growth protein 3 (p47ING3 protein).	AK106146	Q10CW3 (UniProt)	Zinc finger, PHD-type
Os03g0748300	Aldose 1-epimerase family protein.	AK068519	Q8W307 (UniProt)	Glycoside hydrolase-type carbohydrate-binding
Os03g0748400	Serine protein kinase-like protein.	AK070433	Q8W306 (UniProt)	Protein kinase, catalytic domain
Os03g0748500	Similar to flavoprotein wrbA.	AK110695	NP_001147306.1 (RefSeq)	NADPH-dependent FMN reductase
Os03g0748550	Hypothetical protein.	BT017374	longestORF	-
Os03g0748600	Conserved hypothetical protein.	AK062629	Q8W304 (UniProt)	-
Os03g0748700	Iron hydrogenase domain containing protein.	AK067853	Q93YF9 (UniProt)	Iron hydrogenase, small subunit-like
Os03g0748750	Conserved hypothetical protein.	CT835159	NP_001060202.1 (RefSeq)"	-
Os03g0748800	Similar to Helicase associated domain family protein, expressed.	AK243482	Q10CV6 (UniProt)	Double-stranded RNA-binding
Os03g0748900	Aldo/keto reductase domain containing protein.	AK061165	NP_001148213.1 (RefSeq)	RNA recognition motif domain
Os03g0749000	Putative ammonium transporter 4 member 1.	Q10CV4 (UniProt)	Q10CV4 (UniProt)	Ammonium transporter
Os03g0749051	Hypothetical protein.	BT054429	longestORF	-
Os03g0749100	Similar to Beta-glucanase.	AK072485	NP_001105671.1 (RefSeq)	Glycoside hydrolase, family 3, N-terminal
Os03g0749200	Hypothetical protein.	tplb0060e05 (Wheat FLcDNA)	longestORF	-
Os03g0749300	Similar to Exoglucanase precursor.	AK065044	Q8RWR5 (UniProt)	Glycoside hydrolase, family 3, N-terminal
Os03g0749401	Hypothetical protein.	tplb0060e05 (Wheat FLcDNA)	longestORF	-
Os03g0749500	Similar to Exo-beta-glucanase.	AK073110	Q9XE93 (UniProt)	Glycoside hydrolase, family 3, N-terminal
Os03g0749650	Hypothetical protein.	tplb0060e05 (Wheat FLcDNA)	longestORF	-
Os03g0749800	Similar to Tousled-like protein kinase.	AK111770	Q5I6Y0 (UniProt)	Protein kinase, catalytic domain
Os03g0749900	N-6 adenine-specific DNA methylase, conserved site domain containing protein.	AK061252	NP_567065.1 (RefSeq)	DNA methylase, N-6 adenine-specific, conserved site
Os03g0750000	Similar to ethylene-responsive protein.	AK071321	NP_001151646.1 (RefSeq)	UspA
Os03g0750100	Similar to TPR Domain containing protein, expressed.	ab initio prediction	Q10CT3 (UniProt)	Tetratricopeptide-like helical
Os03g0750300	Conserved hypothetical protein.	EU947175	NP_001173642.1 (RefSeq)	-
Os03g0754300	Conserved hypothetical protein.	AK288070	Q6Z9T1 (UniProt)	-
Os03g0754500	Similar to COBRA-like protein 3.	ab initio prediction	Q60E70 (UniProt)	Glycosyl-phosphatidyl inositol-anchored, plant
Os03g0754800	Mitochondrial substrate carrier family protein.	AK101584	B6TV90 (UniProt)	Mitochondrial carrier protein
Os03g0754900	Similar to Cleavage stimulation factor, 50 kDa subunit.	AK101437	B4FBI9 (UniProt)	WD40 repeat
Os03g0755000	Similar to Serine/threonine kinase (Fragment).	AK068540	B3GK00 (UniProt)	Protein kinase, catalytic domain
Os03g0755100	Similar to Transporter associated with antigen processing-like	AK066049 ,AK240921 ,AK28766.	Q9FNU2 (UniProt)	ABC transporter, transmembrane domain
Os03g0755150	Non-protein coding transcript.	tplb0044a23 (Wheat FLcDNA)	NONE	-
Os03g0755600	Similar to 36I5.7.	ab initio prediction	NP_001173645.1 (RefSeq)	-
Os03g0755625	Hypothetical conserved gene.	tplb0013l17 (Wheat FLcDNA)	Q75J15 (UniProt)	-
Os03g0755700	Similar to Ribosomal protein L13a.	CT833550	Q5I7L1 (UniProt)	Ribosomal protein L13

Os03g0755800	Similar to 36I5.7.	AK330449	Q8LPX2 (UniProt)	NLI interacting factor
Os03g0755900	Similar to ABC transporter B family member 25.	ab initio prediction	Q9FNU2 (UniProt)	-
Os03g0756000	Similar to 60S ribosomal protein L13a-4.	AK243083 ,AK103893 ,AK10607	Q5I7L1 (UniProt)	Ribosomal protein L13, eukaryotic/archaeal
Os03g0756200	Protein kinase, core domain containing protein.	AK111668	A3AMU1 (UniProt)	Protein kinase, catalytic domain
Os03g0756300	X8 domain containing protein.	AK103836	NP_001152290.1 (RefSeq)	X8
Os03g0756400	Protein of unknown function, transmembrane-40 domain containing protein.	AK099399	A2XM49 (UniProt)	Uncharacterised protein family, transmembrane-40
Os03g0756500	Similar to PAF1 complex component.	AK373369	XP_002318586.1 (RefSeq)	Leo1-like protein
Os03g0756700	Hypothetical conserved gene.	AK318528	B9FBW4 (UniProt)	-
Os03g0756800	Uncharacterised protein family UPF0139 domain containing	AK120423	NP_001152924.1 (RefSeq)	Uncharacterised protein family UPF0139
Os03g0763000	Similar to Casein kinase II alpha subunit.	AK120812	Q8VX01 (UniProt)	Protein kinase, catalytic domain
Os03g0764000	Acyl-CoA N-acyltransferase domain containing protein.	CT831785	NP_195474.1 (RefSeq)	GCN5-related N-acetyltransferase
Os03g0764100	Zinc finger transcription factor ZF1.	AK072942	Q9AXE9 (UniProt)	Zinc finger, C2H2
Os03g0764125	Hypothetical protein.	ab initio prediction	NONE	-
Os03g0764150	Non-protein coding transcript.	AK243023	NONE	-
Os03g0764250	Hypothetical gene.	AK240671	longestORF	-
Os03g0764300	Serine/threonine protein kinase domain containing protein.	AK068541 ,AK103184	B6SVE4 (UniProt)	Protein kinase, catalytic domain
Os03g0764450	Similar to SWIB/MDM2 domain containing protein.	EU955355	B6SRJ0 (UniProt)	SWIB/MDM2 domain
Os03g0764500	Conserved hypothetical protein.	AK120443	Q8W5G1 (UniProt)	-
Os03g0764550	Hypothetical gene.	AK288256	GeneMark	-
Os03g0764600	Homeodomain-like containing protein.	AK105625	NP_001149103.1 (RefSeq)	Myb-like DNA-binding domain, SHAQKYF class
Os03g0764800	Similar to Serine/threonine-protein kinase SAPK8.	AK060479	Q7Y0B9 (UniProt)	Protein kinase, catalytic domain
Os03g0764900	Similar to Zn finger protein (Fragment).	AK063380	XP_002310753.1 (RefSeq)	Zinc finger, Dof-type
Os03g0765000	Similar to Serine/threonine-protein kinase 12 (EC 2.7.1.37) (Aurora-B) (Fragment).	AK073918	B6SI45 (UniProt)	Protein kinase, catalytic domain
Os03g0765050	Non-protein coding transcript.	BT019247	NONE	-
Os03g0765100	Conserved hypothetical protein.	AK062731	Q7Y0C7 (UniProt)	-
Os03g0765200	Similar to Signal peptidase I family protein, expressed.	AK058692	Q10EP0 (UniProt)	Peptidase S24/S26A/S26B/S26C, beta-ribbon domain
Os03g0765400	Similar to nucleoporin p58/p45.	AK100035	NP_001149260.1 (RefSeq)	-
Os03g0765500	Hypothetical conserved gene.	CT835163	Q7Y0B8 (UniProt)	-
Os03g0765800	Pyridoxal phosphate-dependent transferase, major region domain containing protein.	AK367148	NP_201496.1 (RefSeq)	Pyridoxal phosphate-dependent transferase, major region, subdomain 1
Os03g0765900	PetM of cytochrome b6/f complex subunit 7 domain containing protein.	AK058858	NP_001147411.1 (RefSeq)	PetM of cytochrome b6/f complex subunit 7
Os03g0766000	Similar to seven transmembrane domain protein.	AK070596 ,AK104743 ,AK10607	NP_001147233.1 (RefSeq)	Protein of unknown function DUF2053, membrane
Os03g0766100	10 kDa prolamin precursor.	AK108254 ,AK241987	A2XMB2 (UniProt)	Plant lipid transfer protein/seed storage/trypsin-alpha amylase inhibitor
Os03g0766200	Bifunctional inhibitor/plant lipid transfer protein/seed storage domain containing protein.	AK241901	A2XMB2 (UniProt)	Bifunctional inhibitor/plant lipid transfer protein/seed storage
Os03g0766350	Bifunctional inhibitor/plant lipid transfer protein/seed storage domain containing protein.	AK242216	A2XMB2 (UniProt)	Plant lipid transfer protein/seed storage/trypsin-alpha amylase inhibitor
Os03g0766500	Similar to Two-component response regulator ARR1. Splice isoform 2.	AK108408	NP_001119363.1 (RefSeq)	Myb-like DNA-binding domain, SHAQKYF class
Os03g0766600	Conserved hypothetical protein.	AK063832 ,AK121417	A2XMB7 (UniProt)	-
Os03g0766800	Conserved hypothetical protein.	AK120228	B8AKI9 (UniProt)	-
Os03g0766900	Allene oxide synthase.	AK103989 ,AK071361	B6TKL0 (UniProt)	Proteasome assembly chaperone 2, eukaryotic
Os03g0766950	Non-protein coding transcript.	AK361753	NONE	-
Os03g0767000	Similar to Allene oxide synthase (EC 4.2.1.92).	AK068620	Q7Y0C8 (UniProt)	Cytochrome P450
Os03g0832500	Nucleic acid-binding, OB-fold domain containing protein.	AK106890	B8AMR6 (UniProt)	Nucleic acid-binding, OB-fold
Os03g0832600	Similar to Galactokinase (EC 2.7.1.6) (Galactose kinase).	AK120137	B6TZ40 (UniProt)	Galactokinase
Os03g0832800	Similar to glycerol-3-phosphate acyltransferase 1.	AK064956	NP_001148243.1 (RefSeq)	-
Os03g0832850	Hypothetical protein.	tplb0038i08 (Wheat FLcDNA)	longestORF	-
Os03g0832900	Similar to DNAJ-like protein (Fragment).	AK060945	Q84VA4 (UniProt)	Heat shock protein DnaJ, N-terminal
Os03g0833100	Similar to Tasselseed2 protein (Fragment).	AK068662	Q2EFH2 (UniProt)	Short-chain dehydrogenase/reductase SDR
Os03g0833200	Methyltransferase TRM13 domain containing protein.	AK102870	B8AMR9 (UniProt)	Methyltransferase TRM13
Os03g0833300	Similar to Squamosa promoter-binding-like protein 6.	AK065741	Q75LH6 (UniProt)	Transcription factor, SBP-box
Os03g0833500	Hypothetical protein.	AK119356	longestORF	-
Os03g0833600	X8 domain containing protein.	AK109880	Q10B16 (UniProt)	X8
Os03g0833700	RNA 3'-terminal phosphate cyclase family protein.	AK121918	NP_680196.1 (RefSeq)	RNA 3&apos%3B-terminal phosphate cyclase
Os03g0833800	Pectin lyase fold/virulence factor domain containing protein.	AK066209	NP_001151479.1 (RefSeq)	Glycoside hydrolase, family 28
Os03g0833900	Similar to Cytosine deaminase (EC 3.5.4.1).	AK073655 ,AK104068	NP_001105963.1 (RefSeq)	-
Os03g0834000	Flap endonuclease-1b (EC 3.-.-.-) (OsFEN-1b).	AK062149	Q75LI2 (UniProt)	5'-3' exonuclease, N-terminal
Os03g0834050	Similar to predicted protein.	AK240756	B6TQ05 (UniProt)	HAD-superfamily hydrolase, subfamily IA, variant 3
Os03g0834150	Non-protein coding transcript.	X06284	NONE	-
Os03g0834300	Similar to GRAS family transcription factor containing protein, expressed.	AK241384	Q7XCD7 (UniProt)"	-
Os03g0834466	Similar to MADS-box transcription factor 21.	BT008965	D3U2H6 (UniProt)	Transcription factor, MADS-box
Os03g0857000	Hypothetical conserved gene.	AK120418	B8ANS1 (UniProt)	-
Os03g0857100	Hypothetical protein.	AK105192	longestORF	-
Os03g0857200	Conserved hypothetical protein.	AK287789	NP_001051954.1 (RefSeq)	-
Os03g0857301	Hypothetical protein.	ab initio prediction	NONE	-
Os03g0857400	tRNA-binding arm domain containing protein.	AK098951 ,AK062137	NP_849422.1 (RefSeq)	-
Os03g0857500	Protein of unknown function DUF303, acetylesterase putative domain containing protein.	AK072880	NP_001150914.1 (RefSeq)	Domain of unknown function DUF303, acetylesterase putative
Os03g0857600	Protein of unknown function DUF303, acetylesterase putative domain containing protein.	AK061073 ,AK100733	NP_001150914.1 (RefSeq)	Domain of unknown function DUF303, acetylesterase putative
Os03g0857750	Similar to predicted protein.	CT829136	NP_001173722.1 (RefSeq)	-
Os03g0857900	Similar to Lysine decarboxylase-like protein.	AK071705 ,AK061341	Q8L8B8 (UniProt)	Cytokinin riboside 5&apos%3B-monophosphate phosphoribohydrolase LOG
Os03g0858100	Similar to CRM1 protein (Fragment).	AK100745	D1MAF2 (UniProt)	Importin-beta, N-terminal
Os03g0858200	Conserved hypothetical protein.	AK110744	A2XPA5 (UniProt)	-
Os03g0858400	WD40 repeat-like domain containing protein.	AK102968	NP_567150.1 (RefSeq)	WD40 repeat
Os03g0858600	Protein of unknown function DUF668 family protein.	AK102241	Q84M75 (UniProt)	Protein of unknown function DUF668
Os03g0858700	Protein of unknown function DUF1499 family protein.	AK064976 ,AK061723	B8ANT3 (UniProt)	Protein of unknown function DUF1499
Os03g0858800	Multi antimicrobial extrusion protein MatE family protein.	AK109467	NP_001150832.1 (RefSeq)	Multi antimicrobial extrusion protein
Os03g0859100	Esterase, SGNH hydrolase-type domain containing protein.	AK060992	NP_001150794.1 (RefSeq)	Lipase, GDSL
Os03g0859300	Similar to La domain containing protein, expressed.	AK073135	Q10AA9 (UniProt)	RNA-binding protein Lupus La
Os03g0859500	ABC transporter-like domain containing protein.	AK070637	NP_850111.1 (RefSeq)	ABC transporter-like
Os03g0859550	Non-protein coding transcript.	AK241487	NONE	-
Os03g0859600	RuBisCO subunit binding-protein alpha subunit, chloroplast precursor (60 kDa chaperonin alpha subunit) (CPN-60 alpha).	AK061901	B6SXW8 (UniProt)	Chaperonin Cpn60
Os03g0859700	Similar to RuBisCO subunit binding-protein alpha subunit, chloroplast precursor (60 kDa chaperonin alpha subunit) (CPN-60 alpha) (Fragment).	AY224481	B6SXW8 (UniProt)	Chaperonin Cpn60/TCP-1
Os03g0859800	Ovarian tumour, otubain domain containing protein.	AK071971	NP_001148776.1 (RefSeq)	Ovarian tumour, otubain
Os03g0859850	Non-protein coding transcript.	EU946238	NONE	-
Os03g0859900	Protein of unknown function DUF547 domain containing protein.	AK072837	Q7Y1E8 (UniProt)	Domain of unknown function DUF547
Os03g0860000	ASCH domain domain containing protein.	AK066505	Q7Y1E7 (UniProt)	ASCH domain
Os03g0860050	Hypothetical gene.	AK243056	longestORF	-
Os04g0334700	Peptidase aspartic, catalytic domain containing protein.	AK120870	B7F2P1 (UniProt)	Peptidase A1
Os04g0334825	Conserved hypothetical protein.	ab initio prediction	Q7X7R4 (UniProt)	-
Os04g0334951	Similar to OSIGBa0137O04.7 protein.	AK109756	Q01KZ0 (UniProt)	Peptidase A1
Os04g0335075	Conserved hypothetical protein.	ab initio prediction	Q7X7R4 (UniProt)	-
Os04g0335200	Conserved hypothetical protein.	AK289258	Q2QWP9 (UniProt)	-
Os04g0335400	Non-protein coding transcript.	CT828838	NONE"	-
Os04g0336001	Hypothetical protein.	EU945299	longestORF	-
Os04g0336600	Peptidase aspartic, catalytic domain containing protein.	AK120870	B7F2P1 (UniProt)	Peptidase A1
Os04g0336700	Conserved hypothetical protein.	ab initio prediction	Q7X7R4 (UniProt)	-
Os04g0336801	Conserved hypothetical protein.	AK289258	Q2QWP9 (UniProt)"	-
Os04g0337000	Peptidase aspartic, catalytic domain containing protein.	AK066236	Q01KZ0 (UniProt)	Peptidase A1
Os04g0530100	Similar to Beta-expansin 1 precursor (AtEXPB1) (At-EXPB1) (Ath-ExpBeta-1.5).	AK107184	Q7X6J9 (UniProt)	Expansin/pollen allergen, DPBB domain
Os04g0530150	Non-protein coding transcript.	AK109871	NONE	-
Os04g0530200	Similar to OSIGBa0155K17.10 protein.	AK105586 ,AK121439	Q00RJ0 (UniProt)	-
Os04g0530300	Similar to OSIGBa0155K17.11 protein.	AK066705	Q00RI9 (UniProt)	-
Os04g0530400	t-snare domain containing protein.	AK067634	B6TDT7 (UniProt)	SNARE-complex protein Syntaxin-18 N-terminal
Os04g0530500	Zinc finger, RING/FYVE/PHD-type domain containing protein.	AK070732	NP_001152715.1 (RefSeq)	Zinc finger, RING-type
Os04g0530600	Similar to Thioredoxin 1 (TRX-1) (Thioredoxin M).	AK069195	NP_001150752.1 (RefSeq)	Thioredoxin
Os04g0530700	Similar to Beta-D-xylosidase.	AK120331	NP_196618.1 (RefSeq)	Glycoside hydrolase, family 3, N-terminal
Os04g0530801	Hypothetical gene.	EU973535	longestORF	-
Os04g0530900	Glycosyl transferase, family 8 protein.	AK072297	XP_002312381.1 (RefSeq)	Glycosyl transferase, family 8
Os04g0531100	C2 calcium-dependent membrane targeting domain containing	AK063584	Q0JBH9 (UniProt)	C2 calcium-dependent membrane targeting
Os04g0531200	Hypothetical conserved gene.	AB332066	A2XVT7 (UniProt)	-
Os04g0531300	tRNA-dihydrouridine synthase domain containing protein.	AK103911	Q00RI1 (UniProt)	tRNA-dihydrouridine synthase
Os04g0531400	Similar to Lectin-like receptor kinase 7%3B2.	AK105289	Q00RI0 (UniProt)	Protein kinase, catalytic domain
Os04g0531500	Concanavalin A-like lectin/glucanase domain containing protein.	AK102285	Q00RH9 (UniProt)	Protein kinase, catalytic domain

Os04g0531600	Hypothetical protein.	AK107678	longestORF	-
Os04g0531700	Short-chain dehydrogenase/reductase SDR domain containing protein.	AK119481	Q00RH9 (UniProt)	Short-chain dehydrogenase/reductase SDR
Os04g0531750	Similar to OSIGBa0125M19.13 protein.	ab initio prediction	Q00RH3 (UniProt)	Short-chain dehydrogenase/reductase SDR
Os04g0531800	Conserved hypothetical protein.	AK121355	B8ASD8 (UniProt)	-
Os04g0531900	Short-chain dehydrogenase/reductase SDR domain containing protein.	AK071069	Q00RH3 (UniProt)	Short-chain dehydrogenase/reductase SDR
Os04g0532100	Short-chain dehydrogenase/reductase SDR domain containing protein.	AK109281	Q00RH5 (UniProt)	Short-chain dehydrogenase/reductase SDR
Os04g0532200	Conserved hypothetical protein.	AK110853	B8ASE2 (UniProt)	-
Os04g0532400	Similar to OSIGBa0125M19.13 protein.	AK318556	Q00RH3 (UniProt)	Short-chain dehydrogenase/reductase SDR
Os04g0532500	Similar to Transcription factor L2.	AK069907	Q00RH2 (UniProt)	Zinc finger, LIM-type
Os04g0532700	Hypothetical protein.	AK109796	longestORF	-
Os04g0532800	Myb transcription factor domain containing protein.	AK107135	B4FNQ6 (UniProt)	SANT domain, DNA binding
Os04g0533000	Similar to RNA helicase (Fragment).	AK101477	B7EJQ0 (UniProt)	RNA helicase, ATP-dependent, DEAD-box, conserved site
Os04g0533200	Similar to Myb7 protein (Fragment).	AK376409	Q43598 (UniProt)	-
Os04g0567700	ORMDL family protein.	AK064998	Q01K71 (UniProt)	ORMDL
Os04g0567800	Similar to OSIGBa0103M18.3 protein.	AK060659	Q01K70 (UniProt)	Zinc finger, Dof-type
Os04g0568300	Hypothetical gene.	AK072175	longestORF	-
Os04g0568400	WD40 repeat-like domain containing protein.	AK103546	Q01JP7 (UniProt)	WD40 repeat
Os04g0568500	Conserved hypothetical protein.	AK363942	F2DJ74 (UniProt)	-
Os04g0568600	Similar to 6-phospho-3-hexuloisomerase.	CT836509	Q01JP6 (UniProt)	Sugar isomerase
Os04g0568700	Similar to Heat stress transcription factor Spl7 (Heat shock transcription factor) (Heat shock factor RHSF10).	AY344483	Q01JP5 (UniProt)	Heat shock factor
Os04g0568751	Hypothetical protein.	tplb0033f11 (Wheat FLcDNA)	longestORF	-
Os04g0568800	Similar to OSIGBa0139P06.4 protein.	AK106571	Q01JP4 (UniProt)	Protein of unknown function DUF810
Os04g0568850	Similar to OSIGBa0139P06.4 protein.	CT828553	Q01JP4 (UniProt)	-
Os04g0568900	Similar to RING finger protein 6 (RING-H2 protein).	AK070267	Q01JP3 (UniProt)	Zinc finger, RING-type
Os04g0568950	Hypothetical gene.	EU941385	longestORF	-
Os04g0569000	Similar to Replication factor C 40kDa subunit.	AK069025	Q948P2 (UniProt)	ATPase, AAA+ type, core
Os04g0569100	Similar to OCL1 homeobox protein.	AK112099 ,AK111914	Q7Y0V9-2 (UniProt)	Helix-turn-helix motif, lambda-like repressor
Os04g0569300	Similar to Membrane protein.	AK058648	Q01JP0 (UniProt)	Peptidase S54, rhomboid
Os04g0569400	Similar to OSIGBa0139P06.9 protein.	AK365201	Q01JN9 (UniProt)	Protein of unknown function DUF248, methyltransferase putative
Os04g0569500	Hypothetical protein.	tplb0021o10 (Wheat FLcDNA)	longestORF	-
Os04g0569900	IQ calmodulin-binding region domain containing protein.	AK100392	Q01J01 (UniProt)	IQ motif, EF-hand binding site
Os04g0570000	Cytochrome P450 family protein.	AJ459255	Q7XU38 (UniProt)	Cytochrome P450
Os04g0570125	Hypothetical protein.	tplb0036f19 (Wheat FLcDNA)	longestORF	-
Os04g0669500	Phospholipase/carboxylesterase domain containing protein.	AK069249	Q259P2 (UniProt)	Phospholipase/carboxylesterase/thioesterase
Os04g0669600	Phospholipase/carboxylesterase domain containing protein.	AK110767	Q259P1 (UniProt)	Phospholipase/carboxylesterase/thioesterase
Os04g0669700	Similar to H0818H01.9 protein.	CT835076	Q259P0 (UniProt)	Phospholipase/carboxylesterase/thioesterase
Os04g0669800	Methylthioribose kinase (EC 2.7.1.100).	AK067649	Q7XR61 (UniProt)	Aminoglycoside phosphotransferase
Os04g0669900	Similar to H0818H01.11 protein.	AY593959	Q259N8 (UniProt)	Aminoglycoside phosphotransferase
Os04g0670000	Reticulon family protein.	AK071792	Q259P4 (UniProt)	Reticulon
Os04g0670100	Similar to H0818H01.13 protein.	FP095236	Q259P3 (UniProt)	-
Os04g0670150	Hypothetical protein.	BT068200	longestORF	-
Os04g0670200	Similar to Oryzain beta chain precursor (EC 3.4.22.-).	AK109371 ,AK073373	Q0WXG7 (UniProt)	Granulin
Os04g0670400	Similar to H0624F09.2 protein.	AK103099	Q258Z6 (UniProt)	-
Os04g0670500	Cysteine protease 1 precursor (EC 3.4.22.-) (OsCP1).	AK107506	Q7XR52 (UniProt)	Granulin
Os04g0670600	Similar to H0624F09.5 protein.	AK064255	Q258Z3 (UniProt)	Exostosin-like
Os04g0670700	Similar to H0624F09.4 protein.	AK063069	Q258Z4 (UniProt)	Phosphatidate cytidylyltransferase
Os04g0670800	UBX domain containing protein.	AK073170	Q258Z2 (UniProt)	UBX
Os04g0670900	Homeodomain-like containing protein.	DQ383374	Q258Z1 (UniProt)	MYB-like
Os04g0671100	Similar to H0624F09.8 protein.	AK241125	Q258Z0 (UniProt)	Adenylate kinase
Os04g0671200	Similar to Suppressor of presenilin 5 (P110b homolog).	AK070003	Q258Y9 (UniProt)	Flavin amine oxidase
Os04g0671250	Hypothetical protein.	tplb0048c15 (Wheat FLcDNA)	longestORF	-
Os04g0671300	Similar to Suppressor of presenilin 5 (P110b homolog).	AK072414	Q258Y8 (UniProt)	Flavin amine oxidase
Os04g0671500	Hypothetical protein.	tplb0048c15 (Wheat FLcDNA)	longestORF	-
Os04g0671700	ThiJ/PfpI domain containing protein.	AK100753	NP_001146927.1 (RefSeq)	ThiJ/PfpI
Os04g0671800	Zinc finger, CCCH-type domain containing protein.	AK070857	F1DK83 (UniProt)	Zinc finger, CCCH-type
Os04g0671900	Similar to P-167-1_1 (Fragment).	AK071455	Q0J951 (UniProt)	AUX/IAA protein
Os04g0672100	Similar to Phytosulfokine receptor precursor (EC 2.7.1.37) (Phytosulfokine LRR receptor kinase).	AK121689	Q258Z9 (UniProt)	Protein kinase, catalytic domain
Os04g0672200	Poly(ADP-ribose) polymerase, catalytic region domain containing protein.	AK099725	Q258Z8 (UniProt)	Poly
Os04g0672300	Similar to H0322F07.3 protein.	AK101246	Q259A6 (UniProt)	-
Os04g0672600	Leucine-rich repeat, N-terminal domain containing protein.	AK070283	Q258Z9 (UniProt)	Leucine-rich repeat
Os04g0672700	Pentatricopeptide repeat domain containing protein.	AK100434 ,AK102863	Q259A5 (UniProt)	Pentatricopeptide repeat
Os04g0672800	Similar to H0322F07.5 protein.	AK068413	Q259A4 (UniProt)	-
Os04g0672900	Similar to H0322F07.6 protein.	AK065014	Q259A3 (UniProt)	Domain of unknown function DUF2296
Os04g0673000	Similar to H0322F07.7 protein.	AK068390	Q259A2 (UniProt)	Protein of unknown function DUF3755
Os04g0673050	Non-protein coding transcript.	tplb0055d06 (Wheat FLcDNA)	NONE	-
Os04g0673300	A-type response regulator, Cytokinin signaling	AK059734 ,AB249653 ,BR00031	Q7XQA6 (UniProt)	Signal transduction response regulator, receiver domain
Os04g0674100	Tetratricopeptide-like helical domain containing protein.	AK288533	Q259S2 (UniProt)	Tetratricopeptide TPR-1
Os04g0674200	Coenzyme Q biosynthesis Coq4 family protein.	AK103795 ,AK101965	Q259S1 (UniProt)	Coenzyme Q biosynthesis Coq4
Os04g0674300	NPH3 domain containing protein.	AK119773	Q259S0 (UniProt)	BTB/POZ-like
Os04g0674350	Conserved hypothetical protein.	AK241498	NP_001174139.1 (RefSeq)	-
Os04g0674400	Similar to Anamorsin (Cytokine induced apoptosis inhibitor 1) (CUA001). Splice isoform 2.	AK102124	B8ARI7 (UniProt)	Cytokine-induced anti-apoptosis inhibitor 1
Os04g0674425	Non-protein coding transcript.	BT086960	NONE	-
Os04g0674450	Similar to DHHC zinc finger domain containing protein.	AK376242	NP_001147996.1 (RefSeq)	Zinc finger, DHHC-type, palmitoyltransferase
Os04g0674500	Hypothetical protein.	AK120940	longestORF	-
Os04g0674600	Similar to H0103C06.1 protein.	AK069645	Q259I1 (UniProt)	Oligopeptide transporter OPT superfamily
Os04g0674700	Similar to AMP-binding protein (Adenosine monophosphate binding protein 5 AMPBP5).	AK106615	Q259I0 (UniProt)	AMP-dependent synthetase/ligase
Os04g0674750	Hypothetical protein.	EU941562	longestORF	-
Os04g0674800	Similar to CEL1%3DCELLULASE 1 (Fragment).	AK119913	Q0J930 (UniProt)	Glycoside hydrolase, family 9
Os04g0674900	Hypothetical protein.	tplb0042g22 (Wheat FLcDNA)	longestORF	-
Os04g0675000	Similar to H0103C06.5 protein.	AK103357	Q259G9 (UniProt)	Protein of unknown function DUF789
Os04g0675101	ATPase-like, ATP-binding domain domain containing protein.	BT066996	B9FDA1 (UniProt)	ATPase-like, ATP-binding domain
Os04g0675200	Similar to H0103C06.6 protein.	AK121874	Q259H3 (UniProt)	-
Os04g0675300	Zinc finger, RING/FYVE/PHD-type domain containing protein.	AK101752	Q259H3 (UniProt)	Zinc finger, RING-type
Os04g0675400	Similar to Chaperone protein dnaJ.	AK068186	Q259H2 (UniProt)	Heat shock protein DnaJ, N-terminal
Os04g0675500	Similar to Itm1 protein.	AK067609	Q259H1 (UniProt)	Oligosaccharyl transferase, STT3 subunit
Os04g0675600	Hypothetical conserved gene.	AK242041	NP_001054249.1 (RefSeq)	Pentatricopeptide repeat
Os04g0675700	Hypothetical conserved gene.	ab initio prediction	D0ABD8 (UniProt)	FBD
Os04g0675800	Similar to H0103C06.10 protein.	ab initio prediction	Q259H7 (UniProt)	F-box domain, cyclin-like
Os04g0676000	Non-protein coding transcript.	CT836511	NONE"	-
Os04g0676100	Similar to Thioredoxin X, chloroplast precursor.	AK288094	Q259H6 (UniProt)	Thioredoxin
Os04g0676200	Pentatricopeptide repeat domain containing protein.	AK106412	Q7XKC9 (UniProt)	Pentatricopeptide repeat
Os04g0676300	Similar to H0101F08.3 protein.	AK287813	Q259H4 (UniProt)	Dihydroorotate dehydrogenase, conserved site
Os04g0676400	Similar to H0101F08.4 protein.	AK121530	Q259I9 (UniProt)	Protein of unknown function DUF761, plant
Os04g0682600	Similar to H0502G05.7 protein.	AK062587	Q01JF9 (UniProt)	Ribonuclease H-like
Os04g0682800	Similar to H0124B04.17 protein.	AK121846	Q259F9 (UniProt)	Regulator of K+ conductance, N-terminal
Os04g0682850	Hypothetical protein.	AK363281	longestORF	-
Os04g0682900	Similar to H0124B04.17 protein.	AK065300	Q259F9 (UniProt)	DNA mismatch repair protein MutS-like, N-terminal
Os04g0683100	Similar to Cleavage and polyadenylation specificity factor 5.	AK064962	B6T6P2 (UniProt)	NUDIX hydrolase domain
Os04g0683400	Similar to H0306F12.4 protein.	EU958236	Q259Q7 (UniProt)	Transcription factor CBF/NF-Y/archaeal histone
Os04g0683500	Similar to H0306F12.5 protein.	AK071538	Q259R1 (UniProt)	Adenosine deaminase/editase
Os04g0683600	Similar to H0306F12.6 protein.	AK065557	Q259R0 (UniProt)	Leucine-rich repeat
Os04g0683700	Similar to 4-coumarate-CoA ligase-like protein (Adenosine monophosphate binding protein 3 AMPBP3).	AK119512	Q259Q6 (UniProt)	AMP-dependent synthetase/ligase
Os04g0683800	Similar to Phosphoribosyltransferase (Fragment).	AK073897 ,AK102910	B9FDD7 (UniProt)	C2 calcium-dependent membrane targeting
Os04g0683850	Similar to Glutathione peroxidase.	AK249946	C5YAP1 (UniProt)	Glutathione peroxidase
Os04g0683875	Non-protein coding transcript.	AF366451	NONE	-
Os04g0683900	Protein of unknown function DUF296 domain containing protein.	AK099749	Q259R4 (UniProt)	Domain of unknown function DUF296
Os04g0683950	Non-protein coding transcript.	EU948418	NONE	-
Os04g0684000	Hypothetical protein.	AK102578	longestORF	-
Os04g0684100	Hypothetical conserved gene.	AK060126	B9FDD9 (UniProt)	-
Os04g0684200	Similar to H0913C04.1 protein.	AK101506	Q259U8 (UniProt)	-
Os04g0691300	Similar to ANAC030.	AK334647	NP_001148332.1 (RefSeq)	No apical meristem
Os04g0691366	Similar to POT family protein.	ab initio prediction	B6SXJ7 (UniProt)	-
Os04g0691400	Similar to POT family protein.	ab initio prediction	B6SXJ7 (UniProt)	Oligopeptide transporter
Os04g0691433	Non-protein coding transcript.	EU946594	NONE	-
Os04g0691466	Hypothetical gene.	EU949821	longestORF	-

Os04g0691500	Similar to predicted protein.	AK069073	NP_001031359.1 (RefSeq)	HEAT
Os04g0691600	Similar to 30S ribosomal protein S17.	AK059422	Q9ZST1 (UniProt)	Ribosomal protein S17
Os04g0691700	GCN5-related N-acetyltransferase (GNAT) domain domain containing protein.	AK106305	Q7FAP7 (UniProt)	GCN5-related N-acetyltransferase
Os04g0691750	Similar to phosphoribosylanthranilate transferase.	tplb0048i05 (Wheat FLcDNA)	NP_001152458.1 (RefSeq)	-
Os04g0691800	C2 domain containing protein.	AK318532	NP_001152458.1 (RefSeq)	C2 calcium-dependent membrane targeting
Os04g0691900	Chaperonin Cpn60/TCP-1 family protein.	AK068257	Q7XKA1 (UniProt)	Chaperonin Cpn60/TCP-1
Os04g0692000	Protein of unknown function DUF6, transmembrane domain containing protein.	AK103611 ,AK061991	B4G1R1 (UniProt)	-
Os04g0692100	Similar to Tubulin folding cofactor B.	AK102150	A5CFZ4 (UniProt)	Cytoskeleton-associated protein, Gly-rich domain
Os04g0692200	Protein of unknown function DUF3727 domain containing [illegible]	AK059807 ,AK060875 ,AK07172[illegible]	B9FDS1 (UniProt)	Protein of unknown function DUF3727
Os04g0692300	Peptidase C14, ICE, catalytic subunit p20, active site domain containing protein.	AK073368	XP_002871357.1 (RefSeq)	Zinc finger, RING/FYVE/PHD-type
Os04g0692400	Similar to ankyrin-like protein.	AK070717	NP_001151565.1 (RefSeq)	Protein of unknown function DUF248, methyltransferase putative
Os04g0692500	Similar to antiporter/ drug transporter/ transporter.	AK121096	NP_001147555.1 (RefSeq)	Multi antimicrobial extrusion protein
Os04g0692600	Staygreen protein domain containing protein.	AK105982	B8AS49 (UniProt)	Staygreen protein
Os04g0692700	Similar to CHR24 (chromatin remodeling 24)%3B ATP binding / DNA binding / helicase.	BT087024	NP_201200.2 (RefSeq)	Helicase, C-terminal
Os04g0692725	Hypothetical gene.	AK242828	longestORF	-
Os04g0692750	Hypothetical conserved gene.	BT083703	XP_002309928.1 (RefSeq)	-
Os05g0149300	Similar to 1-aminocyclopropane-1-carboxylate oxidase.	BT087584	O81606 (UniProt)	Oxoglutarate/iron-dependent oxygenase
Os05g0149400	Similar to 1-aminocyclopropane-1-carboxylic acid oxidase.	AK061064	O81606 (UniProt)	Oxoglutarate/iron-dependent oxygenase
Os05g0149450	Non-protein coding transcript.	tplb0008n13 (Wheat FLcDNA)	NONE	-
Os05g0149500	Similar to lipopolysaccharide-modifying protein.	AK064684	NP_001151574.1 (RefSeq)	Lipopolysaccharide-modifying protein
Os05g0149600	Similar to CULLIN1-like protein 1.	AK073947	C7SJ62 (UniProt)	-
Os05g0149701	Conserved hypothetical protein.	BT086859	C4J7R2 (UniProt)	-
Os05g0149800	EF-Hand type domain containing protein.	AK070081	B7UBT1 (UniProt)	EF-hand-like domain
Os05g0149850	Non-protein coding transcript.	BT016490	NONE	-
Os05g0149900	Tetratricopeptide-like helical domain containing protein.	AK101105 ,AK105677	XP_002881630.1 (RefSeq)	Tetratricopeptide-like helical
Os05g0149950	Monothiol glutaredoxin-S8.	P0C290 (UniProt)	P0C290 (UniProt)	Glutaredoxin
Os05g0150000	Proline synthetase co-transcribed bacterial homolog protein.	AK289260	XP_002889904.1 (RefSeq)	Alanine racemase, N-terminal
Os05g0150100	Conserved hypothetical protein.	AK071562	B9FHD8 (UniProt)	-
Os05g0150300	Similar to Possible global transcription activator SNF2L1 (SWI/SNF related matrix associated actin dependent regulator of chromatin subfamily A member 1).	AK100732	XP_002315568.1 (RefSeq)	SNF2-related
Os05g0150400	Double-stranded RNA-binding domain containing protein.	AK110983	B9FMH4 (UniProt)	Double-stranded RNA-binding
Os05g0150500	Similar to Transport inhibitor response 1.	EU400583	D0ES26 (UniProt)	Leucine-rich repeat, cysteine-containing subtype
Os05g0150550	Hypothetical protein.	tplb0032b19 (Wheat FLcDNA)	longestORF	-
Os05g0150600	Hypothetical conserved gene.	AK063338	D7U9T8 (UniProt)	DNA helicase, ATP-dependent, RecQ type
Os05g0150733	Similar to pro-resilin.	ab initio prediction	NP_001148515.1 (RefSeq)	-
Os05g0150800	Similar to Plastid 5,10-methylene-tetrahydrofolate dehydrogenase (Fragment).	AK103505	B6T3G6 (UniProt)	Tetrahydrofolate dehydrogenase/cyclohydrolase
Os05g0150900	Histidyl-tRNA synthetase (EC 6.1.1.21) (Histidine--tRNA ligase) (HisRS).	AK099789	P93422 (UniProt)	Phenylalanine/histidine ammonia-lyase
Os05g0151000	Similar to DNA-directed RNA polymerase.	AK371102	C5YGY9 (UniProt)	RNA polymerase II, heptapeptide repeat, eukaryotic
Os05g0154500	Spc97/Spc98 family protein.	AK119462	XP_002320339.1 (RefSeq)	Spc97/Spc98
Os05g0154600	Similar to VIP2 protein.	AK111816	Q9M4C5 (UniProt)	Zinc finger, RING-type
Os05g0154700	Similar to Kinesin heavy chain (Fragment).	AK064328	B9FMJ3 (UniProt)	Kinesin, motor domain
Os05g0154800	Similar to U1snRNP-specific protein, U1A.	AK102716	Q41498 (UniProt)	RNA recognition motif domain
Os05g0154850	Hypothetical protein.	BT019068	longestORF	-
Os05g0154900	Conserved hypothetical protein.	AK070869	B8AY68 (UniProt)	-
Os05g0155000	Similar to Protein MFP-b.	AK102420	NP_001152306.1 (RefSeq)	Crotonase, core
Os05g0155050	Hypothetical gene.	BT019314	longestORF	-
Os05g0155100	Similar to 60S ribosomal protein L18.	AK120877 ,AK061929 ,AK10281[illegible]	A2Y0K0 (UniProt)	Ribosomal protein L18e
Os05g0155200	Similar to Ethylene receptor.	AK111696	A1IIY0 (UniProt)	GAF
Os05g0155250	Hypothetical protein.	tplb0049a13 (Wheat FLcDNA)	longestORF	-
Os05g0155300	Similar to HIRA interacting protein 5.	AK069217	B8R1J6 (UniProt)	NIF system FeS cluster assembly, NifU, C-terminal
Os05g0155400	Zinc finger, NHR/GATA-type domain containing protein.	AK241016	NP_001054691.2 (RefSeq)	Zinc finger, GATA-type
Os05g0155450	Non-protein coding transcript.	EU970609	NONE	-
Os05g0155500	Hypothetical conserved gene.	AK120498	Q5WMY0 (UniProt)	-
Os05g0155601	Importin alpha-1b subunit.	Q9SLX0 (UniProt)	Q9SLX0 (UniProt)	Armadillo
Os05g0155700	Ribosomal protein S2, conserved site domain containing protein.	AK066581	Q01L45 (UniProt)	Protein of unknown function DUF1624
Os05g0155800	Similar to OSIGBa0157A06.5 protein.	EU968877	Q01IV4 (UniProt)	Transferase
Os05g0156200	Conserved hypothetical protein.	AK071622	B9FHF2 (UniProt)	-
Os05g0156300	Similar to protein disulfide isomerase.	AK099341	Q75M08-2 (UniProt)	Thioredoxin
Os05g0156401	Hypothetical gene.	BT086849	longestORF	-
Os05g0156500	Similar to Apobec-1 binding protein 2.	AK062718	XP_002878466.1 (RefSeq)	Chaperone DnaJ, C-terminal
Os05g0156600	Similar to Tubulin gamma-1 chain (Gamma-1 tubulin).	AK102557	O49068 (UniProt)	Tubulin
Os05g0156700	Similar to Dihydrolipoyl dehydrogenase.	AK242902	B8AYG2 (UniProt)	Fumarate reductase/succinate dehydrogenase flavoprotein, N-terminal
Os05g0328800	Prolamin 7.	X53857	Q5EFA4 (UniProt)	Bifunctional trypsin/alpha-amylase inhibitor
Os05g0328901	Similar to Prolamin.	AK242260	Q5EFA4 (UniProt)	-
Os05g0329001	Similar to Prolamin.	AF042201	Q5EFA4 (UniProt)	-
Os05g0329100	Prolamin.	AK242260	Q5EFA4 (UniProt)	Bifunctional trypsin/alpha-amylase inhibitor
Os05g0329200	Similar to Prolamin.	AK242260	Q5EFA4 (UniProt)	Bifunctional trypsin/alpha-amylase inhibitor
Os05g0329300	Prolamin.	AK242260	Q5EFA4 (UniProt)	Bifunctional trypsin/alpha-amylase inhibitor
Os05g0329350	Prolamin.	EF122440	Q5EFA4 (UniProt)	Bifunctional trypsin/alpha-amylase inhibitor
Os05g0329400	Similar to Prolamin.	AK242260	Q5EFA4 (UniProt)	-
Os05g0329700	Similar to Prolamin.	AK242910	Q5EFA4 (UniProt)	Bifunctional trypsin/alpha-amylase inhibitor
Os05g0330150	Similar to Prolamin.	AK242260	Q5EFA4 (UniProt)	Bifunctional trypsin/alpha-amylase inhibitor
Os05g0515400	Similar to Auxin response factor 14.	AK067927	Q0DGS1 (UniProt)	Transcriptional factor B3
Os05g0515500	Similar to O-methyltransferase ZRP4 (EC 2.1.1.-) (OMT).	AK068905	NP_001105689.1 (RefSeq)	O-methyltransferase, family 2
Os05g0515600	Similar to O-methyltransferase ZRP4 (EC 2.1.1.-) (OMT).	AK287483	NP_001148593.1 (RefSeq)	O-methyltransferase, family 2
Os05g0515700	Colicin E3, catalytic domain containing protein.	AK069076	B4FR91 (UniProt)	-
Os05g0515900	Similar to heat- and acid-stable phosphoprotein.	AK073259	NP_001148324.1 (RefSeq)	-
Os05g0516100	Conserved hypothetical protein.	AK107721	B8AZZ5 (UniProt)	-
Os05g0516300	Conserved hypothetical protein.	AK067311	B9FGS5 (UniProt)	-
Os05g0516400	Similar to Hydroxyproline-rich glycoprotein DZ-HRGP [illegible]	AK110196	Q3LFP8 (UniProt)	Protein kinase, catalytic domain
Os05g0516475	Hypothetical protein.	tplb0031a15 (Wheat FLcDNA)	longestORF	-
Os05g0516550	Hypothetical gene.	AK068854	longestORF	-
Os05g0516600	Small GTP binding protein.	AK112031 ,AK111896	Q40787 (UniProt)	Small GTPase superfamily
Os05g0516700	Conserved hypothetical protein.	AK109131	B9FGS6 (UniProt)	-
Os05g0516800	Similar to Ras-related protein RIC2.	AK061969	B6T0Y1 (UniProt)	Small GTPase superfamily
Os05g0516850	Hypothetical gene.	BT084300	longestORF	-
Os05g0516900	Protein of unknown function DUF300 family protein.	AK069607	O80656 (UniProt)	Protein of unknown function DUF300
Os05g0517100	Hypothetical conserved gene.	BT066510	NP_172174.1 (RefSeq)	Domain of unknown function DUF623
Os05g0517200	HAD-superfamily hydrolase subfamily IIB protein.	AK072066	B2ZAS1 (UniProt)	Glycosyl transferase, family 20
Os05g0517400	Conserved hypothetical protein.	AK106780	B9FGS9 (UniProt)	-
Os05g0517500	Similar to Gamma-glutamyl hydrolase precursor (EC 3.4.19.9) (Gamma-Glu-X carboxypeptidase) (Conjugase) (GH).	AK065245	B6UBJ6 (UniProt)	Peptidase C26
Os05g0517600	Similar to Symbiotic-like hemoglobin 5.	ab initio prediction	A3EX90 (UniProt)	Globin, subset
Os05g0517800	Conserved hypothetical protein.	EU959047	NP_001056054.1 (RefSeq)	-
Os05g0517900	2 , 3 cyclic phosphodiesterase, plant domain containing protein.	AK243412	NP_001151923.1 (RefSeq)	RNA ligase/cyclic nucleotide phosphodiesterase
Os05g0518000	Similar to ZCN12 protein.	EU241924	A9LLY2 (UniProt)	Phosphatidylethanolamine-binding, conserved site
Os05g0566200	NLI interacting factor domain containing protein.	AK099836	B9FLM9 (UniProt)	NLI interacting factor
Os05g0566300	PRC-barrel domain containing protein.	AK099641	B9FI59 (UniProt)	RimM protein
Os05g0566400	Similar to Blast and wounding induced mitogen-activated protein kinase.	AK099472 ,AK061645 ,AK10038[illegible]	B3GCL3 (UniProt)	Protein kinase, catalytic domain
Os05g0566450	Non-protein coding transcript.	tplb0038e20 (Wheat FLcDNA)	NONE	-
Os05g0566500	Similar to Initiation factor 3d (Fragment).	AK073468	B6U4Q2 (UniProt)	Eukaryotic translation initiation factor 3, subunit 7
Os05g0566600	Similar to Negatively light-regulated protein.	AK058475	B6SN23 (UniProt)	Endosulphine
Os05g0566700	Similar to Homeobox protein B-H1 (Homeobox BarH1 protein).	AK121469	Q688W9 (UniProt)	-
Os05g0566800	Cold acclimation protein COR413-TM1.	AK065748	Q84XU8 (UniProt)	Cold acclimation WCOR413
Os05g0566900	Similar to TA9 protein (Fragment).	AK120605 ,AK066026	B8AWS4 (UniProt)	UBA-like
Os05g0567100	Aspartic proteinase oryzasin 1 precursor (EC 3.4.23.-).	AK098911 ,AK067742 ,AK09890[illegible]	NP_001148782.1 (RefSeq)	Peptidase A1
Os05g0567200	Similar to WRKY transcription factor 43.	ab initio prediction	Q6IEN8 (UniProt)	DNA-binding WRKY
Os05g0567300	Similar to GTP-binding protein era.	AK112068	B4FIL9 (UniProt)	Protein synthesis factor, GTP-binding
Os05g0567400	Ribosomal RNA methyltransferase J domain containing protein.	AK101119	Q1EPL1 (UniProt)	Ribosomal RNA methyltransferase RrmJ/FtsJ
Os05g0567500	HhH-GPD domain domain containing protein.	AK121133	NP_001151950.1 (RefSeq)	HhH-GPD domain
Os05g0567600	Similar to SANT/MYB protein.	AK361203	NP_001147389.1 (RefSeq)	SANT domain, DNA binding
Os05g0567650	Hypothetical protein.	tplb0033n03 (Wheat FLcDNA)	longestORF	-
Os05g0567700	Similar to Integral membrane protein.	AK065497	NP_001148779.1 (RefSeq)	General substrate transporter
Os05g0567800	Similar to solute carrier family 2, facilitated glucose transporter member 8.	AK108622	NP_001147831.1 (RefSeq)	-
Os05g0567850	Hypothetical protein.	tplb0033n03 (Wheat FLcDNA)	longestORF	-

Os05g0567900	Hypothetical conserved gene.	GU722206	B9FLN6 (UniProt)	Zinc finger, GATA-type
Os05g0568000	Similar to FAE1.	AK119596 ,AK101103	A2Y7K8 (UniProt)	-
Os05g0568100	Similar to Iron sulfur cluster assembly protein 1, mitochondrial precursor (Iron sulfur cluster scaffold protein 1).	AK069724	B4FTN1 (UniProt)	NIF system FeS cluster assembly, NifU, N-terminal
Os05g0568300	Similar to 50S ribosomal protein L12, chloroplast precursor (CL12)	CU405781	O22386-2 (UniProt)	Ribosomal protein L7/L12
Os05g0568600	Protein of unknown function DUF1645 family protein.	AK063781	B9FI71 (UniProt)	Protein of unknown function DUF1645
Os05g0568800	Protein of unknown function DUF1645 family protein.	AK059883	B8AWS9 (UniProt)	Protein of unknown function DUF1645
Os05g0568900	Similar to Protease Do-like 1, chloroplast precursor (EC 3.4.21.- `	AK120626 ,AK061450 ,AK06736	NP_189431.2 (RefSeq)	Peptidase S1/S6, chymotrypsin/Hap
Os05g0569000	Hypothetical conserved gene.	AK242172	B8AWT1 (UniProt)	-
Os06g0140200	Leucine-rich repeat, plant specific containing protein.	AK287701 ,AK241776 ,AK28787	Q5VPE8 (UniProt)	Leucine-rich repeat
Os06g0140300	Leucine-rich repeat, N-terminal domain containing protein.	AK100368	B8B2B4 (UniProt)	Leucine-rich repeat
Os06g0140400	Similar to HAHB-6 (Fragment).	AK102692	B7E3F1 (UniProt)	Homeobox
Os06g0140700	Similar to Homeodomain leucine zipper protein (Fragment).	AK105150	Q5VPE3 (UniProt)	Helix-turn-helix motif, lambda-like repressor
Os06g0140800	Protein kinase, core domain containing protein.	AK065683	A1YKF2 (UniProt)	Protein kinase, catalytic domain
Os06g0140900	Sigma factor, regions 3 and 4 domain containing protein.	AK058823	B8B2B8 (UniProt)	-
Os06g0140950	Non-protein coding transcript.	AK242656	NONE	-
Os06g0141100	Monosaccharide transporter.	ab initio prediction	C0JSA7 (UniProt)	Sugar/inositol transporter
Os06g0141133	Hypothetical protein.	tplb0053a05 (Wheat FLcDNA)	longestORF	-
Os06g0141166	Similar to hydrolase, NUDIX family protein.	ab initio prediction	NP_001149422.1 (RefSeq)	NUDIX hydrolase domain
Os06g0141200	Similar to RNA-binding protein EWS.	AK061234 ,AK099058	Q9SW92 (UniProt)	Zinc finger, RanBP2-type
Os06g0141400	Similar to Early nodulin.	AK121184	Q9XFD2 (UniProt)	Early nodulin 93 ENOD93 protein
Os06g0141600	Early nodulin 93 ENOD93 protein family protein.	AK059772	O82787 (UniProt)	Early nodulin 93 ENOD93 protein
Os06g0141700	Similar to Early nodulin.	AK121791	O82787 (UniProt)	Early nodulin 93 ENOD93 protein
Os06g0141800	Similar to fasciclin-like arabinogalactan protein 8.	AK107277	NP_001150349.1 (RefSeq)	-
Os06g0141950	Similar to MYB transcription factor.	AJ601380	B4FX89 (UniProt)	Homeodomain-related
Os06g0142000	Hypothetical conserved gene.	CT836263	NP_001056764.1 (RefSeq)	Peptidase cysteine/serine, trypsin-like
Os06g0142050	Similar to H0913C04.9 protein.	BT035075	NP_565878.1 (RefSeq)	Ribosomal biogenesis regulatory protein
Os06g0142100	Cyclin-like F-box domain containing protein.	ab initio prediction	NP_001056765.1 (RefSeq)	-
Os06g0142200	Early nodulin.	AK122162	B6SIM1 (UniProt)	Early nodulin 93 ENOD93 protein
Os06g0142300	Early nodulin 93 ENOD93 protein family protein.	CT837557	O82787 (UniProt)	Early nodulin 93 ENOD93 protein
Os06g0142350	Similar to Early nodulin.	AK242220	O82787 (UniProt)	Early nodulin 93 ENOD93 protein
Os06g0142400	Early nodulin.	AK241230	O82787 (UniProt)	Early nodulin 93 ENOD93 protein
Os06g0142500	Similar to Wall-associated kinase 3.	AK252642	Q4U3Z6 (UniProt)	EGF-type aspartate/asparagine hydroxylation site
Os06g0142550	Non-protein coding transcript.	CT828398	NONE"	-
Os06g0142600	Similar to ELF3 protein.	AK242105	A7YJG7 (UniProt)	-
Os06g0142625	Hypothetical protein.	BT039571	longestORF	-
Os06g0142650	Similar to Avr9/Cf-9 rapidly elicited protein 11 (Fragment).	ab initio prediction	NP_001174594.1 (RefSeq)	Protein kinase, catalytic domain
Os06g0142700	Cytochrome c oxidase, subunit Vb family protein.	AK071423	P92683 (UniProt)	Cytochrome c oxidase, subunit Vb
Os06g0142800	Ribosomal L11 methyltransferase domain containing protein.	AK073269 ,AK103971	NP_563720.1 (RefSeq)	Skb1 methyltransferase
Os06g0142900	Similar to 1-deoxy-D-xylulose 5-phosphate synthase.	AK121920	F2WM28 (UniProt)	-
Os06g0143000	Iron-superoxide dismutase (EC 1.15.1.1).	AK062073 ,AK071301	B8B2C9 (UniProt)	Manganese/iron superoxide dismutase
Os06g0143100	Similar to Cadmium tolerant 1.	AK061597	B5BSU1 (UniProt)	-
Os06g0143400	Similar to Acyl-ACP thioesterase (Fragment).	AK072729 ,AK120946	A1YKG2 (UniProt)	Acyl-ACP thioesterase
Os06g0143500	Pentatricopeptide repeat domain containing protein.	ab initio prediction	Q9SNP3 (UniProt)	Pentatricopeptide repeat
Os06g0143600	Similar to PRP38 pre-mRNA processing factor 38 domain containing B.	AK061914	B6TSE1 (UniProt)	-
Os06g0143650	Non-protein coding transcript.	tplb0040d24 (Wheat FLcDNA)	NONE	-
Os06g0143700	Similar to Sulfate transporter 2.	AK067270	NP_001148179.1 (RefSeq)	Sulphate anion transporter
Os06g0143750	Hypothetical protein.	tplb0059b17 (Wheat FLcDNA)	longestORF	-
Os06g0143800	Non-protein coding transcript.	AK121251	NONE	-
Os06g0143900	Similar to Coatomer protein complex, beta prime%3B beta'-COP protein.	AK111584	A1YKF7 (UniProt)	WD40 repeat
Os06g0143950	Non-protein coding transcript.	BT086934	NONE	-
Os06g0144000	BRCT domain containing protein.	AK068998	A1YKF6 (UniProt)	BRCT
Os06g0144200	Similar to Pectate lyase homolog (EC 4.2.2.2).	AK100191	Q43862 (UniProt)	Pectate lyase/Amb allergen
Os06g0144600	Similar to Zinc carboxy peptidase.	AK067250	A1YKF5 (UniProt)	Peptidase M14, carboxypeptidase A
Os06g0144800	Similar to GTP-binding protein lepA.	AK103630	B8B2R1 (UniProt)	Translation elongation factor EFG/EF2, C-terminal
Os06g0144850	Hypothetical protein.	tplb0047c16 (Wheat FLcDNA)	longestORF	-
Os06g0144900	Pectate lyase/Amb allergen domain containing protein.	AK070509	GeneMark	Pectate lyase/Amb allergen
Os06g0145200	Transferase family protein.	AK109544	A2Y974 (UniProt)	Transferase
Os06g0145300	Transferase domain containing protein.	ab initio prediction	A3B8C8 (UniProt)	Transferase
Os06g0145400	Transferase domain containing protein.	AK332289	Q5VP58 (UniProt)	Transferase
Os06g0513943	Conserved hypothetical protein.	CT833375	B8AJ30 (UniProt)"	-
Os06g0514100	Conserved hypothetical protein.	CT833375	B8AJ30 (UniProt)"	-
Os06g0552900	Similar to SP3D.	CT836192	A8WET3 (UniProt)	Phosphatidylethanolamine-binding, conserved site
Os06g0553001	Conserved hypothetical protein.	EU972049	B6U484 (UniProt)	-
Os06g0553100	Similar to Heat stress transcription factor C-2b.	AK241254	Q0DBL6 (UniProt)	Heat shock factor
Os06g0553200	Similar to Meiosis 5.	AK104528 ,AK060896 ,AK10463	B6SUD3 (UniProt)	-
Os06g0553550	Conserved hypothetical protein.	EU945783	C0PA66 (UniProt)	-
Os06g0553700	Conserved hypothetical protein.	CT836191	Q5Z9C9 (UniProt)	-
Os06g0553800	Cupredoxin domain containing protein.	AK072932	Q5Z9C7 (UniProt)	Plastocyanin-like
Os06g0554100	Hypothetical conserved gene.	CT836317	Q5Z9C5 (UniProt)	Zinc finger, RING-type
Os06g0554200	Similar to F20B17.20.	AK363742	XP_002889325.1 (RefSeq)	Protein of unknown function DUF1677, plant
Os06g0554300	Conserved hypothetical protein.	AK070137 ,AK103962	B8B3L6 (UniProt)	-
Os06g0606599	Hypothetical protein.	ab initio prediction	NONE	-
Os06g0606700	Tetratricopeptide-like helical domain containing protein.	AK359397	Q69Q43 (UniProt)	Tetratricopeptide-like helical
Os06g0606800	Targeting for Xklp2 family protein.	AK066355	B6U8B2 (UniProt)	Xklp2 targeting protein
Os06g0606900	Conserved hypothetical protein.	AK068134	B9FU26 (UniProt)	-
Os06g0607000	Similar to Beta-1,3-glucanase.	AK121115	A4PID2 (UniProt)	Glycoside hydrolase, family 17
Os06g0607100	Similar to phosphatidic acid phosphatase-related / PAP2-related.	AK071250	NP_566527.1 (RefSeq)	-
Os06g0607200	Similar to Cellular retinaldehyde binding/alpha-tocopherol transport%3B Cellular retinaldehyde-binding/triple function, N-terminal.	AK242958	Q2HV32 (UniProt)	Cellular retinaldehyde-binding/triple function, N-terminal
Os06g0607700	ABC transporter-like domain containing protein.	AK106792	C0J9X4 (UniProt)	ABC transporter-like
Os06g0607750	Hypothetical protein.	BT084685	longestORF	-
Os06g0607800	Similar to 26S proteasome regulatory complex subunit p42D.	AK063158 ,AK066695	C0J9X5 (UniProt)	ATPase, AAA+ type, core
Os06g0607850	Hypothetical protein.	tplb0035c03 (Wheat FLcDNA)	longestORF	-
Os06g0607900	GRAM domain containing protein.	AK070879	C0J9X6 (UniProt)	GRAM
Os06g0608000	Similar to PGPS/D10.	AK119328	A3BDF7 (UniProt)	-
Os06g0608050	Non-protein coding transcript.	AK241542	NONE	-
Os06g0608100	Hypothetical conserved gene.	tplb0012d16 (Wheat FLcDNA)	NP_001058037.2 (RefSeq)	-
Os06g0635700	Conserved hypothetical protein.	AK107643	A3BDV0 (UniProt)	-
Os06g0636100	Hypothetical conserved gene.	CT836245	Q67V17 (UniProt)	Pathogenic type III effector avirulence factor Avr cleavage site
Os06g0636201	Conserved hypothetical protein.	EU968448	NP_001144438.1 (RefSeq)	-
Os06g0636600	Protein kinase, core domain containing protein.	AK119586	NP_001147925.1 (RefSeq)	Protein kinase, catalytic domain
Os06g0636700	Esterase, SGNH hydrolase-type domain containing protein.	AK058562	NP_849451.1 (RefSeq)	Lipase, GDSL
Os06g0636800	Conserved hypothetical protein.	EU970200	NP_001058146.1 (RefSeq)	-
Os06g0637400	Conserved hypothetical protein.	AK066059	A2YFE2 (UniProt)	-
Os06g0637500	Similar to MYB transcription factor R2R3 type.	AK121564 ,AK105817	NP_001159044.1 (RefSeq)	-
Os06g0667900	Disease resistance protein domain containing protein.	ab initio prediction	Q655T3 (UniProt)	Disease resistance protein
Os06g0668000	Hypothetical protein.	AK064023	longestORF	-
Os06g0668200	Similar to Phosphoglycerate kinase, cytosolic (EC 2.7.2.3).	AK070705 ,AK101622	A2YG06 (UniProt)	Phosphoglycerate kinase
Os06g0668250	Hypothetical gene.	BT088042	longestORF	-
Os06g0668300	BTB/POZ-like domain containing protein.	AK375661	B9FQE4 (UniProt)	BTB/POZ-like
Os06g0668400	Kelch related domain containing protein.	AK108036	B9FQE4 (UniProt)	BTB/POZ-like
Os06g0669100	Conserved hypothetical protein.	AK099506	Q653E7 (UniProt)"	-
Os06g0669200	Conserved hypothetical protein.	AK062363	Q5Z8D4 (UniProt)	-
Os06g0669225	Hypothetical conserved gene.	ab initio prediction	Q655S3 (UniProt)	MATH
Os06g0669275	Similar to MEE44 (maternal effect embryo arrest 44)%3B nucleotidyltransferase.	BT018457	NP_191917.2 (RefSeq)	PAP/25A-associated
Os06g0669400	Similar to Cell division protease ftsH homolog 2, chloroplastic.	AK064913	Q655S1 (UniProt)	Peptidase M41
Os06g0669600	Similar to predicted protein.	AK288929	NP_199956.1 (RefSeq)	Conserved oligomeric Golgi complex, subunit 7
Os06g0669700	Similar to DNA binding protein.	AK070871	NP_001149442.1 (RefSeq)	Transcription regulator HTH, Myb-type, DNA-binding
Os06g0669800	Ovarian tumour, otubain domain containing protein.	AK073551	B6U3W2 (UniProt)	Ovarian tumour, otubain
Os06g0670000	Similar to Molybdenum cofactor sulfurase.	AK287475	Q655R6 (UniProt)	Aminotransferase, class V/Cysteine desulfurase
Os06g0670100	Conserved hypothetical protein.	AK102577	A3BEH8 (UniProt)	-
Os06g0697300	Orn/DAP/Arg decarboxylase 2 domain containing protein.	AB332079	NP_001174973.1 (RefSeq)	-
Os06g0697400	Similar to DNA-directed RNA polymerases I, II, and III 17.1 kDa polypeptide.	AK068912	B6SRG8 (UniProt)	RNA polymerase, Rpb8
Os06g0697500	ATPase, AAA-type, core domain containing protein.	BT060613	NP_001149719.1 (RefSeq)	ATPase, AAA+ type, core
Os06g0697600	ATPase, AAA-type, core domain containing protein.	AK287436	NP_001149719.1 (RefSeq)	ATPase, AAA+ type, core
Os06g0698000	Hypothetical conserved gene.	AK242699	B9FQP3 (UniProt)	-
Os06g0698200	Similar to Homeobox-leucine zipper protein HOX18.	A2YGL9 (UniProt)	A2YGL9 (UniProt)	Homeobox
Os06g0698300	Protein phosphatase 2C family protein.	AK071637	A7L5U8 (UniProt)	Protein phosphatase 2C, manganese/magnesium aspartate binding site

Os06g0698400	RNA recognition motif domain domain containing protein.	AK108486	XP_002437521.1 (RefSeq)	RNA recognition motif domain
Os06g0698500	Protein of unknown function DUF1336 domain containing	AK062416	B4F841 (UniProt)	Domain of unknown function DUF1336
Os06g0698600	Exo70 exocyst complex subunit family protein.	AK243691	NP_194882.2 (RefSeq)	Exo70 exocyst complex subunit
Os06g0698674	eIF4-gamma/eIF5/eIF2-epsilon domain containing protein.	AK243382	B6T7C2 (UniProt)	Translation initiation factor IF2/IF5
Os06g0698686	Hypothetical protein.	tplb0060f03 (Wheat FLcDNA)	longestORF	-
Os06g0698711	Conserved hypothetical protein.	AK070810	B8B238 (UniProt)	-
Os06g0698748	Similar to cDNA clone:J023083M02, full insert sequence.	AK105781	B7EQC0 (UniProt)	-
Os06g0698785	Similar to Choline monooxygenase.	AK120445	Q7XB43 (UniProt)	Rieske [2Fe-2S] iron-sulphur domain
Os06g0698802	Hypothetical conserved gene.	AK288331	B9FQP9 (UniProt)	-
Os06g0698812	Disease resistance protein domain containing protein.	ab initio prediction	XP_002438943.1 (RefSeq)	Disease resistance protein
Os06g0698822	Hypothetical protein.	AK241532	longestORF	-
Os06g0698859	Similar to C2H2 zinc-finger protein SERRATE (Fragment).	AK120947	Q94FY3 (UniProt)	Arsenite-resistance protein 2
Os06g0698900	Similar to GATA transcription factor 25.	AK243334	NP_001148559.1 (RefSeq)	Zinc finger, GATA-type
Os06g0699050	Hypothetical protein.	tplb0041e20 (Wheat FLcDNA)	longestORF	-
Os06g0699100	Transferase domain containing protein.	tplb0032h14 (Wheat FLcDNA)	A2YGN1 (UniProt)	Transferase
Os06g0699200	Metallophosphoesterase domain containing protein.	AK243173	NP_001149077.1 (RefSeq)	Metallophosphoesterase domain
Os06g0699301	Hypothetical conserved gene.	ab initio prediction	NP_001174980.1 (RefSeq)	-
Os06g0699400	MAP kinase 2.	AK071376	Q5Z859 (UniProt)	Protein kinase, catalytic domain
Os06g0699500	Tautomerase domain containing protein.	AK120103	NP_001150913.1 (RefSeq)	Macrophage migration inhibitory factor
Os06g0699600	CCT domain containing protein.	AK121295	B8B244 (UniProt)	CCT domain
Os06g0699700	Similar to Aminodeoxychorismate synthase/glutamine amidotransferase.	AK059492	A4UIY9 (UniProt)	ADC synthase
Os06g0699800	ENTH/VHS domain containing protein.	EU969260	Q5Z855 (UniProt)	ENTH/VHS
Os06g0699850	Hypothetical protein.	EU975689	longestORF	-
Os06g0699900	Proteasome/cyclosome, regulatory subunit domain containing	AK242770	XP_002884212.1 (RefSeq)	Proteasome/cyclosome, regulatory subunit
Os06g0700000	Peptidase S8, subtilisin-related domain containing protein.	AK102835	B6SWW5 (UniProt)	Peptidase S8/S53, subtilisin/kexin/sedolisin
Os06g0700100	Pentatricopeptide repeat domain containing protein.	AK119443	A2YGP0 (UniProt)	Pentatricopeptide repeat
Os06g0700300	Conserved hypothetical protein.	AK099967	A2YGP2 (UniProt)	-
Os06g0700500	Protein of unknown function DUF266, plant family protein.	AK072266	Q01KD8 (UniProt)	Glycosyl transferase, family 14
Os06g0707400	Conserved hypothetical protein.	AK066341	B8A944 (UniProt)	-
Os06g0707700	NB-ARC domain containing protein.	AK100720	Q84KC8 (UniProt)	Disease resistance protein
Os06g0707733	Disease resistance protein domain containing protein.	ab initio prediction	A2YGU6 (UniProt)	Disease resistance protein
Os06g0707800	Non-protein coding transcript.	AK111291	NONE	-
Os06g0708000	MAP kinase homolog.	AK066531	Q5Z9J0 (UniProt)	Protein kinase, catalytic domain
Os06g0708050	Hypothetical gene.	EU949346	longestORF	-
Os06g0708075	Non-protein coding transcript.	tplb0059e09 (Wheat FLcDNA)	NONE	-
Os06g0708100	Carboxylesterase, type B family protein.	AK102365	B6UAH8 (UniProt)	Alpha/beta hydrolase fold-3
Os06g0708200	Conserved hypothetical protein.	AK103849	B9FQU2 (UniProt)	-
Os06g0708300	Similar to RER1A protein.	AK243324	NP_001151498.1 (RefSeq)	Retrieval of early ER protein Rer1
Os06g0708400	Cyclophilin.	ab initio prediction	A2YGV1 (UniProt)	Peptidyl-prolyl cis-trans isomerase, cyclophilin-type
Os06g0708500	Similar to Peptidyl-prolyl cis-trans isomerase.	AK060865	A2YGV2 (UniProt)	Peptidyl-prolyl cis-trans isomerase, cyclophilin-type
Os06g0708600	Zinc finger, C2H2-like domain containing protein.	AK100915	E4MXX2 (UniProt)	Zinc finger, C2H2-like
Os06g0708700	Similar to Nodulin-like protein.	AK067151	B6SKJ2 (UniProt)	Drug/metabolite transporter
Os06g0708832	Similar to arogenate dehydrogenase.	AK355541	NP_001147429.1 (RefSeq)	Prephenate dehydrogenase
Os06g0708900	Similar to zinc knuckle (CCHC-type) family protein.	AK100402	NP_193654.2 (RefSeq)	CBF1-interacting co-repressor CIR, N-terminal
Os06g0709000	NAD(P)-binding domain containing protein.	AK068653	NP_001147429.1 (RefSeq)	Prephenate dehydrogenase
Os06g0709100	Cyclin-like F-box domain containing protein.	AK059732 ,AK103683	B6TRX2 (UniProt)	F-box domain, cyclin-like
Os06g0709400	Conserved hypothetical protein.	AK108588	A3CGK2 (UniProt)	-
Os06g0711700	Kelch-type beta propeller domain containing protein.	EU956453	NP_001058552.2 (RefSeq)	Galactose oxidase, beta-propeller
Os06g0711800	Pectinesterase inhibitor domain containing protein.	AK069642	NP_001167668.1 (RefSeq)	Pectinesterase inhibitor
Os06g0711900	Bifunctional inhibitor/plant lipid transfer protein/seed storage domain containing protein.	AK105838	A2YGX0 (UniProt)	Plant lipid transfer protein/seed storage/trypsin-alpha amylase inhibitor
Os06g0712200	NUDIX domain containing protein.	AK061313	A2YGX1 (UniProt)	NUDIX hydrolase domain
Os06g0712250	Hypothetical conserved gene.	CT832454	NP_001144189.1 (RefSeq)	-
Os06g0712300	EF-Hand type domain containing protein.	AK070744	E6NU22 (UniProt)	Phospholipid/glycerol acyltransferase
Os06g0712400	Protein of unknown function DUF544 family protein.	AK067573	B8B2H1 (UniProt)	Ubiquitin interacting motif
Os06g0712500	Similar to Glycosyltransferase QUASIMODO1 (EC 2.4.1.-).	AK068531	E6NU27 (UniProt)	Glycosyl transferase, family 8
Os06g0712550	Hypothetical protein.	tplb0056b02 (Wheat FLcDNA)	longestORF	-
Os06g0712600	Similar to SHI.	EU959412	NP_001150905.1 (RefSeq)	Zinc finger, lateral root primordium type 1
Os06g0712700	MADS-box protein SPW1.	AK069317	Q944S9 (UniProt)	Transcription factor, MADS-box
Os06g0712800	Similar to Ankyrin-like protein.	AK121236	NP_190676.1 (RefSeq)	Protein of unknown function DUF248, methyltransferase putative
Os06g0712900	tRNA-dihydrouridine synthase domain containing protein.	AK106648	NP_201523.1 (RefSeq)	tRNA-dihydrouridine synthase
Os06g0713000	Zinc finger, B-box domain containing protein.	AK105957	NP_001150747.1 (RefSeq)	Zinc finger, B-box
Os06g0713100	Protein of unknown function DUF1640 family protein.	AK072606	B6UC57 (UniProt)	Protein of unknown function DUF1640
Os06g0713201	Non-protein coding transcript.	BT087005	NONE	-
Os06g0713300	Conserved hypothetical protein.	AK106687	A3BFC6 (UniProt)	-
Os06g0713400	Cyclin-like F-box domain containing protein.	AK100628	B9FQV7 (UniProt)	F-box domain, cyclin-like
Os06g0713600	Non-protein coding transcript.	tplb0032e04 (Wheat FLcDNA)	NONE	-
Os06g0713800	Alpha-amylase isozyme 2A precursor (EC 3.2.1.1) (1,4-alpha-D-glucan glucanohydrolase).	AK059671 ,AK101018	Q0D9J1 (UniProt)	Glycoside hydrolase, family 13
Os06g0713900	Hypothetical conserved gene.	AK242112	A2YGY3 (UniProt)	-
Os06g0714000	Uncharacterised protein family UPF0183 domain containing	AK069538	XP_002439019.1 (RefSeq)	Uncharacterised protein family UPF0183
Os06g0714100	Complex 1 LYR protein family protein.	AK121079	NP_001148390.1 (RefSeq)	-
Os06g0714200	Similar to calcium dependent protein kinase1.	AK243187	NP_001105740.1 (RefSeq)	Protein kinase, catalytic domain
Os07g0124500	Similar to Eukaryotic translation initiation factor 3 subunit 8 (eIF3 p110) (eIF3c).	AK070529 ,AK101000	Q1SL20 (UniProt)	Proteasome component
Os07g0124600	Nucleotide-binding, alpha-beta plait domain containing protein.	AK073437	A2YHR7 (UniProt)	RNA recognition motif domain
Os07g0124650	Hypothetical gene.	EU942577	longestORF	-
Os07g0124700	Similar to PLETHORA1.	AK109848	Q84Z02 (UniProt)	Pathogenesis-related transcriptional factor/ERF, DNA-binding
Os07g0124725	Hypothetical protein.	EU949353	longestORF	-
Os07g0124750	Similar to cDNA clone:J033097L05, full insert sequence.	AK358114	B7F7S0 (UniProt)	Glycosyl transferase, family 2
Os07g0124800	Similar to Chaperone protein dnaJ.	AK068837	B6U0R7 (UniProt)	Heat shock protein DnaJ, N-terminal
Os07g0124900	Allergen V5/Tpx-1 related family protein.	AK060057	D5KR57 (UniProt)	Allergen V5/Tpx-1-related
Os07g0125000	Allergen V5/Tpx-1 related family protein.	AK060005 ,AK104140	Q8LLU7 (UniProt)	-
Os07g0125201	Allergen V5/Tpx-1 related family protein.	DQ167191	O04000 (UniProt)	Allergen V5/Tpx-1-related
Os07g0125500	Allergen V5/Tpx-1 related family protein.	AK060057	D5KR57 (UniProt)	Allergen V5/Tpx-1-related
Os07g0125600	Allergen V5/Tpx-1 related family protein.	AK060005 ,AK104140	Q8LLU7 (UniProt)	-
Os07g0126100	Allergen V5/Tpx-1 related family protein.	DQ167191	O04000 (UniProt)	Allergen V5/Tpx-1-related
Os07g0126301	Allergen V5/Tpx-1 related family protein.	AK060057	D5KR57 (UniProt)	Allergen V5/Tpx-1-related
Os07g0126401	Allergen V5/Tpx-1 related family protein.	AK060005 ,AK104140	Q8LLU7 (UniProt)	-
Os07g0126500	Allergen V5/Tpx-1 related family protein.	DQ167191	O04000 (UniProt)	Allergen V5/Tpx-1-related
Os07g0127500	Similar to PR-1a pathogenesis related protein (Hv-1a) precursor.	AK062949	C3UZE5 (UniProt)	Allergen V5/Tpx-1-related
Os07g0127600	Allergen V5/Tpx-1 related family protein.	AK063248	Q8LLU7 (UniProt)	Allergen V5/Tpx-1-related
Os07g0155600	Nramp ion-transporter family protein, Ethylene signaling pathway	AY396568	Q6TJY0 (UniProt),Q0D8I9 (	-
Os07g0156200	Haem peroxidase, plant/fungal/bacterial family protein.	AK249509	NP_001152255.1 (RefSeq)	Plant peroxidase
Os07g0156467	Similar to Class III peroxidase 7.	ab initio prediction	Q5U1T6 (UniProt)	Plant peroxidase
Os07g0156732	Similar to EIN2.	AK111802	Q6TJY0 (UniProt)	Natural resistance-associated macrophage protein
Os07g0156910	Similar to peroxidase 1.	ab initio prediction	NP_001152255.1 (RefSeq)	Haem peroxidase, plant/fungal/bacterial
Os07g0157000	Similar to Class III peroxidase 7.	FP094284	Q5U1T6 (UniProt)	Plant peroxidase
Os07g0157401	Similar to EIN2.	AK111802	Q6TJY0 (UniProt)	Natural resistance-associated macrophage protein
Os07g0157600	Similar to peroxidase 1.	AK252026	NP_001152255.1 (RefSeq)	Plant peroxidase
Os07g0157700	Conserved hypothetical protein.	AK069862	B8B7D5 (UniProt)	-
Os07g0564000	Conserved hypothetical protein.	AK069806	B8B7G5 (UniProt)	-
Os07g0564100	UDP-glucuronosyl/UDP-glucosyltransferase family protein.	AK107291	NP_001148465.1 (RefSeq)	UDP-glucuronosyl/UDP-glucosyltransferase
Os07g0564150	Hypothetical gene.	EU956579	longestORF	-
Os07g0564200	Conserved hypothetical protein.	AK072771	A2YMP3 (UniProt)	-
Os07g0564500	Pyridine nucleotide-disulphide oxidoreductase, NAD-binding region domain containing protein.	AK121213	NP_563783.1 (RefSeq)	Pyridine nucleotide-disulphide oxidoreductase, NAD-binding domain
Os07g0564533	Similar to HAT family dimerisation domain containing protein.	AK288170	Q53RM1 (UniProt)	HAT dimerisation
Os07g0564566	Non-protein coding transcript.	CU406694	NONE"	-
Os07g0564600	Similar to Secretory carrier membrane protein.	AK102110	Q8H5X5 (UniProt)	SCAMP
Os07g0564700	Similar to ATMIN7 (ARABIDOPSIS THALIANA HOPM INTERACTOR 7)%3B guanyl-nucleotide exchange factor/ protein binding.	AK059018	NP_189916.4 (RefSeq)	-
Os07g0564750	Conserved hypothetical protein.	FP095875	A2XXG5 (UniProt)	-
Os07g0564800	Protein of unknown function DUF707 family protein.	AK100860	Q8H767 (UniProt)	Protein of unknown function DUF707
Os07g0564901	Non-protein coding transcript.	BT086289	NONE	-
Os07g0565000	Similar to 40S ribosomal protein S11.	AK121056	NP_001105562.1 (RefSeq)	Ribosomal protein S17
Os07g0565100	Similar to 40S ribosomal protein S11.	CT833537	NP_001105562.1 (RefSeq)	Ribosomal protein S17
Os07g0565200	Similar to Cell differentiation protein rcd1.	AK106782	B6TRX0 (UniProt)	Cell differentiation, Rcd1-like
Os07g0565300	Bromodomain containing protein.	AK101955	A2YMP9 (UniProt)	Bromodomain
Os07g0565350	Hypothetical protein.	tplb0052b05 (Wheat FLcDNA)	longestORF	-
Os07g0565400	Similar to SRF8 (STRUBBELIG-RECEPTOR FAMILY 8).	AK242465	NP_001119030.1 (RefSeq)	Protein kinase, catalytic domain
Os07g0565500	Conserved hypothetical protein.	AK107834	F2DAZ7 (UniProt)	-

Os07g0565600	Similar to predicted protein.	AK107481	XP_002885091.1 (RefSeq)	Peptidyl-prolyl cis-trans isomerase, cyclophilin-type
Os07g0565700	Alpha/beta hydrolase family protein.	AK112115	XP_002889847.1 (RefSeq)	Alpha/beta hydrolase fold-1
Os07g0565800	Similar to LLA-115.	AK062834	B2BA79 (UniProt)	-
Os07g0567250	Hypothetical protein.	tplb0054h01 (Wheat FLcDNA)	longestORF	-
Os07g0567300	Hypothetical conserved gene.	AK288489	Q84SM4 (UniProt)	Glycosyltransferase, DXD sugar-binding motif
Os07g0567400	Similar to Cytochrome c6.	AK068245	B6TIZ9 (UniProt)	Cytochrome c domain
Os07g0567500	Similar to predicted protein.	AK373391	Q84SM2 (UniProt)	-
Os07g0567700	Similar to SCARECROW.	AK061242	Q6ULS4 (UniProt)	Transcription factor GRAS
Os07g0567801	Conserved hypothetical protein.	AK242924	NP_001172118.1 (RefSeq)	-
Os07g0567900	Conserved hypothetical protein.	FP097526	B9FXZ7 (UniProt)	-
Os07g0568000	Apolipophorin III-like domain containing protein.	AK072783	B8B7H7 (UniProt)	Cullin repeat-like-containing domain
Os07g0568100	LRR protein kinase, Common symbiosis signaling (SYM) [illegible]	AK099778	Q7F1I0 (UniProt)	Protein kinase, catalytic domain
Os07g0568200	Homeodomain-related containing protein.	AK102232	XP_002463045.1 (RefSeq)	Homeodomain-like
Os07g0568300	Similar to ZF protein (Fragment).	AK067895	Q84SL2 (UniProt)	Zinc finger, CCCH-type
Os07g0568400	TB2/DP1 and HVA22 related protein family protein.	AK241061	A2YMS5 (UniProt)	TB2/DP1/HVA22-related protein
Os07g0568500	Peptidase aspartic, active site domain containing protein.	AK100091	B6SLR0 (UniProt)	Uncharacterised protein family Ycf60
Os07g0568600	Similar to Calcium-dependent protein kinase.	AK068315	B6SKK9 (UniProt)	Protein kinase, catalytic domain
Os07g0568650	Hypothetical protein.	tplb0061j19 (Wheat FLcDNA)	longestORF	-
Os07g0568700	Polygalacturonase inhibitor 1 precursor (Polygalacturonase-inhibiting protein) (Floral organ regulator 1).	AK101897 ,AK061685	Q8GT95 (UniProt)	-
Os07g0568800	Hypothetical protein.	AK111260	longestORF	-
Os07g0568900	Conserved hypothetical protein.	AK062660	B8B7I1 (UniProt)	-
Os07g0569000	Conserved hypothetical protein.	AK073915	B8B7I2 (UniProt)	-
Os07g0569100	Remorin, C-terminal region domain containing protein.	AK120160	NP_001150312.1 (RefSeq)	Remorin, C-terminal
Os07g0569166	Conserved hypothetical protein.	AK240724	NP_001175264.1 (RefSeq)	-
Os07g0571100	Conserved hypothetical protein.	AK119301	A3BLC4 (UniProt)	-
Os07g0571200	Hypothetical protein.	BT067768	longestORF	-
Os07g0571300	Hypothetical protein.	AK107499	longestORF	-
Os07g0571500	Similar to Transmembrane protein 49.	CT831667	B6TDW5 (UniProt)	SNARE associated Golgi protein
Os07g0571600	Chalcone isomerase domain containing protein.	CT837974	NP_001149585.1 (RefSeq)	Chalcone isomerase, subgroup
Os07g0571700	Similar to Transporter-like protein.	AK067178	NP_001151801.1 (RefSeq)	General substrate transporter
Os07g0571800	Similar to Protein YABBY 7.	AK072618	A2PZN8 (UniProt)	YABBY protein
Os07g0571900	Prp18 domain containing protein.	AK101604	B6T2W8 (UniProt)	Splicing factor motif
Os07g0572000	WD40/YVTN repeat-like domain containing protein.	AK111653	A2YMV0 (UniProt)	WD40 repeat
Os07g0572050	Similar to Copper amine oxidase.	ab initio prediction	B7ZYE7 (UniProt)	Copper amine oxidase
Os07g0572075	Hypothetical protein.	BT063833	longestORF	-
Os07g0572100	Similar to Amine oxidase like protein (EC 1.4.3.6) (Copper amine oxidase).	AK099435	B7ZYE7 (UniProt)	Copper amine oxidase
Os07g0572300	Protein of unknown function DUF868, plant family protein.	AK074013	A2YMV3 (UniProt)	Protein of unknown function DUF868, plant
Os07g0572400	Conserved hypothetical protein.	AK067317	B8B7T8 (UniProt)	-
Os07g0572500	Conserved hypothetical protein.	AK108612	B8B7T9 (UniProt)	-
Os07g0572600	Similar to F20D23.3 protein.	AK109147 ,AK072023	Q9SHI5 (UniProt)	GDP-fucose protein O-fucosyltransferase
Os07g0572800	Similar to MAP kinase-like protein.	AK100426	C0M0P2 (UniProt)	Protein kinase, catalytic domain
Os07g0572850	Non-protein coding transcript.	tplb0061f07 (Wheat FLcDNA)	NONE	-
Os07g0572900	Similar to 40S ribosomal protein S13.	AK063015	B4FML8 (UniProt)	Ribosomal protein S15
Os07g0573000	Conserved hypothetical protein.	AK120026	A3BLD8 (UniProt)	-
Os07g0573100	Similar to Adenylyl-sulfate kinase 1, chloroplast precursor (EC 2.7.1.25) (APS kinase) (Adenosine-5'phosphosulfate kinase) (ATP adenosine-5'- phosphosulfate 3'-phosphotransferase).	AK071285	B8B7U1 (UniProt)	Adenylylsulphate kinase, C-terminal
Os07g0573200	Similar to Adenylyl-sulfate kinase.	CT836177	B8B7U1 (UniProt)	-
Os07g0573300	FYVE finger-containing phosphoinositide kinase (EC 2.7.1.68) (1- phosphatidylinositol-4-phosphate 5-kinase) (PIP5K) (PtdIns(4)P-5- kinase) (PIKfyve) (p235). Splice isoform p235S.	AK071682	Q6ZL20 (UniProt)	Zinc finger, FYVE-type
Os07g0573400	Protein of unknown function DUF239, plant domain containing protein.	AK061739	NP_001152099.1 (RefSeq)	Glucoamylase, putatuve
Os07g0573450	Hypothetical protein.	tplb0062i11 (Wheat FLcDNA)	longestORF	-
Os07g0685800	Short-chain dehydrogenase/reductase SDR domain containing protein.	AK064532	B8B650 (UniProt)	Short-chain dehydrogenase/reductase SDR
Os07g0685900	Peptidase S8, subtilisin-related domain containing protein.	AK119348	Q8LIH3 (UniProt)	Peptidase S8/S53, subtilisin/kexin/sedolisin
Os07g0686100	Similar to Abscisic acid responsive elements-binding factor.	AK110915	Q7XIR0 (UniProt)	Basic-leucine zipper
Os07g0686300	Zinc finger, RING/FYVE/PHD-type domain containing protein.	AK062636	NP_176569.1 (RefSeq)	Zinc finger, RING-type
Os07g0686366	Non-protein coding transcript.	CT836160	NONE"	-
Os07g0686400	Conserved hypothetical protein.	FP101530	NP_001060683.2 (RefSeq)	-
Os07g0686500	Protein of unknown function DUF632 domain containing protein.	AK119424	Q8LIG9 (UniProt)	Domain of unknown function DUF632
Os07g0686600	VQ domain containing protein.	AK108527	A2YQ31 (UniProt)	VQ
Os07g0686700	Conserved hypothetical protein.	AK067620	A3BNK3 (UniProt)	-
Os07g0686800	Similar to Serine/threonine protein kinase-like.	AK106421	NP_001147807.1 (RefSeq)	Protein kinase, catalytic domain
Os07g0686825	Hypothetical protein.	EU951577	longestORF	-
Os07g0686900	Similar to Alpha-L-arabinofuranosidase C-terminus family protein, expressed.	AK064838	Q10M79 (UniProt)	-
Os07g0686950	Hypothetical gene.	AK241535	longestORF	-
Os07g0687000	InterPro=Protein kinase, catalytic domain (IPR000719),Serine/threonine-protein kinase, catalytic domain (IPR002290),NAF domain (IPR004041),Serine/threonine-protein kinase, active site (IPR008271),Protein kinase-like domain (IPR011009),Protein kinase, ATP binding site (IPR017441),Serine/threonine-protein kinase-like domain (IPR017442),NAF/FISL domain (IPR018451),Tyrosine-protein kinase, catalytic domain (IPR020635)	GO=Biological Process: protein ph	KEGG=Os07t0687000-00 (E	-
Os07g0687100	Protein of unknown function DUF341 family protein.	AK066780	E4MW38 (UniProt)	Serine hydrolase FSH
Os07g0687200	Similar to Auxin-regulated calmodulin.	AK063566	NP_001167666.1 (RefSeq)	EF-hand-like domain
Os07g0687300	Similar to SNF1 kinase complex anchoring protein (Fragment).	AK073043	NP_001149540.1 (RefSeq)	5-AMP-activated protein kinase, beta subunit, interaction domain
Os07g0687400	Similar to VQ motif family protein, expressed.	CT836093	Q10M91 (UniProt)	VQ
Os07g0687500	Rhodanese-like domain containing protein.	AK073511	B6SS56 (UniProt)	Peptidyl-prolyl cis-trans isomerase, PpiC-type
Os07g0687700	Similar to Transcription factor HBP-1b(C38) (Fragment).	AK100440	Q8W5R9 (UniProt)	Basic-leucine zipper
Os07g0687900	WSI76 protein induced by water stress.	AK107065 ,AK099548	B4G0Z8 (UniProt)	-
Os07g0688000	Metallophosphoesterase domain containing protein.	AK069836	NP_974609.1 (RefSeq)	Metallophosphoesterase domain
Os07g0688100	Pentatricopeptide repeat domain containing protein.	AK101635	B8B6C4 (UniProt)	Pentatricopeptide repeat
Os07g0688200	Similar to Typical P-type R2R3 Myb protein (Fragment).	AK103455	Q8S3Y6 (UniProt)	SANT domain, DNA binding
Os07g0688300	Similar to Importin alpha 1.	AK068325	NP_001149981.1 (RefSeq)	Armadillo
Os07g0688500	Similar to Poly.	CT836073	B6UBW8 (UniProt)	Nucleotidyl transferase domain
Os07g0688700	Hypothetical protein.	AK360624	GeneMark	-
Os07g0688800	Aldehyde dehydrogenase domain containing protein.	AK068462	A7LHM3 (UniProt)	Aldehyde dehydrogenase domain
Os07g0689150	Similar to BADH-like protein.	ab initio prediction	A7LHM3 (UniProt)	Peptidase S16, Lon C-terminal
Os07g0689300	Similar to Lon protease homolog, mitochondrial.	AK068511	A2YQ56 (UniProt)	ATPase, AAA-type, core
Os07g0689400	Similar to predicted protein.	AK120218	NP_850473.1 (RefSeq)	-
Os07g0689600	Nicotianamine synthase 3 (EC 2.5.1.43) (S-adenosyl-L-methionine:S- adenosyl-L-methionine:S-adenosyl-methionine 3-amino-3- carboxypropyltransferase 3) (OsNAS3).	AK070656	Q0D3F2 (UniProt)	Nicotianamine synthase
Os07g0696100	Conserved hypothetical protein.	AK071018	B8BN83 (UniProt)	-
Os08g0124500	Similar to Resistance protein candidate (Fragment).	AK059130	B7F8F0 (UniProt)	Protein kinase, catalytic domain
Os08g0124533	Hypothetical protein.	tplb0022l18 (Wheat FLcDNA)	longestORF	-
Os08g0124566	Hypothetical protein.	tplb0022l18 (Wheat FLcDNA)	longestORF	-
Os08g0124600	Similar to J065032N17, full insert sequence.	AK369941	B7F8F0 (UniProt)	Protein kinase, catalytic domain
Os08g0124651	Conserved hypothetical protein.	EU974589	Q6ZFR7 (UniProt)	-
Os08g0124700	Similar to Resistance protein candidate (Fragment).	AK106798	B7F8F0 (UniProt)	Protein kinase, catalytic domain
Os08g0124750	Hypothetical protein.	tplb0022l18 (Wheat FLcDNA)	longestORF	-
Os08g0124850	Hypothetical protein.	tplb0022l18 (Wheat FLcDNA)	longestORF	-
Os08g0125006	Hypothetical protein.	tplb0022l18 (Wheat FLcDNA)	longestORF	-
Os08g0125059	Hypothetical protein.	tplb0022l18 (Wheat FLcDNA)	longestORF	-
Os08g0161700	NADH dehydrogenase [ubiquinone] (complex I), iron-sulphur protein 6, mitochondria domain containing protein.	AK059272	NP_001151169.1 (RefSeq)	NADH dehydrogenase [ubiquinone]
Os08g0161750	Similar to Secretory carrier-associated membrane protein 2.	AK372856	B7X6S6 (UniProt)	SCAMP
Os08g0161800	Similar to Choline-phosphate cytidylyltransferase B.	AK120881	B6U468 (UniProt)	-
Os08g0161900	Molecular chaperone, heat shock protein, Hsp40, DnaJ domain containing protein.	AK240890	XP_002886287.1 (RefSeq)	Heat shock protein DnaJ, N-terminal
Os08g0161950	Hypothetical protein.	tplb0026i01 (Wheat FLcDNA)	longestORF	-
Os08g0162000	Similar to transmembrane 9 superfamily protein member 1.	AK119463	NP_001148367.1 (RefSeq)	Nonaspanin
Os08g0162033	Similar to 50S ribosomal protein L13.	BT040800	B6TQ75 (UniProt)	Ribosomal protein L13
Os08g0162066	Conserved hypothetical protein.	AK288660	Q6Z3B0 (UniProt)	-
Os08g0162100	Similar to CTV.2.	AK111830	NP_001167872.2 (RefSeq)	WD40 repeat
Os08g0162200	Pentatricopeptide repeat domain containing protein.	AK106876	F2E8M6 (UniProt)	Pentatricopeptide repeat
Os08g0162250	Hypothetical conserved gene.	EU970947	A3BPW9 (UniProt)	-
Os08g0162300	Hypothetical protein.	AK067263	longestORF	-

Os08g0162400	Zinc finger, RING/FYVE/PHD-type domain containing protein.	AK063453	B6TDS7 (UniProt)	Zinc finger, RING-type
Os08g0162500	Conserved hypothetical protein.	AK121633	B8BAY8 (UniProt)	-
Os08g0162600	Rubredoxin-type Fe(Cys)4 protein family protein.	AK060121	NP_568342.1 (RefSeq)	PDZ/DHR/GLGF
Os08g0162650	Hypothetical gene.	BT038966	longestORF	-
Os08g0162700	Aldehyde dehydrogenase, conserved site domain containing	AK062732	A3BPX3 (UniProt)	-
Os08g0162800	Similar to Acyl-CoA-binding protein.	AK122061 ,AK059406	B4FER8 (UniProt)	Acyl-CoA-binding protein, ACBP
Os08g0162900	Similar to 60S ribosomal protein L13.	AJ222784	Q8S9G2 (UniProt)	-
Os08g0282700	Hypothetical gene.	AK071808	longestORF	-
Os08g0283000	Similar to H0315A08.1 protein.	FP099421	Q01I63 (UniProt)	Ribonuclease H1, N-terminal
Os08g0283300	Similar to H0315A08.1 protein.	FP099421	Q01I63 (UniProt)	Ribonuclease H1, N-terminal
Os08g0283600	Similar to H0315A08.1 protein.	FP099421	Q01I63 (UniProt)	Ribonuclease H1, N-terminal
Os08g0283900	Similar to H0315A08.1 protein.	FP099421	Q01I63 (UniProt)	Ribonuclease H1, N-terminal
Os08g0284200	Similar to H0315A08.1 protein.	FP099421	Q01I63 (UniProt)	Ribonuclease H1, N-terminal
Os08g0284500	Similar to H0315A08.1 protein.	FP099421	Q01I63 (UniProt)	Ribonuclease H1, N-terminal
Os08g0358800	A-type response regulator, Cytokinin signaling	BR000320	Q6YZK8 (UniProt)	-
Os08g0359000	Similar to APE1.	AK066474	B6TPW9 (UniProt)	Protein of unknown function DUF2854
Os08g0359050	Hypothetical protein.	FP091988	longestORF	-
Os08g0359100	Similar to Lipid phosphate phosphatase 2 (EC 3.1.3.-) (AtLPP2) (Phosphatidic acid phosphatase 2) (AtPAP2) (Prenyl diphosphate phosphatase).	AK067740	B6SSC1 (UniProt)	Phosphatidic acid phosphatase type 2/haloperoxidase
Os08g0359200	Hypothetical gene.	AK060942	longestORF	-
Os08g0359300	Uncharacterised conserved protein UCP031088, alpha/beta hydrolase, At1g15070 domain containing protein.	AK071671	E4MY19 (UniProt)	Peptidase S9, serine active site
Os08g0359400	Hypothetical protein.	tplb0032d16 (Wheat FLcDNA)	longestORF	-
Os08g0359500	CS domain domain containing protein.	AK112034 ,AK104798	P0C8Z0 (UniProt)	CS-like domain
Os08g0359600	Similar to 30S ribosomal protein S17.	AK070753	B6TH50 (UniProt)	Ribosomal protein S17
Os08g0359900	Conserved hypothetical protein.	tplb0001k01 (Wheat FLcDNA)	NP_001061633.1 (RefSeq)	-
Os08g0360000	Conserved hypothetical protein.	AK111240	B8BA16 (UniProt)	-
Os08g0360100	RNA-binding, CRM domain domain containing protein.	AK066365	NP_001105008.1 (RefSeq)	RNA-binding, CRM domain
Os08g0360150	Hypothetical protein.	tplb0062e03 (Wheat FLcDNA)	longestORF	-
Os08g0360200	Conserved hypothetical protein.	ab initio prediction	Q6YYA2 (UniProt)	-
Os08g0467100	Zinc finger, C2H2 domain containing protein.	AK119595	Q6YSA1 (UniProt)	Zinc finger, C2H2
Os08g0467201	Hypothetical protein.	BT086922	longestORF	-
Os08g0467300	Clathrin adaptor, phosphoinositide-binding, GAT-like domain containing protein.	AK105541	NP_001151341.1 (RefSeq)	ENTH/VHS
Os08g0467400	Zinc/iron permease family protein.	AK070501	XP_002311552.1 (RefSeq)	Zinc/iron permease
Os08g0467500	TB2/DP1 and HVA22 related protein family protein.	AK064041	Q07764 (UniProt)	TB2/DP1/HVA22-related protein
Os08g0467600	Hypothetical conserved gene.	AK370590	Q6YSC2 (UniProt)	Protein of unknown function DUF573
Os08g0468100	Similar to Nitrate reductase.	AK121810	P16081 (UniProt)	Oxidoreductase, molybdopterin-binding domain
Os08g0468200	Hypothetical protein.	AK107363	longestORF	-
Os08g0468300	Conserved hypothetical protein.	ab initio prediction	NP_001175616.1 (RefSeq)	-
Os08g0468400	Kinetochore protein Ndc80 domain containing protein.	AK063363	B6U218 (UniProt)	Kinetochore protein Ndc80
Os08g0468700	Similar to Nitrate reductase [NADH] 1 (EC 1.7.1.1) (NR1).	AK121810	P16081 (UniProt)	Oxidoreductase, molybdopterin-binding domain
Os08g0468801	Hypothetical protein.	AK107363	longestORF	-
Os08g0477800	PWWP domain containing protein.	AK066304	B8BBS0 (UniProt)	PWWP
Os08g0477900	Helix-loop-helix DNA-binding domain containing protein.	EU968808	NP_001159035.1 (RefSeq)	Helix-loop-helix DNA-binding
Os08g0478000	Similar to mucin-2.	AK059493	B6SR31 (UniProt)	-
Os08g0478100	Uncharacterised protein family UPF0029, N-terminal domain containing protein.	AK063259	B8BBS2 (UniProt)	Impact, N-terminal
Os08g0478200	Similar to ATP synthase D chain, mitochondrial (EC 3.6.3.14).	AK068050	NP_001150316.1 (RefSeq)	ATPase, F0 complex, subunit D, mitochondrial
Os08g0478466	Protein of unknown function DUF296 domain containing protein.	AK287800	NP_001152438.1 (RefSeq)	Domain of unknown function DUF296
Os08g0478500	Peptidase C19, ubiquitin carboxyl-terminal hydrolase 2 family	AK099704	A2YWA2 (UniProt)	Peptidase C19, ubiquitin carboxyl-terminal hydrolase 2
Os08g0478566	Similar to Maturase K 2.	AY964640	C6ES29 (UniProt)	-
Os08g0478700	Similar to Mitochondrial uncoupling protein 4.	AK058489	Q66PX4 (UniProt)	Mitochondrial substrate/solute carrier
Os08g0478800	Phosphoglucose isomerase (PGI) family protein.	AK107494	A3BU52 (UniProt)	Phosphoglucose isomerase
Os08g0479300	Cyclin, A/B/D/E domain containing protein.	AK070025	Q4KYM5 (UniProt)	Cyclin, C-terminal
Os08g0479400	Similar to Hydroxyproline-rich glycoprotein DZ-HRGP	AK109528	B6TE62 (UniProt)	Homeobox domain, ZF-HD class
Os08g0486100	Similar to Potential copper-transporting ATPase PAA1 (EC	AK059217	XP_002304082.1 (RefSeq)	ATPase, P-type, H+ transporting proton pump
Os08g0486200	Similar to Splicing factor SC35.	AK103676	B6UG76 (UniProt)	RNA recognition motif domain
Os08g0486233	Similar to EIL transcription factor.	CT835917	Q8W3L9 (UniProt)	Ethylene insensitive 3-like protein, DNA-binding domain
Os08g0486266	Conserved hypothetical protein.	AK288793	Q53LF9 (UniProt)	-
Os08g0486300	Similar to P-type R2R3 Myb protein (Fragment).	BT033636	Q8S416 (UniProt)	SANT domain, DNA binding
Os08g0486400	Hypothetical protein.	EU971393	longestORF	-
Os08g0486500	Conserved hypothetical protein.	AK111410	B9G1H9 (UniProt)	-
Os08g0486700	Conserved hypothetical protein.	AK241195	NP_001062097.1 (RefSeq)	-
Os08g0486750	Non-protein coding transcript.	AK363293	NONE	-
Os08g0486801	Non-protein coding transcript.	AK241869	NONE	-
Os08g0486867	Conserved hypothetical protein.	AK243168	Q75LB0 (UniProt)	-
Os08g0486933	Similar to Peptide transporter-like protein.	ab initio prediction	Q6YS05 (UniProt)	-
Os08g0487000	Hypothetical conserved gene.	AK110869	B8BBV9 (UniProt)	-
Os08g0487050	Hypothetical protein.	EU946896	longestORF	-
Os08g0487100	Similar to BZIP transcription factor BZI-2.	AK107150	NP_001152649.1 (RefSeq)	Basic-leucine zipper
Os08g0487400	Conserved hypothetical protein.	EU949564	B8B3K1 (UniProt)	-
Os08g0487500	Zinc finger, RING/FYVE/PHD-type domain containing protein.	AK066326	B6U108 (UniProt)	Zinc finger, RING-type
Os08g0487700	Helix-loop-helix DNA-binding domain containing protein.	ab initio prediction	NP_001062102.1 (RefSeq)	Helix-loop-helix DNA-binding
Os08g0487800	Similar to Heat-shock protein precursor.	AK122102	Q43638 (UniProt)	Heat shock protein Hsp90
Os08g0487850	Non-protein coding transcript.	BT017162	NONE	-
Os08g0487900	Esterase/lipase/thioesterase domain containing protein.	AK109668	NP_001150405.1 (RefSeq)	-
Os08g0493800	Protein kinase, core domain containing protein.	AK110374	Q6Z8S8 (UniProt)	Protein kinase, catalytic domain
Os08g0493900	WD40 repeat-like domain containing protein.	AK111820	XP_002894196.1 (RefSeq)	WD40 repeat
Os08g0494000	Harpin-induced 1 domain containing protein.	AK110788	NP_001148671.1 (RefSeq)	Late embryogenesis abundant protein, LEA-14
Os08g0494100	Transcription factor, MADS-box domain containing protein.	ab initio prediction	Q6Z5F8 (UniProt)	Transcription factor, MADS-box
Os08g0494200	Conserved hypothetical protein.	AK100830	B8BC24 (UniProt)	-
Os08g0494300	Copine domain containing protein.	AK066150	NP_001147447.1 (RefSeq)	Zinc finger, RING-type
Os08g0494350	Pentatricopeptide repeat domain containing protein.	ab initio prediction	B8BC26 (UniProt)	Pentatricopeptide repeat
Os08g0494375	Conserved hypothetical protein.	ab initio prediction	B8B4W8 (UniProt)	-
Os08g0494400	Conserved hypothetical protein.	AK065332	B9G1K3 (UniProt)	-
Os08g0495300	Similar to cDNA clone:J013000K10, full insert sequence.	AK287721	B9G1K9 (UniProt)	Protein of unknown function DUF3615
Os08g0495500	Kelch related domain containing protein.	AK100472	NP_001150189.1 (RefSeq)	BTB/POZ-like
Os09g0512950	Hypothetical gene.	AK287470	longestORF	-
Os09g0513000	Similar to TGB12K interacting protein 3.	AK062078 ,AK098858 ,AK12137	B7EN30 (UniProt)	Ankyrin repeat
Os09g0513100	Similar to Phospholipase A1.	AK120151	B6T3X6 (UniProt)	Lecithin:cholesterol/phospholipid:diacylglycerol acyltransferase
Os09g0513200	Similar to Solute carrier family 35, member F1.	BT039861	B6U001 (UniProt)	Protein of unknown function DUF914, eukaryotic
Os09g0513400	Hypothetical protein.	AK119919	longestORF	-
Os09g0513500	Similar to ZCN24.	CT841571	A9LLZ6 (UniProt)	Phosphatidylethanolamine-binding, conserved site
Os09g0513600	Similar to predicted protein.	AK242851	Q84P43 (UniProt)	Protein kinase, catalytic domain
Os09g0513700	Nucleotide-binding, alpha-beta plait domain containing protein.	AK071999	B8BDI3 (UniProt)	RNA recognition motif domain
Os09g0513800	Similar to H0425E08.1 protein.	AK106673	Q01IA7 (UniProt)	Zinc finger, RanBP2-type
Os09g0513850	Non-protein coding transcript.	AM939994	NONE	-
Os09g0513900	Conserved hypothetical protein.	AK107699	B9G4J1 (UniProt)	-
Os09g0514100	Similar to oxidoreductase.	AK106812	NP_001147594.1 (RefSeq)	FAD dependent oxidoreductase
Os09g0514200	Similar to Calcium-dependent protein kinase.	AK100474	B3GN93 (UniProt)	Protein kinase, catalytic domain
Os09g0514300	Similar to predicted protein.	AK287431	Q01IA4 (UniProt)	-
Os09g0514350	Hypothetical protein.	EU949684	longestORF	-
Os09g0514400	Similar to Protein farnesyltransferase/geranylgeranyltransferase type I alpha subunit (EC 2.5.1.58) (EC 2.5.1.59) (CAAX farnesyltransferase alpha subunit) (Ras proteins prenyltransferase alpha) (FTase-alpha) (Type I protein geranyl-geranyltransferase alpha subunit) (GGTase-I-alpha).	AK060892	B6TEY3 (UniProt)	Protein prenyltransferase, alpha subunit
Os09g0514500	Conserved hypothetical protein.	AB097944	A3C0L5 (UniProt)	-
Os09g0514550	Hypothetical protein.	tplb0027e15 (Wheat FLcDNA)	longestORF	-
Os09g0514600	Beta-grasp fold, ferredoxin-type domain containing protein.	CU861778	B6SLA5 (UniProt)	Ferredoxin
Os09g0514700	Conserved hypothetical protein.	AK106676	B9G4J7 (UniProt)	-
Os09g0514900	Basic helix-loop-helix, Nulp1-type domain containing protein.	AK102371	B8BDI6 (UniProt)	Basic helix-loop-helix, Nulp1-type
Os09g0515100	Similar to bromodomain protein 103.	AK110315	NP_001105102.1 (RefSeq)	ATPase, AAA+ type, core
Os09g0515200	Beta 7 subunit of 20S proteasome.	AK071380 ,AK103975	B9G4J9 (UniProt)	Proteasome, subunit alpha/beta
Os09g0515300	Conserved hypothetical protein.	AK102157	B9G4K0 (UniProt)	-
Os09g0515400	Trigger factor, ribosome-binding, bacterial domain containing	AK103734	B9G4K1 (UniProt)	Trigger factor, ribosome-binding, bacterial
Os09g0515500	Translation initiation factor 2 related domain containing protein.	AK065778	D7U9V2 (UniProt)	Translation initiation factor aIF-2, bacterial-like
Os09g0515550	Hypothetical protein.	EU971718	GeneMark	-
Os09g0515800	RabGAP/TBC domain containing protein.	AK070794 ,AK066076	NP_001152412.1 (RefSeq)	Rab-GAP/TBC domain
Os09g0516200	Similar to Transcription factor RF2a.	AK102795	Q69IL4 (UniProt)	Basic-leucine zipper
Os09g0516300	Nucleotide-binding, alpha-beta plait domain containing protein.	AK065222	B8BDJ2 (UniProt)	RNA recognition motif domain
Os09g0516451	Hypothetical gene.	AK287468	longestORF	-

Os09g0516500	NAD(P)-binding domain containing protein.	EU969024	Q69IL0 (UniProt)	3-beta hydroxysteroid dehydrogenase/isomerase
Os09g0516600	Glyoxalase II.	AK070780	Q940L0 (UniProt)	Beta-lactamase-like
Os09g0516700	Protein of unknown function DUF936, plant family protein.	AK072458	B9G4K5 (UniProt)	Protein of unknown function DUF936, plant
Os09g0516750	Hypothetical protein.	tplb0057h05 (Wheat FLcDNA)	longestORF	-
Os09g0563800	ATPase, AAA-type, core domain containing protein.	AK068364	B9G539 (UniProt)	ATPase, AAA-type, core
Os09g0563950	Cyclin-like F-box domain containing protein.	ab initio prediction	NP_001175984.1 (RefSeq)	-
Os09g0564000	Peptidase C1A, papain family protein.	AK071733	B9G542 (UniProt)	Peptidase, cysteine peptidase active site
Os09g0564200	Hypothetical conserved gene.	AK243212	NP_001063943.2 (RefSeq)	Peptidase, cysteine peptidase active site
Os09g0564400	Hypothetical conserved gene.	Z97023	B9G543 (UniProt)	Peptidase, cysteine peptidase active site
Os09g0564450	Non-protein coding transcript.	AK288219	NONE	-
Os09g0564600	Peptidase C1A, papain family protein.	AK289220	NP_001175985.1 (RefSeq)	Peptidase, cysteine peptidase active site
Os09g0564700	Similar to Vignain.	ab initio prediction	B6TYM9 (UniProt)	Peptidase, cysteine peptidase active site
Os09g0564800	Conserved hypothetical protein.	AK069191	NP_001175602.1 (RefSeq)	-
Os09g0565000	Peptidase, cysteine peptidase active site domain containing [illegible]	ab initio prediction	Q650W9 (UniProt)	Peptidase, cysteine peptidase active site
Os09g0565200	Similar to Nucleic acid-binding protein precursor.	AK069121 ,AK104354	A6N0D0 (UniProt)	-
Os09g0565250	Hypothetical gene.	BT019263	longestORF	-
Os09g0565300	Similar to RING-finger protein.	AK243511	B8BEL7 (UniProt)	Copine
Os09g0565350	Conserved hypothetical protein.	AK376692	Q650W5 (UniProt)	-
Os09g0565400	Lipoprotein, type 6 family protein.	AK067821	B9G549 (UniProt)	-
Os09g0565450	Hypothetical conserved gene.	CT835840	B9G551 (UniProt)	-
Os09g0565500	Conserved hypothetical protein.	AK063286	B9G551 (UniProt)	-
Os09g0565600	Similar to Delta(14)-sterol reductase (EC 1.3.1.70) (C-14 sterol reductase) (Sterol C14-reductase) (FACKEL protein).	AK242547	B5QT68 (UniProt)	Ergosterol biosynthesis ERG4/ERG24
Os09g0565700	Prephenate dehydratase domain containing protein.	AK066427	A3C1L2 (UniProt)	Prephenate dehydratase
Os09g0565900	Conserved hypothetical protein.	AK100675	B9G555 (UniProt)	-
Os09g0566050	Similar to Prephenate dehydratase.	AK374296	Q01L56 (UniProt)	Prephenate dehydratase
Os09g0566075	Hypothetical gene.	EU975661	longestORF	-
Os09g0566100	ENTH/VHS domain containing protein.	AK065725	Q650V5 (UniProt)	RNA polymerase II, large subunit, CTD
Os09g0566300	Hypothetical protein.	AK102257	longestORF	-
Os09g0566400	Conserved hypothetical protein.	AK105885 ,AK061768	B8BEM3 (UniProt)	-
Os09g0566550	Similar to serine/threonine protein kinase 1, CTR1.	AK318529	XP_002326245.1 (RefSeq)	Protein kinase, catalytic domain
Os09g0566600	Hypothetical conserved gene.	ab initio prediction	Q650V0 (UniProt)	-
Os09g0567500	Similar to Fatty acyl coA reductase.	AK071956	Q8L4V2 (UniProt)	Male sterility
Os09g0567600	Hypothetical protein.	AK106807	longestORF	-
Os09g0567700	WD40 repeat-like domain containing protein.	AK065913	NP_001105835.1 (RefSeq)	WD40 repeat
Os09g0567800	Hypothetical conserved gene.	CT835822	NP_187604.1 (RefSeq)	-
Os09g0567900	Inosine/uridine-preferring nucleoside hydrolase domain containing protein.	AK061415	B6T563 (UniProt)	Inosine/uridine-preferring nucleoside hydrolase domain
Os09g0568000	Single-stranded nucleic acid binding R3H domain containing [illegible]	AK068829	A2Z474 (UniProt)	Single-stranded nucleic acid binding R3H
Os09g0568050	Hypothetical protein.	tplb0025f09 (Wheat FLcDNA)	longestORF	-
Os09g0568100	Conserved hypothetical protein.	FP092518	B9G564 (UniProt)	-
Os09g0568200	Similar to DNA polymerase epsilon subunit 3.	AK242634	NP_001149275.1 (RefSeq)	Transcription factor CBF/NF-Y/archaeal histone
Os09g0568266	Non-protein coding transcript.	BT018987	NONE	-
Os09g0568400	Similar to 60S ribosomal protein L40 (CEP52).	AK059386	Q7XYD4 (UniProt)	Ribosomal protein L40e
Os09g0568500	Germin family protein.	AK108987	Q84XR7 (UniProt)	Germin
Os09g0568600	Putative germin-like protein 9-2.	Q652Q0 (UniProt)	Q652Q0 (UniProt)	Germin
Os09g0568700	RmlC-like jelly roll fold domain containing protein.	AK109010	Q84XR7 (UniProt)	Germin
Os09g0568800	Similar to Ribosomal protein S25 (40S ribosomal 25S subunit).	AK059234	NP_001148017.1 (RefSeq)	Ribosomal protein S25
Os09g0568900	Similar to predicted protein.	AK059442	XP_002879140.1 (RefSeq)	-
Os09g0569000	Hypothetical gene.	AK108627	longestORF	-
Os09g0569100	Haloacid dehalogenase-like hydrolase domain containing protein.	AK071860	B4FI81 (UniProt)	Haloacid dehalogenase-like hydrolase
Os09g0569151	Hypothetical protein.	AK355652	longestORF	-
Os09g0569200	Similar to Beta-amylase (EC 3.2.1.2) (1,4-alpha-D-glucan maltohydrolase).	AK070300	B6SVZ0 (UniProt)	Glycoside hydrolase, family 14B, plant
Os09g0569300	Similar to calmodulin-binding heat-shock protein.	AK069587	NP_001149987.1 (RefSeq)	Lipase, class 3
Os09g0569400	Beta-lactamase-like domain containing protein.	AK070608 ,AK063384	B6U1S6 (UniProt)	Beta-lactamase-like
Os09g0569450	Conserved hypothetical protein.	AK242543	NP_001175993.1 (RefSeq)	-
Os09g0569700	Conserved hypothetical protein.	AK108778	NP_001063979.1 (RefSeq)	-
Os09g0569780	Conserved hypothetical protein.	AK241041	NP_001175963.1 (RefSeq)	-
Os09g0569800	Serine/threonine protein kinase-related domain containing protein.	ab initio prediction	NP_001063980.1 (RefSeq)	Protein kinase, catalytic domain
Os09g0569900	Hypothetical protein.	AK073081	longestORF	-
Os09g0570000	Serine/threonine protein kinase-related domain containing protein.	AK058797	Q2HTK4 (UniProt)	Protein kinase, catalytic domain
Os09g0570100	Protein kinase, catalytic domain domain containing protein.	AK061135	B9G572 (UniProt)	Protein kinase, catalytic domain
Os09g0570150	Hypothetical conserved gene.	EU953102	B6SK37 (UniProt)	-
Os09g0570200	Zinc finger, C2H2-type domain containing protein.	AK073421	A2Z489 (UniProt)	Zinc finger, C2H2
Os09g0570300	Similar to Short-chain dehydrogenase Tic32.	AK061532	B4FKX6 (UniProt)	Short-chain dehydrogenase/reductase SDR
Os09g0570400	Major facilitator superfamily protein.	AK065287	NP_567175.2 (RefSeq)	Major facilitator superfamily
Os09g0570500	Zinc finger, RING-type domain containing protein.	AK069259	B9G575 (UniProt)	Zinc finger, RING-type
Os09g0570600	PAP/25A core domain containing protein.	AK103632	B9G576 (UniProt)	-
Os09g0570800	Isopenicillin N synthase family protein.	AK064211	NP_001149522.1 (RefSeq)	Isopenicillin N synthase
Os09g0570850	Similar to Histone H2B.	ab initio prediction	B6SGC3 (UniProt)	Histone H2B
Os09g0570900	Amino acid-binding ACT domain containing protein.	AK071292	B6TMA2 (UniProt)	Amino acid-binding ACT
Os09g0570951	Hypothetical gene.	tplb0017n19 (Wheat FLcDNA)	longestORF	-
Os09g0571000	Protein of unknown function DUF966 family protein.	ab initio prediction	Q651B3 (UniProt)	Protein of unknown function DUF966
Os09g0571033	Conserved hypothetical protein.	AK241104	NP_001175997.1 (RefSeq)	-
Os09g0571100	Pectin lyase fold/virulence factor domain containing protein.	AK106869	C5X6M1 (UniProt)	Pectinesterase, catalytic
Os09g0571200	C2 domain containing protein.	AK062604	B6TT83 (UniProt)	C2 calcium-dependent membrane targeting
Os09g0571400	Cyclophilin 1.	AK103109	Q40672 (UniProt)	Peptidyl-prolyl cis-trans isomerase, cyclophilin-type
Os09g0571500	Lipase, class 3 family protein.	AK071951	NP_181773.2 (RefSeq)	Lipase, class 3
Os10g0162846	Similar to LRR14.	AK363200	Q9ATQ3 (UniProt)	-
Os10g0162848	Non-protein coding transcript.	AK058701	NONE	-
Os10g0162852	Non-protein coding transcript.	AK058701	NONE	-
Os10g0355800	Similar to ATP synthase CF1 beta subunit.	ab initio prediction	NP_039390.1 (RefSeq)	ATPase, F1/V1/A1 complex, alpha/beta subunit, nucleotide-binding domain
Os10g0356000	Similar to ribulose-1,5-bisphosphate carboxylase/oxygenase large subunit.	ab initio prediction	NP_039391.1 (RefSeq)	Ribulose bisphosphate carboxylase, large subunit, C-terminal
Os10g0410600	Similar to Phosphoprotein phosphatase 2A isoform 4.	AK099604	A3C4N5 (UniProt)	Metallophosphoesterase domain
Os10g0410650	Hypothetical gene.	BT018461	longestORF	-
Os10g0410700	Similar to SET domain protein 123.	AK111981 ,AK111906	NP_001105199.1 (RefSeq)	SET domain
Os10g0410750	Protein kinase, catalytic domain domain containing protein.	EU975108	XP_002450423.1 (RefSeq)	Protein kinase, catalytic domain
Os10g0410900	Conserved hypothetical protein.	AK119279	Q7XER2 (UniProt)	-
Os10g0411100	Protein of unknown function DUF620 domain containing protein.	BT088024	XP_002468447.1 (RefSeq)	Protein of unknown function DUF620
Os10g0411200	Hypothetical protein.	BT085554	longestORF	-
Os10g0411500	IQ calmodulin-binding region domain containing protein.	AK072572	B8BGS6 (UniProt)	-
Os10g0411600	Hypothetical protein.	AK108669	longestORF	-
Os10g0411650	Non-protein coding transcript.	EU947431	NONE	-
Os10g0411700	Similar to S28 ribosomal protein (Fragment).	AK058209 ,AK121099	Q7X9K4 (UniProt)	-
Os10g0411750	Hypothetical conserved gene.	AK241834	Q7XEQ4 (UniProt)	JNK/Rab-associated protein-1, N-terminal
Os10g0411800	Similar to 40S ribosomal protein S17-3.	AK059434	NP_001149330.1 (RefSeq)	Ribosomal protein S17e
Os10g0412000	ATPase, P-type, K/Mg/Cd/Cu/Zn/Na/Ca/Na/H-transporter family protein.	AK066864	XP_002314626.1 (RefSeq)	ATPase, P-type, K/Mg/Cd/Cu/Zn/Na/Ca/Na/H-transporter
Os10g0412050	Similar to aminophospholipid ATPase.	BT035338	NP_176191.1 (RefSeq)	ATPase, P-type, ATPase-associated domain
Os10g0412100	Endonuclease/exonuclease/phosphatase domain containing [illegible]	AK102338	Q10R98 (UniProt)	Endonuclease/exonuclease/phosphatase
Os10g0412350	Similar to H0315A08.1 protein.	FP099421	Q01I63 (UniProt)	Ribonuclease H1, N-terminal
Os10g0428900	Similar to BTB/POZ domain containing protein.	ab initio prediction	Q7XEB6 (UniProt)	MATH
Os10g0429200	BTB/POZ fold domain containing protein.	AK248323	Q7XEA8 (UniProt)	BTB/POZ fold
Os10g0429300	Kelch related domain containing protein.	tplb0003h03 (Wheat FLcDNA)	Q7XEA7 (UniProt)	BTB/POZ-like
Os10g0429600	Similar to BTB/POZ domain containing protein.	AK369602	Q7XEA4 (UniProt)	BTB/POZ-like
Os10g0429651	Hypothetical conserved gene.	AK248323	Q7XEA4 (UniProt)	BTB/POZ-like
Os10g0430200	Similar to Sinapyl alcohol dehydrogenase.	AK069040	Q8S411 (UniProt)	Alcohol dehydrogenase superfamily, zinc-type
Os10g0430600	Aminotransferase, class-II, pyridoxal-phosphate binding site domain containing protein.	AK069549	B8BGZ5 (UniProt)	-
Os10g0430700	Conserved hypothetical protein.	AK072713	B8BGZ5 (UniProt)	-
Os10g0430750	Hypothetical conserved gene.	AK062794	longestORF	-
Os10g0430800	Hypothetical conserved gene.	AK072819	A3C4Z6 (UniProt)	-
Os10g0430900	Tyrosine protein kinase domain containing protein.	AK121718	Q5XPL8 (UniProt)	Protein kinase, catalytic domain
Os10g0431000	Similar to predicted protein.	AK099410	NP_199554.2 (RefSeq)	DWNN domain
Os10g0431100	Hypothetical protein.	ab initio prediction	NONE	-
Os10g0475400	Nucleoside phosphatase GDA1/CD39 domain containing protein.	EU946507	Q8W5Q4 (UniProt)	Nucleoside phosphatase GDA1/CD39
Os10g0475900	Ubiquitin domain containing protein.	AK101853	Q8W5Q6 (UniProt)	Ubiquitin
Os10g0476000	Adaptin ear-binding coat-associated protein 1 NECAP-1 family protein.	AK065629	B4FAZ6 (UniProt)	Adaptin ear-binding coat-associated protein 1 NECAP-1
Os10g0476100	Similar to Protein kinase MK6.	AK375579	Q9LW99 (UniProt)	Protein kinase, catalytic domain
Os10g0476200	Similar to Casein kinase I (Fragment).	AK107522	longestORF	-
Os10g0476300	Similar to casein kinase I isoform delta-like.	AK070314	NP_001151631.1 (RefSeq)	Protein kinase-like domain

Os10g0476400	Snf7 family protein.	AK072826	B6T5E0 (UniProt)	Snf7
Os10g0476500	Conserved hypothetical protein.	AK110990	B9G687 (UniProt)	-
Os10g0476600	Similar to Protein phosphatase 2A 62 kDa B'' regulatory subunit (Protein phosphatase 2A 62 kDa B regulatory subunit) (Protein phosphatase 2A alpha).	AK067811	XP_002872330.1 (RefSeq)	EF-hand-like domain
Os10g0476700	Esterase, SGNH hydrolase-type, subgroup domain containing	AK070445	B6TVA2 (UniProt)	Lipase, GDSL
Os10g0476900	Pentatricopeptide repeat domain containing protein.	AK103437	A3C5Q4 (UniProt)	Pentatricopeptide repeat
Os10g0477000	Armadillo-like helical domain containing protein.	AK106419	Q9AV01 (UniProt)	Armadillo-type fold
Os10g0477100	Similar to Ankyrin-like protein.	AK067258	NP_567427.1 (RefSeq)	Protein of unknown function DUF248, methyltransferase putative
Os10g0477200	Pentatricopeptide repeat domain containing protein.	AK067154	A3C5Q7 (UniProt)	Pentatricopeptide repeat
Os10g0477301	Conserved hypothetical protein.	AK288518	Q69WY5 (UniProt)	-
Os10g0507500	Conserved hypothetical protein.	FP097010	B9G6J5 (UniProt)	-
Os10g0507600	PapD-like domain containing protein.	AK058656	Q8LN94 (UniProt)	Major sperm protein
Os10g0507700	Conserved hypothetical protein.	EU968230	Q8LN95 (UniProt)	-
Os10g0507800	Similar to chaperone protein dnaJ 13.	AK061575	B6T7S1 (UniProt)	Heat shock protein DnaJ, N-terminal
Os10g0508000	Similar to C-terminal peptide-binding protein 1.	CT837938	Q1EPX7 (UniProt)	Multicopper oxidase, type 1
Os10g0508050	Hypothetical protein.	tplb0021n10 (Wheat FLcDNA)	longestORF	-
Os10g0508100	Protein of unknown function DUF641, plant domain containing protein.	AK287902	NP_001065020.1 (RefSeq)	Domain of unknown function DUF641, plant
Os10g0508300	Similar to Protein YABBY 3.	AB274015	Q8L556 (UniProt)	High mobility group, HMG1/HMG2
Os10g0508400	Similar to Methionine aminopeptidase-like protein.	AK066207	B9G6J9 (UniProt)	Peptidase M24, structural domain
Os10g0508500	Similar to tubulin folding cofactor.	BT041825	XP_002320715.1 (RefSeq)	Armadillo-like helical
Os10g0508600	Protein of unknown function DUF1352 domain containing	AK103246	Q8LN74 (UniProt)	Protein of unknown function DUF1352
Os10g0543400	Chitinase 8.	Q7XCK6 (UniProt)	Q7XCK6 (UniProt)	Glycoside hydrolase, family 19, catalytic
Os10g0543500	Six-bladed beta-propeller, TolB-like domain containing protein.	AK106264	Q109A9 (UniProt)	Six-bladed beta-propeller, TolB-like
Os10g0543800	Similar to Glutathione S-transferase GST 11 (EC 2.5.1.18).	CT836577	NP_001104983.1 (RefSeq)	Glutathione S-transferase, N-terminal
Os10g0544200	Basic helix-loop-helix dimerisation region bHLH domain containing protein.	AK063669	NP_001149796.1 (RefSeq)	Helix-loop-helix DNA-binding
Os10g0544500	WD40 repeat-like domain containing protein.	AK073941	NP_001150616.1 (RefSeq)	WD40 repeat
Os10g0544600	Zinc finger, RING/FYVE/PHD-type domain containing protein.	AK061763 ,AK099554 ,AK10588	NP_001151464.1 (RefSeq)	Zinc finger, RING-type
Os10g0544900	Similar to Protein phosphatase 2C-like protein.	AK119635	B6TZR2 (UniProt)	Protein phosphatase 2C, manganese/magnesium aspartate binding site
Os10g0544933	Non-protein coding transcript.	EU943254	NONE	-
Os10g0544950	Hypothetical gene.	AK242432	longestORF	-
Os10g0544966	Hypothetical protein.	tplb0043b08 (Wheat FLcDNA)	longestORF	-
Os10g0545000	Similar to magnesium transporter CorA-like family protein.	AK069472	NP_567076.1 (RefSeq)	Fibronectin-attachment
Os10g0545100	Similar to predicted protein.	AK287840	B9G1Z7 (UniProt)	Uncharacterised conserved protein UCP014543
Os10g0545200	Similar to 4,4-dimethyl-sterol C4-methyl-oxidase (Fragment).	AK121804	B4FN64 (UniProt)	Fatty acid hydroxylase
Os10g0545300	Zinc finger, CCHC retroviral-type domain containing protein.	AK065156 ,AK121559	NP_001149324.1 (RefSeq)	Zinc finger, CCHC-type
Os10g0545500	Similar to xyloglucan endotransglucosylase/hydrolase protein 32.	FJ264508	NP_001148432.1 (RefSeq)	Glycoside hydrolase, family 16
Os10g0545600	Similar to transposon protein.	EU976069	B6UFQ4 (UniProt)	Zinc finger, RING-type
Os10g0545700	Similar to Rhodanese-like protein.	AK073024	Q66T88 (UniProt)	Rhodanese-like
Os10g0545800	Nucleic acid-binding, OB-fold domain containing protein.	AK103222	NP_001152237.1 (RefSeq)	CcmE/CycJ protein
Os10g0546100	Pollen Ole e 1 allergen and extensin domain containing protein.	AK240974	NP_001065213.1 (RefSeq)	Pollen Ole e 1 allergen/extensin
Os10g0546200	Glycosyl transferase, family 1 domain containing protein.	AK372742	Q9AV30 (UniProt)	Glycosyl transferase, family 1
Os10g0546300	Protein of unknown function DUF2451, C-terminal domain containing protein.	AK103033	B9G6U9 (UniProt)	Protein of unknown function DUF2451, C-terminal
Os10g0580750	Hypothetical gene.	BT019171	longestORF	-
Os10g0580800	Similar to NClpP4 (Fragment).	AK104823 ,AK065306 ,AK10439	B4FP39 (UniProt)	Peptidase S14, ClpP
Os10g0580900	Conserved hypothetical protein.	AK072734	Q9FRM3 (UniProt)	-
Os10g0581000	Conserved hypothetical protein.	AK242551	B9G779 (UniProt)	-
Os11g0312400	Adenylate kinase B (EC 2.7.4.3) (ATP-AMP	AK073605	Q08480 (UniProt)	Adenylate kinase
Os11g0323860	Hypothetical conserved gene.	AK243521	B9GCL3 (UniProt)	-
Os11g0334938	Similar to Class III peroxidase 111.	BT085722	B6TU39 (UniProt)	Plant peroxidase
Os11g0340477	Zinc finger, C2H2-type domain containing protein.	AK108997	A2XIC5 (UniProt)	Zinc finger, C2H2
Os11g0346016	Similar to calmodulin binding protein.	AK372659	NP_001151502.1 (RefSeq)	Calmodulin binding protein-like
Os11g0351555	Similar to A.thaliana gene induced upon wounding stress.	AK106105	D6NIX1 (UniProt)	NAD
Os11g0357094	Pentatricopeptide repeat domain containing protein.	ab initio prediction	A2XIB7 (UniProt)	Pentatricopeptide repeat
Os11g0362633	Hypothetical conserved gene.	FP093303	A9NKD9 (UniProt)	Protein of unknown function DUF3743
Os11g0368172	Peptidase S8 and S53, subtilisin, kexin, sedolisin domain containing protein.	AK107787	A9NKD9 (UniProt)	Protein of unknown function DUF3743
Os11g0373711	Hypothetical gene.	AK101901	longestORF	-
Os11g0379251	Similar to calmodulin binding protein.	AK372659	NP_001151502.1 (RefSeq)	Calmodulin binding protein-like
Os11g0709000	Similar to H0124E07.4 protein.	AK241872	NP_001175717.1 (RefSeq)	-
Os12g0109150	Similar to Sodium/hydrogen exchanger family protein.	ab initio prediction	Q2QYR3 (UniProt)	-
Os12g0109200	Similar to Ca(2+)-dependent nuclease.	AK103380	B8BNU8 (UniProt)	-
Os12g0109300	Pentatricopeptide repeat domain containing protein.	AK103426	Q2QYR0 (UniProt)	Pentatricopeptide repeat
Os12g0109375	Hypothetical protein.	tplb0021n11 (Wheat FLcDNA)	longestORF	-
Os12g0109600	Protein of unknown function DUF1677, Oryza sativa family	AK107606	B9GBG9 (UniProt)	Protein of unknown function DUF1677, Oryza sativa
Os12g0109633	Hypothetical conserved gene.	EU955714	A3CE98 (UniProt)	Protein of unknown function DUF2985
Os12g0109700	Protein of unknown function DUF2985 domain containing	AK058609	Q2QYQ5 (UniProt)	Protein of unknown function DUF2985
Os12g0109750	Hypothetical gene.	AK361165	longestORF	-
Os12g0109800	Hypothetical conserved gene.	BT066437	B8BNV5 (UniProt)	Pentatricopeptide repeat
Os12g0109850	Similar to Protein kinase domain containing protein, expressed.	ab initio prediction	Q2RBK1 (UniProt)	Serine-threonine/tyrosine-protein kinase
Os12g0109900	Double-stranded RNA-binding-like domain containing protein.	AK103543	Q0IV63 (UniProt)	Double-stranded RNA-binding
Os12g0110000	Regulatory associated protein of TOR family protein.	AY491431	Q0IQN5 (UniProt)	WD40 repeat
Os12g0110050	Hypothetical conserved gene.	EU953906	Q0IQN5 (UniProt)	-
Os12g0110100	Esterase/lipase/thioesterase domain containing protein.	AK067362	NP_850428.1 (RefSeq)	-
Os12g0110150	Hypothetical gene.	EU949682	longestORF	-
Os12g0110200	Hypothetical protein.	AK105537	longestORF	-
Os12g0110300	Similar to cDNA, clone: J065191E12, full insert sequence.	AK058945	B7F8Y2 (UniProt)	-
Os12g0110400	Similar to PTAC6 (PLASTID TRANSCRIPTIONALLY	AK102323	NP_564144.1 (RefSeq)	-
Os12g0110475	Conserved hypothetical protein.	AK240800	NP_001176757.1 (RefSeq)	-
Os12g0110700	Conserved hypothetical protein.	BT067382	NP_001046339.1 (RefSeq)	-
Os12g0110951	Hypothetical gene.	AK288366	longestORF	-
Os12g0111000	Conserved hypothetical protein.	AK064603	Q2QYP4 (UniProt)	-
Os12g0199000	Similar to Jasmonate-induced protein.	FP099363	Q564C9 (UniProt)	Plant disease resistance response protein
Os12g0199050	Hypothetical protein.	BT016217	longestORF	-
Os12g0199100	NB-ARC domain containing protein.	AK065572	B9GCB3 (UniProt)	Disease resistance protein
Os12g0199200	Non-protein coding transcript.	AK066878	NONE	-
Os12g0199500	Similar to N-methyltransferase.	U43498	Q2QWD0 (UniProt)	O-methyltransferase, family 2
Os12g0199800	Similar to Cyt-P450 monooxygenase.	AK288730	Q8S3F6 (UniProt)	Cytochrome P450
Os12g0469300	Non-protein coding transcript.	tplb0015j08 (Wheat FLcDNA)	NONE	-
Os12g0470000	Similar to Rab28 protein.	tplb0013m03 (Wheat FLcDNA)	Q41850 (UniProt)	Seed maturation protein
Os12g0522516	Similar to ATRL5 (ARABIDOPSIS RAD-LIKE 5)%3B DNA binding / transcription factor.	tplb0003e07 (Wheat FLcDNA)	NP_564087.2 (RefSeq)	SANT domain, DNA binding
Os12g0524201	Hypothetical conserved gene.	AK288953	B6U883 (UniProt)	-
Os12g0524750	Similar to ribosomal protein S7.	ab initio prediction	YP_514635.1 (RefSeq)	Ribosomal protein S7
Os12g0525300	Hypothetical protein.	ab initio prediction	NONE	-
Os12g0641200	Hypothetical protein.	AK064961	longestORF	-
Os12g0641300	Similar to Zn-dependent hydrolases of the beta-lactamase fold.	AK061015	C3TX72 (UniProt)	Beta-lactamase-like
Os12g0641400	Similar to Sucrose transporter.	AK067030	Q0ILJ3 (UniProt)	Major facilitator superfamily domain, general substrate transporter
Os12g0641500	Similar to SMC1 protein.	AK120333	Q8GU56 (UniProt)	RecF/RecN/SMC
Os12g0641600	Non-protein coding transcript.	AK289049	NONE	-
**HHZ pedigree**				
Os01g0180000	Pistil-specific extensin-like protein family protein.	AK103199	NP_001150408.1 (RefSeq)	Leucine-rich repeat
Os01g0180050	Hypothetical protein.	BT068200	longestORF	-
Os01g0180300	Hypothetical conserved gene.	AK061954	B9ETH2 (UniProt)	-
Os01g0180400	Protein of unknown function DUF581 domain containing protein.	AK108226	B9ETH3 (UniProt)	Protein of unknown function DUF581
Os01g0180500	Non-protein coding transcript.	EU973947	NONE	-
Os01g0180600	Similar to MutS homolog 7 (Fragment).	AK122068	Q8RVT1 (UniProt)	DNA mismatch repair protein MutS, C-terminal
Os01g0180700	Hypothetical conserved gene.	AK240913	Q9AQU6 (UniProt)	-
Os01g0180800	Similar to heat shock 70 kDa protein 4.	AK061691	NP_001147805.1 (RefSeq)	Heat shock protein Hsp70
Os01g0180850	Hypothetical protein.	tplb0021a19 (Wheat FLcDNA)	longestORF	-
Os01g0180900	Conserved hypothetical protein.	AK357358	NP_001172205.1 (RefSeq)	-
Os01g0181000	Frigida-like domain containing protein.	EU948578	Q5VR39 (UniProt)	Frigida-like
Os01g0181033	Similar to NADH dehydrogenase subunit 1.	AJ010976	YP_024343.1 (RefSeq)	NADH:ubiquinone oxidoreductase, subunit 1/F420H2 oxidoreductase subunit
Os01g0181166	Hypothetical gene.	CU405586	longestORF	-
Os01g0230700	Hypothetical conserved gene.	AK289150	Q9LE91 (UniProt)	-
Os01g0230800	Conserved hypothetical protein.	AK109258	Q657L2 (UniProt)	-
Os01g0231000	Similar to Auxin-responsive protein (Aux/IAA) (Fragment).	AK104654	Q5NB25 (UniProt)	AUX/IAA protein
Os01g0231500	Similar to casein kinase I isoform delta-like.	AK111602	B9EUI3 (UniProt)	Protein kinase, catalytic domain
Os01g0231600	Pleckstrin homology-type domain containing protein.	ab initio prediction	A2WMF2 (UniProt)	Pleckstrin homology-type
Os01g0231700	Similar to 60S acidic ribosomal protein P2-B (CaRP2B).	AK107453	NP_001147388.1 (RefSeq)	Ribosomal protein 60S
Os01g0231800	Similar to cDNA clone:J033025F17, full insert sequence.	AK062918	B7ELY0 (UniProt)	Protein kinase A anchor protein, nuclear localisation signal domain

Os01g0231900	K Homology, type 1, subgroup domain containing protein.	AK073280	B9EUI5 (UniProt)	K Homology
Os01g0231950	Hypothetical gene.	BT061568	longestORF	-
Os01g0232000	Major intrinsic protein family protein.	Q9LWR2 (UniProt)	Q9LWR2 (UniProt)	Major intrinsic protein
Os01g0232100	Similar to Tonoplast membrane integral protein ZmTIP4-3.	AK069592 ,AK099190	NP_001105035.1 (RefSeq)	Major intrinsic protein
Os01g0232200	WD40 repeat domain containing protein.	EU955858	Q5NB66 (UniProt)	WD40 repeat
Os01g0232300	Similar to TRZ3 (TRNASE Z 3)%3B 3'-tRNA processing endoribonuclease/ catalytic.	AK120694	NP_175628.2 (RefSeq)	-
Os01g0232400	Similar to VHS1 protein (Fragment).	AK106519	B8AB14 (UniProt)	Epsin domain, N-terminal
Os01g0232450	Conserved hypothetical protein.	AK242981	NP_001172244.1 (RefSeq)	-
Os01g0232500	Similar to PFU3/UBC26 (UBIQUITIN-CONJUGATING ENZYME 26)%3B ubiquitin-protein ligase.	AK119659	A2ZR04 (UniProt)	Ubiquitin-conjugating enzyme, E2
Os01g0232550	Hypothetical conserved gene.	EU941213	B9EUI8 (UniProt)	-
Os01g0232700	Similar to Histidinol dehydrogenase.	AK104113 ,AK061778	A2WMG4 (UniProt)	Histidinol dehydrogenase, conserved site
Os01g0232800	Similar to KOB1.	AK318572	NP_001151449.1 (RefSeq)	-
Os01g0233000	DREPP plasma membrane polypeptide family protein.	AK065256	A2WMG6 (UniProt)	DREPP plasma membrane polypeptide
Os01g0233800	Similar to Viroid symptom modulation protein.	AK069983	Q41297 (UniProt)	Protein kinase, catalytic domain
Os01g0233850	Hypothetical gene.	EU943630	longestORF	-
Os01g0233900	Similar to cDNA clone:J013003E06, full insert sequence.	AK100714	B7EAV1 (UniProt)	Ubiquitin-conjugating enzyme, E2
Os01g0234001	Hypothetical conserved gene.	AK241763	B9G6H6 (UniProt)	-
Os01g0234100	Transcriptional factor B3 family protein.	AK106332	Q0JP99 (UniProt)	Transcriptional factor B3
Os01g0234200	Similar to DNA topoisomerase 2.	ab initio prediction	A2WMH7 (UniProt)	DNA topoisomerase, type IIA, subunit B/N-terminal
Os01g0234300	Similar to Pectinesterase.	ab initio prediction	B9EUJ6 (UniProt)	Pectinesterase, catalytic
Os01g0234433	Hypothetical protein.	EU943103	longestORF	-
Os01g0234499	Similar to Pectinesterase.	ab initio prediction	B9EUJ6 (UniProt)	Pectinesterase, catalytic
Os01g0234566	Non-protein coding transcript.	X06283	NONE	-
Os01g0234700	Harpin-induced 1 domain containing protein.	AK108936	NP_001147348.1 (RefSeq)	-
Os01g0234800	Non-protein coding transcript.	AK063827	NONE	-
Os01g0234850	Hypothetical conserved gene.	AK242318	NP_001172247.1 (RefSeq)	Phosphatidylinositol 3-/4-kinase, catalytic
Os01g0234900	Similar to Ubiquitin ligase SINAT5 (EC 6.3.2.-) (Seven in absentia homolog 5). Splice isoform 2.	AK120816	B8Q8B9 (UniProt)	Zinc finger, RING-type
Os01g0235100	Similar to predicted protein.	AK287587	Q5NAX5 (UniProt)	-
Os01g0235200	Conserved hypothetical protein.	AK064013	B9EUK0 (UniProt)	-
Os01g0235300	SOUL haem-binding protein domain containing protein.	AK102961 ,AK104591	B6SR01 (UniProt)	SOUL haem-binding protein
Os01g0235325	Hypothetical protein.	EU943029	longestORF	-
Os01g0235350	Conserved hypothetical protein.	ab initio prediction	B9EUK1 (UniProt)	-
Os01g0235400	Similar to Importin-alpha re-exporter (Cellular apoptosis susceptibility protein homolog).	AK105595	Q9ZPY7 (UniProt)	CAS/CSE, C-terminal
Os01g0235500	Conserved hypothetical protein.	AK121404	A2WMJ1 (UniProt)	-
Os01g0235632	Hypothetical protein.	tplb0028d05 (Wheat FLcDNA)	longestORF	-
Os01g0235700	Similar to BHLH transcription factor (Fragment).	AK121013	Q5IWM1 (UniProt)	Helix-loop-helix DNA-binding
Os01g0235800	K Homology domain containing protein.	AK058731	B9EUK2 (UniProt)	K Homology
Os01g0235850	Hypothetical protein.	EU974948	longestORF	-
Os01g0235900	Thioredoxin fold domain containing protein.	ab initio prediction	NP_001042526.1 (RefSeq)	Glutaredoxin
Os01g0236000	Conserved hypothetical protein.	AK065569	B8AB33 (UniProt)	-
Os01g0252600	Similar to predicted protein.	CT835001	B8ABT0 (UniProt)	-
Os01g0252900	Zinc finger, CCHC-type domain containing protein.	AK105250	XP_002878450.1 (RefSeq)	Zinc finger, CCHC-type
Os01g0253000	Similar to LpimPth3.	AK071644	Q5NBC2 (UniProt)	Protein kinase, catalytic domain
Os01g0253050	Conserved hypothetical protein.	EU957207	NP_001042608.1 (RefSeq)	-
Os01g0253100	Hypothetical conserved gene.	AK061932	B9EV01 (UniProt)	Protein kinase, catalytic domain
Os01g0253200	Similar to pectinesterase inhibitor domain containing protein.	tplb0011f19 (Wheat FLcDNA)	NP_001148594.1 (RefSeq)	Pectinesterase inhibitor
Os01g0253300	Importin alpha-1a subunit.	AK099336	B6T451 (UniProt)	Armadillo
Os01g0253400	Protein of unknown function DUF1218 family protein.	AK108904	Q9S809 (UniProt)	Protein of unknown function DUF1218
Os01g0253500	Conserved hypothetical protein.	AK243648	NP_001172261.1 (RefSeq)	-
Os01g0253600	Replication factor A protein 3 domain containing protein.	AK058837	NP_001152271.1 (RefSeq)	Nucleic acid-binding, OB-fold
Os01g0253800	Conserved hypothetical protein.	ab initio prediction	NP_001172262.1 (RefSeq)	-
Os01g0253900	Lipase, class 3 family protein.	AK067101	NP_001148192.1 (RefSeq)	Lipase, class 3
Os01g0254000	Similar to NTGB2 (Fragment).	AK111904 ,AK111979 ,AK11954	B4FPT6 (UniProt)	Small GTP-binding protein domain
Os01g0254100	Similar to Lissencephaly type-1-like homology motif%3B CTLH, C-terminal to LisH motif%3B Nitrous oxide reductase, N-terminal%3B WD40-like%3B Quinonprotein alcohol [illegible]	AK111583	Q2HW32 (UniProt)	WD40 repeat
Os01g0254200	Conserved hypothetical protein.	AK121256	A2WMZ3 (UniProt)	-
Os01g0254300	Pectinesterase (EC 3.1.1.11) (Fragment).	EU974973	B4F9U3 (UniProt)	Pectinesterase, catalytic
Os01g0254350	Hypothetical gene.	BT085732	longestORF	-
Os01g0654100	Similar to CTP synthase.	AK062263	NP_001148101.1 (RefSeq)	CTP synthase
Os01g0654150	Non-protein coding transcript.	tplb0056h06 (Wheat FLcDNA)	NONE	-
Os01g0654200	Peptidase M50 domain containing protein.	AK100750	B9EYD8 (UniProt)	Peptidase M50
Os01g0654300	Similar to ARP2/3 complex 34 kDa subunit.	AK058593	B6TA29 (UniProt)	Arp2/3 complex, 34kDa subunit p34-Arc
Os01g0654400	Similar to Salt tolerant correlative protein.	AK288103	E6Y2L2 (UniProt)	-
Os01g0654450	Hypothetical gene.	EU946002	longestORF	-
Os01g0654500	Similar to NADP-isocitrate dehydrogenase.	AK061752	Q9XGU8 (UniProt)	Isocitrate dehydrogenase NADP-dependent, eukaryotic-type
Os01g0654650	Non-protein coding transcript.	BT016882	NONE	-
Os01g0654800	Conserved hypothetical protein.	AK359861	B9EYE1 (UniProt)	-
Os01g0654900	Hypothetical conserved gene.	ab initio prediction	NP_001172487.1 (RefSeq)	-
Os01g0655250	PWWP domain containing protein.	ab initio prediction	B9EYE3 (UniProt)	PWWP
Os01g0655300	Similar to Trithorax 4 (Fragment).	AK062870	B3VSN3 (UniProt)	SET domain
Os01g0655400	Hypothetical conserved gene.	AK060601	Q5SN80 (UniProt)	-
Os01g0655500	Similar to protein kinase domain containing protein.	AK108632	NP_001147834.1 (RefSeq)	-
Os01g0655800	Similar to ACS-like protein.	AK067776	Q2I824 (UniProt)	AMP-dependent synthetase/ligase
Os01g0658400	Ubiquitin-conjugating enzyme OsUBC5a.	AK063826 ,AK099284	B8A6V8 (UniProt)	Ubiquitin-conjugating enzyme, E2
Os01g0658450	Non-protein coding transcript.	BT086907	NONE	-
Os01g0658500	Similar to Vacuolar protein sorting protein 25.	AK069050	B6SHU1 (UniProt)	ESCRT-II complex, vps25 subunit
Os01g0658600	Similar to SBH2 (SPHINGOID BASE HYDROXYLASE 2)%3B catalytic/ sphingosine hydroxylase.	BT009316	NP_563944.1 (RefSeq)	-
Os01g0658700	Glycoside hydrolase-type carbohydrate-binding, subgroup domain containing protein.	AK106555	NP_001149116.1 (RefSeq)	Aldose 1-epimerase
Os01g0658800	Hypothetical protein.	tplb0033p24 (Wheat FLcDNA)	longestORF	-
Os01g0658900	OSBZ8.	AK121925	Q40645 (UniProt)	Basic-leucine zipper
Os01g0659200	Similar to Vacuolar ATP synthase subunit E (EC 3.6.3.14) (V-ATPase E subunit) (Vacuolar proton pump E subunit).	AK071200	Q2L9B8 (UniProt)	ATPase, V1/A1 complex, subunit E
Os01g0659400	Non-protein coding transcript.	AK064418	NONE	-
Os01g0659800	C2 calcium-dependent membrane targeting domain containing [illegible]	AK109718	NP_001152648.1 (RefSeq)	C2 calcium-dependent membrane targeting
Os01g0659900	Cyclin-like F-box domain containing protein.	AK121359	E5GCH4 (UniProt)	F-box domain, cyclin-like
Os01g0660000	Conserved hypothetical protein.	AK064265	Q6Z593 (UniProt)	-
Os01g0660100	Conserved hypothetical protein.	AK108487	B9FS42 (UniProt)	-
Os01g0660200	Acidic class III chitinase OsChib3a precursor (Chitinase) (EC 3.2.1.14).	AK100973	GeneMark	Glycoside hydrolase, family 18, catalytic domain
Os01g0660300	Similar to Pyruvate kinase.	AK106457	A5C814 (UniProt)	Pyruvate kinase
Os01g0660700	Protein of unknown function DUF295 family protein.	AK064155	A2WTF8 (UniProt)	Protein of unknown function DUF295
Os01g0660800	3'-5' exonuclease domain containing protein.	AK242290	B6TKY2 (UniProt)	3'-5' exonuclease
Os01g0660850	Non-protein coding transcript.	AK289062	NONE	-
Os01g0660900	Similar to phosphoglycerate mutase family protein.	AK330283	B6TTB7 (UniProt)	Histidine phosphatase superfamily, clade-1
Os01g0661000	Uncharacterised protein family UPF0497, trans-membrane plant domain containing protein.	AK101958	B8A6W8 (UniProt)	Uncharacterised protein family UPF0497, trans-membrane plant
Os01g0661200	Hypothetical protein.	ab initio prediction	NONE	-
Os01g0661400	S1, RNA binding domain containing protein.	AK073113	B6TDY7 (UniProt)	RNA-binding domain, S1
Os01g0661500	Mov34/MPN/PAD-1 family protein.	AK068127	Q6NKP9 (UniProt)	Mov34/MPN/PAD-1
Os01g0661601	Hypothetical gene.	AK241460	longestORF	-
Os01g0661650	Hypothetical conserved gene.	CT834939	NP_001172489.1 (RefSeq)	-
Os01g0661700	Hypothetical gene.	AK072040	longestORF	-
Os01g0661750	Hypothetical conserved gene.	ab initio prediction	NP_001172490.1 (RefSeq)	-
Os01g0666800	Similar to predicted protein.	AK242777	NP_565879.1 (RefSeq)	Vps16, C-terminal
Os01g0666900	Non-protein coding transcript.	CT834937	NONE"	-
Os01g0666950	Hypothetical conserved gene.	BT016634	C4J224 (UniProt)	-
Os01g0667000	No apical meristem (NAM) protein domain containing protein.	FP096727	Q5QLQ4 (UniProt)	No apical meristem
Os01g0667100	Similar to 60S ribosomal protein L18A.	AK287948	NP_001148379.1 (RefSeq)	Ribosomal protein L18a
Os01g0667200	Similar to Glyoxalase II.	AK104104 ,AK104749	B9EYI3 (UniProt)	Beta-lactamase-like
Os01g0667400	Similar to WUSCHEL-related homeobox 7.	AK064236	Q0JKK6 (UniProt)	-
Os01g0667450	Similar to WUSCHEL-related homeobox 7.	AM234752	Q0JKK6 (UniProt)	Homeobox
Os01g0667550	Hypothetical gene.	BT084300	longestORF	-
Os01g0667600	Similar to GTP-binding protein.	AK111561	B6U632 (UniProt)	Small GTPase superfamily
Os01g0667700	Zinc finger, RING/FYVE/PHD-type domain containing protein.	AK069011	B6SVN2 (UniProt)	Zinc finger, RING-type
Os01g0667725	Non-protein coding transcript.	tplb0029e12 (Wheat FLcDNA)	NONE	-
Os01g0667750	Hypothetical gene.	AK288717	longestORF	-
Os01g0667800	Similar to U2 snRNP auxiliary factor, small subunit.	AK241513	NP_001148499.1 (RefSeq)	RNA recognition motif domain

Os01g0667900	Thioredoxin fold domain containing protein.	AK105335	Q5QLR2 (UniProt)	Glutaredoxin
Os01g0668000	CS domain containing protein.	AK101664	B6SNS1 (UniProt)	CS-like domain
Os01g0668100	Similar to Arabinogalactan-like protein.	AK105603	Q06I92 (UniProt)	FAS1 domain
Os01g0668200	Conserved hypothetical protein.	EU964274	B8A778 (UniProt)	-
Os01g0668300	Conserved hypothetical protein.	AK102266	B9EYI8 (UniProt)	-
Os01g0668400	Similar to predicted protein.	AK365081	B9EYI9 (UniProt)	Protein kinase, catalytic domain
Os01g0668500	Hypothetical protein.	AK108767	longestORF	-
Os01g0668600	Curculin-like (mannose-binding) lectin domain containing protein.	AK365081	NP_001043817.2 (RefSeq)	Protein kinase, catalytic domain
Os01g0668700	Hypothetical protein.	AK067957	longestORF	-
Os01g0668901	Protein kinase, catalytic domain domain containing protein.	AK365081	Q5QLR7 (UniProt)	Protein kinase, catalytic domain
Os01g0892500	Similar to carboxylic ester hydrolase.	AK243057	NP_001151408.1 (RefSeq)	Pectinacetylesterase
Os01g0892600	Pectinacetylesterase family protein.	AK104609 ,AK064882	NP_001151408.1 (RefSeq)	-
Os01g0892800	Integrin-linked protein kinase domain containing protein.	AK100268	Q8SAE5 (UniProt)	Protein kinase, catalytic domain
Os01g0893300	Non-protein coding transcript.	AK063187	NONE	-
Os01g0893400	Zinc finger, TAZ-type domain containing protein.	AK061917	B8A2U0 (UniProt)	Zinc finger, TAZ-type
Os01g0893700	DOMON domain domain containing protein.	ab initio prediction	Q7F451 (UniProt)	DOMON domain
Os01g0894000	Similar to predicted protein.	AK121960	NP_566200.1 (RefSeq)	Metallophosphoesterase domain
Os01g0894050	Myb/SANT-like domain domain containing protein.	BT039927	NP_001176912.1 (RefSeq)	Myb/SANT-like domain
Os01g0894075	Hypothetical protein.	tplb0043a14 (Wheat FLcDNA)	longestORF	-
Os01g0894100	Similar to Transposase (Fragment).	AK111398	NP_001176912.1 (RefSeq)	-
Os01g0894300	Fructokinase (Fragment).	AK120887	Q0JGZ6-2 (UniProt)	Carbohydrate/puine kinase, PfkB, conserved site
Os01g0894500	Sep15/SelM redox domain containing protein.	AK103300	B6T652 (UniProt)	Thioredoxin-like fold
Os01g0894600	Hypothetical conserved gene.	AK059715	A3A0G9 (UniProt)	-
Os01g0894700	Trigger factor, ribosome-binding, bacterial domain containing	AK067623 ,AK104876	B8A7T9 (UniProt)	Trigger factor, ribosome-binding, bacterial
Os01g0895000	Hypothetical protein.	AK107053	longestORF	-
Os01g0915900	Conserved hypothetical protein.	AK243101	NP_001172710.1 (RefSeq)	-
Os01g0916000	Conserved hypothetical protein.	AK106300	A2WYA9 (UniProt)	-
Os01g0916100	Similar to loricrin.	AK109173	NP_001147500.1 (RefSeq)	-
Os01g0916200	Clathrin/coatomer adaptor, adaptin-like, N-terminal domain containing protein.	AK101112	A3A0W6 (UniProt)	Clathrin/coatomer adaptor, adaptin-like, N-terminal
Os01g0916300	Similar to protein binding protein.	AK121061 ,AK103107	NP_001147059.1 (RefSeq)	WW/Rsp5/WWP
Os01g0916350	Hypothetical gene.	AK288280	longestORF	-
Os01g0916400	Similar to Selenium binding protein.	AK071710	B6TFC2 (UniProt)	Selenium-binding protein
Os01g0916600	RNA recognition motif, glycine rich protein domain containing protein.	AK121668 ,AK059446	O24188 (UniProt)	RNA recognition motif domain
Os01g0916700	Conserved hypothetical protein.	AK288011	NP_001045196.1 (RefSeq)	-
Os01g0916800	Similar to predicted protein.	ab initio prediction	NP_001045197.2 (RefSeq)	THO complex, subunitTHOC2, C-terminal
Os01g0916950	Hypothetical gene.	tplb0014o19 (Wheat FLcDNA)	longestORF	-
Os01g0917100	Protein of unknown function DUF1675 domain containing	AK107234	A2WYC7 (UniProt)	Protein of unknown function DUF1675
Os01g0917200	Peptidase, trypsin-like serine and cysteine proteases domain containing protein.	AK288071	Q5JLC5 (UniProt)	-
Os01g0917300	Similar to Cysteine-rich peptide.	AK240939	D7NXT9 (UniProt)	-
Os01g0917400	Hypothetical conserved gene.	AK111958	B9EVV7 (UniProt)	Zinc finger, CCCH-type
Os01g0917500	Hypothetical conserved gene.	AK120933	B9EVV8 (UniProt)	Protein kinase, catalytic domain
Os01g0917700	Similar to wiscott-Aldrich syndrome, C-terminal.	EU972186	NP_001150347.1 (RefSeq)	-
Os01g0917801	Hypothetical conserved gene.	BT060591	B9EVV9 (UniProt)	-
Os01g0917900	Zinc finger, RING/FYVE/PHD-type domain containing protein.	AK109460	B8A8H8 (UniProt)	Zinc finger, RING-type
Os01g0918100	Conserved hypothetical protein.	AK108783	B8A8H9 (UniProt)	-
Os01g0921000	Nucleotide-diphospho-sugar transferase domain containing	AK071688	B8A8F3 (UniProt)	Nucleotide-diphospho-sugar transferase
Os01g0921100	Hypothetical conserved gene.	AK243195	B8A8F4 (UniProt)	Nucleotide-diphospho-sugar transferase
Os01g0921200	Similar to predicted protein.	AK289185	NP_176916.2 (RefSeq)	Glycoside hydrolase, family 63
Os01g0921300	Exostosin-like family protein.	AK119614 ,AK106015	NP_001152546.1 (RefSeq)	Exostosin-like
Os01g0921400	Conserved hypothetical protein.	AK099844	Q8RZJ0 (UniProt)	-
Os01g0921450	Hypothetical protein.	AK374499	longestORF	-
Os01g0921500	Similar to SNF1-related protein kinase regulatory gamma subunit 1 (AKIN gamma1) (AKING1).	AK105517	Q01LJ2 (UniProt)	Cystathionine beta-synthase, core
Os01g0921550	Hypothetical protein.	ab initio prediction	NONE	-
Os01g0921600	Similar to Mitochondrial import receptor subunit TOM20 (Translocase of outer membrane 20 kDa subunit).	AK071344	NP_001149254.1 (RefSeq)	Plant specific mitochondrial import receptor subunit TOM20
Os01g0921800	Tetratricopeptide-like helical domain containing protein.	AK334012	XP_002887115.1 (RefSeq)	Zinc finger, MYND-type
Os01g0922000	Hypothetical protein.	AK073034	longestORF	-
Os01g0922100	Conserved hypothetical protein.	AK110737	A2WYH3 (UniProt)	-
Os01g0922350	Hypothetical protein.	AK373674	longestORF	-
Os01g0922600	Similar to SBP-domain protein 4.	AK062581	Q0JGI1 (UniProt)	Transcription factor, SBP-box
Os01g0922700	Conserved hypothetical protein.	AK287528	NP_001172712.1 (RefSeq)	-
Os01g0922800	Similar to MADS-box protein PTM5.	AK066160	Q198I2 (UniProt)	Transcription factor, MADS-box
Os02g0164800	Zinc finger, C2H2-type domain containing protein.	AK062817	NP_175482.1 (RefSeq)	-
Os02g0164900	Similar to Auxin response factor 3.	AK066725	Q6H6V4 (UniProt)	AUX/IAA protein
Os02g0165000	Zinc finger, RING/FYVE/PHD-type domain containing protein.	AK101610	B8AI80 (UniProt)	Zinc finger, RING-CH-type
Os02g0165100	Protein kinase, core domain containing protein.	AK066645	NP_001148145.1 (RefSeq)	Protein kinase, catalytic domain
Os02g0165200	Hypothetical protein.	AK101881	longestORF	-
Os02g0165500	Conserved hypothetical protein.	AK060547	A2X1A6 (UniProt)	-
Os02g0165800	Conserved hypothetical protein.	AK106983	A2X1A7 (UniProt)	-
Os02g0166200	Non-protein coding transcript.	AK066189	NONE	-
Os02g0263432	Conserved hypothetical protein.	ab initio prediction	B9F4W3 (UniProt)	-
Os02g0305600	Spectrin repeat containing protein.	AK102077	B8AFZ4 (UniProt)	-
Os02g0305700	Armadillo-type fold domain containing protein.	AK099254	B6TWZ9 (UniProt)	Armadillo-like helical
Os02g0305800	EKC/KEOPS complex, subunit Pcc1 domain containing protein.	AK066730	NP_001172913.1 (RefSeq)	EKC/KEOPS complex, subunit Pcc1
Os02g0305950	Similar to calmodulin binding protein.	tplb0019a16 (Wheat FLcDNA)	NP_001149909.1 (RefSeq)	Auxin responsive SAUR protein
Os02g0306100	Similar to cation cation antiporter.	AK066324 (DDBJ, Secondary hit)	NP_001148426.1 (RefSeq)	Vesicle transport protein, Use1
Os02g0306250	EKC/KEOPS complex, subunit Pcc1 domain containing protein.	AK058382 (DDBJ, Secondary hit)	NP_001172915.1 (RefSeq)	EKC/KEOPS complex, subunit Pcc1
Os02g0433600	Helix-loop-helix DNA-binding domain containing protein.	AK069790 ,AK103986	B6UE48 (UniProt)	Helix-loop-helix DNA-binding
Os02g0598300	Conserved hypothetical protein.	AK105842	Q6K5K0 (UniProt)	-
Os02g0598350	Hypothetical gene.	BT016907	longestORF	-
Os02g0598400	Lipovitellin, superhelical domain containing protein.	AK068111	Q6K1U1 (UniProt)	Telomere length regulation protein, conserved domain
Os02g0598500	Protein phosphatase 2C family protein.	AK106903 (DDBJ, Secondary hit)	NP_001047333.1 (RefSeq)	Protein phosphatase 2C, manganese/magnesium aspartate binding site
Os02g0598600	Cyclin-like F-box domain containing protein.	AK105564	Q6K1T5 (UniProt)	F-box domain, cyclin-like
Os02g0598800	Telomere length regulation protein, conserved domain domain containing protein.	AK372629	NP_001047336.2 (RefSeq)	Telomere length regulation protein, conserved domain
Os02g0598900	Cyclin-like F-box domain containing protein.	AK105564	Q6K1T5 (UniProt)	F-box domain, cyclin-like
Os02g0599100	Hypothetical conserved gene.	tplb0009a09 (Wheat FLcDNA)	Q6K1U1 (UniProt)	Telomere length regulation protein, conserved domain
Os02g0599150	Hypothetical conserved gene.	AK106903	Q6K1U4 (UniProt)	Protein phosphatase 2C, manganese/magnesium aspartate binding site
Os02g0599151	Probable protein phosphatase 2C 17.	Q6K1U0 (UniProt)	Q6K1U0 (UniProt)	Protein phosphatase 2C, manganese/magnesium aspartate binding site
Os02g0675400	Similar to OSIGBa0111L12.2 protein.	AK362424	Q01J00 (UniProt)	Cytochrome P450
Os02g0675550	Hypothetical protein.	tplb0036f19 (Wheat FLcDNA)	longestORF	-
Os02g0675700	Protein of unknown function DUF248, methyltransferase putative family protein.	AK065315	A6MZN0 (UniProt)	Protein of unknown function DUF248, methyltransferase putative
Os02g0675750	Hypothetical gene.	EU963650	GeneMark	-
Os02g0675800	Cyclin-like F-box domain containing protein.	AK100726	Q01JM6 (UniProt)	F-box domain, cyclin-like
Os02g0676000	Similar to predicted protein.	AK059030	XP_002892700.1 (RefSeq)	Membrane bound O-acyl transferase, MBOAT
Os02g0676400	Multi antimicrobial extrusion protein MatE family protein.	BT083598	Q01JM2 (UniProt)	Multi antimicrobial extrusion protein
Os02g0676450	Non-protein coding transcript.	AK287818	NONE	-
Os02g0676500	Similar to OSIGBa0127A14.6 protein.	CT835321	Q01JM1 (UniProt)	Zinc finger, RING-type
Os02g0770500	Similar to WRKY transcription factor 32.	AK363247	Q6IEP9 (UniProt)	DNA-binding WRKY
Os02g0770600	Protein of unknown function DUF1644 family protein.	AK101234	Q2Z1Y7 (UniProt)	Protein of unknown function DUF1644
Os02g0770700	Peptidase C50, separase domain containing protein.	AK106494	B9F3G4 (UniProt)	Peptidase C50, separase
Os02g0770800	Similar to Nitrate reductase [NAD(P)H] (EC 1.7.1.2).	AK102178 ,AK102602	P27968 (UniProt)	Oxidoreductase, molybdopterin-binding domain
Os02g0771100	Similar to COP1 (Fragment).	AK111614	Q947M8 (UniProt)	WD40 repeat
Os02g0771200	Hypothetical conserved gene.	AK071059	A2XA27 (UniProt)	-
Os02g0771400	Protein-tyrosine phosphatase, SIW14-like domain containing	ab initio prediction	B6TUQ6 (UniProt)	Protein-tyrosine phosphatase, SIW14-like
Os02g0771450	Hypothetical conserved gene.	AK109837 (DDBJ, Secondary hit)	B8A8B2 (UniProt)	-
Os02g0771500	Conserved hypothetical protein.	AK062335 ,AK106018	B9F3G5 (UniProt)	-
Os02g0771600	Similar to 1-aminocyclopropane-1-carboxylate oxidase	AK103969 ,AK071557	O65031 (UniProt)	Oxoglutarate/iron-dependent oxygenase
Os02g0771666	Hypothetical gene.	BT019229	longestORF	-
Os02g0771700	Glycoside hydrolase, family 17 protein.	AK058571 ,AK102185	Q9ZNY6 (UniProt)	Glycoside hydrolase, family 17
Os02g0771800	Similar to predicted protein.	AK243530	B8AJ71 (UniProt)	-
Os02g0771900	Non-protein coding transcript.	FP091874	NONE	-
Os02g0772000	Similar to global transcription factor group.	AK334486	XP_002879484.1 (RefSeq)	Transcription elongation factor Spt5, NGN domain
Os03g0110300	Conserved hypothetical protein.	AK106206 ,AK066190 ,AK06676	B9FAD8 (UniProt)	-
Os03g0110400	Hypothetical conserved gene.	AK059259	B8ALF8 (UniProt)	THO complex, subunit THOC1
Os03g0110500	KIP1-like domain containing protein.	AK101157	NP_001149784.1 (RefSeq)	KIP1-like
Os03g0110600	Similar to LysM domain containing protein, expressed.	AK356815	Q10SU7 (UniProt)"	-
Os03g0110700	Hypothetical protein.	tplb0029n18 (Wheat FLcDNA)	longestORF	-
Os03g0110800	Similar to DNA methyltransferase.	AK065147	B6UD67 (UniProt)	C-5 cytosine methyltransferase

Os03g0110900	Dimeric alpha-beta barrel domain containing protein.	AK070403	Q10SU2 (UniProt)	Dimeric alpha-beta barrel
Os03g0111000	Conserved hypothetical protein.	AK062759	B8ALG1 (UniProt)	-
Os03g0111100	Similar to Dihydrofolate synthetase /folylpolyglutamate	AK102025	B6SW32 (UniProt)	Folylpolyglutamate synthetase
Os03g0111200	Similar to Remorin.	AK060379	B6U3W6 (UniProt)	Remorin, C-terminal
Os03g0111300	Nonspecific lipid-transfer protein 2 (nsLTP2) (7 kDa lipid transfer protein).	AK062506	Q10ST8 (UniProt)	Plant lipid transfer protein/hydrophobic protein, helical domain
Os03g0111400	Heavy metal transport/detoxification protein domain containing protein.	AK243276	Q01II0 (UniProt)	Heavy metal-associated domain, HMA
Os03g0111500	Hypothetical conserved gene.	AK067241	Q10ST5 (UniProt)	-
Os03g0111600	Protein of unknown function DUF1618 domain containing	AK101020	A3ADD8 (UniProt)	Domain of unknown function DUF1618
Os03g0111700	Similar to TWN2 (TWIN 2)%3B ATP binding / aminoacyl-tRNA ligase/ nucleotide binding / valine-tRNA ligase.	AK066973	NP_172913.1 (RefSeq)	-
Os03g0111800	WD40/YVTN repeat-like domain containing protein.	AK121627	B9FAE6 (UniProt)	WD40 repeat
Os03g0112101	Similar to Adaptin N terminal region family protein, expressed.	CT835180	Q10SS6 (UniProt)	Clathrin/coatomer adaptor, adaptin-like, N-terminal
Os03g0112400	Clathrin/coatomer adaptor, adaptin-like, appendage, C-terminal subdomain domain containing protein.	AK063082	Q10SS6 (UniProt)	Clathrin adaptor, alpha-adaptin, appendage, C-terminal subdomain
Os03g0112600	Similar to Plus-3 domain containing protein, expressed.	EU942983	Q7XCX5 (UniProt)	SWIB/MDM2 domain
Os03g0112700	Zinc finger, CCCH-type domain containing protein.	AK112095	Q10SS3 (UniProt)	Zinc finger, CCCH-type
Os03g0112800	Protein of unknown function DUF726 family protein.	AK100572	A2XBP3 (UniProt)	Protein of unknown function DUF726
Os03g0112900	Similar to Aldehyde 5-hydroxylase.	AB207253	B6SWS3 (UniProt)	Cytochrome P450
Os03g0112950	Non-protein coding transcript.	AK242233	NONE	-
Os03g0113000	Serine/threonine protein kinase domain containing protein.	AK058286 ,AK104314	NP_001151054.1 (RefSeq)	Protein kinase, catalytic domain
Os03g0113100	Thymidine kinase family protein.	AK068779	Q10SR8 (UniProt)	Thymidine kinase
Os03g0113200	Protein of unknown function DUF295 family protein.	AK109767	Q10SR6 (UniProt)	Protein of unknown function DUF295
Os03g0113500	Conserved hypothetical protein.	AK241800	B9FAE9 (UniProt)	-
Os03g0113700	Similar to Heat shock 70 kDa protein, mitochondrial precursor.	AK103835	NP_001151739.1 (RefSeq)	Heat shock protein Hsp70
Os03g0113750	Hypothetical protein.	tplb0023b01 (Wheat FLcDNA)	longestORF	-
Os03g0113800	Tetratricopeptide-like helical domain containing protein.	AK065925	XP_002870606.1 (RefSeq)	Tetratricopeptide-like helical
Os03g0124000	Homeodomain-related domain containing protein.	AK121207	B6SZ15 (UniProt)	Homeobox
Os03g0124033	Hypothetical protein.	tplb0025a22 (Wheat FLcDNA)	longestORF	-
Os03g0124100	Protein of unknown function DUF604 domain containing protein.	AK107007	Q01KI0 (UniProt)	Protein of unknown function DUF604
Os03g0124200	Similar to Protein kinase domain containing protein, expressed.	AK105735	A2XBX8 (UniProt)	Protein kinase, catalytic domain
Os03g0124300	Similar to ATP binding protein.	AK069148	B6TWY5 (UniProt)	Malectin-like carbohydrate-binding domain
Os03g0124500	Conserved hypothetical protein.	AK119960	B8ALZ1 (UniProt)	-
Os03g0124800	Non-protein coding transcript.	AK121064	NONE	-
Os03g0124900	Pectin lyase fold/virulence factor domain containing protein.	AK070284	NP_001151102.1 (RefSeq)	Glycoside hydrolase, family 28
Os03g0125000	Similar to 50S ribosomal protein L5, chloroplast.	AK098999 ,AK059557 ,AK10413	Q9ZST0 (UniProt)	Ribosomal protein L5
Os03g0125100	Beta-carotene hydroxylase, Drought and oxidative stress	AK287823 (Genbank)	Q10SE7 (UniProt)	-
Os03g0125300	D111/G-patch domain containing protein.	AK121790 ,AK065004	B6TBI8 (UniProt)	D111/G-patch
Os03g0125400	Similar to D-erythro-sphingosine kinase/ diacylglycerol kinase.	AK101342	B6TDW8 (UniProt)	Diacylglycerol kinase, catalytic domain
Os03g0125600	Ser Thr specific protein kinase-like protein.	AK243600	Q10SE3 (UniProt)	Protein kinase, catalytic domain
Os03g0125650	Hypothetical gene.	AK289045	longestORF	-
Os03g0125700	Ubiquitin ligase, Det1/DDB1-complexing domain containing	AK062913	B8ALZ5 (UniProt)	Ubiquitin ligase, Det1/DDB1-complexing
Os03g0125800	Cystathionine beta-synthase, core domain containing protein.	AK102496 ,AK100164	B6SVJ7 (UniProt)	Domain of unknown function DUF21
Os03g0125900	Hypothetical conserved gene.	AK071921	Q10SD7 (UniProt)	-
Os03g0126000	Similar to Phosphorybosyl anthranilate transferase 1.	AK121680	NP_001130955.1 (RefSeq)	Glycosyl transferase, family 3
Os03g0126100	Similar to Arabidopsis thaliana DNA, 40 kb surrounding ACS1	AK111921	NP_001151564.1 (RefSeq)	D-isomer specific 2-hydroxyacid dehydrogenase, NAD-binding
Os03g0126300	Similar to cDNA clone:001-042-G03, full insert sequence.	AK100841	Q10SD2 (UniProt)	-
Os03g0126450	Domain of unknown function DUF623 domain containing protein.	EU952385	Q8S5W2 (UniProt)	Domain of unknown function DUF623
Os03g0126600	Conserved hypothetical protein.	AK243575	Q8S5W1 (UniProt)	-
Os03g0126700	Similar to Barley stem rust resistance protein.	AK099929	Q2QSQ6 (UniProt)	Heavy metal-associated domain, HMA
Os03g0126800	Similar to CBL-interacting protein kinase 9.	FJ901199	Q10SC8 (UniProt)	Protein kinase, catalytic domain
Os03g0126825	Hypothetical gene.	AK242859	longestORF	-
Os03g0126850	Conserved hypothetical protein.	AK241174	NP_001173247.1 (RefSeq)	-
Os03g0126900	Conserved hypothetical protein.	AK109217	A2XBZ9 (UniProt)	-
Os03g0127000	Similar to cell growth defect factor 2.	AK068479	NP_001148819.1 (RefSeq)	-
Os03g0127100	Hypothetical gene.	AK120780	longestORF	-
Os03g0127500	bZIP transcription factor, bZIP-1 domain containing protein.	EU971509	NP_001151643.1 (RefSeq)	Basic-leucine zipper
Os03g0127600	Forkhead-associated domain containing protein.	AK103695	Q8S5V4 (UniProt)	Forkhead-associated
Os03g0127650	Non-protein coding transcript.	BT017530	NONE	-
Os03g0127700	Serine/threonine protein kinase-related domain containing protein.	AK103401	Q9LQ11 (UniProt)	Protein kinase, catalytic domain
Os03g0127800	Non-protein coding transcript.	EU947022	NONE	-
Os03g0127900	Cation/H+ exchanger domain containing protein.	AK062977	NP_001147290.1 (RefSeq)	Cation/H+ exchanger
Os03g0127950	Similar to inner membrane protein ybaL.	CT835134	NP_001147290.1 (RefSeq)	-
Os03g0128000	Similar to Fasciclin-like protein FLA2.	AK062278	Q06IA4 (UniProt)	FAS1 domain
Os03g0128100	Similar to 1,3-beta-glucan synthase component family protein, expressed.	AK105008	Q8S5U9 (UniProt)	Glycosyl transferase, family 48
Os03g0128200	Similar to predicted protein.	AK243268	Q9LXT9 (UniProt)	Protein of unknown function DUF605
Os03g0128300	Conserved hypothetical protein.	AK064718	B8AMD0 (UniProt)	-
Os03g0128400	Conserved hypothetical protein.	AK063116	A2XC10 (UniProt)	-
Os03g0128500	Similar to DNA polymerase delta small subunit (EC 2.7.7.7).	AK067991	Q9LRE5 (UniProt)	DNA polymerase alpha/epsilon, subunit B
Os03g0128600	Conserved hypothetical protein.	AK242075	NP_001173249.1 (RefSeq)	-
Os03g0128700	Calcium-dependent protein kinase, isoform 11 (EC 2.7.1.-) (CDPK 11).	AK066500	P53684 (UniProt)	Protein kinase, catalytic domain
Os03g0128800	Similar to predicted protein.	AK109497	Q10SA9 (UniProt)	-
Os03g0128866	Non-protein coding transcript.	tplb0045g05 (Wheat FLcDNA)	NONE	-
Os03g0128932	Similar to Major facilitator superfamily protein, expressed.	BT009593	Q8H887 (UniProt)	Sugar/inositol transporter
Os03g0129000	GPI biosynthesis protein Pig-F domain containing protein.	AK073895	Q0PGJ2 (UniProt)	GPI biosynthesis protein Pig-F
Os03g0129100	Seven transmembrane protein MLO2.	AK098993 ,AK111990	Q94EX3 (UniProt)	Mlo-related protein
Os03g0151000	Conserved hypothetical protein.	AF216531	Q5NAS3 (UniProt)"	-
Os03g0151100	Appr-1-p processing domain containing protein.	AK099979	NP_001152046.1 (RefSeq)	Appr-1-p processing
Os03g0151201	Non-protein coding transcript.	AK288979	NONE	-
Os03g0151300	Similar to JmjC domain containing protein, expressed.	AK110927	Q10RP5 (UniProt)	Transcription factor jumonji/aspartyl beta-hydroxylase
Os03g0151400	Similar to Zinc finger, C2H2 type family protein, expressed.	AK110807	Q10RP4 (UniProt)	-
Os03g0151500	Conserved hypothetical protein.	AK109181	A3AE69 (UniProt)	-
Os03g0151600	Similar to N-acetyltransferase.	AK073240	B6T916 (UniProt)	GCN5-related N-acetyltransferase
Os03g0151700	Hypothetical conserved gene.	AK111951	B8ANC7 (UniProt)	WD40 repeat
Os03g0151800	Hypothetical conserved gene.	AK059991	XP_002465842.1 (RefSeq)	ATPase, AAA+ type, core
Os03g0151850	Hypothetical gene.	AK373447	longestORF	-
Os03g0151900	Similar to Small GTP-binding protein.	AK105389	Q9FPK1 (UniProt)	Small GTPase superfamily
Os03g0152000	Heavy metal transport/detoxification protein domain containing protein.	AK102357	A2XCL8 (UniProt)	Heavy metal-associated domain, HMA
Os03g0152100	Similar to E2F-DP transcription factor.	AK242229	B4FHH8 (UniProt)	Transcription factor E2F/dimerisation partner
Os03g0152200	Non-protein coding transcript.	BT009415	NONE	-
Os03g0152300	Haem peroxidase family protein.	AK070875	A2XCM0 (UniProt)	Plant peroxidase
Os03g0152400	Similar to 4-coumarate--CoA ligase-like 1.	AK242087	Q0DV32 (UniProt)	AMP-dependent synthetase/ligase
Os03g0152500	Hypothetical protein.	BT062457	longestORF	-
Os03g0152600	Conserved hypothetical protein.	AK109525	B8AND6 (UniProt)	-
Os03g0152700	Pseudouridine synthase domain containing protein.	AK067387	B6U7U0 (UniProt)	RNA-binding S4
Os03g0152800	Similar to predicted protein.	AK062056	Q10RM8 (UniProt)	-
Os03g0152900	Similar to predicted protein.	AK288414	XP_002876276.1 (RefSeq)	Armadillo
Os03g0152950	Non-protein coding transcript.	BT085237	NONE	-
Os03g0153000	Similar to cDNA clone:001-019-C07, full insert sequence.	CT835172	Q337U3 (UniProt)	RNA recognition motif domain
Os03g0153100	Similar to FAD binding domain containing protein, expressed.	BT054188	Q10RM1 (UniProt)	Monooxygenase, FAD-binding
Os03g0182000	Similar to flavin-dependent monooxygenase 1.	BT040713	XP_002893036.1 (RefSeq)	Flavin monooxygenase-like
Os03g0182350	Non-protein coding transcript.	AK241509	NONE	-
Os03g0182400	Similar to SAC domain protein 1 (FIG4-like protein AtFIG4).	AK100037	Q10QV0 (UniProt)	Synaptojanin, N-terminal
Os03g0182600	Similar to 40S ribosomal protein SA.	AK066899	Q10QU9 (UniProt)	Ribosomal protein S2
Os03g0182700	Eukaryotic translation initiation factor 3 subunit 12 (eIF-3 p25) (eIF3k).	AK073293 ,AK120235	Q94HF1 (UniProt)	-
Os03g0182800	Similar to Ethylene responsive element binding factor3	AK073133	Q8VXC3 (UniProt)	Pathogenesis-related transcriptional factor/ERF, DNA-binding
Os03g0182900	Non-protein coding transcript.	AK103663	NONE	-
Os03g0183000	Similar to Root abundant factor.	AK060929	Q4F8A4 (UniProt)	-
Os03g0183050	Hypothetical gene.	EU941438	longestORF	-
Os03g0183100	SAP-like protein BP-73 (OsBP-73) (Riaa1).	AK067631	Q8L4E7 (UniProt)	Rho termination factor, N-terminal
Os03g0183200	Similar to AP2 domain containing protein, expressed.	AK106987	Q10QU3 (UniProt)	-
Os03g0183300	Similar to BTH-induced ERF transcriptional factor 4.	AK058349	Q5MFV0 (UniProt)	-
Os03g0183500	Protein of unknown function DUF581 family protein.	AK063042	NP_197570.1 (RefSeq)	Protein of unknown function DUF581
Os03g0183600	Similar to Alanine aminotransferase.	Z26322	Q9S768 (UniProt)	1-aminocyclopropane-1-carboxylate synthase
Os03g0183800	Similar to Leucine-rich repeat transmembrane protein kinase 1 (Fragment).	AK112046	O81105 (UniProt)	Protein kinase, catalytic domain
Os03g0183850	Hypothetical protein.	tplb0047d04 (Wheat FLcDNA)	longestORF	-
Os03g0183900	Similar to Plasma membrane H+-ATPase.	AK242894	Q8RW26 (UniProt)	ATPase, P-type, H+ transporting proton pump
Os03g0183950	Hypothetical protein.	tplb0039e05 (Wheat FLcDNA)	longestORF	-

Os03g0304800	Lg106-like family protein.	AK062656	NP_001148326.1 (RefSeq)	Endosulphine
Os03g0305000	AMP-binding protein.	AK243161	Q10ML0 (UniProt)	AMP-dependent synthetase/ligase
Os03g0305050	Hypothetical gene.	EU941562	longestORF	-
Os03g0305100	Similar to AMP-binding protein.	AK101765	B6SS27 (UniProt)	AMP-dependent synthetase/ligase
Os03g0305150	Hypothetical protein.	EU941562	longestORF	-
Os03g0305200	Conserved hypothetical protein.	AK108450	A2XFT0 (UniProt)	-
Os03g0305400	UspA domain containing protein.	AK071397	Q10MK6 (UniProt)	Universal stress protein A
Os03g0305500	Similar to Argininosuccinate lyase.	AK070233	B4FAV4 (UniProt)	Fumarate lyase
Os03g0305550	Hypothetical gene.	EU949424	longestORF	-
Os03g0305600	Mitochondrial import inner membrane translocase, subunit Tim17/22 family protein.	AK063714	Q2HU23 (UniProt)	Mitochondrial inner membrane translocase subunit Tim17/Tim22/Tim23/peroxisomal protein PMP24
Os03g0305700	Similar to Peptide chain release factor 2 (Fragment).	AK073807	NP_001148129.1 (RefSeq)	Peptide chain release factor class I/class II
Os03g0305800	Galactosyl transferase family protein.	AK105101 ,AK105005	A2XFT5 (UniProt)	-
Os03g0305950	Similar to Xyloglucan 6-xylosyltransferase (EC 2.4.2.39) [illegible]	AK241375	longestORF	-
Os03g0306100	Galactosyl transferase family protein.	AK335833	NP_001151451.1 (RefSeq)	Galactosyl transferase
Os03g0306200	Similar to transducin family protein / WD-40 repeat family [illegible]	AK241828	XP_002865733.1 (RefSeq)	Bromodomain
Os03g0306302	Hypothetical conserved gene.	DQ245981	Q10MJ9 (UniProt)	-
Os03g0306400	Similar to Lon protease homolog, mitochondrial.	AK288546	A2YQ56 (UniProt)	-
Os03g0306700	Conserved hypothetical protein.	AK109929	A3AH67 (UniProt)	-
Os03g0306800	Similar to CP12 (Fragment).	AK103722	B7XB92 (UniProt)	Domain of unknown function CP12
Os03g0306900	Similar to Seed maturation protein PM36.	AK063807	B6TPF2 (UniProt)	TENA/THI-4 protein/Coenzyme PQQ biosynthesis protein C domain
Os03g0307000	Conserved hypothetical protein.	AK243201	NP_001049892.2 (RefSeq)	-
Os03g0307100	Peptidase S9A, oligopeptidase, N-terminal beta-propeller domain containing protein.	AK102205	XP_002882118.1 (RefSeq)	Peptidase S9, prolyl oligopeptidase, catalytic domain
Os03g0803900	Similar to predicted protein.	AK288442	NP_201068.1 (RefSeq)	Galectin, carbohydrate recognition domain
Os03g0804000	Hypothetical protein.	AK059908	longestORF	-
Os03g0804100	Conserved hypothetical protein.	FP100876	NP_001051616.1 (RefSeq)	-
Os03g0804200	Bifunctional inhibitor/plant lipid transfer protein/seed storage domain containing protein.	AK241129	A2XN28 (UniProt)	Plant lipid transfer protein/Par allergen
Os03g0804300	Zinc finger, DHHC-type domain containing protein.	AK104353 ,AK106099	NP_001148846.1 (RefSeq)	Zinc finger, DHHC-type, palmitoyltransferase
Os03g0804400	Conserved hypothetical protein.	AK073430	Q75HJ9 (UniProt)	-
Os03g0804500	Similar to Germin-like protein subfamily T member 1 precursor.	AK062698	Q10BU2 (UniProt)	Germin
Os03g0804600	Similar to Germin-like protein 3-7.	AK248846	NP_001051622.2 (RefSeq)	-
Os03g0804700	Germin-like protein 3-8.	Q75HJ4 (UniProt)	Q75HJ4 (UniProt)	Germin
Os03g0804800	Similar to Cct8-prov protein.	AK100651	C6F1N7 (UniProt)	Chaperonin TCP-1, conserved site
Os03g0804900	UDP-glucuronosyl/UDP-glucosyltransferase family protein.	AK373315	NP_001051624.1 (RefSeq)	UDP-glucuronosyl/UDP-glucosyltransferase
Os03g0805100	Similar to Squalene synthase (EC 2.5.1.21).	AK242592	NP_001104839.1 (RefSeq)	Squalene/phytoene synthase
Os03g0805200	Similar to RNA helicase (Fragment).	AK066048	Q75HJ0 (UniProt)	Helicase, C-terminal
Os03g0805300	Similar to Phosphoprotein phosphatase 2A isoform 4.	AK060885 ,AK099257 ,AK10035[illegible]	Q10BT5 (UniProt)	Metallophosphoesterase domain
Os03g0805350	Hypothetical protein.	BT018461	longestORF	-
Os03g0805400	Similar to phosphoric ester hydrolase.	AK060174	NP_001151171.1 (RefSeq)	Phosphatidic acid phosphatase type 2/haloperoxidase
Os03g0805500	Similar to AAE18 (ACYL-ACTIVATING ENZYME 18)%3B catalytic/ ligase.	AK067857	NP_175929.3 (RefSeq)	-
Os03g0805600	Similar to pheophorbide a oxygenase.	CT835283	B6ST74 (UniProt)	Rieske [2Fe-2S] iron-sulphur domain
Os03g0805700	Similar to Pheophorbide a oxygenase.	EU955939	B6ST74 (UniProt)	Pheophorbide a oxygenase
Os03g0805733	Hypothetical protein.	tplb0031c13 (Wheat FLcDNA)	longestORF	-
Os03g0805766	Hypothetical protein.	tplb0031c13 (Wheat FLcDNA)	longestORF	-
Os03g0806400	Similar to Elongation factor P family protein, expressed.	AK073074	Q10BS5 (UniProt)	-
Os03g0806500	Thioredoxin domain 2 containing protein.	AK073308 ,AK100198	Q84M47 (UniProt)	Thioredoxin-like fold
Os03g0806600	Conserved hypothetical protein.	AK070959	B9F6V2 (UniProt)	-
Os03g0806700	Protein of unknown function DUF868, plant family protein.	AK062034	XP_002466252.1 (RefSeq)	Protein of unknown function DUF868, plant
Os03g0806800	Conserved hypothetical protein.	AK120337	A2XN54 (UniProt)	-
Os03g0806900	Similar to Cytochrome-C reductase 14 kDa subunit (EC 1.10.2.2) (Fragment).	AK121708	B6UBZ9 (UniProt)	Cytochrome d ubiquinol oxidase, 14kDa subunit
Os03g0807000	Conserved hypothetical protein.	AK241139	NP_001173679.1 (RefSeq)	-
Os03g0807100	Protein of unknown function DUF239 domain containing protein.	AK242472	B6TV70 (UniProt)	Glucoamylase, putatuve
Os03g0807150	Hypothetical protein.	tplb0032e19 (Wheat FLcDNA)	longestORF	-
Os03g0807200	Non-protein coding transcript.	AK122072	NONE	-
Os03g0807400	Pentatricopeptide repeat domain containing protein.	AK065345	Q84M45 (UniProt)	Pentatricopeptide repeat
Os03g0807500	Cupredoxin domain containing protein.	CU406844	B6TG00 (UniProt)	Plastocyanin-like
Os03g0807600	Similar to Cyclopropane-fatty-acyl-phospholipid synthase family protein, expressed.	CT835101	Q10BR3 (UniProt)	Methyltransferase type 11
Os03g0807700	Similar to predicted protein.	AK121037	A2XN62 (UniProt)	Protein of unknown function DUF642
Os03g0807800	Similar to 40S ribosomal protein S2 (Fragment).	AK064984	B6TNR8 (UniProt)	Ribosomal protein S5
Os03g0807900	Chaperonin-like RbcX family protein.	AK070287	A2XN64 (UniProt)	Chaperonin-like RbcX
Os03g0808000	Glycoside hydrolase, family 28 domain containing protein.	BT042622	NP_001051647.1 (RefSeq)	Glycoside hydrolase, family 28
Os03g0808100	Similar to Cellulose synthase BoCesA5.	AK061688	Q4U0Z5 (UniProt)	Cellulose synthase
Os03g0808150	Hypothetical gene.	BT019277	longestORF	-
Os03g0808175	Non-protein coding transcript.	U48693	NONE	-
Os03g0808200	UDP-glucuronosyl/UDP-glucosyltransferase family protein.	BT037819	NP_001149762.1 (RefSeq)	UDP-glucuronosyl/UDP-glucosyltransferase
Os04g0334700	Similar to OSIGBa0137O04.7 protein.	AK109756	Q01KZ0 (UniProt)	Peptidase A1
Os04g0334825	Conserved hypothetical protein.	ab initio prediction	Q7X7R4 (UniProt)	-
Os04g0334951	Similar to OSIGBa0137O04.7 protein.	AK109756	Q01KZ0 (UniProt)	Peptidase A1
Os04g0335075	Conserved hypothetical protein.	ab initio prediction	Q7X7R4 (UniProt)	-
Os04g0335200	Conserved hypothetical protein.	AK289258	Q2QWP9 (UniProt)	-
Os04g0335400	Non-protein coding transcript.	CT828838	NONE"	-
Os04g0336001	Hypothetical protein.	EU945299	longestORF	-
Os04g0336600	Peptidase aspartic, catalytic domain containing protein.	AK120870 (DDBJ, Secondary hit)	B7F2P1 (UniProt)	Peptidase A1
Os04g0336700	Conserved hypothetical protein.	ab initio prediction	Q7X7R4 (UniProt)	-
Os04g0336801	Conserved hypothetical protein.	AK289258 (DDBJ, Secondary hit)	Q2QWP9 (UniProt)"	-
Os04g0337000	Peptidase aspartic, catalytic domain containing protein.	AK066236	Q01KZ0 (UniProt)	Peptidase A1
Os04g0417600	Similar to OSIGBa0092M08.12 protein.	EU953424	Q01I40 (UniProt)	VQ
Os04g0417750	Non-protein coding transcript.	CT835081	NONE"	-
Os04g0417800	WD40 repeat-like domain containing protein.	AK067483	Q01I39 (UniProt)	WD40 repeat
Os04g0418000	Conserved hypothetical protein.	AK106155 ,AK061030 ,AK09912[illegible]	Q7X6C3 (UniProt)	-
Os04g0418100	Hypothetical conserved gene.	CT835067	B9FF49 (UniProt)	-
Os04g0418300	Conserved hypothetical protein.	ab initio prediction	Q7XQY6 (UniProt)	-
Os04g0418500	Similar to photoperiod responsive protein.	AK121255	NP_001150615.1 (RefSeq)	U box domain
Os04g0418600	Conserved hypothetical protein.	ab initio prediction	Q7XQY4 (UniProt)	-
Os04g0418800	Hypothetical protein.	AK102919	GeneMark	-
Os04g0418900	Non-protein coding transcript.	AK073909	NONE	-
Os04g0419100	Conserved hypothetical protein.	AK107777	B8ATQ0 (UniProt)	-
Os04g0419200	Hypothetical conserved gene.	AK064626	B9FF52 (UniProt)	-
Os04g0419400	Conserved hypothetical protein.	AK107801 (DDBJ, Secondary hit)	NP_001056956.1 (RefSeq)"	-
Os04g0419550	Zinc finger, RING-type domain containing protein.	AK243335	Q7XQX6 (UniProt)	Zinc finger, RING-type
Os04g0419600	Histone H3.	AK288497	XP_003082036.1 (RefSeq)	Histone H3
Os04g0419700	Similar to H0525E10.7 protein.	AK357509	Q01K01 (UniProt)	Protein kinase, catalytic domain
Os04g0419750	Hypothetical protein.	tplb0036o12 (Wheat FLcDNA)	longestORF	-
Os04g0419800	Hypothetical conserved gene.	AK121609	B9FF57 (UniProt)	-
Os04g0419900	Similar to H0525E10.7 protein.	AK110836	Q01K01 (UniProt)	Protein kinase, catalytic domain
Os04g0420033	Similar to H0525E10.1 protein.	BT084608	Q01K07 (UniProt)	Protein kinase, catalytic domain
Os04g0420166	Hypothetical protein.	tplb0036o12 (Wheat FLcDNA)	longestORF	-
Os04g0420300	Similar to H0525E10.1 protein.	AK371960	Q01K07 (UniProt)	Protein kinase, catalytic domain
Os04g0420500	Hypothetical protein.	AK073522	longestORF	-
Os04g0420600	Similar to H0525E10.2 protein.	tplb0059a02 (Wheat FLcDNA)	Q01K06 (UniProt)	Protein kinase, catalytic domain
Os04g0420700	Hypothetical protein.	tplb0028c09 (Wheat FLcDNA)	longestORF	-
Os04g0420801	Hypothetical protein.	tplb0028c09 (Wheat FLcDNA)	longestORF	-
Os04g0420900	Similar to Receptor-like protein kinase.	AK101902	Q01K01 (UniProt)	Protein kinase, catalytic domain
Os04g0429900	Conserved hypothetical protein.	AK063076	Q7XT85 (UniProt)	-
Os04g0430000	Similar to Ser Thr specific protein kinase-like protein.	AK100142	NP_001167697.1 (RefSeq)	Protein kinase, catalytic domain
Os04g0430100	Similar to 40S ribosomal protein S10-1.	AK102190	Q9AYP4 (UniProt)	Plectin/S10, N-terminal
Os04g0430150	Hypothetical gene.	BT019280	longestORF	-
Os04g0430200	Similar to phospholipase C.	AK104956	NP_001150982.1 (RefSeq)	-
Os04g0430400	Hypothetical conserved gene.	AK064161	B9FF85 (UniProt)	Protein kinase, catalytic domain
Os04g0430600	Similar to OSIGBa0160I14.1 protein.	AK106337 ,AK072050	Q01JS2 (UniProt)	-
Os04g0430700	Peptidase S8, subtilisin-related domain containing protein.	AK105112	Q01JS1 (UniProt)	Peptidase S8/S53, subtilisin/kexin/sedolisin
Os04g0430800	Similar to OSIGBa0160I14.3 protein.	AK287766	Q01JS0 (UniProt)	Thioredoxin
Os04g0430900	Magnesium transporter MRS2-F.	Q7XQQ1 (UniProt)	Q8L4S2 (UniProt)	-
Os04g0430950	Hypothetical protein.	tplb0059h12 (Wheat FLcDNA)	longestORF	-
Os04g0431000	Conserved hypothetical protein.	AK111961	Q7XQQ0 (UniProt)	-
Os04g0431100	Similar to GrpE protein homolog.	AK105181	A2XTK3 (UniProt)	GrpE nucleotide exchange factor
Os04g0431200	Similar to OSIGBa0160I14.7 protein.	AK064823	Q01JR6 (UniProt)	F-box domain, cyclin-like
Os04g0431300	Acyl-CoA N-acyltransferase domain containing protein.	AK063893	NP_565157.1 (RefSeq)	Acyl-CoA N-acyltransferase
Os04g0431700	Similar to OSIGBa0160I14.9 protein.	AK243005	Q01JR4 (UniProt)	Leucine-rich repeat

Os04g0431800	Conserved hypothetical protein.	AK068094	Q7XQP5 (UniProt)	-
Os04g0432000	Serine/threonine-protein kinase SAPK7 (EC 2.7.1.37) (Osmotic stress/abscisic acid-activated protein kinase 7).	AK122087	Q7XQP4 (UniProt)	Protein kinase, catalytic domain
Os04g0432100	Transcription factor GRAS domain containing protein.	BT018988	Q7XQP2 (UniProt)	Transcription factor GRAS
Os04g0432200	Hypothetical protein.	CT837490	longestORF"	-
Os04g0432250	Conserved hypothetical protein.	FP091622	Q7XQP1 (UniProt)	-
Os04g0432300	Similar to 4-alpha-L-fucosyltransferase.	AM162282	Q2WBL8 (UniProt)	Glycosyl transferase, family 10
Os04g0529400	Similar to OO_Ba0013J05-OO_Ba0033A15.30 protein.	AK099627	D0ABH3 (UniProt)	WD40 repeat
Os04g0529500	Similar to cDNA clone:J023022A09, full insert sequence.	AK059419	B7EGJ9 (UniProt)	Double-stranded RNA-binding
Os04g0529600	Lanthionine synthetase C-like family protein.	AK288141	Q00RJ6 (UniProt)	Lanthionine synthetase C-like
Os04g0529700	Glycosyltransferase sugar-binding region containing DXD motif domain containing protein.	AK121532	Q00RJ5 (UniProt)	Glycosyltransferase, DXD sugar-binding motif
Os04g0529800	Similar to OSIGBa0155K17.6 protein.	AK101735	Q00RJ4 (UniProt)	Sugar/inositol transporter
Os04g0530000	Similar to OSIGBa0155K17.8 protein.	AK066169	Q00RJ2 (UniProt)	Transcription factor TFIIIC, tau55-related
Os04g0530050	Hypothetical protein.	ab initio prediction	NONE	-
Os04g0530100	Similar to Beta-expansin 1 precursor (AtEXPB1) (At-EXPB1) (Ath-ExpBeta-1.5).	AK107184	Q7X6J9 (UniProt)	Expansin/pollen allergen, DPBB domain
Os04g0530150	Non-protein coding transcript.	AK109871	NONE	-
Os04g0530200	Similar to OSIGBa0155K17.10 protein.	AK105586 ,AK121439	Q00RJ0 (UniProt)	-
Os04g0530300	Similar to OSIGBa0155K17.11 protein.	AK066705	Q00RI9 (UniProt)	-
Os04g0530400	t-snare domain containing protein.	AK067634	B6TDT7 (UniProt)	SNARE-complex protein Syntaxin-18 N-terminal
Os04g0530500	Zinc finger, RING/FYVE/PHD-type domain containing protein.	AK104359	NP_001152715.1 (RefSeq)	Zinc finger, RING-type
Os04g0530600	Similar to Thioredoxin 1 (TRX-1) (Thioredoxin M).	AK069195	NP_001150752.1 (RefSeq)	Thioredoxin
Os04g0530700	Similar to Beta-D-xylosidase.	AK120331	NP_196618.1 (RefSeq)	Glycoside hydrolase, family 3, N-terminal
Os04g0530801	Hypothetical gene.	EU973535	longestORF	-
Os04g0530900	Glycosyl transferase, family 8 protein.	AK120509	XP_002312381.1 (RefSeq)	Glycosyl transferase, family 8
Os04g0531100	C2 calcium-dependent membrane targeting domain containing	AK063584	Q0JBH9 (UniProt)	C2 calcium-dependent membrane targeting
Os04g0531200	Hypothetical conserved gene.	AB332066	A2XVT7 (UniProt)	-
Os04g0531300	tRNA-dihydrouridine synthase domain containing protein.	AK072647	Q00RI1 (UniProt)	tRNA-dihydrouridine synthase
Os04g0531400	Similar to Lectin-like receptor kinase 7%3B2.	AK105289	Q00RI0 (UniProt)	Protein kinase, catalytic domain
Os04g0531500	Concanavalin A-like lectin/glucanase domain containing protein.	AK102285	Q00RH9 (UniProt)	Protein kinase, catalytic domain
Os04g0531600	Hypothetical protein.	AK107678	longestORF	-
Os04g0531700	Short-chain dehydrogenase/reductase SDR domain containing protein.	AK119481	Q00RH9 (UniProt)	Short-chain dehydrogenase/reductase SDR
Os04g0531750	Similar to OSIGBa0125M19.13 protein.	ab initio prediction	Q00RH3 (UniProt)	Short-chain dehydrogenase/reductase SDR
Os04g0531800	Conserved hypothetical protein.	AK121355	B8ASD8 (UniProt)	-
Os04g0531900	Short-chain dehydrogenase/reductase SDR domain containing protein.	AK071069	Q00RH3 (UniProt)	Short-chain dehydrogenase/reductase SDR
Os04g0532100	Short-chain dehydrogenase/reductase SDR domain containing protein.	AK109281	Q00RH5 (UniProt)	Short-chain dehydrogenase/reductase SDR
Os04g0532200	Conserved hypothetical protein.	AK110853	B8ASE2 (UniProt)	-
Os04g0532400	Similar to OSIGBa0125M19.13 protein.	AK318556	Q00RH3 (UniProt)	Short-chain dehydrogenase/reductase SDR
Os04g0532500	Similar to Transcription factor L2.	AK069907	Q00RH2 (UniProt)	Zinc finger, LIM-type
Os04g0532700	Hypothetical protein.	AK109796	longestORF	-
Os04g0532800	Myb transcription factor domain containing protein.	AK107135	B4FNQ6 (UniProt)	SANT domain, DNA binding
Os04g0533000	Similar to RNA helicase (Fragment).	AK071636	B7EKD5 (UniProt)	RNA helicase, ATP-dependent, DEAD-box, conserved site
Os04g0533200	Similar to Myb7 protein (Fragment).	AK376409	Q43598 (UniProt)	-
Os04g0533250	Hypothetical gene.	EU971393	longestORF	-
Os04g0533300	Similar to remorin.	AK104678 ,AK061351	NP_001159012.1 (RefSeq)	Remorin, C-terminal
Os04g0533500	Cytochrome b561 family protein.	AK061426 ,AK069219	Q01II4 (UniProt)	-
Os04g0533602	Hypothetical genes.	ab initio prediction	NONE	-
Os04g0533700	Putative non-inhibitory serpin-10.	Q7XMK1 (UniProt)	Q7XMK1 (UniProt)	Protease inhibitor I4, serpin
Os04g0565750	Hypothetical gene.	CT837494	longestORF	-
Os04g0565900	Basic helix-loop-helix dimerisation region bHLH domain containing protein.	AK109094	Q01KG1 (UniProt)	Helix-loop-helix DNA-binding
Os04g0566000	Similar to OSIGBa0158F05.10 protein.	AK107723	Q01KG0 (UniProt)	-
Os04g0566100	Similar to OSIGBa0158F05.11 protein.	tplb0009k14 (Wheat FLcDNA)	Q01KF9 (UniProt)	SNF2-related
Os04g0566300	Hypothetical protein.	AK062357	longestORF	-
Os04g0566400	Similar to INDETERMINATE-related protein 9.	AK120723	Q5UDB6 (UniProt)	Zinc finger, C2H2
Os04g0566450	Hypothetical gene.	BT086922	longestORF	-
Os04g0566500	Similar to Argonaute protein.	AK111587	Q7XSA2-2 (UniProt)	Argonaute/Dicer protein, PAZ
Os04g0566550	Hypothetical gene.	BT016774	longestORF	-
Os04g0567700	ORMDL family protein.	AK064998	Q01K71 (UniProt)	ORMDL
Os04g0567800	Similar to OSIGBa0103M18.3 protein.	AK060659	Q01K70 (UniProt)	Zinc finger, Dof-type
Os04g0568300	Hypothetical gene.	AK072175	longestORF	-
Os04g0568400	WD40 repeat-like domain containing protein.	AK103546	Q01JP7 (UniProt)	WD40 repeat
Os04g0568500	Conserved hypothetical protein.	AK363942	F2DJ74 (UniProt)	-
Os04g0568600	Similar to 6-phospho-3-hexuloisomerase.	CT836509	Q01JP6 (UniProt)	Sugar isomerase
Os04g0568700	Similar to Heat stress transcription factor Spl7 (Heat shock transcription factor) (Heat shock factor RHSF10).	AY344483	Q01JP5 (UniProt)	Heat shock factor
Os04g0568751	Hypothetical protein.	tplb0033f11 (Wheat FLcDNA)	longestORF	-
Os04g0568800	Similar to OSIGBa0139P06.4 protein.	AK106571	Q01JP4 (UniProt)	Protein of unknown function DUF810
Os04g0568850	Similar to OSIGBa0139P06.4 protein.	CT828553	Q01JP4 (UniProt)	-
Os04g0568900	Similar to RING finger protein 6 (RING-H2 protein).	AK070267	Q01JP3 (UniProt)	Zinc finger, RING-type
Os04g0568950	Hypothetical gene.	EU941385	longestORF	-
Os04g0569000	Similar to Replication factor C 40kDa subunit.	AK069025	Q948P2 (UniProt)	ATPase, AAA+ type, core
Os04g0569100	Similar to OCL1 homeobox protein.	AK112099 ,AK111914	Q7Y0V9-2 (UniProt)	Helix-turn-helix motif, lambda-like repressor
Os04g0569300	Similar to Membrane protein.	AK099082	Q01JP0 (UniProt)	Peptidase S54, rhomboid
Os04g0569400	Similar to OSIGBa0139P06.9 protein.	AK365201	Q01JN9 (UniProt)	Protein of unknown function DUF248, methyltransferase putative
Os04g0569500	Hypothetical protein.	tplb0021o10 (Wheat FLcDNA)	longestORF	-
Os04g0569900	Similar to OSIGBa0111L12.1 protein.	AK059100	Q01J01 (UniProt)	-
Os04g0570000	Cytochrome P450 family protein.	AJ459255	Q7XU38 (UniProt)	Cytochrome P450
Os04g0570125	Hypothetical protein.	tplb0036f19 (Wheat FLcDNA)	longestORF	-
Os04g0580700	MADS box transcription factor MADS17.	AK070540	Q7XUN2 (UniProt)	Transcription factor, MADS-box
Os04g0580800	Zinc finger, RING/FYVE/PHD-type domain containing protein.	AK067005	NP_001150265.1 (RefSeq)	Zinc finger, RING-type
Os04g0580866	Hypothetical conserved gene.	EU969180	Q7XSR3 (UniProt)	Zinc finger, RING-type
Os04g0581000	Similar to Flavanone 3-hydroxylase (Fragment).	AK104142 ,AK061337	Q0H3G8 (UniProt)	Oxoglutarate/iron-dependent oxygenase
Os04g0581050	Hypothetical protein.	tplb0008n13 (Wheat FLcDNA)	longestORF	-
Os04g0581100	2OG-Fe(II) oxygenase domain containing protein.	AK100853	XP_002872519.1 (RefSeq)	Oxoglutarate/iron-dependent oxygenase
Os04g0581300	Similar to Mitochondrial import inner membrane translocase subunit TIM13.	AK063093	Q9XGY5 (UniProt)	Mitochondrial inner membrane translocase complex, Tim8/9/10/13-zinc finger-like
Os04g0581400	B3 DNA binding domain containing protein, Brassinosteroid homeostasis	HM450152 (Genbank),(This transc	D9MNL6 (UniProt)	-
Os04g0581451	Hypothetical protein.	tplb0021g13 (Wheat FLcDNA)	longestORF	-
Os04g0581502	Zinc finger, RING-CH-type domain containing protein.	CT834317	NP_001172654.1 (RefSeq)	Zinc finger, RING-CH-type
Os04g0581600	Similar to UDP-glucose dehydrogenase.	AK101692	NP_001151861.1 (RefSeq)	UDP-glucose/GDP-mannose dehydrogenase, N-terminal
Os04g0581700	Similar to predicted protein.	AK063676	B9FC51 (UniProt)	-
Os04g0581800	Heavy metal transport/detoxification protein domain containing protein.	AK107739	NP_001151445.1 (RefSeq)	Heavy metal-associated domain, HMA
Os04g0642000	Similar to H0423H10.7 protein.	AK105961	Q9FSQ6 (UniProt)	ABC transporter, transmembrane domain
Os04g0642100	Microtubule-associated protein EB1.	AK240692	Q9FSQ5 (UniProt)	Calponin homology domain
Os04g0642300	Similar to H0423H10.10 protein.	ab initio prediction	Q9FSQ3 (UniProt)	-
Os04g0643000	Similar to Cullin-3 (CUL-3). Splice isoform 2.	AK069061	Q25A70 (UniProt)	Cullin, N-terminal
Os04g0643100	Similar to Vacuolar ATP synthase subunit D (EC 3.6.3.14) (V-ATPase D subunit) (Vacuolar proton pump D subunit).	AK106989	Q25A69 (UniProt)	ATPase, V1/A1 complex, subunit D
Os04g0643200	Amidase family protein.	ab initio prediction	Q25A68 (UniProt)	Amidase
Os04g0643300	Similar to 3-ketoacyl carrier protein synthase III.	AK120067	Q25A72 (UniProt)	Beta-ketoacyl-acyl carrier protein synthase III
Os04g0643350	Non-protein coding transcript.	X06283	NONE	-
Os04g0643401	Non-protein coding transcript.	EU947745	NONE	-
Os04g0643500	Similar to H0306F03.8 protein.	AK365086	Q25A67 (UniProt)	Oxoglutarate/iron-dependent oxygenase
Os04g0643550	Hypothetical protein.	tplb0021e03 (Wheat FLcDNA)	longestORF	-
Os04g0643600	Similar to Cyclic nucleotide-gated channel C (Fragment).	AK105170	Q25A66 (UniProt)	IQ motif, EF-hand binding site
Os04g0643700	Similar to H0306F03.10 protein.	AK108565	Q25A65 (UniProt)	Pentatricopeptide repeat
Os04g0643750	Hypothetical protein.	tplb0043j02 (Wheat FLcDNA)	longestORF	-
Os04g0643800	Similar to H0306F03.11 protein.	FP100404	Q25A64 (UniProt)	-
Os04g0644000	Similar to H0306F03.12 protein.	AK100283	Q25A63 (UniProt)	-
Os04g0644050	Hypothetical protein.	tplb0048p04 (Wheat FLcDNA)	longestORF	-
Os04g0644100	Sterile alpha motif homology domain containing protein.	AK106954	Q25A62 (UniProt)	Sterile alpha motif, type 2
Os04g0644200	Similar to H0306F03.14 protein.	AK367692	Q25A61 (UniProt)	-
Os04g0644250	Non-protein coding transcript.	EU949846	NONE	-
Os04g0668600	Kelch-type beta propeller domain containing protein.	AK287832	B4FF73 (UniProt)	F-box domain, cyclin-like
Os04g0668700	Similar to phosphatidylinositol 3- and 4-kinase family protein.	AK062068	NP_001151804.1 (RefSeq)	Phosphatidylinositol 3-/4-kinase, catalytic
Os04g0668800	Putative thiol-disulphide oxidoreductase DCC family protein.	AK288046	Q8W437 (UniProt)	Putative thiol-disulphide oxidoreductase DCC
Os04g0668900	Similar to Plastid terminal oxidase.	AK067891	B8AW16 (UniProt)	Alternative oxidase

Os04g0669100	Conserved hypothetical protein.	AK103123 ,AK070188	B8AW17 (UniProt)	-
Os04g0669200	Similar to Ethylene response factor 2 (Ethylene response factor 2)	AK067373	NP_001148189.1 (RefSeq)	Pathogenesis-related transcriptional factor/ERF, DNA-binding
Os04g0669250	Non-protein coding transcript.	CU406947	NONE"	-
Os04g0669300	EF hand domain containing protein.	AK071148	NP_001030731.1 (RefSeq)	EPS15 homology
Os04g0669375	Non-protein coding transcript.	tplb0032i10 (Wheat FLcDNA)	NONE	-
Os04g0669450	Hypothetical gene.	EU941171	longestORF	-
Os04g0669475	Conserved hypothetical protein.	FP101466	Q7XR65 (UniProt)	-
Os04g0669500	Phospholipase/carboxylesterase domain containing protein.	AK069249	Q259P2 (UniProt)	Phospholipase/carboxylesterase/thioesterase
Os04g0669600	Phospholipase/carboxylesterase domain containing protein.	AK110767	Q259P1 (UniProt)	Phospholipase/carboxylesterase/thioesterase
Os04g0669700	Similar to H0818H01.9 protein.	CT835076	Q259P0 (UniProt)	Phospholipase/carboxylesterase/thioesterase
Os04g0669800	Methylthioribose kinase (EC 2.7.1.100).	AK067649	Q7XR61 (UniProt)	Aminoglycoside phosphotransferase
Os04g0669900	Similar to H0818H01.11 protein.	AY593959 (DDBJ, Secondary hit)	Q259N8 (UniProt)	Aminoglycoside phosphotransferase
Os04g0670000	Reticulon family protein.	AK071792	Q259P4 (UniProt)	Reticulon
Os04g0670100	Similar to H0818H01.13 protein.	FP095236	Q259P3 (UniProt)	-
Os04g0670150	Hypothetical protein.	BT068200	longestORF	-
Os04g0670200	Granulin domain containing protein.	AK099358	GeneMark	Granulin
Os04g0670400	Similar to H0624F09.2 protein.	AK064003	Q258Z6 (UniProt)	Ovarian tumour, otubain
Os04g0670500	Cysteine protease 1 precursor (EC 3.4.22.-) (OsCP1).	AK107506	Q7XR52 (UniProt)	Granulin
Os04g0670600	Similar to H0624F09.5 protein.	AK065924	Q258Z3 (UniProt)	Exostosin-like
Os04g0670700	Similar to H0624F09.4 protein.	AK063069	Q258Z4 (UniProt)	Phosphatidate cytidylyltransferase
Os04g0670800	UBX domain containing protein.	AK072097	Q258Z2 (UniProt)	UBX
Os04g0670900	Homeodomain-like containing protein.	DQ383374	Q258Z1 (UniProt)	MYB-like
Os04g0671100	Similar to H0624F09.8 protein.	AK241125	Q258Z0 (UniProt)	Adenylate kinase
Os04g0671200	Similar to H0624F09.9 protein.	AK106044	Q258Y9 (UniProt)	Amine oxidase
Os04g0671250	Hypothetical protein.	tplb0048c15 (Wheat FLcDNA)	longestORF	-
Os04g0671300	Similar to Suppressor of presenilin 5 (P110b homolog).	AK072414	Q258Y8 (UniProt)	Flavin amine oxidase
Os04g0671500	Hypothetical protein.	tplb0048c15 (Wheat FLcDNA)	longestORF	-
Os04g0671700	ThiJ/PfpI domain containing protein.	AK100753	NP_001146927.1 (RefSeq)	ThiJ/PfpI
Os04g0671800	Similar to H0624F09.12 protein.	AK059421	F1DK83 (UniProt)	-
Os04g0671900	Similar to Auxin response factor 12.	AK104920	Q0J951 (UniProt)	AUX/IAA protein
Os04g0672100	Similar to Phytosulfokine receptor precursor (EC 2.7.1.37) (Phytosulfokine LRR receptor kinase).	AK121689	Q258Z9 (UniProt)	Protein kinase, catalytic domain
Os04g0672200	Poly(ADP-ribose) polymerase, catalytic region domain containing protein.	AK099725	Q258Z8 (UniProt)	Poly
Os04g0672300	Similar to H0322F07.3 protein.	AK101246	Q259A6 (UniProt)	-
Os04g0672600	Leucine-rich repeat, N-terminal domain containing protein.	AK070283	Q258Z9 (UniProt)	Leucine-rich repeat
Os04g0672700	Pentatricopeptide repeat domain containing protein.	AK100434 ,AK102863	Q259A5 (UniProt)	Pentatricopeptide repeat
Os04g0672800	Similar to H0322F07.5 protein.	AK068413	Q259A4 (UniProt)	-
Os04g0672900	Similar to H0322F07.6 protein.	AK062248	Q259A3 (UniProt)	Domain of unknown function DUF2296
Os04g0673000	Similar to H0322F07.7 protein.	AK066211	Q259A2 (UniProt)	Protein of unknown function DUF3755
Os04g0673050	Non-protein coding transcript.	tplb0055d06 (Wheat FLcDNA)	NONE	-
Os04g0673300	A-type response regulator, Cytokinin signaling	AK059734 (Genbank),AB249653 (	Q7XQA6 (UniProt)	Signal transduction response regulator, receiver domain
Os04g0673700	Hypothetical protein.	AK105405	longestORF	-
Os04g0673800	Cupredoxin domain containing protein.	AK071236	Q259S3 (UniProt)	Plastocyanin-like
Os04g0674000	Similar to H0403D02.10 protein.	AK119671 ,AK071276	Q259S8 (UniProt)	-
Os04g0674025	Similar to H0403D02.10 protein.	ab initio prediction	Q259S8 (UniProt)	-
Os04g0674100	Similar to H0403D02.12 protein.	AK242456	Q259S2 (UniProt)	Tetratricopeptide-like helical
Os04g0674200	Coenzyme Q biosynthesis Coq4 family protein.	AK103795 ,AK101965	Q259S1 (UniProt)	Coenzyme Q biosynthesis Coq4
Os04g0674300	Similar to H0403D02.14 protein.	AK059048	Q259S0 (UniProt)	NPH3
Os04g0674350	Conserved hypothetical protein.	AK241498	NP_001174139.1 (RefSeq)	-
Os04g0674400	Similar to Anamorsin (Cytokine induced apoptosis inhibitor 1) (CUA001). Splice isoform 2.	AK102124	B8ARI7 (UniProt)	Cytokine-induced anti-apoptosis inhibitor 1
Os04g0674425	Non-protein coding transcript.	BT086960	NONE	-
Os04g0674450	Similar to DHHC zinc finger domain containing protein.	AK376242	NP_001147996.1 (RefSeq)	Zinc finger, DHHC-type, palmitoyltransferase
Os04g0674500	Hypothetical protein.	AK120940	longestORF	-
Os04g0674600	Similar to H0103C06.1 protein.	AK069645	Q259I1 (UniProt)	Oligopeptide transporter OPT superfamily
Os04g0674700	Similar to AMP-binding protein (Adenosine monophosphate binding protein 5 AMPBP5).	AK106615	Q259I0 (UniProt)	AMP-dependent synthetase/ligase
Os04g0674750	Hypothetical protein.	EU941562	longestORF	-
Os04g0674800	Similar to CEL1%3DCELLULASE 1 (Fragment).	AK119913	Q0J930 (UniProt)	Glycoside hydrolase, family 9
Os04g0674900	Hypothetical protein.	tplb0042g22 (Wheat FLcDNA)	longestORF	-
Os04g0675000	Non-protein coding transcript.	AK063870	NONE	-
Os04g0675101	ATPase-like, ATP-binding domain domain containing protein.	BT066996	B9FDA1 (UniProt)	ATPase-like, ATP-binding domain
Os04g0675200	Similar to H0103C06.6 protein.	AK121874	Q259H3 (UniProt)	-
Os04g0675300	Similar to H0103C06.6 protein.	AK111982	Q259H3 (UniProt)	Zinc finger, RING-type
Os04g0675400	Similar to Chaperone protein dnaJ.	AK068186	Q259H2 (UniProt)	Heat shock protein DnaJ, N-terminal
Os04g0675500	Similar to Itm1 protein.	AK099138	Q259H1 (UniProt)	Oligosaccharyl transferase, STT3 subunit
Os04g0675600	Hypothetical conserved gene.	AK242041	NP_001054249.1 (RefSeq)	Pentatricopeptide repeat
Os04g0675700	Hypothetical conserved gene.	ab initio prediction	D0ABD8 (UniProt)	FBD
Os04g0675800	Similar to H0103C06.10 protein.	ab initio prediction	Q259H7 (UniProt)	F-box domain, cyclin-like
Os04g0676000	Non-protein coding transcript.	CT836511	NONE"	-
Os04g0676100	Similar to Thioredoxin X, chloroplast precursor.	AK288094	Q259H6 (UniProt)	Thioredoxin
Os04g0676200	Pentatricopeptide repeat domain containing protein.	AK106412	Q7XKC9 (UniProt)	Pentatricopeptide repeat
Os04g0676300	Similar to H0101F08.3 protein.	AK287813	Q259H4 (UniProt)	Dihydroorotate dehydrogenase, conserved site
Os04g0687800	Protein of unknown function DUF6, transmembrane domain containing protein.	AK107360	Q259K9 (UniProt)	Drug/metabolite transporter
Os04g0687900	Similar to T24D18.17 protein (Tubby-like protein TULP8).	AK104333	Q9ST78 (UniProt)	Tubby, C-terminal
Os04g0688000	Similar to CAA303718.1 protein.	CT835043	Q9ST79 (UniProt)	Protein BYPASS-related
Os04g0688100	Peroxidase (EC 1.11.1.7).	AK060789 ,AK104833	Q5U1N4 (UniProt)	Plant peroxidase
Os04g0688200	Similar to Peroxidase (EC 1.11.1.7).	AK103558	Q9ST81 (UniProt)	Plant peroxidase
Os04g0688300	Haem peroxidase, plant/fungal/bacterial family protein.	AK104928 ,AK065090	Q9ST82 (UniProt)	Plant peroxidase
Os04g0688500	Peroxidase (EC 1.11.1.7).	AK067667	Q5U1N1 (UniProt)	Plant peroxidase
Os04g0688600	Peroxidase (EC 1.11.1.7).	AK070680	Q5U1N0 (UniProt)	Plant peroxidase
Os04g0689000	Similar to Peroxidase (EC 1.11.1.7).	AK073360	Q00RN3 (UniProt)	Plant peroxidase
Os04g0689300	PLC-like phosphodiesterase, TIM beta/alpha-barrel domain domain containing protein.	AK100293	Q00RM9 (UniProt)	Phospholipase C, phosphatidylinositol-specific , X domain
Os04g0689400	Protein kinase, core domain containing protein.	AK102046	Q00RM8 (UniProt)	Protein kinase, catalytic domain
Os04g0689500	Similar to H0814G11.12 protein.	AK060983	Q00RM8 (UniProt)	Late embryogenesis abundant protein, LEA-14
Os04g0690100	Similar to H0814G11.16 protein.	AK105834	Q00RM4 (UniProt)	Zinc finger, C2H2
Os04g0690300	Similar to H0814G11.17 protein.	AK289176	Q00RM3 (UniProt)	Tetratricopeptide TPR-1
Os04g0690400	Similar to H0814G11.18 protein.	AK102981 ,AK103240	Q00RM2 (UniProt)	-
Os04g0690451	Hypothetical gene.	CT836480	longestORF	-
Os04g0690500	Similar to CAA30371.1 protein (Fragment).	AK105853	Q9ST96 (UniProt)	-
Os04g0690600	Similar to Auxin response factor 13.	AK109449	Q7XSS9 (UniProt)	Transcriptional factor B3
Os04g0690800	22 kDa protein of photosystem II.	AK071638	Q40716 (UniProt)	Chlorophyll A-B binding protein
Os04g0690900	Similar to H0323C08.15 protein.	AK241685	Q25A34 (UniProt)	-
Os04g0690932	Hypothetical gene.	AK062616	longestORF	-
Os04g0691000	Conserved hypothetical protein.	AK063241	NP_001043476.1 (RefSeq)	-
Os04g0691100	Serine/threonine-protein kinase SAPK5 (EC 2.7.1.37) (Osmotic stress/abscisic acid-activated protein kinase 5).	AK100269	Q7XKA8 (UniProt)	Protein kinase, catalytic domain
Os04g0691200	Similar to predicted protein.	AK369781	XP_002869342.1 (RefSeq)	WD40 repeat
Os04g0691300	Similar to ANAC030.	AK334647	NP_001148332.1 (RefSeq)	No apical meristem
Os04g0691366	Similar to POT family protein.	ab initio prediction	B6SXJ7 (UniProt)	-
Os04g0691400	Similar to POT family protein.	ab initio prediction	B6SXJ7 (UniProt)	Oligopeptide transporter
Os04g0691433	Non-protein coding transcript.	EU946594	NONE	-
Os04g0691466	Hypothetical gene.	EU949821	longestORF	-
Os04g0691500	Similar to TRN1 (TRANSPORTIN 1)%3B protein transporter.	AK100077	NP_001031359.1 (RefSeq)	HEAT
Os04g0691600	Similar to 30S ribosomal protein S17.	AK099298	Q9ZST1 (UniProt)	Ribosomal protein S17
Os04g0691700	GCN5-related N-acetyltransferase (GNAT) domain domain containing protein.	AK106305	Q7FAP7 (UniProt)	GCN5-related N-acetyltransferase
Os04g0691750	Similar to phosphoribosylanthranilate transferase.	tplb0048i05 (Wheat FLcDNA)	NP_001152458.1 (RefSeq)	-
Os04g0691800	C2 domain containing protein.	AK318532	NP_001152458.1 (RefSeq)	C2 calcium-dependent membrane targeting
Os04g0691900	Chaperonin Cpn60/TCP-1 family protein.	AK068257	Q7XKA1 (UniProt)	Chaperonin Cpn60/TCP-1
Os04g0692000	Protein of unknown function DUF6, transmembrane domain containing protein.	AK103611 ,AK061991	B4G1R1 (UniProt)	-
Os04g0692100	Similar to Tubulin folding cofactor B.	AK102150	A5CFZ4 (UniProt)	Cytoskeleton-associated protein, Gly-rich domain
Os04g0692200	Non-protein coding transcript.	AK059342	NONE	-
Os04g0692300	Peptidase C14, ICE, catalytic subunit p20, active site domain containing protein.	AK073368	XP_002871357.1 (RefSeq)	Zinc finger, RING/FYVE/PHD-type
Os04g0692400	Similar to ankyrin-like protein.	AK070717	NP_001151565.1 (RefSeq)	Protein of unknown function DUF248, methyltransferase putative
Os04g0692500	Similar to antiporter/ drug transporter/ transporter.	AK101528	NP_001147555.1 (RefSeq)	Ribonuclease Zc3h12a-like
Os04g0692600	Hypothetical gene.	AK318609	longestORF	-
Os04g0692700	Similar to CHR24 (chromatin remodeling 24)%3B ATP binding / DNA binding / helicase.	BT087024	NP_201200.2 (RefSeq)	Helicase, C-terminal

Os04g0692725	Hypothetical gene.	AK242828	longestORF	-
Os04g0692750	Hypothetical conserved gene.	BT083703	XP_002309928.1 (RefSeq)	-
Os05g0149300	Similar to 1-aminocyclopropane-1-carboxylate oxidase.	BT087584	O81606 (UniProt)	Oxoglutarate/iron-dependent oxygenase
Os05g0149400	Similar to 1-aminocyclopropane-1-carboxylic acid oxidase.	AK061064	O81606 (UniProt)	Oxoglutarate/iron-dependent oxygenase
Os05g0149450	Non-protein coding transcript.	tplb0008n13 (Wheat FLcDNA)	NONE	-
Os05g0149500	Similar to lipopolysaccharide-modifying protein.	AK064684	NP_001151574.1 (RefSeq)	Lipopolysaccharide-modifying protein
Os05g0149600	Similar to predicted protein.	AK061409	C7SJ62 (UniProt)	Cullin, N-terminal
Os05g0149701	Conserved hypothetical protein.	BT086859	C4J7R2 (UniProt)	-
Os05g0149800	Similar to Discordia 1.	AK058366	B7UBT1 (UniProt)	EF-hand-like domain
Os05g0149850	Non-protein coding transcript.	BT016490	NONE	-
Os05g0149900	Similar to tetratricopeptide repeat-containing protein.	AK119376	XP_002881630.1 (RefSeq)	Tetratricopeptide-like helical
Os05g0149950	Monothiol glutaredoxin-S8.	P0C290 (UniProt)	P0C290 (UniProt)	Glutaredoxin
Os05g0150000	Proline synthetase co-transcribed bacterial homolog protein.	AK289260	XP_002889904.1 (RefSeq)	Alanine racemase, N-terminal
Os05g0150100	Conserved hypothetical protein.	AK071562	B9FHD8 (UniProt)	-
Os05g0150300	Similar to Possible global transcription activator SNF2L1 (SWI/SNF related matrix associated actin dependent regulator of chromatin subfamily A member 1).	AK100732	XP_002315568.1 (RefSeq)	SNF2-related
Os05g0150400	Double-stranded RNA-binding domain containing protein.	AK110983	B9FMH4 (UniProt)	Double-stranded RNA-binding
Os05g0150500	Similar to Transport inhibitor response 1.	EU400583	D0ES26 (UniProt)	Leucine-rich repeat, cysteine-containing subtype
Os05g0150550	Hypothetical protein.	tplb0032b19 (Wheat FLcDNA)	longestORF	-
Os05g0150600	DNA helicase, ATP-dependent, RecQ type domain containing protein.	AK072977	D7U9T8 (UniProt)	Helicase, C-terminal
Os05g0150733	Similar to pro-resilin.	ab initio prediction	NP_001148515.1 (RefSeq)	-
Os05g0150800	Similar to C-1-tetrahydrofolate synthase, cytoplasmic.	AK104502 ,AK060882	B6T3G6 (UniProt)	Tetrahydrofolate dehydrogenase/cyclohydrolase, catalytic domain
Os05g0150900	Similar to Histidyl-tRNA synthetase (Fragment).	AK059520	Q9ZTW6 (UniProt)	Aminoacyl-tRNA synthetase, class II
Os05g0151000	Similar to DNA-directed RNA polymerase.	AK371102	C5YGY9 (UniProt)	RNA polymerase II, heptapeptide repeat, eukaryotic
Os05g0328800	Prolamin 7.	X53857	Q5EFA4 (UniProt)	Bifunctional trypsin/alpha-amylase inhibitor
Os05g0328901	Similar to Prolamin.	AK242260 (DDBJ, Secondary hit)	Q5EFA4 (UniProt)	-
Os05g0329001	Similar to Prolamin.	AF042201 (DDBJ, Secondary hit)	Q5EFA4 (UniProt)	-
Os05g0329100	Prolamin.	AK242910	Q5EFA4 (UniProt)	Bifunctional trypsin/alpha-amylase inhibitor
Os05g0329200	Similar to Prolamin.	AK242260 (DDBJ, Secondary hit)	Q5EFA4 (UniProt)	Bifunctional trypsin/alpha-amylase inhibitor
Os05g0329300	Prolamin.	AK242910	Q5EFA4 (UniProt)	Bifunctional trypsin/alpha-amylase inhibitor
Os05g0329350	Prolamin.	EF122440	Q5EFA4 (UniProt)	Bifunctional trypsin/alpha-amylase inhibitor
Os05g0329400	Similar to Prolamin.	AK242260 (DDBJ, Secondary hit)	Q5EFA4 (UniProt)	-
Os05g0329700	Similar to Prolamin.	AK242910	Q5EFA4 (UniProt)	Bifunctional trypsin/alpha-amylase inhibitor
Os05g0330150	Similar to Prolamin.	AK242260 (DDBJ, Secondary hit)	Q5EFA4 (UniProt)	Bifunctional trypsin/alpha-amylase inhibitor
Os06g0137400	Hypothetical conserved gene.	AK375274	XP_002310856.1 (RefSeq)	Auxin responsive SAUR protein
Os06g0137500	Brix domain containing protein.	AK072896	NP_567213.1 (RefSeq)	Anticodon-binding
Os06g0137600	K homology-like, alpha/beta domain containing protein.	AK072214 ,AK059782	B6TDG2 (UniProt)	Ribosome-binding factor A
Os06g0137650	UDP-glucuronosyl/UDP-glucosyltransferase domain containing protein.	AK373894	NP_001173311.1 (RefSeq)	UDP-glucuronosyl/UDP-glucosyltransferase
Os06g0137700	Similar to GDP-mannose 4,6 dehydratase 1 (EC 4.2.1.47) (GDP-D-mannose dehydratase 1) (GMD 1).	AK106455	NP_001149057.1 (RefSeq)	NAD-dependent epimerase/dehydratase
Os06g0138000	Similar to IMB1.	AK063880	B6UCC7 (UniProt)	-
Os06g0138100	Methionine sulphoxide reductase A domain containing protein.	AK069328	Q5VPG8 (UniProt)	Peptide methionine sulphoxide reductase MsrA
Os06g0138200	Similar to Oxidoreductase.	AK058914	A1YKE1 (UniProt)	-
Os06g0138400	Hypothetical conserved gene.	ab initio prediction	Q5VPG5 (UniProt)	Ribonuclease CAF1
Os06g0138600	Protein of unknown function DUF248, methyltransferase putative domain containing protein.	AK069536	A2Y920 (UniProt)	Protein of unknown function DUF248, methyltransferase putative
Os06g0138700	Cyclin-like F-box domain containing protein.	AK119470	Q01K44 (UniProt)	F-box domain, cyclin-like
Os06g0138900	Conserved hypothetical protein.	AK105368	A2Y922 (UniProt)	-
Os06g0139000	Conserved hypothetical protein.	AK073027	B9EZS4 (UniProt)	-
Os06g0139150	Conserved hypothetical protein.	AK105368	A2Y922 (UniProt)	-
Os06g0139200	Conserved hypothetical protein.	AK063851	B9FJK4 (UniProt)	-
Os06g0139400	Conserved hypothetical protein.	AK110768	Q5VQG5 (UniProt)	-
Os06g0139700	Protein of unknown function DUF2052, coiled-coil domain containing protein.	AK106220 ,AK067884 ,AK10324[illegible]	A2Y924 (UniProt)	Protein of unknown function DUF2052, coiled-coil
Os06g0139800	Similar to Acid phosphatase (EC 3.1.3.2) 1 allozyme 1.	AK058604	NP_001150931.1 (RefSeq)	Acid phosphatase
Os06g0139900	Similar to Beta 1 subunit of 20S proteasome.	AK104152	A2Y926 (UniProt)	Peptidase T1A, proteasome beta-subunit
Os06g0140200	Leucine-rich repeat, plant specific containing protein.	AK287701 ,AK241776 ,AK28787[illegible]	Q5VPE8 (UniProt)	Leucine-rich repeat
Os06g0140300	Leucine-rich repeat, N-terminal domain containing protein.	AK100368	B8B2B4 (UniProt)	Leucine-rich repeat
Os06g0140400	Similar to HAHB-6 (Fragment).	AK059116	Q5VPE5 (UniProt)	Homeobox
Os06g0140700	Similar to Homeodomain leucine zipper protein (Fragment).	AK105150	Q5VPE3 (UniProt)	Helix-turn-helix motif, lambda-like repressor
Os06g0140800	Similar to Serine threonine kinase.	AK105657	A1YKF2 (UniProt)	Protein kinase, catalytic domain
Os06g0140900	Sigma factor, regions 3 and 4 domain containing protein.	AK058823	B8B2B8 (UniProt)	-
Os06g0140950	Non-protein coding transcript.	AK242656	NONE	-
Os06g0141100	Monosaccharide transporter.	ab initio prediction	C0JSA7 (UniProt)	Sugar/inositol transporter
Os06g0141133	Hypothetical protein.	tplb0053a05 (Wheat FLcDNA)	longestORF	-
Os06g0141166	Similar to hydrolase, NUDIX family protein.	ab initio prediction	NP_001149422.1 (RefSeq)	NUDIX hydrolase domain
Os06g0141200	Similar to RNA-binding protein EWS.	AK061234 ,AK099058	Q9SW92 (UniProt)	Zinc finger, RanBP2-type
Os06g0141400	Similar to Early nodulin.	AK121184	Q9XFD2 (UniProt)	Early nodulin 93 ENOD93 protein
Os06g0141600	Early nodulin 93 ENOD93 protein family protein.	AK059772	O82787 (UniProt)	Early nodulin 93 ENOD93 protein
Os06g0141700	Similar to Early nodulin.	AK121791	O82787 (UniProt)	Early nodulin 93 ENOD93 protein
Os06g0141800	Similar to fasciclin-like arabinogalactan protein 8.	AK107277	NP_001150349.1 (RefSeq)	-
Os06g0141950	Similar to MYB transcription factor.	AJ601380	B4FX89 (UniProt)	Homeodomain-related
Os06g0142000	Hypothetical conserved gene.	CT836263	NP_001056764.1 (RefSeq)	Peptidase cysteine/serine, trypsin-like
Os06g0142050	Similar to H0913C04.9 protein.	BT035075	NP_565878.1 (RefSeq)	Ribosomal biogenesis regulatory protein
Os06g0142100	Cyclin-like F-box domain containing protein.	ab initio prediction	NP_001056765.1 (RefSeq)	-
Os06g0142200	Similar to cDNA clone:J033147G13, full insert sequence.	AK105832	B6UHN2 (UniProt)	Early nodulin 93 ENOD93 protein
Os06g0142300	Early nodulin 93 ENOD93 protein family protein.	CT837557	O82787 (UniProt)	Early nodulin 93 ENOD93 protein
Os06g0142350	Similar to Early nodulin.	AK242220	O82787 (UniProt)	Early nodulin 93 ENOD93 protein
Os06g0142400	Early nodulin.	AK241230	O82787 (UniProt)	Early nodulin 93 ENOD93 protein
Os06g0142500	Similar to Wall-associated kinase 3.	AK252642	Q4U3Z6 (UniProt)	EGF-type aspartate/asparagine hydroxylation site
Os06g0142550	Non-protein coding transcript.	CT828398	NONE"	-
Os06g0142600	Similar to ELF3 protein.	AK242105	A7YJG7 (UniProt)	-
Os06g0142625	Hypothetical protein.	BT039571	longestORF	-
Os06g0142650	Similar to Avr9/Cf-9 rapidly elicited protein 11 (Fragment).	ab initio prediction	NP_001174594.1 (RefSeq)	Protein kinase, catalytic domain
Os06g0142700	Cytochrome c oxidase, subunit Vb family protein.	AK071423	P92683 (UniProt)	Cytochrome c oxidase, subunit Vb
Os06g0142800	Ribosomal L11 methyltransferase domain containing protein.	AK073269 ,AK103971	NP_563720.1 (RefSeq)	Skb1 methyltransferase
Os06g0142900	Similar to predicted protein.	AK061829	F2WM28 (UniProt)	Transketolase-like, pyrimidine-binding domain
Os06g0143000	Iron-superoxide dismutase (EC 1.15.1.1).	AK062073 ,AK071301	B8B2C9 (UniProt)	Manganese/iron superoxide dismutase
Os06g0143100	Similar to Cadmium tolerant 1.	AK121057	B5BSU1 (UniProt)	-
Os06g0143400	Similar to Acyl-ACP thioesterase (Fragment).	AK072729 ,AK120946	A1YKG2 (UniProt)	Acyl-ACP thioesterase
Os06g0143500	Pentatricopeptide repeat domain containing protein.	ab initio prediction	Q9SNP3 (UniProt)	Pentatricopeptide repeat
Os06g0143600	Similar to PRP38 pre-mRNA processing factor 38 domain containing B.	AK061914	B6TSE1 (UniProt)	-
Os06g0143650	Non-protein coding transcript.	tplb0040d24 (Wheat FLcDNA)	NONE	-
Os06g0513943	Conserved hypothetical protein.	CT833375 (DDBJ, Secondary hit)	B8AJ30 (UniProt)"	-
Os06g0514100	Conserved hypothetical protein.	CT833375 (DDBJ, Secondary hit)	B8AJ30 (UniProt)"	-
Os06g0606900	Conserved hypothetical protein.	AK068134	B9FU26 (UniProt)	-
Os06g0607000	Similar to Beta-1,3-glucanase.	AK121115	A4PID2 (UniProt)	Glycoside hydrolase, family 17
Os06g0607100	Similar to phosphatidic acid phosphatase-related / PAP2-related.	AK071250	NP_566527.1 (RefSeq)	-
Os06g0607200	Similar to Cellular retinaldehyde binding/alpha-tocopherol transport%3B Cellular retinaldehyde-binding/triple function, N-terminal.	AK242958	Q2HV32 (UniProt)	Cellular retinaldehyde-binding/triple function, N-terminal
Os06g0607700	ABC transporter-like domain containing protein.	AK106792	C0J9X4 (UniProt)	ABC transporter-like
Os06g0607750	Hypothetical protein.	BT084685	longestORF	-
Os06g0607800	Similar to 26S proteasome regulatory complex subunit p42D.	AK063158 ,AK066695	C0J9X5 (UniProt)	ATPase, AAA+ type, core
Os06g0607850	Hypothetical protein.	tplb0035c03 (Wheat FLcDNA)	longestORF	-
Os06g0607900	GRAM domain containing protein.	AK070879	C0J9X6 (UniProt)	GRAM
Os06g0608000	Similar to PGPS/D10.	AK119328	A3BDF7 (UniProt)	-
Os06g0608050	Hypothetical gene.	AK241742	longestORF	-
Os06g0608100	Hypothetical conserved gene.	tplb0012d16 (Wheat FLcDNA)	NP_001058037.2 (RefSeq)	-
Os06g0635700	Conserved hypothetical protein.	AK107643	A3BDV0 (UniProt)	-
Os06g0636100	Hypothetical conserved gene.	CT836245	Q67V17 (UniProt)	Pathogenic type III effector avirulence factor Avr cleavage site
Os06g0636201	Conserved hypothetical protein.	EU968448	NP_001144438.1 (RefSeq)	-
Os06g0636600	Protein kinase, core domain containing protein.	AK119586	NP_001147925.1 (RefSeq)	Protein kinase, catalytic domain
Os06g0636700	Esterase, SGNH hydrolase-type domain containing protein.	AK058562	NP_849451.1 (RefSeq)	Lipase, GDSL
Os06g0645400	Similar to ATP binding protein (Fragment).	AK105391	A6N078 (UniProt)	-
Os06g0645500	Sterile alpha motif homology domain containing protein.	AK109234	A2YFK5 (UniProt)	Sterile alpha motif/pointed domain
Os06g0645600	Hypothetical conserved gene.	BT085127	NP_001058193.1 (RefSeq)	-
Os06g0645650	Hypothetical protein.	tplb0048p04 (Wheat FLcDNA)	longestORF	-
Os06g0645700	Similar to HAF1.	AK108404	Q67W65 (UniProt)	Bromodomain
Os06g0645800	Similar to Transcription initiation factor TFIID subunit 1.	AK064421	Q67W65 (UniProt)	Zinc finger, CCHC-type

Os06g0645901	Similar to Transcription initiation factor TFIID subunit 1.	BT037416	Q67W65 (UniProt)	-
Os06g0646000	Methyltransferase type 11 domain containing protein.	AK104339	B6SRT6 (UniProt)	Methyltransferase type 11
Os06g0646100	Conserved hypothetical protein.	AK062869	A2YFL1 (UniProt)	-
Os06g0646400	Tyrosine protein kinase domain containing protein.	AK243413	B9FQ73 (UniProt)	Phox/Bem1p
Os06g0646500	Similar to ATP synthase delta chain, mitochondrial precursor (EC 3.6.3.14) (Oligomycin sensitivity conferral protein) (OSCP).	AK071267	B6TBW2 (UniProt)	ATPase, F1 complex, OSCP/delta subunit
Os06g0646600	Similar to cDNA, clone: J065162G03, full insert sequence.	AK289177	B7F8T4 (UniProt)	Homeobox
Os06g0646700	Conserved hypothetical protein.	AK065143	B9FQ75 (UniProt)	-
Os06g0646801	Hypothetical conserved gene.	AK287534	B9FQ76 (UniProt)	-
Os06g0646900	Similar to Homogentisic acid geranylgeranyl transferase.	AK063383	Q7XB12 (UniProt)	-
Os06g0647000	Hypothetical protein.	ab initio prediction	NONE	-
Os06g0647100	Similar to 50S ribosomal protein L35, chloroplast precursor [illegible]	AK066837	A2YFL7 (UniProt)	Ribosomal protein L35, non-mitochondrial
Os06g0676500	Hypothetical protein.	AK069470	longestORF	-
Os06g0676600	Protein kinase, core domain containing protein.	AK103523	NP_001148145.1 (RefSeq)	Protein kinase, catalytic domain
Os06g0676700	Similar to High pI alpha-glucosidase.	AK063175	Q653V3 (UniProt)	-
Os06g0676801	Hypothetical gene.	BT019289	longestORF	-
Os06g0676900	Non-protein coding transcript.	AK111124	NONE	-
Os06g0677000	Hypothetical conserved gene.	AK357609	NP_001058354.2 (RefSeq)	Patatin/Phospholipase A2-related
Os06g0677300	Zinc finger, RING/FYVE/PHD-type domain containing protein.	AK100432	B9FQH3 (UniProt)	Zinc finger, RING-CH-type
Os06g0677400	Similar to 3-hydroxyisobutyrate dehydrogenase.	AK073050	B6SHU3 (UniProt)	3-hydroxyisobutyrate dehydrogenase-related, conserved site
Os06g0677500	Protein prenyltransferase domain containing protein.	AK102430	NP_001151503.1 (RefSeq)	Leucine-rich repeat
Os06g0677600	Like-Sm ribonucleoprotein, core family protein.	AK063252	NP_001147152.1 (RefSeq)	Like-Sm ribonucleoprotein
Os06g0677700	YT521-B-like protein family protein.	tplb0017e12 (Wheat FLcDNA)	NP_174334.2 (RefSeq)	Zinc finger, CCCH-type
Os06g0677800	Similar to P-167-1_1 (Fragment).	AK103280	Q653U3 (UniProt)	AUX/IAA protein
Os06g0678100	Conserved hypothetical protein.	EU975100	NP_001058361.1 (RefSeq)	-
Os06g0678200	Similar to Geranyl diphosphate synthase.	AK071299 ,AK059456	B6TA22 (UniProt)	Polyprenyl synthetase
Os06g0678500	Hypothetical conserved gene.	ab initio prediction	NP_001058363.2 (RefSeq)	-
Os06g0678650	WD40 repeat-like domain containing protein.	AK241346	NP_564728.2 (RefSeq)	WD40 repeat
Os06g0678651	Similar to WD-40 repeat family protein / beige-related.	EU954809	NP_564728.2 (RefSeq)	BEACH domain
Os06g0678700	Hypothetical conserved gene.	AK101184	B9FQH6 (UniProt)	-
Os06g0678750	Non-protein coding transcript.	EU943604	NONE	-
Os06g0678800	Similar to Pollen-specific protein NTP303 precursor.	AK071465	B6ST56 (UniProt)	Multicopper oxidase, type 1
Os06g0678875	Non-protein coding transcript.	tplb0021n10 (Wheat FLcDNA)	NONE	-
Os06g0698600	Exo70 exocyst complex subunit family protein.	AK243691	NP_194882.2 (RefSeq)	Exo70 exocyst complex subunit
Os06g0698674	eIF4-gamma/eIF5/eIF2-epsilon domain containing protein.	AK243382	B6T7C2 (UniProt)	Translation initiation factor IF2/IF5
Os06g0698686	Hypothetical protein.	tplb0060f03 (Wheat FLcDNA)	longestORF	-
Os06g0698711	Conserved hypothetical protein.	AK070810	B8B238 (UniProt)	-
Os06g0698748	Similar to MTD1.	AK100361	NP_001148854.1 (RefSeq)	-
Os06g0698785	Similar to Choline monooxygenase.	AK120445	Q7XB43 (UniProt)	Rieske [2Fe-2S] iron-sulphur domain
Os06g0698802	Hypothetical conserved gene.	AK288331	B9FQP9 (UniProt)	-
Os06g0698812	Disease resistance protein domain containing protein.	ab initio prediction	XP_002438943.1 (RefSeq)	Disease resistance protein
Os06g0698822	Hypothetical protein.	AK241532	longestORF	-
Os06g0698859	Similar to C2H2 zinc-finger protein SERRATE (Fragment).	AK120947	Q94FY3 (UniProt)	Arsenite-resistance protein 2
Os06g0704000	Protein of unknown function DUF659 domain containing protein.	AK073579	B8B263 (UniProt)	Domain of unknown function DUF659
Os06g0704100	Protein of unknown function DUF547 domain containing protein.	AK103758	E4MVN4 (UniProt)	Domain of unknown function DUF547
Os06g0704200	Conserved hypothetical protein.	AK241846	Q5Z808 (UniProt)	-
Os06g0704300	Zinc finger, CCCH-type domain containing protein.	AK107008	NP_001147895.1 (RefSeq)	Zinc finger, CCCH-type
Os06g0704400	Similar to cDNA clone:001-132-E04, full insert sequence.	AK062169	B7F4U2 (UniProt)	Zinc finger, PMZ-type
Os06g0704450	Hypothetical protein.	BT068200	longestORF	-
Os06g0704500	Leucine-rich repeat, plant specific containing protein.	AK105531	Q5Z8W0 (UniProt)	Leucine-rich repeat
Os06g0704600	Similar to Delta-aminolevulinic acid dehydratase (Fragment).	AK065784 ,AK119551	Q5Z8V9 (UniProt)	Tetrapyrrole biosynthesis, porphobilinogen synthase
Os06g0704700	NAD(P)-binding domain containing protein.	AK120907	NP_173116.1 (RefSeq)	NmrA-like
Os06g0704800	Probable histone acetyltransferase HAC-like 2.	Q5Z8V7 (UniProt)	Q5Z8V7 (UniProt)	Zinc finger, TAZ-type
Os06g0704900	Similar to Cell division-like protein.	AK103054	NP_001152207.1 (RefSeq)	Ribosomal RNA methyltransferase RrmJ/FtsJ
Os06g0705000	Similar to predicted protein.	AK242204	XP_002967921.1 (RefSeq)	EXTL2, alpha-1,4-N-acetylhexosaminyltransferase
Os06g0705100	Similar to Thylakoid lumenal 13.3 kDa protein (P16.5) [illegible]	AK060969	B6SU36 (UniProt)	-
Os06g0705200	Hypothetical conserved gene.	EU953189	Q5Z8V2 (UniProt)	EGF-type aspartate/asparagine hydroxylation site
Os06g0705250	Hypothetical gene.	EU955465	longestORF	-
Os06g0705300	Similar to plant-specific domain TIGR01589 family protein.	AK064803	NP_001149236.1 (RefSeq)	Conserved hypothetical protein CHP01589, plant
Os06g0705350	Similar to pentatricopeptide (PPR) repeat-containing protein.	BT055332	NP_199702.1 (RefSeq)	Pentatricopeptide repeat
Os06g0705400	Similar to Nonspecific lipid-transfer protein 2P (LTP2P) (Lipid transfer protein 2 isoform 2) (LTP2-2) (7 kDa lipid transfer [illegible]	AK062667	NP_001149680.1 (RefSeq)	Plant lipid transfer protein/seed storage/trypsin-alpha amylase inhibitor
Os06g0705500	Ataxin-2, C-terminal domain containing protein.	AK060142	B8B276 (UniProt)	Ubiquitin system component Cue
Os06g0705600	Oligopeptide transporter domain containing protein.	ab initio prediction	NP_001058518.2 (RefSeq)	Oligopeptide transporter
Os06g0705651	Hypothetical protein.	EU955776	longestORF	-
Os06g0705700	TGF-beta receptor, type I/II extracellular region family protein.	AK119527	A2YGT5 (UniProt)	Oligopeptide transporter
Os06g0705901	Hypothetical protein.	tplb0058e01 (Wheat FLcDNA)	longestORF	-
Os06g0706100	Similar to Peptide transporter PTR2.	AK334364	B6TVK5 (UniProt)	Oligopeptide transporter
Os06g0706700	Similar to PsAD1.	EU965944	Q9LRI5 (UniProt)	-
Os06g0706850	Similar to Glycosyltransferase.	tplb0046e07 (Wheat FLcDNA)	B6UFI8 (UniProt)	-
Os06g0707000	Glycosyltransferase AER61, uncharacterised domain containing protein.	ab initio prediction	Q5Z8T8 (UniProt)	Glycosyltransferase AER61, uncharacterised
Os06g0707100	Multi antimicrobial extrusion protein MatE family protein.	AK066647	NP_200058.1 (RefSeq)	Multi antimicrobial extrusion protein
Os06g0707200	Glycosyltransferase AER61, uncharacterized domain containing protein.	AK059411 ,AK071568	NP_001151219.1 (RefSeq)	Glycosyltransferase AER61, uncharacterised
Os06g0707250	Hypothetical genes.	ab initio prediction	NONE	-
Os06g0707300	Cyclin-like F-box domain containing protein.	AK101481 ,AK063291	A2YGU4 (UniProt)	-
Os06g0707350	Similar to NBS-LRR disease resistance protein homologue.	AK355483	Q84KC8 (UniProt)	-
Os06g0707400	Similar to NBS-LRR disease resistance protein homologue.	AK067727	Q84KC2 (UniProt)	-
Os06g0707700	NB-ARC domain containing protein.	AK100720	Q84KC8 (UniProt)	Disease resistance protein
Os06g0707733	Disease resistance protein domain containing protein.	ab initio prediction	A2YGU6 (UniProt)	Disease resistance protein
Os06g0707800	Similar to OSIGBa0148A10.13 protein.	AK102129	Q5Z9J2 (UniProt)	Disease resistance protein
Os06g0708000	Similar to Isoform 2 of Mitogen-activated protein kinase 12.	AK070823	Q5Z9J0-2 (UniProt)	-
Os06g0708050	Hypothetical gene.	EU949346	longestORF	-
Os06g0708075	Non-protein coding transcript.	tplb0059e09 (Wheat FLcDNA)	NONE	-
Os06g0708100	Similar to Carboxylesterase-like protein.	AK066901	A2YGU8 (UniProt)	-
Os06g0708200	Hypothetical conserved gene.	AK068246	B9FQU2 (UniProt)	-
Os06g0708300	Similar to RER1A protein.	AK243324	NP_001151498.1 (RefSeq)	Retrieval of early ER protein Rer1
Os06g0708400	Cyclophilin.	ab initio prediction	A2YGV1 (UniProt)	Peptidyl-prolyl cis-trans isomerase, cyclophilin-type
Os06g0708500	Similar to Peptidyl-prolyl cis-trans isomerase.	AK072490	A2YGV2 (UniProt)	Peptidyl-prolyl cis-trans isomerase, cyclophilin-type
Os06g0708600	Zinc finger, C2H2-like domain containing protein.	AK100915	E4MXX2 (UniProt)	Zinc finger, C2H2-like
Os06g0708700	Similar to nodulin-like protein.	AK071974	B6SKJ2 (UniProt)	Drug/metabolite transporter
Os06g0708832	Similar to arogenate dehydrogenase.	AK355541	NP_001147429.1 (RefSeq)	Prephenate dehydrogenase
Os06g0708900	Similar to zinc knuckle (CCHC-type) family protein.	AK100402	NP_193654.2 (RefSeq)	CBF1-interacting co-repressor CIR, N-terminal
Os06g0709000	NAD(P)-binding domain containing protein.	AK068653	NP_001147429.1 (RefSeq)	Prephenate dehydrogenase
Os06g0709100	Cyclin-like F-box domain containing protein.	AK070881	B6TRX2 (UniProt)	F-box domain, cyclin-like
Os06g0709400	Conserved hypothetical protein.	AK108588	A3CGK2 (UniProt)	-
Os06g0711700	Kelch-type beta propeller domain containing protein.	EU956453	NP_001058552.2 (RefSeq)	Galactose oxidase, beta-propeller
Os06g0711800	Pectinesterase inhibitor domain containing protein.	AK069642	NP_001167668.1 (RefSeq)	Pectinesterase inhibitor
Os06g0711900	Bifunctional inhibitor/plant lipid transfer protein/seed storage domain containing protein.	AK105838	A2YGX0 (UniProt)	Plant lipid transfer protein/seed storage/trypsin-alpha amylase inhibitor
Os06g0712200	NUDIX domain containing protein.	AK061313	A2YGX1 (UniProt)	NUDIX hydrolase domain
Os06g0712250	Hypothetical conserved gene.	CT832454	NP_001144189.1 (RefSeq)	-
Os06g0712300	Hypothetical conserved gene.	AK058857	B9FQV0 (UniProt)	EF-hand-like domain
Os06g0712400	Similar to predicted protein.	AK104913	B9FQV1 (UniProt)	Protein of unknown function DUF544
Os06g0712500	Similar to Glycosyltransferase QUASIMODO1 (EC 2.4.1.-).	AK068531	E6NU27 (UniProt)	Glycosyl transferase, family 8
Os06g0712550	Hypothetical protein.	tplb0056b02 (Wheat FLcDNA)	longestORF	-
Os06g0712600	Similar to SHI.	EU959412	NP_001150905.1 (RefSeq)	Zinc finger, lateral root primordium type 1
Os06g0712700	MADS-box protein SPW1.	AK069317	Q944S9 (UniProt)	Transcription factor, MADS-box
Os06g0712800	Similar to Ankyrin-like protein.	AK121236	NP_190676.1 (RefSeq)	Protein of unknown function DUF248, methyltransferase putative
Os06g0712900	tRNA-dihydrouridine synthase domain containing protein.	AK106648	NP_201523.1 (RefSeq)	tRNA-dihydrouridine synthase
Os06g0713000	Zinc finger, B-box domain containing protein.	AK105957	NP_001150747.1 (RefSeq)	Zinc finger, B-box
Os06g0713100	Protein of unknown function DUF1640 family protein.	AK072606	B6UC57 (UniProt)	Protein of unknown function DUF1640
Os06g0713201	Non-protein coding transcript.	BT087005	NONE	-
Os06g0713300	Conserved hypothetical protein.	AK106687	A3BFC6 (UniProt)	-
Os06g0713400	Cyclin-like F-box domain containing protein.	AK100628	B9FQV7 (UniProt)	F-box domain, cyclin-like
Os06g0713600	Non-protein coding transcript.	tplb0032e04 (Wheat FLcDNA)	NONE	-
Os06g0713800	Alpha-amylase isozyme 2A precursor (EC 3.2.1.1) (1,4-alpha-D-glucan glucanohydrolase).	AK059671 ,AK101018	Q0D9J1 (UniProt)	Glycoside hydrolase, family 13
Os06g0713900	Hypothetical conserved gene.	AK242112	A2YGY3 (UniProt)	-
Os06g0714000	Uncharacterised protein family UPF0183 domain containing [illegible]	AK069538	XP_002439019.1 (RefSeq)	Uncharacterised protein family UPF0183
Os06g0714100	Complex 1 LYR protein family protein.	AK121079	NP_001148390.1 (RefSeq)	-
Os06g0714200	Similar to calcium dependent protein kinase1.	AK243187	NP_001105740.1 (RefSeq)	Protein kinase, catalytic domain
Os06g0714300	Auxin responsive SAUR protein family protein.	AK107043	XP_002318465.1 (RefSeq)	Auxin responsive SAUR protein

Os06g0714366	Hypothetical protein.	EU977023	longestORF	-
Os06g0714400	Conserved hypothetical protein.	AK108610	Q5NAL1 (UniProt)	-
Os06g0714432	Non-protein coding transcript.	CT836310	NONE"	-
Os06g0714500	ATPase, AAA-type, core domain containing protein.	AK100047 ,AK119628	NP_001150200.1 (RefSeq)	ATPase, AAA+ type, core
Os06g0714600	ADP-ribosylation factor domain containing protein.	AK318603	A6MD16 (UniProt)	Small GTPase superfamily
Os06g0714700	Conserved hypothetical protein.	CU406678	NP_001058577.1 (RefSeq)	-
Os06g0714800	Protein of unknown function DUF581 family protein.	AK059793 ,AK071528	Q5Z9Q5 (UniProt)	Protein of unknown function DUF581
Os06g0714900	Serine/threonine protein kinase domain containing protein.	AK071975	NP_001147668.1 (RefSeq)	Protein kinase, catalytic domain
Os06g0714950	Hypothetical gene.	EU943065	longestORF	-
Os06g0714951	Hypothetical gene.	CT832209	longestORF	-
Os07g0125000	Allergen V5/Tpx-1 related family protein.	AK060005 ,AK104140	Q8LLU7 (UniProt)	-
Os07g0125201	Allergen V5/Tpx-1 related family protein.	DQ167191	O04000 (UniProt)	Allergen V5/Tpx-1-related
Os07g0125500	Allergen V5/Tpx-1 related family protein.	AK060057	D5KR57 (UniProt)	Allergen V5/Tpx-1-related
Os07g0125600	Allergen V5/Tpx-1 related family protein.	AK060005 ,AK104140	Q8LLU7 (UniProt)	-
Os07g0126100	Allergen V5/Tpx-1 related family protein.	DQ167191	O04000 (UniProt)	Allergen V5/Tpx-1-related
Os07g0126301	Allergen V5/Tpx-1 related family protein.	AK060057	D5KR57 (UniProt)	Allergen V5/Tpx-1-related
Os07g0126401	Allergen V5/Tpx-1 related family protein.	AK060005 ,AK104140	Q8LLU7 (UniProt)	-
Os07g0126500	Allergen V5/Tpx-1 related family protein.	DQ167191	O04000 (UniProt)	Allergen V5/Tpx-1-related
Os07g0155600	Nramp ion-transporter family protein, Ethylene signaling pathway	AY396568	Q6TJY0 (UniProt),Q0D8I9 (	-
Os07g0156200	Haem peroxidase, plant/fungal/bacterial family protein.	AK249509	NP_001152255.1 (RefSeq)	Plant peroxidase
Os07g0156467	Similar to Class III peroxidase 7.	ab initio prediction	Q5U1T6 (UniProt)	Plant peroxidase
Os07g0156732	Similar to EIN2.	AK111802	Q6TJY0 (UniProt)	Natural resistance-associated macrophage protein
Os07g0156910	Similar to peroxidase 1.	ab initio prediction	NP_001152255.1 (RefSeq)	Haem peroxidase, plant/fungal/bacterial
Os07g0157000	Similar to Class III peroxidase 7.	FP094284	Q5U1T6 (UniProt)	Plant peroxidase
Os07g0157401	Similar to EIN2.	AK111802	Q6TJY0 (UniProt)	Natural resistance-associated macrophage protein
Os07g0157600	Similar to peroxidase 1.	AK252026	NP_001152255.1 (RefSeq)	Plant peroxidase
Os07g0157700	Conserved hypothetical protein.	AK069862	B8B7D5 (UniProt)	-
Os07g0157900	Deoxyribonuclease, TatD domain containing protein.	AK067778	B6TDE6 (UniProt)	Deoxyribonuclease, TatD-related
Os07g0158000	Hypothetical conserved gene.	ab initio prediction	A3BGQ5 (UniProt)	-
Os07g0158100	Hypothetical gene.	AK108021	longestORF	-
Os07g0158200	Deoxyribonuclease, TatD Mg-dependent domain containing	AK103591 ,AK103532	B6TDE6 (UniProt)	Deoxyribonuclease, TatD-related
Os07g0158300	Similar to RNA binding protein.	AK067376 ,AK099188 ,AK10477	O81989 (UniProt)	RNA recognition motif domain
Os07g0158400	Hypothetical conserved gene.	EU966727	Q9LMF3 (UniProt)	GCK
Os07g0158500	Similar to HAP2 subunit of HAP complex.	FP101566	B0S4T5 (UniProt)	CCAAT-binding transcription factor, subunit B
Os07g0171100	Similar to predicted protein.	AK062132	XP_002891836.1 (RefSeq)	PDZ/DHR/GLGF
Os07g0171200	Similar to Galactose-1-phosphate uridyl transferase-like protein.	AK071075	B6TFN9 (UniProt)	Galactose-1-phosphate uridyl transferase, class I
Os07g0171300	Protein kinase, core domain containing protein.	AK100663 ,AK066357	NP_001152463.1 (RefSeq)	Protein kinase, catalytic domain
Os07g0171350	Non-protein coding transcript.	BT084747	NONE	-
Os07g0172200	Similar to ROOT HAIRLESS 1.	AK103352	B6UAF0 (UniProt)	-
Os07g0172500	Similar to DNA-directed RNA polymerase II 13.6 kDa polypeptide (EC 2.7.7.6).	AK068132 ,AK099622	B4FJS1 (UniProt)	DNA-directed RNA polymerase Rpb11, 13-16kDa subunit, conserved site
Os07g0172600	Pentatricopeptide repeat domain containing protein.	AK369145	NP_001058999.1 (RefSeq)	Pentatricopeptide repeat
Os07g0172900	Hypothetical conserved gene.	AK067304	Q6ZA56 (UniProt)	-
Os07g0173100	HSP20-like chaperone domain containing protein.	CT836094	NP_001059002.1 (RefSeq)	-
Os07g0173200	Frigida-like family protein.	AK061624	Q6ZA59 (UniProt)	Frigida-like
Os07g0173300	Conserved hypothetical protein.	AK110935	A3BH18 (UniProt)	-
Os07g0173400	Conserved hypothetical protein.	AK107434	Q6ZA58 (UniProt)	-
Os07g0173501	Similar to 40S ribosomal protein S18.	CT837772	NP_001151612.1 (RefSeq)	Ribosomal protein S13
Os07g0184900	Hypothetical conserved gene.	AK064466	B8B7W2 (UniProt)	-
Os07g0184950	Conserved hypothetical protein.	CT831736	Q6ZGF9 (UniProt)"	-
Os07g0185000	Similar to cDNA clone:J023135J22, full insert sequence.	AK060552	B7ER38 (UniProt)	-
Os07g0185100	Hypothetical protein.	AK119508	longestORF	-
Os07g0185200	Similar to predicted protein.	AK060743	NP_176653.1 (RefSeq)	Lipid-binding START
Os07g0185300	Protein of unknown function YGGT family protein.	AK071133	NP_194528.1 (RefSeq)	Uncharacterised protein family Ycf19
Os07g0185432	Similar to Seed specific protein Bn15D14A.	ab initio prediction	B6TVC9 (UniProt)	Xklp2 targeting protein
Os07g0185500	Non-protein coding transcript.	AK058768	NONE	-
Os07g0185700	Conserved hypothetical protein.	AK070572	B8B7W7 (UniProt)	-
Os07g0185800	Similar to C-Myc binding protein (Associate of Myc 1) (AMY-1)	AK063035	NP_001149560.1 (RefSeq)	-
Os07g0185900	Conserved hypothetical protein.	AK070315	A2YIW6 (UniProt)	-
Os07g0186000	Similar to Thioredoxin h isoform 1.	AK121423 ,AK059196	A2YIW7 (UniProt)	-
Os07g0564000	Conserved hypothetical protein.	AK069806	B8B7G5 (UniProt)	-
Os07g0564100	UDP-glucuronosyl/UDP-glucosyltransferase family protein.	AK107291	NP_001148465.1 (RefSeq)	UDP-glucuronosyl/UDP-glucosyltransferase
Os07g0564150	Hypothetical gene.	EU956579	longestORF	-
Os07g0564200	Conserved hypothetical protein.	AK072771	A2YMP3 (UniProt)	-
Os07g0564500	Pyridine nucleotide-disulphide oxidoreductase, NAD-binding region domain containing protein.	AK121213	NP_563783.1 (RefSeq)	Pyridine nucleotide-disulphide oxidoreductase, NAD-binding domain
Os07g0564533	Similar to HAT family dimerisation domain containing protein.	AK288170 (DDBJ, Secondary hit)	Q53RM1 (UniProt)	HAT dimerisation
Os07g0564566	Non-protein coding transcript.	CU406694	NONE"	-
Os07g0564600	Similar to Secretory carrier membrane protein.	AK061125	Q8H5X5 (UniProt)	SCAMP
Os07g0564700	Similar to ATMIN7 (ARABIDOPSIS THALIANA HOPM INTERACTOR 7)%3B guanyl-nucleotide exchange factor/ protein binding.	AK059018	NP_189916.4 (RefSeq)	-
Os07g0564750	Conserved hypothetical protein.	FP095875	A2XXG5 (UniProt)	-
Os07g0564800	Protein of unknown function DUF707 family protein.	AK100860	Q8H767 (UniProt)	Protein of unknown function DUF707
Os07g0564901	Non-protein coding transcript.	BT086289	NONE	-
Os07g0565000	Similar to 40S ribosomal protein S11.	AK121056	NP_001105562.1 (RefSeq)	Ribosomal protein S17
Os07g0565100	Similar to 40S ribosomal protein S11.	CT833537	NP_001105562.1 (RefSeq)	Ribosomal protein S17
Os07g0565200	Similar to Cell differentiation protein rcd1.	AK106782	B6TRX0 (UniProt)	Cell differentiation, Rcd1-like
Os07g0565300	Bromodomain containing protein.	AK101955	A2YMP9 (UniProt)	Bromodomain
Os07g0565350	Hypothetical protein.	tplb0052b05 (Wheat FLcDNA)	longestORF	-
Os07g0565400	Similar to SRF8 (STRUBBELIG-RECEPTOR FAMILY 8).	AK242465	NP_001119030.1 (RefSeq)	Protein kinase, catalytic domain
Os07g0565500	Conserved hypothetical protein.	AK107834	F2DAZ7 (UniProt)	-
Os07g0565600	Similar to Peptidyl-prolyl cis-trans isomerase TLP38, chloroplast precursor (EC 5.2.1.8) (PPIase) (Rotamase) (Thylakoid lumen PPIase of 38 kDa) (p38).	AK071983	XP_002885091.1 (RefSeq)	Peptidyl-prolyl cis-trans isomerase, cyclophilin-type
Os07g0565700	Alpha/beta hydrolase family protein.	AK111937	XP_002889847.1 (RefSeq)	Alpha/beta hydrolase fold-1
Os07g0565800	Similar to LLA-115.	AK062834	B2BA79 (UniProt)	-
Os07g0567250	Hypothetical protein.	tplb0054h01 (Wheat FLcDNA)	longestORF	-
Os07g0567300	Hypothetical conserved gene.	AK288489	Q84SM4 (UniProt)	Glycosyltransferase, DXD sugar-binding motif
Os07g0567400	Similar to Cytochrome c6.	AK068245	B6TIZ9 (UniProt)	Cytochrome c domain
Os07g0567500	Similar to predicted protein.	AK373391	Q84SM2 (UniProt)	-
Os07g0567700	Similar to Scarecrow-like 23 (Fragment).	AK059008	Q6ULS4 (UniProt)	Transcription factor GRAS
Os07g0567801	Conserved hypothetical protein.	AK242924	NP_001172118.1 (RefSeq)	-
Os07g0567900	Conserved hypothetical protein.	FP097526	B9FXZ7 (UniProt)	-
Os07g0568000	Apolipophorin III-like domain containing protein.	AK069485	B8B7H7 (UniProt)	Cullin repeat-like-containing domain
Os07g0568100	LRR protein kinase, Common symbiosis signaling (SYM	AK099778 (Genbank)	Q7F1I0 (UniProt)	Protein kinase, catalytic domain
Os07g0568200	Homeodomain-related containing protein.	AK102232	XP_002463045.1 (RefSeq)	Homeodomain-like
Os07g0568300	Similar to ZF protein (Fragment).	AK067784	Q84SL2 (UniProt)	Zinc finger, CCCH-type
Os07g0568400	TB2/DP1 and HVA22 related protein family protein.	AK241061	A2YMS5 (UniProt)	TB2/DP1/HVA22-related protein
Os07g0568500	Peptidase aspartic, active site domain containing protein.	AK059994	B6SLR0 (UniProt)	Uncharacterised protein family Ycf60
Os07g0568600	Similar to calcium-dependent protein kinase, isoform AK1.	AK073000	B6SKK9 (UniProt)	Protein kinase, catalytic domain
Os07g0568650	Hypothetical protein.	tplb0061j19 (Wheat FLcDNA)	longestORF	-
Os07g0568700	Polygalacturonase inhibitor 1 precursor (Polygalacturonase-inhibiting protein) (Floral organ regulator 1).	AK101897 ,AK061685	Q8GT95 (UniProt)	-
Os07g0568800	Hypothetical protein.	AK111260	longestORF	-
Os07g0568900	Conserved hypothetical protein.	AK062660	B8B7I1 (UniProt)	-
Os07g0569000	Conserved hypothetical protein.	AK073915	B8B7I2 (UniProt)	-
Os07g0569100	Remorin, C-terminal region domain containing protein.	AK120160	NP_001150312.1 (RefSeq)	Remorin, C-terminal
Os07g0569166	Conserved hypothetical protein.	AK240724	NP_001175264.1 (RefSeq)	-
Os07g0571100	Hypothetical conserved gene.	AK067380	Q6YTV3 (UniProt)	-
Os07g0571200	Hypothetical protein.	BT067768	longestORF	-
Os07g0571300	Hypothetical protein.	AK107499	longestORF	-
Os07g0571500	Similar to Transmembrane protein 49.	CT831667	B6TDW5 (UniProt)	SNARE associated Golgi protein
Os07g0571600	Chalcone isomerase domain containing protein.	CT837974	NP_001149585.1 (RefSeq)	Chalcone isomerase, subgroup
Os07g0571700	Similar to Transporter-like protein.	AK067178	NP_001151801.1 (RefSeq)	General substrate transporter
Os07g0571800	Similar to Protein YABBY 7.	AK072618	A2PZN8 (UniProt)	YABBY protein
Os07g0571900	Prp18 domain containing protein.	AK101604	B6T2W8 (UniProt)	Splicing factor motif
Os07g0572000	WD40/YVTN repeat-like domain containing protein.	AK111653	A2YMV0 (UniProt)	WD40 repeat
Os07g0572050	Similar to Copper amine oxidase.	ab initio prediction	B7ZYE7 (UniProt)	Copper amine oxidase
Os07g0572075	Hypothetical protein.	BT063833	longestORF	-
Os07g0572100	Similar to Amine oxidase like protein (EC 1.4.3.6) (Copper amine oxidase).	AK099435	B7ZYE7 (UniProt)	Copper amine oxidase
Os07g0572300	Protein of unknown function DUF868, plant family protein.	AK074013	A2YMV3 (UniProt)	Protein of unknown function DUF868, plant
Os07g0572400	Conserved hypothetical protein.	AK067317	B8B7T8 (UniProt)	-

Os07g0572500	Conserved hypothetical protein.	AK108612	B8B7T9 (UniProt)	-
Os07g0572600	Similar to F20D23.3 protein.	AK109147 ,AK072023	Q9SHI5 (UniProt)	GDP-fucose protein O-fucosyltransferase
Os07g0572800	Hypothetical conserved gene.	AK060818	C0M0P2 (UniProt)	Protein kinase, catalytic domain
Os07g0572850	Non-protein coding transcript.	tplb0061f07 (Wheat FLcDNA)	NONE	-
Os07g0572900	Similar to 40S ribosomal protein S13.	AK063015	B4FML8 (UniProt)	Ribosomal protein S15
Os07g0573000	Conserved hypothetical protein.	AK120026	A3BLD8 (UniProt)	-
Os07g0573100	Similar to Adenylyl-sulfate kinase 1, chloroplast precursor (EC 2.7.1.25) (APS kinase) (Adenosine-5'phosphosulfate kinase) (ATP adenosine-5'- phosphosulfate 3'-phosphotransferase).	AK071285	B8B7U1 (UniProt)	Adenylylsulphate kinase, C-terminal
Os07g0573200	Similar to Adenylyl-sulfate kinase.	CT836177	B8B7U1 (UniProt)	-
Os07g0573300	Similar to FYVE finger-containing phosphoinositide kinase (EC 2.7.1.68) (1- phosphatidylinositol-4-phosphate 5-kinase) (PIP5K) (PtdIns(4)P-5- kinase) (PIKfyve) (p235).	AK122181	NP_564103.1 (RefSeq)	Zinc finger, FYVE-type
Os07g0573400	Protein of unknown function DUF239, plant domain containing protein.	AK102732 ,AK065116 ,AK11963	NP_001152099.1 (RefSeq)	Glucoamylase, putatuve
Os07g0573450	Hypothetical protein.	tplb0062i11 (Wheat FLcDNA)	longestORF	-
Os07g0573500	Hypothetical gene.	AK106040	longestORF	-
Os07g0573600	Nucleotide excision repair, TFIIH, subunit TTDA domain containing protein.	AK073925	NP_001148022.1 (RefSeq)	Nucleotide excision repair, TFIIH, subunit TTDA
Os07g0573700	Nucleotide-sugar transporter family protein.	AK070473	NP_001149907.1 (RefSeq)	UDP-galactose transporter
Os07g0573800	Hypothetical protein.	AK107962	longestORF	-
Os07g0573900	Hypothetical conserved gene.	CT836098	NP_001060071.2 (RefSeq)	-
Os07g0574100	Similar to receptor-kinase isolog.	BT083633	NP_001151891.1 (RefSeq)	Protein kinase, catalytic domain
Os07g0574150	Hypothetical protein.	EU951884	longestORF	-
Os07g0574200	Ubiquitin supergroup domain containing protein.	EU972055	Q6ZL10 (UniProt)	Ubiquitin supergroup
Os07g0574400	Hypothetical gene.	AK061367	longestORF	-
Os07g0574500	Ubiquitin domain containing protein.	AK107923	B8YBH7 (UniProt)	Ubiquitin supergroup
Os07g0580200	Conserved hypothetical protein.	AK108520	Q7XI99 (UniProt)	-
Os07g0580300	Hypothetical conserved gene.	CT837805	Q7XI99 (UniProt)	-
Os07g0580500	Similar to BES1/BZR1 protein.	AK106748	NP_001151195.1 (RefSeq)	BZR1, transcriptional repressor
Os07g0580700	Integrin alpha chain, C-terminal cytoplasmic region, conserved site domain containing protein.	AK119427	Q7XI95 (UniProt)	Protein of unknown function DUF2921
Os07g0580733	Protein of unknown function DUF2921 domain containing	ab initio prediction	Q7XI95 (UniProt)	Protein of unknown function DUF2921
Os07g0580766	Protein of unknown function DUF2921 domain containing	ab initio prediction	B8B7S6 (UniProt)	Protein of unknown function DUF2921
Os07g0580800	Hypothetical protein.	AK243347	longestORF	-
Os07g0580900	Similar to GGDP synthase.	AK121529	A9ZN20 (UniProt)	Polyprenyl synthetase
Os07g0581000	Protein of unknown function DUF250 domain containing protein.	AK064035	B6TCN0 (UniProt)	Domain of unknown function DUF250
Os07g0581100	Similar to ruvB-like 2.	ab initio prediction	NP_001148563.1 (RefSeq)	TIP49, C-terminal
Os07g0581300	Similar to predicted protein.	AK061656	XP_002460920.1 (RefSeq)	-
Os07g0581333	Hypothetical protein.	EU945312	longestORF	-
Os07g0581366	Zinc finger, C2H2 domain containing protein.	FP098399	Q7XI88 (UniProt)	Zinc finger, C2H2
Os07g0581400	Hypothetical gene.	AK063137	longestORF	-
Os07g0581550	Hypothetical protein.	EU945738	longestORF	-
Os07g0581700	Similar to HAHB-7 (Fragment).	AK121889	Q7XI85 (UniProt)	Helix-turn-helix motif, lambda-like repressor
Os07g0586500	Similar to SUMO activating enzyme 2.	AK120683	NP_973506.1 (RefSeq)	Ubiquitin-activating enzyme repeat
Os07g0586550	Hypothetical protein.	tplb0044i16 (Wheat FLcDNA)	longestORF	-
Os07g0586600	Conserved hypothetical protein.	AK287951	NP_001175279.1 (RefSeq)	-
Os07g0586700	Similar to HRT transcription factor (Fragment).	AK102792	F1DJZ9 (UniProt)	-
Os07g0586800	Similar to triacylglycerol lipase.	AK287769	NP_001149039.1 (RefSeq)	-
Os07g0586900	GRAS transcription factor domain containing protein.	AK120959	Q8H2X8 (UniProt)	Transcription factor GRAS
Os07g0587000	F-box domain, cyclin-like domain containing protein.	AK107187	GeneMark	F-box domain, cyclin-like
Os07g0587100	Reticulon family protein.	AK243436	NP_565102.1 (RefSeq)	Reticulon
Os07g0587200	NPH3 domain containing protein.	AK108192	B8B841 (UniProt)	NPH3
Os07g0587250	Hypothetical conserved gene.	EU949232	B9FY60 (UniProt)	-
Os07g0587300	Hypothetical conserved gene.	AK287835	NP_001175280.1 (RefSeq)	-
Os07g0587400	Similar to eukaryotic peptide chain release factor subunit 1-1.	AK065768	NP_001151538.1 (RefSeq)	Peptide chain release factor eRF1/aRF1
Os07g0587450	Non-protein coding transcript.	tplb0034d14 (Wheat FLcDNA)	NONE	-
Os07g0587500	Armadillo-like helical domain containing protein.	AK242730	NP_001148265.1 (RefSeq)	Armadillo
Os07g0588000	Interferon-related developmental regulator domain containing	AK099482	B4FQN7 (UniProt)	Interferon-related developmental regulator, N-terminal
Os07g0588100	Pentatricopeptide repeat domain containing protein.	BT084276	Q6ZIP5 (UniProt)	Pentatricopeptide repeat
Os07g0588200	Similar to Formin-like protein 13.	AK059131	Q0D519 (UniProt)	Actin-binding FH2
Os07g0588300	Hypothetical conserved gene.	BT062538	Q6ZIP2 (UniProt)	Protein of unknown function DUF936, plant
Os07g0588400	Similar to basic helix-loop-helix protein / bHLH protein.	ab initio prediction	NP_179083.2 (RefSeq)	Helix-loop-helix DNA-binding
Os07g0696100	Conserved hypothetical protein.	AK071018	B8BN83 (UniProt)	-
Os08g0124500	Similar to Resistance protein candidate (Fragment).	AK098961	B7F8F0 (UniProt)	Protein kinase, catalytic domain
Os08g0124533	Hypothetical protein.	tplb0022l18 (Wheat FLcDNA)	longestORF	-
Os08g0124566	Hypothetical protein.	tplb0022l18 (Wheat FLcDNA)	longestORF	-
Os08g0124600	Similar to J065032N17, full insert sequence.	AK369941	B7F8F0 (UniProt)	Protein kinase, catalytic domain
Os08g0124651	Conserved hypothetical protein.	EU974589	Q6ZFR7 (UniProt)	-
Os08g0124700	Similar to Resistance protein candidate (Fragment).	AK106798	B7F8F0 (UniProt)	Protein kinase, catalytic domain
Os08g0124750	Hypothetical protein.	tplb0022l18 (Wheat FLcDNA)	longestORF	-
Os08g0124850	Hypothetical protein.	tplb0022l18 (Wheat FLcDNA)	longestORF	-
Os08g0125006	Hypothetical protein.	tplb0022l18 (Wheat FLcDNA)	longestORF	-
Os08g0125059	Hypothetical protein.	tplb0022l18 (Wheat FLcDNA)	longestORF	-
Os08g0129500	Conserved hypothetical protein.	AK122021	B9FYX0 (UniProt)	-
Os08g0129600	Conserved hypothetical protein.	AK102166	A2YQW7 (UniProt)	-
Os08g0129700	NAD(P)-binding domain containing protein.	AK099222	Q2LC81 (UniProt)	NAD-dependent epimerase/dehydratase
Os08g0129800	Hypothetical conserved gene.	AK065009	A6N1B0 (UniProt)	-
Os08g0129900	Conserved hypothetical protein.	ab initio prediction	NP_001175367.1 (RefSeq)	-
Os08g0130000	Putative DNA binding domain containing protein.	ab initio prediction	NP_178511.2 (RefSeq)	Mg2+ transporter protein, CorA-like/Zinc transport protein ZntB
Os08g0130100	Zinc finger, LSD1-type domain containing protein.	AK120454	Q84UR0 (UniProt)	Zinc finger, LSD1-type
Os08g0130250	Non-protein coding transcript.	AK240839	NONE	-
Os08g0130300	Hypothetical conserved gene.	AK376532	NP_001060921.2 (RefSeq)	Tetratricopeptide TPR-1
Os08g0130400	AMP-dependent synthetase/ligase domain containing protein.	AK065929	A2YQX4 (UniProt)	AMP-dependent synthetase/ligase
Os08g0130500	Similar to 60S acidic ribosomal protein p0.	AK062008	A6N1T9 (UniProt)	Ribosomal protein 60S
Os08g0130550	Hypothetical protein.	AK371367	longestORF	-
Os08g0130600	BTB domain containing protein.	AK100956	D9ZIR1 (UniProt)	BTB/POZ-like
Os08g0130700	Conserved hypothetical protein.	AK242232	NP_001175368.1 (RefSeq)	-
Os08g0130900	Similar to galactosyltransferase family.	AK243240	B6SXL2 (UniProt)	Galectin, carbohydrate recognition domain
Os08g0131000	Pentatricopeptide repeat domain containing protein.	AK067165 ,AK100132 ,AK10109	F1BL93 (UniProt)	Pentatricopeptide repeat
Os08g0131100	Similar to Cytochrome P450.	AK106843	B6TC85 (UniProt)	Cytochrome P450
Os08g0131150	Hypothetical protein.	EU942896	longestORF	-
Os08g0131200	Similar to Non-specific lipid-transfer protein.	AK063493	A2YQX8 (UniProt)	-
Os08g0131300	Phospholipid/glycerol acyltransferase domain containing protein.	EU965540	Q69R60 (UniProt)	Phospholipid/glycerol acyltransferase
Os08g0282700	Hypothetical gene.	AK071808	longestORF	-
Os08g0283000	Similar to H0315A08.1 protein.	FP099421	Q01I63 (UniProt)	Ribonuclease H1, N-terminal
Os08g0283300	Similar to H0315A08.1 protein.	FP099421	Q01I63 (UniProt)	Ribonuclease H1, N-terminal
Os08g0283600	Similar to H0315A08.1 protein.	FP099421	Q01I63 (UniProt)	Ribonuclease H1, N-terminal
Os08g0283900	Similar to H0315A08.1 protein.	FP099421	Q01I63 (UniProt)	Ribonuclease H1, N-terminal
Os08g0284200	Similar to H0315A08.1 protein.	FP099421	Q01I63 (UniProt)	Ribonuclease H1, N-terminal
Os08g0284500	Similar to H0315A08.1 protein.	FP099421	Q01I63 (UniProt)	Ribonuclease H1, N-terminal
Os08g0477800	PWWP domain containing protein.	AK066304	B8BBS0 (UniProt)	PWWP
Os08g0477900	Helix-loop-helix DNA-binding domain containing protein.	EU968808	NP_001159035.1 (RefSeq)	Helix-loop-helix DNA-binding
Os08g0478000	Conserved hypothetical protein.	AK099412	Q6ZJC6 (UniProt)	-
Os08g0478100	Uncharacterised protein family UPF0029, N-terminal domain containing protein.	AK063259	B8BBS2 (UniProt)	Impact, N-terminal
Os08g0478200	Similar to ATP synthase D chain, mitochondrial (EC 3.6.3.14).	AK068050	NP_001150316.1 (RefSeq)	ATPase, F0 complex, subunit D, mitochondrial
Os08g0478466	Protein of unknown function DUF296 domain containing protein.	AK287800	NP_001152438.1 (RefSeq)	Domain of unknown function DUF296
Os08g0478500	Peptidase C19, ubiquitin carboxyl-terminal hydrolase 2 family	AK099704	A2YWA2 (UniProt)	Peptidase C19, ubiquitin carboxyl-terminal hydrolase 2
Os08g0478566	Similar to Maturase K 2.	AY964640	C6ES29 (UniProt)	-
Os08g0478700	Similar to Mitochondrial uncoupling protein 4.	AK058489	Q66PX4 (UniProt)	Mitochondrial substrate/solute carrier
Os08g0478800	Phosphoglucose isomerase (PGI) family protein.	AK107494	A3BU52 (UniProt)	Phosphoglucose isomerase
Os08g0479300	Cyclin, A/B/D/E domain containing protein.	AK070025	Q4KYM5 (UniProt)	Cyclin, C-terminal
Os08g0479400	Similar to Hydroxyproline-rich glycoprotein DZ-HRGP	AK109528	B6TE62 (UniProt)	Homeobox domain, ZF-HD class
Os08g0486100	Similar to Potential copper-transporting ATPase PAA1 (EC	AK059217	XP_002304082.1 (RefSeq)	ATPase, P-type, H+ transporting proton pump
Os08g0486200	Non-protein coding transcript.	AK063813	NONE	-
Os08g0486233	Similar to EIL transcription factor.	CT835917	Q8W3L9 (UniProt)	Ethylene insensitive 3-like protein, DNA-binding domain
Os08g0486266	Conserved hypothetical protein.	AK288793	Q53LF9 (UniProt)	-
Os08g0486300	Similar to P-type R2R3 Myb protein (Fragment).	BT033636	Q8S416 (UniProt)	SANT domain, DNA binding
Os08g0486400	Hypothetical protein.	EU971393	longestORF	-
Os08g0486500	Conserved hypothetical protein.	AK111410	B9G1H9 (UniProt)	-
Os08g0486700	Conserved hypothetical protein.	AK241195	NP_001062097.1 (RefSeq)	-
Os08g0486750	Non-protein coding transcript.	AK363293	NONE	-
Os08g0486801	Non-protein coding transcript.	AK241869	NONE	-

Os08g0486867	Conserved hypothetical protein.	AK243168	Q75LB0 (UniProt)	-
Os08g0486933	Similar to Peptide transporter-like protein.	ab initio prediction	Q6YS05 (UniProt)	-
Os08g0487000	Conserved hypothetical protein.	AK103460	B8BBV9 (UniProt)	-
Os08g0487050	Hypothetical protein.	EU946896	longestORF	-
Os08g0487100	Similar to BZIP transcription factor BZI-2.	AK107150	NP_001152649.1 (RefSeq)	Basic-leucine zipper
Os08g0487400	Conserved hypothetical protein.	EU949564	B8B3K1 (UniProt)	-
Os08g0487500	Zinc finger, RING/FYVE/PHD-type domain containing protein.	AK066326	B6U108 (UniProt)	Zinc finger, RING-type
Os08g0487700	Helix-loop-helix DNA-binding domain containing protein.	ab initio prediction	NP_001062102.1 (RefSeq)	Helix-loop-helix DNA-binding
Os08g0487800	Similar to Heat-shock protein precursor.	AK122102	Q43638 (UniProt)	Heat shock protein Hsp90
Os08g0487850	Non-protein coding transcript.	BT017162	NONE	-
Os08g0487900	Esterase/lipase/thioesterase domain containing protein.	AK109668	NP_001150405.1 (RefSeq)	-
Os08g0489300	Similar to GMP synthase.	AK065590	B4FL85 (UniProt)	-
Os08g0489800	Phosphatidylinositol 3- and 4-kinase, catalytic domain containing protein.	AK072785	NP_001151804.1 (RefSeq)	Phosphatidylinositol 3-/4-kinase, catalytic
Os08g0490000	Similar to Transcription factor BIM2.	AK070237	B6SVP6 (UniProt)	Helix-loop-helix DNA-binding
Os08g0490100	Similar to PBF protein.	AK241364	Q1HFQ1 (UniProt)	Zinc finger, Dof-type
Os08g0490300	RNA recognition motif, glycine rich protein domain containing protein.	AK066895	A2YWH8 (UniProt)	RNA recognition motif domain
Os08g0490400	Hypothetical gene.	AK334302	GeneMark	-
Os08g0490600	FAS1 domain domain containing protein.	AK108305	Q06IA2 (UniProt)	FAS1 domain
Os08g0490700	Conserved hypothetical protein.	AK069034 ,AK070422	A2YWI2 (UniProt)	-
Os08g0490800	Similar to Histone H2B.	FP097117	B9G1J0 (UniProt)	Histone H2B
Os08g0490900	Similar to Histone H2B.2.	FP098904	C5XPC6 (UniProt)	Histone H2B
Os08g0491000	Hypothetical conserved gene.	AK073673	B9G1J1 (UniProt)	-
Os08g0491100	Conserved hypothetical protein.	AK060215	B8BC14 (UniProt)	-
Os08g0491200	Serine/threonine protein kinase domain containing protein.	AK103656	B7EQ74 (UniProt)	Protein kinase, catalytic domain
Os08g0533900	Hypothetical conserved gene.	AY302058 (DDBJ, Secondary hit)	Q94DG6 (UniProt)	-
Os08g0534200	Similar to HEAT repeat family protein.	AK066926	B6TWZ9 (UniProt)	-
Os08g0534300	Similar to calmodulin binding protein.	tplb0019a16 (Wheat FLcDNA)	NP_001149909.1 (RefSeq)	Auxin responsive SAUR protein
Os08g0534350	Similar to cation cation antiporter.	AK066324 (DDBJ, Secondary hit)	NP_001148426.1 (RefSeq)	Vesicle transport protein, Use1
Os08g0534400	EKC/KEOPS complex, subunit Pcc1 domain containing protein.	AK058382	NP_001172915.1 (RefSeq)	EKC/KEOPS complex, subunit Pcc1
Os08g0534900	Armadillo-type fold domain containing protein.	AK109343 ,AK061777	B6TWZ9 (UniProt)	Armadillo-like helical
Os08g0534950	Similar to calmodulin binding protein.	tplb0019a16 (Wheat FLcDNA)	NP_001149909.1 (RefSeq)	Auxin responsive SAUR protein
Os08g0535000	Similar to cation cation antiporter.	AK066324	NP_001148426.1 (RefSeq)	Vesicle transport protein, Use1
Os08g0535050	Conserved hypothetical protein.	AK101573	A3BV79 (UniProt)	-
Os08g0558200	Thioredoxin fold domain containing protein.	AK069761	B4FN60 (UniProt)	Glutathione S-transferase, N-terminal
Os08g0558300	Harpin-induced 1 domain containing protein.	CT835925	NP_001062493.1 (RefSeq)	Late embryogenesis abundant protein, LEA-14
Os08g0558400	Similar to cDNA clone:J023090L01, full insert sequence.	AK060581	B7EJ91 (UniProt)	-
Os08g0558600	Synaptobrevin domain containing protein.	AK104800	B4FK20 (UniProt)	Synaptobrevin
Os08g0558700	Similar to Multiple myeloma tumor-associated protein 2.	AK103638	B6TFM2 (UniProt)	Kinase phosphorylation domain
Os08g0558800	Similar to Ribosomal protein.	AK071249	B8B9K6 (UniProt)	Ribosomal protein L1
Os08g0558900	Similar to F1F0-ATPase inhibitor protein.	AK099200	Q9XIV2 (UniProt)	ATPase inhibitor, IATP, mitochondria
Os08g0559000	Similar to F1F0-ATPase inhibitor protein.	AK242369	Q9SXG0 (UniProt)	-
Os08g0559200	Similar to Ribosomal protein S25 (40S ribosomal 25S subunit).	AK059582 ,AK068626 ,AK09901[illegible]	NP_001148017.1 (RefSeq)	Ribosomal protein S25
Os08g0559300	Similar to Gibberellin action negative regulator SPY.	AK065854	NP_187761.1 (RefSeq)	Tetratricopeptide TPR-1
Os08g0559400	Similar to Cyclophilin-like protein.	AK072675	Q93VG0 (UniProt)	Peptidyl-prolyl cis-trans isomerase, cyclophilin-type
Os08g0559501	Conserved hypothetical protein.	CT837577	NP_001175701.1 (RefSeq)"	-
Os08g0559600	Similar to Dihydroxy-acid dehydratase (EC 4.2.1.9) (DAD).	AK058527	D2DKF0 (UniProt)	Dihydroxy-acid/6-phosphogluconate dehydratase
Os08g0559700	Protein of unknown function DUF3245 domain containing [illegible]	AK250759	NP_001062504.1 (RefSeq)	Protein of unknown function DUF3245
Os08g0559800	Hypothetical conserved gene.	AK242197	B9FYJ9 (UniProt)	-
Os08g0559900	PAP/25A core domain containing protein.	AK065627	B8B9L0 (UniProt)	Nucleotidyl transferase domain
Os09g0470900	Similar to predicted protein.	AK072191	B8BCN9 (UniProt)	Protein of unknown function DUF3550/UPF0682
Os09g0471000	Similar to cupin, RmlC-type.	AK107828	NP_001147194.1 (RefSeq)	Cysteamine dioxygenase
Os09g0471100	Similar to Peroxidase 17 precursor (EC 1.11.1.7) (Atperox P17) (ATP25a).	AK069281	Q5I3F1 (UniProt)	Plant peroxidase
Os09g0471150	Hypothetical gene.	EU942261	longestORF	-
Os09g0471200	Similar to WAK80 - OsWAK receptor-like protein kinase.	ab initio prediction	NP_001147058.1 (RefSeq)	Protein kinase, catalytic domain
Os09g0471300	Non-protein coding transcript.	AK108005	NONE	-
Os09g0471400	Similar to WAK80 - OsWAK receptor-like protein kinase.	AK060211	NP_001147058.1 (RefSeq)	Protein kinase, catalytic domain
Os09g0471500	Conserved hypothetical protein.	AK111541	A3BZT3 (UniProt)	-
Os09g0471550	EGF-like calcium-binding domain containing protein.	AK241661	A3BZT5 (UniProt)	EGF-like calcium-binding
Os09g0471600	EGF-like calcium-binding domain containing protein.	AK119296	B9G444 (UniProt)	EGF-like calcium-binding
Os09g0471800	Similar to WAK80 - OsWAK receptor-like protein kinase.	AK071360	NP_001147058.1 (RefSeq)	EGF-type aspartate/asparagine hydroxylation site
Os09g0471900	Hypothetical conserved gene.	AK062110	B8BCP5 (UniProt)	-
Os09g0472000	Hypothetical protein.	tplb0055k03 (Wheat FLcDNA)	longestORF	-
Os09g0567500	Similar to Fatty acyl coA reductase.	AK061142	Q8L4V2 (UniProt)	Male sterility
Os09g0567600	Hypothetical protein.	AK106807	longestORF	-
Os09g0567700	WD40 repeat-like domain containing protein.	AK065913	NP_001105835.1 (RefSeq)	WD40 repeat
Os09g0567800	Hypothetical conserved gene.	CT835822	NP_187604.1 (RefSeq)	-
Os09g0567900	Inosine/uridine-preferring nucleoside hydrolase domain containing protein.	AK061415	B6T563 (UniProt)	Inosine/uridine-preferring nucleoside hydrolase domain
Os09g0568000	Conserved hypothetical protein.	AK104856	Q652Q6 (UniProt)	-
Os09g0568050	Hypothetical protein.	tplb0025f09 (Wheat FLcDNA)	longestORF	-
Os09g0568100	Conserved hypothetical protein.	FP092518	B9G564 (UniProt)	-
Os09g0568200	Similar to DNA polymerase epsilon subunit 3.	AK242634	NP_001149275.1 (RefSeq)	Transcription factor CBF/NF-Y/archaeal histone
Os09g0568266	Non-protein coding transcript.	BT018987	NONE	-
Os09g0568400	Similar to Ubiquitin/ribosomal fusion protein (Fragment).	AK060445	Q7XYD4 (UniProt)	Ribosomal protein L40e
Os09g0568500	Germin family protein.	AK108987	Q84XR7 (UniProt)	Germin
Os09g0568600	Putative germin-like protein 9-2.	Q652Q0 (UniProt)	Q652Q0 (UniProt)	Germin
Os09g0568700	RmlC-like jelly roll fold domain containing protein.	AK109010	Q84XR7 (UniProt)	Germin
Os09g0568800	Similar to Ribosomal protein S25 (40S ribosomal 25S subunit).	AK059234	NP_001148017.1 (RefSeq)	Ribosomal protein S25
Os09g0568900	Similar to predicted protein.	AK059442	XP_002879140.1 (RefSeq)	-
Os09g0569000	Hypothetical gene.	AK108627	longestORF	-
Os09g0569100	Haloacid dehalogenase-like hydrolase domain containing protein.	AK071860	B4FI81 (UniProt)	Haloacid dehalogenase-like hydrolase
Os09g0569151	Hypothetical protein.	AK355652	longestORF	-
Os09g0569200	Similar to Beta-amylase (EC 3.2.1.2) (1,4-alpha-D-glucan maltohydrolase).	AK070300	B6SVZ0 (UniProt)	Glycoside hydrolase, family 14B, plant
Os09g0569300	Similar to calmodulin-binding heat-shock protein.	AK069587	NP_001149987.1 (RefSeq)	Lipase, class 3
Os09g0569400	Beta-lactamase-like domain containing protein.	AK070608 ,AK063384	B6U1S6 (UniProt)	Beta-lactamase-like
Os09g0569450	Conserved hypothetical protein.	AK242543	NP_001175993.1 (RefSeq)	-
Os09g0569700	Conserved hypothetical protein.	AK108778	NP_001063979.1 (RefSeq)	-
Os09g0569780	Conserved hypothetical protein.	AK241041	NP_001175963.1 (RefSeq)	-
Os09g0569800	Serine/threonine protein kinase-related domain containing protein.	ab initio prediction	NP_001063980.1 (RefSeq)	Protein kinase, catalytic domain
Os09g0569900	Hypothetical protein.	AK073081	longestORF	-
Os09g0570000	Serine/threonine protein kinase-related domain containing protein.	AK058797	Q2HTK4 (UniProt)	Protein kinase, catalytic domain
Os09g0570100	Hypothetical conserved gene.	AK059199	B9G572 (UniProt)	Protein kinase, catalytic domain
Os09g0570150	Hypothetical conserved gene.	EU953102	B6SK37 (UniProt)	-
Os09g0570200	Zinc finger, C2H2-type domain containing protein.	AK102035	A2Z489 (UniProt)	Zinc finger, C2H2
Os09g0570300	Similar to Short-chain dehydrogenase Tic32.	AK058687	B4FKX6 (UniProt)	Short-chain dehydrogenase/reductase SDR
Os09g0570400	Non-protein coding transcript.	AK061710	NONE	-
Os09g0570500	Zinc finger, RING-type domain containing protein.	AK069259	B9G575 (UniProt)	Zinc finger, RING-type
Os09g0570600	Non-protein coding transcript.	AK105456	NONE	-
Os09g0570800	Isopenicillin N synthase family protein.	AK064211	NP_001149522.1 (RefSeq)	Isopenicillin N synthase
Os09g0570850	Similar to Histone H2B.	ab initio prediction	B6SGC3 (UniProt)	Histone H2B
Os09g0570900	Similar to amino acid binding protein.	AK068397	B6TMA2 (UniProt)	-
Os09g0570951	Hypothetical gene.	tplb0017n19 (Wheat FLcDNA)	longestORF	-
Os09g0571000	Protein of unknown function DUF966 family protein.	ab initio prediction	Q651B3 (UniProt)	Protein of unknown function DUF966
Os09g0571033	Conserved hypothetical protein.	AK241104	NP_001175997.1 (RefSeq)	-
Os09g0571100	Pectin lyase fold/virulence factor domain containing protein.	AK106869	C5X6M1 (UniProt)	Pectinesterase, catalytic
Os09g0571200	C2 domain containing protein.	AK062604	B6TT83 (UniProt)	C2 calcium-dependent membrane targeting
Os09g0571400	Cyclophilin 1.	AK103109	Q40672 (UniProt)	Peptidyl-prolyl cis-trans isomerase, cyclophilin-type
Os09g0571500	Similar to lipase class 3 family protein.	AK106610	NP_181773.2 (RefSeq)	Lipase, class 3
Os10g0355800	Similar to ATP synthase CF1 beta subunit.	ab initio prediction	NP_039390.1 (RefSeq)	ATPase, F1/V1/A1 complex, alpha/beta subunit, nucleotide-binding domain
Os10g0356000	Similar to ribulose-1,5-bisphosphate carboxylase/oxygenase large subunit.	ab initio prediction	NP_039391.1 (RefSeq)	Ribulose bisphosphate carboxylase, large subunit, C-terminal
Os10g0357700	Conserved hypothetical protein.	AK106693	NP_001053142.1 (RefSeq)	-
Os10g0410600	Similar to Phosphoprotein phosphatase 2A isoform 4.	AK099604	A3C4N5 (UniProt)	Metallophosphoesterase domain
Os10g0410650	Hypothetical gene.	BT018461	longestORF	-
Os10g0410700	Similar to SET domain protein 123.	AK111981 ,AK111906	NP_001105199.1 (RefSeq)	SET domain
Os10g0410750	Protein kinase, catalytic domain domain containing protein.	EU975108	XP_002450423.1 (RefSeq)	Protein kinase, catalytic domain
Os10g0410900	Conserved hypothetical protein.	AK119279	Q7XER2 (UniProt)	-
Os10g0411100	Protein of unknown function DUF620 domain containing protein.	BT088024	XP_002468447.1 (RefSeq)	Protein of unknown function DUF620
Os10g0411200	Hypothetical protein.	BT085554	longestORF	-
Os10g0411500	IQ calmodulin-binding region domain containing protein.	AK072572	B8BGS6 (UniProt)	-

Os10g0411600	Hypothetical protein.	AK108669	longestORF	-
Os10g0411650	Non-protein coding transcript.	EU947431	NONE	-
Os10g0411700	Similar to S28 ribosomal protein (Fragment).	AK058209 ,AK121099	Q7X9K4 (UniProt)	-
Os10g0411750	Hypothetical conserved gene.	AK241834	Q7XEQ4 (UniProt)	JNK/Rab-associated protein-1, N-terminal
Os10g0411800	Similar to 40S ribosomal protein S17-3.	AK059434	NP_001149330.1 (RefSeq)	Ribosomal protein S17e
Os10g0412000	Similar to aminophospholipid ATPase.	AK062158	XP_002314626.1 (RefSeq)	ATPase, P-type, K/Mg/Cd/Cu/Zn/Na/Ca/Na/H-transporter
Os10g0412050	Similar to aminophospholipid ATPase.	BT035338	NP_176191.1 (RefSeq)	ATPase, P-type, ATPase-associated domain
Os10g0412100	Similar to Endonuclease/Exonuclease/phosphatase family protein, expressed.	AK105868	Q10R98 (UniProt)	Endonuclease/exonuclease/phosphatase
Os10g0412350	Similar to H0315A08.1 protein.	FP099421	Q01I63 (UniProt)	Ribonuclease H1, N-terminal
Os10g0428900	Similar to BTB/POZ domain containing protein.	ab initio prediction	Q7XEB6 (UniProt)	MATH
Os10g0429200	BTB/POZ fold domain containing protein.	AK248323	Q7XEA8 (UniProt)	BTB/POZ fold
Os10g0429300	Kelch related domain containing protein.	tplb0003h03 (Wheat FLcDNA)	Q7XEA7 (UniProt)	BTB/POZ-like
Os10g0429600	Similar to BTB/POZ domain containing protein.	AK369602	Q7XEA4 (UniProt)	BTB/POZ-like
Os10g0429651	Hypothetical conserved gene.	AK248323	Q7XEA4 (UniProt)	BTB/POZ-like
Os10g0430200	Similar to Sinapyl alcohol dehydrogenase.	AK099176	Q8S411 (UniProt)	Alcohol dehydrogenase superfamily, zinc-type
Os10g0430600	Aminotransferase, class-II, pyridoxal-phosphate binding site domain containing protein.	AK069549	B8BGZ5 (UniProt)	-
Os10g0430700	Conserved hypothetical protein.	AK072713	B8BGZ5 (UniProt)	-
Os10g0430750	Hypothetical conserved gene.	AK062794 (DDBJ, Secondary hit)	longestORF	-
Os10g0430800	Conserved hypothetical protein.	AK111261	A3C4Z6 (UniProt)	-
Os10g0430900	Tyrosine protein kinase domain containing protein.	AK121718	Q5XPL8 (UniProt)	Protein kinase, catalytic domain
Os10g0475400	Nucleoside phosphatase GDA1/CD39 domain containing protein.	EU946507	Q8W5Q4 (UniProt)	Nucleoside phosphatase GDA1/CD39
Os10g0475900	Ubiquitin domain containing protein.	AK101853	Q8W5Q6 (UniProt)	Ubiquitin
Os10g0476000	Similar to Adaptin ear-binding coat-associated protein 2.	AK100161	B4FAZ6 (UniProt)	Adaptin ear-binding coat-associated protein 1 NECAP-1
Os10g0476100	Similar to Protein kinase MK6.	AK375579	Q9LW99 (UniProt)	Protein kinase, catalytic domain
Os10g0476200	Hypothetical gene.	AK066729	longestORF	-
Os10g0476300	Similar to Dual specificity kinase 1.	AK120765	NP_193170.1 (RefSeq)	Protein kinase, catalytic domain
Os10g0476400	Snf7 family protein.	AK104071	B6T5E0 (UniProt)	Snf7
Os10g0476500	Conserved hypothetical protein.	AK110990	B9G687 (UniProt)	-
Os10g0476600	Similar to Protein phosphatase 2A 62 kDa B" regulatory subunit (Protein phosphatase 2A 62 kDa B regulatory subunit) (Protein phosphatase 2A alpha).	AK101394	NP_175847.1 (RefSeq)	EF-hand-like domain
Os10g0476700	Esterase, SGNH hydrolase-type, subgroup domain containing [illegible]	AK060164	B6TVA2 (UniProt)	Lipase, GDSL
Os10g0476900	Pentatricopeptide repeat domain containing protein.	AK103437	A3C5Q4 (UniProt)	Pentatricopeptide repeat
Os10g0477000	Armadillo-like helical domain containing protein.	AK106419	Q9AV01 (UniProt)	Armadillo-type fold
Os10g0477100	Similar to Ankyrin-like protein.	AK067258	NP_567427.1 (RefSeq)	Protein of unknown function DUF248, methyltransferase putative
Os10g0477200	Pentatricopeptide repeat domain containing protein.	AK067154	A3C5Q7 (UniProt)	Pentatricopeptide repeat
Os10g0477301	Conserved hypothetical protein.	AK288518	Q69WY5 (UniProt)	-
Os10g0507500	Conserved hypothetical protein.	FP097010	B9G6J5 (UniProt)	-
Os10g0507600	PapD-like domain containing protein.	AK058656	Q8LN94 (UniProt)	Major sperm protein
Os10g0507700	Conserved hypothetical protein.	EU968230	Q8LN95 (UniProt)	-
Os10g0507800	Similar to Chaperone protein dnaJ 13.	AK066499	B6T7S1 (UniProt)	Heat shock protein DnaJ, N-terminal
Os10g0508000	Similar to C-terminal peptide-binding protein 1.	CT837938	Q1EPX7 (UniProt)	Multicopper oxidase, type 1
Os10g0508050	Hypothetical protein.	tplb0021n10 (Wheat FLcDNA)	longestORF	-
Os10g0508100	Protein of unknown function DUF641, plant domain containing protein.	AK287902	NP_001065020.1 (RefSeq)	Domain of unknown function DUF641, plant
Os10g0508300	Similar to Protein YABBY 3.	AB274015	Q8L556 (UniProt)	High mobility group, HMG1/HMG2
Os10g0508400	Similar to Methionine aminopeptidase-like protein.	AK066207	B9G6J9 (UniProt)	Peptidase M24, structural domain
Os10g0508500	Similar to tubulin folding cofactor.	BT041825	XP_002320715.1 (RefSeq)	Armadillo-like helical
Os10g0508600	Protein of unknown function DUF1352 domain containing [illegible]	AK103246	Q8LN74 (UniProt)	Protein of unknown function DUF1352
Os10g0510000	Similar to actin.	AK070531 ,AK101613	A3C6D7 (UniProt)	Actin-like
Os10g0510050	Hypothetical gene.	BT018641	longestORF	-
Os10g0510200	Hypothetical conserved gene.	AK374096	Q94GX3 (UniProt)	Protein of unknown function DUF241, plant
Os10g0510300	Peptidase C12, ubiquitin carboxyl-terminal hydrolase 1 domain containing protein.	AK288012	NP_172132.1 (RefSeq)	Plant organelle RNA recognition domain
Os10g0510400	Protein of unknown function DUF248, methyltransferase putative family protein.	AK073237	NP_001148962.1 (RefSeq)	Protein of unknown function DUF248, methyltransferase putative
Os10g0510500	Auxin responsive SAUR protein family protein.	AK068270 ,AK109491	B6TBH1 (UniProt)	Auxin responsive SAUR protein
Os10g0510700	Similar to calcium dependent protein kinase1.	AK376289	NP_001105740.1 (RefSeq)	Protein kinase, catalytic domain
Os10g0511200	Similar to Cytochrome P450 family protein.	ab initio prediction	Q337C6 (UniProt)	Cytochrome P450
Os10g0511300	Similar to mTERF domain-containing protein, mitochondrial.	ab initio prediction	E1UHI8 (UniProt)"	-
Os10g0511400	Peptidase S28 family protein.	AK070496	Q9FW17 (UniProt)	Peptidase S28
Os10g0511600	Similar to prolyl carboxypeptidase like protein.	AK073197	NP_001149727.1 (RefSeq)	Peptidase S28
Os10g0511800	Similar to Plus-3 domain containing protein, expressed.	EU942983	Q7XCX5 (UniProt)	SWIB/MDM2 domain
Os10g0511900	Similar to Plus-3 domain containing protein, expressed.	AK060521	Q7XCX5 (UniProt)	Zinc finger, CCCH-type
Os10g0512001	Hypothetical gene.	AK242263	NP_001176238.1 (RefSeq)	-
Os10g0512100	Protein of unknown function DUF726 family protein.	BT033755	NP_001176237.1 (RefSeq)	Protein of unknown function DUF726
Os10g0512400	Cytochrome P450 family protein.	AK067847	B6SWS3 (UniProt)	Cytochrome P450
Os10g0512450	Hypothetical protein.	EU942896	longestORF	-
Os10g0512500	Similar to Crs1/yhby domain containing protein (Fragment).	AK067203	A6N061 (UniProt)	RNA-binding, CRM domain
Os10g0512700	Similar to SH3 domain-containing protein 3.	AK067223	B6STF7 (UniProt)	Src homology-3 domain
Os10g0512800	Similar to predicted protein.	AK242756	NP_195616.2 (RefSeq)	Kinesin, motor domain
Os10g0536000	Similar to SUMO E2 conjugating enzyme SCE1.	AK105227	B8BI11 (UniProt)	Ubiquitin-conjugating enzyme, E2
Os10g0536050	Similar to Ubiquitin conjugating enzyme (Fragment).	U49913	Q41762 (UniProt)	-
Os10g0536100	Similar to MADS-box transcription factor 56.	AK070135	P0C5B2 (UniProt)	Transcription factor, MADS-box
Os10g0536400	Similar to Oxidoreductase, 2OG-Fe oxygenase family protein, expressed.	AK061357	Q7G1T5 (UniProt)	-
Os10g0536450	Hypothetical protein.	tplb0008n13 (Wheat FLcDNA)	longestORF	-
Os10g0536500	Peptidase S10, serine carboxypeptidase, active site domain containing protein.	AK066849 ,AK103995	A2Z9R0 (UniProt)	-
Os10g0536600	Plant peroxidase domain containing protein.	ab initio prediction	A2Z9R1 (UniProt)	Plant peroxidase
Os10g0536700	Similar to Peroxidase 1.	AK105708	Q8LMR7 (UniProt)	Plant peroxidase
Os10g0536900	Hypothetical protein.	tplb0030i03 (Wheat FLcDNA)	longestORF	-
Os10g0537100	Hypothetical conserved gene.	FP096459	Q8LNN8 (UniProt)	Transcriptional factor B3
Os10g0537300	Conserved hypothetical protein.	AK068850	A2Z9R4 (UniProt)	-
Os10g0537400	Similar to metal ion binding protein.	AK366348	B6T4Q0 (UniProt)	Heavy metal-associated domain, HMA
Os10g0537450	Hypothetical protein.	tplb0046l08 (Wheat FLcDNA)	longestORF	-
Os10g0537500	Similar to Tobamovirus multiplication 3.	AK061395 ,AK101318	Q402F3 (UniProt)	Domain of unknown function DUF1084
Os10g0537600	Conserved hypothetical protein.	AK103560	Q8LNN4 (UniProt)	-
Os10g0543400	Chitinase 8.	Q7XCK6 (UniProt)	Q7XCK6 (UniProt)	Glycoside hydrolase, family 19, catalytic
Os10g0543500	Six-bladed beta-propeller, TolB-like domain containing protein.	AK106264	Q109A9 (UniProt)	Six-bladed beta-propeller, TolB-like
Os10g0543800	Similar to Glutathione S-transferase GST 11 (EC 2.5.1.18).	CT836577	NP_001104983.1 (RefSeq)	Glutathione S-transferase, N-terminal
Os10g0544200	Basic helix-loop-helix dimerisation region bHLH domain containing protein.	AK063669	NP_001149796.1 (RefSeq)	Helix-loop-helix DNA-binding
Os10g0544500	Similar to nucleotide binding protein.	AK060182	NP_001150616.1 (RefSeq)	WD40 repeat
Os10g0544600	Zinc finger, RING/FYVE/PHD-type domain containing protein.	AK119332	NP_001151464.1 (RefSeq)	Zinc finger, RING-type
Os10g0544900	Similar to Protein phosphatase 2C-like protein.	AK119635	B6TZR2 (UniProt)	Protein phosphatase 2C, manganese/magnesium aspartate binding site
Os10g0544933	Non-protein coding transcript.	EU943254	NONE	-
Os10g0544950	Hypothetical gene.	AK242432	longestORF	-
Os10g0544966	Hypothetical protein.	tplb0043b08 (Wheat FLcDNA)	longestORF	-
Os10g0545000	Similar to magnesium transporter CorA-like family protein.	AK069472	NP_567076.1 (RefSeq)	Fibronectin-attachment
Os10g0545100	Similar to predicted protein.	AK287840	B9G1Z7 (UniProt)	Uncharacterised conserved protein UCP014543
Os10g0545200	Similar to 4,4-dimethyl-sterol C4-methyl-oxidase (Fragment).	AK098884 ,AK061779	B4FN64 (UniProt)	Fatty acid hydroxylase
Os10g0545300	Zinc finger, CCHC retroviral-type domain containing protein.	AK065156 ,AK121559	NP_001149324.1 (RefSeq)	Zinc finger, CCHC-type
Os10g0545500	Similar to xyloglucan endotransglucosylase/hydrolase protein 32.	FJ264508	NP_001148432.1 (RefSeq)	Glycoside hydrolase, family 16
Os10g0545600	Similar to transposon protein.	EU976069	B6UFQ4 (UniProt)	Zinc finger, RING-type
Os10g0545700	Dual-specificity tyrosine-phosphatase CDC25, Arsenic [illegible]	AY860059 (Genbank)	Q336V5 (UniProt)	-
Os10g0545800	Nucleic acid-binding, OB-fold domain containing protein.	AK103222	NP_001152237.1 (RefSeq)	CcmE/CycJ protein
Os10g0546100	Pollen Ole e 1 allergen and extensin domain containing protein.	AK240974	NP_001065213.1 (RefSeq)	Pollen Ole e 1 allergen/extensin
Os10g0546200	Glycosyl transferase, family 1 domain containing protein.	AK372742	Q9AV30 (UniProt)	Glycosyl transferase, family 1
Os10g0546300	Protein of unknown function DUF2451, C-terminal domain containing protein.	AK103033	B9G6U9 (UniProt)	Protein of unknown function DUF2451, C-terminal
Os10g0546400	Conserved hypothetical protein.	AK069919	B9G6V0 (UniProt)	-
Os10g0546600	Similar to Chloroplast carotenoid epsilon-ring hydroxylase.	AK065689	NP_190881.2 (RefSeq)	Cytochrome P450
Os10g0580500	Essential protein Yae1, N-terminal domain containing protein.	CT837648	NP_001045408.2 (RefSeq)	Essential protein Yae1, N-terminal
Os10g0580600	Protein of unknown function DUF1997 domain containing [illegible]	AK370224	Q9FRM6 (UniProt)	Protein of unknown function DUF1997
Os10g0580700	Ankyrin repeat containing protein.	AK071604	NP_193650.2 (RefSeq)	Ankyrin repeat
Os10g0580750	Hypothetical gene.	BT019171	longestORF	-
Os10g0580800	Similar to NClpP4 (Fragment).	AK104823 ,AK065306 ,AK1043[illegible]	B4FP39 (UniProt)	Peptidase S14, ClpP
Os10g0580900	Non-protein coding transcript.	AK062821	NONE	-
Os10g0581000	Conserved hypothetical protein.	AK242551	B9G779 (UniProt)	-
Os11g0127600	No apical meristem (NAM) protein domain containing protein.	AK067922	Q5CD17 (UniProt)	No apical meristem
Os11g0127700	Conserved hypothetical protein.	AK103742	A2ZAZ8 (UniProt)	-

Os11g0127800	Similar to FHA domain containing protein, expressed.	AK104250 ,AK104015	Q2QYC9 (UniProt)	Forkhead-associated
Os11g0127900	Similar to 40S ribosomal protein S16.	AK059550	Q0IQF7 (UniProt)	Ribosomal protein S9
Os11g0127951	Hypothetical protein.	ab initio prediction	NONE	-
Os11g0128000	Similar to secondary cell wall-related glycosyltransferase family [illegible]	AK243483	NP_001151894.1 (RefSeq)	Exostosin-like
Os11g0128300	Similar to ZF-HD homeobox protein (Fragment).	AK063715	Q2QYC5 (UniProt)	ZF-HD homeobox protein, Cys/His-rich dimerisation domain
Os11g0128400	CDC45-like protein family protein.	AK102291	NP_189146.1 (RefSeq)	CDC45 family
Os11g0128500	Similar to Myb factor.	AK241549	O04109 (UniProt)	SANT domain, DNA binding
Os11g0128600	Conserved hypothetical protein.	ab initio prediction	NP_001065637.1 (RefSeq)	-
Os11g0128700	Mad3/BUB1 homology region 1 domain containing protein.	AK102502 (DDBJ, Secondary hit)	Q2RB24 (UniProt)	Mad3/BUB1 homology region 1
Os11g0128800	Similar to Homoserine dehydrogenase-like protein.	AK071434	B8AES5 (UniProt)	Homoserine dehydrogenase, catalytic
Os11g0128932	Hypothetical gene.	BT054648	longestORF	-
Os11g0129000	Similar to Transparent testa 12 protein.	AK067875 (DDBJ, Secondary hit)	B6SSE0 (UniProt)	Multi antimicrobial extrusion protein
Os11g0129101	Non-protein coding transcript.	BT054648	NONE	-
Os11g0129200	Multi antimicrobial extrusion protein MatE family protein.	AK067521 (DDBJ, Secondary hit)	Q2RB20 (UniProt)	Multi antimicrobial extrusion protein
Os11g0129301	Hypothetical gene.	BT054648	longestORF	-
Os11g0129500	Similar to GDSL-like Lipase/Acylhydrolase family protein, [illegible]	AK107889	Q2RB18 (UniProt)	-
Os11g0129600	Hypothetical conserved gene.	AK241463	NP_001066048.1 (RefSeq)	Engulfment/cell motility, ELMO
Os11g0129700	Similar to AP2-1 protein (Fragment).	AK112088	Q2RB16 (UniProt)	-
Os11g0312400	Adenylate kinase B (EC 2.7.4.3) (ATP-AMP [illegible]	AK073605	Q08480 (UniProt)	Adenylate kinase
Os11g0323860	Hypothetical conserved gene.	AK243521	B9GCL3 (UniProt)	-
Os11g0334938	Similar to Class III peroxidase 111.	BT085722	B6TU39 (UniProt)	Plant peroxidase
Os11g0340477	Zinc finger, C2H2-type domain containing protein.	AK108997	A2XIC5 (UniProt)	Zinc finger, C2H2
Os11g0346016	Similar to calmodulin binding protein.	AK372659	NP_001151502.1 (RefSeq)	Calmodulin binding protein-like
Os11g0351555	Similar to A.thaliana gene induced upon wounding stress.	AK060848	D6NIX1 (UniProt)	NAD
Os11g0357094	Pentatricopeptide repeat domain containing protein.	ab initio prediction	A2XIB7 (UniProt)	Pentatricopeptide repeat
Os11g0362633	Hypothetical conserved gene.	FP093303	A9NKD9 (UniProt)	Protein of unknown function DUF3743
Os11g0368172	Peptidase S8 and S53, subtilisin, kexin, sedolisin domain containing protein.	AK107787	A9NKD9 (UniProt)	Protein of unknown function DUF3743
Os11g0373711	Hypothetical gene.	AK101901	longestORF	-
Os11g0379251	Similar to calmodulin binding protein.	AK372659	NP_001151502.1 (RefSeq)	Calmodulin binding protein-like
Os11g0393097	Similar to chlorophyll a-b binding protein M9, chloroplastic precursor.	AJ234427	NP_001105545.1 (RefSeq)"	-
Os11g0406945	Similar to Phenazine biosynthesis-like protein, expressed.	AK333982	Q10J41 (UniProt)	Phenazine biosynthesis PhzC/PhzF protein
Os11g0412484	Hypothetical conserved gene.	AK243521	B9GCL3 (UniProt)	-
Os11g0697100	Non-protein coding transcript.	AK067885	NONE	-
Os11g0697350	Conserved hypothetical protein.	AK241561 (DDBJ, Secondary hit)	NP_001176733.1 (RefSeq)"	-
Os11g0697600	Hypothetical gene.	CT841618 (DDBJ, Secondary hit)	longestORF	-
Os11g0698567	Conserved hypothetical protein.	AK241561	NP_001176733.1 (RefSeq)	-
Os11g0699532	Hypothetical gene.	CT841618	longestORF	-
Os11g0709000	Similar to H0124E07.4 protein.	AK241872 (DDBJ, Secondary hit)	NP_001175717.1 (RefSeq)	-
Os12g0199100	NB-ARC domain containing protein.	AK065572	B9GCB3 (UniProt)	Disease resistance protein
Os12g0199200	Non-protein coding transcript.	AK066878	NONE	-
Os12g0199500	Similar to N-methyltransferase.	U43498	Q2QWD0 (UniProt)	O-methyltransferase, family 2
Os12g0199800	Similar to Cyt-P450 monooxygenase.	AK288730	Q8S3F6 (UniProt)	Cytochrome P450
Os12g0469300	Non-protein coding transcript.	tplb0015j08 (Wheat FLcDNA)	NONE	-
Os12g0498650	Protein kinase, catalytic domain domain containing protein.	AK375837	Q2QQD0 (UniProt)	Protein kinase, catalytic domain
Os12g0498700	Mannosyltransferase, PIG-V family protein.	AK073582	NP_172652.2 (RefSeq)	GPI mannosyltransferase 2-like
Os12g0498800	Conserved hypothetical protein.	AK067767	B8BI52 (UniProt)	-
Os12g0498900	Conserved hypothetical protein.	AK073621	A3CHK6 (UniProt)	-
Os12g0499700	Similar to Protein kinase domain containing protein.	EU955852	Q2QQC1 (UniProt)	Protein kinase, catalytic domain
Os12g0500050	Non-protein coding transcript.	AK242983	NONE	-
Os12g0500225	Conserved hypothetical protein.	AK359595	A2ZNF9 (UniProt)	-
Os12g0500400	Similar to Protein kinase domain containing protein.	AK334099	Q2QQB6 (UniProt)	Protein kinase, catalytic domain
Os12g0522516	Similar to ATRL5 (ARABIDOPSIS RAD-LIKE 5)%3B DNA binding / transcription factor.	tplb0003e07 (Wheat FLcDNA)	NP_564087.2 (RefSeq)	SANT domain, DNA binding
Os12g0524201	Hypothetical conserved gene.	AK288953	B6U883 (UniProt)	-
Os12g0524750	Similar to ribosomal protein S7.	ab initio prediction	YP_514635.1 (RefSeq)	Ribosomal protein S7
Os12g0525300	Hypothetical protein.	ab initio prediction	NONE	-
Os12g0613000	Similar to NF-YA subunit.	AK065163	B6TY54 (UniProt)	CCAAT-binding transcription factor, subunit B
Os12g0613100	Similar to Amino acid permease family protein.	AK332141	Q2QM92 (UniProt)	Amino acid/polyamine transporter I
Os12g0613150	Hypothetical protein.	tplb0044b08 (Wheat FLcDNA)	longestORF	-
Os12g0613200	Hypothetical conserved gene.	AK069599	B9GE94 (UniProt)	SET domain
Os12g0613250	Similar to BTB/POZ%3B Superoxide dismutase, copper/zinc binding%3B NPH3.	EU368707	A2Q365 (UniProt)	BTB/POZ-like
Os12g0613300	Similar to Single myb histone 6.	AK105660	Q761Y7 (UniProt)	SANT domain, DNA binding
Os12g0613500	Similar to SKP40, C-terminal domain containing protein, [illegible]	AK058205	Q2QM88 (UniProt)	LisH dimerisation motif
Os12g0613600	Remorin, C-terminal region domain containing protein.	AK111002	Q2QM85 (UniProt)	Remorin, C-terminal
Os12g0613700	Similar to Auxin response factor 25.	AK065025	Q2QM84 (UniProt)	DNA-binding pseudobarrel domain
Os12g0613850	Similar to Galactosyltransferase family.	ab initio prediction	B6SVW0 (UniProt)	Galectin, carbohydrate recognition domain
Os12g0614000	Conserved hypothetical protein.	AK068768	B8BN00 (UniProt)	-
Os12g0614050	Hypothetical gene.	BT019058	longestORF	-
Os12g0614100	Similar to Lipase family protein.	BT042072	Q2QM82 (UniProt)	Lipase, class 3
Os12g0614200	Similar to Lipase family protein.	BT042072	Q2QM81 (UniProt)	Lipase, class 3
Os12g0614201	Conserved hypothetical protein.	AK243381	NP_001177060.1 (RefSeq)	-
Os12g0641200	Hypothetical protein.	AK064961	longestORF	-
Os12g0641300	Similar to Zn-dependent hydrolases of the beta-lactamase fold.	AK061015	C3TX72 (UniProt)	Beta-lactamase-like
Os12g0641400	Similar to Sucrose transporter.	AK067030	Q0ILJ3 (UniProt)	Major facilitator superfamily domain, general substrate transporter
Os12g0641500	Similar to RecF/RecN/SMC N terminal domain containing protein, expressed.	AK065733	Q2QLI0 (UniProt)	RecF/RecN/SMC
Os12g0641600	Non-protein coding transcript.	AK289049	NONE	-
**SH527 pedigree**				
Os01g0169851	Hypothetical conserved gene.	ab initio prediction	Q5VQG8	-
Os01g0169900	Protein of unknown function DUF1421 family protein.	AK121293	B6SV28	Protein of unknown function DUF1421
Os01g0170000	Raffinose synthase family protein.	AK061224	Q5VQG4	Raffinose synthase
Os01g0170051	Hypothetical protein.	tplb0031h14 (Wheat FLcDNA)	longestORF	-
Os01g0170100	D111/G-patch domain containing protein.	AK242074	Q5VQM8	D111/G-patch
Os01g0170300	Serine/threonine protein kinase-related domain containing protein.	AK241418	NP_001042139.2	Protein kinase, catalytic domain
Os01g0170500	Protein of unknown function DUF239, plant domain containing protein.	AK106837	B6SXW9	Glucoamylase, putatuve
Os01g0170600	UspA domain containing protein.	AK067202	D2JYS0	Universal stress protein A
Os01g0170700	Thiol-activated cytolysin family protein.	AK101269	A2ZPR3	-
Os01g0170800	Pentatricopeptide repeat domain containing protein.	AK101200	A2WL64	Pentatricopeptide repeat
Os01g0170900	Conserved hypothetical protein.	AK111084	A2WL65	-
Os01g0171000	Similar to Leucine-rich repeat receptor-like kinase.	AK060282	Q75UP2	Protein kinase, catalytic domain
Os01g0171100	Ankyrin repeat domain containing protein.	AK367141	B8ADH1	Ankyrin repeat
Os01g0171200	Conserved hypothetical protein.	AK107515	B9ET36	-
Os01g0171800	Conserved hypothetical protein.	AK067987	Q5VQL4	-
Os01g0172000	Glycosyltransferase, ALG3 domain containing protein.	AK068405	A2ZPS3	Glycosyltransferase, ALG3
Os01g0172100	Similar to Triose phosphate/phosphate translocator, non-green plastid, chloroplast precursor (CTPT).	AK060343	Q5VQL3	Drug/metabolite transporter
Os01g0172200	DNA/RNA helicase, DEAD/DEAH box type, N-terminal domain containing protein.	AK100326	C6KML6	RNA helicase, ATP-dependent, DEAD-box, conserved site
Os01g0172300	Conserved hypothetical protein.	AK106113	B8ADH6	-
Os01g0172400	Similar to Phospholipase D alpha 1.	AK061865	B8ADH7	Phospholipase D/Transphosphatidylase
Os01g0172600	Similar to electron carrier/ heme binding / peroxidase.	AK058554	NP_181679.3	Plant peroxidase
Os01g0172701	Regulator of chromosome condensation, RCC1 domain containing protein.	CT833782	longestORF	-
Os01g0172800	Embryo-specific 3 family protein.	AK067273	B6U3F4	Lipase/lipooxygenase, PLAT/LH2
Os01g0172900	Glycoside hydrolase, family 28 domain containing protein.	ab initio prediction	B9ETD9	Glycoside hydrolase, family 28
Os01g0173000	Putative thiol-disulphide oxidoreductase DCC domain containing protein.	AK287669	GeneMark	Putative thiol-disulphide oxidoreductase DCC
Os01g0173100	Alba, DNA/RNA-binding protein family protein.	AK121114	NP_564325.1	DNA/RNA-binding protein Alba-like
Os01g0173600	Glyoxalase/bleomycin resistance protein/dioxygenase domain containing protein.	CT835513	B4ERW1	-
Os01g0179800	TMS membrane protein/tumour differentially expressed protein family protein.	AK069734	B6SVV5	TMS membrane protein/tumour differentially expressed protein
Os01g0179901	Conserved hypothetical protein.	AK242152	NP_001172204.1	-
Os01g0180000	Pistil-specific extensin-like protein family protein.	AK103199	NP_001150408.1	Leucine-rich repeat
Os01g0180050	Hypothetical protein.	BT068200	longestORF	-
Os01g0180300	Hypothetical conserved gene.	AK061954	B9ETH2	-
Os01g0180400	Protein of unknown function DUF581 domain containing protein.	AK108226	B9ETH3	Protein of unknown function DUF581
Os01g0180500	Non-protein coding transcript.	EU973947	NONE	-
Os01g0180600	Similar to MutS homolog 7 (Fragment).	AK122068	Q8RVT1	DNA mismatch repair protein MutS, C-terminal
Os01g0180700	Hypothetical conserved gene.	AK240913	Q9AQU6	-

Os01g0180800	Similar to heat shock 70 kDa protein 4.	AK061691	NP_001147805.1	Heat shock protein Hsp70
Os01g0180850	Hypothetical protein.	tplb0021a19 (Wheat FLcDNA)	longestORF	-
Os01g0180900	Conserved hypothetical protein.	AK357358	NP_001172205.1	-
Os01g0181000	Frigida-like domain containing protein.	EU948578	Q5VR39	Frigida-like
Os01g0181033	Similar to NADH dehydrogenase subunit 1.	AJ010976	YP_024343.1	NADH:ubiquinone oxidoreductase, subunit 1/F420H2 oxidoreductase subunit [illegible]
Os01g0181166	Hypothetical gene.	CU405586	longestORF	-
Os01g0182832	Hypothetical conserved gene.	EU940917	B9ETI1	-
Os01g0182900	Conserved hypothetical protein.	AK061255	B9ETI1	-
Os01g0183000	Similar to GmCK3p (EC 2.7.1.32) (Fragment).	AK287974	B6UIG4	Choline/ethanolamine kinase
Os01g0183100	Cytochrome cd1-nitrite reductase-like, C-terminal haem d1 domain containing protein.	AK111760	NP_187664.2	WD40 repeat
Os01g0183300	Endonuclease/exonuclease/phosphatase domain containing [illegible]	AK110423	B6SXL1	Inositol polyphosphate-related phosphatase
Os01g0183400	Histone-fold domain containing protein.	AK373759	B6U0K8	Transcription factor CBF/NF-Y/archaeal histone
Os01g0183500	Cytochrome P450 family protein.	AK242195	Q6RYE0	Cytochrome P450
Os01g0183600	Cytochrome P450 family protein.	AK110852	B9TST2	Cytochrome P450
Os01g0183633	Similar to predicted protein.	AK318597	NP_001172209.1	Ribosomal protein L18a
Os01g0183800	Cyclin-like F-box domain containing protein.	AK106385	NP_001182794.1	F-box domain, cyclin-like
Os01g0183850	Conserved hypothetical protein.	ab initio prediction	B8ADM3	-
Os01g0183950	Hypothetical protein.	EU942531	longestORF	-
Os01g0184000	NUC156 family protein.	AK071130	NP_974765.1	Pre-mRNA cleavage complex II Clp1
Os01g0184050	Hypothetical protein.	BT016978	longestORF	-
Os01g0184100	Similar to 17.5 kDa class II heat shock protein.	AK071240	E5D3J5	Heat shock protein Hsp20
Os01g0184200	Similar to OSIGBa0132G14.1 protein.	AK120391	Q01MX2	-
Os01g0184500	DEAD-like helicase, N-terminal domain containing protein.	AK060699	Q56X76	Helicase, C-terminal
Os01g0184700	Conserved hypothetical protein.	AK061353	A2WLG9	-
Os01g0184800	Thioredoxin fold domain containing protein.	AK073377 ,AK102544	B4FF90	Thioredoxin-like fold
Os01g0184900	Similar to SSRP1 protein.	AK068959	Q9LGR0	High mobility group, HMG1/HMG2
Os01g0185000	Hypothetical conserved gene.	ab initio prediction	Q69TJ5	-
Os01g0185100	Similar to predicted protein.	BT065044	Q9LGQ8	Protein of unknown function DUF1664
Os01g0185200	Glutaminyl-tRNA synthetase, class Ic domain containing protein.	AK065125	NP_001152305.1	Glutamyl/glutaminyl-tRNA synthetase, class Ib
Os01g0191000	Similar to H0717B12.1 protein.	EU963117	Q01JC1	-
Os01g0191100	Similar to Acidic ribosomal protein P2a-4 (Fragment).	AK058815	B6U978	Ribosomal protein 60S
Os01g0191150	Similar to profilin-5.	AK375203	NP_001105622.1	Profilin/allergen
Os01g0191200	Similar to Acid phosphatase.	AK060964 ,AK061466 ,AK10418	A6N0F9	-
Os01g0191300	Similar to NAC-type transcription factor.	AK121339	B2CPX4	No apical meristem
Os01g0191400	Hypothetical protein.	AK101179	longestORF	-
Os01g0191500	Similar to Mitochondrial processing peptidase.	AK101856 ,AK071930	NP_001150614.1	Peptidase M16, C-terminal
Os01g0191700	Similar to Pyrophosphate-fructose-6-phosphate 1-phosphotransferase-like protein (Pyrophosphate-dependent phosphofructo-1-kinase-like protein).	AK121093	B6TE72	Phosphofructokinase domain
Os01g0191800	Similar to Aurora kinase.	AK061360	Q4R1K7	Protein kinase, catalytic domain
Os01g0191900	Similar to Blind.	AK109125	Q8S3Y3	SANT domain, DNA binding
Os01g0192000	Similar to C3H transcription factor.	AK073660	B7EQW0	-
Os01g0192101	Hypothetical protein.	EU957563	longestORF	-
Os01g0192500	Similar to H0525G02.3 protein.	EU964716	Q00RR7	Heavy metal-associated domain, HMA
Os01g0192550	Conserved hypothetical protein.	AK241461	NP_001172217.1	-
Os01g0192600	Ndr family protein.	AK100731	B6TFK0	Ndr
Os01g0192700	Non-protein coding transcript.	tplb0040a08 (Wheat FLcDNA)	NONE	-
Os01g0192900	1-aminocyclopropane-1-carboxylate synthase family protein.	BT068927	O24220	1-aminocyclopropane-1-carboxylate synthase
Os01g0193250	Hypothetical gene.	EU959976	longestORF	-
Os01g0193400	Hypothetical gene.	AK287954	longestORF	-
Os01g0193500	Conserved hypothetical protein.	AK353821	NP_001042281.1	-
Os01g0193600	Methyltransferase small domain containing protein.	AK068784	NP_188767.2	23S rRNA methyltransferase/RumA
Os01g0193700	Hypothetical protein.	tplb0022j08 (Wheat FLcDNA)	longestORF	-
Os01g0193900	Hypothetical conserved gene.	AK110899	B9ETM1	-
Os01g0194000	Hypothetical conserved gene.	AK058353	A2WLM7	-
Os01g0194200	IQ calmodulin-binding region domain containing protein.	AK073282	NP_001151471.1	IQ motif, EF-hand binding site
Os01g0194300	Similar to NPR1.	AK120715	Q5D0W8	BTB/POZ-like
Os01g0194600	Thioredoxin fold domain containing protein.	AK102031 ,AK101203	Q5SMY5	Glutaredoxin
Os01g0195000	Zinc finger, C2H2-type domain containing protein.	AK105663	A2WLN2	Zinc finger, C2H2
Os01g0195066	Non-protein coding transcript.	CT834794	NONE	-
Os01g0195100	Non-protein coding transcript.	AK121095	NONE	-
Os01g0195200	Similar to Serine/threonine-protein kinase PBS1 (EC 2.7.1.37) (AvrPphB susceptible protein 1).	AK105501	C6ZRT8	Protein kinase, catalytic domain
Os01g0231000	Similar to Auxin-responsive protein (Aux/IAA) (Fragment).	AK104654	Q5NB25	AUX/IAA protein
Os01g0231500	Similar to casein kinase I isoform delta-like.	AK111602	B9EUI3	Protein kinase, catalytic domain
Os01g0231600	Pleckstrin homology-type domain containing protein.	ab initio prediction	A2WMF2	Pleckstrin homology-type
Os01g0231700	Similar to 60S acidic ribosomal protein P2-B (CaRP2B).	AK107453	NP_001147388.1	Ribosomal protein 60S
Os01g0231800	Similar to cDNA clone:J033025F17, full insert sequence.	AK062918	B7ELY0	Protein kinase A anchor protein, nuclear localisation signal domain
Os01g0231900	K Homology, type 1, subgroup domain containing protein.	AK073280	B9EUI5	K Homology
Os01g0231950	Hypothetical gene.	BT061568	longestORF	-
Os01g0232000	Major intrinsic protein family protein.	Q9LWR2 (UniProt)	Q9LWR2	Major intrinsic protein
Os01g0232100	Similar to Tonoplast membrane integral protein ZmTIP4-3.	AK069592 ,AK099190	NP_001105035.1	Major intrinsic protein
Os01g0232200	WD40 repeat domain containing protein.	EU955858	Q5NB66	WD40 repeat
Os01g0232300	Similar to TRZ3 (TRNASE Z 3)%3B 3'-tRNA processing endoribonuclease/ catalytic.	AK120694	NP_175628.2	-
Os01g0232400	Similar to VHS1 protein (Fragment).	AK106519	B8AB14	Epsin domain, N-terminal
Os01g0232450	Conserved hypothetical protein.	AK242981	NP_001172244.1	-
Os01g0232500	Similar to PFU3/UBC26 (UBIQUITIN-CONJUGATING ENZYME 26)%3B ubiquitin-protein ligase.	AK119659	A2ZR04	Ubiquitin-conjugating enzyme, E2
Os01g0232550	Hypothetical conserved gene.	EU941213	B9EUI8	-
Os01g0232700	Similar to Histidinol dehydrogenase.	AK104113 ,AK061778	A2WMG4	Histidinol dehydrogenase, conserved site
Os01g0232800	Similar to KOB1.	AK318572	NP_001151449.1	-
Os01g0233000	DREPP plasma membrane polypeptide family protein.	AK065256	A2WMG6	DREPP plasma membrane polypeptide
Os01g0233100	Similar to Cyclin-A1-2.	tplb0028l11 (Wheat FLcDNA)	Q0JPA4	Cyclin, C-terminal
Os01g0233400	Histone H4 acetyltransferase, NuA4 complex, Eaf6 domain containing protein.	CT830710 (DDBJ, Secondary hit)	NP_001042511.2	Histone H4 acetyltransferase, NuA4 complex, Eaf6
Os01g0233500	Cyclin.	AK063476	Q7F830	Cyclin, C-terminal
Os01g0233800	Similar to Viroid symptom modulation protein.	AK069983	Q41297	Protein kinase, catalytic domain
Os01g0233850	Hypothetical gene.	EU943630	longestORF	-
Os01g0233900	Similar to cDNA clone:J013003E06, full insert sequence.	AK100714	B7EAV1	Ubiquitin-conjugating enzyme, E2
Os01g0234001	Hypothetical conserved gene.	AK241763	B9G6H6	-
Os01g0234100	Transcriptional factor B3 family protein.	AK106332	Q0JP99	Transcriptional factor B3
Os01g0234200	Similar to DNA topoisomerase 2.	ab initio prediction	A2WMH7	DNA topoisomerase, type IIA, subunit B/N-terminal
Os01g0234300	Similar to Pectinesterase.	ab initio prediction	B9EUJ6	Pectinesterase, catalytic
Os01g0234433	Hypothetical protein.	EU943103	longestORF	-
Os01g0234499	Similar to Pectinesterase.	ab initio prediction	B9EUJ6	Pectinesterase, catalytic
Os01g0234566	Non-protein coding transcript.	X06283	NONE	-
Os01g0234700	Harpin-induced 1 domain containing protein.	AK108936	NP_001147348.1	-
Os01g0234800	Non-protein coding transcript.	AK063827	NONE	-
Os01g0234850	Hypothetical conserved gene.	AK242318	NP_001172247.1	Phosphatidylinositol 3-/4-kinase, catalytic
Os01g0234900	Similar to Ubiquitin ligase SINAT5 (EC 6.3.2.-) (Seven in absentia homolog 5). Splice isoform 2.	AK120816	B8Q8B9	Zinc finger, RING-type
Os01g0235100	Similar to predicted protein.	AK287587	Q5NAX5	-
Os01g0235200	Conserved hypothetical protein.	AK064013	B9EUK0	-
Os01g0235300	SOUL haem-binding protein domain containing protein.	AK102961 ,AK104591	B6SR01	SOUL haem-binding protein
Os01g0235325	Hypothetical protein.	EU943029	longestORF	-
Os01g0235350	Conserved hypothetical protein.	ab initio prediction	B9EUK1	-
Os01g0235400	Similar to Importin-alpha re-exporter (Cellular apoptosis susceptibility protein homolog).	AK105595	Q9ZPY7	CAS/CSE, C-terminal
Os01g0235500	Conserved hypothetical protein.	AK121404	A2WMJ1	-
Os01g0235632	Hypothetical protein.	tplb0028d05 (Wheat FLcDNA)	longestORF	-
Os01g0235700	Similar to BHLH transcription factor (Fragment).	AK121013	Q5IWM1	Helix-loop-helix DNA-binding
Os01g0235800	K Homology domain containing protein.	AK058731	B9EUK2	K Homology
Os01g0235850	Hypothetical protein.	EU974948	longestORF	-
Os01g0235900	Thioredoxin fold domain containing protein.	ab initio prediction	NP_001042526.1	Glutaredoxin
Os01g0236000	Conserved hypothetical protein.	AK065569	B8AB33	-
Os01g0559600	Similar to Asparaginyl endopeptidase.	AK068011	Q9SSZ4	Peptidase C13, legumain
Os01g0559750	Hypothetical gene.	AK241989	GeneMark	-
Os01g0559825	Hypothetical protein.	EU942443	longestORF	-
Os01g0559900	Non-protein coding transcript.	AK243203	NONE	-
Os01g0560000	Similar to cDNA clone:001-205-F05, full insert sequence.	AK059399	B7EYM8	Peptidase M20

Os01g0560200	Similar to Vesicle transport v-SNARE 13 (AtVTI13) (Vesicle transport v-SNARE protein VTI13) (Vesicle soluble NSF attachment protein receptor 13).	AK102003	NP_001147199.1	Vesicle transport v-SNARE, N-terminal
Os01g0658400	Ubiquitin-conjugating enzyme OsUBC5a.	AK063826 ,AK099284	B8A6V8	Ubiquitin-conjugating enzyme, E2
Os01g0658450	Non-protein coding transcript.	BT086907	NONE	-
Os01g0658500	Similar to Vacuolar protein sorting protein 25.	AK069050	B6SHU1	ESCRT-II complex, vps25 subunit
Os01g0658600	Similar to SBH2 (SPHINGOID BASE HYDROXYLASE 2)%3B catalytic/ sphingosine hydroxylase.	BT009316	NP_563944.1	-
Os01g0658700	Glycoside hydrolase-type carbohydrate-binding, subgroup domain containing protein.	AK106555	NP_001149116.1	Aldose 1-epimerase
Os01g0658800	Hypothetical protein.	tplb0033p24 (Wheat FLcDNA)	longestORF	-
Os01g0658900	OSBZ8.	AK121925	Q40645	Basic-leucine zipper
Os01g0659200	Similar to Vacuolar ATP synthase subunit E (EC 3.6.3.14) (V-ATPase E subunit) (Vacuolar proton pump E subunit).	AK071200	Q2L9B8	ATPase, V1/A1 complex, subunit E
Os01g0659400	Non-protein coding transcript.	AK064418	NONE	-
Os01g0659800	C2 calcium-dependent membrane targeting domain containing [illegible]	AK109718	NP_001152648.1	C2 calcium-dependent membrane targeting
Os01g0659900	Cyclin-like F-box domain containing protein.	AK121359	E5GCH4	F-box domain, cyclin-like
Os01g0660000	Conserved hypothetical protein.	AK064265	Q6Z593	-
Os01g0660100	Conserved hypothetical protein.	AK108487	B9FS42	-
Os01g0660200	Acidic class III chitinase OsChib3a precursor (Chitinase) (EC 3.2.1.14).	AK100973	GeneMark	Glycoside hydrolase, family 18, catalytic domain
Os01g0660300	Similar to Pyruvate kinase.	AK106457	A5C814	Pyruvate kinase
Os01g0660450	Similar to 3-5 exonuclease family protein.	BT019050	B6TC91	-
Os01g0660500	Similar to 3-5 exonuclease family protein.	FP099855	B6TC91	3'-5' exonuclease
Os01g0660550	Conserved hypothetical protein.	AK242175	NP_001055948.1	-
Os01g0660700	Protein of unknown function DUF295 family protein.	AK064155	A2WTF8	Protein of unknown function DUF295
Os01g0660800	3'-5' exonuclease domain containing protein.	AK242290	B6TKY2	3'-5' exonuclease
Os01g0660850	Non-protein coding transcript.	AK289062	NONE	-
Os01g0660900	Similar to phosphoglycerate mutase family protein.	AK330283	B6TTB7	Histidine phosphatase superfamily, clade-1
Os01g0661000	Uncharacterised protein family UPF0497, trans-membrane plant domain containing protein.	AK101958	B8A6W8	Uncharacterised protein family UPF0497, trans-membrane plant
Os01g0661200	Hypothetical protein.	ab initio prediction	NONE	-
Os01g0661400	S1, RNA binding domain containing protein.	AK073113	B6TDY7	RNA-binding domain, S1
Os01g0661500	Mov34/MPN/PAD-1 family protein.	AK068127	Q6NKP9	Mov34/MPN/PAD-1
Os01g0661601	Hypothetical gene.	AK241460	longestORF	-
Os01g0661650	Hypothetical conserved gene.	CT834939	NP_001172489.1	-
Os01g0661700	Hypothetical gene.	AK072040	longestORF	-
Os01g0661750	Hypothetical conserved gene.	ab initio prediction	NP_001172490.1	-
Os01g0662300	Similar to Isoform 2 of 50S ribosomal protein L12, chloroplastic.	AK062414	O22386-2	Ribosomal protein L7/L12, C-terminal
Os01g0662600	Non-protein coding transcript.	AK105041	NONE	-
Os01g0662700	Similar to Naphthoate synthase (EC 4.1.3.36).	AK068152	E5GBI7	Crotonase, core
Os01g0662800	Zinc finger, NHR/GATA-type domain containing protein.	GU722206	NP_001172493.1	Zinc finger, GATA-type
Os01g0663051	Similar to SANT/MYB protein.	CT834958	NP_001148292.1	SANT domain, DNA binding
Os01g0663300	Similar to (1-4)-beta-mannan endohydrolase-like protein.	AK071667	Q0JKM9-2	Glycoside hydrolase, family 5
Os01g0663400	Similar to aspartic proteinase oryzasin-1.	AK072645	NP_001148782.1	Peptidase A1
Os01g0663500	Transcriptional coactivator/pterin dehydratase family protein.	AK058452 ,AK060072	NP_001148979.1	Transcriptional coactivator/pterin dehydratase
Os01g0663800	Hypothetical conserved gene.	AK062567	B8A764	-
Os01g0664000	Similar to carboxyl-terminal-processing protease.	EU954703	NP_001147330.1	PDZ/DHR/GLGF
Os01g0664100	Mg2+ transporter protein, CorA-like/Zinc transport protein ZntB domain containing protein.	AK065234	B8A765	Mg2+ transporter protein, CorA-like/Zinc transport protein ZntB
Os01g0664200	Similar to Ser Thr specific protein kinase-like protein.	AK066072	Q1EPK4	Protein kinase, catalytic domain
Os01g0664500	Lg106-like family protein.	AK059427	B6SN23	Endosulphine
Os01g0673500	Similar to predicted protein.	AK062045	XP_001698289.1	ATPase, AAA-type, core
Os01g0673600	Similar to Ubiquitin-conjugating enzyme E2.	AK122067	C5XG33	Ubiquitin-conjugating enzyme, E2
Os01g0673650	Non-protein coding transcript.	BT016196	NONE	-
Os01g0673700	Similar to Dof3 (Fragment).	AK376848	Q0GLE9	Zinc finger, Dof-type
Os01g0673800	Similar to predicted protein.	AK120641	NP_565711.1	-
Os01g0673900	Zinc finger, RING-type domain containing protein.	AK063791	F2E206	Zinc finger, RING-type
Os01g0674000	Homeodomain-like containing protein.	AK099396	NP_001149030.1	SANT domain, DNA binding
Os01g0674100	Similar to ATP binding protein.	AK111983	NP_001152076.1	Protein kinase, catalytic domain
Os01g0674125	Hypothetical gene.	BT017362	longestORF	-
Os01g0674150	Conserved hypothetical protein.	AK241731	NP_001172499.1	-
Os01g0674400	Hypothetical conserved gene.	CT834954	NP_001172501.1	-
Os01g0674450	Non-protein coding transcript.	AK241551	NONE	-
Os01g0674500	Similar to cDNA clone:J023048A08, full insert sequence.	AK072815	B7EHT3	Tubby, C-terminal
Os01g0674700	Similar to EMB2279 (EMBRYO DEFECTIVE 2279).	AK376580	B9EYK3	Pentatricopeptide repeat
Os01g0674800	Serine/threonine-specific protein kinase NPK15-like.	AK059169	NP_193599.3	Protein kinase, catalytic domain
Os01g0675000	Rop nucleotide exchanger, PRONE domain containing protein.	AK100701	NP_001148162.1	Plant specific Rop nucleotide exchanger, PRONE
Os01g0675100	Similar to peroxiredoxin.	AK058509	Q9FR35	Thioredoxin-like fold
Os01g0675400	DNA-binding pseudobarrel domain domain containing protein.	EU944481	Q5QM34	DNA-binding pseudobarrel domain
Os01g0675500	Similar to galactosylgalactosylxylosylprotein 3-beta-glucuronosyltransferase 1.	AK062112	NP_001151473.1	Glycosyl transferase, family 43
Os01g0675700	Similar to Auxin-responsive protein IAA14 (Indoleacetic acid-induced protein 14) (SOLITARY-ROOT protein).	AK061495 ,AK102396	Q0JKG7	AUX/IAA protein
Os01g0705000	Hypothetical conserved gene.	AK243273	Q5N8G0	-
Os01g0705100	Similar to Germin-like protein.	Q94JF3 (UniProt)	Q94JF3	Germin
Os01g0705200	Late embryogenesis abundant protein repeat containing protein.	AK064074 ,AK073837	Q40709	Late embryogenesis abundant protein, LEA-3
Os01g0705300	Similar to predicted protein.	AK103903	XP_002872105.1	-
Os01g0705400	Hypothetical conserved gene.	AK336128	A2ZX08	-
Os01g0705500	Similar to fiber protein Fb11.	AK063120	NP_001151960.1	-
Os01g0705700	Similar to Transcription factor ICE1 (Inducer of CBF expression 1) (Basic helix- loop-helix protein 116) (bHLH116) [illegible]	AK064946	NP_001150726.1	Helix-loop-helix DNA-binding
Os01g0705750	Hypothetical protein.	tplb0026g06 (Wheat FLcDNA)	longestORF	-
Os01g0705800	Conserved hypothetical protein.	AK106968	Q851I8	-
Os01g0706000	Similar to RNA polymerase II transcriptional coactivator KELP.	AK070545	NP_192830.1	Transcriptional coactivator p15
Os01g0706100	Conserved hypothetical protein.	AK072799	B8A8B9	-
Os01g0706200	Cullin, N-terminal domain containing protein.	AK241711	B9EZ23	Cullin, N-terminal
Os01g0706266	Conserved hypothetical protein.	tplb0024d06 (Wheat FLcDNA)	Q8S0P4	-
Os01g0706332	Hypothetical protein.	tplb0028o22 (Wheat FLcDNA)	longestORF	-
Os01g0706400	Hypothetical conserved gene.	AK070004	B9EZ25	-
Os01g0706500	Ribosomal protein L28e domain containing protein.	CT837912	A6MZK4	Ribosomal protein L28e
Os01g0706600	Conserved hypothetical protein.	CT835009	NP_001172531.1	-
Os01g0706700	Protein of unknown function DUF590 family protein.	AK072738	NP_001170473.1	Anoctamin/TMEM 16
Os01g0706800	Similar to agglutinin.	EU971654	B6T6H0	Mannose-binding lectin
Os01g0706850	Non-protein coding transcript.	FP096644	NONE	-
Os01g0706900	Similar to Auxin amidohydrolase.	AK105646	Q5N8F2	Peptidase M20
Os01g0719300	Similar to Sulfate transporter 3.1 (AST12) (AtST1).	AK121195	Q6ZZ94	Sulphate anion transporter
Os01g0719350	Hypothetical protein.	tplb0050b20 (Wheat FLcDNA)	longestORF	-
Os01g0719400	CASC3/Barentsz eIF4AIII binding domain containing protein.	AK063516	B9EZ63	CASC3/Barentsz eIF4AIII binding
Os01g0719600	Heavy metal transport/detoxification protein domain containing protein.	AK070988	NP_001148191.1	Heavy metal-associated domain, HMA
Os01g0719700	Similar to HCF101 (HIGH-CHLOROPHYLL-FLUORESCENCE 101)%3B ATP binding.	AK360193	NP_189086.1	Domain of unknown function, DUF971
Os01g0719800	Hypothetical protein.	AK107082	longestORF	-
Os01g0719900	Similar to predicted protein.	BT069573	NP_001149280.1	Lipase, class 3
Os01g0720000	Peptidase A1 domain containing protein.	AK332688	Q8W0E9	Peptidase A1
Os01g0720200	Non-protein coding transcript.	AK058236	NONE	-
Os01g0720300	NADH-ubiquinone oxidoreductase 20 kDa subunit, mitochondrial precursor (EC 1.6.5.3) (EC 1.6.99.3) (Complex I-20KD) (CI-[illegible]	ab initio prediction	B6TDQ5	NADH:ubiquinone oxidoreductase-like, 20kDa subunit
Os01g0720400	HAD-superfamily subfamily IB hydrolase, hypothetical 1 [illegible]	AK061237	Q06C47	HAD-superfamily hydrolase, subfamily IB, PSPase-like
Os01g0720500	Non-protein coding transcript.	AK060524	NONE	-
Os01g0720600	Similar to starch synthase IV.	AK066808	D0TZV4	Glycosyl transferase, family 1
Os01g0720700	Similar to satase isoform I.	CT830895	NP_001105082.1	-
Os01g0720800	Hypothetical conserved gene.	AK287755	NP_001172542.1	NB-ARC
Os01g0720801	Hypothetical gene.	AK240850	longestORF	-
Os01g0720900	Hypothetical conserved gene.	ab initio prediction	A2ZXB8	NB-ARC
Os01g0721000	NB-ARC domain containing protein.	AK105712	A2WUK0	NB-ARC
Os01g0721100	Conserved hypothetical protein.	AK065291	NP_001044097.1	-
Os01g0721200	Similar to cDNA clone:J013002N02, full insert sequence.	ab initio prediction	B7EAQ6	NB-ARC
Os01g0721300	Similar to cDNA clone:J013002N02, full insert sequence.	ab initio prediction	B7EAQ6	NB-ARC
Os01g0721400	NB-ARC domain containing protein.	AK063472	B7EAQ6	NB-ARC
Os01g0721500	NB-ARC domain containing protein.	AK241666	B7EAQ6	NB-ARC
Os01g0721700	Hypothetical genes.	ab initio prediction	NONE	-
Os01g0721800	Protein kinase-like domain containing protein.	AK120924	B7EAQ6	-

Os01g0721900	Similar to plasminogen activator inhibitor 1 RNA-binding protein.	AK059581	NP_001148933.1	-
Os01g0722100	Bacterial transferase hexapeptide repeat domain containing [illegible]	AK066657	NP_194683.2	Bacterial transferase hexapeptide repeat
Os01g0722300	Myb transcription factor domain containing protein.	AK241370	Q8S3Z0	SANT domain, DNA binding
Os01g0722425	Hypothetical protein.	EU971393	longestORF	-
Os01g0722550	Similar to HAT family dimerisation domain containing protein.	AK122139 (DDBJ, Secondary hit)	Q2QPA8	Zinc finger, BED-type predicted
Os01g0722700	Similar to Hexokinase.	AK069917	Q2KNB7	Hexokinase
Os01g0722800	Dimethylmenaquinone methyltransferase family protein.	CT828155	D6C5C5	Ribonuclease E inhibitor RraA/Dimethylmenaquinone methyltransferase
Os01g0813800	Similar to Beta-glucosidase 3.	AK067934	Q8RZL1	Glycoside hydrolase, family 1
Os01g0813900	Similar to ZIGA1 protein (Fragment).	AK101729	A2WWA8	Arf GTPase activating protein
Os01g0814000	Nuclear fragile X mental retardation-interacting protein 1, conserved domain domain containing protein.	AK073886	B8AB98	Nuclear fragile X mental retardation-interacting protein 1, conserved domain
Os01g0814100	Similar to Bindin (Fragment).	AK061173 ,AK059376	B6TD92	Plant lipid transfer protein/seed storage/trypsin-alpha amylase inhibitor
Os01g0814150	Conserved hypothetical protein.	CT834420	NP_001172620.1	-
Os01g0814200	Hypothetical conserved gene.	AK070675	Q5N766	-
Os01g0814300	Pentatricopeptide repeat domain containing protein.	AK103158	E6NUE8	Pentatricopeptide repeat
Os01g0814400	Similar to 91A protein (Fragment).	AK061117	B8ABA0	-
Os01g0814550	Non-protein coding transcript.	BT086943	NONE	-
Os01g0814700	Cyclin-like F-box domain containing protein.	AK068533	NP_001147428.1	F-box domain, Skp2-like
Os01g0814800	Similar to Cysteine synthase, chloroplast precursor (EC 2.5.1.47) (O-acetylserine sulfhydrylase) (O-acetylserine (Thiol)-lyase) (CSase B) (CS-B) (OAS-TL B).	AK065664	B8ABA1	Cysteine synthase/cystathionine beta-synthase P-phosphate-binding site
Os01g0814900	Similar to Cytochrome b5 reductase.	AK058594 ,AK071859 ,AK10396[illegible]	Q9ZPN0	Oxidoreductase FAD/NAD
Os01g0815100	Hypothetical conserved gene.	EU970732	XP_002884292.1	Ribosomal protein L34Ae
Os01g0815400	Conserved hypothetical protein.	AK121388	A2WWB7	-
Os01g0815700	Zinc finger, RanBP2-type domain containing protein.	AK073519	B8ABA2	Zinc finger, RanBP2-type
Os01g0815800	Non-protein coding transcript.	AK058706	NONE	-
Os01g0815850	Hypothetical conserved gene.	BT065526	C0HHH7	-
Os01g0815900	Hypothetical conserved gene.	AK241656	A2ZYY7	Pentatricopeptide repeat
Os01g0816000	Protein prenyltransferase domain containing protein.	EU951349	XP_002871645.1	Pentatricopeptide repeat
Os01g0816100	Similar to NAC domain protein.	AK073848	Q52QH4	No apical meristem
Os01g0816400	Similar to Microtubule bundling polypeptide TMBP200.	AK121331	Q5N749	Armadillo-like helical
Os01g0816450	Non-protein coding transcript.	BT085435	NONE	-
Os01g0816500	Similar to microtubule organization protein.	AK242550	Q5N749	Armadillo-like helical
Os01g0816525	Non-protein coding transcript.	BT085435	NONE	-
Os01g0816550	Non-protein coding transcript.	AK242081	NONE	-
Os01g0816600	Similar to predicted protein.	AK243409	C5DB47	Protein kinase, catalytic domain
Os01g0816700	Similar to L-ascorbate oxidase homolog precursor (EC 1.10.3.3) (Ascorbase).	AK100654	E0X9N2	Multicopper oxidase, type 1
Os01g0816850	Hypothetical protein.	tplb0021n10 (Wheat FLcDNA)	longestORF	-
Os01g0818000	Auxin efflux carrier domain containing protein.	AK070194	XP_002870888.1	Auxin efflux carrier
Os01g0818100	Hypothetical conserved gene.	CT830790	Q5QMK9	C2 calcium-dependent membrane targeting
Os01g0818200	Hypothetical gene.	AK103555	longestORF	-
Os01g0818300	K Homology domain containing protein.	AK063274	NP_001148784.1	K Homology
Os01g0818400	Homeodomain-like containing protein.	AK068585	Q5QMM3	Homeobox
Os01g0818600	Similar to cDNA clone:J013021N20, full insert sequence.	AK066883	B7EP37	Protein kinase, catalytic domain
Os01g0818650	Non-protein coding transcript.	AK368632	NONE	-
Os01g0818700	Leucine-rich repeat, N-terminal domain containing protein.	AK066550	B9ETW6	Leucine-rich repeat
Os01g0818800	Non-protein coding transcript.	AK106940	NONE	-
Os01g0818900	Conserved hypothetical protein.	AK063195	B8ABB1	-
Os01g0819000	Similar to transposon protein CACTA, En/Spm sub-class.	AK073866	NP_001147994.1	-
Os01g0819100	Similar to predicted protein.	AK242625	C0LGW1	Protein kinase, catalytic domain
Os01g0819200	Similar to calcium lipid binding protein-like.	ab initio prediction	NP_001152047.1	C2 calcium-dependent membrane targeting
Os01g0819233	Hypothetical gene.	AK288527	longestORF	-
Os01g0819266	Non-protein coding transcript.	CT835003	NONE"	-
Os01g0819300	Similar to calcium lipid binding protein-like.	AK107799	NP_001152047.1	-
Os01g0819400	Similar to Ubiquitin carrier protein.	FP101418	B8ABB5	Ubiquitin-conjugating enzyme, E2
Os01g0819433	Non-protein coding transcript.	BT086907	NONE	-
Os01g0835800	Hypothetical conserved gene.	AK288705	Q5QMF3	KIP1-like
Os01g0835900	Similar to Histone H4.	CU405994	XP_002310853.1	Histone H4
Os01g0836400	SAC3/GANP family protein.	AK073540	B8ABQ2	SAC3/GANP/Nin1/mts3/eIF-3 p25
Os01g0836600	ABC transporter-like domain containing protein.	AK067556	NP_181467.1	ABC transporter-like
Os01g0836700	Hypothetical conserved gene.	AK354161	Q5QME9	Transmembrane receptor, eukaryota
Os01g0836800	Transmembrane receptor, eukaryota domain containing protein.	AK061720 ,AK106145	NP_001150692.1	Transmembrane receptor, eukaryota
Os01g0836900	Conserved hypothetical protein.	AK064231	A2WWR9	-
Os01g0837000	Ankyrin repeat containing protein.	AK243678	A2CIR5	Ankyrin repeat
Os01g0837100	Similar to predicted protein.	BT018120	B6TJY9	Pectate lyase/Amb allergen
Os01g0837200	Similar to esterase/lipase/thioesterase family protein.	AK062368	NP_191078.1	-
Os01g0837300	Similar to UDP-glucuronic acid decarboxylase 1.	AK104675	NP_001151221.1	NAD-dependent epimerase/dehydratase
Os01g0837350	Hypothetical protein.	tplb0036f05 (Wheat FLcDNA)	longestORF	-
Os01g0837500	Similar to DNA helicase, TBP-interacting protein.	AK064668	B7FAF4	TIP49, C-terminal
Os01g0837600	Similar to plant-specific domain TIGR01589 family protein.	AK108007	NP_001148858.1	Conserved hypothetical protein CHP01589, plant
Os01g0837700	Hypothetical genes.	ab initio prediction	Q943N0	-
Os01g0837800	Similar to metal tolerance protein.	AK061539	Q5NA18	Cation efflux protein
Os01g0837900	Non-protein coding transcript.	AK106580	NONE	-
Os01g0838100	Conserved hypothetical protein.	AK065393	Q7XS07	-
Os01g0867600	Similar to UDP-glucose:sterol glucosyltransferase (EC [illegible]	AK102226	B6SKE1	UDP-glucuronosyl/UDP-glucosyltransferase
Os01g0867700	Yip1 domain containing protein.	AK108852	B6TPW7	Yip1 domain
Os01g0867800	Similar to RBP50.	AK062260	D8L9M8	RNA recognition motif domain
Os01g0867900	Similar to COV1-like protein.	AK121441	B4FES4	Protein of unknown function DUF502
Os01g0868000	Similar to F-box domain containing protein, expressed.	ab initio prediction	D8L9N0	Pathogenesis-related transcriptional factor/ERF, DNA-binding
Os01g0868200	Zinc finger, DHHC-type domain containing protein.	AK119393	B6TJT8	Zinc finger, DHHC-type, palmitoyltransferase
Os01g0868300	Similar to DNA polymerase alpha catalytic subunit (EC 2.7.7.7).	AK288311	B8A6Y4	DNA-directed DNA polymerase, family B, pol2
Os01g0868301	Hypothetical protein.	tplb0058b01 (Wheat FLcDNA)	longestORF	-
Os01g0868400	Hypothetical protein.	AK103345	longestORF	-
Os01g0868550	Hypothetical protein.	AK360759	longestORF	-
Os01g0868600	Hypothetical conserved gene.	AK289227	Q8S1N5	Peptidase A1
Os01g0868800	Similar to Subtilase.	ab initio prediction	Q84TR6	Peptidase S8/S53, subtilisin/kexin/sedolisin
Os01g0868900	Similar to predicted protein.	AK062271	C7E4J6	Peptidase S8/S53, subtilisin/kexin/sedolisin
Os01g0869000	Protein of unknown function DUF639 domain containing protein.	AK068777	XP_002458831.1	Protein of unknown function DUF639
Os01g0869200	Mg2+ transporter protein, CorA-like domain containing protein.	AK073453	Q8S1N1	-
Os01g0869300	Conserved hypothetical protein.	AK059347	B9EUV6	-
Os01g0869400	Similar to Anthocyanidin 5,3-O-glucosyltransferase.	AK063342	B4FG90	UDP-glucuronosyl/UDP-glucosyltransferase
Os01g0869450	Hypothetical protein.	EU964458	longestORF	-
Os01g0869500	Plant nuclear matrix 1 family protein.	AK106718	Q94G01	Plant nuclear matrix 1
Os01g0869550	Conserved hypothetical protein.	BT017027	A2WXD6	-
Os01g0869600	TRAM, LAG1 and CLN8 homology domain containing protein.	AK060596 ,AK070678	B8A6Z0	TRAM/LAG1/CLN8 homology domain
Os01g0869750	Conserved hypothetical protein.	AK288119	NP_001172656.1	-
Os01g0869800	Similar to Photosystem II subunit PsbS.	AK058284 ,AK071827 ,AK10427[illegible]	E0WBG6	Chlorophyll A-B binding protein
Os01g0892500	Similar to carboxylic ester hydrolase.	AK243057	NP_001151408.1	Pectinacetylesterase
Os01g0892600	Pectinacetylesterase family protein.	AK104609 ,AK064882	NP_001151408.1	-
Os01g0892800	Integrin-linked protein kinase domain containing protein.	AK100268	Q8SAE5	Protein kinase, catalytic domain
Os01g0893300	Non-protein coding transcript.	AK063187	NONE	-
Os01g0893400	Zinc finger, TAZ-type domain containing protein.	AK061917	B8A2U0	Zinc finger, TAZ-type
Os01g0893700	DOMON domain domain containing protein.	ab initio prediction	Q7F451	DOMON domain
Os01g0894000	Similar to predicted protein.	AK121960	NP_566200.1	Metallophosphoesterase domain
Os01g0894050	Myb/SANT-like domain domain containing protein.	BT039927	NP_001176912.1	Myb/SANT-like domain
Os01g0894075	Hypothetical protein.	tplb0043a14 (Wheat FLcDNA)	longestORF	-
Os01g0894100	Similar to Transposase (Fragment).	AK111398	NP_001176912.1	-
Os01g0894300	Fructokinase (Fragment).	AK120887	Q0JGZ6-2	Carbohydrate/puine kinase, PfkB, conserved site
Os01g0894500	Sep15/SelM redox domain containing protein.	AK103300	B6T652	Thioredoxin-like fold
Os01g0894600	Hypothetical conserved gene.	AK059715	A3A0G9	-
Os01g0894700	Trigger factor, ribosome-binding, bacterial domain containing [illegible]	AK067623 ,AK104876	B8A7T9	Trigger factor, ribosome-binding, bacterial
Os01g0895000	Hypothetical protein.	AK107053	longestORF	-
Os01g0895100	Similar to Membrane-associated 30 kDa protein, chloroplast precursor (M30).	AK067698 ,AK099166	B6T6V3	PspA/IM30
Os01g0895200	DOMON related domain containing protein.	AK063282	A3A0H2	Cytochrome b561, eukaryote
Os01g0895300	Cytochrome b561, eukaryote domain containing protein.	AK073544	A2WXW5	Cytochrome b561, eukaryote
Os01g0895500	Peptidase S54, rhomboid domain containing protein.	AK063649	E5GC88	Peptidase S54, rhomboid domain
Os01g0914000	Conserved hypothetical protein.	AK101364	A3A0V2	-
Os01g0914100	Plant lipid transfer protein/seed storage/trypsin-alpha amylase inhibitor domain containing protein.	ab initio prediction	NP_001045177.1	Plant lipid transfer protein/hydrophobic protein, helical domain
Os01g0914200	Hypothetical protein.	AK106995	longestORF	-
Os01g0914300	Plant lipid transfer protein/seed storage/trypsin-alpha amylase inhibitor domain containing protein.	AK105463 ,AK103489	B8A848	Plant lipid transfer protein/seed storage/trypsin-alpha amylase inhibitor

Os01g0914400	Similar to EPIDERMAL PATTERNING FACTOR-like protein [illegible]	AK063138	Q9SV72	-
Os01g0914600	Pentatricopeptide repeat domain containing protein.	AK106012	B8A850	Pentatricopeptide repeat
Os01g0914700	Peptidase A22B, signal peptide peptidase domain containing [illegible]	AK069261	B8A851	Protease-associated domain, PA
Os01g0914800	Protein of unknown function DUF231, plant domain containing protein.	AK102791	XP_002865870.1	Domain of unknown function DUF231, plant
Os01g0915000	Protein of unknown function DUF506, plant family protein.	AK107951	A2WYA2	Protein of unknown function DUF506, plant
Os01g0915200	Similar to cysteine proteinase inhibitor B.	AK121863	NP_001146946.1	Proteinase inhibitor I25, cystatin
Os01g0915300	Non-protein coding transcript.	AK119435	NONE	-
Os01g0915350	Hypothetical protein.	tplb0021a04 (Wheat FLcDNA)	longestORF	-
Os01g0915400	Similar to SEC (SECRET AGENT)%3B transferase, transferring glycosyl groups.	CT834784	NP_187074.1	-
Os01g0915401	Proteinase inhibitor I25, cystatin domain containing protein.	Q0JGM8 (UniProt)	Q0JGM8	Proteinase inhibitor I25, cystatin
Os01g0915600	Similar to TA1 protein (Fragment).	AK101063	Q70KS8	Helix-loop-helix DNA-binding
Os01g0915666	Hypothetical protein.	tplb0045a15 (Wheat FLcDNA)	longestORF	-
Os01g0915732	Hypothetical protein.	EU973377	longestORF	-
Os01g0915800	Similar to FK506-binding protein 2-2 precursor (EC 5.2.1.8) (Peptidyl-prolyl cis- trans isomerase) (PPIase) (Rotamase) (15 kDa FKBP) (FKBP-15-2).	AK103859	NP_001152120.1	Peptidyl-prolyl cis-trans isomerase, FKBP-type, domain
Os01g0915900	Conserved hypothetical protein.	AK243101	NP_001172710.1	-
Os01g0916000	Conserved hypothetical protein.	AK106300	A2WYA9	-
Os01g0916100	Similar to loricrin.	AK109173	NP_001147500.1	-
Os01g0916200	Clathrin/coatomer adaptor, adaptin-like, N-terminal domain containing protein.	AK101112	A3A0W6	Clathrin/coatomer adaptor, adaptin-like, N-terminal
Os01g0916300	Similar to protein binding protein.	AK121061 ,AK103107	NP_001147059.1	WW/Rsp5/WWP
Os01g0916350	Hypothetical gene.	AK288280	longestORF	-
Os01g0916400	Similar to Selenium binding protein.	AK071710	B6TFC2	Selenium-binding protein
Os01g0916600	RNA recognition motif, glycine rich protein domain containing protein.	AK121668 ,AK059446	O24188	RNA recognition motif domain
Os01g0916700	Conserved hypothetical protein.	AK288011	NP_001045196.1	-
Os01g0916800	Similar to predicted protein.	ab initio prediction	NP_001045197.2	THO complex, subunitTHOC2, C-terminal
Os01g0916950	Hypothetical gene.	tplb0014o19 (Wheat FLcDNA)	longestORF	-
Os01g0917100	Protein of unknown function DUF1675 domain containing [illegible]	AK107234	A2WYC7	Protein of unknown function DUF1675
Os01g0917200	Peptidase, trypsin-like serine and cysteine proteases domain containing protein.	AK288071	Q5JLC5	-
Os01g0917300	Similar to Cysteine-rich peptide.	AK240939	D7NXT9	-
Os01g0917400	Hypothetical conserved gene.	AK111958	B9EVV7	Zinc finger, CCCH-type
Os01g0921000	Nucleotide-diphospho-sugar transferase domain containing [illegible]	AK071688	B8A8F3	Nucleotide-diphospho-sugar transferase
Os01g0921100	Hypothetical conserved gene.	AK243195	B8A8F4	Nucleotide-diphospho-sugar transferase
Os01g0921200	Similar to predicted protein.	AK289185	NP_176916.2	Glycoside hydrolase, family 63
Os01g0921300	Exostosin-like family protein.	AK119614 ,AK106015	NP_001152546.1	Exostosin-like
Os01g0921400	Conserved hypothetical protein.	AK099844	Q8RZJ0	-
Os01g0921450	Hypothetical protein.	AK374499	longestORF	-
Os01g0921500	Similar to SNF1-related protein kinase regulatory gamma subunit 1 (AKIN gamma1) (AKING1).	AK105517	Q01LJ2	Cystathionine beta-synthase, core
Os01g0921550	Hypothetical protein.	ab initio prediction	NONE	-
Os01g0921600	Similar to Mitochondrial import receptor subunit TOM20 (Translocase of outer membrane 20 kDa subunit).	AK071344	NP_001149254.1	Plant specific mitochondrial import receptor subunit TOM20
Os01g0921800	Tetratricopeptide-like helical domain containing protein.	AK334012	XP_002887115.1	Zinc finger, MYND-type
Os01g0922000	Hypothetical protein.	AK073034	longestORF	-
Os01g0922100	Conserved hypothetical protein.	AK110737	A2WYH3	-
Os01g0922350	Hypothetical protein.	AK373674	longestORF	-
Os01g0922600	Similar to SBP-domain protein 4.	AK062581	Q0JGI1	Transcription factor, SBP-box
Os01g0922700	Conserved hypothetical protein.	AK287528	NP_001172712.1	-
Os01g0922800	Similar to MADS-box protein PTM5.	AK066160	Q198I2	Transcription factor, MADS-box
Os01g0926200	Similar to RING-H2 finger protein RHF1a (Fragment).	AK066457	B4FYL2	Zinc finger, RING-type
Os01g0926300	Similar to Transaldolase (EC 2.2.1.2).	AK105800 ,AK067632 ,AK07270	B4FRC9	-
Os01g0926350	Hypothetical gene.	BT019303	longestORF	-
Os01g0926400	Similar to Pectin-glucuronyltransferase.	AK063916	B6U2C1	Exostosin-like
Os01g0926450	Hypothetical protein.	AK241690	longestORF	-
Os01g0926501	Non-protein coding transcript.	X02683	NONE	-
Os01g0926600	Similar to Pectin-glucuronyltransferase.	AK068465	E0ZPV1	Exostosin-like
Os01g0926700	Similar to secondary cell wall-related glycosyltransferase family [illegible]	CU405596	NP_001150738.1	Exostosin-like
Os01g0926800	Cellular retinaldehyde-binding/triple function, N-terminal domain containing protein.	AK121373	O24659	Cellular retinaldehyde-binding/triple function, C-terminal
Os01g0927000	Similar to SET domain-containing protein SET118.	AK106700	Q8L821	SET domain
Os01g0927300	Heavy metal transport/detoxification protein domain containing protein.	AK073201	NP_565819.1	Heavy metal-associated domain, HMA
Os01g0927400	Molecular chaperone, heat shock protein, Hsp40, DnaJ domain containing protein.	AK242854	NP_001045268.2	Domain of unknown function DUF3444
Os01g0927500	Serine/threonine protein kinase-related domain containing protein.	AK101312	NP_001151856.1	Protein kinase, catalytic domain
Os01g0927600	Similar to Auxin response factor 2 (ARF1-binding protein) (ARF1-BP).	AK072309	Q5JK20	AUX/IAA protein
Os01g0927900	Similar to Aspartate kinase precursor (EC 2.7.2.4).	AK073189	B8A8I6	Aspartate/glutamate/uridylate kinase
Os01g0927950	Hypothetical gene.	BT068036	longestORF	-
Os01g0928000	Similar to Transcription factor ICE1 (Inducer of CBF expression 1) (Basic helix- loop-helix protein 116) (bHLH116) [illegible]	AK102594	B6UB60	Helix-loop-helix DNA-binding
Os01g0928100	Similar to expressed protein.	AK120714	XP_002869690.1	-
Os01g0928200	Conserved hypothetical protein.	CT834799	B6TUM4	-
Os01g0928300	Prefoldin domain containing protein.	AK068358	B9EW06	-
Os01g0928400	Conserved hypothetical protein.	AK111454	B8A8V0	-
Os01g0928600	Serine palmitoyltransferase.	AK241034	B6SMZ5	Aminotransferase, class-II, pyridoxal-phosphate binding site
Os01g0928700	Similar to Serine palmitoyltransferase.	ab initio prediction	B6SMZ5	Aminotransferase, class-II, pyridoxal-phosphate binding site
Os01g0928800	Similar to Serine palmitoyltransferase.	AK120617	B6SMZ5	Aminotransferase, class-II, pyridoxal-phosphate binding site
Os01g0929000	Similar to aminopeptidase.	AK073334	NP_196898.5	Protein of unknown function DUF3754
Os01g0929100	Similar to predicted protein.	AK060605	Q5JK31	-
Os01g0929200	Serine/threonine protein kinase domain containing protein.	AK242279	C6ZRX2	Protein kinase, catalytic domain
Os01g0929500	Similar to Carbonyl reductase-like protein.	AK111399	E6NU09	Short-chain dehydrogenase/reductase SDR
Os01g0929600	Similar to Anther specific.	AK070978	Q40629	-
Os01g0929800	Hypothetical gene.	CT837487	GeneMark	-
Os01g0942900	Similar to cDNA clone:J023028M23, full insert sequence.	AK243304	B7E3R6	Ankyrin repeat
Os01g0943000	Hypothetical conserved gene.	AK240931	Q94CS2	-
Os01g0943100	Conserved hypothetical protein.	EU956243	A2ZPV3	-
Os01g0943400	Hypothetical protein.	ab initio prediction	NONE	-
Os01g0943700	Conserved hypothetical protein.	AK066782	Q8H4Z9	-
Os01g0943800	Similar to cDNA clone:001-029-B04, full insert sequence.	AK069731	B7E3R6	Ankyrin repeat
Os01g0943900	Hypothetical conserved gene.	AK240931	Q94CS2	-
Os01g0943950	Hypothetical conserved gene.	ab initio prediction	XP_002885094.1	-
Os01g0944000	Conserved hypothetical protein.	AK120633	Q94CR7	-
Os01g0944100	Conserved hypothetical protein.	AK103580	NP_001173817.1	-
Os02g0130600	Conserved hypothetical protein.	AK120974	B8AH13	-
Os02g0130700	Hypothetical genes.	ab initio prediction	NP_001172785.1	-
Os02g0130800	F-box domain, cyclin-like domain containing protein.	EU975717	B9F2A0	F-box domain, cyclin-like
Os02g0130900	Ubiquitin-conjugating enzyme E2C-binding protein domain containing protein.	AK102856	A2X0I7	Ubiquitin-conjugating enzyme E2C-binding protein
Os02g0131000	Conserved hypothetical protein.	AK062905	A2X0I8	-
Os02g0131050	Hypothetical gene.	AK242812	longestORF	-
Os02g0131100	Conserved hypothetical protein.	AK060381	B9F2A1	-
Os02g0131200	Similar to RNA-binding region RNP-1 (RNA recognition motif).	AK069931	A3A2T3	Glycosyl transferase, family 3
Os02g0131300	Non-protein coding transcript.	AK061919	NONE	-
Os02g0131400	Beta-D-glucan exohydrolase, isoenzyme ExoII (EC 3.2.1.58).	AK363981	NP_001130296.1	Glycoside hydrolase, family 3, N-terminal
Os02g0131500	Hypothetical protein.	tplb0060e05 (Wheat FLcDNA)	longestORF	-
Os02g0131600	Similar to Mitochondrial import receptor subunit TOM22 homolog (Translocase of outer membrane 22 kDa subunit [illegible]	CT828266	B6U2X6	Mitochondrial outer membrane translocase complex, subunit Tom22, plant
Os02g0131700	Similar to RNA binding protein.	CT830787	B6T5S5	RNA recognition motif domain
Os02g0131800	Similar to Root-specific metal transporter.	AK102180	Q6ZG85	Natural resistance-associated macrophage protein
Os02g0131850	Hypothetical gene.	BT086902	longestORF	-
Os02g0132100	Pentatricopeptide repeat domain containing protein.	ab initio prediction	NP_001045795.1	Pentatricopeptide repeat
Os02g0132200	Conserved hypothetical protein.	AK370222	NP_001045796.1	-
Os02g0132300	Similar to Erythrocyte membrane protein PFEMP3 (Fragment).	AK103414	NP_001151550.1	Zinc finger, RING-CH-type
Os02g0164800	Zinc finger, C2H2-type domain containing protein.	AK062817	NP_175482.1	-
Os02g0164900	Similar to Auxin response factor 3.	AK066725	Q6H6V4	AUX/IAA protein
Os02g0165000	Zinc finger, RING/FYVE/PHD-type domain containing protein.	AK101610	B8AI80	Zinc finger, RING-CH-type
Os02g0165100	Protein kinase, core domain containing protein.	AK066645	NP_001148145.1	Protein kinase, catalytic domain
Os02g0165200	Hypothetical protein.	AK101881	longestORF	-

Os02g0165500	Conserved hypothetical protein.	AK060547	A2X1A6	-
Os02g0165800	Conserved hypothetical protein.	AK106983	A2X1A7	-
Os02g0166200	Non-protein coding transcript.	AK066189	NONE	-
Os02g0166501	Hypothetical conserved gene.	CT835482	Q6H4W8	-
Os02g0166600	Hypothetical protein.	AK069371	longestORF	-
Os02g0166800	Protein of unknown function DUF640 domain containing protein.	AK106854	D1MYE3	Domain of unknown function DUF640
Os02g0166875	Hypothetical protein.	EU970153	longestORF	-
Os02g0166950	Hypothetical gene.	AK243419	longestORF	-
Os02g0167000	Similar to holocarboxylase synthetase 1.	AK375247	XP_002878877.1	Biotin protein ligase, C-terminal
Os02g0167100	Similar to 3-mercaptopyruvate sulfurtransferase precursor (EC 2.8.1.1).	AK066425	Q94C43	Thiosulphate sulfurtransferase, conserved site
Os02g0167200	Pentatricopeptide repeat domain containing protein.	AK106491	Q6H4W1	Pentatricopeptide repeat
Os02g0167300	Similar to Tubulin beta-5 chain.	AK061659 ,AK119164	P46265	Tubulin
Os02g0167366	Hypothetical protein.	tplb0045b10 (Wheat FLcDNA)	longestORF	-
Os02g0167400	Non-protein coding transcript.	X06283	NONE	-
Os02g0167500	RNA recognition motif domain domain containing protein.	AK069161	B9F385	RNA recognition motif domain
Os02g0167600	Hypothetical gene.	AK242059	longestORF	-
Os02g0167700	Armadillo-like helical domain containing protein.	AK069128	NP_001030954.1	Armadillo-like helical
Os02g0167850	Non-protein coding transcript.	CT835346	NONE"	-
Os02g0168000	Similar to UreF.	AK106654	E0ZS46	Urease accessory protein UreF
Os02g0168100	Similar to 4-hydroxyphenylpyruvate dioxygenase.	AK058890	O48604	Glyoxalase/fosfomycin resistance/dioxygenase
Os02g0168200	Similar to Transfactor-like protein.	AK108211	NP_001147627.1	Myb-like DNA-binding domain, SHAQKYF class
Os02g0168250	Hypothetical protein.	tplb0050a13 (Wheat FLcDNA)	longestORF	-
Os02g0168300	Conserved hypothetical protein.	AK103301	B8AIH7	-
Os02g0168400	Hypothetical conserved gene.	AK241031	A3A3J4	Armadillo-like helical
Os02g0168500	Similar to Protein-O-fucosyltransferase 1.	AK073338	Q659R8	GDP-fucose protein O-fucosyltransferase
Os02g0168550	Non-protein coding transcript.	tplb0046d03 (Wheat FLcDNA)	NONE	-
Os02g0168600	Ovarian tumour, otubain domain containing protein.	AK067291	B6U3W2	Ovarian tumour, otubain
Os02g0168700	Peptidyl-prolyl cis-trans isomerase, FKBP-type domain containing protein.	AK061562	NP_001148684.1	Peptidyl-prolyl cis-trans isomerase, FKBP-type, domain
Os02g0168800	Similar to Porphobilinogen deaminase, chloroplastic.	AK102265	Q6H6D2	Tetrapyrrole biosynthesis, hydroxymethylbilane synthase
Os02g0168850	Hypothetical gene.	Y12809	longestORF	-
Os02g0168900	Ribonuclease H2, subunit C domain containing protein.	AK102098	A2X1D0	Ribonuclease H2, subunit C
Os02g0169000	Similar to T6H22.2 protein.	AK101628	Q9SGU5	-
Os02g0169151	Hypothetical gene.	BT016355	longestORF	-
Os02g0169300	Similar to Phosphoglycerate kinase, cytosolic (EC 2.7.2.3).	AK070041	B8AIH2	Phosphoglycerate kinase
Os02g0224100	Similar to catalytic/ protein phosphatase type 2C.	AK068836	NP_001151594.1	Protein phosphatase 2C-like
Os02g0224200	Similar to Ser/Thr specific protein phosphatase 2A B regulatory subunit beta isoform.	AK101100	A2X2K3	Protein phosphatase 2A, regulatory subunit PR55
Os02g0224300	Hypothetical conserved gene.	AK119200	B9F4C9	-
Os02g0224400	Similar to Xaa-Pro dipeptidase (EC 3.4.13.9) (X-Pro dipeptidase) (Proline dipeptidase) (Prolidase) (Imidodipeptidase)	AK064268	B6TL64	Peptidase M24, structural domain
Os02g0224800	Similar to predicted protein.	AK062204	B6TFK0	Ndr
Os02g0224850	Non-protein coding transcript.	tplb0040g21 (Wheat FLcDNA)	NONE	-
Os02g0224900	Similar to ATPP2-A13.	AK101829	NP_001149956.1	-
Os02g0225000	Similar to cDNA clone:0014-E12, full insert sequence.	AK241075	B7EBN1	Mitochondrial substrate/solute carrier
Os02g0225100	SKP1 component domain containing protein.	FP094482	Q6Z8A9	SKP1 component
Os02g0559400	Similar to 12-oxo-phytodienoic acid reductase5.	AK059887	NP_001105909.1	NADH:flavin oxidoreductase/NADH oxidase, N-terminal
Os02g0559500	Conserved hypothetical protein.	CT837652	Q6YU31	-
Os02g0559800	E3 ubiquitin ligase EL5 (EC 6.3.2.-).	AK243670	Q9LRB7	Zinc finger, RING-type
Os02g0559900	Conserved hypothetical protein.	CT837652	Q6YU31	-
Os02g0559925	Hypothetical protein.	EU947281	longestORF	-
Os02g0560200	E3 ubiquitin ligase EL5 (EC 6.3.2.-).	AK243670	Q9LRB7	Zinc finger, RING-type
Os02g0560300	Conserved hypothetical protein.	CT837652	Q6YU31	-
Os02g0560450	Cytochrome c oxidase biogenesis protein Cmc1-like domain containing protein.	CT836531	B8BJN1	Cytochrome c oxidase biogenesis protein Cmc1-like
Os02g0560600	E3 ubiquitin ligase EL5 (EC 6.3.2.-).	AK243670	Q9LRB7	Zinc finger, RING-type
Os02g0560700	Conserved hypothetical protein.	CT837652	Q6YU31	-
Os02g0561000	E3 ubiquitin ligase EL5 (EC 6.3.2.-).	AK243670	Q9LRB7	Zinc finger, RING-type
Os02g0561100	Conserved hypothetical protein.	CT837652	Q6YU31	-
Os02g0561400	E3 ubiquitin ligase EL5 (EC 6.3.2.-).	AK243670	Q9LRB7	Zinc finger, RING-type
Os02g0561500	Monooxygenase, FAD-binding domain containing protein.	AK367305	Q6YU31	Monooxygenase, FAD-binding
Os02g0561800	E3 ubiquitin ligase EL5 (EC 6.3.2.-).	AK243670	Q9LRB7	Zinc finger, RING-type
Os02g0561900	Similar to E3 ubiquitin-protein ligase EL5.	BT040259	Q9LRB7	Zinc finger, RING-type
Os02g0562000	Hypothetical protein.	AK375340	longestORF	-
Os02g0562100	DNA repair and recombination, RecA-like domain containing [illegible]	AK332394	NP_001047150.1	DNA repair protein RadA
Os02g0562300	Hypothetical conserved gene.	AK104737 ,AK061441	B8ADY6	Calmodulin binding protein-like
Os02g0562350	Non-protein coding transcript.	EU949450	NONE	-
Os02g0562400	Ankyrin repeat containing protein.	AK106570	Q9M1Y3	Ankyrin repeat-containing domain
Os02g0562600	Similar to Seven trans-membrane protein (Fragment).	AK121163	E2RZR9	Mlo-related protein
Os02g0562700	Similar to Gamma hydroxybutyrate dehydrogenase (EC 1.1.1.61)	AK071800 ,AK104168	NP_001148591.1	3-hydroxyisobutyrate dehydrogenase-related, conserved site
Os02g0563000	Hypothetical conserved gene.	BT083559	Q6ZGB6	F-box domain, cyclin-like
Os02g0591500	Similar to H0305E08.4 protein.	AK241753	Q6YY66	-
Os02g0591600	Conserved hypothetical protein.	AK060151	B8AEP7	-
Os02g0591700	Similar to Candida glabrata strain CBS138 chromosome L complete sequence.	AK072777	A2X6N1	Ribosomal protein L6E
Os02g0591800	Brix domain containing protein.	AK102534	B6TPS2	Anticodon-binding
Os02g0591850	Non-protein coding transcript.	AK242359	NONE	-
Os02g0591900	Protein kinase, catalytic domain domain containing protein.	AK100504	Q69L76	Protein kinase, catalytic domain
Os02g0592000	Similar to OSIGBa0106G07.8 protein.	AK361746	Q01IX6	Oxoglutarate/iron-dependent oxygenase
Os02g0592200	Similar to alkaline phosphatase D.	AK069742	NP_001149298.1	Alkaline phosphatase D-related
Os02g0592300	Similar to PMS5 protein (HPMS5 protein) (Fragment).	AK102601	B8AEQ2	DNA mismatch repair protein
Os02g0592400	Hypoxia induced protein conserved region family protein.	AK121468 ,AK105384	NP_001148018.1	Hypoxia induced protein, domain
Os02g0592500	Folate receptor, conserved region domain containing protein.	AK070526	B9F0U2	Folate receptor-like
Os02g0592600	Phospholipase C, phosphatidylinositol-specific, Y domain domain containing protein.	AK103224	A2WUV9	Phospholipase C, phosphatidylinositol-specific, Y domain
Os02g0592700	Conserved hypothetical protein.	AK108162	A2X6P0	-
Os02g0592833	Protein of unknown function DUF581 family protein.	CT841776	NP_001047297.1	Protein of unknown function DUF581
Os02g0593100	Similar to OSIGBa0106G07.12 protein.	AK333669	Q01IY5	Copper amine oxidase
Os02g0598500	Protein phosphatase 2C family protein.	AK106903 (DDBJ, Secondary hit)	NP_001047333.1	Protein phosphatase 2C, manganese/magnesium aspartate binding site
Os02g0598600	Cyclin-like F-box domain containing protein.	AK105564	Q6K1T5	F-box domain, cyclin-like
Os02g0598800	Telomere length regulation protein, conserved domain domain containing protein.	AK372629	NP_001047336.2	Telomere length regulation protein, conserved domain
Os02g0598900	Cyclin-like F-box domain containing protein.	AK105564	Q6K1T5	F-box domain, cyclin-like
Os02g0599100	Hypothetical conserved gene.	tplb0009a09 (Wheat FLcDNA)	Q6K1U1	Telomere length regulation protein, conserved domain
Os02g0599150	Hypothetical conserved gene.	AK106903	Q6K1U4	Protein phosphatase 2C, manganese/magnesium aspartate binding site
Os02g0770500	Similar to WRKY transcription factor 32.	AK363247	Q6IEP9	DNA-binding WRKY
Os02g0770600	Protein of unknown function DUF1644 family protein.	AK101234	Q2Z1Y7	Protein of unknown function DUF1644
Os02g0770700	Peptidase C50, separase domain containing protein.	AK106494	B9F3G4	Peptidase C50, separase
Os02g0770800	Similar to Nitrate reductase [NAD(P)H] (EC 1.7.1.2).	AK102178 ,AK102602	P27968	Oxidoreductase, molybdopterin-binding domain
Os02g0771100	Similar to COP1 (Fragment).	AK111614	Q947M8	WD40 repeat
Os02g0771200	Hypothetical conserved gene.	AK071059	A2XA27	-
Os02g0771400	Protein-tyrosine phosphatase, SIW14-like domain containing [illegible]	ab initio prediction	B6TUQ6	Protein-tyrosine phosphatase, SIW14-like
Os02g0771450	Hypothetical conserved gene.	AK109837 (DDBJ, Secondary hit)	B8A8B2	-
Os02g0771500	Conserved hypothetical protein.	AK062335 ,AK106018	B9F3G5	-
Os02g0771600	Similar to 1-aminocyclopropane-1-carboxylate oxidase (Fragment)	AK103969 ,AK071557	O65031	Oxoglutarate/iron-dependent oxygenase
Os02g0771666	Hypothetical gene.	BT019229	longestORF	-
Os02g0771700	Glycoside hydrolase, family 17 protein.	AK058571 ,AK102185	Q9ZNY6	Glycoside hydrolase, family 17
Os02g0771800	Similar to predicted protein.	AK243530	B8AJ71	-
Os02g0771900	Non-protein coding transcript.	FP091874	NONE	-
Os02g0772000	Similar to global transcription factor group.	AK334486	XP_002879484.1	Transcription elongation factor Spt5, NGN domain
Os03g0124000	Homeodomain-related domain containing protein.	AK121207	B6SZ15	Homeobox
Os03g0124033	Hypothetical protein.	tplb0025a22 (Wheat FLcDNA)	longestORF	-
Os03g0124100	Protein of unknown function DUF604 domain containing protein.	AK107007	Q01KI0	Protein of unknown function DUF604
Os03g0124200	Similar to Protein kinase domain containing protein, expressed.	AK105735	A2XBX8	Protein kinase, catalytic domain
Os03g0124300	Similar to ATP binding protein.	AK069148	B6TWY5	Malectin-like carbohydrate-binding domain
Os03g0124500	Conserved hypothetical protein.	AK119960	B8ALZ1	-
Os03g0124800	Non-protein coding transcript.	AK121064	NONE	-
Os03g0124900	Pectin lyase fold/virulence factor domain containing protein.	AK070284	NP_001151102.1	Glycoside hydrolase, family 28
Os03g0125000	Similar to 50S ribosomal protein L5, chloroplast.	AK098999 ,AK059557 ,AK10413'	Q9ZST0	Ribosomal protein L5
Os03g0125100	Beta-carotene hydroxylase, Drought and oxidative stress [illegible]	AK287823 (Genbank)	Q10SE7	-
Os03g0125300	D111/G-patch domain containing protein.	AK121790 ,AK065004	B6TBI8	D111/G-patch
Os03g0125400	Similar to D-erythro-sphingosine kinase/ diacylglycerol kinase.	AK101342	B6TDW8	Diacylglycerol kinase, catalytic domain
Os03g0125600	Ser Thr specific protein kinase-like protein.	AK243600	Q10SE3	Protein kinase, catalytic domain

Os03g0125650	Hypothetical gene.	AK289045	longestORF	-
Os03g0125700	Ubiquitin ligase, Det1/DDB1-complexing domain containing	AK062913	B8ALZ5	Ubiquitin ligase, Det1/DDB1-complexing
Os03g0125800	Cystathionine beta-synthase, core domain containing protein.	AK102496 ,AK100164	B6SVJ7	Domain of unknown function DUF21
Os03g0125900	Hypothetical conserved gene.	AK071921	Q10SD7	-
Os03g0126000	Similar to Phosphorybosyl anthranilate transferase 1.	AK121680	NP_001130955.1	Glycosyl transferase, family 3
Os03g0126100	Similar to Arabidopsis thaliana DNA, 40 kb surrounding ACS1	AK111921	NP_001151564.1	D-isomer specific 2-hydroxyacid dehydrogenase, NAD-binding
Os03g0126300	Similar to cDNA clone:0012-G03, full insert sequence.	AK100841	Q10SD2	-
Os03g0126450	Domain of unknown function DUF623 domain containing protein.	EU952385	Q8S5W2	Domain of unknown function DUF623
Os03g0126600	Conserved hypothetical protein.	AK243575	Q8S5W1	-
Os03g0126700	Similar to Barley stem rust resistance protein.	AK099929	Q2QSQ6	Heavy metal-associated domain, HMA
Os03g0126800	Similar to CBL-interacting protein kinase 9.	FJ901199	Q10SC8	Protein kinase, catalytic domain
Os03g0126825	Hypothetical gene.	AK242859	longestORF	-
Os03g0127100	Hypothetical gene.	AK120780	longestORF	-
Os03g0127500	bZIP transcription factor, bZIP-1 domain containing protein.	EU971509	NP_001151643.1	Basic-leucine zipper
Os03g0127600	Forkhead-associated domain containing protein.	AK103695	Q8S5V4	Forkhead-associated
Os03g0127650	Non-protein coding transcript.	BT017530	NONE	-
Os03g0127700	Serine/threonine protein kinase-related domain containing protein.	AK103401	Q9LQ11	Protein kinase, catalytic domain
Os03g0127800	Non-protein coding transcript.	EU947022	NONE	-
Os03g0127900	Cation/H+ exchanger domain containing protein.	AK062977	NP_001147290.1	Cation/H+ exchanger
Os03g0127950	Similar to inner membrane protein ybaL.	CT835134	NP_001147290.1	-
Os03g0128000	Similar to Fasciclin-like protein FLA2.	AK062278	Q06IA4	FAS1 domain
Os03g0128100	Similar to 1,3-beta-glucan synthase component family protein, expressed.	AK105008	Q8S5U9	Glycosyl transferase, family 48
Os03g0128200	Similar to predicted protein.	AK243268	Q9LXT9	Protein of unknown function DUF605
Os03g0128300	Conserved hypothetical protein.	AK064718	B8AMD0	-
Os03g0128400	Conserved hypothetical protein.	AK063116	A2XC10	-
Os03g0128500	Similar to DNA polymerase delta small subunit (EC 2.7.7.7).	AK067991	Q9LRE5	DNA polymerase alpha/epsilon, subunit B
Os03g0128600	Conserved hypothetical protein.	AK242075	NP_001173249.1	-
Os03g0128700	Calcium-dependent protein kinase, isoform 11 (EC 2.7.1.-) (CDPK 11).	AK066500	P53684	Protein kinase, catalytic domain
Os03g0128800	Similar to predicted protein.	AK109497	Q10SA9	-
Os03g0128866	Non-protein coding transcript.	tplb0045g05 (Wheat FLcDNA)	NONE	-
Os03g0128932	Similar to Major facilitator superfamily protein, expressed.	BT009593	Q8H887	Sugar/inositol transporter
Os03g0129000	GPI biosynthesis protein Pig-F domain containing protein.	AK073895	Q0PGJ2	GPI biosynthesis protein Pig-F
Os03g0129100	Seven transmembrane protein MLO2.	AK098993 ,AK111990	Q94EX3	Mlo-related protein
Os03g0151000	Conserved hypothetical protein.	AF216531	Q5NAS3	-
Os03g0151100	Appr-1-p processing domain containing protein.	AK099979	NP_001152046.1	Appr-1-p processing
Os03g0151201	Non-protein coding transcript.	AK288979	NONE	-
Os03g0151300	Similar to JmjC domain containing protein, expressed.	AK110927	Q10RP5	Transcription factor jumonji/aspartyl beta-hydroxylase
Os03g0151400	Similar to Zinc finger, C2H2 type family protein, expressed.	AK110807	Q10RP4	-
Os03g0151500	Conserved hypothetical protein.	AK109181	A3AE69	-
Os03g0151600	Similar to N-acetyltransferase.	AK073240	B6T916	GCN5-related N-acetyltransferase
Os03g0151700	Hypothetical conserved gene.	AK111951	B8ANC7	WD40 repeat
Os03g0151800	Hypothetical conserved gene.	AK059991	XP_002465842.1	ATPase, AAA+ type, core
Os03g0151850	Hypothetical gene.	AK373447	longestORF	-
Os03g0151900	Similar to Small GTP-binding protein.	AK105389	Q9FPK1	Small GTPase superfamily
Os03g0152000	Heavy metal transport/detoxification protein domain containing protein.	AK102357	A2XCL8	Heavy metal-associated domain, HMA
Os03g0152100	Similar to E2F-DP transcription factor.	AK242229	B4FHH8	Transcription factor E2F/dimerisation partner
Os03g0152200	Non-protein coding transcript.	BT009415	NONE	-
Os03g0152300	Haem peroxidase family protein.	AK070875	A2XCM0	Plant peroxidase
Os03g0152400	Similar to 4-coumarate--CoA ligase-like 1.	AK242087	Q0DV32	AMP-dependent synthetase/ligase
Os03g0152500	Hypothetical protein.	BT062457	longestORF	-
Os03g0152600	Conserved hypothetical protein.	AK109525	B8AND6	-
Os03g0152700	Pseudouridine synthase domain containing protein.	AK067387	B6U7U0	RNA-binding S4
Os03g0152800	Similar to predicted protein.	AK062056	Q10RM8	-
Os03g0152900	Similar to predicted protein.	AK288414	XP_002876276.1	Armadillo
Os03g0152950	Non-protein coding transcript.	BT085237	NONE	-
Os03g0153000	Similar to cDNA clone:001-019-C07, full insert sequence.	CT835172	Q337U3	RNA recognition motif domain
Os03g0153100	Similar to FAD binding domain containing protein, expressed.	BT054188	Q10RM1	Monooxygenase, FAD-binding
Os03g0153400	Similar to EMB1374.	AK069915	B6UE68	BolA protein
Os03g0181400	Similar to ubiquitin thiolesterase.	AK064006	NP_680185.1	Plant organelle RNA recognition domain
Os03g0181500	Similar to (Rice Genome Annotation Project) rudienead-like	AK100798	Q10QW0	3-Oxoacyl-[acyl-carrier-protein
Os03g0181550	Hypothetical protein.	tplb0055l20 (Wheat FLcDNA)	longestORF	-
Os03g0181600	Similar to GATA transcription factor 25 (ZIM-like 2 protein).	AK067807	B8A2M6	Zinc finger, GATA-type
Os03g0181675	ABC transporter, transmembrane domain domain containing	ab initio prediction	B8APN9	ABC transporter, transmembrane domain
Os03g0181750	ABC transporter, transmembrane domain domain containing	BT066367	Q8H7L0	ABC transporter, transmembrane domain
Os03g0181800	Hypothetical conserved gene.	AK058391	A2XD85	Protein of unknown function DUF936, plant
Os03g0182000	Similar to flavin-dependent monooxygenase 1.	BT040713	XP_002893036.1	Flavin monooxygenase-like
Os03g0182350	Non-protein coding transcript.	AK241509	NONE	-
Os03g0182400	Similar to SAC domain protein 1 (FIG4-like protein AtFIG4).	AK100037	Q10QV0	Synaptojanin, N-terminal
Os03g0182600	Similar to 40S ribosomal protein SA.	AK066899	Q10QU9	Ribosomal protein S2
Os03g0182700	Eukaryotic translation initiation factor 3 subunit 12 (eIF-3 p25) (eIF3k).	AK073293 ,AK120235	Q94HF1	-
Os03g0182800	Similar to Ethylene responsive element binding factor3	AK073133	Q8VXC3	Pathogenesis-related transcriptional factor/ERF, DNA-binding
Os03g0182900	Non-protein coding transcript.	AK103663	NONE	-
Os03g0183000	Similar to Root abundant factor.	AK060929	Q4F8A4	-
Os03g0183050	Hypothetical gene.	EU941438	longestORF	-
Os03g0183100	SAP-like protein BP-73 (OsBP-73) (Riaa1).	AK067631	Q8L4E7	Rho termination factor, N-terminal
Os03g0183200	Similar to AP2 domain containing protein, expressed.	AK106987	Q10QU3	-
Os03g0183300	Similar to BTH-induced ERF transcriptional factor 4.	AK058349	Q5MFV0	-
Os03g0183500	Protein of unknown function DUF581 family protein.	AK063042	NP_197570.1	Protein of unknown function DUF581
Os03g0183600	Similar to Alanine aminotransferase.	Z26322	Q9S768	1-aminocyclopropane-1-carboxylate synthase
Os03g0183800	Similar to Leucine-rich repeat transmembrane protein kinase 1 (Fragment).	AK112046	O81105	Protein kinase, catalytic domain
Os03g0183850	Hypothetical protein.	tplb0047d04 (Wheat FLcDNA)	longestORF	-
Os03g0183900	Similar to Plasma membrane H+-ATPase.	AK242894	Q8RW26	ATPase, P-type, H+ transporting proton pump
Os03g0183950	Hypothetical protein.	tplb0039e05 (Wheat FLcDNA)	longestORF	-
Os03g0632200	Similar to F-box domain containing protein.	AK374846	Q53K61	-
Os03g0632666	Non-protein coding transcript.	AK242727	NONE	-
Os03g0632800	Similar to Zinc finger, C3HC4 type (RING finger) containing	AK242791	Q60DL1	Zinc finger, RING-type
Os03g0632950	Hypothetical protein.	tplb0048g08 (Wheat FLcDNA)	longestORF	-
Os03g0633100	Similar to Leucine Rich Repeat family protein, expressed.	ab initio prediction	Q60DL3	F-box domain, cyclin-like
Os03g0633400	Similar to Leucine Rich Repeat family protein, expressed.	AK240801	Q60DL3	-
Os03g0633500	Similar to Auxin-responsive protein IAA11.	EU973294	Q75GK0	AUX/IAA protein
Os03g0633800	Similar to IAA6 (Fragment).	AK073044	Q75GK1	AUX/IAA protein
Os03g0633900	Nucleic acid-binding, OB-fold-like domain containing protein.	AK068169 ,AK059181	NP_001148836.1	Primosome PriB/single-strand DNA-binding
Os03g0634000	Similar to THA4.	AK063673	NP_001104942.1	Bacterial sec-independent translocation protein MttA/Hcf106
Os03g0634400	Serine/threonine protein kinase domain containing protein.	AK111510	Q75GK4	Protein kinase, catalytic domain
Os03g0635000	Hypothetical conserved gene.	AK371052	Q75GK7	Pentatricopeptide repeat
Os03g0635100	Heterotrimeric G protein gamma subunit 1.	AK241226	B8AN27	-
Os03g0797600	Similar to BHLH transcription factor.	AK068388	F1DKA9	-
Os03g0797700	rRNA-processing protein EFG1 domain containing protein.	AK098876	B8ALB3	rRNA-processing protein EFG1
Os03g0797800	AUX/IAA protein family protein.	AK059619	Q7Y1H8	AUX/IAA protein
Os03g0797902	Conserved hypothetical protein.	FP099914	B9F6G7	-
Os03g0798000	Similar to Aldehyde oxidase-2.	AK242875	O23888	Aldehyde oxidase/xanthine dehydrogenase, molybdopterin binding
Os03g0798101	Similar to Aldehyde oxidase.	ab initio prediction	O23887	Aldehyde oxidase/xanthine dehydrogenase, molybdopterin binding
Os03g0798200	Zinc finger, RING/FYVE/PHD-type domain containing protein.	AK071950 ,AK071454	NP_001150954.1	Zinc finger, RING-type
Os03g0798233	Non-protein coding transcript.	BT084541	NONE	-
Os03g0798266	Non-protein coding transcript.	FP100236	NONE	-
Os03g0798300	Similar to Cytosine-5 DNA methyltransferase MET1 (Fragment).	AK108034	B8ALB7	C-5 cytosine methyltransferase
Os03g0798400	Prenylated rab acceptor PRA1 family protein.	AK109070	NP_001147211.1	Prenylated rab acceptor PRA1
Os03g0798500	Similar to WRKY DNA binding domain containing protein, expressed.	CT835182	Q10C13	-
Os03g0798600	Similar to 40S ribosomal protein S15 (Fragment).	AK121716	A6MZB5	Ribosomal protein S19/S15
Os03g0799000	Similar to Histone H1.	AK067840 ,AK099211	B6TC95	Histone H1/H5
Os03g0799050	Hypothetical protein.	EU961904	longestORF	-
Os03g0799100	Similar to WAVE-DAMPENED2.	AK102772 ,AK058959	Q10C08	Xklp2 targeting protein
Os03g0799200	Protein of unknown function DUF593 family protein.	AK067976	Q851Q1	Protein of unknown function DUF593
Os03g0799250	Non-protein coding transcript.	tplb0044i24 (Wheat FLcDNA)	NONE	-
Os03g0799300	Conserved hypothetical protein.	AK108023	A2XMY9	-
Os03g0799400	Hypothetical conserved gene.	AK362736	Q851Q3	-
Os03g0799500	Protein of unknown function DUF778 family protein.	AK071709	D9J224	Protein of unknown function DUF778
Os03g0799600	Similar to ES43 like protein.	AK104450	NP_001151899.1	Bromo adjacent homology

Os03g0799700	GTP1/OBG subdomain containing protein.	AK109922	B6SV10	GTP-binding domain, HSR1-related
Os03g0799801	Hypothetical conserved gene.	CT835268	NP_001051592.1	-
Os03g0800200	Similar to Protein argonaute MEL1.	AB297928	Q851R2	Argonaute/Dicer protein, PAZ
Os03g0800400	Protein of unknown function DUF1618 domain containing	AK071430	Q851R3	Domain of unknown function DUF1618
Os03g0800500	Putative small multi-drug export family protein.	AK070229	NP_001148347.1	Putative small multi-drug export
Os03g0800700	Similar to Thioredoxin H-type 5 (TRX-H-5).	AK062383	B8ALD1	Thioredoxin
Os03g0800800	Forkhead-associated domain containing protein.	AK065406	NP_001147954.1	Forkhead-associated
Os03g0800900	Hypothetical conserved gene.	AK241533	Q851R7	-
Os03g0801000	Conserved hypothetical protein.	AK111357	Q6YXA4	-
Os03g0801300	Similar to H0219H12.9 protein.	AK241187	Q01II8	-
Os03g0801500	Conserved hypothetical protein.	AK122059	B8ALT9	-
Os03g0801600	Similar to vacuolar protein sorting 35.	AK099884	NP_001151633.1	Vacuolar protein sorting-associated protein 35
Os03g0801700	Rossmann-like alpha/beta/alpha sandwich fold domain containing protein.	AK069505	NP_001151161.1	Rossmann-like alpha/beta/alpha sandwich fold
Os03g0801800	Nucleotide-binding, alpha-beta plait domain containing protein.	AK067130	Q84T02	RNA recognition motif domain
Os03g0801900	Protein of unknown function DUF569 family protein.	AK106415	B8ALU1	Protein of unknown function DUF569
Os03g0802100	Similar to F-box domain containing protein, expressed.	AK288197	Q10BX1	F-box domain, cyclin-like
Os03g0802200	Non-protein coding transcript.	AK111769	NONE	-
Os03g0802300	Hypothetical conserved gene.	AK105727	Q10BW7	-
Os03g0802400	Similar to Avr9/Cf-9 rapidly elicited protein 102 (Fragment).	AK110996	longestORF	-
Os03g0802500	ATPase, AAA-type, core domain containing protein.	AK104696 ,AK070731 ,AK09921	B6SVY2	ATPase, AAA+ type, core
Os03g0802600	ATPase, AAA-type, core domain containing protein.	AK243420	B6SVY2	ATPase, AAA+ type, core
Os03g0802650	Hypothetical gene.	CT837941	longestORF	-
Os03g0802700	RH27 helicase (Fragment).	AK288909	XP_003081470.1	RNA helicase, ATP-dependent, DEAD-box, conserved site
Os03g0802800	Conserved hypothetical protein.	AK059670	Q84T06	-
Os03g0802900	Similar to MYC1.	AK100177	F1DK89	Helix-loop-helix DNA-binding
Os03g0803000	Synaptobrevin domain containing protein.	AK102944	NP_001149329.1	Synaptobrevin
Os03g0803066	Non-protein coding transcript.	AK242539	NONE	-
Os03g0803100	Unknow protein.	ab initio prediction	NP_001051607.1	-
Os03g0803200	Similar to CLE family OsCLE306 protein.	AK111283	A8R3N5	-
Os03g0803250	Non-protein coding transcript.	EU958975	NONE	-
Os03g0803300	Non-protein coding transcript.	AK061608	NONE	-
Os03g0803500	Similar to Prolyl 4-hydroxylase alpha-1 subunit-like protein.	AK059759	B6TAV1	Metridin-like ShK toxin
Os03g0803600	Glycosyl transferase, family 31 protein.	AK241975	B6SJ23	Glycosyl transferase, family 31
Os03g0803700	Hypothetical conserved gene.	AK064481	Q84T10	-
Os03g0803800	Conserved hypothetical protein.	AK063484	Q10BV4	-
Os03g0803900	Similar to predicted protein.	AK288442	NP_201068.1	Galectin, carbohydrate recognition domain
Os03g0804000	Hypothetical protein.	AK059908	longestORF	-
Os03g0804100	Conserved hypothetical protein.	FP100876	NP_001051616.1	-
Os03g0804200	Bifunctional inhibitor/plant lipid transfer protein/seed storage domain containing protein.	AK241129	A2XN28	Plant lipid transfer protein/Par allergen
Os03g0804300	Zinc finger, DHHC-type domain containing protein.	AK104353 ,AK106099	NP_001148846.1	Zinc finger, DHHC-type, palmitoyltransferase
Os03g0804400	Conserved hypothetical protein.	AK073430	Q75HJ9	-
Os03g0804500	Similar to Germin-like protein subfamily T member 1 precursor.	AK062698	Q10BU2	Germin
Os03g0804600	Similar to Germin-like protein 3-7.	AK248846	NP_001051622.2	-
Os03g0804700	Germin-like protein 3-8.	Q75HJ4 (UniProt)	Q75HJ4	Germin
Os03g0804800	Similar to Cct8-prov protein.	AK100651	C6F1N7	Chaperonin TCP-1, conserved site
Os03g0804900	UDP-glucuronosyl/UDP-glucosyltransferase family protein.	AK373315	NP_001051624.1	UDP-glucuronosyl/UDP-glucosyltransferase
Os03g0805100	Similar to Squalene synthase (EC 2.5.1.21).	AK242592	NP_001104839.1	Squalene/phytoene synthase
Os03g0805200	Similar to RNA helicase (Fragment).	AK066048	Q75HJ0	Helicase, C-terminal
Os03g0805300	Similar to Phosphoprotein phosphatase 2A isoform 4.	AK060885 ,AK099257 ,AK10035	Q10BT5	Metallophosphoesterase domain
Os03g0805350	Hypothetical protein.	BT018461	longestORF	-
Os03g0805400	Similar to phosphoric ester hydrolase.	AK060174	NP_001151171.1	Phosphatidic acid phosphatase type 2/haloperoxidase
Os03g0805500	Similar to AAE18 (ACYL-ACTIVATING ENZYME 18)%3B catalytic/ ligase.	AK067857	NP_175929.3	-
Os03g0805600	Similar to pheophorbide a oxygenase.	CT835283	B6ST74	Rieske [2Fe-2S] iron-sulphur domain
Os03g0805700	Similar to Pheophorbide a oxygenase.	EU955939	B6ST74	Pheophorbide a oxygenase
Os03g0805733	Hypothetical protein.	tplb0031c13 (Wheat FLcDNA)	longestORF	-
Os03g0805766	Hypothetical protein.	tplb0031c13 (Wheat FLcDNA)	longestORF	-
Os03g0806100	Hypothetical protein.	tplb0031c13 (Wheat FLcDNA)	longestORF	-
Os03g0806300	Pheophorbide a oxygenase domain containing protein.	AK376575	Q84M41	Pheophorbide a oxygenase
Os03g0806400	Similar to Elongation factor P family protein, expressed.	AK073074	Q10BS5	-
Os03g0806500	Thioredoxin domain 2 containing protein.	AK073308 ,AK100198	Q84M47	Thioredoxin-like fold
Os03g0806600	Conserved hypothetical protein.	AK070959	B9F6V2	-
Os03g0806700	Protein of unknown function DUF868, plant family protein.	AK062034	XP_002466252.1	Protein of unknown function DUF868, plant
Os03g0806800	Conserved hypothetical protein.	AK120337	A2XN54	-
Os03g0806900	Similar to Cytochrome-C reductase 14 kDa subunit (EC 1.10.2.2) (Fragment).	AK121708	B6UBZ9	Cytochrome d ubiquinol oxidase, 14kDa subunit
Os03g0807000	Conserved hypothetical protein.	AK241139	NP_001173679.1	-
Os03g0807100	Protein of unknown function DUF239 domain containing protein.	AK242472	B6TV70	Glucoamylase, putatuve
Os03g0807150	Hypothetical protein.	tplb0032e19 (Wheat FLcDNA)	longestORF	-
Os03g0807200	Non-protein coding transcript.	AK122072	NONE	-
Os03g0807400	Pentatricopeptide repeat domain containing protein.	AK065345	Q84M45	Pentatricopeptide repeat
Os03g0807500	Cupredoxin domain containing protein.	CU406844	B6TG00	Plastocyanin-like
Os03g0807600	Similar to Cyclopropane-fatty-acyl-phospholipid synthase family protein, expressed.	CT835101	Q10BR3	Methyltransferase type 11
Os03g0807700	Similar to predicted protein.	AK121037	A2XN62	Protein of unknown function DUF642
Os03g0807800	Similar to 40S ribosomal protein S2 (Fragment).	AK064984	B6TNR8	Ribosomal protein S5
Os03g0807900	Chaperonin-like RbcX family protein.	AK070287	A2XN64	Chaperonin-like RbcX
Os03g0808000	Glycoside hydrolase, family 28 domain containing protein.	BT042622	NP_001051647.1	Glycoside hydrolase, family 28
Os03g0808100	Similar to Cellulose synthase BoCesA5.	AK061688	Q4U0Z5	Cellulose synthase
Os03g0808150	Hypothetical gene.	BT019277	longestORF	-
Os03g0808175	Non-protein coding transcript.	U48693	NONE	-
Os03g0808200	UDP-glucuronosyl/UDP-glucosyltransferase family protein.	BT037819	NP_001149762.1	UDP-glucuronosyl/UDP-glucosyltransferase
Os03g0808300	Remorin, C-terminal region domain containing protein.	AK109389	NP_001152428.1	Remorin, C-terminal
Os03g0808350	Hypothetical protein.	AK361927	longestORF	-
Os03g0808400	Ubiquitin domain containing protein.	ab initio prediction	Q84M51	Ubiquitin
Os03g0808500	Plant lipid transfer protein/Par allergen family protein.	CT835148	Q5NE32	Plant lipid transfer protein/Par allergen
Os03g0823301	Hypothetical conserved gene.	EU942158	B6TW49	-
Os03g0823350	Conserved hypothetical protein.	EU943107	B6TW49	-
Os03g0823400	Similar to Bowman-Birk type trypsin inhibitor (WTI).	AK120562	A1EGX1	Proteinase inhibitor I12, Bowman-Birk
Os03g0823500	TGF-beta receptor, type I/II extracellular region family protein.	AK058972 ,AK099193	NP_181326.1	-
Os03g0823550	Hypothetical protein.	tplb0038k08 (Wheat FLcDNA)	longestORF	-
Os03g0823700	Similar to Ras-related protein Rab11C.	AK067508	B4F7V4	Small GTPase superfamily
Os03g0823750	Non-protein coding transcript.	BT084300	NONE	-
Os03g0823800	Similar to permease I.	AK058223	NP_001149779.1	Xanthine/uracil/vitamin C permease
Os03g0823900	Calmodulin binding protein-like domain containing protein.	EU970985	NP_001050439.2	Calmodulin binding protein-like
Os03g0824000	Peptidase S8 and S53, subtilisin, kexin, sedolisin domain containing protein.	AK111129	A9NKD9	Protein of unknown function DUF3743
Os03g0824100	Tetratricopeptide-like helical domain containing protein.	AK065308	Q852B8	Smr protein/MutS2 C-terminal
Os03g0824200	Methyltransferase small domain containing protein.	AK059711	B6TML1	DNA methylase, N-6 adenine-specific, conserved site
Os03g0824300	Hypothetical conserved gene.	EU944857	NP_001051750.2	RNA recognition motif domain
Os03g0824350	Conserved hypothetical protein.	AK243596	B9EWT4	-
Os03g0824400	Similar to Dolichol-phosphate mannosyltransferase (EC 2.4.1.83) (Dolichol- phosphate mannose synthase) (Dolichyl-phosphate beta-D- mannosyltransferase) (Mannose-P-dolichol synthase) (MPD synthase) (DPM synthase).	AK104298 ,AK070153	Q9LMW5	Glycosyl transferase, family 2
Os03g0824500	Conserved hypothetical protein.	AK058990	A2XNI9	-
Os03g0824600	Similar to cytokinin-N-glucosyltransferase 1.	tplb0017i08 (Wheat FLcDNA)	NP_001149878.1	UDP-glucuronosyl/UDP-glucosyltransferase
Os03g0824650	Hypothetical protein.	AK358998	longestORF	-
Os03g0825300	Serine/threonine protein kinase domain containing protein.	AK072216	B6UD08	Protein kinase, catalytic domain
Os03g0825400	Similar to Mitochondrial import inner membrane translocase subunit Tim10.	AK059163	B6U5I0	Mitochondrial inner membrane translocase complex, Tim8/9/10/13-zinc finger-like
Os03g0825500	Transcription antitermination protein, NusG, N-terminal domain containing protein.	AK058472	NP_001151355.1	KOW
Os03g0825600	Conserved hypothetical protein.	AK063312	Q94GE3	-
Os03g0825700	Similar to DEX1 (DEFECTIVE IN EXINE FORMATION 1).	AK104830	NP_566343.1	-
Os03g0825800	Protein kinase, core domain containing protein.	AK108602	Q10BA3	Protein kinase, catalytic domain
Os03g0825850	Hypothetical protein.	AK110992	longestORF	-
Os04g0334700	Similar to OSIGBa0137O04.7 protein.	AK109756	Q01KZ0	Peptidase A1
Os04g0334825	Conserved hypothetical protein.	ab initio prediction	Q7X7R4	-
Os04g0334951	Similar to OSIGBa0137O04.7 protein.	AK109756	Q01KZ0	Peptidase A1
Os04g0335075	Conserved hypothetical protein.	ab initio prediction	Q7X7R4	-
Os04g0335200	Conserved hypothetical protein.	AK289258	Q2QWP9	-

Os04g0335400	Non-protein coding transcript.	CT828838	NONE"	-
Os04g0336001	Hypothetical protein.	EU945299	longestORF	-
Os04g0336600	Peptidase aspartic, catalytic domain containing protein.	AK120870 (DDBJ, Secondary hit)	B7F2P1	Peptidase A1
Os04g0336700	Conserved hypothetical protein.	ab initio prediction	Q7X7R4	-
Os04g0336801	Conserved hypothetical protein.	AK289258 (DDBJ, Secondary hit)	Q2QWP9	-
Os04g0337000	Peptidase aspartic, catalytic domain containing protein.	AK066236	Q01KZ0	Peptidase A1
Os04g0526800	GRAM domain containing protein.	AK061581	Q01HP4	GRAM
Os04g0527000	GRAM domain containing protein.	AK099545 ,AK070324 ,AK10443	Q01HP3	-
Os04g0527300	Non-protein coding transcript.	AK110653	NONE	-
Os04g0527400	BRO1 domain containing protein.	AK101489	Q01HP2	BRO1 domain
Os04g0527500	Similar to OSIGBa0115K01-H0319F09.11 protein.	AK059144	Q01HP1	Mitochondrial carrier domain
Os04g0527700	CHCH domain containing protein.	AK072980	Q01HP0	-
Os04g0527800	Similar to OSIGBa0115K01-H0319F09.13 protein.	AK104019	Q01HN9	-
Os04g0527900	Similar to Tonoplast membrane integral protein ZmTIP3-2.	AK108116	Q01HN8	Major intrinsic protein
Os04g0528000	Similar to OSIGBa0115K01-H0319F09.15 protein.	BT018594	Q01HN7	Protein of unknown function DUF789
Os04g0528100	Similar to OSIGBa0115K01-H0319F09.16 protein.	AK065957	Q01HN6	-
Os04g0528200	Similar to OSIGBa0115K01-H0319F09.17 protein.	AK064693	Q01HN5	-
Os04g0528300	Similar to OSIGBa0115K01-H0319F09.18 protein.	AK058387	Q01HN4	ABC-2 type transporter
Os04g0528400	Hypothetical gene.	AK107445	longestORF	-
Os04g0528600	Similar to OSIGBa0115K01-H0319F09.22 protein.	AK240918	Q01HN0	-
Os04g0528651	Hypothetical protein.	tplb0055d11 (Wheat FLcDNA)	longestORF	-
Os04g0528800	Similar to OSIGBa0115K01-H0319F09.23 protein.	AK062397	Q01HM9	Lipoate synthase
Os04g0529100	Pathogenesis-related transcriptional factor and ERF domain containing protein.	AK107680	Q01HM7	Pathogenesis-related transcriptional factor/ERF, DNA-binding
Os04g0529300	Hypothetical conserved gene.	AK288129	B8ASC6	-
Os04g0529400	Similar to OO_Ba0013J05-OO_Ba0033A15.30 protein.	AK099627	D0ABH3	WD40 repeat
Os04g0529500	Similar to cDNA clone:J023022A09, full insert sequence.	AK059419	B7EGJ9	Double-stranded RNA-binding
Os04g0529600	Lanthionine synthetase C-like family protein.	AK288141	Q00RJ6	Lanthionine synthetase C-like
Os04g0529700	Glycosyltransferase sugar-binding region containing DXD motif domain containing protein.	AK121532	Q00RJ5	Glycosyltransferase, DXD sugar-binding motif
Os04g0529800	Similar to OSIGBa0155K17.6 protein.	AK101735	Q00RJ4	Sugar/inositol transporter
Os04g0530000	Similar to OSIGBa0155K17.8 protein.	AK066169	Q00RJ2	Transcription factor TFIIIC, tau55-related
Os04g0530050	Hypothetical protein.	ab initio prediction	NONE	-
Os04g0530100	Similar to Beta-expansin 1 precursor (AtEXPB1) (At-EXPB1) (Ath-ExpBeta-1.5).	AK107184	Q7X6J9	Expansin/pollen allergen, DPBB domain
Os04g0530150	Non-protein coding transcript.	AK109871	NONE	-
Os04g0530200	Similar to OSIGBa0155K17.10 protein.	AK105586 ,AK121439	Q00RJ0	-
Os04g0530300	Similar to OSIGBa0155K17.11 protein.	AK066705	Q00RI9	-
Os04g0530400	t-snare domain containing protein.	AK067634	B6TDT7	SNARE-complex protein Syntaxin-18 N-terminal
Os04g0530500	Zinc finger, RING/FYVE/PHD-type domain containing protein.	AK104359	NP_001152715.1	Zinc finger, RING-type
Os04g0530600	Similar to Thioredoxin 1 (TRX-1) (Thioredoxin M).	AK069195	NP_001150752.1	Thioredoxin
Os04g0530700	Similar to Beta-D-xylosidase.	AK120331	NP_196618.1	Glycoside hydrolase, family 3, N-terminal
Os04g0530801	Hypothetical gene.	EU973535	longestORF	-
Os04g0530900	Glycosyl transferase, family 8 protein.	AK120509	XP_002312381.1	Glycosyl transferase, family 8
Os04g0531100	C2 calcium-dependent membrane targeting domain containing	AK063584	Q0JBH9	C2 calcium-dependent membrane targeting
Os04g0531200	Hypothetical conserved gene.	AB332066	A2XVT7	-
Os04g0531300	tRNA-dihydrouridine synthase domain containing protein.	AK072647	Q00RI1	tRNA-dihydrouridine synthase
Os04g0531400	Similar to Lectin-like receptor kinase 7%3B2.	AK105289	Q00RI0	Protein kinase, catalytic domain
Os04g0531500	Concanavalin A-like lectin/glucanase domain containing protein.	AK102285	Q00RH9	Protein kinase, catalytic domain
Os04g0531600	Hypothetical protein.	AK107678	longestORF	-
Os04g0531700	Short-chain dehydrogenase/reductase SDR domain containing protein.	AK119481	Q00RH9	Short-chain dehydrogenase/reductase SDR
Os04g0531750	Similar to OSIGBa0125M19.13 protein.	ab initio prediction	Q00RH3	Short-chain dehydrogenase/reductase SDR
Os04g0531800	Conserved hypothetical protein.	AK121355	B8ASD8	-
Os04g0531900	Short-chain dehydrogenase/reductase SDR domain containing protein.	AK071069	Q00RH3	Short-chain dehydrogenase/reductase SDR
Os04g0532100	Short-chain dehydrogenase/reductase SDR domain containing protein.	AK109281	Q00RH5	Short-chain dehydrogenase/reductase SDR
Os04g0532200	Conserved hypothetical protein.	AK110853	B8ASE2	-
Os04g0532400	Similar to OSIGBa0125M19.13 protein.	AK318556	Q00RH3	Short-chain dehydrogenase/reductase SDR
Os04g0532500	Similar to Transcription factor L2.	AK069907	Q00RH2	Zinc finger, LIM-type
Os04g0532700	Hypothetical protein.	AK109796	longestORF	-
Os04g0532800	Myb transcription factor domain containing protein.	AK107135	B4FNQ6	SANT domain, DNA binding
Os04g0533000	Similar to RNA helicase (Fragment).	AK071636	B7EKD5	RNA helicase, ATP-dependent, DEAD-box, conserved site
Os04g0533200	Similar to Myb7 protein (Fragment).	AK376409	Q43598	-
Os04g0533250	Hypothetical gene.	EU971393	longestORF	-
Os04g0533300	Similar to remorin.	AK104678 ,AK061351	NP_001159012.1	Remorin, C-terminal
Os04g0533500	Cytochrome b561 family protein.	AK061426 ,AK069219	Q01II4	-
Os04g0533602	Hypothetical genes.	ab initio prediction	NONE	-
Os04g0533700	Putative non-inhibitory serpin-10.	Q7XMK1 (UniProt)	Q7XMK1	Protease inhibitor I4, serpin
Os04g0542800	Similar to Metal-nicotianamine transporter YSL2.	AK070304	Q6H3Z6	Oligopeptide transporter OPT superfamily
Os04g0542900	Similar to endonuclease, polyU-specific.	AK068610	NP_001150308.1	Endoribonuclease XendoU
Os04g0543000	Similar to Protein kinase.	AK064119	A7UGD8	Protein kinase, catalytic domain
Os04g0543033	Hypothetical protein.	EU951577	longestORF	-
Os04g0543100	Hypothetical gene.	AK062379	longestORF	-
Os04g0543200	Nucleotide-binding, alpha-beta plait domain containing protein.	AK102135 ,AK101961	NP_001105099.1	RNA recognition motif domain
Os04g0543500	Hypothetical conserved gene.	CT836502	NP_001053454.2	-
Os04g0543600	Amino acid/polyamine transporter I family protein.	AK064289	XP_002326482.1	Amino acid/polyamine transporter I
Os04g0543650	Hypothetical protein.	EU957609	longestORF	-
Os04g0543700	Similar to Serine proteinase (Fragment).	AK106823	Q68Q08	Peptidase S8/S53, subtilisin/kexin/sedolisin
Os04g0543900	Similar to Glutamate dehydrogenase 2.	AK063467	Q33E23	Glutamate/phenylalanine/leucine/valine dehydrogenase
Os04g0544000	Hypothetical conserved gene.	EU972251	Q7XN05	-
Os04g0544100	Similar to SET domain-containing protein SET104.	AK066297	NP_001105193.1	SET domain
Os04g0544200	Cyclin-related 2 domain containing protein.	AK287844	Q949F8	Cyclin P/U
Os04g0544400	Hypothetical conserved gene.	AK371099	Q7XN02	Pentatricopeptide repeat
Os04g0544500	Similar to Similarities with spP40209 Saccharomyces cerevisiae YMR136w GAT2.	AK070729	NP_001149109.1	Zinc finger, GATA-type
Os04g0544700	Hypothetical protein.	tplb0054e15 (Wheat FLcDNA)	longestORF	-
Os04g0544900	Alg9-like mannosyltransferase family protein.	AK069661	Q01J26	GPI mannosyltransferase
Os04g0545000	Similar to WRKY transcription factor 34.	AK073695	Q01J25	DNA-binding WRKY
Os04g0545100	Engulfment and cell motility, ELM domain containing protein.	AK071451	Q01J24	Engulfment/cell motility, ELMO
Os04g0545200	Similar to OSIGBa0101C23.4 protein.	AK360700	Q01J23	Domain of unknown function DUF3598
Os04g0545250	Hypothetical protein.	AK241990	longestORF	-
Os04g0545300	Cupredoxin domain containing protein.	ab initio prediction	Q01J22	-
Os04g0545400	Cupredoxin domain containing protein.	EU961255	B6T8E0	Plastocyanin-like
Os04g0568300	Hypothetical gene.	AK072175	longestORF	-
Os04g0568400	WD40 repeat-like domain containing protein.	AK103546	Q01JP7	WD40 repeat
Os04g0568500	Conserved hypothetical protein.	AK363942	F2DJ74	-
Os04g0568600	Similar to 6-phospho-3-hexuloisomerase.	CT836509	Q01JP6	Sugar isomerase
Os04g0568700	Similar to Heat stress transcription factor Spl7 (Heat shock transcription factor) (Heat shock factor RHSF10).	AY344483	Q01JP5	Heat shock factor
Os04g0568751	Hypothetical protein.	tplb0033f11 (Wheat FLcDNA)	longestORF	-
Os04g0568800	Similar to OSIGBa0139P06.4 protein.	AK106571	Q01JP4	Protein of unknown function DUF810
Os04g0568850	Similar to OSIGBa0139P06.4 protein.	CT828553	Q01JP4	-
Os04g0568900	Similar to RING finger protein 6 (RING-H2 protein).	AK070267	Q01JP3	Zinc finger, RING-type
Os04g0568950	Hypothetical gene.	EU941385	longestORF	-
Os04g0569000	Similar to Replication factor C 40kDa subunit.	AK069025	Q948P2	ATPase, AAA+ type, core
Os04g0569100	Similar to OCL1 homeobox protein.	AK112099 ,AK111914	Q7Y0V9-2	Helix-turn-helix motif, lambda-like repressor
Os04g0569300	Similar to Membrane protein.	AK099082	Q01JP0	Peptidase S54, rhomboid
Os04g0569400	Similar to OSIGBa0139P06.9 protein.	AK365201	Q01JN9	Protein of unknown function DUF248, methyltransferase putative
Os04g0569500	Hypothetical protein.	tplb0021o10 (Wheat FLcDNA)	longestORF	-
Os04g0569900	Similar to OSIGBa0111L12.1 protein.	AK059100	Q01J01	-
Os04g0570000	Cytochrome P450 family protein.	AJ459255	Q7XU38	Cytochrome P450
Os04g0570125	Hypothetical protein.	tplb0036f19 (Wheat FLcDNA)	longestORF	-
Os04g0583000	Conserved hypothetical protein.	AK068074	A2XWT1	-
Os04g0583101	Pentatricopeptide repeat domain containing protein.	AK065769	NP_001174064.1	Pentatricopeptide repeat
Os04g0583200	Conserved hypothetical protein.	AK073181	A3AWR9	-
Os04g0583266	Hypothetical protein.	BT019268	longestORF	-
Os04g0583332	Non-protein coding transcript.	CT834365	NONE"	-
Os04g0583500	Similar to Expansin-A10.	AK066414	Q7XUD0	Expansin
Os04g0583550	Non-protein coding transcript.	BT016845	NONE	-
Os04g0583600	Similar to Histone H4.	AK059019	XP_002310853.1	Histone H4
Os04g0583700	Zinc finger, FYVE-type domain containing protein.	ab initio prediction	Q7F9W1	Zinc finger, FYVE-type

Os04g0583800	RNA-binding region RNP-1 (RNA recognition motif) domain containing protein.	AK241387	NP_001151732.1	Nucleotide-binding, alpha-beta plait
Os04g0583900	Similar to LHY protein.	AK101209	D0AB68	SANT domain, DNA binding
Os04g0584002	Non-protein coding transcript.	EU951957	NONE	-
Os04g0584100	Serine/threonine protein kinase domain containing protein.	AK108779	NP_196292.1	Protein kinase, catalytic domain
Os04g0584201	Hypothetical protein.	AK105292	longestORF	-
Os04g0584300	Similar to Catalytic/ protein phosphatase type 2C.	AK289111	B6TAL6	Protein phosphatase 2C, manganese/magnesium aspartate binding site
Os04g0584500	U box domain domain containing protein.	AK099968	Q7XND9	U box domain
Os04g0584550	Hypothetical gene.	AK287512	longestORF	-
Os04g0584600	Similar to cDNA clone:0011-E12, full insert sequence.	AK059786	Q5UKX8	Protein kinase, catalytic domain
Os04g0584700	Pentatricopeptide repeat domain containing protein.	AK108387	Q7FA49	Pentatricopeptide repeat
Os04g0584750	Hypothetical protein.	BT085496	longestORF	-
Os04g0584800	Hypothetical conserved gene.	AK058492	B8ATD0	Alpha/gamma-adaptin-binding protein p34
Os04g0584900	Hypothetical conserved gene.	AK058633	A2XWU9	-
Os04g0601200	Hypothetical gene.	AK063203	longestORF	-
Os04g0601400	EF-HAND 2 domain containing protein.	AK069777	Q01HT8	Calcium-binding EF-hand
Os04g0601500	Similar to B0403H10-OSIGBa0105A11.11 protein.	EU955565	Q01HT7	-
Os04g0601700	Similar to Vacuolar ATP synthase subunit G 1 (EC 3.6.3.14) (V-ATPase G subunit 1) (Vacuolar proton pump G subunit 1).	AK067286	Q01HT5	Vacuolar
Os04g0601800	Similar to Plastid protein.	AK062641	Q01HT4	-
Os04g0602000	Similar to B0403H10-OSIGBa0105A11.12 protein.	ab initio prediction	Q01HT6	-
Os04g0602100	Similar to B0403H10-OSIGBa0105A11.15 protein.	AK121197	Q01HT3	Haem peroxidase, plant/fungal/bacterial
Os04g0602200	Bacterial extracellular solute-binding protein, family 1 protein.	AK120467	Q01HT2	Bacterial extracellular solute-binding, family 1
Os04g0602250	Non-protein coding transcript.	EU946899	NONE	-
Os04g0602300	Similar to B0403H10-OSIGBa0105A11.17 protein.	AK105720	Q01HT1	-
Os04g0602400	Similar to Maltose excess protein 1, chloroplast precursor (Root cap protein 1).	AK071005	Q01HT0	-
Os04g0602500	Similar to B0403H10-OSIGBa0105A11.19 protein.	AK058634	Q01HS9	Pectinacetylesterase
Os04g0602600	Pentatricopeptide repeat domain containing protein.	AK120600	Q01HS8	Pentatricopeptide repeat
Os04g0602700	RNA recognition motif, RNP-1 domain containing protein.	EU959252	Q01HS7	RNA recognition motif domain
Os04g0602800	Similar to B0403H10-OSIGBa0105A11.22 protein.	AK060878	Q01HS6	-
Os04g0602900	Similar to Protein-S-isoprenylcysteine O-methyltransferase.	AK100414	B6TWA2	-
Os04g0603000	Similar to OSIGBa0118P15.1 protein.	BT064689	Q01KL0	Aldose 1-epimerase
Os04g0603100	Hypothetical protein.	AK073515	longestORF	-
Os04g0603200	Similar to OSIGBa0118P15.2 protein.	AK059404	Q01KK9	-
Os04g0603300	Hypothetical protein.	AK106704	longestORF	-
Os04g0603400	Similar to OSIGBa0118P15.3 protein.	AK107076	Q01KK8	-
Os04g0603601	Non-protein coding transcript.	CT835044	NONE"	-
Os04g0669500	Phospholipase/carboxylesterase domain containing protein.	AK069249	Q259P2	Phospholipase/carboxylesterase/thioesterase
Os04g0669600	Phospholipase/carboxylesterase domain containing protein.	AK110767	Q259P1	Phospholipase/carboxylesterase/thioesterase
Os04g0669700	Similar to H0818H01.9 protein.	CT835076	Q259P0	Phospholipase/carboxylesterase/thioesterase
Os04g0669800	Methylthioribose kinase (EC 2.7.1.100).	AK067649	Q7XR61	Aminoglycoside phosphotransferase
Os04g0669900	Similar to H0818H01.11 protein.	AY593959 (DDBJ, Secondary hit)	Q259N8	Aminoglycoside phosphotransferase
Os04g0670000	Reticulon family protein.	AK071792	Q259P4	Reticulon
Os04g0670100	Similar to H0818H01.13 protein.	FP095236	Q259P3	-
Os04g0670150	Hypothetical protein.	BT068200	longestORF	-
Os04g0670200	Granulin domain containing protein.	AK099358	GeneMark	Granulin
Os04g0670400	Similar to H0624F09.2 protein.	AK064003	Q258Z6	Ovarian tumour, otubain
Os04g0670500	Cysteine protease 1 precursor (EC 3.4.22.-) (OsCP1).	AK107506	Q7XR52	Granulin
Os04g0670600	Similar to H0624F09.5 protein.	AK065924	Q258Z3	Exostosin-like
Os04g0670700	Similar to H0624F09.4 protein.	AK063069	Q258Z4	Phosphatidate cytidylyltransferase
Os04g0670800	UBX domain containing protein.	AK072097	Q258Z2	UBX
Os04g0670900	Homeodomain-like containing protein.	DQ383374	Q258Z1	MYB-like
Os04g0671100	Similar to H0624F09.8 protein.	AK241125	Q258Z0	Adenylate kinase
Os04g0671200	Similar to H0624F09.9 protein.	AK106044	Q258Y9	Amine oxidase
Os04g0671250	Hypothetical protein.	tplb0048c15 (Wheat FLcDNA)	longestORF	-
Os04g0671300	Similar to Suppressor of presenilin 5 (P110b homolog).	AK072414	Q258Y8	Flavin amine oxidase
Os04g0671500	Hypothetical protein.	tplb0048c15 (Wheat FLcDNA)	longestORF	-
Os04g0671700	ThiJ/PfpI domain containing protein.	AK100753	NP_001146927.1	ThiJ/PfpI
Os04g0671800	Similar to H0624F09.12 protein.	AK059421	F1DK83	-
Os04g0671900	Similar to Auxin response factor 12.	AK104920	Q0J951	AUX/IAA protein
Os04g0672100	Similar to Phytosulfokine receptor precursor (EC 2.7.1.37) (Phytosulfokine LRR receptor kinase).	AK121689	Q258Z9	Protein kinase, catalytic domain
Os04g0672200	Poly(ADP-ribose) polymerase, catalytic region domain containing protein.	AK099725	Q258Z8	Poly
Os04g0672300	Similar to H0322F07.3 protein.	AK101246	Q259A6	-
Os04g0672600	Leucine-rich repeat, N-terminal domain containing protein.	AK070283	Q258Z9	Leucine-rich repeat
Os04g0672800	Similar to H0322F07.5 protein.	AK068413	Q259A4	-
Os04g0672900	Similar to H0322F07.6 protein.	AK062248	Q259A3	Domain of unknown function DUF2296
Os04g0673000	Similar to H0322F07.7 protein.	AK066211	Q259A2	Protein of unknown function DUF3755
Os04g0673050	Non-protein coding transcript.	tplb0055d06 (Wheat FLcDNA)	NONE	-
Os04g0673300	A-type response regulator, Cytokinin signaling	AK059734 (Genbank),AB249653 (	Q7XQA6	Signal transduction response regulator, receiver domain
Os04g0673400	Similar to Uracil-DNA glycosylase.	AK287652	D0ABD0	Uracil-DNA glycosylase
Os04g0673700	Hypothetical protein.	AK105405	longestORF	-
Os04g0673800	Cupredoxin domain containing protein.	AK071236	Q259S3	Plastocyanin-like
Os04g0674000	Similar to H0403D02.10 protein.	AK119671 ,AK071276	Q259S8	-
Os04g0674025	Similar to H0403D02.10 protein.	ab initio prediction	Q259S8	-
Os04g0674100	Similar to H0403D02.12 protein.	AK242456	Q259S2	Tetratricopeptide-like helical
Os04g0674200	Coenzyme Q biosynthesis Coq4 family protein.	AK103795 ,AK101965	Q259S1	Coenzyme Q biosynthesis Coq4
Os04g0674300	Similar to H0403D02.14 protein.	AK059048	Q259S0	NPH3
Os04g0674350	Conserved hypothetical protein.	AK241498	NP_001174139.1	-
Os04g0674400	Similar to Anamorsin (Cytokine induced apoptosis inhibitor 1) (CUA001). Splice isoform 2.	AK102124	B8ARI7	Cytokine-induced anti-apoptosis inhibitor 1
Os04g0674425	Non-protein coding transcript.	BT086960	NONE	-
Os04g0674450	Similar to DHHC zinc finger domain containing protein.	AK376242	NP_001147996.1	Zinc finger, DHHC-type, palmitoyltransferase
Os04g0674500	Hypothetical protein.	AK120940	longestORF	-
Os04g0674600	Similar to H0103C06.1 protein.	AK069645	Q259I1	Oligopeptide transporter OPT superfamily
Os04g0674700	Similar to AMP-binding protein (Adenosine monophosphate binding protein 5 AMPBP5).	AK106615	Q259I0	AMP-dependent synthetase/ligase
Os04g0674750	Hypothetical protein.	EU941562	longestORF	-
Os04g0674800	Similar to CEL1%3DCELLULASE 1 (Fragment).	AK119913	Q0J930	Glycoside hydrolase, family 9
Os04g0674900	Hypothetical protein.	tplb0042g22 (Wheat FLcDNA)	longestORF	-
Os04g0675000	Non-protein coding transcript.	AK063870	NONE	-
Os04g0675101	ATPase-like, ATP-binding domain domain containing protein.	BT066996	B9FDA1	ATPase-like, ATP-binding domain
Os04g0675200	Similar to H0103C06.6 protein.	AK121874	Q259H3	-
Os04g0675300	Similar to H0103C06.6 protein.	AK111982	Q259H3	Zinc finger, RING-type
Os04g0675400	Similar to Chaperone protein dnaJ.	AK068186	Q259H2	Heat shock protein DnaJ, N-terminal
Os04g0675500	Similar to Itm1 protein.	AK099138	Q259H1	Oligosaccharyl transferase, STT3 subunit
Os04g0675600	Hypothetical conserved gene.	AK242041	NP_001054249.1	Pentatricopeptide repeat
Os04g0675700	Hypothetical conserved gene.	ab initio prediction	D0ABD8	FBD
Os04g0675800	Similar to H0103C06.10 protein.	ab initio prediction	Q259H7	F-box domain, cyclin-like
Os04g0676000	Non-protein coding transcript.	CT836511	NONE"	-
Os04g0676100	Similar to Thioredoxin X, chloroplast precursor.	AK288094	Q259H6	Thioredoxin
Os04g0676200	Pentatricopeptide repeat domain containing protein.	AK106412	Q7XKC9	Pentatricopeptide repeat
Os04g0676300	Similar to H0101F08.3 protein.	AK287813	Q259H4	Dihydroorotate dehydrogenase, conserved site
Os04g0676400	Similar to H0101F08.4 protein.	AK121530	Q259I9	Protein of unknown function DUF761, plant
Os04g0676600	Similar to H0101F08.6 protein.	AK072874	Q259I7	Transcriptional factor B3
Os04g0676650	Putative B3 domain-containing protein Os04g0676650.	Q7XKC4 (UniProt)	Q7XKC4	Transcriptional factor B3
Os04g0690100	Similar to H0814G11.16 protein.	AK105834	Q00RM4	Zinc finger, C2H2
Os04g0690300	Similar to H0814G11.17 protein.	AK289176	Q00RM3	Tetratricopeptide TPR-1
Os04g0690400	Similar to H0814G11.18 protein.	AK102981 ,AK103240	Q00RM2	-
Os04g0690451	Hypothetical gene.	CT836480	longestORF	-
Os04g0690500	Similar to CAA30371.1 protein (Fragment).	AK105853	Q9ST96	-
Os04g0690600	Similar to Auxin response factor 13.	AK109449	Q7XSS9	Transcriptional factor B3
Os04g0690800	22 kDa protein of photosystem II.	AK071638	Q40716	Chlorophyll A-B binding protein
Os04g0690900	Similar to H0323C08.15 protein.	AK241685	Q25A34	-
Os04g0690932	Hypothetical gene.	AK062616	longestORF	-
Os04g0691000	Conserved hypothetical protein.	AK063241	NP_001043476.1	-
Os04g0691100	Serine/threonine-protein kinase SAPK5 (EC 2.7.1.37) (Osmotic stress/abscisic acid-activated protein kinase 5).	AK100269	Q7XKA8	Protein kinase, catalytic domain
Os04g0691200	Similar to predicted protein.	AK369781	XP_002869342.1	WD40 repeat
Os04g0691300	Similar to ANAC030.	AK334647	NP_001148332.1	No apical meristem
Os04g0691366	Similar to POT family protein.	ab initio prediction	B6SXJ7	-
Os04g0691400	Similar to POT family protein.	ab initio prediction	B6SXJ7	Oligopeptide transporter

Os04g0691433	Non-protein coding transcript.	EU946594	NONE	-
Os04g0691466	Hypothetical gene.	EU949821	longestORF	-
Os04g0691500	Similar to TRN1 (TRANSPORTIN 1)%3B protein transporter.	AK100077	NP_001031359.1	HEAT
Os04g0691600	Similar to 30S ribosomal protein S17.	AK099298	Q9ZST1	Ribosomal protein S17
Os04g0691700	GCN5-related N-acetyltransferase (GNAT) domain domain containing protein.	AK106305	Q7FAP7	GCN5-related N-acetyltransferase
Os04g0691750	Similar to phosphoribosylanthranilate transferase.	tplb0048i05 (Wheat FLcDNA)	NP_001152458.1	-
Os04g0691800	C2 domain containing protein.	AK318532	NP_001152458.1	C2 calcium-dependent membrane targeting
Os04g0691900	Chaperonin Cpn60/TCP-1 family protein.	AK068257	Q7XKA1	Chaperonin Cpn60/TCP-1
Os04g0692000	Protein of unknown function DUF6, transmembrane domain containing protein.	AK103611 ,AK061991	B4G1R1	-
Os04g0692100	Similar to Tubulin folding cofactor B.	AK102150	A5CFZ4	Cytoskeleton-associated protein, Gly-rich domain
Os04g0692200	Non-protein coding transcript.	AK059342	NONE	-
Os04g0692300	Peptidase C14, ICE, catalytic subunit p20, active site domain containing protein.	AK073368	XP_002871357.1	Zinc finger, RING/FYVE/PHD-type
Os04g0692400	Similar to ankyrin-like protein.	AK070717	NP_001151565.1	Protein of unknown function DUF248, methyltransferase putative
Os04g0692500	Similar to antiporter/ drug transporter/ transporter.	AK101528	NP_001147555.1	Ribonuclease Zc3h12a-like
Os04g0692600	Hypothetical gene.	AK318609	longestORF	-
Os04g0692700	Similar to CHR24 (chromatin remodeling 24)%3B ATP binding / DNA binding / helicase.	BT087024	NP_201200.2	Helicase, C-terminal
Os04g0692725	Hypothetical gene.	AK242828	longestORF	-
Os04g0692750	Hypothetical conserved gene.	BT083703	XP_002309928.1	-
Os05g0149300	Similar to 1-aminocyclopropane-1-carboxylate oxidase.	BT087584	O81606	Oxoglutarate/iron-dependent oxygenase
Os05g0149400	Similar to 1-aminocyclopropane-1-carboxylic acid oxidase.	AK061064	O81606	Oxoglutarate/iron-dependent oxygenase
Os05g0149450	Non-protein coding transcript.	tplb0008n13 (Wheat FLcDNA)	NONE	-
Os05g0149500	Similar to lipopolysaccharide-modifying protein.	AK064684	NP_001151574.1	Lipopolysaccharide-modifying protein
Os05g0149600	Similar to predicted protein.	AK061409	C7SJ62	Cullin, N-terminal
Os05g0149701	Conserved hypothetical protein.	BT086859	C4J7R2	-
Os05g0149800	Similar to Discordia 1.	AK058366	B7UBT1	EF-hand-like domain
Os05g0149850	Non-protein coding transcript.	BT016490	NONE	-
Os05g0149900	Similar to tetratricopeptide repeat-containing protein.	AK119376	XP_002881630.1	Tetratricopeptide-like helical
Os05g0149950	Monothiol glutaredoxin-S8.	P0C290 (UniProt)	P0C290	Glutaredoxin
Os05g0150000	Proline synthetase co-transcribed bacterial homolog protein.	AK289260	XP_002889904.1	Alanine racemase, N-terminal
Os05g0150100	Conserved hypothetical protein.	AK071562	B9FHD8	-
Os05g0150300	Similar to Possible global transcription activator SNF2L1 (SWI/SNF related matrix associated actin dependent regulator of chromatin subfamily A member 1).	AK100732	XP_002315568.1	SNF2-related
Os05g0150400	Double-stranded RNA-binding domain containing protein.	AK110983	B9FMH4	Double-stranded RNA-binding
Os05g0150500	Similar to Transport inhibitor response 1.	EU400583	D0ES26	Leucine-rich repeat, cysteine-containing subtype
Os05g0150550	Hypothetical protein.	tplb0032b19 (Wheat FLcDNA)	longestORF	-
Os05g0150600	DNA helicase, ATP-dependent, RecQ type domain containing protein.	AK072977	D7U9T8	Helicase, C-terminal
Os05g0150733	Similar to pro-resilin.	ab initio prediction	NP_001148515.1	-
Os05g0150800	Similar to C-1-tetrahydrofolate synthase, cytoplasmic.	AK104502 ,AK060882	B6T3G6	Tetrahydrofolate dehydrogenase/cyclohydrolase, catalytic domain
Os05g0150900	Similar to Histidyl-tRNA synthetase (Fragment).	AK059520	Q9ZTW6	Aminoacyl-tRNA synthetase, class II
Os05g0151000	Similar to DNA-directed RNA polymerase.	AK371102	C5YGY9	RNA polymerase II, heptapeptide repeat, eukaryotic
Os05g0295100	Protein of unknown function DUF1253 family protein.	AK100239	B7F1E3	Digestive organ expansion factor, predicted
Os05g0295200	Conserved hypothetical protein.	AK073450	B9FNQ1	-
Os05g0295300	Similar to Acetyl-coenzyme A carboxylase (EC 6.4.1.2).	AK059261	O48959	Carboxyl transferase
Os05g0295500	Non-protein coding transcript.	BT019212	NONE	-
Os05g0295700	Mitochondrial matrix Mmp37 domain containing protein.	AK100560	B9FNQ2	Mitochondrial matrix Mmp37
Os05g0295800	Similar to Glyoxalase I (EC 4.4.1.5).	AK070232	NP_001146873.1	Glyoxalase/fosfomycin resistance/dioxygenase
Os05g0295900	Conserved hypothetical protein.	AK069962	B9FNQ4	-
Os06g0178900	Vacuolar H+-pyrophosphatase (EC 3.6.1.1) (Ovp2).	AK066933	P93410	Pyrophosphate-energised proton pump
Os06g0178950	Hypothetical protein.	tplb0034o06 (Wheat FLcDNA)	longestORF	-
Os06g0179000	Glycoside hydrolase family 79, N-terminal protein.	AK065263	A2Y9Y7	Glycoside hydrolase, family 79
Os06g0179050	Hypothetical gene.	BT085373	longestORF	-
Os06g0179100	Similar to H0306F03.13 protein.	EU963950	Q25A62	Sterile alpha motif/pointed domain
Os06g0179200	Similar to Nodulin-like protein.	AK101156	Q8H6R3	Nodulin-like
Os06g0179400	Hypothetical protein.	AK103645	longestORF	-
Os06g0179500	Protein of unknown function DUF247, plant family protein.	ab initio prediction	NP_001056969.1	Protein of unknown function DUF247, plant
Os06g0179700	Similar to DNA-binding protein phosphatase 2C.	AK065602	C4JAG1	Protein phosphatase 2C, manganese/magnesium aspartate binding site
Os06g0179800	Hypothetical conserved gene.	AK065817	Q5SMK3	Leucine-rich repeat-containing N-terminal, type 2
Os06g0179900	Major facilitator superfamily protein.	AK287929	NP_001147465.1	Major facilitator superfamily
Os06g0179950	Hypothetical protein.	AK369010	longestORF	-
Os06g0180000	Similar to Root determined nodulation 1.	AK069615	E9KID2	-
Os06g0180050	Hypothetical protein.	tplb0025h12 (Wheat FLcDNA)	longestORF	-
Os06g0180100	Hypothetical conserved gene.	ab initio prediction	Q8H604	-
Os06g0180300	Conserved hypothetical protein.	ab initio prediction	Q5SMJ6	-
Os06g0180533	Similar to predicted protein.	EU943442	XP_002321824.1	Cysteine, histidine-dependent amidohydrolase/peptidase
Os06g0180666	Hypothetical gene.	BT055164	longestORF	-
Os06g0181100	Octicosapeptide/Phox/Bem1p domain containing protein.	AK073372	NP_001056976.1	Phox/Bem1p
Os06g0181200	Serine/threonine protein kinase domain containing protein.	AK067771	XP_002877531.1	Protein kinase, catalytic domain
Os06g0181300	Similar to terminal acidic SANT 1.	AK241456	Q6T804	-
Os06g0181400	Similar to oxidoreductase.	AK242975	NP_001152353.1	FAD dependent oxidoreductase
Os06g0606599	Hypothetical protein.	ab initio prediction	NONE	-
Os06g0606700	Tetratricopeptide-like helical domain containing protein.	AK359397	Q69Q43	Tetratricopeptide-like helical
Os06g0606800	Similar to WDL1.	AK061909	B6U8B2	Xklp2 targeting protein
Os06g0606900	Conserved hypothetical protein.	AK068134	B9FU26	-
Os06g0607000	Similar to Beta-1,3-glucanase.	AK121115	A4PID2	Glycoside hydrolase, family 17
Os06g0607100	Similar to phosphatidic acid phosphatase-related / PAP2-related.	AK071250	NP_566527.1	-
Os06g0607200	Similar to Cellular retinaldehyde binding/alpha-tocopherol transport%3B Cellular retinaldehyde-binding/triple function, N-terminal.	AK242958	Q2HV32	Cellular retinaldehyde-binding/triple function, N-terminal
Os06g0607700	ABC transporter-like domain containing protein.	AK106792	C0J9X4	ABC transporter-like
Os06g0607750	Hypothetical protein.	BT084685	longestORF	-
Os06g0607800	Similar to 26S proteasome regulatory complex subunit p42D.	AK063158 ,AK066695	C0J9X5	ATPase, AAA+ type, core
Os06g0607850	Hypothetical protein.	tplb0035c03 (Wheat FLcDNA)	longestORF	-
Os06g0607900	GRAM domain containing protein.	AK070879	C0J9X6	GRAM
Os06g0608000	Similar to PGPS/D10.	AK119328	A3BDF7	-
Os06g0608050	Hypothetical gene.	AK241742	longestORF	-
Os06g0608100	Hypothetical conserved gene.	tplb0012d16 (Wheat FLcDNA)	NP_001058037.2	-
Os06g0635700	Conserved hypothetical protein.	AK107643	A3BDV0	-
Os06g0636100	Hypothetical conserved gene.	CT836245	Q67V17	Pathogenic type III effector avirulence factor Avr cleavage site
Os06g0636201	Conserved hypothetical protein.	EU968448	NP_001144438.1	-
Os06g0636600	Protein kinase, core domain containing protein.	AK119586	NP_001147925.1	Protein kinase, catalytic domain
Os06g0636700	Esterase, SGNH hydrolase-type domain containing protein.	AK058562	NP_849451.1	Lipase, GDSL
Os06g0677400	Similar to 3-hydroxyisobutyrate dehydrogenase.	AK073050	B6SHU3	3-hydroxyisobutyrate dehydrogenase-related, conserved site
Os06g0677500	Protein prenyltransferase domain containing protein.	AK102430	NP_001151503.1	Leucine-rich repeat
Os06g0677600	Like-Sm ribonucleoprotein, core family protein.	AK063252	NP_001147152.1	Like-Sm ribonucleoprotein
Os06g0677700	YT521-B-like protein family protein.	tplb0017e12 (Wheat FLcDNA)	NP_174334.2	Zinc finger, CCCH-type
Os06g0677800	Similar to P-167-1_1 (Fragment).	AK103280	Q653U3	AUX/IAA protein
Os06g0678100	Conserved hypothetical protein.	EU975100	NP_001058361.1	-
Os06g0678200	Similar to Geranyl diphosphate synthase.	AK071299 ,AK059456	B6TA22	Polyprenyl synthetase
Os06g0678500	Hypothetical conserved gene.	ab initio prediction	NP_001058363.2	-
Os06g0678650	WD40 repeat-like domain containing protein.	AK241346	NP_564728.2	WD40 repeat
Os06g0678651	Similar to WD-40 repeat family protein / beige-related.	EU954809	NP_564728.2	BEACH domain
Os06g0678700	Hypothetical conserved gene.	AK101184	B9FQH6	-
Os06g0678750	Non-protein coding transcript.	EU943604	NONE	-
Os06g0678800	Similar to Pollen-specific protein NTP303 precursor.	AK071465	B6ST56	Multicopper oxidase, type 1
Os06g0678875	Non-protein coding transcript.	tplb0021n10 (Wheat FLcDNA)	NONE	-
Os06g0680500	Similar to Glutamate receptor 3.4 precursor (Ligand-gated ion channel 3.4) (AtGLR4). Splice isoform 2.	AK099745	A3BEP2	Ionotropic glutamate receptor
Os06g0680700	Cytochrome P450 family protein.	AK064920	XP_002889880.1	Cytochrome P450
Os06g0680800	Hypothetical protein.	AK357549	longestORF	-
Os06g0680900	Exostosin-like family protein.	AK121510	A2YG89	Exostosin-like
Os06g0681000	Conserved hypothetical protein.	AK062780	B9FQI5	-
Os06g0681100	Hypothetical conserved gene.	ab initio prediction	Q653Y1	Domain of unknown function DUF629
Os06g0681200	Cupredoxin domain containing protein.	AK107980	Q4KXE1	Plastocyanin-like
Os06g0681300	PAK-box/P21-Rho-binding domain containing protein.	AK110977	B8B1G9	PAK-box/P21-Rho-binding
Os06g0681400	Ubiquitin domain containing protein.	AK121590	GeneMark	Ubiquitin
Os06g0681600	Similar to peroxidase 39.	AK071908	Q5U1K4	Plant peroxidase
Os06g0681700	Protein of unknown function DUF6, transmembrane domain containing protein.	AK104993	NP_187364.1	Protein of unknown function DUF914, eukaryotic

Os06g0707400	Similar to NBS-LRR disease resistance protein homologue.	AK067727	Q84KC2	-
Os06g0707700	NB-ARC domain containing protein.	AK100720	Q84KC8	Disease resistance protein
Os06g0707733	Disease resistance protein domain containing protein.	ab initio prediction	A2YGU6	Disease resistance protein
Os06g0707800	Similar to OSIGBa0148A10.13 protein.	AK102129	Q5Z9J2	Disease resistance protein
Os06g0708000	Similar to Isoform 2 of Mitogen-activated protein kinase 12.	AK070823	Q5Z9J0-2	-
Os06g0708050	Hypothetical gene.	EU949346	longestORF	-
Os06g0708075	Non-protein coding transcript.	tplb0059e09 (Wheat FLcDNA)	NONE	-
Os06g0708100	Similar to Carboxylesterase-like protein.	AK066901	A2YGU8	-
Os06g0708200	Hypothetical conserved gene.	AK068246	B9FQU2	-
Os06g0708300	Similar to RER1A protein.	AK243324	NP_001151498.1	Retrieval of early ER protein Rer1
Os06g0708400	Cyclophilin.	ab initio prediction	A2YGV1	Peptidyl-prolyl cis-trans isomerase, cyclophilin-type
Os06g0708500	Similar to Peptidyl-prolyl cis-trans isomerase.	AK072490	A2YGV2	Peptidyl-prolyl cis-trans isomerase, cyclophilin-type
Os06g0708600	Zinc finger, C2H2-like domain containing protein.	AK100915	E4MXX2	Zinc finger, C2H2-like
Os06g0708700	Similar to nodulin-like protein.	AK071974	B6SKJ2	Drug/metabolite transporter
Os06g0708832	Similar to arogenate dehydrogenase.	AK355541	NP_001147429.1	Prephenate dehydrogenase
Os06g0708900	Similar to zinc knuckle (CCHC-type) family protein.	AK100402	NP_193654.2	CBF1-interacting co-repressor CIR, N-terminal
Os06g0709000	NAD(P)-binding domain containing protein.	AK068653	NP_001147429.1	Prephenate dehydrogenase
Os06g0709100	Cyclin-like F-box domain containing protein.	AK070881	B6TRX2	F-box domain, cyclin-like
Os06g0709400	Conserved hypothetical protein.	AK108588	A3CGK2	-
Os06g0712800	Similar to Ankyrin-like protein.	AK121236	NP_190676.1	Protein of unknown function DUF248, methyltransferase putative
Os06g0712900	tRNA-dihydrouridine synthase domain containing protein.	AK106648	NP_201523.1	tRNA-dihydrouridine synthase
Os06g0713000	Zinc finger, B-box domain containing protein.	AK105957	NP_001150747.1	Zinc finger, B-box
Os06g0713100	Protein of unknown function DUF1640 family protein.	AK072606	B6UC57	Protein of unknown function DUF1640
Os06g0713201	Non-protein coding transcript.	BT087005	NONE	-
Os06g0713300	Conserved hypothetical protein.	AK106687	A3BFC6	-
Os06g0713400	Cyclin-like F-box domain containing protein.	AK100628	B9FQV7	F-box domain, cyclin-like
Os06g0713600	Non-protein coding transcript.	tplb0032e04 (Wheat FLcDNA)	NONE	-
Os06g0713800	Alpha-amylase isozyme 2A precursor (EC 3.2.1.1) (1,4-alpha-D-glucan glucanohydrolase).	AK059671 ,AK101018	Q0D9J1	Glycoside hydrolase, family 13
Os06g0713900	Hypothetical conserved gene.	AK242112	A2YGY3	-
Os06g0714000	Uncharacterised protein family UPF0183 domain containing	AK069538	XP_002439019.1	Uncharacterised protein family UPF0183
Os06g0714100	Complex 1 LYR protein family protein.	AK121079	NP_001148390.1	-
Os06g0714200	Similar to calcium dependent protein kinase1.	AK243187	NP_001105740.1	Protein kinase, catalytic domain
Os06g0714300	Auxin responsive SAUR protein family protein.	AK107043	XP_002318465.1	Auxin responsive SAUR protein
Os06g0714366	Hypothetical protein.	EU977023	longestORF	-
Os06g0714400	Conserved hypothetical protein.	AK108610	Q5NAL1	-
Os06g0714432	Non-protein coding transcript.	CT836310	NONE"	-
Os06g0714500	ATPase, AAA-type, core domain containing protein.	AK100047 ,AK119628	NP_001150200.1	ATPase, AAA+ type, core
Os06g0714600	ADP-ribosylation factor domain containing protein.	AK318603	A6MD16	Small GTPase superfamily
Os06g0714700	Conserved hypothetical protein.	CU406678	NP_001058577.1	-
Os06g0731700	Similar to predicted protein.	AK241390	Q5Z403	Tetratricopeptide-like helical
Os06g0731750	Hypothetical gene.	BT016392	longestORF	-
Os06g0731800	Clathrin light chain family protein.	AK069288	Q259D4	Clathrin light chain
Os06g0731900	Similar to Lysine ketoglutarate reductase trans-splicing related 1.	AK073333	B4FAK0	Protein of unknown function DUF707
Os06g0731950	Conserved hypothetical protein.	AK241967	NP_001175012.1	-
Os06g0732000	Similar to 60S ribosomal protein L35.	FP092387	NP_001148517.1	-
Os07g0124800	Similar to Chaperone protein dnaJ.	AK068837	B6U0R7	Heat shock protein DnaJ, N-terminal
Os07g0124900	Allergen V5/Tpx-1 related family protein.	AK060057	D5KR57	Allergen V5/Tpx-1-related
Os07g0125000	Allergen V5/Tpx-1 related family protein.	AK060005 ,AK104140	Q8LLU7	-
Os07g0125201	Allergen V5/Tpx-1 related family protein.	DQ167191	O04000	Allergen V5/Tpx-1-related
Os07g0125500	Allergen V5/Tpx-1 related family protein.	AK060057	D5KR57	Allergen V5/Tpx-1-related
Os07g0125600	Allergen V5/Tpx-1 related family protein.	AK060005 ,AK104140	Q8LLU7	-
Os07g0126100	Allergen V5/Tpx-1 related family protein.	DQ167191	O04000	Allergen V5/Tpx-1-related
Os07g0126301	Allergen V5/Tpx-1 related family protein.	AK060057	D5KR57	Allergen V5/Tpx-1-related
Os07g0126401	Allergen V5/Tpx-1 related family protein.	AK060005 ,AK104140	Q8LLU7	-
Os07g0126500	Allergen V5/Tpx-1 related family protein.	DQ167191	O04000	Allergen V5/Tpx-1-related
Os07g0146700	Hypothetical protein.	AK063631 ,AK101150	GeneMark	-
Os07g0146800	Non-protein coding transcript.	AK106695	NONE	-
Os07g0147500	Similar to Photosystem II 10 kDa polypeptide, chloroplast	AK062172 ,AK120691	Q40070	Photosystem II PsbR
Os07g0147550	Similar to Photosystem II 10 kDa polypeptide, chloroplast.	CT837962	Q40070	Photosystem II PsbR
Os07g0147600	Protein kinase, core domain containing protein.	AK099551	Q01JV1	Protein kinase, catalytic domain
Os07g0147700	ATPase, BadF/BadG/BcrA/BcrD type domain containing	AK242508	NP_001152135.1	ATPase, BadF/BadG/BcrA/BcrD type
Os07g0147800	Hypothetical conserved gene.	ab initio prediction	Q6ZF68	-
Os07g0147900	Similar to Ferredoxin--NADP reductase.	AK072768	A2YI62	Flavoprotein pyridine nucleotide cytochrome reductase
Os07g0150100	Protein of unknown function DUF221 domain containing protein.	AK098912 ,AK065241	NP_001108122.1	Domain of unknown function DUF221
Os07g0150200	Similar to 40S ribosomal protein S12-1.	AK121635 ,AK058680	B8B7B6	Ribosomal protein S12e
Os07g0150500	Zinc finger, C3HC-like domain containing protein.	AK073749	A2YI76	Zinc finger, C3HC-like
Os07g0150600	Non-protein coding transcript.	BT085109	NONE	-
Os07g0150700	Serine/threonine protein kinase, Pollination and drought stress responses	AK069726 (Genbank)	Q6ZLP5	Protein kinase, catalytic domain
Os07g0150901	Conserved hypothetical protein.	AK241621	B9FVI6	-
Os07g0151100	Similar to Glutaredoxin-C9.	Q7XIZ1 (UniProt)	Q7XIZ1	Glutaredoxin
Os07g0151200	Sugar/inositol transporter domain containing protein.	BT068655	XP_002893607.1	Sugar/inositol transporter
Os07g0151250	Hypothetical protein.	EU940776	longestORF	-
Os07g0151300	MAGE protein family protein.	AK102057	B4FM01	MAGE protein
Os07g0155600	Nramp ion-transporter family protein, Ethylene signaling pathway	AY396568	Q6TJY0	-
Os07g0156200	Haem peroxidase, plant/fungal/bacterial family protein.	AK249509	NP_001152255.1	Plant peroxidase
Os07g0156467	Similar to Class III peroxidase 7.	ab initio prediction	Q5U1T6	Plant peroxidase
Os07g0156732	Similar to EIN2.	AK111802	Q6TJY0	Natural resistance-associated macrophage protein
Os07g0156910	Similar to peroxidase 1.	ab initio prediction	NP_001152255.1	Haem peroxidase, plant/fungal/bacterial
Os07g0157000	Similar to Class III peroxidase 7.	FP094284	Q5U1T6	Plant peroxidase
Os07g0157401	Similar to EIN2.	AK111802	Q6TJY0	Natural resistance-associated macrophage protein
Os07g0157600	Similar to peroxidase 1.	AK252026	NP_001152255.1	Plant peroxidase
Os07g0157700	Conserved hypothetical protein.	AK069862	B8B7D5	-
Os07g0157900	Deoxyribonuclease, TatD domain containing protein.	AK067778	B6TDE6	Deoxyribonuclease, TatD-related
Os07g0158000	Hypothetical conserved gene.	ab initio prediction	A3BGQ5	-
Os07g0158100	Hypothetical gene.	AK108021	longestORF	-
Os07g0158200	Deoxyribonuclease, TatD Mg-dependent domain containing	AK103591 ,AK103532	B6TDE6	Deoxyribonuclease, TatD-related
Os07g0158300	Similar to RNA binding protein.	AK067376 ,AK099188 ,AK10477	O81989	RNA recognition motif domain
Os07g0158400	Hypothetical conserved gene.	EU966727	Q9LMF3	GCK
Os07g0158500	Similar to HAP2 subunit of HAP complex.	FP101566	B0S4T5	CCAAT-binding transcription factor, subunit B
Os07g0170800	Similar to F-box domain containing protein.	AK105849	NP_001151302.1	F-box domain, Skp2-like
Os07g0170900	Conserved hypothetical protein.	AK106442	A3BH03	-
Os07g0171100	Similar to predicted protein.	AK062132	XP_002891836.1	PDZ/DHR/GLGF
Os07g0171200	Similar to Galactose-1-phosphate uridyl transferase-like protein.	AK071075	B6TFN9	Galactose-1-phosphate uridyl transferase, class I
Os07g0171300	Protein kinase, core domain containing protein.	AK100663 ,AK066357	NP_001152463.1	Protein kinase, catalytic domain
Os07g0171350	Non-protein coding transcript.	BT084747	NONE	-
Os07g0172200	Similar to ROOT HAIRLESS 1.	AK103352	B6UAF0	-
Os07g0172500	Similar to DNA-directed RNA polymerase II 13.6 kDa polypeptide (EC 2.7.7.6).	AK068132 ,AK099622	B4FJS1	DNA-directed RNA polymerase Rpb11, 13-16kDa subunit, conserved site
Os07g0172600	Pentatricopeptide repeat domain containing protein.	AK369145	NP_001058999.1	Pentatricopeptide repeat
Os07g0172900	Hypothetical conserved gene.	AK067304	Q6ZA56	-
Os07g0173100	HSP20-like chaperone domain containing protein.	CT836094	NP_001059002.1	-
Os07g0173200	Frigida-like family protein.	AK061624	Q6ZA59	Frigida-like
Os07g0173300	Conserved hypothetical protein.	AK110935	A3BH18	-
Os07g0173400	Conserved hypothetical protein.	AK107434	Q6ZA58	-
Os07g0173501	Similar to 40S ribosomal protein S18.	CT837772	NP_001151612.1	Ribosomal protein S13
Os07g0173601	Hypothetical conserved gene.	ab initio prediction	Q8H589	-
Os07g0173700	40S ribosomal protein S18.	FP101812	NP_001151612.1	Ribosomal protein S13
Os07g0173850	Hypothetical gene.	CU406775	longestORF	-
Os07g0174200	Similar to 40S ribosomal protein S18.	tplb0009n18 (Wheat FLcDNA)	NP_001151612.1	Ribosomal protein S13
Os07g0174400	Similar to Non-specific lipid-transfer protein.	ab initio prediction	A2YIM6	Plant lipid transfer protein/Par allergen
Os07g0174700	Conserved hypothetical protein.	ab initio prediction	NP_001059008.2	-
Os07g0174766	Hypothetical conserved gene.	AK241687	B9F7S1	-
Os07g0515700	Similar to cDNA, clone: J100054L12, full insert sequence.	AK103117	B7FA83	Ankyrin repeat
Os07g0515900	Similar to cDNA, clone: J100054L12, full insert sequence.	ab initio prediction	B7FA83	Ankyrin repeat
Os07g0516000	Neuraminidase domain containing protein.	AK106685	NP_001078763.1	BNR repeat
Os07g0516050	Similar to predicted protein.	CT836059	NP_001078763.1	BNR repeat
Os07g0516100	Hypothetical conserved gene.	tplb0011b20 (Wheat FLcDNA)	A2YLU4	Protein phosphatase 2C
Os07g0516200	Similar to Endoribonuclease, L-PSP family.	AK061373	Q84UT6	YjgF-like protein
Os07g0516250	Non-protein coding transcript.	BT018946	NONE	-
Os07g0516300	Protein of unknown function DUF584 family protein.	AK102726 ,AK069570	Q01IB5	Protein of unknown function DUF584
Os07g0516400	Hypothetical conserved gene.	DQ244334	NP_001059783.2	-
Os07g0516500	Hypothetical conserved gene.	AK061672	B9F1Y0	-

Os07g0696100	Conserved hypothetical protein.	AK071018	B8BN83	-
Os08g0124500	Similar to Resistance protein candidate (Fragment).	AK098961	B7F8F0	Protein kinase, catalytic domain
Os08g0124533	Hypothetical protein.	tplb0022l18 (Wheat FLcDNA)	longestORF	-
Os08g0124566	Hypothetical protein.	tplb0022l18 (Wheat FLcDNA)	longestORF	-
Os08g0124600	Similar to J065032N17, full insert sequence.	AK369941	B7F8F0	Protein kinase, catalytic domain
Os08g0124651	Conserved hypothetical protein.	EU974589	Q6ZFR7	-
Os08g0124700	Similar to Resistance protein candidate (Fragment).	AK106798	B7F8F0	Protein kinase, catalytic domain
Os08g0124750	Hypothetical protein.	tplb0022l18 (Wheat FLcDNA)	longestORF	-
Os08g0124850	Hypothetical protein.	tplb0022l18 (Wheat FLcDNA)	longestORF	-
Os08g0125006	Hypothetical protein.	tplb0022l18 (Wheat FLcDNA)	longestORF	-
Os08g0125059	Hypothetical protein.	tplb0022l18 (Wheat FLcDNA)	longestORF	-
Os08g0519400	Apolipoprotein/apolipophorin domain containing protein.	AK070323	NP_567873.1	Apolipoprotein/apolipophorin
Os08g0519501	Hypothetical gene.	AK109201	longestORF	-
Os08g0519600	Similar to predicted protein.	AK072374	B9G1U1	Ribosomal protein L34Ae
Os08g0519700	Hypothetical conserved gene.	AK110779	A2YX44	Domain of unknown function DUF630
Os08g0519750	Hypothetical conserved gene.	EU950331	A2YX44	Domain of unknown function DUF632
Os08g0519800	Pumilio RNA-binding repeat domain containing protein.	AK064999	B9G1U3	Pumilio RNA-binding repeat
Os08g0519900	Conserved hypothetical protein.	ab initio prediction	NP_001062263.1	-
Os08g0520000	Mitochondrial substrate carrier family protein.	AK063556	NP_001151333.1	Mitochondrial carrier protein
Os08g0520100	Similar to pseudouridine synthase family protein.	AK059686	NP_178012.2	Pseudouridine synthase, RsuA and RluB/C/D/E/F
Os08g0520200	Similar to predicted protein.	AK288100	Q287W6	Tetratricopeptide-like helical
Os08g0520300	Similar to Oligouridylate binding protein.	AK069912	NP_001148487.1	RNA recognition motif domain
Os08g0520400	Conserved hypothetical protein.	AK119371 ,AK064309 ,AK10242	A2YX50	-
Os08g0520500	Similar to Isoform 2 of Auxin response factor 21.	AK059543	Q6YZW0-2	-
Os08g0520550	Similar to Auxin response factor 21.	AK111639	Q6YZW0	Transcriptional factor B3
Os08g0520600	Conserved hypothetical protein.	EU945787	NP_001062269.1	-
Os08g0520700	Non-protein coding transcript.	AK122042	NONE	-
Os08g0520850	Similar to chaperone protein dnaJ-related.	ab initio prediction	NP_850052.1	-
Os08g0520900	Similar to Isoamylase (Fragment).	AB015615	D0TZF9	Glycoside hydrolase, family 13, N-terminal
Os08g0521000	Protein of unknown function DUF599 family protein.	AK108985	A2YX57	Protein of unknown function DUF599
Os08g0533900	Hypothetical conserved gene.	AY302058 (DDBJ, Secondary hit)	Q94DG6	-
Os08g0534200	Similar to HEAT repeat family protein.	AK066926	B6TWZ9	-
Os08g0534300	Similar to calmodulin binding protein.	tplb0019a16 (Wheat FLcDNA)	NP_001149909.1	Auxin responsive SAUR protein
Os08g0534350	Similar to cation cation antiporter.	AK066324 (DDBJ, Secondary hit)	NP_001148426.1	Vesicle transport protein, Use1
Os08g0534400	EKC/KEOPS complex, subunit Pcc1 domain containing protein.	AK058382	NP_001172915.1	EKC/KEOPS complex, subunit Pcc1
Os08g0534900	Armadillo-type fold domain containing protein.	AK109343 ,AK061777	B6TWZ9	Armadillo-like helical
Os08g0534950	Similar to calmodulin binding protein.	tplb0019a16 (Wheat FLcDNA)	NP_001149909.1	Auxin responsive SAUR protein
Os08g0535000	Similar to cation cation antiporter.	AK066324	NP_001148426.1	Vesicle transport protein, Use1
Os08g0535050	Conserved hypothetical protein.	AK101573	A3BV79	-
Os09g0426800	Similar to Glossy1 protein.	AK060786 ,AK120227	NP_001105247.1	Fatty acid hydroxylase
Os09g0427100	Conserved hypothetical protein.	AK100693	B8BFB6	-
Os09g0427125	Hypothetical conserved gene.	CT835830	B8BFB5	-
Os09g0427300	Similar to amino acid carrier.	AK121239	NP_001149036.1	Amino acid transporter, transmembrane
Os09g0427551	Hypothetical gene.	AK242706	longestORF	-
Os09g0427800	Similar to Trehalose-6-phosphate synthase.	AK366091	B6T0F0	Glycosyl transferase, family 20
Os09g0428000	Glycosyl transferase, family 2 domain containing protein.	AF435650	NP_194887.1	Glycosyl transferase, family 2
Os09g0428266	Conserved hypothetical protein.	AK241163	NP_001175850.1	-
Os09g0428300	Similar to Glutamate receptor.	AK371221	C5XZJ7	GPCR, family 3
Os09g0428500	Conserved hypothetical protein.	AK060993	B9G3Q0	-
Os09g0428600	Similar to Glutamate receptor.	AK362522	C5XZJ7	GPCR, family 3
Os09g0428750	Conserved hypothetical protein.	AK241051	B9G3Q1	-
Os09g0428900	Similar to Conserved protein.	AK100970	B6U6F7	Uncharacterised domain UPF0066
Os09g0429000	Similar to Glutamate receptor.	AK371221	A2Z1H2	Ionotropic glutamate receptor
Os09g0567500	Similar to Fatty acyl coA reductase.	AK061142	Q8L4V2	Male sterility
Os09g0567600	Hypothetical protein.	AK106807	longestORF	-
Os09g0567700	WD40 repeat-like domain containing protein.	AK065913	NP_001105835.1	WD40 repeat
Os09g0567800	Hypothetical conserved gene.	CT835822	NP_187604.1	-
Os09g0567900	Inosine/uridine-preferring nucleoside hydrolase domain containing protein.	AK061415	B6T563	Inosine/uridine-preferring nucleoside hydrolase domain
Os09g0568000	Conserved hypothetical protein.	AK104856	Q652Q6	-
Os09g0568050	Hypothetical protein.	tplb0025f09 (Wheat FLcDNA)	longestORF	-
Os09g0568100	Conserved hypothetical protein.	FP092518	B9G564	-
Os09g0568200	Similar to DNA polymerase epsilon subunit 3.	AK242634	NP_001149275.1	Transcription factor CBF/NF-Y/archaeal histone
Os09g0568266	Non-protein coding transcript.	BT018987	NONE	-
Os09g0568400	Similar to Ubiquitin/ribosomal fusion protein (Fragment).	AK060445	Q7XYD4	Ribosomal protein L40e
Os09g0568500	Germin family protein.	AK108987	Q84XR7	Germin
Os09g0568600	Putative germin-like protein 9-2.	Q652Q0 (UniProt)	Q652Q0	Germin
Os09g0568700	RmlC-like jelly roll fold domain containing protein.	AK109010	Q84XR7	Germin
Os09g0568800	Similar to Ribosomal protein S25 (40S ribosomal 25S subunit).	AK059234	NP_001148017.1	Ribosomal protein S25
Os09g0568900	Similar to predicted protein.	AK059442	XP_002879140.1	-
Os09g0569000	Hypothetical gene.	AK108627	longestORF	-
Os09g0569100	Haloacid dehalogenase-like hydrolase domain containing protein.	AK071860	B4FI81	Haloacid dehalogenase-like hydrolase
Os09g0569151	Hypothetical protein.	AK355652	longestORF	-
Os09g0569200	Similar to Beta-amylase (EC 3.2.1.2) (1,4-alpha-D-glucan maltohydrolase).	AK070300	B6SVZ0	Glycoside hydrolase, family 14B, plant
Os09g0569300	Similar to calmodulin-binding heat-shock protein.	AK069587	NP_001149987.1	Lipase, class 3
Os09g0569400	Beta-lactamase-like domain containing protein.	AK070608 ,AK063384	B6U1S6	Beta-lactamase-like
Os09g0569450	Conserved hypothetical protein.	AK242543	NP_001175993.1	-
Os09g0569700	Conserved hypothetical protein.	AK108778	NP_001063979.1	-
Os09g0569780	Conserved hypothetical protein.	AK241041	NP_001175963.1	-
Os09g0569800	Serine/threonine protein kinase-related domain containing protein.	ab initio prediction	NP_001063980.1	Protein kinase, catalytic domain
Os09g0569900	Hypothetical protein.	AK073081	longestORF	-
Os09g0570000	Serine/threonine protein kinase-related domain containing protein.	AK058797	Q2HTK4	Protein kinase, catalytic domain
Os09g0570100	Hypothetical conserved gene.	AK059199	B9G572	Protein kinase, catalytic domain
Os09g0570150	Hypothetical conserved gene.	EU953102	B6SK37	-
Os09g0570200	Zinc finger, C2H2-type domain containing protein.	AK102035	A2Z489	Zinc finger, C2H2
Os09g0570300	Similar to Short-chain dehydrogenase Tic32.	AK058687	B4FKX6	Short-chain dehydrogenase/reductase SDR
Os09g0570400	Non-protein coding transcript.	AK061710	NONE	-
Os09g0570500	Zinc finger, RING-type domain containing protein.	AK069259	B9G575	Zinc finger, RING-type
Os09g0570600	Non-protein coding transcript.	AK105456	NONE	-
Os09g0570800	Isopenicillin N synthase family protein.	AK064211	NP_001149522.1	Isopenicillin N synthase
Os09g0570850	Similar to Histone H2B.	ab initio prediction	B6SGC3	Histone H2B
Os09g0570900	Similar to amino acid binding protein.	AK068397	B6TMA2	-
Os09g0570951	Hypothetical gene.	tplb0017n19 (Wheat FLcDNA)	longestORF	-
Os10g0162846	Similar to LRR14.	AK363200	Q9ATQ3	-
Os10g0162848	Non-protein coding transcript.	AK058701	NONE	-
Os10g0162852	Non-protein coding transcript.	AK058701	NONE	-
Os10g0410600	Similar to Phosphoprotein phosphatase 2A isoform 4.	AK099604	A3C4N5	Metallophosphoesterase domain
Os10g0410650	Hypothetical gene.	BT018461	longestORF	-
Os10g0410700	Similar to SET domain protein 123.	AK111981 ,AK111906	NP_001105199.1	SET domain
Os10g0410750	Protein kinase, catalytic domain domain containing protein.	EU975108	XP_002450423.1	Protein kinase, catalytic domain
Os10g0410900	Conserved hypothetical protein.	AK119279	Q7XER2	-
Os10g0411100	Protein of unknown function DUF620 domain containing protein.	BT088024	XP_002468447.1	Protein of unknown function DUF620
Os10g0411200	Hypothetical protein.	BT085554	longestORF	-
Os10g0411500	IQ calmodulin-binding region domain containing protein.	AK072572	B8BGS6	-
Os10g0411600	Hypothetical protein.	AK108669	longestORF	-
Os10g0411650	Non-protein coding transcript.	EU947431	NONE	-
Os10g0411700	Similar to S28 ribosomal protein (Fragment).	AK058209 ,AK121099	Q7X9K4	-
Os10g0411750	Hypothetical conserved gene.	AK241834	Q7XEQ4	JNK/Rab-associated protein-1, N-terminal
Os10g0411800	Similar to 40S ribosomal protein S17-3.	AK059434	NP_001149330.1	Ribosomal protein S17e
Os10g0412000	Similar to aminophospholipid ATPase.	AK062158	XP_002314626.1	ATPase, P-type, K/Mg/Cd/Cu/Zn/Na/Ca/Na/H-transporter
Os10g0412050	Similar to aminophospholipid ATPase.	BT035338	NP_176191.1	ATPase, P-type, ATPase-associated domain
Os10g0412100	Similar to Endonuclease/Exonuclease/phosphatase family protein, expressed.	AK105868	Q10R98	Endonuclease/exonuclease/phosphatase
Os10g0412350	Similar to H0315A08.1 protein.	FP099421	Q01I63	Ribonuclease H1, N-terminal
Os10g0542800	Similar to ATP binding protein.	AK121234	NP_001151339.1	Tetratricopeptide-like helical
Os10g0542900	Similar to chitinase.	AK070067	Q7XCK6	Glycoside hydrolase, family 19, catalytic
Os10g0543150	Conserved hypothetical protein.	ab initio prediction	NP_001176258.1	-
Os10g0543400	Chitinase 8.	Q7XCK6 (UniProt)	Q7XCK6	Glycoside hydrolase, family 19, catalytic
Os10g0543500	Six-bladed beta-propeller, TolB-like domain containing protein.	AK106264	Q109A9	Six-bladed beta-propeller, TolB-like
Os10g0543800	Similar to Glutathione S-transferase GST 11 (EC 2.5.1.18).	CT836577	NP_001104983.1	Glutathione S-transferase, N-terminal
Os10g0544200	Basic helix-loop-helix dimerisation region bHLH domain containing protein.	AK063669	NP_001149796.1	Helix-loop-helix DNA-binding
Os10g0544500	Similar to nucleotide binding protein.	AK060182	NP_001150616.1	WD40 repeat

Os10g0544600	Zinc finger, RING/FYVE/PHD-type domain containing protein.	AK119332	NP_001151464.1	Zinc finger, RING-type
Os10g0544900	Similar to Protein phosphatase 2C-like protein.	AK119635	B6TZR2	Protein phosphatase 2C, manganese/magnesium aspartate binding site
Os10g0544933	Non-protein coding transcript.	EU943254	NONE	-
Os10g0544950	Hypothetical gene.	AK242432	longestORF	-
Os10g0544966	Hypothetical protein.	tplb0043b08 (Wheat FLcDNA)	longestORF	-
Os10g0545000	Similar to magnesium transporter CorA-like family protein.	AK069472	NP_567076.1	Fibronectin-attachment
Os10g0545100	Similar to predicted protein.	AK287840	B9G1Z7	Uncharacterised conserved protein UCP014543
Os10g0545200	Similar to 4,4-dimethyl-sterol C4-methyl-oxidase (Fragment).	AK098884 ,AK061779	B4FN64	Fatty acid hydroxylase
Os10g0545300	Zinc finger, CCHC retroviral-type domain containing protein.	AK065156 ,AK121559	NP_001149324.1	Zinc finger, CCHC-type
Os10g0545500	Similar to xyloglucan endotransglucosylase/hydrolase protein 32.	FJ264508	NP_001148432.1	Glycoside hydrolase, family 16
Os10g0545600	Similar to transposon protein.	EU976069	B6UFQ4	Zinc finger, RING-type
Os10g0545700	Dual-specificity tyrosine-phosphatase CDC25, Arsenic [illegible]	AY860059 (Genbank)	Q336V5	-
Os10g0545800	Nucleic acid-binding, OB-fold domain containing protein.	AK103222	NP_001152237.1	CcmE/CycJ protein
Os10g0546100	Pollen Ole e 1 allergen and extensin domain containing protein.	AK240974	NP_001065213.1	Pollen Ole e 1 allergen/extensin
Os10g0546200	Glycosyl transferase, family 1 domain containing protein.	AK372742	Q9AV30	Glycosyl transferase, family 1
Os10g0546300	Protein of unknown function DUF2451, C-terminal domain containing protein.	AK103033	B9G6U9	Protein of unknown function DUF2451, C-terminal
Os10g0546400	Conserved hypothetical protein.	AK069919	B9G6V0	-
Os10g0546600	Similar to Chloroplast carotenoid epsilon-ring hydroxylase.	AK065689	NP_190881.2	Cytochrome P450
Os10g0556600	Similar to predicted protein.	EU971180	NP_171710.3	CCR4-Not complex, Not1 subunit, domain of unknown function DUF3819
Os10g0556700	Hypothetical conserved gene.	BT068414	Q10T37	-
Os10g0556801	Non-protein coding transcript.	CU406112	NONE"	-
Os10g0556900	Conserved hypothetical protein.	AK318544	NP_001176269.1	-
Os10g0557133	Similar to H0315A08.1 protein.	FP099421	Q01I63	Ribonuclease H1, N-terminal
Os10g0557366	Conserved hypothetical protein.	FP100733	A2XC28	-
Os10g0557600	Similar to GATA transcription factor 9.	CT837819	NP_001147669.1	Zinc finger, GATA-type
Os10g0557700	Conserved hypothetical protein.	AK241699	NP_001176270.1	-
Os10g0557750	Hypothetical genes.	ab initio prediction	NONE	-
Os10g0557800	Conserved hypothetical protein.	AK073483	B9G6Y4	-
Os10g0557900	Peptidase M10A and M12B, matrixin and adamalysin family [illegible]	CT835768	Q8LET7	Peptidase M10, metallopeptidase
Os10g0558100	Similar to Matrixin family protein, expressed.	AK362972	Q108Z3	Peptidase M10, metallopeptidase
Os10g0558125	Similar to Matrixin family protein, expressed.	AK335065	Q108Z3	Peptidase M10, metallopeptidase
Os10g0558200	2OG-Fe(II) oxygenase domain containing protein.	ab initio prediction	Q7XC87	Oxoglutarate/iron-dependent oxygenase
Os10g0558301	Hypothetical gene.	EU953848	longestORF	-
Os10g0558400	Similar to Oxidoreductase, 2OG-Fe oxygenase family protein.	BT085094	Q7XC87	Oxoglutarate/iron-dependent oxygenase
Os10g0558466	Hypothetical protein.	EU953848	longestORF	-
Os10g0558600	Pentatricopeptide repeat domain containing protein.	ab initio prediction	Q94LP5	Pentatricopeptide repeat
Os10g0558650	Non-protein coding transcript.	EU946768	NONE	-
Os10g0558700	Similar to Oxidoreductase, 2OG-Fe oxygenase family protein, expressed.	AK099678	Q336T3	Oxoglutarate/iron-dependent oxygenase
Os10g0558725	Hypothetical protein.	EU953848	longestORF	-
Os10g0559200	Similar to Oxidoreductase, 2OG-Fe oxygenase family protein, expressed.	AK066405	Q336T1	-
Os10g0559300	Hypothetical protein.	AK063546	longestORF	-
Os10g0559400	Similar to cDNA clone:J023138C05, full insert sequence.	AK065705	Q7XC75	-
Os10g0559450	Conserved hypothetical protein.	AK241769	NP_001176272.1	-
Os10g0559500	2OG-Fe(II) oxygenase domain containing protein.	AK108419	Q7XC75	Oxoglutarate/iron-dependent oxygenase
Os10g0559550	Conserved hypothetical protein.	ab initio prediction	B9G6Z3	-
Os10g0559600	Agenet domain containing protein.	AK066219	Q108Y7	Agenet
Os10g0559650	Hypothetical protein.	tplb0029f23 (Wheat FLcDNA)	longestORF	-
Os10g0559700	Similar to Agenet domain containing protein, expressed.	AK068632	Q7XC74	-
Os10g0559800	Protein of unknown function DUF547 domain containing protein.	AK105615	B9G6Z5	Domain of unknown function DUF547
Os10g0559833	Hypothetical protein.	tplb0038j02 (Wheat FLcDNA)	longestORF	-
Os10g0559866	Hypothetical protein.	ab initio prediction	NONE	-
Os10g0559900	Ribosomal protein L18/L5 domain containing protein.	AK070091	NP_849617.1	Ribosomal protein L18/L5
Os10g0560000	Similar to predicted protein.	AK059709	Q8SB99	Pleckstrin homology domain
Os10g0560200	Protein of unknown function Cys-rich family protein.	AK069913	B6SGC5	Uncharacterised protein family Cys-rich
Os10g0560400	Similar to CONSTANS-like protein CO9 (Fragment).	AK109732	Q8SBA3	CCT domain
Os10g0560450	Similar to Autophagy-related protein 3.	AK241567	B9G6Z8	Autophagy-related protein 3, N-terminal
Os10g0560451	Hypothetical gene.	EU940960	longestORF	-
Os10g0560500	Conserved hypothetical protein.	AK100157	Q8SBA5	-
Os10g0560600	Non-protein coding transcript.	AK064207	NONE	-
Os10g0560700	Conserved hypothetical protein.	CT837759	NP_001065380.1	-
Os10g0560850	Hypothetical gene.	EU940765	longestORF	-
Os10g0560900	Pyridoxal phosphate-dependent transferase, major region, subdomain 1 domain containing protein.	AK071526	B6TFA9	Aminotransferase, class I/classII
Os10g0561000	Hypothetical conserved gene.	BT064708	Q9AUT7	-
Os10g0561100	Similar to Quinone oxidoreductase.	AK110882	Q10T48	Alcohol dehydrogenase superfamily, zinc-type
Os10g0580500	Essential protein Yae1, N-terminal domain containing protein.	CT837648	NP_001045408.2	Essential protein Yae1, N-terminal
Os10g0580600	Protein of unknown function DUF1997 domain containing [illegible]	AK370224	Q9FRM6	Protein of unknown function DUF1997
Os10g0580700	Ankyrin repeat containing protein.	AK071604	NP_193650.2	Ankyrin repeat
Os10g0580750	Hypothetical gene.	BT019171	longestORF	-
Os10g0580800	Similar to NClpP4 (Fragment).	AK104823 ,AK065306 ,AK10439[illegible]	B4FP39	Peptidase S14, ClpP
Os10g0580900	Non-protein coding transcript.	AK062821	NONE	-
Os10g0581000	Conserved hypothetical protein.	AK242551	B9G779	-
Os11g0127600	No apical meristem (NAM) protein domain containing protein.	AK067922	Q5CD17	No apical meristem
Os11g0127700	Conserved hypothetical protein.	AK103742	A2ZAZ8	-
Os11g0127800	Similar to FHA domain containing protein, expressed.	AK104250 ,AK104015	Q2QYC9	Forkhead-associated
Os11g0127900	Similar to 40S ribosomal protein S16.	AK059550	Q0IQF7	Ribosomal protein S9
Os11g0127951	Hypothetical protein.	ab initio prediction	NONE	-
Os11g0128000	Similar to secondary cell wall-related glycosyltransferase family [illegible]	AK243483	NP_001151894.1	Exostosin-like
Os11g0128300	Similar to ZF-HD homeobox protein (Fragment).	AK063715	Q2QYC5	ZF-HD homeobox protein, Cys/His-rich dimerisation domain
Os11g0128400	CDC45-like protein family protein.	AK102291	NP_189146.1	CDC45 family
Os11g0128500	Similar to Myb factor.	AK241549	O04109	SANT domain, DNA binding
Os11g0128600	Conserved hypothetical protein.	ab initio prediction	NP_001065637.1	-
Os11g0128700	Mad3/BUB1 homology region 1 domain containing protein.	AK102502 (DDBJ, Secondary hit)	Q2RB24	Mad3/BUB1 homology region 1
Os11g0128800	Similar to Homoserine dehydrogenase-like protein.	AK071434	B8AES5	Homoserine dehydrogenase, catalytic
Os11g0128932	Hypothetical gene.	BT054648	longestORF	-
Os11g0129000	Similar to Transparent testa 12 protein.	AK067875 (DDBJ, Secondary hit)	B6SSE0	Multi antimicrobial extrusion protein
Os11g0129101	Non-protein coding transcript.	BT054648	NONE	-
Os11g0129200	Multi antimicrobial extrusion protein MatE family protein.	AK067521 (DDBJ, Secondary hit)	Q2RB20	Multi antimicrobial extrusion protein
Os11g0129301	Hypothetical gene.	BT054648	longestORF	-
Os11g0129500	Similar to GDSL-like Lipase/Acylhydrolase family protein, [illegible]	AK107889	Q2RB18	-
Os11g0129600	Hypothetical conserved gene.	AK241463	NP_001066048.1	Engulfment/cell motility, ELMO
Os11g0129700	Similar to AP2-1 protein (Fragment).	AK112088	Q2RB16	-
Os11g0129800	Hypothetical conserved gene.	AK287524	B8BIV0	-
Os11g0130100	Conserved hypothetical protein.	AK063914	Q2RB13	-
Os11g0130200	Protein of unknown function DUF309 family protein.	AK059458	B8BIV2	Protein of unknown function DUF309
Os11g0130300	Nse1 non-SMC component of SMC5-6 complex family protein.	AK101224	Q2QYA0	Nse1 non-SMC component of SMC5-6 complex
Os11g0130400	Harpin-induced 1 domain containing protein.	AK243509	NP_001065651.2	Late embryogenesis abundant protein, LEA-14
Os11g0130500	Cyclin-like F-box domain containing protein.	AK241516	Q2RB09	F-box domain, cyclin-like
Os11g0130600	Conserved hypothetical protein.	AK066342	NP_001066054.1	-
Os11g0130700	Conserved hypothetical protein.	ab initio prediction	B9G976	-
Os11g0130800	Similar to Ulp1 protease family, C-terminal catalytic domain containing protein, expressed.	AK242802	Q2QY95	-
Os11g0130900	Similar to NB-ARC domain containing protein, expressed.	tplb0036i24 (Wheat FLcDNA)	Q2RB05	Disease resistance protein
Os11g0131100	VQ domain containing protein.	CT835703	Q2RB04	VQ
Os11g0131200	Similar to Mpv17/PMP22 family protein, expressed.	AK240867	Q2RB03	Mpv17/PMP22
Os11g0131300	Hypothetical conserved gene.	ab initio prediction	Q2RB02	F-box domain, cyclin-like
Os11g0131400	Hypothetical conserved gene.	AK109466 (DDBJ, Secondary hit)	Q2RB01	Pentatricopeptide repeat
Os11g0131500	Hypothetical protein.	BT085496	longestORF	-
Os11g0131600	Similar to YDG/SRA domain containing protein.	BT055690	Q2RB00	SET domain
Os11g0131700	Zinc finger, Tim10/DDP-type domain containing protein.	AK243327	B6SLJ2	Mitochondrial inner membrane translocase complex, Tim8/9/10/13-zinc finger-[illegible]
Os11g0131800	Similar to H0114G12.9 protein.	AK288048	A8B8B1	-
Os11g0131900	Similar to alpha-N-arabinofuranosidase A.	AK061612	B6T9B9	Alpha-L-arabinofuranosidase, C-terminal
Os11g0132000	Similar to Arabinoxylan arabinofuranohydrolase isoenzyme [illegible]	AK099668	Q9ATV7	-
Os11g0132100	Similar to Pectinesterase inhibitor domain containing protein.	DQ245602	Q2RAZ5	Pectinesterase inhibitor
Os11g0312400	Adenylate kinase B (EC 2.7.4.3) (ATP-AMP [illegible]	AK073605	Q08480	Adenylate kinase
Os11g0323860	Hypothetical conserved gene.	AK243521	B9GCL3	-
Os11g0334938	Similar to Class III peroxidase 111.	BT085722	B6TU39	Plant peroxidase
Os11g0340477	Zinc finger, C2H2-type domain containing protein.	AK108997	A2XIC5	Zinc finger, C2H2
Os11g0346016	Similar to calmodulin binding protein.	AK372659	NP_001151502.1	Calmodulin binding protein-like
Os11g0351555	Similar to A.thaliana gene induced upon wounding stress.	AK060848	D6NIX1	NAD
Os11g0357094	Pentatricopeptide repeat domain containing protein.	ab initio prediction	A2XIB7	Pentatricopeptide repeat

Os11g0362633	Hypothetical conserved gene.	FP093303	A9NKD9	Protein of unknown function DUF3743
Os11g0368172	Peptidase S8 and S53, subtilisin, kexin, sedolisin domain containing protein.	AK107787	A9NKD9	Protein of unknown function DUF3743
Os11g0373711	Hypothetical gene.	AK101901	longestORF	-
Os11g0379251	Similar to calmodulin binding protein.	AK372659	NP_001151502.1	Calmodulin binding protein-like
Os11g0393097	Similar to chlorophyll a-b binding protein M9, chloroplastic precursor.	AJ234427	NP_001105545.1	-
Os11g0406945	Similar to Phenazine biosynthesis-like protein, expressed.	AK333982	Q10J41	Phenazine biosynthesis PhzC/PhzF protein
Os11g0412484	Hypothetical conserved gene.	AK243521	B9GCL3	-
Os12g0177500	Peptidase A1 domain containing protein.	AK064771	Q2QWY6	Peptidase A1
Os12g0177800	Similar to D-mannose binding lectin family protein, expressed.	AK105397	Q2QWY4	S-locus glycoprotein
Os12g0177900	Similar to Aldehyde dehydrogenase.	ab initio prediction	Q2QWY3	Aldehyde dehydrogenase, N-terminal
Os12g0178000	Similar to Aldehyde dehydrogenase family protein, expressed.	AK241698	Q2QWY3	Aldehyde dehydrogenase NAD
Os12g0178100	Haem peroxidase family protein.	AK061107 ,AK099201	Q9XPR6	Haem peroxidase, plant/fungal/bacterial
Os12g0178200	Similar to Thylakoid-bound ascorbate peroxidase (EC 1.11.1.11) (Fragment).	AK073910	Q01IY9	Haem peroxidase, plant/fungal/bacterial
Os12g0178250	Similar to dehydration-responsive protein-related.	ab initio prediction	NP_001031109.1	Protein of unknown function DUF248, methyltransferase putative
Os12g0178300	Similar to ankyrin-like protein.	ab initio prediction	NP_001151565.1	Protein of unknown function DUF248, methyltransferase putative
Os12g0178500	Similar to seven-transmembrane-domain protein 1.	FP100089	Q2QWX8	RAG1-activating protein-1-related
Os12g0178600	Similar to dehydration-responsive protein-related.	ab initio prediction	NP_190676.1	Protein of unknown function DUF248, methyltransferase putative
Os12g0178633	WD40 repeat domain containing protein.	AK369075	B8BNG0	WD40 repeat
Os12g0178700	Similar to ARC5 (ACCUMULATION AND REPLICATION OF CHLOROPLAST 5)%3B GTP binding / GTPase.	AK072318	NP_188606.2	Dynamin, GTPase domain
Os12g0178800	Hypothetical protein.	AK063709	longestORF	-
Os12g0179100	Hypothetical gene.	AK106975	longestORF	-
Os12g0179700	Domain of unknown function DUF1618 domain containing [illegible]	AK073945 ,AK120612	Q2QWX0	Domain of unknown function DUF1618
Os12g0179800	NAD-dependent histone deacetylase, silent information regulator Sir2 domain containing protein.	AK067069	XP_002306275.1	NAD-dependent deacetylase, sirtuin family
Os12g0199800	Similar to Cyt-P450 monooxygenase.	AK288730	Q8S3F6	Cytochrome P450
Os12g0469300	Non-protein coding transcript.	tplb0015j08 (Wheat FLcDNA)	NONE	-
Os12g0522516	Similar to ATRL5 (ARABIDOPSIS RAD-LIKE 5)%3B DNA binding / transcription factor.	tplb0003e07 (Wheat FLcDNA)	NP_564087.2	SANT domain, DNA binding
Os12g0524201	Hypothetical conserved gene.	AK288953	B6U883	-
Os12g0524750	Similar to ribosomal protein S7.	ab initio prediction	YP_514635.1	Ribosomal protein S7
Os12g0525300	Hypothetical protein.	ab initio prediction	NONE	-
Os12g0600400	Similar to Pyridoxal kinase.	AK066514	Q69G22	Pyridoxal phosphate
Os12g0600550	Non-protein coding transcript.	AK288257	NONE	-
Os12g0600701	Conserved hypothetical protein.	CU406704	NP_001177048.1	-
Os12g0601000	Similar to Leucine Rich Repeat family protein, expressed.	EU973703	Q2QMK5	F-box domain, Skp2-like
Os12g0601200	Similar to ATP binding protein.	AK073304	B6TCV6	Phosphoribulokinase/uridine kinase
Os12g0601300	Similar to Auxin-responsive protein (Aux/IAA) (Fragment).	AK068213	P0C132	AUX/IAA protein
Os12g0601400	Similar to Auxin-responsive protein (Aux/IAA) (Fragment).	AK073361	P0C133	AUX/IAA protein
Os12g0601500	Conserved hypothetical protein.	AK242321	NP_001177049.1	-
Os12g0601800	Similar to BZIP transcription factor family protein, expressed.	AK287981	Q2QMJ7	Basic-leucine zipper
Os12g0602200	Conserved hypothetical protein.	AK119494	NP_001059593.2	-
Os12g0613000	Similar to NF-YA subunit.	AK065163	B6TY54	CCAAT-binding transcription factor, subunit B
Os12g0613100	Similar to Amino acid permease family protein.	AK332141	Q2QM92	Amino acid/polyamine transporter I
Os12g0613150	Hypothetical protein.	tplb0044b08 (Wheat FLcDNA)	longestORF	-
Os12g0613200	Hypothetical conserved gene.	AK069599	B9GE94	SET domain
Os12g0613250	Similar to BTB/POZ%3B Superoxide dismutase, copper/zinc binding%3B NPH3.	EU368707	A2Q365	BTB/POZ-like
Os12g0613300	Similar to Single myb histone 6.	AK105660	Q761Y7	SANT domain, DNA binding
Os12g0613500	Similar to SKP40, C-terminal domain containing protein, [illegible]	AK058205	Q2QM88	LisH dimerisation motif
Os12g0613600	Remorin, C-terminal region domain containing protein.	AK111002	Q2QM85	Remorin, C-terminal
Os12g0613700	Similar to Auxin response factor 25.	AK065025	Q2QM84	DNA-binding pseudobarrel domain
Os12g0613850	Similar to Galactosyltransferase family.	ab initio prediction	B6SVW0	Galectin, carbohydrate recognition domain
Os12g0614000	Conserved hypothetical protein.	AK068768	B8BN00	-
Os12g0614050	Hypothetical gene.	BT019058	longestORF	-
Os12g0614100	Similar to Lipase family protein.	BT042072	Q2QM82	Lipase, class 3
Os12g0614200	Similar to Lipase family protein.	BT042072	Q2QM81	Lipase, class 3
Os12g0614201	Conserved hypothetical protein.	AK243381	NP_001177060.1	-
Os12g0641200	Hypothetical protein.	AK064961	longestORF	-
Os12g0641300	Similar to Zn-dependent hydrolases of the beta-lactamase fold.	AK061015	C3TX72	Beta-lactamase-like
Os12g0641400	Similar to Sucrose transporter.	AK067030	Q0ILJ3	Major facilitator superfamily domain, general substrate transporter
Os12g0641500	Similar to RecF/RecN/SMC N terminal domain containing protein, expressed.	AK065733	Q2QLI0	RecF/RecN/SMC
Os12g0641600	Non-protein coding transcript.	AK289049	NONE	-

Pedigree-based genome re-sequencing reveals genetic variation patterns of elite backbone varieties during modern rice improvement

Xingfei Zheng, Lanzhi Li, Fan Liang, Changjun Tan, Shuzhu Tang, Sibin Yu, Ying Diao, Shuangcheng Li,and Zhongli Hu

Supplementary Table S13 Cloned genes from that identified in zero-diversity genes and low-diversity regions and sweeps from SH527 pedigree

Phenotypic classification	Gene ID	Gene symbols	Low-diveristy DNA regions			Zero-diversity genes			Sweeps from SH527 pedigree
			GC2H	HHZ	SH527	GC2H	HHZ	SH527	
	Os02g0152500	*LC2*	×	×	×	√	√	√	×
	Os04g0469800	*d11; CYP724B1*	×	×	×	×	√	×	×
	Os01g0257300	*OsRAA1*	×	×	×	√	√	×	×
	Os02g0134300	*OsTEF1*	×	×	×	√	√	×	×
	Os07g0153600	*PROG1*	×	×	×	√	√	√	×
	Os08g0509600	*OsSPL14*	×	×	×	√	×	×	×
	Os09g0114500	*BC12*	×	×	×	√	√	√	×
	Os09g0395300	*SLL1*	×	×	×	√	√	√	×
	Os03g0203200	*HTD2*	√	×	×	×	×	×	×
	Os06g0704300	*LIC*	×	√	×	×	×	×	×
Plant architecture and yield	Os06g0677000	*DEP3*	×	√	×	×	×	×	×
	Os06g0139000	*DDF1*	×	√	×	×	×	×	×
	Os01g0667400	*DWT1*	×	√	×	×	×	×	×
	Os03g0706500	*OsTB1; FC1*	√	×	×	×	×	×	×
	Os07g0685800	*OsSDR*	√	×	×	×	×	×	×
	Os03g0110800	*OsDRM2*	√	√	×	×	×	×	×
	Os12g0641400	*OsSUT2*	√	√	√	×	×	×	×
	Os07g0129700	*d6*	×	×	×	×	×	×	√
	Os07g0261200	*Ghd7/Hd4*	×	×	×	×	×	×	√
	Os03g0171300	*PGL1*	√	×	×	×	×	×	×
	Os04g0671900	*OsARF12*	√	√	√	×	×	×	×
	Os04g0457800	*OsSERK2*	×	×	×	√	√	×	×
	Os03g0805100	*SQS*	×	√	√	×	√	√	×
	Os06g0208800	*LYP6*	×	×	×	√	√	√	×
	Os07g0186000	*OsTRXh1*	×	√	×	×	×	×	×
Desease Resistance	Os01g0917400	*C3H12*	×	√	√	×	×	×	×
	Os03g0125100	*DSM2*	√	√	√	×	×	×	×
	Os06g0130100	*OsSIK1*	×	×	×	×	×	×	√
	Os07g0257200	*OsNRAMP5*	×	×	×	×	×	×	√
	Os09g0516200	*RF2a; OsbZIP75*	√	×	×	×	×	×	×
	Os08g0454000	*OsDERF1*	×	×	×	√	√	√	×
	Os03g0835800	*GPA3*	×	×	×	√	×	√	×
	Os05g0329200	*Prol14*	√	√	×	√	√	√	×
Grain quality	Os07g0206500	*RM1*	×	×	×	√	×	×	×
	Os09g0569200	*GPA3*	×	√	√	×	×	×	×
	Os03g0766100	*RP10*	√	×	×	×	×	×	×
	Os06g0133000	*wx*	×	×	×	×	×	×	√
	Os08g0162100	*OsLIS-L1; ASP1;*	√	×	×	×	×	×	×
Pollen development	Os07g0151100	*MIL1*	×	×	√	×	×	×	×
	Os01g0274800	*CSA*	×	×	×	√	√	√	×
	Os08g0494100	*MADS62*	√	×	×	√	×	√	×
	Os07g0204900	*PHS2*	×	×	×	√	×	×	×
	Os04g0670900	*sh4; SHA1*	√	√	√	√	√	√	×
	Os03g0112700	*Ehd4*	√	√	×	×	×	×	×
Others	Os01g0927000	*SDG714*	×	×	√	×	×	×	×
	Os04g0463400	*OsVIT1*	×	×	×	√	√	√	×
	Os09g0396900	*OsVIT2*	×	×	×	√	√	√	×
	Os04g0518800	*LABA1; An-2;*	√	√	√	×	×	×	×

Note: " √ " means that genes are found in pedigree; "×" means that genes are not found in pedigree

Pedigree-based genome re-sequencing reveals genetic variation patterns of elite backbone varieties during modern rice improvement
Xingfei Zheng, Lanzhi Li, Fan Liang, Changjun Tan, Shuzhu Tang, Sibin Yu, Ying Diao, Shuangcheng Li,and Zhongli Hu

Supplementary Table S14 Zero-diversity genes and their functional annotations during pedigree breeding

Gene name	Nr annotation	Transcript evidence	ORF evidence	InterPro annotation
**GC2H pedigree**				
Os01g0113150	Disease resistance protein domain containing protein.	AK367884	A2ZNH1	Disease resistance protein
Os01g0114050	Non-protein coding transcript.	EU941912	NONE	-
Os01g0121300	Conserved hypothetical protein.	AK103404	B8ACZ7	-
Os01g0121400	Hypothetical protein.	tplb0026h09 (Wheat FLcDNA)	longestORF	-
Os01g0121500	Conserved hypothetical protein.	AK107776	A2WK24	-
Os01g0122200	Seven In Absentia Homolog-type domain containing protein.	AK107171	A2WK31	Seven-in-absentia protein, sina
Os01g0122367	Hypothetical protein.	AK365307	longestORF	-
Os01g0133766	Non-protein coding transcript.	X02683	NONE	-
Os01g0152900	Similar to Histone H2B.1.	CT837552	B9A1G8	Histone H2B
Os01g0154200	Protein of unknown function DUF674 domain containing protein.	FP098916	Q5ZCE4	Protein of unknown function DUF674
Os01g0154250	Hypothetical protein.	BT085097	longestORF	-
Os01g0154550	Non-protein coding transcript.	AK288511	NONE	-
Os01g0155200	Similar to H0525E10.8 protein.	AK252147	Q01K00	Protein kinase, catalytic domain
Os01g0155500	Protein kinase, catalytic domain domain containing protein.	AK099840	A2ZPF5	Protein kinase, catalytic domain
Os01g0155600	Similar to Splicing factor RSZ33.	AK071635	A6MZ96	RNA recognition motif domain
Os01g0156300	Similar to Cappuccino protein.	AK107993	A2WKV9	Protein of unknown function DUF1110
Os01g0157200	Conserved hypothetical protein.	AK108577	A2ZPG5	-
Os01g0157400	Conserved hypothetical protein.	EU974248	Q5ZCC9	-
Os01g0157500	Protein of unknown function DUF1110 domain containing protein.	EU965613	NP_001172188.1	Protein of unknown function DUF1110
Os01g0157600	Conserved hypothetical protein.	AK106766	Q9FYQ2	-
Os01g0157700	Glycosyl transferase, family 43 domain containing protein.	AK376884	Q5ZCC5	Glycosyl transferase, family 43
Os01g0159600	Small hydrophilic plant seed protein family protein.	AK122153 ,AK064055	Q3I3Z0	Stress induced protein
Os01g0159800	Similar to DNA binding protein.	AK106292	NP_001152068.1	Helix-loop-helix DNA-binding
Os01g0164650	Non-protein coding transcript.	EU944689	NONE	-
Os01g0165100	Conserved hypothetical protein.	AK103011 ,AK059335	A2WL20	-
Os01g0165600	Similar to H0323C08.17 protein.	AK335607	Q942P3	-
Os01g0166700	Saposin family protein.	AK067805	B6T780	Saposin-like type B, 1
Os01g0172400	Similar to Phospholipase D alpha 1.	AK066556	B8ADH7	Phospholipase D/Transphosphatidylase
Os01g0172900	Glycoside hydrolase, family 28 domain containing protein.	ab initio prediction	B9ETD9	Glycoside hydrolase, family 28
Os01g0173000	Putative thiol-disulphide oxidoreductase DCC domain containing protein.	AK287669	GeneMark	Putative thiol-disulphide oxidoreductase DCC
Os01g0173100	Alba, DNA/RNA-binding protein family protein.	AK121114	NP_564325.1	DNA/RNA-binding protein Alba-like
Os01g0173950	Non-protein coding transcript.	tplb0011b09 (Wheat FLcDNA)	NONE	-
Os01g0176300	Pentatricopeptide repeat domain containing protein.	AK249469	NP_001042180.1	Pentatricopeptide repeat
Os01g0177150	Non-protein coding transcript.	AK288777	NONE	-
Os01g0178300	Hypothetical protein.	AK062450	GeneMark	-
Os01g0178400	Similar to Protein phosphatase 2A B'kappa subunit.	AK108850	XP_002885457.1	Protein phosphatase 2A, regulatory B subunit, B56
Os01g0178500	Similar to Auxin-responsive protein (Aux/IAA) (Fragment).	AK109373 ,AK060091 ,AK06924	Q5VRD1	-
Os01g0178700	Similar to Protein binding protein.	AK103147	B6TV66	Zinc finger, RING-type
Os01g0178775	Non-protein coding transcript.	tplb0030e06 (Wheat FLcDNA)	NONE	-
Os01g0178900	Similar to Symbiosis-related disease resistance protein (Fragment).	AK110770	Q8RVG6	-
Os01g0179000	Transferase family protein.	AK288049	B6TZV1	Transferase
Os01g0179600	UDP-glucuronosyl/UDP-glucosyltransferase family protein.	AK243646	NP_001042201.2	UDP-glucuronosyl/UDP-glucosyltransferase
Os01g0179750	Non-protein coding transcript.	BT019232	NONE	-
Os01g0181033	Similar to NADH dehydrogenase subunit 1.	AJ010976	YP_024343.1	NADH:ubiquinone oxidoreductase, subunit 1/F420H2 oxidoreductase subunit H
Os01g0182300	Lipocalin domain containing protein.	AK062827	A2WLF0	-
Os01g0187200	Non-protein coding transcript.	AK110797	NONE	-
Os01g0194000	Conserved hypothetical protein.	AK071994	A2WLM7	-
Os01g0206650	Hypothetical protein.	tplb0042a18 (Wheat FLcDNA)	longestORF	-
Os01g0206700	Similar to Serine/threonine protein kinase (CBL-interacting protein kinase 20).	AK065589	Q9LWM4	Protein kinase, catalytic domain
Os01g0206800	Protein kinase, core domain containing protein.	AK100525	NP_001186540.1	Protein kinase, catalytic domain
Os01g0207001	Hypothetical protein.	tplb0058b08 (Wheat FLcDNA)	longestORF	-
Os01g0208400	Similar to peptide-N4-asparagine amidase A.	AK105750	NP_001152324.1	Peptide-N4-
Os01g0208800	Similar to Phosphoenolpyruvate carboxylase (Fragment).	AK109734	longestORF	-
Os01g0211200	Cytochrome P450 family protein.	ab initio prediction	Q2MIZ9	Cytochrome P450
Os01g0224700	Flavin monooxygenase-like enzyme, Auxin biosynthesis	AK070386	Q9LG41	FAD-dependent pyridine nucleotide-disulphide oxidoreductase
Os01g0225200	Similar to predicted protein.	AK121971	XP_002868685.1	GTP-binding domain, HSR1-related
Os01g0225400	Pyruvate/Phosphoenolpyruvate kinase, catalytic core domain containing protein.	AK242525	NP_001150103.1	Ketopantoate hydroxymethyltransferase
Os01g0225600	Similar to Dehydrin.	CU405989	Q5CAQ2	Late embryogenesis abundant protein, LEA-14
Os01g0233850	Hypothetical gene.	EU943630	longestORF	-
Os01g0235400	Similar to predicted protein.	AK064696	Q9ZPY7	Importin-beta, N-terminal
Os01g0236700	Hypothetical conserved gene.	AK061701	Q5NB82	Phox/Bem1p
Os01g0243400	Hypothetical conserved gene.	AK070651	A2ZR73	-
Os01g0243450	Non-protein coding transcript.	FP095602	NONE	-
Os01g0243600	Conserved hypothetical protein.	EU974727	NP_001042565.1	-
Os01g0243901	Hypothetical protein.	AK376036	longestORF	-
Os01g0244150	Similar to J075123K08, full insert sequence.	AK374764	B7F983	Domain of unknown function DUF1618
Os01g0244400	Protein of unknown function DUF1618 domain containing protein.	AK242054	NP_001172253.1	Domain of unknown function DUF1618
Os01g0245000	Conserved hypothetical protein.	AK372630	Q5NA94	-
Os01g0245600	Conserved hypothetical protein.	ab initio prediction	NP_001042571.2	-
Os01g0245700	Domain of unknown function DUF1618 domain containing protein.	AK373668	NP_001172254.1	Domain of unknown function DUF1618
Os01g0245901	Domain of unknown function DUF1618 domain containing protein.	ab initio prediction	Q9XHZ1	Domain of unknown function DUF1618
Os01g0246400	Similar to Low molecular mass early light-inducible protein HV90, chloroplast precursor (ELIP).	AK062972	NP_001152927.1	Chlorophyll A-B binding protein
Os01g0246601	Protein of unknown function DUF296 domain containing protein.	BT064989	NP_001150963.1	Domain of unknown function DUF296
Os01g0246700	Similar to WRKY transcription factor 1.	AK105509	Q6IET0	-
Os01g0247600	Conserved hypothetical protein.	AK109701	B9EUN9	-
Os01g0248300	Similar to Pathogen-related protein.	ab initio prediction	B6TQA1	-
Os01g0248350	Hypothetical protein.	AK366258	longestORF	-
Os01g0248600	GINS complex, Psf2 component family protein.	AK061039 ,AK100824	B6SJM9	GINS complex, subunit Psf2
Os01g0248701	Terpenoid synthase domain containing protein.	BT009461	A9ZN20	Polyprenyl synthetase
Os01g0248800	Conserved hypothetical protein.	AK062766	XP_002455366.1	-
Os01g0248850	Hypothetical gene.	BT062860	longestORF	-
Os01g0248900	Similar to Expansin Os-EXPA3.	AK111100	Q9XHX0	Expansin
Os01g0249700	Heavy metal-associated domain, HMA domain containing protein.	AK361799	Q9LIU6	Heavy metal-associated domain, HMA
Os01g0249800	Heavy metal-associated domain, HMA domain containing protein.	AK361799	Q9LIU5	Heavy metal-associated domain, HMA
Os01g0249900	Protein of unknown function DUF573 family protein.	AK072886	B9EUP5	Protein of unknown function DUF573
Os01g0251200	Zinc finger, C2H2-like domain containing protein.	AK063298	B9EUQ0	Zinc finger, RING-type
Os01g0251400	Conserved hypothetical protein.	AK060341	B8ABS6	-
Os01g0252100	Similar to Glycogen synthase kinase-3 homolog MsK-3 (EC 2.7.1.-).	AK068737	Q7GC12	Protein kinase, catalytic domain
Os01g0252150	Hypothetical protein.	tplb0034j24 (Wheat FLcDNA)	longestORF	-
Os01g0252600	Similar to predicted protein.	CT835001	B8ABT0	-
Os01g0252900	Zinc finger, CCHC-type domain containing protein.	AK105250	XP_002878450.1	Zinc finger, CCHC-type
Os01g0253000	Similar to LpimPth3.	AK071644	Q5NBC2	Protein kinase, catalytic domain
Os01g0253050	Conserved hypothetical protein.	EU957207	NP_001042608.1	-
Os01g0253100	Similar to Avr9/Cf-9 induced kinase 1.	AK067133	B9EV01	Protein kinase, catalytic domain
Os01g0253200	Similar to pectinesterase inhibitor domain containing protein.	tplb0011f19 (Wheat FLcDNA)	NP_001148594.1	Pectinesterase inhibitor
Os01g0253400	Protein of unknown function DUF1218 family protein.	AK108904	Q9S809	Protein of unknown function DUF1218
Os01g0253500	Conserved hypothetical protein.	AK243648	NP_001172261.1	-
Os01g0253600	Replication factor A protein 3 domain containing protein.	AK058837	NP_001152271.1	Nucleic acid-binding, OB-fold
Os01g0253800	Conserved hypothetical protein.	ab initio prediction	NP_001172262.1	-
Os01g0253900	Similar to triacylglycerol lipase.	AK072113	NP_001148192.1	Lipase, class 3
Os01g0254000	Similar to NTGB2 (Fragment).	AK111904 ,AK111979 ,AK11954	B4FPT6	Small GTP-binding protein domain
Os01g0254100	Similar to CTV.2.	AK111762	Q2HW32	WD40 repeat
Os01g0254200	Conserved hypothetical protein.	AK121256	A2WMZ3	-
Os01g0254850	Hypothetical conserved gene.	CT834921	Q5NBT3	-
Os01g0254900	Similar to Syntaxin 22 (AtSYP22) (AtVAM3).	AK068204	Q5S1U0	Target SNARE coiled-coil domain
Os01g0254950	Hypothetical gene.	AK287984	longestORF	-
Os01g0255000	Similar to Soluble epoxide hydrolase.	AK109484 ,AK102341	NP_001148885.1	Epoxide hydrolase-like
Os01g0255100	Similar to Soluble epoxide hydrolase.	AK058528	NP_001148885.1	Epoxide hydrolase-like

Os01g0255700	Conserved hypothetical protein.	EU947173	Q5NBS7	-
Os01g0256300	Conserved hypothetical protein.	AK357815	Q1EHU3	-
Os01g0256500	Similar to ZnI.	AK069098 ,AK119698	B1PXF8	-
Os01g0256600	Ribosomal protein L18/L5 domain containing protein.	AK063884 ,AK099373	NP_001150322.1	Ribosomal protein L18/L5
Os01g0256800	Similar to zinc finger helicase family protein.	AK100311	XP_002882127.1	Zinc finger, CCCH-type
Os01g0256900	Similar to SmX6 protein.	AK100819	NP_001152235.1	Like-Sm ribonucleoprotein
Os01g0257100	Rapid ALkalinization Factor family protein.	AK243243	NP_001150840.1	Rapid ALkalinization Factor
Os01g0257300	Similar to FPF1.	AK070626	NP_001151726.1	-
Os01g0257400	Zinc finger, CCCH-type domain containing protein.	AK073920	Q5NAV3	Zinc finger, CCCH-type
Os01g0258600	Similar to predicted protein.	CT828253	E3W9Z9	-
Os01g0258700	Zinc finger, CCCH-type domain containing protein.	AK111903 ,AK111727	B7F3Z8	Zinc finger, CCCH-type
Os01g0259200	Similar to serine/threonine protein kinase PBS1.	AK067283	XP_002312759.1	Protein kinase, catalytic domain
Os01g0259300	Non-protein coding transcript.	AK105158	NONE	-
Os01g0259400	Armadillo-type fold domain containing protein.	AK102537	XP_002873912.1	Protein kinase, catalytic domain
Os01g0260000	Protein prenyltransferase domain containing protein.	AK108460	Q5NAR1	Pentatricopeptide repeat
Os01g0262000	Similar to agmatine coumaroyltransferase.	BT033950	B6SJ46	Transferase
Os01g0272700	Hypothetical conserved gene.	AK248395	NP_001150380.1	Plastocyanin-like
Os01g0272800	Conserved hypothetical protein.	AK242815	B9EV58	-
Os01g0273150	Hypothetical gene.	CU861759	longestORF	-
Os01g0273200	Conserved hypothetical protein.	ab initio prediction	A2YSX7	-
Os01g0274500	Similar to Expansin-A11.	DQ062981	Q4PNY1	Expansin
Os01g0274800	R2R3-type MYB transcription factor, Sugar partitioning into anther	AK107461	Q5NBM8	SANT domain, DNA binding
Os01g0275700	Non-protein coding transcript.	EU949846	NONE	-
Os01g0275950	Hypothetical conserved gene.	ab initio prediction	NP_001042723.2	-
Os01g0276066	Non-protein coding transcript.	BT061579	NONE	-
Os01g0276200	Similar to Mitochondrial import receptor subunit TOM40.	BT040757	B6TEP3	Porin, eukaryotic type
Os01g0276400	Similar to presenilin.	AK106403	NP_001149271.1	Peptidase A22A, presenilin
Os01g0277600	Similar to aminophospholipid ATPase.	tplb0014e24 (Wheat FLcDNA)	XP_002315406.1	ATPase, P-type, K/Mg/Cd/Cu/Zn/Na/Ca/Na/H-transporter
Os01g0278466	Non-protein coding transcript.	CT835260	NONE	-
Os01g0278900	Similar to 50S ribosomal protein L40.	AK120429	NP_001147184.1	-
Os01g0279100	Similar to Magnesium-protoporphyrin ix monomethyl ester cyclase (Fragment).	AK061646	A6N1H0	-
Os01g0279700	Non-protein coding transcript.	AK058762	NONE	-
Os01g0281000	Cyclin-like F-box domain containing protein.	AK064952	NP_001151624.1	F-box domain, cyclin-like
Os01g0281050	Hypothetical protein.	ab initio prediction	NONE	-
Os01g0281100	Conserved hypothetical protein.	AK109672	B9EVJ4	-
Os01g0281200	Similar to Type B-like cyclin (Fragment).	AK107209	Q0JNK6-2	Cyclin, C-terminal
Os01g0281250	Hypothetical protein.	tplb0047b05 (Wheat FLcDNA)	longestORF	-
Os01g0281301	Protein of unknown function DUF1645 domain containing protein.	BT065146	Q9LG63	Protein of unknown function DUF1645
Os01g0283000	Conserved hypothetical protein.	AK073165	A2WNJ8	-
Os01g0283400	Conserved hypothetical protein.	AK119993	A2WNK1	-
Os01g0285300	Myb transcription factor domain containing protein.	AK111803	D6BV29	SANT domain, DNA binding
Os01g0286100	Helix-loop-helix DNA-binding domain containing protein.	AK102252	B9EVK4	Helix-loop-helix DNA-binding
Os01g0286550	Hypothetical gene.	EU943867	GeneMark	-
Os01g0286900	Similar to Auxin-responsive protein IAA31 (Indoleacetic acid-induced protein 31).	AK103865	A2ZRY8	AUX/IAA protein
Os01g0287400	Similar to Hydrophobic protein LTI6A (Low temperature-induced protein 6A).	AK058343	Q01L12	-
Os01g0287900	Hypothetical protein.	AK372761	longestORF	-
Os01g0290000	Similar to Cyprosin precursor (EC 3.4.23.-) (Fragment).	AK066523	NP_001152501.1	Peptidase A1
Os01g0290600	Similar to isopenicillin N epimerase.	AK366122	NP_001151142.1	Aminotransferase, class V/Cysteine desulfurase
Os01g0290800	Hypothetical protein.	AK119474	longestORF	-
Os01g0308700	Similar to H0315A08.1 protein.	FP099421	Q01I63	Ribonuclease H1, N-terminal
Os01g0324400	Hypothetical conserved gene.	AK111299	Q657G4	-
Os01g0366300	Similar to Receptor protein kinase.	AK064115	Q33B66	Protein kinase, catalytic domain
Os01g0379800	Similar to HAT family dimerisation domain containing protein.	AK122139	Q2QPA8	Zinc finger, BED-type predicted
Os01g0523600	Hypothetical protein.	BT019283	longestORF	-
Os01g0536200	Conserved hypothetical protein.	AK372466	Q6K2H9	-
Os01g0539900	Conserved hypothetical protein.	AK242029	B9EXE2	-
Os01g0540000	Similar to T22C5.14.	BT086321	Q9SFZ0	-
Os01g0540300	Rac-like GTP-binding protein 2.	Q68Y52	Q68Y52	Small GTPase superfamily
Os01g0553901	HGWP repeat domain containing protein.	AK288464	Q6YWG6	HGWP repeat
Os01g0556800	Hypothetical conserved gene.	ab initio prediction	NP_001172418.1	Protein of unknown function DUF724
Os01g0557100	Alpha/beta hydrolase family protein.	AK287842	NP_001043328.1	-
Os01g0557200	Conserved hypothetical protein.	EU972429	Q8S0U8	-
Os01g0558100	Similar to Glutathione S-transferase TSI-1 (EC 2.5.1.18) (Glutathione S- transferase 1).	AK111021	NP_001152229.1	Glutathione S-transferase, N-terminal
Os01g0558500	PWWP domain containing protein.	AK099982	A2WRD3	PWWP
Os01g0558600	Ras-related protein RIC1.	AK243597	P40392	Small GTPase superfamily
Os01g0558700	Conserved hypothetical protein.	AK287514	NP_001172419.1	-
Os01g0558800	Similar to oxidoreductase/ transition metal ion binding protein.	AK068120	XP_002869432.1	Protein of unknown function DUF3531
Os01g0558825	Hypothetical protein.	AF207545	longestORF	-
Os01g0558850	Similar to peptidase M16 family protein / insulinase family protein.	BT037467	XP_002866163.1	Peptidase M16, zinc-binding site
Os01g0558900	Conserved hypothetical protein.	AK058447	Q5JKR1	-
Os01g0559000	Protein of unknown function DUF1000 family protein.	AK061456	B6T8X4	Galactose-binding domain-like
Os01g0559100	Similar to Seryl-tRNA synthetase (EC 6.1.1.11) (Serine--tRNA ligase) (SerRS) (Fragment).	AK105730	B4FRC4	Aminoacyl-tRNA synthetase, class II
Os01g0559150	Hypothetical protein.	tplb0033a05 (Wheat FLcDNA)	longestORF	-
Os01g0559200	Phosphorylated adapter RNA export protein, RNA-binding domain domain containing protein.	AK102611	B9EXJ7	Phosphorylated adapter RNA export protein, RNA-binding domain
Os01g0559300	Conserved hypothetical protein.	AK064666	A2WRD9	-
Os01g0559600	Similar to Asparaginyl endopeptidase.	AK068011	Q9SSZ4	Peptidase C13, legumain
Os01g0600900	Similar to Chlorophyll a-b binding protein 2, chloroplastic.	AK061619	P12331	Chlorophyll A-B binding protein, plant
Os01g0607800	Hypothetical conserved gene.	AK058392	A2ZV92	Pentatricopeptide repeat
Os01g0613400	Non-protein coding transcript.	AK058952	NONE	-
Os01g0613500	Peptidase C1A, papain family protein.	AK070448	A2WSK3	Peptidase, cysteine peptidase active site
Os01g0618400	Similar to RNA helicase (Fragment).	AK067570	Q9FFQ1	RNA helicase, ATP-dependent, DEAD-box, conserved site
Os01g0618450	Non-protein coding transcript.	AF366447	NONE	-
Os01g0618500	Similar to RNA helicase (Fragment).	AK067476	B7EDN0	RNA helicase, ATP-dependent, DEAD-box, conserved site
Os01g0618601	Non-protein coding transcript.	AF366447	NONE	-
Os01g0618900	Similar to polygalacturonase.	AK099329	B6TDS0	Glycoside hydrolase, family 28
Os01g0619900	Similar to DNA binding.	AK060239	NP_001118417.1	-
Os01g0629400	Similar to Ctd-phosphatase-like protein (Fragment).	AK063853	A6N1L0	NLI interacting factor
Os01g0637600	Similar to Peptide deformylase, chloroplast precursor (EC 3.5.1.88) (PDF) (Polypeptide deformylase	AK106980	Q5VNN5	Formylmethionine deformylase
Os01g0639900	Non-protein coding transcript.	AK058203	NONE	-
Os01g0640700	Similar to vegetative cell wall protein gp1.	AK334548	C5XEK4	Uncharacterised protein family UPF0497, trans-membrane plant
Os01g0641750	Hypothetical protein.	AK361892	longestORF	-
Os01g0641800	Similar to Carbonic anhydrase.	BT060613	B8ACD0	ATPase, AAA+ type, core
Os01g0642000	Carboxylesterase, type B family protein.	AK110911	NP_173937.2	Carboxylesterase, type B
Os01g0642100	Hypothetical conserved gene.	AK063793	B9EYA5	-
Os01g0642200	Conserved hypothetical protein.	AK105174 ,AK071521	B8ACD2	-
Os01g0644200	Similar to Little protein 1.	AK063634	B4UWC1	-
Os01g0647000	Similar to F-box domain containing protein.	AK061541	NP_001149398.1	-
Os01g0648500	Similar to Transcription initiation factor TFIID subunit 7.	AK241160	B6UGL3	TAFII55 protein, conserved region
Os01g0648700	Bromodomain containing protein.	AK111374	Q94JA6	Bromodomain
Os01g0649100	Malate dehydrogenase.	AK071699	Q7FSL4	Lactate/malate dehydrogenase, N-terminal
Os01g0650501	Non-protein coding transcript.	EU942775	NONE	-
Os01g0650900	Lipase, GDSL domain containing protein.	AK375707	NP_001043727.1	Lipase, GDSL
Os01g0650950	Hypothetical protein.	tplb0048c08 (Wheat FLcDNA)	longestORF	-
Os01g0651000	Lipase, GDSL domain containing protein.	AK242032	Q8RZ48	Lipase, GDSL
Os01g0651100	Pentatricopeptide repeat domain containing protein.	AK119380	B8A6T7	Pentatricopeptide repeat
Os01g0651300	Similar to GDSL-motif lipase/hydrolase-like protein.	AK111325	NP_001151904.1	Lipase, GDSL
Os01g0651400	Lipase, GDSL domain containing protein.	AK059784	NP_001151904.1	-
Os01g0651450	Hypothetical protein.	EU954131	longestORF	-
Os01g0651500	Wax synthase domain containing protein.	EU959294	B6T2S9	Wax synthase
Os01g0652000	Similar to proline-rich family protein.	AK058938	NP_193591.2	-
Os01g0652450	Hypothetical gene.	CT834813	Q6L4J0	-
Os01g0652600	Similar to Ketol-acid reductoisomerase, chloroplast precursor (EC 1.1.1.86) (Acetohydroxy-acid redu	AK072075	B6TF69	Acetohydroxy acid isomeroreductase C-terminal
Os01g0652650	Non-protein coding transcript.	D21110	NONE	-
Os01g0652700	Similar to predicted protein.	EU967350	NP_001043739.2	Protein of unknown function DUF538
Os01g0652800	Protein of unknown function DUF231, plant domain containing protein.	AK061145 ,AK103547 ,AK10431	A2WTA8	Domain of unknown function DUF231, plant

Os01g0653500	Conserved hypothetical protein.	ab initio prediction	Q5VPB2	-
Os01g0654100	Similar to CTP synthase (EC 6.3.4.2) (UTP--ammonia ligase) (CTP synthetase).	AK069482	NP_001148101.1	CTP synthase
Os01g0654150	Non-protein coding transcript.	tplb0056h06 (Wheat FLcDNA)	NONE	-
Os01g0654200	Peptidase M50 domain containing protein.	AK100750	B9EYD8	Peptidase M50
Os01g0654300	Similar to ARP2/3 complex 34 kDa subunit.	AK058593	B6TA29	Arp2/3 complex, 34kDa subunit p34-Arc
Os01g0654400	Similar to Salt tolerant correlative protein.	AK288103	E6Y2L2	-
Os01g0654450	Hypothetical gene.	EU946002	longestORF	-
Os01g0654500	Similar to NADP-isocitrate dehydrogenase.	AK061752	Q9XGU8	Isocitrate dehydrogenase NADP-dependent, eukaryotic-type
Os01g0654650	Non-protein coding transcript.	BT016882	NONE	-
Os01g0654800	Conserved hypothetical protein.	AK359861	B9EYE1	-
Os01g0655300	Similar to Trithorax 4 (Fragment).	AK062870	B3VSN3	SET domain
Os01g0655400	Hypothetical conserved gene.	AK069873	B8A6V1	-
Os01g0668000	CS domain containing protein.	AK061159	B6SNS1	CS-like domain
Os01g0668600	Curculin-like (mannose-binding) lectin domain containing protein.	AK365081	NP_001043817.2	Protein kinase, catalytic domain
Os01g0705400	Hypothetical conserved gene.	AK336128	A2ZX08	-
Os01g0707500	Similar to Transcription factor LAX PANICLE.	AK100208	A2WUA1	Helix-loop-helix DNA-binding
Os01g0708100	Similar to N-myristoyl transferase (EC 2.3.1.97).	AK101457	A2WUA4	Myristoyl-CoA:protein N-myristoyltransferase
Os01g0708201	Hypothetical gene.	BT024151	longestORF	-
Os01g0708400	Non-protein coding transcript.	AK242236	NONE	-
Os01g0710100	Hypothetical conserved gene.	AK357053	NP_001044037.2	Protein of unknown function DUF761, plant
Os01g0711900	Conserved hypothetical protein.	AK108302	A2ZX51	-
Os01g0718300	Similar to Brassinosteroid-insensitive 1.	AK121760	Q0ZA03	Protein kinase, catalytic domain
Os01g0720200	Non-protein coding transcript.	AK058236	NONE	-
Os01g0720700	Similar to satase isoform I.	CT830895	NP_001105082.1	-
Os01g0722550	Similar to HAT family dimerisation domain containing protein.	AK122139	Q2QPA8	Zinc finger, BED-type predicted
Os01g0732300	Protein of unknown function DUF623, plant domain containing protein.	AK108391	A2WUS7	Domain of unknown function DUF623
Os01g0745700	Hypothetical conserved gene.	AK062377	Q5JLX9	-
Os01g0748200	Conserved hypothetical protein.	AK372402	Q5JNJ4	-
Os01g0755700	Zinc finger, RING/FYVE/PHD-type domain containing protein.	AK108853	NP_001148026.1	Zinc finger, RING-type
Os01g0763850	Hypothetical protein.	BT085554	longestORF	-
Os01g0766966	Hypothetical gene.	BT066271	longestORF	-
Os01g0769100	Non-protein coding transcript.	AK242413	NONE	-
Os01g0777650	Hypothetical gene.	AK289196	longestORF	-
Os01g0791033	Similar to ribulose-1,5-bisphosphate carboxylase/oxygenase large subunit.	ab initio prediction	NP_039391.1	Ribulose bisphosphate carboxylase, large subunit, C-terminal
Os01g0793900	Conserved hypothetical protein.	AK062811	A2WVX8	-
Os01g0800200	Conserved hypothetical protein.	ab initio prediction	NP_001172605.1	-
Os01g0805750	Non-protein coding transcript.	EU951443	NONE	-
Os01g0807000	Conserved hypothetical protein.	AK109751	Q53NH3	-
Os01g0810000	Utp11 family protein.	AK102143	NP_001150340.1	Small-subunit processome, Utp11
Os01g0814400	Conserved hypothetical protein.	AK104661	B8ABA0	-
Os01g0814550	Non-protein coding transcript.	BT086943	NONE	-
Os01g0819100	Similar to predicted protein.	AK242625	C0LGW1	Protein kinase, catalytic domain
Os01g0823600	Conserved hypothetical protein.	AK242079	NP_001044661.1	-
Os01g0825166	Similar to Splicing coactivator subunit-like protein.	EU944053	C8TFH6	-
Os01g0825332	Similar to Splicing coactivator subunit-like protein.	EU944053	C8TFH6	-
Os01g0828900	Similar to predicted protein.	AB512496	NP_563780.1	Domain of unknown function DUF640
Os01g0829000	Thioredoxin-like fold domain containing protein.	AK065327	B6TC88	Thioredoxin-like fold
Os01g0829100	Ankyrin domain containing protein.	AK060644	A2Z5H4	Major sperm protein
Os01g0829183	Non-protein coding transcript.	X06283	NONE	-
Os01g0829266	Hypothetical conserved gene.	AK241659	A2XX31	-
Os01g0829500	Conserved hypothetical protein.	EU962098	Q941V6	-
Os01g0829600	Protein of unknown function DUF688 domain containing protein.	AK060725	Q5QLS4	Protein of unknown function DUF688
Os01g0829700	Hypothetical protein.	AK109789	longestORF	-
Os01g0830000	Similar to Plastid sufB (Fragment).	AK121100	A2WWL8	SUF system FeS cluster assembly, SufBD
Os01g0830100	Pyridine nucleotide-disulphide oxidoreductase, NAD-binding region domain containing protein.	AK069755	XP_002881079.1	Pyridine nucleotide-disulphide oxidoreductase, NAD-binding domain
Os01g0831250	Hypothetical gene.	BT054429	longestORF	-
Os01g0831300	Similar to Ammonium transporter.	AK109023	Q8S233	Ammonium transporter
Os01g0831900	Similar to Ammonium transporter.	AK102106	Q8S230	Ammonium transporter
Os01g0831950	Hypothetical protein.	BT054429	longestORF	-
Os01g0835500	Similar to respiratory burst oxidase protein A.	AK068400	B2D0P0	Cytochrome b245, heavy chain
Os01g0836400	Hypothetical conserved gene.	AK068741	Q5QMF1	-
Os01g0836600	ABC transporter-like domain containing protein.	AK067556	NP_181467.1	ABC transporter-like
Os01g0836800	Transmembrane receptor, eukaryota domain containing protein.	AK066629 ,AK098958 ,AK11978	NP_001150692.1	Transmembrane receptor, eukaryota
Os01g0836900	Conserved hypothetical protein.	AK064231	A2WWR9	-
Os01g0837000	Ankyrin repeat containing protein.	AK243678	A2CIR5	Ankyrin repeat
Os01g0837200	Similar to esterase/lipase/thioesterase family protein.	AK062368	NP_191078.1	-
Os01g0837300	Similar to UDP-glucuronic acid decarboxylase 1.	AK104675	NP_001151221.1	NAD-dependent epimerase/dehydratase
Os01g0837600	Similar to plant-specific domain TIGR01589 family protein.	AK108007	NP_001148858.1	Conserved hypothetical protein CHP01589, plant
Os01g0837800	Similar to metal tolerance protein.	AK061539	Q5NA18	Cation efflux protein
Os01g0837900	Similar to Protein kinase AFC1 (EC 2.7.1.-).	AK103207	NP_850695.2	Protein kinase, catalytic domain
Os01g0839200	Protein of unknown function DUF966 family protein.	AK068926	A2WWT9	Protein of unknown function DUF966
Os01g0844500	Peptidase aspartic, catalytic domain containing protein.	EU944384	NP_001044781.1	Peptidase A1
Os01g0849050	Non-protein coding transcript.	DQ185898	NONE	-
Os01g0853000	Conserved hypothetical protein.	AK099561	XP_002458778.1	-
Os01g0853700	Similar to MCB1 protein.	AK111988	B7ZY89	SANT domain, DNA binding
Os01g0854400	Conserved hypothetical protein.	AK108330	A2WX36	-
Os01g0855400	SANT domain, DNA binding domain containing protein.	FJ940215	NP_001044841.1	SANT domain, DNA binding
Os01g0855600	Similar to Hs1pro-1 protein.	AK099776	Q9FUH4	Hs1pro-1, C-terminal
Os01g0855700	Hypothetical protein.	AK106740	longestORF	-
Os01g0856850	Similar to ribosomal protein S7.	EU957350	NP_039458.1	Ribosomal protein S7 domain
Os01g0858350	Similar to cytochrome P450.	AK061128 ,AK100746	XP_002328165.1	Cytochrome P450
Os01g0858900	Glycosyl transferase, family 29 protein.	AK107493	NP_001151877.1	Glycosyl transferase, family 29
Os01g0866600	Similar to bolA-like protein.	AK242498	NP_001148455.1	BolA protein
Os01g0869900	Similar to Serine/threonine-protein kinase SAPK4.	AK060576	C8CBK4	-
Os01g0872650	Non-protein coding transcript.	CT835515	NONE	-
Os01g0875500	Similar to Beta-galactosidase (EC 3.2.1.23).	AK102715	B6U0W2	D-galactoside/L-rhamnose binding SUEL lectin domain
Os01g0876650	Hypothetical conserved gene.	CT834967	Q654G2	-
Os01g0877500	Hypothetical conserved gene.	AK058582 ,AK064177	A2WXJ6	Zinc finger, PHD-type
Os01g0878000	Conserved hypothetical protein.	AK104289 ,AK060162	A3A062	-
Os01g0878550	Hypothetical protein.	tplb0036o08 (Wheat FLcDNA)	longestORF	-
Os01g0882900	Hypothetical protein.	tplb0036o08 (Wheat FLcDNA)	longestORF	-
Os01g0883850	Hypothetical protein.	DQ334868	longestORF	-
Os01g0884350	Hypothetical protein.	FP093663	longestORF	-
Os01g0884450	Non-protein coding transcript.	BT084688	NONE	-
Os01g0885500	Hypothetical conserved gene.	AK071788 ,AK106609	Q5N8H2	-
Os01g0886600	Similar to CLP protease regulatory subunit CLPX precursor.	AK070098	B7FAD8	ATPase, AAA+ type, core
Os01g0887100	Similar to 4-(Cytidine 5'-diphospho)-2-C-methyl-D-erythritol synthase.	AK240934	A4PIB9	4-diphosphocytidyl-2C-methyl-D-erythritol synthase
Os01g0890100	Similar to S-domain class receptor-like kinase3.	tplb0056i22 (Wheat FLcDNA)	NP_001105655.1	Protein kinase, catalytic domain
Os01g0890500	Conserved hypothetical protein.	ab initio prediction	Q5N832	-
Os01g0892800	Similar to Ankyrin-kinase.	AK070097	Q8SAE5	Protein kinase, catalytic domain
Os01g0893400	Zinc finger, TAZ-type domain containing protein.	AK071112	B8A2U0	Zinc finger, TAZ-type
Os01g0894075	Hypothetical protein.	tplb0043a14 (Wheat FLcDNA)	longestORF	-
Os01g0894100	Similar to Transposase (Fragment).	AK111398	NP_001176912.1	-
Os01g0895200	DOMON related domain containing protein.	AK063282	A3A0H2	Cytochrome b561, eukaryote
Os01g0900900	Ovarian tumour, otubain domain containing protein.	AK120577	NP_001078307.1	Ovarian tumour, otubain
Os01g0901101	Non-protein coding transcript.	BT084794	NONE	-
Os01g0916100	Similar to loricrin.	AK109173	NP_001147500.1	-
Os01g0916300	Similar to PQBP-1 protein (Nuclear protein containing a WW domain) (Npw38) (JM26 protein).	AK063922	NP_001147059.1	WW/Rsp5/WWP
Os01g0916350	Hypothetical gene.	AK288280	longestORF	-
Os01g0916400	Similar to Selenium binding protein.	AK071710	B6TFC2	Selenium-binding protein
Os01g0916800	Similar to predicted protein.	ab initio prediction	NP_001045197.2	THO complex, subunitTHOC2, C-terminal
Os01g0917300	Similar to Cysteine-rich peptide.	AK240939	D7NXT9	-
Os01g0920200	Similar to E(Y)2 homolog (DC6) (Enhancer of yellow 2 homolog).	AK120182	B6T6J7	Transcription factor, enhancer of yellow 2
Os01g0920400	Hypothetical conserved gene.	AK063897	B8A8F0	-
Os01g0920450	Conserved hypothetical protein.	ab initio prediction	B9EVX1	-

Os01g0921300	Exostosin-like family protein.	AK119614 ,AK106015	NP_001152546.1	Exostosin-like
Os01g0921450	Hypothetical protein.	AK374499	longestORF	-
Os01g0921550	Hypothetical protein.	ab initio prediction	NONE	-
Os01g0921800	Tetratricopeptide-like helical domain containing protein.	AK334012	XP_002887115.1	Zinc finger, MYND-type
Os01g0922000	Hypothetical protein.	AK073034	longestORF	-
Os01g0922700	Conserved hypothetical protein.	AK287528	NP_001172712.1	-
Os01g0923200	Similar to AT.I.24-6 protein (Fragment).	AK060288	Q96310	Protein of unknown function DUF3339
Os01g0923600	Hypothetical conserved gene.	AK059687	B8A8F8	IQ motif, EF-hand binding site
Os01g0923700	Similar to Histidine kinase.	AK121135	A1A696	-
Os01g0923750	Hypothetical gene.	BT086966	longestORF	-
Os01g0924100	Non-protein coding transcript.	AK062847	NONE	-
Os01g0927900	Similar to Aspartate kinase precursor (EC 2.7.2.4).	AK073189	B8A8I6	Aspartate/glutamate/uridylate kinase
Os01g0927950	Hypothetical gene.	BT068036	longestORF	-
Os01g0928000	Similar to Transcription factor ICE1 (Inducer of CBF expression 1) (Basic helix- loop-helix protein 1	AK102594	B6UB60	Helix-loop-helix DNA-binding
Os01g0929100	Similar to predicted protein.	AK060605	Q5JK31	-
Os01g0933200	Heavy metal transport/detoxification protein domain containing protein.	ab initio prediction	NP_001147140.1	Heavy metal-associated domain, HMA
Os01g0933300	Non-protein coding transcript.	AK060386	NONE	-
Os01g0942900	Similar to cDNA clone:J023028M23, full insert sequence.	AK243304	B7E3R6	Ankyrin repeat
Os01g0943800	Similar to cDNA, clone: J100054L12, full insert sequence.	AK059510	B7FA83	Ankyrin repeat
Os01g0947500	Conserved hypothetical protein.	Z93114	F2DK87	-
Os01g0947833	Conserved hypothetical protein.	AK062969	NP_001045425.1	-
Os01g0952300	Similar to Ran GTPase binding / chromatin binding / zinc ion binding.	AK333491	NP_197443.3	Zinc finger, FYVE-type
Os01g0958000	Similar to Cyclin-dependent kinase C-2.	AK109352	Q5JK68	Protein kinase, catalytic domain
Os01g0959900	Similar to predicted protein.	AK066497	NP_565093.1	NAD
Os01g0960000	Gamma-secretase aspartyl protease complex, presenilin enhancer-2 subunit domain containing prote	AK108183	B8A9J7	Gamma-secretase aspartyl protease complex, presenilin enhancer-2 subunit
Os01g0960101	Non-protein coding transcript.	X02683	NONE	-
Os01g0961000	Conserved hypothetical protein.	AK062919	B8A9K4	-
Os01g0962000	Hypothetical conserved gene.	AK059675	A2WZC9	-
Os01g0962400	Ubiquitin-like, Ufm1 domain containing protein.	AK059165	NP_001151442.1	Ubiquitin-fold modifier 1
Os01g0962500	Zinc finger, HIT-type domain containing protein.	AK073163	A2WZD4	Zinc finger, HIT-type
Os01g0962600	Similar to 40S ribosomal protein S10-1.	AK073846	NP_001152734.1	Plectin/S10, N-terminal
Os01g0962650	Hypothetical gene.	BT019280	longestORF	-
Os01g0962900	Similar to Cationic peroxidase SPC4 (Fragment).	ab initio prediction	P84516	Plant peroxidase
Os01g0963000	Similar to Peroxidase BP 1 precursor.	AK061131 ,AK102172 ,AK10948	Q5U1S3	Plant peroxidase
Os01g0966100	Similar to Peroxisomal ABC transporter.	AK110805	Q5JJV5	ABC transporter-like
Os01g0966200	Protein of unknown function YGGT family protein.	AK058564 ,AK098935	NP_198461.2	Uncharacterised protein family Ycf19
Os01g0966300	Similar to Mitochondrial processing peptidase.	AK073175	P29677	Peptidase M16, C-terminal
Os01g0966400	Leucine-rich repeat, SDS22 containing protein.	AK103064	B6TDM9	Leucine-rich repeat
Os01g0966500	Similar to Vacuolar protein sorting 55 containing protein.	AK062986	A6N1U6	Vacuolar protein sorting 55
Os01g0968600	Leucine-rich repeat, cysteine-containing subtype domain containing protein.	AK356760	B8A9V8	Leucine-rich repeat, cysteine-containing subtype
Os01g0968700	tRNA isopentenyltransferase family protein.	AK066574	Q33CD2	tRNA isopentenyltransferase
Os01g0968800	Similar to Dehydration responsive element binding protein 1F (DREB1F protein).	AY345234	Q8S9Z5	Pathogenesis-related transcriptional factor/ERF, DNA-binding
Os01g0970600	Similar to ATP binding protein.	AK288993	NP_001147746.1	Helicase, C-terminal
Os01g0970700	Peptidase M48, Ste24p family protein.	AK098940	B6TA12	Peptidase M48
Os01g0971000	Conserved hypothetical protein.	AK061660 ,AK120145	A2WZJ6	-
Os01g0971100	Drought induced 19 family protein.	AK069516	Q5JME8	Drought induced 19/ RING finger protein 114
Os01g0971200	Similar to Lysine ketoglutarate reductase trans-splicing related 1.	AK064075	B4FSQ8	Protein of unknown function DUF707
Os01g0971301	Non-protein coding transcript.	BT016843	NONE	-
Os01g0971600	Similar to Sn-glycerol-3-phosphate dehydrogenase (Fragment).	AK070366	NP_001150493.1	Glycerol-3-phosphate dehydrogenase, NAD-dependent, C-terminal
Os02g0100250	Hypothetical protein.	BT083581	longestORF	-
Os02g0114033	Glutamine-Leucine-Glutamine, QLQ domain containing protein.	tplb0057e06 (Wheat FLcDNA)	Q6Z7C5	Glutamine-Leucine-Glutamine, QLQ
Os02g0114066	Non-protein coding transcript.	EU948018	NONE	-
Os02g0124000	Arbuscular mycorrhizal specific marker 31.	EU953683	Q6Z711	Proteinase inhibitor I13, potato inhibitor I
Os02g0124300	Arbuscular mycorrhizal specific marker 24.	FP099153	Q6Z708	Proteinase inhibitor I13, potato inhibitor I
Os02g0124800	Conserved hypothetical protein.	AK058547	Q6Z705	-
Os02g0124866	Hypothetical gene.	AK288719	longestORF	-
Os02g0128501	Non-protein coding transcript.	BT016330	NONE	-
Os02g0128600	Similar to ADP-ribosylation factor-like protein.	AK059865 ,AK103009	B6TGE5	Small GTP-binding protein domain
Os02g0128800	TRAF-type domain containing protein.	AK111795	NP_001123598.1	Zinc finger, RING-type
Os02g0129300	Conserved hypothetical protein.	AK065700	B8AH08	-
Os02g0129700	Hypothetical protein.	AK065610 ,AK063802 ,AK28749	longestORF	-
Os02g0129800	Conserved hypothetical protein.	AK109213	A2X0H5	-
Os02g0130000	Similar to SWIb domain-containing protein (Fragment).	AK106464	B6THI7	SWIB/MDM2 domain
Os02g0130100	SNO glutamine amidotransferase family protein.	AK121866	B6SNW4	Glutamine amidotransferase subunit PdxT
Os02g0130300	Hypothetical conserved gene.	AK106339	A2X0I0	Zinc finger, RING-type
Os02g0130700	Hypothetical genes.	ab initio prediction	NP_001172785.1	-
Os02g0130800	F-box domain, cyclin-like domain containing protein.	EU975717	B9F2A0	F-box domain, cyclin-like
Os02g0130900	Ubiquitin-conjugating enzyme E2C-binding protein domain containing protein.	AK102856	A2X0I7	Ubiquitin-conjugating enzyme E2C-binding protein
Os02g0131000	Conserved hypothetical protein.	AK062905	A2X0I8	-
Os02g0131050	Hypothetical gene.	AK242812	longestORF	-
Os02g0131100	Hypothetical conserved gene.	AK105854	B9F2A1	-
Os02g0131300	Non-protein coding transcript.	AK061919	NONE	-
Os02g0131850	Hypothetical gene.	BT086902	longestORF	-
Os02g0132100	Pentatricopeptide repeat domain containing protein.	ab initio prediction	NP_001045795.1	Pentatricopeptide repeat
Os02g0132200	Conserved hypothetical protein.	AK370222	NP_001045796.1	-
Os02g0132950	Hypothetical gene.	AK241678	longestORF	-
Os02g0133000	Cyclin-T1-1.	Q0E474	Q0E474	Cyclin, N-terminal
Os02g0133100	Similar to Phosphatidylinositol transfer-like protein III.	AK060180	Q94FN1	Cellular retinaldehyde-binding/triple function, C-terminal
Os02g0133200	Similar to Phosphatidylinositol transfer-like protein IV.	AK100898 ,AK072646	NP_568006.1	Cellular retinaldehyde binding/alpha-tocopherol transport
Os02g0133800	Proteasome subunit alpha type 1 (EC 3.4.25.1) (20S proteasome alpha subunit F) (20S proteasome s	AK103646 ,AK109376	B8AHG2	Proteasome, alpha-subunit, conserved site
Os02g0133900	Protein of unknown function DUF829, eukaryotic family protein.	AK107180	NP_001130250.1	Protein of unknown function DUF829, TMEM53
Os02g0134000	Similar to bifunctional coenzyme A synthase.	AK355133	NP_001147323.1	-
Os02g0134100	Hypothetical gene.	AK069055	longestORF	-
Os02g0134200	Protein of unknown function DUF1645 family protein.	AK108331	A2X0L4	Protein of unknown function DUF1645
Os02g0134300	Protein of unknown function DUF701, zinc-binding putative family protein.	AK120292	B6SX26	Protein of unknown function DUF701, zinc-binding putative
Os02g0134400	Fumarate reductase/succinate dehydrogenase flavoprotein, C-terminal domain containing protein.	AK072394	B6U0K6	Fumarate reductase/succinate dehydrogenase flavoprotein, N-terminal
Os02g0135000	Conserved hypothetical protein.	AK069693	A2X0L9	-
Os02g0135200	Similar to Mitogen-activated protein kinase 13.	CT836561	Q0E459	Protein kinase, catalytic domain
Os02g0135250	Hypothetical protein.	tplb0059e09 (Wheat FLcDNA)	longestORF	-
Os02g0135300	Similar to Wee1-like protein kinase.	AK372043	Q6Z829	Protein kinase, catalytic domain
Os02g0135600	Conserved hypothetical protein.	AK069843	A2X0M4	-
Os02g0135700	DNA polymerase V family protein.	AK100570	B9F2L5	DNA polymerase V
Os02g0135800	Similar to Sec13-like protein (Fragment).	AK112028	Q8W403	WD40 repeat
Os02g0135850	Hypothetical conserved gene.	ab initio prediction	B9F2L6	Ubiquitin system component Cue
Os02g0135900	Similar to 5'-AMP-activated protein kinase beta-1 subunit-related.	AK072017	NP_568573.2	-
Os02g0135950	Non-protein coding transcript.	BT016931	NONE	-
Os02g0136150	Hypothetical gene.	CT835435	longestORF	-
Os02g0136400	Conserved hypothetical protein.	ab initio prediction	B8AHD1	-
Os02g0136850	Non-protein coding transcript.	CU406396	NONE	-
Os02g0136900	Protein kinase, core domain containing protein.	AK111638	B8AHD4	Protein kinase, catalytic domain
Os02g0136933	Hypothetical conserved gene.	ab initio prediction	B9F2M0	WD40 repeat-like-containing domain
Os02g0136966	Hypothetical gene.	AK288412	longestORF	-
Os02g0137000	WD40 repeat-like domain containing protein.	AK111719	XP_002875574.1	WD40 repeat
Os02g0137100	Similar to plant-specific domain TIGR01589 family protein.	AK073353	NP_001148712.1	Conserved hypothetical protein CHP01589, plant
Os02g0137200	Similar to 50S ribosomal protein L3-1, chloroplast precursor.	AK099849	B6UF84	Ribosomal protein L3
Os02g0137400	Similar to Spliceosomal-like protein.	AK068480	Q9LD60	Cleavage/polyadenylation specificity factor, A subunit, C-terminal
Os02g0137450	Conserved hypothetical protein.	AK369368	B9F2M2	-
Os02g0137500	Similar to histone acetyltransferase.	AK241505	XP_002330477.1	Zinc finger, TAZ-type
Os02g0137600	Bacterial methyltransferase family protein.	AK102168	NP_196652.1	S-adenosyl-L-methionine-dependent methyltransferase, MraW
Os02g0137700	NAD(P)-binding domain containing protein.	AK241047	XP_002890185.1	NmrA-like
Os02g0150600	Similar to Pyridine nucleotide-disulphide oxidoreductase (Fragment).	AK105844	B9F2R3	-
Os02g0150700	Zinc finger, RING-type domain containing protein.	AK120070	B9F2R3	Zinc finger, RING-type
Os02g0150900	Protein of unknown function DUF1644 family protein.	AK121424	Q67UW9	Zinc finger, C2H2
Os02g0151100	Hypothetical conserved gene.	AK243362	Q67UW6	Protein kinase, catalytic domain
Os02g0151501	Similar to SUI1 protein.	GU565696	Q84LW9	Translation initiation factor SUI1

Os02g0151600	Ketol-acid reductoisomerase, chloroplast precursor (EC 1.1.1.86) (Acetohydroxy-acid reductoisomer	ab initio prediction	B6TF69	Acetohydroxy acid isomeroreductase
Os02g0152200	Protein of unknown function DUF1677, Oryza sativa family protein.	AK070155	A2X0Z5	Protein of unknown function DUF1677, Oryza sativa
Os02g0152300	Serine/threonine protein kinase-related domain containing protein.	AK069368	B8AHT6	Protein kinase, catalytic domain
Os02g0152400	Similar to Ribulose bisphosphate carboxylase (EC 4.1.1.39) (Fragment).	AK059909	B8AHT7	Ribulose bisphosphate carboxylase small chain, domain
Os02g0152500	Non-protein coding transcript.	AK062072	NONE	-
Os02g0156800	Leucine-rich repeat-containing N-terminal, type 2 domain containing protein.	AK067358	B9F2U1	Leucine-rich repeat-containing N-terminal, type 2
Os02g0157000	Leucine-rich repeat domain containing protein.	AK371462	NP_001172810.1	Leucine-rich repeat
Os02g0157100	Leucine-rich repeat domain containing protein.	AK371462	XP_002453319.1	Leucine-rich repeat
Os02g0157200	Leucine-rich repeat domain containing protein.	ab initio prediction	NP_001045949.1	Leucine-rich repeat
Os02g0157300	Hypothetical protein.	AK102244	longestORF	-
Os02g0157400	Leucine-rich repeat-containing N-terminal, type 2 domain containing protein.	AK067358	B9F2U1	Leucine-rich repeat-containing N-terminal, type 2
Os02g0157501	Leucine-rich repeat domain containing protein.	AK369574	longestORF	Leucine-rich repeat
Os02g0157600	Similar to senescence-associated protein DIN1.	AK243231	NP_001151916.1	Rhodanese-like
Os02g0157700	Non-protein coding transcript.	AK242201	NONE	-
Os02g0157900	Hypothetical conserved gene.	AB244282	Q6ET49	RNA recognition motif domain
Os02g0158100	Similar to EH-domain-containing protein 1.	AK058991	B6U193	-
Os02g0158600	Conserved hypothetical protein.	AK066467	A2X145	-
Os02g0161000	Similar to Serine/threonine protein kinase-like protein.	AK111660	Q6H7U5	Protein kinase, catalytic domain
Os02g0161100	Actin-binding FH2 domain containing protein.	AK072377	Q6H7U3	Actin-binding FH2/DRF autoregulatory
Os02g0161251	Hypothetical gene.	AK288500	longestORF	-
Os02g0161300	Similar to Mot1 (Fragment).	AK070938	C6ERB4	Domain of unknown function DUF3535
Os02g0161900	Similar to polyubiquitin containing 7 ubiquitin monomers.	AK101547 ,AK061956	NP_001147027.1	Ubiquitin
Os02g0162050	Non-protein coding transcript.	BT016940	NONE	-
Os02g0162150	Hypothetical protein.	ab initio prediction	NONE	-
Os02g0162600	Conserved hypothetical protein.	AK242133	NP_001045983.1	-
Os02g0162700	Non-protein coding transcript.	AK058629	NONE	-
Os02g0162783	Non-protein coding transcript.	EU943604	NONE	-
Os02g0163533	Conserved hypothetical protein.	AK241536	NP_001172814.1	-
Os02g0163600	Conserved hypothetical protein.	AK068043	B8AI72	-
Os02g0164800	Zinc finger, C2H2-type domain containing protein.	AK062817	NP_175482.1	-
Os02g0164900	Similar to Auxin response factor 3.	AK070569	Q6H6V4	AUX/IAA protein
Os02g0165000	Zinc finger, RING/FYVE/PHD-type domain containing protein.	AK069562	B8AI80	Zinc finger, RING-CH-type
Os02g0165100	Protein kinase, core domain containing protein.	AK066645	NP_001148145.1	Protein kinase, catalytic domain
Os02g0165200	Hypothetical protein.	AK101881	longestORF	-
Os02g0165500	Conserved hypothetical protein.	AK060547	A2X1A6	-
Os02g0165800	Conserved hypothetical protein.	AK106983	A2X1A7	-
Os02g0166200	Non-protein coding transcript.	AK066189	NONE	-
Os02g0166501	Hypothetical conserved gene.	CT835482	Q6H4W8	-
Os02g0166600	Hypothetical protein.	AK069371	longestORF	-
Os02g0166875	Hypothetical protein.	EU970153	longestORF	-
Os02g0166950	Hypothetical gene.	AK243419	longestORF	-
Os02g0167000	Similar to holocarboxylase synthetase 1.	AK375247	XP_002878877.1	Biotin protein ligase, C-terminal
Os02g0167100	Similar to 3-mercaptopyruvate sulfurtransferase precursor (EC 2.8.1.1).	AK066425	Q94C43	Thiosulphate sulfurtransferase, conserved site
Os02g0167200	Pentatricopeptide repeat domain containing protein.	AK106491	Q6H4W1	Pentatricopeptide repeat
Os02g0167300	Similar to Tubulin beta-5 chain.	AK061659 ,AK119164	P46265	Tubulin
Os02g0167400	Non-protein coding transcript.	X06283	NONE	-
Os02g0167500	RNA recognition motif domain domain containing protein.	AK069161	B9F385	RNA recognition motif domain
Os02g0167600	Hypothetical gene.	AK242059	longestORF	-
Os02g0167850	Non-protein coding transcript.	CT835346	NONE	-
Os02g0168100	Similar to 4-hydroxyphenylpyruvate dioxygenase.	AK058890	O48604	Glyoxalase/fosfomycin resistance/dioxygenase
Os02g0168550	Non-protein coding transcript.	tplb0046d03 (Wheat FLcDNA)	NONE	-
Os02g0168700	Peptidyl-prolyl cis-trans isomerase, FKBP-type domain containing protein.	AK061562	NP_001148684.1	Peptidyl-prolyl cis-trans isomerase, FKBP-type, domain
Os02g0169000	Similar to T6H22.2 protein.	AK101628	Q9SGU5	-
Os02g0169151	Hypothetical gene.	BT016355	longestORF	-
Os02g0169275	Non-protein coding transcript.	FP098893	NONE	-
Os02g0169300	Similar to Phosphoglycerate kinase, cytosolic (EC 2.7.2.3).	AK070041	B8AIH2	Phosphoglycerate kinase
Os02g0169400	Similar to Argonaute 4 protein.	AK106247	Q6H6C3	Argonaute/Dicer protein, PAZ
Os02g0169450	Non-protein coding transcript.	BT016774	NONE	-
Os02g0169900	Inositol monophosphatase family protein.	AK105887 ,AK070107	NP_564376.1	Inositol monophosphatase
Os02g0170000	Conserved hypothetical protein.	AK065022	A3A3K8	-
Os02g0175250	Hypothetical protein.	AM939967	longestORF	-
Os02g0177666	Hypothetical protein.	BT017473	longestORF	-
Os02g0180400	GCN5-related N-acetyltransferase (GNAT) domain domain containing protein.	AK355812	Q6H820	GCN5-related N-acetyltransferase
Os02g0190800	Alpha/beta hydrolase fold-1 domain containing protein.	AK099853	NP_001151903.1	-
Os02g0204000	Tetratricopeptide-like helical domain containing protein.	AK066727	NP_001147153.1	Tetratricopeptide-like helical
Os02g0220050	Hypothetical protein.	AK375091	longestORF	-
Os02g0220100	Similar to Cytokinin dehydrogenase 7.	GU160403	Q6YW50	Oxygen oxidoreductase covalent FAD-binding site
Os02g0230300	En/Spm-like transposon protein (Protodermal factor 1).	AK058752	Q56UU6	-
Os02g0258000	Hypothetical conserved gene.	CT835450	Q6ETP6	-
Os02g0258200	Similar to high mobility group family.	AK070270	XP_002303636.1	High mobility group, HMG1/HMG2
Os02g0258250	Hypothetical conserved gene.	CT837539	XP_002299486.1	High mobility group, HMG1/HMG2
Os02g0258300	Similar to extra-large G-protein-related.	AK104067	NP_192018.1	-
Os02g0260800	Non-protein coding transcript.	EU941912	NONE	-
Os02g0260900	Similar to H0315A08.1 protein.	FP099421	Q01I63	Ribonuclease H1, N-terminal
Os02g0289300	Hypothetical conserved gene.	AK062485	NP_001173375.1	-
Os02g0305950	Similar to calmodulin binding protein.	tplb0019a16 (Wheat FLcDNA)	NP_001149909.1	Auxin responsive SAUR protein
Os02g0306801	Hypothetical gene.	EU943056	longestORF	-
Os02g0306900	Similar to DnaJ-like protein.	AK100011 ,AK070441	A2Q505	Heat shock protein DnaJ, N-terminal
Os02g0508650	Non-protein coding transcript.	FP094256	NONE	-
Os02g0508801	Similar to predicted protein.	EU959920	Q6K2G2	Protein of unknown function DUF3339
Os02g0519900	Similar to H0613H07.5 protein.	AK060953	Q01MK8	Translation elongation factor EFG/EF2, C-terminal
Os02g0521300	C2 domain containing protein.	AK120851	NP_001147038.1	C2 calcium-dependent membrane targeting
Os02g0521366	Similar to HAT family dimerisation domain containing protein.	tplb0053n06 (Wheat FLcDNA)	Q7XE06	Zinc finger, BED-type predicted
Os02g0521432	Hypothetical protein.	tplb0030l08 (Wheat FLcDNA)	longestORF	-
Os02g0521500	Peptidase C65, otubain domain containing protein.	AK335298	B9F0A6	Peptidase C65, otubain
Os02g0521600	Ovarian tumour, otubain domain containing protein.	AK362134	B9F0A7	Ovarian tumour, otubain
Os02g0549200	Similar to Ser Thr specific protein kinase-like protein.	AK069491	NP_001167697.1	Protein kinase, catalytic domain
Os02g0549467	Non-protein coding transcript.	EU947769	NONE	-
Os02g0557800	A-type response regulator, Cytokinin signaling	AK070645 ,AB249662 ,BR00031	Q6YVX7	Signal transduction response regulator, receiver domain
Os02g0562300	Hypothetical conserved gene.	AK104737 ,AK061441	B8ADY6	Calmodulin binding protein-like
Os02g0562350	Non-protein coding transcript.	EU949450	NONE	-
Os02g0563000	Hypothetical conserved gene.	BT083559	Q6ZGB6	F-box domain, cyclin-like
Os02g0563800	Cyclin-like F-box domain containing protein.	AK067547	A2Y6M3	F-box domain, cyclin-like
Os02g0564200	Conserved hypothetical protein.	AK072445	A2X661	-
Os02g0564300	Conserved hypothetical protein.	AK242358	NP_001047159.1	-
Os02g0564400	Similar to CLPX (Clp protease regulatory subunit X)%3B ATPase.	AK243408	E5GBA0	ATPase, AAA+ type, core
Os02g0564500	SFT2-like family protein.	AK103450	B6TQJ1	Vesicle transport protein, Got1/SFT2-like
Os02g0564600	Late embryogenesis abundant protein 3 family protein.	AK318610	NP_001147317.1	Late embryogenesis abundant protein, LEA-5
Os02g0565000	Homeodomain-like containing protein.	AK120665	NP_001151900.1	-
Os02g0565500	Similar to Pto kinase interactor 1.	AK072522	Q709M1	Protein kinase, catalytic domain
Os02g0565600	Similar to Homeodomain leucine zipper protein (Fragment).	AK064118	Q0E0A6	Homeobox
Os02g0567000	Conserved hypothetical protein.	AK068282	B8AEC4	-
Os02g0567200	Protein phosphatase 2C domain containing protein.	AK069840	NP_001151313.1	Protein phosphatase 2C-like
Os02g0567450	Hypothetical gene.	BT085317	GeneMark	-
Os02g0568600	Hypothetical conserved gene.	ab initio prediction	Q6YTF9	-
Os02g0583300	Similar to OSIGBa0132O24.3 protein.	EU951279	Q01MB4	-
Os02g0583500	Transposase, Ptta/En/Spm, plant domain containing protein.	EU976982	NP_001047258.2	Transposase, Ptta/En/Spm, plant
Os02g0585200	Heavy metal transport/detoxification protein domain containing protein.	AK287653	NP_001152411.1	Heavy metal-associated domain, HMA
Os02g0587600	Conserved hypothetical protein.	ab initio prediction	NP_001047273.1	-
Os02g0587800	Virulence factor, pectin lyase fold family protein.	AK243611	NP_001173047.1	-
Os02g0588300	Similar to Fimbrin 1 (AtFIM1).	AK119439	NP_001147889.1	Actinin-type, actin-binding, conserved site
Os02g0590200	Lecithin:cholesterol/phospholipid:diacylglycerol acyltransferase domain containing protein.	AK241245	NP_001047281.2	Lecithin:cholesterol/phospholipid:diacylglycerol acyltransferase
Os02g0591850	Non-protein coding transcript.	AK242359	NONE	-
Os02g0591900	Protein kinase, catalytic domain domain containing protein.	AK060750	Q69L76	Protein kinase, catalytic domain
Os02g0592000	Similar to OSIGBa0106G07.8 protein.	AK361746	Q01IX6	Oxoglutarate/iron-dependent oxygenase

Os02g0592300	Similar to predicted protein.	AK073685	NP_567236.1	DNA mismatch repair protein
Os02g0592500	Folate receptor, conserved region domain containing protein.	AK070526	B9F0U2	Folate receptor-like
Os02g0592600	Phospholipase C, phosphatidylinositol-specific, Y domain domain containing protein.	AK099683	A2WUV9	Phospholipase C, phosphatidylinositol-specific, Y domain
Os02g0592700	Conserved hypothetical protein.	AK107229	A2X6P0	-
Os02g0593400	Conserved hypothetical protein.	AK064226	XP_002454106.1	-
Os02g0593500	Phosphate transporter family protein.	AK067498	Q7YME0	Phosphate transporter
Os02g0593550	Hypothetical gene.	EU972267	longestORF	-
Os02g0593600	Leucine-rich repeat, typical subtype containing protein.	AK069976	Q01IY1	Leucine-rich repeat
Os02g0593700	Similar to TA4 protein (Fragment).	AK072838	B8K1X5	Phox/Bem1p
Os02g0593900	Methyltransferase-related domain containing protein.	AK288086	B6TN81	Methyltransferase-related
Os02g0594100	Similar to Protein kinase ATN1.	AK067412	Q7XJ65	Protein kinase, catalytic domain
Os02g0594300	Similar to Enhancer of shoot regeneration ESR1.	EU946638	NP_001147345.1	Pathogenesis-related transcriptional factor/ERF, DNA-binding
Os02g0598500	Protein phosphatase 2C family protein.	AK106903	NP_001047333.1	Protein phosphatase 2C, manganese/magnesium aspartate binding site
Os02g0598800	Telomere length regulation protein, conserved domain domain containing protein.	AK372629	NP_001047336.2	Telomere length regulation protein, conserved domain
Os02g0644400	Similar to Kinesin heavy chain (Fragment).	ab initio prediction	Q93XG3	Kinesin, motor domain
Os02g0658800	Beta-expansin.	AK059638	Q6H676	Major pollen allergen Lol pI
Os02g0658850	Hypothetical protein.	EU947703	longestORF	-
Os02g0662300	Conserved hypothetical protein.	AK121727	B8AGC6	-
Os02g0663900	Hypothetical conserved gene.	AK064204	Q6EUH6	Pentatricopeptide repeat
Os02g0664200	Similar to UDP-glucose:glycoprotein glucosyltransferase 1 precursor (EC 2.4.1.-) (UDP-glucose cera	AK059298	NP_177278.3	Glycosyl transferase, family 8
Os02g0665250	Conserved hypothetical protein.	AK242407	NP_001173107.1	-
Os02g0666250	Hypothetical gene.	EU947829	longestORF	-
Os02g0666550	Hypothetical protein.	ab initio prediction	NONE	-
Os02g0667100	Conserved hypothetical protein.	AK370258	NP_001047679.1	-
Os02g0668000	Hypothetical protein.	AK108411	GeneMark	-
Os02g0668100	Similar to Geranylgeranyl-diphosphate synthase.	AK111401	A9ZN21	Polyprenyl synthetase
Os02g0668900	Similar to H0211B05.3 protein.	ab initio prediction	Q01JA9	Lipase, GDSL
Os02g0669100	Dehydrin family protein.	AK070197	B1NEV7	Dehydrin
Os02g0669366	Hypothetical gene.	EU942482	longestORF	-
Os02g0669500	Similar to H0211B05.7 protein.	AK111117	Q01JA5	Protein of unknown function DUF241, plant
Os02g0672400	Sugar transporter, conserved site domain containing protein.	AK064365	B8AGF1	-
Os02g0673100	Similar to ALMT3.	AK108963	A5YBP9	Malate transporter, aliminium toerance
Os02g0673301	Hypothetical gene.	AK335180	longestORF	-
Os02g0674050	Non-protein coding transcript.	EU970724	NONE	-
Os02g0679200	Pentatricopeptide repeat domain containing protein.	AK110789	B8AGT4	Pentatricopeptide repeat
Os02g0679700	Protein of unknown function DUF623, plant domain containing protein.	AK108178	B8AGP6	Domain of unknown function DUF623
Os02g0680500	Pentatricopeptide repeat domain containing protein.	AK106401	B8AGP8	Pentatricopeptide repeat
Os02g0681100	Protein of unknown function DUF604 family protein.	AK100584	Q01KI0	Protein of unknown function DUF604
Os02g0682700	Similar to protein binding protein.	ab initio prediction	NP_001150294.1	-
Os02g0683100	Hypothetical protein.	tplb0021g13 (Wheat FLcDNA)	longestORF	-
Os02g0683500	Similar to DNA-binding protein RAV1.	AK109908	D9MNL6	Transcriptional factor B3
Os02g0684300	Nucleoporin protein Ndc1-Nup domain containing protein.	AK108514	A3AA67	Nucleoporin protein Ndc1-Nup
Os02g0684500	Similar to Histone H4.	FP095140	XP_002310853.1	Histone H4
Os02g0685350	Hypothetical gene.	EU951957	longestORF	-
Os02g0689550	Hypothetical protein.	tplb0058j15 (Wheat FLcDNA)	longestORF	-
Os02g0691650	Hypothetical gene.	EU948983	longestORF	-
Os02g0695900	Similar to oligopeptide transporter 4.	AK062910	NP_001152089.1	Oligopeptide transporter OPT superfamily
Os02g0696200	Hypothetical protein.	AK353783	longestORF	-
Os02g0697400	Non-protein coding transcript.	AK060627	NONE	-
Os02g0697650	Hypothetical gene.	EU963805	longestORF	-
Os02g0697900	Hypothetical protein.	AK119485	longestORF	-
Os02g0698000	Similar to Phosphoribulokinase, chloroplast precursor (EC 2.7.1.19) (Phosphopentokinase) (PRKase	AK099461	Q8GRU9	Phosphoribulokinase
Os02g0704262	Conserved hypothetical protein.	CT835588	B9GCT9	-
Os02g0717550	Non-protein coding transcript.	BT018944	NONE	-
Os02g0718100	Non-protein coding transcript.	AK109216	NONE	-
Os02g0720000	Conserved hypothetical protein.	AK108806	Q6ZI16	-
Os02g0720100	Cupredoxin domain containing protein.	AK063724	A3AAS9	Plastocyanin-like
Os02g0720250	Non-protein coding transcript.	tplb0026b06 (Wheat FLcDNA)	NONE	-
Os02g0720450	Hypothetical protein.	EU947645	longestORF	-
Os02g0720500	Peptidase A1 domain containing protein.	AK108932	B6SL29	Peptidase A1
Os02g0720900	Similar to Aspartic proteinase nepenthesin-1.	AK072228	B6TP02	Peptidase A1
Os02g0721000	Hypothetical protein.	AK108039	longestORF	-
Os02g0721100	Similar to E2 ubiquitin-conjugating enzyme UbcH5B (Fragment).	AK108167	A2X916	Ubiquitin-conjugating enzyme, E2
Os02g0725300	Similar to cDNA clone:J033042D19, full insert sequence.	AK111344	B7ERT8	-
Os02g0725600	tRNA (guanine-N1-)-methyltransferase, eukaryotic domain containing protein.	AK058341	A2Q1R6	tRNA
Os02g0726300	Similar to AOBP (Ascorbate oxidase promoter-binding protein).	AK101321 ,AK100892	Q58FK8	Zinc finger, Dof-type
Os02g0729700	Similar to HAHB-7 (Fragment).	AK058364	B7F510	Helix-turn-helix motif, lambda-like repressor
Os02g0731600	Similar to Threonine endopeptidase.	AK058271	B6TCN7	-
Os02g0735900	Zinc finger, RING/FYVE/PHD-type domain containing protein.	AK072424	B6THV3	Zinc finger, RING-type
Os02g0736700	Conserved hypothetical protein.	EU945911	NP_001048056.1	-
Os02g0736800	Conserved hypothetical protein.	AK073311	B8AI34	-
Os02g0737401	Hypothetical conserved gene.	AK241122	Q6Z733	-
Os02g0738100	Conserved hypothetical protein.	AK241905	NP_001173146.1	-
Os02g0743200	Conserved hypothetical protein.	AK108304	A3AB98	-
Os02g0744150	Hypothetical gene.	BT016845	longestORF	-
Os02g0745250	Similar to NAC domain class transcription factor.	AY742218	D9ZJ98	No apical meristem
Os02g0745300	Conserved hypothetical protein.	ab initio prediction	Q6ZGU0	-
Os02g0748100	Hypothetical conserved gene.	AK111248	Q6YUW6	-
Os02g0748300	Similar to Kelch motif family protein.	AK100919	B6SXJ9	Kelch repeat type 1
Os02g0750400	Pentatricopeptide repeat domain containing protein.	AK059134	A3ABE1	Pentatricopeptide repeat
Os02g0753100	Non-protein coding transcript.	BT086933	NONE	-
Os02g0753800	Similar to Annexin p35.	AK101787	A2X9Q4	Annexin
Os02g0753850	Non-protein coding transcript.	tplb0051f01 (Wheat FLcDNA)	NONE	-
Os02g0753900	Hypothetical conserved gene.	AK242419	NP_001048150.2	-
Os02g0755450	Hypothetical gene.	EU941442	longestORF	-
Os02g0758000	Similar to Low molecular weight heat shock protein precursor (Mitochondrial small heat shock prote	AK105464 ,AK064389 ,AK07400	E5D3K1	Heat shock protein Hsp20
Os02g0762400	Cyclin-dependent kinase inhibitor family protein.	AK103084	Q6Z6G5	Cyclin-dependent kinase inhibitor
Os02g0767900	Glutaredoxin 2 family protein.	AK099885	NP_001151051.1	Glutaredoxin-like
Os02g0768800	Similar to predicted protein.	AK063192	E5GCG2	-
Os02g0768950	Non-protein coding transcript.	CU405652	NONE	-
Os02g0785000	Glycosyl transferase, family 31 protein.	AK103670	NP_200558.1	Glycosyl transferase, family 31
Os02g0787000	Allergen V5/Tpx-1 related family protein.	ab initio prediction	NP_001048338.1	Allergen V5/Tpx-1-related
Os02g0796750	Non-protein coding transcript.	EU943327	NONE	-
Os02g0800500	Similar to Cyclin-B1-2.	AK099813	Q0DWQ7	Cyclin, N-terminal
Os02g0801200	Similar to Cyclin-B1-2.	AK069086	Q0DWQ7	-
Os02g0801210	Non-protein coding transcript.	CT831260	NONE	-
Os02g0801450	Similar to OSJNba0093F12.23 protein.	AK065428	Q5JQR9	-
Os02g0807600	Hypothetical gene.	AK062825	GeneMark	-
Os02g0811101	Conserved hypothetical protein.	FP095473	NP_001147425.1	-
Os02g0813183	Hypothetical protein.	tplb0043a14 (Wheat FLcDNA)	longestORF	-
Os02g0813200	Myb/SANT-like domain domain containing protein.	EU975340	NP_001048483.2	Myb/SANT-like domain
Os02g0814700	Similar to Ribosomal Pr 117 (Fragment).	AK121994	Q7X9K1	Ribosomal protein L14b/L23e
Os02g0814900	Similar to nicotinamide-nucleotide adenylyltransferase 1.	ab initio prediction	NP_001150381.1	Cytidylyltransferase
Os02g0820100	Hypothetical protein.	FP092063	longestORF	-
Os02g0823500	Non-protein coding transcript.	U16257	NONE	-
Os02g0824400	Conserved hypothetical protein.	AK121390	B8AF55	-
Os02g0824500	Similar to Remorin.	AK111296	NP_001159012.1	Remorin, C-terminal
Os02g0826016	Hypothetical protein.	tplb0042k12 (Wheat FLcDNA)	longestORF	-
Os02g0826533	Non-protein coding transcript.	tplb0033e11 (Wheat FLcDNA)	NONE	-
Os02g0826566	F-box domain, cyclin-like domain containing protein.	AK367617	B8AF61	F-box domain, cyclin-like
Os02g0826700	F-box domain, cyclin-like domain containing protein.	ab initio prediction	Q6K7R1	F-box domain, cyclin-like
Os02g0827200	Similar to Sucrose transporter.	AK065430	Q6YK44	Major facilitator superfamily
Os02g0827300	Protein of unknown function DUF382 domain containing protein.	AK069159	A2Q200	PSP, proline-rich
Os02g0827400	Similar to predicted protein.	CT835495	NP_001048593.1	Protein of unknown function DUF789
Os02g0827500	Hypothetical conserved gene.	AK242677	Q6K7Q5	Domain of unknown function DUF2404

Os02g0828300	Conserved hypothetical protein.	AK108744	B8BG05	-
Os02g0828400	Conserved hypothetical protein.	AK107588	Q6ZAX3	-
Os02g0830750	Hypothetical protein.	BT086967	longestORF	-
Os03g0100010	Conserved hypothetical protein.	AK333943	A2XBE9	-
Os03g0100020	Similar to predicted protein.	AK073982	NP_199482.1	Tetraspanin, subgroup
Os03g0100030	CAAX amino terminal protease domain containing protein.	AK068177	A3AD50	CAAX amino terminal protease
Os03g0100050	Inner nuclear membrane protein MAN1 domain containing protein.	AK066383	Q10T70	Inner nuclear membrane protein MAN1
Os03g0100225	Non-protein coding transcript.	EU949846	NONE	-
Os03g0100300	Peptidyl-prolyl cis-trans isomerase, cyclophilin-type domain containing protein.	AK106235	NP_567522.5	Peptidyl-prolyl cis-trans isomerase, cyclophilin-type
Os03g0100400	Similar to DNA repair endonuclease UVH1 (EC 3.1.-.-) (Ultraviolet hypersensitive 1) (AtRAD1) (D	AK068556 ,AK121880	NP_198931.1	ERCC4 domain
Os03g0100900	Protein of unknown function DUF862, eukaryotic domain containing protein.	AK103335	B4FKS4	Domain of unknown function DUF862, eukaryotic
Os03g0101100	Similar to Palmitoyl-protein thioesterase-like.	AK120839	Q10T54	Palmitoyl protein thioesterase
Os03g0101200	Similar to U-box domain containing protein, expressed.	ab initio prediction	Q10T52	Protein kinase, catalytic domain
Os03g0101250	Hypothetical gene.	AK241727	longestORF	-
Os03g0101300	Similar to Hexose transporter.	AK100524	NP_001149551.1	Sugar/inositol transporter
Os03g0101400	Hypothetical protein.	AK120679	longestORF	-
Os03g0101500	Similar to Phosphoglycerate mutase family protein.	AK109299	C3SAD0	PRIB5
Os03g0101600	Similar to Oxidoreductase, zinc-binding dehydrogenase family protein, expressed.	AK061143 ,AK069374	Q10T48	Alcohol dehydrogenase superfamily, zinc-type
Os03g0101700	Similar to SNF2P.	AK066228	Q8GSF4	SNF2-related
Os03g0101800	Similar to DUF614 containing protein.	AK103196 ,AK061092 ,AK10439	C3SAD5	Uncharacterised protein family Cys-rich
Os03g0102000	Conserved hypothetical protein.	AK062867	B9FA98	-
Os03g0102100	Similar to formyltetrahydrofolate deformylase.	AK071358	NP_001152471.1	Formyl transferase, N-terminal
Os03g0102200	Similar to DNA-directed RNA polymerase II 14.5 kDa polypeptide (EC 2.7.7.6) (RPB9) (RPB14.5).	AK120183	Q10T39	Zinc finger, TFIIS-type
Os03g0102300	Hypothetical conserved gene.	BT068414	Q10T37	-
Os03g0102333	Hypothetical conserved gene.	EU971180	Q10T37	CCR4-Not complex, Not1 subunit, domain of unknown function DUF3819
Os03g0102366	Similar to transcriptional regulator-related.	AK362299	NP_171710.3	CCR4-Not complex component, Not1, C-terminal
Os03g0102400	Similar to 2-phosphoglycerate kinase-related.	AK121721	NP_200953.1	-
Os03g0108400	Similar to Charged multivesicular body protein 3.	AK242193	B6SM72	Snf7
Os03g0108500	Similar to 4,4-dimethyl-sterol C4-methyl-oxidase (Fragment).	AK108704	B4FN64	Fatty acid hydroxylase
Os03g0108600	Similar to DEAD-box ATP-dependent RNA helicase 50.	AK065776	Q8GUG7	Helicase, C-terminal
Os03g0108700	Conserved hypothetical protein.	AK105115	Q8H7P7	-
Os03g0109350	Non-protein coding transcript.	AK374302	NONE	-
Os03g0109650	Hypothetical conserved gene.	AK289246 ,AK243040	Q8H7V1	Protein of unknown function DUF3755
Os03g0109800	Hypothetical conserved gene.	AK066015	Q8H7V3	-
Os03g0110300	Conserved hypothetical protein.	AK106206 ,AK066190 ,AK06676	B9FAD8	-
Os03g0110400	Similar to nuclear matrix protein-related.	AK100395	NP_568219.1	THO complex, subunit THOC1
Os03g0110900	Dimeric alpha-beta barrel domain containing protein.	AK070403	Q10SU2	Dimeric alpha-beta barrel
Os03g0111000	Conserved hypothetical protein.	AK062759	B8ALG1	-
Os03g0111100	Similar to Dihydrofolate synthetase /folylpolyglutamate synthetase.	AK102025	B6SW32	Folylpolyglutamate synthetase
Os03g0111500	Hypothetical conserved gene.	AK067241	Q10ST5	-
Os03g0112400	Clathrin/coatomer adaptor, adaptin-like, appendage, C-terminal subdomain domain containing protei	AK063082	Q10SS6	Clathrin adaptor, alpha-adaptin, appendage, C-terminal subdomain
Os03g0112600	Similar to Plus-3 domain containing protein, expressed.	EU942983	Q7XCX5	SWIB/MDM2 domain
Os03g0113000	Serine/threonine protein kinase domain containing protein.	AK071309	NP_001151054.1	Protein kinase, catalytic domain
Os03g0113100	Similar to Thymidine kinase.	AK066124	O81263	Thymidine kinase
Os03g0113500	Conserved hypothetical protein.	AK241800	B9FAE9	-
Os03g0113800	Tetratricopeptide-like helical domain containing protein.	AK065925	XP_002870606.1	Tetratricopeptide-like helical
Os03g0114850	Non-protein coding transcript.	BT063951	NONE	-
Os03g0117050	Non-protein coding transcript.	AK241608	NONE	-
Os03g0121300	Similar to Peroxidase 1.	AK064633	Q8LMR7	Plant peroxidase
Os03g0122034	Non-protein coding transcript.	EU950997	NONE	-
Os03g0124300	Similar to ATP binding protein.	AK106076	B6TWY5	Malectin-like carbohydrate-binding domain
Os03g0127000	Similar to cell growth defect factor 2.	AK068479	NP_001148819.1	-
Os03g0127800	Non-protein coding transcript.	EU947022	NONE	-
Os03g0127950	Similar to inner membrane protein ybaL.	CT835134	NP_001147290.1	-
Os03g0130800	Non-protein coding transcript.	AK058647	NONE	-
Os03g0137050	Non-protein coding transcript.	BT086855	NONE	-
Os03g0137600	Conserved hypothetical protein.	AK061189	B9FAZ2	-
Os03g0137700	Mg2+ transporter protein, CorA-like domain containing protein.	AK069498	Q10S25	-
Os03g0137750	Hypothetical protein.	tplb0043b08 (Wheat FLcDNA)	longestORF	-
Os03g0172000	Peptidase S59, nucleoporin family protein.	AK122024	NP_178183.2	Peptidase S59, nucleoporin
Os03g0174200	Conserved hypothetical protein.	AK103762 ,AK060616	A2XD32	-
Os03g0174400	Hypothetical conserved gene.	EF028768	Q10R18	Pathogenesis-related transcriptional factor/ERF, DNA-binding
Os03g0174800	Conserved hypothetical protein.	AK069179	B9FBL5	-
Os03g0174900	Similar to RAPB protein.	AK069348	O49915	CCAAT-binding transcription factor, subunit B
Os03g0175200	Similar to predicted protein.	AK242803	NP_001147647.1	Mitochondrial substrate/solute carrier
Os03g0176200	Hypothetical protein.	EU949353	longestORF	-
Os03g0176300	Hypothetical conserved gene.	AY685115	B9FBM1	Pathogenesis-related transcriptional factor/ERF, DNA-binding
Os03g0177000	Acyl-CoA N-acyltransferase domain containing protein.	AK071368	NP_671784.2	GCN5-related N-acetyltransferase
Os03g0177900	EF-1 alpha.	AK119647 ,AK119528 ,AK07134	B8APM5	Protein synthesis factor, GTP-binding
Os03g0178000	EF-1 alpha.	AK072285	B8APM5	Protein synthesis factor, GTP-binding
Os03g0178150	Hypothetical gene.	BT086892	longestORF	-
Os03g0178200	Protein of unknown function YGGT family protein.	AK106024	A6MZH3	Uncharacterised protein family Ycf19
Os03g0178300	Hypothetical conserved gene.	AK121217	A2XD59	-
Os03g0178400	Alpha/beta hydrolase fold-1 domain containing protein.	AK108257	NP_001152422.1	Alpha/beta hydrolase fold-1
Os03g0178550	Hypothetical gene.	AK289159	longestORF	-
Os03g0179000	Similar to Tubulin-tyrosine ligase family protein, expressed.	AK069518	Q10QY3	Tubulin-tyrosine ligase
Os03g0179100	Similar to predicted protein.	CT835279	Q8H021	Germin
Os03g0179700	Similar to OSIGBa0140O07.11 protein.	CT835157	Q01J32	Tubby, C-terminal
Os03g0179950	Non-protein coding transcript.	CT835230	NONE	-
Os03g0180000	Sodium/calcium exchanger membrane region domain containing protein.	AK360521	NP_564650.1	Sodium/calcium exchanger membrane region
Os03g0180100	Protein of unknown function DUF1677, plant domain containing protein.	AK108326	B8APN4	Protein of unknown function DUF1677, plant
Os03g0180300	Hypothetical conserved gene.	AK061621	Q10QX0	Smr protein/MutS2 C-terminal
Os03g0180400	Proteasome subunit alpha type 6 (EC 3.4.25.1) (20S proteasome alpha subunit A) (20S proteasome s	AK101031 ,AK059929 ,AK06002	A2XD75	Proteasome, alpha-subunit, conserved site
Os03g0180700	Glycosyl transferase, group 1 domain containing protein.	AK069776	NP_001149006.1	Glycosyl transferase, family 1
Os03g0180800	Similar to ZIM motif family protein.	AK070649	NP_001151145.1	Tify
Os03g0181500	Similar to (Rice Genome Annotation Project) fiddlehead-like protein.	AK100798	Q10QW0	3-Oxoacyl-[acyl-carrier-protein
Os03g0182700	Eukaryotic translation initiation factor 3 subunit 12 (eIF-3 p25) (eIF3k).	AK073293 ,AK120235	Q94HF1	-
Os03g0183000	Similar to AP2 domain containing protein RAP2.6 (Fragment).	AK060527	Q94HF3	Pathogenesis-related transcriptional factor/ERF, DNA-binding
Os03g0183050	Hypothetical gene.	EU941438	longestORF	-
Os03g0183300	Pathogenesis-related transcriptional factor and ERF domain containing protein.	AK106163 ,AK105922 ,AK11955	Q5MFV0	Pathogenesis-related transcriptional factor/ERF, DNA-binding
Os03g0183800	Similar to Leucine-rich repeat transmembrane protein kinase 1 (Fragment).	AK112046	O81105	Protein kinase, catalytic domain
Os03g0184050	Non-protein coding transcript.	AK058668	NONE	-
Os03g0187200	Non-protein coding transcript.	AK060541	NONE	-
Os03g0187500	Leucine-rich repeat, cysteine-containing subtype containing protein.	AK059536	Q8H7P5	-
Os03g0188200	Zinc finger, RING/FYVE/PHD-type domain containing protein.	AK058578	NP_001149891.1	Zinc finger, RING-type
Os03g0190100	UbiA prenyltransferase family protein.	AK061907 ,AK066332	B6UA12	UbiA prenyltransferase family
Os03g0190500	Hypothetical gene.	AK110854	longestORF	-
Os03g0191200	WW/Rsp5/WWP domain containing protein.	AK070228	A2XDF1	WW/Rsp5/WWP
Os03g0191550	Non-protein coding transcript.	X06284	NONE	-
Os03g0192550	Hypothetical gene.	AK111026	longestORF	-
Os03g0192900	Similar to Ubiquitin-specific protease 26.	AK105104	B9F5M9	-
Os03g0195400	Hypothetical gene.	AK064567	longestORF	-
Os03g0195450	Similar to sulfate/bicarbonate/oxalate exchanger and transporter sat-1.	CT835280	D4IIA9	Sulphate transporter/antisigma-factor antagonist STAS
Os03g0197050	Non-protein coding transcript.	CT835219	NONE	-
Os03g0197100	Similar to Sugar transporter protein.	AK099273 ,AK070417 ,AK10391	B6TSL9	Sugar/inositol transporter
Os03g0197125	Hypothetical gene.	tplb0043f10 (Wheat FLcDNA)	longestORF	-
Os03g0197825	Hypothetical protein.	BT086922	longestORF	-
Os03g0230300	Poly(ADP-ribose) polymerase, catalytic region domain containing protein.	AK102303	B8AJL8	Poly
Os03g0231000	Conserved hypothetical protein.	AY554031	A3AFR6	-
Os03g0231150	Similar to Hox19 (Fragment).	CU406260	Q6Q7D4	Leucine zipper, homeobox-associated
Os03g0231600	Similar to Branched-chain-amino-acid aminotransferase 3, chloroplast precursor (EC 2.6.1.42) (Atbc	AK120579 ,AK105963	A2XE78	Aminotransferase, class IV
Os03g0231650	Hypothetical protein.	tplb0043l14 (Wheat FLcDNA)	longestORF	-
Os03g0243700	Similar to Glycosyl hydrolase family 10 protein, expressed.	AK059149	Q10P81	Glycoside hydrolase, family 10
Os03g0243750	Hypothetical protein.	tplb0048l17 (Wheat FLcDNA)	longestORF	-
Os03g0244750	Conserved hypothetical protein.	ab initio prediction	NP_001183257.1	-

Os03g0246500	Non-protein coding transcript.	AK105050	NONE	-
Os03g0275350	Non-protein coding transcript.	EU948346	NONE	-
Os03g0280800	Similar to UDP-D-glucuronate decarboxylase.	AK071034	NP_001151221.1	NAD-dependent epimerase/dehydratase
Os03g0280900	Hypothetical protein.	tplb0036f05 (Wheat FLcDNA)	longestORF	-
Os03g0281100	Ankyrin repeat containing protein.	BT086250	NP_172250.1	Ankyrin repeat
Os03g0281201	Conserved hypothetical protein.	AK287938	Q10N64	-
Os03g0281466	Similar to Protein kinase (Pto kinase) (Serine/threonine protein kinase Pto).	AK241347	longestORF	-
Os03g0281500	Similar to Resistance protein candidate (Fragment).	AK100137	C6ZRT9	Protein kinase, catalytic domain
Os03g0281600	Similar to Ca2+-ATPase.	AK070260	Q4PKC7	ATPase, P-type, K/Mg/Cd/Cu/Zn/Na/Ca/Na/H-transporter
Os03g0281800	Conserved hypothetical protein.	AK067596	Q8H8V8	-
Os03g0281900	ABC transporter-like domain containing protein.	AK072135	NP_001151504.1	ABC transporter-like
Os03g0282232	Conserved hypothetical protein.	ab initio prediction	B9F7D7	-
Os03g0282300	Hypothetical conserved gene.	BT038365	B9F7D7	-
Os03g0282700	DEAD-like helicase, N-terminal domain containing protein.	ab initio prediction	Q8H8U9	Helicase, C-terminal
Os03g0282800	Conserved hypothetical protein.	AK101658	B8ALI3	-
Os03g0282900	Conserved hypothetical protein.	AK102161 ,AK061166	B8ALI4	-
Os03g0283000	Similar to In2-1 protein.	AY332468	Q8H8U5	Glutathione S-transferase, N-terminal
Os03g0283100	Similar to In2-1 protein.	AK061849	Q8H8U5	Glutathione S-transferase, N-terminal
Os03g0283300	Conserved hypothetical protein.	AK070169	Q10N42	-
Os03g0283400	Conserved hypothetical protein.	AK064670	B9F7E1	-
Os03g0283500	Pentatricopeptide repeat domain containing protein.	AK103244	B8ALI6	Pentatricopeptide repeat
Os03g0283600	CDP-alcohol phosphatidyltransferase domain containing protein.	AK070093	B6SU69	CDP-alcohol phosphatidyltransferase
Os03g0283650	Hypothetical protein.	ab initio prediction	NONE	-
Os03g0283750	Conserved hypothetical protein.	AK241622	NP_001173371.1	-
Os03g0283800	Similar to TBC domain containing protein, expressed.	AK072879	Q10N37	-
Os03g0283850	Hypothetical protein.	tplb0061a18 (Wheat FLcDNA)	longestORF	-
Os03g0283900	Similar to Serine/threonine-protein kinase PBS1 (EC 2.7.1.37) (AvrPphB susceptible protein 1).	AK105569	Q32SF8	Protein kinase, catalytic domain
Os03g0284100	Similar to Two-component response regulator-like PRR73.	AK121136	Q10N34	Signal transduction response regulator, receiver domain
Os03g0284150	Hypothetical protein.	tplb0030a07 (Wheat FLcDNA)	longestORF	-
Os03g0284200	Non-protein coding transcript.	AK062789	NONE	-
Os03g0286200	Similar to P-protein.	AK105065	B6SYB7	Prephenate dehydratase
Os03g0295800	Similar to gamma-interferon-inducible lysosomal thiol reductase.	AK106050	B6TUH5	Gamma interferon inducible lysosomal thiol reductase GILT
Os03g0295866	Transcription factor IIS, N-terminal domain containing protein.	BT040964	Q10MT6	Transcription factor IIS, N-terminal
Os03g0295932	Hypothetical protein.	EU965145	longestORF	-
Os03g0296600	Similar to ECA1 protein.	AK109176	Q9SES8	Protein of unknown function DUF784
Os03g0296700	Non-protein coding transcript.	AK121470	NONE	-
Os03g0298100	Conserved hypothetical protein.	AK369609	NP_001173382.1	-
Os03g0298400	Similar to 26S protease regulatory subunit 4 homolog.	AK058779	P46466	ATPase, AAA-type, core
Os03g0299200	Similar to Alcohol dehydrogenase (Fragment).	AK100767	P93795	Short-chain dehydrogenase/reductase SDR
Os03g0299400	Hypothetical protein.	EU954997	longestORF	-
Os03g0299600	Wound-induced protein, Wun1 domain containing protein.	AK068151	XP_002468012.1	Wound-induced protein, Wun1
Os03g0299800	Hypothetical conserved gene.	AK100929	B8AM23	Uncharacterised protein family Cys-rich
Os03g0300000	Similar to Xyloglucan 6-xylosyltransferase (EC 2.4.2.39) (AtXT1).	AK060863	NP_001150077.1	Galactosyl transferase
Os03g0300300	WD40 repeat-like domain containing protein.	AK099693	B8AM26	WD40 repeat
Os03g0300400	Pathogen-related protein (JIOsPR10).	AK121376	Q945E9	Bet v I allergen
Os03g0300500	Similar to Pectin methylesterase 6 (Fragment).	AK070513	A2XFP7	Pectinesterase, catalytic
Os03g0300600	Similar to Chaperone protein dnaJ.	AK066250	B6U761	Heat shock protein DnaJ, N-terminal
Os03g0300700	Retrotransposon gag protein family protein.	AK071770	A2XFP9	Retrotransposon gag protein
Os03g0301550	Hypothetical gene.	AK288301	longestORF	-
Os03g0301600	Conserved hypothetical protein.	AK062764	B9F7W2	-
Os03g0301700	Similar to Calmodulin-binding protein phosphatase.	AK101878	B6TFE7	Protein phosphatase 2C-like
Os03g0301950	Peroxisomal biogenesis factor 11 family protein.	Q10MN3	Q10MN3	Peroxisomal biogenesis factor 11
Os03g0302000	Peroxisomal biogenesis factor 11 family protein.	AK287976	Q10MN2	Peroxisomal biogenesis factor 11
Os03g0302100	Non-protein coding transcript.	AK063197	NONE	-
Os03g0302200	Zinc finger, RING/FYVE/PHD-type domain containing protein.	AK120853	A2XFQ9	Zinc finger, RING-type
Os03g0302700	Zinc finger, RING/FYVE/PHD-type domain containing protein.	AK112010	NP_001152943.1	Zinc finger, RING-type
Os03g0304100	Conserved hypothetical protein.	AK111121	B8AMI6	-
Os03g0304500	Tetratricopeptide-like helical domain containing protein.	AK101290	XP_002874939.1	Tetratricopeptide TPR-1
Os03g0304550	Hypothetical conserved gene.	BT085356	longestORF	-
Os03g0304800	Lg106-like family protein.	AK062656	NP_001148326.1	Endosulphine
Os03g0305500	Similar to Argininosuccinate lyase.	AK070233	B4FAV4	Fumarate lyase
Os03g0305550	Hypothetical gene.	EU949424	longestORF	-
Os03g0305600	Mitochondrial import inner membrane translocase, subunit Tim17/22 family protein.	AK063714	Q2HU23	Mitochondrial inner membrane translocase subunit Tim17/Tim22/Tim23/peroxisomal p
Os03g0305800	Galactosyl transferase family protein.	AK105101 ,AK105005	A2XFT5	-
Os03g0306200	Similar to transducin family protein / WD-40 repeat family protein.	AK241828	XP_002865733.1	Bromodomain
Os03g0306302	Hypothetical conserved gene.	DQ245981	Q10MJ9	-
Os03g0306400	Similar to Lon protease homolog, mitochondrial.	AK288546	A2YQ56	-
Os03g0306800	Similar to CP12 (Fragment).	AK103722	B7XB92	Domain of unknown function CP12
Os03g0306900	Haem oxygenase-like, multi-helical domain containing protein.	AK073626	B6TPF2	TENA/THI-4 protein/Coenzyme PQQ biosynthesis protein C domain
Os03g0307000	Conserved hypothetical protein.	AK243201	NP_001049892.2	-
Os03g0320100	Alpha-L-arabinofuranosidase, C-terminal domain containing protein.	AK062471	Q10M79	Alpha-L-arabinofuranosidase, C-terminal
Os03g0320800	Similar to Leucine Rich Repeat family protein, expressed.	AK103265	Q10M75	Leucine-rich repeat
Os03g0320850	Hypothetical gene.	BT061105	longestORF	-
Os03g0320900	Guanylate kinase family protein.	AK106255	NP_001149581.1	Guanylate kinase
Os03g0321700	Similar to WRKY transcription factor 55.	AK101653	A6N066	DNA-binding WRKY
Os03g0321800	Similar to WRKY transcription factor 55.	AK111447	NP_001030961.1	Octanoyltransferase
Os03g0322000	Similar to Centromere protein-like protein (Fragment).	AY224484	Q10M62	-
Os03g0324650	Non-protein coding transcript.	BT085189	NONE	-
Os03g0325500	Similar to Phosphate starvation regulator protein (Regulatory protein of P- starvation acclimation res	AK100282	A2XG76	Myb-like DNA-binding domain, SHAQKYF class
Os03g0325600	Similar to WUSCHEL-related homeobox 6.	AK111000	Q10M29	Homeobox
Os03g0327100	Similar to CUC1.	AK065989	NP_001147731.1	No apical meristem
Os03g0327900	Conserved hypothetical protein.	EU963434	Q8H7M0	-
Os03g0328000	Dedicator of cytokinesis family protein.	AK059469	NP_193367.7	Dedicator of cytokinesis
Os03g0328400	Similar to Apyrase GS50 (Fragment).	AF358764	D3Y1I0	Nucleoside phosphatase GDA1/CD39
Os03g0328450	Non-protein coding transcript.	BT017220	NONE	-
Os03g0328600	Hypothetical protein.	EU949280	longestORF	-
Os03g0329200	Similar to cDNA clone:J013002B05, full insert sequence.	AK064318	B7F3U2	-
Os03g0329700	Similar to predicted protein.	AK242883	Q10LZ2	Protein kinase, catalytic domain
Os03g0336100	11-S plant seed storage protein family protein.	AK105307	Q10LT1	Cupin 1
Os03g0336400	Similar to Alpha-expansin OsEXPA4.	AK107698	Q9M4X7	Expansin
Os03g0336700	PAP/25A core domain containing protein.	AK120611	Q10LS4	PAP/25A-associated
Os03g0336900	Similar to Uncharacterized plant-specific domain TIGR01568 family protein, expressed.	EU972618	NP_001050045.2	Domain of unknown function DUF623
Os03g0338800	Hypothetical conserved gene.	AK064660	B8B3T4	-
Os03g0339150	Hypothetical gene.	AK241704	longestORF	-
Os03g0342900	Dormancyauxin associated family protein.	AK104391 ,AK060136 ,AK06098	B4FA62	-
Os03g0343225	Hypothetical gene.	EU951910	longestORF	-
Os03g0343300	Small-subunit processome, Utp14 domain containing protein.	AK243183	Q10LM2	Small-subunit processome, Utp14
Os03g0343400	Similar to Photolyase/blue-light receptor PHR2.	AK105611	B6U742	DNA photolyase, FAD-binding/Cryptochrome, C-terminal
Os03g0343500	Similar to 60S ribosomal protein L22-2.	AK059599	B6U998	Ribosomal protein L22e
Os03g0343700	Brix domain containing protein.	AK060603	XP_002864754.1	Brix domain
Os03g0343800	Conserved hypothetical protein.	EU976763	NP_001050080.1	-
Os03g0343900	Photosystem II PsbX domain containing protein.	AK105813	GeneMark	Photosystem II PsbX
Os03g0344232	Conserved hypothetical protein.	CT835238	Q10RS8	-
Os03g0344650	Hypothetical protein.	EU943048	longestORF	-
Os03g0345100	Similar to auxin response factor 75.	AK065579	NP_001149782.1	Rad9
Os03g0345200	40S ribosomal protein S21.	AK073724 ,AK104084 ,AK11926	P35687	Ribosomal protein S21e
Os03g0345300	Similar to Desiccation-related protein Lb_13-62 (Fragment).	AK108277	B1PMG8	-
Os03g0345500	Non-protein coding transcript.	EU975566	NONE	-
Os03g0345700	Similar to Heavy metal-associated domain containing protein, expressed.	AK066080	Q10LJ9	-
Os03g0345901	Hypothetical gene.	EU973702	longestORF	-
Os03g0348200	Terpene synthase-like domain containing protein.	EU954571	NP_001050103.1	Terpene synthase-like
Os03g0348900	Similar to CHY zinc finger family protein, expressed.	AK073680	Q10LI0	Zinc finger, RING-type
Os03g0349200	Similar to transposon protein.	AK101089	NP_001148044.1	-
Os03g0349400	Hypothetical protein.	tplb0040h06 (Wheat FLcDNA)	longestORF	-
Os03g0351100	Similar to CONSTANS-like protein CO9 (Fragment).	AK101822	C0PI33	Zinc finger, B-box

Os03g0351200	NAD(P)-binding domain containing protein.	AK353935	D5L1S4	NmrA-like
Os03g0351300	Similar to beta-amylase.	BT041352	NP_001151271.1	Glycoside hydrolase, family 14
Os03g0351400	Similar to Tubby-like protein 3.	AK102370	Q10LG8	Tubby, C-terminal
Os03g0355200	WD40 repeat-like domain containing protein.	BT055024	NP_181681.1	WD40 repeat
Os03g0355400	WD40 repeat-like domain containing protein.	BT055024	NP_181681.1	WD40 repeat
Os03g0355800	Similar to Diphthine synthase (EC 2.1.1.98) (Diphthamide biosynthesis methyltransferase).	AK103902	B8APT0	Tetrapyrrole methylase
Os03g0355900	Hypothetical conserved gene.	CT835254	NP_001050138.1	-
Os03g0356414	Similar to Ubiquitin ligase SINAT5 (EC 6.3.2.-) (Seven in absentia homolog 5). Splice isoform 2.	AK058336	B6SRI2	Zinc finger, RING-type
Os03g0363700	Pentatricopeptide repeat domain containing protein.	EU954511	NP_001050167.1	Pentatricopeptide repeat
Os03g0368150	Hypothetical protein.	BT085962	longestORF	-
Os03g0373300	Protein of unknown function DUF1110 domain containing protein.	AK107897	Q84TT5	Protein of unknown function DUF1110
Os03g0383800	Similar to SAP domain containing protein, expressed.	AK062998	Q10KI0	DNA-binding SAP
Os03g0387900	Hypothetical conserved gene.	AK063085	Q10KE2	-
Os03g0388000	Similar to Splicing factor SC35.	AK073057	B6T6J9	RNA recognition motif domain
Os03g0388500	Similar to Anther ethylene-upregulated protein ER1 (Fragment).	AK070350	Q10KD1	IQ motif, EF-hand binding site
Os03g0388650	Hypothetical protein.	EU971393	longestORF	-
Os03g0392250	Hypothetical gene.	AK288273	longestORF	-
Os03g0392300	Similar to GTP-binding protein-like.	AK071779	B4FF78	Small GTP-binding protein domain
Os03g0392400	Heat shock protein DnaJ, N-terminal domain containing protein.	AK106027 ,AK073726	B6TYB9	Heat shock protein DnaJ, N-terminal
Os03g0392833	Hypothetical protein.	EU949881	longestORF	-
Os03g0395200	EF-hand-like domain domain containing protein.	ab initio prediction	Q94LH2	EF-hand-like domain
Os03g0419200	Hypothetical gene.	AK106284	GeneMark	-
Os03g0419700	Hypothetical conserved gene.	ab initio prediction	Q75GC7	-
Os03g0437100	Zinc finger, C2H2-type domain containing protein.	AK108997	A2XIC5	Zinc finger, C2H2
Os03g0437200	Zinc finger, C2H2-type domain containing protein.	AK059839	A2XIC8	Zinc finger, C2H2
Os03g0564600	Protein of unknown function DUF26 domain containing protein.	AK108950	NP_001148207.1	Gnk2-homologous domain
Os03g0589500	Conserved hypothetical protein.	AK105481	Q5W6J4	-
Os03g0593100	Conserved hypothetical protein.	AK287562	NP_001050587.1	-
Os03g0605400	Non-protein coding transcript.	AK063612	NONE	-
Os03g0609400	Non-protein coding transcript.	AK241806	NONE	-
Os03g0609500	Similar to LOB domain protein 39.	AK288002	NP_201543.1	Lateral organ boundaries, LOB
Os03g0617101	Non-protein coding transcript.	CT835260	NONE	-
Os03g0627300	Similar to nucleotide-binding protein-like.	AK106686	NP_001150831.1	Cobyrinic acid a,c-diamide synthase
Os03g0633900	Nucleic acid-binding, OB-fold-like domain containing protein.	AK068169 ,AK059181	NP_001148836.1	Primosome PriB/single-strand DNA-binding
Os03g0635100	Heterotrimeric G protein gamma subunit 1.	AK241226	B8AN27	-
Os03g0637800	Regulator of chromosome condensation/beta-lactamase-inhibitor protein II domain containing protei	AK101339	Q948R7	Regulator of chromosome condensation, RCC1
Os03g0637900	Similar to Centromere protein-like protein (Fragment).	AK060224	Q84VB6	-
Os03g0638000	Similar to Centromere protein-like protein (Fragment).	AY224555	Q84VD9	-
Os03g0642900	Similar to Hydrolase-like protein.	BT055664	B4FSG2	-
Os03g0643000	Similar to Hydrolase-like protein.	AK366873	B4FSG2	-
Os03g0643100	Homeodomain-like containing protein.	AK106587	Q60DP5	Myb/SANT-like domain
Os03g0643300	Similar to Ornithine-oxo-acid transaminase.	AK108010	A7Y209	Aminotransferase class-III
Os03g0643611	Hypothetical protein.	ab initio prediction	NONE	-
Os03g0644200	Pentatricopeptide repeat domain containing protein.	AK073773	B6SWY6	Pentatricopeptide repeat
Os03g0644400	Amino acid permease.	AK067118	Q9LUH7	Amino acid transporter, transmembrane
Os03g0644600	Protein of unknown function DUF761, plant domain containing protein.	AK376083	Q60DN1	Protein of unknown function DUF761, plant
Os03g0645100	Similar to pyruvate dehydrogenase E1 component subunit beta.	AK103256	NP_001150473.1	Transketolase-like, pyrimidine-binding domain
Os03g0645200	Similar to Translation initiation factor eIF-2B delta subunit (eIF-2B GDP-GTP exchange factor).	AK060478	NP_001159071.1	Initiation factor 2B-related
Os03g0645250	Hypothetical protein.	tplb0051c11 (Wheat FLcDNA)	longestORF	-
Os03g0646850	Non-protein coding transcript.	BT017109	NONE	-
Os03g0647400	GCK domain containing protein.	AK073665	Q9LMF3	GCK
Os03g0647500	Similar to GTP binding protein.	AK064308	B6U832	-
Os03g0647550	Non-protein coding transcript.	AF124739	NONE	-
Os03g0647575	Hypothetical protein.	BT086909	longestORF	-
Os03g0648100	Protein of unknown function DUF266, plant family protein.	AK109329	A3AKV3	Glycosyl transferase, family 14
Os03g0648400	Non-protein coding transcript.	AK058555	NONE	-
Os03g0648500	Cupredoxin domain containing protein.	AK107766	A2XV11	Plastocyanin-like
Os03g0650300	Hypothetical conserved gene.	tplb0014g09 (Wheat FLcDNA)	Q43713	-
Os03g0650350	Hypothetical gene.	EU947173	longestORF	-
Os03g0650400	Similar to ameiotic 1.	AK064367	NP_001139538.1	-
Os03g0650700	Translation elongation factor EFG/EF2, C-terminal domain containing protein.	AK102195	XP_002885535.1	Translation elongation factor EFG/EF2, C-terminal
Os03g0650800	Protein of unknown function DUF593 family protein.	ab initio prediction	NP_001050787.1	Protein of unknown function DUF593
Os03g0650850	Non-protein coding transcript.	AK242527	NONE	-
Os03g0650900	Zinc finger, RING/FYVE/PHD-type domain containing protein.	AK103022	NP_181426.2	Zinc finger, RING-type
Os03g0651950	Hypothetical protein.	tplb0048i05 (Wheat FLcDNA)	longestORF	-
Os03g0652000	C2 domain containing protein.	AK110820 ,AK107712	Q7XZZ4	-
Os03g0653601	Non-protein coding transcript.	AK242503	NONE	-
Os03g0653900	Hypothetical conserved gene.	ab initio prediction	B9FA32	Ribosome-inactivating protein
Os03g0655200	Similar to Membrin 11 (AtMEMB11) (Golgi SNAP receptor complex member 2-1) (27 kDa Golgi S	AK111247	B6TMP4	-
Os03g0655300	CS domain domain containing protein.	BT061748	B6T9V0	CS-like domain
Os03g0655400	Similar to LIP5.	AK059150	GeneMark	-
Os03g0656201	Similar to chaperone protein dnaJ-related.	AK240680	NP_197286.1	Heat shock protein DnaJ, cysteine-rich domain
Os03g0656500	Similar to K-exchanger-like protein.	AK121052	Q8W0Q9	Sodium/calcium exchanger membrane region
Os03g0656800	Similar to 3-glucanase.	AK069506	NP_001147672.1	Glycoside hydrolase, family 17
Os03g0656850	Conserved hypothetical protein.	BT019195	C4J0K6	-
Os03g0657000	TATA-binding protein TBP2.	AK061103 ,AK099162	Q8W0W4	TATA-box binding protein
Os03g0657100	Similar to predicted protein.	AK066133	NP_564125.2	Armadillo-like helical
Os03g0657400	Hypothetical conserved gene.	ab initio prediction	NP_001050814.2	DNA-binding WRKY
Os03g0659200	Similar to Ferredoxin.	AK062598	NP_001151112.1	Ferredoxin
Os03g0659233	Cytochrome b559 subunit alpha.	P0C368	P0C368	Photosystem II cytochrome b559, conserved site
Os03g0659266	Cytochrome b559 subunit beta.	P0C399	P0C399	Photosystem II cytochrome b559, conserved site
Os03g0659300	Glyoxalase/bleomycin resistance protein/dioxygenase domain containing protein.	AK241252	NP_001148888.1	-
Os03g0659350	Hypothetical gene.	AK288422	longestORF	-
Os03g0659400	Hypothetical conserved gene.	AK111930	A2XK97	-
Os03g0660500	Auxin responsive SAUR protein domain containing protein.	AK361840	Q75GQ1	Auxin responsive SAUR protein
Os03g0660700	Conserved hypothetical protein.	AK109543	B9FA57	-
Os03g0661250	Hypothetical protein.	EU941142	longestORF	-
Os03g0661300	Beta-tubulin (Beta-3 tubulin) (Tubulin beta subunit).	AK121321	Q76FS2	Tubulin
Os03g0661500	Conserved hypothetical protein.	AK100254	A2XKB1	-
Os03g0661600	Similar to Alpha-amylase/trypsin inhibitor (Antifungal protein).	AK058637	O04364	Thaumatin, pathogenesis-related
Os03g0661700	Non-protein coding transcript.	AK073701	NONE	-
Os03g0661900	Peptidase, trypsin-like serine and cysteine domain containing protein.	AK072616	A3AL41	Peptidase S7, flavivirus helicase
Os03g0663400	Similar to Thaumatin-like protein.	AK102970 ,AK102798 ,AK11916	Q40630	-
Os03g0663500	Similar to Osmotin precursor.	U77656	NP_001147526.1	Thaumatin, pathogenesis-related
Os03g0663600	Similar to Pathogenesis-related thaumatin-like protein.	EU967040	O04363	-
Os03g0663750	Hypothetical protein.	tplb0024b15 (Wheat FLcDNA)	longestORF	-
Os03g0663800	Globulin 1 (Fragment).	AK105347	Q10FL8	Cupin 1
Os03g0663900	Hypothetical conserved gene.	EU948060	NP_001050836.1	Bifunctional inhibitor/plant lipid transfer protein/seed storage
Os03g0664300	F-box domain, cyclin-like domain containing protein.	tplb0015o19 (Wheat FLcDNA)	Q75GY3	F-box domain, cyclin-like
Os03g0664400	Similar to Protease inhibitor/seed storage/LTP family protein.	AK058628	B6U491	Plant lipid transfer protein/seed storage/trypsin-alpha amylase inhibitor
Os03g0664600	Plant lipid transfer protein/seed storage/trypsin-alpha amylase inhibitor domain containing protein.	AK370248	Q75GY8	Plant lipid transfer protein/seed storage/trypsin-alpha amylase inhibitor
Os03g0664650	Hypothetical gene.	CT835252	longestORF	-
Os03g0664700	RNA polymerase II accessory factor, Cdc73 domain containing protein.	AK100794	B8ANM7	RNA polymerase II accessory factor, Cdc73
Os03g0664800	Acyl-CoA N-acyltransferase domain containing protein.	AK243094	NP_001150788.1	GCN5-related N-acetyltransferase
Os03g0664901	Hypothetical protein.	EU957022	longestORF	-
Os03g0665000	GCN5-related N-acetyltransferase (GNAT) domain domain containing protein.	EU969219	NP_001050840.2	GCN5-related N-acetyltransferase
Os03g0665200	Protein of unknown function DUF581 family protein.	AK066202	B8ANM9	Protein of unknown function DUF581
Os03g0665700	Similar to predicted protein.	DQ245183	NP_001050842.2	Protein of unknown function DUF1677, plant
Os03g0665751	Hypothetical gene.	BT086957	longestORF	-
Os03g0665800	Armadillo-type fold domain containing protein.	AK107693	NP_191180.2	Armadillo-like helical
Os03g0665950	Conserved hypothetical protein.	AK241231	NP_001173576.1	-
Os03g0666300	Hypothetical conserved gene.	BT083602	Q75H97	MYB-like
Os03g0666500	Similar to Ras-related protein RHA1.	AK061203	B4F7V6	Small GTPase superfamily
Os03g0666550	Hypothetical protein.	tplb0055b17 (Wheat FLcDNA)	longestORF	-
Os03g0666600	UDP-glucuronosyl/UDP-glucosyltransferase family protein.	AK064709	NP_001151546.1	UDP-glucuronosyl/UDP-glucosyltransferase

Os03g0666700	TRAM, LAG1 and CLN8 homology domain containing protein.	AK061721 ,AK061309 ,AK10201	NP_001149791.1	TRAM/LAG1/CLN8 homology domain
Os03g0666850	Hypothetical conserved gene.	AK240914	B9ETH0	-
Os03g0667100	Similar to NPR1-like protein.	AK065952	B6VAC8	BTB/POZ-like
Os03g0667150	Hypothetical protein.	tplb0036n13 (Wheat FLcDNA)	longestORF	-
Os03g0667201	Photosystem II reaction center protein L.	P0C416	P0C416	Photosystem II PsbL
Os03g0667400	Similar to Iron regulated metal transporter.	AK119409	NP_178033.1	Plant organelle RNA recognition domain
Os03g0667700	Similar to RanBP1 domain containing protein, expressed.	AK240716	Q10FH4	Ran binding protein 1
Os03g0667800	Ribosomal protein S21e family protein.	ab initio prediction	P35687	Ribosomal protein S21e
Os03g0668033	Hypothetical gene.	AK288472	longestORF	-
Os03g0668100	F-box domain, cyclin-like domain containing protein.	tplb0009f23 (Wheat FLcDNA)	GeneMark	F-box domain, cyclin-like
Os03g0668500	Conserved hypothetical protein.	AK064871	Q7Y198	-
Os03g0669100	Similar to predicted protein.	AK063742	Q10FF9	DeoxyUTP pyrophosphatase
Os03g0669200	Similar to GTP binding protein2.	AK242811	E0AEC9	G-protein, beta subunit
Os03g0669300	Beta-1,3-glucanase-like protein.	AK120903	NP_001148381.1	Glycoside hydrolase, family 17
Os03g0669450	Hypothetical protein.	BT019229	longestORF	-
Os03g0672950	Hypothetical protein.	tplb0048l17 (Wheat FLcDNA)	longestORF	-
Os03g0673400	Conserved hypothetical protein.	AK243310	NP_001173580.1	-
Os03g0681201	Similar to predicted protein.	CT835232	NP_176121.2	-
Os03g0681750	Hypothetical gene.	CT835257	longestORF	-
Os03g0685750	Hypothetical conserved gene.	CT833489	NP_195008.5	-
Os03g0685800	Hypothetical protein.	AK058278	longestORF	-
Os03g0698400	Non-protein coding transcript.	AK058933	NONE	-
Os03g0699100	WD40 repeat-like domain containing protein.	AK110715	B6TYC2	WD40 repeat
Os03g0701100	Similar to Prp18 domain containing protein, expressed.	AK109455 ,AK110740	Q53RB6	Splicing factor motif
Os03g0702600	Hypothetical conserved gene.	CT828111	Q75I87	-
Os03g0705433	Conserved hypothetical protein.	ab initio prediction	NP_001173605.1	-
Os03g0707250	Non-protein coding transcript.	AK242476	NONE	-
Os03g0708000	Phospholipase A2 family protein.	AK105828	Q10E50	Phospholipase A2, eukaryotic
Os03g0708100	Phytanoyl-CoA dioxygenase family protein.	AK103821	NP_565262.1	Phytanoyl-CoA dioxygenase
Os03g0708400	Protein of unknown function DUF1295 family protein.	AK066216	NP_565068.1	3-oxo-5-alpha-steroid 4-dehydrogenase, C-terminal
Os03g0708700	Protein of unknown function DUF593 domain containing protein.	EU940948	NP_001051039.2	Protein of unknown function DUF593
Os03g0708750	Hypothetical gene.	AK288565	longestORF	-
Os03g0708800	Hypothetical conserved gene.	AK121625	F1DK22	Protein of unknown function DUF573
Os03g0709100	Similar to Basic blue protein (Cusacyanin) (Plantacyanin) (CBP).	AK061596	B4FTG0	Plastocyanin-like
Os03g0709200	NB-ARC domain containing protein.	AK318615	NP_001173609.1	Disease resistance protein
Os03g0709300	Similar to Chemocyanin precursor (Basic blue protein) (Plantacyanin).	AK100153	B6SXE9	Blue
Os03g0710000	Protein of unknown function DUF292, eukaryotic domain containing protein.	AK060065	A2XL85	Domain of unknown function DUF292, eukaryotic
Os03g0710100	Similar to pairing protein meu13-like.	AK102972	B6TAH2	Tat binding protein 1-interacting
Os03g0710550	Conserved hypothetical protein.	AK288133	NP_001173611.1	-
Os03g0710600	Conserved hypothetical protein.	AK068746	A2XL91	-
Os03g0710900	Similar to CCAAT displacement protein-related / CDP-related.	AK369948	NP_566611.1	CASP, C-terminal
Os03g0711200	Similar to Phosphatidylinositol 4-kinase.	AK063479	Q8L5D9	Phosphatidylinositol 3-/4-kinase, catalytic
Os03g0711525	Hypothetical protein.	tplb0039i16 (Wheat FLcDNA)	longestORF	-
Os03g0711600	Similar to DNA binding protein (Fragment).	AK288161	Q10E13	Histone H1/H5
Os03g0712000	Similar to Lipase family protein.	ab initio prediction	Q10E06	Lipase, class 3
Os03g0712200	Zinc finger, RanBP2-type domain containing protein.	AK073205 ,AK098968	Q9AUQ9	Zinc finger, RanBP2-type
Os03g0712400	Similar to atypical receptor-like kinase MARK.	AK111063	NP_001105207.1	Protein kinase, catalytic domain
Os03g0712500	Hypothetical protein.	BT086967	longestORF	-
Os03g0712700	Similar to Phosphoglucomutase, cytoplasmic 2 (EC 5.4.2.2) (Glucose phosphomutase 2) (PGM 2).	AK099746 ,AK072313	A5HSI1	Alpha-D-phosphohexomutase superfamily
Os03g0712733	Hypothetical gene.	BT016600	longestORF	-
Os03g0712900	Pentatricopeptide repeat domain containing protein.	AK103803	Q10DZ7	Pentatricopeptide repeat
Os03g0713100	Similar to dynamin-related protein 1C.	AK243639	NP_001151519.1	Dynamin central domain
Os03g0713150	Hypothetical protein.	tplb0034e16 (Wheat FLcDNA)	longestORF	-
Os03g0713200	Conserved hypothetical protein.	AK060373	A2XLC2	-
Os03g0713400	Similar to NADH-ubiquinone oxidoreductase 75 kDa subunit, mitochondrial precursor (EC 1.6.5.3)	AK071307	C5WN22	NADH:ubiquinone oxidoreductase, 75kDa subunit, conserved site
Os03g0713600	Aldo/keto reductase domain containing protein.	AK074014	NP_001149104.1	RNA recognition motif domain
Os03g0714100	Conserved hypothetical protein.	AK241691	NP_001173615.1	-
Os03g0715332	Hypothetical conserved gene.	CT828621	Q8W337	-
Os03g0715400	Membrane protein,Tapt1/CMV receptor domain containing protein.	AK101931	B9FB76	Membrane protein,Tapt1/CMV receptor
Os03g0717000	Similar to TMK protein.	AK062249	Q10DW6	Protein kinase, catalytic domain
Os03g0717200	Cytochrome b561 family protein.	EU969254	B6TW89	Cytochrome b561, eukaryote
Os03g0717700	Histidine kinase, Cytokinin signaling	BR000246	A1A698	-
Os03g0717900	Similar to ABRH7 (Fragment).	AK099485	E4MX37	Protein of unknown function DUF760
Os03g0717950	Hypothetical protein.	tplb0028e15 (Wheat FLcDNA)	longestORF	-
Os03g0718000	Similar to Anthranilate synthase beta chain.	AK105178	Q8L9I8	Carbamoyl-phosphate synthase, GATase domain
Os03g0718100	Actin 1.	AK100267 ,AK058421	Q10DV7	Actin-like
Os03g0718150	Hypothetical protein.	BT018641	longestORF	-
Os03g0718200	Zinc finger, SWIM-type domain containing protein.	AK063808	Q10DV6	Zinc finger, PMZ-type
Os03g0718300	Hypothetical protein.	AK108509	longestORF	-
Os03g0718650	Hypothetical protein.	EU955192	longestORF	-
Os03g0718800	Similar to Physical impedance induced protein.	AK288124	NP_001149478.1	Plant lipid transfer protein/seed storage/trypsin-alpha amylase inhibitor
Os03g0719000	MAP65/ASE1 family protein.	AK108923	XP_002875099.1	Microtubule-associated protein, MAP65/ASE1-type
Os03g0719450	Hypothetical gene.	EU946282	longestORF	-
Os03g0719850	Similar to Protein kinase 2.	BT038499	Q709M0	Protein kinase, catalytic domain
Os03g0739500	Conserved hypothetical protein.	AK241255	Q5ZAF7	-
Os03g0745000	Similar to Heat stress transcription factor A-2a.	AK069579	Q84MN7	Heat shock factor
Os03g0746500	Similar to predicted protein.	AK058367	NP_193215.2	Helicase, C-terminal
Os03g0773150	40S ribosomal protein S29.	CU406248	A2ZG71	Ribosomal protein S14
Os03g0773300	Protein kinase, core domain containing protein.	AK100394	NP_001105795.1	Protein kinase, catalytic domain
Os03g0773450	Hypothetical protein.	tplb0046o04 (Wheat FLcDNA)	longestORF	-
Os03g0773700	Similar to Receptor-like protein kinase 2.	AK102670	Q7XZW7	Protein kinase, catalytic domain
Os03g0774400	Similar to predicted protein.	AK109683	Q10E11	-
Os03g0775200	Protein of unknown function DUF248, methyltransferase putative family protein.	AK120397	NP_564084.1	Protein of unknown function DUF248, methyltransferase putative
Os03g0775250	Hypothetical protein.	AK358342	longestORF	-
Os03g0775300	Flavodoxin/nitric oxide synthase domain containing protein.	AK100507	Q8H8N3-2	Radical SAM
Os03g0775400	Tetratricopeptide-like helical domain containing protein.	AK107354	A2XMF9	Pentatricopeptide repeat
Os03g0775650	Non-protein coding transcript.	tplb0059h22 (Wheat FLcDNA)	NONE	-
Os03g0775700	Conserved hypothetical protein.	AK067093	A2XMG2	-
Os03g0775900	Conserved hypothetical protein.	AK374530	Q8H8M7	-
Os03g0777000	Similar to NAC-domain containing protein 19 (ANAC019) (ANAC) (Abscicic-acid- responsive NAC	AK073539	F1DJT1	No apical meristem
Os03g0778100	Similar to Photosystem-1 F subunit.	AK060493	Q9ZSU5	Photosystem I PsaF, reaction centre subunit III
Os03g0778400	Similar to predicted protein.	CT835114	NP_001051445.1	-
Os03g0780200	Conserved hypothetical protein.	AK107920	A2XMI6	-
Os03g0780350	Similar to SAUR1 - auxin-responsive SAUR family member.	EU974244	NP_001152203.1	Auxin responsive SAUR protein
Os03g0780400	Similar to Actin-depolymerizing factor 6 (ADF-6) (AtADF6).	AK073162 ,AK061347	Q9AY76	Actin-binding, cofilin/tropomyosin type
Os03g0780550	Hypothetical protein.	tplb0045b10 (Wheat FLcDNA)	longestORF	-
Os03g0780600	Tubulin beta-1 chain (Beta-1 tubulin).	AK120180	P37832	Tubulin
Os03g0780800	Fatty acid hydroxylase domain containing protein.	AK103085	NP_001149139.1	Fatty acid hydroxylase
Os03g0781000	Conserved hypothetical protein.	AK065910	Q9AY71	-
Os03g0781100	Pentatricopeptide repeat domain containing protein.	EU950024	Q9AY70	Pentatricopeptide repeat
Os03g0781200	Hypothetical protein.	AK110946	longestORF	-
Os03g0781300	Conserved hypothetical protein.	AK120661	F2EFE7	-
Os03g0781400	Ribose 5-phosphate isomerase family protein.	AK071970	NP_001151663.1	Ribose 5-phosphate isomerase, type A
Os03g0781501	Non-protein coding transcript.	AK243070	NONE	-
Os03g0782200	Hypothetical protein.	AK069119	GeneMark	-
Os03g0782300	Conserved hypothetical protein.	AK070713	B9F3B9	-
Os03g0782350	Hypothetical gene.	CT832459	longestORF	-
Os03g0782400	Conserved hypothetical protein.	ab initio prediction	Q10CH8	-
Os03g0782900	Pentatricopeptide repeat domain containing protein.	AK108367	Q9AY61	Pentatricopeptide repeat
Os03g0782950	Hypothetical protein.	tplb0031e02 (Wheat FLcDNA)	longestORF	-
Os03g0783000	Similar to Actin-related protein 7.	AK069426	A3ANB5	Actin-like
Os03g0783051	Conserved hypothetical protein.	FP098389	A3ANB6	-
Os03g0799900	Similar to sec12-like protein 1.	AK111711	NP_001148204.1	WD40 repeat
Os03g0800400	Protein of unknown function DUF1618 domain containing protein.	AK071430	Q851R3	Domain of unknown function DUF1618
Os03g0800900	Hypothetical conserved gene.	AK241533	Q851R7	-

Os03g0802650	Hypothetical gene.	CT837941	longestORF	-
Os03g0808100	Similar to Cellulose synthase BoCesA5.	AK058615	Q4U0Z5	Cellulose synthase
Os03g0808150	Hypothetical gene.	BT019277	longestORF	-
Os03g0808175	Non-protein coding transcript.	U48693	NONE	-
Os03g0808200	UDP-glucuronosyl/UDP-glucosyltransferase family protein.	BT037819	NP_001149762.1	UDP-glucuronosyl/UDP-glucosyltransferase
Os03g0808350	Hypothetical protein.	AK361927	longestORF	-
Os03g0809800	Conserved hypothetical protein.	AK241877	Q10BN7	-
Os03g0810900	ARF/SAR superfamily domain containing protein.	AK121284	XP_002871068.1	Patatin/Phospholipase A2-related
Os03g0811600	Similar to Peptidyl prolyl isomerase H.	AK071076	Q1KL26	Peptidyl-prolyl cis-trans isomerase, cyclophilin-type
Os03g0811700	Similar to Pre-mRNA branch site protein p14 (SF3B 14 kDa subunit).	AK121661 ,AK059288	B6T0W0	RNA recognition motif domain
Os03g0811800	Ribosomal protein L36 family protein.	AK063320	B8AM87	Ribosomal protein L36
Os03g0811900	Similar to ADP-ribosylation factor.	AK122007	Q6S4R7	Small GTP-binding protein domain
Os03g0812000	DNA topoisomerase, type IIA, subunit A or C-terminal domain containing protein.	AK059281	Q7XZF7	DNA gyrase/topoisomerase IV, subunit A, C-terminal beta-pinwheel
Os03g0824000	Peptidase S8 and S53, subtilisin, kexin, sedolisin domain containing protein.	AK111129	A9NKD9	Protein of unknown function DUF3743
Os03g0831300	Protein of unknown function DUF789 family protein.	AK073782	Q850Z6	Protein of unknown function DUF789
Os03g0831400	Beta tubulin, autoregulation binding site domain containing protein.	AK071202	A2XNN8	Protein of unknown function DUF3511
Os03g0831500	Similar to Phosphoribosylformylglycinamidine cyclo-ligase, chloroplast/mitochondrial precursor (EC	AK101211	B8AMR2	AIR synthase-related protein
Os03g0832400	Similar to Protein phosphatase 2C-like protein.	AK120748	B4G0F7	Protein phosphatase 2C-like
Os03g0832800	Non-protein coding transcript.	AK062843	NONE	-
Os03g0832900	Similar to DNAJ-like protein (Fragment).	AK060945	Q84VA4	Heat shock protein DnaJ, N-terminal
Os03g0833200	Methyltransferase TRM13 domain containing protein.	AK102870	B8AMR9	Methyltransferase TRM13
Os03g0833500	Hypothetical protein.	AK119356	longestORF	-
Os03g0833600	X8 domain containing protein.	AK109880	Q10B16	X8
Os03g0833700	RNA 3'-terminal phosphate cyclase family protein.	AK121918	NP_680196.1	RNA 3&apos%3B-terminal phosphate cyclase
Os03g0833900	Similar to Cytosine deaminase (EC 3.5.4.1).	AK073655 ,AK104068	NP_001105963.1	-
Os03g0834000	Flap endonuclease-1b (EC 3.-.-.-) (OsFEN-1b).	AK062149	Q75LI2	5'-3' exonuclease, N-terminal
Os03g0834050	Similar to predicted protein.	AK240756	B6TQ05	HAD-superfamily hydrolase, subfamily IA, variant 3
Os03g0834150	Non-protein coding transcript.	X06284	NONE	-
Os03g0834466	Similar to MADS-box transcription factor 21.	BT008965	D3U2H6	Transcription factor, MADS-box
Os03g0835100	Similar to Chloroplast protein import component Toc159.	AK373768	Q75LI9	Chloroplast protein import component Toc86/159
Os03g0835150	Conserved hypothetical protein.	AK243368	NP_001173702.1	-
Os03g0835300	Hypothetical protein.	AK064539	longestORF	-
Os03g0835400	Similar to Uvs101.	AK061773 ,AK098986 ,AK10589	Q75LJ3	Electron transfer flavoprotein, alpha subunit
Os03g0835800	Galactose oxidase/kelch, beta-propeller domain containing protein.	AK102903	Q10AZ7	Kelch repeat type 1
Os03g0835900	Similar to Ferredoxin III, chloroplast precursor (Fd III).	AK121063	D3JUX1	Ferredoxin
Os03g0835950	Non-protein coding transcript.	AK370418	NONE	-
Os03g0842700	Conserved hypothetical protein.	AK062057	B9F7I9	-
Os03g0842900	Similar to Steroleosin-B.	AK107425	Q8LKV5	Short-chain dehydrogenase/reductase SDR
Os03g0843000	Non-protein coding transcript.	AK064669	NONE	-
Os03g0843100	Ras GTPase family protein.	AK111499	B4FEN1	Small GTPase superfamily
Os03g0843150	Non-protein coding transcript.	BT084300	NONE	-
Os03g0843200	Protein of unknown function DUF901 family protein.	AK063613	B6TD02	Protein of unknown function DUF901
Os03g0843300	Late embryogenesis abundant protein 2 family protein.	AK099274	B6T887	Late embryogenesis abundant protein, LEA-14
Os03g0844000	Hypothetical conserved gene.	AK059033	B9F7J4	Pentatricopeptide repeat
Os03g0844301	Hypothetical gene.	CT835264	longestORF	-
Os03g0847851	Non-protein coding transcript.	CT835272	NONE	-
Os03g0856200	Similar to Longin-like.	AK058786	Q2HUA7	Longin-like
Os03g0856300	Similar to Acyl carrier protein III, chloroplast precursor (ACP III).	AK108342	A2XP91	Acyl carrier protein
Os03g0856500	Similar to Plastid-specific 30S ribosomal protein 1, chloroplast precursor (CS- S5) (CS5) (S22) (Rib	AK104733 ,AK061449	C6YXL0	Ribosomal protein S30Ae/sigma 54 modulation protein
Os04g0102600	Similar to Splicing factor 3B subunit 2 (Spliceosome associated protein 145) (SAP 145) (SF3b150) (	AK104417 ,AK070814	Q01N45	Peptidase S24/S26A/S26B/S26C, beta-ribbon domain
Os04g0125800	Similar to Growth-regulating factor 1.	EF515848	Q9SDZ5	-
Os04g0171232	Conserved hypothetical protein.	BT039178	A2WJQ3	-
Os04g0213800	Similar to Bacterial blight resistance protein XA26.	DQ119307	Q2EZ14	Protein kinase, catalytic domain
Os04g0234600	Similar to Sedoheptulose-1,7-bisphosphatase.	AK062081	Q84JG8	Fructose-1,6-bisphosphatase class 1/Sedoheputulose-1,7-bisphosphatase
Os04g0247875	Non-protein coding transcript.	EU941912	NONE	-
Os04g0249550	Hypothetical protein.	tplb0054p19 (Wheat FLcDNA)	longestORF	-
Os04g0250200	Conserved hypothetical protein.	AK058584	B8ARM3	-
Os04g0255525	Hypothetical gene.	AK242256	longestORF	-
Os04g0255600	Cytochrome P450 domain containing protein.	BT064329	Q7XQ53	Cytochrome P450
Os04g0259200	Conserved hypothetical protein.	AK119366	B8B6C2	-
Os04g0267950	Conserved hypothetical protein.	AK355731	NP_001172891.1	-
Os04g0286500	Homeodomain-like containing protein.	AK064584	B8BCY2	MYB-like
Os04g0309751	Hypothetical protein.	tplb0054p19 (Wheat FLcDNA)	longestORF	-
Os04g0313600	Zinc finger, CCHC-type domain containing protein.	AK288309	A2XRV4	Zinc finger, CCHC-type
Os04g0313900	Similar to cytokinin-O-glucosyltransferase 2.	EU976007	NP_001152029.1	UDP-glucuronosyl/UDP-glucosyltransferase
Os04g0319600	tRNA methyltransferase complex GCD14 subunit domain containing protein.	AK243633	Q01M41	tRNA methyltransferase complex GCD14 subunit
Os04g0319800	Similar to Cytokinin-O-glucosyltransferase 2 (EC 2.4.1.-) (Zeatin O- glucosyltransferase 2).	AK241629	Q01M39	UDP-glucuronosyl/UDP-glucosyltransferase
Os04g0320000	Similar to H0107B07.3 protein.	ab initio prediction	Q01M51	-
Os04g0320200	Tetratricopeptide-like helical domain containing protein.	AK069513 ,AK104028 ,AK11925	Q01M49	Pentatricopeptide repeat
Os04g0320700	Similar to Glucosyltransferase (Fragment).	AK119530 ,AK105966	Q01M48	UDP-glucuronosyl/UDP-glucosyltransferase
Os04g0321700	Similar to Serine carboxypeptidase I precursor (EC 3.4.16.5) (Carboxypeptidase C).	AK068732	Q01LN2	Peptidase S10, serine carboxypeptidase
Os04g0321800	Similar to OSIGBa0097I24.2 protein.	AK069106	Q01LN1	Protein phosphatase 2C-like
Os04g0326000	Similar to H0107B07.3 protein.	ab initio prediction	Q01M51	-
Os04g0326100	Similar to H0107B07.2 protein.	CT837883	Q01M39	UDP-glucuronosyl/UDP-glucosyltransferase
Os04g0326300	Hypothetical conserved gene.	AK242372	NP_001052474.2	-
Os04g0332700	Conserved hypothetical protein.	AK071842	Q6MWJ9	-
Os04g0333850	Non-protein coding transcript.	CT835070	NONE	-
Os04g0334700	Peptidase aspartic, catalytic domain containing protein.	AK120870	B7F2P1	Peptidase A1
Os04g0336001	Hypothetical protein.	EU945299	longestORF	-
Os04g0336600	Peptidase aspartic, catalytic domain containing protein.	AK120870	B7F2P1	Peptidase A1
Os04g0336801	Conserved hypothetical protein.	AK289258	Q2QWP9	-
Os04g0337300	Similar to OSIGBa0137O04.8 protein.	AK060427	Q01KY9	-
Os04g0340100	Hypothetical conserved gene.	ab initio prediction	B9FEH9	-
Os04g0340550	Hypothetical gene.	AK068264	longestORF	-
Os04g0341500	Similar to OSIGBa0159H11-OSIGBa0137A07.8 protein.	AK289110	Q01HV1	Terpene synthase, metal-binding domain
Os04g0344400	Similar to OSIGBa0106G08.5 protein.	BT041617	Q01KN9	Terpene synthase-like
Os04g0345400	Similar to OSIGBa0106G08.7 protein.	AK062280	Q01KN7	Terpene synthase-like
Os04g0351800	Conserved hypothetical protein.	FP101309	B8ASJ0	-
Os04g0351933	Conserved hypothetical protein.	FP101309	A3ASQ2	-
Os04g0352066	Conserved hypothetical protein.	AK375252	B9FEL4	-
Os04g0352200	Conserved hypothetical protein.	AK103395	A2XS74	-
Os04g0354200	Similar to OSIGBa0092G14.5 protein.	ab initio prediction	Q01KX7	FAR1 DNA binding domain
Os04g0356600	Similar to OSIGBa0105P02.3 protein.	AK066063	Q01KU2	Protein kinase, catalytic domain
Os04g0356800	Hypothetical protein.	tplb0036o12 (Wheat FLcDNA)	longestORF	-
Os04g0357500	Conserved hypothetical protein.	EU966738	B8ASQ8	-
Os04g0357700	Hypothetical conserved gene.	AK250527	NP_001052546.2	-
Os04g0360400	Hypothetical protein.	EU945918	longestORF	-
Os04g0375750	Similar to H0315A08.1 protein.	FP099421	Q01I63	Ribonuclease H1, N-terminal
Os04g0395600	Similar to TRANSPORT INHIBITOR RESPONSE 1 protein (F-box/LRR-repeat protein 1).	AK100862	XP_002328871.1	F-box domain, cyclin-like
Os04g0395651	Hypothetical protein.	tplb0032b19 (Wheat FLcDNA)	longestORF	-
Os04g0397100	Similar to ClpC protease.	AK058510	E5GBL8	Chaperonin ClpA/B
Os04g0397400	Conserved hypothetical protein.	AK059476	Q7F9I0	-
Os04g0397601	Non-protein coding transcript.	CT835050	NONE	-
Os04g0398700	Similar to Pollen specific protein C13 precursor.	AK375503	Q01L34	Allergen Ole e 1, conserved site
Os04g0402700	Hypothetical protein.	AK058995	longestORF	-
Os04g0403200	Zinc finger, RING-type domain containing protein.	AK120702	B8Q8A9	Zinc finger, RING-type
Os04g0403300	Similar to Alpha-amylase.	CT836492	C3W8N1	Alpha-amylase, C-terminal beta-sheet
Os04g0403400	Tyrosyl-DNA phosphodiesterase family protein.	AK120071	B9FEZ9	Tyrosyl-DNA phosphodiesterase
Os04g0403450	Non-protein coding transcript.	BT086661	NONE	-
Os04g0403600	Conserved hypothetical protein.	tplb0006a06 (Wheat FLcDNA)	NP_001052700.2	-
Os04g0405000	Similar to ATP-dependent Clp protease ATP-binding subunit.	AK061666	Q01L44	-
Os04g0405100	Similar to H0502B11.8 protein.	AK288033	Q01L43	Sterile alpha motif domain
Os04g0405150	Hypothetical gene.	BT019295	longestORF	-
Os04g0405700	Similar to H0502B11.13 protein.	tplb0022b18 (Wheat FLcDNA)	Q01L38	Protein of unknown function DUF677
Os04g0405800	Similar to OSIGBa0142C11.1 protein.	AK108757	Q01L58	Aspartate/glutamate/uridylate kinase

Os04g0406600	Prephenate dehydratase domain containing protein.	AK103609	Q01L56	Prephenate dehydratase
Os04g0407850	Similar to H0321H01.4 protein.	EU942896	Q01L52	-
Os04g0407900	Similar to Cytochrom P450-like protein.	AK064758	Q01L52	Cytochrome P450
Os04g0411200	Similar to ZPR3 (LITTLE ZIPPER 3)%3B protein binding.	FP099759	NP_190845.1	-
Os04g0411800	Similar to H0717B12.5 protein.	AK102032	Q01JB7	-
Os04g0414300	Similar to H0622F05.1 protein.	EU965676	Q01L95	RNA recognition motif domain
Os04g0414400	Non-protein coding transcript.	AK058617	NONE	-
Os04g0414500	Similar to H0622F05.2 protein.	AK121479	Q01L94	-
Os04g0416000	Protein of unknown function DUF1279 domain containing protein.	AK241337	Q01I48	Domain of unknown function DUF1279
Os04g0416100	Similar to Transcription factor E2F1 (E2F-1) (Fragment).	AK067581	Q01I47	Transcription factor E2F/dimerisation partner
Os04g0418000	Conserved hypothetical protein.	AK106155 ,AK061030 ,AK09912	Q7X6C3	-
Os04g0418100	Hypothetical conserved gene.	CT835067	B9FF49	-
Os04g0420900	Similar to H0525E10.7 protein.	AK064414	Q01K01	S-locus glycoprotein
Os04g0421700	FAR1 domain containing protein.	AK072998	Q01JZ9	FAR1 DNA binding domain
Os04g0421800	EF-Hand type domain containing protein.	AK068951	Q01JZ8	EF-hand-like domain
Os04g0421850	Hypothetical protein.	EU949573	longestORF	-
Os04g0431000	Conserved hypothetical protein.	AK111961	Q7XQQ0	-
Os04g0431100	Similar to GrpE protein homolog.	AK105181	A2XTK3	GrpE nucleotide exchange factor
Os04g0435700	Similar to UVB-resistance protein UVR8.	AK100857	A2XTP3	Regulator of chromosome condensation, RCC1
Os04g0436100	Thioesterase superfamily domain containing protein.	AK072631	B6SNT9	Phenylacetic acid degradation-related protein
Os04g0436300	Protein disulphide isomerase isoform/multifunctional endoplasmic reticulum luminal polypeptide (D	AK101074	B9FFA7	Thioredoxin
Os04g0436700	Similar to H0315A08.1 protein.	FP099421	Q01I63	Ribonuclease H1, N-terminal
Os04g0436800	Pentatricopeptide repeat domain containing protein.	AK101115	Q01I62	Pentatricopeptide repeat
Os04g0437000	No apical meristem (NAM) protein domain containing protein.	AK243585	Q01I69	No apical meristem
Os04g0437300	Zinc finger, RING/FYVE/PHD-type domain containing protein.	AK121647	Q01I67	U box domain
Os04g0437401	Non-protein coding transcript.	X02683	NONE	-
Os04g0439900	Similar to Translocon Tic40 precursor.	AK065119	Q01K76	Heat shock chaperonin-binding
Os04g0440000	Protein of unknown function DUF2040, coiled-coil domain containing protein.	AK102407 ,AK101691	Q01K75	Domain of unknown function DUF2040
Os04g0442700	Hypothetical gene.	AK119975	longestORF	-
Os04g0443801	Cytochrome c oxidase biogenesis protein Cmc1-like domain containing protein.	AK104938	B8BJN1	Cytochrome c oxidase biogenesis protein Cmc1-like
Os04g0446700	Hypothetical protein.	AK073019	GeneMark	-
Os04g0446750	Hypothetical gene.	AK289000	GeneMark	-
Os04g0447100	Similar to Lipoxygenase.	AK287825	Q01J86	Lipoxygenase
Os04g0447166	Hypothetical protein.	EU945269	longestORF	-
Os04g0447300	Conserved hypothetical protein.	AK111006	Q5NA50	-
Os04g0447400	Similar to Glutamate decarboxylase 2 (EC 4.1.1.15) (GAD 2).	AK070858	Q01J85	Pyridoxal phosphate-dependent decarboxylase
Os04g0447533	Hypothetical protein.	AK375890	longestORF	-
Os04g0447650	Hypothetical gene.	EU946224	longestORF	-
Os04g0447800	Glutamate decarboxylase (EC 4.1.1.15).	AK068340	Q01J81	Pyridoxal phosphate-dependent decarboxylase
Os04g0447900	Domain of unknown function DUF623 domain containing protein.	AK376132	A2XTZ6	Domain of unknown function DUF623
Os04g0448100	Protein of unknown function DUF1644 family protein.	AK108771	Q01J79	Protein of unknown function DUF1644
Os04g0448200	Similar to OSIGBa0152K17.9 protein.	AK068842	Q01J78	-
Os04g0448250	Non-protein coding transcript.	tplb0058f15 (Wheat FLcDNA)	NONE	-
Os04g0448300	Peptidase A1 domain containing protein.	AK120786	Q01J77	Peptidase A1
Os04g0448600	ChaC-like protein family protein.	AK068709	Q01J75	ChaC-like protein
Os04g0448700	Similar to H0818E04.4 protein.	AK334544	Q01JH9	-
Os04g0449000	Similar to H0818E04.8 protein.	AK105976	Q01J70	Mitochondrial substrate/solute carrier
Os04g0449400	Similar to H0818E04.11 protein.	AK241313	Q01JH2	Protein phosphatase 2C-like
Os04g0449450	Hypothetical gene.	BT085317	longestORF	-
Os04g0449900	Similar to RNA Binding Protein 47.	AK069926	Q01JG9	RNA recognition motif domain
Os04g0449950	Non-protein coding transcript.	BT009377	NONE	-
Os04g0450000	Zinc finger, FYVE/PHD-type domain containing protein.	AK108406	Q01JG8	-
Os04g0450150	Hypothetical gene.	EU948502	longestORF	-
Os04g0450200	Pentatricopeptide repeat containing protein.	AK061063	Q01JG6	Pentatricopeptide repeat
Os04g0450400	Similar to H0818E04.19 protein.	AK121979	Q01JI7	Zinc finger, RING-type
Os04g0450500	Similar to H0818E04.20 protein.	AK058975	Q01JI6	-
Os04g0450600	Similar to H0818E04.21 protein.	CT835041	Q01JI5	-
Os04g0450701	Hypothetical protein.	EU969685	GeneMark	-
Os04g0453450	Hypothetical gene.	BT061593	longestORF	-
Os04g0454900	Similar to Mitotic spindle checkpoint component mad3.	AK073191	B6U2B3	-
Os04g0455800	Similar to Lipoic acid synthetase, mitochondrial precursor (Lip-syn) (Lipoate synthase) (mLIP1).	AK120652	Q7XRF1	Lipoate synthase
Os04g0455900	Protein of unknown function DUF1692 domain containing protein.	AK121783	Q01JQ8	Domain of unknown function DUF1692
Os04g0457400	Conserved hypothetical protein.	EU953664	NP_001052973.2	-
Os04g0457500	Similar to H0523F07.13 protein.	AK241500	Q01JQ0	Gamma-glutamyltranspeptidase
Os04g0457600	Non-protein coding transcript.	X02683	NONE	-
Os04g0457700	Similar to H0523F07.14 protein.	AK242111	Q01JP9	-
Os04g0457800	Similar to SERK1 (Fragment).	AK099777	Q5Y8C8	Protein kinase, catalytic domain
Os04g0457900	Non-protein coding transcript.	AM939987	NONE	-
Os04g0458200	Similar to ORW1943Ba0077G13.1 protein.	AK101398	F2QA78	-
Os04g0458250	Hypothetical protein.	tplb0041i03 (Wheat FLcDNA)	longestORF	-
Os04g0458600	Similar to ORW1943Ba0077G13.3 protein.	AK106058	F2QA80	Aldose 1-epimerase
Os04g0459200	Hypothetical protein.	AK107705	longestORF	-
Os04g0459500	Similar to H0219H12.1 protein.	AK067817	Q01IJ6	Glyceraldehyde 3-phosphate dehydrogenase, catalytic domain
Os04g0459600	Mog1/PsbP, alpha/beta/alpha sandwich domain containing protein.	AK061516	Q01IJ5	Ran-interacting Mog1 protein
Os04g0459700	Similar to H0219H12.3 protein.	AK109192	Q01IJ4	Nucleosome assembly protein
Os04g0459800	Similar to H0219H12.4 protein.	AK073573	Q01IJ3	Helicase, C-terminal
Os04g0459900	Peptidase, trypsin-like serine and cysteine domain containing protein.	AK065850	Q01IJ2	Peptidase S1/S6, chymotrypsin/Hap
Os04g0460050	Hypothetical conserved gene.	AB332064	F2QA89	-
Os04g0460300	Amino acid transporter, transmembrane domain containing protein.	AK106202	Q01IJ0	Amino acid transporter, transmembrane
Os04g0460350	Similar to predicted protein.	CT836521	B6U9R4	-
Os04g0460400	Uncharacterised protein family UPF0497, trans-membrane plant subgroup domain containing protei	FP091536	GeneMark	Uncharacterised protein family UPF0497, trans-membrane plant subgroup
Os04g0460500	Similar to H0219H12.9 protein.	BT037263	Q01II8	Molybdopterin cofactor biosynthesis C
Os04g0460600	Similar to H0219H12.10 protein.	AK071020	Q01II7	No apical meristem
Os04g0460750	Non-protein coding transcript.	X02683	NONE	-
Os04g0460900	Non-protein coding transcript.	AK066716 ,AK065774 ,AK10402	NONE	-
Os04g0461050	Hypothetical protein.	EU971393	longestORF	-
Os04g0461100	Ribosomal protein, PSRP-3/Ycf65 domain containing protein.	AK068022	Q01II5	Ribosomal protein PSRP-3/Ycf65
Os04g0461700	Non-protein coding transcript.	AK120338	NONE	-
Os04g0461800	Actin-binding FH2 domain containing protein.	AK106770	Q7XUV2	Actin-binding FH2/DRF autoregulatory
Os04g0461900	Hypothetical protein.	AK064527	longestORF	-
Os04g0462300	Conserved hypothetical protein.	AK109651	B8AUR5	-
Os04g0462500	Similar to 14-3-3-like protein GF14-6.	AK101938	Q7XTE8	14-3-3 protein
Os04g0462550	Hypothetical gene.	BT017754	longestORF	-
Os04g0463000	Serine/threonine protein kinase domain containing protein.	AK120857	Q25A94	Protein kinase, catalytic domain
Os04g0463200	Protein of unknown function DUF593 family protein.	AK243661	NP_001053008.1	Protein of unknown function DUF593
Os04g0463300	Pentatricopeptide repeat containing protein.	AK119417	Q6MWD7	Pentatricopeptide repeat
Os04g0463400	Protein of unknown function DUF125, transmembrane family protein.	AK059730	C4B8E4	Domain of unknown function DUF125, transmembrane
Os04g0467550	Non-protein coding transcript.	BT017430	NONE	-
Os04g0467700	Similar to Indole-3-glycerol phosphate synthase, chloroplast precursor (EC 4.1.1.48) (IGPS).	AK101835	Q01IS1	Indole-3-glycerol phosphate synthase, conserved site
Os04g0469000	Heavy metal transport/detoxification protein domain containing protein.	AK062816	Q01IL4	-
Os04g0469400	Pentatricopeptide repeat domain containing protein.	AK060385	Q7XJU7	Pentatricopeptide repeat
Os04g0469425	Hypothetical protein.	AK357511	longestORF	-
Os04g0507400	Similar to OSIGBa0157A06.5 protein.	EU957725	Q01IV4	Transferase
Os04g0507450	Hypothetical gene.	BT035701	longestORF	-
Os04g0508500	Similar to OSIGBa0101P20.9 protein.	AK109620	Q01I73	SANT domain, DNA binding
Os04g0509100	Similar to triacylglycerol lipase.	AK359843	NP_193091.5	C2 calcium-dependent membrane targeting
Os04g0509600	Similar to Ammonium transporter 1 member 1.	AK073718	Q7XQ12	Ammonium transporter
Os04g0510200	Similar to STYLOSA protein.	AK108706	Q01HE9	LisH dimerisation motif
Os04g0510900	Similar to Embryo-specific protein 1 (ATS1).	AK063625	Q01HE4	Caleosin
Os04g0511200	EFA27 for EF hand, abscisic acid, 27kD.	AK243204	Q40679	Caleosin
Os04g0511500	Hypothetical protein.	EU940776	longestORF	-
Os04g0511600	Similar to RING-H2 finger protein ATL3G.	AK073609	Q01HE0	Zinc finger, RING-type
Os04g0511700	Similar to OSIGBa0157K09-H0214G12.18 protein.	AK102761	Q01HD9	-
Os04g0512225	Non-protein coding transcript.	FP092318	NONE	-
Os04g0512250	Hypothetical gene.	AK288761	longestORF	-

Os04g0512300	Arp2/3 complex, 34kDa subunit p34-Arc family protein.	AK071791	Q01HD5	Arp2/3 complex, 34kDa subunit p34-Arc
Os04g0512400	Similar to Midline 1 protein (Tripartite motif protein 18).	AK103203	Q01HD4	BRCT
Os04g0512900	NB-ARC domain containing protein.	AK067204	Q7XPY8	Disease resistance protein
Os04g0513000	Conserved hypothetical protein.	AK102028	B9FG47	-
Os04g0513700	Similar to Beta-glucosidase 16.	BT039444	Q7XSK2	Glycoside hydrolase, family 1
Os04g0513800	Hypothetical protein.	tplb0059k05 (Wheat FLcDNA)	longestORF	-
Os04g0514500	Similar to OSIGBa0140J09.3 protein.	AK073502	Q01I18	Pentatricopeptide repeat
Os04g0514800	Similar to Dual specificity kinase 1.	AK111987	Q01I14	Protein kinase, catalytic domain
Os04g0514900	Hypothetical protein.	tplb0035e01 (Wheat FLcDNA)	longestORF	-
Os04g0515400	NTP pyrophosphohydrolase MazG, putative catalytic core domain containing protein.	AK336096	Q01I10	NTP pyrophosphohydrolase MazG, putative catalytic core
Os04g0516550	Conserved hypothetical protein.	AK333114	NP_001047774.1	-
Os04g0516600	Pyridoxal phosphate-dependent transferase, major region, subdomain 1 domain containing protein.	AK059652 ,AK099280	Q01J56	Aromatic amino acid beta-eliminating lyase/threonine aldolase
Os04g0516701	Hypothetical gene.	BT017119	longestORF	-
Os04g0516800	Hypothetical protein.	AK105554	longestORF	-
Os04g0516900	Conserved hypothetical protein.	AK108714	Q7XKW4	-
Os04g0517100	Similar to OSIGBa0145M07.4 protein.	AK111798	Q01J53	Homeodomain-like
Os04g0517300	Protein of unknown function DUF647 family protein.	AK069879	Q01J52	Protein of unknown function DUF647
Os04g0517500	Similar to Phosphoenolpyruvate carboxylase kinase.	AK066885	Q2ABP5	Protein kinase, catalytic domain
Os04g0517900	Auxin responsive SAUR protein family protein.	AK241307	B5RHT9	Auxin responsive SAUR protein
Os04g0518000	Similar to Adenosine kinase (Fragment).	AK101926	Q01J47	Adenosine kinase
Os04g0518100	Phenylalanine ammonia-lyase.	AK100346	A2XVK1	Phenylalanine/histidine ammonia-lyase
Os04g0518400	Similar to Phenylalanine ammonia-lyase (Fragment).	AK067801	A2XVK3	Phenylalanine/histidine ammonia-lyase
Os04g0518650	Similar to OSIGBa0102I15.5 protein.	AK242408	Q01IW5	Small subunit of serine palmitoyltransferase-like
Os04g0518800	Similar to Lysine decarboxylase-like protein.	AK069293	Q8RUN2	Cytokinin riboside 5&apos%3B-monophosphate phosphoribohydrolase LOG
Os04g0519200	Similar to OSIGBa0132O24.3 protein.	EU951279	Q01MB4	-
Os04g0519700	Similar to Auxin response factor 10.	AK100795	Q7XKK6	Transcriptional factor B3
Os04g0519900	Similar to OSIGBa0145G11.5 protein.	AK071665	Q01I34	-
Os04g0519912	Hypothetical protein.	tplb0025b01 (Wheat FLcDNA)	longestORF	-
Os04g0519925	Similar to OSIGBa0145G11.5 protein.	ab initio prediction	Q01I34	Peptidase M28
Os04g0519950	Non-protein coding transcript.	EU954591	NONE	-
Os04g0519975	Similar to photosystem I subunit IX.	EU954469	YP_784493.1	Photosystem I PsaJ, reaction centre subunit IX
Os04g0520000	S1, RNA binding domain containing protein.	AK102988	Q01I33	RNA-binding domain, S1
Os04g0520700	Protein of unknown function DUF584 family protein.	AK065832	Q01IB5	Protein of unknown function DUF584
Os04g0520900	Pentatricopeptide repeat domain containing protein.	AK068793	Q01IB9	Pentatricopeptide repeat
Os04g0521001	Hypothetical protein.	FP096444	longestORF	-
Os04g0521100	Major intrinsic protein family protein.	AK243592	Q01IB8	Major intrinsic protein
Os04g0521300	Conserved hypothetical protein.	AK071350	B9FG70	-
Os04g0521700	Similar to OSIGBa0131L05.9 protein.	AK073622	Q01IB2	-
Os04g0521900	Similar to Vegetative cell wall protein gp1.	AK105173	B6SY74	-
Os04g0522100	Similar to OSIGBa0131L05.12 protein.	tplb0012i18 (Wheat FLcDNA)	Q01IA9	Knottin
Os04g0523200	Up-frameshift suppressor 2 domain containing protein.	AK119440	B9FG77	Up-frameshift suppressor 2
Os04g0523350	Similar to Initiation factor eIF-4 gamma, middle%3B Up-frameshift suppressor 2.	EU942804	Q2HSQ9	MIF4-like, type 1/2/3
Os04g0523600	Similar to OSIGBa0153E02-OSIGBa0093I20.8 protein.	AK070948	Q01HR7	UDP-glucuronosyl/UDP-glucosyltransferase
Os04g0523650	Hypothetical protein.	AK242174	longestORF	-
Os04g0524300	Signal transduction response regulator, receiver domain (IPR001789),CheY-like superfamily (IPR01	GO=Biological Process: two-comp	NIAS_FLcDNA=002-1(	-
Os04g0524500	Oligopeptide transporter OPT superfamily protein.	AK067235	Q01HR1	Oligopeptide transporter OPT superfamily
Os04g0524800	Hypothetical protein.	AK064753	longestORF	-
Os04g0524900	Similar to OSIGBa0153E02-OSIGBa0093I20.16 protein.	AB190921	Q01HQ9	Oligopeptide transporter OPT superfamily
Os04g0524950	Hypothetical protein.	ab initio prediction	NONE	-
Os04g0525000	Protein of unknown function DUF177 domain containing protein.	AK067753	Q01HQ8	Protein of unknown function DUF177
Os04g0525100	UDP-glucuronosyl/UDP-glucosyltransferase family protein.	AK109806	Q01HQ7	UDP-glucuronosyl/UDP-glucosyltransferase
Os04g0525600	ATP-dependent Clp protease proteolytic subunit (EC 3.4.21.92) (Endopeptidase Clp).	AK121934	Q01HQ5	Peptidase S14, ClpP
Os04g0525700	Similar to Serine carboxypeptidase II precursor (EC 3.4.16.6) (Carboxypeptidase D) (Bri1 suppressc	AK105830	Q01HQ4	Peptidase S10, serine carboxypeptidase
Os04g0525800	Similar to OSIGBa0115K01-H0319F09.1 protein.	ab initio prediction	Q01HQ1	Isochorismatase-like
Os04g0525850	Conserved hypothetical protein.	EU971861	NP_001144947.1	-
Os04g0526300	Sulfotransferase family protein.	ab initio prediction	Q01HP8	Sulfotransferase domain
Os04g0526800	GRAM domain containing protein.	AK061581	Q01HP4	GRAM
Os04g0527000	GRAM domain containing protein.	AK099545 ,AK070324 ,AK10443	Q01HP3	-
Os04g0527300	Non-protein coding transcript.	AK110653	NONE	-
Os04g0527400	BRO1 domain containing protein.	AK107295	Q01HP2	BRO1 domain
Os04g0527500	Similar to OSIGBa0115K01-H0319F09.11 protein.	AK059144	Q01HP1	Mitochondrial carrier domain
Os04g0527700	CHCH domain containing protein.	AK072980	Q01HP0	-
Os04g0527800	Similar to OSIGBa0115K01-H0319F09.13 protein.	AK104019	Q01HN9	-
Os04g0527900	Similar to Tonoplast membrane integral protein ZmTIP3-2.	AK108116	Q01HN8	Major intrinsic protein
Os04g0528000	Similar to OSIGBa0115K01-H0319F09.15 protein.	BT018594	Q01HN7	Protein of unknown function DUF789
Os04g0528100	Similar to OSIGBa0115K01-H0319F09.16 protein.	AK065957	Q01HN6	-
Os04g0528200	Similar to OSIGBa0115K01-H0319F09.17 protein.	AK064693	Q01HN5	-
Os04g0528400	Similar to ABC transporter.	AK064120	Q01HN3	Hus1-like protein
Os04g0528600	Similar to OSIGBa0115K01-H0319F09.22 protein.	AK240918	Q01HN0	-
Os04g0528651	Hypothetical protein.	tplb0055d11 (Wheat FLcDNA)	longestORF	-
Os04g0528800	Similar to Ferredoxin-thioredoxin reductase, variable chain (FTR-V) (Ferredoxin- thioredoxin reduc	AK103671	Q01HM9	Lipoate synthase
Os04g0529100	Pathogenesis-related transcriptional factor and ERF domain containing protein.	AK107680	Q01HM7	Pathogenesis-related transcriptional factor/ERF, DNA-binding
Os04g0529400	Similar to OO_Ba0013J05-OO_Ba0033A15.30 protein.	AK099627	D0ABH3	WD40 repeat
Os04g0529500	Similar to cDNA clone:J023022A09, full insert sequence.	AK059419	B7EGJ9	Double-stranded RNA-binding
Os04g0529700	Glycosyltransferase sugar-binding region containing DXD motif domain containing protein.	AK121532	Q00RJ5	Glycosyltransferase, DXD sugar-binding motif
Os04g0529800	Sugar/inositol transporter domain containing protein.	AK069679	Q00RJ4	Sugar/inositol transporter
Os04g0530801	Hypothetical gene.	EU973535	longestORF	-
Os04g0530900	Glycosyl transferase, family 8 protein.	AK072297	XP_002312381.1	Glycosyl transferase, family 8
Os04g0531300	tRNA-dihydrouridine synthase domain containing protein.	AK103911	Q00RI1	tRNA-dihydrouridine synthase
Os04g0531400	Similar to Lectin-like receptor kinase 7%3B2.	AK105289	Q00RI0	Protein kinase, catalytic domain
Os04g0531500	Concanavalin A-like lectin/glucanase domain containing protein.	AK102285	Q00RH9	Protein kinase, catalytic domain
Os04g0531600	Hypothetical protein.	AK107678	longestORF	-
Os04g0531750	Similar to OSIGBa0125M19.13 protein.	ab initio prediction	Q00RH3	Short-chain dehydrogenase/reductase SDR
Os04g0531800	Conserved hypothetical protein.	AK121355	B8ASD8	-
Os04g0532100	Short-chain dehydrogenase/reductase SDR domain containing protein.	AK109281	Q00RH5	Short-chain dehydrogenase/reductase SDR
Os04g0532400	Similar to OSIGBa0125M19.13 protein.	AK318556	Q00RH3	Short-chain dehydrogenase/reductase SDR
Os04g0532500	Similar to Transcription factor L2.	AK069907	Q00RH2	Zinc finger, LIM-type
Os04g0533200	Similar to Myb7 protein (Fragment).	AK376409	Q43598	-
Os04g0533250	Hypothetical gene.	EU971393	longestORF	-
Os04g0533500	Cytochrome b561 family protein.	AK061426 ,AK069219	Q01II4	-
Os04g0533800	Similar to OSIGBa0159I10.4 protein.	BT040291	Q01II1	Protease inhibitor I4, serpin
Os04g0534166	Similar to OSIGBa0159I10.8 protein.	ab initio prediction	Q01IH7	Leucine-rich repeat-containing N-terminal, type 2
Os04g0534200	Protein kinase, core domain containing protein.	AK109583	Q01IH7	Protein kinase, catalytic domain
Os04g0534500	Similar to OSIGBa0159I10.11 protein.	AK105805	Q01IH4	-
Os04g0534600	Peroxisomal biogenesis factor 11 family protein.	AK073835 ,AK287635	Q7XU74	Peroxisomal biogenesis factor 11
Os04g0535200	Peptidase A1 domain containing protein.	AK101505 ,AK060585 ,AK11976	Q01IH2	-
Os04g0535600	Similar to Beta-fructofuranosidase 1 precursor (EC 3.2.1.26) (Sucrose 1) (Invertase 1).	AK099312 ,AK065130	Q01IH0	Glycoside hydrolase, family 32
Os04g0536300	Transcription factor with zinc finger domain and helix-loop-helix domain (YABBY domain), Leaf de	AK070205 ,AB274017	B7EHI2,Q0JBF0	-
Os04g0536500	Similar to H0502G05.5 protein.	AK109552	Q01JG1	No apical meristem
Os04g0537100	Similar to Auxin-induced protein X15.	AK108118	Q01JF8	Auxin responsive SAUR protein
Os04g0537450	Non-protein coding transcript.	CT835085	NONE	-
Os04g0538166	Similar to Elongation factor G, chloroplastic.	P35450	P35450	Protein synthesis factor, GTP-binding
Os04g0538300	Hypothetical conserved gene.	ab initio prediction	NP_001174027.1	-
Os04g0538400	Similar to Nodulin 21 (N-21).	AK108230	Q9M2C3	Domain of unknown function DUF125, transmembrane
Os04g0538700	Autophagy-related protein 13 domain containing protein.	AK105628	Q7XU21	Autophagy-related protein 13
Os04g0538900	Glyoxalase/bleomycin resistance protein/dioxygenase domain containing protein.	AK333604	NP_001053434.1	-
Os04g0539000	Protein of unknown function DUF2485 domain containing protein.	AK105343	Q7FB12	NAD
Os04g0539100	Conserved hypothetical protein.	AK069820	A2XVZ2	-
Os04g0539300	Similar to Aldehyde 5-hydroxylase.	AK248286	Q9SWR1	Cytochrome P450
Os04g0539500	Similar to H0115B09.1 protein.	AK060574 ,AK099144	Q01IQ1	Zinc finger, GATA-type
Os04g0539601	Hypothetical gene.	EU947136	longestORF	-
Os04g0539701	Conserved hypothetical protein.	CT835025	NP_001060202.1	-
Os04g0539800	Src homology-3 domain containing protein.	AK240927	Q01IQ0	Src homology-3 domain
Os04g0540200	Zinc finger, B-box domain containing protein.	AK061333	Q01IP9	Zinc finger, B-box
Os04g0540300	COBRA-like protein 7.	Q7XR91	Q7XR91	Glycosyl-phosphatidyl inositol-anchored, plant

Os04g0540401	Hypothetical protein.	tplb0046p06 (Wheat FLcDNA)	longestORF	-
Os04g0540900	Serine/threonine protein kinase domain containing protein.	AK066397	Q01IP5	Protein kinase, catalytic domain
Os04g0542100	Similar to HAT family dimerisation domain containing protein.	AK288170	Q53RM1	HAT dimerisation
Os04g0586200	Similar to H0307D04.13 protein.	AK120315	Q01K53	Protein of unknown function DUF581
Os04g0590283	Hypothetical protein.	BT019283	longestORF	-
Os04g0590650	Hypothetical gene.	EU948983	longestORF	-
Os04g0613500	Similar to 40S ribosomal protein S11.	D29727	NP_001105562.1	Ribosomal protein S17
Os04g0616650	Non-protein coding transcript.	EU949024	NONE	-
Os04g0638700	Conserved hypothetical protein.	AK062886	B8AV68	-
Os04g0639100	Conserved hypothetical protein.	AK063036	Q7XPH6	-
Os04g0639300	Conserved hypothetical protein.	AK058483	Q7XPH4	-
Os04g0639800	Similar to OSIGBa0138H21-OSIGBa0138E01.9 protein.	AK243114	Q01HX4	-
Os04g0640600	Shikimate kinase.	AK070318	Q01HY3	Shikimate kinase
Os04g0640700	Similar to Alpha-L-arabinofuranosidase/beta-D-xylosidase isoenzyme ARA-I.	AK101782	Q01HY2	Glycoside hydrolase, family 3, N-terminal
Os04g0640750	Hypothetical protein.	tplb0030f15 (Wheat FLcDNA)	longestORF	-
Os04g0664400	Auxin response factor 1.	AK103452	Q8S983	AUX/IAA protein
Os04g0665900	Similar to H1005F08.13 protein.	FP097147	Q01MN1	VQ
Os04g0667033	Non-protein coding transcript.	BT018595	NONE	-
Os04g0668000	Conserved hypothetical protein.	AK062740	Q7XR77	-
Os04g0668700	Similar to phosphatidylinositol 3- and 4-kinase family protein.	AK062068	NP_001151804.1	Phosphatidylinositol 3-/4-kinase, catalytic
Os04g0668800	Putative thiol-disulphide oxidoreductase DCC family protein.	AK288046	Q8W437	Putative thiol-disulphide oxidoreductase DCC
Os04g0668900	Similar to Plastid terminal oxidase.	AK067891	B8AW16	Alternative oxidase
Os04g0669300	EF hand domain containing protein.	AK071148	NP_001030731.1	EPS15 homology
Os04g0669375	Non-protein coding transcript.	tplb0032i10 (Wheat FLcDNA)	NONE	-
Os04g0669600	Phospholipase/carboxylesterase domain containing protein.	AK110767	Q259P1	Phospholipase/carboxylesterase/thioesterase
Os04g0670000	Reticulon family protein.	AK071792	Q259P4	Reticulon
Os04g0670200	Granulin domain containing protein.	AK099358	GeneMark	Granulin
Os04g0670900	Homeodomain-like containing protein.	DQ383374	Q258Z1	MYB-like
Os04g0671100	Similar to H0624F09.8 protein.	AK241125	Q258Z0	Adenylate kinase
Os04g0671300	Similar to Suppressor of presenilin 5 (P110b homolog).	AK072414	Q258Y8	Flavin amine oxidase
Os04g0671500	Hypothetical protein.	tplb0048c15 (Wheat FLcDNA)	longestORF	-
Os04g0691600	Similar to 30S ribosomal protein S17.	AK059422	Q9ZST1	Ribosomal protein S17
Os05g0102300	HAT dimerisation domain containing protein.	AK106387	longestORF	HAT dimerisation
Os05g0119700	Conserved hypothetical protein.	AK062482	Q9LIW7	-
Os05g0119850	Hypothetical protein.	tplb0022d09 (Wheat FLcDNA)	longestORF	-
Os05g0120300	Conserved hypothetical protein.	AK109108	Q9LIW0	-
Os05g0127266	Non-protein coding transcript.	AK248585	NONE	-
Os05g0127400	Similar to DnaJ subfamily B member 13.	AK069823	B6TKS5	Heat shock protein DnaJ, N-terminal
Os05g0127450	Non-protein coding transcript.	tplb0026i01 (Wheat FLcDNA)	NONE	-
Os05g0129700	KNOX class homeodomain protein (Knotted1-type homeobox protein OSH71).	AK111878	Q7GDL5	Homeobox
Os05g0129800	DNA-binding WRKY domain containing protein.	AK241799	NP_001174199.1	DNA-binding WRKY
Os05g0129900	Tetratricopeptide-like helical domain containing protein.	AK060436	B9FMA1	Tetratricopeptide TPR-1
Os05g0138300	Similar to ICT protein (Fragment).	AK119249	Q9SXZ4	-
Os05g0142500	Hypothetical conserved gene.	ab initio prediction	A2Y0A3	-
Os05g0143000	Conserved hypothetical protein.	AK064113	B8AXW7	-
Os05g0143300	Conserved hypothetical protein.	AK072841 ,AK099030	B9FH60	-
Os05g0143500	Similar to Kinase (Fragment).	AK061851	C4PE55	-
Os05g0143600	Similar to Jasmonate-induced protein.	AK107844	A2Y0B0	Mannose-binding lectin
Os05g0144100	WD40 repeat-like domain containing protein.	AK069280	B6U179	WD40 repeat
Os05g0144150	Hypothetical gene.	BT084538	longestORF	-
Os05g0144200	Similar to glioma tumor suppressor-like protein.	AK242272	B6T3I5	Tumour suppressor protein Gltscr2
Os05g0145400	Fibronectin, type III-like fold domain containing protein.	AK106860	A0SQ42	Fibronectin, type III
Os05g0145500	Hypothetical protein.	tplb0048h04 (Wheat FLcDNA)	longestORF	-
Os05g0145700	Peptidase, trypsin-like serine and cysteine domain containing protein.	AK069656	B9FH68	Peptidase cysteine/serine, trypsin-like
Os05g0149300	Similar to 1-aminocyclopropane-1-carboxylate oxidase.	BT087584	O81606	Oxoglutarate/iron-dependent oxygenase
Os05g0149400	Similar to 1-aminocyclopropane-1-carboxylic acid oxidase.	AK061064	O81606	Oxoglutarate/iron-dependent oxygenase
Os05g0149450	Non-protein coding transcript.	tplb0008n13 (Wheat FLcDNA)	NONE	-
Os05g0149600	Similar to CULLIN1-like protein 1.	AK073947	C7SJ62	-
Os05g0149950	Monothiol glutaredoxin-S8.	P0C290	P0C290	Glutaredoxin
Os05g0150550	Hypothetical protein.	tplb0032b19 (Wheat FLcDNA)	longestORF	-
Os05g0150600	Hypothetical conserved gene.	AK063338	D7U9T8	DNA helicase, ATP-dependent, RecQ type
Os05g0150900	Histidyl-tRNA synthetase (EC 6.1.1.21) (Histidine--tRNA ligase) (HisRS).	AK099789	P93422	Phenylalanine/histidine ammonia-lyase
Os05g0151000	Similar to DNA-directed RNA polymerase.	AK371102	C5YGY9	RNA polymerase II, heptapeptide repeat, eukaryotic
Os05g0151100	Similar to desiccation-related protein PCC13-62.	EU967146	B6TNA6	-
Os05g0151150	Hypothetical gene.	EU975566	GeneMark	-
Os05g0151200	TspO/MBR-related protein family protein.	AK240936	NP_001147341.1	TspO/MBR-related protein
Os05g0151300	Rubber elongation factor family protein.	AK243548	A2Y0H2	Rubber elongation factor
Os05g0151400	AIG1 domain containing protein.	AK102924	XP_002874910.1	Chloroplast protein import component Toc86/159
Os05g0152266	Hypothetical gene.	EU943470	longestORF	-
Os05g0159400	Non-protein coding transcript.	EU942788	NONE	-
Os05g0160000	Similar to Ferric leghemoglobin reductase.	AK063865	B9FML1	Pyridine nucleotide-disulphide oxidoreductase, dimerisation
Os05g0160200	Ubiquitin.	AK119731	A6MZJ4	Ubiquitin
Os05g0173700	Similar to DNA replication licensing factor MCM3 homolog (Replication origin activator) (ROA pro	AK063470	NP_001105718.1	Mini-chromosome maintenance, DNA-dependent ATPase
Os05g0176100	Similar to Cellulose synthase BoCesA1.	AK067850	D5FJ45	Cellulose synthase
Os05g0176700	Conserved hypothetical protein.	AK071729	A2Y0X6	-
Os05g0177100	F-box domain, cyclin-like domain containing protein.	AK064652	B9FMR7	F-box domain, cyclin-like
Os05g0178950	Hypothetical protein.	tplb0040d24 (Wheat FLcDNA)	longestORF	-
Os05g0179050	Hypothetical protein.	tplb0030e06 (Wheat FLcDNA)	longestORF	-
Os05g0182600	Similar to SSRP1 protein.	AK102592	Q65WY8	High mobility group, HMG1/HMG2
Os05g0182650	Conserved hypothetical protein.	AK240762	NP_001174249.1	-
Os05g0182700	Protein of unknown function DUF1664 family protein.	AK065911 ,AK104343	A2Y139	Protein of unknown function DUF1664
Os05g0182800	Similar to glutaminyl-tRNA synthetase.	AK061951	NP_001152305.1	Glutamyl/glutaminyl-tRNA synthetase, class Ib
Os05g0183100	Similar to WRKY transcription factor 16 (Fragment).	AK066252	B2KJ59	DNA-binding WRKY
Os05g0183200	Similar to barley mlo defense gene homolog6.	ab initio prediction	NP_001105170.1	Mlo-related protein
Os05g0183300	Similar to barley mlo defense gene homolog6.	ab initio prediction	NP_001105170.1	Mlo-related protein
Os05g0183900	Similar to transposon protein CACTA, En/Spm sub-class.	AK360948	Q65WX7	Putative harbinger transposase-derived nuclease
Os05g0184016	Hypothetical protein.	AK372466	longestORF	-
Os05g0184901	Phospholipase A2, active site domain containing protein.	AK287961	B6SGX9	-
Os05g0185650	Similar to predicted protein.	AK242365	XP_002867220.1	-
Os05g0185700	Similar to predicted protein.	AK064724	NP_200842.1	MaoC-like dehydratase
Os05g0185767	Hypothetical gene.	AK288224	longestORF	-
Os05g0185800	Conserved hypothetical protein.	AK065050	A2Y150	-
Os05g0186000	Similar to PSRP4.	AK058675	B6T2D1	-
Os05g0186601	Hypothetical gene.	EU969101	longestORF	-
Os05g0186900	Similar to Auxin-responsive protein IAA16.	AK111403	P0C127	-
Os05g0187000	Beta 2 subunit of 20S proteasome (20S proteasome beta subunit).	AK103126	A2Y157	Peptidase T1A, proteasome beta-subunit
Os05g0187100	Similar to Hexokinase.	AK067988	Q1WM16	Hexokinase
Os05g0206000	Conserved hypothetical protein.	AK107740	A2Y1G1	-
Os05g0206100	Conserved hypothetical protein.	CT836392	NP_001174274.1	-
Os05g0207500	Similar to Shaggy-related protein kinase eta (EC 2.7.1.-) (ASK-eta) (BRASSINOSTEROID-INSENS	AK102147	NP_001149135.1	Protein kinase, catalytic domain
Os05g0207600	Hypothetical protein.	tplb0034j24 (Wheat FLcDNA)	longestORF	-
Os05g0207700	Similar to Serine/threonine-protein kinase PBS1 (EC 2.7.1.37) (AvrPphB susceptible protein 1).	AK102665	B4FFH0	Protein kinase, catalytic domain
Os05g0208000	Similar to Mitochondrial 2-oxoglutarate/malate carrier protein.	AK103990	NP_001182793.1	Mitochondrial substrate/solute carrier
Os05g0208050	Hypothetical gene.	BT016874	longestORF	-
Os05g0208100	Similar to CBL-interacting serine/threonine-protein kinase 15 (EC 2.7.1.37) (Serine/threonine-prote	AK107068	Q60EY8	Protein kinase, catalytic domain
Os05g0217950	Non-protein coding transcript.	tplb0043a14 (Wheat FLcDNA)	NONE	-
Os05g0218001	Myb/SANT-like domain domain containing protein.	BT039927	NP_001176912.1	Myb/SANT-like domain
Os05g0231600	Similar to Aquaglyceroporin (Tonoplast intrinsic protein (Tipa)).	AK119476	longestORF	-
Os05g0247900	DEAD-like helicase, N-terminal domain containing protein.	AK071717	XP_002328055.1	SNF2-related
Os05g0248200	Glycoside hydrolase, family 18 protein.	AK241114	Q53NL5	Glycoside hydrolase, family 18, catalytic domain
Os05g0249800	Domain of unknown function DUF1618 domain containing protein.	tplb0019i13 (Wheat FLcDNA)	NP_001174299.1	Domain of unknown function DUF1618
Os05g0250000	Domain of unknown function DUF1618 domain containing protein.	AK377023	B9FJN4	Domain of unknown function DUF1618
Os05g0250200	Hypothetical conserved gene.	AK330366	B8B052	Domain of unknown function DUF1618
Os05g0250500	Similar to selT/selW/selH selenoprotein domain containing protein.	ab initio prediction	NP_001149449.1	Chloramphenicol acetyltransferase-like domain
Os05g0251200	Domain of unknown function DUF1618 domain containing protein.	ab initio prediction	NP_001174301.1	Domain of unknown function DUF1618

Os05g0251400	Conserved hypothetical protein.	AK103883	A2Y2F6	-
Os05g0251500	Similar to Similarities with spP29295 Saccharomyces cerevisiae YPL204w HRR25 casein kinase I.	AK058649	B6SPS1	Protein of unknown function DUF1242
Os05g0251700	Hypothetical protein.	tplb0036f21 (Wheat FLcDNA)	longestORF	-
Os05g0251900	Hypothetical conserved gene.	AB190913	Q6AVD0-2	Oligopeptide transporter OPT superfamily
Os05g0252000	Oligopeptide transporter OPT superfamily protein.	AK068865	B9FNH8	Oligopeptide transporter OPT superfamily
Os05g0252050	Hypothetical protein.	tplb0036f21 (Wheat FLcDNA)	longestORF	-
Os05g0252100	Protein of unknown function DUF620 family protein.	AK288714	NP_001055033.1	Protein of unknown function DUF620
Os05g0252250	Hypothetical protein.	BT085554	longestORF	-
Os05g0252701	Non-protein coding transcript.	CT836375	NONE	-
Os05g0252801	Transposase, IS4-like domain containing protein.	BT037367	Q8S819	Transposase, IS4-like
Os05g0253100	Conserved hypothetical protein.	AK288186	Q8LM57	-
Os05g0253301	Non-protein coding transcript.	EU949024	NONE	-
Os05g0255600	Thioredoxin domain 2 containing protein.	AK073067	NP_190665.2	Thioredoxin-like fold
Os05g0255800	Domain of unknown function DUF632 domain containing protein.	AK063562	Q65XR1	Domain of unknown function DUF632
Os05g0256350	Similar to HAT family dimerisation domain containing protein.	AK122139	Q2QPA8	Zinc finger, BED-type predicted
Os05g0258501	Non-protein coding transcript.	EU949024	NONE	-
Os05g0259601	Non-protein coding transcript.	EU949024	NONE	-
Os05g0261001	Non-protein coding transcript.	EU949024	NONE	-
Os05g0267100	Hypothetical conserved gene.	AK065811	Q6ATF1	-
Os05g0267800	Cellular retinaldehyde-binding/triple function, C-terminal domain containing protein.	AK061207	B6TF16	Cellular retinaldehyde-binding/triple function, C-terminal
Os05g0268400	Similar to Major facilitator superfamily antiporter.	AK288425	Q8H6D6	-
Os05g0268500	Similar to Serine carboxypeptidase 2.	AK119979	P08819	Peptidase S10, serine carboxypeptidase
Os05g0269100	Cyclin-like F-box domain containing protein.	AK066309	A2Y2K4	F-box domain, cyclin-like
Os05g0269200	Similar to inner membrane transport protein.	CT836417	NP_001147344.1	C4-dicarboxylate transporter/malic acid transport protein
Os05g0270000	Ribosomal protein L25 family protein.	AK072793	NP_194093.1	Ribosomal protein L25/Gln-tRNA synthetase, anti-codon-binding domain
Os05g0270400	Protein phosphatase inhibitor family protein.	AK121231	A2Q5P6	Protein phosphatase inhibitor
Os05g0271300	Hypothetical conserved gene.	Q6ATG6	Q6ATG6	Ribonuclease III
Os05g0272300	NmrA-like domain containing protein.	ab initio prediction	NP_001041775.1	NmrA-like
Os05g0272400	Hypothetical protein.	AK110636	longestORF	-
Os05g0272800	Similar to F20D23.27 protein.	AK068945	Q9SHG2	-
Os05g0272900	B-cell receptor-associated 31-like family protein.	AK107093	B6TG43	B-cell receptor-associated 31-like
Os05g0274300	Similar to ATP binding protein.	AK071399	NP_001150449.1	-
Os05g0274950	Similar to homogentisate phytyltransferase VTE2-1.	HM212530	NP_001105877.1	-
Os05g0275000	Pentatricopeptide repeat domain containing protein.	AK067135	NP_001157212.1	Pentatricopeptide repeat
Os05g0275100	Pentatricopeptide repeat domain containing protein.	ab initio prediction	B9FNL7	Pentatricopeptide repeat
Os05g0275600	Conserved hypothetical protein.	AK108098	XP_002464690.1	-
Os05g0275700	Similar to Peroxisome assembly protein 2 (Peroxin-2) (AthPEX2) (Pex2p).	AK111642	B4FCL8	Zinc finger, RING-type
Os05g0276100	Similar to Na+/H+ exchangeing protein-like (Cation/H+ exchanger).	AK069092	XP_002314350.1	Cation/H+ exchanger
Os05g0276500	Expansin Os-EXPA3.	AK060313	Q40637	Expansin
Os05g0276750	Hypothetical protein.	BT016845	longestORF	-
Os05g0277000	Similar to Expansin Os-EXPA3.	P0C1Y4	P0C1Y4	Expansin
Os05g0277100	Hypothetical protein.	BT016845	longestORF	-
Os05g0277200	Conserved hypothetical protein.	AK110612	A2Y2P1	-
Os05g0277300	Similar to Peptide chain release factor 1.	AK065607	B6U6C9	-
Os05g0277500	Similar to Germin-like protein subfamily 2 member 4 precursor.	AK105351	NP_001151047.1	Germin
Os05g0278150	Non-protein coding transcript.	EU949846	NONE	-
Os05g0278500	Transferase family protein.	AK067851	B6TZX2	Transferase
Os05g0278550	Hypothetical gene.	EU944389	longestORF	-
Os05g0278950	Similar to ATP-dependent Clp protease proteolytic subunit.	BT033932	B8AXJ0	-
Os05g0279850	Hypothetical protein.	tplb0028j17 (Wheat FLcDNA)	longestORF	-
Os05g0279900	Similar to glycoside hydrolase family 28 protein / polygalacturonase (pectinase) family protein.	ab initio prediction	NP_175244.1	Glycoside hydrolase, family 28
Os05g0280200	Similar to Ras-related protein RGP2.	AK120316	A2Y2Q3	Small GTPase superfamily
Os05g0280350	Hypothetical gene.	BT084300	longestORF	-
Os05g0283200	Pectinesterase inhibitor domain containing protein.	AK120717	A2Y2R2	Pectinesterase inhibitor
Os05g0286100	Similar to Zinc-finger protein KNUCKLES.	AK108829	A2Y2R5	Zinc finger, C2H2
Os05g0287800	Similar to Phosphatidic acid phosphatase-like protein.	AK071727	E5FY32	-
Os05g0289400	Similar to CRN (Crooked neck) protein.	AK100478	Q9FJ37	RNA-processing protein, HAT helix
Os05g0289700	Arbuscular mycorrhizal specific marker 10.,Benzyl alcohol benzoyl transferase.	BT086008	Q01IV4	Transferase
Os05g0290000	Hypothetical protein.	BT035701	longestORF	-
Os05g0291600	Hypothetical conserved gene.	AK073226	NP_001059231.1	-
Os05g0291700	Similar to PTAC16 (PLASTID TRANSCRIPTIONALLY ACTIVE18)%3B binding / catalytic.	AK108453	XP_002875803.1	NmrA-like
Os05g0292800	Similar to One helix protein (OHP).	AK059562	Q8LPV2	Chlorophyll a/b binding protein domain
Os05g0294800	Hypothetical gene.	AK065926	longestORF	-
Os05g0295200	Conserved hypothetical protein.	AK073450	B9FNQ1	-
Os05g0295300	Similar to Acetyl-coenzyme A carboxylase (EC 6.4.1.2).	AK059261	O48959	Carboxyl transferase
Os05g0295500	Non-protein coding transcript.	BT019212	NONE	-
Os05g0295800	Similar to Glyoxalase I (EC 4.4.1.5).	AK070232	NP_001146873.1	Glyoxalase/fosfomycin resistance/dioxygenase
Os05g0295900	Conserved hypothetical protein.	AK069962	B9FNQ4	-
Os05g0296530	Non-protein coding transcript.	AK356614	NONE	-
Os05g0296600	Non-protein coding transcript.	AK059372	NONE	-
Os05g0296750	Hypothetical gene.	AK063686	longestORF	-
Os05g0296900	Hypothetical conserved gene.	AK100725	B8AWA5	-
Os05g0297900	Similar to Signal peptidase 18 subunit (Fragment).	AK071238	B6T545	Peptidase S26B, eukaryotic signal peptidase
Os05g0298200	Ankyrin repeat containing protein.	AK066758	A2Y2V7	Ankyrin repeat
Os05g0298300	Hypothetical protein.	tplb0059e11 (Wheat FLcDNA)	longestORF	-
Os05g0299000	Conserved hypothetical protein.	AK108720	B8AWB0	-
Os05g0299200	Hypothetical conserved gene.	EU956990	NP_001055128.1	Nucleotide-sugar transporter
Os05g0299250	Hypothetical protein.	tplb0062m11 (Wheat FLcDNA)	longestORF	-
Os05g0299300	Conserved hypothetical protein.	AK111812	B9FNQ8	-
Os05g0300700	Cell division cycle-associated protein domain containing protein.	AK067846	B9FK50	Zinc-finger domain of monoamine-oxidase A repressor R1
Os05g0301500	Similar to Ribophorin I (Fragment).	AK070329	NP_001151620.1	Ribophorin I
Os05g0302916	Hypothetical protein.	tplb0044f08 (Wheat FLcDNA)	longestORF	-
Os05g0303000	Similar to Chloroplast heat shock protein 70.	AK060410	A4ZYQ0	Heat shock protein Hsp70
Os05g0303700	Hypothetical conserved gene.	ab initio prediction	A2Y2Y7	RNA recognition motif domain
Os05g0304501	Hypothetical gene.	CT833082	longestORF	-
Os05g0304701	Hypothetical gene.	EU945269	longestORF	-
Os05g0311075	Non-protein coding transcript.	EU943375	NONE	-
Os05g0324100	N2,N2-dimethylguanosine tRNA methyltransferase family protein.	AK059071	NP_191192.1	N2,N2-dimethylguanosine tRNA methyltransferase
Os05g0328901	Similar to Prolamin.	AK242260	Q5EFA4	-
Os05g0329001	Similar to Prolamin.	AF042201	Q5EFA4	-
Os05g0329100	Prolamin.	AK242260	Q5EFA4	Bifunctional trypsin/alpha-amylase inhibitor
Os05g0329200	Similar to Prolamin.	AK242260	Q5EFA4	Bifunctional trypsin/alpha-amylase inhibitor
Os05g0329350	Prolamin.	EF122440	Q5EFA4	Bifunctional trypsin/alpha-amylase inhibitor
Os05g0329700	Similar to Prolamin.	AK242910	Q5EFA4	Bifunctional trypsin/alpha-amylase inhibitor
Os05g0330600	Similar to Prolamin.	FJ940201	Q5EFA4	Bifunctional trypsin/alpha-amylase inhibitor
Os05g0355800	Similar to Lipoxygenase.	DQ658739	Q01J86	Lipoxygenase
Os05g0356500	Hypothetical conserved gene.	AB332071	B8AX35	-
Os05g0356600	Hypothetical conserved gene.	AB332072	A2Y3M6	-
Os05g0356700	Protein of unknown function DUF231, plant domain containing protein.	AK071031	XP_002884829.1	Domain of unknown function DUF231, plant
Os05g0356800	Protein of unknown function DUF3511 domain containing protein.	AK121901	A2Y3M8	Protein of unknown function DUF3511
Os05g0363533	Non-protein coding transcript.	EU948047	NONE	-
Os05g0363566	Hypothetical conserved gene.	CT836447	Q688I1	-
Os05g0375532	Hypothetical gene.	AK287793	longestORF	-
Os05g0375600	Similar to Peptide chain release factor 2.	AK376656	B6SSD2	-
Os05g0375700	Hypothetical conserved gene.	BT054511	Q6AUQ1	Peptidase A1
Os05g0375850	Hypothetical protein.	AK360759	longestORF	-
Os05g0377450	Similar to H0315A08.1 protein.	FP099421	Q01I63	Ribonuclease H1, N-terminal
Os05g0377600	Non-protein coding transcript.	EU941912	NONE	-
Os05g0377750	Non-protein coding transcript.	EU941912	NONE	-
Os05g0391400	Non-protein coding transcript.	EU941912	NONE	-
Os05g0392300	Similar to Cyclin-dependent kinase D-1.	AK062401	P29620	-
Os05g0399900	Conserved hypothetical protein.	EU976876	B9FVF5	-
Os05g0408800	Similar to zinc finger, C3HC4 type family protein.	EU964917	NP_001149860.1	Zinc finger, RING-type
Os05g0408850	Hypothetical gene.	CT836350	longestORF	-
Os05g0409500	Similar to MtN21 protein.	BT084636	NP_001150619.1	Drug/metabolite transporter

Os05g0410750	Hypothetical protein.	tplb0038k08 (Wheat FLcDNA)	longestORF	-
Os05g0410800	TGF-beta receptor, type I/II extracellular region family protein.	AK108312	NP_001147810.1	Oligopeptide transporter
Os05g0411150	Hypothetical protein.	tplb0038k08 (Wheat FLcDNA)	longestORF	-
Os05g0411300	Similar to DNA binding protein.	AK073142	NP_001148077.1	Basic-leucine zipper
Os05g0413575	Hypothetical gene.	BT016337	longestORF	-
Os05g0419500	K Homology domain containing protein.	AK073986	NP_001148920.1	K Homology
Os05g0473500	Exo70 exocyst complex subunit family protein.	AK101630	B6U0J9	Exo70 exocyst complex subunit
Os05g0476250	Non-protein coding transcript.	EU951628	NONE	-
Os05g0476466	Similar to CBL-interacting protein kinase 28.	A3B529	A3B529	Protein kinase, catalytic domain
Os05g0476583	Non-protein coding transcript.	tplb0042a18 (Wheat FLcDNA)	NONE	-
Os05g0477666	Hypothetical gene.	BT016845	longestORF	-
Os05g0478250	Hypothetical protein.	AK369221	longestORF	-
Os05g0478300	Hypothetical conserved gene.	AK242838	NP_001055847.2	Protein kinase, catalytic domain
Os05g0481500	K Homology domain containing protein.	AK072560	NP_001150991.1	K Homology
Os05g0486833	Hypothetical conserved gene.	BT065526	C0HHH7	-
Os05g0487300	Conserved hypothetical protein.	AK062926	B8AZG8	-
Os05g0487600	Similar to Vacuolar ATPase B subunit.	AK059850	Q7FV25	-
Os05g0488100	Conserved hypothetical protein.	AK109773	B8AZH0	-
Os05g0489301	Non-protein coding transcript.	CT836470	NONE	-
Os05g0489800	Centromeric histone 3 (Histone H3).	AK101975	Q6T367	Histone H3
Os05g0491800	Hypothetical protein.	tplb0061j19 (Wheat FLcDNA)	longestORF	-
Os05g0494100	Similar to dr1-associated corepressor.	AK060192	A2Y627	-
Os05g0494700	Hypothetical conserved gene.	AK243175	A2Y632	-
Os05g0494800	Hypothetical protein.	AK069939	GeneMark	-
Os05g0494900	SH2 motif domain containing protein.	AK106396	NP_001077775.1	SH2 motif
Os05g0495000	Hypothetical conserved gene.	CT829003	B9FKX7	Zinc finger, RING-type
Os05g0495200	Basic leucine zipper domain containing protein.	AK072281	D6MKB2	Basic-leucine zipper
Os05g0495250	Non-protein coding transcript.	CT836397	NONE	-
Os05g0495700	Similar to Glycerol-3-phosphate dehydrogenase.	AK101484	B6SKP4	Glycerol-3-phosphate dehydrogenase, NAD-dependent, C-terminal
Os05g0496100	Translation initiation factor eIF3 subunit domain containing protein.	ab initio prediction	NP_001055936.1	Translation initiation factor eIF3 subunit
Os05g0496200	Similar to 3-phosphoglycerate kinase (Fragment).	AK287421	P12782	Phosphoglycerate kinase
Os05g0496300	Chalcone/stilbene synthase, N-terminal domain containing protein.	AY286097	A2Y651	Chalcone/stilbene synthase, N-terminal
Os05g0509101	Conserved hypothetical protein.	AK110690	Q6L4X0	-
Os05g0528101	Hypothetical gene.	EU950506	longestORF	-
Os05g0547200	Conserved hypothetical protein.	AK375772	NP_001047613.2	-
Os05g0547700	Conserved hypothetical protein.	AK070926	B9G5C2	-
Os05g0557500	Protein of unknown function DUF2921 domain containing protein.	AK067152	B9FLK1	Protein of unknown function DUF2921
Os05g0557800	Similar to 50S ribosomal protein L21.	AK105327	B6TJJ8	Ribosomal protein L21
Os05g0557950	Hypothetical protein.	EU949682	longestORF	-
Os05g0558101	Conserved hypothetical protein.	EU957461	Q6ZJX2	-
Os05g0558501	Conserved hypothetical protein.	EU957461	Q6ZJX2	-
Os05g0565100	Cytochrome b561/ferric reductase transmembrane domain containing protein.	AK063277	B8AWR7	Cytochrome b561, eukaryote
Os05g0565900	DNA-binding WRKY domain containing protein.	FP101665	Q688X5	DNA-binding WRKY
Os05g0566200	NLI interacting factor domain containing protein.	AK099836	B9FLM9	NLI interacting factor
Os05g0566300	PRC-barrel domain containing protein.	AK099641	B9FI59	RimM protein
Os05g0566500	Similar to Initiation factor 3d (Fragment).	AK073468	B6U4Q2	Eukaryotic translation initiation factor 3, subunit 7
Os05g0566800	Cold acclimation protein COR413-TM1.	AK065748	Q84XU8	Cold acclimation WCOR413
Os05g0567100	Similar to Phytepsin.	AK100749	P42210	Peptidase A1
Os05g0568600	Protein of unknown function DUF1645 family protein.	AK063781	B9FI71	Protein of unknown function DUF1645
Os05g0570350	Protein of unknown function DUF295 domain containing protein.	AK241838	GeneMark	Protein of unknown function DUF295
Os05g0571100	Peptidase C12, ubiquitin carboxyl-terminal hydrolase 1 domain containing protein.	AK068658	NP_001118908.1	Plant organelle RNA recognition domain
Os05g0574600	Similar to 3-ketoacyl-CoA synthase.	AK070754	A2Y7R6	Very-long-chain 3-ketoacyl-CoA synthase
Os05g0595100	Similar to UDP-glucose-4-epimerase.	AK066249	A2Y861	NAD-dependent epimerase/dehydratase
Os05g0595200	Allergen V5/Tpx-1 related family protein.	AK070317	B8AXH6	Allergen V5/Tpx-1-related
Os05g0595800	Serine-threonine/tyrosine-protein kinase domain containing protein.	ab initio prediction	NP_001174552.1	Serine-threonine/tyrosine-protein kinase
Os05g0596000	Peptidase A1 domain containing protein.	AK121242 ,AK068911	NP_001149154.1	Peptidase A1
Os05g0596200	Similar to cDNA clone:001-104-D04, full insert sequence.	AK107887	B7E7K4	-
Os05g0596500	Similar to RING-H2 finger protein ATL3I (YGHL1-C3HC4 RING fusion protein).	AK070817	XP_002440385.1	Zinc finger, RING-type
Os05g0596700	Similar to cDNA clone:J033067M13, full insert sequence.	AK103507	B7EMM6	Peptidyl-tRNA hydrolase
Os05g0597100	Similar to Nucleolar histone deacetylase HD2-p39.	AK072845	NP_001105631.1	Zinc finger, C2H2
Os05g0597200	Similar to AT1 protein.	AK064798	Q40725	High mobility group, HMG-I/HMG-Y
Os05g0597400	Conserved hypothetical protein.	AK375939	NP_001174555.1	-
Os05g0597700	Conserved hypothetical protein.	AK121194	A2Y879	-
Os06g0100550	Conserved hypothetical protein.	EU951430	Q65WW4	-
Os06g0122800	Hypothetical conserved gene.	AK289091	NP_001041816.1	Peptidase C48, SUMO/Sentrin/Ubl1
Os06g0131900	Non-protein coding transcript.	EU941912	NONE	-
Os06g0132000	Similar to H0315A08.1 protein.	FP099421	Q01I63	Ribonuclease H1, N-terminal
Os06g0140200	Leucine-rich repeat, plant specific containing protein.	AK287701 ,AK241776 ,AK28787	Q5VPE8	Leucine-rich repeat
Os06g0159750	Hypothetical conserved gene.	EU964122	Q01I89	-
Os06g0184100	Conserved hypothetical protein.	AK064179	B8B3I7	-
Os06g0188400	Extracellular ligand-binding receptor domain containing protein.	AK065496	B9FRX3	Extracellular ligand-binding receptor
Os06g0188600	Hypothetical conserved gene.	AK242845	Q69KL2	Ionotropic glutamate receptor
Os06g0189600	Protein of unknown function DUF1677, Oryza sativa family protein.	AK109331	B8B3M7	Protein of unknown function DUF1677, Oryza sativa
Os06g0190950	Similar to H0418A01.5 protein.	BT061604	Q01J63	Putative harbinger transposase-derived nuclease
Os06g0198100	Similar to H0315A08.1 protein.	FP099421	Q01I63	Ribonuclease H1, N-terminal
Os06g0198500	RmlC-like jelly roll fold domain containing protein.	EU945799	Q9LMB4	Protein of unknown function DUF985
Os06g0198600	Protein of unknown function DUF985 domain containing protein.	AK070593	Q69K63	Protein of unknown function DUF985
Os06g0198800	Similar to 25.3 kDa vesicle transport protein.	AK064781	B6TMY9	Longin
Os06g0199000	Similar to Glycine-rich cell wall structural protein 2 precursor.	AK106381	A2YAD1	-
Os06g0199050	Similar to predicted protein.	EU948644	XP_001779700.1	-
Os06g0200500	Hypothetical conserved gene.	ab initio prediction	Q69K48	-
Os06g0201000	Conserved hypothetical protein.	AK108188	A2YAE2	-
Os06g0202100	Conserved hypothetical protein.	AK106373	B8B3R6	-
Os06g0202400	Conserved hypothetical protein.	AK062755	A2YAE9	-
Os06g0202800	Conserved hypothetical protein.	AK119480	B9FS12	-
Os06g0202825	Non-protein coding transcript.	tplb0055c23 (Wheat FLcDNA)	NONE	-
Os06g0203550	Non-protein coding transcript.	AK288217	NONE	-
Os06g0204250	Hypothetical protein.	EU955821	longestORF	-
Os06g0204400	Similar to ECPT-type aminoalcoholphosphotransferase.	AK067531	C7G530	-
Os06g0204600	Conserved hypothetical protein.	AK354215	XP_002459790.1	-
Os06g0204700	Hypothetical protein.	tplb0041l09 (Wheat FLcDNA)	longestORF	-
Os06g0205100	Similar to Transcriptional activator.	AK062487	GeneMark	-
Os06g0205600	Similar to mechanosensitive ion channel domain-containing protein.	AK357076	XP_002873549.1	Mechanosensitive ion channel MscS
Os06g0206900	Conserved hypothetical protein.	AK071212	A2YAH7	-
Os06g0207500	Hypothetical conserved gene.	AK067995	Q69T67	-
Os06g0207600	Hypothetical conserved gene.	AK332154	Q69T66	WRC
Os06g0207783	Hypothetical protein.	tplb0025a09 (Wheat FLcDNA)	longestORF	-
Os06g0207866	Conserved hypothetical protein.	BT085445	B9FAZ8	-
Os06g0207950	Hypothetical gene.	CT836248	longestORF	-
Os06g0208100	Similar to Homeodomain protein HOX3.	AB101651	Q69T58	Helix-turn-helix motif, lambda-like repressor
Os06g0208151	Similar to quinone oxidoreductase.	BT040177	B8AEF1	GroES-like
Os06g0208200	Conserved hypothetical protein.	AK242462	NP_001174659.1	-
Os06g0208300	KOW domain containing protein.	AK059092	A6N0S0	KOW
Os06g0208700	Similar to Dual-specificity phosphatase protein.	AK065860	Q6DL00	Protein-tyrosine phosphatase, SIW14-like
Os06g0208800	Similar to LysM-domain GPI-anchored protein.	AK062542	Q4VVD8	-
Os06g0208951	Conserved hypothetical protein.	EU943155	NP_001057106.1	-
Os06g0209100	Peptidase A1 domain containing protein.	ab initio prediction	NP_001057106.1	Peptidase A1
Os06g0209300	Conserved hypothetical protein.	AK065500	Q69T47	-
Os06g0209400	Conserved hypothetical protein.	AK376937	NP_001057108.2	-
Os06g0212600	Xyloglucan fucosyltransferase family protein.	tplb0053e16 (Wheat FLcDNA)	NP_001057125.1	Xyloglucan fucosyltransferase
Os06g0214850	Similar to gibberellin receptor GID1L2.	tplb0013o20 (Wheat FLcDNA)	NP_001150234.1	Alpha/beta hydrolase fold-3
Os06g0214900	FAD-dependent pyridine nucleotide-disulphide oxidoreductase domain containing protein.	AK064752	B6T7Z1	FAD-dependent pyridine nucleotide-disulphide oxidoreductase
Os06g0215200	Zinc finger, U1-C type domain containing protein.	AK065717	NP_001151811.1	Zinc finger, C2H2-type matrin
Os06g0215600	Similar to Oxo-phytodienoic acid reductase.	AK061212 ,AK104039 ,AK10419	A8DA33	-
Os06g0215750	Hypothetical gene.	EU947281	longestORF	-

Os06g0215950	Non-protein coding transcript.	EU947281	NONE	-
Os06g0216200	Similar to Oxo-phytodienoic acid reductase.	ab initio prediction	Q8H9F1	NADH:flavin oxidoreductase/NADH oxidase, N-terminal
Os06g0216250	Non-protein coding transcript.	EU947281	NONE	-
Os06g0216400	Tetratricopeptide-like helical domain containing protein.	AK101267	B8B3Z8	Pentatricopeptide repeat
Os06g0216700	Cupredoxin domain containing protein.	AK058410 ,AK101397	B6TG98	Plastocyanin-like
Os06g0216800	Similar to Cyclophilin-40 (Expressed protein).	AK068112	NP_565381.1	Tetratricopeptide TPR-1
Os06g0217600	Similar to kinesin motor protein-related.	AK071349	NP_201349.2	-
Os06g0218000	Similar to pre-mRNA-splicing factor prp45.	tplb0006k10 (Wheat FLcDNA)	Q84UD2	SKI-interacting protein SKIP, SNW domain
Os06g0226950	Fatty acid hydroxylase domain containing protein.	AK241878	B6TLG7	Fatty acid hydroxylase
Os06g0231100	Pentatricopeptide repeat domain containing protein.	BT054912	Q67X39	Pentatricopeptide repeat
Os06g0231300	CS domain domain containing protein.	AK073934	B6T728	CS-like domain
Os06g0231350	Hypothetical protein.	tplb0021n11 (Wheat FLcDNA)	longestORF	-
Os06g0231400	Pentatricopeptide repeat domain containing protein.	AK072084 ,AK120621	NP_001147320.1	Pentatricopeptide repeat
Os06g0239100	Peptidase M10A and M12B, matrixin and adamalysin family protein.	AK287609	NP_001151749.1	Peptidase M10, metallopeptidase
Os06g0240700	Similar to OSIGBa0132O24.3 protein.	EU951279	Q01MB4	-
Os06g0272700	Protein of unknown function DUF231, plant domain containing protein.	BT062756	NP_001174707.1	Domain of unknown function DUF231, plant
Os06g0274100	Armadillo-like helical domain containing protein.	AK242109	Q5VN12	Armadillo-like helical
Os06g0274800	Similar to Peroxidase 11 precursor (EC 1.11.1.7) (Atperox P11) (ATP23a/ATP23b).	AK060295	Q96519	Plant peroxidase
Os06g0274875	Hypothetical protein.	EU942261	longestORF	-
Os06g0275600	Hypothetical conserved gene.	AK101279	F1DKB4	Helix-loop-helix DNA-binding
Os06g0275700	Similar to Beta-catenin repeat family protein.	AK110058	C3SA81	Armadillo
Os06g0276001	Non-protein coding transcript.	AK287734	NONE	-
Os06g0278450	Hypothetical gene.	CT837498	longestORF	-
Os06g0282750	Hypothetical protein.	AK372466	longestORF	-
Os06g0293000	Hypothetical conserved gene.	CT836321	Q5Z570	-
Os06g0293050	Hypothetical protein.	FP101342	longestORF	-
Os06g0309550	Hypothetical gene.	CU406696	longestORF	-
Os06g0317100	Similar to glycine-rich cell wall structural protein.	CT837813	NP_001151377.1	Glycine rich protein
Os06g0318700	Non-protein coding transcript.	AK061908	NONE	-
Os06g0320100	Protein of unknown function DUF773 family protein.	AK101600 ,AK067233	B6T2L5	Protein of unknown function DUF773
Os06g0320900	Conserved hypothetical protein.	ab initio prediction	Q5ZA95	-
Os06g0323550	Similar to H0315A08.1 protein.	FP099421	Q01I63	Ribonuclease H1, N-terminal
Os06g0324800	Major facilitator superfamily protein.	AK109109	E8Z9A7	General substrate transporter
Os06g0326400	Phosphofructokinase domain containing protein.	AK121116 ,AK065052	B6SHF8	Phosphofructokinase domain
Os06g0328900	Cytochrome P450 family protein.	AK110659	C3SAE5	Cytochrome P450
Os06g0328966	Hypothetical protein.	AK357549	longestORF	-
Os06g0329300	Conserved hypothetical protein.	AK067924	A2YCF3	-
Os06g0332500	Conserved hypothetical protein.	AK121262	B8B1C1	-
Os06g0332700	Hypothetical conserved gene.	CT836256	NP_001057547.2	Tesmin/TSO1-like, CXC
Os06g0335500	Similar to Auxin-responsive protein IAA21.	AK063854	Q5Z749	AUX/IAA protein
Os06g0336001	Non-protein coding transcript.	AK289086	NONE	-
Os06g0338801	Hypothetical protein.	BT063833	longestORF	-
Os06g0339500	Similar to OSIGBa0137A06.2 protein.	AK105220	Q01N23	-
Os06g0340001	Hypothetical conserved gene.	ab initio prediction	NP_001174769.1	-
Os06g0340600	Similar to phosphatidylinositol 3- and 4-kinase family protein.	AK065176	NP_001147061.1	Phosphatidylinositol 3-/4-kinase, catalytic
Os06g0341400	Hypothetical protein.	AK110872	longestORF	-
Os06g0341500	Plant lipid transfer protein/seed storage/trypsin-alpha amylase inhibitor domain containing protein.	AK106755	Q5Z9Y7	Plant lipid transfer protein/seed storage/trypsin-alpha amylase inhibitor
Os06g0341600	Conserved hypothetical protein.	AK067335	B9FT43	-
Os06g0345732	Non-protein coding transcript.	AK241165	NONE	-
Os06g0476400	Conserved hypothetical protein.	ab initio prediction	NP_001174788.1	-
Os06g0481800	Myb/SANT-like domain domain containing protein.	BT039927	NP_001176912.1	Myb/SANT-like domain
Os06g0481850	Hypothetical conserved gene.	AK111398	NP_001176912.1	Putative harbinger transposase-derived nuclease
Os06g0488600	Similar to Potential phospholipid-transporting ATPase 7 (EC 3.6.3.1) (Aminophospholipid flippase 7	AK058968	XP_002318557.1	HAD-like domain
Os06g0497350	Similar to Cytochrome P450 CYP71K14.	ab initio prediction	B7F8Q5	Cytochrome P450
Os06g0500100	Conserved hypothetical protein.	AK241750	NP_001057706.2	-
Os06g0509600	Glycoside hydrolase, family 28 domain containing protein.	BT043275	Q5Z4W9	Glycoside hydrolase, family 28
Os06g0513781	Conserved hypothetical protein.	CT833375	B8AJ30	-
Os06g0513862	Conserved hypothetical protein.	CT833375	B8AJ30	-
Os06g0513943	Conserved hypothetical protein.	CT833375	B8AJ30	-
Os06g0514800	Conserved hypothetical protein.	CT833375	B8AJ30	-
Os06g0521300	Similar to Harpin-induced protein 1 containing protein, expressed.	AK358816	Q2RB97	Late embryogenesis abundant protein, LEA-14
Os06g0526600	Similar to predicted protein.	AK241349	Q9FFQ1	Helicase, C-terminal
Os06g0526650	Non-protein coding transcript.	AF366447	NONE	-
Os06g0526700	Probable protein phosphatase 2C 55.	Q652Z7	Q652Z7	Protein phosphatase 2C, manganese/magnesium aspartate binding site
Os06g0539801	Conserved hypothetical protein.	BT064098	A3BCH5	-
Os06g0540050	Hypothetical conserved gene.	ab initio prediction	B9FTM8	Zinc finger, RING-type
Os06g0541600	Conserved hypothetical protein.	AK356929	Q5Z6Z4	-
Os06g0554100	Hypothetical conserved gene.	CT836317	Q5Z9C5	Zinc finger, RING-type
Os06g0557200	Hypothetical protein.	AK063039	longestORF	-
Os06g0562300	Similar to BEL1-type homeodomain protein.	AB546644	D4QFI3	Homeobox
Os06g0563000	Ndr family protein.	AK099669	B6TFK0	Ndr
Os06g0563125	Hypothetical protein.	tplb0040g21 (Wheat FLcDNA)	longestORF	-
Os06g0564800	Similar to beta-1,4-mannosyltransferase.	AK288135	NP_001149006.1	-
Os06g0573800	Fanconi Anaemia group E protein, C-terminal domain containing protein.	AK106254	A2YEA6	Fanconi Anaemia group E protein, C-terminal
Os06g0579100	Conserved hypothetical protein.	AK068691	B8ATT0	-
Os06g0580100	Similar to cDNA clone:006-306-H07, full insert sequence.	AK059529	B7E6C5	-
Os06g0586000	Conserved hypothetical protein.	AK119252 ,AK063903	A2XQZ9	-
Os06g0609400	Peptidase S8, subtilisin-related domain containing protein.	AK109185	C0JA17	-
Os06g0609450	Similar to ZAC.	AK287598	C0JA35	C2 calcium-dependent membrane targeting
Os06g0609500	Homeodomain-like containing protein.	AK071731	C0JA19	Myb-like DNA-binding domain, SHAQKYF class
Os06g0609600	EF-Hand type domain containing protein.	AK072533 ,AK099872	C0JA20	-
Os06g0609700	Esterase/lipase/thioesterase domain containing protein.	AK067228	C0JA21	-
Os06g0609775	Hypothetical protein.	tplb0061n07 (Wheat FLcDNA)	longestORF	-
Os06g0612300	Similar to T6J4.5 protein (WIP6 protein).	AK071104	A6N8R9	Zinc finger, C2H2
Os06g0614400	Conserved hypothetical protein.	AK120383	Q5Z7S3	-
Os06g0632400	Similar to OSIGBa0115M15.3 protein.	EU976099	D0VLU6	-
Os06g0632700	MULE transposase, conserved domain domain containing protein.	AK101148	D0VLU5	Phox/Bem1p
Os06g0632800	Hypothetical protein.	tplb0035l23 (Wheat FLcDNA)	longestORF	-
Os06g0633100	Similar to P10Sh249C12.	AK107791	D0VLU4	-
Os06g0633375	Hypothetical protein.	FP099929	longestORF	-
Os06g0633450	Hypothetical conserved gene.	ab initio prediction	Q67VL1	-
Os06g0633500	Zinc finger, RING/FYVE/PHD-type domain containing protein.	AK108505	B6TQ30	Zinc finger, RING-type
Os06g0633800	Amino acid transporter, transmembrane domain containing protein.	AK073428	B6TE03	Amino acid transporter, transmembrane
Os06g0635700	Conserved hypothetical protein.	AK107643	A3BDV0	-
Os06g0636201	Conserved hypothetical protein.	EU968448	NP_001144438.1	-
Os06g0636600	Protein kinase, core domain containing protein.	AK119586	NP_001147925.1	Protein kinase, catalytic domain
Os06g0636800	Conserved hypothetical protein.	EU970200	NP_001058146.1	-
Os06g0638100	Similar to Tic21.	AK073270	Q09S70	Protein of unknown function DUF3611
Os06g0638200	Uncharacterised protein family UPF0047 domain containing protein.	AK059450	B9FQ34	Uncharacterised protein family UPF0047
Os06g0638500	Tyrosine protein kinase domain containing protein.	AK360940	Q67WE5	Protein kinase, catalytic domain
Os06g0639250	Conserved hypothetical protein.	ab initio prediction	Q67WD8	-
Os06g0639550	Non-protein coding transcript.	AK099542	NONE	-
Os06g0645700	Similar to HAF1.	AK108404	Q67W65	Bromodomain
Os06g0645901	Similar to Transcription initiation factor TFIID subunit 1.	BT037416	Q67W65	-
Os06g0649550	Non-protein coding transcript.	BT086930	NONE	-
Os06g0652501	Non-protein coding transcript.	X06283	NONE	-
Os06g0656566	Hypothetical gene.	BT062457	longestORF	-
Os06g0656800	FAS1 domain domain containing protein.	AK109762	NP_001150349.1	FAS1 domain
Os06g0662000	Similar to Vacuolar ATP synthase catalytic subunit A.	AK071775	B6UH55	ATPase, F1/V1/A1 complex, alpha/beta subunit, N-terminal
Os06g0665100	Similar to Dreg-2 like protein.	AK101330	NP_199286.1	Haloacid dehalogenase-like hydrolase
Os06g0667100	Similar to Nuclear Y/CCAAT-box binding factor C subunit NF-YC.	AK105079 ,AK066630	NP_001149626.1	Transcription factor CBF/NF-Y/archaeal histone
Os06g0667300	Non-protein coding transcript.	AK102272	NONE	-
Os06g0667400	Conserved hypothetical protein.	AK065424	Q655T7	-
Os06g0667600	Similar to Glycine decarboxylase complex H-protein.	AK071621 ,AK058606	NP_001152670.1	Glycine cleavage H-protein
Os06g0667900	Disease resistance protein domain containing protein.	ab initio prediction	Q655T3	Disease resistance protein

Os06g0668000	Hypothetical protein.	AK064023	longestORF	-
Os06g0668200	Similar to Phosphoglycerate kinase, cytosolic (EC 2.7.2.3).	AK070705 ,AK101622	A2YG06	Phosphoglycerate kinase
Os06g0668250	Hypothetical gene.	BT088042	longestORF	-
Os06g0668400	Kelch related domain containing protein.	AK108036	B9FQE4	BTB/POZ-like
Os06g0669100	Conserved hypothetical protein.	AK099506	Q653E7	-
Os06g0669200	Conserved hypothetical protein.	AK062363	Q5Z8D4	-
Os06g0669225	Hypothetical conserved gene.	ab initio prediction	Q655S3	MATH
Os06g0669275	Similar to MEE44 (maternal effect embryo arrest 44)%3B nucleotidyltransferase.	BT018457	NP_191917.2	PAP/25A-associated
Os06g0669700	Similar to DNA binding protein.	AK070871	NP_001149442.1	Transcription regulator HTH, Myb-type, DNA-binding
Os06g0669800	Ovarian tumour, otubain domain containing protein.	AK073551	B6U3W2	Ovarian tumour, otubain
Os06g0670000	Similar to Molybdenum cofactor sulfurase.	AK287475	Q655R6	Aminotransferase, class V/Cysteine desulfurase
Os06g0670100	Conserved hypothetical protein.	AK102577	A3BEH8	-
Os06g0670300	Homeodomain-like containing protein.	AK070966	NP_001147627.1	Myb-like DNA-binding domain, SHAQKYF class
Os06g0674800	Hypothetical conserved gene.	AK241848	B9FQG9	-
Os06g0678700	Hypothetical gene.	AK107560	Q655H6	-
Os06g0683925	Hypothetical protein.	tplb0033d10 (Wheat FLcDNA)	longestORF	-
Os06g0690400	Non-protein coding transcript.	EU941368	NONE	-
Os06g0691600	EF-Hand type domain containing protein.	AK070430	NP_001150907.1	Calcium-binding EF-hand
Os06g0692050	Conserved hypothetical protein.	FP100342	Q5Z672	-
Os06g0698812	Disease resistance protein domain containing protein.	ab initio prediction	XP_002438943.1	Disease resistance protein
Os06g0703900	Homeodomain-like containing protein.	AK067950	B8B262	Myb-like DNA-binding domain, SHAQKYF class
Os06g0705300	Similar to plant-specific domain TIGR01589 family protein.	AK064803	NP_001149236.1	Conserved hypothetical protein CHP01589, plant
Os06g0705350	Similar to pentatricopeptide (PPR) repeat-containing protein.	BT055332	NP_199702.1	Pentatricopeptide repeat
Os06g0705400	Hypothetical conserved gene.	AK062493	C0KHK5	Plant lipid transfer protein/hydrophobic protein, helical domain
Os06g0705651	Hypothetical protein.	EU955776	longestORF	-
Os06g0705700	TGF-beta receptor, type I/II extracellular region family protein.	AK119527	A2YGT5	Oligopeptide transporter
Os06g0705901	Hypothetical protein.	tplb0058e01 (Wheat FLcDNA)	longestORF	-
Os06g0707800	Non-protein coding transcript.	AK111291	NONE	-
Os06g0708300	Similar to RER1A protein.	AK243324	NP_001151498.1	Retrieval of early ER protein Rer1
Os06g0708600	Zinc finger, C2H2-like domain containing protein.	AK100915	E4MXX2	Zinc finger, C2H2-like
Os06g0708700	Similar to Nodulin-like protein.	AK067151	B6SKJ2	Drug/metabolite transporter
Os06g0708832	Similar to arogenate dehydrogenase.	AK355541	NP_001147429.1	Prephenate dehydrogenase
Os06g0710001	Hypothetical conserved gene.	AK249657	Q5Z9G7	-
Os06g0710300	Uncharacterised conserved protein UCP022348 domain containing protein.	AK121344	B6U4B2	Uncharacterised protein family UPF0114
Os06g0710401	Non-protein coding transcript.	AK330826	NONE	-
Os06g0710850	Non-protein coding transcript.	EU950496	NONE	-
Os06g0710900	Conserved hypothetical protein.	AK073326	A2YGW4	-
Os06g0711800	Pectinesterase inhibitor domain containing protein.	AK069642	NP_001167668.1	Pectinesterase inhibitor
Os06g0712250	Hypothetical conserved gene.	CT832454	NP_001144189.1	-
Os06g0712300	EF-Hand type domain containing protein.	AK070744	E6NU22	Phospholipid/glycerol acyltransferase
Os06g0712400	Protein of unknown function DUF544 family protein.	AK067573	B8B2H1	Ubiquitin interacting motif
Os06g0712550	Hypothetical protein.	tplb0056b02 (Wheat FLcDNA)	longestORF	-
Os06g0712600	Similar to SHI.	EU959412	NP_001150905.1	Zinc finger, lateral root primordium type 1
Os06g0712900	tRNA-dihydrouridine synthase domain containing protein.	AK106648	NP_201523.1	tRNA-dihydrouridine synthase
Os06g0713400	Cyclin-like F-box domain containing protein.	AK100628	B9FQV7	F-box domain, cyclin-like
Os06g0714400	Conserved hypothetical protein.	AK108610	Q5NAL1	-
Os06g0714432	Non-protein coding transcript.	CT836310	NONE	-
Os06g0714600	ADP-ribosylation factor domain containing protein.	AK318603	A6MD16	Small GTPase superfamily
Os06g0714700	Conserved hypothetical protein.	CU406678	NP_001058577.1	-
Os06g0716050	Hypothetical gene.	EU948676	longestORF	-
Os06g0716700	Similar to Heat shock protein 90.	AK063385	Q9MB32	Heat shock protein Hsp90
Os06g0718400	Hypothetical conserved gene.	CT836320	Q8SB26	Blue
Os06g0719600	Domain of unknown function DUF1618 domain containing protein.	AK099272	Q8SB18	Domain of unknown function DUF1618
Os06g0719900	Similar to endo/excinuclease amino terminal domain-containing protein.	CT834222	XP_002881103.1	-
Os06g0721600	Hypothetical conserved gene.	ab initio prediction	NP_001058606.2	Protein of unknown function DUF544
Os06g0721800	Cupredoxin domain containing protein.	AK243644	A2YH12	Blue
Os06g0722100	Hypothetical conserved gene.	AK068839	NP_001058607.1	Domain of unknown function DUF1618
Os06g0723400	Hypothetical conserved gene.	ab initio prediction	Q5YLZ3	Protein of unknown function DUF544
Os06g0725350	Hypothetical gene.	tplb0017d24 (Wheat FLcDNA)	longestORF	-
Os06g0725400	Similar to BLE1 protein.	AK241158	Q8S937	-
Os06g0726200	Similar to Chitinase 1.	AK061042 ,AK099339 ,AK10402	Q42993	Glycoside hydrolase, family 19, catalytic
Os06g0726501	Non-protein coding transcript.	tplb0001a06 (Wheat FLcDNA)	NONE	-
Os06g0727000	Zinc finger, C2H2-type domain containing protein.	AK108097	A2YH63	Zinc finger, C2H2
Os06g0727400	Similar to Protein kinase APK1A, chloroplast precursor (EC 2.7.1.-).	AK069558	NP_001147720.1	Protein kinase, catalytic domain
Os06g0727500	Hypothetical protein.	tplb0023d22 (Wheat FLcDNA)	longestORF	-
Os06g0728902	Similar to predicted protein.	AK240761	NP_001154712.1	Protein of unknown function DUF605
Os06g0729150	Hypothetical protein.	tplb0026f23 (Wheat FLcDNA)	longestORF	-
Os06g0729300	Similar to Protein argonaute 1D.	AK111833	Q5Z5B2	Stem cell self-renewal protein Piwi
Os06g0729350	Non-protein coding transcript.	BT016774	NONE	-
Os06g0729650	Similar to Photosystem II stability/assembly factor HCF136, chloroplastic.	FP096975	B9FR06	Twin-arginine translocation pathway, signal sequence
Os06g0729800	Hypothetical gene.	AK241958	longestORF	-
Os06g0730000	Similar to Serine carboxypeptidase II-like protein.	AK242427	NP_001152245.1	Peptidase S10, serine carboxypeptidase
Os06g0731000	Conserved hypothetical protein.	ab initio prediction	NP_001175010.1	-
Os06g0731200	Similar to ABC transporter-like protein.	AK071489	XP_002303777.1	ABC transporter-like
Os06g0731300	Surface protein from Gram-positive cocci, anchor region domain containing protein.	AK358759	Q5Z408	Heat shock protein DnaJ, cysteine-rich domain
Os06g0731400	Hypothetical conserved gene.	AK120175	B8B2Y0	-
Os06g0731500	Conserved hypothetical protein.	AK062465	Q5Z405	-
Os06g0731750	Hypothetical gene.	BT016392	longestORF	-
Os06g0731800	Clathrin light chain family protein.	AK069288	Q259D4	Clathrin light chain
Os06g0732000	Similar to 60S ribosomal protein L35.	FP092387	NP_001148517.1	-
Os07g0116050	Transposase, IS4-like domain containing protein.	BT037367	XP_002449014.1	Transposase, IS4-like
Os07g0117600	Conserved hypothetical protein.	ab initio prediction	Q8H5J6	-
Os07g0132800	Hypothetical gene.	AK240792	longestORF	-
Os07g0144900	Similar to ASK11 (ARABIDOPSIS SKP1-LIKE 11)%3B ubiquitin-protein ligase.	AK242552	NP_567959.1	SKP1 component
Os07g0147500	Similar to Photosystem II 10 kDa polypeptide, chloroplast precursor.	AK062172 ,AK120691	Q40070	Photosystem II PsbR
Os07g0147550	Similar to Photosystem II 10 kDa polypeptide, chloroplast.	CT837962	Q40070	Photosystem II PsbR
Os07g0147700	ATPase, BadF/BadG/BcrA/BcrD type domain containing protein.	AK242508	NP_001152135.1	ATPase, BadF/BadG/BcrA/BcrD type
Os07g0148800	Conserved hypothetical protein.	AK062246	Q69VA2	-
Os07g0148900	Photosystem I protein-like protein.	AK058788	P36886	Photosystem I PsaG/PsaK protein
Os07g0151800	Conserved hypothetical protein.	AK105339	A2YI84	-
Os07g0151900	Conserved hypothetical protein.	AK240814	NP_001175061.1	-
Os07g0152800	Similar to Glycine-rich protein.	AK065458	B6TIW3	-
Os07g0152900	Similar to Glycolate oxidase (EC 1.1.3.15) (Fragment).	AK120929	Q6YT73	FMN-dependent dehydrogenase
Os07g0152950	Hypothetical gene.	BT066752	longestORF	-
Os07g0153000	Tify domain containing protein.	AK374356	NP_001058910.2	Tify
Os07g0153300	Protein of unknown function DUF1365 family protein.	AK066146	B9FVJ7	Protein of unknown function DUF1365
Os07g0153400	Kelch-type beta propeller domain containing protein.	AK069618	XP_002893599.1	F-box domain, cyclin-like
Os07g0153600	Similar to Prostrate growth 1.	FJ155665	D9ZKN9	Zinc finger, C2H2
Os07g0154201	Hypothetical gene.	BT085311	longestORF	-
Os07g0154300	Conserved hypothetical protein.	AK105386 ,AK122093	A2YIA2	-
Os07g0154400	Armadillo-type fold domain containing protein.	AK288457	B9FVJ8	Armadillo-type fold
Os07g0154800	Conserved hypothetical protein.	AK111389	B8B7D0	-
Os07g0156200	Haem peroxidase, plant/fungal/bacterial family protein.	AK249509	NP_001152255.1	Plant peroxidase
Os07g0157600	Similar to peroxidase 1.	AK252026	NP_001152255.1	Plant peroxidase
Os07g0161000	Tetratricopeptide-like helical domain containing protein.	AK065877	B8B7F4	Tetratricopeptide TPR-1
Os07g0162200	Protein of unknown function DUF593 domain containing protein.	tplb0058k18 (Wheat FLcDNA)	Q8H538	Protein of unknown function DUF593
Os07g0162300	Similar to RIO kinase.	AK066456	B9FVM0	RIO kinase
Os07g0168000	Similar to Polyribonucleotide phophorylase (Fragment).	AK065622	O24288	Exoribonuclease, phosphorolytic domain 1
Os07g0168300	Similar to Glutathione S-transferase GSTU6.	AK061436 ,AK099137	A2YIJ4	Glutathione S-transferase, N-terminal
Os07g0168800	Zinc finger, AN1-type domain containing protein.	AK121813	Q7Y1W9	Zinc finger, AN1-type
Os07g0168900	Hypothetical gene.	AK062924	longestORF	-
Os07g0169600	2OG-Fe(II) oxygenase domain containing protein.	AK121818 ,AK060318	NP_001152182.1	Oxoglutarate/iron-dependent oxygenase
Os07g0169700	GA 20-oxidase3, GA metabolism	Inferred from literature	Q69LD8	-
Os07g0169800	Ribonuclease CAF1 domain containing protein.	AK332128	Q69LD7	Ribonuclease CAF1
Os07g0170000	Similar to Brn1-like protein.	EU972358	NP_193054.1	Short-chain dehydrogenase/reductase SDR

Os07g0170016	Hypothetical gene.	AK289254	longestORF	-
Os07g0170200	Similar to predicted protein.	CT836091	NP_179964.2	HNH endonuclease
Os07g0170300	YT521-B-like protein family protein.	AK072392	B6U208	YTH domain
Os07g0170400	Non-protein coding transcript.	BT016473	NONE	-
Os07g0170500	Conserved hypothetical protein.	AK240864	NP_001175070.1	-
Os07g0171200	Similar to Galactose-1-phosphate uridyl transferase-like protein.	AK071075	B6TFN9	Galactose-1-phosphate uridyl transferase, class I
Os07g0171350	Non-protein coding transcript.	BT084747	NONE	-
Os07g0172200	Similar to ROOT HAIRLESS 1.	AK103352	B6UAF0	-
Os07g0172900	Hypothetical conserved gene.	AK067304	Q6ZA56	-
Os07g0173100	HSP20-like chaperone domain containing protein.	CT836094	NP_001059002.1	-
Os07g0173300	Conserved hypothetical protein.	AK110935	A3BH18	-
Os07g0175200	MTCP1 domain containing protein.	AK058437	A2YIN2	MTCP1
Os07g0175350	Hypothetical protein.	tplb0022c02 (Wheat FLcDNA)	longestORF	-
Os07g0175400	Hypothetical conserved gene.	AK331548	NP_001175074.1	Cyclic nucleotide-binding domain
Os07g0175801	Non-protein coding transcript.	X02683	NONE	-
Os07g0175900	Similar to Inositol-1, 4, 5-trisphosphate 5-Phosphatase-like protein.	AK067194	NP_001151627.1	Inositol polyphosphate-related phosphatase
Os07g0176200	Similar to Transcription factor CPP.	AK062003	B3IX45	Tesmin/TSO1-like, CXC
Os07g0176400	Hypothetical protein.	BT085651	longestORF	-
Os07g0176401	Hypothetical protein.	tplb0061g22 (Wheat FLcDNA)	longestORF	-
Os07g0176500	Leucine-rich repeat, plant specific containing protein.	AK062887	A2YIP1	Leucine-rich repeat
Os07g0176600	Similar to Kinase-like protein.	AK067073 ,AK066812	Q6ZEZ5	Protein kinase, catalytic domain
Os07g0176900	Similar to ribose-5-phosphate isomerase.	AK067117	Q8RU73	Ribose 5-phosphate isomerase, type A
Os07g0177100	Armadillo-type fold domain containing protein.	AK070264	B9FVT4	Armadillo-type fold
Os07g0177150	Non-protein coding transcript.	AK288093	NONE	-
Os07g0177200	Similar to glutamate binding protein.	AK070925	XP_002878509.1	Inhibitor of apoptosis-promoting Bax1-related
Os07g0177300	Uncharacterised protein family UPF0005 domain containing protein.	AK073781	B6T7B7	Inhibitor of apoptosis-promoting Bax1-related
Os07g0178950	Hypothetical protein.	tplb0047n14 (Wheat FLcDNA)	longestORF	-
Os07g0179000	Pentatricopeptide repeat domain containing protein.	AK100008	A2YIR0	Pentatricopeptide repeat
Os07g0190900	Forkhead-associated domain containing protein.	AK073533	A3BHE5	Forkhead-associated
Os07g0191200	Plasma membrane H+ ATPase (EC 3.6.3.6).	AK121402	Q43002	ATPase, P-type, H+ transporting proton pump
Os07g0191250	Hypothetical protein.	tplb0039e05 (Wheat FLcDNA)	longestORF	-
Os07g0191600	Protein of unknown function DUF569 domain containing protein.	AK102563	B8B850	Protein of unknown function DUF569
Os07g0191625	Hypothetical protein.	AK361241	longestORF	-
Os07g0191801	Non-protein coding transcript.	AK288998	NONE	-
Os07g0192000	ATPase, AAA-type, core domain containing protein.	AK065505	B6SVY2	ATPase, AAA+ type, core
Os07g0192800	Similar to ATPase 3.	AK288139	NP_001148126.1	ATPase, AAA+ type, core
Os07g0193100	Non-protein coding transcript.	EU949559	NONE	-
Os07g0193200	Ankyrin repeat containing protein.	ab initio prediction	NP_001059105.1	Ankyrin repeat
Os07g0193701	Hypothetical protein.	BT086982	longestORF	-
Os07g0193800	Similar to BHLH transcription factor (Fragment).	AK242645	F1DK89	Helix-loop-helix DNA-binding
Os07g0194400	Conserved hypothetical protein.	AB332080	NP_001175091.1	-
Os07g0194500	2OG-Fe(II) oxygenase domain containing protein.	AK121816	XP_002324024.1	Metridin-like ShK toxin
Os07g0194550	Non-protein coding transcript.	BT084776	NONE	-
Os07g0204900	Similar to Zeta-carotene desaturase (Fragment).	AK065213	A9UKC6	Amine oxidase
Os07g0205000	Similar to Ubiquinol-cytochrome c reductase complex 14 kDa protein.	AK103963	B6TMP9	Cytochrome d ubiquinol oxidase, 14kDa subunit
Os07g0205500	Protein of unknown function DUF239, plant domain containing protein.	AK103449	B6TV70	Glucoamylase, putatuve
Os07g0205600	Hypothetical protein.	tplb0032e19 (Wheat FLcDNA)	longestORF	-
Os07g0206300	Conserved hypothetical protein.	CT836132	NP_001175100.1	-
Os07g0206400	13 kDa prolamin precursor.	AK242940	P17048	Gliadin/LMW glutenin
Os07g0206500	13 kDa prolamin precursor.	X60979	Q9SAY8	Gliadin/LMW glutenin
Os07g0209401	Similar to DNA polymerase.	ab initio prediction	B9FUS4	-
Os07g0209500	Similar to DNA polymerase.	AK108416	B9FUS4	-
Os07g0210800	Hypothetical conserved gene.	AK106587	Q60DP5	Myb/SANT-like domain
Os07g0211601	Hypothetical gene.	AK288862	longestORF	-
Os07g0211700	Similar to isopentenyl transferase IPT4.	AB239801	NP_001121195.1	tRNA isopentenyltransferase
Os07g0211900	Protein of unknown function DUF632 domain containing protein.	AK243281	Q8H4U8	Domain of unknown function DUF632
Os07g0213600	Bifunctional inhibitor/plant lipid transfer protein/seed storage domain containing protein.	AK107696	A2YJF5	Plant lipid transfer protein/seed storage/trypsin-alpha amylase inhibitor
Os07g0214100	Seed allergenic protein RA17 precursor.	AK242324	Q01883	Cereal allergen/alpha-amylase inhibitor, rice-type
Os07g0214600	Similar to Seed allergenic protein RA17 precursor.	AK242298	Q40654	Cereal allergen/alpha-amylase inhibitor, rice-type
Os07g0214900	Similar to Chalcone synthase C2 (EC 2.3.1.74) (Naringenin-chalcone synthase C2).	FP093770	Q8H4L3	Chalcone/stilbene synthase, N-terminal
Os07g0215050	Hypothetical protein.	BT063109	longestORF	-
Os07g0215500	Allergenic protein.	AK242333	Q01881	Cereal allergen/alpha-amylase inhibitor, rice-type
Os07g0218700	Cytochrome P450 family protein.	AK108382	Q01LP1	Cytochrome P450
Os07g0218766	Hypothetical protein.	tplb0041k22 (Wheat FLcDNA)	longestORF	-
Os07g0220466	Similar to H0315A08.1 protein.	FP099421	Q01I63	Ribonuclease H1, N-terminal
Os07g0223733	Non-protein coding transcript.	BT016899	NONE	-
Os07g0290500	Plant lipid transfer/seed storage/trypsin-alpha amylase inhibitor domain containing protein.	EU957442	NP_001151008.1	-
Os07g0301050	Non-protein coding transcript.	BT068036	NONE	-
Os07g0468900	Hypothetical gene.	AK109042	longestORF	-
Os07g0487198	Hypothetical protein.	tplb0040j01 (Wheat FLcDNA)	longestORF	-
Os07g0487300	Protein kinase, catalytic domain domain containing protein.	ab initio prediction	Q84ZD7	Protein kinase, catalytic domain
Os07g0488215	Hypothetical protein.	tplb0040j01 (Wheat FLcDNA)	longestORF	-
Os07g0488400	Protein kinase, catalytic domain domain containing protein.	ab initio prediction	Q84ZD7	Protein kinase, catalytic domain
Os07g0523400	Glucose-6-phosphate/phosphate-translocator precursor.	AK070124	NP_001147439.1	Drug/metabolite transporter
Os07g0536800	Hypothetical conserved gene.	AK109751	Q53NH3	-
Os07g0555200	Similar to predicted protein.	AK062438	D4N2J6	MIF4G-like, type 3
Os07g0562900	Hypothetical conserved gene.	CT836099	B9FXX6	-
Os07g0564533	Similar to HAT family dimerisation domain containing protein.	AK288170	Q53RM1	HAT dimerisation
Os07g0567700	Similar to Scarecrow-like 23 (Fragment).	AK059008	Q6ULS4	Transcription factor GRAS
Os07g0569600	Chaperonin-like RbcX domain containing protein.	AK067599	A3BLB7	Chaperonin-like RbcX
Os07g0574150	Hypothetical protein.	EU951884	longestORF	-
Os07g0575000	Pathogenesis-related transcriptional factor/ERF, DNA-binding domain containing protein.	AK374106	Q6ZL38	Pathogenesis-related transcriptional factor/ERF, DNA-binding
Os07g0575733	Conserved hypothetical protein.	ab initio prediction	NP_001053398.1	-
Os07g0575900	Protein of unknown function DUF946, plant family protein.	AK105640	B9FY25	Vacuolar protein sorting-associated protein 62
Os07g0585200	Bifunctional inhibitor/plant lipid transfer protein/seed storage domain containing protein.	AK242443	A2YL71	Plant lipid transfer protein/seed storage/trypsin-alpha amylase inhibitor
Os07g0585500	Conserved hypothetical protein.	AK070285	B9FY48	-
Os07g0585700	Zinc finger protein, Pre-harvest sprouting resistance	AB510199	Q8H5K8	-
Os07g0585900	Hypothetical conserved gene.	FP093392	Q8GRW3	-
Os07g0586000	Similar to ZCW7.	AK069212	B6TC50	-
Os07g0586266	Hypothetical protein.	EU949155	longestORF	-
Os07g0586600	Conserved hypothetical protein.	AK287951	NP_001175279.1	-
Os07g0586700	Similar to HRT transcription factor (Fragment).	AK102792	F1DJZ9	-
Os07g0591202	Hypothetical gene.	BT017174	longestORF	-
Os07g0599500	Hypothetical protein.	AK068696 ,AK106010	GeneMark	-
Os07g0599600	Hypothetical protein.	AK105918 ,AK070771 ,AK10621	GeneMark	-
Os07g0600000	Conserved hypothetical protein.	AK105907	A3BLW3	-
Os07g0633450	Hypothetical gene.	EU947431	longestORF	-
Os07g0639400	Similar to Peroxidase 1.	AK110555	A3BMN4	Plant peroxidase
Os07g0646300	Hypothetical conserved gene.	AK063331	NP_001060464.1	-
Os07g0646800	Similar to Avr9/Cf-9 rapidly elicited protein 231.	AK071716	XP_002311936.1	Glycosyl transferase, family 8
Os07g0648566	Hypothetical gene.	AK059417	longestORF	-
Os07g0648700	Conserved hypothetical protein.	ab initio prediction	NP_001175315.1	-
Os07g0666900	Vacuolar Na+/H+ antiporter, Salt tolerance	AB021878	Q9SXJ8	-
Os07g0667900	Hypothetical protein.	tplb0040h06 (Wheat FLcDNA)	longestORF	-
Os07g0668000	Similar to cDNA clone:J033024L12, full insert sequence.	CT836044	Q7F0M8	-
Os07g0668100	Conserved hypothetical protein.	AK106702	B9FUS0	-
Os07g0668200	Similar to adenyl cyclase.	AK064146	NP_001149561.1	-
Os07g0668300	Lipase, GDSL domain containing protein.	AK061122	NP_001148291.1	Lipase, GDSL
Os07g0668500	Protein kinase, catalytic domain domain containing protein.	EU955462	Q7F0M5	Protein kinase, catalytic domain
Os07g0669200	Similar to GTP1/OBG family protein.	BT063209	NP_197358.2	GTP-binding domain, HSR1-related
Os07g0669250	Similar to predicted protein.	EU950976	XP_002873904.1	GTP-binding protein GTP1/OBG, C-terminal
Os07g0669401	Conserved hypothetical protein.	FP091568	NP_001042416.1	-
Os07g0669500	Similar to Branched silkless1.	AK105365	A1KZE8	Pathogenesis-related transcriptional factor/ERF, DNA-binding
Os07g0669600	Conserved hypothetical protein.	AK066595	B9FUS7	-
Os07g0669700	Potassium transporter 4 (AtPOT4) (AtKUP3) (AtKT4).	AK100652	Q8H3P9-2	K+ potassium transporter

Os07g0669900	Non-protein coding transcript.	AK062892	NONE	-
Os07g0670000	Pentatricopeptide repeat domain containing protein.	AK109518	A2YPS5	Pentatricopeptide repeat
Os07g0670100	Hypothetical conserved gene.	CT836088	Q8GSF2	-
Os07g0670800	Similar to 60S ribosomal protein L19-3.	AK241287	NP_001148813.1	-
Os07g0671000	Similar to FPF1.	AK358497	NP_001151726.1	-
Os07g0671200	Pentatricopeptide repeat domain containing protein.	EU971084	NP_001060597.1	Pentatricopeptide repeat
Os07g0671301	Hypothetical gene.	EU973702	longestORF	-
Os07g0671400	Heavy metal transport/detoxification protein domain containing protein.	AK067933	NP_001149888.1	Heavy metal-associated domain, HMA
Os07g0674100	Hypothetical conserved gene.	AK099614	B9FUU7	NAD-dependent epimerase/dehydratase
Os07g0674150	Hypothetical protein.	tplb0036f05 (Wheat FLcDNA)	longestORF	-
Os07g0674200	Similar to 60S ribosomal protein L22-2.	AK058245	B6U998	Ribosomal protein L22e
Os07g0674300	Protein kinase, ATP binding site domain containing protein.	AK110511	A2YPV3	Pentatricopeptide repeat
Os07g0674500	Pollen Ole e 1 allergen/extensin domain containing protein.	AK107802	A2YPV5	Pollen Ole e 1 allergen/extensin
Os07g0674800	Similar to AP2 domain transcription factor EREBP.	AK288040	Q7Y047	Pathogenesis-related transcriptional factor/ERF, DNA-binding
Os07g0675166	Non-protein coding transcript.	AK110786	NONE	-
Os07g0675200	Conserved hypothetical protein.	AK063620	B8B5V2	-
Os07g0676200	Non-protein coding transcript.	AK061503	NONE	-
Os07g0676400	Hypothetical conserved gene.	CT836080	B8B5V6	Protein kinase, catalytic domain
Os07g0677400	Peroxidase.	AF014469	Q9LKY9	Plant peroxidase
Os07g0677600	Similar to Cationic peroxidase.	AK060984	NP_001147443.1	Plant peroxidase
Os07g0677900	Protein of unknown function DUF827, plant family protein.	AK103884 ,AK106293	A2YPX8	Protein of unknown function DUF827, plant
Os07g0684500	Non-protein coding transcript.	AK060591	NONE	-
Os07g0687200	Similar to CAM7 (CALMODULIN 7)%3B calcium ion binding.	AK104031	NP_850344.1	Calcium-binding EF-hand
Os07g0690600	Protein of unknown function DUF1677, Oryza sativa domain containing protein.	AK365840	Q8H3Z2	Protein of unknown function DUF1677, Oryza sativa
Os07g0691300	Wound-induced protein, Wun1, subgroup domain containing protein.	AK069499	B8B6A4	Wound-induced protein, Wun1
Os07g0692500	Conserved hypothetical protein.	AK108769	Q7XI64	-
Os07g0692900	Similar to Ubiquitin-activating enzyme E1.	AK074007	B8B6A7	Ubiquitin/SUMO-activating enzyme E1
Os07g0692950	Hypothetical protein.	tplb0053b11 (Wheat FLcDNA)	longestORF	-
Os08g0105850	Hypothetical protein.	tplb0042b08 (Wheat FLcDNA)	longestORF	-
Os08g0117800	Cation/H+ exchanger domain containing protein.	AK100696	A2YQP4	Cation/H+ exchanger
Os08g0117900	Myeloid leukemia factor domain containing protein.	AK102753	B8BAC8	Myeloid leukemia factor
Os08g0118500	Similar to Auxin-induced SAUR-like protein.	CT835918	NP_001148413.1	Auxin responsive SAUR protein
Os08g0118700	Non-protein coding transcript.	X06283	NONE	-
Os08g0118800	Similar to SAUR31 - auxin-responsive SAUR family member.	ab initio prediction	NP_001148413.1	Auxin responsive SAUR protein
Os08g0119000	CSN3 (Fragment).	AK058565	B6TEU1	Proteasome component
Os08g0119100	Protein of unknown function DUF1517 domain containing protein.	AK353591	Q6ZJ46	Protein of unknown function DUF1517
Os08g0119500	Methyltransferase type 11 domain containing protein.	AK242531	NP_680738.1	Methyltransferase type 11
Os08g0127300	Conserved hypothetical protein.	AK058548	Q6ZK51	-
Os08g0127750	Non-protein coding transcript.	AK242810	NONE	-
Os08g0127800	Conserved hypothetical protein.	AK103321 ,AK108548	Q6ZK47	-
Os08g0132500	Conserved hypothetical protein.	AK363691	Q69R49	-
Os08g0133200	Similar to copine-3.	AK241367	NP_001147038.1	Copine
Os08g0140600	Conserved hypothetical protein.	ab initio prediction	Q6YZ17	-
Os08g0141000	Conserved hypothetical protein.	EU960934	NP_001060971.2	-
Os08g0141300	PapD-like domain containing protein.	AK058337 ,AK067917	B6TP74	Major sperm protein
Os08g0142500	Pectinesterase inhibitor domain containing protein.	AK242564	NP_001175375.1	Pectinesterase inhibitor
Os08g0143150	Similar to retrotransposon protein.	EU953494	NP_001158937.1	-
Os08g0143400	Amine oxidase domain containing protein.	AK120532	NP_187981.1	Amine oxidase
Os08g0143500	Glycosyl transferase, family 14 protein.	AK071122	NP_001147601.1	Glycosyl transferase, family 14
Os08g0144000	Myb, DNA-binding domain containing protein.	AK073681	B4FLJ6	SANT domain, DNA binding
Os08g0144400	Hypothetical conserved gene.	AK108707	B9FZ15	-
Os08g0145501	Conserved hypothetical protein.	AK241170	B9FSE2	-
Os08g0146001	Hypothetical conserved gene.	ab initio prediction	B9FZ18	-
Os08g0148300	Conserved hypothetical protein.	AK105581	A3BPL9	-
Os08g0163700	Conserved hypothetical protein.	AK375159	Q84S46	-
Os08g0166750	Hypothetical protein.	AK372466	longestORF	-
Os08g0172433	Non-protein coding transcript.	EU941912	NONE	-
Os08g0182400	Conserved hypothetical protein.	AK059491	NP_001061148.1	-
Os08g0216450	Conserved hypothetical protein.	EU951430	Q65WW4	-
Os08g0240966	Non-protein coding transcript.	CT836001	NONE	-
Os08g0241300	Similar to OSIGBa0118P15.3 protein.	AK108203	Q01KK8	-
Os08g0241400	Similar to protein binding protein.	EU961893	NP_001149055.1	Zinc finger, RING-type
Os08g0241800	Similar to Plasma membrane H+-ATPase (EC 3.6.1.3).	AK108449	Q8RW30	ATPase, P-type, transmembrane domain
Os08g0241900	Hypothetical protein.	AK106690	longestORF	-
Os08g0242400	Similar to WUSCHEL-related homeobox 8.	AK107305	Q6Z3L4	Homeobox
Os08g0242900	Afadin/alpha-actinin-binding domain containing protein.	AK060264 ,AK103204	B9FZT2	Afadin/alpha-actinin-binding
Os08g0243001	Non-protein coding transcript.	BT017491	NONE	-
Os08g0243500	Similar to NADPH-cytochrome P450 oxydoreductase isoform 2.	AK099083 ,AK068915	B6S78	Flavodoxin
Os08g0248900	Similar to ADP-ribosylation factor 3.	AK073613	B4FM64	Small GTP-binding protein domain
Os08g0299200	Adenylate cyclase domain containing protein.	AK241974	B6TQK5	CYTH domain
Os08g0301551	Non-protein coding transcript.	AJ238715	NONE	-
Os08g0323266	Similar to Signal recognition particle 68 kDa protein.	ab initio prediction	B6U4F2	-
Os08g0323800	Conserved hypothetical protein.	AK108337	Q6Z0E2	-
Os08g0328050	Hypothetical protein.	EU945299	longestORF	-
Os08g0334900	Xyloglucan fucosyltransferase family protein.	AK060562	A2YU28	Xyloglucan fucosyltransferase
Os08g0338500	Pectinesterase inhibitor domain containing protein.	AK107751	NP_001148732.1	Pectinesterase inhibitor
Os08g0347500	Similar to PsbP family protein, expressed.	ab initio prediction	Q109K8	Photosystem II PsbP, oxygen evolving complex
Os08g0393150	Similar to OSIGBa0110B10.2 protein.	EU973161	Q01KQ6	Zinc finger, BED-type predicted
Os08g0404350	Similar to FACT complex subunit SPT16.	ab initio prediction	Q7X923	-
Os08g0411900	Conserved hypothetical protein.	AK243392	B9G0V4	-
Os08g0433000	Similar to Arginine decarboxylase.	ab initio prediction	A3B825	Arginine decarboxylase
Os08g0433050	Similar to Arginine decarboxylase.	EU944353	A3B825	-
Os08g0433200	Conserved hypothetical protein.	AK099471	B8BB11	-
Os08g0433400	Hypothetical conserved gene.	AK059468	A2YVI0	SANT domain, DNA binding
Os08g0433600	Protein of unknown function DUF962 family protein.	AK067255	NP_001151002.1	Protein of unknown function DUF962
Os08g0434300	Similar to Malate dehydrogenase precursor (EC 1.1.1.37).	AK058477	A2YVI5	Lactate/malate dehydrogenase, N-terminal
Os08g0434500	Hypothetical protein.	ab initio prediction	NONE	-
Os08g0434700	Homeodomain-like containing protein.	AK070338	B8BB14	Myb-like DNA-binding domain, SHAQKYF class
Os08g0436700	Similar to NAC transcription factor.	AK073013	A2YVJ8	No apical meristem
Os08g0437566	Similar to H0315A08.1 protein.	FP099421	Q01I63	Ribonuclease H1, N-terminal
Os08g0438801	Hypothetical protein.	AK287665	longestORF	-
Os08g0439000	Similar to pyrophosphate--fructose 6-phosphate 1-phosphotransferase.	AK287794	NP_001147085.1	Phosphofructokinase domain
Os08g0439200	Non-protein coding transcript.	AK059480	NONE	-
Os08g0439600	Protein of unknown function DUF868, plant family protein.	BT070111	NP_001061898.1	Protein of unknown function DUF868, plant
Os08g0440100	Similar to Temperature stress-induced lipocalin.	AK068551	Q38JE5	Lipocalin/cytosolic fatty-acid binding protein domain
Os08g0440800	Glyceraldehyde-3-phosphate dehydrogenase.	AK071520	Q8S4Y9	Aldehyde dehydrogenase domain
Os08g0440850	Hypothetical gene.	EU945888	longestORF	-
Os08g0441100	Similar to PnC401 homologue.	AK108225	Q6Z9F4	Protein kinase, catalytic domain
Os08g0441300	Hypothetical conserved gene.	ab initio prediction	Q6Z9F3	Proteinase inhibitor I13, potato inhibitor I
Os08g0441500	Similar to cinnamoyl CoA reductase.	AK102061	XP_002299260.1	NAD-dependent epimerase/dehydratase
Os08g0441600	Similar to Chorismate mutase CM2 (EC 5.4.99.5) (Chorimate mutase).	AK069725	B4FUP5	Chorismate mutase, AroQ class, eukaryotic type
Os08g0442600	Hypothetical protein.	AK105576	longestORF	-
Os08g0442900	Hypothetical protein.	AK110520	GeneMark	-
Os08g0443800	Tetraspanin domain containing protein.	AK110630	NP_001105285.1	Tetraspanin
Os08g0444100	Similar to Splicing factor 3b, subunit 5 (Splicing factor 3B subunit 10).	AK073746	E5GC24	Splicing factor 3B subunit 5/RDS3 complex subunit 10
Os08g0444440	Hypothetical conserved gene.	AK241238	B9G163	-
Os08g0444500	Conserved hypothetical protein.	CT835928	NP_001061923.1	-
Os08g0445000	Similar to RING-H2 finger protein ATL5A.	AK106866	B6T239	Zinc finger, RING-type
Os08g0445250	Hypothetical protein.	tplb0042b14 (Wheat FLcDNA)	longestORF	-
Os08g0445700	HAD-superfamily hydrolase subfamily IIB protein.	AK072132	B6T0F0	Glycosyl transferase, family 20
Os08g0446200	Similar to Receptor-like protein kinase precursor (EC 2.7.1.37). Splice isoform INRPK1a.	AK120424	XP_002445581.1	Protein kinase, catalytic domain
Os08g0446250	Hypothetical gene.	BT068372	longestORF	-
Os08g0446350	Non-protein coding transcript.	BT068372	NONE	-
Os08g0446750	Hypothetical protein.	EU976033	longestORF	-
Os08g0446800	Similar to GDU1.	AK243202	NP_001151217.1	-

Os08g0447000	Similar to D-3-phosphoglycerate dehydrogenase.	AK243399	B6SKK1	Amino acid-binding ACT
Os08g0447500	Hypothetical conserved gene.	AK240682	A3BTN1	-
Os08g0448000	Similar to 4-coumarate--CoA ligase 1 (EC 6.2.1.12) (4CL 1) (4-coumaroyl-CoA synthase 1) (Clone 4	AK120964	Q9LL50	AMP-dependent synthetase/ligase
Os08g0448050	Hypothetical protein.	BT062457	longestORF	-
Os08g0448900	Conserved hypothetical protein.	CT832739	NP_001175602.1	-
Os08g0449500	Conserved hypothetical protein.	AK120389	B8BB90	-
Os08g0449901	Hypothetical protein.	BT085732	longestORF	-
Os08g0450100	Similar to Pectinesterase (EC 3.1.1.11) (Fragment).	AK058651	A2YVS3	Pectinesterase, catalytic
Os08g0450200	Similar to Pectin methylesterase (Fragment).	AK072779	A2YVS4	Pectinesterase, catalytic
Os08g0450800	Phosphatidylinositol-4-phosphate 5-kinase family protein.	AK102479	XP_002882874.1	Phosphatidylinositol-4-phosphate 5-kinase, core
Os08g0450900	SANT domain, DNA binding domain containing protein.	ab initio prediction	Q6ZLF1	SANT domain, DNA binding
Os08g0452200	Hypothetical protein.	AK100150	longestORF	-
Os08g0452500	Auxin responsive SAUR protein family protein.	AK106714	B6TUQ7	Auxin responsive SAUR protein
Os08g0453733	Non-protein coding transcript.	CT836006	NONE	-
Os08g0454000	Pathogenesis-related transcriptional factor/ERF, DNA-binding domain containing protein.	EU968979	Q6ZAG1	Pathogenesis-related transcriptional factor/ERF, DNA-binding
Os08g0455700	Similar to predicted protein.	AK240992 ,AK240990	NP_001061962.1	Exo70 exocyst complex subunit
Os08g0456600	Hypothetical protein.	AK059009	longestORF	-
Os08g0457300	Hypothetical conserved gene.	CT836013	Q6Z0P9	-
Os08g0457400	Similar to Avr9/Cf-9 induced kinase 1.	AK106955	B6ST24	Protein kinase, catalytic domain
Os08g0457901	Hypothetical genes.	ab initio prediction	NONE	-
Os08g0458200	Similar to MTD1.	AK061399	NP_001148816.1	-
Os08g0459100	Leucine-rich repeat, cysteine-containing containing protein.	AK121795	A3BTU1	-
Os08g0460000	Similar to Germin-like protein 1 precursor.	AK104729 ,AK065284	Q6ZBZ2	Germin
Os08g0460900	Conserved hypothetical protein.	ab initio prediction	NP_001061979.1	-
Os08g0461300	Cyclin-like F-box domain containing protein.	AK065651	A3BTV7	F-box domain, cyclin-like
Os08g0463500	Zinc finger, C2H2-type domain containing protein.	AK058457	A2YVZ8	Zinc finger, C2H2
Os08g0464950	Hypothetical gene.	AK241196	longestORF	-
Os08g0465000	Hypothetical conserved gene.	CT835984	Q6YPD0	-
Os08g0467201	Hypothetical protein.	BT086922	longestORF	-
Os08g0467300	Clathrin adaptor, phosphoinositide-binding, GAT-like domain containing protein.	AK105541	NP_001151341.1	ENTH/VHS
Os08g0467500	Abscisic acid and stress inducible (A22) gene.	AK100937	Q07764	TB2/DP1/HVA22-related protein
Os08g0469500	Similar to F-box domain containing protein.	AK109599	NP_001150287.1	-
Os08g0469600	Conserved hypothetical protein.	AK062647	B8BBK4	-
Os08g0471000	Similar to cDNA, clone: J065120N14, full insert sequence.	AK356002	B7F8M8	Heat shock factor
Os08g0471150	Hypothetical protein.	tplb0033f11 (Wheat FLcDNA)	longestORF	-
Os08g0471800	Remorin, C-terminal region domain containing protein.	AK105281	B9G1D0	Remorin, C-terminal
Os08g0471850	Hypothetical protein.	tplb0035l08 (Wheat FLcDNA)	longestORF	-
Os08g0471950	bZIP transcription factor TRAB1.	Q6ZDF3	Q6ZDF3	Basic-leucine zipper
Os08g0472400	Conserved hypothetical protein.	FP097825	Q6ZDE7	-
Os08g0472800	Cytochrome P450 family protein.	AK120757	Q6ZDE3	Cytochrome P450
Os08g0473200	Hypothetical protein.	tplb0043h07 (Wheat FLcDNA)	longestORF	-
Os08g0475400	Alpha/beta hydrolase fold-3 domain containing protein.	AK060706 ,AK073975	NP_001150584.1	Alpha/beta hydrolase fold-3
Os08g0476400	Similar to phosphoglycerate mutase gpmB.	AK241011	NP_001150922.1	Phosphoglycerate/bisphosphoglycerate mutase, active site
Os08g0477600	Conserved hypothetical protein.	AK069216	A3BU43	-
Os08g0477900	Helix-loop-helix DNA-binding domain containing protein.	EU968808	NP_001159035.1	Helix-loop-helix DNA-binding
Os08g0478566	Similar to Maturase K 2.	AY964640	C6ES29	-
Os08g0478800	Phosphoglucose isomerase (PGI) family protein.	AK107494	A3BU52	Phosphoglucose isomerase
Os08g0479400	Similar to Hydroxyproline-rich glycoprotein DZ-HRGP precursor.	AK109528	B6TE62	Homeobox domain, ZF-HD class
Os08g0480400	Similar to cupin, RmlC-type.	AK241844	NP_001147194.1	Cupin, RmlC-type
Os08g0480800	Similar to TaWIN2.	AK101599	Q84J55	14-3-3 protein
Os08g0480901	Non-protein coding transcript.	BT017754	NONE	-
Os08g0481000	Hypothetical conserved gene.	AK111069	Q6Z235	Pentatricopeptide repeat
Os08g0481100	Similar to SKIP interacting protein 3 (Fragment).	AK240733	B8Q889	-
Os08g0481500	Conserved hypothetical protein.	AK241875	NP_001062065.2	-
Os08g0481700	Hypothetical conserved gene.	CT835933	Q6Z230	-
Os08g0482025	Hypothetical protein.	BT009228	longestORF	-
Os08g0482300	Pistil-specific extensin-like protein domain containing protein.	AK107940	longestORF	-
Os08g0482600	Cupredoxin domain containing protein.	AK063639	A2YWC7	Blue
Os08g0482700	Cupredoxin domain containing protein.	AK060044	A2YWC8	Plastocyanin-like
Os08g0483100	Conserved hypothetical protein.	AK099821	A3BU75	-
Os08g0483600	Conserved hypothetical protein.	AK099543	B9G1G7	-
Os08g0483800	Hypothetical gene.	AK100866	longestORF	-
Os08g0483900	Helix-loop-helix DNA-binding domain containing protein.	AK107626	NP_001151793.1	Helix-loop-helix DNA-binding
Os08g0484100	Similar to predicted protein.	AK242582	A3BU81	Glycoside hydrolase, subgroup, catalytic domain
Os08g0484200	Zinc finger, RING/FYVE/PHD-type domain containing protein.	AK287991 ,AK241976	NP_001062080.1	Zinc finger, RING-type
Os08g0484300	Conserved hypothetical protein.	BT019110	C0P2Z7	-
Os08g0484450	Hypothetical protein.	tplb0055k03 (Wheat FLcDNA)	longestORF	-
Os08g0484500	Similar to Phospho-2-dehydro-3-deoxyheptonate aldolase 2.	AK106196	B6UAK5	DAHP synthetase, class II
Os08g0484533	Hypothetical protein.	tplb0022g15 (Wheat FLcDNA)	longestORF	-
Os08g0484633	Hypothetical protein.	tplb0031c07 (Wheat FLcDNA)	longestORF	-
Os08g0484800	Hypothetical conserved gene.	FP092392	Q6YTS7	-
Os08g0485000	Similar to PHI-1.	AK073011	NP_001150825.1	Phosphate-induced protein 1
Os08g0485500	Conserved hypothetical protein.	AK241136	A3BU93	-
Os08g0485600	Hypothetical conserved gene.	AK103243	B8BBU4	Zinc finger, C2H2
Os08g0485800	Barwin-related endoglucanase domain containing protein.	AK242586	NP_001148968.1	Expansin/pollen allergen, DPBB domain
Os08g0485900	Haloacid dehalogenase-like hydrolase domain containing protein.	AK110716	NP_568077.1	Haloacid dehalogenase-like hydrolase
Os08g0486100	Similar to Potential copper-transporting ATPase PAA1 (EC 3.6.3.4).	AK059217	XP_002304082.1	ATPase, P-type, H+ transporting proton pump
Os08g0486200	Similar to Splicing factor SC35.	AK103676	B6UG76	RNA recognition motif domain
Os08g0486233	Similar to EIL transcription factor.	CT835917	Q8W3L9	Ethylene insensitive 3-like protein, DNA-binding domain
Os08g0486300	Similar to P-type R2R3 Myb protein (Fragment).	BT033636	Q8S416	SANT domain, DNA binding
Os08g0486400	Hypothetical protein.	EU971393	longestORF	-
Os08g0486700	Conserved hypothetical protein.	AK241195	NP_001062097.1	-
Os08g0486750	Non-protein coding transcript.	AK363293	NONE	-
Os08g0486801	Non-protein coding transcript.	AK241869	NONE	-
Os08g0486867	Conserved hypothetical protein.	AK243168	Q75LB0	-
Os08g0486933	Similar to Peptide transporter-like protein.	ab initio prediction	Q6YS05	-
Os08g0487100	Similar to BZIP transcription factor BZI-2.	AK107150	NP_001152649.1	Basic-leucine zipper
Os08g0487500	Zinc finger, RING/FYVE/PHD-type domain containing protein.	AK066326	B6U108	Zinc finger, RING-type
Os08g0487700	Helix-loop-helix DNA-binding domain containing protein.	ab initio prediction	NP_001062102.1	Helix-loop-helix DNA-binding
Os08g0487800	Similar to Heat-shock protein precursor.	AK122102	Q43638	Heat shock protein Hsp90
Os08g0487850	Non-protein coding transcript.	BT017162	NONE	-
Os08g0489100	Hypothetical conserved gene.	ab initio prediction	Q6ZBR2	UDP-glucuronosyl/UDP-glucosyltransferase
Os08g0489300	Methyladenine glycosylase domain containing protein.	AK104597 ,AK059000	B4FL85	Methyladenine glycosylase
Os08g0490000	Helix-loop-helix DNA-binding domain containing protein.	AK100183	B6SVP6	Helix-loop-helix DNA-binding
Os08g0490100	Similar to PBF protein.	AK241364	Q1HFQ1	Zinc finger, Dof-type
Os08g0490300	RNA recognition motif, glycine rich protein domain containing protein.	AK066895	A2YWH8	RNA recognition motif domain
Os08g0490600	FAS1 domain containing protein.	AK108305	Q06IA2	FAS1 domain
Os08g0490800	Similar to Histone H2B.	FP097117	B9G1J0	Histone H2B
Os08g0490900	Similar to Histone H2B.2.	FP098904	C5XPC6	Histone H2B
Os08g0491000	Hypothetical conserved gene.	AK073673	B9G1J1	-
Os08g0491200	Serine/threonine protein kinase domain containing protein.	AK103656	B7EQ74	Protein kinase, catalytic domain
Os08g0491400	Interferon-related developmental regulator domain containing protein.	AK109684	A2YWI6	Interferon-related developmental regulator, N-terminal
Os08g0491700	Putative zinc finger CCCH domain-containing protein 57.	A3BUD2	A3BUD2	Zinc finger, CCCH-type
Os08g0492000	Similar to Glutathione transporter.	AK100814	XP_002323113.1	Tetrapeptide transporter, OPT1/isp4
Os08g0492400	C2 calcium-dependent membrane targeting domain containing protein.	AK241274	B9G1J5	C2 calcium-dependent membrane targeting
Os08g0494000	Harpin-induced 1 domain containing protein.	AK110788	NP_001148671.1	Late embryogenesis abundant protein, LEA-14
Os08g0494100	Transcription factor, MADS-box domain containing protein.	ab initio prediction	Q6Z5F8	Transcription factor, MADS-box
Os08g0494200	Conserved hypothetical protein.	AK100830	B8BC24	-
Os08g0494300	Copine domain containing protein.	AK066150	NP_001147447.1	Zinc finger, RING-type
Os08g0494350	Pentatricopeptide repeat domain containing protein.	ab initio prediction	B8BC26	Pentatricopeptide repeat
Os08g0494375	Conserved hypothetical protein.	ab initio prediction	B8B4W8	-
Os08g0494400	Conserved hypothetical protein.	AK065332	B9G1K3	-
Os08g0495300	Similar to cDNA clone:J013000K10, full insert sequence.	AK287721	B9G1K9	Protein of unknown function DUF3615
Os08g0495500	Kelch related domain containing protein.	AK100472	NP_001150189.1	BTB/POZ-like
Os08g0496100	Hypothetical conserved gene.	EU952779	Q6Z5C4	-

Os08g0496600	Hypothetical conserved gene.	FP095819	Q7F8V1	-
Os08g0498100	Similar to Caffeoyl-CoA O-methyltransferase 2 (EC 2.1.1.104) (Trans-caffeoyl-CoA 3-O-methyltrans	AK071482 ,AK104326 ,AK10480	B4G080	O-methyltransferase, family 3
Os08g0498400	Similar to Caffeoyl-CoA O-methyltransferase (Fragment).	AK065515	A5A5Y9	O-methyltransferase, family 3
Os08g0499000	Conserved hypothetical protein.	AK107539	A2YWN4	-
Os08g0499100	ATPase, AAA+ type, core domain containing protein.	AK243638	XP_002864442.1	NB-ARC
Os08g0499250	Hypothetical conserved gene.	EU971673	B6U358	-
Os08g0499300	WRKY transcription factor 30.	AK065518	B8BC43	DNA-binding WRKY
Os08g0499400	Conserved hypothetical protein.	AK241142	NP_001175641.1	-
Os08g0499700	Conserved hypothetical protein.	AK119271	B9G1M7	-
Os08g0499800	Pentatricopeptide repeat domain containing protein.	AK102014	Q2L3A2	Pentatricopeptide repeat
Os08g0500400	Hypothetical protein.	AK111024	longestORF	-
Os08g0500700	Similar to Heat shock protein 82.	AK102426	E0X6U7	Heat shock protein Hsp90
Os08g0500900	Similar to Phosphoribosylglycinamide formyltransferase, chloroplast precursor (EC 2.1.2.2) (GART)	AK102314	XP_002334132.1	Phosphoribosylglycinamide formyltransferase, active site
Os08g0501600	Similar to Wall associated kinase.	DQ177499	Q2L3D5	Protein kinase, catalytic domain
Os08g0501800	Non-protein coding transcript.	EU955321	NONE	-
Os08g0501900	Non-protein coding transcript.	AK063130	NONE	-
Os08g0502101	Conserved hypothetical protein.	AK241735	NP_001175644.1	-
Os08g0502600	EF hand domain containing protein.	AK107730	A2YWR5	Calcium-binding EF-hand
Os08g0503600	Hypothetical protein.	AK119300	longestORF	-
Os08g0503700	Sodium/sulphate symporter family protein.	AK072183	NP_001148320.1	Sodium/sulphate symporter
Os08g0504000	Similar to WIP5 protein.	AK287566	A6N8R9	Zinc finger, C2H2
Os08g0504200	Similar to H0315A08.1 protein.	FP099421	Q01I63	Ribonuclease H1, N-terminal
Os08g0506700	Helix-loop-helix DNA-binding domain containing protein.	AK100106	NP_001148741.1	Helix-loop-helix DNA-binding
Os08g0506850	Hypothetical gene.	CT836002	longestORF	-
Os08g0509400	Non-protein coding transcript.	AK060593	NONE	-
Os08g0509600	Squamosa promter-binding-like transcription factor, Regulation of branching in panicles and vegetat	AK107191	Q7EXZ2,B7F043	Transcription factor, SBP-box
Os08g0511800	Conserved hypothetical protein.	AK063264	A2YWY7	-
Os08g0519501	Hypothetical gene.	AK109201	longestORF	-
Os08g0524600	Similar to 60S ribosomal protein L32.	P51421	P51421	Ribosomal protein L32e
Os08g0525000	Ras GTPase family protein.	AK103220	NP_001152371.1	Small GTPase superfamily
Os08g0525050	Non-protein coding transcript.	BT084300	NONE	-
Os08g0533300	Similar to ACR5.	AK287480	B6SYQ4	Amino acid-binding ACT
Os08g0533450	Hypothetical protein.	tplb0061b06 (Wheat FLcDNA)	longestORF	-
Os08g0535000	Similar to cation cation antiporter.	AK066324	NP_001148426.1	Vesicle transport protein, Use1
Os08g0536333	Non-protein coding transcript.	CT835951	NONE	-
Os08g0543650	Non-protein coding transcript.	BT019236	NONE	-
Os08g0547600	Similar to SRS7 (SHI-RELATED SEQUENCE 7).	AK335496	NP_001031069.1	Zinc finger, lateral root primordium type 1
Os08g0557400	Protein tyrosine phosphatase-like protein.	AK060067	Q84PD6	Protein-tyrosine phosphatase/arsenate reductase
Os08g0557500	Similar to predicted protein.	AK111941	XP_002877347.1	Peptidyl-prolyl cis-trans isomerase, cyclophilin-type
Os08g0557600	Similar to Monodehydroascorbate reductase (EC 1.6.5.4) (MDAR) (Ascorbate free radical reductase	AK102459	Q9SXX0	Pyridine nucleotide-disulphide oxidoreductase, NAD-binding domain
Os08g0557700	Similar to Histidine-containing phosphotransfer protein.	AK061111	Q6PT60	Signal transduction histidine kinase, phosphotransfer
Os08g0558800	Similar to Ribosomal protein.	AK122095	B8B9K6	Ribosomal protein L1
Os08g0559501	Conserved hypothetical protein.	CT837577	NP_001175701.1	-
Os08g0559800	Hypothetical conserved gene.	AK242197	B9FYJ9	-
Os08g0564300	Hypothetical conserved gene.	AK106260	Q8GU78	ABC transporter, transmembrane domain
Os09g0113500	Similar to H0315A08.1 protein.	FP099421	Q01I63	Ribonuclease H1, N-terminal
Os09g0114500	Hypothetical protein.	AK059015	longestORF	-
Os09g0114566	Non-protein coding transcript.	X06284	NONE	-
Os09g0115900	Non-protein coding transcript.	AK060240	NONE	-
Os09g0116800	Similar to DNA binding protein.	EU975895	B6TCD9	Transcription factor, MADS-box
Os09g0119600	UDP-glucuronosyl/UDP-glucosyltransferase family protein.	AK059022	C4MF39	UDP-glucuronosyl/UDP-glucosyltransferase
Os09g0123400	Hypothetical conserved gene.	EU941192	Q6K269	-
Os09g0124232	Ankyrin repeat domain containing protein.	ab initio prediction	A3BW61	Ankyrin repeat
Os09g0125400	Hypothetical conserved gene.	ab initio prediction	Q6K385	-
Os09g0127800	Similar to cDNA clone:J023075G08, full insert sequence.	AK058402	B7EQ85	Coatomer, WD associated region
Os09g0129301	Non-protein coding transcript.	AK058758	NONE	-
Os09g0129800	Conserved hypothetical protein.	ab initio prediction	Q6ERA9	-
Os09g0133800	Conserved hypothetical protein.	AK069372	A2YYQ1	-
Os09g0134900	Hypothetical gene.	AK063225	longestORF	-
Os09g0240500	Similar to Sulfate transporter 4.1.	AK287664	B6SXI4	Sulphate transporter/antisigma-factor antagonist STAS
Os09g0241100	Similar to nucleotide binding.	AK109602	NP_189852.2	WD40 repeat
Os09g0241200	Hypothetical protein.	AK120155	longestORF	-
Os09g0241550	Similar to F-box domain containing protein, expressed.	ab initio prediction	D8L9K3	-
Os09g0242000	Similar to F-box domain containing protein, expressed.	ab initio prediction	D8L9K3	-
Os09g0247700	Similar to Zinc finger, ZZ-type%3B Zinc finger, C2H2-type.	AK059400	A2Q2P2	-
Os09g0251400	Non-protein coding transcript.	AK059379	NONE	-
Os09g0253775	Hypothetical protein.	AK372466	longestORF	-
Os09g0264011	Conserved hypothetical protein.	AK372466	Q6K2H9	-
Os09g0273600	Hypothetical gene.	CT835836	longestORF	-
Os09g0310800	Similar to Glycosyl hydrolase family 9 protein, expressed.	AK067555	Q2QSI0	-
Os09g0311600	Similar to NBS-LRR type resistance protein (Fragment).	AK102321	Q69KE4	Disease resistance protein
Os09g0325140	Hypothetical conserved gene.	ab initio prediction	D7UPN3	-
Os09g0334600	Similar to Cytochrome b5.	AK335015	B6UG94	Cytochrome b5
Os09g0343600	Non-protein coding transcript.	AK061057	NONE	-
Os09g0365450	Similar to DNA binding protein.	AK367679	NP_001146992.1	-
Os09g0376900	Similar to Potassium transporter 13 (AtPOT13) (AtKT5).	AK070831	Q6H4R6	K+ potassium transporter
Os09g0380400	Conserved hypothetical protein.	AK073073 ,AK063909	B8BF06	-
Os09g0386600	Conserved hypothetical protein.	AK064522	B9G3D2	-
Os09g0391501	Hypothetical gene.	CT835895	longestORF	-
Os09g0392400	Similar to Pleiotropic drug resistance protein 13.	AK072152	Q8S628	-
Os09g0395300	GARP DNA-binding protien, Leaf polarity modeling	FJ268748	Q0J235	-
Os09g0396300	Peptidase C15, pyroglutamyl peptidase I family protein.	AK060069 ,AK105043	NP_001149461.1	Peptidase C15, pyroglutamyl peptidase I
Os09g0396900	Protein of unknown function DUF125, transmembrane family protein.	AK071589	NP_001150466.1	Domain of unknown function DUF125, transmembrane
Os09g0397100	Conserved hypothetical protein.	AK243387	NP_001063102.1	-
Os09g0397200	Conserved hypothetical protein.	AK241559	NP_001063103.2	-
Os09g0397400	Protein of unknown function DUF231, plant domain containing protein.	AK063997	Q6ERD8	Domain of unknown function DUF231, plant
Os09g0397700	Glutathione-dependent formaldehyde-activating, GFA family protein.	AK111009	B6T550	Glutathione-dependent formaldehyde-activating family, GFA
Os09g0397800	Hypothetical gene.	AK062361	longestORF	-
Os09g0397900	Similar to FEG protein.	AK101306	Q6E435	Beta-lactamase-like
Os09g0397950	Hypothetical protein.	tplb0030m03 (Wheat FLcDNA)	longestORF	-
Os09g0400200	Similar to Cinnamyl alcohol dehydrogenase (EC 1.1.1.195).	AK072000	longestORF	-
Os09g0400300	Similar to Cinnamyl alcohol dehydrogenase (EC 1.1.1.195).	AK067085	Q2HZE4	Alcohol dehydrogenase superfamily, zinc-type
Os09g0400350	Conserved hypothetical protein.	EU947353	NP_001173247.1	-
Os09g0405900	Similar to Uncharacterized protein OsI_030282.	AK288278	A2Z139	-
Os09g0406000	Similar to Uncharacterized protein OsI_031781.	AK120862	A2Z5D8	-
Os09g0406100	Similar to Uncharacterized protein OsI_030282.	AK288278	A2Z139	-
Os09g0406300	Similar to Uncharacterized protein OsI_031781.	AK288278	A2Z5D8	-
Os09g0406500	Similar to Uncharacterized protein OsI_030282.	AK288278	A2Z139	-
Os09g0444600	Non-protein coding transcript.	AK059315	NONE	-
Os09g0445700	ATPase, AAA+ type, core domain containing protein.	ab initio prediction	B8BCH8	ATPase, AAA+ type, core
Os09g0447000	Homeodomain-related domain containing protein.	AY559046	Q67UE2	Homeobox
Os09g0473050	Hypothetical protein.	AK372466	longestORF	-
Os09g0473400	Peptidase C12, ubiquitin carboxyl-terminal hydrolase 1 domain containing protein.	EU976366	NP_192633.2	Plant organelle RNA recognition domain
Os09g0473502	Hypothetical gene.	CT835876	longestORF	-
Os09g0473600	Hypothetical conserved gene.	EU973630	B9G446	-
Os09g0474000	bZIP transcription factor, bZIP-1 domain containing protein.	AK108319	B8BCQ0	Basic-leucine zipper
Os09g0480532	Conserved hypothetical protein.	ab initio prediction	Q69QS1	-
Os09g0526300	Similar to Homeobox protein GLABRA2 (Homeobox-leucine zipper protein ATHB-10) (HD-ZIP pro	AB101649	Q7Y0V7	Homeobox
Os09g0526500	C2 calcium/lipid-binding region, CaLB domain containing protein.	CT841705	NP_001175951.1	-
Os09g0526600	Similar to Isoform 2 of Heat stress transcription factor B-2c.	AK106525	Q652B0-2	Heat shock factor
Os09g0526650	Conserved hypothetical protein.	tplb0033f11 (Wheat FLcDNA)	B8AME9	-
Os09g0526700	Similar to UDP-glucose 4-epimerase (EC 5.1.3.2) (Galactowaldenase) (UDP-galactose 4-epimerase)	AK073610	A6MD13	NAD-dependent epimerase/dehydratase
Os09g0526800	NC domain containing protein.	AK105121 ,AK060048	A6MD14	NC
Os09g0527100	Similar to RNA-binding protein.	AK073499	NP_001063730.1	RNA recognition motif domain
Os09g0527500	Similar to MEG5.	AK110546	B7EM90	RNA recognition motif domain

Os09g0527600	Similar to GTP-binding protein (Fragment).	AK111543	A6MD16	Small GTPase superfamily
Os09g0527650	Hypothetical protein.	BT084300	longestORF	-
Os09g0527700	Similar to Auxin-induced protein IAA4.	AK111128	Q652A1	AUX/IAA protein
Os09g0528000	Hypothetical conserved gene.	AK060588	B9G4P1	Protein of unknown function DUF3490
Os09g0528050	Hypothetical protein.	tplb0030a08 (Wheat FLcDNA)	longestORF	-
Os09g0528100	Similar to 30S ribosomal protein S31, chloroplast (Fragment).	AK059096	B6UIF3	-
Os09g0528200	Similar to Homeobox-leucine zipper protein HOX6.	AK103160	Q651Z5	Helix-turn-helix motif, lambda-like repressor
Os09g0528500	Hypothetical protein.	tplb0044m18 (Wheat FLcDNA)	longestORF	-
Os09g0528700	Similar to Cytochrome p450 (CYP78A9).	ab initio prediction	B6ST44	Cytochrome P450
Os09g0528750	Non-protein coding transcript.	CT835864	NONE	-
Os09g0542600	Similar to RING-H2 finger protein ATL1I.	AK060109	B8BE25	Zinc finger, RING-type
Os09g0542800	Pentatricopeptide repeat domain containing protein.	AK240855	XP_002890377.1	Pentatricopeptide repeat
Os09g0542900	Conserved hypothetical protein.	AK073400	B8BE26	-
Os09g0543400	Ornithine decarboxylase (EC 4.1.1.17).	BT042764	Q01N22	Ornithine/DAP/Arg decarboxylase
Os09g0545250	Hypothetical protein.	EU976224	GeneMark	-
Os09g0545500	Similar to SAUR55 - auxin-responsive SAUR family member.	EU975305	A3C156	Auxin responsive SAUR protein
Os09g0545801	Non-protein coding transcript.	CT835855	NONE	-
Os09g0545901	Non-protein coding transcript.	FP098421	NONE	-
Os09g0545999	Hypothetical protein.	FP098421	longestORF	-
Os09g0546200	Similar to auxin induced protein.	CT837633	NP_001105462.1	Auxin responsive SAUR protein
Os09g0546400	Similar to Auxin induced protein.	AK072378	longestORF	-
Os09g0546700	Similar to SAUR55 - auxin-responsive SAUR family member.	EU975305	B6TXI9	Auxin responsive SAUR protein
Os09g0546900	Similar to Auxin induced protein.	AK062365	A2Z3Q4	Auxin responsive SAUR protein
Os09g0547033	Similar to SAUR44 - auxin-responsive SAUR family member.	AK287456	NP_001149574.1	Auxin responsive SAUR protein
Os09g0547067	Conserved hypothetical protein.	FP098421	A3C166	-
Os09g0547100	Similar to Auxin induced protein.	AK058556	NP_001105462.1	Auxin responsive SAUR protein
Os09g0547200	Protein of unknown function DUF292, eukaryotic domain containing protein.	AK073078	B8BE42	Domain of unknown function DUF292, eukaryotic
Os09g0548083	Non-protein coding transcript.	tplb0058a02 (Wheat FLcDNA)	NONE	-
Os09g0548200	Peptidoglycan-binding Lysin subgroup domain containing protein.	AK060664	Q651P3	Peptidoglycan-binding Lysin subgroup
Os09g0549425	Conserved hypothetical protein.	AK374610	F2EEN4	-
Os09g0549500	Nucleotide-binding, alpha-beta plait domain containing protein.	FP096667	NP_001150292.1	RNA recognition motif domain
Os09g0551100	Hypothetical protein.	AK107540	longestORF	-
Os09g0551251	Similar to H0105C05.10 protein.	ab initio prediction	Q25A21	Protein kinase, catalytic domain
Os09g0552800	No apical meristem (NAM) protein domain containing protein.	AK361030	B9G4Y8	No apical meristem
Os09g0552900	Similar to NAC domain protein, IPR003441.	EU942489	XP_002318332.1	No apical meristem
Os09g0553100	Similar to Histone H4.	CU406630	B6T0P4	Histone H4
Os09g0553200	UDPase, Pollen mother cell (PMC) meiosis, Pollen development	DQ395328 ,AB062606 ,AK11919	A3QQQ3,Q93X08	-
Os09g0553250	Hypothetical gene.	BT016879	longestORF	-
Os09g0553700	Similar to PRLI-interacting factor A (Fragment).	AK070189	B6TGN5	-
Os09g0553750	Hypothetical gene.	EU970031	longestORF	-
Os09g0554200	Zinc finger, RING/FYVE/PHD-type domain containing protein.	AK108361	NP_001146938.1	Zinc finger, RING-type
Os09g0555400	Pentatricopeptide repeat domain containing protein.	AK073402	A2Z3X5	Pentatricopeptide repeat
Os09g0555450	Conserved hypothetical protein.	EU950356	F2EDP0	-
Os09g0555700	Zinc finger, C2H2-type domain containing protein.	AK061264	Q5UDB7	Zinc finger, C2H2
Os09g0556300	Non-protein coding transcript.	AK105452	NONE	-
Os09g0556800	Conserved hypothetical protein.	AK101051	A2Z3Y8	-
Os09g0557600	Non-protein coding transcript.	AK058533	NONE	-
Os09g0557900	Similar to Protein phosphatase 2C gamma isoform (EC 3.1.3.16) (PP2C-gamma) (Protein phosphata	AK108807	NP_001149513.1	Plastocyanin-like
Os09g0565400	Lipoprotein, type 6 family protein.	AK067821	B9G549	-
Os09g0567300	Similar to Monodehydroascorbate reductase (EC 1.6.5.4) (MDAR) (Ascorbate free radical reductase	AK071558	Q9XFZ3	Pyridine nucleotide-disulphide oxidoreductase, NAD-binding domain
Os09g0567400	Similar to Histidine-containing phosphotransfer protein.	AK072521	Q6VAK4	Signal transduction histidine kinase, phosphotransfer
Os09g0568100	Conserved hypothetical protein.	FP092518	B9G564	-
Os09g0568400	Similar to 60S ribosomal protein L40 (CEP52).	AK059386	Q7XYD4	Ribosomal protein L40e
Os09g0568500	Germin family protein.	AK108987	Q84XR7	Germin
Os09g0568700	RmlC-like jelly roll fold domain containing protein.	AK109010	Q84XR7	Germin
Os09g0568900	Similar to predicted protein.	AK059442	XP_002879140.1	-
Os09g0569000	Hypothetical gene.	AK108627	longestORF	-
Os09g0569100	Haloacid dehalogenase-like hydrolase domain containing protein.	AK071860	B4FI81	Haloacid dehalogenase-like hydrolase
Os09g0569200	Similar to Beta-amylase (EC 3.2.1.2) (1,4-alpha-D-glucan maltohydrolase).	AK070300	B6SVZ0	Glycoside hydrolase, family 14B, plant
Os09g0569300	Similar to calmodulin-binding heat-shock protein.	AK069587	NP_001149987.1	Lipase, class 3
Os09g0569450	Conserved hypothetical protein.	AK242543	NP_001175993.1	-
Os09g0569700	Conserved hypothetical protein.	AK108778	NP_001063979.1	-
Os09g0569780	Conserved hypothetical protein.	AK241041	NP_001175963.1	-
Os09g0569800	Serine/threonine protein kinase-related domain containing protein.	ab initio prediction	NP_001063980.1	Protein kinase, catalytic domain
Os09g0570000	Serine/threonine protein kinase-related domain containing protein.	AK058797	Q2HTK4	Protein kinase, catalytic domain
Os09g0570100	Protein kinase, catalytic domain domain containing protein.	AK061135	B9G572	Protein kinase, catalytic domain
Os09g0570150	Hypothetical conserved gene.	EU953102	B6SK37	-
Os09g0570300	Similar to Short-chain dehydrogenase Tic32.	AK061532	B4FKX6	Short-chain dehydrogenase/reductase SDR
Os09g0570400	Non-protein coding transcript.	AK061710	NONE	-
Os09g0570500	Zinc finger, RING-type domain containing protein.	AK069259	B9G575	Zinc finger, RING-type
Os09g0570600	Non-protein coding transcript.	AK105456	NONE	-
Os09g0570850	Similar to Histone H2B.	ab initio prediction	B6SGC3	Histone H2B
Os09g0571000	Protein of unknown function DUF966 family protein.	ab initio prediction	Q651B3	Protein of unknown function DUF966
Os09g0571033	Conserved hypothetical protein.	AK241104	NP_001175997.1	-
Os09g0571100	Pectin lyase fold/virulence factor domain containing protein.	AK106869	C5X6M1	Pectinesterase, catalytic
Os09g0571200	C2 domain containing protein.	AK062604	B6TT83	C2 calcium-dependent membrane targeting
Os09g0572000	Pathogenesis-related transcriptional factor and ERF domain containing protein.	AK241281	XP_002306842.1	Pathogenesis-related transcriptional factor/ERF, DNA-binding
Os09g0572550	Hypothetical gene.	AK243102	longestORF	-
Os09g0572600	Similar to Receptor protein kinase-like protein.	AK070226	NP_188689.1	Protein kinase, catalytic domain
Os09g0572950	Non-protein coding transcript.	tplb0034e16 (Wheat FLcDNA)	NONE	-
Os09g0573000	Pentatricopeptide repeat domain containing protein.	AK073399	B9G584	Pentatricopeptide repeat
Os09g0573100	Ubiquitin domain containing protein.	AK121232	XP_002881393.1	Uncharacterised domain SAYSvFN
Os10g0122100	Hypothetical protein.	AK372466	longestORF	-
Os10g0141300	Similar to Protein kinase domain containing protein.	ab initio prediction	Q7XGY5	Protein kinase, catalytic domain
Os10g0141400	Similar to 26S proteasome non-ATPase regulatory subunit 4.	BT063362	NP_001150010.1	von Willebrand factor, type A
Os10g0141900	Queuine/other tRNA-ribosyltransferase family protein.	AK106467	Q948C4	Queuine/other tRNA-ribosyltransferase
Os10g0142100	Similar to Zinc finger, C3HC4 type family protein, expressed.	AK058523	Q10A50	Zinc finger, RING-type
Os10g0142500	Cyclin-like F-box domain containing protein.	AK072788	Q948C2	F-box domain, cyclin-like
Os10g0144000	Conserved hypothetical protein.	EU945087	NP_001064152.1	-
Os10g0167401	Non-protein coding transcript.	BT084318	NONE	-
Os10g0180600	Conserved hypothetical protein.	ab initio prediction	NP_001176047.1	-
Os10g0186200	Pectinesterase inhibitor domain containing protein.	EU971858	NP_001176048.1	Pectinesterase inhibitor
Os10g0192301	Non-protein coding transcript.	FP100159	NONE	-
Os10g0208900	Hypothetical conserved gene.	AK109751	Q53NH3	-
Os10g0339650	Non-protein coding transcript.	AK109165	NONE	-
Os10g0340950	Non-protein coding transcript.	EU941912	NONE	-
Os10g0351414	Endonuclease/exonuclease/phosphatase domain containing protein.	tplb0014n19 (Wheat FLcDNA)	NP_001057661.2	Endonuclease/exonuclease/phosphatase
Os10g0355800	Similar to ATP synthase CF1 beta subunit.	ab initio prediction	NP_039390.1	ATPase, F1/V1/A1 complex, alpha/beta subunit, nucleotide-binding domain
Os10g0356000	Similar to ribulose-1,5-bisphosphate carboxylase/oxygenase large subunit.	ab initio prediction	NP_039391.1	Ribulose bisphosphate carboxylase, large subunit, C-terminal
Os10g0390100	Non-protein coding transcript.	AK111272	NONE	-
Os10g0410650	Hypothetical gene.	BT018461	longestORF	-
Os10g0411500	IQ calmodulin-binding region domain containing protein.	AK072572	B8BGS6	-
Os10g0411600	Hypothetical protein.	AK108669	longestORF	-
Os10g0411650	Non-protein coding transcript.	EU947431	NONE	-
Os10g0411700	Similar to S28 ribosomal protein (Fragment).	AK058209 ,AK121099	Q7X9K4	-
Os10g0412100	Similar to Endonuclease/Exonuclease/phosphatase family protein, expressed.	AK102038	Q10R98	Endonuclease/exonuclease/phosphatase
Os10g0413400	Similar to Glycerol-3-phosphate acyltransferase 6 (EC 2.3.1.15) (AtGPAT6).	BT039538	NP_001176155.1	Phospholipid/glycerol acyltransferase
Os10g0429600	Similar to BTB/POZ domain containing protein.	AK369602	Q7XEA4	BTB/POZ-like
Os10g0430800	Conserved hypothetical protein.	AK111261	A3C4Z6	-
Os10g0432001	Hypothetical gene.	EU943065	longestORF	-
Os10g0432200	Conserved hypothetical protein.	AK106572	Q337X2	-
Os10g0432300	Non-protein coding transcript.	AK111369	NONE	-
Os10g0432725	Similar to cDNA, clone: J075155G16, full insert sequence.	ab initio prediction	Q337W9	-
Os10g0434200	Similar to BTB/POZ domain containing protein.	AK369602	Q337W6	BTB/POZ-like
Os10g0438000	Similar to Potassium channel tetramerisation domain-containing protein 9.	ab initio prediction	B6TKK7	BTB/POZ-like

Os10g0438800	Similar to RING-H2 finger protein ATL2E.	AK063228	NP_001151998.1	Zinc finger, RING-type
Os10g0439333	Similar to BTB/POZ domain containing protein.	AK376276	Q7XE33	BTB/POZ-like
Os10g0440050	Non-protein coding transcript.	BT009415	NONE	-
Os10g0457000	Hypothetical conserved gene.	AK061041	B9G629	RNA recognition motif domain
Os10g0459300	Exostosin-like family protein.	tplb0047d21 (Wheat FLcDNA)	NP_001150032.1	Exostosin-like
Os10g0464050	Non-protein coding transcript.	EU969129	NONE	-
Os10g0468250	Putative harbinger transposase-derived nuclease domain containing protein.	AK335074	Q8S819	Putative harbinger transposase-derived nuclease
Os10g0468301	Hypothetical conserved gene.	AK106587	Q60DP5	Myb/SANT-like domain
Os10g0472501	Hypothetical conserved gene.	tplb0015l09 (Wheat FLcDNA)	Q9FW60	-
Os10g0478350	Hypothetical protein.	EU971393	longestORF	-
Os10g0479800	Protein of unknown function DUF1336 domain containing protein.	AK107572	NP_180399.2	Domain of unknown function DUF1336
Os10g0488400	Protein of unknown function DUF761, plant domain containing protein.	AK108465	Q8W5I9	Protein of unknown function DUF761, plant
Os10g0492000	Conserved hypothetical protein.	AK064354	B9G6E3	-
Os10g0492101	Hypothetical conserved gene.	CT841749	Q9FWV2	-
Os10g0492200	Glycosyltransferase AER61, uncharacterized domain containing protein.	AK109476	NP_001105165.1	Glycosyltransferase AER61, uncharacterised
Os10g0493600	Similar to Alpha-galactosidase (Fragment).	AK058373	O04944	Glycosyl hydrolase, family 13, all-beta
Os10g0493850	Hypothetical protein.	tplb0029b01 (Wheat FLcDNA)	longestORF	-
Os10g0493900	Protein of unknown function DUF6, transmembrane domain containing protein.	AK071193	XP_002882247.1	Drug/metabolite transporter
Os10g0494000	Protein of unknown function DUF789 family protein.	AK106067 ,AK071373	A2Z8W3	Protein of unknown function DUF789
Os10g0495050	Non-protein coding transcript.	EU946286	NONE	-
Os10g0497500	K Homology, type 1, subgroup domain containing protein.	AK107213	A3C631	K Homology
Os10g0497550	Non-protein coding transcript.	EU946286	NONE	-
Os10g0499600	Similar to predicted protein.	AK107549	Q8LNG9	-
Os10g0503550	Non-protein coding transcript.	CT835611	NONE	-
Os10g0504650	Similar to nonspecific lipid-transfer protein.	ab initio prediction	Q7G2C5	Plant lipid transfer protein/seed storage/trypsin-alpha amylase inhibitor
Os10g0511800	Similar to Plus-3 domain containing protein, expressed.	EU942983	Q7XCX5	SWIB/MDM2 domain
Os10g0516500	Conserved hypothetical protein.	AK059055	Q94HZ3	-
Os10g0516551	Hypothetical protein.	BT088333	longestORF	-
Os10g0518150	Non-protein coding transcript.	BT016918	NONE	-
Os10g0518200	Cytochrome b5 domain containing protein.	AK058780	B6SNI9	Cytochrome b5
Os10g0524300	Peptidoglycan-binding lysin domain domain containing protein.	AK063244	NP_001150468.1	Peptidoglycan-binding lysin domain
Os10g0524400	Similar to Phospholipase D beta 2.	AK121075	B9G6P8	C2 calcium-dependent membrane targeting
Os10g0524766	Hypothetical gene.	EU941347	longestORF	-
Os10g0525000	Similar to Cytochrome P450 family protein, expressed.	AK105884	Q109D2	Cytochrome P450
Os10g0525101	Hypothetical gene.	BT065154	longestORF	-
Os10g0525301	Hypothetical gene.	BT065154	longestORF	-
Os10g0527601	Glutathione S-transferase, C-terminal-like domain containing protein.	ab initio prediction	Q6WSC2	Glutathione S-transferase, N-terminal
Os10g0528900	Similar to Tau class GST protein 3.	AK059726	Q6WSC2	Glutathione S-transferase, N-terminal
Os10g0529400	Similar to Glutathione S-transferase GSTU6.	AK102196	B6U486	Glutathione S-transferase, N-terminal
Os10g0529450	Hypothetical protein.	AK364077	longestORF	-
Os10g0530300	Similar to Glutathione S-transferase GST 42 (Fragment).	ab initio prediction	Q9FQ97	Glutathione S-transferase, C-terminal
Os10g0530800	Hypothetical protein.	AK111429	longestORF	-
Os10g0530900	Similar to Glutathione S-transferase GST 30 (EC 2.5.1.18).	AK103699 ,AK059760	Q8RW01	Glutathione S-transferase, N-terminal
Os10g0532100	Similar to Amine oxidase, flavin-containing.	AK065403	Q336Y0	Amine oxidase
Os10g0532150	Similar to Amine oxidase, flavin-containing family protein, expressed.	AK372474	Q336Y0	-
Os10g0534400	Similar to E4 protein (Fragment).	AK105216	B8BI06	-
Os10g0534500	Similar to Resistance protein candidate (Fragment).	AK070172	Q8LN27	Protein kinase, catalytic domain
Os10g0534600	Hypothetical protein.	AK103845	longestORF	-
Os10g0539900	Similar to Hexose transporter.	AK059705	B6U6U2	General substrate transporter
Os10g0540100	Pentatricopeptide repeat domain containing protein.	AK074016	Q9FRJ8	Pentatricopeptide repeat
Os10g0542100	Plant EC metallothionein-like protein, family 15 protein.	AK063504 ,AK102405	Q109B0	Plant EC metallothionein-like protein, family 15
Os10g0542800	Similar to ATP binding protein.	AK121234	NP_001151339.1	Tetratricopeptide-like helical
Os10g0543800	Similar to Glutathione S-transferase GST 11 (EC 2.5.1.18).	CT836577	NP_001104983.1	Glutathione S-transferase, N-terminal
Os10g0544933	Non-protein coding transcript.	EU943254	NONE	-
Os10g0544966	Hypothetical protein.	tplb0043b08 (Wheat FLcDNA)	longestORF	-
Os10g0545000	Similar to magnesium transporter CorA-like family protein.	AK069472	NP_567076.1	Fibronectin-attachment
Os10g0545100	Similar to predicted protein.	AK287840	B9G1Z7	Uncharacterised conserved protein UCP014543
Os10g0545200	Similar to 4,4-dimethyl-sterol C4-methyl-oxidase (Fragment).	AK121804	B4FN64	Fatty acid hydroxylase
Os10g0545300	Zinc finger, CCHC retroviral-type domain containing protein.	AK065156 ,AK121559	NP_001149324.1	Zinc finger, CCHC-type
Os10g0545700	Similar to Rhodanese-like protein.	AK073024	Q66T88	Rhodanese-like
Os10g0546400	Conserved hypothetical protein.	AK069919	B9G6V0	-
Os10g0546600	Similar to Chloroplast carotenoid epsilon-ring hydroxylase.	AK065689	NP_190881.2	Cytochrome P450
Os10g0546900	Similar to Zinc finger, C3HC4 type family protein, expressed.	AK368666	Q109A4	Zinc finger, RING-type
Os10g0547200	Harpin-induced 1 domain containing protein.	AK060129	A2Z9Y3	Late embryogenesis abundant protein, LEA-14
Os10g0549500	Similar to Branched-chain-amino-acid aminotransferase.	AK330986	A2ZA03	Aminotransferase, class IV
Os10g0550066	Hypothetical conserved gene.	BT067999	XP_002466024.1	Mediator complex, subunit Med12
Os10g0550100	Conserved hypothetical protein.	AK106583	Q94LW0	-
Os10g0550725	Hypothetical gene.	AK240685	longestORF	-
Os10g0550750	Protein of unknown function DUF3527 domain containing protein.	AK375090	B9G6W8	Protein of unknown function DUF3527
Os10g0551100	Conserved hypothetical protein.	AK106659	Q9FWN4	-
Os10g0551200	Similar to Scl1 protein (Fragment).	AK106239	Q7XCD7	Transcription factor GRAS
Os10g0551700	Plant lipid transfer protein and hydrophobic protein, helical domain containing protein.	AK107851	A2ZA19	Plant lipid transfer protein/seed storage/trypsin-alpha amylase inhibitor
Os10g0551800	Root-specific protein (RCc2 protein).	L27209	A2ZA20	Plant lipid transfer protein/seed storage/trypsin-alpha amylase inhibitor
Os10g0553900	Hypothetical conserved gene.	AK060446	A2ZA32	Protein of unknown function DUF863, plant
Os10g0554050	Non-protein coding transcript.	tplb0022l14 (Wheat FLcDNA)	NONE	-
Os10g0554100	Similar to Oxysterol-binding protein.	AK071048	C3SAC1	Oxysterol-binding protein
Os10g0557133	Similar to H0315A08.1 protein.	FP099421	Q01I63	Ribonuclease H1, N-terminal
Os10g0560500	Conserved hypothetical protein.	AK100157	Q8SBA5	-
Os10g0560850	Hypothetical gene.	EU940765	longestORF	-
Os10g0561000	Hypothetical conserved gene.	BT064708	Q9AUT7	-
Os10g0561200	Similar to CigA protein.	AK066634	Q9AUT5	-
Os10g0561250	Hypothetical protein.	tplb0041o18 (Wheat FLcDNA)	longestORF	-
Os10g0561300	Similar to Monosaccharid transporter.	AK068343	NP_001149551.1	Sugar/inositol transporter
Os10g0562000	Similar to glycoprotein.	AK106294	NP_001151220.1	-
Os10g0566800	Haem peroxidase, plant/fungal/bacterial family protein.	AK063299	Q5U1H3	Plant peroxidase
Os10g0567000	Camphor resistance CrcB protein family protein.	AK103373	NP_001147074.1	Camphor resistance CrcB protein
Os10g0567100	Similar to Chlorophyll b synthase (Fragment).	AK063367	Q8S7E1	Pheophorbide a oxygenase
Os10g0567200	Hypothetical protein.	AK100517	longestORF	-
Os10g0567300	SH2 motif domain containing protein.	AK070829	Q336Q9	SH2 motif
Os10g0567400	Similar to Rieske.	AK065124 ,AK071899	Q8S7E1	Pheophorbide a oxygenase
Os10g0567500	RNA-directed DNA polymerase (reverse transcriptase) domain containing protein.	AK072336	B6SWF1	Reverse transcriptase
Os10g0567600	Uncharacterised protein family UPF0503 domain containing protein.	AK105119	Q8S7E3	Uncharacterised protein family UPF0503
Os10g0567800	Similar to predicted protein.	AK061703	NP_567094.1	Dynamin central domain
Os10g0567900	Similar to F-box protein interaction domain containing protein, expressed.	AK064083	Q108X5	F-box associated interaction domain
Os10g0571250	Non-protein coding transcript.	EU950639	NONE	-
Os10g0571600	No apical meristem (NAM) protein domain containing protein.	AK110684	XP_002329572.1	No apical meristem
Os10g0572100	Uncharacterised protein family UPF0005 domain containing protein.	AK073002 ,AK105742	O49696	Malate transporter, aliminium toerance
Os10g0572400	Conserved hypothetical protein.	AK062550	Q8S7M6	-
Os10g0572950	Hypothetical protein.	EU959880	longestORF	-
Os10g0575650	Hypothetical gene.	BT086509	longestORF	-
Os10g0575950	Similar to F5D14.6 protein.	AK241904	Q9LQM9	-
Os10g0577000	Conserved hypothetical protein.	AK059043	Q8W3G9	-
Os10g0578500	Hypothetical conserved gene.	ab initio prediction	Q7XBT9	Pentatricopeptide repeat
Os10g0579150	Hypothetical gene.	EU951391	longestORF	-
Os10g0579400	Conserved hypothetical protein.	AK108346	Q9FRP5	-
Os10g0579800	TGF-beta receptor, type I/II extracellular region family protein.	AK121847	NP_001148010.1	Oligopeptide transporter
Os10g0579850	Hypothetical protein.	tplb0038k08 (Wheat FLcDNA)	longestORF	-
Os10g0580300	Serine/threonine protein kinase domain containing protein.	AK066824 ,AK104880	Q336M2	Protein kinase, catalytic domain
Os10g0580900	Non-protein coding transcript.	AK062821	NONE	-
Os11g0100300	Conserved hypothetical protein.	AK369990	NP_001176293.1	-
Os11g0100600	Alpha/beta hydrolase family protein.	AK060331	B4FK75	Alpha/beta hydrolase fold-1
Os11g0100800	Similar to Exo70 exocyst complex subunit family protein.	AK287644	Q2RBS0	Exo70 exocyst complex subunit
Os11g0101200	Similar to RNase P subunit p30 family protein, expressed.	AK058862	Q2RBR8	-
Os11g0102100	WUS-type homenodomain protein, Leaf development	AM490243	A8Y7W4	-
Os11g0104900	Similar to Clathrin heavy chain.	ab initio prediction	Q39834	Clathrin, heavy chain/VPS, 7-fold repeat

Os11g0104933	Non-protein coding transcript.	AK242528	NONE	-
Os11g0112400	Peroxidase (EC 1.11.1.7).	AK242814	Q5U1F8	Plant peroxidase
Os11g0127800	Similar to FHA domain containing protein, expressed.	AK059969 ,AK104179	Q2QYC9	Forkhead-associated
Os11g0127900	Similar to 40S ribosomal protein S16.	AK059550	Q0IQF7	Ribosomal protein S9
Os11g0127951	Hypothetical protein.	ab initio prediction	NONE	-
Os11g0128500	Similar to Myb factor.	AK241549	O04109	SANT domain, DNA binding
Os11g0128600	Conserved hypothetical protein.	ab initio prediction	NP_001065637.1	-
Os11g0128700	Mad3/BUB1 homology region 1 domain containing protein.	AK102502	Q2RB24	Mad3/BUB1 homology region 1
Os11g0128800	Similar to Homoserine dehydrogenase-like protein.	AK071434	B8AES5	Homoserine dehydrogenase, catalytic
Os11g0128932	Hypothetical gene.	BT054648	longestORF	-
Os11g0129500	Similar to GDSL-like Lipase/Acylhydrolase family protein, expressed.	AK107889	Q2RB18	-
Os11g0130100	Conserved hypothetical protein.	AK063914	Q2RB13	-
Os11g0130400	Harpin-induced 1 domain containing protein.	AK243509	NP_001065651.2	Late embryogenesis abundant protein, LEA-14
Os11g0130500	Cyclin-like F-box domain containing protein.	AK241516	Q2RB09	F-box domain, cyclin-like
Os11g0130600	Conserved hypothetical protein.	AK066342	NP_001066054.1	-
Os11g0130700	Conserved hypothetical protein.	ab initio prediction	B9G976	-
Os11g0131100	VQ domain containing protein.	CT835703	Q2RB04	VQ
Os11g0131400	Hypothetical conserved gene.	AK109466	Q2RB01	Pentatricopeptide repeat
Os11g0131500	Hypothetical protein.	BT085496	longestORF	-
Os11g0131600	Similar to YDG/SRA domain containing protein.	BT055690	Q2RB00	SET domain
Os11g0131700	Zinc finger, Tim10/DDP-type domain containing protein.	AK243327	B6SLJ2	Mitochondrial inner membrane translocase complex, Tim8/9/10/13-zinc finger-like
Os11g0131900	Similar to alpha-N-arabinofuranosidase A.	AK061612	B6T9B9	Alpha-L-arabinofuranosidase, C-terminal
Os11g0132400	Uncharacterised protein family UPF0414 domain containing protein.	AK059074	NP_001176772.1	Protein of unknown function DUF872, transmembrane
Os11g0133300	Similar to D-mannose binding lectin family protein, expressed.	AK121819 ,AK102340	Q2QY68	Epidermal growth factor-like, type 3
Os11g0133700	Similar to Glucosamine-fructose-6-phosphate aminotransferase, isomerizing family protein, expresse	AK058784	Q2RAY0	-
Os11g0133800	Similar to zinc finger (DHHC type) family protein.	AK358270	NP_191252.1	Zinc finger, DHHC-type, palmitoyltransferase
Os11g0133850	Hypothetical gene.	EU946073	longestORF	-
Os11g0134400	Calmodulin-like protein 3.	Q0IQB6	Q0IQB6	Calcium-binding EF-hand
Os11g0137050	Hypothetical protein.	tplb0036f07 (Wheat FLcDNA)	longestORF	-
Os11g0137250	Hypothetical gene.	AK242555	longestORF	-
Os11g0143300	A-type response regulator, Cytokinin signaling	AK058585 ,AB249659 ,BR00025(	Q2RAP3	Signal transduction response regulator, receiver domain
Os11g0144950	Hypothetical protein.	FP101342	longestORF	-
Os11g0149900	PAN-like, type 2 domain containing protein.	BT034293	NP_001176354.1	S-locus glycoprotein
Os11g0166800	Transcription elongation factor, TFIIS/CRSP70, N-terminal domain containing protein.	AK070928	Q53PV2	Transcription factor IIS, N-terminal
Os11g0166866	Non-protein coding transcript.	BT065504	NONE	-
Os11g0166932	Non-protein coding transcript.	EU949846	NONE	-
Os11g0168000	Protein of unknown function DUF1278 family protein.	AK062599	Q9SES8	Protein of unknown function DUF784
Os11g0168200	60S ribosomal protein L3.	AK073392 ,AK101469	A5D934	Ribosomal protein L3
Os11g0168250	Hypothetical gene.	BT016883	longestORF	-
Os11g0168300	Alba, DNA/RNA-binding protein family protein.	AK061362 ,AK104405	B6T7F3	DNA/RNA-binding protein Alba-like
Os11g0168600	Protein kinase, core domain containing protein.	AK063805	Q53JG3	Protein kinase, catalytic domain
Os11g0180000	Plant disease resistance response protein family protein.	AK060077	NP_001150515.1	Plant disease resistance response protein
Os11g0180100	Conserved hypothetical protein.	AK375448	Q53NQ8	-
Os11g0180900	Myb transcription factor domain containing protein.	BT069656	Q6SL45	SANT domain, DNA binding
Os11g0181000	Hypothetical protein.	EU971393	longestORF	-
Os11g0181200	Hypothetical protein.	AK063747	longestORF	-
Os11g0182100	Protein of unknown function DUF688 domain containing protein.	AK107888	Q53MM1	Protein of unknown function DUF688
Os11g0193300	Hypothetical conserved gene.	AK109751	Q53NH3	-
Os11g0201175	Similar to H0315A08.1 protein.	FP099421	Q01I63	-
Os11g0203400	Hypothetical conserved gene.	AK105579	Q6ZI94	-
Os11g0222800	Similar to LGC1.	EU947580	Q53P07	-
Os11g0272900	Conserved hypothetical protein.	AK068971	Q53MK6	-
Os11g0300501	Non-protein coding transcript.	AK242366	NONE	-
Os11g0440200	Similar to GDA1/CD39 family protein, expressed.	ab initio prediction	Q53KM8	Nucleoside phosphatase GDA1/CD39
Os11g0460851	Non-protein coding transcript.	tplb0037b22 (Wheat FLcDNA)	NONE	-
Os11g0574725	Hypothetical conserved gene.	ab initio prediction	Q2R289	F-box domain, cyclin-like
Os11g0596000	Hypothetical conserved gene.	AK288199	Q2R1R7	-
Os11g0613950	Non-protein coding transcript.	DQ246094	NONE	-
Os11g0623650	Non-protein coding transcript.	BT065526	NONE	-
Os11g0629900	Conserved hypothetical protein.	AK288199	Q2R0W5	-
Os11g0636600	Conserved hypothetical protein.	AK107824	Q8S7N2	-
Os11g0649801	Similar to E3 ubiquitin-protein ligase EL5.	FP092295	Q9LRB7	Zinc finger, RING-type
Os11g0650600	Similar to von Willebrand factor type A domain containing protein, expressed.	FP091693	Q2R0C5	von Willebrand factor, type A
Os11g0656500	Integrase, catalytic core domain containing protein.	AK072370	Q01M13	Integrase, catalytic core
Os11g0671600	Conserved hypothetical protein.	AK288199	Q2QZU2	-
Os11g0697350	Conserved hypothetical protein.	AK241561	NP_001176733.1	-
Os11g0697600	Hypothetical gene.	CT841618	longestORF	-
Os11g0699532	Hypothetical gene.	CT841618	longestORF	-
Os12g0100200	Conserved hypothetical protein.	AK369990	NP_001176293.1	-
Os12g0100700	Similar to Exo70 exocyst complex subunit family protein.	AK287644	Q2RBS0	Exo70 exocyst complex subunit
Os12g0101000	Similar to CCT motif family protein.	AK100957	Q2QYZ4	CCT domain
Os12g0101600	WUS-type homenodomain protein, Leaf development	AM490243	A8Y7W4	-
Os12g0101901	Hypothetical gene.	EU949907	longestORF	-
Os12g0102300	Similar to WRKY transcription factor 57.	AK242855	Q6IEM4	-
Os12g0106801	Hypothetical genes.	ab initio prediction	NONE	-
Os12g0118400	Similar to Inwardly rectifying potassium channel subunit.	AK062407	Q6ZXS4	Potassium channel, voltage-dependent, EAG/ELK/ERG
Os12g0131150	Similar to Glucosamine-fructose-6-phosphate aminotransferase, isomerizing family protein, expresse	AK058784	Q2RAY0	-
Os12g0132200	Similar to CBL-interacting protein kinase 33.	AK111953	Q2RAX3	Protein kinase, catalytic domain
Os12g0132300	Similar to Calmodulin (CaM).	AK111834 ,AK119956	Q0IQB6	-
Os12g0132400	Similar to Grwd-pending-prov protein.	AK111507	Q1HFN6	WD40 repeat
Os12g0132500	Similar to Major facilitator superfamily antiporter.	CT833630	Q8H6D5	-
Os12g0132650	Non-protein coding transcript.	tplb0023l07 (Wheat FLcDNA)	NONE	-
Os12g0132900	Hypothetical gene.	AK068041	GeneMark	-
Os12g0133000	Similar to Major facilitator superfamily antiporter.	EU970439	B6TZM4	Major facilitator superfamily
Os12g0133025	Non-protein coding transcript.	tplb0023l07 (Wheat FLcDNA)	NONE	-
Os12g0133050	Similar to 60S acidic ribosomal protein P0.	AK361198	NP_001105482.1	Ribosomal protein L10/acidic P0
Os12g0133350	Non-protein coding transcript.	tplb0023l07 (Wheat FLcDNA)	NONE	-
Os12g0133700	Similar to Dual specificity protein phosphatase 19 (EC 3.1.3.48) (EC 3.1.3.16) (Stress-activated prot	CU405769	Q2HSZ6	Dual specificity phosphatase, catalytic domain
Os12g0133750	Hypothetical protein.	ab initio prediction	NONE	-
Os12g0133850	Hypothetical protein.	tplb0036f07 (Wheat FLcDNA)	longestORF	-
Os12g0133900	Similar to Epa4p.	AK102656	Q2RAU6	Mpp10 protein
Os12g0133950	Non-protein coding transcript.	BT016200	NONE	-
Os12g0134000	Similar to hydroxymethylglutaryl-CoA lyase.	AK061875	B6TKJ5	Pyruvate carboxyltransferase
Os12g0134500	Similar to Zinc finger, C3HC4 type family protein.	AK358384	Q2RAT8	Zinc finger, RING-type
Os12g0135100	Similar to Phosphoglycerate mutase family protein.	BT062522	Q2QY22	Phosphoglycerate/bisphosphoglycerate mutase, active site
Os12g0135850	Similar to No apical meristem protein.	ab initio prediction	Q2QSX2	No apical meristem
Os12g0139400	A-type response regulator, Cytokinin signaling	AB249660 ,BR000257	Q2QXY3	Signal transduction response regulator, receiver domain
Os12g0140300	FBD domain containing protein.	AK102092	B9G816	-
Os12g0140550	Hypothetical gene.	EU945989	longestORF	-
Os12g0149300	Similar to galactosyl transferase GMA12/MNN10 family protein.	AK110842	XP_002887538.1	Galactosyl transferase
Os12g0149750	Hypothetical protein.	EU943630	longestORF	-
Os12g0149800	Similar to G10.	AK103299	NP_001148940.1	G10 protein
Os12g0150200	Similar to Cytochrome P450 CYP94C20.	AK064287	B6STM6	Cytochrome P450
Os12g0150901	Conserved hypothetical protein.	tplb0051a13 (Wheat FLcDNA)	Q2QXM5	-
Os12g0151600	Hypothetical conserved gene.	BT061672	NP_190845.1	-
Os12g0152000	Similar to Terminal flower 1-like protein.	AF159883	Q9XGS5	Phosphatidylethanolamine-binding, conserved site
Os12g0152200	Cyclin-like F-box domain containing protein.	ab initio prediction	NP_001066174.1	F-box domain, cyclin-like
Os12g0152500	Hypothetical conserved gene.	BT039089	A2ZI44	-
Os12g0152800	Rhodanese-like domain containing protein.	AK073401	NP_001152230.1	Dual specificity phosphatase, catalytic domain
Os12g0153200	Conserved hypothetical protein.	AK108198	A2ZI60	-
Os12g0153400	Cyclin-like F-box domain containing protein.	ab initio prediction	NP_001066174.1	F-box domain, cyclin-like
Os12g0153433	Hypothetical gene.	CT835608	longestORF	-
Os12g0153500	Carbonic anhydrase, CAH1-like domain containing protein.	AK070276	A2ZI48	Carbonic anhydrase, alpha-class, catalytic domain
Os12g0153700	Peptidase aspartic, catalytic domain containing protein.	ab initio prediction	Q2QXK3	Peptidase A1
Os12g0154000	Protein of unknown function DUF250 domain containing protein.	AK105118	NP_001150661.1	Domain of unknown function DUF250

Os12g0154400	t-snare domain containing protein.	BT035718	B6TDT7	SNARE-complex protein Syntaxin-18 N-terminal
Os12g0154900	Germin-like protein 12-3, Disease resistance	Inferred from literature	Q2QXJ1	Germin
Os12g0155000	Germin-like protein 12-4, Disease resistance	CT837600	Q2QXJ0	-
Os12g0155100	Protein of unknown function DUF3049 domain containing protein.	AK060647	A2ZI76	Protein of unknown function DUF3049
Os12g0155200	Similar to Rac GTPase activating protein.	AK067300	B6SWP2	PAK-box/P21-Rho-binding
Os12g0155250	Hypothetical protein.	tplb0049d09 (Wheat FLcDNA)	longestORF	-
Os12g0155300	Conserved hypothetical protein.	AK060480	Q2QXI6	-
Os12g0155400	Similar to Leucine Rich Repeat family protein, expressed.	X79130	Q8S5D5	-
Os12g0155600	Non-protein coding transcript.	X06283	NONE	-
Os12g0156100	Similar to NAC-domain containing protein 90 (ANAC090).	AK109534	XP_002874067.1	No apical meristem
Os12g0156400	Dcp1-like decapping family protein.	AK072086 ,AK099278	NP_001149166.1	Dcp1-like decapping
Os12g0156500	Cyclin-like F-box domain containing protein.	EU969867	NP_001151212.1	F-box domain, cyclin-like
Os12g0156900	Pentatricopeptide repeat domain containing protein.	BT018623	Q2QXH1	Pentatricopeptide repeat
Os12g0157000	DNA-binding pseudobarrel domain domain containing protein.	AK110593	B8BNA0	DNA-binding pseudobarrel domain
Os12g0157066	Hypothetical protein.	AK375277	longestORF	-
Os12g0157200	EF-Hand type domain containing protein.	AK064843	NP_196244.1	TLDc
Os12g0159600	Harpin-induced 1 domain containing protein.	AK070481	NP_001149809.1	Late embryogenesis abundant protein, LEA-14
Os12g0159700	Similar to protein binding protein.	tplb0017m18 (Wheat FLcDNA)	NP_001147814.1	Exo70 exocyst complex subunit
Os12g0163000	Similar to CNGC18 (CYCLIC NUCLEOTIDE-GATED CHANNEL 18)%3B calmodulin binding / c	ab initio prediction	NP_196991.1	Cyclic nucleotide-binding domain
Os12g0169300	Conserved hypothetical protein.	AK287997	NP_001176998.1	-
Os12g0177800	Similar to D-mannose binding lectin family protein, expressed.	AK105397	Q2QWY4	S-locus glycoprotein
Os12g0178250	Similar to dehydration-responsive protein-related.	ab initio prediction	NP_001031109.1	Protein of unknown function DUF248, methyltransferase putative
Os12g0178300	Similar to ankyrin-like protein.	ab initio prediction	NP_001151565.1	Protein of unknown function DUF248, methyltransferase putative
Os12g0178600	Similar to dehydration-responsive protein-related.	ab initio prediction	NP_190676.1	Protein of unknown function DUF248, methyltransferase putative
Os12g0178633	WD40 repeat domain containing protein.	AK369075	B8BNG0	WD40 repeat
Os12g0178800	Hypothetical protein.	AK063709	longestORF	-
Os12g0179100	Hypothetical gene.	AK106975	longestORF	-
Os12g0179700	Hypothetical conserved gene.	AK068305	Q2QWX0	-
Os12g0189700	Twin arginine translocation signal, Tat domain containing protein.	AK287777	A9P324	Twin-arginine translocation pathway, signal sequence
Os12g0190875	Similar to H0315A08.1 protein.	FP099421	Q01I63	Ribonuclease H1, N-terminal
Os12g0191300	Similar to Phage head-tail adaptor family protein, expressed.	BT067332	Q2QWM4	Aminotransferase, class V/Cysteine desulfurase
Os12g0191400	Conserved hypothetical protein.	BT085962	NP_001183161.1	-
Os12g0191500	Similar to Peroxidase 43 precursor (EC 1.11.1.7) (Atperox P43).	AK061040	B9V0M3	Plant peroxidase
Os12g0191901	Hypothetical gene.	CU406111	longestORF	-
Os12g0195100	Similar to amino acid permease.	EU962989	O82044	Amino acid transporter, transmembrane
Os12g0195183	Hypothetical protein.	tplb0036o08 (Wheat FLcDNA)	longestORF	-
Os12g0198500	Conserved hypothetical protein.	AK242191	Q2QWE0	-
Os12g0202700	Similar to N-methyltransferase.	CU406772	Q96565	O-methyltransferase, family 2
Os12g0203000	Similar to Cyclin-dependent protein kinase-like protein.	AK100360	Q2QW88	Protein kinase, catalytic domain
Os12g0203300	Similar to amino acid permease 1.	EU949945	NP_001149517.1	Amino acid transporter, transmembrane
Os12g0207500	Similar to ATP synthase subunit beta.	ab initio prediction	Q2QW50	ATPase, F1/V1/A1 complex, alpha/beta subunit, nucleotide-binding domain
Os12g0207600	Similar to ribulose-1,5-bisphosphate carboxylase/oxygenase large subunit.	ab initio prediction	YP_654221.1	Ribulose bisphosphate carboxylase, large subunit, C-terminal
Os12g0222800	Leucine-rich repeat, N-terminal domain containing protein.	ab initio prediction	Q2QVP4	Leucine-rich repeat
Os12g0228500	Hypothetical conserved gene.	AK287997	NP_001176998.1	-
Os12g0233366	Non-protein coding transcript.	CT835611	NONE	-
Os12g0299650	Myb/SANT-like domain domain containing protein.	BT063151	NP_001176912.1	Myb/SANT-like domain
Os12g0424180	Similar to ATP synthase F0 subunit 6.	GU075810	YP_514657.1	ATPase, F0 complex, subunit A
Os12g0484375	Similar to H0315A08.1 protein.	FP099421	Q01I63	Ribonuclease H1, N-terminal
Os12g0510766	Non-protein coding transcript.	EU942847	NONE	-
Os12g0510900	Similar to LIM domain protein WLIM-1.	AK058220	Q2QQ10	Zinc finger, LIM-type
Os12g0511400	Similar to NB-ARC domain containing protein.	AK105287	Q2QQ03	Disease resistance protein
Os12g0511750	Nucleic acid-binding, OB-fold domain containing protein.	AK287637	NP_001175163.1	Nucleic acid-binding, OB-fold
Os12g0520200	Similar to phenylalanine ammonia-lyase.	AK240729	Q2QPR0	Phenylalanine/histidine ammonia-lyase
Os12g0521101	Hypothetical protein.	tplb0048a09 (Wheat FLcDNA)	longestORF	-
Os12g0521300	Similar to HAT family dimerisation domain containing protein.	EU942994	Q2QPQ7	HAT dimerisation
Os12g0524201	Hypothetical conserved gene.	AK288953	B6U883	-
Os12g0524750	Similar to ribosomal protein S7.	ab initio prediction	YP_514635.1	Ribosomal protein S7
Os12g0528400	Similar to Glutathione synthetase (Fragment).	AK058922	Q2PZ33	Glutathione synthase, eukaryotic
Os12g0534700	Protein kinase-like domain containing protein.	AK122037	B8BMA3	-
Os12g0538066	Similar to HAT family dimerisation domain containing protein.	AK122139	Q2QPA8	Zinc finger, BED-type predicted
Os12g0539333	Conserved hypothetical protein.	AK242608	NP_001176995.1	-
Os12g0539400	Hypothetical conserved gene.	CT828588	Q2QP77	-
Os12g0562400	Similar to Phospholipase C (Fragment).	CT835563	B6SKE7	C2 calcium-dependent membrane targeting
Os12g0562433	Hypothetical gene.	AK248585	longestORF	-
Os12g0562455	Non-protein coding transcript.	tplb0025h13 (Wheat FLcDNA)	NONE	-
Os12g0564600	Zinc finger, CCHC-type domain containing protein.	AK101383	XP_002865765.1	Zinc finger, CCHC-type
Os12g0564701	Hypothetical protein.	tplb0029h05 (Wheat FLcDNA)	longestORF	-
Os12g0564800	NB-ARC domain containing protein.	AK103886	A3CIH5	Disease resistance protein
Os12g0564866	Hypothetical protein.	tplb0040n12 (Wheat FLcDNA)	longestORF	-
Os12g0564932	Hypothetical protein.	ab initio prediction	NONE	-
Os12g0565166	Non-protein coding transcript.	tplb0040n12 (Wheat FLcDNA)	NONE	-
Os12g0565200	Conserved hypothetical protein.	ab initio prediction	NP_001067046.2	-
Os12g0565300	Similar to Calmodulin-binding protein MPCBP.	AK073968	B6U2D5	Tetratricopeptide-like helical
Os12g0572201	Hypothetical gene.	AK068492	longestORF	-
Os12g0572400	Similar to SC35-like splicing factor SCL30, 30 kD.	AK065531	Q7XXQ1	RNA recognition motif domain
Os12g0572500	Region of unknown function XH domain containing protein.	AK064511	Q2QNA8	Uncharacterised domain XH
Os12g0572601	Hypothetical gene.	AK287811	longestORF	-
Os12g0572700	Exostosin-like domain containing protein.	ab initio prediction	NP_001177020.1	Exostosin-like
Os12g0576100	Conserved hypothetical protein.	CU406037	NP_001067107.1	-
Os12g0587116	Non-protein coding transcript.	AK358137	NONE	-
Os12g0590550	Hypothetical gene.	AK062999	longestORF	-
Os12g0592832	Hypothetical protein.	ab initio prediction	NONE	-
Os12g0594000	WD40 repeat-like domain containing protein.	AK354406	Q1EPE0	WD40 repeat
Os12g0594200	Hypothetical conserved gene.	EU942864	NP_001067177.2	-
Os12g0594201	Hypothetical gene.	CU406459	longestORF	-
Os12g0594300	Hypothetical conserved gene.	ab initio prediction	B9GE20	Phox/Bem1p
Os12g0594600	BTB/POZ fold domain containing protein.	AK101239	A2ZMA8	SKP1 component
Os12g0594700	Similar to F-box domain containing protein.	ab initio prediction	Q2QMR2	F-box domain, Skp2-like
Os12g0594950	Hypothetical conserved gene.	AK287432	NP_001177039.1	-
Os12g0595000	Similar to EXS, C-terminal.	AK065252	NP_001151325.1	EXS, C-terminal
Os12g0595300	Conserved hypothetical protein.	AK106840	A3CJ25	-
Os12g0595600	Hypothetical conserved gene.	ab initio prediction	B9GE25	SKP1 component
Os12g0596401	Non-protein coding transcript.	X06283	NONE	-
Os12g0596501	Hypothetical protein.	tplb0045o13 (Wheat FLcDNA)	longestORF	-
Os12g0596600	Conserved hypothetical protein.	AK064196	B9GE28	-
Os12g0596800	Zinc finger, LIM-type domain containing protein.	AK065760	B6SPK3	Zinc finger, LIM-type
Os12g0596850	Non-protein coding transcript.	tplb0034a09 (Wheat FLcDNA)	NONE	-
Os12g0597000	Similar to Calcineurin B-like protein 2 (SOS3-like calcium binding protein 1).	AK111887	Q3HRP5	Recoverin
Os12g0597051	Non-protein coding transcript.	BT018557	NONE	-
Os12g0597200	Similar to MATH domain containing protein, expressed.	AK059486	Q2QMP1	-
Os12g0597400	HI0933-like protein family protein.	AK071949 ,AK099006	NP_566235.1	-
Os12g0597600	Similar to KH domain containing protein.	AK066451	NP_001148654.1	K Homology, type 1
Os12g0597650	Non-protein coding transcript.	AK288557	NONE	-
Os12g0597700	Similar to WRKY DNA binding domain containing protein, expressed.	AK104890	Q2QMN5	DNA-binding WRKY
Os12g0598000	Non-protein coding transcript.	AK242434	NONE	-
Os12g0599400	Helix-loop-helix DNA-binding domain containing protein.	ab initio prediction	Q2QML8	Helix-loop-helix DNA-binding
Os12g0599550	Similar to Helix-loop-helix DNA-binding domain containing protein.	ab initio prediction	Q2QML8	-
Os12g0599800	Pectinesterase inhibitor domain containing protein.	AK111139	A2ZME3	Pectinesterase inhibitor
Os12g0599900	Tetratricopeptide-like helical domain containing protein.	AK101252	B4FG08	Tetratricopeptide TPR-1
Os12g0600000	Conserved hypothetical protein.	AK109945	NP_001067202.1	-
Os12g0600100	Tetratricopeptide-like helical domain containing protein.	AK063560	B4FG08	Tetratricopeptide TPR-1
Os12g0600150	Hypothetical gene.	AK241829	longestORF	-
Os12g0600200	Similar to Zinc finger, C3HC4 type (RING finger) containing protein.	AK102450	Q60DL1	Zinc finger, RING/FYVE/PHD-type
Os12g0600301	Hypothetical protein.	tplb0048g08 (Wheat FLcDNA)	longestORF	-
Os12g0600400	Similar to Pyridoxal kinase.	AK066514	Q69G22	Pyridoxal phosphate

Os12g0600550	Non-protein coding transcript.	AK288257	NONE	-
Os12g0601000	Similar to Leucine Rich Repeat family protein, expressed.	EU973703	Q2QMK5	F-box domain, Skp2-like
Os12g0601200	Uridine kinase family protein.	AK059928	B6TCV6	Phosphoribulokinase/uridine kinase
Os12g0604000	Non-protein coding transcript.	AK067077 ,AK067588	NONE	-
Os12g0604200	Similar to phosphoinositide binding.	ab initio prediction	NP_188920.1	Domain of unknown function DUF828
Os12g0604300	Hypothetical gene.	AK241135	longestORF	-
Os12g0604500	IQ motif, EF-hand binding site domain containing protein.	AK372379	NP_001177052.1	IQ motif, EF-hand binding site
Os12g0604650	Non-protein coding transcript.	EU948222	NONE	-
Os12g0605400	Similar to CROC-1-like protein (Fragment).	AK068060	B6SK03	Ubiquitin-conjugating enzyme, E2
Os12g0605500	Tesmin/TSO1-like, CXC domain containing protein.	AK073942	B8BMW2	Tesmin/TSO1-like, CXC
Os12g0606900	Similar to Ulp1 protease family, C-terminal catalytic domain containing protein, expressed.	AK072659	Q2QME9	-
Os12g0607000	Similar to homocysteine S-methyltransferase-2.	AK103293	NP_001105012.1	Homocysteine S-methyltransferase
Os12g0607100	Similar to Eukaryotic translation initiation factor 2 gamma subunit.	AK061482	B6TNU0	Protein synthesis factor, GTP-binding
Os12g0611500	Similar to H0315A08.1 protein.	FP099421	Q01I63	Ribonuclease H1, N-terminal
Os12g0613000	Similar to NF-YA subunit.	AK065163	B6TY54	CCAAT-binding transcription factor, subunit B
Os12g0613200	Hypothetical conserved gene.	AK069599	B9GE94	SET domain
Os12g0613700	Transcriptional factor B3 family protein.	AK121703	Q2QM84	AUX/IAA protein
Os12g0613850	Similar to Galactosyltransferase family.	ab initio prediction	B6SVW0	Galectin, carbohydrate recognition domain
Os12g0614100	Similar to Lipase family protein.	BT042072	Q2QM82	Lipase, class 3
Os12g0614200	Similar to Lipase family protein.	BT042072	Q2QM81	Lipase, class 3
Os12g0614201	Conserved hypothetical protein.	AK243381	NP_001177060.1	-
Os12g0615700	Similar to TRANSPARENT TESTA 12 protein.	AK058512	E3SHE0	Multi antimicrobial extrusion protein
Os12g0617100	Similar to Translation initiation factor eIF-2B delta subunit (eIF-2B GDP-GTP exchange factor) (Gu	AK103148	NP_001159071.1	Initiation factor 2B-related
Os12g0617175	Non-protein coding transcript.	tplb0051c11 (Wheat FLcDNA)	NONE	-
Os12g0621766	Hypothetical protein.	EU947100	GeneMark	-
**HHZ pedigree**				
Os01g0113150	Disease resistance protein domain containing protein.	AK367884	A2ZNH1	Disease resistance protein
Os01g0114050	Non-protein coding transcript.	EU941912	NONE	-
Os01g0121300	Conserved hypothetical protein.	AK103404	B8ACZ7	-
Os01g0121400	Hypothetical protein.	tplb0026h09 (Wheat FLcDNA)	longestORF	-
Os01g0121500	Conserved hypothetical protein.	AK107776	A2WK24	-
Os01g0128300	Similar to predicted protein.	CT841996	D7R521	-
Os01g0128400	Protein of unknown function DUF2359, TMEM214 domain containing protein.	AK121214	B8AD25	Protein of unknown function DUF2359, TMEM214
Os01g0133766	Non-protein coding transcript.	X02683	NONE	-
Os01g0155500	Protein kinase, catalytic domain domain containing protein.	AK099840	A2ZPF5	Protein kinase, catalytic domain
Os01g0155600	Similar to Splicing factor RSZ33.	AK071635	A6MZ96	RNA recognition motif domain
Os01g0156300	Similar to Cappuccino protein.	AK107993	A2WKV9	Protein of unknown function DUF1110
Os01g0157200	Conserved hypothetical protein.	AK108577	A2ZPG5	-
Os01g0157500	Protein of unknown function DUF1110 domain containing protein.	EU965613	NP_001172188.1 (RefS	Protein of unknown function DUF1110
Os01g0157600	Conserved hypothetical protein.	AK106766	Q9FYQ2	-
Os01g0157700	Glycosyl transferase, family 43 domain containing protein.	AK376884	Q5ZCC5	Glycosyl transferase, family 43
Os01g0159600	Small hydrophilic plant seed protein family protein.	AK122153 ,AK064055	Q3I3Z0	Stress induced protein
Os01g0164650	Non-protein coding transcript.	EU944689	NONE	-
Os01g0165100	Conserved hypothetical protein.	AK103011 ,AK059335	A2WL20	-
Os01g0165600	Similar to H0323C08.17 protein.	AK335607	Q942P3	-
Os01g0166700	Saposin family protein.	AK067805	B6T780	Saposin-like type B, 1
Os01g0172400	Similar to Phospholipase D alpha 1.	AK066556	B8ADH7	Phospholipase D/Transphosphatidylase
Os01g0172900	Glycoside hydrolase, family 28 domain containing protein.	ab initio prediction	B9ETD9	Glycoside hydrolase, family 28
Os01g0173000	Putative thiol-disulphide oxidoreductase DCC domain containing protein.	AK287669	GeneMark	Putative thiol-disulphide oxidoreductase DCC
Os01g0173100	Alba, DNA/RNA-binding protein family protein.	AK121114	NP_564325.1 (RefSeq)	DNA/RNA-binding protein Alba-like
Os01g0173950	Non-protein coding transcript.	tplb0011b09 (Wheat FLcDNA)	NONE	-
Os01g0176300	Pentatricopeptide repeat domain containing protein.	AK249469	NP_001042180.1 (RefS	Pentatricopeptide repeat
Os01g0177150	Non-protein coding transcript.	AK288777	NONE	-
Os01g0178300	Hypothetical protein.	AK062450	GeneMark	-
Os01g0178400	Similar to Protein phosphatase 2A B'kappa subunit.	AK108850	XP_002885457.1 (RefS	Protein phosphatase 2A, regulatory B subunit, B56
Os01g0178500	Similar to Auxin-responsive protein (Aux/IAA) (Fragment).	AK109373 ,AK060091 ,AK06924	Q5VRD1	-
Os01g0178700	Similar to Protein binding protein.	AK103147	B6TV66	Zinc finger, RING-type
Os01g0178775	Non-protein coding transcript.	tplb0030e06 (Wheat FLcDNA)	NONE	-
Os01g0178900	Similar to Symbiosis-related disease resistance protein (Fragment).	AK110770	Q8RVG6	-
Os01g0179000	Transferase family protein.	AK288049	B6TZV1	Transferase
Os01g0179600	UDP-glucuronosyl/UDP-glucosyltransferase family protein.	AK243646	NP_001042201.2 (RefS	UDP-glucuronosyl/UDP-glucosyltransferase
Os01g0179750	Non-protein coding transcript.	BT019232	NONE	-
Os01g0181033	Similar to NADH dehydrogenase subunit 1.	AJ010976	YP_024343.1 (RefSeq)	NADH:ubiquinone oxidoreductase, subunit 1/F420H2 oxidoreductase subunit H
Os01g0182300	Lipocalin domain containing protein.	AK062827	A2WLF0	-
Os01g0187200	Non-protein coding transcript.	AK110797	NONE	-
Os01g0194000	Conserved hypothetical protein.	AK071994	A2WLM7	-
Os01g0206650	Hypothetical protein.	tplb0042a18 (Wheat FLcDNA)	longestORF	-
Os01g0206700	Similar to Serine/threonine protein kinase (CBL-interacting protein kinase 20).	AK065589	Q9LWM4	Protein kinase, catalytic domain
Os01g0207200	Similar to Peptide-N4-(N-acetyl-beta-glucosaminyl)asparagine amidase A (EC 3.5.1.52) (PNGase A	AK103341	P81898	Peptide-N4-
Os01g0211000	Cytochrome P450 family protein.	ab initio prediction	Q2MIZ9	Cytochrome P450
Os01g0224700	Flavin monooxygenase-like enzyme, Auxin biosynthesis	AK070386	Q9LG41	FAD-dependent pyridine nucleotide-disulphide oxidoreductase
Os01g0225200	Similar to predicted protein.	AK121971	XP_002868685.1 (RefS	GTP-binding domain, HSR1-related
Os01g0225400	Pyruvate/Phosphoenolpyruvate kinase, catalytic core domain containing protein.	AK242525	NP_001150103.1 (RefS	Ketopantoate hydroxymethyltransferase
Os01g0225600	Similar to Dehydrin.	CU405989	Q5CAQ2	Late embryogenesis abundant protein, LEA-14
Os01g0233850	Hypothetical gene.	EU943630	longestORF	-
Os01g0235400	Similar to predicted protein.	AK064696	Q9ZPY7	Importin-beta, N-terminal
Os01g0236700	Hypothetical conserved gene.	AK061701	Q5NB82	Phox/Bem1p
Os01g0243400	Hypothetical conserved gene.	AK070651	A2ZR73	-
Os01g0243450	Non-protein coding transcript.	FP095602	NONE	-
Os01g0243600	Conserved hypothetical protein.	EU974727	NP_001042565.1 (RefS	-
Os01g0243901	Hypothetical protein.	AK376036	longestORF	-
Os01g0244150	Similar to J075123K08, full insert sequence.	AK374764	B7F983	Domain of unknown function DUF1618
Os01g0244400	Protein of unknown function DUF1618 domain containing protein.	AK242054	NP_001172253.1 (RefS	Domain of unknown function DUF1618
Os01g0245000	Conserved hypothetical protein.	AK372630	Q5NA94	-
Os01g0245600	Conserved hypothetical protein.	ab initio prediction	NP_001042571.2 (RefS	-
Os01g0245700	Domain of unknown function DUF1618 domain containing protein.	AK373668	NP_001172254.1 (RefS	Domain of unknown function DUF1618
Os01g0245901	Domain of unknown function DUF1618 domain containing protein.	ab initio prediction	Q9XHZ1	Domain of unknown function DUF1618
Os01g0246400	Similar to Low molecular mass early light-inducible protein HV90, chloroplast precursor (ELIP).	AK062972	NP_001152927.1 (RefS	Chlorophyll A-B binding protein
Os01g0246601	Protein of unknown function DUF296 domain containing protein.	BT064989	NP_001150963.1 (RefS	Domain of unknown function DUF296
Os01g0246700	Similar to WRKY transcription factor 1.	AK105509	Q6IET0	-
Os01g0247600	Conserved hypothetical protein.	AK109701	B9EUN9	-
Os01g0248300	Similar to Pathogen-related protein.	ab initio prediction	B6TQA1	-
Os01g0248350	Hypothetical protein.	AK366258	longestORF	-
Os01g0248600	GINS complex, Psf2 component family protein.	AK061039 ,AK100824	B6SJM9	GINS complex, subunit Psf2
Os01g0248701	Terpenoid synthase domain containing protein.	BT009461	A9ZN20	Polyprenyl synthetase
Os01g0248800	Conserved hypothetical protein.	AK062766	XP_002455366.1 (RefS	-
Os01g0248850	Hypothetical gene.	BT062860	longestORF	-
Os01g0248900	Similar to Expansin Os-EXPA3.	AK111100	Q9XHX0	Expansin
Os01g0249050	Hypothetical protein.	BT016845	longestORF	-
Os01g0249100	Similar to Expansin-A9.	DQ061054	Q4PR53	Expansin
Os01g0249700	Heavy metal-associated domain, HMA domain containing protein.	AK361799	Q9LIU6	Heavy metal-associated domain, HMA
Os01g0249800	Heavy metal-associated domain, HMA domain containing protein.	AK361799	Q9LIU5	Heavy metal-associated domain, HMA
Os01g0251200	Zinc finger, C2H2-like domain containing protein.	AK063298	B9EUQ0	Zinc finger, RING-type
Os01g0251400	Conserved hypothetical protein.	AK060341	B8ABS6	-
Os01g0252100	Similar to Glycogen synthase kinase-3 homolog MsK-3 (EC 2.7.1.-).	AK068737	Q7GC12	Protein kinase, catalytic domain
Os01g0252150	Hypothetical protein.	tplb0034j24 (Wheat FLcDNA)	longestORF	-
Os01g0252600	Similar to predicted protein.	CT835001	B8ABT0	-
Os01g0252900	Zinc finger, CCHC-type domain containing protein.	AK105250	XP_002878450.1 (RefS	Zinc finger, CCHC-type
Os01g0253000	Similar to LpimPth3.	AK071644	Q5NBC2	Protein kinase, catalytic domain
Os01g0253050	Conserved hypothetical protein.	EU957207	NP_001042608.1 (RefS	-
Os01g0253100	Similar to Avr9/Cf-9 induced kinase 1.	AK067133	B9EV01	Protein kinase, catalytic domain
Os01g0253200	Similar to pectinesterase inhibitor domain containing protein.	tplb0011f19 (Wheat FLcDNA)	NP_001148594.1 (RefS	Pectinesterase inhibitor
Os01g0253400	Protein of unknown function DUF1218 family protein.	AK108904	Q9S809	Protein of unknown function DUF1218
Os01g0253500	Conserved hypothetical protein.	AK243648	NP_001172261.1 (RefS	-
Os01g0253600	Replication factor A protein 3 domain containing protein.	AK058837	NP_001152271.1 (RefS	Nucleic acid-binding, OB-fold

Os01g0253800	Conserved hypothetical protein.	ab initio prediction	NP_001172262.1 (RefS	-
Os01g0253900	Similar to triacylglycerol lipase.	AK072113	NP_001148192.1 (RefS	Lipase, class 3
Os01g0254000	Similar to NTGB2 (Fragment).	AK111904 ,AK111979 ,AK11954	B4FPT6	Small GTP-binding protein domain
Os01g0254100	Similar to CTV.2.	AK111762	Q2HW32	WD40 repeat
Os01g0254200	Conserved hypothetical protein.	AK121256	A2WMZ3	-
Os01g0254850	Hypothetical conserved gene.	CT834921	Q5NBT3	-
Os01g0254900	Similar to Syntaxin 22 (AtSYP22) (AtVAM3).	AK068204	Q5S1U0	Target SNARE coiled-coil domain
Os01g0254950	Hypothetical gene.	AK287984	longestORF	-
Os01g0255100	Similar to Soluble epoxide hydrolase.	AK058528	NP_001148885.1 (RefS	Epoxide hydrolase-like
Os01g0255700	Conserved hypothetical protein.	EU947173	Q5NBS7	-
Os01g0256300	Conserved hypothetical protein.	AK357815	Q1EHU3	-
Os01g0256500	Similar to ZnI.	AK069098 ,AK119698	B1PXF8	-
Os01g0256600	Ribosomal protein L18/L5 domain containing protein.	AK063884 ,AK099373	NP_001150322.1 (RefS	Ribosomal protein L18/L5
Os01g0256900	Similar to SmX6 protein.	AK100819	NP_001152235.1 (RefS	Like-Sm ribonucleoprotein
Os01g0257100	Rapid ALkalinization Factor family protein.	AK243243	NP_001150840.1 (RefS	Rapid ALkalinization Factor
Os01g0257300	Similar to FPF1.	AK070626	NP_001151726.1 (RefS	-
Os01g0257400	Zinc finger, CCCH-type domain containing protein.	AK073920	Q5NAV3	Zinc finger, CCCH-type
Os01g0258600	Similar to predicted protein.	CT828253	E3W9Z9	-
Os01g0258700	Zinc finger, CCCH-type domain containing protein.	AK111903 ,AK111727	B7F3Z8	Zinc finger, CCCH-type
Os01g0259200	Similar to serine/threonine protein kinase PBS1.	AK067283	XP_002312759.1 (RefS	Protein kinase, catalytic domain
Os01g0259300	Non-protein coding transcript.	AK105158	NONE	-
Os01g0259400	Armadillo-type fold domain containing protein.	AK102537	XP_002873912.1 (RefS	Protein kinase, catalytic domain
Os01g0260000	Protein prenyltransferase domain containing protein.	AK108460	Q5NAR1	Pentatricopeptide repeat
Os01g0262000	Similar to agmatine coumaroyltransferase.	BT033950	B6SJ46	Transferase
Os01g0272700	Hypothetical conserved gene.	AK248395	NP_001150380.1 (RefS	Plastocyanin-like
Os01g0272800	Conserved hypothetical protein.	AK242815	B9EV58	-
Os01g0273150	Hypothetical gene.	CU861759	longestORF	-
Os01g0273200	Conserved hypothetical protein.	ab initio prediction	A2YSX7	-
Os01g0274500	Similar to Expansin-A11.	DQ062981	Q4PNY1	Expansin
Os01g0274800	R2R3-type MYB transcription factor, Sugar partitioning into anther	AK107461	Q5NBM8	SANT domain, DNA binding
Os01g0275700	Non-protein coding transcript.	EU949846	NONE	-
Os01g0275950	Hypothetical conserved gene.	ab initio prediction	NP_001042723.2 (RefS	-
Os01g0276066	Non-protein coding transcript.	BT061579	NONE	-
Os01g0276200	Similar to Mitochondrial import receptor subunit TOM40.	BT040757	B6TEP3	Porin, eukaryotic type
Os01g0276400	Similar to presenilin.	AK106403	NP_001149271.1 (RefS	Peptidase A22A, presenilin
Os01g0277600	Similar to aminophospholipid ATPase.	tplb0014e24 (Wheat FLcDNA)	XP_002315406.1 (RefS	ATPase, P-type, K/Mg/Cd/Cu/Zn/Na/Ca/Na/H-transporter
Os01g0278466	Non-protein coding transcript.	CT835260	NONE	-
Os01g0278900	Similar to 50S ribosomal protein L40.	AK120429	NP_001147184.1 (RefS	-
Os01g0279100	Similar to Magnesium-protoporphyrin ix monomethyl ester cyclase (Fragment).	AK061646	A6N1H0	-
Os01g0279700	Non-protein coding transcript.	AK058762	NONE	-
Os01g0281100	Conserved hypothetical protein.	AK109672	B9EVJ4	-
Os01g0281200	Similar to Type B-like cyclin (Fragment).	AK107209	Q0JNK6-2	Cyclin, C-terminal
Os01g0281250	Hypothetical protein.	tplb0047b05 (Wheat FLcDNA)	longestORF	-
Os01g0281301	Protein of unknown function DUF1645 domain containing protein.	BT065146	Q9LG63	Protein of unknown function DUF1645
Os01g0285300	Myb transcription factor domain containing protein.	AK111803	D6BV29	SANT domain, DNA binding
Os01g0286100	Helix-loop-helix DNA-binding domain containing protein.	AK102252	B9EVK4	Helix-loop-helix DNA-binding
Os01g0286550	Hypothetical gene.	EU943867	GeneMark	-
Os01g0286900	Similar to Auxin-responsive protein IAA31 (Indoleacetic acid-induced protein 31).	AK103865	A2ZRY8	AUX/IAA protein
Os01g0287400	Similar to Hydrophobic protein LTI6A (Low temperature-induced protein 6A).	AK058343	Q01L12	-
Os01g0287900	Hypothetical protein.	AK372761	longestORF	-
Os01g0290000	Similar to Cyprosin precursor (EC 3.4.23.-) (Fragment).	AK066523	NP_001152501.1 (RefS	Peptidase A1
Os01g0290600	Similar to isopenicillin N epimerase.	AK366122	NP_001151142.1 (RefS	Aminotransferase, class V/Cysteine desulfurase
Os01g0290800	Hypothetical protein.	AK119474	longestORF	-
Os01g0308700	Similar to H0315A08.1 protein.	FP099421	Q01I63	Ribonuclease H1, N-terminal
Os01g0324400	Hypothetical conserved gene.	AK111299	Q657G4	-
Os01g0366300	Similar to Receptor protein kinase.	AK064115	Q33B66	Protein kinase, catalytic domain
Os01g0379800	Similar to HAT family dimerisation domain containing protein.	AK122139	Q2QPA8	Zinc finger, BED-type predicted
Os01g0536200	Conserved hypothetical protein.	AK372466	Q6K2H9	-
Os01g0539900	Conserved hypothetical protein.	AK242029	B9EXE2	-
Os01g0540000	Similar to T22C5.14.	BT086321	Q9SFZ0	-
Os01g0540300	Rac-like GTP-binding protein 2.	Q68Y52	Q68Y52	Small GTPase superfamily
Os01g0556800	Hypothetical conserved gene.	ab initio prediction	NP_001172418.1 (RefS	Protein of unknown function DUF724
Os01g0557100	Alpha/beta hydrolase family protein.	AK287842	NP_001043328.1 (RefS	-
Os01g0557200	Conserved hypothetical protein.	EU972429	Q8S0U8	-
Os01g0558500	PWWP domain containing protein.	AK099982	A2WRD3	PWWP
Os01g0558600	Ras-related protein RIC1.	AK243597	P40392	Small GTPase superfamily
Os01g0558700	Conserved hypothetical protein.	AK287514	NP_001172419.1 (RefS	-
Os01g0558800	Similar to oxidoreductase/ transition metal ion binding protein.	AK068120	XP_002869432.1 (RefS	Protein of unknown function DUF3531
Os01g0558900	Conserved hypothetical protein.	AK058447	Q5JKR1	-
Os01g0559000	Protein of unknown function DUF1000 family protein.	AK061456	B6T8X4	Galactose-binding domain-like
Os01g0559200	Phosphorylated adapter RNA export protein, RNA-binding domain domain containing protein.	AK102611	B9EXJ7	Phosphorylated adapter RNA export protein, RNA-binding domain
Os01g0559300	Conserved hypothetical protein.	AK064666	A2WRD9	-
Os01g0564950	Protein of unknown function DUF247, plant domain containing protein.	AK375832	Q656U6	Protein of unknown function DUF247, plant
Os01g0607800	Hypothetical conserved gene.	AK058392	A2ZV92	Pentatricopeptide repeat
Os01g0613400	Non-protein coding transcript.	AK058952	NONE	-
Os01g0613500	Peptidase C1A, papain family protein.	AK070448	A2WSK3	Peptidase, cysteine peptidase active site
Os01g0618400	Similar to RNA helicase (Fragment).	AK067570	Q9FFQ1	RNA helicase, ATP-dependent, DEAD-box, conserved site
Os01g0618450	Non-protein coding transcript.	AF366447	NONE	-
Os01g0618500	Similar to RNA helicase (Fragment).	AK067476	B7EDN0	RNA helicase, ATP-dependent, DEAD-box, conserved site
Os01g0618601	Non-protein coding transcript.	AF366447	NONE	-
Os01g0619900	Similar to DNA binding.	AK060239	NP_001118417.1 (RefS	-
Os01g0637600	Similar to Peptide deformylase, chloroplast precursor (EC 3.5.1.88) (PDF) (Polypeptide deformylase	AK106980	Q5VNN5	Formylmethionine deformylase
Os01g0639900	Non-protein coding transcript.	AK058203	NONE	-
Os01g0641750	Hypothetical protein.	AK361892	longestORF	-
Os01g0642000	Carboxylesterase, type B family protein.	AK110911	NP_173937.2 (RefSeq)	Carboxylesterase, type B
Os01g0642100	Hypothetical conserved gene.	AK063793	B9EYA5	-
Os01g0642200	Conserved hypothetical protein.	AK105174 ,AK071521	B8ACD2	-
Os01g0644200	Similar to Little protein 1.	AK063634	B4UWC1	-
Os01g0647000	Similar to F-box domain containing protein.	AK061541	NP_001149398.1 (RefS	-
Os01g0648500	Similar to Transcription initiation factor TFIID subunit 7.	AK241160	B6UGL3	TAFII55 protein, conserved region
Os01g0648700	Bromodomain containing protein.	AK111374	Q94JA6	Bromodomain
Os01g0649000	WD40 repeat-like domain containing protein.	AK073564	A2WT83	WD40 repeat
Os01g0649100	Malate dehydrogenase.	AK071699	Q7FSL4	Lactate/malate dehydrogenase, N-terminal
Os01g0649566	Conserved hypothetical protein.	AK372466	Q6K2H9	-
Os01g0650501	Non-protein coding transcript.	EU942775	NONE	-
Os01g0650900	Lipase, GDSL domain containing protein.	AK375707	NP_001043727.1 (RefS	Lipase, GDSL
Os01g0650950	Hypothetical protein.	tplb0048c08 (Wheat FLcDNA)	longestORF	-
Os01g0651000	Lipase, GDSL domain containing protein.	AK242032	Q8RZ48	Lipase, GDSL
Os01g0651100	Pentatricopeptide repeat domain containing protein.	AK119380	B8A6T7	Pentatricopeptide repeat
Os01g0651300	Similar to GDSL-motif lipase/hydrolase-like protein.	AK111325	NP_001151904.1 (RefS	Lipase, GDSL
Os01g0651400	Lipase, GDSL domain containing protein.	AK059784	NP_001151904.1 (RefS	-
Os01g0651450	Hypothetical protein.	EU954131	longestORF	-
Os01g0651500	Wax synthase domain containing protein.	EU959294	B6T2S9	Wax synthase
Os01g0652000	Similar to proline-rich family protein.	AK058938	NP_193591.2 (RefSeq)	-
Os01g0652450	Hypothetical gene.	CT834813	Q6L4J0	-
Os01g0652600	Similar to Ketol-acid reductoisomerase, chloroplast precursor (EC 1.1.1.86) (Acetohydroxy-acid redu	AK072075	B6TF69	Acetohydroxy acid isomeroreductase C-terminal
Os01g0652650	Non-protein coding transcript.	D21110	NONE	-
Os01g0652700	Similar to predicted protein.	EU967350	NP_001043739.2 (RefS	Protein of unknown function DUF538
Os01g0652800	Protein of unknown function DUF231, plant domain containing protein.	AK061145 ,AK103547 ,AK10431	A2WTA8	Domain of unknown function DUF231, plant
Os01g0653500	Conserved hypothetical protein.	ab initio prediction	Q5VPB2	-
Os01g0654100	Similar to CTP synthase (EC 6.3.4.2) (UTP--ammonia ligase) (CTP synthetase).	AK069482	NP_001148101.1 (RefS	CTP synthase
Os01g0654150	Non-protein coding transcript.	tplb0056h06 (Wheat FLcDNA)	NONE	-
Os01g0654200	Peptidase M50 domain containing protein.	AK100750	B9EYD8	Peptidase M50
Os01g0654300	Similar to ARP2/3 complex 34 kDa subunit.	AK058593	B6TA29	Arp2/3 complex, 34kDa subunit p34-Arc
Os01g0654400	Similar to Salt tolerant correlative protein.	AK288103	E6Y2L2	-

Os01g0654450	Hypothetical gene.	EU946002	longestORF	-
Os01g0654800	Conserved hypothetical protein.	AK359861	B9EYE1	-
Os01g0655300	Similar to Trithorax 4 (Fragment).	AK062870	B3VSN3	SET domain
Os01g0668000	CS domain containing protein.	AK061159	B6SNS1	CS-like domain
Os01g0668600	Curculin-like (mannose-binding) lectin domain containing protein.	AK365081	NP_001043817.2 (RefS	Protein kinase, catalytic domain
Os01g0705400	Hypothetical conserved gene.	AK336128	A2ZX08	-
Os01g0707500	Similar to Transcription factor LAX PANICLE.	AK100208	A2WUA1	Helix-loop-helix DNA-binding
Os01g0708100	Similar to N-myristoyl transferase (EC 2.3.1.97).	AK101457	A2WUA4	Myristoyl-CoA:protein N-myristoyltransferase
Os01g0708201	Hypothetical gene.	BT024151	longestORF	-
Os01g0708400	Non-protein coding transcript.	AK242236	NONE	-
Os01g0710100	Hypothetical conserved gene.	AK357053	NP_001044037.2 (RefS	Protein of unknown function DUF761, plant
Os01g0711900	Conserved hypothetical protein.	AK108302	A2ZX51	-
Os01g0718300	Similar to Brassinosteroid-insensitive 1.	AK121760	Q0ZA03	Protein kinase, catalytic domain
Os01g0720200	Non-protein coding transcript.	AK058236	NONE	-
Os01g0720700	Similar to satase isoform I.	CT830895	NP_001105082.1 (RefS	-
Os01g0722550	Similar to HAT family dimerisation domain containing protein.	AK122139	Q2QPA8	Zinc finger, BED-type predicted
Os01g0723800	Hypothetical conserved gene.	AK061902	B8A926	ABC transporter, transmembrane domain
Os01g0732300	Protein of unknown function DUF623, plant domain containing protein.	AK108391	A2WUS7	Domain of unknown function DUF623
Os01g0742000	tRNA/rRNA methyltransferase, SpoU domain containing protein.	AK062779	NP_849992.1 (RefSeq)	tRNA/rRNA methyltransferase, SpoU
Os01g0745700	Hypothetical conserved gene.	AK062377	Q5JLX9	-
Os01g0748200	Conserved hypothetical protein.	AK372402	Q5JNJ4	-
Os01g0755700	Zinc finger, RING/FYVE/PHD-type domain containing protein.	AK108853	NP_001148026.1 (RefS	Zinc finger, RING-type
Os01g0766966	Hypothetical gene.	BT066271	longestORF	-
Os01g0769100	Non-protein coding transcript.	AK242413	NONE	-
Os01g0777650	Hypothetical gene.	AK289196	longestORF	-
Os01g0791033	Similar to ribulose-1,5-bisphosphate carboxylase/oxygenase large subunit.	ab initio prediction	NP_039391.1 (RefSeq)	Ribulose bisphosphate carboxylase, large subunit, C-terminal
Os01g0793900	Conserved hypothetical protein.	AK062811	A2WVX8	-
Os01g0800200	Conserved hypothetical protein.	ab initio prediction	NP_001172605.1 (RefS	-
Os01g0807000	Conserved hypothetical protein.	AK109751	Q53NH3	-
Os01g0810000	Utp11 family protein.	AK102143	NP_001150340.1 (RefS	Small-subunit processome, Utp11
Os01g0814400	Conserved hypothetical protein.	AK104661	B8ABA0	-
Os01g0814550	Non-protein coding transcript.	BT086943	NONE	-
Os01g0819100	Similar to predicted protein.	AK242625	C0LGW1	Protein kinase, catalytic domain
Os01g0823600	Conserved hypothetical protein.	AK242079	NP_001044661.1 (RefS	-
Os01g0825166	Similar to Splicing coactivator subunit-like protein.	EU944053	C8TFH6	-
Os01g0825332	Similar to Splicing coactivator subunit-like protein.	EU944053	C8TFH6	-
Os01g0828900	Similar to predicted protein.	AB512496	NP_563780.1 (RefSeq)	Domain of unknown function DUF640
Os01g0829100	Ankyrin domain containing protein.	AK060644	A2Z5H4	Major sperm protein
Os01g0829183	Non-protein coding transcript.	X06283	NONE	-
Os01g0829266	Hypothetical conserved gene.	AK241659	A2XX31	-
Os01g0829500	Conserved hypothetical protein.	EU962098	Q941V6	-
Os01g0829600	Protein of unknown function DUF688 domain containing protein.	AK060725	Q5QLS4	Protein of unknown function DUF688
Os01g0829700	Hypothetical protein.	AK109789	longestORF	-
Os01g0830000	Similar to Plastid sufB (Fragment).	AK121100	A2WWL8	SUF system FeS cluster assembly, SufBD
Os01g0830100	Pyridine nucleotide-disulphide oxidoreductase, NAD-binding region domain containing protein.	AK069755	XP_002881079.1 (RefS	Pyridine nucleotide-disulphide oxidoreductase, NAD-binding domain
Os01g0831250	Hypothetical gene.	BT054429	longestORF	-
Os01g0831300	Similar to Ammonium transporter.	AK109023	Q8S233	Ammonium transporter
Os01g0831900	Similar to Ammonium transporter.	AK102106	Q8S230	Ammonium transporter
Os01g0831950	Hypothetical protein.	BT054429	longestORF	-
Os01g0835500	Similar to respiratory burst oxidase protein A.	AK068400	B2D0P0	Cytochrome b245, heavy chain
Os01g0836400	Hypothetical conserved gene.	AK068741	Q5QMF1	-
Os01g0836600	ABC transporter-like domain containing protein.	AK067556	NP_181467.1 (RefSeq)	ABC transporter-like
Os01g0836800	Transmembrane receptor, eukaryota domain containing protein.	AK066629 ,AK098958 ,AK11978	NP_001150692.1 (RefS	Transmembrane receptor, eukaryota
Os01g0836900	Conserved hypothetical protein.	AK064231	A2WWR9	-
Os01g0837000	Ankyrin repeat containing protein.	AK243678	A2CIR5	Ankyrin repeat
Os01g0837200	Similar to esterase/lipase/thioesterase family protein.	AK062368	NP_191078.1 (RefSeq)	-
Os01g0837300	Similar to UDP-glucuronic acid decarboxylase 1.	AK104675	NP_001151221.1 (RefS	NAD-dependent epimerase/dehydratase
Os01g0837350	Hypothetical protein.	tplb0036f05 (Wheat FLcDNA)	longestORF	-
Os01g0837600	Similar to plant-specific domain TIGR01589 family protein.	AK108007	NP_001148858.1 (RefS	Conserved hypothetical protein CHP01589, plant
Os01g0837800	Similar to metal tolerance protein.	AK061539	Q5NA18	Cation efflux protein
Os01g0837900	Similar to Protein kinase AFC1 (EC 2.7.1.-).	AK103207	NP_850695.2 (RefSeq)	Protein kinase, catalytic domain
Os01g0839200	Protein of unknown function DUF966 family protein.	AK068926	A2WWT9	Protein of unknown function DUF966
Os01g0849050	Non-protein coding transcript.	DQ185898	NONE	-
Os01g0853000	Conserved hypothetical protein.	AK099561	XP_002458778.1 (RefS	-
Os01g0853700	Similar to MCB1 protein.	AK111988	B7ZY89	SANT domain, DNA binding
Os01g0854400	Conserved hypothetical protein.	AK108330	A2WX36	-
Os01g0855400	SANT domain, DNA binding domain containing protein.	FJ940215	NP_001044841.1 (RefS	SANT domain, DNA binding
Os01g0855600	Similar to Hs1pro-1 protein.	AK099776	Q9FUH4	Hs1pro-1, C-terminal
Os01g0855700	Hypothetical protein.	AK106740	longestORF	-
Os01g0856850	Similar to ribosomal protein S7.	EU957350	NP_039458.1 (RefSeq)	Ribosomal protein S7 domain
Os01g0858350	Similar to cytochrome P450.	AK061128 ,AK100746	XP_002328165.1 (RefS	Cytochrome P450
Os01g0858900	Glycosyl transferase, family 29 protein.	AK107493	NP_001151877.1 (RefS	Glycosyl transferase, family 29
Os01g0863800	Protein of unknown function DUF623, plant domain containing protein.	FP100668	B6TL44	Domain of unknown function DUF623
Os01g0866600	Similar to bolA-like protein.	AK242498	NP_001148455.1 (RefS	BolA protein
Os01g0869900	Similar to Serine/threonine-protein kinase SAPK4.	AK060576	C8CBK4	-
Os01g0872650	Non-protein coding transcript.	CT835515	NONE	-
Os01g0875500	Similar to Beta-galactosidase (EC 3.2.1.23).	AK102715	B6U0W2	D-galactoside/L-rhamnose binding SUEL lectin domain
Os01g0876650	Hypothetical conserved gene.	CT834967	Q654G2	-
Os01g0877500	Hypothetical conserved gene.	AK058582 ,AK064177	A2WXJ6	Zinc finger, PHD-type
Os01g0878000	Conserved hypothetical protein.	AK104289 ,AK060162	A3A062	-
Os01g0878550	Hypothetical protein.	tplb0036o08 (Wheat FLcDNA)	longestORF	-
Os01g0881950	Non-protein coding transcript.	CT834826	NONE	-
Os01g0881966	Hypothetical conserved gene.	AK242526	Q5N7J0	-
Os01g0882900	Hypothetical protein.	tplb0036o08 (Wheat FLcDNA)	longestORF	-
Os01g0883850	Hypothetical protein.	DQ334868	longestORF	-
Os01g0884350	Hypothetical protein.	FP093663	longestORF	-
Os01g0884450	Non-protein coding transcript.	BT084688	NONE	-
Os01g0884850	Hypothetical protein.	BT085496	longestORF	-
Os01g0884900	Similar to DNA glycosylase.	AK063875	XP_003057876.1 (RefS	SNARE associated Golgi protein
Os01g0885500	Hypothetical conserved gene.	AK071788 ,AK106609	Q5N8H2	-
Os01g0886600	Similar to CLP protease regulatory subunit CLPX precursor.	AK070098	B7FAD8	ATPase, AAA+ type, core
Os01g0887100	Similar to 4-(Cytidine 5'-diphospho)-2-C-methyl-D-erythritol synthase.	AK240934	A4PIB9	4-diphosphocytidyl-2C-methyl-D-erythritol synthase
Os01g0890001	Hypothetical conserved gene.	AK242501	B9EVA1	-
Os01g0890050	Hypothetical protein.	tplb0028c09 (Wheat FLcDNA)	longestORF	-
Os01g0890100	Similar to S-domain class receptor-like kinase3.	tplb0056i22 (Wheat FLcDNA)	NP_001105655.1 (RefS	Protein kinase, catalytic domain
Os01g0890500	Conserved hypothetical protein.	ab initio prediction	Q5N832	-
Os01g0892800	Similar to Ankyrin-kinase.	AK070097	Q8SAE5	Protein kinase, catalytic domain
Os01g0894075	Hypothetical protein.	tplb0043a14 (Wheat FLcDNA)	longestORF	-
Os01g0894100	Similar to Transposase (Fragment).	AK111398	NP_001176912.1 (RefS	-
Os01g0895200	DOMON related domain containing protein.	AK063282	A3A0H2	Cytochrome b561, eukaryote
Os01g0900900	Ovarian tumour, otubain domain containing protein.	AK120577	NP_001078307.1 (RefS	Ovarian tumour, otubain
Os01g0901101	Non-protein coding transcript.	BT084794	NONE	-
Os01g0916100	Similar to loricrin.	AK109173	NP_001147500.1 (RefS	-
Os01g0916300	Similar to PQBP-1 protein (Nuclear protein containing a WW domain) (Npw38) (JM26 protein).	AK063922	NP_001147059.1 (RefS	WW/Rsp5/WWP
Os01g0916350	Hypothetical gene.	AK288280	longestORF	-
Os01g0916400	Similar to Selenium binding protein.	AK071710	B6TFC2	Selenium-binding protein
Os01g0916800	Similar to predicted protein.	ab initio prediction	NP_001045197.2 (RefS	THO complex, subunitTHOC2, C-terminal
Os01g0917300	Similar to Cysteine-rich peptide.	AK240939	D7NXT9	-
Os01g0920200	Similar to E(Y)2 homolog (DC6) (Enhancer of yellow 2 homolog).	AK120182	B6T6J7	Transcription factor, enhancer of yellow 2
Os01g0920400	Hypothetical conserved gene.	AK063897	B8A8F0	-
Os01g0920450	Conserved hypothetical protein.	ab initio prediction	B9EVX1	-
Os01g0921550	Hypothetical protein.	ab initio prediction	NONE	-
Os01g0921800	Tetratricopeptide-like helical domain containing protein.	AK334012	XP_002887115.1 (RefS	Zinc finger, MYND-type
Os01g0922000	Hypothetical protein.	AK073034	longestORF	-
Os01g0922700	Conserved hypothetical protein.	AK287528	NP_001172712.1 (RefS	-

Os01g0923200	Similar to AT.I.24-6 protein (Fragment).	AK060288	Q96310	Protein of unknown function DUF3339
Os01g0923600	Hypothetical conserved gene.	AK059687	B8A8F8	IQ motif, EF-hand binding site
Os01g0923700	Similar to Histidine kinase.	AK121135	A1A696	-
Os01g0923750	Hypothetical gene.	BT086966	longestORF	-
Os01g0924100	Non-protein coding transcript.	AK062847	NONE	-
Os01g0924300	Similar to periodic tryptophan protein 1.	AK112027	B6UFL7	WD40 repeat
Os01g0927900	Similar to Aspartate kinase precursor (EC 2.7.2.4).	AK073189	B8A8I6	Aspartate/glutamate/uridylate kinase
Os01g0927950	Hypothetical gene.	BT068036	longestORF	-
Os01g0928000	Similar to Transcription factor ICE1 (Inducer of CBF expression 1) (Basic helix- loop-helix protein 1	AK102594	B6UB60	Helix-loop-helix DNA-binding
Os01g0928800	Similar to Serine palmitoyltransferase.	AK120617	B6SMZ5	Aminotransferase, class-II, pyridoxal-phosphate binding site
Os01g0929000	Similar to aminopeptidase.	AK073334	NP_196898.5 (RefSeq)	Protein of unknown function DUF3754
Os01g0929100	Similar to predicted protein.	AK060605	Q5JK31	-
Os01g0933200	Heavy metal transport/detoxification protein domain containing protein.	ab initio prediction	NP_001147140.1 (RefS	Heavy metal-associated domain, HMA
Os01g0942900	Similar to cDNA clone:J023028M23, full insert sequence.	AK243304	B7E3R6	Ankyrin repeat
Os01g0943400	Hypothetical protein.	ab initio prediction	NONE	-
Os01g0943800	Similar to cDNA, clone: J100054L12, full insert sequence.	AK059510	B7FA83	Ankyrin repeat
Os01g0947500	Conserved hypothetical protein.	Z93114	F2DK87	-
Os01g0947767	Hypothetical gene.	CT828816	longestORF	-
Os01g0947833	Conserved hypothetical protein.	AK062969	NP_001045425.1 (RefS	-
Os01g0958000	Similar to Cyclin-dependent kinase C-2.	AK109352	Q5JK68	Protein kinase, catalytic domain
Os01g0969400	Similar to cation proton exchanger.	BT086324	XP_002310518.1 (RefS	-
Os01g0970400	Eukaryotic translation initiation factor 4E-1 (eIF4E-1) (eIF-4E-1) (mRNA cap-binding protein) (eIF-	AK069207 ,AK099169	NP_001149588.1 (RefS	Translation Initiation factor eIF- 4e
Os01g0970600	Similar to ATP binding protein.	AK288993	NP_001147746.1 (RefS	Helicase, C-terminal
Os01g0970700	Peptidase M48, Ste24p family protein.	AK098940	B6TA12	Peptidase M48
Os01g0971000	Conserved hypothetical protein.	AK061660 ,AK120145	A2WZJ6	-
Os01g0971100	Drought induced 19 family protein.	AK069516	Q5JME8	Drought induced 19/ RING finger protein 114
Os01g0971200	Similar to Lysine ketoglutarate reductase trans-splicing related 1.	AK064075	B4FSQ8	Protein of unknown function DUF707
Os01g0971301	Non-protein coding transcript.	BT016843	NONE	-
Os01g0971600	Similar to Sn-glycerol-3-phosphate dehydrogenase (Fragment).	AK070366	NP_001150493.1 (RefS	Glycerol-3-phosphate dehydrogenase, NAD-dependent, C-terminal
Os02g0100250	Hypothetical protein.	BT083581	longestORF	-
Os02g0114033	Glutamine-Leucine-Glutamine, QLQ domain containing protein.	tplb0057e06 (Wheat FLcDNA)	Q6Z7C5	Glutamine-Leucine-Glutamine, QLQ
Os02g0114066	Non-protein coding transcript.	EU948018	NONE	-
Os02g0121300	Cyclophilin 2.	AK061894 ,AK098919	Q40673	Peptidyl-prolyl cis-trans isomerase, cyclophilin-type
Os02g0121500	Conserved hypothetical protein.	AK065791	Q6YUR3	-
Os02g0121600	Similar to Zinc finger A20 domain-containing stress-associated protein 18.	AK105698	P0C282	-
Os02g0121800	Succinate dehydrogenase, cytochrome b subunit family protein.	AK103260	Q94FP2	Succinate dehydrogenase/Fumarate reductase, transmembrane subunit
Os02g0122000	Similar to P18.	AK121653	B4FIB4	Sin3 associated polypeptide p18
Os02g0122300	Translation initiation factor eIF3 subunit domain containing protein.	AK106553	Q761Z3	Translation initiation factor eIF3 subunit
Os02g0122700	Similar to SET domain protein.	ab initio prediction	XP_002318442.1 (RefS	SET domain
Os02g0122800	Similar to Arginine/serine-rich splicing factor.	AK065148	D8L816	RNA recognition motif domain
Os02g0122900	Similar to Dimethyladenosine transferase.	AK073966	D8L817	Ribosomal RNA adenine methylase transferase
Os02g0123032	Hypothetical gene.	BT016935	longestORF	-
Os02g0123100	Similar to cDNA clone:J033034K16, full insert sequence.	AK064363	B7ERM3	Protein kinase, catalytic domain
Os02g0123300	Eukaryotic translation initiation factor 3 subunit 8, N-terminal domain containing protein.	FN556363	NP_001058804.1 (RefS	Eukaryotic translation initiation factor 3 subunit 8, N-terminal
Os02g0123500	Similar to NADPH-dependent mannose 6-phosphate reductase.	AK070168	D8L838	Aldo/keto reductase
Os02g0124000	Arbuscular mycorrhizal specific marker 31.	EU953683	Q6Z711	Proteinase inhibitor I13, potato inhibitor I
Os02g0124550	Non-protein coding transcript.	FP099177	NONE	-
Os02g0124599	Similar to FAD-binding domain-containing protein.	ab initio prediction	NP_182199.1 (RefSeq)	D-arabinono-1,4-lactone oxidase
Os02g0124800	Conserved hypothetical protein.	AK058547	Q6Z705	-
Os02g0124866	Hypothetical gene.	AK288719	longestORF	-
Os02g0125000	Similar to LPA2 (low psii accumulation2).	AK105873	NP_001032057.1 (RefS	-
Os02g0128501	Non-protein coding transcript.	BT016330	NONE	-
Os02g0128600	Similar to ADP-ribosylation factor-like protein.	AK059865 ,AK103009	B6TGE5	Small GTP-binding protein domain
Os02g0128800	TRAF-type domain containing protein.	AK111795	NP_001123598.1 (RefS	Zinc finger, RING-type
Os02g0130700	Hypothetical genes.	ab initio prediction	NP_001172785.1 (RefS	-
Os02g0130800	F-box domain, cyclin-like domain containing protein.	EU975717	B9F2A0	F-box domain, cyclin-like
Os02g0130900	Ubiquitin-conjugating enzyme E2C-binding protein domain containing protein.	AK102856	A2X0I7	Ubiquitin-conjugating enzyme E2C-binding protein
Os02g0131000	Conserved hypothetical protein.	AK062905	A2X0I8	-
Os02g0131050	Hypothetical gene.	AK242812	longestORF	-
Os02g0131100	Hypothetical conserved gene.	AK105854	B9F2A1	-
Os02g0131300	Non-protein coding transcript.	AK061919	NONE	-
Os02g0131850	Hypothetical gene.	BT086902	longestORF	-
Os02g0132100	Pentatricopeptide repeat domain containing protein.	ab initio prediction	NP_001045795.1 (RefS	Pentatricopeptide repeat
Os02g0132300	Similar to Erythrocyte membrane protein PFEMP3 (Fragment).	AK070078	NP_001151550.1 (RefS	Zinc finger, RING-CH-type
Os02g0132950	Hypothetical gene.	AK241678	longestORF	-
Os02g0133000	Cyclin-T1-1.	Q0E474	Q0E474	Cyclin, N-terminal
Os02g0133100	Similar to Phosphatidylinositol transfer-like protein III.	AK060180	Q94FN1	Cellular retinaldehyde-binding/triple function, C-terminal
Os02g0133200	Similar to Phosphatidylinositol transfer-like protein IV.	AK100898 ,AK072646	NP_568006.1 (RefSeq)	Cellular retinaldehyde binding/alpha-tocopherol transport
Os02g0133800	Proteasome subunit alpha type 1 (EC 3.4.25.1) (20S proteasome alpha subunit F) (20S proteasome s	AK103646 ,AK109376	B8AHG2	Proteasome, alpha-subunit, conserved site
Os02g0133900	Protein of unknown function DUF829, eukaryotic family protein.	AK107180	NP_001130250.1 (RefS	Protein of unknown function DUF829, TMEM53
Os02g0134000	Similar to bifunctional coenzyme A synthase.	AK355133	NP_001147323.1 (RefS	-
Os02g0134100	Hypothetical gene.	AK069055	longestORF	-
Os02g0134200	Protein of unknown function DUF1645 family protein.	AK108331	A2X0L4	Protein of unknown function DUF1645
Os02g0134300	Protein of unknown function DUF701, zinc-binding putative family protein.	AK120292	B6SX26	Protein of unknown function DUF701, zinc-binding putative
Os02g0134400	Fumarate reductase/succinate dehydrogenase flavoprotein, C-terminal domain containing protein.	AK072394	B6U0K6	Fumarate reductase/succinate dehydrogenase flavoprotein, N-terminal
Os02g0135000	Conserved hypothetical protein.	AK069693	A2X0L9	-
Os02g0135200	Similar to Mitogen-activated protein kinase 13.	CT836561	Q0E459	Protein kinase, catalytic domain
Os02g0135250	Hypothetical protein.	tplb0059e09 (Wheat FLcDNA)	longestORF	-
Os02g0135300	Similar to Wee1-like protein kinase.	AK372043	Q6Z829	Protein kinase, catalytic domain
Os02g0135600	Conserved hypothetical protein.	AK069843	A2X0M4	-
Os02g0135700	DNA polymerase V family protein.	AK100570	B9F2L5	DNA polymerase V
Os02g0135800	Similar to Sec13-like protein (Fragment).	AK112028	Q8W403	WD40 repeat
Os02g0135850	Hypothetical conserved gene.	ab initio prediction	B9F2L6	Ubiquitin system component Cue
Os02g0135900	Similar to 5'-AMP-activated protein kinase beta-1 subunit-related.	AK072017	NP_568573.2 (RefSeq)	-
Os02g0135950	Non-protein coding transcript.	BT016931	NONE	-
Os02g0136150	Hypothetical gene.	CT835435	longestORF	-
Os02g0136400	Conserved hypothetical protein.	ab initio prediction	B8AHD1	-
Os02g0136850	Non-protein coding transcript.	CU406396	NONE	-
Os02g0136900	Protein kinase, core domain containing protein.	AK111638	B8AHD4	Protein kinase, catalytic domain
Os02g0136933	Hypothetical conserved gene.	ab initio prediction	B9F2M0	WD40 repeat-like-containing domain
Os02g0136966	Hypothetical gene.	AK288412	longestORF	-
Os02g0137000	WD40 repeat-like domain containing protein.	AK111719	XP_002875574.1 (RefS	WD40 repeat
Os02g0137100	Similar to plant-specific domain TIGR01589 family protein.	AK073353	NP_001148712.1 (RefS	Conserved hypothetical protein CHP01589, plant
Os02g0137200	Similar to 50S ribosomal protein L3-1, chloroplast precursor.	AK099849	B6UF84	Ribosomal protein L3
Os02g0137400	Similar to Spliceosomal-like protein.	AK068480	Q9LD60	Cleavage/polyadenylation specificity factor, A subunit, C-terminal
Os02g0137450	Conserved hypothetical protein.	AK369368	B9F2M2	-
Os02g0137500	Similar to histone acetyltransferase.	AK241505	XP_002330477.1 (RefS	Zinc finger, TAZ-type
Os02g0137600	Bacterial methyltransferase family protein.	AK102168	NP_196652.1 (RefSeq)	S-adenosyl-L-methionine-dependent methyltransferase, MraW
Os02g0137700	NAD(P)-binding domain containing protein.	AK241047	XP_002890185.1 (RefS	NmrA-like
Os02g0150600	Similar to Pyridine nucleotide-disulphide oxidoreductase (Fragment).	AK105844	B9F2R3	-
Os02g0150700	Zinc finger, RING-type domain containing protein.	AK120070	B9F2R3	Zinc finger, RING-type
Os02g0150900	Protein of unknown function DUF1644 family protein.	AK121424	Q67UW9	Zinc finger, C2H2
Os02g0151100	Hypothetical conserved gene.	AK243362	Q67UW6	Protein kinase, catalytic domain
Os02g0151300	Similar to Cellulase (Fragment).	AK120536	XP_002321149.1 (RefS	Glycoside hydrolase, family 9
Os02g0151350	Hypothetical protein.	tplb0042g22 (Wheat FLcDNA)	longestORF	-
Os02g0151501	Similar to SUI1 protein.	GU565696	Q84LW9	Translation initiation factor SUI1
Os02g0151600	Ketol-acid reductoisomerase, chloroplast precursor (EC 1.1.1.86) (Acetohydroxy-acid reductoisomera	ab initio prediction	B6TF69	Acetohydroxy acid isomeroreductase
Os02g0152200	Protein of unknown function DUF1677, Oryza sativa family protein.	AK070155	A2X0Z5	Protein of unknown function DUF1677, Oryza sativa
Os02g0152300	Serine/threonine protein kinase-related domain containing protein.	AK069368	B8AHT6	Protein kinase, catalytic domain
Os02g0152400	Similar to Ribulose bisphosphate carboxylase (EC 4.1.1.39) (Fragment).	AK059909	B8AHT7	Ribulose bisphosphate carboxylase small chain, domain
Os02g0152500	Non-protein coding transcript.	AK062072	NONE	-
Os02g0152700	Similar to DNA-directed RNA polymerase.	AK063373	B9F2S6	Copper amine oxidase, N2/N3-terminal
Os02g0152750	Non-protein coding transcript.	EU944670	NONE	-
Os02g0152900	Conserved hypothetical protein.	AK061136	Q67IU2	-
Os02g0156800	Leucine-rich repeat-containing N-terminal, type 2 domain containing protein.	AK067358	B9F2U1	Leucine-rich repeat-containing N-terminal, type 2

Os02g0157000	Leucine-rich repeat domain containing protein.	AK371462	NP_001172810.1 (RefS	Leucine-rich repeat
Os02g0157100	Leucine-rich repeat domain containing protein.	AK371462	XP_002453319.1 (RefS	Leucine-rich repeat
Os02g0157501	Leucine-rich repeat domain containing protein.	AK369574	longestORF	Leucine-rich repeat
Os02g0158600	Conserved hypothetical protein.	AK066467	A2X145	-
Os02g0161000	Similar to Serine/threonine protein kinase-like protein.	AK111660	Q6H7U5	Protein kinase, catalytic domain
Os02g0161100	Actin-binding FH2 domain containing protein.	AK072377	Q6H7U3	Actin-binding FH2/DRF autoregulatory
Os02g0161251	Hypothetical gene.	AK288500	longestORF	-
Os02g0161300	Similar to Mot1 (Fragment).	AK070938	C6ERB4	Domain of unknown function DUF3535
Os02g0161900	Similar to polyubiquitin containing 7 ubiquitin monomers.	AK101547 ,AK061956	NP_001147027.1 (RefS	Ubiquitin
Os02g0162050	Non-protein coding transcript.	BT016940	NONE	-
Os02g0162150	Hypothetical protein.	ab initio prediction	NONE	-
Os02g0162600	Conserved hypothetical protein.	AK242133	NP_001045983.1 (RefS	-
Os02g0162700	Non-protein coding transcript.	AK058629	NONE	-
Os02g0162783	Non-protein coding transcript.	EU943604	NONE	-
Os02g0163533	Conserved hypothetical protein.	AK241536	NP_001172814.1 (RefS	-
Os02g0163600	Conserved hypothetical protein.	AK068043	B8AI72	-
Os02g0164800	Zinc finger, C2H2-type domain containing protein.	AK062817	NP_175482.1 (RefSeq)	-
Os02g0164900	Similar to Auxin response factor 3.	AK070569	Q6H6V4	AUX/IAA protein
Os02g0165000	Zinc finger, RING/FYVE/PHD-type domain containing protein.	AK069562	B8AI80	Zinc finger, RING-CH-type
Os02g0165100	Protein kinase, core domain containing protein.	AK066645	NP_001148145.1 (RefS	Protein kinase, catalytic domain
Os02g0165500	Conserved hypothetical protein.	AK060547	A2X1A6	-
Os02g0165800	Conserved hypothetical protein.	AK106983	A2X1A7	-
Os02g0166501	Hypothetical conserved gene.	CT835482	Q6H4W8	-
Os02g0166600	Hypothetical protein.	AK069371	longestORF	-
Os02g0166800	Protein of unknown function DUF640 domain containing protein.	AK106854	D1MYE3	Domain of unknown function DUF640
Os02g0166875	Hypothetical protein.	EU970153	longestORF	-
Os02g0166950	Hypothetical gene.	AK243419	longestORF	-
Os02g0167000	Similar to holocarboxylase synthetase 1.	AK375247	XP_002878877.1 (RefS	Biotin protein ligase, C-terminal
Os02g0167100	Similar to 3-mercaptopyruvate sulfurtransferase precursor (EC 2.8.1.1).	AK066425	Q94C43	Thiosulphate sulfurtransferase, conserved site
Os02g0167200	Pentatricopeptide repeat domain containing protein.	AK106491	Q6H4W1	Pentatricopeptide repeat
Os02g0167300	Similar to Tubulin beta-5 chain.	AK061659 ,AK119164	P46265	Tubulin
Os02g0167400	Non-protein coding transcript.	X06283	NONE	-
Os02g0167500	RNA recognition motif domain domain containing protein.	AK069161	B9F385	RNA recognition motif domain
Os02g0167600	Hypothetical gene.	AK242059	longestORF	-
Os02g0167850	Non-protein coding transcript.	CT835346	NONE	-
Os02g0168100	Similar to 4-hydroxyphenylpyruvate dioxygenase.	AK058890	O48604	Glyoxalase/fosfomycin resistance/dioxygenase
Os02g0168550	Non-protein coding transcript.	tplb0046d03 (Wheat FLcDNA)	NONE	-
Os02g0168600	Ovarian tumour, otubain domain containing protein.	AK067291	B6U3W2	Ovarian tumour, otubain
Os02g0168700	Peptidyl-prolyl cis-trans isomerase, FKBP-type domain containing protein.	AK061562	NP_001148684.1 (RefS	Peptidyl-prolyl cis-trans isomerase, FKBP-type, domain
Os02g0169000	Similar to T6H22.2 protein.	AK101628	Q9SGU5	-
Os02g0169151	Hypothetical gene.	BT016355	longestORF	-
Os02g0169275	Non-protein coding transcript.	FP098893	NONE	-
Os02g0169300	Similar to Phosphoglycerate kinase, cytosolic (EC 2.7.2.3).	AK070041	B8AIH2	Phosphoglycerate kinase
Os02g0169400	Similar to Argonaute 4 protein.	AK106247	Q6H6C3	Argonaute/Dicer protein, PAZ
Os02g0169450	Non-protein coding transcript.	BT016774	NONE	-
Os02g0169900	Inositol monophosphatase family protein.	AK105887 ,AK070107	NP_564376.1 (RefSeq)	Inositol monophosphatase
Os02g0170000	Conserved hypothetical protein.	AK065022	A3A3K8	-
Os02g0174900	Hypothetical gene.	AK121973	longestORF	-
Os02g0175000	Ion transporter , Na+ transport	AJ491818	Q6H501 ,Q0E3H2	-
Os02g0175100	Similar to RISBZ4.	AK069575	Q9AVD0	Basic-leucine zipper
Os02g0175200	Domain of unknown function DUF632 domain containing protein.	ab initio prediction	Q6H4Z9	Domain of unknown function DUF632
Os02g0175250	Hypothetical protein.	AM939967	longestORF	-
Os02g0175800	Similar to fiber protein Fb15.	AK120215	NP_001150164.1 (RefS	-
Os02g0175850	Non-protein coding transcript.	BT017226	NONE	-
Os02g0177666	Hypothetical protein.	BT017473	longestORF	-
Os02g0180400	GCN5-related N-acetyltransferase (GNAT) domain domain containing protein.	AK355812	Q6H820	GCN5-related N-acetyltransferase
Os02g0190800	Alpha/beta hydrolase fold-1 domain containing protein.	AK099853	NP_001151903.1 (RefS	-
Os02g0204000	Tetratricopeptide-like helical domain containing protein.	AK066727	NP_001147153.1 (RefS	Tetratricopeptide-like helical
Os02g0213100	Conserved hypothetical protein.	AK062445	Q6H826	-
Os02g0220050	Hypothetical protein.	AK375091	longestORF	-
Os02g0220100	Similar to Cytokinin dehydrogenase 7.	GU160403	Q6YW50	Oxygen oxidoreductase covalent FAD-binding site
Os02g0226300	Mitochondrial carrier protein domain containing protein.	ab initio prediction	B8AEI5	Mitochondrial carrier protein
Os02g0226350	Non-protein coding transcript.	AK242418	NONE	-
Os02g0230300	En/Spm-like transposon protein (Protodermal factor 1).	AK058752	Q56UU6	-
Os02g0258000	Hypothetical conserved gene.	CT835450	Q6ETP6	-
Os02g0258200	Similar to high mobility group family.	AK070270	XP_002303636.1 (RefS	High mobility group, HMG1/HMG2
Os02g0258250	Hypothetical conserved gene.	CT837539	XP_002299486.1 (RefS	High mobility group, HMG1/HMG2
Os02g0258300	Similar to extra-large G-protein-related.	AK104067	NP_192018.1 (RefSeq)	-
Os02g0260800	Non-protein coding transcript.	EU941912	NONE	-
Os02g0260900	Similar to H0315A08.1 protein.	FP099421	Q01I63	Ribonuclease H1, N-terminal
Os02g0268400	Similar to Expansin-A23.	DQ061064	Q4PR43	Expansin
Os02g0268500	Hypothetical protein.	BT064922	longestORF	-
Os02g0305950	Similar to calmodulin binding protein.	tplb0019a16 (Wheat FLcDNA)	NP_001149909.1 (RefS	Auxin responsive SAUR protein
Os02g0306801	Hypothetical gene.	EU943056	longestORF	-
Os02g0306900	Similar to DnaJ-like protein.	AK100011 ,AK070441	A2Q505	Heat shock protein DnaJ, N-terminal
Os02g0508650	Non-protein coding transcript.	FP094256	NONE	-
Os02g0508801	Similar to predicted protein.	EU959920	Q6K2G2	Protein of unknown function DUF3339
Os02g0519900	Similar to H0613H07.5 protein.	AK060953	Q01MK8	Translation elongation factor EFG/EF2, C-terminal
Os02g0521300	C2 domain containing protein.	AK120851	NP_001147038.1 (RefS	C2 calcium-dependent membrane targeting
Os02g0521366	Similar to HAT family dimerisation domain containing protein.	tplb0053n06 (Wheat FLcDNA)	Q7XE06	Zinc finger, BED-type predicted
Os02g0521432	Hypothetical protein.	tplb0030l08 (Wheat FLcDNA)	longestORF	-
Os02g0521500	Peptidase C65, otubain domain containing protein.	AK335298	B9F0A6	Peptidase C65, otubain
Os02g0521600	Ovarian tumour, otubain domain containing protein.	AK362134	B9F0A7	Ovarian tumour, otubain
Os02g0549467	Non-protein coding transcript.	EU947769	NONE	-
Os02g0557800	A-type response regulator, Cytokinin signaling	AK070645 ,AB249662 ,BR00031	Q6YVX7	Signal transduction response regulator, receiver domain
Os02g0561500	Monooxygenase, FAD-binding domain containing protein.	AK367305	Q6YU31	Monooxygenase, FAD-binding
Os02g0562300	Hypothetical conserved gene.	AK104737 ,AK061441	B8ADY6	Calmodulin binding protein-like
Os02g0562350	Non-protein coding transcript.	EU949450	NONE	-
Os02g0563000	Hypothetical conserved gene.	BT083559	Q6ZGB6	F-box domain, cyclin-like
Os02g0563800	Cyclin-like F-box domain containing protein.	AK067547	A2Y6M3	F-box domain, cyclin-like
Os02g0564200	Conserved hypothetical protein.	AK072445	A2X661	-
Os02g0564300	Conserved hypothetical protein.	AK242358	NP_001047159.1 (RefS	-
Os02g0564400	Similar to CLPX (Clp protease regulatory subunit X)%3B ATPase.	AK243408	E5GBA0	ATPase, AAA+ type, core
Os02g0564500	SFT2-like family protein.	AK103450	B6TQJ1	Vesicle transport protein, Got1/SFT2-like
Os02g0564600	Late embryogenesis abundant protein 3 family protein.	AK318610	NP_001147317.1 (RefS	Late embryogenesis abundant protein, LEA-5
Os02g0565000	Homeodomain-like containing protein.	AK120665	NP_001151900.1 (RefS	-
Os02g0565500	Similar to Pto kinase interactor 1.	AK072522	Q709M1	Protein kinase, catalytic domain
Os02g0565600	Similar to Homeodomain leucine zipper protein (Fragment).	AK064118	Q0E0A6	Homeobox
Os02g0567000	Conserved hypothetical protein.	AK068282	B8AEC4	-
Os02g0567200	Protein phosphatase 2C domain containing protein.	AK069840	NP_001151313.1 (RefS	Protein phosphatase 2C-like
Os02g0567450	Hypothetical gene.	BT085317	GeneMark	-
Os02g0568600	Hypothetical conserved gene.	ab initio prediction	Q6YTF9	-
Os02g0583300	Similar to OSIGBa0132O24.3 protein.	EU951279	Q01MB4	-
Os02g0583500	Transposase, Ptta/En/Spm, plant domain containing protein.	EU976982	NP_001047258.2 (RefS	Transposase, Ptta/En/Spm, plant
Os02g0585200	Heavy metal transport/detoxification protein domain containing protein.	AK287653	NP_001152411.1 (RefS	Heavy metal-associated domain, HMA
Os02g0587600	Conserved hypothetical protein.	ab initio prediction	NP_001047273.1 (RefS	-
Os02g0587800	Virulence factor, pectin lyase fold family protein.	AK243611	NP_001173047.1 (RefS	-
Os02g0588300	Similar to Fimbrin 1 (AtFIM1).	AK119439	NP_001147889.1 (RefS	Actinin-type, actin-binding, conserved site
Os02g0590200	Lecithin:cholesterol/phospholipid:diacylglycerol acyltransferase domain containing protein.	AK241245	NP_001047281.2 (RefS	Lecithin:cholesterol/phospholipid:diacylglycerol acyltransferase
Os02g0591850	Non-protein coding transcript.	AK242359	NONE	-
Os02g0592000	Similar to OSIGBa0106G07.8 protein.	AK361746	Q01IX6	Oxoglutarate/iron-dependent oxygenase
Os02g0592300	Similar to predicted protein.	AK073685	NP_567236.1 (RefSeq)	DNA mismatch repair protein
Os02g0592500	Folate receptor, conserved region domain containing protein.	AK070526	B9F0U2	Folate receptor-like
Os02g0592600	Phospholipase C, phosphatidylinositol-specific, Y domain domain containing protein.	AK099683	A2WUV9	Phospholipase C, phosphatidylinositol-specific, Y domain
Os02g0592700	Conserved hypothetical protein.	AK107229	A2X6P0	-

Os02g0593400	Conserved hypothetical protein.	AK064226	XP_002454106.1 (RefS	-
Os02g0593500	Phosphate transporter family protein.	AK067498	Q7YME0	Phosphate transporter
Os02g0593550	Hypothetical gene.	EU972267	longestORF	-
Os02g0593600	Leucine-rich repeat, typical subtype containing protein.	AK069976	Q01IY1	Leucine-rich repeat
Os02g0593700	Similar to TA4 protein (Fragment).	AK072838	B8K1X5	Phox/Bem1p
Os02g0593900	Methyltransferase-related domain containing protein.	AK288086	B6TN81	Methyltransferase-related
Os02g0594100	Similar to Protein kinase ATN1.	AK067412	Q7XJ65	Protein kinase, catalytic domain
Os02g0594300	Similar to Enhancer of shoot regeneration ESR1.	EU946638	NP_001147345.1 (RefS	Pathogenesis-related transcriptional factor/ERF, DNA-binding
Os02g0598500	Protein phosphatase 2C family protein.	AK106903	NP_001047333.1 (RefS	Protein phosphatase 2C, manganese/magnesium aspartate binding site
Os02g0598800	Telomere length regulation protein, conserved domain domain containing protein.	AK372629	NP_001047336.2 (RefS	Telomere length regulation protein, conserved domain
Os02g0599100	Hypothetical conserved gene.	tplb0009a09 (Wheat FLcDNA)	Q6K1U1	Telomere length regulation protein, conserved domain
Os02g0644400	Similar to Kinesin heavy chain (Fragment).	ab initio prediction	Q93XG3	Kinesin, motor domain
Os02g0658800	Beta-expansin.	AK059638	Q6H676	Major pollen allergen Lol pI
Os02g0658850	Hypothetical protein.	EU947703	longestORF	-
Os02g0662300	Conserved hypothetical protein.	AK121727	B8AGC6	-
Os02g0664200	Similar to UDP-glucose:glycoprotein glucosyltransferase 1 precursor (EC 2.4.1.-) (UDP-glucose cera	AK059298	NP_177278.3 (RefSeq)	Glycosyl transferase, family 8
Os02g0666250	Hypothetical gene.	EU947829	longestORF	-
Os02g0666550	Hypothetical protein.	ab initio prediction	NONE	-
Os02g0667100	Conserved hypothetical protein.	AK370258	NP_001047679.1 (RefS	-
Os02g0668000	Hypothetical protein.	AK108411	GeneMark	-
Os02g0668100	Similar to Geranylgeranyl-diphosphate synthase.	AK111401	A9ZN21	Polyprenyl synthetase
Os02g0668900	Similar to H0211B05.3 protein.	ab initio prediction	Q01JA9	Lipase, GDSL
Os02g0669100	Dehydrin family protein.	AK070197	B1NEV7	Dehydrin
Os02g0669366	Hypothetical gene.	EU942482	longestORF	-
Os02g0669500	Similar to H0211B05.7 protein.	AK111117	Q01JA5	Protein of unknown function DUF241, plant
Os02g0672400	Sugar transporter, conserved site domain containing protein.	AK064365	B8AGF1	-
Os02g0674050	Non-protein coding transcript.	EU970724	NONE	-
Os02g0679200	Pentatricopeptide repeat domain containing protein.	AK110789	B8AGT4	Pentatricopeptide repeat
Os02g0679700	Protein of unknown function DUF623, plant domain containing protein.	AK108178	B8AGP6	Domain of unknown function DUF623
Os02g0680500	Pentatricopeptide repeat domain containing protein.	AK106401	B8AGP8	Pentatricopeptide repeat
Os02g0681100	Protein of unknown function DUF604 family protein.	AK100584	Q01KI0	Protein of unknown function DUF604
Os02g0682700	Similar to protein binding protein.	ab initio prediction	NP_001150294.1 (RefS	-
Os02g0683100	Hypothetical protein.	tplb0021g13 (Wheat FLcDNA)	longestORF	-
Os02g0683500	Similar to DNA-binding protein RAV1.	AK109908	D9MNL6	Transcriptional factor B3
Os02g0684300	Nucleoporin protein Ndc1-Nup domain containing protein.	AK108514	A3AA67	Nucleoporin protein Ndc1-Nup
Os02g0685350	Hypothetical gene.	EU951957	longestORF	-
Os02g0691650	Hypothetical gene.	EU948983	longestORF	-
Os02g0695900	Similar to oligopeptide transporter 4.	AK062910	NP_001152089.1 (RefS	Oligopeptide transporter OPT superfamily
Os02g0696200	Hypothetical protein.	AK353783	longestORF	-
Os02g0697400	Non-protein coding transcript.	AK060627	NONE	-
Os02g0704262	Conserved hypothetical protein.	CT835588	B9GCT9	-
Os02g0717550	Non-protein coding transcript.	BT018944	NONE	-
Os02g0718100	Non-protein coding transcript.	AK109216	NONE	-
Os02g0720000	Conserved hypothetical protein.	AK108806	Q6ZI16	-
Os02g0720100	Cupredoxin domain containing protein.	AK063724	A3AAS9	Plastocyanin-like
Os02g0720250	Non-protein coding transcript.	tplb0026b06 (Wheat FLcDNA)	NONE	-
Os02g0720450	Hypothetical protein.	EU947645	longestORF	-
Os02g0720500	Peptidase A1 domain containing protein.	AK108932	B6SL29	Peptidase A1
Os02g0720900	Similar to Aspartic proteinase nepenthesin-1.	AK072228	B6TP02	Peptidase A1
Os02g0721000	Hypothetical protein.	AK108039	longestORF	-
Os02g0721100	Similar to E2 ubiquitin-conjugating enzyme UbcH5B (Fragment).	AK108167	A2X916	Ubiquitin-conjugating enzyme, E2
Os02g0725300	Similar to cDNA clone:J033042D19, full insert sequence.	AK111344	B7ERT8	-
Os02g0725600	tRNA (guanine-N1-)-methyltransferase, eukaryotic domain containing protein.	AK058341	A2Q1R6	tRNA
Os02g0726300	Similar to AOBP (Ascorbate oxidase promoter-binding protein).	AK101321 ,AK100892	Q58FK8	Zinc finger, Dof-type
Os02g0731600	Similar to Threonine endopeptidase.	AK058271	B6TCN7	-
Os02g0735900	Zinc finger, RING/FYVE/PHD-type domain containing protein.	AK072424	B6THV3	Zinc finger, RING-type
Os02g0736700	Conserved hypothetical protein.	EU945911	NP_001048056.1 (RefS	-
Os02g0736800	Conserved hypothetical protein.	AK073311	B8AI34	-
Os02g0737401	Hypothetical conserved gene.	AK241122	Q6Z733	-
Os02g0738100	Conserved hypothetical protein.	AK241905	NP_001173146.1 (RefS	-
Os02g0744150	Hypothetical gene.	BT016845	longestORF	-
Os02g0745250	Similar to NAC domain class transcription factor.	AY742218	D9ZJ98	No apical meristem
Os02g0745300	Conserved hypothetical protein.	ab initio prediction	Q6ZGU0	-
Os02g0748100	Hypothetical conserved gene.	AK111248	Q6YUW6	-
Os02g0748300	Similar to Kelch motif family protein.	AK100919	B6SXJ9	Kelch repeat type 1
Os02g0750400	Pentatricopeptide repeat domain containing protein.	AK059134	A3ABE1	Pentatricopeptide repeat
Os02g0753100	Non-protein coding transcript.	BT086933	NONE	-
Os02g0755450	Hypothetical gene.	EU941442	longestORF	-
Os02g0762400	Cyclin-dependent kinase inhibitor family protein.	AK103084	Q6Z6G5	Cyclin-dependent kinase inhibitor
Os02g0768800	Similar to predicted protein.	AK063192	E5GCG2	-
Os02g0768950	Non-protein coding transcript.	CU405652	NONE	-
Os02g0785000	Glycosyl transferase, family 31 protein.	AK103670	NP_200558.1 (RefSeq)	Glycosyl transferase, family 31
Os02g0787000	Allergen V5/Tpx-1 related family protein.	ab initio prediction	NP_001048338.1 (RefS	Allergen V5/Tpx-1-related
Os02g0796750	Non-protein coding transcript.	EU943327	NONE	-
Os02g0800500	Similar to Cyclin-B1-2.	AK099813	Q0DWQ7	Cyclin, N-terminal
Os02g0801200	Similar to Cyclin-B1-2.	AK069086	Q0DWQ7	-
Os02g0801210	Non-protein coding transcript.	CT831260	NONE	-
Os02g0801255	Similar to cDNA clone:J013118P06, full insert sequence.	ab initio prediction	B7EE20	-
Os02g0801300	Similar to Cyclin-B1-2.	AK069086	Q0DWQ7	-
Os02g0801450	Similar to OSJNba0093F12.23 protein.	AK065428	Q5JQR9	-
Os02g0807600	Hypothetical gene.	AK062825	GeneMark	-
Os02g0811101	Conserved hypothetical protein.	FP095473	NP_001147425.1 (RefS	-
Os02g0813183	Hypothetical protein.	tplb0043a14 (Wheat FLcDNA)	longestORF	-
Os02g0813200	Myb/SANT-like domain domain containing protein.	EU975340	NP_001048483.2 (RefS	Myb/SANT-like domain
Os02g0814900	Similar to nicotinamide-nucleotide adenylyltransferase 1.	ab initio prediction	NP_001150381.1 (RefS	Cytidylyltransferase
Os02g0823500	Non-protein coding transcript.	U16257	NONE	-
Os02g0824400	Conserved hypothetical protein.	AK121390	B8AF55	-
Os02g0824500	Similar to Remorin.	AK111296	NP_001159012.1 (RefS	Remorin, C-terminal
Os02g0826016	Hypothetical protein.	tplb0042k12 (Wheat FLcDNA)	longestORF	-
Os02g0826533	Non-protein coding transcript.	tplb0033e11 (Wheat FLcDNA)	NONE	-
Os02g0826566	F-box domain, cyclin-like domain containing protein.	AK367617	B8AF61	F-box domain, cyclin-like
Os02g0826700	F-box domain, cyclin-like domain containing protein.	ab initio prediction	Q6K7R1	F-box domain, cyclin-like
Os02g0827300	Protein of unknown function DUF382 domain containing protein.	AK069159	A2Q200	PSP, proline-rich
Os02g0827400	Similar to predicted protein.	CT835495	NP_001048593.1 (RefS	Protein of unknown function DUF789
Os02g0827500	Hypothetical conserved gene.	AK242677	Q6K7Q5	Domain of unknown function DUF2404
Os02g0830750	Hypothetical protein.	BT086967	longestORF	-
Os03g0100010	Conserved hypothetical protein.	AK333943	A2XBE9	-
Os03g0100020	Similar to predicted protein.	AK073982	NP_199482.1 (RefSeq)	Tetraspanin, subgroup
Os03g0100050	Inner nuclear membrane protein MAN1 domain containing protein.	AK066383	Q10T70	Inner nuclear membrane protein MAN1
Os03g0100225	Non-protein coding transcript.	EU949846	NONE	-
Os03g0100300	Peptidyl-prolyl cis-trans isomerase, cyclophilin-type domain containing protein.	AK106235	NP_567522.5 (RefSeq)	Peptidyl-prolyl cis-trans isomerase, cyclophilin-type
Os03g0100400	Similar to DNA repair endonuclease UVH1 (EC 3.1.-.-) (Ultraviolet hypersensitive 1) (AtRAD1) (D	AK068556 ,AK121880	NP_198931.1 (RefSeq)	ERCC4 domain
Os03g0100900	Protein of unknown function DUF862, eukaryotic domain containing protein.	AK103335	B4FKS4	Domain of unknown function DUF862, eukaryotic
Os03g0101100	Similar to Palmitoyl-protein thioesterase-like.	AK120839	Q10T54	Palmitoyl protein thioesterase
Os03g0101200	Similar to U-box domain containing protein, expressed.	ab initio prediction	Q10T52	Protein kinase, catalytic domain
Os03g0101250	Hypothetical gene.	AK241727	longestORF	-
Os03g0101300	Similar to Hexose transporter.	AK100524	NP_001149551.1 (RefS	Sugar/inositol transporter
Os03g0101400	Hypothetical protein.	AK120679	longestORF	-
Os03g0101500	Similar to Phosphoglycerate mutase family protein.	AK109299	C3SAD0	PRIB5
Os03g0101600	Similar to Oxidoreductase, zinc-binding dehydrogenase family protein, expressed.	AK061143 ,AK069374	Q10T48	Alcohol dehydrogenase superfamily, zinc-type
Os03g0102000	Conserved hypothetical protein.	AK062867	B9FA98	-
Os03g0102100	Similar to formyltetrahydrofolate deformylase.	AK071358	NP_001152471.1 (RefS	Formyl transferase, N-terminal
Os03g0102200	Similar to DNA-directed RNA polymerase II 14.5 kDa polypeptide (EC 2.7.7.6) (RPB9) (RPB14.5).	AK120183	Q10T39	Zinc finger, TFIIS-type
Os03g0108400	Similar to Charged multivesicular body protein 3.	AK242193	B6SM72	Snf7
Os03g0108500	Similar to 4,4-dimethyl-sterol C4-methyl-oxidase (Fragment).	AK108704	B4FN64	Fatty acid hydroxylase

Os03g0108600	Similar to DEAD-box ATP-dependent RNA helicase 50.	AK065776	Q8GUG7	Helicase, C-terminal
Os03g0108700	Conserved hypothetical protein.	AK105115	Q8H7P7	-
Os03g0109350	Non-protein coding transcript.	AK374302	NONE	-
Os03g0109650	Hypothetical conserved gene.	AK289246 ,AK243040	Q8H7V1	Protein of unknown function DUF3755
Os03g0109800	Hypothetical conserved gene.	AK066015	Q8H7V3	-
Os03g0110400	Similar to nuclear matrix protein-related.	AK100395	NP_568219.1 (RefSeq)	THO complex, subunit THOC1
Os03g0110900	Dimeric alpha-beta barrel domain containing protein.	AK070403	Q10SU2	Dimeric alpha-beta barrel
Os03g0111000	Conserved hypothetical protein.	AK062759	B8ALG1	-
Os03g0111100	Similar to Dihydrofolate synthetase /folylpolyglutamate synthetase.	AK102025	B6SW32	Folylpolyglutamate synthetase
Os03g0111500	Hypothetical conserved gene.	AK067241	Q10ST5	-
Os03g0112400	Clathrin/coatomer adaptor, adaptin-like, appendage, C-terminal subdomain domain containing protei	AK063082	Q10SS6	Clathrin adaptor, alpha-adaptin, appendage, C-terminal subdomain
Os03g0112600	Similar to Plus-3 domain containing protein, expressed.	EU942983	Q7XCX5	SWIB/MDM2 domain
Os03g0113000	Serine/threonine protein kinase domain containing protein.	AK071309	NP_001151054.1 (RefS	Protein kinase, catalytic domain
Os03g0113100	Similar to Thymidine kinase.	AK066124	O81263	Thymidine kinase
Os03g0113500	Conserved hypothetical protein.	AK241800	B9FAE9	-
Os03g0113800	Tetratricopeptide-like helical domain containing protein.	AK065925	XP_002870606.1 (RefS	Tetratricopeptide-like helical
Os03g0114850	Non-protein coding transcript.	BT063951	NONE	-
Os03g0117050	Non-protein coding transcript.	AK241608	NONE	-
Os03g0121300	Similar to Peroxidase 1.	AK064633	Q8LMR7	Plant peroxidase
Os03g0122034	Non-protein coding transcript.	EU950997	NONE	-
Os03g0124300	Similar to ATP binding protein.	AK106076	B6TWY5	Malectin-like carbohydrate-binding domain
Os03g0127000	Similar to cell growth defect factor 2.	AK068479	NP_001148819.1 (RefS	-
Os03g0127800	Non-protein coding transcript.	EU947022	NONE	-
Os03g0127950	Similar to inner membrane protein ybaL.	CT835134	NP_001147290.1 (RefS	-
Os03g0130800	Non-protein coding transcript.	AK058647	NONE	-
Os03g0137050	Non-protein coding transcript.	BT086855	NONE	-
Os03g0137600	Conserved hypothetical protein.	AK061189	B9FAZ2	-
Os03g0137700	Mg2+ transporter protein, CorA-like domain containing protein.	AK069498	Q10S25	-
Os03g0137750	Hypothetical protein.	tplb0043b08 (Wheat FLcDNA)	longestORF	-
Os03g0172000	Peptidase S59, nucleoporin family protein.	AK122024	NP_178183.2 (RefSeq)	Peptidase S59, nucleoporin
Os03g0174200	Conserved hypothetical protein.	AK103762 ,AK060616	A2XD32	-
Os03g0174400	Hypothetical conserved gene.	EF028768	Q10R18	Pathogenesis-related transcriptional factor/ERF, DNA-binding
Os03g0174800	Conserved hypothetical protein.	AK069179	B9FBL5	-
Os03g0174900	Similar to RAPB protein.	AK069348	O49915	CCAAT-binding transcription factor, subunit B
Os03g0175200	Similar to predicted protein.	AK242803	NP_001147647.1 (RefS	Mitochondrial substrate/solute carrier
Os03g0176200	Hypothetical protein.	EU949353	longestORF	-
Os03g0177000	Acyl-CoA N-acyltransferase domain containing protein.	AK071368	NP_671784.2 (RefSeq)	GCN5-related N-acetyltransferase
Os03g0177900	EF-1 alpha.	AK119647 ,AK119528 ,AK07134	B8APM5	Protein synthesis factor, GTP-binding
Os03g0178000	EF-1 alpha.	AK072285	B8APM5	Protein synthesis factor, GTP-binding
Os03g0178150	Hypothetical gene.	BT086892	longestORF	-
Os03g0178200	Protein of unknown function YGGT family protein.	AK106024	A6MZH3	Uncharacterised protein family Ycf19
Os03g0178300	Hypothetical conserved gene.	AK121217	A2XD59	-
Os03g0178550	Hypothetical gene.	AK289159	longestORF	-
Os03g0179000	Similar to Tubulin-tyrosine ligase family protein, expressed.	AK069518	Q10QY3	Tubulin-tyrosine ligase
Os03g0179100	Similar to predicted protein.	CT835279	Q8H021	Germin
Os03g0179700	Similar to OSIGBa0140O07.11 protein.	CT835157	Q01J32	Tubby, C-terminal
Os03g0179950	Non-protein coding transcript.	CT835230	NONE	-
Os03g0180000	Sodium/calcium exchanger membrane region domain containing protein.	AK360521	NP_564650.1 (RefSeq)	Sodium/calcium exchanger membrane region
Os03g0180100	Protein of unknown function DUF1677, plant domain containing protein.	AK108326	B8APN4	Protein of unknown function DUF1677, plant
Os03g0180300	Hypothetical conserved gene.	AK061621	Q10QX0	Smr protein/MutS2 C-terminal
Os03g0180400	Proteasome subunit alpha type 6 (EC 3.4.25.1) (20S proteasome alpha subunit A) (20S proteasome s	AK101031 ,AK059929 ,AK06002	A2XD75	Proteasome, alpha-subunit, conserved site
Os03g0180700	Glycosyl transferase, group 1 domain containing protein.	AK069776	NP_001149006.1 (RefS	Glycosyl transferase, family 1
Os03g0180800	Similar to ZIM motif family protein.	AK070649	NP_001151145.1 (RefS	Tify
Os03g0181500	Similar to (Rice Genome Annotation Project) fiddlehead-like protein.	AK100798	Q10QW0	3-Oxoacyl-[acyl-carrier-protein
Os03g0182700	Eukaryotic translation initiation factor 3 subunit 12 (eIF-3 p25) (eIF3k).	AK073293 ,AK120235	Q94HF1	-
Os03g0183000	Similar to AP2 domain containing protein RAP2.6 (Fragment).	AK060527	Q94HF3	Pathogenesis-related transcriptional factor/ERF, DNA-binding
Os03g0183050	Hypothetical gene.	EU941438	longestORF	-
Os03g0183300	Pathogenesis-related transcriptional factor and ERF domain containing protein.	AK106163 ,AK105922 ,AK11955	Q5MFV0	Pathogenesis-related transcriptional factor/ERF, DNA-binding
Os03g0183800	Similar to Leucine-rich repeat transmembrane protein kinase 1 (Fragment).	AK112046	O81105	Protein kinase, catalytic domain
Os03g0184050	Non-protein coding transcript.	AK058668	NONE	-
Os03g0187200	Non-protein coding transcript.	AK060541	NONE	-
Os03g0188200	Zinc finger, RING/FYVE/PHD-type domain containing protein.	AK058578	NP_001149891.1 (RefS	Zinc finger, RING-type
Os03g0190100	UbiA prenyltransferase family protein.	AK061907 ,AK066332	B6UA12	UbiA prenyltransferase family
Os03g0190500	Hypothetical gene.	AK110854	longestORF	-
Os03g0191200	WW/Rsp5/WWP domain containing protein.	AK070228	A2XDF1	WW/Rsp5/WWP
Os03g0191550	Non-protein coding transcript.	X06284	NONE	-
Os03g0192550	Hypothetical gene.	AK111026	longestORF	-
Os03g0192900	Similar to Ubiquitin-specific protease 26.	AK105104	B9F5M9	-
Os03g0195400	Hypothetical gene.	AK064567	longestORF	-
Os03g0195450	Similar to sulfate/bicarbonate/oxalate exchanger and transporter sat-1.	CT835280	D4IIA9	Sulphate transporter/antisigma-factor antagonist STAS
Os03g0197050	Non-protein coding transcript.	CT835219	NONE	-
Os03g0197100	Similar to Sugar transporter protein.	AK099273 ,AK070417 ,AK10391	B6TSL9	Sugar/inositol transporter
Os03g0197125	Hypothetical gene.	tplb0043f10 (Wheat FLcDNA)	longestORF	-
Os03g0197825	Hypothetical protein.	BT086922	longestORF	-
Os03g0217900	Hypothetical protein.	AK119980	longestORF	-
Os03g0243700	Similar to Glycosyl hydrolase family 10 protein, expressed.	AK059149	Q10P81	Glycoside hydrolase, family 10
Os03g0243750	Hypothetical protein.	tplb0048l17 (Wheat FLcDNA)	longestORF	-
Os03g0244750	Conserved hypothetical protein.	ab initio prediction	NP_001183257.1 (RefS	-
Os03g0246500	Non-protein coding transcript.	AK105050	NONE	-
Os03g0275350	Non-protein coding transcript.	EU948346	NONE	-
Os03g0280800	Similar to UDP-D-glucuronate decarboxylase.	AK071034	NP_001151221.1 (RefS	NAD-dependent epimerase/dehydratase
Os03g0280900	Hypothetical protein.	tplb0036f05 (Wheat FLcDNA)	longestORF	-
Os03g0281100	Ankyrin repeat containing protein.	BT086250	NP_172250.1 (RefSeq)	Ankyrin repeat
Os03g0281466	Similar to Protein kinase (Pto kinase) (Serine/threonine protein kinase Pto).	AK241347	longestORF	-
Os03g0281500	Similar to Resistance protein candidate (Fragment).	AK100137	C6ZRT9	Protein kinase, catalytic domain
Os03g0281600	Similar to Ca2+-ATPase.	AK070260	Q4PKC7	ATPase, P-type, K/Mg/Cd/Cu/Zn/Na/Ca/Na/H-transporter
Os03g0281900	ABC transporter-like domain containing protein.	AK072135	NP_001151504.1 (RefS	ABC transporter-like
Os03g0282300	Hypothetical conserved gene.	BT038365	B9F7D7	-
Os03g0282700	DEAD-like helicase, N-terminal domain containing protein.	ab initio prediction	Q8H8U9	Helicase, C-terminal
Os03g0282800	Conserved hypothetical protein.	AK101658	B8ALI3	-
Os03g0282900	Conserved hypothetical protein.	AK102161 ,AK061166	B8ALI4	-
Os03g0283000	Similar to In2-1 protein.	AY332468	Q8H8U5	Glutathione S-transferase, N-terminal
Os03g0283100	Similar to In2-1 protein.	AK061849	Q8H8U5	Glutathione S-transferase, N-terminal
Os03g0283500	Pentatricopeptide repeat domain containing protein.	AK103244	B8ALI6	Pentatricopeptide repeat
Os03g0283650	Hypothetical protein.	ab initio prediction	NONE	-
Os03g0286200	Similar to P-protein.	AK105065	B6SYB7	Prephenate dehydratase
Os03g0295800	Similar to gamma-interferon-inducible lysosomal thiol reductase.	AK106050	B6TUH5	Gamma interferon inducible lysosomal thiol reductase GILT
Os03g0295866	Transcription factor IIS, N-terminal domain containing protein.	BT040964	Q10MT6	Transcription factor IIS, N-terminal
Os03g0295932	Hypothetical protein.	EU965145	longestORF	-
Os03g0296600	Similar to ECA1 protein.	AK109176	Q9SES8	Protein of unknown function DUF784
Os03g0296700	Non-protein coding transcript.	AK121470	NONE	-
Os03g0296800	Mitochondrial carrier protein domain containing protein.	AK058471	NP_001151324.1 (RefS	Mitochondrial carrier protein
Os03g0298100	Conserved hypothetical protein.	AK369609	NP_001173382.1 (RefS	-
Os03g0298400	Similar to 26S protease regulatory subunit 4 homolog.	AK058779	P46466	ATPase, AAA-type, core
Os03g0299200	Similar to Alcohol dehydrogenase (Fragment).	AK100767	P93795	Short-chain dehydrogenase/reductase SDR
Os03g0299400	Hypothetical protein.	EU954997	longestORF	-
Os03g0299600	Wound-induced protein, Wun1 domain containing protein.	AK068151	XP_002468012.1 (RefS	Wound-induced protein, Wun1
Os03g0299800	Hypothetical conserved gene.	AK100929	B8AM23	Uncharacterised protein family Cys-rich
Os03g0300000	Similar to Xyloglucan 6-xylosyltransferase (EC 2.4.2.39) (AtXT1).	AK060863	NP_001150077.1 (RefS	Galactosyl transferase
Os03g0300300	WD40 repeat-like domain containing protein.	AK099693	B8AM26	WD40 repeat
Os03g0300400	Pathogen-related protein (JIOsPR10).	AK121376	Q945E9	Bet v I allergen
Os03g0300500	Similar to Pectin methylesterase 6 (Fragment).	AK070513	A2XFP7	Pectinesterase, catalytic
Os03g0300600	Similar to Chaperone protein dnaJ.	AK066250	B6U761	Heat shock protein DnaJ, N-terminal
Os03g0300700	Retrotransposon gag protein family protein.	AK071770	A2XFP9	Retrotransposon gag protein
Os03g0301600	Conserved hypothetical protein.	AK062764	B9F7W2	-

Os03g0301700	Similar to Calmodulin-binding protein phosphatase.	AK101878	B6TFE7	Protein phosphatase 2C-like
Os03g0301950	Peroxisomal biogenesis factor 11 family protein.	Q10MN3	Q10MN3	Peroxisomal biogenesis factor 11
Os03g0302000	Peroxisomal biogenesis factor 11 family protein.	AK287976	Q10MN2	Peroxisomal biogenesis factor 11
Os03g0302100	Non-protein coding transcript.	AK063197	NONE	-
Os03g0302200	Zinc finger, RING/FYVE/PHD-type domain containing protein.	AK120853	A2XFQ9	Zinc finger, RING-type
Os03g0302700	Zinc finger, RING/FYVE/PHD-type domain containing protein.	AK112010	NP_001152943.1 (RefS	Zinc finger, RING-type
Os03g0304100	Conserved hypothetical protein.	AK111121	B8AMI6	-
Os03g0304500	Tetratricopeptide-like helical domain containing protein.	AK101290	XP_002874939.1 (RefS	Tetratricopeptide TPR-1
Os03g0304550	Hypothetical conserved gene.	BT085356	longestORF	-
Os03g0304800	Lg106-like family protein.	AK062656	NP_001148326.1 (RefS	Endosulphine
Os03g0305500	Similar to Argininosuccinate lyase.	AK070233	B4FAV4	Fumarate lyase
Os03g0305550	Hypothetical gene.	EU949424	longestORF	-
Os03g0305600	Mitochondrial import inner membrane translocase, subunit Tim17/22 family protein.	AK063714	Q2HU23	Mitochondrial inner membrane translocase subunit Tim17/Tim22/Tim23/peroxisomal p
Os03g0305800	Galactosyl transferase family protein.	AK105101 ,AK105005	A2XFT5	-
Os03g0306200	Similar to transducin family protein / WD-40 repeat family protein.	AK241828	XP_002865733.1 (RefS	Bromodomain
Os03g0306302	Hypothetical conserved gene.	DQ245981	Q10MJ9	-
Os03g0306800	Similar to CP12 (Fragment).	AK103722	B7XB92	Domain of unknown function CP12
Os03g0306900	Haem oxygenase-like, multi-helical domain containing protein.	AK073626	B6TPF2	TENA/THI-4 protein/Coenzyme PQQ biosynthesis protein C domain
Os03g0307000	Conserved hypothetical protein.	AK243201	NP_001049892.2 (RefS	-
Os03g0307300	Nicotianamine synthase 1 (EC 2.5.1.43) (S-adenosyl-L-methionine:S- adenosyl-L-methionine:S-aden	AK112069	Q0DSH9	Nicotianamine synthase
Os03g0320800	Similar to Leucine Rich Repeat family protein, expressed.	AK103265	Q10M75	Leucine-rich repeat
Os03g0320850	Hypothetical gene.	BT061105	longestORF	-
Os03g0320900	Guanylate kinase family protein.	AK106255	NP_001149581.1 (RefS	Guanylate kinase
Os03g0321700	Similar to WRKY transcription factor 55.	AK101653	A6N066	DNA-binding WRKY
Os03g0321800	Similar to WRKY transcription factor 55.	AK111447	NP_001030961.1 (RefS	Octanoyltransferase
Os03g0322000	Similar to Centromere protein-like protein (Fragment).	AY224484	Q10M62	-
Os03g0324650	Non-protein coding transcript.	BT085189	NONE	-
Os03g0325500	Similar to Phosphate starvation regulator protein (Regulatory protein of P- starvation acclimation res	AK100282	A2XG76	Myb-like DNA-binding domain, SHAQKYF class
Os03g0325600	Similar to WUSCHEL-related homeobox 6.	AK111000	Q10M29	Homeobox
Os03g0327100	Similar to CUC1.	AK065989	NP_001147731.1 (RefS	No apical meristem
Os03g0328000	Dedicator of cytokinesis family protein.	AK059469	NP_193367.7 (RefSeq)	Dedicator of cytokinesis
Os03g0328400	Similar to Apyrase GS50 (Fragment).	AF358764	D3Y1I0	Nucleoside phosphatase GDA1/CD39
Os03g0328450	Non-protein coding transcript.	BT017220	NONE	-
Os03g0328600	Hypothetical protein.	EU949280	longestORF	-
Os03g0329200	Similar to cDNA clone:J013002B05, full insert sequence.	AK064318	B7F3U2	-
Os03g0329700	Similar to predicted protein.	AK242883	Q10LZ2	Protein kinase, catalytic domain
Os03g0333100	Hypothetical conserved gene.	AK060242	B9F8A6	AARP2CN
Os03g0336400	Similar to Alpha-expansin OsEXPA4.	AK107698	Q9M4X7	Expansin
Os03g0336700	PAP/25A core domain containing protein.	AK120611	Q10LS4	PAP/25A-associated
Os03g0336900	Similar to Uncharacterized plant-specific domain TIGR01568 family protein, expressed.	EU972618	NP_001050045.2 (RefS	Domain of unknown function DUF623
Os03g0338400	Basic helix-loop-helix dimerisation region bHLH domain containing protein.	AK073378	B4FLH1	Helix-loop-helix DNA-binding
Os03g0338800	Hypothetical conserved gene.	AK064660	B8B3T4	-
Os03g0339150	Hypothetical gene.	AK241704	longestORF	-
Os03g0342900	Dormancyauxin associated family protein.	AK104391 ,AK060136 ,AK06098	B4FA62	-
Os03g0343225	Hypothetical gene.	EU951910	longestORF	-
Os03g0343300	Small-subunit processome, Utp14 domain containing protein.	AK243183	Q10LM2	Small-subunit processome, Utp14
Os03g0343400	Similar to Photolyase/blue-light receptor PHR2.	AK105611	B6U742	DNA photolyase, FAD-binding/Cryptochrome, C-terminal
Os03g0343500	Similar to 60S ribosomal protein L22-2.	AK059599	B6U998	Ribosomal protein L22e
Os03g0343700	Brix domain containing protein.	AK060603	XP_002864754.1 (RefS	Brix domain
Os03g0343900	Photosystem II PsbX domain containing protein.	AK105813	GeneMark	Photosystem II PsbX
Os03g0344232	Conserved hypothetical protein.	CT835238	Q10RS8	-
Os03g0344300	Similar to Ulp1 protease family, C-terminal catalytic domain containing protein, expressed.	AK073314	Q10LL1	Peptidase C48, SUMO/Sentrin/Ubl1
Os03g0344650	Hypothetical protein.	EU943048	longestORF	-
Os03g0345100	Similar to auxin response factor 75.	AK065579	NP_001149782.1 (RefS	Rad9
Os03g0345200	40S ribosomal protein S21.	AK073724 ,AK104084 ,AK11926	P35687	Ribosomal protein S21e
Os03g0345300	Similar to Desiccation-related protein Lb_13-62 (Fragment).	AK108277	B1PMG8	-
Os03g0345500	Non-protein coding transcript.	EU975566	NONE	-
Os03g0345700	Similar to Heavy metal-associated domain containing protein, expressed.	AK066080	Q10LJ9	-
Os03g0345901	Hypothetical gene.	EU973702	longestORF	-
Os03g0346150	Non-protein coding transcript.	CU861814	NONE	-
Os03g0346200	Conserved hypothetical protein.	ab initio prediction	A2XGM1	-
Os03g0347700	NPH3 domain containing protein.	AK110492	E9NZU3	NPH3
Os03g0347750	Hypothetical gene.	BT083870	longestORF	-
Os03g0347800	Peptidyl-tRNA hydrolase family protein.	AK073756	XP_002871908.1 (RefS	Peptidyl-tRNA hydrolase
Os03g0348200	Terpene synthase-like domain containing protein.	EU954571	NP_001050103.1 (RefS	Terpene synthase-like
Os03g0348900	Similar to CHY zinc finger family protein, expressed.	AK073680	Q10LI0	Zinc finger, RING-type
Os03g0349200	Similar to transposon protein.	AK101089	NP_001148044.1 (RefS	-
Os03g0349400	Hypothetical protein.	tplb0040h06 (Wheat FLcDNA)	longestORF	-
Os03g0351100	Similar to CONSTANS-like protein CO9 (Fragment).	AK101822	C0PI33	Zinc finger, B-box
Os03g0351200	NAD(P)-binding domain containing protein.	AK353935	D5L1S4	NmrA-like
Os03g0351300	Similar to beta-amylase.	BT041352	NP_001151271.1 (RefS	Glycoside hydrolase, family 14
Os03g0351400	Similar to Tubby-like protein 3.	AK102370	Q10LG8	Tubby, C-terminal
Os03g0355400	WD40 repeat-like domain containing protein.	BT055024	NP_181681.1 (RefSeq)	WD40 repeat
Os03g0355800	Similar to Diphthine synthase (EC 2.1.1.98) (Diphthamide biosynthesis methyltransferase).	AK103902	B8APT0	Tetrapyrrole methylase
Os03g0356414	Similar to Ubiquitin ligase SINAT5 (EC 6.3.2.-) (Seven in absentia homolog 5). Splice isoform 2.	AK058336	B6SRI2	Zinc finger, RING-type
Os03g0363700	Pentatricopeptide repeat domain containing protein.	EU954511	NP_001050167.1 (RefS	Pentatricopeptide repeat
Os03g0368150	Hypothetical protein.	BT085962	longestORF	-
Os03g0387900	Hypothetical conserved gene.	AK063085	Q10KE2	-
Os03g0388000	Similar to Splicing factor SC35.	AK073057	B6T6J9	RNA recognition motif domain
Os03g0388500	Similar to Anther ethylene-upregulated protein ER1 (Fragment).	AK070350	Q10KD1	IQ motif, EF-hand binding site
Os03g0388650	Hypothetical protein.	EU971393	longestORF	-
Os03g0392250	Hypothetical gene.	AK288273	longestORF	-
Os03g0392300	Similar to GTP-binding protein-like.	AK071779	B4FF78	Small GTP-binding protein domain
Os03g0392400	Heat shock protein DnaJ, N-terminal domain containing protein.	AK106027 ,AK073726	B6TYB9	Heat shock protein DnaJ, N-terminal
Os03g0392833	Hypothetical protein.	EU949881	longestORF	-
Os03g0393350	Hypothetical genes.	ab initio prediction	A3AIQ6	-
Os03g0419200	Hypothetical gene.	AK106284	GeneMark	-
Os03g0419700	Hypothetical conserved gene.	ab initio prediction	Q75GC7	-
Os03g0437100	Zinc finger, C2H2-type domain containing protein.	AK108997	A2XIC5	Zinc finger, C2H2
Os03g0589500	Conserved hypothetical protein.	AK105481	Q5W6J4	-
Os03g0593100	Conserved hypothetical protein.	AK287562	NP_001050587.1 (RefS	-
Os03g0605400	Non-protein coding transcript.	AK063612	NONE	-
Os03g0609400	Non-protein coding transcript.	AK241806	NONE	-
Os03g0609500	Similar to LOB domain protein 39.	AK288002	NP_201543.1 (RefSeq)	Lateral organ boundaries, LOB
Os03g0617101	Non-protein coding transcript.	CT835260	NONE	-
Os03g0627300	Similar to nucleotide-binding protein-like.	AK106686	NP_001150831.1 (RefS	Cobyrinic acid a,c-diamide synthase
Os03g0633900	Nucleic acid-binding, OB-fold-like domain containing protein.	AK068169 ,AK059181	NP_001148836.1 (RefS	Primosome PriB/single-strand DNA-binding
Os03g0637800	Regulator of chromosome condensation/beta-lactamase-inhibitor protein II domain containing protei	AK101339	Q948R7	Regulator of chromosome condensation, RCC1
Os03g0637900	Similar to Centromere protein-like protein (Fragment).	AK060224	Q84VB6	-
Os03g0638000	Similar to Centromere protein-like protein (Fragment).	AY224555	Q84VD9	-
Os03g0643100	Homeodomain-like containing protein.	AK106587	Q60DP5	Myb/SANT-like domain
Os03g0646850	Non-protein coding transcript.	BT017109	NONE	-
Os03g0647400	GCK domain containing protein.	AK073665	Q9LMF3	GCK
Os03g0647500	Similar to GTP binding protein.	AK064308	B6U832	-
Os03g0647550	Non-protein coding transcript.	AF124739	NONE	-
Os03g0647575	Hypothetical protein.	BT086909	longestORF	-
Os03g0648100	Protein of unknown function DUF266, plant family protein.	AK109329	A3AKV3	Glycosyl transferase, family 14
Os03g0648250	Zinc finger, GRF-type domain containing protein.	AK241508	Q259A1	Zinc finger, GRF-type
Os03g0648400	Non-protein coding transcript.	AK058555	NONE	-
Os03g0648500	Cupredoxin domain containing protein.	AK107766	A2XV11	Plastocyanin-like
Os03g0650300	Hypothetical conserved gene.	tplb0014g09 (Wheat FLcDNA)	Q43713	-
Os03g0650350	Hypothetical gene.	EU947173	longestORF	-
Os03g0650400	Similar to ameiotic 1.	AK064367	NP_001139538.1 (RefS	-
Os03g0650700	Translation elongation factor EFG/EF2, C-terminal domain containing protein.	AK102195	XP_002885535.1 (RefS	Translation elongation factor EFG/EF2, C-terminal
Os03g0650800	Protein of unknown function DUF593 family protein.	ab initio prediction	NP_001050787.1 (RefS	Protein of unknown function DUF593

Os03g0650850	Non-protein coding transcript.	AK242527	NONE	-
Os03g0651950	Hypothetical protein.	tplb0048i05 (Wheat FLcDNA)	longestORF	-
Os03g0652000	C2 domain containing protein.	AK110820 ,AK107712	Q7XZZ4	-
Os03g0655300	CS domain domain containing protein.	BT061748	B6T9V0	CS-like domain
Os03g0655400	Similar to LIP5.	AK059150	GeneMark	-
Os03g0656100	Similar to Hsp20/alpha crystallin family protein, expressed.	AK062338	Q10FT7	-
Os03g0656800	Similar to 3-glucanase.	AK069506	NP_001147672.1 (RefS	Glycoside hydrolase, family 17
Os03g0656850	Conserved hypothetical protein.	BT019195	C4J0K6	-
Os03g0657100	Similar to predicted protein.	AK066133	NP_564125.2 (RefSeq)	Armadillo-like helical
Os03g0660500	Auxin responsive SAUR protein domain containing protein.	AK361840	Q75GQ1	Auxin responsive SAUR protein
Os03g0660700	Conserved hypothetical protein.	AK109543	B9FA57	-
Os03g0661250	Hypothetical protein.	EU941142	longestORF	-
Os03g0661300	Beta-tubulin (Beta-3 tubulin) (Tubulin beta subunit).	AK121321	Q76FS2	Tubulin
Os03g0661600	Similar to Alpha-amylase/trypsin inhibitor (Antifungal protein).	AK058637	O04364	Thaumatin, pathogenesis-related
Os03g0661700	Non-protein coding transcript.	AK073701	NONE	-
Os03g0663400	Similar to Thaumatin-like protein.	AK102970 ,AK102798 ,AK11916	Q40630	-
Os03g0663500	Similar to Osmotin precursor.	U77656	NP_001147526.1 (RefS	Thaumatin, pathogenesis-related
Os03g0663600	Similar to Pathogenesis-related thaumatin-like protein.	EU967040	O04363	-
Os03g0663750	Hypothetical protein.	tplb0024b15 (Wheat FLcDNA)	longestORF	-
Os03g0663800	Globulin 1 (Fragment).	AK105347	Q10FL8	Cupin 1
Os03g0664300	F-box domain, cyclin-like domain containing protein.	tplb0015o19 (Wheat FLcDNA)	Q75GY3	F-box domain, cyclin-like
Os03g0664400	Similar to Protease inhibitor/seed storage/LTP family protein.	AK058628	B6U491	Plant lipid transfer protein/seed storage/trypsin-alpha amylase inhibitor
Os03g0664700	RNA polymerase II accessory factor, Cdc73 domain containing protein.	AK100794	B8ANM7	RNA polymerase II accessory factor, Cdc73
Os03g0664800	Acyl-CoA N-acyltransferase domain containing protein.	AK243094	NP_001150788.1 (RefS	GCN5-related N-acetyltransferase
Os03g0664901	Hypothetical protein.	EU957022	longestORF	-
Os03g0665000	GCN5-related N-acetyltransferase (GNAT) domain domain containing protein.	EU969219	NP_001050840.2 (RefS	GCN5-related N-acetyltransferase
Os03g0665200	Protein of unknown function DUF581 family protein.	AK066202	B8ANM9	Protein of unknown function DUF581
Os03g0665700	Similar to predicted protein.	DQ245183	NP_001050842.2 (RefS	Protein of unknown function DUF1677, plant
Os03g0665751	Hypothetical gene.	BT086957	longestORF	-
Os03g0665800	Armadillo-type fold domain containing protein.	AK107693	NP_191180.2 (RefSeq)	Armadillo-like helical
Os03g0666300	Hypothetical conserved gene.	BT083602	Q75H97	MYB-like
Os03g0666500	Similar to Ras-related protein RHA1.	AK061203	B4F7V6	Small GTPase superfamily
Os03g0666550	Hypothetical protein.	tplb0055b17 (Wheat FLcDNA)	longestORF	-
Os03g0666600	UDP-glucuronosyl/UDP-glucosyltransferase family protein.	AK064709	NP_001151546.1 (RefS	UDP-glucuronosyl/UDP-glucosyltransferase
Os03g0666700	TRAM, LAG1 and CLN8 homology domain containing protein.	AK061721 ,AK061309 ,AK10201	NP_001149791.1 (RefS	TRAM/LAG1/CLN8 homology domain
Os03g0666850	Hypothetical conserved gene.	AK240914	B9ETH0	-
Os03g0685750	Hypothetical conserved gene.	CT833489	NP_195008.5 (RefSeq)	-
Os03g0685800	Hypothetical protein.	AK058278	longestORF	-
Os03g0698400	Non-protein coding transcript.	AK058933	NONE	-
Os03g0699100	WD40 repeat-like domain containing protein.	AK110715	B6TYC2	WD40 repeat
Os03g0701100	Similar to Prp18 domain containing protein, expressed.	AK109455 ,AK110740	Q53RB6	Splicing factor motif
Os03g0702600	Hypothetical conserved gene.	CT828111	Q75I87	-
Os03g0705433	Conserved hypothetical protein.	ab initio prediction	NP_001173605.1 (RefS	-
Os03g0707250	Non-protein coding transcript.	AK242476	NONE	-
Os03g0718100	Actin 1.	AK100267 ,AK058421	Q10DV7	Actin-like
Os03g0718150	Hypothetical protein.	BT018641	longestORF	-
Os03g0718200	Zinc finger, SWIM-type domain containing protein.	AK063808	Q10DV6	Zinc finger, PMZ-type
Os03g0718300	Hypothetical protein.	AK108509	longestORF	-
Os03g0718650	Hypothetical protein.	EU955192	longestORF	-
Os03g0718800	Similar to Physical impedance induced protein.	AK288124	NP_001149478.1 (RefS	Plant lipid transfer protein/seed storage/trypsin-alpha amylase inhibitor
Os03g0739500	Conserved hypothetical protein.	AK241255	Q5ZAF7	-
Os03g0746500	Similar to predicted protein.	AK058367	NP_193215.2 (RefSeq)	Helicase, C-terminal
Os03g0773150	40S ribosomal protein S29.	CU406248	A2ZG71	Ribosomal protein S14
Os03g0773300	Protein kinase, core domain containing protein.	AK100394	NP_001105795.1 (RefS	Protein kinase, catalytic domain
Os03g0773450	Hypothetical protein.	tplb0046o04 (Wheat FLcDNA)	longestORF	-
Os03g0774400	Similar to predicted protein.	AK109683	Q10E11	-
Os03g0775200	Protein of unknown function DUF248, methyltransferase putative family protein.	AK120397	NP_564084.1 (RefSeq)	Protein of unknown function DUF248, methyltransferase putative
Os03g0775250	Hypothetical protein.	AK358342	longestORF	-
Os03g0775300	Flavodoxin/nitric oxide synthase domain containing protein.	AK100507	Q8H8N3-2	Radical SAM
Os03g0775400	Tetratricopeptide-like helical domain containing protein.	AK107354	A2XMF9	Pentatricopeptide repeat
Os03g0775700	Conserved hypothetical protein.	AK067093	A2XMG2	-
Os03g0775900	Conserved hypothetical protein.	AK374530	Q8H8M7	-
Os03g0777000	Similar to NAC-domain containing protein 19 (ANAC019) (ANAC) (Abscicic-acid- responsive NAC	AK073539	F1DJT1	No apical meristem
Os03g0778400	Similar to predicted protein.	CT835114	NP_001051445.1 (RefS	-
Os03g0779800	Protein of unknown function DUF295 domain containing protein.	tplb0004o19 (Wheat FLcDNA)	Q9AY78	Protein of unknown function DUF295
Os03g0780200	Conserved hypothetical protein.	AK107920	A2XMI6	-
Os03g0780350	Similar to SAUR1 - auxin-responsive SAUR family member.	EU974244	NP_001152203.1 (RefS	Auxin responsive SAUR protein
Os03g0780400	Similar to Actin-depolymerizing factor 6 (ADF-6) (AtADF6).	AK073162 ,AK061347	Q9AY76	Actin-binding, cofilin/tropomyosin type
Os03g0780550	Hypothetical protein.	tplb0045b10 (Wheat FLcDNA)	longestORF	-
Os03g0780600	Tubulin beta-1 chain (Beta-1 tubulin).	AK120180	P37832	Tubulin
Os03g0780800	Fatty acid hydroxylase domain containing protein.	AK103085	NP_001149139.1 (RefS	Fatty acid hydroxylase
Os03g0780900	Protein of unknown function DUF858, methyltransferase-like family protein.	AK100619	A2XMJ1	Protein of unknown function DUF858, methyltransferase-like
Os03g0781000	Conserved hypothetical protein.	AK065910	Q9AY71	-
Os03g0781300	Conserved hypothetical protein.	AK120661	F2EFE7	-
Os03g0781400	Ribose 5-phosphate isomerase family protein.	AK071970	NP_001151663.1 (RefS	Ribose 5-phosphate isomerase, type A
Os03g0781501	Non-protein coding transcript.	AK243070	NONE	-
Os03g0782200	Hypothetical protein.	AK069119	GeneMark	-
Os03g0782300	Conserved hypothetical protein.	AK070713	B9F3B9	-
Os03g0782350	Hypothetical gene.	CT832459	longestORF	-
Os03g0782400	Conserved hypothetical protein.	ab initio prediction	Q10CH8	-
Os03g0782900	Pentatricopeptide repeat domain containing protein.	AK108367	Q9AY61	Pentatricopeptide repeat
Os03g0782950	Hypothetical protein.	tplb0031e02 (Wheat FLcDNA)	longestORF	-
Os03g0783000	Similar to Actin-related protein 7.	AK069426	A3ANB5	Actin-like
Os03g0783051	Conserved hypothetical protein.	FP098389	A3ANB6	-
Os03g0799900	Similar to sec12-like protein 1.	AK111711	NP_001148204.1 (RefS	WD40 repeat
Os03g0800400	Protein of unknown function DUF1618 domain containing protein.	AK071430	Q851R3	Domain of unknown function DUF1618
Os03g0800900	Hypothetical conserved gene.	AK241533	Q851R7	-
Os03g0802650	Hypothetical gene.	CT837941	longestORF	-
Os03g0803200	Similar to CLE family OsCLE306 protein.	AK111283	A8R3N5	-
Os03g0803250	Non-protein coding transcript.	EU958975	NONE	-
Os03g0803500	Similar to Prolyl 4-hydroxylase alpha-1 subunit-like protein.	AK059759	B6TAV1	Metridin-like ShK toxin
Os03g0803600	Glycosyl transferase, family 31 protein.	AK241975	B6SJ23	Glycosyl transferase, family 31
Os03g0804000	Hypothetical protein.	AK059908	longestORF	-
Os03g0804100	Conserved hypothetical protein.	FP100876	NP_001051616.1 (RefS	-
Os03g0804300	Zinc finger, DHHC-type domain containing protein.	AK067295	NP_001148846.1 (RefS	Zinc finger, DHHC-type, palmitoyltransferase
Os03g0804400	Conserved hypothetical protein.	AK073430	Q75HJ9	-
Os03g0804500	Similar to Germin-like protein subfamily T member 1 precursor.	AK062698	Q10BU2	Germin
Os03g0804600	Similar to Germin-like protein 3-7.	AK248846	NP_001051622.2 (RefS	-
Os03g0804700	Germin-like protein 3-8.	Q75HJ4	Q75HJ4	Germin
Os03g0805100	Similar to Squalene synthase (EC 2.5.1.21).	AK242592	NP_001104839.1 (RefS	Squalene/phytoene synthase
Os03g0805400	Phosphatidic acid phosphatase type 2/haloperoxidase domain containing protein.	AK071708	NP_001151171.1 (RefS	Phosphatidic acid phosphatase type 2/haloperoxidase
Os03g0805500	Similar to AAE18 (ACYL-ACTIVATING ENZYME 18)%3B catalytic/ ligase.	AK067857	NP_175929.3 (RefSeq)	-
Os03g0805600	Similar to pheophorbide a oxygenase.	CT835283	B6ST74	Rieske [2Fe-2S] iron-sulphur domain
Os03g0805700	Similar to Pheophorbide a oxygenase.	EU955939	B6ST74	Pheophorbide a oxygenase
Os03g0805733	Hypothetical protein.	tplb0031c13 (Wheat FLcDNA)	longestORF	-
Os03g0806400	Similar to Elongation factor P family protein, expressed.	AK073074	Q10BS5	-
Os03g0806600	Conserved hypothetical protein.	AK070959	B9F6V2	-
Os03g0806700	Protein of unknown function DUF868, plant family protein.	AK062034	XP_002466252.1 (RefS	Protein of unknown function DUF868, plant
Os03g0806800	Conserved hypothetical protein.	AK061623	A2XN54	-
Os03g0806900	Similar to Cytochrome-C reductase 14 kDa subunit (EC 1.10.2.2) (Fragment).	AK121708	B6UBZ9	Cytochrome d ubiquinol oxidase, 14kDa subunit
Os03g0807200	Protein of unknown function DUF1639 family protein.	AK103496 ,AK063932	A2XN58	Protein of unknown function DUF1639
Os03g0807700	Protein of unknown function DUF642 domain containing protein.	AK059286	A2XN62	Protein of unknown function DUF642
Os03g0807900	Chaperonin-like RbcX family protein.	AK060962	A2XN64	Chaperonin-like RbcX
Os03g0808100	Similar to Cellulose synthase BoCesA5.	AK058615	Q4U0Z5	Cellulose synthase
Os03g0808150	Hypothetical gene.	BT019277	longestORF	-

Os03g0808175	Non-protein coding transcript.	U48693	NONE	-
Os03g0808200	UDP-glucuronosyl/UDP-glucosyltransferase family protein.	BT037819	NP_001149762.1 (RefS	UDP-glucuronosyl/UDP-glucosyltransferase
Os03g0808350	Hypothetical protein.	AK361927	longestORF	-
Os03g0809800	Conserved hypothetical protein.	AK241877	Q10BN7	-
Os03g0810900	ARF/SAR superfamily domain containing protein.	AK121284	XP_002871068.1 (RefS	Patatin/Phospholipase A2-related
Os03g0820600	Similar to cDNA clone:001-009-H02, full insert sequence.	AK105336	B7E3A0	Actin-binding, cofilin/tropomyosin type
Os03g0824000	Peptidase S8 and S53, subtilisin, kexin, sedolisin domain containing protein.	AK111129	A9NKD9	Protein of unknown function DUF3743
Os03g0832900	Similar to DNAJ-like protein (Fragment).	AK060945	Q84VA4	Heat shock protein DnaJ, N-terminal
Os03g0833100	Similar to Tasselseed2 protein (Fragment).	AK068662	Q2EFH2	Short-chain dehydrogenase/reductase SDR
Os03g0833200	Methyltransferase TRM13 domain containing protein.	AK102870	B8AMR9	Methyltransferase TRM13
Os03g0833600	X8 domain containing protein.	AK109880	Q10B16	X8
Os03g0833700	RNA 3'-terminal phosphate cyclase family protein.	AK121918	NP_680196.1 (RefSeq)	RNA 3&apos%3B-terminal phosphate cyclase
Os03g0833900	Similar to Cytosine deaminase (EC 3.5.4.1).	AK073655 ,AK104068	NP_001105963.1 (RefS	-
Os03g0834050	Similar to predicted protein.	AK240756	B6TQ05	HAD-superfamily hydrolase, subfamily IA, variant 3
Os03g0835100	Similar to Chloroplast protein import component Toc159.	AK373768	Q75LI9	Chloroplast protein import component Toc86/159
Os03g0835600	Galactose oxidase/kelch, beta-propeller domain containing protein.	AK101677	NP_001152073.1 (RefS	Acyl-CoA-binding protein, ACBP
Os03g0843100	Ras GTPase family protein.	AK111499	B4FEN1	Small GTPase superfamily
Os03g0843150	Non-protein coding transcript.	BT084300	NONE	-
Os03g0843200	Protein of unknown function DUF901 family protein.	AK063613	B6TD02	Protein of unknown function DUF901
Os03g0843300	Late embryogenesis abundant protein 2 family protein.	AK099274	B6T887	Late embryogenesis abundant protein, LEA-14
Os03g0844000	Hypothetical conserved gene.	AK059033	B9F7J4	Pentatricopeptide repeat
Os03g0844301	Hypothetical gene.	CT835264	longestORF	-
Os03g0847851	Non-protein coding transcript.	CT835272	NONE	-
Os03g0856200	Similar to Longin-like.	AK058786	Q2HUA7	Longin-like
Os04g0102600	Similar to Splicing factor 3B subunit 2 (Spliceosome associated protein 145) (SAP 145) (SF3b150) (	AK104417 ,AK070814	Q01N45	Peptidase S24/S26A/S26B/S26C, beta-ribbon domain
Os04g0120000	Hypothetical protein.	AK372466	longestORF	-
Os04g0125800	Similar to Growth-regulating factor 1.	EF515848	Q9SDZ5	-
Os04g0171232	Conserved hypothetical protein.	BT039178	A2WJQ3	-
Os04g0213800	Similar to Bacterial blight resistance protein XA26.	DQ119307	Q2EZ14	Protein kinase, catalytic domain
Os04g0247875	Non-protein coding transcript.	EU941912	NONE	-
Os04g0249550	Hypothetical protein.	tplb0054p19 (Wheat FLcDNA)	longestORF	-
Os04g0250200	Conserved hypothetical protein.	AK058584	B8ARM3	-
Os04g0255525	Hypothetical gene.	AK242256	longestORF	-
Os04g0255600	Cytochrome P450 domain containing protein.	BT064329	Q7XQ53	Cytochrome P450
Os04g0259200	Conserved hypothetical protein.	AK119366	B8B6C2	-
Os04g0286500	Homeodomain-like containing protein.	AK064584	B8BCY2	MYB-like
Os04g0293600	Hypothetical protein.	AK063003	longestORF	-
Os04g0294812	EF-hand-like domain domain containing protein.	FP097437	Q33AZ2	EF-hand-like domain
Os04g0309751	Hypothetical protein.	tplb0054p19 (Wheat FLcDNA)	longestORF	-
Os04g0313600	Zinc finger, CCHC-type domain containing protein.	AK288309	A2XRV4	Zinc finger, CCHC-type
Os04g0313900	Similar to cytokinin-O-glucosyltransferase 2.	EU976007	NP_001152029.1 (RefS	UDP-glucuronosyl/UDP-glucosyltransferase
Os04g0319600	tRNA methyltransferase complex GCD14 subunit domain containing protein.	AK243633	Q01M41	tRNA methyltransferase complex GCD14 subunit
Os04g0319800	Similar to Cytokinin-O-glucosyltransferase 2 (EC 2.4.1.-) (Zeatin O- glucosyltransferase 2).	AK241629	Q01M39	UDP-glucuronosyl/UDP-glucosyltransferase
Os04g0320000	Similar to H0107B07.3 protein.	ab initio prediction	Q01M51	-
Os04g0320200	Tetratricopeptide-like helical domain containing protein.	AK069513 ,AK104028 ,AK11925	Q01M49	Pentatricopeptide repeat
Os04g0320700	Similar to Glucosyltransferase (Fragment).	AK119530 ,AK105966	Q01M48	UDP-glucuronosyl/UDP-glucosyltransferase
Os04g0321700	Similar to Serine carboxypeptidase I precursor (EC 3.4.16.5) (Carboxypeptidase C).	AK068732	Q01LN2	Peptidase S10, serine carboxypeptidase
Os04g0321800	Similar to OSIGBa0097I24.2 protein.	AK069106	Q01LN1	Protein phosphatase 2C-like
Os04g0326000	Similar to H0107B07.3 protein.	ab initio prediction	Q01M51	-
Os04g0326300	Hypothetical conserved gene.	AK242372	NP_001052474.2 (RefS	-
Os04g0332700	Conserved hypothetical protein.	AK071842	Q6MWJ9	-
Os04g0333850	Non-protein coding transcript.	CT835070	NONE	-
Os04g0334700	Peptidase aspartic, catalytic domain containing protein.	AK120870	B7F2P1	Peptidase A1
Os04g0336001	Hypothetical protein.	EU945299	longestORF	-
Os04g0336600	Peptidase aspartic, catalytic domain containing protein.	AK120870	B7F2P1	Peptidase A1
Os04g0336801	Conserved hypothetical protein.	AK289258	Q2QWP9	-
Os04g0337300	Similar to OSIGBa0137O04.8 protein.	AK060427	Q01KY9	-
Os04g0340100	Hypothetical conserved gene.	ab initio prediction	B9FEH9	-
Os04g0340550	Hypothetical gene.	AK068264	longestORF	-
Os04g0341500	Similar to OSIGBa0159H11-OSIGBa0137A07.8 protein.	AK289110	Q01HV1	Terpene synthase, metal-binding domain
Os04g0345400	Similar to OSIGBa0106G08.7 protein.	AK062280	Q01KN7	Terpene synthase-like
Os04g0351800	Conserved hypothetical protein.	FP101309	B8ASJ0	-
Os04g0351933	Conserved hypothetical protein.	FP101309	A3ASQ2	-
Os04g0352066	Conserved hypothetical protein.	AK375252	B9FEL4	-
Os04g0352200	Conserved hypothetical protein.	AK103395	A2XS74	-
Os04g0354200	Similar to OSIGBa0092G14.5 protein.	ab initio prediction	Q01KX7	FAR1 DNA binding domain
Os04g0357500	Conserved hypothetical protein.	EU966738	B8ASQ8	-
Os04g0357700	Hypothetical conserved gene.	AK250527	NP_001052546.2 (RefS	-
Os04g0358000	Similar to OSIGBa0104J13.1 protein.	AK287896	Q01KT9	Alcohol dehydrogenase superfamily, zinc-type
Os04g0358150	Hypothetical protein.	EU945918	longestORF	-
Os04g0360400	Hypothetical protein.	EU945918	longestORF	-
Os04g0395600	Similar to TRANSPORT INHIBITOR RESPONSE 1 protein (F-box/LRR-repeat protein 1).	AK100862	XP_002328871.1 (RefS	F-box domain, cyclin-like
Os04g0395651	Hypothetical protein.	tplb0032b19 (Wheat FLcDNA)	longestORF	-
Os04g0397100	Similar to ClpC protease.	AK058510	E5GBL8	Chaperonin ClpA/B
Os04g0397400	Conserved hypothetical protein.	AK059476	Q7F9I0	-
Os04g0397601	Non-protein coding transcript.	CT835050	NONE	-
Os04g0398700	Similar to Pollen specific protein C13 precursor.	AK375503	Q01L34	Allergen Ole e 1, conserved site
Os04g0403200	Zinc finger, RING-type domain containing protein.	AK120702	B8Q8A9	Zinc finger, RING-type
Os04g0403300	Similar to Alpha-amylase.	CT836492	C3W8N1	Alpha-amylase, C-terminal beta-sheet
Os04g0403400	Tyrosyl-DNA phosphodiesterase family protein.	AK120071	B9FEZ9	Tyrosyl-DNA phosphodiesterase
Os04g0403450	Non-protein coding transcript.	BT086661	NONE	-
Os04g0403600	Conserved hypothetical protein.	tplb0006a06 (Wheat FLcDNA)	NP_001052700.2 (RefS	-
Os04g0405000	Similar to ATP-dependent Clp protease ATP-binding subunit.	AK061666	Q01L44	-
Os04g0405100	Similar to H0502B11.8 protein.	AK288033	Q01L43	Sterile alpha motif domain
Os04g0405150	Hypothetical gene.	BT019295	longestORF	-
Os04g0405700	Similar to H0502B11.13 protein.	tplb0022b18 (Wheat FLcDNA)	Q01L38	Protein of unknown function DUF677
Os04g0405800	Similar to OSIGBa0142C11.1 protein.	AK108757	Q01L58	Aspartate/glutamate/uridylate kinase
Os04g0406600	Prephenate dehydratase domain containing protein.	AK103609	Q01L56	Prephenate dehydratase
Os04g0407850	Similar to H0321H01.4 protein.	EU942896	Q01L52	-
Os04g0407900	Similar to Cytochrom P450-like protein.	AK064758	Q01L52	Cytochrome P450
Os04g0411200	Similar to ZPR3 (LITTLE ZIPPER 3)%3B protein binding.	FP099759	NP_190845.1 (RefSeq)	-
Os04g0411800	Similar to H0717B12.5 protein.	AK102032	Q01JB7	-
Os04g0414500	Similar to H0622F05.2 protein.	AK121479	Q01L94	-
Os04g0416000	Protein of unknown function DUF1279 domain containing protein.	AK241337	Q01I48	Domain of unknown function DUF1279
Os04g0416100	Similar to Transcription factor E2F1 (E2F-1) (Fragment).	AK067581	Q01I47	Transcription factor E2F/dimerisation partner
Os04g0418000	Conserved hypothetical protein.	AK106155 ,AK061030 ,AK09912	Q7X6C3	-
Os04g0418100	Hypothetical conserved gene.	CT835067	B9FF49	-
Os04g0420700	Hypothetical protein.	tplb0028c09 (Wheat FLcDNA)	longestORF	-
Os04g0420900	Similar to H0525E10.7 protein.	AK064414	Q01K01	S-locus glycoprotein
Os04g0421700	FAR1 domain containing protein.	AK072998	Q01JZ9	FAR1 DNA binding domain
Os04g0421800	EF-Hand type domain containing protein.	AK068951	Q01JZ8	EF-hand-like domain
Os04g0421850	Hypothetical protein.	EU949573	longestORF	-
Os04g0430800	Similar to OSIGBa0160I14.3 protein.	AK287766	Q01JS0	Thioredoxin
Os04g0431000	Conserved hypothetical protein.	AK111961	Q7XQQ0	-
Os04g0431100	Similar to GrpE protein homolog.	AK105181	A2XTK3	GrpE nucleotide exchange factor
Os04g0431800	Conserved hypothetical protein.	AK068094	Q7XQP5	-
Os04g0432100	Transcription factor GRAS domain containing protein.	BT018988	Q7XQP2	Transcription factor GRAS
Os04g0432250	Conserved hypothetical protein.	FP091622	Q7XQP1	-
Os04g0432300	Similar to 4-alpha-L-fucosyltransferase.	AM162282	Q2WBL8	Glycosyl transferase, family 10
Os04g0432400	Similar to neutral/alkaline invertase.	AK061496	NP_001147920.1 (RefS	Plant neutral invertase
Os04g0432500	Similar to Protein disulfide isomerase.	AK069367	Q01JF2	Thioredoxin
Os04g0432800	Similar to H0315E07.3 protein.	tplb0040l12 (Wheat FLcDNA)	Q01JF0	Pentatricopeptide repeat
Os04g0435700	Similar to UVB-resistance protein UVR8.	AK100857	A2XTP3	Regulator of chromosome condensation, RCC1
Os04g0436300	Protein disulphide isomerase isoform/multifunctional endoplasmic reticulum luminal polypeptide (D	AK101074	B9FFA7	Thioredoxin
Os04g0436700	Similar to H0315A08.1 protein.	FP099421	Q01I63	Ribonuclease H1, N-terminal

Os04g0436800	Pentatricopeptide repeat domain containing protein.	AK101115	Q01I62	Pentatricopeptide repeat
Os04g0437000	No apical meristem (NAM) protein domain containing protein.	AK243585	Q01I69	No apical meristem
Os04g0437300	Zinc finger, RING/FYVE/PHD-type domain containing protein.	AK121647	Q01I67	U box domain
Os04g0437401	Non-protein coding transcript.	X02683	NONE	-
Os04g0439900	Similar to Translocon Tic40 precursor.	AK065119	Q01K76	Heat shock chaperonin-binding
Os04g0440000	Protein of unknown function DUF2040, coiled-coil domain containing protein.	AK102407 ,AK101691	Q01K75	Domain of unknown function DUF2040
Os04g0441600	Similar to protein AIG1.	AK364676	NP_001148533.1 (RefS	AIG1
Os04g0441650	Non-protein coding transcript.	BT017369	NONE	-
Os04g0442700	Hypothetical gene.	AK119975	longestORF	-
Os04g0443200	Protein of unknown function DUF538 family protein.	AK107991	Q7XTH5	Protein of unknown function DUF538
Os04g0443750	Similar to CPRD8 protein.	ab initio prediction	P93699	NADH:flavin oxidoreductase/NADH oxidase, N-terminal
Os04g0443801	Cytochrome c oxidase biogenesis protein Cmc1-like domain containing protein.	AK104938	B8BJN1	Cytochrome c oxidase biogenesis protein Cmc1-like
Os04g0443850	Hypothetical protein.	CT831918	longestORF	-
Os04g0446700	Hypothetical protein.	AK073019	GeneMark	-
Os04g0446750	Hypothetical gene.	AK289000	GeneMark	-
Os04g0447100	Similar to Lipoxygenase.	AK287825	Q01J86	Lipoxygenase
Os04g0447166	Hypothetical protein.	EU945269	longestORF	-
Os04g0447300	Conserved hypothetical protein.	AK111006	Q5NA50	-
Os04g0447400	Similar to Glutamate decarboxylase 2 (EC 4.1.1.15) (GAD 2).	AK070858	Q01J85	Pyridoxal phosphate-dependent decarboxylase
Os04g0447533	Hypothetical protein.	AK375890	longestORF	-
Os04g0447650	Hypothetical gene.	EU946224	longestORF	-
Os04g0447800	Glutamate decarboxylase (EC 4.1.1.15).	AK068340	Q01J81	Pyridoxal phosphate-dependent decarboxylase
Os04g0447900	Domain of unknown function DUF623 domain containing protein.	AK376132	A2XTZ6	Domain of unknown function DUF623
Os04g0448100	Protein of unknown function DUF1644 family protein.	AK108771	Q01J79	Protein of unknown function DUF1644
Os04g0448200	Similar to OSIGBa0152K17.9 protein.	AK068842	Q01J78	-
Os04g0448250	Non-protein coding transcript.	tplb0058f15 (Wheat FLcDNA)	NONE	-
Os04g0448300	Peptidase A1 domain containing protein.	AK120786	Q01J77	Peptidase A1
Os04g0448600	ChaC-like protein family protein.	AK068709	Q01J75	ChaC-like protein
Os04g0448700	Similar to H0818E04.4 protein.	AK334544	Q01JH9	-
Os04g0449000	Similar to H0818E04.8 protein.	AK105976	Q01J70	Mitochondrial substrate/solute carrier
Os04g0449400	Similar to H0818E04.11 protein.	AK241313	Q01JH2	Protein phosphatase 2C-like
Os04g0449900	Similar to RNA Binding Protein 47.	AK069926	Q01JG9	RNA recognition motif domain
Os04g0449950	Non-protein coding transcript.	BT009377	NONE	-
Os04g0450000	Zinc finger, FYVE/PHD-type domain containing protein.	AK108406	Q01JG8	-
Os04g0450150	Hypothetical gene.	EU948502	longestORF	-
Os04g0450200	Pentatricopeptide repeat containing protein.	AK061063	Q01JG6	Pentatricopeptide repeat
Os04g0450400	Similar to H0818E04.19 protein.	AK121979	Q01JI7	Zinc finger, RING-type
Os04g0450500	Similar to H0818E04.20 protein.	AK058975	Q01JI6	-
Os04g0450600	Similar to H0818E04.21 protein.	CT835041	Q01JI5	-
Os04g0450701	Hypothetical protein.	EU969685	GeneMark	-
Os04g0453450	Hypothetical gene.	BT061593	longestORF	-
Os04g0455800	Similar to Lipoic acid synthetase, mitochondrial precursor (Lip-syn) (Lipoate synthase) (mLIP1).	AK120652	Q7XRF1	Lipoate synthase
Os04g0455900	Protein of unknown function DUF1692 domain containing protein.	AK121783	Q01JQ8	Domain of unknown function DUF1692
Os04g0457400	Conserved hypothetical protein.	EU953664	NP_001052973.2 (RefS	-
Os04g0457500	Similar to H0523F07.13 protein.	AK241500	Q01JQ0	Gamma-glutamyltranspeptidase
Os04g0457600	Non-protein coding transcript.	X02683	NONE	-
Os04g0457700	Similar to H0523F07.14 protein.	AK242111	Q01JP9	-
Os04g0457800	Similar to SERK1 (Fragment).	AK099777	Q5Y8C8	Protein kinase, catalytic domain
Os04g0457900	Non-protein coding transcript.	AM939987	NONE	-
Os04g0458200	Similar to ORW1943Ba0077G13.1 protein.	AK101398	F2QA78	-
Os04g0458250	Hypothetical protein.	tplb0041i03 (Wheat FLcDNA)	longestORF	-
Os04g0458600	Similar to ORW1943Ba0077G13.3 protein.	AK106058	F2QA80	Aldose 1-epimerase
Os04g0459500	Similar to H0219H12.1 protein.	AK067817	Q01IJ6	Glyceraldehyde 3-phosphate dehydrogenase, catalytic domain
Os04g0459600	Mog1/PsbP, alpha/beta/alpha sandwich domain containing protein.	AK061516	Q01IJ5	Ran-interacting Mog1 protein
Os04g0459700	Similar to H0219H12.3 protein.	AK109192	Q01IJ4	Nucleosome assembly protein
Os04g0459800	Similar to H0219H12.4 protein.	AK073573	Q01IJ3	Helicase, C-terminal
Os04g0459900	Peptidase, trypsin-like serine and cysteine domain containing protein.	AK065850	Q01IJ2	Peptidase S1/S6, chymotrypsin/Hap
Os04g0460050	Hypothetical conserved gene.	AB332064	F2QA89	-
Os04g0460300	Amino acid transporter, transmembrane domain containing protein.	AK106202	Q01IJ0	Amino acid transporter, transmembrane
Os04g0460350	Similar to predicted protein.	CT836521	B6U9R4	-
Os04g0460400	Uncharacterised protein family UPF0497, trans-membrane plant subgroup domain containing protein	FP091536	GeneMark	Uncharacterised protein family UPF0497, trans-membrane plant subgroup
Os04g0460500	Similar to H0219H12.9 protein.	BT037263	Q01II8	Molybdopterin cofactor biosynthesis C
Os04g0460600	Similar to H0219H12.10 protein.	AK071020	Q01II7	No apical meristem
Os04g0460750	Non-protein coding transcript.	X02683	NONE	-
Os04g0460900	Non-protein coding transcript.	AK066716 ,AK065774 ,AK10402	NONE	-
Os04g0461050	Hypothetical protein.	EU971393	longestORF	-
Os04g0461100	Ribosomal protein, PSRP-3/Ycf65 domain containing protein.	AK068022	Q01II5	Ribosomal protein PSRP-3/Ycf65
Os04g0461700	Non-protein coding transcript.	AK120338	NONE	-
Os04g0461800	Actin-binding FH2 domain containing protein.	AK106770	Q7XUV2	Actin-binding FH2/DRF autoregulatory
Os04g0461900	Hypothetical protein.	AK064527	longestORF	-
Os04g0462300	Conserved hypothetical protein.	AK109651	B8AUR5	-
Os04g0462500	Similar to 14-3-3-like protein GF14-6.	AK101938	Q7XTE8	14-3-3 protein
Os04g0462550	Hypothetical gene.	BT017754	longestORF	-
Os04g0463000	Serine/threonine protein kinase domain containing protein.	AK120857	Q25A94	Protein kinase, catalytic domain
Os04g0463200	Protein of unknown function DUF593 family protein.	AK243661	NP_001053008.1 (RefS	Protein of unknown function DUF593
Os04g0463300	Pentatricopeptide repeat containing protein.	AK119417	Q6MWD7	Pentatricopeptide repeat
Os04g0463400	Protein of unknown function DUF125, transmembrane family protein.	AK059730	C4B8E4	Domain of unknown function DUF125, transmembrane
Os04g0467550	Non-protein coding transcript.	BT017430	NONE	-
Os04g0467700	Similar to Indole-3-glycerol phosphate synthase, chloroplast precursor (EC 4.1.1.48) (IGPS).	AK101835	Q01IS1	Indole-3-glycerol phosphate synthase, conserved site
Os04g0469000	Heavy metal transport/detoxification protein domain containing protein.	AK062816	Q01IL4	-
Os04g0469400	Pentatricopeptide repeat domain containing protein.	AK060385	Q7XJU7	Pentatricopeptide repeat
Os04g0469425	Hypothetical protein.	AK357511	longestORF	-
Os04g0469500	Similar to OSIGBa0124N08.2 protein.	AK071065	Q01IL1	Phosphofructokinase domain
Os04g0469700	Hypothetical protein.	AK068788	longestORF	-
Os04g0469800	Cytochrome P450 724B1 (EC 1.14.-.-) (OsDWARF11) (Dwarf protein 11).	AK106528 ,AK119772	Q01JN4	Cytochrome P450
Os04g0507400	Similar to OSIGBa0157A06.5 protein.	EU957725	Q01IV4	Transferase
Os04g0507450	Hypothetical gene.	BT035701	longestORF	-
Os04g0507800	UbiE/COQ5 methyltransferase family protein.	AK111060	Q01I80	UbiE/COQ5 methyltransferase
Os04g0508500	Similar to OSIGBa0101P20.9 protein.	AK109620	Q01I73	SANT domain, DNA binding
Os04g0509100	Similar to triacylglycerol lipase.	AK359843	NP_193091.5 (RefSeq)	C2 calcium-dependent membrane targeting
Os04g0511200	EFA27 for EF hand, abscisic acid, 27kD.	AK243204	Q40679	Caleosin
Os04g0511500	Hypothetical protein.	EU940776	longestORF	-
Os04g0511600	Similar to RING-H2 finger protein ATL3G.	AK073609	Q01HE0	Zinc finger, RING-type
Os04g0511700	Similar to OSIGBa0157K09-H0214G12.18 protein.	AK102761	Q01HD9	-
Os04g0512250	Hypothetical gene.	AK288761	longestORF	-
Os04g0512300	Arp2/3 complex, 34kDa subunit p34-Arc family protein.	AK071791	Q01HD5	Arp2/3 complex, 34kDa subunit p34-Arc
Os04g0512400	Similar to Midline 1 protein (Tripartite motif protein 18).	AK103203	Q01HD4	BRCT
Os04g0512900	NB-ARC domain containing protein.	AK067204	Q7XPY8	Disease resistance protein
Os04g0513000	Conserved hypothetical protein.	AK102028	B9FG47	-
Os04g0513700	Similar to Beta-glucosidase 16.	BT039444	Q7XSK2	Glycoside hydrolase, family 1
Os04g0513800	Hypothetical protein.	tplb0059k05 (Wheat FLcDNA)	longestORF	-
Os04g0514500	Similar to OSIGBa0140J09.3 protein.	AK073502	Q01I18	Pentatricopeptide repeat
Os04g0514800	Similar to Dual specificity kinase 1.	AK111987	Q01I14	Protein kinase, catalytic domain
Os04g0514900	Hypothetical protein.	tplb0035e01 (Wheat FLcDNA)	longestORF	-
Os04g0515400	NTP pyrophosphohydrolase MazG, putative catalytic core domain containing protein.	AK336096	Q01I10	NTP pyrophosphohydrolase MazG, putative catalytic core
Os04g0516550	Conserved hypothetical protein.	AK333114	NP_001047774.1 (RefS	-
Os04g0516600	Pyridoxal phosphate-dependent transferase, major region, subdomain 1 domain containing protein.	AK059652 ,AK099280	Q01J56	Aromatic amino acid beta-eliminating lyase/threonine aldolase
Os04g0516701	Hypothetical gene.	BT017119	longestORF	-
Os04g0516800	Hypothetical protein.	AK105554	longestORF	-
Os04g0516900	Conserved hypothetical protein.	AK108714	Q7XKW4	-
Os04g0517100	Similar to OSIGBa0145M07.4 protein.	AK111798	Q01J53	Homeodomain-like
Os04g0517500	Similar to Phosphoenolpyruvate carboxylase kinase.	AK066885	Q2ABP5	Protein kinase, catalytic domain
Os04g0517900	Auxin responsive SAUR protein family protein.	AK241307	B5RHT9	Auxin responsive SAUR protein
Os04g0518000	Similar to Adenosine kinase (Fragment).	AK101926	Q01J47	Adenosine kinase
Os04g0518100	Phenylalanine ammonia-lyase.	AK100346	A2XVK1	Phenylalanine/histidine ammonia-lyase

Os04g0518400	Similar to Phenylalanine ammonia-lyase (Fragment).	AK067801	A2XVK3	Phenylalanine/histidine ammonia-lyase
Os04g0518650	Similar to OSIGBa0102I15.5 protein.	AK242408	Q01IW5	Small subunit of serine palmitoyltransferase-like
Os04g0518800	Similar to Lysine decarboxylase-like protein.	AK069293	Q8RUN2	Cytokinin riboside 5&apos%3B-monophosphate phosphoribohydrolase LOG
Os04g0519200	Similar to OSIGBa0132O24.3 protein.	EU951279	Q01MB4	-
Os04g0519700	Similar to Auxin response factor 10.	AK100795	Q7XKK6	Transcriptional factor B3
Os04g0519900	Similar to OSIGBa0145G11.5 protein.	AK071665	Q01I34	-
Os04g0519912	Hypothetical protein.	tplb0025b01 (Wheat FLcDNA)	longestORF	-
Os04g0519925	Similar to OSIGBa0145G11.5 protein.	ab initio prediction	Q01I34	Peptidase M28
Os04g0519950	Non-protein coding transcript.	EU954591	NONE	-
Os04g0519975	Similar to photosystem I subunit IX.	EU954469	YP_784493.1 (RefSeq)	Photosystem I PsaJ, reaction centre subunit IX
Os04g0520000	S1, RNA binding domain containing protein.	AK102988	Q01I33	RNA-binding domain, S1
Os04g0520700	Protein of unknown function DUF584 family protein.	AK065832	Q01IB5	Protein of unknown function DUF584
Os04g0520900	Pentatricopeptide repeat domain containing protein.	AK068793	Q01IB9	Pentatricopeptide repeat
Os04g0521001	Hypothetical protein.	FP096444	longestORF	-
Os04g0521100	Major intrinsic protein family protein.	AK243592	Q01IB8	Major intrinsic protein
Os04g0521300	Conserved hypothetical protein.	AK071350	B9FG70	-
Os04g0521700	Similar to OSIGBa0131L05.9 protein.	AK073622	Q01IB2	-
Os04g0521900	Similar to Vegetative cell wall protein gp1.	AK105173	B6SY74	-
Os04g0522100	Similar to OSIGBa0131L05.12 protein.	tplb0012i18 (Wheat FLcDNA)	Q01IA9	Knottin
Os04g0523200	Up-frameshift suppressor 2 domain containing protein.	AK119440	B9FG77	Up-frameshift suppressor 2
Os04g0523350	Similar to Initiation factor eIF-4 gamma, middle%3B Up-frameshift suppressor 2.	EU942804	Q2HSQ9	MIF4-like, type 1/2/3
Os04g0523600	Similar to OSIGBa0153E02-OSIGBa0093I20.8 protein.	AK070948	Q01HR7	UDP-glucuronosyl/UDP-glucosyltransferase
Os04g0523650	Hypothetical protein.	AK242174	longestORF	-
Os04g0524300	InterPro=Signal transduction response regulator, receiver domain (IPR001789),CheY-like superfami	GO=Biological Process: two-comp	NIAS_FLcDNA=002-1(	-
Os04g0524500	Oligopeptide transporter OPT superfamily protein.	AK067235	Q01HR1	Oligopeptide transporter OPT superfamily
Os04g0524800	Hypothetical protein.	AK064753	longestORF	-
Os04g0524900	Similar to OSIGBa0153E02-OSIGBa0093I20.16 protein.	AB190921	Q01HQ9	Oligopeptide transporter OPT superfamily
Os04g0524950	Hypothetical protein.	ab initio prediction	NONE	-
Os04g0525000	Protein of unknown function DUF177 domain containing protein.	AK067753	Q01HQ8	Protein of unknown function DUF177
Os04g0525600	ATP-dependent Clp protease proteolytic subunit (EC 3.4.21.92) (Endopeptidase Clp).	AK121934	Q01HQ5	Peptidase S14, ClpP
Os04g0525700	Similar to Serine carboxypeptidase II precursor (EC 3.4.16.6) (Carboxypeptidase D) (Bri1 suppressc	AK105830	Q01HQ4	Peptidase S10, serine carboxypeptidase
Os04g0525800	Similar to OSIGBa0115K01-H0319F09.1 protein.	ab initio prediction	Q01HQ1	Isochorismatase-like
Os04g0525850	Conserved hypothetical protein.	EU971861	NP_001144947.1 (RefS	-
Os04g0526300	Sulfotransferase family protein.	ab initio prediction	Q01HP8	Sulfotransferase domain
Os04g0526800	GRAM domain containing protein.	AK061581	Q01HP4	GRAM
Os04g0527000	GRAM domain containing protein.	AK099545 ,AK070324 ,AK10443	Q01HP3	-
Os04g0527300	Non-protein coding transcript.	AK110653	NONE	-
Os04g0527400	BRO1 domain containing protein.	AK107295	Q01HP2	BRO1 domain
Os04g0527500	Similar to OSIGBa0115K01-H0319F09.11 protein.	AK059144	Q01HP1	Mitochondrial carrier domain
Os04g0527700	CHCH domain containing protein.	AK072980	Q01HP0	-
Os04g0527800	Similar to OSIGBa0115K01-H0319F09.13 protein.	AK104019	Q01HN9	-
Os04g0527900	Similar to Tonoplast membrane integral protein ZmTIP3-2.	AK108116	Q01HN8	Major intrinsic protein
Os04g0528000	Similar to OSIGBa0115K01-H0319F09.15 protein.	BT018594	Q01HN7	Protein of unknown function DUF789
Os04g0528100	Similar to OSIGBa0115K01-H0319F09.16 protein.	AK065957	Q01HN6	-
Os04g0528200	Similar to OSIGBa0115K01-H0319F09.17 protein.	AK064693	Q01HN5	-
Os04g0528400	Similar to ABC transporter.	AK064120	Q01HN3	Hus1-like protein
Os04g0528600	Similar to OSIGBa0115K01-H0319F09.22 protein.	AK240918	Q01HN0	-
Os04g0528651	Hypothetical protein.	tplb0055d11 (Wheat FLcDNA)	longestORF	-
Os04g0528800	Similar to Ferredoxin-thioredoxin reductase, variable chain (FTR-V) (Ferredoxin- thioredoxin reduc	AK103671	Q01HM9	Lipoate synthase
Os04g0529100	Pathogenesis-related transcriptional factor and ERF domain containing protein.	AK107680	Q01HM7	Pathogenesis-related transcriptional factor/ERF, DNA-binding
Os04g0529400	Similar to OO_Ba0013J05-OO_Ba0033A15.30 protein.	AK099627	D0ABH3	WD40 repeat
Os04g0529500	Similar to cDNA clone:J023022A09, full insert sequence.	AK059419	B7EGJ9	Double-stranded RNA-binding
Os04g0529700	Glycosyltransferase sugar-binding region containing DXD motif domain containing protein.	AK121532	Q00RJ5	Glycosyltransferase, DXD sugar-binding motif
Os04g0529800	Sugar/inositol transporter domain containing protein.	AK069679	Q00RJ4	Sugar/inositol transporter
Os04g0530801	Hypothetical gene.	EU973535	longestORF	-
Os04g0530900	Glycosyl transferase, family 8 protein.	AK072297	XP_002312381.1 (RefS	Glycosyl transferase, family 8
Os04g0531300	tRNA-dihydrouridine synthase domain containing protein.	AK103911	Q00RI1	tRNA-dihydrouridine synthase
Os04g0531400	Similar to Lectin-like receptor kinase 7%3B2.	AK105289	Q00RI0	Protein kinase, catalytic domain
Os04g0531500	Concanavalin A-like lectin/glucanase domain containing protein.	AK102285	Q00RH9	Protein kinase, catalytic domain
Os04g0531600	Hypothetical protein.	AK107678	longestORF	-
Os04g0531750	Similar to OSIGBa0125M19.13 protein.	ab initio prediction	Q00RH3	Short-chain dehydrogenase/reductase SDR
Os04g0531800	Conserved hypothetical protein.	AK121355	B8ASD8	-
Os04g0532100	Short-chain dehydrogenase/reductase SDR domain containing protein.	AK109281	Q00RH5	Short-chain dehydrogenase/reductase SDR
Os04g0532400	Similar to OSIGBa0125M19.13 protein.	AK318556	Q00RH3	Short-chain dehydrogenase/reductase SDR
Os04g0532500	Similar to Transcription factor L2.	AK069907	Q00RH2	Zinc finger, LIM-type
Os04g0533200	Similar to Myb7 protein (Fragment).	AK376409	Q43598	-
Os04g0533250	Hypothetical gene.	EU971393	longestORF	-
Os04g0533500	Cytochrome b561 family protein.	AK061426 ,AK069219	Q01II4	-
Os04g0533800	Similar to OSIGBa0159I10.4 protein.	BT040291	Q01II1	Protease inhibitor I4, serpin
Os04g0534166	Similar to OSIGBa0159I10.8 protein.	ab initio prediction	Q01IH7	Leucine-rich repeat-containing N-terminal, type 2
Os04g0534200	Protein kinase, core domain containing protein.	AK109583	Q01IH7	Protein kinase, catalytic domain
Os04g0534500	Similar to OSIGBa0159I10.11 protein.	AK105805	Q01IH4	-
Os04g0534600	Peroxisomal biogenesis factor 11 family protein.	AK073835 ,AK287635	Q7XU74	Peroxisomal biogenesis factor 11
Os04g0535200	Peptidase A1 domain containing protein.	AK101505 ,AK060585 ,AK11976	Q01IH2	-
Os04g0535600	Similar to Beta-fructofuranosidase 1 precursor (EC 3.2.1.26) (Sucrose 1) (Invertase 1).	AK099312 ,AK065130	Q01IH0	Glycoside hydrolase, family 32
Os04g0536300	Transcription factor with zinc finger domain and helix-loop-helix domain (YABBY domain), Leaf de	AK070205 ,AB274017	B7EHI2 ,Q0JBF0	-
Os04g0536500	Similar to H0502G05.5 protein.	AK109552	Q01JG1	No apical meristem
Os04g0537100	Similar to Auxin-induced protein X15.	AK108118	Q01JF8	Auxin responsive SAUR protein
Os04g0537450	Non-protein coding transcript.	CT835085	NONE	-
Os04g0538166	Similar to Elongation factor G, chloroplastic.	P35450	P35450	Protein synthesis factor, GTP-binding
Os04g0538300	Hypothetical conserved gene.	ab initio prediction	NP_001174027.1 (RefS	-
Os04g0538400	Similar to Nodulin 21 (N-21).	AK108230	Q9M2C3	Domain of unknown function DUF125, transmembrane
Os04g0538700	Autophagy-related protein 13 domain containing protein.	AK105628	Q7XU21	Autophagy-related protein 13
Os04g0538900	Glyoxalase/bleomycin resistance protein/dioxygenase domain containing protein.	AK333604	NP_001053434.1 (RefS	-
Os04g0539000	Protein of unknown function DUF2485 domain containing protein.	AK105343	Q7FB12	NAD
Os04g0539100	Conserved hypothetical protein.	AK069820	A2XVZ2	-
Os04g0539300	Similar to Aldehyde 5-hydroxylase.	AK248286	Q9SWR1	Cytochrome P450
Os04g0539500	Similar to H0115B09.1 protein.	AK060574 ,AK099144	Q01IQ1	Zinc finger, GATA-type
Os04g0539601	Hypothetical gene.	EU947136	longestORF	-
Os04g0539701	Conserved hypothetical protein.	CT835025	NP_001060202.1 (RefS	-
Os04g0539800	Src homology-3 domain containing protein.	AK240927	Q01IQ0	Src homology-3 domain
Os04g0540200	Zinc finger, B-box domain containing protein.	AK061333	Q01IP9	Zinc finger, B-box
Os04g0540300	COBRA-like protein 7.	Q7XR91	Q7XR91	Glycosyl-phosphatidyl inositol-anchored, plant
Os04g0540401	Hypothetical protein.	tplb0046p06 (Wheat FLcDNA)	longestORF	-
Os04g0540900	Serine/threonine protein kinase domain containing protein.	AK066397	Q01IP5	Protein kinase, catalytic domain
Os04g0542100	Similar to HAT family dimerisation domain containing protein.	AK288170	Q53RM1	HAT dimerisation
Os04g0586200	Similar to H0307D04.13 protein.	AK120315	Q01K53	Protein of unknown function DUF581
Os04g0590283	Hypothetical protein.	BT019283	longestORF	-
Os04g0609700	Similar to H0702G05.10 protein.	AK058541	Q25AI3	-
Os04g0610500	Peptidase M24A, methionine aminopeptidase, subfamily 1 protein.	AK066289 ,AK104817	B8ATX6	Peptidase M24, structural domain
Os04g0610700	Similar to Potassium transporter 13 (AtPOT13) (AtKT5).	AK100672	Q01JS6	K+ potassium transporter
Os04g0611267	Similar to OSIGBa0152L12.11 protein.	ab initio prediction	Q01JS3	-
Os04g0613500	Similar to 40S ribosomal protein S11.	D29727	NP_001105562.1 (RefS	Ribosomal protein S17
Os04g0616650	Non-protein coding transcript.	EU949024	NONE	-
Os04g0638700	Conserved hypothetical protein.	AK062886	B8AV68	-
Os04g0639100	Conserved hypothetical protein.	AK063036	Q7XPH6	-
Os04g0639300	Conserved hypothetical protein.	AK058483	Q7XPH4	-
Os04g0639800	Similar to OSIGBa0138H21-OSIGBa0138E01.9 protein.	AK243114	Q01HX4	-
Os04g0640200	Conserved hypothetical protein.	AK103423	Q7XPG4	-
Os04g0640600	Shikimate kinase.	AK070318	Q01HY3	Shikimate kinase
Os04g0640700	Similar to Alpha-L-arabinofuranosidase/beta-D-xylosidase isoenzyme ARA-I.	AK101782	Q01HY2	Glycoside hydrolase, family 3, N-terminal
Os04g0640750	Hypothetical protein.	tplb0030f15 (Wheat FLcDNA)	longestORF	-
Os04g0664400	Auxin response factor 1.	AK103452	Q8S983	AUX/IAA protein
Os04g0665900	Similar to H1005F08.13 protein.	FP097147	Q01MN1	VQ
Os04g0667033	Non-protein coding transcript.	BT018595	NONE	-

Os04g0668000	Conserved hypothetical protein.	AK062740	Q7XR77	-
Os04g0668700	Similar to phosphatidylinositol 3- and 4-kinase family protein.	AK062068	NP_001151804.1 (RefS	Phosphatidylinositol 3-/4-kinase, catalytic
Os04g0668800	Putative thiol-disulphide oxidoreductase DCC family protein.	AK288046	Q8W437	Putative thiol-disulphide oxidoreductase DCC
Os04g0668900	Similar to Plastid terminal oxidase.	AK067891	B8AW16	Alternative oxidase
Os04g0669300	EF hand domain containing protein.	AK071148	NP_001030731.1 (RefS	EPS15 homology
Os04g0669375	Non-protein coding transcript.	tplb0032i10 (Wheat FLcDNA)	NONE	-
Os04g0669600	Phospholipase/carboxylesterase domain containing protein.	AK110767	Q259P1	Phospholipase/carboxylesterase/thioesterase
Os04g0670000	Reticulon family protein.	AK071792	Q259P4	Reticulon
Os04g0670200	Granulin domain containing protein.	AK099358	GeneMark	Granulin
Os04g0670900	Homeodomain-like containing protein.	DQ383374	Q258Z1	MYB-like
Os04g0671100	Similar to H0624F09.8 protein.	AK241125	Q258Z0	Adenylate kinase
Os04g0671300	Similar to Suppressor of presenilin 5 (P110b homolog).	AK072414	Q258Y8	Flavin amine oxidase
Os04g0671500	Hypothetical protein.	tplb0048c15 (Wheat FLcDNA)	longestORF	-
Os04g0688500	Peroxidase (EC 1.11.1.7).	AK067667	Q5U1N1	Plant peroxidase
Os04g0691600	Similar to 30S ribosomal protein S17.	AK059422	Q9ZST1	Ribosomal protein S17
Os05g0119700	Conserved hypothetical protein.	AK062482	Q9LIW7	-
Os05g0119850	Hypothetical protein.	tplb0022d09 (Wheat FLcDNA)	longestORF	-
Os05g0127266	Non-protein coding transcript.	AK248585	NONE	-
Os05g0127400	Similar to DnaJ subfamily B member 13.	AK069823	B6TKS5	Heat shock protein DnaJ, N-terminal
Os05g0127450	Non-protein coding transcript.	tplb0026i01 (Wheat FLcDNA)	NONE	-
Os05g0129700	KNOX class homeodomain protein (Knotted1-type homeobox protein OSH71).	AK111878	Q7GDL5	Homeobox
Os05g0129800	DNA-binding WRKY domain containing protein.	AK241799	NP_001174199.1 (RefS	DNA-binding WRKY
Os05g0129900	Tetratricopeptide-like helical domain containing protein.	AK060436	B9FMA1	Tetratricopeptide TPR-1
Os05g0138300	Similar to ICT protein (Fragment).	AK119249	Q9SXZ4	-
Os05g0139300	F-box domain, cyclin-like domain containing protein.	ab initio prediction	NP_001054595.1 (RefS	F-box domain, cyclin-like
Os05g0142500	Hypothetical conserved gene.	ab initio prediction	A2Y0A3	-
Os05g0143000	Conserved hypothetical protein.	AK064113	B8AXW7	-
Os05g0143300	Conserved hypothetical protein.	AK072841 ,AK099030	B9FH60	-
Os05g0143500	Similar to Kinase (Fragment).	AK061851	C4PE55	-
Os05g0143600	Similar to Jasmonate-induced protein.	AK107844	A2Y0B0	Mannose-binding lectin
Os05g0144100	WD40 repeat-like domain containing protein.	AK069280	B6U179	WD40 repeat
Os05g0144150	Hypothetical gene.	BT084538	longestORF	-
Os05g0144200	Similar to glioma tumor suppressor-like protein.	AK242272	B6T3I5	Tumour suppressor protein Gltscr2
Os05g0149300	Similar to 1-aminocyclopropane-1-carboxylate oxidase.	BT087584	O81606	Oxoglutarate/iron-dependent oxygenase
Os05g0149400	Similar to 1-aminocyclopropane-1-carboxylic acid oxidase.	AK061064	O81606	Oxoglutarate/iron-dependent oxygenase
Os05g0149450	Non-protein coding transcript.	tplb0008n13 (Wheat FLcDNA)	NONE	-
Os05g0149600	Similar to CULLIN1-like protein 1.	AK073947	C7SJ62	-
Os05g0149950	Monothiol glutaredoxin-S8.	P0C290	P0C290	Glutaredoxin
Os05g0150550	Hypothetical protein.	tplb0032b19 (Wheat FLcDNA)	longestORF	-
Os05g0150600	Hypothetical conserved gene.	AK063338	D7U9T8	DNA helicase, ATP-dependent, RecQ type
Os05g0150900	Histidyl-tRNA synthetase (EC 6.1.1.21) (Histidine--tRNA ligase) (HisRS).	AK099789	P93422	Phenylalanine/histidine ammonia-lyase
Os05g0151000	Similar to DNA-directed RNA polymerase.	AK371102	C5YGY9	RNA polymerase II, heptapeptide repeat, eukaryotic
Os05g0151100	Similar to desiccation-related protein PCC13-62.	EU967146	B6TNA6	-
Os05g0151150	Hypothetical gene.	EU975566	GeneMark	-
Os05g0151200	TspO/MBR-related protein family protein.	AK240936	NP_001147341.1 (RefS	TspO/MBR-related protein
Os05g0151300	Rubber elongation factor family protein.	AK243548	A2Y0H2	Rubber elongation factor
Os05g0151400	AIG1 domain containing protein.	AK102924	XP_002874910.1 (RefS	Chloroplast protein import component Toc86/159
Os05g0152266	Hypothetical gene.	EU943470	longestORF	-
Os05g0159400	Non-protein coding transcript.	EU942788	NONE	-
Os05g0160000	Similar to Ferric leghemoglobin reductase.	AK063865	B9FML1	Pyridine nucleotide-disulphide oxidoreductase, dimerisation
Os05g0160200	Ubiquitin.	AK119731	A6MZJ4	Ubiquitin
Os05g0170250	Hypothetical gene.	AK243238	longestORF	-
Os05g0170300	Leucine-rich repeat domain containing protein.	ab initio prediction	Q65XS3	Leucine-rich repeat
Os05g0173700	Similar to DNA replication licensing factor MCM3 homolog (Replication origin activator) (ROA pro	AK063470	NP_001105718.1 (RefS	Mini-chromosome maintenance, DNA-dependent ATPase
Os05g0176100	Similar to Cellulose synthase BoCesA1.	AK067850	D5FJ45	Cellulose synthase
Os05g0176700	Conserved hypothetical protein.	AK071729	A2Y0X6	-
Os05g0177100	F-box domain, cyclin-like domain containing protein.	AK064652	B9FMR7	F-box domain, cyclin-like
Os05g0178950	Hypothetical protein.	tplb0040d24 (Wheat FLcDNA)	longestORF	-
Os05g0179050	Hypothetical protein.	tplb0030e06 (Wheat FLcDNA)	longestORF	-
Os05g0182600	Similar to SSRP1 protein.	AK102592	Q65WY8	High mobility group, HMG1/HMG2
Os05g0182650	Conserved hypothetical protein.	AK240762	NP_001174249.1 (RefS	-
Os05g0182700	Protein of unknown function DUF1664 family protein.	AK065911 ,AK104343	A2Y139	Protein of unknown function DUF1664
Os05g0182800	Similar to glutaminyl-tRNA synthetase.	AK061951	NP_001152305.1 (RefS	Glutamyl/glutaminyl-tRNA synthetase, class Ib
Os05g0183200	Similar to barley mlo defense gene homolog6.	ab initio prediction	NP_001105170.1 (RefS	Mlo-related protein
Os05g0183300	Similar to barley mlo defense gene homolog6.	ab initio prediction	NP_001105170.1 (RefS	Mlo-related protein
Os05g0183900	Similar to transposon protein CACTA, En/Spm sub-class.	AK360948	Q65WX7	Putative harbinger transposase-derived nuclease
Os05g0184016	Hypothetical protein.	AK372466	longestORF	-
Os05g0184901	Phospholipase A2, active site domain containing protein.	AK287961	B6SGX9	-
Os05g0185650	Similar to predicted protein.	AK242365	XP_002867220.1 (RefS	-
Os05g0185700	Similar to predicted protein.	AK064724	NP_200842.1 (RefSeq)	MaoC-like dehydratase
Os05g0185767	Hypothetical gene.	AK288224	longestORF	-
Os05g0185800	Conserved hypothetical protein.	AK065050	A2Y150	-
Os05g0186000	Similar to PSRP4.	AK058675	B6T2D1	-
Os05g0186601	Hypothetical gene.	EU969101	longestORF	-
Os05g0186900	Similar to Auxin-responsive protein IAA16.	AK111403	P0C127	-
Os05g0187000	Beta 2 subunit of 20S proteasome (20S proteasome beta subunit).	AK103126	A2Y157	Peptidase T1A, proteasome beta-subunit
Os05g0187100	Similar to Hexokinase.	AK067988	Q1WM16	Hexokinase
Os05g0217950	Non-protein coding transcript.	tplb0043a14 (Wheat FLcDNA)	NONE	-
Os05g0218001	Myb/SANT-like domain domain containing protein.	BT039927	NP_001176912.1 (RefS	Myb/SANT-like domain
Os05g0259601	Non-protein coding transcript.	EU949024	NONE	-
Os05g0263500	Non-protein coding transcript.	AK101739	NONE	-
Os05g0267100	Hypothetical conserved gene.	AK065811	Q6ATF1	-
Os05g0268400	Similar to Major facilitator superfamily antiporter.	AK288425	Q8H6D6	-
Os05g0268500	Similar to Serine carboxypeptidase 2.	AK119979	P08819	Peptidase S10, serine carboxypeptidase
Os05g0269100	Cyclin-like F-box domain containing protein.	AK066309	A2Y2K4	F-box domain, cyclin-like
Os05g0269200	Similar to inner membrane transport protein.	CT836417	NP_001147344.1 (RefS	C4-dicarboxylate transporter/malic acid transport protein
Os05g0269500	Generative cell specific-1, HAP2-GCS1 domain containing protein.	AK072871	Q2PGG7	Generative cell specific-1, HAP2-GCS1
Os05g0272300	NmrA-like domain containing protein.	ab initio prediction	NP_001041775.1 (RefS	NmrA-like
Os05g0272400	Hypothetical protein.	AK110636	longestORF	-
Os05g0272632	Non-protein coding transcript.	EU949846	NONE	-
Os05g0272900	B-cell receptor-associated 31-like family protein.	AK107093	B6TG43	B-cell receptor-associated 31-like
Os05g0273500	Conserved hypothetical protein.	AK066374	B8AW21	-
Os05g0274300	Similar to ATP binding protein.	AK071399	NP_001150449.1 (RefS	-
Os05g0274900	Similar to 60S ribosomal protein L15-1.	O23515	O23515	Ribosomal protein L15e
Os05g0275100	Pentatricopeptide repeat domain containing protein.	ab initio prediction	B9FNL7	Pentatricopeptide repeat
Os05g0275600	Conserved hypothetical protein.	AK108098	XP_002464690.1 (RefS	-
Os05g0275700	Similar to Peroxisome assembly protein 2 (Peroxin-2) (AthPEX2) (Pex2p).	AK111642	B4FCL8	Zinc finger, RING-type
Os05g0276100	Similar to Na+/H+ exchangeing protein-like (Cation/H+ exchanger).	AK069092	XP_002314350.1 (RefS	Cation/H+ exchanger
Os05g0276500	Expansin Os-EXPA3.	AK060313	Q40637	Expansin
Os05g0276750	Hypothetical protein.	BT016845	longestORF	-
Os05g0277000	Similar to Expansin Os-EXPA3.	P0C1Y4	P0C1Y4	Expansin
Os05g0277100	Hypothetical protein.	BT016845	longestORF	-
Os05g0277300	Similar to Peptide chain release factor 1.	AK065607	B6U6C9	-
Os05g0277500	Similar to Germin-like protein subfamily 2 member 4 precursor.	AK105351	NP_001151047.1 (RefS	Germin
Os05g0278150	Non-protein coding transcript.	EU949846	NONE	-
Os05g0278500	Transferase family protein.	AK067851	B6TZX2	Transferase
Os05g0278550	Hypothetical gene.	EU944389	longestORF	-
Os05g0279850	Hypothetical protein.	tplb0028j17 (Wheat FLcDNA)	longestORF	-
Os05g0279900	Similar to glycoside hydrolase family 28 protein / polygalacturonase (pectinase) family protein.	ab initio prediction	NP_175244.1 (RefSeq)	Glycoside hydrolase, family 28
Os05g0280200	Similar to Ras-related protein RGP2.	AK120316	A2Y2Q3	Small GTPase superfamily
Os05g0280350	Hypothetical gene.	BT084300	longestORF	-
Os05g0283200	Pectinesterase inhibitor domain containing protein.	AK120717	A2Y2R2	Pectinesterase inhibitor
Os05g0286100	Similar to Zinc-finger protein KNUCKLES.	AK108829	A2Y2R5	Zinc finger, C2H2
Os05g0287800	Similar to Phosphatidic acid phosphatase-like protein.	AK071727	E5FY32	-
Os05g0289400	Similar to CRN (Crooked neck) protein.	AK100478	Q9FJ37	RNA-processing protein, HAT helix

Os05g0289700	Arbuscular mycorrhizal specific marker 10.,Benzyl alcohol benzoyl transferase.	BT086008	Q01IV4	Transferase
Os05g0290000	Hypothetical protein.	BT035701	longestORF	-
Os05g0291600	Hypothetical conserved gene.	AK073226	NP_001059231.1 (RefS	-
Os05g0292800	Similar to One helix protein (OHP).	AK059562	Q8LPV2	Chlorophyll a/b binding protein domain
Os05g0294800	Hypothetical gene.	AK065926	longestORF	-
Os05g0295200	Conserved hypothetical protein.	AK073450	B9FNQ1	-
Os05g0295900	Conserved hypothetical protein.	AK069962	B9FNQ4	-
Os05g0296600	Non-protein coding transcript.	AK059372	NONE	-
Os05g0296750	Hypothetical gene.	AK063686	longestORF	-
Os05g0296900	Hypothetical conserved gene.	AK100725	B8AWA5	-
Os05g0297900	Similar to Signal peptidase 18 subunit (Fragment).	AK071238	B6T545	Peptidase S26B, eukaryotic signal peptidase
Os05g0298200	Ankyrin repeat containing protein.	AK066758	A2Y2V7	Ankyrin repeat
Os05g0298300	Hypothetical protein.	tplb0059e11 (Wheat FLcDNA)	longestORF	-
Os05g0299000	Conserved hypothetical protein.	AK108720	B8AWB0	-
Os05g0299200	Hypothetical conserved gene.	EU956990	NP_001055128.1 (RefS	Nucleotide-sugar transporter
Os05g0299250	Hypothetical protein.	tplb0062m11 (Wheat FLcDNA)	longestORF	-
Os05g0299300	Conserved hypothetical protein.	AK111812	B9FNQ8	-
Os05g0301500	Similar to Ribophorin I (Fragment).	AK070329	NP_001151620.1 (RefS	Ribophorin I
Os05g0302916	Hypothetical protein.	tplb0044f08 (Wheat FLcDNA)	longestORF	-
Os05g0303000	Similar to Chloroplast heat shock protein 70.	AK060410	A4ZYQ0	Heat shock protein Hsp70
Os05g0303700	Hypothetical conserved gene.	ab initio prediction	A2Y2Y7	RNA recognition motif domain
Os05g0304501	Hypothetical gene.	CT833082	longestORF	-
Os05g0304701	Hypothetical gene.	EU945269	longestORF	-
Os05g0328901	Similar to Prolamin.	AK242260	Q5EFA4	-
Os05g0329001	Similar to Prolamin.	AF042201	Q5EFA4	-
Os05g0329100	Prolamin.	AK242260	Q5EFA4	Bifunctional trypsin/alpha-amylase inhibitor
Os05g0329200	Similar to Prolamin.	AK242260	Q5EFA4	Bifunctional trypsin/alpha-amylase inhibitor
Os05g0329300	Prolamin.	AK242260	Q5EFA4	Bifunctional trypsin/alpha-amylase inhibitor
Os05g0329350	Prolamin.	EF122440	Q5EFA4	Bifunctional trypsin/alpha-amylase inhibitor
Os05g0329700	Similar to Prolamin.	AK242910	Q5EFA4	Bifunctional trypsin/alpha-amylase inhibitor
Os05g0355800	Similar to Lipoxygenase.	DQ658739	Q01J86	Lipoxygenase
Os05g0356700	Protein of unknown function DUF231, plant domain containing protein.	AK071031	XP_002884829.1 (RefS	Domain of unknown function DUF231, plant
Os05g0356800	Protein of unknown function DUF3511 domain containing protein.	AK121901	A2Y3M8	Protein of unknown function DUF3511
Os05g0363533	Non-protein coding transcript.	EU948047	NONE	-
Os05g0363566	Hypothetical conserved gene.	CT836447	Q688I1	-
Os05g0375532	Hypothetical gene.	AK287793	longestORF	-
Os05g0375600	Similar to Peptide chain release factor 2.	AK376656	B6SSD2	-
Os05g0375700	Hypothetical conserved gene.	BT054511	Q6AUQ1	Peptidase A1
Os05g0375850	Hypothetical protein.	AK360759	longestORF	-
Os05g0377450	Similar to H0315A08.1 protein.	FP099421	Q01I63	Ribonuclease H1, N-terminal
Os05g0377600	Non-protein coding transcript.	EU941912	NONE	-
Os05g0377750	Non-protein coding transcript.	EU941912	NONE	-
Os05g0391400	Non-protein coding transcript.	EU941912	NONE	-
Os05g0392300	Similar to Cyclin-dependent kinase D-1.	AK062401	P29620	-
Os05g0399900	Conserved hypothetical protein.	EU976876	B9FVF5	-
Os05g0408800	Similar to zinc finger, C3HC4 type family protein.	EU964917	NP_001149860.1 (RefS	Zinc finger, RING-type
Os05g0408850	Hypothetical gene.	CT836350	longestORF	-
Os05g0409500	Similar to MtN21 protein.	BT084636	NP_001150619.1 (RefS	Drug/metabolite transporter
Os05g0410750	Hypothetical protein.	tplb0038k08 (Wheat FLcDNA)	longestORF	-
Os05g0410800	TGF-beta receptor, type I/II extracellular region family protein.	AK108312	NP_001147810.1 (RefS	Oligopeptide transporter
Os05g0411150	Hypothetical protein.	tplb0038k08 (Wheat FLcDNA)	longestORF	-
Os05g0411300	Similar to DNA binding protein.	AK073142	NP_001148077.1 (RefS	Basic-leucine zipper
Os05g0419500	K Homology domain containing protein.	AK073986	NP_001148920.1 (RefS	K Homology
Os05g0473500	Exo70 exocyst complex subunit family protein.	AK101630	B6U0J9	Exo70 exocyst complex subunit
Os05g0476250	Non-protein coding transcript.	EU951628	NONE	-
Os05g0476466	Similar to CBL-interacting protein kinase 28.	A3B529	A3B529	Protein kinase, catalytic domain
Os05g0476583	Non-protein coding transcript.	tplb0042a18 (Wheat FLcDNA)	NONE	-
Os05g0477666	Hypothetical gene.	BT016845	longestORF	-
Os05g0478250	Hypothetical protein.	AK369221	longestORF	-
Os05g0478300	Hypothetical conserved gene.	AK242838	NP_001055847.2 (RefS	Protein kinase, catalytic domain
Os05g0481500	K Homology domain containing protein.	AK072560	NP_001150991.1 (RefS	K Homology
Os05g0486833	Hypothetical conserved gene.	BT065526	C0HHH7	-
Os05g0487300	Conserved hypothetical protein.	AK062926	B8AZG8	-
Os05g0489301	Non-protein coding transcript.	CT836470	NONE	-
Os05g0489800	Centromeric histone 3 (Histone H3).	AK101975	Q6T367	Histone H3
Os05g0491800	Hypothetical protein.	tplb0061j19 (Wheat FLcDNA)	longestORF	-
Os05g0494100	Similar to dr1-associated corepressor.	AK060192	A2Y627	-
Os05g0494800	Hypothetical protein.	AK069939	GeneMark	-
Os05g0494900	SH2 motif domain containing protein.	AK106396	NP_001077775.1 (RefS	SH2 motif
Os05g0495000	Hypothetical conserved gene.	CT829003	B9FKX7	Zinc finger, RING-type
Os05g0495200	Basic leucine zipper domain containing protein.	AK072281	D6MKB2	Basic-leucine zipper
Os05g0495250	Non-protein coding transcript.	CT836397	NONE	-
Os05g0495700	Similar to Glycerol-3-phosphate dehydrogenase.	AK101484	B6SKP4	Glycerol-3-phosphate dehydrogenase, NAD-dependent, C-terminal
Os05g0496100	Translation initiation factor eIF3 subunit domain containing protein.	ab initio prediction	NP_001055936.1 (RefS	Translation initiation factor eIF3 subunit
Os05g0496200	Similar to 3-phosphoglycerate kinase (Fragment).	AK287421	P12782	Phosphoglycerate kinase
Os05g0496300	Chalcone/stilbene synthase, N-terminal domain containing protein.	AY286097	A2Y651	Chalcone/stilbene synthase, N-terminal
Os05g0509101	Conserved hypothetical protein.	AK110690	Q6L4X0	-
Os05g0528101	Hypothetical gene.	EU950506	longestORF	-
Os05g0547200	Conserved hypothetical protein.	AK375772	NP_001047613.2 (RefS	-
Os05g0547600	Hypothetical protein.	AK102181	longestORF	-
Os05g0547700	Conserved hypothetical protein.	AK070926	B9G5C2	-
Os05g0557500	Protein of unknown function DUF2921 domain containing protein.	AK067152	B9FLK1	Protein of unknown function DUF2921
Os05g0557800	Similar to 50S ribosomal protein L21.	AK105327	B6TJJ8	Ribosomal protein L21
Os05g0557950	Hypothetical protein.	EU949682	longestORF	-
Os05g0558101	Conserved hypothetical protein.	EU957461	Q6ZJX2	-
Os05g0558501	Conserved hypothetical protein.	EU957461	Q6ZJX2	-
Os05g0565100	Cytochrome b561/ferric reductase transmembrane domain containing protein.	AK063277	B8AWR7	Cytochrome b561, eukaryote
Os05g0565900	DNA-binding WRKY domain containing protein.	FP101665	Q688X5	DNA-binding WRKY
Os05g0566200	NLI interacting factor domain containing protein.	AK099836	B9FLM9	NLI interacting factor
Os05g0566300	PRC-barrel domain containing protein.	AK099641	B9FI59	RimM protein
Os05g0566500	Similar to Initiation factor 3d (Fragment).	AK073468	B6U4Q2	Eukaryotic translation initiation factor 3, subunit 7
Os05g0566800	Cold acclimation protein COR413-TM1.	AK065748	Q84XU8	Cold acclimation WCOR413
Os05g0567100	Similar to Phytepsin.	AK100749	P42210	Peptidase A1
Os05g0567900	Hypothetical conserved gene.	GU722206	B9FLN6	Zinc finger, GATA-type
Os05g0568600	Protein of unknown function DUF1645 family protein.	AK063781	B9FI71	Protein of unknown function DUF1645
Os05g0570350	Protein of unknown function DUF295 domain containing protein.	AK241838	GeneMark	Protein of unknown function DUF295
Os05g0571100	Peptidase C12, ubiquitin carboxyl-terminal hydrolase 1 domain containing protein.	AK068658	NP_001118908.1 (RefS	Plant organelle RNA recognition domain
Os05g0594800	Similar to Adhesion regulating molecule conserved region family protein.	AK058332 ,AK068013	B4G1K7	26S proteasome complex ubiquitin receptor, subunit Rpn13
Os05g0594900	Similar to U6 snRNA-associated Sm-like protein LSm8.	AK121070	B4FNR5	Like-Sm ribonucleoprotein
Os05g0595000	Allergen V5/Tpx-1 related family protein.	AK071326	A2Y860	Allergen V5/Tpx-1-related
Os05g0595100	Similar to UDP-glucose-4-epimerase.	AK066249	A2Y861	NAD-dependent epimerase/dehydratase
Os05g0595200	Allergen V5/Tpx-1 related family protein.	AK070317	B8AXH6	Allergen V5/Tpx-1-related
Os05g0595300	Similar to CCT motif family protein.	AK059614	NP_001151864.1 (RefS	-
Os05g0595500	Pleckstrin homology-type domain containing protein.	AK242184	NP_001078437.1 (RefS	Pleckstrin homology domain
Os05g0596000	Peptidase A1 domain containing protein.	AK121242 ,AK068911	NP_001149154.1 (RefS	Peptidase A1
Os05g0596200	Similar to cDNA clone:001-104-D04, full insert sequence.	AK107887	B7E7K4	-
Os05g0596500	Similar to RING-H2 finger protein ATL3I (YGHL1-C3HC4 RING fusion protein).	AK070817	XP_002440385.1 (RefS	Zinc finger, RING-type
Os05g0596700	Similar to cDNA clone:J033067M13, full insert sequence.	AK103507	B7EMM6	Peptidyl-tRNA hydrolase
Os05g0597100	Similar to Nucleolar histone deacetylase HD2-p39.	AK072845	NP_001105631.1 (RefS	Zinc finger, C2H2
Os05g0597200	Similar to AT1 protein.	AK064798	Q40725	High mobility group, HMG-I/HMG-Y
Os05g0597400	Conserved hypothetical protein.	AK375939	NP_001174555.1 (RefS	-
Os05g0597700	Conserved hypothetical protein.	AK121194	A2Y879	-
Os06g0122800	Hypothetical conserved gene.	AK289091	NP_001041816.1 (RefS	Peptidase C48, SUMO/Sentrin/Ubl1
Os06g0131900	Non-protein coding transcript.	EU941912	NONE	-
Os06g0132000	Similar to H0315A08.1 protein.	FP099421	Q01I63	Ribonuclease H1, N-terminal

Os06g0140200	Leucine-rich repeat, plant specific containing protein.	AK287701 ,AK241776 ,AK28787	Q5VPE8	Leucine-rich repeat
Os06g0159750	Hypothetical conserved gene.	EU964122	Q01I89	-
Os06g0159900	Similar to Arm repeat containing protein.	AK063418	GeneMark	U box domain
Os06g0184100	Conserved hypothetical protein.	AK064179	B8B3I7	-
Os06g0188400	Extracellular ligand-binding receptor domain containing protein.	AK065496	B9FRX3	Extracellular ligand-binding receptor
Os06g0188600	Hypothetical conserved gene.	AK242845	Q69KL2	Ionotropic glutamate receptor
Os06g0189600	Protein of unknown function DUF1677, Oryza sativa family protein.	AK109331	B8B3M7	Protein of unknown function DUF1677, Oryza sativa
Os06g0190950	Similar to H0418A01.5 protein.	BT061604	Q01J63	Putative harbinger transposase-derived nuclease
Os06g0198100	Similar to H0315A08.1 protein.	FP099421	Q01I63	Ribonuclease H1, N-terminal
Os06g0200500	Hypothetical conserved gene.	ab initio prediction	Q69K48	-
Os06g0207500	Hypothetical conserved gene.	AK067995	Q69T67	-
Os06g0207600	Hypothetical conserved gene.	AK332154	Q69T66	WRC
Os06g0207866	Conserved hypothetical protein.	BT085445	B9FAZ8	-
Os06g0207950	Hypothetical gene.	CT836248	longestORF	-
Os06g0208100	Similar to Homeodomain protein HOX3.	AB101651	Q69T58	Helix-turn-helix motif, lambda-like repressor
Os06g0208151	Similar to quinone oxidoreductase.	BT040177	B8AEF1	GroES-like
Os06g0208200	Conserved hypothetical protein.	AK242462	NP_001174659.1 (RefS	-
Os06g0208300	KOW domain containing protein.	AK059092	A6N0S0	KOW
Os06g0208700	Similar to Dual-specificity phosphatase protein.	AK065860	Q6DL00	Protein-tyrosine phosphatase, SIW14-like
Os06g0208800	Similar to LysM-domain GPI-anchored protein.	AK062542	Q4VVD8	-
Os06g0208951	Conserved hypothetical protein.	EU943155	NP_001057106.1 (RefS	-
Os06g0209100	Peptidase A1 domain containing protein.	ab initio prediction	NP_001057106.1 (RefS	Peptidase A1
Os06g0209300	Conserved hypothetical protein.	AK065500	Q69T47	-
Os06g0226950	Fatty acid hydroxylase domain containing protein.	AK241878	B6TLG7	Fatty acid hydroxylase
Os06g0240700	Similar to OSIGBa0132O24.3 protein.	EU951279	Q01MB4	-
Os06g0272700	Protein of unknown function DUF231, plant domain containing protein.	BT062756	NP_001174707.1 (RefS	Domain of unknown function DUF231, plant
Os06g0274800	Similar to Peroxidase 11 precursor (EC 1.11.1.7) (Atperox P11) (ATP23a/ATP23b).	AK060295	Q96519	Plant peroxidase
Os06g0274875	Hypothetical protein.	EU942261	longestORF	-
Os06g0275600	Hypothetical conserved gene.	AK101279	F1DKB4	Helix-loop-helix DNA-binding
Os06g0275700	Similar to Beta-catenin repeat family protein.	AK110058	C3SA81	Armadillo
Os06g0276001	Non-protein coding transcript.	AK287734	NONE	-
Os06g0278450	Hypothetical gene.	CT837498	longestORF	-
Os06g0282750	Hypothetical protein.	AK372466	longestORF	-
Os06g0288000	Non-protein coding transcript.	CT835691	NONE	-
Os06g0293000	Hypothetical conserved gene.	CT836321	Q5Z570	-
Os06g0293050	Hypothetical protein.	FP101342	longestORF	-
Os06g0317100	Similar to glycine-rich cell wall structural protein.	CT837813	NP_001151377.1 (RefS	Glycine rich protein
Os06g0318700	Non-protein coding transcript.	AK061908	NONE	-
Os06g0320100	Protein of unknown function DUF773 family protein.	AK101600 ,AK067233	B6T2L5	Protein of unknown function DUF773
Os06g0323550	Similar to H0315A08.1 protein.	FP099421	Q01I63	Ribonuclease H1, N-terminal
Os06g0324800	Major facilitator superfamily protein.	AK109109	E8Z9A7	General substrate transporter
Os06g0326400	Phosphofructokinase domain containing protein.	AK121116 ,AK065052	B6SHF8	Phosphofructokinase domain
Os06g0328900	Cytochrome P450 family protein.	AK110659	C3SAE5	Cytochrome P450
Os06g0328966	Hypothetical protein.	AK357549	longestORF	-
Os06g0329300	Conserved hypothetical protein.	AK067924	A2YCF3	-
Os06g0332500	Conserved hypothetical protein.	AK121262	B8B1C1	-
Os06g0335500	Similar to Auxin-responsive protein IAA21.	AK063854	Q5Z749	AUX/IAA protein
Os06g0336001	Non-protein coding transcript.	AK289086	NONE	-
Os06g0338801	Hypothetical protein.	BT063833	longestORF	-
Os06g0339500	Similar to OSIGBa0137A06.2 protein.	AK105220	Q01N23	-
Os06g0340001	Hypothetical conserved gene.	ab initio prediction	NP_001174769.1 (RefS	-
Os06g0340600	Similar to phosphatidylinositol 3- and 4-kinase family protein.	AK065176	NP_001147061.1 (RefS	Phosphatidylinositol 3-/4-kinase, catalytic
Os06g0341400	Hypothetical protein.	AK110872	longestORF	-
Os06g0341500	Plant lipid transfer protein/seed storage/trypsin-alpha amylase inhibitor domain containing protein.	AK106755	Q5Z9Y7	Plant lipid transfer protein/seed storage/trypsin-alpha amylase inhibitor
Os06g0341600	Conserved hypothetical protein.	AK067335	B9FT43	-
Os06g0345732	Non-protein coding transcript.	AK241165	NONE	-
Os06g0476400	Conserved hypothetical protein.	ab initio prediction	NP_001174788.1 (RefS	-
Os06g0481800	Myb/SANT-like domain domain containing protein.	BT039927	NP_001176912.1 (RefS	Myb/SANT-like domain
Os06g0481850	Hypothetical conserved gene.	AK111398	NP_001176912.1 (RefS	Putative harbinger transposase-derived nuclease
Os06g0513781	Conserved hypothetical protein.	CT833375	B8AJ30	-
Os06g0513862	Conserved hypothetical protein.	CT833375	B8AJ30	-
Os06g0513943	Conserved hypothetical protein.	CT833375	B8AJ30	-
Os06g0514800	Conserved hypothetical protein.	CT833375	B8AJ30	-
Os06g0521300	Similar to Harpin-induced protein 1 containing protein, expressed.	AK358816	Q2RB97	Late embryogenesis abundant protein, LEA-14
Os06g0526700	Probable protein phosphatase 2C 55.	Q652Z7	Q652Z7	Protein phosphatase 2C, manganese/magnesium aspartate binding site
Os06g0539801	Conserved hypothetical protein.	BT064098	A3BCH5	-
Os06g0541600	Conserved hypothetical protein.	AK356929	Q5Z6Z4	-
Os06g0554100	Hypothetical conserved gene.	CT836317	Q5Z9C5	Zinc finger, RING-type
Os06g0557200	Hypothetical protein.	AK063039	longestORF	-
Os06g0566100	RNA recognition motif domain domain containing protein.	AK059225	A3BCV5	RNA recognition motif domain
Os06g0566201	Hypothetical gene.	AK241111	longestORF	-
Os06g0566300	Similar to zinc transporter 4.	AK363919	Q5Z653	Zinc/iron permease
Os06g0567200	Cupredoxin domain containing protein.	AK243375	B6SZ55	Multicopper oxidase, type 1
Os06g0567433	Hypothetical protein.	BT019237	longestORF	-
Os06g0579100	Conserved hypothetical protein.	AK068691	B8ATT0	-
Os06g0580100	Similar to cDNA clone:006-306-H07, full insert sequence.	AK059529	B7E6C5	-
Os06g0582600	Similar to Cysteine proteinase.	AK067453	NP_001151293.1 (RefS	Peptidase, cysteine peptidase active site
Os06g0586000	Conserved hypothetical protein.	AK119252 ,AK063903	A2XQZ9	-
Os06g0612300	Similar to T6J4.5 protein (WIP6 protein).	AK071104	A6N8R9	Zinc finger, C2H2
Os06g0614400	Conserved hypothetical protein.	AK120383	Q5Z7S3	-
Os06g0626050	Similar to potassium transporter 10.	ab initio prediction	NP_001147472.1 (RefS	K+ potassium transporter
Os06g0632400	Similar to OSIGBa0115M15.3 protein.	EU976099	D0VLU6	-
Os06g0632700	MULE transposase, conserved domain domain containing protein.	AK101148	D0VLU5	Phox/Bem1p
Os06g0632800	Hypothetical protein.	tplb0035l23 (Wheat FLcDNA)	longestORF	-
Os06g0633100	Similar to P10Sh249C12.	AK107791	D0VLU4	-
Os06g0633450	Hypothetical conserved gene.	ab initio prediction	Q67VL1	-
Os06g0633500	Zinc finger, RING/FYVE/PHD-type domain containing protein.	AK108505	B6TQ30	Zinc finger, RING-type
Os06g0635700	Conserved hypothetical protein.	AK107643	A3BDV0	-
Os06g0636201	Conserved hypothetical protein.	EU968448	NP_001144438.1 (RefS	-
Os06g0638100	Similar to Tic21.	AK073270	Q09S70	Protein of unknown function DUF3611
Os06g0638200	Uncharacterised protein family UPF0047 domain containing protein.	AK059450	B9FQ34	Uncharacterised protein family UPF0047
Os06g0638500	Tyrosine protein kinase domain containing protein.	AK360940	Q67WE5	Protein kinase, catalytic domain
Os06g0639250	Conserved hypothetical protein.	ab initio prediction	Q67WD8	-
Os06g0639550	Non-protein coding transcript.	AK099542	NONE	-
Os06g0645700	Similar to HAF1.	AK108404	Q67W65	Bromodomain
Os06g0645901	Similar to Transcription initiation factor TFIID subunit 1.	BT037416	Q67W65	-
Os06g0649550	Non-protein coding transcript.	BT086930	NONE	-
Os06g0652501	Non-protein coding transcript.	X06283	NONE	-
Os06g0656566	Hypothetical gene.	BT062457	longestORF	-
Os06g0656800	FAS1 domain domain containing protein.	AK109762	NP_001150349.1 (RefS	FAS1 domain
Os06g0662000	Similar to Vacuolar ATP synthase catalytic subunit A.	AK071775	B6UH55	ATPase, F1/V1/A1 complex, alpha/beta subunit, N-terminal
Os06g0665100	Similar to Dreg-2 like protein.	AK101330	NP_199286.1 (RefSeq)	Haloacid dehalogenase-like hydrolase
Os06g0667100	Similar to Nuclear Y/CCAAT-box binding factor C subunit NF-YC.	AK105079 ,AK066630	NP_001149626.1 (RefS	Transcription factor CBF/NF-Y/archaeal histone
Os06g0667300	Non-protein coding transcript.	AK102272	NONE	-
Os06g0667400	Conserved hypothetical protein.	AK065424	Q655T7	-
Os06g0667600	Similar to Glycine decarboxylase complex H-protein.	AK071621 ,AK058606	NP_001152670.1 (RefS	Glycine cleavage H-protein
Os06g0667900	Disease resistance protein domain containing protein.	ab initio prediction	Q655T3	Disease resistance protein
Os06g0668000	Hypothetical protein.	AK064023	longestORF	-
Os06g0668200	Similar to Phosphoglycerate kinase, cytosolic (EC 2.7.2.3).	AK070705 ,AK101622	A2YG06	Phosphoglycerate kinase
Os06g0668250	Hypothetical gene.	BT088042	longestORF	-
Os06g0668400	Kelch related domain containing protein.	AK108036	B9FQE4	BTB/POZ-like
Os06g0669275	Similar to MEE44 (maternal effect embryo arrest 44)%3B nucleotidyltransferase.	BT018457	NP_191917.2 (RefSeq)	PAP/25A-associated
Os06g0669700	Similar to DNA binding protein.	AK070871	NP_001149442.1 (RefS	Transcription regulator HTH, Myb-type, DNA-binding
Os06g0669800	Ovarian tumour, otubain domain containing protein.	AK073551	B6U3W2	Ovarian tumour, otubain
Os06g0670000	Similar to Molybdenum cofactor sulfurase.	AK287475	Q655R6	Aminotransferase, class V/Cysteine desulfurase

Os06g0670633	Similar to embryo-sac basal-endosperm layer embryo-surrounding-region.	AJ437283	NP_001105581.1 (RefS	Glucoamylase, putatuve
Os06g0671000	Similar to Potassium transporter 1 (AtPOT1) (AtKUP1) (AtKT1).	AK111663	B6U7Z6	K+ potassium transporter
Os06g0671075	Hypothetical gene.	EU967027	longestORF	-
Os06g0671600	Beta tubulin, autoregulation binding site domain containing protein.	AK111011	B8B176	-
Os06g0671700	Similar to toprim domain-containing protein.	AK108468	NP_849735.1 (RefSeq)	DNA helicase, DnaB-like, C-terminal
Os06g0673500	Similar to polyubiquitin containing 7 ubiquitin monomers.	AK243474	NP_001147027.1 (RefS	Ubiquitin
Os06g0678700	Hypothetical gene.	AK107560	Q655H6	-
Os06g0683925	Hypothetical protein.	tplb0033d10 (Wheat FLcDNA)	longestORF	-
Os06g0690400	Non-protein coding transcript.	EU941368	NONE	-
Os06g0691900	Similar to F-box domain containing protein.	EU962397	NP_001149196.1 (RefS	F-box domain, cyclin-like
Os06g0692050	Conserved hypothetical protein.	FP100342	Q5Z672	-
Os06g0698812	Disease resistance protein domain containing protein.	ab initio prediction	XP_002438943.1 (RefS	Disease resistance protein
Os06g0703900	Homeodomain-like containing protein.	AK067950	B8B262	Myb-like DNA-binding domain, SHAQKYF class
Os06g0705300	Similar to plant-specific domain TIGR01589 family protein.	AK064803	NP_001149236.1 (RefS	Conserved hypothetical protein CHP01589, plant
Os06g0705350	Similar to pentatricopeptide (PPR) repeat-containing protein.	BT055332	NP_199702.1 (RefSeq)	Pentatricopeptide repeat
Os06g0705400	Hypothetical conserved gene.	AK062493	C0KHK5	Plant lipid transfer protein/hydrophobic protein, helical domain
Os06g0705651	Hypothetical protein.	EU955776	longestORF	-
Os06g0705700	TGF-beta receptor, type I/II extracellular region family protein.	AK119527	A2YGT5	Oligopeptide transporter
Os06g0705901	Hypothetical protein.	tplb0058e01 (Wheat FLcDNA)	longestORF	-
Os06g0707800	Non-protein coding transcript.	AK111291	NONE	-
Os06g0708300	Similar to RER1A protein.	AK243324	NP_001151498.1 (RefS	Retrieval of early ER protein Rer1
Os06g0708600	Zinc finger, C2H2-like domain containing protein.	AK100915	E4MXX2	Zinc finger, C2H2-like
Os06g0708700	Similar to Nodulin-like protein.	AK067151	B6SKJ2	Drug/metabolite transporter
Os06g0708832	Similar to arogenate dehydrogenase.	AK355541	NP_001147429.1 (RefS	Prephenate dehydrogenase
Os06g0710001	Hypothetical conserved gene.	AK249657	Q5Z9G7	-
Os06g0710300	Uncharacterised conserved protein UCP022348 domain containing protein.	AK121344	B6U4B2	Uncharacterised protein family UPF0114
Os06g0710401	Non-protein coding transcript.	AK330826	NONE	-
Os06g0710850	Non-protein coding transcript.	EU950496	NONE	-
Os06g0710900	Conserved hypothetical protein.	AK073326	A2YGW4	-
Os06g0711800	Pectinesterase inhibitor domain containing protein.	AK069642	NP_001167668.1 (RefS	Pectinesterase inhibitor
Os06g0712250	Hypothetical conserved gene.	CT832454	NP_001144189.1 (RefS	-
Os06g0712300	EF-Hand type domain containing protein.	AK070744	E6NU22	Phospholipid/glycerol acyltransferase
Os06g0712400	Protein of unknown function DUF544 family protein.	AK067573	B8B2H1	Ubiquitin interacting motif
Os06g0712550	Hypothetical protein.	tplb0056b02 (Wheat FLcDNA)	longestORF	-
Os06g0712600	Similar to SHI.	EU959412	NP_001150905.1 (RefS	Zinc finger, lateral root primordium type 1
Os06g0712900	tRNA-dihydrouridine synthase domain containing protein.	AK106648	NP_201523.1 (RefSeq)	tRNA-dihydrouridine synthase
Os06g0713400	Cyclin-like F-box domain containing protein.	AK100628	B9FQV7	F-box domain, cyclin-like
Os06g0714400	Conserved hypothetical protein.	AK108610	Q5NAL1	-
Os06g0714432	Non-protein coding transcript.	CT836310	NONE	-
Os06g0714600	ADP-ribosylation factor domain containing protein.	AK318603	A6MD16	Small GTPase superfamily
Os06g0714700	Conserved hypothetical protein.	CU406678	NP_001058577.1 (RefS	-
Os06g0714800	Protein of unknown function DUF581 family protein.	AK059793 ,AK071528	Q5Z9Q5	Protein of unknown function DUF581
Os06g0716050	Hypothetical gene.	EU948676	longestORF	-
Os06g0718400	Hypothetical conserved gene.	CT836320	Q8SB26	Blue
Os06g0719600	Domain of unknown function DUF1618 domain containing protein.	AK099272	Q8SB18	Domain of unknown function DUF1618
Os06g0719900	Similar to endo/excinuclease amino terminal domain-containing protein.	CT834222	XP_002881103.1 (RefS	-
Os06g0721600	Hypothetical conserved gene.	ab initio prediction	NP_001058606.2 (RefS	Protein of unknown function DUF544
Os06g0721800	Cupredoxin domain containing protein.	AK243644	A2YH12	Blue
Os06g0722100	Hypothetical conserved gene.	AK068839	NP_001058607.1 (RefS	Domain of unknown function DUF1618
Os06g0723400	Hypothetical conserved gene.	ab initio prediction	Q5YLZ3	Protein of unknown function DUF544
Os06g0725350	Hypothetical gene.	tplb0017d24 (Wheat FLcDNA)	longestORF	-
Os06g0725400	Similar to BLE1 protein.	AK241158	Q8S937	-
Os06g0726200	Similar to Chitinase 1.	AK061042 ,AK099339 ,AK10402	Q42993	Glycoside hydrolase, family 19, catalytic
Os06g0726501	Non-protein coding transcript.	tplb0001a06 (Wheat FLcDNA)	NONE	-
Os06g0727000	Zinc finger, C2H2-type domain containing protein.	AK108097	A2YH63	Zinc finger, C2H2
Os06g0727400	Similar to Protein kinase APK1A, chloroplast precursor (EC 2.7.1.-).	AK069558	NP_001147720.1 (RefS	Protein kinase, catalytic domain
Os06g0727500	Hypothetical protein.	tplb0023d22 (Wheat FLcDNA)	longestORF	-
Os06g0728902	Similar to predicted protein.	AK240761	NP_001154712.1 (RefS	Protein of unknown function DUF605
Os06g0729300	Similar to Protein argonaute 1D.	AK111833	Q5Z5B2	Stem cell self-renewal protein Piwi
Os06g0729350	Non-protein coding transcript.	BT016774	NONE	-
Os06g0729650	Similar to Photosystem II stability/assembly factor HCF136, chloroplastic.	FP096975	B9FR06	Twin-arginine translocation pathway, signal sequence
Os06g0729800	Hypothetical gene.	AK241958	longestORF	-
Os06g0730000	Similar to Serine carboxypeptidase II-like protein.	AK242427	NP_001152245.1 (RefS	Peptidase S10, serine carboxypeptidase
Os06g0731000	Conserved hypothetical protein.	ab initio prediction	NP_001175010.1 (RefS	-
Os06g0731200	Similar to ABC transporter-like protein.	AK071489	XP_002303777.1 (RefS	ABC transporter-like
Os06g0731300	Surface protein from Gram-positive cocci, anchor region domain containing protein.	AK358759	Q5Z408	Heat shock protein DnaJ, cysteine-rich domain
Os06g0731400	Hypothetical conserved gene.	AK120175	B8B2Y0	-
Os06g0731500	Conserved hypothetical protein.	AK062465	Q5Z405	-
Os06g0731750	Hypothetical gene.	BT016392	longestORF	-
Os06g0731800	Clathrin light chain family protein.	AK069288	Q259D4	Clathrin light chain
Os06g0732000	Similar to 60S ribosomal protein L35.	FP092387	NP_001148517.1 (RefS	-
Os07g0116050	Transposase, IS4-like domain containing protein.	BT037367	XP_002449014.1 (RefS	Transposase, IS4-like
Os07g0117600	Conserved hypothetical protein.	ab initio prediction	Q8H5J6	-
Os07g0132800	Hypothetical gene.	AK240792	longestORF	-
Os07g0144900	Similar to ASK11 (ARABIDOPSIS SKP1-LIKE 11)%3B ubiquitin-protein ligase.	AK242552	NP_567959.1 (RefSeq)	SKP1 component
Os07g0146800	Non-protein coding transcript.	AK106695	NONE	-
Os07g0147500	Similar to Photosystem II 10 kDa polypeptide, chloroplast precursor.	AK062172 ,AK120691	Q40070	Photosystem II PsbR
Os07g0147550	Similar to Photosystem II 10 kDa polypeptide, chloroplast.	CT837962	Q40070	Photosystem II PsbR
Os07g0147700	ATPase, BadF/BadG/BcrA/BcrD type domain containing protein.	AK242508	NP_001152135.1 (RefS	ATPase, BadF/BadG/BcrA/BcrD type
Os07g0148800	Conserved hypothetical protein.	AK062246	Q69VA2	-
Os07g0148900	Photosystem I protein-like protein.	AK058788	P36886	Photosystem I PsaG/PsaK protein
Os07g0151900	Conserved hypothetical protein.	AK240814	NP_001175061.1 (RefS	-
Os07g0152800	Similar to Glycine-rich protein.	AK065458	B6TIW3	-
Os07g0152900	Similar to Glycolate oxidase (EC 1.1.3.15) (Fragment).	AK120929	Q6YT73	FMN-dependent dehydrogenase
Os07g0152950	Hypothetical gene.	BT066752	longestORF	-
Os07g0153000	Tify domain containing protein.	AK374356	NP_001058910.2 (RefS	Tify
Os07g0153300	Protein of unknown function DUF1365 family protein.	AK066146	B9FVJ7	Protein of unknown function DUF1365
Os07g0153400	Kelch-type beta propeller domain containing protein.	AK069618	XP_002893599.1 (RefS	F-box domain, cyclin-like
Os07g0153600	Similar to Prostrate growth 1.	FJ155665	D9ZKN9	Zinc finger, C2H2
Os07g0154201	Hypothetical gene.	BT085311	longestORF	-
Os07g0154300	Conserved hypothetical protein.	AK105386 ,AK122093	A2YIA2	-
Os07g0154400	Armadillo-type fold domain containing protein.	AK288457	B9FVJ8	Armadillo-type fold
Os07g0154800	Conserved hypothetical protein.	AK111389	B8B7D0	-
Os07g0156200	Haem peroxidase, plant/fungal/bacterial family protein.	AK249509	NP_001152255.1 (RefS	Plant peroxidase
Os07g0159800	MD-2-related lipid-recognition domain containing protein.	AK060475	B6TM82	MD-2-related lipid-recognition
Os07g0159900	Thioredoxin fold domain containing protein.	AK062586	A2YID0	Glutaredoxin
Os07g0161000	Tetratricopeptide-like helical domain containing protein.	AK065877	B8B7F4	Tetratricopeptide TPR-1
Os07g0162200	Protein of unknown function DUF593 domain containing protein.	tplb0058k18 (Wheat FLcDNA)	Q8H538	Protein of unknown function DUF593
Os07g0162300	Similar to RIO kinase.	AK066456	B9FVM0	RIO kinase
Os07g0168000	Similar to Polyribonucleotide phophorylase (Fragment).	AK065622	O24288	Exoribonuclease, phosphorolytic domain 1
Os07g0168300	Similar to Glutathione S-transferase GSTU6.	AK061436 ,AK099137	A2YIJ4	Glutathione S-transferase, N-terminal
Os07g0168800	Zinc finger, AN1-type domain containing protein.	AK121813	Q7Y1W9	Zinc finger, AN1-type
Os07g0168900	Hypothetical gene.	AK062924	longestORF	-
Os07g0169600	2OG-Fe(II) oxygenase domain containing protein.	AK121818 ,AK060318	NP_001152182.1 (RefS	Oxoglutarate/iron-dependent oxygenase
Os07g0169700	GA 20-oxidase3, GA metabolism	Inferred from literature	Q69LD8	-
Os07g0170000	Similar to Brn1-like protein.	EU972358	NP_193054.1 (RefSeq)	Short-chain dehydrogenase/reductase SDR
Os07g0170200	Similar to predicted protein.	CT836091	NP_179964.2 (RefSeq)	HNH endonuclease
Os07g0170300	YT521-B-like protein family protein.	AK072392	B6U208	YTH domain
Os07g0170400	Non-protein coding transcript.	BT016473	NONE	-
Os07g0170500	Conserved hypothetical protein.	AK240864	NP_001175070.1 (RefS	-
Os07g0171200	Similar to Galactose-1-phosphate uridyl transferase-like protein.	AK071075	B6TFN9	Galactose-1-phosphate uridyl transferase, class I
Os07g0171350	Non-protein coding transcript.	BT084747	NONE	-
Os07g0172200	Similar to ROOT HAIRLESS 1.	AK103352	B6UAF0	-
Os07g0172900	Hypothetical conserved gene.	AK067304	Q6ZA56	-
Os07g0173100	HSP20-like chaperone domain containing protein.	CT836094	NP_001059002.1 (RefS	-

Os07g0173300	Conserved hypothetical protein.	AK110935	A3BH18	-
Os07g0175200	MTCP1 domain containing protein.	AK058437	A2YIN2	MTCP1
Os07g0175801	Non-protein coding transcript.	X02683	NONE	-
Os07g0176200	Similar to Transcription factor CPP.	AK062003	B3IX45	Tesmin/TSO1-like, CXC
Os07g0176400	Hypothetical protein.	BT085651	longestORF	-
Os07g0176401	Hypothetical protein.	tplb0061g22 (Wheat FLcDNA)	longestORF	-
Os07g0176500	Leucine-rich repeat, plant specific containing protein.	AK062887	A2YIP1	Leucine-rich repeat
Os07g0176600	Similar to Kinase-like protein.	AK067073 ,AK066812	Q6ZEZ5	Protein kinase, catalytic domain
Os07g0176900	Similar to ribose-5-phosphate isomerase.	AK067117	Q8RU73	Ribose 5-phosphate isomerase, type A
Os07g0177100	Armadillo-type fold domain containing protein.	AK070264	B9FVT4	Armadillo-type fold
Os07g0177150	Non-protein coding transcript.	AK288093	NONE	-
Os07g0177200	Similar to glutamate binding protein.	AK070925	XP_002878509.1 (RefS	Inhibitor of apoptosis-promoting Bax1-related
Os07g0177300	Uncharacterised protein family UPF0005 domain containing protein.	AK073781	B6T7B7	Inhibitor of apoptosis-promoting Bax1-related
Os07g0178950	Hypothetical protein.	tplb0047n14 (Wheat FLcDNA)	longestORF	-
Os07g0179000	Pentatricopeptide repeat domain containing protein.	AK100008	A2YIR0	Pentatricopeptide repeat
Os07g0190900	Forkhead-associated domain containing protein.	AK073533	A3BHE5	Forkhead-associated
Os07g0191200	Plasma membrane H+ ATPase (EC 3.6.3.6).	AK121402	Q43002	ATPase, P-type, H+ transporting proton pump
Os07g0191250	Hypothetical protein.	tplb0039e05 (Wheat FLcDNA)	longestORF	-
Os07g0191600	Protein of unknown function DUF569 domain containing protein.	AK102563	B8B850	Protein of unknown function DUF569
Os07g0191625	Hypothetical protein.	AK361241	longestORF	-
Os07g0191801	Non-protein coding transcript.	AK288998	NONE	-
Os07g0192000	ATPase, AAA-type, core domain containing protein.	AK065505	B6SVY2	ATPase, AAA+ type, core
Os07g0192400	Non-protein coding transcript.	AK120724	NONE	-
Os07g0192800	Similar to ATPase 3.	AK288139	NP_001148126.1 (RefS	ATPase, AAA+ type, core
Os07g0193100	Non-protein coding transcript.	EU949559	NONE	-
Os07g0193200	Ankyrin repeat containing protein.	ab initio prediction	NP_001059105.1 (RefS	Ankyrin repeat
Os07g0193701	Hypothetical protein.	BT086982	longestORF	-
Os07g0193800	Similar to BHLH transcription factor (Fragment).	AK242645	F1DK89	Helix-loop-helix DNA-binding
Os07g0194300	Hypothetical protein.	AK062637	GeneMark	-
Os07g0194400	Conserved hypothetical protein.	AB332080	NP_001175091.1 (RefS	-
Os07g0194500	2OG-Fe(II) oxygenase domain containing protein.	AK121816	XP_002324024.1 (RefS	Metridin-like ShK toxin
Os07g0194550	Non-protein coding transcript.	BT084776	NONE	-
Os07g0210800	Hypothetical conserved gene.	AK106587	Q60DP5	Myb/SANT-like domain
Os07g0211800	Protein of unknown function DUF1749 family protein.	AK065959	B7EB72	Protein of unknown function DUF1749
Os07g0212300	Similar to Nudix hydrolase 16, mitochondrial precursor (EC 3.6.1.-) (AtNUDT16).	AK107375	B6SQG1	NUDIX hydrolase domain
Os07g0213400	Conserved hypothetical protein.	AK062273	B9FW62	-
Os07g0213500	Hypothetical conserved gene.	AK070126	B6SL19	-
Os07g0213600	Bifunctional inhibitor/plant lipid transfer protein/seed storage domain containing protein.	AK107696	A2YJF5	Plant lipid transfer protein/seed storage/trypsin-alpha amylase inhibitor
Os07g0213800	Similar to Allergenic protein.	AK107215	Q01882	Cereal allergen/alpha-amylase inhibitor, rice-type
Os07g0214100	Seed allergenic protein RA17 precursor.	AK242324	Q01883	Cereal allergen/alpha-amylase inhibitor, rice-type
Os07g0214600	Similar to Seed allergenic protein RA17 precursor.	AK242298	Q40654	Cereal allergen/alpha-amylase inhibitor, rice-type
Os07g0214900	Similar to Chalcone synthase C2 (EC 2.3.1.74) (Naringenin-chalcone synthase C2).	FP093770	Q8H4L3	Chalcone/stilbene synthase, N-terminal
Os07g0215050	Hypothetical protein.	BT063109	longestORF	-
Os07g0215500	Allergenic protein.	AK242333	Q01881	Cereal allergen/alpha-amylase inhibitor, rice-type
Os07g0218700	Cytochrome P450 family protein.	AK108382	Q01LP1	Cytochrome P450
Os07g0218766	Hypothetical protein.	tplb0041k22 (Wheat FLcDNA)	longestORF	-
Os07g0220466	Similar to H0315A08.1 protein.	FP099421	Q01I63	Ribonuclease H1, N-terminal
Os07g0223733	Non-protein coding transcript.	BT016899	NONE	-
Os07g0301050	Non-protein coding transcript.	BT068036	NONE	-
Os07g0468900	Hypothetical gene.	AK109042	longestORF	-
Os07g0472900	Conserved hypothetical protein.	ab initio prediction	NP_001059615.1 (RefS	-
Os07g0487198	Hypothetical protein.	tplb0040j01 (Wheat FLcDNA)	longestORF	-
Os07g0487300	Protein kinase, catalytic domain domain containing protein.	ab initio prediction	Q84ZD7	Protein kinase, catalytic domain
Os07g0488215	Hypothetical protein.	tplb0040j01 (Wheat FLcDNA)	longestORF	-
Os07g0488400	Protein kinase, catalytic domain domain containing protein.	ab initio prediction	Q84ZD7	Protein kinase, catalytic domain
Os07g0523400	Glucose-6-phosphate/phosphate-translocator precursor.	AK070124	NP_001147439.1 (RefS	Drug/metabolite transporter
Os07g0536800	Hypothetical conserved gene.	AK109751	Q53NH3	-
Os07g0555200	Similar to predicted protein.	AK062438	D4N2J6	MIF4G-like, type 3
Os07g0562900	Hypothetical conserved gene.	CT836099	B9FXX6	-
Os07g0567700	Similar to Scarecrow-like 23 (Fragment).	AK059008	Q6ULS4	Transcription factor GRAS
Os07g0569600	Chaperonin-like RbcX domain containing protein.	AK067599	A3BLB7	Chaperonin-like RbcX
Os07g0571200	Hypothetical protein.	BT067768	longestORF	-
Os07g0574150	Hypothetical protein.	EU951884	longestORF	-
Os07g0575000	Pathogenesis-related transcriptional factor/ERF, DNA-binding domain containing protein.	AK374106	Q6ZL38	Pathogenesis-related transcriptional factor/ERF, DNA-binding
Os07g0575733	Conserved hypothetical protein.	ab initio prediction	NP_001053398.1 (RefS	-
Os07g0575900	Protein of unknown function DUF946, plant family protein.	AK105640	B9FY25	Vacuolar protein sorting-associated protein 62
Os07g0585600	Similar to N-acetyl-glutamate synthase.	AK240739	C4NFG1	GCN5-related N-acetyltransferase
Os07g0585700	Zinc finger protein, Pre-harvest sprouting resistance	AB510199	Q8H5K8	-
Os07g0585900	Hypothetical conserved gene.	FP093392	Q8GRW3	-
Os07g0586000	Similar to ZCW7.	AK069212	B6TC50	-
Os07g0586266	Hypothetical protein.	EU949155	longestORF	-
Os07g0586600	Conserved hypothetical protein.	AK287951	NP_001175279.1 (RefS	-
Os07g0586700	Similar to HRT transcription factor (Fragment).	AK102792	F1DJZ9	-
Os07g0591202	Hypothetical gene.	BT017174	longestORF	-
Os07g0599500	Hypothetical protein.	AK068696 ,AK106010	GeneMark	-
Os07g0599600	Hypothetical protein.	AK105918 ,AK070771 ,AK10621	GeneMark	-
Os07g0600000	Conserved hypothetical protein.	AK105907	A3BLW3	-
Os07g0633450	Hypothetical gene.	EU947431	longestORF	-
Os07g0639400	Similar to Peroxidase 1.	AK110555	A3BMN4	Plant peroxidase
Os07g0669200	Similar to GTP1/OBG family protein.	BT063209	NP_197358.2 (RefSeq)	GTP-binding domain, HSR1-related
Os07g0669250	Similar to predicted protein.	EU950976	XP_002873904.1 (RefS	GTP-binding protein GTP1/OBG, C-terminal
Os07g0669401	Conserved hypothetical protein.	FP091568	NP_001042416.1 (RefS	-
Os07g0669500	Similar to Branched silkless1.	AK105365	A1KZE8	Pathogenesis-related transcriptional factor/ERF, DNA-binding
Os07g0669600	Conserved hypothetical protein.	AK066595	B9FUS7	-
Os07g0669900	Non-protein coding transcript.	AK062892	NONE	-
Os07g0670000	Pentatricopeptide repeat domain containing protein.	AK109518	A2YPS5	Pentatricopeptide repeat
Os07g0670100	Hypothetical conserved gene.	CT836088	Q8GSF2	-
Os07g0670800	Similar to 60S ribosomal protein L19-3.	AK241287	NP_001148813.1 (RefS	-
Os07g0671000	Similar to FPF1.	AK358497	NP_001151726.1 (RefS	-
Os07g0671200	Pentatricopeptide repeat domain containing protein.	EU971084	NP_001060597.1 (RefS	Pentatricopeptide repeat
Os07g0671301	Hypothetical gene.	EU973702	longestORF	-
Os07g0671400	Heavy metal transport/detoxification protein domain containing protein.	AK067933	NP_001149888.1 (RefS	Heavy metal-associated domain, HMA
Os07g0674100	Hypothetical conserved gene.	AK099614	B9FUU7	NAD-dependent epimerase/dehydratase
Os07g0674150	Hypothetical protein.	tplb0036f05 (Wheat FLcDNA)	longestORF	-
Os07g0674200	Similar to 60S ribosomal protein L22-2.	AK058245	B6U998	Ribosomal protein L22e
Os07g0674300	Protein kinase, ATP binding site domain containing protein.	AK110511	A2YPV3	Pentatricopeptide repeat
Os07g0674500	Pollen Ole e 1 allergen/extensin domain containing protein.	AK107802	A2YPV5	Pollen Ole e 1 allergen/extensin
Os07g0674800	Similar to AP2 domain transcription factor EREBP.	AK288040	Q7Y047	Pathogenesis-related transcriptional factor/ERF, DNA-binding
Os07g0675166	Non-protein coding transcript.	AK110786	NONE	-
Os07g0675200	Conserved hypothetical protein.	AK063620	B8B5V2	-
Os07g0676200	Non-protein coding transcript.	AK061503	NONE	-
Os07g0677600	Similar to Cationic peroxidase.	AK060984	NP_001147443.1 (RefS	Plant peroxidase
Os07g0677900	Protein of unknown function DUF827, plant family protein.	AK103884 ,AK106293	A2YPX8	Protein of unknown function DUF827, plant
Os07g0687200	Similar to CAM7 (CALMODULIN 7)%3B calcium ion binding.	AK104031	NP_850344.1 (RefSeq)	Calcium-binding EF-hand
Os07g0692500	Conserved hypothetical protein.	AK108769	Q7XI64	-
Os07g0692900	Similar to Ubiquitin-activating enzyme E1.	AK074007	B8B6A7	Ubiquitin/SUMO-activating enzyme E1
Os07g0692950	Hypothetical protein.	tplb0053b11 (Wheat FLcDNA)	longestORF	-
Os07g0693100	Similar to Pyruvate decarboxylase isozyme 3 (EC 4.1.1.1) (PDC).	AK100433	Q0D3D2	Thiamine pyrophosphate enzyme, C-terminal TPP-binding
Os08g0117800	Cation/H+ exchanger domain containing protein.	AK100696	A2YQP4	Cation/H+ exchanger
Os08g0118500	Similar to Auxin-induced SAUR-like protein.	CT835918	NP_001148413.1 (RefS	Auxin responsive SAUR protein
Os08g0118700	Non-protein coding transcript.	X06283	NONE	-
Os08g0118800	Similar to SAUR31 - auxin-responsive SAUR family member.	ab initio prediction	NP_001148413.1 (RefS	Auxin responsive SAUR protein
Os08g0119100	Protein of unknown function DUF1517 domain containing protein.	AK353591	Q6ZJ46	Protein of unknown function DUF1517
Os08g0119500	Methyltransferase type 11 domain containing protein.	AK242531	NP_680738.1 (RefSeq)	Methyltransferase type 11
Os08g0122900	Conserved hypothetical protein.	AK288623	Q6YX29	-

Os08g0127300	Conserved hypothetical protein.	AK058548	Q6ZK51	-
Os08g0127800	Conserved hypothetical protein.	AK103321 ,AK108548	Q6ZK47	-
Os08g0132500	Conserved hypothetical protein.	AK363691	Q69R49	-
Os08g0133200	Similar to copine-3.	AK241367	NP_001147038.1 (RefS	Copine
Os08g0140600	Conserved hypothetical protein.	ab initio prediction	Q6YZ17	-
Os08g0141000	Conserved hypothetical protein.	EU960934	NP_001060971.2 (RefS	-
Os08g0141300	PapD-like domain containing protein.	AK058337 ,AK067917	B6TP74	Major sperm protein
Os08g0142500	Pectinesterase inhibitor domain containing protein.	AK242564	NP_001175375.1 (RefS	Pectinesterase inhibitor
Os08g0143150	Similar to retrotransposon protein.	EU953494	NP_001158937.1 (RefS	-
Os08g0143400	Amine oxidase domain containing protein.	AK120532	NP_187981.1 (RefSeq)	Amine oxidase
Os08g0143500	Glycosyl transferase, family 14 protein.	AK071122	NP_001147601.1 (RefS	Glycosyl transferase, family 14
Os08g0144000	Myb, DNA-binding domain containing protein.	AK073681	B4FLJ6	SANT domain, DNA binding
Os08g0144400	Hypothetical conserved gene.	AK108707	B9FZ15	-
Os08g0145501	Conserved hypothetical protein.	AK241170	B9FSE2	-
Os08g0146001	Hypothetical conserved gene.	ab initio prediction	B9FZ18	-
Os08g0148300	Conserved hypothetical protein.	AK105581	A3BPL9	-
Os08g0166750	Hypothetical protein.	AK372466	longestORF	-
Os08g0172433	Non-protein coding transcript.	EU941912	NONE	-
Os08g0176200	Serine/threonine protein kinase-related domain containing protein.	AK241001	Q5KS46	Protein kinase, catalytic domain
Os08g0182400	Conserved hypothetical protein.	AK059491	NP_001061148.1 (RefS	-
Os08g0216450	Conserved hypothetical protein.	EU951430	Q65WW4	-
Os08g0283000	Similar to H0315A08.1 protein.	FP099421	Q01I63	Ribonuclease H1, N-terminal
Os08g0283300	Similar to H0315A08.1 protein.	FP099421	Q01I63	Ribonuclease H1, N-terminal
Os08g0299200	Adenylate cyclase domain containing protein.	AK241974	B6TQK5	CYTH domain
Os08g0301551	Non-protein coding transcript.	AJ238715	NONE	-
Os08g0323266	Similar to Signal recognition particle 68 kDa protein.	ab initio prediction	B6U4F2	-
Os08g0323800	Conserved hypothetical protein.	AK108337	Q6Z0E2	-
Os08g0328050	Hypothetical protein.	EU945299	longestORF	-
Os08g0334900	Xyloglucan fucosyltransferase family protein.	AK060562	A2YU28	Xyloglucan fucosyltransferase
Os08g0347500	Similar to PsbP family protein, expressed.	ab initio prediction	Q109K8	Photosystem II PsbP, oxygen evolving complex
Os08g0393150	Similar to OSIGBa0110B10.2 protein.	EU973161	Q01KQ6	Zinc finger, BED-type predicted
Os08g0401200	Non-protein coding transcript.	BT069163	NONE	-
Os08g0404350	Similar to FACT complex subunit SPT16.	ab initio prediction	Q7X923	-
Os08g0411900	Conserved hypothetical protein.	AK243392	B9G0V4	-
Os08g0422200	Cation efflux protein family protein.	AY305863	Q8H329	Cation efflux protein
Os08g0433000	Similar to Arginine decarboxylase.	ab initio prediction	A3B825	Arginine decarboxylase
Os08g0433050	Similar to Arginine decarboxylase.	EU944353	A3B825	-
Os08g0433400	Hypothetical conserved gene.	AK059468	A2YVI0	SANT domain, DNA binding
Os08g0433600	Protein of unknown function DUF962 family protein.	AK067255	NP_001151002.1 (RefS	Protein of unknown function DUF962
Os08g0434300	Similar to Malate dehydrogenase precursor (EC 1.1.1.37).	AK058477	A2YVI5	Lactate/malate dehydrogenase, N-terminal
Os08g0434500	Hypothetical protein.	ab initio prediction	NONE	-
Os08g0434700	Homeodomain-like containing protein.	AK070338	B8BB14	Myb-like DNA-binding domain, SHAQKYF class
Os08g0436700	Similar to NAC transcription factor.	AK073013	A2YVJ8	No apical meristem
Os08g0437566	Similar to H0315A08.1 protein.	FP099421	Q01I63	Ribonuclease H1, N-terminal
Os08g0437832	Non-protein coding transcript.	EU941912	NONE	-
Os08g0438801	Hypothetical protein.	AK287665	longestORF	-
Os08g0439000	Similar to pyrophosphate--fructose 6-phosphate 1-phosphotransferase.	AK287794	NP_001147085.1 (RefS	Phosphofructokinase domain
Os08g0439200	Non-protein coding transcript.	AK059480	NONE	-
Os08g0439600	Protein of unknown function DUF868, plant family protein.	BT070111	NP_001061898.1 (RefS	Protein of unknown function DUF868, plant
Os08g0440100	Similar to Temperature stress-induced lipocalin.	AK068551	Q38JE5	Lipocalin/cytosolic fatty-acid binding protein domain
Os08g0440800	Glyceraldehyde-3-phosphate dehydrogenase.	AK071520	Q8S4Y9	Aldehyde dehydrogenase domain
Os08g0440850	Hypothetical gene.	EU945888	longestORF	-
Os08g0441100	Similar to PnC401 homologue.	AK108225	Q6Z9F4	Protein kinase, catalytic domain
Os08g0441300	Hypothetical conserved gene.	ab initio prediction	Q6Z9F3	Proteinase inhibitor I13, potato inhibitor I
Os08g0441500	Similar to cinnamoyl CoA reductase.	AK102061	XP_002299260.1 (RefS	NAD-dependent epimerase/dehydratase
Os08g0441600	Similar to Chorismate mutase CM2 (EC 5.4.99.5) (Chorimate mutase).	AK069725	B4FUP5	Chorismate mutase, AroQ class, eukaryotic type
Os08g0442600	Hypothetical protein.	AK105576	longestORF	-
Os08g0442900	Hypothetical protein.	AK110520	GeneMark	-
Os08g0443800	Tetraspanin domain containing protein.	AK110630	NP_001105285.1 (RefS	Tetraspanin
Os08g0444100	Similar to Splicing factor 3b, subunit 5 (Splicing factor 3B subunit 10).	AK073746	E5GC24	Splicing factor 3B subunit 5/RDS3 complex subunit 10
Os08g0444440	Hypothetical conserved gene.	AK241238	B9G163	-
Os08g0444500	Conserved hypothetical protein.	CT835928	NP_001061923.1 (RefS	-
Os08g0445000	Similar to RING-H2 finger protein ATL5A.	AK106866	B6T239	Zinc finger, RING-type
Os08g0445250	Hypothetical protein.	tplb0042b14 (Wheat FLcDNA)	longestORF	-
Os08g0445700	HAD-superfamily hydrolase subfamily IIB protein.	AK072132	B6T0F0	Glycosyl transferase, family 20
Os08g0446200	Similar to Receptor-like protein kinase precursor (EC 2.7.1.37). Splice isoform INRPK1a.	AK120424	XP_002445581.1 (RefS	Protein kinase, catalytic domain
Os08g0446250	Hypothetical gene.	BT068372	longestORF	-
Os08g0446350	Non-protein coding transcript.	BT068372	NONE	-
Os08g0446750	Hypothetical protein.	EU976033	longestORF	-
Os08g0446800	Similar to GDU1.	AK243202	NP_001151217.1 (RefS	-
Os08g0447000	Similar to D-3-phosphoglycerate dehydrogenase.	AK243399	B6SKK1	Amino acid-binding ACT
Os08g0447500	Hypothetical conserved gene.	AK240682	A3BTN1	-
Os08g0448000	Similar to 4-coumarate--CoA ligase 1 (EC 6.2.1.12) (4CL 1) (4-coumaroyl-CoA synthase 1) (Clone 4	AK120964	Q9LL50	AMP-dependent synthetase/ligase
Os08g0448050	Hypothetical protein.	BT062457	longestORF	-
Os08g0448900	Conserved hypothetical protein.	CT832739	NP_001175602.1 (RefS	-
Os08g0449500	Conserved hypothetical protein.	AK120389	B8BB90	-
Os08g0449901	Hypothetical protein.	BT085732	longestORF	-
Os08g0450100	Similar to Pectinesterase (EC 3.1.1.11) (Fragment).	AK058651	A2YVS3	Pectinesterase, catalytic
Os08g0450200	Similar to Pectin methylesterase (Fragment).	AK072779	A2YVS4	Pectinesterase, catalytic
Os08g0450800	Phosphatidylinositol-4-phosphate 5-kinase family protein.	AK102479	XP_002882874.1 (RefS	Phosphatidylinositol-4-phosphate 5-kinase, core
Os08g0450900	SANT domain, DNA binding domain containing protein.	ab initio prediction	Q6ZLF1	SANT domain, DNA binding
Os08g0452200	Hypothetical protein.	AK100150	longestORF	-
Os08g0452500	Auxin responsive SAUR protein family protein.	AK106714	B6TUQ7	Auxin responsive SAUR protein
Os08g0453733	Non-protein coding transcript.	CT836006	NONE	-
Os08g0454000	Pathogenesis-related transcriptional factor/ERF, DNA-binding domain containing protein.	EU968979	Q6ZAG1	Pathogenesis-related transcriptional factor/ERF, DNA-binding
Os08g0455700	Similar to predicted protein.	AK240992 ,AK240990	NP_001061962.1 (RefS	Exo70 exocyst complex subunit
Os08g0456600	Hypothetical protein.	AK059009	longestORF	-
Os08g0457901	Hypothetical genes.	ab initio prediction	NONE	-
Os08g0458200	Similar to MTD1.	AK061399	NP_001148816.1 (RefS	-
Os08g0459100	Leucine-rich repeat, cysteine-containing containing protein.	AK121795	A3BTU1	-
Os08g0460000	Similar to Germin-like protein 1 precursor.	AK104729 ,AK065284	Q6ZBZ2	Germin
Os08g0460900	Conserved hypothetical protein.	ab initio prediction	NP_001061979.1 (RefS	-
Os08g0461300	Cyclin-like F-box domain containing protein.	AK065651	A3BTV7	F-box domain, cyclin-like
Os08g0463500	Zinc finger, C2H2-type domain containing protein.	AK058457	A2YVZ8	Zinc finger, C2H2
Os08g0464950	Hypothetical gene.	AK241196	longestORF	-
Os08g0465000	Hypothetical conserved gene.	CT835984	Q6YPD0	-
Os08g0467201	Hypothetical protein.	BT086922	longestORF	-
Os08g0467300	Clathrin adaptor, phosphoinositide-binding, GAT-like domain containing protein.	AK105541	NP_001151341.1 (RefS	ENTH/VHS
Os08g0467500	Abscisic acid and stress inducible (A22) gene.	AK100937	Q07764	TB2/DP1/HVA22-related protein
Os08g0468700	Similar to Nitrate reductase [NADH] 1 (EC 1.7.1.1) (NR1).	AK121810	P16081	Oxidoreductase, molybdopterin-binding domain
Os08g0468801	Hypothetical protein.	AK107363	longestORF	-
Os08g0469500	Similar to F-box domain containing protein.	AK109599	NP_001150287.1 (RefS	-
Os08g0469600	Conserved hypothetical protein.	AK062647	B8BBK4	-
Os08g0471000	Similar to cDNA, clone: J065120N14, full insert sequence.	AK356002	B7F8M8	Heat shock factor
Os08g0471150	Hypothetical protein.	tplb0033f11 (Wheat FLcDNA)	longestORF	-
Os08g0471950	bZIP transcription factor TRAB1.	Q6ZDF3	Q6ZDF3	Basic-leucine zipper
Os08g0472400	Conserved hypothetical protein.	FP097825	Q6ZDE7	-
Os08g0472800	Cytochrome P450 family protein.	AK120757	Q6ZDE3	Cytochrome P450
Os08g0473200	Hypothetical protein.	tplb0043h07 (Wheat FLcDNA)	longestORF	-
Os08g0475400	Alpha/beta hydrolase fold-3 domain containing protein.	AK060706 ,AK073975	NP_001150584.1 (RefS	Alpha/beta hydrolase fold-3
Os08g0476400	Similar to phosphoglycerate mutase gpmB.	AK241011	NP_001150922.1 (RefS	Phosphoglycerate/bisphosphoglycerate mutase, active site
Os08g0477600	Conserved hypothetical protein.	AK069216	A3BU43	-
Os08g0477900	Helix-loop-helix DNA-binding domain containing protein.	EU968808	NP_001159035.1 (RefS	Helix-loop-helix DNA-binding
Os08g0478566	Similar to Maturase K 2.	AY964640	C6ES29	-
Os08g0478800	Phosphoglucose isomerase (PGI) family protein.	AK107494	A3BU52	Phosphoglucose isomerase

Os08g0479400	Similar to Hydroxyproline-rich glycoprotein DZ-HRGP precursor.	AK109528	B6TE62	Homeobox domain, ZF-HD class
Os08g0480400	Similar to cupin, RmlC-type.	AK241844	NP_001147194.1 (RefS	Cupin, RmlC-type
Os08g0480800	Similar to TaWIN2.	AK101599	Q84J55	14-3-3 protein
Os08g0480901	Non-protein coding transcript.	BT017754	NONE	-
Os08g0481000	Hypothetical conserved gene.	AK111069	Q6Z235	Pentatricopeptide repeat
Os08g0481100	Similar to SKIP interacting protein 3 (Fragment).	AK240733	B8Q889	-
Os08g0481500	Conserved hypothetical protein.	AK241875	NP_001062065.2 (RefS	-
Os08g0481700	Hypothetical conserved gene.	CT835933	Q6Z230	-
Os08g0482025	Hypothetical protein.	BT009228	longestORF	-
Os08g0482300	Pistil-specific extensin-like protein domain containing protein.	AK107940	longestORF	-
Os08g0482600	Cupredoxin domain containing protein.	AK063639	A2YWC7	Blue
Os08g0482700	Cupredoxin domain containing protein.	AK060044	A2YWC8	Plastocyanin-like
Os08g0483100	Conserved hypothetical protein.	AK099821	A3BU75	-
Os08g0483600	Conserved hypothetical protein.	AK099543	B9G1G7	-
Os08g0483800	Hypothetical gene.	AK100866	longestORF	-
Os08g0483900	Helix-loop-helix DNA-binding domain containing protein.	AK107626	NP_001151793.1 (RefS	Helix-loop-helix DNA-binding
Os08g0484100	Similar to predicted protein.	AK242582	A3BU81	Glycoside hydrolase, subgroup, catalytic domain
Os08g0484200	Zinc finger, RING/FYVE/PHD-type domain containing protein.	AK287991 ,AK241976	NP_001062080.1 (RefS	Zinc finger, RING-type
Os08g0484300	Conserved hypothetical protein.	BT019110	C0P2Z7	-
Os08g0484450	Hypothetical protein.	tplb0055k03 (Wheat FLcDNA)	longestORF	-
Os08g0484500	Similar to Phospho-2-dehydro-3-deoxyheptonate aldolase 2.	AK106196	B6UAK5	DAHP synthetase, class II
Os08g0484633	Hypothetical protein.	tplb0031c07 (Wheat FLcDNA)	longestORF	-
Os08g0484800	Hypothetical conserved gene.	FP092392	Q6YTS7	-
Os08g0485000	Similar to PHI-1.	AK073011	NP_001150825.1 (RefS	Phosphate-induced protein 1
Os08g0485500	Conserved hypothetical protein.	AK241136	A3BU93	-
Os08g0485600	Hypothetical conserved gene.	AK103243	B8BBU4	Zinc finger, C2H2
Os08g0485800	Barwin-related endoglucanase domain containing protein.	AK242586	NP_001148968.1 (RefS	Expansin/pollen allergen, DPBB domain
Os08g0485900	Haloacid dehalogenase-like hydrolase domain containing protein.	AK110716	NP_568077.1 (RefSeq)	Haloacid dehalogenase-like hydrolase
Os08g0486100	Similar to Potential copper-transporting ATPase PAA1 (EC 3.6.3.4).	AK059217	XP_002304082.1 (RefS	ATPase, P-type, H+ transporting proton pump
Os08g0486200	Similar to Splicing factor SC35.	AK103676	B6UG76	RNA recognition motif domain
Os08g0486233	Similar to EIL transcription factor.	CT835917	Q8W3L9	Ethylene insensitive 3-like protein, DNA-binding domain
Os08g0486300	Similar to P-type R2R3 Myb protein (Fragment).	BT033636	Q8S416	SANT domain, DNA binding
Os08g0486400	Hypothetical protein.	EU971393	longestORF	-
Os08g0486700	Conserved hypothetical protein.	AK241195	NP_001062097.1 (RefS	-
Os08g0486750	Non-protein coding transcript.	AK363293	NONE	-
Os08g0486801	Non-protein coding transcript.	AK241869	NONE	-
Os08g0486867	Conserved hypothetical protein.	AK243168	Q75LB0	-
Os08g0486933	Similar to Peptide transporter-like protein.	ab initio prediction	Q6YS05	-
Os08g0487100	Similar to BZIP transcription factor BZI-2.	AK107150	NP_001152649.1 (RefS	Basic-leucine zipper
Os08g0487500	Zinc finger, RING/FYVE/PHD-type domain containing protein.	AK066326	B6U108	Zinc finger, RING-type
Os08g0487700	Helix-loop-helix DNA-binding domain containing protein.	ab initio prediction	NP_001062102.1 (RefS	Helix-loop-helix DNA-binding
Os08g0487800	Similar to Heat-shock protein precursor.	AK122102	Q43638	Heat shock protein Hsp90
Os08g0487850	Non-protein coding transcript.	BT017162	NONE	-
Os08g0489100	Hypothetical conserved gene.	ab initio prediction	Q6ZBR2	UDP-glucuronosyl/UDP-glucosyltransferase
Os08g0489300	Methyladenine glycosylase domain containing protein.	AK104597 ,AK059000	B4FL85	Methyladenine glycosylase
Os08g0490000	Helix-loop-helix DNA-binding domain containing protein.	AK100183	B6SVP6	Helix-loop-helix DNA-binding
Os08g0490100	Similar to PBF protein.	AK241364	Q1HFQ1	Zinc finger, Dof-type
Os08g0490600	FAS1 domain domain containing protein.	AK108305	Q06IA2	FAS1 domain
Os08g0490800	Similar to Histone H2B.	FP097117	B9G1J0	Histone H2B
Os08g0491000	Hypothetical conserved gene.	AK073673	B9G1J1	-
Os08g0491200	Serine/threonine protein kinase domain containing protein.	AK103656	B7EQ74	Protein kinase, catalytic domain
Os08g0491400	Interferon-related developmental regulator domain containing protein.	AK109684	A2YWI6	Interferon-related developmental regulator, N-terminal
Os08g0491700	Putative zinc finger CCCH domain-containing protein 57.	A3BUD2	A3BUD2	Zinc finger, CCCH-type
Os08g0492400	C2 calcium-dependent membrane targeting domain containing protein.	AK241274	B9G1J5	C2 calcium-dependent membrane targeting
Os08g0496100	Hypothetical conserved gene.	EU952779	Q6Z5C4	-
Os08g0499800	Pentatricopeptide repeat domain containing protein.	AK102014	Q2L3A2	Pentatricopeptide repeat
Os08g0500400	Hypothetical protein.	AK111024	longestORF	-
Os08g0500700	Similar to Heat shock protein 82.	AK102426	E0X6U7	Heat shock protein Hsp90
Os08g0501600	Similar to Wall associated kinase.	DQ177499	Q2L3D5	Protein kinase, catalytic domain
Os08g0501900	Non-protein coding transcript.	AK063130	NONE	-
Os08g0502101	Conserved hypothetical protein.	AK241735	NP_001175644.1 (RefS	-
Os08g0502600	EF hand domain containing protein.	AK107730	A2YWR5	Calcium-binding EF-hand
Os08g0504200	Similar to H0315A08.1 protein.	FP099421	Q01I63	Ribonuclease H1, N-terminal
Os08g0509400	Non-protein coding transcript.	AK060593	NONE	-
Os08g0510601	Non-protein coding transcript.	X02683	NONE	-
Os08g0511800	Conserved hypothetical protein.	AK063264	A2YWY7	-
Os08g0511900	Similar to aminoacylase-1.	AK240689	NP_001148589.1 (RefS	Peptidase M20
Os08g0512100	Conserved hypothetical protein.	AK359537	Q6Z8P1	-
Os08g0512200	Similar to heavy metal-associated domain containing protein.	EU952883	NP_001147000.1 (RefS	Heavy metal-associated domain, HMA
Os08g0512300	Similar to Rab geranylgeranyl transferase beta subunit (Fragment).	AK102273	XP_002871518.1 (RefS	Prenyltransferase/squalene oxidase
Os08g0512400	Protein of unknown function DUF296 domain containing protein.	AK067561	NP_001149717.1 (RefS	Domain of unknown function DUF296
Os08g0512700	Similar to 3-hydroxy-3-methylglutaryl coenzyme A reductase (EC 1.1.1.34) (Fragment).	AK060545	Q9XHL5	Hydroxymethylglutaryl-CoA reductase, class I/II
Os08g0512801	Hypothetical gene.	BT019297	longestORF	-
Os08g0519501	Hypothetical gene.	AK109201	longestORF	-
Os08g0524600	Similar to 60S ribosomal protein L32.	P51421	P51421	Ribosomal protein L32e
Os08g0525000	Ras GTPase family protein.	AK103220	NP_001152371.1 (RefS	Small GTPase superfamily
Os08g0525050	Non-protein coding transcript.	BT084300	NONE	-
Os08g0533300	Similar to ACR5.	AK287480	B6SYQ4	Amino acid-binding ACT
Os08g0533450	Hypothetical protein.	tplb0061b06 (Wheat FLcDNA)	longestORF	-
Os08g0535000	Similar to cation cation antiporter.	AK066324	NP_001148426.1 (RefS	Vesicle transport protein, Use1
Os08g0536333	Non-protein coding transcript.	CT835951	NONE	-
Os08g0557600	Similar to Monodehydroascorbate reductase (EC 1.6.5.4) (MDAR) (Ascorbate free radical reductase	AK102459	Q9SXX0	Pyridine nucleotide-disulphide oxidoreductase, NAD-binding domain
Os08g0557700	Similar to Histidine-containing phosphotransfer protein.	AK061111	Q6PT60	Signal transduction histidine kinase, phosphotransfer
Os08g0558800	Similar to Ribosomal protein.	AK122095	B8B9K6	Ribosomal protein L1
Os08g0559501	Conserved hypothetical protein.	CT837577	NP_001175701.1 (RefS	-
Os08g0559800	Hypothetical conserved gene.	AK242197	B9FYJ9	-
Os08g0564300	Hypothetical conserved gene.	AK106260	Q8GU78	ABC transporter, transmembrane domain
Os09g0114500	Hypothetical protein.	AK059015	longestORF	-
Os09g0114566	Non-protein coding transcript.	X06284	NONE	-
Os09g0115900	Non-protein coding transcript.	AK060240	NONE	-
Os09g0116800	Similar to DNA binding protein.	EU975895	B6TCD9	Transcription factor, MADS-box
Os09g0119600	UDP-glucuronosyl/UDP-glucosyltransferase family protein.	AK059022	C4MF39	UDP-glucuronosyl/UDP-glucosyltransferase
Os09g0122200	Hypothetical conserved gene.	AK106587	Q60DP5	Myb/SANT-like domain
Os09g0125400	Hypothetical conserved gene.	ab initio prediction	Q6K385	-
Os09g0127800	Similar to cDNA clone:J023075G08, full insert sequence.	AK058402	B7EQ85	Coatomer, WD associated region
Os09g0129800	Conserved hypothetical protein.	ab initio prediction	Q6ERA9	-
Os09g0134900	Hypothetical gene.	AK063225	longestORF	-
Os09g0240500	Similar to Sulfate transporter 4.1.	AK287664	B6SXI4	Sulphate transporter/antisigma-factor antagonist STAS
Os09g0242000	Similar to F-box domain containing protein, expressed.	ab initio prediction	D8L9K3	-
Os09g0247700	Similar to Zinc finger, ZZ-type%3B Zinc finger, C2H2-type.	AK059400	A2Q2P2	-
Os09g0251400	Non-protein coding transcript.	AK059379	NONE	-
Os09g0253775	Hypothetical protein.	AK372466	longestORF	-
Os09g0264011	Conserved hypothetical protein.	AK372466	Q6K2H9	-
Os09g0273600	Hypothetical gene.	CT835836	longestORF	-
Os09g0311600	Similar to NBS-LRR type resistance protein (Fragment).	AK102321	Q69KE4	Disease resistance protein
Os09g0322500	Hypothetical conserved gene.	AK243660	A3BXF1	Ankyrin repeat
Os09g0325140	Hypothetical conserved gene.	ab initio prediction	D7UPN3	-
Os09g0334050	Hypothetical gene.	tplb0008l20 (Wheat FLcDNA)	longestORF	-
Os09g0334600	Similar to Cytochrome b5.	AK335015	B6UG94	Cytochrome b5
Os09g0343600	Non-protein coding transcript.	AK061057	NONE	-
Os09g0365450	Similar to DNA binding protein.	AK367679	NP_001146992.1 (RefS	-
Os09g0376900	Similar to Potassium transporter 13 (AtPOT13) (AtKT5).	AK070831	Q6H4R6	K+ potassium transporter
Os09g0380400	Conserved hypothetical protein.	AK073073 ,AK063909	B8BF06	-
Os09g0391501	Hypothetical gene.	CT835895	longestORF	-
Os09g0392400	Similar to Pleiotropic drug resistance protein 13.	AK072152	Q8S628	-

Os09g0395300	GARP DNA-binding protien, Leaf polarity modeling	FJ268748	Q0J235	-
Os09g0396300	Peptidase C15, pyroglutamyl peptidase I family protein.	AK060069 ,AK105043	NP_001149461.1 (RefS	Peptidase C15, pyroglutamyl peptidase I
Os09g0396900	Protein of unknown function DUF125, transmembrane family protein.	AK071589	NP_001150466.1 (RefS	Domain of unknown function DUF125, transmembrane
Os09g0397100	Conserved hypothetical protein.	AK243387	NP_001063102.1 (RefS	-
Os09g0397200	Conserved hypothetical protein.	AK241559	NP_001063103.2 (RefS	-
Os09g0397400	Protein of unknown function DUF231, plant domain containing protein.	AK063997	Q6ERD8	Domain of unknown function DUF231, plant
Os09g0397700	Glutathione-dependent formaldehyde-activating, GFA family protein.	AK111009	B6T550	Glutathione-dependent formaldehyde-activating family, GFA
Os09g0397800	Hypothetical gene.	AK062361	longestORF	-
Os09g0406000	Similar to Uncharacterized protein OsI_031781.	AK120862	A2Z5D8	-
Os09g0406100	Similar to Uncharacterized protein OsI_030282.	AK288278	A2Z139	-
Os09g0406300	Similar to Uncharacterized protein OsI_031781.	AK288278	A2Z5D8	-
Os09g0406500	Similar to Uncharacterized protein OsI_030282.	AK288278	A2Z139	-
Os09g0409312	Non-protein coding transcript.	EU941912	NONE	-
Os09g0426050	Hypothetical gene.	BT084142	longestORF	-
Os09g0445700	ATPase, AAA+ type, core domain containing protein.	ab initio prediction	B8BCH8	ATPase, AAA+ type, core
Os09g0447000	Homeodomain-related domain containing protein.	AY559046	Q67UE2	Homeobox
Os09g0470000	Similar to Plastidic general dicarboxylate transporter.	AK059359	Q9ZTT4	Sodium/sulphate symporter
Os09g0473050	Hypothetical protein.	AK372466	longestORF	-
Os09g0473200	Similar to blight-associated protein p12.	EU961552	NP_001148968.1 (RefS	Expansin/pollen allergen, DPBB domain
Os09g0473350	Similar to methylcytosine binding domain protein.	CT835883	B3GS43	-
Os09g0473400	Peptidase C12, ubiquitin carboxyl-terminal hydrolase 1 domain containing protein.	EU976366	NP_192633.2 (RefSeq)	Plant organelle RNA recognition domain
Os09g0473502	Hypothetical gene.	CT835876	longestORF	-
Os09g0473600	Hypothetical conserved gene.	EU973630	B9G446	-
Os09g0473800	Hypothetical conserved gene.	AK108803	A3BZV2	-
Os09g0474000	bZIP transcription factor, bZIP-1 domain containing protein.	AK108319	B8BCQ0	Basic-leucine zipper
Os09g0480532	Conserved hypothetical protein.	ab initio prediction	Q69QS1	-
Os09g0480600	Conserved hypothetical protein.	AK107853	A3BZZ7	-
Os09g0526300	Similar to Homeobox protein GLABRA2 (Homeobox-leucine zipper protein ATHB-10) (HD-ZIP pro	AB101649	Q7Y0V7	Homeobox
Os09g0526500	C2 calcium/lipid-binding region, CaLB domain containing protein.	CT841705	NP_001175951.1 (RefS	-
Os09g0526600	Similar to Isoform 2 of Heat stress transcription factor B-2c.	AK106525	Q652B0-2	Heat shock factor
Os09g0526650	Conserved hypothetical protein.	tplb0033f11 (Wheat FLcDNA)	B8AME9	-
Os09g0526700	Similar to UDP-glucose 4-epimerase (EC 5.1.3.2) (Galactowaldenase) (UDP-galactose 4-epimerase)	AK073610	A6MD13	NAD-dependent epimerase/dehydratase
Os09g0526800	NC domain containing protein.	AK105121 ,AK060048	A6MD14	NC
Os09g0527500	Similar to MEG5.	AK110546	B7EM90	RNA recognition motif domain
Os09g0527600	Similar to GTP-binding protein (Fragment).	AK111543	A6MD16	Small GTPase superfamily
Os09g0527650	Hypothetical protein.	BT084300	longestORF	-
Os09g0527700	Similar to Auxin-induced protein IAA4.	AK111128	Q652A1	AUX/IAA protein
Os09g0528000	Hypothetical conserved gene.	AK060588	B9G4P1	Protein of unknown function DUF3490
Os09g0528050	Hypothetical protein.	tplb0030a08 (Wheat FLcDNA)	longestORF	-
Os09g0528100	Similar to 30S ribosomal protein S31, chloroplast (Fragment).	AK059096	B6UIF3	-
Os09g0528200	Similar to Homeobox-leucine zipper protein HOX6.	AK103160	Q651Z5	Helix-turn-helix motif, lambda-like repressor
Os09g0528500	Hypothetical protein.	tplb0044m18 (Wheat FLcDNA)	longestORF	-
Os09g0528700	Similar to Cytochrome p450 (CYP78A9).	ab initio prediction	B6ST44	Cytochrome P450
Os09g0528750	Non-protein coding transcript.	CT835864	NONE	-
Os09g0541700	Similar to mRNA, clone: RTFL01-25-P14.	AK104841	E4MY13	UspA
Os09g0541900	Similar to 26S proteasome subunit RPN3a.	AK101536	B6TAE2	Proteasome component
Os09g0542000	Similar to Ribonuclease P.	AK102114	B6T7F1	DNA/RNA-binding protein Alba-like
Os09g0542100	Peptidase A1 domain containing protein.	AK105001	Q01MD1	Peptidase A1
Os09g0542600	Similar to RING-H2 finger protein ATL1I.	AK060109	B8BE25	Zinc finger, RING-type
Os09g0542800	Pentatricopeptide repeat domain containing protein.	AK240855	XP_002890377.1 (RefS	Pentatricopeptide repeat
Os09g0542900	Conserved hypothetical protein.	AK073400	B8BE26	-
Os09g0546100	Auxin responsive SAUR protein family protein.	AK240906	NP_001150183.1 (RefS	Auxin responsive SAUR protein
Os09g0547100	Similar to Auxin induced protein.	AK058556	NP_001105462.1 (RefS	Auxin responsive SAUR protein
Os09g0548083	Non-protein coding transcript.	tplb0058a02 (Wheat FLcDNA)	NONE	-
Os09g0548200	Peptidoglycan-binding Lysin subgroup domain containing protein.	AK060664	Q651P3	Peptidoglycan-binding Lysin subgroup
Os09g0549425	Conserved hypothetical protein.	AK374610	F2EEN4	-
Os09g0549500	Nucleotide-binding, alpha-beta plait domain containing protein.	FP096667	NP_001150292.1 (RefS	RNA recognition motif domain
Os09g0551100	Hypothetical protein.	AK107540	longestORF	-
Os09g0551251	Similar to H0105C05.10 protein.	ab initio prediction	Q25A21	Protein kinase, catalytic domain
Os09g0552500	RmlC-like jelly roll fold domain containing protein.	AK108790	B9G4Y6	Cupin 1
Os09g0552800	No apical meristem (NAM) protein domain containing protein.	AK361030	B9G4Y8	No apical meristem
Os09g0552900	Similar to NAC domain protein, IPR003441.	EU942489	XP_002318332.1 (RefS	No apical meristem
Os09g0553100	Similar to Histone H4.	CU406630	B6T0P4	Histone H4
Os09g0553200	UDPase, Pollen mother cell (PMC) meiosis, Pollen development	DQ395328 ,AB062606 ,AK11919	A3QQQ3 ,Q93X08	-
Os09g0553250	Hypothetical gene.	BT016879	longestORF	-
Os09g0553700	Similar to PRLI-interacting factor A (Fragment).	AK070189	B6TGN5	-
Os09g0553750	Hypothetical gene.	EU970031	longestORF	-
Os09g0554200	Zinc finger, RING/FYVE/PHD-type domain containing protein.	AK108361	NP_001146938.1 (RefS	Zinc finger, RING-type
Os09g0555400	Pentatricopeptide repeat domain containing protein.	AK073402	A2Z3X5	Pentatricopeptide repeat
Os09g0555450	Conserved hypothetical protein.	EU950356	F2EDP0	-
Os09g0555700	Zinc finger, C2H2-type domain containing protein.	AK061264	Q5UDB7	Zinc finger, C2H2
Os09g0564400	Hypothetical conserved gene.	Z97023	B9G543	Peptidase, cysteine peptidase active site
Os09g0567300	Similar to Monodehydroascorbate reductase (EC 1.6.5.4) (MDAR) (Ascorbate free radical reductase	AK071558	Q9XFZ3	Pyridine nucleotide-disulphide oxidoreductase, NAD-binding domain
Os09g0567400	Similar to Histidine-containing phosphotransfer protein.	AK072521	Q6VAK4	Signal transduction histidine kinase, phosphotransfer
Os09g0568100	Conserved hypothetical protein.	FP092518	B9G564	-
Os09g0568400	Similar to 60S ribosomal protein L40 (CEP52).	AK059386	Q7XYD4	Ribosomal protein L40e
Os09g0568500	Germin family protein.	AK108987	Q84XR7	Germin
Os09g0568900	Similar to predicted protein.	AK059442	XP_002879140.1 (RefS	-
Os09g0569000	Hypothetical gene.	AK108627	longestORF	-
Os09g0569100	Haloacid dehalogenase-like hydrolase domain containing protein.	AK071860	B4FI81	Haloacid dehalogenase-like hydrolase
Os09g0569200	Similar to Beta-amylase (EC 3.2.1.2) (1,4-alpha-D-glucan maltohydrolase).	AK070300	B6SVZ0	Glycoside hydrolase, family 14B, plant
Os09g0569300	Similar to calmodulin-binding heat-shock protein.	AK069587	NP_001149987.1 (RefS	Lipase, class 3
Os09g0569450	Conserved hypothetical protein.	AK242543	NP_001175993.1 (RefS	-
Os09g0569700	Conserved hypothetical protein.	AK108778	NP_001063979.1 (RefS	-
Os09g0569780	Conserved hypothetical protein.	AK241041	NP_001175963.1 (RefS	-
Os09g0569800	Serine/threonine protein kinase-related domain containing protein.	ab initio prediction	NP_001063980.1 (RefS	Protein kinase, catalytic domain
Os09g0570000	Serine/threonine protein kinase-related domain containing protein.	AK058797	Q2HTK4	Protein kinase, catalytic domain
Os09g0570100	Protein kinase, catalytic domain domain containing protein.	AK061135	B9G572	Protein kinase, catalytic domain
Os09g0570150	Hypothetical conserved gene.	EU953102	B6SK37	-
Os09g0570300	Similar to Short-chain dehydrogenase Tic32.	AK061532	B4FKX6	Short-chain dehydrogenase/reductase SDR
Os09g0570400	Non-protein coding transcript.	AK061710	NONE	-
Os09g0570500	Zinc finger, RING-type domain containing protein.	AK069259	B9G575	Zinc finger, RING-type
Os09g0570600	Non-protein coding transcript.	AK105456	NONE	-
Os09g0570850	Similar to Histone H2B.	ab initio prediction	B6SGC3	Histone H2B
Os09g0571033	Conserved hypothetical protein.	AK241104	NP_001175997.1 (RefS	-
Os09g0571100	Pectin lyase fold/virulence factor domain containing protein.	AK106869	C5X6M1	Pectinesterase, catalytic
Os09g0571200	C2 domain containing protein.	AK062604	B6TT83	C2 calcium-dependent membrane targeting
Os09g0572000	Pathogenesis-related transcriptional factor and ERF domain containing protein.	AK241281	XP_002306842.1 (RefS	Pathogenesis-related transcriptional factor/ERF, DNA-binding
Os09g0572550	Hypothetical gene.	AK243102	longestORF	-
Os09g0572600	Similar to Receptor protein kinase-like protein.	AK070226	NP_188689.1 (RefSeq)	Protein kinase, catalytic domain
Os09g0572950	Non-protein coding transcript.	tplb0034e16 (Wheat FLcDNA)	NONE	-
Os09g0573000	Pentatricopeptide repeat domain containing protein.	AK073399	B9G584	Pentatricopeptide repeat
Os09g0573100	Ubiquitin domain containing protein.	AK121232	XP_002881393.1 (RefS	Uncharacterised domain SAYSvFN
Os10g0116600	Serine/threonine protein kinase domain containing protein.	ab initio prediction	Q7XH75	Protein kinase, catalytic domain
Os10g0116650	Hypothetical protein.	tplb0029l08 (Wheat FLcDNA)	longestORF	-
Os10g0116701	Hypothetical conserved gene.	ab initio prediction	Q7XH75	EGF-type aspartate/asparagine hydroxylation site
Os10g0122100	Hypothetical protein.	AK372466	longestORF	-
Os10g0141300	Similar to Protein kinase domain containing protein.	ab initio prediction	Q7XGY5	Protein kinase, catalytic domain
Os10g0141400	Similar to 26S proteasome non-ATPase regulatory subunit 4.	BT063362	NP_001150010.1 (RefS	von Willebrand factor, type A
Os10g0141900	Queuine/other tRNA-ribosyltransferase family protein.	AK106467	Q948C4	Queuine/other tRNA-ribosyltransferase
Os10g0142100	Similar to Zinc finger, C3HC4 type family protein, expressed.	AK058523	Q10A50	Zinc finger, RING-type
Os10g0142500	Cyclin-like F-box domain containing protein.	AK072788	Q948C2	F-box domain, cyclin-like
Os10g0144000	Conserved hypothetical protein.	EU945087	NP_001064152.1 (RefS	-
Os10g0167401	Non-protein coding transcript.	BT084318	NONE	-
Os10g0192301	Non-protein coding transcript.	FP100159	NONE	-

Os10g0208900	Hypothetical conserved gene.	AK109751	Q53NH3	-
Os10g0339650	Non-protein coding transcript.	AK109165	NONE	-
Os10g0340950	Non-protein coding transcript.	EU941912	NONE	-
Os10g0351414	Endonuclease/exonuclease/phosphatase domain containing protein.	tplb0014n19 (Wheat FLcDNA)	NP_001057661.2 (RefS	Endonuclease/exonuclease/phosphatase
Os10g0355800	Similar to ATP synthase CF1 beta subunit.	ab initio prediction	NP_039390.1 (RefSeq)	ATPase, F1/V1/A1 complex, alpha/beta subunit, nucleotide-binding domain
Os10g0356000	Similar to ribulose-1,5-bisphosphate carboxylase/oxygenase large subunit.	ab initio prediction	NP_039391.1 (RefSeq)	Ribulose bisphosphate carboxylase, large subunit, C-terminal
Os10g0377150	Hypothetical protein.	FP097651	longestORF	-
Os10g0410650	Hypothetical gene.	BT018461	longestORF	-
Os10g0411500	IQ calmodulin-binding region domain containing protein.	AK072572	B8BGS6	-
Os10g0411600	Hypothetical protein.	AK108669	longestORF	-
Os10g0411650	Non-protein coding transcript.	EU947431	NONE	-
Os10g0411700	Similar to S28 ribosomal protein (Fragment).	AK058209 ,AK121099	Q7X9K4	-
Os10g0412100	Similar to Endonuclease/Exonuclease/phosphatase family protein, expressed.	AK102038	Q10R98	Endonuclease/exonuclease/phosphatase
Os10g0413400	Similar to Glycerol-3-phosphate acyltransferase 6 (EC 2.3.1.15) (AtGPAT6).	BT039538	NP_001176155.1 (RefS	Phospholipid/glycerol acyltransferase
Os10g0429600	Similar to BTB/POZ domain containing protein.	AK369602	Q7XEA4	BTB/POZ-like
Os10g0430800	Conserved hypothetical protein.	AK111261	A3C4Z6	-
Os10g0432001	Hypothetical gene.	EU943065	longestORF	-
Os10g0432200	Conserved hypothetical protein.	AK106572	Q337X2	-
Os10g0432300	Non-protein coding transcript.	AK111369	NONE	-
Os10g0434200	Similar to BTB/POZ domain containing protein.	AK369602	Q337W6	BTB/POZ-like
Os10g0438000	Similar to Potassium channel tetramerisation domain-containing protein 9.	ab initio prediction	B6TKK7	BTB/POZ-like
Os10g0438800	Similar to RING-H2 finger protein ATL2E.	AK063228	NP_001151998.1 (RefS	Zinc finger, RING-type
Os10g0439333	Similar to BTB/POZ domain containing protein.	AK376276	Q7XE33	BTB/POZ-like
Os10g0440050	Non-protein coding transcript.	BT009415	NONE	-
Os10g0457000	Hypothetical conserved gene.	AK061041	B9G629	RNA recognition motif domain
Os10g0459300	Exostosin-like family protein.	tplb0047d21 (Wheat FLcDNA)	NP_001150032.1 (RefS	Exostosin-like
Os10g0464050	Non-protein coding transcript.	EU969129	NONE	-
Os10g0468250	Putative harbinger transposase-derived nuclease domain containing protein.	AK335074	Q8S819	Putative harbinger transposase-derived nuclease
Os10g0468301	Hypothetical conserved gene.	AK106587	Q60DP5	Myb/SANT-like domain
Os10g0472501	Hypothetical conserved gene.	tplb0015l09 (Wheat FLcDNA)	Q9FW60	-
Os10g0478350	Hypothetical protein.	EU971393	longestORF	-
Os10g0479800	Protein of unknown function DUF1336 domain containing protein.	AK107572	NP_180399.2 (RefSeq)	Domain of unknown function DUF1336
Os10g0488400	Protein of unknown function DUF761, plant domain containing protein.	AK108465	Q8W5I9	Protein of unknown function DUF761, plant
Os10g0489650	Conserved hypothetical protein.	AK242732	B9G6D4	-
Os10g0492101	Hypothetical conserved gene.	CT841749	Q9FWV2	-
Os10g0492200	Glycosyltransferase AER61, uncharacterized domain containing protein.	AK109476	NP_001105165.1 (RefS	Glycosyltransferase AER61, uncharacterised
Os10g0492500	Similar to Tonoplast membrane integral protein ZmTIP3-1.	AK106383	longestORF	-
Os10g0492600	Similar to Tonoplast membrane integral protein ZmTIP3-1.	AK111931	NP_001105032.1 (RefS	Major intrinsic protein
Os10g0493600	Similar to Alpha-galactosidase (Fragment).	AK058373	O04944	Glycosyl hydrolase, family 13, all-beta
Os10g0493850	Hypothetical protein.	tplb0029b01 (Wheat FLcDNA)	longestORF	-
Os10g0493900	Protein of unknown function DUF6, transmembrane domain containing protein.	AK071193	XP_002882247.1 (RefS	Drug/metabolite transporter
Os10g0494000	Protein of unknown function DUF789 family protein.	AK106067 ,AK071373	A2Z8W3	Protein of unknown function DUF789
Os10g0495050	Non-protein coding transcript.	EU946286	NONE	-
Os10g0497500	K Homology, type 1, subgroup domain containing protein.	AK107213	A3C631	K Homology
Os10g0497550	Non-protein coding transcript.	EU946286	NONE	-
Os10g0503550	Non-protein coding transcript.	CT835611	NONE	-
Os10g0504650	Similar to nonspecific lipid-transfer protein.	ab initio prediction	Q7G2C5	Plant lipid transfer protein/seed storage/trypsin-alpha amylase inhibitor
Os10g0511800	Similar to Plus-3 domain containing protein, expressed.	EU942983	Q7XCX5	SWIB/MDM2 domain
Os10g0516500	Conserved hypothetical protein.	AK059055	Q94HZ3	-
Os10g0516551	Hypothetical protein.	BT088333	longestORF	-
Os10g0518150	Non-protein coding transcript.	BT016918	NONE	-
Os10g0524300	Peptidoglycan-binding lysin domain domain containing protein.	AK063244	NP_001150468.1 (RefS	Peptidoglycan-binding lysin domain
Os10g0524400	Similar to Phospholipase D beta 2.	AK121075	B9G6P8	C2 calcium-dependent membrane targeting
Os10g0524766	Hypothetical gene.	EU941347	longestORF	-
Os10g0525000	Similar to Cytochrome P450 family protein, expressed.	AK105884	Q109D2	Cytochrome P450
Os10g0525101	Hypothetical gene.	BT065154	longestORF	-
Os10g0525301	Hypothetical gene.	BT065154	longestORF	-
Os10g0527601	Glutathione S-transferase, C-terminal-like domain containing protein.	ab initio prediction	Q6WSC2	Glutathione S-transferase, N-terminal
Os10g0528900	Similar to Tau class GST protein 3.	AK059726	Q6WSC2	Glutathione S-transferase, N-terminal
Os10g0529400	Similar to Glutathione S-transferase GSTU6.	AK102196	B6U486	Glutathione S-transferase, N-terminal
Os10g0529450	Hypothetical protein.	AK364077	longestORF	-
Os10g0530300	Similar to Glutathione S-transferase GST 42 (Fragment).	ab initio prediction	Q9FQ97	Glutathione S-transferase, C-terminal
Os10g0530800	Hypothetical protein.	AK111429	longestORF	-
Os10g0530900	Similar to Glutathione S-transferase GST 30 (EC 2.5.1.18).	AK103699 ,AK059760	Q8RW01	Glutathione S-transferase, N-terminal
Os10g0532100	Similar to Amine oxidase, flavin-containing.	AK065403	Q336Y0	Amine oxidase
Os10g0532150	Similar to Amine oxidase, flavin-containing family protein, expressed.	AK372474	Q336Y0	-
Os10g0534400	Similar to E4 protein (Fragment).	AK105216	B8BI06	-
Os10g0534500	Similar to Resistance protein candidate (Fragment).	AK070172	Q8LN27	Protein kinase, catalytic domain
Os10g0534600	Hypothetical protein.	AK103845	longestORF	-
Os10g0534700	Protein of unknown function DUF604 family protein.	AK071861	Q01KI0	Protein of unknown function DUF604
Os10g0539900	Similar to Hexose transporter.	AK059705	B6U6U2	General substrate transporter
Os10g0540100	Pentatricopeptide repeat domain containing protein.	AK074016	Q9FRJ8	Pentatricopeptide repeat
Os10g0542100	Plant EC metallothionein-like protein, family 15 protein.	AK063504 ,AK102405	Q109B0	Plant EC metallothionein-like protein, family 15
Os10g0542800	Similar to ATP binding protein.	AK121234	NP_001151339.1 (RefS	Tetratricopeptide-like helical
Os10g0543800	Similar to Glutathione S-transferase GST 11 (EC 2.5.1.18).	CT836577	NP_001104983.1 (RefS	Glutathione S-transferase, N-terminal
Os10g0544933	Non-protein coding transcript.	EU943254	NONE	-
Os10g0544966	Hypothetical protein.	tplb0043b08 (Wheat FLcDNA)	longestORF	-
Os10g0545000	Similar to magnesium transporter CorA-like family protein.	AK069472	NP_567076.1 (RefSeq)	Fibronectin-attachment
Os10g0545100	Similar to predicted protein.	AK287840	B9G1Z7	Uncharacterised conserved protein UCP014543
Os10g0545200	Similar to 4,4-dimethyl-sterol C4-methyl-oxidase (Fragment).	AK121804	B4FN64	Fatty acid hydroxylase
Os10g0545300	Zinc finger, CCHC retroviral-type domain containing protein.	AK065156 ,AK121559	NP_001149324.1 (RefS	Zinc finger, CCHC-type
Os10g0546400	Conserved hypothetical protein.	AK069919	B9G6V0	-
Os10g0546600	Similar to Chloroplast carotenoid epsilon-ring hydroxylase.	AK065689	NP_190881.2 (RefSeq)	Cytochrome P450
Os10g0546900	Similar to Zinc finger, C3HC4 type family protein, expressed.	AK368666	Q109A4	Zinc finger, RING-type
Os10g0547200	Harpin-induced 1 domain containing protein.	AK060129	A2Z9Y3	Late embryogenesis abundant protein, LEA-14
Os10g0549600	Non-protein coding transcript.	EU947769	NONE	-
Os10g0549650	Hypothetical protein.	EU951562	longestORF	-
Os10g0550066	Hypothetical conserved gene.	BT067999	XP_002466024.1 (RefS	Mediator complex, subunit Med12
Os10g0550100	Conserved hypothetical protein.	AK106583	Q94LW0	-
Os10g0550725	Hypothetical gene.	AK240685	longestORF	-
Os10g0550750	Protein of unknown function DUF3527 domain containing protein.	AK375090	B9G6W8	Protein of unknown function DUF3527
Os10g0551100	Conserved hypothetical protein.	AK106659	Q9FWN4	-
Os10g0551700	Plant lipid transfer protein and hydrophobic protein, helical domain containing protein.	AK107851	A2ZA19	Plant lipid transfer protein/seed storage/trypsin-alpha amylase inhibitor
Os10g0551800	Root-specific protein (RCc2 protein).	L27209	A2ZA20	Plant lipid transfer protein/seed storage/trypsin-alpha amylase inhibitor
Os10g0553900	Hypothetical conserved gene.	AK060446	A2ZA32	Protein of unknown function DUF863, plant
Os10g0554050	Non-protein coding transcript.	tplb0022l14 (Wheat FLcDNA)	NONE	-
Os10g0554100	Similar to Oxysterol-binding protein.	AK071048	C3SAC1	Oxysterol-binding protein
Os10g0557133	Similar to H0315A08.1 protein.	FP099421	Q01I63	Ribonuclease H1, N-terminal
Os10g0560500	Conserved hypothetical protein.	AK100157	Q8SBA5	-
Os10g0560850	Hypothetical gene.	EU940765	longestORF	-
Os10g0561000	Hypothetical conserved gene.	BT064708	Q9AUT7	-
Os10g0561300	Similar to Monosaccharid transporter.	AK068343	NP_001149551.1 (RefS	Sugar/inositol transporter
Os10g0566800	Haem peroxidase, plant/fungal/bacterial family protein.	AK063299	Q5U1H3	Plant peroxidase
Os10g0567000	Camphor resistance CrcB protein family protein.	AK103373	NP_001147074.1 (RefS	Camphor resistance CrcB protein
Os10g0567300	SH2 motif domain containing protein.	AK070829	Q336Q9	SH2 motif
Os10g0567500	RNA-directed DNA polymerase (reverse transcriptase) domain containing protein.	AK072336	B6SWF1	Reverse transcriptase
Os10g0567800	Similar to predicted protein.	AK061703	NP_567094.1 (RefSeq)	Dynamin central domain
Os10g0567900	Similar to F-box protein interaction domain containing protein, expressed.	AK064083	Q108X5	F-box associated interaction domain
Os10g0571250	Non-protein coding transcript.	EU950639	NONE	-
Os10g0571600	No apical meristem (NAM) protein domain containing protein.	AK110684	XP_002329572.1 (RefS	No apical meristem
Os10g0572400	Conserved hypothetical protein.	AK062550	Q8S7M6	-
Os10g0572950	Hypothetical protein.	EU959880	longestORF	-
Os10g0575650	Hypothetical gene.	BT086509	longestORF	-
Os10g0575950	Similar to F5D14.6 protein.	AK241904	Q9LQM9	-
Os10g0577000	Conserved hypothetical protein.	AK059043	Q8W3G9	-

Os10g0578500	Hypothetical conserved gene.	ab initio prediction	Q7XBT9	Pentatricopeptide repeat
Os10g0578900	Similar to CTP:phosphorylcholine cytidylyltransferase (EC 2.7.7.15).	AK073939	NP_001150315.1 (RefS	Cytidylyltransferase
Os10g0579150	Hypothetical gene.	EU951391	longestORF	-
Os10g0579400	Conserved hypothetical protein.	AK108346	Q9FRP5	-
Os10g0579800	TGF-beta receptor, type I/II extracellular region family protein.	AK121847	NP_001148010.1 (RefS	Oligopeptide transporter
Os10g0579850	Hypothetical protein.	tplb0038k08 (Wheat FLcDNA)	longestORF	-
Os10g0580300	Serine/threonine protein kinase domain containing protein.	AK066824 ,AK104880	Q336M2	Protein kinase, catalytic domain
Os10g0580900	Non-protein coding transcript.	AK062821	NONE	-
Os11g0100300	Conserved hypothetical protein.	AK369990	NP_001176293.1 (RefS	-
Os11g0100600	Alpha/beta hydrolase family protein.	AK060331	B4FK75	Alpha/beta hydrolase fold-1
Os11g0100800	Similar to Exo70 exocyst complex subunit family protein.	AK287644	Q2RBS0	Exo70 exocyst complex subunit
Os11g0101200	Similar to RNase P subunit p30 family protein, expressed.	AK058862	Q2RBR8	-
Os11g0102100	WUS-type homenodomain protein, Leaf development	AM490243	A8Y7W4	-
Os11g0104900	Similar to Clathrin heavy chain.	ab initio prediction	Q39834	Clathrin, heavy chain/VPS, 7-fold repeat
Os11g0104933	Non-protein coding transcript.	AK242528	NONE	-
Os11g0112400	Peroxidase (EC 1.11.1.7).	AK242814	Q5U1F8	Plant peroxidase
Os11g0127800	Similar to FHA domain containing protein, expressed.	AK059969 ,AK104179	Q2QYC9	Forkhead-associated
Os11g0127900	Similar to 40S ribosomal protein S16.	AK059550	Q0IQF7	Ribosomal protein S9
Os11g0127951	Hypothetical protein.	ab initio prediction	NONE	-
Os11g0128500	Similar to Myb factor.	AK241549	O04109	SANT domain, DNA binding
Os11g0128600	Conserved hypothetical protein.	ab initio prediction	NP_001065637.1 (RefS	-
Os11g0128700	Mad3/BUB1 homology region 1 domain containing protein.	AK102502	Q2RB24	Mad3/BUB1 homology region 1
Os11g0128800	Similar to Homoserine dehydrogenase-like protein.	AK071434	B8AES5	Homoserine dehydrogenase, catalytic
Os11g0128932	Hypothetical gene.	BT054648	longestORF	-
Os11g0129500	Similar to GDSL-like Lipase/Acylhydrolase family protein, expressed.	AK107889	Q2RB18	-
Os11g0130100	Conserved hypothetical protein.	AK063914	Q2RB13	-
Os11g0130400	Harpin-induced 1 domain containing protein.	AK243509	NP_001065651.2 (RefS	Late embryogenesis abundant protein, LEA-14
Os11g0130500	Cyclin-like F-box domain containing protein.	AK241516	Q2RB09	F-box domain, cyclin-like
Os11g0130600	Conserved hypothetical protein.	AK066342	NP_001066054.1 (RefS	-
Os11g0130700	Conserved hypothetical protein.	ab initio prediction	B9G976	-
Os11g0131100	VQ domain containing protein.	CT835703	Q2RB04	VQ
Os11g0131400	Hypothetical conserved gene.	AK109466	Q2RB01	Pentatricopeptide repeat
Os11g0131500	Hypothetical protein.	BT085496	longestORF	-
Os11g0131600	Similar to YDG/SRA domain containing protein.	BT055690	Q2RB00	SET domain
Os11g0131700	Zinc finger, Tim10/DDP-type domain containing protein.	AK243327	B6SLJ2	Mitochondrial inner membrane translocase complex, Tim8/9/10/13-zinc finger-like
Os11g0131900	Similar to alpha-N-arabinofuranosidase A.	AK061612	B6T9B9	Alpha-L-arabinofuranosidase, C-terminal
Os11g0132400	Uncharacterised protein family UPF0414 domain containing protein.	AK059074	NP_001176772.1 (RefS	Protein of unknown function DUF872, transmembrane
Os11g0133300	Similar to D-mannose binding lectin family protein, expressed.	AK121819 ,AK102340	Q2QY68	Epidermal growth factor-like, type 3
Os11g0133700	Similar to Glucosamine-fructose-6-phosphate aminotransferase, isomerizing family protein, expresse	AK058784	Q2RAY0	-
Os11g0133850	Hypothetical gene.	EU946073	longestORF	-
Os11g0134400	Calmodulin-like protein 3.	Q0IQB6	Q0IQB6	Calcium-binding EF-hand
Os11g0137050	Hypothetical protein.	tplb0036f07 (Wheat FLcDNA)	longestORF	-
Os11g0137250	Hypothetical gene.	AK242555	longestORF	-
Os11g0144950	Hypothetical protein.	FP101342	longestORF	-
Os11g0149900	PAN-like, type 2 domain containing protein.	BT034293	NP_001176354.1 (RefS	S-locus glycoprotein
Os11g0166800	Transcription elongation factor, TFIIS/CRSP70, N-terminal domain containing protein.	AK070928	Q53PV2	Transcription factor IIS, N-terminal
Os11g0166866	Non-protein coding transcript.	BT065504	NONE	-
Os11g0166932	Non-protein coding transcript.	EU949846	NONE	-
Os11g0168000	Protein of unknown function DUF1278 family protein.	AK062599	Q9SES8	Protein of unknown function DUF784
Os11g0168200	60S ribosomal protein L3.	AK073392 ,AK101469	A5D934	Ribosomal protein L3
Os11g0168250	Hypothetical gene.	BT016883	longestORF	-
Os11g0168300	Alba, DNA/RNA-binding protein family protein.	AK061362 ,AK104405	B6T7F3	DNA/RNA-binding protein Alba-like
Os11g0168600	Protein kinase, core domain containing protein.	AK063805	Q53JG3	Protein kinase, catalytic domain
Os11g0180000	Plant disease resistance response protein family protein.	AK060077	NP_001150515.1 (RefS	Plant disease resistance response protein
Os11g0180100	Conserved hypothetical protein.	AK375448	Q53NQ8	-
Os11g0180900	Myb transcription factor domain containing protein.	BT069656	Q6SL45	SANT domain, DNA binding
Os11g0181000	Hypothetical protein.	EU971393	longestORF	-
Os11g0181200	Hypothetical protein.	AK063747	longestORF	-
Os11g0182100	Protein of unknown function DUF688 domain containing protein.	AK107888	Q53MM1	Protein of unknown function DUF688
Os11g0182400	WD40/YVTN repeat-like domain containing protein.	AK112089	B6TCZ0	WD40 repeat
Os11g0193300	Hypothetical conserved gene.	AK109751	Q53NH3	-
Os11g0201175	Similar to H0315A08.1 protein.	FP099421	Q01I63	-
Os11g0222800	Similar to LGC1.	EU947580	Q53P07	-
Os11g0247900	Non-protein coding transcript.	EU948158	NONE	-
Os11g0272675	Similar to H0315A08.1 protein.	FP099421	Q01I63	Ribonuclease H1, N-terminal
Os11g0272900	Conserved hypothetical protein.	AK068971	Q53MK6	-
Os11g0334938	Similar to Class III peroxidase 111.	BT085722	B6TU39	Plant peroxidase
Os11g0384789	Similar to Hydrolase.	AK104400 ,AK073355 ,AK10413	NP_001151350.1 (RefS	-
Os11g0440200	Similar to GDA1/CD39 family protein, expressed.	ab initio prediction	Q53KM8	Nucleoside phosphatase GDA1/CD39
Os11g0460851	Non-protein coding transcript.	tplb0037b22 (Wheat FLcDNA)	NONE	-
Os11g0596000	Hypothetical conserved gene.	AK288199	Q2R1R7	-
Os11g0623650	Non-protein coding transcript.	BT065526	NONE	-
Os11g0629900	Conserved hypothetical protein.	AK288199	Q2R0W5	-
Os11g0656500	Integrase, catalytic core domain containing protein.	AK072370	Q01M13	Integrase, catalytic core
Os11g0671600	Conserved hypothetical protein.	AK288199	Q2QZU2	-
Os11g0697350	Conserved hypothetical protein.	AK241561	NP_001176733.1 (RefS	-
Os11g0697600	Hypothetical gene.	CT841618	longestORF	-
Os11g0699532	Hypothetical gene.	CT841618	longestORF	-
Os12g0100200	Conserved hypothetical protein.	AK369990	NP_001176293.1 (RefS	-
Os12g0100700	Similar to Exo70 exocyst complex subunit family protein.	AK287644	Q2RBS0	Exo70 exocyst complex subunit
Os12g0101000	Similar to CCT motif family protein.	AK100957	Q2QYZ4	CCT domain
Os12g0101600	WUS-type homenodomain protein, Leaf development	AM490243	A8Y7W4	-
Os12g0101901	Hypothetical gene.	EU949907	longestORF	-
Os12g0102100	Alcohol dehydrogenase superfamily, zinc-containing protein.	AK068187	C5MRG3	Alcohol dehydrogenase superfamily, zinc-type
Os12g0102300	Similar to WRKY transcription factor 57.	AK242855	Q6IEM4	-
Os12g0106801	Hypothetical genes.	ab initio prediction	NONE	-
Os12g0118400	Similar to Inwardly rectifying potassium channel subunit.	AK062407	Q6ZXS4	Potassium channel, voltage-dependent, EAG/ELK/ERG
Os12g0124700	CDC45-like protein family protein.	AK073156	NP_189146.1 (RefSeq)	CDC45 family
Os12g0124900	Non-protein coding transcript.	AK106719	NONE	-
Os12g0125000	Myb factor.	AK061437	O04109	SANT domain, DNA binding
Os12g0125550	Hypothetical gene.	BT054648	longestORF	-
Os12g0125850	Hypothetical gene.	BT054648	longestORF	-
Os12g0131150	Similar to Glucosamine-fructose-6-phosphate aminotransferase, isomerizing family protein, expresse	AK058784	Q2RAY0	-
Os12g0132200	Similar to CBL-interacting protein kinase 33.	AK111953	Q2RAX3	Protein kinase, catalytic domain
Os12g0132300	Similar to Calmodulin (CaM).	AK111834 ,AK119956	Q0IQB6	-
Os12g0132400	Similar to Grwd-pending-prov protein.	AK111507	Q1HFN6	WD40 repeat
Os12g0132500	Similar to Major facilitator superfamily antiporter.	CT833630	Q8H6D5	-
Os12g0132650	Non-protein coding transcript.	tplb0023l07 (Wheat FLcDNA)	NONE	-
Os12g0132900	Hypothetical gene.	AK068041	GeneMark	-
Os12g0133000	Similar to Major facilitator superfamily antiporter.	EU970439	B6TZM4	Major facilitator superfamily
Os12g0133025	Non-protein coding transcript.	tplb0023l07 (Wheat FLcDNA)	NONE	-
Os12g0133050	Similar to 60S acidic ribosomal protein P0.	AK361198	NP_001105482.1 (RefS	Ribosomal protein L10/acidic P0
Os12g0133350	Non-protein coding transcript.	tplb0023l07 (Wheat FLcDNA)	NONE	-
Os12g0133700	Similar to Dual specificity protein phosphatase 19 (EC 3.1.3.48) (EC 3.1.3.16) (Stress-activated prot	CU405769	Q2HSZ6	Dual specificity phosphatase, catalytic domain
Os12g0133750	Hypothetical protein.	ab initio prediction	NONE	-
Os12g0133850	Hypothetical protein.	tplb0036f07 (Wheat FLcDNA)	longestORF	-
Os12g0133950	Non-protein coding transcript.	BT016200	NONE	-
Os12g0134000	Similar to hydroxymethylglutaryl-CoA lyase.	AK061875	B6TKJ5	Pyruvate carboxyltransferase
Os12g0134500	Similar to Zinc finger, C3HC4 type family protein.	AK358384	Q2RAT8	Zinc finger, RING-type
Os12g0135100	Similar to Phosphoglycerate mutase family protein.	BT062522	Q2QY22	Phosphoglycerate/bisphosphoglycerate mutase, active site
Os12g0139400	A-type response regulator, Cytokinin signaling	AB249660 ,BR000257	Q2QXY3	Signal transduction response regulator, receiver domain
Os12g0140300	FBD domain containing protein.	AK102092	B9G816	-
Os12g0140550	Hypothetical gene.	EU945989	longestORF	-
Os12g0149300	Similar to galactosyl transferase GMA12/MNN10 family protein.	AK110842	XP_002887538.1 (RefS	Galactosyl transferase
Os12g0149800	Similar to G10.	AK103299	NP_001148940.1 (RefS	G10 protein

Os12g0150200	Similar to Cytochrome P450 CYP94C20.	AK064287	B6STM6	Cytochrome P450
Os12g0150901	Conserved hypothetical protein.	tplb0051a13 (Wheat FLcDNA)	Q2QXM5	-
Os12g0151600	Hypothetical conserved gene.	BT061672	NP_190845.1 (RefSeq)	-
Os12g0152000	Similar to Terminal flower 1-like protein.	AF159883	Q9XGS5	Phosphatidylethanolamine-binding, conserved site
Os12g0152200	Cyclin-like F-box domain containing protein.	ab initio prediction	NP_001066174.1 (RefS	F-box domain, cyclin-like
Os12g0152500	Hypothetical conserved gene.	BT039089	A2ZI44	-
Os12g0152800	Rhodanese-like domain containing protein.	AK073401	NP_001152230.1 (RefS	Dual specificity phosphatase, catalytic domain
Os12g0153200	Conserved hypothetical protein.	AK108198	A2ZI60	-
Os12g0154900	Germin-like protein 12-3, Disease resistance	Inferred from literature	Q2QXJ1	Germin
Os12g0155300	Conserved hypothetical protein.	AK060480	Q2QXI6	-
Os12g0155400	Similar to Leucine Rich Repeat family protein, expressed.	X79130	Q8S5D5	-
Os12g0155600	Non-protein coding transcript.	X06283	NONE	-
Os12g0156100	Similar to NAC-domain containing protein 90 (ANAC090).	AK109534	XP_002874067.1 (RefS	No apical meristem
Os12g0156400	Dcp1-like decapping family protein.	AK072086 ,AK099278	NP_001149166.1 (RefS	Dcp1-like decapping
Os12g0156500	Cyclin-like F-box domain containing protein.	EU969867	NP_001151212.1 (RefS	F-box domain, cyclin-like
Os12g0156900	Pentatricopeptide repeat domain containing protein.	BT018623	Q2QXH1	Pentatricopeptide repeat
Os12g0169300	Conserved hypothetical protein.	AK287997	NP_001176998.1 (RefS	-
Os12g0177800	Similar to D-mannose binding lectin family protein, expressed.	AK105397	Q2QWY4	S-locus glycoprotein
Os12g0178250	Similar to dehydration-responsive protein-related.	ab initio prediction	NP_001031109.1 (RefS	Protein of unknown function DUF248, methyltransferase putative
Os12g0178300	Similar to ankyrin-like protein.	ab initio prediction	NP_001151565.1 (RefS	Protein of unknown function DUF248, methyltransferase putative
Os12g0178600	Similar to dehydration-responsive protein-related.	ab initio prediction	NP_190676.1 (RefSeq)	Protein of unknown function DUF248, methyltransferase putative
Os12g0178633	WD40 repeat domain containing protein.	AK369075	B8BNG0	WD40 repeat
Os12g0178800	Hypothetical protein.	AK063709	longestORF	-
Os12g0179100	Hypothetical gene.	AK106975	longestORF	-
Os12g0182200	Similar to Dihydrolipoamide S-acetyltransferase.	AK099737	B4G1C9	Biotin/lipoyl attachment
Os12g0182600	Hypothetical conserved gene.	AK120295	B8BNH4	MTCP1
Os12g0189700	Twin arginine translocation signal, Tat domain containing protein.	AK287777	A9P324	Twin-arginine translocation pathway, signal sequence
Os12g0190875	Similar to H0315A08.1 protein.	FP099421	Q01I63	Ribonuclease H1, N-terminal
Os12g0191300	Similar to Phage head-tail adaptor family protein, expressed.	BT067332	Q2QWM4	Aminotransferase, class V/Cysteine desulfurase
Os12g0191400	Conserved hypothetical protein.	BT085962	NP_001183161.1 (RefS	-
Os12g0191500	Similar to Peroxidase 43 precursor (EC 1.11.1.7) (Atperox P43).	AK061040	B9V0M3	Plant peroxidase
Os12g0191901	Hypothetical gene.	CU406111	longestORF	-
Os12g0195100	Similar to amino acid permease.	EU962989	O82044	Amino acid transporter, transmembrane
Os12g0195183	Hypothetical protein.	tplb0036o08 (Wheat FLcDNA)	longestORF	-
Os12g0202700	Similar to N-methyltransferase.	CU406772	Q96565	O-methyltransferase, family 2
Os12g0203000	Similar to Cyclin-dependent protein kinase-like protein.	AK100360	Q2QW88	Protein kinase, catalytic domain
Os12g0203300	Similar to amino acid permease 1.	EU949945	NP_001149517.1 (RefS	Amino acid transporter, transmembrane
Os12g0203900	Hypothetical gene.	AK072494	longestORF	-
Os12g0207500	Similar to ATP synthase subunit beta.	ab initio prediction	Q2QW50	ATPase, F1/V1/A1 complex, alpha/beta subunit, nucleotide-binding domain
Os12g0207600	Similar to ribulose-1,5-bisphosphate carboxylase/oxygenase large subunit.	ab initio prediction	YP_654221.1 (RefSeq)	Ribulose bisphosphate carboxylase, large subunit, C-terminal
Os12g0215100	Hypothetical conserved gene.	AK109751	Q53NH3	-
Os12g0220800	Hypothetical protein.	AK108124	longestORF	-
Os12g0222800	Leucine-rich repeat, N-terminal domain containing protein.	ab initio prediction	Q2QVP4	Leucine-rich repeat
Os12g0228500	Hypothetical conserved gene.	AK287997	NP_001176998.1 (RefS	-
Os12g0233366	Non-protein coding transcript.	CT835611	NONE	-
Os12g0299650	Myb/SANT-like domain domain containing protein.	BT063151	NP_001176912.1 (RefS	Myb/SANT-like domain
Os12g0484375	Similar to H0315A08.1 protein.	FP099421	Q01I63	Ribonuclease H1, N-terminal
Os12g0510766	Non-protein coding transcript.	EU942847	NONE	-
Os12g0510900	Similar to LIM domain protein WLIM-1.	AK058220	Q2QQ10	Zinc finger, LIM-type
Os12g0511400	Similar to NB-ARC domain containing protein.	AK105287	Q2QQ03	Disease resistance protein
Os12g0511750	Nucleic acid-binding, OB-fold domain containing protein.	AK287637	NP_001175163.1 (RefS	Nucleic acid-binding, OB-fold
Os12g0520200	Similar to phenylalanine ammonia-lyase.	AK240729	Q2QPR0	Phenylalanine/histidine ammonia-lyase
Os12g0521101	Hypothetical protein.	tplb0048a09 (Wheat FLcDNA)	longestORF	-
Os12g0521300	Similar to HAT family dimerisation domain containing protein.	EU942994	Q2QPQ7	HAT dimerisation
Os12g0524201	Hypothetical conserved gene.	AK288953	B6U883	-
Os12g0524750	Similar to ribosomal protein S7.	ab initio prediction	YP_514635.1 (RefSeq)	Ribosomal protein S7
Os12g0538066	Similar to HAT family dimerisation domain containing protein.	AK122139	Q2QPA8	Zinc finger, BED-type predicted
Os12g0538800	Kelch-type beta propeller domain containing protein.	AK100645	Q2R069	Kelch repeat type 2
Os12g0538850	Non-protein coding transcript.	tplb0031a05 (Wheat FLcDNA)	NONE	-
Os12g0538900	Armadillo-type fold domain containing protein.	AK066962	NP_566820.1 (RefSeq)	Vesicle tethering protein Uso1/P115-like , head domain
Os12g0539400	Hypothetical conserved gene.	CT828588	Q2QP77	-
Os12g0541300	Similar to Respiratory burst oxidase homolog protein B.	AK318553	Q948T9	Cytochrome b245, heavy chain
Os12g0541350	Hypothetical protein.	tplb0025i04 (Wheat FLcDNA)	longestORF	-
Os12g0541500	Similar to Elongation factor Ts.	AK062564	A2ZLC1	Translation elongation factor EFTs/EF1B
Os12g0541600	Non-protein coding transcript.	AK058334	NONE	-
Os12g0541700	Similar to Rapid alkalinization factor 2.	AK103420	NP_001147163.1 (RefS	Rapid ALkalinization Factor
Os12g0564600	Zinc finger, CCHC-type domain containing protein.	AK101383	XP_002865765.1 (RefS	Zinc finger, CCHC-type
Os12g0564701	Hypothetical protein.	tplb0029h05 (Wheat FLcDNA)	longestORF	-
Os12g0564800	NB-ARC domain containing protein.	AK103886	A3CIH5	Disease resistance protein
Os12g0564866	Hypothetical protein.	tplb0040n12 (Wheat FLcDNA)	longestORF	-
Os12g0564932	Hypothetical protein.	ab initio prediction	NONE	-
Os12g0565166	Non-protein coding transcript.	tplb0040n12 (Wheat FLcDNA)	NONE	-
Os12g0565200	Conserved hypothetical protein.	ab initio prediction	NP_001067046.2 (RefS	-
Os12g0565300	Similar to Calmodulin-binding protein MPCBP.	AK073968	B6U2D5	Tetratricopeptide-like helical
Os12g0572201	Hypothetical gene.	AK068492	longestORF	-
Os12g0572400	Similar to SC35-like splicing factor SCL30, 30 kD.	AK065531	Q7XXQ1	RNA recognition motif domain
Os12g0572601	Hypothetical gene.	AK287811	longestORF	-
Os12g0572700	Exostosin-like domain containing protein.	ab initio prediction	NP_001177020.1 (RefS	Exostosin-like
Os12g0576100	Conserved hypothetical protein.	CU406037	NP_001067107.1 (RefS	-
Os12g0587116	Non-protein coding transcript.	AK358137	NONE	-
Os12g0590550	Hypothetical gene.	AK062999	longestORF	-
Os12g0592832	Hypothetical protein.	ab initio prediction	NONE	-
Os12g0594000	WD40 repeat-like domain containing protein.	AK354406	Q1EPE0	WD40 repeat
Os12g0594200	Hypothetical conserved gene.	EU942864	NP_001067177.2 (RefS	-
Os12g0594201	Hypothetical gene.	CU406459	longestORF	-
Os12g0594300	Hypothetical conserved gene.	ab initio prediction	B9GE20	Phox/Bem1p
Os12g0594600	BTB/POZ fold domain containing protein.	AK101239	A2ZMA8	SKP1 component
Os12g0594700	Similar to F-box domain containing protein.	ab initio prediction	Q2QMR2	F-box domain, Skp2-like
Os12g0594950	Hypothetical conserved gene.	AK287432	NP_001177039.1 (RefS	-
Os12g0595000	Similar to EXS, C-terminal.	AK065252	NP_001151325.1 (RefS	EXS, C-terminal
Os12g0595300	Conserved hypothetical protein.	AK106840	A3CJ25	-
Os12g0595600	Hypothetical conserved gene.	ab initio prediction	B9GE25	SKP1 component
Os12g0596401	Non-protein coding transcript.	X06283	NONE	-
Os12g0596501	Hypothetical protein.	tplb0045o13 (Wheat FLcDNA)	longestORF	-
Os12g0596600	Conserved hypothetical protein.	AK064196	B9GE28	-
Os12g0596800	Zinc finger, LIM-type domain containing protein.	AK065760	B6SPK3	Zinc finger, LIM-type
Os12g0596850	Non-protein coding transcript.	tplb0034a09 (Wheat FLcDNA)	NONE	-
Os12g0597200	Similar to MATH domain containing protein, expressed.	AK059486	Q2QMP1	-
Os12g0597400	HI0933-like protein family protein.	AK071949 ,AK099006	NP_566235.1 (RefSeq)	-
Os12g0598000	Non-protein coding transcript.	AK242434	NONE	-
Os12g0599400	Helix-loop-helix DNA-binding domain containing protein.	ab initio prediction	Q2QML8	Helix-loop-helix DNA-binding
Os12g0599550	Similar to Helix-loop-helix DNA-binding domain containing protein.	ab initio prediction	Q2QML8	-
Os12g0599800	Pectinesterase inhibitor domain containing protein.	AK111139	A2ZME3	Pectinesterase inhibitor
Os12g0599900	Tetratricopeptide-like helical domain containing protein.	AK101252	B4FG08	Tetratricopeptide TPR-1
Os12g0600000	Conserved hypothetical protein.	AK109945	NP_001067202.1 (RefS	-
Os12g0600100	Tetratricopeptide-like helical domain containing protein.	AK063560	B4FG08	Tetratricopeptide TPR-1
Os12g0600150	Hypothetical gene.	AK241829	longestORF	-
Os12g0600400	Similar to Pyridoxal kinase.	AK066514	Q69G22	Pyridoxal phosphate
Os12g0600550	Non-protein coding transcript.	AK288257	NONE	-
Os12g0601000	Similar to Leucine Rich Repeat family protein, expressed.	EU973703	Q2QMK5	F-box domain, Skp2-like
Os12g0603700	Similar to CBL-interacting protein kinase 4.	Q2QMI0	Q2QMI0	Protein kinase, catalytic domain
Os12g0604000	Non-protein coding transcript.	AK067077 ,AK067588	NONE	-
Os12g0604650	Non-protein coding transcript.	EU948222	NONE	-
Os12g0605200	KIP1-like domain containing protein.	AK102398	Q2QMG9	KIP1-like
Os12g0605400	Similar to CROC-1-like protein (Fragment).	AK068060	B6SK03	Ubiquitin-conjugating enzyme, E2

Os12g0605500	Tesmin/TSO1-like, CXC domain containing protein.	AK073942	B8BMW2	Tesmin/TSO1-like, CXC
Os12g0606900	Similar to Ulp1 protease family, C-terminal catalytic domain containing protein, expressed.	AK072659	Q2QME9	-
Os12g0607100	Similar to Eukaryotic translation initiation factor 2 gamma subunit.	AK061482	B6TNU0	Protein synthesis factor, GTP-binding
Os12g0611500	Similar to H0315A08.1 protein.	FP099421	Q01I63	Ribonuclease H1, N-terminal
Os12g0613000	Similar to NF-YA subunit.	AK065163	B6TY54	CCAAT-binding transcription factor, subunit B
Os12g0613200	Hypothetical conserved gene.	AK069599	B9GE94	SET domain
Os12g0613700	Transcriptional factor B3 family protein.	AK121703	Q2QM84	AUX/IAA protein
Os12g0613850	Similar to Galactosyltransferase family.	ab initio prediction	B6SVW0	Galectin, carbohydrate recognition domain
Os12g0614100	Similar to Lipase family protein.	BT042072	Q2QM82	Lipase, class 3
Os12g0614200	Similar to Lipase family protein.	BT042072	Q2QM81	Lipase, class 3
Os12g0614201	Conserved hypothetical protein.	AK243381	NP_001177060.1 (RefS	-
Os12g0617100	Similar to Translation initiation factor eIF-2B delta subunit (eIF-2B GDP-GTP exchange factor) (Gu	AK103148	NP_001159071.1 (RefS	Initiation factor 2B-related
Os12g0617175	Non-protein coding transcript.	tplb0051c11 (Wheat FLcDNA)	NONE	-
Os12g0621766	Hypothetical protein.	EU947100	GeneMark	-
**SH527 pedigree**				
Os01g0113150	Disease resistance protein domain containing protein.	AK367884	A2ZNH1	Disease resistance protein
Os01g0114050	Non-protein coding transcript.	EU941912	NONE	-
Os01g0121300	Conserved hypothetical protein.	AK103404	B8ACZ7	-
Os01g0121400	Hypothetical protein.	tplb0026h09 (Wheat FLcDNA)	longestORF	-
Os01g0121500	Conserved hypothetical protein.	AK107776	A2WK24	-
Os01g0133766	Non-protein coding transcript.	X02683	NONE	-
Os01g0154200	Protein of unknown function DUF674 domain containing protein.	FP098916	Q5ZCE4	Protein of unknown function DUF674
Os01g0154250	Hypothetical protein.	BT085097	longestORF	-
Os01g0154550	Non-protein coding transcript.	AK288511	NONE	-
Os01g0155200	Similar to H0525E10.8 protein.	AK252147	Q01K00	Protein kinase, catalytic domain
Os01g0155500	Protein kinase, catalytic domain containing protein.	AK099840	A2ZPF5	Protein kinase, catalytic domain
Os01g0155600	Similar to Splicing factor RSZ33.	AK071635	A6MZ96	RNA recognition motif domain
Os01g0156300	Similar to Cappuccino protein.	AK107993	A2WKV9	Protein of unknown function DUF1110
Os01g0157200	Conserved hypothetical protein.	AK108577	A2ZPG5	-
Os01g0157400	Conserved hypothetical protein.	EU974248	Q5ZCC9	-
Os01g0157700	Glycosyl transferase, family 43 domain containing protein.	AK376884	Q5ZCC5	Glycosyl transferase, family 43
Os01g0159600	Small hydrophilic plant seed protein family protein.	AK122153,AK064055	Q3I3Z0	Stress induced protein
Os01g0159800	Similar to DNA binding protein.	AK106292	NP_001152068.1	Helix-loop-helix DNA-binding
Os01g0160200	Leucine-rich repeat, N-terminal domain containing protein.	AK102533,AK073444	Q9LGL4	-
Os01g0164650	Non-protein coding transcript.	EU944689	NONE	-
Os01g0165100	Conserved hypothetical protein.	AK103011,AK059335	A2WL20	-
Os01g0165600	Similar to H0323C08.17 protein.	AK335607	Q942P3	-
Os01g0166700	Saposin family protein.	AK067805	B6T780	Saposin-like type B, 1
Os01g0170900	Conserved hypothetical protein.	AK111084	A2WL65	-
Os01g0172400	Similar to Phospholipase D alpha 1.	AK066556	B8ADH7	Phospholipase D/Transphosphatidylase
Os01g0172900	Glycoside hydrolase, family 28 domain containing protein.	ab initio prediction	B9ETD9	Glycoside hydrolase, family 28
Os01g0173000	Putative thiol-disulphide oxidoreductase DCC domain containing protein.	AK287669	GeneMark	Putative thiol-disulphide oxidoreductase DCC
Os01g0173100	Alba, DNA/RNA-binding protein family protein.	AK121114	NP_564325.1	DNA/RNA-binding protein Alba-like
Os01g0173950	Non-protein coding transcript.	tplb0011b09 (Wheat FLcDNA)	NONE	-
Os01g0176300	Pentatricopeptide repeat domain containing protein.	AK249469	NP_001042180.1	Pentatricopeptide repeat
Os01g0177150	Non-protein coding transcript.	AK288777	NONE	-
Os01g0178300	Hypothetical protein.	AK062450	GeneMark	-
Os01g0178400	Similar to Protein phosphatase 2A B'kappa subunit.	AK108850	XP_002885457.1	Protein phosphatase 2A, regulatory B subunit, B56
Os01g0178500	Similar to Auxin-responsive protein (Aux/IAA) (Fragment).	AK109373,AK060091,AK069242	Q5VRD1	-
Os01g0178700	Similar to Protein binding protein.	AK103147	B6TV66	Zinc finger, RING-type
Os01g0178775	Non-protein coding transcript.	tplb0030e06 (Wheat FLcDNA)	NONE	-
Os01g0178900	Similar to Symbiosis-related disease resistance protein (Fragment).	AK110770	Q8RVG6	-
Os01g0179000	Transferase family protein.	AK288049	B6TZV1	Transferase
Os01g0179600	UDP-glucuronosyl/UDP-glucosyltransferase family protein.	AK243646	NP_001042201.2	UDP-glucuronosyl/UDP-glucosyltransferase
Os01g0179750	Non-protein coding transcript.	BT019232	NONE	-
Os01g0181033	Similar to NADH dehydrogenase subunit 1.	AJ010976	YP_024343.1	NADH:ubiquinone oxidoreductase, subunit 1/F420H2 oxidoreductase subunit H
Os01g0182300	Lipocalin domain containing protein.	AK062827	A2WLF0	-
Os01g0186600	Conserved hypothetical protein.	AK108138	A2WLI4	-
Os01g0186900	Similar to cDNA clone:001-117-G12, full insert sequence.	AK106168	B7EXU7	-
Os01g0186950	Hypothetical protein.	tplb0058c08 (Wheat FLcDNA)	longestORF	-
Os01g0187200	Non-protein coding transcript.	AK110797	NONE	-
Os01g0193250	Hypothetical gene.	EU959976	longestORF	-
Os01g0194000	Conserved hypothetical protein.	AK071994	A2WLM7	-
Os01g0196133	Similar to H0315A08.1 protein.	FP099421	Q01I63	Ribonuclease H1, N-terminal
Os01g0224700	Flavin monooxygenase-like enzyme, Auxin biosynthesis	AK070386	Q9LG41	FAD-dependent pyridine nucleotide-disulphide oxidoreductase
Os01g0225400	Pyruvate/Phosphoenolpyruvate kinase, catalytic core domain containing protein.	AK242525	NP_001150103.1	Ketopantoate hydroxymethyltransferase
Os01g0225600	Similar to Dehydrin.	CU405989	Q5CAQ2	Late embryogenesis abundant protein, LEA-14
Os01g0233850	Hypothetical gene.	EU943630	longestORF	-
Os01g0235400	Similar to predicted protein.	AK064696	Q9ZPY7	Importin-beta, N-terminal
Os01g0250200	Conserved hypothetical protein.	ab initio prediction	A2ZQT9	-
Os01g0262000	Similar to agmatine coumaroyltransferase.	BT033950	B6SJ46	Transferase
Os01g0265700	Conserved hypothetical protein.	AK111252	A2WN70	-
Os01g0265800	RNA-binding region RNP-1 (RNA recognition motif) domain containing protein.	AK099896	NP_001149939.1	RNA recognition motif domain
Os01g0265900	Similar to translin family protein.	tplb0054b23 (Wheat FLcDNA)	NP_178473.2	Translin
Os01g0267050	Non-protein coding transcript.	AK105155	NONE	-
Os01g0267100	MT-A70 family protein.	AK065023	Q10RS1	DNA methylase, N-6 adenine-specific, conserved site
Os01g0272700	Hypothetical conserved gene.	AK248395	NP_001150380.1	Plastocyanin-like
Os01g0272800	Conserved hypothetical protein.	AK242815	B9EV58	-
Os01g0274500	Similar to Expansin-A11.	DQ062981	Q4PNY1	Expansin
Os01g0274800	R2R3-type MYB transcription factor, Sugar partitioning into anther	AK107461	Q5NBM8	SANT domain, DNA binding
Os01g0275700	Non-protein coding transcript.	EU949846	NONE	-
Os01g0275950	Hypothetical conserved gene.	ab initio prediction	NP_001042723.2	-
Os01g0276066	Non-protein coding transcript.	BT061579	NONE	-
Os01g0276200	Similar to Mitochondrial import receptor subunit TOM40.	BT040757	B6TEP3	Porin, eukaryotic type
Os01g0276400	Similar to presenilin.	AK106403	NP_001149271.1	Peptidase A22A, presenilin
Os01g0278466	Non-protein coding transcript.	CT835260	NONE	-
Os01g0278900	Similar to 50S ribosomal protein L40.	AK120429	NP_001147184.1	-
Os01g0279100	Similar to Magnesium-protoporphyrin ix monomethyl ester cyclase (Fragment).	AK061646	A6N1H0	-
Os01g0279700	Non-protein coding transcript.	AK058762	NONE	-
Os01g0281000	Cyclin-like F-box domain containing protein.	AK064952	NP_001151624.1	F-box domain, cyclin-like
Os01g0281050	Hypothetical protein.	ab initio prediction	NONE	-
Os01g0281100	Conserved hypothetical protein.	AK109672	B9EVJ4	-
Os01g0281200	Similar to Type B-like cyclin (Fragment).	AK107209	Q0JNK6-2	Cyclin, C-terminal
Os01g0281250	Hypothetical protein.	tplb0047b05 (Wheat FLcDNA)	longestORF	-
Os01g0281301	Protein of unknown function DUF1645 domain containing protein.	BT065146	Q9LG63	Protein of unknown function DUF1645
Os01g0283000	Conserved hypothetical protein.	AK073165	A2WNJ8	-
Os01g0283400	Conserved hypothetical protein.	AK119993	A2WNK1	-
Os01g0285300	Myb transcription factor domain containing protein.	AK111803	D6BV29	SANT domain, DNA binding
Os01g0286100	Helix-loop-helix DNA-binding domain containing protein.	AK102252	B9EVK4	Helix-loop-helix DNA-binding
Os01g0287400	Similar to Hydrophobic protein LTI6A (Low temperature-induced protein 6A).	AK058343	Q01L12	-
Os01g0287900	Hypothetical protein.	AK372761	longestORF	-
Os01g0290000	Similar to Cyprosin precursor (EC 3.4.23.-) (Fragment).	AK066523	NP_001152501.1	Peptidase A1
Os01g0290600	Similar to isopenicillin N epimerase.	AK366122	NP_001151142.1	Aminotransferase, class V/Cysteine desulfurase
Os01g0299200	Pentatricopeptide repeat domain containing protein.	BT084276	Q5JNP8	Pentatricopeptide repeat
Os01g0308700	Similar to H0315A08.1 protein.	FP099421	Q01I63	Ribonuclease H1, N-terminal
Os01g0324400	Hypothetical conserved gene.	AK111299	Q657G4	-
Os01g0366300	Similar to Receptor protein kinase.	AK064115	Q33B66	Protein kinase, catalytic domain
Os01g0368900	Similar to Glutaredoxin-C1.	AK107858	Q7G8Y5	Glutaredoxin
Os01g0379800	Similar to HAT family dimerisation domain containing protein.	AK122139	Q2QPA8	Zinc finger, BED-type predicted
Os01g0393100	Similar to CUC2.	AK107407	A2ZVS5	No apical meristem
Os01g0393200	Conserved hypothetical protein.	FM878729	C0PHZ5	-
Os01g0523600	Hypothetical protein.	BT019283	longestORF	-
Os01g0536200	Conserved hypothetical protein.	AK372466	Q6K2H9	-
Os01g0539900	Conserved hypothetical protein.	AK242029	B9EXE2	-
Os01g0540000	Similar to T22C5.14.	BT086321	Q9SFZ0	-

Os01g0540300	Rac-like GTP-binding protein 2.	Q68Y52	Q68Y52	Small GTPase superfamily
Os01g0544700	Hypothetical conserved gene.	AK318584	B9EWA7	-
Os01g0557200	Conserved hypothetical protein.	EU972429	Q8S0U8	-
Os01g0558700	Conserved hypothetical protein.	AK287514	NP_001172419.1	-
Os01g0558800	Similar to oxidoreductase/ transition metal ion binding protein.	AK068120	XP_002869432.1	Protein of unknown function DUF3531
Os01g0559600	Similar to Asparaginyl endopeptidase.	AK068011	Q9SSZ4	Peptidase C13, legumain
Os01g0613400	Non-protein coding transcript.	AK058952	NONE	-
Os01g0613500	Peptidase C1A, papain family protein.	AK070448	A2WSK3	Peptidase, cysteine peptidase active site
Os01g0618500	Similar to RNA helicase (Fragment).	AK067476	B7EDN0	RNA helicase, ATP-dependent, DEAD-box, conserved site
Os01g0618601	Non-protein coding transcript.	AF366447	NONE	-
Os01g0618900	Similar to polygalacturonase.	AK099329	B6TDS0	Glycoside hydrolase, family 28
Os01g0619900	Similar to DNA binding.	AK060239	NP_001118417.1	-
Os01g0629400	Similar to Ctd-phosphatase-like protein (Fragment).	AK063853	A6N1L0	NLI interacting factor
Os01g0637600	Similar to Peptide deformylase, chloroplast precursor (EC 3.5.1.88) (PDF) (Polypeptide deformylase	AK106980	Q5VNN5	Formylmethionine deformylase
Os01g0639900	Non-protein coding transcript.	AK058203	NONE	-
Os01g0641750	Hypothetical protein.	AK361892	longestORF	-
Os01g0642000	Carboxylesterase, type B family protein.	AK110911	NP_173937.2	Carboxylesterase, type B
Os01g0642100	Hypothetical conserved gene.	AK063793	B9EYA5	-
Os01g0642200	Conserved hypothetical protein.	AK105174,AK071521	B8ACD2	-
Os01g0642600	Protein of unknown function DUF617, plant family protein.	AK364722	NP_001149649.1	Protein of unknown function DUF617, plant
Os01g0642900	Nucleic acid-binding, OB-fold domain containing protein.	AK073560,AK063836	NP_001151839.1	Primosome PriB/single-strand DNA-binding
Os01g0643900	Oleosin family protein.	AK108288	B8ACE2	Oleosin
Os01g0644200	Similar to Little protein 1.	AK063634	B4UWC1	-
Os01g0645200	Hypothetical conserved gene.	AK111326	B8ACE5	-
Os01g0647000	Similar to F-box domain containing protein.	AK061541	NP_001149398.1	-
Os01g0648500	Similar to Transcription initiation factor TFIID subunit 7.	AK241160	B6UGL3	TAFII55 protein, conserved region
Os01g0648700	Bromodomain containing protein.	AK111374	Q94JA6	Bromodomain
Os01g0650501	Non-protein coding transcript.	EU942775	NONE	-
Os01g0650900	Lipase, GDSL domain containing protein.	AK375707	NP_001043727.1	Lipase, GDSL
Os01g0650950	Hypothetical protein.	tplb0048c08 (Wheat FLcDNA)	longestORF	-
Os01g0651000	Lipase, GDSL domain containing protein.	AK242032	Q8RZ48	Lipase, GDSL
Os01g0651100	Pentatricopeptide repeat domain containing protein.	AK119380	B8A6T7	Pentatricopeptide repeat
Os01g0651300	Similar to GDSL-motif lipase/hydrolase-like protein.	AK111325	NP_001151904.1	Lipase, GDSL
Os01g0651400	Lipase, GDSL domain containing protein.	AK059784	NP_001151904.1	-
Os01g0651450	Hypothetical protein.	EU954131	longestORF	-
Os01g0651500	Wax synthase domain containing protein.	EU959294	B6T2S9	Wax synthase
Os01g0652000	Similar to proline-rich family protein.	AK058938	NP_193591.2	-
Os01g0652450	Hypothetical gene.	CT834813	Q6L4J0	-
Os01g0652600	Similar to Ketol-acid reductoisomerase, chloroplast precursor (EC 1.1.1.86) (Acetohydroxy-acid redu	AK072075	B6TF69	Acetohydroxy acid isomeroreductase C-terminal
Os01g0652650	Non-protein coding transcript.	D21110	NONE	-
Os01g0652700	Similar to predicted protein.	EU967350	NP_001043739.2	Protein of unknown function DUF538
Os01g0652800	Protein of unknown function DUF231, plant domain containing protein.	AK061145,AK103547,AK104312	A2WTA8	Domain of unknown function DUF231, plant
Os01g0653500	Conserved hypothetical protein.	ab initio prediction	Q5VPB2	-
Os01g0654300	Similar to ARP2/3 complex 34 kDa subunit.	AK058593	B6TA29	Arp2/3 complex, 34kDa subunit p34-Arc
Os01g0654400	Similar to Salt tolerant correlative protein.	AK288103	E6Y2L2	-
Os01g0654450	Hypothetical gene.	EU946002	longestORF	-
Os01g0654500	Similar to NADP-isocitrate dehydrogenase.	AK061752	Q9XGU8	Isocitrate dehydrogenase NADP-dependent, eukaryotic-type
Os01g0654650	Non-protein coding transcript.	BT016882	NONE	-
Os01g0654800	Conserved hypothetical protein.	AK359861	B9EYE1	-
Os01g0655300	Similar to Trithorax 4 (Fragment).	AK062870	B3VSN3	SET domain
Os01g0655400	Hypothetical conserved gene.	AK069873	B8A6V1	-
Os01g0705400	Hypothetical conserved gene.	AK336128	A2ZX08	-
Os01g0707500	Similar to Transcription factor LAX PANICLE.	AK100208	A2WUA1	Helix-loop-helix DNA-binding
Os01g0708201	Hypothetical gene.	BT024151	longestORF	-
Os01g0708400	Non-protein coding transcript.	AK242236	NONE	-
Os01g0710100	Hypothetical conserved gene.	AK357053	NP_001044037.2	Protein of unknown function DUF761, plant
Os01g0711900	Conserved hypothetical protein.	AK108302	A2ZX51	-
Os01g0714600	Similar to cDNA clone:J023088C01, full insert sequence.	AK059855	B7EQF1	-
Os01g0718300	Similar to Brassinosteroid-insensitive 1.	AK121760	Q0ZA03	Protein kinase, catalytic domain
Os01g0720200	Non-protein coding transcript.	AK058236	NONE	-
Os01g0720700	Similar to satase isoform I.	CT830895	NP_001105082.1	-
Os01g0722550	Similar to HAT family dimerisation domain containing protein.	AK122139	Q2QPA8	Zinc finger, BED-type predicted
Os01g0723800	Hypothetical conserved gene.	AK061902	B8A926	ABC transporter, transmembrane domain
Os01g0732300	Protein of unknown function DUF623, plant domain containing protein.	AK108391	A2WUS7	Domain of unknown function DUF623
Os01g0742000	tRNA/rRNA methyltransferase, SpoU domain containing protein.	AK062779	NP_849992.1	tRNA/rRNA methyltransferase, SpoU
Os01g0745700	Hypothetical conserved gene.	AK062377	Q5JLX9	-
Os01g0748200	Conserved hypothetical protein.	AK372402	Q5JNJ4	-
Os01g0755700	Zinc finger, RING/FYVE/PHD-type domain containing protein.	AK108853	NP_001148026.1	Zinc finger, RING-type
Os01g0769100	Non-protein coding transcript.	AK242413	NONE	-
Os01g0777650	Hypothetical gene.	AK289196	longestORF	-
Os01g0791033	Similar to ribulose-1,5-bisphosphate carboxylase/oxygenase large subunit.	ab initio prediction	NP_039391.1	Ribulose bisphosphate carboxylase, large subunit, C-terminal
Os01g0793900	Conserved hypothetical protein.	AK062811	A2WVX8	-
Os01g0800200	Conserved hypothetical protein.	ab initio prediction	NP_001172605.1	-
Os01g0805750	Non-protein coding transcript.	EU951443	NONE	-
Os01g0807000	Conserved hypothetical protein.	AK109751	Q53NH3	-
Os01g0810000	Utp11 family protein.	AK102143	NP_001150340.1	Small-subunit processome, Utp11
Os01g0813350	Hypothetical gene.	EU949353	longestORF	-
Os01g0814400	Conserved hypothetical protein.	AK104661	B8ABA0	-
Os01g0814550	Non-protein coding transcript.	BT086943	NONE	-
Os01g0819100	Similar to predicted protein.	AK242625	C0LGW1	Protein kinase, catalytic domain
Os01g0823600	Conserved hypothetical protein.	AK242079	NP_001044661.1	-
Os01g0825166	Similar to Splicing coactivator subunit-like protein.	EU944053	C8TFH6	-
Os01g0825332	Similar to Splicing coactivator subunit-like protein.	EU944053	C8TFH6	-
Os01g0828900	Similar to predicted protein.	AB512496	NP_563780.1	Domain of unknown function DUF640
Os01g0829000	Thioredoxin-like fold domain containing protein.	AK065327	B6TC88	Thioredoxin-like fold
Os01g0829100	Ankyrin domain containing protein.	AK060644	A2Z5H4	Major sperm protein
Os01g0829183	Non-protein coding transcript.	X06283	NONE	-
Os01g0829266	Hypothetical conserved gene.	AK241659	A2XX31	-
Os01g0829500	Conserved hypothetical protein.	EU962098	Q941V6	-
Os01g0829600	Protein of unknown function DUF688 domain containing protein.	AK060725	Q5QLS4	Protein of unknown function DUF688
Os01g0829700	Hypothetical protein.	AK109789	longestORF	-
Os01g0830000	Similar to Plastid sufB (Fragment).	AK121100	A2WWL8	SUF system FeS cluster assembly, SufBD
Os01g0830100	Pyridine nucleotide-disulphide oxidoreductase, NAD-binding region domain containing protein.	AK069755	XP_002881079.1	Pyridine nucleotide-disulphide oxidoreductase, NAD-binding domain
Os01g0830200	Zinc finger, RING/FYVE/PHD-type domain containing protein.	AK099788,AK069312,AK073766	B6UIK4	Zinc finger, RING-type
Os01g0831250	Hypothetical gene.	BT054429	longestORF	-
Os01g0831300	Similar to Ammonium transporter.	AK109023	Q8S233	Ammonium transporter
Os01g0831900	Similar to Ammonium transporter.	AK102106	Q8S230	Ammonium transporter
Os01g0831950	Hypothetical protein.	BT054429	longestORF	-
Os01g0835500	Similar to respiratory burst oxidase protein A.	AK068400	B2D0P0	Cytochrome b245, heavy chain
Os01g0836400	Hypothetical conserved gene.	AK068741	Q5QMF1	-
Os01g0836600	ABC transporter-like domain containing protein.	AK067556	NP_181467.1	ABC transporter-like
Os01g0836800	Transmembrane receptor, eukaryota domain containing protein.	AK066629,AK098958,AK119783	NP_001150692.1	Transmembrane receptor, eukaryota
Os01g0836900	Conserved hypothetical protein.	AK064231	A2WWR9	-
Os01g0837000	Ankyrin repeat containing protein.	AK243678	A2CIR5	Ankyrin repeat
Os01g0837200	Similar to esterase/lipase/thioesterase family protein.	AK062368	NP_191078.1	-
Os01g0837300	Similar to UDP-glucuronic acid decarboxylase 1.	AK104675	NP_001151221.1	NAD-dependent epimerase/dehydratase
Os01g0837600	Similar to plant-specific domain TIGR01589 family protein.	AK108007	NP_001148858.1	Conserved hypothetical protein CHP01589, plant
Os01g0837800	Similar to metal tolerance protein.	AK061539	Q5NA18	Cation efflux protein
Os01g0837900	Similar to Protein kinase AFC1 (EC 2.7.1.-).	AK103207	NP_850695.2	Protein kinase, catalytic domain
Os01g0839200	Protein of unknown function DUF966 family protein.	AK068926	A2WWT9	Protein of unknown function DUF966
Os01g0844500	Peptidase aspartic, catalytic domain containing protein.	EU944384	NP_001044781.1	Peptidase A1
Os01g0846150	Hypothetical gene.	BT086014	longestORF	-
Os01g0849050	Non-protein coding transcript.	DQ185898	NONE	-
Os01g0853000	Conserved hypothetical protein.	AK099561	XP_002458778.1	-
Os01g0853700	Similar to MCB1 protein.	AK111988	B7ZY89	SANT domain, DNA binding

Os01g0854400	Conserved hypothetical protein.	AK108330	A2WX36	-
Os01g0855400	SANT domain, DNA binding domain containing protein.	FJ940215	NP_001044841.1	SANT domain, DNA binding
Os01g0855600	Similar to Hs1pro-1 protein.	AK099776	Q9FUH4	Hs1pro-1, C-terminal
Os01g0855700	Hypothetical protein.	AK106740	longestORF	-
Os01g0856850	Similar to ribosomal protein S7.	EU957350	NP_039458.1	Ribosomal protein S7 domain
Os01g0858350	Similar to cytochrome P450.	AK061128,AK100746	XP_002328165.1	Cytochrome P450
Os01g0858900	Glycosyl transferase, family 29 protein.	AK107493	NP_001151877.1	Glycosyl transferase, family 29
Os01g0861000	Conserved hypothetical protein.	AK058707	A2ZZT0	-
Os01g0862850	Hypothetical protein.	FP093663	longestORF	-
Os01g0863800	Protein of unknown function DUF623, plant domain containing protein.	FP100668	B6TL44	Domain of unknown function DUF623
Os01g0866600	Similar to bolA-like protein.	AK242498	NP_001148455.1	BolA protein
Os01g0868400	Hypothetical protein.	AK103345	longestORF	-
Os01g0869750	Conserved hypothetical protein.	AK288119	NP_001172656.1	-
Os01g0869900	Similar to Serine/threonine-protein kinase SAPK4.	AK060576	C8CBK4	-
Os01g0872650	Non-protein coding transcript.	CT835515	NONE	-
Os01g0875500	Similar to Beta-galactosidase (EC 3.2.1.23).	AK102715	B6U0W2	D-galactoside/L-rhamnose binding SUEL lectin domain
Os01g0876650	Hypothetical conserved gene.	CT834967	Q654G2	-
Os01g0877500	Hypothetical conserved gene.	AK058582,AK064177	A2WXJ6	Zinc finger, PHD-type
Os01g0878000	Conserved hypothetical protein.	AK104289,AK060162	A3A062	-
Os01g0878550	Hypothetical protein.	tplb0036o08 (Wheat FLcDNA)	longestORF	-
Os01g0881950	Non-protein coding transcript.	CT834826	NONE	-
Os01g0881966	Hypothetical conserved gene.	AK242526	Q5N7J0	-
Os01g0882900	Hypothetical protein.	tplb0036o08 (Wheat FLcDNA)	longestORF	-
Os01g0883850	Hypothetical protein.	DQ334868	longestORF	-
Os01g0884350	Hypothetical protein.	FP093663	longestORF	-
Os01g0884450	Non-protein coding transcript.	BT084688	NONE	-
Os01g0884850	Hypothetical protein.	BT085496	longestORF	-
Os01g0884900	Similar to DNA glycosylase.	AK063875	XP_003057876.1	SNARE associated Golgi protein
Os01g0885500	Hypothetical conserved gene.	AK071788,AK106609	Q5N8H2	-
Os01g0886600	Similar to CLP protease regulatory subunit CLPX precursor.	AK070098	B7FAD8	ATPase, AAA+ type, core
Os01g0887100	Similar to 4-(Cytidine 5'-diphospho)-2-C-methyl-D-erythritol synthase.	AK240934	A4PIB9	4-diphosphocytidyl-2C-methyl-D-erythritol synthase
Os01g0890001	Hypothetical conserved gene.	AK242501	B9EVA1	-
Os01g0890050	Hypothetical protein.	tplb0028c09 (Wheat FLcDNA)	longestORF	-
Os01g0890100	Similar to S-domain class receptor-like kinase3.	tplb0056i22 (Wheat FLcDNA)	NP_001105655.1	Protein kinase, catalytic domain
Os01g0890500	Conserved hypothetical protein.	ab initio prediction	Q5N832	-
Os01g0892800	Similar to Ankyrin-kinase.	AK070097	Q8SAE5	Protein kinase, catalytic domain
Os01g0894075	Hypothetical protein.	tplb0043a14 (Wheat FLcDNA)	longestORF	-
Os01g0894100	Similar to Transposase (Fragment).	AK111398	NP_001176912.1	-
Os01g0895200	DOMON related domain containing protein.	AK063282	A3A0H2	Cytochrome b561, eukaryote
Os01g0900900	Ovarian tumour, otubain domain containing protein.	AK120577	NP_001078307.1	Ovarian tumour, otubain
Os01g0901101	Non-protein coding transcript.	BT084794	NONE	-
Os01g0916100	Similar to loricrin.	AK109173	NP_001147500.1	-
Os01g0916300	Similar to PQBP-1 protein (Nuclear protein containing a WW domain) (Npw38) (JM26 protein).	AK063922	NP_001147059.1	WW/Rsp5/WWP
Os01g0916800	Similar to predicted protein.	ab initio prediction	NP_001045197.2	THO complex, subunitTHOC2, C-terminal
Os01g0917300	Similar to Cysteine-rich peptide.	AK240939	D7NXT9	-
Os01g0920200	Similar to E(Y)2 homolog (DC6) (Enhancer of yellow 2 homolog).	AK120182	B6T6J7	Transcription factor, enhancer of yellow 2
Os01g0920400	Hypothetical conserved gene.	AK063897	B8A8F0	-
Os01g0920450	Conserved hypothetical protein.	ab initio prediction	B9EVX1	-
Os01g0921300	Exostosin-like family protein.	AK119614,AK106015	NP_001152546.1	Exostosin-like
Os01g0921450	Hypothetical protein.	AK374499	longestORF	-
Os01g0921550	Hypothetical protein.	ab initio prediction	NONE	-
Os01g0921800	Tetratricopeptide-like helical domain containing protein.	AK334012	XP_002887115.1	Zinc finger, MYND-type
Os01g0922000	Hypothetical protein.	AK073034	longestORF	-
Os01g0922700	Conserved hypothetical protein.	AK287528	NP_001172712.1	-
Os01g0923200	Similar to AT.I.24-6 protein (Fragment).	AK060288	Q96310	Protein of unknown function DUF3339
Os01g0923600	Hypothetical conserved gene.	AK059687	B8A8F8	IQ motif, EF-hand binding site
Os01g0923700	Similar to Histidine kinase.	AK121135	A1A696	-
Os01g0923750	Hypothetical gene.	BT086966	longestORF	-
Os01g0924100	Non-protein coding transcript.	AK062847	NONE	-
Os01g0924500	Non-protein coding transcript.	AK060236	NONE	-
Os01g0927900	Similar to Aspartate kinase precursor (EC 2.7.2.4).	AK073189	B8A8I6	Aspartate/glutamate/uridylate kinase
Os01g0927950	Hypothetical gene.	BT068036	longestORF	-
Os01g0928000	Similar to Transcription factor ICE1 (Inducer of CBF expression 1) (Basic helix- loop-helix protein 1	AK102594	B6UB60	Helix-loop-helix DNA-binding
Os01g0929000	Similar to aminopeptidase.	AK073334	NP_196898.5	Protein of unknown function DUF3754
Os01g0929100	Similar to predicted protein.	AK060605	Q5JK31	-
Os01g0929800	Hypothetical gene.	CT837487	GeneMark	-
Os01g0942900	Similar to cDNA clone:J023028M23, full insert sequence.	AK243304	B7E3R6	Ankyrin repeat
Os01g0943800	Similar to cDNA, clone: J100054L12, full insert sequence.	AK059510	B7FA83	Ankyrin repeat
Os01g0947500	Conserved hypothetical protein.	Z93114	F2DK87	-
Os01g0947767	Hypothetical gene.	CT828816	longestORF	-
Os01g0947833	Conserved hypothetical protein.	AK062969	NP_001045425.1	-
Os01g0958000	Similar to Cyclin-dependent kinase C-2.	AK109352	Q5JK68	Protein kinase, catalytic domain
Os01g0969400	Similar to cation proton exchanger.	BT086324	XP_002310518.1	-
Os01g0970400	Eukaryotic translation initiation factor 4E-1 (eIF4E-1) (eIF-4E-1) (mRNA cap-binding protein) (eIF-	AK069207,AK099169	NP_001149588.1	Translation Initiation factor eIF- 4e
Os01g0970600	Similar to ATP binding protein.	AK288993	NP_001147746.1	Helicase, C-terminal
Os01g0970700	Peptidase M48, Ste24p family protein.	AK098940	B6TA12	Peptidase M48
Os01g0971000	Conserved hypothetical protein.	AK061660,AK120145	A2WZJ6	-
Os01g0971100	Drought induced 19 family protein.	AK069516	Q5JME8	Drought induced 19/ RING finger protein 114
Os01g0971200	Similar to Lysine ketoglutarate reductase trans-splicing related 1.	AK064075	B4FSQ8	Protein of unknown function DUF707
Os01g0971301	Non-protein coding transcript.	BT016843	NONE	-
Os01g0971600	Similar to Sn-glycerol-3-phosphate dehydrogenase (Fragment).	AK070366	NP_001150493.1	Glycerol-3-phosphate dehydrogenase, NAD-dependent, C-terminal
Os02g0130800	F-box domain, cyclin-like domain containing protein.	EU975717	B9F2A0	F-box domain, cyclin-like
Os02g0130900	Ubiquitin-conjugating enzyme E2C-binding protein domain containing protein.	AK102856	A2X0I7	Ubiquitin-conjugating enzyme E2C-binding protein
Os02g0131000	Conserved hypothetical protein.	AK062905	A2X0I8	-
Os02g0131050	Hypothetical gene.	AK242812	longestORF	-
Os02g0131100	Hypothetical conserved gene.	AK105854	B9F2A1	-
Os02g0131200	Similar to RNA-binding region RNP-1 (RNA recognition motif).	AK069931	A3A2T3	Glycosyl transferase, family 3
Os02g0131300	Non-protein coding transcript.	AK061919	NONE	-
Os02g0131400	Beta-D-glucan exohydrolase, isoenzyme ExoII (EC 3.2.1.58).	AK363981	NP_001130296.1	Glycoside hydrolase, family 3, N-terminal
Os02g0131500	Hypothetical protein.	tplb0060e05 (Wheat FLcDNA)	longestORF	-
Os02g0131600	Similar to Mitochondrial import receptor subunit TOM22 homolog (Translocase of outer membrane	CT828266	B6U2X6	Mitochondrial outer membrane translocase complex, subunit Tom22, plant
Os02g0131850	Hypothetical gene.	BT086902	longestORF	-
Os02g0132100	Pentatricopeptide repeat domain containing protein.	ab initio prediction	NP_001045795.1	Pentatricopeptide repeat
Os02g0132300	Similar to Erythrocyte membrane protein PFEMP3 (Fragment).	AK070078	NP_001151550.1	Zinc finger, RING-CH-type
Os02g0132950	Hypothetical gene.	AK241678	longestORF	-
Os02g0133000	Cyclin-T1-1.	Q0E474	Q0E474	Cyclin, N-terminal
Os02g0133100	Similar to Phosphatidylinositol transfer-like protein III.	AK060180	Q94FN1	Cellular retinaldehyde-binding/triple function, C-terminal
Os02g0133300	Similar to SMC3 protein.	AK105135	Q8GU54	RecF/RecN/SMC
Os02g0133800	Proteasome subunit alpha type 1 (EC 3.4.25.1) (20S proteasome alpha subunit F) (20S proteasome s	AK103646,AK109376	B8AHG2	Proteasome, alpha-subunit, conserved site
Os02g0133900	Protein of unknown function DUF829, eukaryotic family protein.	AK107180	NP_001130250.1	Protein of unknown function DUF829, TMEM53
Os02g0134000	Similar to bifunctional coenzyme A synthase.	AK355133	NP_001147323.1	-
Os02g0134100	Hypothetical gene.	AK069055	longestORF	-
Os02g0134200	Protein of unknown function DUF1645 family protein.	AK108331	A2X0L4	Protein of unknown function DUF1645
Os02g0134400	Fumarate reductase/succinate dehydrogenase flavoprotein, C-terminal domain containing protein.	AK072394	B6U0K6	Fumarate reductase/succinate dehydrogenase flavoprotein, N-terminal
Os02g0135000	Conserved hypothetical protein.	AK069693	A2X0L9	-
Os02g0135200	Similar to Mitogen-activated protein kinase 13.	CT836561	Q0E459	Protein kinase, catalytic domain
Os02g0135250	Hypothetical protein.	tplb0059e09 (Wheat FLcDNA)	longestORF	-
Os02g0135300	Similar to Wee1-like protein kinase.	AK372043	Q6Z829	Protein kinase, catalytic domain
Os02g0135600	Conserved hypothetical protein.	AK069843	A2X0M4	-
Os02g0135700	DNA polymerase V family protein.	AK100570	B9F2L5	DNA polymerase V
Os02g0135850	Hypothetical conserved gene.	ab initio prediction	B9F2L6	Ubiquitin system component Cue
Os02g0135900	Similar to 5'-AMP-activated protein kinase beta-1 subunit-related.	AK072017	NP_568573.2	-
Os02g0135950	Non-protein coding transcript.	BT016931	NONE	-
Os02g0136150	Hypothetical gene.	CT835435	longestORF	-
Os02g0136850	Non-protein coding transcript.	CU406396	NONE	-

Os02g0136900	Protein kinase, core domain containing protein.	AK111638	B8AHD4	Protein kinase, catalytic domain
Os02g0136966	Hypothetical gene.	AK288412	longestORF	-
Os02g0137000	WD40 repeat-like domain containing protein.	AK111719	XP_002875574.1	WD40 repeat
Os02g0137200	Similar to 50S ribosomal protein L3-1, chloroplast precursor.	AK099849	B6UF84	Ribosomal protein L3
Os02g0137400	Similar to Spliceosomal-like protein.	AK068480	Q9LD60	Cleavage/polyadenylation specificity factor, A subunit, C-terminal
Os02g0137450	Conserved hypothetical protein.	AK369368	B9F2M2	-
Os02g0137600	Bacterial methyltransferase family protein.	AK102168	NP_196652.1	S-adenosyl-L-methionine-dependent methyltransferase, MraW
Os02g0138200	RNA recognition motif, RNP-1 domain containing protein.	AK064810	A0SPK3	RNA recognition motif domain
Os02g0152500	Non-protein coding transcript.	AK062072	NONE	-
Os02g0156800	Leucine-rich repeat-containing N-terminal, type 2 domain containing protein.	AK067358	B9F2U1	Leucine-rich repeat-containing N-terminal, type 2
Os02g0161000	Similar to Serine/threonine protein kinase-like protein.	AK111660	Q6H7U5	Protein kinase, catalytic domain
Os02g0161100	Actin-binding FH2 domain containing protein.	AK072377	Q6H7U3	Actin-binding FH2/DRF autoregulatory
Os02g0161200	Zinc finger, CCCH-type domain containing protein.	AK065198,AK122103	B6TAM4	Zinc finger, CCCH-type
Os02g0161251	Hypothetical gene.	AK288500	longestORF	-
Os02g0161300	Similar to Mot1 (Fragment).	AK070938	C6ERB4	Domain of unknown function DUF3535
Os02g0161900	Similar to polyubiquitin containing 7 ubiquitin monomers.	AK101547,AK061956	NP_001147027.1	Ubiquitin
Os02g0162050	Non-protein coding transcript.	BT016940	NONE	-
Os02g0162150	Hypothetical protein.	ab initio prediction	NONE	-
Os02g0162600	Conserved hypothetical protein.	AK242133	NP_001045983.1	-
Os02g0162783	Non-protein coding transcript.	EU943604	NONE	-
Os02g0163533	Conserved hypothetical protein.	AK241536	NP_001172814.1	-
Os02g0164800	Zinc finger, C2H2-type domain containing protein.	AK062817	NP_175482.1	-
Os02g0164900	Similar to Auxin response factor 3.	AK070569	Q6H6V4	AUX/IAA protein
Os02g0165000	Zinc finger, RING/FYVE/PHD-type domain containing protein.	AK069562	B8AI80	Zinc finger, RING-CH-type
Os02g0165100	Protein kinase, core domain containing protein.	AK066645	NP_001148145.1	Protein kinase, catalytic domain
Os02g0165800	Conserved hypothetical protein.	AK106983	A2X1A7	-
Os02g0166501	Hypothetical conserved gene.	CT835482	Q6H4W8	-
Os02g0166600	Hypothetical protein.	AK069371	longestORF	-
Os02g0166800	Protein of unknown function DUF640 domain containing protein.	AK106854	D1MYE3	Domain of unknown function DUF640
Os02g0166875	Hypothetical protein.	EU970153	longestORF	-
Os02g0166950	Hypothetical gene.	AK243419	longestORF	-
Os02g0167000	Similar to holocarboxylase synthetase 1.	AK375247	XP_002878877.1	Biotin protein ligase, C-terminal
Os02g0167100	Similar to 3-mercaptopyruvate sulfurtransferase precursor (EC 2.8.1.1).	AK066425	Q94C43	Thiosulphate sulfurtransferase, conserved site
Os02g0167200	Pentatricopeptide repeat domain containing protein.	AK106491	Q6H4W1	Pentatricopeptide repeat
Os02g0167300	Similar to Tubulin beta-5 chain.	AK061659,AK119164	P46265	Tubulin
Os02g0167366	Hypothetical protein.	tplb0045b10 (Wheat FLcDNA)	longestORF	-
Os02g0167400	Non-protein coding transcript.	X06283	NONE	-
Os02g0167500	RNA recognition motif domain domain containing protein.	AK069161	B9F385	RNA recognition motif domain
Os02g0167600	Hypothetical gene.	AK242059	longestORF	-
Os02g0167850	Non-protein coding transcript.	CT835346	NONE	-
Os02g0168100	Similar to 4-hydroxyphenylpyruvate dioxygenase.	AK058890	O48604	Glyoxalase/fosfomycin resistance/dioxygenase
Os02g0168600	Ovarian tumour, otubain domain containing protein.	AK067291	B6U3W2	Ovarian tumour, otubain
Os02g0168700	Peptidyl-prolyl cis-trans isomerase, FKBP-type domain containing protein.	AK061562	NP_001148684.1	Peptidyl-prolyl cis-trans isomerase, FKBP-type, domain
Os02g0169000	Similar to T6H22.2 protein.	AK101628	Q9SGU5	-
Os02g0169151	Hypothetical gene.	BT016355	longestORF	-
Os02g0169275	Non-protein coding transcript.	FP098893	NONE	-
Os02g0169300	Similar to Phosphoglycerate kinase, cytosolic (EC 2.7.2.3).	AK070041	B8AIH2	Phosphoglycerate kinase
Os02g0169400	Similar to Argonaute 4 protein.	AK106247	Q6H6C3	Argonaute/Dicer protein, PAZ
Os02g0169450	Non-protein coding transcript.	BT016774	NONE	-
Os02g0169900	Inositol monophosphatase family protein.	AK105887,AK070107	NP_564376.1	Inositol monophosphatase
Os02g0170000	Conserved hypothetical protein.	AK065022	A3A3K8	-
Os02g0175800	Similar to fiber protein Fb15.	AK120215	NP_001150164.1	-
Os02g0175850	Non-protein coding transcript.	BT017226	NONE	-
Os02g0177666	Hypothetical protein.	BT017473	longestORF	-
Os02g0180400	GCN5-related N-acetyltransferase (GNAT) domain domain containing protein.	AK355812	Q6H820	GCN5-related N-acetyltransferase
Os02g0204000	Tetratricopeptide-like helical domain containing protein.	AK066727	NP_001147153.1	Tetratricopeptide-like helical
Os02g0213100	Conserved hypothetical protein.	AK062445	Q6H826	-
Os02g0220050	Hypothetical protein.	AK375091	longestORF	-
Os02g0226300	Mitochondrial carrier protein domain containing protein.	ab initio prediction	B8AEI5	Mitochondrial carrier protein
Os02g0226350	Non-protein coding transcript.	AK242418	NONE	-
Os02g0258000	Hypothetical conserved gene.	CT835450	Q6ETP6	-
Os02g0258200	Similar to high mobility group family.	AK070270	XP_002303636.1	High mobility group, HMG1/HMG2
Os02g0258250	Hypothetical conserved gene.	CT837539	XP_002299486.1	High mobility group, HMG1/HMG2
Os02g0258300	Similar to extra-large G-protein-related.	AK104067	NP_192018.1	-
Os02g0260800	Non-protein coding transcript.	EU941912	NONE	-
Os02g0260900	Similar to H0315A08.1 protein.	FP099421	Q01I63	Ribonuclease H1, N-terminal
Os02g0268400	Similar to Expansin-A23.	DQ061064	Q4PR43	Expansin
Os02g0268500	Hypothetical protein.	BT064922	longestORF	-
Os02g0303000	Hypothetical gene.	AK111330	longestORF	-
Os02g0305950	Similar to calmodulin binding protein.	tplb0019a16 (Wheat FLcDNA)	NP_001149909.1	Auxin responsive SAUR protein
Os02g0306801	Hypothetical gene.	EU943056	longestORF	-
Os02g0306900	Similar to DnaJ-like protein.	AK100011,AK070441	A2Q505	Heat shock protein DnaJ, N-terminal
Os02g0508650	Non-protein coding transcript.	FP094256	NONE	-
Os02g0508801	Similar to predicted protein.	EU959920	Q6K2G2	Protein of unknown function DUF3339
Os02g0519900	Similar to H0613H07.5 protein.	AK060953	Q01MK8	Translation elongation factor EFG/EF2, C-terminal
Os02g0521300	C2 domain containing protein.	AK120851	NP_001147038.1	C2 calcium-dependent membrane targeting
Os02g0521366	Similar to HAT family dimerisation domain containing protein.	tplb0053n06 (Wheat FLcDNA)	Q7XE06	Zinc finger, BED-type predicted
Os02g0521432	Hypothetical protein.	tplb0030l08 (Wheat FLcDNA)	longestORF	-
Os02g0521500	Peptidase C65, otubain domain containing protein.	AK335298	B9F0A6	Peptidase C65, otubain
Os02g0521600	Ovarian tumour, otubain domain containing protein.	AK362134	B9F0A7	Ovarian tumour, otubain
Os02g0530600	Similar to Poly.	AK105277	B7ETH1	Poly
Os02g0531200	Bile acid:sodium symporter family protein.	AK064103	B6TYA0	Bile acid:sodium symporter
Os02g0531300	Hypothetical protein.	AK068180	longestORF	-
Os02g0531450	Conserved hypothetical protein.	AK242157	NP_001173005.1	-
Os02g0549467	Non-protein coding transcript.	EU947769	NONE	-
Os02g0557800	A-type response regulator, Cytokinin signaling	AK070645,AB249662,BR000312	Q6YVX7	Signal transduction response regulator, receiver domain
Os02g0562300	Hypothetical conserved gene.	AK104737,AK061441	B8ADY6	Calmodulin binding protein-like
Os02g0562350	Non-protein coding transcript.	EU949450	NONE	-
Os02g0563000	Hypothetical conserved gene.	BT083559	Q6ZGB6	F-box domain, cyclin-like
Os02g0564200	Conserved hypothetical protein.	AK072445	A2X661	-
Os02g0564300	Conserved hypothetical protein.	AK242358	NP_001047159.1	-
Os02g0564400	Similar to CLPX (Clp protease regulatory subunit X)%3B ATPase.	AK243408	E5GBA0	ATPase, AAA+ type, core
Os02g0564500	SFT2-like family protein.	AK103450	B6TQJ1	Vesicle transport protein, Got1/SFT2-like
Os02g0564600	Late embryogenesis abundant protein 3 family protein.	AK318610	NP_001147317.1	Late embryogenesis abundant protein, LEA-5
Os02g0565000	Homeodomain-like containing protein.	AK120665	NP_001151900.1	-
Os02g0565500	Similar to Pto kinase interactor 1.	AK072522	Q709M1	Protein kinase, catalytic domain
Os02g0565600	Similar to Homeodomain leucine zipper protein (Fragment).	AK064118	Q0E0A6	Homeobox
Os02g0567000	Conserved hypothetical protein.	AK068282	B8AEC4	-
Os02g0567200	Protein phosphatase 2C domain containing protein.	AK069840	NP_001151313.1	Protein phosphatase 2C-like
Os02g0567450	Hypothetical gene.	BT085317	GeneMark	-
Os02g0568600	Hypothetical conserved gene.	ab initio prediction	Q6YTF9	-
Os02g0583300	Similar to OSIGBa0132O24.3 protein.	EU951279	Q01MB4	-
Os02g0583500	Transposase, Ptta/En/Spm, plant domain containing protein.	EU976982	NP_001047258.2	Transposase, Ptta/En/Spm, plant
Os02g0585200	Heavy metal transport/detoxification protein domain containing protein.	AK287653	NP_001152411.1	Heavy metal-associated domain, HMA
Os02g0587600	Conserved hypothetical protein.	ab initio prediction	NP_001047273.1	-
Os02g0587800	Virulence factor, pectin lyase fold family protein.	AK243611	NP_001173047.1	-
Os02g0590200	Lecithin:cholesterol/phospholipid:diacylglycerol acyltransferase domain containing protein.	AK241245	NP_001047281.2	Lecithin:cholesterol/phospholipid:diacylglycerol acyltransferase
Os02g0591850	Non-protein coding transcript.	AK242359	NONE	-
Os02g0591900	Protein kinase, catalytic domain domain containing protein.	AK060750	Q69L76	Protein kinase, catalytic domain
Os02g0592000	Similar to OSIGBa0106G07.8 protein.	AK361746	Q01IX6	Oxoglutarate/iron-dependent oxygenase
Os02g0592300	Similar to predicted protein.	AK073685	NP_567236.1	DNA mismatch repair protein
Os02g0592500	Folate receptor, conserved region domain containing protein.	AK070526	B9F0U2	Folate receptor-like
Os02g0592600	Phospholipase C, phosphatidylinositol-specific, Y domain domain containing protein.	AK099683	A2WUV9	Phospholipase C, phosphatidylinositol-specific, Y domain
Os02g0592700	Conserved hypothetical protein.	AK107229	A2X6P0	-
Os02g0593400	Conserved hypothetical protein.	AK064226	XP_002454106.1	-

Os02g0593500	Phosphate transporter family protein.	AK067498	Q7YME0	Phosphate transporter
Os02g0593550	Hypothetical gene.	EU972267	longestORF	-
Os02g0593600	Leucine-rich repeat, typical subtype containing protein.	AK069976	Q01IY1	Leucine-rich repeat
Os02g0593700	Similar to TA4 protein (Fragment).	AK072838	B8K1X5	Phox/Bem1p
Os02g0593900	Methyltransferase-related domain containing protein.	AK288086	B6TN81	Methyltransferase-related
Os02g0594100	Similar to Protein kinase ATN1.	AK067412	Q7XJ65	Protein kinase, catalytic domain
Os02g0598500	Protein phosphatase 2C family protein.	AK106903	NP_001047333.1	Protein phosphatase 2C, manganese/magnesium aspartate binding site
Os02g0598800	Telomere length regulation protein, conserved domain domain containing protein.	AK372629	NP_001047336.2	Telomere length regulation protein, conserved domain
Os02g0599100	Hypothetical conserved gene.	tplb0009a09 (Wheat FLcDNA)	Q6K1U1	Telomere length regulation protein, conserved domain
Os02g0599150	Hypothetical conserved gene.	AK106903	Q6K1U4	Protein phosphatase 2C, manganese/magnesium aspartate binding site
Os02g0644400	Similar to Kinesin heavy chain (Fragment).	ab initio prediction	Q93XG3	Kinesin, motor domain
Os02g0663900	Hypothetical conserved gene.	AK064204	Q6EUH6	Pentatricopeptide repeat
Os02g0665250	Conserved hypothetical protein.	AK242407	NP_001173107.1	-
Os02g0666250	Hypothetical gene.	EU947829	longestORF	-
Os02g0666550	Hypothetical protein.	ab initio prediction	NONE	-
Os02g0667100	Conserved hypothetical protein.	AK370258	NP_001047679.1	-
Os02g0668000	Hypothetical protein.	AK108411	GeneMark	-
Os02g0668100	Similar to Geranylgeranyl-diphosphate synthase.	AK111401	A9ZN21	Polyprenyl synthetase
Os02g0668900	Similar to H0211B05.3 protein.	ab initio prediction	Q01JA9	Lipase, GDSL
Os02g0669100	Dehydrin family protein.	AK070197	B1NEV7	Dehydrin
Os02g0672400	Sugar transporter, conserved site domain containing protein.	AK064365	B8AGF1	-
Os02g0679200	Pentatricopeptide repeat domain containing protein.	AK110789	B8AGT4	Pentatricopeptide repeat
Os02g0679700	Protein of unknown function DUF623, plant domain containing protein.	AK108178	B8AGP6	Domain of unknown function DUF623
Os02g0680500	Pentatricopeptide repeat domain containing protein.	AK106401	B8AGP8	Pentatricopeptide repeat
Os02g0681100	Protein of unknown function DUF604 family protein.	AK100584	Q01KI0	Protein of unknown function DUF604
Os02g0683100	Hypothetical protein.	tplb0021g13 (Wheat FLcDNA)	longestORF	-
Os02g0683500	Similar to DNA-binding protein RAV1.	AK109908	D9MNL6	Transcriptional factor B3
Os02g0684300	Nucleoporin protein Ndc1-Nup domain containing protein.	AK108514	A3AA67	Nucleoporin protein Ndc1-Nup
Os02g0685350	Hypothetical gene.	EU951957	longestORF	-
Os02g0689550	Hypothetical protein.	tplb0058j15 (Wheat FLcDNA)	longestORF	-
Os02g0695900	Similar to oligopeptide transporter 4.	AK062910	NP_001152089.1	Oligopeptide transporter OPT superfamily
Os02g0696200	Hypothetical protein.	AK353783	longestORF	-
Os02g0697400	Non-protein coding transcript.	AK060627	NONE	-
Os02g0704262	Conserved hypothetical protein.	CT835588	B9GCT9	-
Os02g0715300	Conserved hypothetical protein.	AK059816	A2X8X5	-
Os02g0717550	Non-protein coding transcript.	BT018944	NONE	-
Os02g0718100	Non-protein coding transcript.	AK109216	NONE	-
Os02g0720000	Conserved hypothetical protein.	AK108806	Q6ZI16	-
Os02g0720100	Cupredoxin domain containing protein.	AK063724	A3AAS9	Plastocyanin-like
Os02g0720250	Non-protein coding transcript.	tplb0026b06 (Wheat FLcDNA)	NONE	-
Os02g0720500	Peptidase A1 domain containing protein.	AK108932	B6SL29	Peptidase A1
Os02g0720900	Similar to Aspartic proteinase nepenthesin-1.	AK072228	B6TP02	Peptidase A1
Os02g0721000	Hypothetical protein.	AK108039	longestORF	-
Os02g0721100	Similar to E2 ubiquitin-conjugating enzyme UbcH5B (Fragment).	AK108167	A2X916	Ubiquitin-conjugating enzyme, E2
Os02g0725300	Similar to cDNA clone:J033042D19, full insert sequence.	AK111344	B7ERT8	-
Os02g0725600	tRNA (guanine-N1-)-methyltransferase, eukaryotic domain containing protein.	AK058341	A2Q1R6	tRNA
Os02g0726300	Similar to AOBP (Ascorbate oxidase promoter-binding protein).	AK101321,AK100892	Q58FK8	Zinc finger, Dof-type
Os02g0730900	Pentatricopeptide repeat domain containing protein.	tplb0054c14 (Wheat FLcDNA)	NP_001048017.1	Pentatricopeptide repeat
Os02g0731600	Similar to Threonine endopeptidase.	AK058271	B6TCN7	-
Os02g0735900	Zinc finger, RING/FYVE/PHD-type domain containing protein.	AK072424	B6THV3	Zinc finger, RING-type
Os02g0736700	Conserved hypothetical protein.	EU945911	NP_001048056.1	-
Os02g0736800	Conserved hypothetical protein.	AK073311	B8AI34	-
Os02g0737401	Hypothetical conserved gene.	AK241122	Q6Z733	-
Os02g0738100	Conserved hypothetical protein.	AK241905	NP_001173146.1	-
Os02g0744150	Hypothetical gene.	BT016845	longestORF	-
Os02g0745250	Similar to NAC domain class transcription factor.	AY742218	D9ZJ98	No apical meristem
Os02g0745300	Conserved hypothetical protein.	ab initio prediction	Q6ZGU0	-
Os02g0748300	Similar to Kelch motif family protein.	AK100919	B6SXJ9	Kelch repeat type 1
Os02g0750400	Pentatricopeptide repeat domain containing protein.	AK059134	A3ABE1	Pentatricopeptide repeat
Os02g0753100	Non-protein coding transcript.	BT086933	NONE	-
Os02g0753800	Similar to Annexin p35.	AK101787	A2X9Q4	Annexin
Os02g0755450	Hypothetical gene.	EU941442	longestORF	-
Os02g0762400	Cyclin-dependent kinase inhibitor family protein.	AK103084	Q6Z6G5	Cyclin-dependent kinase inhibitor
Os02g0768800	Similar to predicted protein.	AK063192	E5GCG2	-
Os02g0768950	Non-protein coding transcript.	CU405652	NONE	-
Os02g0769100	Auxin responsive SAUR protein family protein.	AK107987	B6SNK6	Auxin responsive SAUR protein
Os02g0785000	Glycosyl transferase, family 31 protein.	AK103670	NP_200558.1	Glycosyl transferase, family 31
Os02g0787000	Allergen V5/Tpx-1 related family protein.	ab initio prediction	NP_001048338.1	Allergen V5/Tpx-1-related
Os02g0796750	Non-protein coding transcript.	EU943327	NONE	-
Os02g0800500	Similar to Cyclin-B1-2.	AK099813	Q0DWQ7	Cyclin, N-terminal
Os02g0801200	Similar to Cyclin-B1-2.	AK069086	Q0DWQ7	-
Os02g0801255	Similar to cDNA clone:J013118P06, full insert sequence.	ab initio prediction	B7EE20	-
Os02g0801300	Similar to Cyclin-B1-2.	AK069086	Q0DWQ7	-
Os02g0801450	Similar to OSJNba0093F12.23 protein.	AK065428	Q5JQR9	-
Os02g0811101	Conserved hypothetical protein.	FP095473	NP_001147425.1	-
Os02g0813183	Hypothetical protein.	tplb0043a14 (Wheat FLcDNA)	longestORF	-
Os02g0813200	Myb/SANT-like domain domain containing protein.	EU975340	NP_001048483.2	Myb/SANT-like domain
Os02g0814700	Similar to Ribosomal Pr 117 (Fragment).	AK121994	Q7X9K1	Ribosomal protein L14b/L23e
Os02g0814900	Similar to nicotinamide-nucleotide adenylyltransferase 1.	ab initio prediction	NP_001150381.1	Cytidylyltransferase
Os02g0823500	Non-protein coding transcript.	U16257	NONE	-
Os02g0824400	Conserved hypothetical protein.	AK121390	B8AF55	-
Os02g0824500	Similar to Remorin.	AK111296	NP_001159012.1	Remorin, C-terminal
Os02g0824700	Similar to Vacuolar ATP synthase subunit F (EC 3.6.3.14) (V-ATPase F subunit) (Vacuolar proton p	AK240995	A2XQ09	ATPase, V1 complex, subunit F, eukaryotic
Os02g0826533	Non-protein coding transcript.	tplb0033e11 (Wheat FLcDNA)	NONE	-
Os02g0826566	F-box domain, cyclin-like domain containing protein.	AK367617	B8AF61	F-box domain, cyclin-like
Os02g0826700	F-box domain, cyclin-like domain containing protein.	ab initio prediction	Q6K7R1	F-box domain, cyclin-like
Os02g0827200	Similar to Sucrose transporter.	AK065430	Q6YK44	Major facilitator superfamily
Os02g0827400	Similar to predicted protein.	CT835495	NP_001048593.1	Protein of unknown function DUF789
Os02g0828300	Conserved hypothetical protein.	AK108744	B8BG05	-
Os02g0828400	Conserved hypothetical protein.	AK107588	Q6ZAX3	-
Os03g0100010	Conserved hypothetical protein.	AK333943	A2XBE9	-
Os03g0100050	Inner nuclear membrane protein MAN1 domain containing protein.	AK066383	Q10T70	Inner nuclear membrane protein MAN1
Os03g0100225	Non-protein coding transcript.	EU949846	NONE	-
Os03g0100300	Peptidyl-prolyl cis-trans isomerase, cyclophilin-type domain containing protein.	AK106235	NP_567522.5	Peptidyl-prolyl cis-trans isomerase, cyclophilin-type
Os03g0100400	Similar to DNA repair endonuclease UVH1 (EC 3.1.-.-) (Ultraviolet hypersensitive 1) (AtRAD1) (D	AK068556,AK121880	NP_198931.1	ERCC4 domain
Os03g0101100	Similar to Palmitoyl-protein thioesterase-like.	AK120839	Q10T54	Palmitoyl protein thioesterase
Os03g0101200	Similar to U-box domain containing protein, expressed.	ab initio prediction	Q10T52	Protein kinase, catalytic domain
Os03g0101250	Hypothetical gene.	AK241727	longestORF	-
Os03g0101300	Similar to Hexose transporter.	AK100524	NP_001149551.1	Sugar/inositol transporter
Os03g0101400	Hypothetical protein.	AK120679	longestORF	-
Os03g0101500	Similar to Phosphoglycerate mutase family protein.	AK109299	C3SAD0	PRIB5
Os03g0101600	Similar to Oxidoreductase, zinc-binding dehydrogenase family protein, expressed.	AK061143,AK069374	Q10T48	Alcohol dehydrogenase superfamily, zinc-type
Os03g0101800	Similar to DUF614 containing protein.	AK103196,AK061092,AK104399	C3SAD5	Uncharacterised protein family Cys-rich
Os03g0102000	Conserved hypothetical protein.	AK062867	B9FA98	-
Os03g0102200	Similar to DNA-directed RNA polymerase II 14.5 kDa polypeptide (EC 2.7.7.6) (RPB9) (RPB14.5).	AK120183	Q10T39	Zinc finger, TFIIS-type
Os03g0102300	Hypothetical conserved gene.	BT068414	Q10T37	-
Os03g0102333	Hypothetical conserved gene.	EU971180	Q10T37	CCR4-Not complex, Not1 subunit, domain of unknown function DUF3819
Os03g0102366	Similar to transcriptional regulator-related.	AK362299	NP_171710.3	CCR4-Not complex component, Not1, C-terminal
Os03g0102400	Similar to 2-phosphoglycerate kinase-related.	AK121721	NP_200953.1	-
Os03g0102700	Similar to Expansin-B7.	AK061423	Q9LD07	Major pollen allergen Lol pI
Os03g0102775	Hypothetical protein.	EU947703	longestORF	-
Os03g0106250	Non-protein coding transcript.	EU948014	NONE	-
Os03g0108400	Similar to Charged multivesicular body protein 3.	AK242193	B6SM72	Snf7
Os03g0108500	Similar to 4,4-dimethyl-sterol C4-methyl-oxidase (Fragment).	AK108704	B4FN64	Fatty acid hydroxylase
Os03g0108600	Similar to DEAD-box ATP-dependent RNA helicase 50.	AK065776	Q8GUG7	Helicase, C-terminal

Os03g0108700	Conserved hypothetical protein.	AK105115	Q8H7P7	-
Os03g0109650	Hypothetical conserved gene.	AK289246,AK243040	Q8H7V1	Protein of unknown function DUF3755
Os03g0109800	Hypothetical conserved gene.	AK066015	Q8H7V3	-
Os03g0110400	Similar to nuclear matrix protein-related.	AK100395	NP_568219.1	THO complex, subunit THOC1
Os03g0110900	Dimeric alpha-beta barrel domain containing protein.	AK070403	Q10SU2	Dimeric alpha-beta barrel
Os03g0111000	Conserved hypothetical protein.	AK062759	B8ALG1	-
Os03g0112400	Clathrin/coatomer adaptor, adaptin-like, appendage, C-terminal subdomain domain containing protei	AK063082	Q10SS6	Clathrin adaptor, alpha-adaptin, appendage, C-terminal subdomain
Os03g0112600	Similar to Plus-3 domain containing protein, expressed.	EU942983	Q7XCX5	SWIB/MDM2 domain
Os03g0112950	Non-protein coding transcript.	AK242233	NONE	-
Os03g0113000	Serine/threonine protein kinase domain containing protein.	AK071309	NP_001151054.1	Protein kinase, catalytic domain
Os03g0113100	Similar to Thymidine kinase.	AK066124	O81263	Thymidine kinase
Os03g0113500	Conserved hypothetical protein.	AK241800	B9FAE9	-
Os03g0113800	Tetratricopeptide-like helical domain containing protein.	AK065925	XP_002870606.1	Tetratricopeptide-like helical
Os03g0114850	Non-protein coding transcript.	BT063951	NONE	-
Os03g0117050	Non-protein coding transcript.	AK241608	NONE	-
Os03g0118700	Similar to predicted protein.	BT085971	Q8H052	Glucose-methanol-choline oxidoreductase, N-terminal
Os03g0121300	Similar to Peroxidase 1.	AK064633	Q8LMR7	Plant peroxidase
Os03g0122034	Non-protein coding transcript.	EU950997	NONE	-
Os03g0124300	Similar to ATP binding protein.	AK106076	B6TWY5	Malectin-like carbohydrate-binding domain
Os03g0127000	Similar to cell growth defect factor 2.	AK068479	NP_001148819.1	-
Os03g0127800	Non-protein coding transcript.	EU947022	NONE	-
Os03g0127950	Similar to inner membrane protein ybaL.	CT835134	NP_001147290.1	-
Os03g0128300	Conserved hypothetical protein.	AK064718	B8AMD0	-
Os03g0130800	Non-protein coding transcript.	AK058647	NONE	-
Os03g0137050	Non-protein coding transcript.	BT086855	NONE	-
Os03g0137600	Conserved hypothetical protein.	AK061189	B9FAZ2	-
Os03g0137700	Mg2+ transporter protein, CorA-like domain containing protein.	AK069498	Q10S25	-
Os03g0137750	Hypothetical protein.	tplb0043b08 (Wheat FLcDNA)	longestORF	-
Os03g0172000	Peptidase S59, nucleoporin family protein.	AK122024	NP_178183.2	Peptidase S59, nucleoporin
Os03g0174200	Conserved hypothetical protein.	AK103762,AK060616	A2XD32	-
Os03g0174400	Hypothetical conserved gene.	EF028768	Q10R18	Pathogenesis-related transcriptional factor/ERF, DNA-binding
Os03g0174800	Conserved hypothetical protein.	AK069179	B9FBL5	-
Os03g0174900	Similar to RAPB protein.	AK069348	O49915	CCAAT-binding transcription factor, subunit B
Os03g0175200	Similar to predicted protein.	AK242803	NP_001147647.1	Mitochondrial substrate/solute carrier
Os03g0176200	Hypothetical protein.	EU949353	longestORF	-
Os03g0176500	Hypothetical protein.	AK101385	longestORF	-
Os03g0177000	Acyl-CoA N-acyltransferase domain containing protein.	AK071368	NP_671784.2	GCN5-related N-acetyltransferase
Os03g0177900	EF-1 alpha.	AK119647,AK119528,AK071343	B8APM5	Protein synthesis factor, GTP-binding
Os03g0178150	Hypothetical gene.	BT086892	longestORF	-
Os03g0178200	Protein of unknown function YGGT family protein.	AK106024	A6MZH3	Uncharacterised protein family Ycf19
Os03g0178300	Hypothetical conserved gene.	AK121217	A2XD59	-
Os03g0178550	Hypothetical gene.	AK289159	longestORF	-
Os03g0179000	Similar to Tubulin-tyrosine ligase family protein, expressed.	AK069518	Q10QY3	Tubulin-tyrosine ligase
Os03g0179100	Similar to predicted protein.	CT835279	Q8H021	Germin
Os03g0179700	Similar to OSIGBa0140O07.11 protein.	CT835157	Q01J32	Tubby, C-terminal
Os03g0179950	Non-protein coding transcript.	CT835230	NONE	-
Os03g0180000	Sodium/calcium exchanger membrane region domain containing protein.	AK360521	NP_564650.1	Sodium/calcium exchanger membrane region
Os03g0180100	Protein of unknown function DUF1677, plant domain containing protein.	AK108326	B8APN4	Protein of unknown function DUF1677, plant
Os03g0180300	Hypothetical conserved gene.	AK061621	Q10QX0	Smr protein/MutS2 C-terminal
Os03g0180400	Proteasome subunit alpha type 6 (EC 3.4.25.1) (20S proteasome alpha subunit A) (20S proteasome s	AK101031,AK059929,AK060026	A2XD75	Proteasome, alpha-subunit, conserved site
Os03g0180700	Glycosyl transferase, group 1 domain containing protein.	AK069776	NP_001149006.1	Glycosyl transferase, family 1
Os03g0181500	Similar to (Rice Genome Annotation Project) fiddlehead-like protein.	AK100798	Q10QW0	3-Oxoacyl-[acyl-carrier-protein
Os03g0182700	Eukaryotic translation initiation factor 3 subunit 12 (eIF-3 p25) (eIF3k).	AK073293,AK120235	Q94HF1	-
Os03g0183000	Similar to AP2 domain containing protein RAP2.6 (Fragment).	AK060527	Q94HF3	Pathogenesis-related transcriptional factor/ERF, DNA-binding
Os03g0183050	Hypothetical gene.	EU941438	longestORF	-
Os03g0183300	Pathogenesis-related transcriptional factor and ERF domain containing protein.	AK106163,AK105922,AK119553	Q5MFV0	Pathogenesis-related transcriptional factor/ERF, DNA-binding
Os03g0183800	Similar to Leucine-rich repeat transmembrane protein kinase 1 (Fragment).	AK112046	O81105	Protein kinase, catalytic domain
Os03g0184050	Non-protein coding transcript.	AK058668	NONE	-
Os03g0187200	Non-protein coding transcript.	AK060541	NONE	-
Os03g0188200	Zinc finger, RING/FYVE/PHD-type domain containing protein.	AK058578	NP_001149891.1	Zinc finger, RING-type
Os03g0191200	WW/Rsp5/WWP domain containing protein.	AK070228	A2XDF1	WW/Rsp5/WWP
Os03g0192550	Hypothetical gene.	AK111026	longestORF	-
Os03g0197050	Non-protein coding transcript.	CT835219	NONE	-
Os03g0197825	Hypothetical protein.	BT086922	longestORF	-
Os03g0217900	Hypothetical protein.	AK119980	longestORF	-
Os03g0243700	Similar to Glycosyl hydrolase family 10 protein, expressed.	AK059149	Q10P81	Glycoside hydrolase, family 10
Os03g0243750	Hypothetical protein.	tplb0048l17 (Wheat FLcDNA)	longestORF	-
Os03g0244750	Conserved hypothetical protein.	ab initio prediction	NP_001183257.1	-
Os03g0246500	Non-protein coding transcript.	AK105050	NONE	-
Os03g0251950	Hypothetical protein.	BT016922	longestORF	-
Os03g0275350	Non-protein coding transcript.	EU948346	NONE	-
Os03g0286200	Similar to P-protein.	AK105065	B6SYB7	Prephenate dehydratase
Os03g0289133	Non-protein coding transcript.	BT018428	NONE	-
Os03g0292100	Hypothetical conserved gene.	AK287695	A3F4K2	Protein phosphatase 2C, manganese/magnesium aspartate binding site
Os03g0295800	Similar to gamma-interferon-inducible lysosomal thiol reductase.	AK106050	B6TUH5	Gamma interferon inducible lysosomal thiol reductase GILT
Os03g0295866	Transcription factor IIS, N-terminal domain containing protein.	BT040964	Q10MT6	Transcription factor IIS, N-terminal
Os03g0295932	Hypothetical protein.	EU965145	longestORF	-
Os03g0296600	Similar to ECA1 protein.	AK109176	Q9SES8	Protein of unknown function DUF784
Os03g0296700	Non-protein coding transcript.	AK121470	NONE	-
Os03g0298100	Conserved hypothetical protein.	AK369609	NP_001173382.1	-
Os03g0298400	Similar to 26S protease regulatory subunit 4 homolog.	AK058779	P46466	ATPase, AAA-type, core
Os03g0299200	Similar to Alcohol dehydrogenase (Fragment).	AK100767	P93795	Short-chain dehydrogenase/reductase SDR
Os03g0299400	Hypothetical protein.	EU954997	longestORF	-
Os03g0299600	Wound-induced protein, Wun1 domain containing protein.	AK068151	XP_002468012.1	Wound-induced protein, Wun1
Os03g0299800	Hypothetical conserved gene.	AK100929	B8AM23	Uncharacterised protein family Cys-rich
Os03g0300000	Similar to Xyloglucan 6-xylosyltransferase (EC 2.4.2.39) (AtXT1).	AK060863	NP_001150077.1	Galactosyl transferase
Os03g0300400	Pathogen-related protein (JIOsPR10).	AK121376	Q945E9	Bet v I allergen
Os03g0300500	Similar to Pectin methylesterase 6 (Fragment).	AK070513	A2XFP7	Pectinesterase, catalytic
Os03g0300600	Similar to Chaperone protein dnaJ.	AK066250	B6U761	Heat shock protein DnaJ, N-terminal
Os03g0301600	Conserved hypothetical protein.	AK062764	B9F7W2	-
Os03g0301950	Peroxisomal biogenesis factor 11 family protein.	Q10MN3	Q10MN3	Peroxisomal biogenesis factor 11
Os03g0302000	Peroxisomal biogenesis factor 11 family protein.	AK287976	Q10MN2	Peroxisomal biogenesis factor 11
Os03g0302100	Non-protein coding transcript.	AK063197	NONE	-
Os03g0302200	Zinc finger, RING/FYVE/PHD-type domain containing protein.	AK120853	A2XFQ9	Zinc finger, RING-type
Os03g0302700	Zinc finger, RING/FYVE/PHD-type domain containing protein.	AK112010	NP_001152943.1	Zinc finger, RING-type
Os03g0304100	Conserved hypothetical protein.	AK111121	B8AMI6	-
Os03g0304800	Lg106-like family protein.	AK062656	NP_001148326.1	Endosulphine
Os03g0305500	Similar to Argininosuccinate lyase.	AK070233	B4FAV4	Fumarate lyase
Os03g0305550	Hypothetical gene.	EU949424	longestORF	-
Os03g0305600	Mitochondrial import inner membrane translocase, subunit Tim17/22 family protein.	AK063714	Q2HU23	Mitochondrial inner membrane translocase subunit Tim17/Tim22/Tim23/peroxisomal p
Os03g0305800	Galactosyl transferase family protein.	AK105101,AK105005	A2XFT5	-
Os03g0306200	Similar to transducin family protein / WD-40 repeat family protein.	AK241828	XP_002865733.1	Bromodomain
Os03g0306302	Hypothetical conserved gene.	DQ245981	Q10MJ9	-
Os03g0306800	Similar to CP12 (Fragment).	AK103722	B7XB92	Domain of unknown function CP12
Os03g0306900	Haem oxygenase-like, multi-helical domain containing protein.	AK073626	B6TPF2	TENA/THI-4 protein/Coenzyme PQQ biosynthesis protein C domain
Os03g0307000	Conserved hypothetical protein.	AK243201	NP_001049892.2	-
Os03g0307300	Nicotianamine synthase 1 (EC 2.5.1.43) (S-adenosyl-L-methionine:S- adenosyl-L-methionine:S-aden	AK112069	Q0DSH9	Nicotianamine synthase
Os03g0311401	Conserved hypothetical protein.	AK242599	NP_001173394.1	-
Os03g0320800	Similar to Leucine Rich Repeat family protein, expressed.	AK103265	Q10M75	Leucine-rich repeat
Os03g0320900	Guanylate kinase family protein.	AK106255	NP_001149581.1	Guanylate kinase
Os03g0321700	Similar to WRKY transcription factor 55.	AK101653	A6N066	DNA-binding WRKY
Os03g0321800	Similar to WRKY transcription factor 55.	AK111447	NP_001030961.1	Octanoyltransferase
Os03g0324650	Non-protein coding transcript.	BT085189	NONE	-
Os03g0325500	Similar to Phosphate starvation regulator protein (Regulatory protein of P- starvation acclimation res	AK100282	A2XG76	Myb-like DNA-binding domain, SHAQKYF class
Os03g0327100	Similar to CUC1.	AK065989	NP_001147731.1	No apical meristem

Os03g0327600	Ricin B-related lectin domain containing protein.	AK069815	Q40705	Ricin B-related lectin
Os03g0328000	Dedicator of cytokinesis family protein.	AK059469	NP_193367.7	Dedicator of cytokinesis
Os03g0328400	Similar to Apyrase GS50 (Fragment).	AF358764	D3Y1I0	Nucleoside phosphatase GDA1/CD39
Os03g0328450	Non-protein coding transcript.	BT017220	NONE	-
Os03g0328600	Hypothetical protein.	EU949280	longestORF	-
Os03g0329200	Similar to cDNA clone:J013002B05, full insert sequence.	AK064318	B7F3U2	-
Os03g0336400	Similar to Alpha-expansin OsEXPA4.	AK107698	Q9M4X7	Expansin
Os03g0336700	PAP/25A core domain containing protein.	AK120611	Q10LS4	PAP/25A-associated
Os03g0338800	Hypothetical conserved gene.	AK064660	B8B3T4	-
Os03g0339150	Hypothetical gene.	AK241704	longestORF	-
Os03g0342900	Dormancyauxin associated family protein.	AK104391,AK060136,AK060980	B4FA62	-
Os03g0343225	Hypothetical gene.	EU951910	longestORF	-
Os03g0343300	Small-subunit processome, Utp14 domain containing protein.	AK243183	Q10LM2	Small-subunit processome, Utp14
Os03g0343400	Similar to Photolyase/blue-light receptor PHR2.	AK105611	B6U742	DNA photolyase, FAD-binding/Cryptochrome, C-terminal
Os03g0343500	Similar to 60S ribosomal protein L22-2.	AK059599	B6U998	Ribosomal protein L22e
Os03g0343700	Brix domain containing protein.	AK060603	XP_002864754.1	Brix domain
Os03g0343900	Photosystem II PsbX domain containing protein.	AK105813	GeneMark	Photosystem II PsbX
Os03g0344232	Conserved hypothetical protein.	CT835238	Q10RS8	-
Os03g0344300	Similar to Ulp1 protease family, C-terminal catalytic domain containing protein, expressed.	AK073314	Q10LL1	Peptidase C48, SUMO/Sentrin/Ubl1
Os03g0344650	Hypothetical protein.	EU943048	longestORF	-
Os03g0345100	Similar to auxin response factor 75.	AK065579	NP_001149782.1	Rad9
Os03g0345200	40S ribosomal protein S21.	AK073724,AK104084,AK119262	P35687	Ribosomal protein S21e
Os03g0345300	Similar to Desiccation-related protein Lb_13-62 (Fragment).	AK108277	B1PMG8	-
Os03g0345500	Non-protein coding transcript.	EU975566	NONE	-
Os03g0345700	Similar to Heavy metal-associated domain containing protein, expressed.	AK066080	Q10LJ9	-
Os03g0345901	Hypothetical gene.	EU973702	longestORF	-
Os03g0347700	NPH3 domain containing protein.	AK110492	E9NZU3	NPH3
Os03g0347750	Hypothetical gene.	BT083870	longestORF	-
Os03g0347800	Peptidyl-tRNA hydrolase family protein.	AK073756	XP_002871908.1	Peptidyl-tRNA hydrolase
Os03g0348200	Terpene synthase-like domain containing protein.	EU954571	NP_001050103.1	Terpene synthase-like
Os03g0348900	Similar to CHY zinc finger family protein, expressed.	AK073680	Q10LI0	Zinc finger, RING-type
Os03g0349200	Similar to transposon protein.	AK101089	NP_001148044.1	-
Os03g0349400	Hypothetical protein.	tplb0040h06 (Wheat FLcDNA)	longestORF	-
Os03g0351100	Similar to CONSTANS-like protein CO9 (Fragment).	AK101822	C0PI33	Zinc finger, B-box
Os03g0351200	NAD(P)-binding domain containing protein.	AK353935	D5L1S4	NmrA-like
Os03g0351300	Similar to beta-amylase.	BT041352	NP_001151271.1	Glycoside hydrolase, family 14
Os03g0351400	Similar to Tubby-like protein 3.	AK102370	Q10LG8	Tubby, C-terminal
Os03g0353701	Similar to aberrant pollen transmission 1.	AK242536	NP_001106066.1	-
Os03g0355400	WD40 repeat-like domain containing protein.	BT055024	NP_181681.1	WD40 repeat
Os03g0355700	Hypothetical conserved gene.	BT018850	NP_001050136.1	IQ motif, EF-hand binding site
Os03g0355800	Similar to Diphthine synthase (EC 2.1.1.98) (Diphthamide biosynthesis methyltransferase).	AK103902	B8APT0	Tetrapyrrole methylase
Os03g0356300	Ribosomal protein L6 family protein.	AK061469,AK099034	NP_001148555.1	Ribosomal protein L6
Os03g0363700	Pentatricopeptide repeat domain containing protein.	EU954511	NP_001050167.1	Pentatricopeptide repeat
Os03g0368150	Hypothetical protein.	BT085962	longestORF	-
Os03g0387900	Hypothetical conserved gene.	AK063085	Q10KE2	-
Os03g0388000	Similar to Splicing factor SC35.	AK073057	B6T6J9	RNA recognition motif domain
Os03g0388650	Hypothetical protein.	EU971393	longestORF	-
Os03g0392400	Heat shock protein DnaJ, N-terminal domain containing protein.	AK106027,AK073726	B6TYB9	Heat shock protein DnaJ, N-terminal
Os03g0392833	Hypothetical protein.	EU949881	longestORF	-
Os03g0393350	Hypothetical genes.	ab initio prediction	A3AIQ6	-
Os03g0419200	Hypothetical gene.	AK106284	GeneMark	-
Os03g0419700	Hypothetical conserved gene.	ab initio prediction	Q75GC7	-
Os03g0437100	Zinc finger, C2H2-type domain containing protein.	AK108997	A2XIC5	Zinc finger, C2H2
Os03g0563200	Hypothetical conserved gene.	AK062665	Q10I63	-
Os03g0589500	Conserved hypothetical protein.	AK105481	Q5W6J4	-
Os03g0593100	Conserved hypothetical protein.	AK287562	NP_001050587.1	-
Os03g0605400	Non-protein coding transcript.	AK063612	NONE	-
Os03g0609400	Non-protein coding transcript.	AK241806	NONE	-
Os03g0609500	Similar to LOB domain protein 39.	AK288002	NP_201543.1	Lateral organ boundaries, LOB
Os03g0617101	Non-protein coding transcript.	CT835260	NONE	-
Os03g0627300	Similar to nucleotide-binding protein-like.	AK106686	NP_001150831.1	Cobyrinic acid a,c-diamide synthase
Os03g0633900	Nucleic acid-binding, OB-fold-like domain containing protein.	AK068169,AK059181	NP_001148836.1	Primosome PriB/single-strand DNA-binding
Os03g0637800	Regulator of chromosome condensation/beta-lactamase-inhibitor protein II domain containing protei	AK101339	Q948R7	Regulator of chromosome condensation, RCC1
Os03g0637900	Similar to Centromere protein-like protein (Fragment).	AK060224	Q84VB6	-
Os03g0638000	Similar to Centromere protein-like protein (Fragment).	AY224555	Q84VD9	-
Os03g0643100	Homeodomain-like containing protein.	AK106587	Q60DP5	Myb/SANT-like domain
Os03g0646850	Non-protein coding transcript.	BT017109	NONE	-
Os03g0647400	GCK domain containing protein.	AK073665	Q9LMF3	GCK
Os03g0647500	Similar to GTP binding protein.	AK064308	B6U832	-
Os03g0647550	Non-protein coding transcript.	AF124739	NONE	-
Os03g0647575	Hypothetical protein.	BT086909	longestORF	-
Os03g0648100	Protein of unknown function DUF266, plant family protein.	AK109329	A3AKV3	Glycosyl transferase, family 14
Os03g0648250	Zinc finger, GRF-type domain containing protein.	AK241508	Q259A1	Zinc finger, GRF-type
Os03g0648400	Non-protein coding transcript.	AK058555	NONE	-
Os03g0648500	Cupredoxin domain containing protein.	AK107766	A2XV11	Plastocyanin-like
Os03g0650300	Hypothetical conserved gene.	tplb0014g09 (Wheat FLcDNA)	Q43713	-
Os03g0650350	Hypothetical gene.	EU947173	longestORF	-
Os03g0650400	Similar to ameiotic 1.	AK064367	NP_001139538.1	-
Os03g0650700	Translation elongation factor EFG/EF2, C-terminal domain containing protein.	AK102195	XP_002885535.1	Translation elongation factor EFG/EF2, C-terminal
Os03g0655100	Similar to Cupin, RmlC-type.	EU963588	B6TF23	Cupin, RmlC-type
Os03g0655200	Similar to Membrin 11 (AtMEMB11) (Golgi SNAP receptor complex member 2-1) (27 kDa Golgi S	AK111247	B6TMP4	-
Os03g0655300	CS domain domain containing protein.	BT061748	B6T9V0	CS-like domain
Os03g0655400	Similar to LIP5.	AK059150	GeneMark	-
Os03g0655600	MULE transposase, conserved domain domain containing protein.	AK120778	Q7Y091	FAR1 DNA binding domain
Os03g0655700	Similar to 3-isopropylmalate dehydrogenase.	AK066745	NP_001149749.1	Isocitrate/isopropylmalate dehydrogenase
Os03g0656000	Hypothetical conserved gene.	ab initio prediction	NP_001173570.1	Heat shock protein Hsp20
Os03g0656051	Hypothetical conserved gene.	EU974920	NP_001145365.1	-
Os03g0656100	Similar to Hsp20/alpha crystallin family protein, expressed.	AK062338	Q10FT7	-
Os03g0656800	Similar to 3-glucanase.	AK069506	NP_001147672.1	Glycoside hydrolase, family 17
Os03g0656850	Conserved hypothetical protein.	BT019195	C4J0K6	-
Os03g0657100	Similar to predicted protein.	AK066133	NP_564125.2	Armadillo-like helical
Os03g0657400	Hypothetical conserved gene.	ab initio prediction	NP_001050814.2	DNA-binding WRKY
Os03g0658000	Cytochrome P450 domain containing protein.	EU969052	Q7Y098	Cytochrome P450
Os03g0658800	Hypothetical conserved gene.	AK063030	Q850S8	-
Os03g0659200	Similar to Ferredoxin.	AK062598	NP_001151112.1	Ferredoxin
Os03g0659233	Cytochrome b559 subunit alpha.	P0C368	P0C368	Photosystem II cytochrome b559, conserved site
Os03g0659266	Cytochrome b559 subunit beta.	P0C399	P0C399	Photosystem II cytochrome b559, conserved site
Os03g0659300	Glyoxalase/bleomycin resistance protein/dioxygenase domain containing protein.	AK241252	NP_001148888.1	-
Os03g0659350	Hypothetical gene.	AK288422	longestORF	-
Os03g0659400	Hypothetical conserved gene.	AK111930	A2XK97	-
Os03g0659450	Hypothetical protein.	tplb0028l15 (Wheat FLcDNA)	longestORF	-
Os03g0659700	Similar to LOB domain protein 12.	AK071550	NP_174417.1	Lateral organ boundaries, LOB
Os03g0659800	Similar to H0525G02.7 protein.	AK062366	Q00RR3	-
Os03g0660000	Conserved hypothetical protein.	AK111052	B8ANL4	-
Os03g0660050	Non-protein coding transcript.	CT835270	NONE	-
Os03g0660500	Auxin responsive SAUR protein domain containing protein.	AK361840	Q75GQ1	Auxin responsive SAUR protein
Os03g0661250	Hypothetical protein.	EU941142	longestORF	-
Os03g0661300	Beta-tubulin (Beta-3 tubulin) (Tubulin beta subunit).	AK121321	Q76FS2	Tubulin
Os03g0661700	Non-protein coding transcript.	AK073701	NONE	-
Os03g0663500	Similar to Osmotin precursor.	U77656	NP_001147526.1	Thaumatin, pathogenesis-related
Os03g0663600	Similar to Pathogenesis-related thaumatin-like protein.	EU967040	O04363	-
Os03g0663750	Hypothetical protein.	tplb0024b15 (Wheat FLcDNA)	longestORF	-
Os03g0663800	Globulin 1 (Fragment).	AK105347	Q10FL8	Cupin 1
Os03g0664300	F-box domain, cyclin-like domain containing protein.	tplb0015o19 (Wheat FLcDNA)	Q75GY3	F-box domain, cyclin-like
Os03g0664400	Similar to Protease inhibitor/seed storage/LTP family protein.	AK058628	B6U491	Plant lipid transfer protein/seed storage/trypsin-alpha amylase inhibitor

Os03g0664700	RNA polymerase II accessory factor, Cdc73 domain containing protein.	AK100794	B8ANM7	RNA polymerase II accessory factor, Cdc73
Os03g0664800	Acyl-CoA N-acyltransferase domain containing protein.	AK243094	NP_001150788.1	GCN5-related N-acetyltransferase
Os03g0664901	Hypothetical protein.	EU957022	longestORF	-
Os03g0665700	Similar to predicted protein.	DQ245183	NP_001050842.2	Protein of unknown function DUF1677, plant
Os03g0665751	Hypothetical gene.	BT086957	longestORF	-
Os03g0665800	Armadillo-type fold domain containing protein.	AK107693	NP_191180.2	Armadillo-like helical
Os03g0666300	Hypothetical conserved gene.	BT083602	Q75H97	MYB-like
Os03g0666500	Similar to Ras-related protein RHA1.	AK061203	B4F7V6	Small GTPase superfamily
Os03g0666550	Hypothetical protein.	tplb0055b17 (Wheat FLcDNA)	longestORF	-
Os03g0666600	UDP-glucuronosyl/UDP-glucosyltransferase family protein.	AK064709	NP_001151546.1	UDP-glucuronosyl/UDP-glucosyltransferase
Os03g0666700	TRAM, LAG1 and CLN8 homology domain containing protein.	AK061721,AK061309,AK102010	NP_001149791.1	TRAM/LAG1/CLN8 homology domain
Os03g0685750	Hypothetical conserved gene.	CT833489	NP_195008.5	-
Os03g0685800	Hypothetical protein.	AK058278	longestORF	-
Os03g0698400	Non-protein coding transcript.	AK058933	NONE	-
Os03g0707250	Non-protein coding transcript.	AK242476	NONE	-
Os03g0746500	Similar to predicted protein.	AK058367	NP_193215.2	Helicase, C-terminal
Os03g0773150	40S ribosomal protein S29.	CU406248	A2ZG71	Ribosomal protein S14
Os03g0773300	Protein kinase, core domain containing protein.	AK100394	NP_001105795.1	Protein kinase, catalytic domain
Os03g0773450	Hypothetical protein.	tplb0046o04 (Wheat FLcDNA)	longestORF	-
Os03g0773700	Similar to Receptor-like protein kinase 2.	AK102670	Q7XZW7	Protein kinase, catalytic domain
Os03g0774400	Similar to predicted protein.	AK109683	Q10E11	-
Os03g0775200	Protein of unknown function DUF248, methyltransferase putative family protein.	AK120397	NP_564084.1	Protein of unknown function DUF248, methyltransferase putative
Os03g0775250	Hypothetical protein.	AK358342	longestORF	-
Os03g0775300	Flavodoxin/nitric oxide synthase domain containing protein.	AK100507	Q8H8N3-2	Radical SAM
Os03g0775400	Tetratricopeptide-like helical domain containing protein.	AK107354	A2XMF9	Pentatricopeptide repeat
Os03g0775650	Non-protein coding transcript.	tplb0059h22 (Wheat FLcDNA)	NONE	-
Os03g0775700	Conserved hypothetical protein.	AK067093	A2XMG2	-
Os03g0775900	Conserved hypothetical protein.	AK374530	Q8H8M7	-
Os03g0777000	Similar to NAC-domain containing protein 19 (ANAC019) (ANAC) (Abscicic-acid- responsive NAC	AK073539	F1DJT1	No apical meristem
Os03g0778100	Similar to Photosystem-1 F subunit.	AK060493	Q9ZSU5	Photosystem I PsaF, reaction centre subunit III
Os03g0778400	Similar to predicted protein.	CT835114	NP_001051445.1	-
Os03g0780200	Conserved hypothetical protein.	AK107920	A2XMI6	-
Os03g0780350	Similar to SAUR1 - auxin-responsive SAUR family member.	EU974244	NP_001152203.1	Auxin responsive SAUR protein
Os03g0780400	Similar to Actin-depolymerizing factor 6 (ADF-6) (AtADF6).	AK073162,AK061347	Q9AY76	Actin-binding, cofilin/tropomyosin type
Os03g0780550	Hypothetical protein.	tplb0045b10 (Wheat FLcDNA)	longestORF	-
Os03g0780600	Tubulin beta-1 chain (Beta-1 tubulin).	AK120180	P37832	Tubulin
Os03g0780800	Fatty acid hydroxylase domain containing protein.	AK103085	NP_001149139.1	Fatty acid hydroxylase
Os03g0780900	Protein of unknown function DUF858, methyltransferase-like family protein.	AK100619	A2XMJ1	Protein of unknown function DUF858, methyltransferase-like
Os03g0781000	Conserved hypothetical protein.	AK065910	Q9AY71	-
Os03g0781100	Pentatricopeptide repeat domain containing protein.	EU950024	Q9AY70	Pentatricopeptide repeat
Os03g0781200	Hypothetical protein.	AK110946	longestORF	-
Os03g0781300	Conserved hypothetical protein.	AK120661	F2EFE7	-
Os03g0781400	Ribose 5-phosphate isomerase family protein.	AK071970	NP_001151663.1	Ribose 5-phosphate isomerase, type A
Os03g0781501	Non-protein coding transcript.	AK243070	NONE	-
Os03g0781600	Conserved hypothetical protein.	AK064719	A2XMJ5	-
Os03g0782200	Hypothetical protein.	AK069119	GeneMark	-
Os03g0782300	Conserved hypothetical protein.	AK070713	B9F3B9	-
Os03g0782350	Hypothetical gene.	CT832459	longestORF	-
Os03g0782400	Conserved hypothetical protein.	ab initio prediction	Q10CH8	-
Os03g0782900	Pentatricopeptide repeat domain containing protein.	AK108367	Q9AY61	Pentatricopeptide repeat
Os03g0782950	Hypothetical protein.	tplb0031e02 (Wheat FLcDNA)	longestORF	-
Os03g0783000	Similar to Actin-related protein 7.	AK069426	A3ANB5	Actin-like
Os03g0783051	Conserved hypothetical protein.	FP098389	A3ANB6	-
Os03g0799900	Similar to sec12-like protein 1.	AK111711	NP_001148204.1	WD40 repeat
Os03g0800400	Protein of unknown function DUF1618 domain containing protein.	AK071430	Q851R3	Domain of unknown function DUF1618
Os03g0800900	Hypothetical conserved gene.	AK241533	Q851R7	-
Os03g0802650	Hypothetical gene.	CT837941	longestORF	-
Os03g0803200	Similar to CLE family OsCLE306 protein.	AK111283	A8R3N5	-
Os03g0803250	Non-protein coding transcript.	EU958975	NONE	-
Os03g0803500	Similar to Prolyl 4-hydroxylase alpha-1 subunit-like protein.	AK059759	B6TAV1	Metridin-like ShK toxin
Os03g0803600	Glycosyl transferase, family 31 protein.	AK241975	B6SJ23	Glycosyl transferase, family 31
Os03g0803700	Hypothetical conserved gene.	AK064481	Q84T10	-
Os03g0804100	Conserved hypothetical protein.	FP100876	NP_001051616.1	-
Os03g0804300	Zinc finger, DHHC-type domain containing protein.	AK067295	NP_001148846.1	Zinc finger, DHHC-type, palmitoyltransferase
Os03g0804400	Conserved hypothetical protein.	AK073430	Q75HJ9	-
Os03g0804500	Similar to Germin-like protein subfamily T member 1 precursor.	AK062698	Q10BU2	Germin
Os03g0804600	Similar to Germin-like protein 3-7.	AK248846	NP_001051622.2	-
Os03g0804700	Germin-like protein 3-8.	Q75HJ4	Q75HJ4	Germin
Os03g0805100	Similar to Squalene synthase (EC 2.5.1.21).	AK242592	NP_001104839.1	Squalene/phytoene synthase
Os03g0805300	Similar to Phosphoprotein phosphatase 2A isoform 4.	AK060885,AK099257,AK100350	Q10BT5	Metallophosphoesterase domain
Os03g0805350	Hypothetical protein.	BT018461	longestORF	-
Os03g0805400	Phosphatidic acid phosphatase type 2/haloperoxidase domain containing protein.	AK071708	NP_001151171.1	Phosphatidic acid phosphatase type 2/haloperoxidase
Os03g0805500	Similar to AAE18 (ACYL-ACTIVATING ENZYME 18)%3B catalytic/ ligase.	AK067857	NP_175929.3	-
Os03g0805600	Similar to pheophorbide a oxygenase.	CT835283	B6ST74	Rieske [2Fe-2S] iron-sulphur domain
Os03g0805700	Similar to Pheophorbide a oxygenase.	EU955939	B6ST74	Pheophorbide a oxygenase
Os03g0805733	Hypothetical protein.	tplb0031c13 (Wheat FLcDNA)	longestORF	-
Os03g0806400	Similar to Elongation factor P family protein, expressed.	AK073074	Q10BS5	-
Os03g0806500	Thioredoxin domain 2 containing protein.	AK073308,AK100198	Q84M47	Thioredoxin-like fold
Os03g0806600	Conserved hypothetical protein.	AK070959	B9F6V2	-
Os03g0806700	Protein of unknown function DUF868, plant family protein.	AK062034	XP_002466252.1	Protein of unknown function DUF868, plant
Os03g0806800	Conserved hypothetical protein.	AK061623	A2XN54	-
Os03g0806900	Similar to Cytochrome-C reductase 14 kDa subunit (EC 1.10.2.2) (Fragment).	AK121708	B6UBZ9	Cytochrome d ubiquinol oxidase, 14kDa subunit
Os03g0807100	Protein of unknown function DUF239 domain containing protein.	AK242472	B6TV70	Glucoamylase, putatuve
Os03g0807150	Hypothetical protein.	tplb0032e19 (Wheat FLcDNA)	longestORF	-
Os03g0807200	Protein of unknown function DUF1639 family protein.	AK103496,AK063932	A2XN58	Protein of unknown function DUF1639
Os03g0807400	Pentatricopeptide repeat domain containing protein.	AK065345	Q84M45	Pentatricopeptide repeat
Os03g0807600	Similar to Cyclopropane-fatty-acyl-phospholipid synthase family protein, expressed.	CT835101	Q10BR3	Methyltransferase type 11
Os03g0807700	Protein of unknown function DUF642 domain containing protein.	AK059286	A2XN62	Protein of unknown function DUF642
Os03g0807900	Chaperonin-like RbcX family protein.	AK060962	A2XN64	Chaperonin-like RbcX
Os03g0808000	Glycoside hydrolase, family 28 domain containing protein.	BT042622	NP_001051647.1	Glycoside hydrolase, family 28
Os03g0808100	Similar to Cellulose synthase BoCesA5.	AK058615	Q4U0Z5	Cellulose synthase
Os03g0808150	Hypothetical gene.	BT019277	longestORF	-
Os03g0808175	Non-protein coding transcript.	U48693	NONE	-
Os03g0808200	UDP-glucuronosyl/UDP-glucosyltransferase family protein.	BT037819	NP_001149762.1	UDP-glucuronosyl/UDP-glucosyltransferase
Os03g0808350	Hypothetical protein.	AK361927	longestORF	-
Os03g0808400	Ubiquitin domain containing protein.	ab initio prediction	Q84M51	Ubiquitin
Os03g0809800	Conserved hypothetical protein.	AK241877	Q10BN7	-
Os03g0810900	ARF/SAR superfamily domain containing protein.	AK121284	XP_002871068.1	Patatin/Phospholipase A2-related
Os03g0820600	Similar to cDNA clone:001-009-H02, full insert sequence.	AK105336	B7E3A0	Actin-binding, cofilin/tropomyosin type
Os03g0822400	Conserved hypothetical protein.	AK102425,AK070075	A2XNH3	-
Os03g0824000	Peptidase S8 and S53, subtilisin, kexin, sedolisin domain containing protein.	AK111129	A9NKD9	Protein of unknown function DUF3743
Os03g0832400	Similar to Protein phosphatase 2C-like protein.	AK120748	B4G0F7	Protein phosphatase 2C-like
Os03g0832800	Non-protein coding transcript.	AK062843	NONE	-
Os03g0832850	Hypothetical protein.	tplb0038i08 (Wheat FLcDNA)	longestORF	-
Os03g0832900	Similar to DNAJ-like protein (Fragment).	AK060945	Q84VA4	Heat shock protein DnaJ, N-terminal
Os03g0833100	Similar to Tasselseed2 protein (Fragment).	AK068662	Q2EFH2	Short-chain dehydrogenase/reductase SDR
Os03g0833200	Methyltransferase TRM13 domain containing protein.	AK102870	B8AMR9	Methyltransferase TRM13
Os03g0833600	X8 domain containing protein.	AK109880	Q10B16	X8
Os03g0833700	RNA 3'-terminal phosphate cyclase family protein.	AK121918	NP_680196.1	RNA 3&apos%3B-terminal phosphate cyclase
Os03g0833900	Similar to Cytosine deaminase (EC 3.5.4.1).	AK073655,AK104068	NP_001105963.1	-
Os03g0834000	Flap endonuclease-1b (EC 3.-.-.-) (OsFEN-1b).	AK062149	Q75LI2	5'-3' exonuclease, N-terminal
Os03g0834050	Similar to predicted protein.	AK240756	B6TQ05	HAD-superfamily hydrolase, subfamily IA, variant 3
Os03g0834150	Non-protein coding transcript.	X06284	NONE	-
Os03g0835100	Similar to Chloroplast protein import component Toc159.	AK373768	Q75LI9	Chloroplast protein import component Toc86/159
Os03g0835150	Conserved hypothetical protein.	AK243368	NP_001173702.1	-

Os03g0835300	Hypothetical protein.	AK064539	longestORF	-
Os03g0835400	Similar to Uvs101.	AK061773,AK098986,AK105896	Q75LJ3	Electron transfer flavoprotein, alpha subunit
Os03g0835600	Galactose oxidase/kelch, beta-propeller domain containing protein.	AK101677	NP_001152073.1	Acyl-CoA-binding protein, ACBP
Os03g0835800	Galactose oxidase/kelch, beta-propeller domain containing protein.	AK102903	Q10AZ7	Kelch repeat type 1
Os03g0835900	Similar to Ferredoxin III, chloroplast precursor (Fd III).	AK121063	D3JUX1	Ferredoxin
Os03g0835950	Non-protein coding transcript.	AK370418	NONE	-
Os03g0836000	Similar to Actin 7 (Actin 2).	AK063598	Q10AZ4	Actin-like
Os03g0836050	Hypothetical gene.	BT018641	longestORF	-
Os03g0844301	Hypothetical gene.	CT835264	longestORF	-
Os03g0856200	Similar to Longin-like.	AK058786	Q2HUA7	Longin-like
Os03g0856300	Similar to Acyl carrier protein III, chloroplast precursor (ACP III).	AK108342	A2XP91	Acyl carrier protein
Os03g0856500	Similar to Plastid-specific 30S ribosomal protein 1, chloroplast precursor (CS- S5) (CS5) (S22) (Rib	AK104733,AK061449	C6YXL0	Ribosomal protein S30Ae/sigma 54 modulation protein
Os04g0102600	Similar to Splicing factor 3B subunit 2 (Spliceosome associated protein 145) (SAP 145) (SF3b150) (	AK104417,AK070814	Q01N45	Peptidase S24/S26A/S26B/S26C, beta-ribbon domain
Os04g0120000	Hypothetical protein.	AK372466	longestORF	-
Os04g0125800	Similar to Growth-regulating factor 1.	EF515848	Q9SDZ5	-
Os04g0171232	Conserved hypothetical protein.	BT039178	A2WJQ3	-
Os04g0213800	Similar to Bacterial blight resistance protein XA26.	DQ119307	Q2EZ14	Protein kinase, catalytic domain
Os04g0234600	Similar to Sedoheptulose-1,7-bisphosphatase.	AK062081	Q84JG8	Fructose-1,6-bisphosphatase class 1/Sedoheputulose-1,7-bisphosphatase
Os04g0250200	Conserved hypothetical protein.	AK058584	B8ARM3	-
Os04g0252850	Hypothetical gene.	EU961904	longestORF	-
Os04g0253000	Similar to Histone H1.	AK073668	Q01M78	Histone H1/H5
Os04g0255525	Hypothetical gene.	AK242256	longestORF	-
Os04g0255600	Cytochrome P450 domain containing protein.	BT064329	Q7XQ53	Cytochrome P450
Os04g0259200	Conserved hypothetical protein.	AK119366	B8B6C2	-
Os04g0286500	Homeodomain-like containing protein.	AK064584	B8BCY2	MYB-like
Os04g0294812	EF-hand-like domain domain containing protein.	FP097437	Q33AZ2	EF-hand-like domain
Os04g0309751	Hypothetical protein.	tplb0054p19 (Wheat FLcDNA)	longestORF	-
Os04g0313600	Zinc finger, CCHC-type domain containing protein.	AK288309	A2XRV4	Zinc finger, CCHC-type
Os04g0313900	Similar to cytokinin-O-glucosyltransferase 2.	EU976007	NP_001152029.1	UDP-glucuronosyl/UDP-glucosyltransferase
Os04g0326100	Similar to H0107B07.2 protein.	CT837883	Q01M39	UDP-glucuronosyl/UDP-glucosyltransferase
Os04g0326300	Hypothetical conserved gene.	AK242372	NP_001052474.2	-
Os04g0332700	Conserved hypothetical protein.	AK071842	Q6MWJ9	-
Os04g0333850	Non-protein coding transcript.	CT835070	NONE	-
Os04g0334700	Peptidase aspartic, catalytic domain containing protein.	AK120870	B7F2P1	Peptidase A1
Os04g0336001	Hypothetical protein.	EU945299	longestORF	-
Os04g0336600	Peptidase aspartic, catalytic domain containing protein.	AK120870	B7F2P1	Peptidase A1
Os04g0336801	Conserved hypothetical protein.	AK289258	Q2QWP9	-
Os04g0340550	Hypothetical gene.	AK068264	longestORF	-
Os04g0344400	Similar to OSIGBa0106G08.5 protein.	BT041617	Q01KN9	Terpene synthase-like
Os04g0351800	Conserved hypothetical protein.	FP101309	B8ASJ0	-
Os04g0351933	Conserved hypothetical protein.	FP101309	A3ASQ2	-
Os04g0352066	Conserved hypothetical protein.	AK375252	B9FEL4	-
Os04g0352200	Conserved hypothetical protein.	AK103395	A2XS74	-
Os04g0353000	Similar to Cation-transporting ATPase.	AK063167	NP_568633.1	-
Os04g0354200	Similar to OSIGBa0092G14.5 protein.	ab initio prediction	Q01KX7	FAR1 DNA binding domain
Os04g0356600	Similar to OSIGBa0105P02.3 protein.	AK066063	Q01KU2	Protein kinase, catalytic domain
Os04g0356800	Hypothetical protein.	tplb0036o12 (Wheat FLcDNA)	longestORF	-
Os04g0357500	Conserved hypothetical protein.	EU966738	B8ASQ8	-
Os04g0357700	Hypothetical conserved gene.	AK250527	NP_001052546.2	-
Os04g0358000	Similar to OSIGBa0104J13.1 protein.	AK287896	Q01KT9	Alcohol dehydrogenase superfamily, zinc-type
Os04g0358150	Hypothetical protein.	EU945918	longestORF	-
Os04g0360400	Hypothetical protein.	EU945918	longestORF	-
Os04g0362501	Hypothetical conserved gene.	AK074006	Q7XMF4	-
Os04g0375400	Similar to Calmodulin.	Y13974	B1NDI5	EF-hand-like domain
Os04g0377500	Non-protein coding transcript.	CT834365	NONE	-
Os04g0383100	Conserved hypothetical protein.	ab initio prediction	NP_001173906.1	-
Os04g0386500	Similar to OSIGBa0148P16.4 protein.	AK070265	Q01L01	Uncharacterised protein family UPF0250
Os04g0386600	Pyruvate/Phosphoenolpyruvate kinase, catalytic core domain containing protein.	AK067193	Q01L00	Isocitrate lyase/phosphorylmutase
Os04g0386650	Hypothetical gene.	BT016201	longestORF	-
Os04g0386700	Similar to OSIGBa0148P16.6 protein.	AK240915	Q01KZ9	-
Os04g0386900	Transcriptional factor B3 family protein.	AK119999	Q01KZ7	Transcriptional factor B3
Os04g0387000	Hypothetical conserved gene.	ab initio prediction	A3AT78	-
Os04g0387900	Similar to AT.I.24-6 protein (Fragment).	AK063212	Q01LF5	Protein of unknown function DUF3339
Os04g0395600	Similar to TRANSPORT INHIBITOR RESPONSE 1 protein (F-box/LRR-repeat protein 1).	AK100862	XP_002328871.1	F-box domain, cyclin-like
Os04g0395651	Hypothetical protein.	tplb0032b19 (Wheat FLcDNA)	longestORF	-
Os04g0397100	Similar to ClpC protease.	AK058510	E5GBL8	Chaperonin ClpA/B
Os04g0397400	Conserved hypothetical protein.	AK059476	Q7F9I0	-
Os04g0397601	Non-protein coding transcript.	CT835050	NONE	-
Os04g0398700	Similar to Pollen specific protein C13 precursor.	AK375503	Q01L34	Allergen Ole e 1, conserved site
Os04g0402700	Hypothetical protein.	AK058995	longestORF	-
Os04g0403200	Zinc finger, RING-type domain containing protein.	AK120702	B8Q8A9	Zinc finger, RING-type
Os04g0403300	Similar to Alpha-amylase.	CT836492	C3W8N1	Alpha-amylase, C-terminal beta-sheet
Os04g0403400	Tyrosyl-DNA phosphodiesterase family protein.	AK120071	B9FEZ9	Tyrosyl-DNA phosphodiesterase
Os04g0403450	Non-protein coding transcript.	BT086661	NONE	-
Os04g0403600	Conserved hypothetical protein.	tplb0006a06 (Wheat FLcDNA)	NP_001052700.2	-
Os04g0405000	Similar to ATP-dependent Clp protease ATP-binding subunit.	AK061666	Q01L44	-
Os04g0405100	Similar to H0502B11.8 protein.	AK288033	Q01L43	Sterile alpha motif domain
Os04g0405150	Hypothetical gene.	BT019295	longestORF	-
Os04g0405700	Similar to H0502B11.13 protein.	tplb0022b18 (Wheat FLcDNA)	Q01L38	Protein of unknown function DUF677
Os04g0405800	Similar to OSIGBa0142C11.1 protein.	AK108757	Q01L58	Aspartate/glutamate/uridylate kinase
Os04g0406600	Prephenate dehydratase domain containing protein.	AK103609	Q01L56	Prephenate dehydratase
Os04g0407850	Similar to H0321H01.4 protein.	EU942896	Q01L52	-
Os04g0407900	Similar to Cytochrom P450-like protein.	AK064758	Q01L52	Cytochrome P450
Os04g0411800	Similar to H0717B12.5 protein.	AK102032	Q01JB7	-
Os04g0414300	Similar to H0622F05.1 protein.	EU965676	Q01L95	RNA recognition motif domain
Os04g0414400	Non-protein coding transcript.	AK058617	NONE	-
Os04g0414500	Similar to H0622F05.2 protein.	AK121479	Q01L94	-
Os04g0416000	Protein of unknown function DUF1279 domain containing protein.	AK241337	Q01I48	Domain of unknown function DUF1279
Os04g0416100	Similar to Transcription factor E2F1 (E2F-1) (Fragment).	AK067581	Q01I47	Transcription factor E2F/dimerisation partner
Os04g0418000	Conserved hypothetical protein.	AK106155,AK061030,AK099124	Q7X6C3	-
Os04g0418100	Hypothetical conserved gene.	CT835067	B9FF49	-
Os04g0420700	Hypothetical protein.	tplb0028c09 (Wheat FLcDNA)	longestORF	-
Os04g0420900	Similar to H0525E10.7 protein.	AK064414	Q01K01	S-locus glycoprotein
Os04g0421700	FAR1 domain containing protein.	AK072998	Q01JZ9	FAR1 DNA binding domain
Os04g0421800	EF-Hand type domain containing protein.	AK068951	Q01JZ8	EF-hand-like domain
Os04g0421850	Hypothetical protein.	EU949573	longestORF	-
Os04g0435700	Similar to UVB-resistance protein UVR8.	AK100857	A2XTP3	Regulator of chromosome condensation, RCC1
Os04g0436000	Conserved hypothetical protein.	AK372391	NP_001052850.2	-
Os04g0436300	Protein disulphide isomerase isoform/multifunctional endoplasmic reticulum luminal polypeptide (D	AK101074	B9FFA7	Thioredoxin
Os04g0436700	Similar to H0315A08.1 protein.	FP099421	Q01I63	Ribonuclease H1, N-terminal
Os04g0436800	Pentatricopeptide repeat domain containing protein.	AK101115	Q01I62	Pentatricopeptide repeat
Os04g0437000	No apical meristem (NAM) protein domain containing protein.	AK243585	Q01I69	No apical meristem
Os04g0437300	Zinc finger, RING/FYVE/PHD-type domain containing protein.	AK121647	Q01I67	U box domain
Os04g0437401	Non-protein coding transcript.	X02683	NONE	-
Os04g0437500	Tetratricopeptide-like helical domain containing protein.	AK110861	Q01I66	Tetratricopeptide TPR-1
Os04g0437600	Mitogen activated protein kinase kinase kinase 3 domain containing protein.	AK068725	Q01I65	Protein kinase, catalytic domain
Os04g0437700	Hypothetical protein.	AK111449	longestORF	-
Os04g0439900	Similar to Translocon Tic40 precursor.	AK065119	Q01K76	Heat shock chaperonin-binding
Os04g0440000	Protein of unknown function DUF2040, coiled-coil domain containing protein.	AK102407,AK101691	Q01K75	Domain of unknown function DUF2040
Os04g0459500	Similar to H0219H12.1 protein.	AK067817	Q01IJ6	Glyceraldehyde 3-phosphate dehydrogenase, catalytic domain
Os04g0459600	Mog1/PsbP, alpha/beta/alpha sandwich domain containing protein.	AK061516	Q01IJ5	Ran-interacting Mog1 protein
Os04g0459700	Similar to H0219H12.3 protein.	AK109192	Q01IJ4	Nucleosome assembly protein
Os04g0459800	Similar to H0219H12.4 protein.	AK073573	Q01IJ3	Helicase, C-terminal
Os04g0459900	Peptidase, trypsin-like serine and cysteine domain containing protein.	AK065850	Q01IJ2	Peptidase S1/S6, chymotrypsin/Hap
Os04g0460050	Hypothetical conserved gene.	AB332064	F2QA89	-

Os04g0460350	Similar to predicted protein.	CT836521	B6U9R4	-
Os04g0460400	Uncharacterised protein family UPF0497, trans-membrane plant subgroup domain containing protein	FP091536	GeneMark	Uncharacterised protein family UPF0497, trans-membrane plant subgroup
Os04g0460500	Similar to H0219H12.9 protein.	BT037263	Q01II8	Molybdopterin cofactor biosynthesis C
Os04g0460750	Non-protein coding transcript.	X02683	NONE	-
Os04g0460900	Non-protein coding transcript.	AK066716,AK065774,AK104026	NONE	-
Os04g0461050	Hypothetical protein.	EU971393	longestORF	-
Os04g0461100	Ribosomal protein, PSRP-3/Ycf65 domain containing protein.	AK068022	Q01II5	Ribosomal protein PSRP-3/Ycf65
Os04g0461700	Non-protein coding transcript.	AK120338	NONE	-
Os04g0461800	Actin-binding FH2 domain containing protein.	AK106770	Q7XUV2	Actin-binding FH2/DRF autoregulatory
Os04g0461900	Hypothetical protein.	AK064527	longestORF	-
Os04g0462300	Conserved hypothetical protein.	AK109651	B8AUR5	-
Os04g0462500	Similar to 14-3-3-like protein GF14-6.	AK101938	Q7XTE8	14-3-3 protein
Os04g0462550	Hypothetical gene.	BT017754	longestORF	-
Os04g0463000	Serine/threonine protein kinase domain containing protein.	AK120857	Q25A94	Protein kinase, catalytic domain
Os04g0463200	Protein of unknown function DUF593 family protein.	AK243661	NP_001053008.1	Protein of unknown function DUF593
Os04g0463300	Pentatricopeptide repeat containing protein.	AK119417	Q6MWD7	Pentatricopeptide repeat
Os04g0463400	Protein of unknown function DUF125, transmembrane family protein.	AK059730	C4B8E4	Domain of unknown function DUF125, transmembrane
Os04g0467550	Non-protein coding transcript.	BT017430	NONE	-
Os04g0467700	Similar to Indole-3-glycerol phosphate synthase, chloroplast precursor (EC 4.1.1.48) (IGPS).	AK101835	Q01IS1	Indole-3-glycerol phosphate synthase, conserved site
Os04g0467901	Similar to OSIGBa0128P10.5 protein.	AK374065	Q01IR9	Calponin homology domain
Os04g0469000	Heavy metal transport/detoxification protein domain containing protein.	AK062816	Q01IL4	-
Os04g0481800	Membrane bound O-acyl transferase, MBOAT family protein.	AK109152	Q25AJ4	Membrane bound O-acyl transferase, MBOAT
Os04g0482000	Similar to Peptide methionine sulfoxide reductase.	AK064801	Q7XUP7	Peptide methionine sulphoxide reductase MsrA
Os04g0482300	Kelch related domain containing protein.	AK064143	Q25AK5	Zinc finger, TAZ-type
Os04g0482700	Similar to H0525D09.10 protein.	AK111072	Q01JV5	Peptidase M76, ATP23
Os04g0482800	Similar to Topoisomerase-like protein.	AK068497	Q01JV4	Initiation factor eIF-4 gamma, MA3
Os04g0483400	Similar to H0805A05.2 protein.	AK063531	Q01IG4	-
Os04g0507400	Similar to OSIGBa0157A06.5 protein.	EU957725	Q01IV4	Transferase
Os04g0507450	Hypothetical gene.	BT035701	longestORF	-
Os04g0507800	UbiE/COQ5 methyltransferase family protein.	AK111060	Q01I80	UbiE/COQ5 methyltransferase
Os04g0509100	Similar to triacylglycerol lipase.	AK359843	NP_193091.5	C2 calcium-dependent membrane targeting
Os04g0509400	RmlC-like jelly roll fold domain containing protein.	AK240893	Q01HF4	Domain of unknown function DUF861, cupin-3
Os04g0509600	Similar to Ammonium transporter 1 member 1.	AK073718	Q7XQ12	Ammonium transporter
Os04g0510200	Similar to STYLOSA protein.	AK108706	Q01HE9	LisH dimerisation motif
Os04g0511200	EFA27 for EF hand, abscisic acid, 27kD.	AK243204	Q40679	Caleosin
Os04g0511500	Hypothetical protein.	EU940776	longestORF	-
Os04g0511600	Similar to RING-H2 finger protein ATL3G.	AK073609	Q01HE0	Zinc finger, RING-type
Os04g0511700	Similar to OSIGBa0157K09-H0214G12.18 protein.	AK102761	Q01HD9	-
Os04g0512225	Non-protein coding transcript.	FP092318	NONE	-
Os04g0512250	Hypothetical gene.	AK288761	longestORF	-
Os04g0512300	Arp2/3 complex, 34kDa subunit p34-Arc family protein.	AK071791	Q01HD5	Arp2/3 complex, 34kDa subunit p34-Arc
Os04g0512400	Similar to Midline 1 protein (Tripartite motif protein 18).	AK103203	Q01HD4	BRCT
Os04g0512900	NB-ARC domain containing protein.	AK067204	Q7XPY8	Disease resistance protein
Os04g0513000	Conserved hypothetical protein.	AK102028	B9FG47	-
Os04g0513700	Similar to Beta-glucosidase 16.	BT039444	Q7XSK2	Glycoside hydrolase, family 1
Os04g0513800	Hypothetical protein.	tplb0059k05 (Wheat FLcDNA)	longestORF	-
Os04g0514500	Similar to OSIGBa0140J09.3 protein.	AK073502	Q01I18	Pentatricopeptide repeat
Os04g0514600	Similar to NBS-LRR type disease resistance protein O2 (Fragment).	AK365389	O81527	Disease resistance protein
Os04g0516550	Conserved hypothetical protein.	AK333114	NP_001047774.1	-
Os04g0516600	Pyridoxal phosphate-dependent transferase, major region, subdomain 1 domain containing protein.	AK059652,AK099280	Q01J56	Aromatic amino acid beta-eliminating lyase/threonine aldolase
Os04g0516701	Hypothetical gene.	BT017119	longestORF	-
Os04g0516800	Hypothetical protein.	AK105554	longestORF	-
Os04g0516900	Conserved hypothetical protein.	AK108714	Q7XKW4	-
Os04g0517100	Similar to OSIGBa0145M07.4 protein.	AK111798	Q01J53	Homeodomain-like
Os04g0517300	Protein of unknown function DUF647 family protein.	AK069879	Q01J52	Protein of unknown function DUF647
Os04g0517500	Similar to Phosphoenolpyruvate carboxylase kinase.	AK066885	Q2ABP5	Protein kinase, catalytic domain
Os04g0517900	Auxin responsive SAUR protein family protein.	AK241307	B5RHT9	Auxin responsive SAUR protein
Os04g0518000	Similar to Adenosine kinase (Fragment).	AK101926	Q01J47	Adenosine kinase
Os04g0518100	Phenylalanine ammonia-lyase.	AK100346	A2XVK1	Phenylalanine/histidine ammonia-lyase
Os04g0518400	Similar to Phenylalanine ammonia-lyase (Fragment).	AK067801	A2XVK3	Phenylalanine/histidine ammonia-lyase
Os04g0518600	Similar to OSIGBa0102I15.5 protein.	AK063087	Q01IW5	-
Os04g0518650	Similar to OSIGBa0102I15.5 protein.	AK242408	Q01IW5	Small subunit of serine palmitoyltransferase-like
Os04g0518800	Similar to Lysine decarboxylase-like protein.	AK069293	Q8RUN2	Cytokinin riboside 5&apos%3B-monophosphate phosphoribohydrolase LOG
Os04g0519200	Similar to OSIGBa0132O24.3 protein.	EU951279	Q01MB4	-
Os04g0519700	Similar to Auxin response factor 10.	AK100795	Q7XKK6	Transcriptional factor B3
Os04g0519900	Similar to OSIGBa0145G11.5 protein.	AK071665	Q01I34	-
Os04g0519912	Hypothetical protein.	tplb0025b01 (Wheat FLcDNA)	longestORF	-
Os04g0519925	Similar to OSIGBa0145G11.5 protein.	ab initio prediction	Q01I34	Peptidase M28
Os04g0519950	Non-protein coding transcript.	EU954591	NONE	-
Os04g0519975	Similar to photosystem I subunit IX.	EU954469	YP_784493.1	Photosystem I PsaJ, reaction centre subunit IX
Os04g0520000	S1, RNA binding domain containing protein.	AK102988	Q01I33	RNA-binding domain, S1
Os04g0520700	Protein of unknown function DUF584 family protein.	AK065832	Q01IB5	Protein of unknown function DUF584
Os04g0520900	Pentatricopeptide repeat domain containing protein.	AK068793	Q01IB9	Pentatricopeptide repeat
Os04g0521001	Hypothetical protein.	FP096444	longestORF	-
Os04g0521100	Major intrinsic protein family protein.	AK243592	Q01IB8	Major intrinsic protein
Os04g0521300	Conserved hypothetical protein.	AK071350	B9FG70	-
Os04g0521700	Similar to OSIGBa0131L05.9 protein.	AK073622	Q01IB2	-
Os04g0521800	Peptidase M20 family protein.	AK102934,AK061536	Q01IB1	Peptidase M20
Os04g0521900	Similar to Vegetative cell wall protein gp1.	AK105173	B6SY74	-
Os04g0522100	Similar to OSIGBa0131L05.12 protein.	tplb0012i18 (Wheat FLcDNA)	Q01IA9	Knottin
Os04g0523200	Up-frameshift suppressor 2 domain containing protein.	AK119440	B9FG77	Up-frameshift suppressor 2
Os04g0523350	Similar to Initiation factor eIF-4 gamma, middle%3B Up-frameshift suppressor 2.	EU942804	Q2HSQ9	MIF4-like, type 1/2/3
Os04g0523812	Conserved hypothetical protein.	EU964909	B9FG79	-
Os04g0524300	InterPro=Signal transduction response regulator, receiver domain (IPR001789),CheY-like superfami	Biological Process: two-componen	NIAS_FLcDNA=002-1(	-
Os04g0524400	Similar to OSIGBa0153E02-OSIGBa0093I20.13 protein.	AK103349	Q01HR2	Protein of unknown function DUF3411
Os04g0524500	Oligopeptide transporter OPT superfamily protein.	AK067235	Q01HR1	Oligopeptide transporter OPT superfamily
Os04g0524800	Hypothetical protein.	AK064753	longestORF	-
Os04g0524900	Similar to OSIGBa0153E02-OSIGBa0093I20.16 protein.	AB190921	Q01HQ9	Oligopeptide transporter OPT superfamily
Os04g0524950	Hypothetical protein.	ab initio prediction	NONE	-
Os04g0525000	Protein of unknown function DUF177 domain containing protein.	AK067753	Q01HQ8	Protein of unknown function DUF177
Os04g0525100	UDP-glucuronosyl/UDP-glucosyltransferase family protein.	AK109806	Q01HQ7	UDP-glucuronosyl/UDP-glucosyltransferase
Os04g0525600	ATP-dependent Clp protease proteolytic subunit (EC 3.4.21.92) (Endopeptidase Clp).	AK121934	Q01HQ5	Peptidase S14, ClpP
Os04g0525700	Similar to Serine carboxypeptidase II precursor (EC 3.4.16.6) (Carboxypeptidase D) (Bri1 suppresso	AK105830	Q01HQ4	Peptidase S10, serine carboxypeptidase
Os04g0525800	Similar to OSIGBa0115K01-H0319F09.1 protein.	ab initio prediction	Q01HQ1	Isochorismatase-like
Os04g0525850	Conserved hypothetical protein.	EU971861	NP_001144947.1	-
Os04g0526300	Sulfotransferase family protein.	ab initio prediction	Q01HP8	Sulfotransferase domain
Os04g0526800	GRAM domain containing protein.	AK061581	Q01HP4	GRAM
Os04g0527000	GRAM domain containing protein.	AK099545,AK070324,AK104433	Q01HP3	-
Os04g0527300	Non-protein coding transcript.	AK110653	NONE	-
Os04g0527400	BRO1 domain containing protein.	AK107295	Q01HP2	BRO1 domain
Os04g0527500	Similar to OSIGBa0115K01-H0319F09.11 protein.	AK059144	Q01HP1	Mitochondrial carrier domain
Os04g0527700	CHCH domain containing protein.	AK072980	Q01HP0	-
Os04g0527800	Similar to OSIGBa0115K01-H0319F09.13 protein.	AK104019	Q01HN9	-
Os04g0527900	Similar to Tonoplast membrane integral protein ZmTIP3-2.	AK108116	Q01HN8	Major intrinsic protein
Os04g0528000	Similar to OSIGBa0115K01-H0319F09.15 protein.	BT018594	Q01HN7	Protein of unknown function DUF789
Os04g0528100	Similar to OSIGBa0115K01-H0319F09.16 protein.	AK065957	Q01HN6	-
Os04g0528200	Similar to OSIGBa0115K01-H0319F09.17 protein.	AK064693	Q01HN5	-
Os04g0528300	Similar to OSIGBa0115K01-H0319F09.18 protein.	AK058387	Q01HN4	ABC-2 type transporter
Os04g0528400	Similar to ABC transporter.	AK064120	Q01HN3	Hus1-like protein
Os04g0528600	Similar to OSIGBa0115K01-H0319F09.22 protein.	AK240918	Q01HN0	-
Os04g0528651	Hypothetical protein.	tplb0055d11 (Wheat FLcDNA)	longestORF	-
Os04g0528800	Similar to Ferredoxin-thioredoxin reductase, variable chain (FTR-V) (Ferredoxin- thioredoxin reduc	AK103671	Q01HM9	Lipoate synthase
Os04g0529100	Pathogenesis-related transcriptional factor and ERF domain containing protein.	AK107680	Q01HM7	Pathogenesis-related transcriptional factor/ERF, DNA-binding
Os04g0529500	Similar to cDNA clone:J023022A09, full insert sequence.	AK059419	B7EGJ9	Double-stranded RNA-binding
Os04g0529700	Glycosyltransferase sugar-binding region containing DXD motif domain containing protein.	AK121532	Q00RJ5	Glycosyltransferase, DXD sugar-binding motif

Os04g0529800	Sugar/inositol transporter domain containing protein.	AK069679	Q00RJ4	Sugar/inositol transporter
Os04g0530050	Hypothetical protein.	ab initio prediction	NONE	-
Os04g0530400	t-snare domain containing protein.	AK067634	B6TDT7	SNARE-complex protein Syntaxin-18 N-terminal
Os04g0530801	Hypothetical gene.	EU973535	longestORF	-
Os04g0530900	Glycosyl transferase, family 8 protein.	AK072297	XP_002312381.1	Glycosyl transferase, family 8
Os04g0531200	Hypothetical conserved gene.	AB332066	A2XVT7	-
Os04g0531300	tRNA-dihydrouridine synthase domain containing protein.	AK103911	Q00RI1	tRNA-dihydrouridine synthase
Os04g0531500	Concanavalin A-like lectin/glucanase domain containing protein.	AK102285	Q00RH9	Protein kinase, catalytic domain
Os04g0531600	Hypothetical protein.	AK107678	longestORF	-
Os04g0531750	Similar to OSIGBa0125M19.13 protein.	ab initio prediction	Q00RH3	Short-chain dehydrogenase/reductase SDR
Os04g0531800	Conserved hypothetical protein.	AK121355	B8ASD8	-
Os04g0531900	Short-chain dehydrogenase/reductase SDR domain containing protein.	AK071069	Q00RH3	Short-chain dehydrogenase/reductase SDR
Os04g0532100	Short-chain dehydrogenase/reductase SDR domain containing protein.	AK109281	Q00RH5	Short-chain dehydrogenase/reductase SDR
Os04g0533200	Similar to Myb7 protein (Fragment).	AK376409	Q43598	-
Os04g0533250	Hypothetical gene.	EU971393	longestORF	-
Os04g0533500	Cytochrome b561 family protein.	AK061426,AK069219	Q01II4	-
Os04g0533800	Similar to OSIGBa0159I10.4 protein.	BT040291	Q01II1	Protease inhibitor I4, serpin
Os04g0534166	Similar to OSIGBa0159I10.8 protein.	ab initio prediction	Q01IH7	Leucine-rich repeat-containing N-terminal, type 2
Os04g0534200	Protein kinase, core domain containing protein.	AK109583	Q01IH7	Protein kinase, catalytic domain
Os04g0534500	Similar to OSIGBa0159I10.11 protein.	AK105805	Q01IH4	-
Os04g0534600	Peroxisomal biogenesis factor 11 family protein.	AK073835,AK287635	Q7XU74	Peroxisomal biogenesis factor 11
Os04g0535200	Peptidase A1 domain containing protein.	AK101505,AK060585,AK119767	Q01IH2	-
Os04g0535600	Similar to Beta-fructofuranosidase 1 precursor (EC 3.2.1.26) (Sucrose 1) (Invertase 1).	AK099312,AK065130	Q01IH0	Glycoside hydrolase, family 32
Os04g0536300	Transcription factor with zinc finger domain and helix-loop-helix domain (YABBY domain), Leaf de	AK070205,AB274017	B7EHI2,Q0JBF0	-
Os04g0536500	Similar to H0502G05.5 protein.	AK109552	Q01JG1	No apical meristem
Os04g0537450	Non-protein coding transcript.	CT835085	NONE	-
Os04g0538166	Similar to Elongation factor G, chloroplastic.	P35450	P35450	Protein synthesis factor, GTP-binding
Os04g0538300	Hypothetical conserved gene.	ab initio prediction	NP_001174027.1	-
Os04g0538400	Similar to Nodulin 21 (N-21).	AK108230	Q9M2C3	Domain of unknown function DUF125, transmembrane
Os04g0538700	Autophagy-related protein 13 domain containing protein.	AK105628	Q7XU21	Autophagy-related protein 13
Os04g0538900	Glyoxalase/bleomycin resistance protein/dioxygenase domain containing protein.	AK333604	NP_001053434.1	-
Os04g0539000	Protein of unknown function DUF2485 domain containing protein.	AK105343	Q7FB12	NAD
Os04g0539100	Conserved hypothetical protein.	AK069820	A2XVZ2	-
Os04g0539300	Similar to Aldehyde 5-hydroxylase.	AK248286	Q9SWR1	Cytochrome P450
Os04g0539500	Similar to H0115B09.1 protein.	AK060574,AK099144	Q01IQ1	Zinc finger, GATA-type
Os04g0539601	Hypothetical gene.	EU947136	longestORF	-
Os04g0539701	Conserved hypothetical protein.	CT835025	NP_001060202.1	-
Os04g0539800	Src homology-3 domain containing protein.	AK240927	Q01IQ0	Src homology-3 domain
Os04g0540200	Zinc finger, B-box domain containing protein.	AK061333	Q01IP9	Zinc finger, B-box
Os04g0540300	COBRA-like protein 7.	Q7XR91	Q7XR91	Glycosyl-phosphatidyl inositol-anchored, plant
Os04g0540401	Hypothetical protein.	tplb0046p06 (Wheat FLcDNA)	longestORF	-
Os04g0540900	Serine/threonine protein kinase domain containing protein.	AK066397	Q01IP5	Protein kinase, catalytic domain
Os04g0542100	Similar to HAT family dimerisation domain containing protein.	AK288170	Q53RM1	HAT dimerisation
Os04g0586200	Similar to H0307D04.13 protein.	AK120315	Q01K53	Protein of unknown function DUF581
Os04g0590283	Hypothetical protein.	BT019283	longestORF	-
Os04g0602200	Bacterial extracellular solute-binding protein, family 1 protein.	AK120467	Q01HT2	Bacterial extracellular solute-binding, family 1
Os04g0602250	Non-protein coding transcript.	EU946899	NONE	-
Os04g0602500	Similar to Pectin acetylesterase.	AK073958	Q01HS9	Pectinacetylesterase
Os04g0602600	Pentatricopeptide repeat domain containing protein.	AK120600	Q01HS8	Pentatricopeptide repeat
Os04g0602700	RNA recognition motif, RNP-1 domain containing protein.	EU959252	Q01HS7	RNA recognition motif domain
Os04g0602800	Similar to Yarrowia lipolytica chromosome D of strain CLIB99 of Yarrowia lipolytica.	AK100925	Q01HS6	Protein kinase, catalytic domain
Os04g0602900	Similar to cDNA clone:J023087M06, full insert sequence.	AK065982	B7EQE6	Isoprenylcysteine carboxyl methyltransferase
Os04g0605100	WRKY transcription factor 68.	AK061266,AK072938	B6U7T4	DNA-binding WRKY
Os04g0605200	Protein-tyrosine phosphatase, low molecular weight domain containing protein.	AK108075	XP_002873471.1	Peptidyl-tRNA hydrolase, PTH2
Os04g0609700	Similar to H0702G05.10 protein.	AK058541	Q25AI3	-
Os04g0610500	Peptidase M24A, methionine aminopeptidase, subfamily 1 protein.	AK066289,AK104817	B8ATX6	Peptidase M24, structural domain
Os04g0610700	Similar to Potassium transporter 13 (AtPOT13) (AtKT5).	AK100672	Q01JS6	K+ potassium transporter
Os04g0611267	Similar to OSIGBa0152L12.11 protein.	ab initio prediction	Q01JS3	-
Os04g0613500	Similar to 40S ribosomal protein S11.	D29727	NP_001105562.1	Ribosomal protein S17
Os04g0639800	Similar to OSIGBa0138H21-OSIGBa0138E01.9 protein.	AK243114	Q01HX4	-
Os04g0656100	Similar to Plasma membrane H+-ATPase-like protein (Fragment).	AK100435	Q01KM8	ATPase, P-type, H+ transporting proton pump
Os04g0664400	Auxin response factor 1.	AK103452	Q8S983	AUX/IAA protein
Os04g0665900	Similar to H1005F08.13 protein.	FP097147	Q01MN1	VQ
Os04g0668000	Conserved hypothetical protein.	AK062740	Q7XR77	-
Os04g0668700	Similar to phosphatidylinositol 3- and 4-kinase family protein.	AK062068	NP_001151804.1	Phosphatidylinositol 3-/4-kinase, catalytic
Os04g0668900	Similar to Plastid terminal oxidase.	AK067891	B8AW16	Alternative oxidase
Os04g0669300	EF hand domain containing protein.	AK071148	NP_001030731.1	EPS15 homology
Os04g0669375	Non-protein coding transcript.	tplb0032i10 (Wheat FLcDNA)	NONE	-
Os04g0669600	Phospholipase/carboxylesterase domain containing protein.	AK110767	Q259P1	Phospholipase/carboxylesterase/thioesterase
Os04g0670000	Reticulon family protein.	AK071792	Q259P4	Reticulon
Os04g0670200	Granulin domain containing protein.	AK099358	GeneMark	Granulin
Os04g0670900	Homeodomain-like containing protein.	DQ383374	Q258Z1	MYB-like
Os04g0671100	Similar to H0624F09.8 protein.	AK241125	Q258Z0	Adenylate kinase
Os04g0671300	Similar to Suppressor of presenilin 5 (P110b homolog).	AK072414	Q258Y8	Flavin amine oxidase
Os04g0671500	Hypothetical protein.	tplb0048c15 (Wheat FLcDNA)	longestORF	-
Os05g0102300	HAT dimerisation domain containing protein.	AK106387	longestORF	HAT dimerisation
Os05g0119700	Conserved hypothetical protein.	AK062482	Q9LIW7	-
Os05g0119850	Hypothetical protein.	tplb0022d09 (Wheat FLcDNA)	longestORF	-
Os05g0127266	Non-protein coding transcript.	AK248585	NONE	-
Os05g0127300	Serine/threonine protein kinase domain containing protein.	AK121766	B9FGM2	Protein kinase, catalytic domain
Os05g0127400	Similar to DnaJ subfamily B member 13.	AK069823	B6TKS5	Heat shock protein DnaJ, N-terminal
Os05g0127450	Non-protein coding transcript.	tplb0026i01 (Wheat FLcDNA)	NONE	-
Os05g0129700	KNOX class homeodomain protein (Knotted1-type homeobox protein OSH71).	AK111878	Q7GDL5	Homeobox
Os05g0129800	DNA-binding WRKY domain containing protein.	AK241799	NP_001174199.1	DNA-binding WRKY
Os05g0129900	Tetratricopeptide-like helical domain containing protein.	AK060436	B9FMA1	Tetratricopeptide TPR-1
Os05g0138300	Similar to ICT protein (Fragment).	AK119249	Q9SXZ4	-
Os05g0142400	Conserved hypothetical protein.	AK062395	A2Y0A2	-
Os05g0142500	Hypothetical conserved gene.	ab initio prediction	A2Y0A3	-
Os05g0143300	Conserved hypothetical protein.	AK072841,AK099030	B9FH60	-
Os05g0143500	Similar to Kinase (Fragment).	AK061851	C4PE55	-
Os05g0143600	Similar to Jasmonate-induced protein.	AK107844	A2Y0B0	Mannose-binding lectin
Os05g0144100	WD40 repeat-like domain containing protein.	AK069280	B6U179	WD40 repeat
Os05g0145400	Fibronectin, type III-like fold domain containing protein.	AK106860	A0SQ42	Fibronectin, type III
Os05g0145500	Hypothetical protein.	tplb0048h04 (Wheat FLcDNA)	longestORF	-
Os05g0145600	Pentatricopeptide repeat containing protein.	AK063945,AK101887	NP_567990.1	-
Os05g0145650	Hypothetical gene.	BT019052	longestORF	-
Os05g0145700	Peptidase, trypsin-like serine and cysteine domain containing protein.	AK069656	B9FH68	Peptidase cysteine/serine, trypsin-like
Os05g0149300	Similar to 1-aminocyclopropane-1-carboxylate oxidase.	BT087584	O81606	Oxoglutarate/iron-dependent oxygenase
Os05g0149400	Similar to 1-aminocyclopropane-1-carboxylic acid oxidase.	AK061064	O81606	Oxoglutarate/iron-dependent oxygenase
Os05g0149450	Non-protein coding transcript.	tplb0008n13 (Wheat FLcDNA)	NONE	-
Os05g0149600	Similar to CULLIN1-like protein 1.	AK073947	C7SJ62	-
Os05g0149950	Monothiol glutaredoxin-S8.	P0C290	P0C290	Glutaredoxin
Os05g0150550	Hypothetical protein.	tplb0032b19 (Wheat FLcDNA)	longestORF	-
Os05g0150600	Hypothetical conserved gene.	AK063338	D7U9T8	DNA helicase, ATP-dependent, RecQ type
Os05g0150900	Histidyl-tRNA synthetase (EC 6.1.1.21) (Histidine--tRNA ligase) (HisRS).	AK099789	P93422	Phenylalanine/histidine ammonia-lyase
Os05g0151100	Similar to desiccation-related protein PCC13-62.	EU967146	B6TNA6	-
Os05g0151150	Hypothetical gene.	EU975566	GeneMark	-
Os05g0151200	TspO/MBR-related protein family protein.	AK240936	NP_001147341.1	TspO/MBR-related protein
Os05g0151400	AIG1 domain containing protein.	AK102924	XP_002874910.1	Chloroplast protein import component Toc86/159
Os05g0152266	Hypothetical gene.	EU943470	longestORF	-
Os05g0159400	Non-protein coding transcript.	EU942788	NONE	-
Os05g0160000	Similar to Ferric leghemoglobin reductase.	AK063865	B9FML1	Pyridine nucleotide-disulphide oxidoreductase, dimerisation
Os05g0160200	Ubiquitin.	AK119731	A6MZJ4	Ubiquitin
Os05g0170250	Hypothetical gene.	AK243238	longestORF	-
Os05g0170300	Leucine-rich repeat domain containing protein.	ab initio prediction	Q65XS3	Leucine-rich repeat

Os05g0176100	Similar to Cellulose synthase BoCesA1.	AK067850	D5FJ45	Cellulose synthase
Os05g0176700	Conserved hypothetical protein.	AK071729	A2Y0X6	-
Os05g0177100	F-box domain, cyclin-like domain containing protein.	AK064652	B9FMR7	F-box domain, cyclin-like
Os05g0178950	Hypothetical protein.	tplb0040d24 (Wheat FLcDNA)	longestORF	-
Os05g0179050	Hypothetical protein.	tplb0030e06 (Wheat FLcDNA)	longestORF	-
Os05g0182650	Conserved hypothetical protein.	AK240762	NP_001174249.1	-
Os05g0182700	Protein of unknown function DUF1664 family protein.	AK065911,AK104343	A2Y139	Protein of unknown function DUF1664
Os05g0182800	Similar to glutaminyl-tRNA synthetase.	AK061951	NP_001152305.1	Glutamyl/glutaminyl-tRNA synthetase, class Ib
Os05g0183100	Similar to WRKY transcription factor 16 (Fragment).	AK066252	B2KJ59	DNA-binding WRKY
Os05g0183200	Similar to barley mlo defense gene homolog6.	ab initio prediction	NP_001105170.1	Mlo-related protein
Os05g0183300	Similar to barley mlo defense gene homolog6.	ab initio prediction	NP_001105170.1	Mlo-related protein
Os05g0184016	Hypothetical protein.	AK372466	longestORF	-
Os05g0184901	Phospholipase A2, active site domain containing protein.	AK287961	B6SGX9	-
Os05g0185650	Similar to predicted protein.	AK242365	XP_002867220.1	-
Os05g0185700	Similar to predicted protein.	AK064724	NP_200842.1	MaoC-like dehydratase
Os05g0185800	Conserved hypothetical protein.	AK065050	A2Y150	-
Os05g0186000	Similar to PSRP4.	AK058675	B6T2D1	-
Os05g0186100	Similar to Histidine-containing phosphotransfer protein 4.	AK240821	B6SY22	Signal transduction histidine kinase, phosphotransfer
Os05g0186601	Hypothetical gene.	EU969101	longestORF	-
Os05g0186900	Similar to Auxin-responsive protein IAA16.	AK111403	P0C127	-
Os05g0187000	Beta 2 subunit of 20S proteasome (20S proteasome beta subunit).	AK103126	A2Y157	Peptidase T1A, proteasome beta-subunit
Os05g0203912	Similar to Histone deacetylase.	tplb0047a04 (Wheat FLcDNA)	A2Z0V8	Peptidase A1
Os05g0217950	Non-protein coding transcript.	tplb0043a14 (Wheat FLcDNA)	NONE	-
Os05g0218001	Myb/SANT-like domain domain containing protein.	BT039927	NP_001176912.1	Myb/SANT-like domain
Os05g0257333	Non-protein coding transcript.	EU949024	NONE	-
Os05g0259601	Non-protein coding transcript.	EU949024	NONE	-
Os05g0267100	Hypothetical conserved gene.	AK065811	Q6ATF1	-
Os05g0268400	Similar to Major facilitator superfamily antiporter.	AK288425	Q8H6D6	-
Os05g0268500	Similar to Serine carboxypeptidase 2.	AK119979	P08819	Peptidase S10, serine carboxypeptidase
Os05g0269200	Similar to inner membrane transport protein.	CT836417	NP_001147344.1	C4-dicarboxylate transporter/malic acid transport protein
Os05g0270000	Ribosomal protein L25 family protein.	AK072793	NP_194093.1	Ribosomal protein L25/Gln-tRNA synthetase, anti-codon-binding domain
Os05g0271300	Hypothetical conserved gene.	Q6ATG6	Q6ATG6	Ribonuclease III
Os05g0272900	B-cell receptor-associated 31-like family protein.	AK107093	B6TG43	B-cell receptor-associated 31-like
Os05g0273500	Conserved hypothetical protein.	AK066374	B8AW21	-
Os05g0274300	Similar to ATP binding protein.	AK071399	NP_001150449.1	-
Os05g0274950	Similar to homogentisate phytyltransferase VTE2-1.	HM212530	NP_001105877.1	-
Os05g0275000	Pentatricopeptide repeat domain containing protein.	AK067135	NP_001157212.1	Pentatricopeptide repeat
Os05g0275100	Pentatricopeptide repeat domain containing protein.	ab initio prediction	B9FNL7	Pentatricopeptide repeat
Os05g0275600	Conserved hypothetical protein.	AK108098	XP_002464690.1	-
Os05g0275700	Similar to Peroxisome assembly protein 2 (Peroxin-2) (AthPEX2) (Pex2p).	AK111642	B4FCL8	Zinc finger, RING-type
Os05g0276500	Expansin Os-EXPA3.	AK060313	Q40637	Expansin
Os05g0276750	Hypothetical protein.	BT016845	longestORF	-
Os05g0277000	Similar to Expansin Os-EXPA3.	P0C1Y4	P0C1Y4	Expansin
Os05g0277100	Hypothetical protein.	BT016845	longestORF	-
Os05g0277300	Similar to Peptide chain release factor 1.	AK065607	B6U6C9	-
Os05g0277500	Similar to Germin-like protein subfamily 2 member 4 precursor.	AK105351	NP_001151047.1	Germin
Os05g0278150	Non-protein coding transcript.	EU949846	NONE	-
Os05g0278550	Hypothetical gene.	EU944389	longestORF	-
Os05g0279850	Hypothetical protein.	tplb0028j17 (Wheat FLcDNA)	longestORF	-
Os05g0279900	Similar to glycoside hydrolase family 28 protein / polygalacturonase (pectinase) family protein.	ab initio prediction	NP_175244.1	Glycoside hydrolase, family 28
Os05g0280200	Similar to Ras-related protein RGP2.	AK120316	A2Y2Q3	Small GTPase superfamily
Os05g0280350	Hypothetical gene.	BT084300	longestORF	-
Os05g0283200	Pectinesterase inhibitor domain containing protein.	AK120717	A2Y2R2	Pectinesterase inhibitor
Os05g0286100	Similar to Zinc-finger protein KNUCKLES.	AK108829	A2Y2R5	Zinc finger, C2H2
Os05g0287800	Similar to Phosphatidic acid phosphatase-like protein.	AK071727	E5FY32	-
Os05g0289400	Similar to CRN (Crooked neck) protein.	AK100478	Q9FJ37	RNA-processing protein, HAT helix
Os05g0289700	Arbuscular mycorrhizal specific marker 10.,Benzyl alcohol benzoyl transferase.	BT086008	Q01IV4	Transferase
Os05g0290000	Hypothetical protein.	BT035701	longestORF	-
Os05g0291600	Hypothetical conserved gene.	AK073226	NP_001059231.1	-
Os05g0292800	Similar to One helix protein (OHP).	AK059562	Q8LPV2	Chlorophyll a/b binding protein domain
Os05g0294800	Hypothetical gene.	AK065926	longestORF	-
Os05g0295200	Conserved hypothetical protein.	AK073450	B9FNQ1	-
Os05g0295300	Similar to Acetyl-coenzyme A carboxylase (EC 6.4.1.2).	AK059261	O48959	Carboxyl transferase
Os05g0295500	Non-protein coding transcript.	BT019212	NONE	-
Os05g0295700	Mitochondrial matrix Mmp37 domain containing protein.	AK100560	B9FNQ2	Mitochondrial matrix Mmp37
Os05g0296600	Non-protein coding transcript.	AK059372	NONE	-
Os05g0296750	Hypothetical gene.	AK063686	longestORF	-
Os05g0296900	Hypothetical conserved gene.	AK100725	B8AWA5	-
Os05g0297600	Non-protein coding transcript.	AK367245	NONE	-
Os05g0297900	Similar to Signal peptidase 18 subunit (Fragment).	AK071238	B6T545	Peptidase S26B, eukaryotic signal peptidase
Os05g0298200	Ankyrin repeat containing protein.	AK066758	A2Y2V7	Ankyrin repeat
Os05g0298300	Hypothetical protein.	tplb0059e11 (Wheat FLcDNA)	longestORF	-
Os05g0298900	Hypothetical protein.	AK105356	longestORF	-
Os05g0299000	Conserved hypothetical protein.	AK108720	B8AWB0	-
Os05g0299101	Hypothetical gene.	CT836471	longestORF	-
Os05g0299200	Hypothetical conserved gene.	EU956990	NP_001055128.1	Nucleotide-sugar transporter
Os05g0299250	Hypothetical protein.	tplb0062m11 (Wheat FLcDNA)	longestORF	-
Os05g0299300	Conserved hypothetical protein.	AK111812	B9FNQ8	-
Os05g0301500	Similar to Ribophorin I (Fragment).	AK070329	NP_001151620.1	Ribophorin I
Os05g0303700	Hypothetical conserved gene.	ab initio prediction	A2Y2Y7	RNA recognition motif domain
Os05g0304501	Hypothetical gene.	CT833082	longestORF	-
Os05g0311075	Non-protein coding transcript.	EU943375	NONE	-
Os05g0328901	Similar to Prolamin.	AK242260	Q5EFA4	-
Os05g0329001	Similar to Prolamin.	AF042201	Q5EFA4	-
Os05g0329200	Similar to Prolamin.	AK242260	Q5EFA4	Bifunctional trypsin/alpha-amylase inhibitor
Os05g0329350	Prolamin.	EF122440	Q5EFA4	Bifunctional trypsin/alpha-amylase inhibitor
Os05g0329700	Similar to Prolamin.	AK242910	Q5EFA4	Bifunctional trypsin/alpha-amylase inhibitor
Os05g0363533	Non-protein coding transcript.	EU948047	NONE	-
Os05g0363566	Hypothetical conserved gene.	CT836447	Q688I1	-
Os05g0375532	Hypothetical gene.	AK287793	longestORF	-
Os05g0375600	Similar to Peptide chain release factor 2.	AK376656	B6SSD2	-
Os05g0377450	Similar to H0315A08.1 protein.	FP099421	Q01I63	Ribonuclease H1, N-terminal
Os05g0377600	Non-protein coding transcript.	EU941912	NONE	-
Os05g0377750	Non-protein coding transcript.	EU941912	NONE	-
Os05g0391400	Non-protein coding transcript.	EU941912	NONE	-
Os05g0392300	Similar to Cyclin-dependent kinase D-1.	AK062401	P29620	-
Os05g0399900	Conserved hypothetical protein.	EU976876	B9FVF5	-
Os05g0408800	Similar to zinc finger, C3HC4 type family protein.	EU964917	NP_001149860.1	Zinc finger, RING-type
Os05g0408850	Hypothetical gene.	CT836350	longestORF	-
Os05g0409500	Similar to MtN21 protein.	BT084636	NP_001150619.1	Drug/metabolite transporter
Os05g0411150	Hypothetical protein.	tplb0038k08 (Wheat FLcDNA)	longestORF	-
Os05g0411300	Similar to DNA binding protein.	AK073142	NP_001148077.1	Basic-leucine zipper
Os05g0413575	Hypothetical gene.	BT016337	longestORF	-
Os05g0435400	Conserved hypothetical protein.	AK109595	A3A6X8	-
Os05g0435700	Similar to (3R)-hydroxymyristoyl-[acyl carrier protein] dehydratase (EC 4.2.1.-) ((3R)-hydroxymyris	AK069677	NP_001149606.1	Beta-hydroxyacyl-
Os05g0435800	Similar to Subtilisin-like protease.	AK288972,AK067138	C7E4J6	Peptidase S8/S53, subtilisin/kexin/sedolisin
Os05g0437300	Nucleotide-binding, alpha-beta plait domain containing protein.	AK102760	D8L9M8	RNA recognition motif domain
Os05g0437800	Conserved hypothetical protein.	AK106328	Q75HX6	-
Os05g0473500	Exo70 exocyst complex subunit family protein.	AK101630	B6U0J9	Exo70 exocyst complex subunit
Os05g0476250	Non-protein coding transcript.	EU951628	NONE	-
Os05g0477666	Hypothetical gene.	BT016845	longestORF	-
Os05g0478250	Hypothetical protein.	AK369221	longestORF	-
Os05g0478300	Hypothetical conserved gene.	AK242838	NP_001055847.2	Protein kinase, catalytic domain
Os05g0486833	Hypothetical conserved gene.	BT065526	C0HHH7	-
Os05g0487300	Conserved hypothetical protein.	AK062926	B8AZG8	-

Os05g0489301	Non-protein coding transcript.	CT836470	NONE	-
Os05g0489800	Centromeric histone 3 (Histone H3).	AK101975	Q6T367	Histone H3
Os05g0491800	Hypothetical protein.	tplb0061j19 (Wheat FLcDNA)	longestORF	-
Os05g0494800	Hypothetical protein.	AK069939	GeneMark	-
Os05g0494900	SH2 motif domain containing protein.	AK106396	NP_001077775.1	SH2 motif
Os05g0495000	Hypothetical conserved gene.	CT829003	B9FKX7	Zinc finger, RING-type
Os05g0495250	Non-protein coding transcript.	CT836397	NONE	-
Os05g0496100	Translation initiation factor eIF3 subunit domain containing protein.	ab initio prediction	NP_001055936.1	Translation initiation factor eIF3 subunit
Os05g0496200	Similar to 3-phosphoglycerate kinase (Fragment).	AK287421	P12782	Phosphoglycerate kinase
Os05g0509101	Conserved hypothetical protein.	AK110690	Q6L4X0	-
Os05g0528101	Hypothetical gene.	EU950506	longestORF	-
Os05g0547200	Conserved hypothetical protein.	AK375772	NP_001047613.2	-
Os05g0547700	Conserved hypothetical protein.	AK070926	B9G5C2	-
Os05g0557800	Similar to 50S ribosomal protein L21.	AK105327	B6TJJ8	Ribosomal protein L21
Os05g0557950	Hypothetical protein.	EU949682	longestORF	-
Os05g0558101	Conserved hypothetical protein.	EU957461	Q6ZJX2	-
Os05g0558501	Conserved hypothetical protein.	EU957461	Q6ZJX2	-
Os05g0565100	Cytochrome b561/ferric reductase transmembrane domain containing protein.	AK063277	B8AWR7	Cytochrome b561, eukaryote
Os05g0565900	DNA-binding WRKY domain containing protein.	FP101665	Q688X5	DNA-binding WRKY
Os05g0566200	NLI interacting factor domain containing protein.	AK099836	B9FLM9	NLI interacting factor
Os05g0566300	PRC-barrel domain containing protein.	AK099641	B9FI59	RimM protein
Os05g0566500	Similar to Initiation factor 3d (Fragment).	AK073468	B6U4Q2	Eukaryotic translation initiation factor 3, subunit 7
Os05g0566800	Cold acclimation protein COR413-TM1.	AK065748	Q84XU8	Cold acclimation WCOR413
Os05g0567100	Similar to Phytepsin.	AK100749	P42210	Peptidase A1
Os05g0567900	Hypothetical conserved gene.	GU722206	B9FLN6	Zinc finger, GATA-type
Os05g0568600	Protein of unknown function DUF1645 family protein.	AK063781	B9FI71	Protein of unknown function DUF1645
Os05g0571100	Peptidase C12, ubiquitin carboxyl-terminal hydrolase 1 domain containing protein.	AK068658	NP_001118908.1	Plant organelle RNA recognition domain
Os05g0595100	Similar to UDP-glucose-4-epimerase.	AK066249	A2Y861	NAD-dependent epimerase/dehydratase
Os05g0595200	Allergen V5/Tpx-1 related family protein.	AK070317	B8AXH6	Allergen V5/Tpx-1-related
Os05g0596000	Peptidase A1 domain containing protein.	AK121242,AK068911	NP_001149154.1	Peptidase A1
Os05g0596200	Similar to cDNA clone:001-104-D04, full insert sequence.	AK107887	B7E7K4	-
Os05g0596500	Similar to RING-H2 finger protein ATL3I (YGHL1-C3HC4 RING fusion protein).	AK070817	XP_002440385.1	Zinc finger, RING-type
Os05g0596700	Similar to cDNA clone:J033067M13, full insert sequence.	AK103507	B7EMM6	Peptidyl-tRNA hydrolase
Os05g0597100	Similar to Nucleolar histone deacetylase HD2-p39.	AK072845	NP_001105631.1	Zinc finger, C2H2
Os05g0597700	Conserved hypothetical protein.	AK121194	A2Y879	-
Os06g0100550	Conserved hypothetical protein.	EU951430	Q65WW4	-
Os06g0122800	Hypothetical conserved gene.	AK289091	NP_001041816.1	Peptidase C48, SUMO/Sentrin/Ubl1
Os06g0131900	Non-protein coding transcript.	EU941912	NONE	-
Os06g0132000	Similar to H0315A08.1 protein.	FP099421	Q01I63	Ribonuclease H1, N-terminal
Os06g0138000	Similar to IMB1.	AK063880	B6UCC7	-
Os06g0140200	Leucine-rich repeat, plant specific containing protein.	AK287701,AK241776,AK287878	Q5VPE8	Leucine-rich repeat
Os06g0159750	Hypothetical conserved gene.	EU964122	Q01I89	-
Os06g0159900	Similar to Arm repeat containing protein.	AK063418	GeneMark	U box domain
Os06g0172800	Similar to alkaline alpha galactosidase 2.	BT061992	NP_001105794.1	Raffinose synthase
Os06g0172901	Hypothetical protein.	tplb0060c15 (Wheat FLcDNA)	longestORF	-
Os06g0173000	Armadillo-type fold domain containing protein.	ab initio prediction	A3B8V4	Armadillo-type fold
Os06g0173100	Similar to 26S protease regulatory subunit 6A homolog (TAT-binding protein homolog 1) (TBP-1) (I	AK065612	P46465	ATPase, AAA+ type, core
Os06g0173450	Hypothetical protein.	BT085228	longestORF	-
Os06g0173800	SANT domain, DNA binding domain containing protein.	EU951889	Q5SNB3	SANT domain, DNA binding
Os06g0174375	Conserved hypothetical protein.	AK242835	NP_001174628.1	-
Os06g0174700	Hypothetical protein.	AK099635	longestORF	-
Os06g0174800	Conserved hypothetical protein.	tplb0014k20 (Wheat FLcDNA)	A2Y9X0	-
Os06g0175800	Similar to Cystathionine beta-lyase, chloroplast precursor (EC 4.4.1.8) (CBL) (Beta-cystathionase) (	AK119521	B4FU01	Cys/Met metabolism, pyridoxal phosphate-dependent enzyme
Os06g0178700	Similar to Iron/ascorbate-dependent oxidoreductase.	AK102979	NP_001152138.1	Oxoglutarate/iron-dependent oxygenase
Os06g0178800	Hypothetical protein.	AK373427	longestORF	-
Os06g0179050	Hypothetical gene.	BT085373	longestORF	-
Os06g0179100	Similar to H0306F03.13 protein.	EU963950	Q25A62	Sterile alpha motif/pointed domain
Os06g0179200	Similar to Nodulin-like protein.	AK101156	Q8H6R3	Nodulin-like
Os06g0179400	Hypothetical protein.	AK103645	longestORF	-
Os06g0179500	Protein of unknown function DUF247, plant family protein.	ab initio prediction	NP_001056969.1	Protein of unknown function DUF247, plant
Os06g0179800	Leucine-rich repeat, N-terminal domain containing protein.	AK121252	A2Y9Z2	Leucine-rich repeat
Os06g0180000	Similar to Root determined nodulation 1.	AK069615	E9KID2	-
Os06g0180050	Hypothetical protein.	tplb0025h12 (Wheat FLcDNA)	longestORF	-
Os06g0180100	Hypothetical conserved gene.	ab initio prediction	Q8H604	-
Os06g0180533	Similar to predicted protein.	EU943442	XP_002321824.1	Cysteine, histidine-dependent amidohydrolase/peptidase
Os06g0181200	Serine/threonine protein kinase domain containing protein.	AK067771	XP_002877531.1	Protein kinase, catalytic domain
Os06g0181400	Similar to oxidoreductase.	AK242975	NP_001152353.1	FAD dependent oxidoreductase
Os06g0181500	Similar to Plasma membrane H+-ATPase.	AK068950	Q8RW25	ATPase, P-type, H+ transporting proton pump
Os06g0181800	Protein of unknown function DUF868, plant domain containing protein.	AK362596	Q5SMI1	Protein of unknown function DUF868, plant
Os06g0188400	Extracellular ligand-binding receptor domain containing protein.	AK065496	B9FRX3	Extracellular ligand-binding receptor
Os06g0188600	Hypothetical conserved gene.	AK242845	Q69KL2	Ionotropic glutamate receptor
Os06g0189600	Protein of unknown function DUF1677, Oryza sativa family protein.	AK109331	B8B3M7	Protein of unknown function DUF1677, Oryza sativa
Os06g0190950	Similar to H0418A01.5 protein.	BT061604	Q01J63	Putative harbinger transposase-derived nuclease
Os06g0198100	Similar to H0315A08.1 protein.	FP099421	Q01I63	Ribonuclease H1, N-terminal
Os06g0198500	RmlC-like jelly roll fold domain containing protein.	EU945799	Q9LMB4	Protein of unknown function DUF985
Os06g0198800	Similar to 25.3 kDa vesicle transport protein.	AK064781	B6TMY9	Longin
Os06g0198850	Non-protein coding transcript.	EU947580	NONE	-
Os06g0199000	Similar to Glycine-rich cell wall structural protein 2 precursor.	AK106381	A2YAD1	-
Os06g0199050	Similar to predicted protein.	EU948644	XP_001779700.1	-
Os06g0199500	Similar to Phosphopantothenoylcysteine decarboxylase (EC 4.1.1.36) (Halotolerance protein Hal3a)	AK100138	C3UZA3	Flavoprotein
Os06g0199601	Non-protein coding transcript.	EU942505	NONE	-
Os06g0200350	Conserved hypothetical protein.	CT836307	B8AJ56	-
Os06g0200500	Hypothetical conserved gene.	ab initio prediction	Q69K48	-
Os06g0201000	Conserved hypothetical protein.	AK108188	A2YAE2	-
Os06g0202100	Conserved hypothetical protein.	AK106373	B8B3R6	-
Os06g0202800	Conserved hypothetical protein.	AK119480	B9FS12	-
Os06g0202825	Non-protein coding transcript.	tplb0055c23 (Wheat FLcDNA)	NONE	-
Os06g0202900	Serine/threonine protein kinase domain containing protein.	AK109607	Q84QD9	Protein kinase, catalytic domain
Os06g0203000	Hypothetical protein.	AK108437	longestORF	-
Os06g0203400	Flavin monooxygenase-like domain containing protein.	AK355934	NP_001159167.1	Flavin monooxygenase-like
Os06g0203550	Non-protein coding transcript.	AK288217	NONE	-
Os06g0204250	Hypothetical protein.	EU955821	longestORF	-
Os06g0205100	Similar to Transcriptional activator.	AK062487	GeneMark	-
Os06g0205600	Similar to mechanosensitive ion channel domain-containing protein.	AK357076	XP_002873549.1	Mechanosensitive ion channel MscS
Os06g0206000	Similar to carbon-nitrogen hydrolase family protein.	FP096100	NP_196765.2	Nitrilase/cyanide hydratase and apolipoprotein N-acyltransferase
Os06g0207500	Hypothetical conserved gene.	AK067995	Q69T67	-
Os06g0207600	Hypothetical conserved gene.	AK332154	Q69T66	WRC
Os06g0207783	Hypothetical protein.	tplb0025a09 (Wheat FLcDNA)	longestORF	-
Os06g0207950	Hypothetical gene.	CT836248	longestORF	-
Os06g0208100	Similar to Homeodomain protein HOX3.	AB101651	Q69T58	Helix-turn-helix motif, lambda-like repressor
Os06g0208151	Similar to quinone oxidoreductase.	BT040177	B8AEF1	GroES-like
Os06g0208200	Conserved hypothetical protein.	AK242462	NP_001174659.1	-
Os06g0208300	KOW domain containing protein.	AK059092	A6N0S0	KOW
Os06g0208700	Similar to Dual-specificity phosphatase protein.	AK065860	Q6DL00	Protein-tyrosine phosphatase, SIW14-like
Os06g0208800	Similar to LysM-domain GPI-anchored protein.	AK062542	Q4VVD8	-
Os06g0208951	Conserved hypothetical protein.	EU943155	NP_001057106.1	-
Os06g0209100	Peptidase A1 domain containing protein.	ab initio prediction	NP_001057106.1	Peptidase A1
Os06g0209300	Conserved hypothetical protein.	AK065500	Q69T47	-
Os06g0226950	Fatty acid hydroxylase domain containing protein.	AK241878	B6TLG7	Fatty acid hydroxylase
Os06g0235800	Similar to predicted protein.	AK248752	NP_198226.1	RNA-processing protein, HAT helix
Os06g0235850	Conserved hypothetical protein.	AK242020	NP_001174681.1	-
Os06g0235900	Conserved hypothetical protein.	tplb0032o13 (Wheat FLcDNA)	Q67V84	-
Os06g0240700	Similar to OSIGBa0132O24.3 protein.	EU951279	Q01MB4	-
Os06g0272700	Protein of unknown function DUF231, plant domain containing protein.	BT062756	NP_001174707.1	Domain of unknown function DUF231, plant
Os06g0274100	Armadillo-like helical domain containing protein.	AK242109	Q5VN12	Armadillo-like helical

Os06g0274800	Similar to Peroxidase 11 precursor (EC 1.11.1.7) (Atperox P11) (ATP23a/ATP23b).	AK060295	Q96519	Plant peroxidase
Os06g0274875	Hypothetical protein.	EU942261	longestORF	-
Os06g0274950	Hypothetical gene.	AK241018	longestORF	-
Os06g0275000	Zinc finger protein, Heading date	AB041838	Q9FDX8	Zinc finger, B-box
Os06g0275600	Hypothetical conserved gene.	AK101279	F1DKB4	Helix-loop-helix DNA-binding
Os06g0275700	Similar to Beta-catenin repeat family protein.	AK110058	C3SA81	Armadillo
Os06g0276001	Non-protein coding transcript.	AK287734	NONE	-
Os06g0278450	Hypothetical gene.	CT837498	longestORF	-
Os06g0282750	Hypothetical protein.	AK372466	longestORF	-
Os06g0293000	Hypothetical conserved gene.	CT836321	Q5Z570	-
Os06g0293050	Hypothetical protein.	FP101342	longestORF	-
Os06g0293100	Conserved hypothetical protein.	ab initio prediction	B8B0U0	-
Os06g0293300	Hypothetical protein.	tplb0033h24 (Wheat FLcDNA)	longestORF	-
Os06g0294200	Conserved hypothetical protein.	AK119403	A2X425	-
Os06g0294300	Hypothetical protein.	tplb0033h24 (Wheat FLcDNA)	longestORF	-
Os06g0309550	Hypothetical gene.	CU406696	longestORF	-
Os06g0317100	Similar to glycine-rich cell wall structural protein.	CT837813	NP_001151377.1	Glycine rich protein
Os06g0318700	Non-protein coding transcript.	AK061908	NONE	-
Os06g0320900	Conserved hypothetical protein.	ab initio prediction	Q5ZA95	-
Os06g0323550	Similar to H0315A08.1 protein.	FP099421	Q01I63	Ribonuclease H1, N-terminal
Os06g0324800	Major facilitator superfamily protein.	AK109109	E8Z9A7	General substrate transporter
Os06g0328900	Cytochrome P450 family protein.	AK110659	C3SAE5	Cytochrome P450
Os06g0328966	Hypothetical protein.	AK357549	longestORF	-
Os06g0329300	Conserved hypothetical protein.	AK067924	A2YCF3	-
Os06g0335500	Similar to Auxin-responsive protein IAA21.	AK063854	Q5Z749	AUX/IAA protein
Os06g0336001	Non-protein coding transcript.	AK289086	NONE	-
Os06g0338801	Hypothetical protein.	BT063833	longestORF	-
Os06g0339500	Similar to OSIGBa0137A06.2 protein.	AK105220	Q01N23	-
Os06g0340001	Hypothetical conserved gene.	ab initio prediction	NP_001174769.1	-
Os06g0340600	Similar to phosphatidylinositol 3- and 4-kinase family protein.	AK065176	NP_001147061.1	Phosphatidylinositol 3-/4-kinase, catalytic
Os06g0341400	Hypothetical protein.	AK110872	longestORF	-
Os06g0341500	Plant lipid transfer protein/seed storage/trypsin-alpha amylase inhibitor domain containing protein.	AK106755	Q5Z9Y7	Plant lipid transfer protein/seed storage/trypsin-alpha amylase inhibitor
Os06g0341600	Conserved hypothetical protein.	AK067335	B9FT43	-
Os06g0481800	Myb/SANT-like domain domain containing protein.	BT039927	NP_001176912.1	Myb/SANT-like domain
Os06g0481850	Hypothetical conserved gene.	AK111398	NP_001176912.1	Putative harbinger transposase-derived nuclease
Os06g0513781	Conserved hypothetical protein.	CT833375	B8AJ30	-
Os06g0513862	Conserved hypothetical protein.	CT833375	B8AJ30	-
Os06g0513943	Conserved hypothetical protein.	CT833375	B8AJ30	-
Os06g0514800	Conserved hypothetical protein.	CT833375	B8AJ30	-
Os06g0526700	Probable protein phosphatase 2C 55.	Q652Z7	Q652Z7	Protein phosphatase 2C, manganese/magnesium aspartate binding site
Os06g0539801	Conserved hypothetical protein.	BT064098	A3BCH5	-
Os06g0541600	Conserved hypothetical protein.	AK356929	Q5Z6Z4	-
Os06g0554100	Hypothetical conserved gene.	CT836317	Q5Z9C5	Zinc finger, RING-type
Os06g0554200	Similar to F20B17.20.	AK363742	XP_002889325.1	Protein of unknown function DUF1677, plant
Os06g0557200	Hypothetical protein.	AK063039	longestORF	-
Os06g0573800	Fanconi Anaemia group E protein, C-terminal domain containing protein.	AK106254	A2YEA6	Fanconi Anaemia group E protein, C-terminal
Os06g0579100	Conserved hypothetical protein.	AK068691	B8ATT0	-
Os06g0580100	Similar to cDNA clone:006-306-H07, full insert sequence.	AK059529	B7E6C5	-
Os06g0586000	Conserved hypothetical protein.	AK119252,AK063903	A2XQZ9	-
Os06g0612300	Similar to T6J4.5 protein (WIP6 protein).	AK071104	A6N8R9	Zinc finger, C2H2
Os06g0633100	Similar to P10Sh249C12.	AK107791	D0VLU4	-
Os06g0633450	Hypothetical conserved gene.	ab initio prediction	Q67VL1	-
Os06g0633500	Zinc finger, RING/FYVE/PHD-type domain containing protein.	AK108505	B6TQ30	Zinc finger, RING-type
Os06g0633800	Amino acid transporter, transmembrane domain containing protein.	AK073428	B6TE03	Amino acid transporter, transmembrane
Os06g0635700	Conserved hypothetical protein.	AK107643	A3BDV0	-
Os06g0636800	Conserved hypothetical protein.	EU970200	NP_001058146.1	-
Os06g0638100	Similar to Tic21.	AK073270	Q09S70	Protein of unknown function DUF3611
Os06g0638200	Uncharacterised protein family UPF0047 domain containing protein.	AK059450	B9FQ34	Uncharacterised protein family UPF0047
Os06g0638500	Tyrosine protein kinase domain containing protein.	AK360940	Q67WE5	Protein kinase, catalytic domain
Os06g0639250	Conserved hypothetical protein.	ab initio prediction	Q67WD8	-
Os06g0639550	Non-protein coding transcript.	AK099542	NONE	-
Os06g0645700	Similar to HAF1.	AK108404	Q67W65	Bromodomain
Os06g0645901	Similar to Transcription initiation factor TFIID subunit 1.	BT037416	Q67W65	-
Os06g0649550	Non-protein coding transcript.	BT086930	NONE	-
Os06g0652501	Non-protein coding transcript.	X06283	NONE	-
Os06g0662000	Similar to Vacuolar ATP synthase catalytic subunit A.	AK071775	B6UH55	ATPase, F1/V1/A1 complex, alpha/beta subunit, N-terminal
Os06g0667100	Similar to Nuclear Y/CCAAT-box binding factor C subunit NF-YC.	AK105079,AK066630	NP_001149626.1	Transcription factor CBF/NF-Y/archaeal histone
Os06g0667300	Non-protein coding transcript.	AK102272	NONE	-
Os06g0667400	Conserved hypothetical protein.	AK065424	Q655T7	-
Os06g0667600	Similar to Glycine decarboxylase complex H-protein.	AK071621,AK058606	NP_001152670.1	Glycine cleavage H-protein
Os06g0667900	Disease resistance protein domain containing protein.	ab initio prediction	Q655T3	Disease resistance protein
Os06g0668000	Hypothetical protein.	AK064023	longestORF	-
Os06g0668200	Similar to Phosphoglycerate kinase, cytosolic (EC 2.7.2.3).	AK070705,AK101622	A2YG06	Phosphoglycerate kinase
Os06g0668250	Hypothetical gene.	BT088042	longestORF	-
Os06g0669200	Conserved hypothetical protein.	AK062363	Q5Z8D4	-
Os06g0669225	Hypothetical conserved gene.	ab initio prediction	Q655S3	MATH
Os06g0669275	Similar to MEE44 (maternal effect embryo arrest 44)%3B nucleotidyltransferase.	BT018457	NP_191917.2	PAP/25A-associated
Os06g0669800	Ovarian tumour, otubain domain containing protein.	AK073551	B6U3W2	Ovarian tumour, otubain
Os06g0670000	Similar to Molybdenum cofactor sulfurase.	AK287475	Q655R6	Aminotransferase, class V/Cysteine desulfurase
Os06g0670633	Similar to embryo-sac basal-endosperm layer embryo-surrounding-region.	AJ437283	NP_001105581.1	Glucoamylase, putatuve
Os06g0678700	Hypothetical gene.	AK107560	Q655H6	-
Os06g0683925	Hypothetical protein.	tplb0033d10 (Wheat FLcDNA)	longestORF	-
Os06g0690400	Non-protein coding transcript.	EU941368	NONE	-
Os06g0691900	Similar to F-box domain containing protein.	EU962397	NP_001149196.1	F-box domain, cyclin-like
Os06g0692050	Conserved hypothetical protein.	FP100342	Q5Z672	-
Os06g0698812	Disease resistance protein domain containing protein.	ab initio prediction	XP_002438943.1	Disease resistance protein
Os06g0703900	Homeodomain-like containing protein.	AK067950	B8B262	Myb-like DNA-binding domain, SHAQKYF class
Os06g0705300	Similar to plant-specific domain TIGR01589 family protein.	AK064803	NP_001149236.1	Conserved hypothetical protein CHP01589, plant
Os06g0705350	Similar to pentatricopeptide (PPR) repeat-containing protein.	BT055332	NP_199702.1	Pentatricopeptide repeat
Os06g0705400	Hypothetical conserved gene.	AK062493	C0KHK5	Plant lipid transfer protein/hydrophobic protein, helical domain
Os06g0705651	Hypothetical protein.	EU955776	longestORF	-
Os06g0705700	TGF-beta receptor, type I/II extracellular region family protein.	AK119527	A2YGT5	Oligopeptide transporter
Os06g0705901	Hypothetical protein.	tplb0058e01 (Wheat FLcDNA)	longestORF	-
Os06g0707800	Non-protein coding transcript.	AK111291	NONE	-
Os06g0708300	Similar to RER1A protein.	AK243324	NP_001151498.1	Retrieval of early ER protein Rer1
Os06g0708600	Zinc finger, C2H2-like domain containing protein.	AK100915	E4MXX2	Zinc finger, C2H2-like
Os06g0708700	Similar to Nodulin-like protein.	AK067151	B6SKJ2	Drug/metabolite transporter
Os06g0708832	Similar to arogenate dehydrogenase.	AK355541	NP_001147429.1	Prephenate dehydrogenase
Os06g0710001	Hypothetical conserved gene.	AK249657	Q5Z9G7	-
Os06g0710300	Uncharacterised conserved protein UCP022348 domain containing protein.	AK121344	B6U4B2	Uncharacterised protein family UPF0114
Os06g0710401	Non-protein coding transcript.	AK330826	NONE	-
Os06g0710850	Non-protein coding transcript.	EU950496	NONE	-
Os06g0710900	Conserved hypothetical protein.	AK073326	A2YGW4	-
Os06g0712250	Hypothetical conserved gene.	CT832454	NP_001144189.1	-
Os06g0712300	EF-Hand type domain containing protein.	AK070744	E6NU22	Phospholipid/glycerol acyltransferase
Os06g0712400	Protein of unknown function DUF544 family protein.	AK067573	B8B2H1	Ubiquitin interacting motif
Os06g0712550	Hypothetical protein.	tplb0056b02 (Wheat FLcDNA)	longestORF	-
Os06g0712600	Similar to SHI.	EU959412	NP_001150905.1	Zinc finger, lateral root primordium type 1
Os06g0712900	tRNA-dihydrouridine synthase domain containing protein.	AK106648	NP_201523.1	tRNA-dihydrouridine synthase
Os06g0713400	Cyclin-like F-box domain containing protein.	AK100628	B9FQV7	F-box domain, cyclin-like
Os06g0714400	Conserved hypothetical protein.	AK108610	Q5NAL1	-
Os06g0714432	Non-protein coding transcript.	CT836310	NONE	-
Os06g0714600	ADP-ribosylation factor domain containing protein.	AK318603	A6MD16	Small GTPase superfamily
Os06g0714700	Conserved hypothetical protein.	CU406678	NP_001058577.1	-
Os06g0716700	Similar to Heat shock protein 90.	AK063385	Q9MB32	Heat shock protein Hsp90

Os06g0719600	Domain of unknown function DUF1618 domain containing protein.	AK099272	Q8SB18	Domain of unknown function DUF1618
Os06g0719900	Similar to endo/excinuclease amino terminal domain-containing protein.	CT834222	XP_002881103.1	-
Os06g0721600	Hypothetical conserved gene.	ab initio prediction	NP_001058606.2	Protein of unknown function DUF544
Os06g0721800	Cupredoxin domain containing protein.	AK243644	A2YH12	Blue
Os06g0722100	Hypothetical conserved gene.	AK068839	NP_001058607.1	Domain of unknown function DUF1618
Os06g0723400	Hypothetical conserved gene.	ab initio prediction	Q5YLZ3	Protein of unknown function DUF544
Os06g0725350	Hypothetical gene.	tplb0017d24 (Wheat FLcDNA)	longestORF	-
Os06g0725400	Similar to BLE1 protein.	AK241158	Q8S937	-
Os06g0726200	Similar to Chitinase 1.	AK061042,AK099339,AK104020	Q42993	Glycoside hydrolase, family 19, catalytic
Os06g0726501	Non-protein coding transcript.	tplb0001a06 (Wheat FLcDNA)	NONE	-
Os06g0727400	Similar to Protein kinase APK1A, chloroplast precursor (EC 2.7.1.-).	AK069558	NP_001147720.1	Protein kinase, catalytic domain
Os06g0727500	Hypothetical protein.	tplb0023d22 (Wheat FLcDNA)	longestORF	-
Os06g0728902	Similar to predicted protein.	AK240761	NP_001154712.1	Protein of unknown function DUF605
Os06g0729150	Hypothetical protein.	tplb0026f23 (Wheat FLcDNA)	longestORF	-
Os06g0729300	Similar to Protein argonaute 1D.	AK111833	Q5Z5B2	Stem cell self-renewal protein Piwi
Os06g0729350	Non-protein coding transcript.	BT016774	NONE	-
Os06g0729650	Similar to Photosystem II stability/assembly factor HCF136, chloroplastic.	FP096975	B9FR06	Twin-arginine translocation pathway, signal sequence
Os06g0729800	Hypothetical gene.	AK241958	longestORF	-
Os06g0731000	Conserved hypothetical protein.	ab initio prediction	NP_001175010.1	-
Os06g0731200	Similar to ABC transporter-like protein.	AK071489	XP_002303777.1	ABC transporter-like
Os06g0731300	Surface protein from Gram-positive cocci, anchor region domain containing protein.	AK358759	Q5Z408	Heat shock protein DnaJ, cysteine-rich domain
Os06g0731400	Hypothetical conserved gene.	AK120175	B8B2Y0	-
Os06g0731500	Conserved hypothetical protein.	AK062465	Q5Z405	-
Os06g0731750	Hypothetical gene.	BT016392	longestORF	-
Os06g0731800	Clathrin light chain family protein.	AK069288	Q259D4	Clathrin light chain
Os06g0732000	Similar to 60S ribosomal protein L35.	FP092387	NP_001148517.1	-
Os07g0116050	Transposase, IS4-like domain containing protein.	BT037367	XP_002449014.1	Transposase, IS4-like
Os07g0117600	Conserved hypothetical protein.	ab initio prediction	Q8H5J6	-
Os07g0132800	Hypothetical gene.	AK240792	longestORF	-
Os07g0144900	Similar to ASK11 (ARABIDOPSIS SKP1-LIKE 11)%3B ubiquitin-protein ligase.	AK242552	NP_567959.1	SKP1 component
Os07g0146700	Hypothetical protein.	AK063631,AK101150	GeneMark	-
Os07g0146800	Non-protein coding transcript.	AK106695	NONE	-
Os07g0147500	Similar to Photosystem II 10 kDa polypeptide, chloroplast precursor.	AK062172,AK120691	Q40070	Photosystem II PsbR
Os07g0147550	Similar to Photosystem II 10 kDa polypeptide, chloroplast.	CT837962	Q40070	Photosystem II PsbR
Os07g0147700	ATPase, BadF/BadG/BcrA/BcrD type domain containing protein.	AK242508	NP_001152135.1	ATPase, BadF/BadG/BcrA/BcrD type
Os07g0148800	Conserved hypothetical protein.	AK062246	Q69VA2	-
Os07g0148900	Photosystem I protein-like protein.	AK058788	P36886	Photosystem I PsaG/PsaK protein
Os07g0151800	Conserved hypothetical protein.	AK105339	A2YI84	-
Os07g0151900	Conserved hypothetical protein.	AK240814	NP_001175061.1	-
Os07g0152000	Transcription factor, TCP domain containing protein.	AK370348	B7F9P5	Transcription factor, TCP
Os07g0152500	Similar to H0315A08.1 protein.	FP099421	Q01I63	Ribonuclease H1, N-terminal
Os07g0152900	Similar to Glycolate oxidase (EC 1.1.3.15) (Fragment).	AK120929	Q6YT73	FMN-dependent dehydrogenase
Os07g0152950	Hypothetical gene.	BT066752	longestORF	-
Os07g0153000	Tify domain containing protein.	AK374356	NP_001058910.2	Tify
Os07g0153300	Protein of unknown function DUF1365 family protein.	AK066146	B9FVJ7	Protein of unknown function DUF1365
Os07g0153600	Similar to Prostrate growth 1.	FJ155665	D9ZKN9	Zinc finger, C2H2
Os07g0154201	Hypothetical gene.	BT085311	longestORF	-
Os07g0154300	Conserved hypothetical protein.	AK105386,AK122093	A2YIA2	-
Os07g0154400	Armadillo-type fold domain containing protein.	AK288457	B9FVJ8	Armadillo-type fold
Os07g0154800	Conserved hypothetical protein.	AK111389	B8B7D0	-
Os07g0156200	Haem peroxidase, plant/fungal/bacterial family protein.	AK249509	NP_001152255.1	Plant peroxidase
Os07g0158400	Hypothetical conserved gene.	EU966727	Q9LMF3	GCK
Os07g0159800	MD-2-related lipid-recognition domain containing protein.	AK060475	B6TM82	MD-2-related lipid-recognition
Os07g0159900	Thioredoxin fold domain containing protein.	AK062586	A2YID0	Glutaredoxin
Os07g0161000	Tetratricopeptide-like helical domain containing protein.	AK065877	B8B7F4	Tetratricopeptide TPR-1
Os07g0162200	Protein of unknown function DUF593 domain containing protein.	tplb0058k18 (Wheat FLcDNA)	Q8H538	Protein of unknown function DUF593
Os07g0162300	Similar to RIO kinase.	AK066456	B9FVM0	RIO kinase
Os07g0167700	Conserved hypothetical protein.	AK099436	B8B7M1	-
Os07g0168000	Similar to Polyribonucleotide phophorylase (Fragment).	AK065622	O24288	Exoribonuclease, phosphorolytic domain 1
Os07g0168800	Zinc finger, AN1-type domain containing protein.	AK121813	Q7Y1W9	Zinc finger, AN1-type
Os07g0168900	Hypothetical gene.	AK062924	longestORF	-
Os07g0169600	2OG-Fe(II) oxygenase domain containing protein.	AK121818,AK060318	NP_001152182.1	Oxoglutarate/iron-dependent oxygenase
Os07g0169700	GA 20-oxidase3, GA metabolism	Inferred from literature	Q69LD8	-
Os07g0170016	Hypothetical gene.	AK289254	longestORF	-
Os07g0170200	Similar to predicted protein.	CT836091	NP_179964.2	HNH endonuclease
Os07g0170300	YT521-B-like protein family protein.	AK072392	B6U208	YTH domain
Os07g0170400	Non-protein coding transcript.	BT016473	NONE	-
Os07g0170500	Conserved hypothetical protein.	AK240864	NP_001175070.1	-
Os07g0171200	Similar to Galactose-1-phosphate uridyl transferase-like protein.	AK071075	B6TFN9	Galactose-1-phosphate uridyl transferase, class I
Os07g0171300	Protein kinase, core domain containing protein.	AK100663,AK066357	NP_001152463.1	Protein kinase, catalytic domain
Os07g0171350	Non-protein coding transcript.	BT084747	NONE	-
Os07g0172200	Similar to ROOT HAIRLESS 1.	AK103352	B6UAF0	-
Os07g0172900	Hypothetical conserved gene.	AK067304	Q6ZA56	-
Os07g0173100	HSP20-like chaperone domain containing protein.	CT836094	NP_001059002.1	-
Os07g0173300	Conserved hypothetical protein.	AK110935	A3BH18	-
Os07g0175200	MTCP1 domain containing protein.	AK058437	A2YIN2	MTCP1
Os07g0175801	Non-protein coding transcript.	X02683	NONE	-
Os07g0176200	Similar to Transcription factor CPP.	AK062003	B3IX45	Tesmin/TSO1-like, CXC
Os07g0176400	Hypothetical protein.	BT085651	longestORF	-
Os07g0176401	Hypothetical protein.	tplb0061g22 (Wheat FLcDNA)	longestORF	-
Os07g0176500	Leucine-rich repeat, plant specific containing protein.	AK062887	A2YIP1	Leucine-rich repeat
Os07g0176600	Similar to Kinase-like protein.	AK067073,AK066812	Q6ZEZ5	Protein kinase, catalytic domain
Os07g0176900	Similar to ribose-5-phosphate isomerase.	AK067117	Q8RU73	Ribose 5-phosphate isomerase, type A
Os07g0177150	Non-protein coding transcript.	AK288093	NONE	-
Os07g0177200	Similar to glutamate binding protein.	AK070925	XP_002878509.1	Inhibitor of apoptosis-promoting Bax1-related
Os07g0177300	Uncharacterised protein family UPF0005 domain containing protein.	AK073781	B6T7B7	Inhibitor of apoptosis-promoting Bax1-related
Os07g0178950	Hypothetical protein.	tplb0047n14 (Wheat FLcDNA)	longestORF	-
Os07g0179000	Pentatricopeptide repeat domain containing protein.	AK100008	A2YIR0	Pentatricopeptide repeat
Os07g0210800	Hypothetical conserved gene.	AK106587	Q60DP5	Myb/SANT-like domain
Os07g0211601	Hypothetical gene.	AK288862	longestORF	-
Os07g0211700	Similar to isopentenyl transferase IPT4.	AB239801	NP_001121195.1	tRNA isopentenyltransferase
Os07g0211800	Protein of unknown function DUF1749 family protein.	AK065959	B7EB72	Protein of unknown function DUF1749
Os07g0211900	Protein of unknown function DUF632 domain containing protein.	AK243281	Q8H4U8	Domain of unknown function DUF632
Os07g0212300	Similar to Nudix hydrolase 16, mitochondrial precursor (EC 3.6.1.-) (AtNUDT16).	AK107375	B6SQG1	NUDIX hydrolase domain
Os07g0213500	Hypothetical conserved gene.	AK070126	B6SL19	-
Os07g0213600	Bifunctional inhibitor/plant lipid transfer protein/seed storage domain containing protein.	AK107696	A2YJF5	Plant lipid transfer protein/seed storage/trypsin-alpha amylase inhibitor
Os07g0213800	Similar to Allergenic protein.	AK107215	Q01882	Cereal allergen/alpha-amylase inhibitor, rice-type
Os07g0214100	Seed allergenic protein RA17 precursor.	AK242324	Q01883	Cereal allergen/alpha-amylase inhibitor, rice-type
Os07g0214300	Seed allergenic protein RAG2 precursor.	AK107328	Q01882	Cereal allergen/alpha-amylase inhibitor, rice-type
Os07g0214600	Similar to Seed allergenic protein RA17 precursor.	AK242298	Q40654	Cereal allergen/alpha-amylase inhibitor, rice-type
Os07g0215500	Allergenic protein.	AK242333	Q01881	Cereal allergen/alpha-amylase inhibitor, rice-type
Os07g0218700	Cytochrome P450 family protein.	AK108382	Q01LP1	Cytochrome P450
Os07g0220466	Similar to H0315A08.1 protein.	FP099421	Q01I63	Ribonuclease H1, N-terminal
Os07g0223733	Non-protein coding transcript.	BT016899	NONE	-
Os07g0283050	Concanavalin A-like lectin/glucanase, subgroup domain containing protein.	BT041367	NP_001147473.1	Protein kinase, catalytic domain
Os07g0301050	Non-protein coding transcript.	BT068036	NONE	-
Os07g0417683	Hypothetical protein.	EU967040	longestORF	-
Os07g0479500	Hypothetical protein.	AK109231	longestORF	-
Os07g0487198	Hypothetical protein.	tplb0040j01 (Wheat FLcDNA)	longestORF	-
Os07g0487300	Protein kinase, catalytic domain domain containing protein.	ab initio prediction	Q84ZD7	Protein kinase, catalytic domain
Os07g0488215	Hypothetical protein.	tplb0040j01 (Wheat FLcDNA)	longestORF	-
Os07g0488400	Protein kinase, catalytic domain domain containing protein.	ab initio prediction	Q84ZD7	Protein kinase, catalytic domain
Os07g0490500	Xanthine/uracil/vitamin C permease family protein.	AK059640,AK061151	XP_002866918.1	Xanthine/uracil/vitamin C permease
Os07g0497500	Similar to MYB transcription factor.	BT038744	Q71KV1	SANT domain, DNA binding
Os07g0523400	Glucose-6-phosphate/phosphate-translocator precursor.	AK070124	NP_001147439.1	Drug/metabolite transporter

Os07g0523965	Similar to Glucose-6-phosphate/phosphate-translocator precursor.	AK059906	NP_001147439.1	Drug/metabolite transporter
Os07g0536800	Hypothetical conserved gene.	AK109751	Q53NH3	-
Os07g0555200	Similar to predicted protein.	AK062438	D4N2J6	MIF4G-like, type 3
Os07g0564533	Similar to HAT family dimerisation domain containing protein.	AK288170	Q53RM1	HAT dimerisation
Os07g0569600	Chaperonin-like RbcX domain containing protein.	AK067599	A3BLB7	Chaperonin-like RbcX
Os07g0574150	Hypothetical protein.	EU951884	longestORF	-
Os07g0575733	Conserved hypothetical protein.	ab initio prediction	NP_001053398.1	-
Os07g0611400	Protein kinase C-like, phorbol ester/diacylglycerol binding domain containing protein.	EU972444	Q8H5Y7	Protein kinase C-like, phorbol ester/diacylglycerol binding
Os07g0633450	Hypothetical gene.	EU947431	longestORF	-
Os07g0639400	Similar to Peroxidase 1.	AK110555	A3BMN4	Plant peroxidase
Os07g0648566	Hypothetical gene.	AK059417	longestORF	-
Os07g0648700	Conserved hypothetical protein.	ab initio prediction	NP_001175315.1	-
Os07g0669200	Similar to GTP1/OBG family protein.	BT063209	NP_197358.2	GTP-binding domain, HSR1-related
Os07g0669250	Similar to predicted protein.	EU950976	XP_002873904.1	GTP-binding protein GTP1/OBG, C-terminal
Os07g0669401	Conserved hypothetical protein.	FP091568	NP_001042416.1	-
Os07g0669600	Conserved hypothetical protein.	AK066595	B9FUS7	-
Os07g0674100	Hypothetical conserved gene.	AK099614	B9FUU7	NAD-dependent epimerase/dehydratase
Os07g0674150	Hypothetical protein.	tplb0036f05 (Wheat FLcDNA)	longestORF	-
Os07g0674200	Similar to 60S ribosomal protein L22-2.	AK058245	B6U998	Ribosomal protein L22e
Os07g0674300	Protein kinase, ATP binding site domain containing protein.	AK110511	A2YPV3	Pentatricopeptide repeat
Os07g0674500	Pollen Ole e 1 allergen/extensin domain containing protein.	AK107802	A2YPV5	Pollen Ole e 1 allergen/extensin
Os07g0675166	Non-protein coding transcript.	AK110786	NONE	-
Os07g0675200	Conserved hypothetical protein.	AK063620	B8B5V2	-
Os07g0676400	Hypothetical conserved gene.	CT836080	B8B5V6	Protein kinase, catalytic domain
Os07g0677600	Similar to Cationic peroxidase.	AK060984	NP_001147443.1	Plant peroxidase
Os07g0678600	Similar to cDNA clone:J023141O10, full insert sequence.	AK058819	B3GLZ6	Protein kinase, catalytic domain
Os07g0679000	Similar to Potassium transporter 2 (AtPOT2) (AtKUP2) (AtKT2).	AK070738	Q1T761	K+ potassium transporter
Os07g0679050	Hypothetical protein.	EU968841	longestORF	-
Os07g0687200	Similar to CAM7 (CALMODULIN 7)%3B calcium ion binding.	AK104031	NP_850344.1	Calcium-binding EF-hand
Os07g0690600	Protein of unknown function DUF1677, Oryza sativa domain containing protein.	AK365840	Q8H3Z2	Protein of unknown function DUF1677, Oryza sativa
Os07g0692500	Conserved hypothetical protein.	AK108769	Q7XI64	-
Os07g0692900	Similar to Ubiquitin-activating enzyme E1.	AK074007	B8B6A7	Ubiquitin/SUMO-activating enzyme E1
Os07g0692950	Hypothetical protein.	tplb0053b11 (Wheat FLcDNA)	longestORF	-
Os08g0114150	Similar to predicted protein.	EU943426	XP_002327722.1	-
Os08g0127300	Conserved hypothetical protein.	AK058548	Q6ZK51	-
Os08g0127750	Non-protein coding transcript.	AK242810	NONE	-
Os08g0127800	Conserved hypothetical protein.	AK103321,AK108548	Q6ZK47	-
Os08g0132500	Conserved hypothetical protein.	AK363691	Q69R49	-
Os08g0133200	Similar to copine-3.	AK241367	NP_001147038.1	Copine
Os08g0141300	PapD-like domain containing protein.	AK058337,AK067917	B6TP74	Major sperm protein
Os08g0142500	Pectinesterase inhibitor domain containing protein.	AK242564	NP_001175375.1	Pectinesterase inhibitor
Os08g0143150	Similar to retrotransposon protein.	EU953494	NP_001158937.1	-
Os08g0143400	Amine oxidase domain containing protein.	AK120532	NP_187981.1	Amine oxidase
Os08g0143500	Glycosyl transferase, family 14 protein.	AK071122	NP_001147601.1	Glycosyl transferase, family 14
Os08g0145501	Conserved hypothetical protein.	AK241170	B9FSE2	-
Os08g0146001	Hypothetical conserved gene.	ab initio prediction	B9FZ18	-
Os08g0163700	Conserved hypothetical protein.	AK375159	Q84S46	-
Os08g0166750	Hypothetical protein.	AK372466	longestORF	-
Os08g0172433	Non-protein coding transcript.	EU941912	NONE	-
Os08g0182400	Conserved hypothetical protein.	AK059491	NP_001061148.1	-
Os08g0216450	Conserved hypothetical protein.	EU951430	Q65WW4	-
Os08g0328050	Hypothetical protein.	EU945299	longestORF	-
Os08g0393150	Similar to OSIGBa0110B10.2 protein.	EU973161	Q01KQ6	Zinc finger, BED-type predicted
Os08g0404350	Similar to FACT complex subunit SPT16.	ab initio prediction	Q7X923	-
Os08g0411900	Conserved hypothetical protein.	AK243392	B9G0V4	-
Os08g0433300	Sialidase domain containing protein.	EU965133	NP_001061873.1	Neuraminidase
Os08g0433400	Hypothetical conserved gene.	AK059468	A2YVI0	SANT domain, DNA binding
Os08g0433600	Protein of unknown function DUF962 family protein.	AK067255	NP_001151002.1	Protein of unknown function DUF962
Os08g0434300	Similar to Malate dehydrogenase precursor (EC 1.1.1.37).	AK058477	A2YVI5	Lactate/malate dehydrogenase, N-terminal
Os08g0434700	Homeodomain-like containing protein.	AK070338	B8BB14	Myb-like DNA-binding domain, SHAQKYF class
Os08g0435725	Conserved hypothetical protein.	ab initio prediction	A2YVI9	-
Os08g0436700	Similar to NAC transcription factor.	AK073013	A2YVJ8	No apical meristem
Os08g0437566	Similar to H0315A08.1 protein.	FP099421	Q01I63	Ribonuclease H1, N-terminal
Os08g0437832	Non-protein coding transcript.	EU941912	NONE	-
Os08g0438801	Hypothetical protein.	AK287665	longestORF	-
Os08g0439000	Similar to pyrophosphate--fructose 6-phosphate 1-phosphotransferase.	AK287794	NP_001147085.1	Phosphofructokinase domain
Os08g0439200	Non-protein coding transcript.	AK059480	NONE	-
Os08g0439600	Protein of unknown function DUF868, plant family protein.	BT070111	NP_001061898.1	Protein of unknown function DUF868, plant
Os08g0440100	Similar to Temperature stress-induced lipocalin.	AK068551	Q38JE5	Lipocalin/cytosolic fatty-acid binding protein domain
Os08g0440800	Glyceraldehyde-3-phosphate dehydrogenase.	AK071520	Q8S4Y9	Aldehyde dehydrogenase domain
Os08g0440850	Hypothetical gene.	EU945888	longestORF	-
Os08g0441100	Similar to PnC401 homologue.	AK108225	Q6Z9F4	Protein kinase, catalytic domain
Os08g0441300	Hypothetical conserved gene.	ab initio prediction	Q6Z9F3	Proteinase inhibitor I13, potato inhibitor I
Os08g0441500	Similar to cinnamoyl CoA reductase.	AK102061	XP_002299260.1	NAD-dependent epimerase/dehydratase
Os08g0441600	Similar to Chorismate mutase CM2 (EC 5.4.99.5) (Chorimate mutase).	AK069725	B4FUP5	Chorismate mutase, AroQ class, eukaryotic type
Os08g0442600	Hypothetical protein.	AK105576	longestORF	-
Os08g0442900	Hypothetical protein.	AK110520	GeneMark	-
Os08g0444100	Similar to Splicing factor 3b, subunit 5 (Splicing factor 3B subunit 10).	AK073746	E5GC24	Splicing factor 3B subunit 5/RDS3 complex subunit 10
Os08g0444440	Hypothetical conserved gene.	AK241238	B9G163	-
Os08g0444500	Conserved hypothetical protein.	CT835928	NP_001061923.1	-
Os08g0445000	Similar to RING-H2 finger protein ATL5A.	AK106866	B6T239	Zinc finger, RING-type
Os08g0445250	Hypothetical protein.	tplb0042b14 (Wheat FLcDNA)	longestORF	-
Os08g0445700	HAD-superfamily hydrolase subfamily IIB protein.	AK072132	B6T0F0	Glycosyl transferase, family 20
Os08g0446200	Similar to Receptor-like protein kinase precursor (EC 2.7.1.37). Splice isoform INRPK1a.	AK120424	XP_002445581.1	Protein kinase, catalytic domain
Os08g0446250	Hypothetical gene.	BT068372	longestORF	-
Os08g0446350	Non-protein coding transcript.	BT068372	NONE	-
Os08g0446750	Hypothetical protein.	EU976033	longestORF	-
Os08g0446800	Similar to GDU1.	AK243202	NP_001151217.1	-
Os08g0447000	Similar to D-3-phosphoglycerate dehydrogenase.	AK243399	B6SKK1	Amino acid-binding ACT
Os08g0447500	Hypothetical conserved gene.	AK240682	A3BTN1	-
Os08g0448000	Similar to 4-coumarate--CoA ligase 1 (EC 6.2.1.12) (4CL 1) (4-coumaroyl-CoA synthase 1) (Clone 4	AK120964	Q9LL50	AMP-dependent synthetase/ligase
Os08g0448050	Hypothetical protein.	BT062457	longestORF	-
Os08g0448900	Conserved hypothetical protein.	CT832739	NP_001175602.1	-
Os08g0449500	Conserved hypothetical protein.	AK120389	B8BB90	-
Os08g0449901	Hypothetical protein.	BT085732	longestORF	-
Os08g0450100	Similar to Pectinesterase (EC 3.1.1.11) (Fragment).	AK058651	A2YVS3	Pectinesterase, catalytic
Os08g0450200	Similar to Pectin methylesterase (Fragment).	AK072779	A2YVS4	Pectinesterase, catalytic
Os08g0450800	Phosphatidylinositol-4-phosphate 5-kinase family protein.	AK102479	XP_002882874.1	Phosphatidylinositol-4-phosphate 5-kinase, core
Os08g0450900	SANT domain, DNA binding domain containing protein.	ab initio prediction	Q6ZLF1	SANT domain, DNA binding
Os08g0452200	Hypothetical protein.	AK100150	longestORF	-
Os08g0452500	Auxin responsive SAUR protein family protein.	AK106714	B6TUQ7	Auxin responsive SAUR protein
Os08g0453733	Non-protein coding transcript.	CT836006	NONE	-
Os08g0454000	Pathogenesis-related transcriptional factor/ERF, DNA-binding domain containing protein.	EU968979	Q6ZAG1	Pathogenesis-related transcriptional factor/ERF, DNA-binding
Os08g0455700	Similar to predicted protein.	AK240992,AK240990	NP_001061962.1	Exo70 exocyst complex subunit
Os08g0456600	Hypothetical protein.	AK059009	longestORF	-
Os08g0457901	Hypothetical genes.	ab initio prediction	NONE	-
Os08g0460000	Similar to Germin-like protein 1 precursor.	AK104729,AK065284	Q6ZBZ2	Germin
Os08g0460900	Conserved hypothetical protein.	ab initio prediction	NP_001061979.1	-
Os08g0461300	Cyclin-like F-box domain containing protein.	AK065651	A3BTV7	F-box domain, cyclin-like
Os08g0463500	Zinc finger, C2H2-type domain containing protein.	AK058457	A2YVZ8	Zinc finger, C2H2
Os08g0467201	Hypothetical protein.	BT086922	longestORF	-
Os08g0467300	Clathrin adaptor, phosphoinositide-binding, GAT-like domain containing protein.	AK105541	NP_001151341.1	ENTH/VHS
Os08g0467500	Abscisic acid and stress inducible (A22) gene.	AK100937	Q07764	TB2/DP1/HVA22-related protein
Os08g0468700	Similar to Nitrate reductase [NADH] 1 (EC 1.7.1.1) (NR1).	AK121810	P16081	Oxidoreductase, molybdopterin-binding domain
Os08g0468801	Hypothetical protein.	AK107363	longestORF	-

Os08g0469500	Similar to F-box domain containing protein.	AK109599	NP_001150287.1	-
Os08g0469600	Conserved hypothetical protein.	AK062647	B8BBK4	-
Os08g0471000	Similar to cDNA, clone: J065120N14, full insert sequence.	AK356002	B7F8M8	Heat shock factor
Os08g0471150	Hypothetical protein.	tplb0033f11 (Wheat FLcDNA)	longestORF	-
Os08g0471800	Remorin, C-terminal region domain containing protein.	AK105281	B9G1D0	Remorin, C-terminal
Os08g0471850	Hypothetical protein.	tplb0035l08 (Wheat FLcDNA)	longestORF	-
Os08g0471950	bZIP transcription factor TRAB1.	Q6ZDF3	Q6ZDF3	Basic-leucine zipper
Os08g0472400	Conserved hypothetical protein.	FP097825	Q6ZDE7	-
Os08g0472800	Cytochrome P450 family protein.	AK120757	Q6ZDE3	Cytochrome P450
Os08g0473200	Hypothetical protein.	tplb0043h07 (Wheat FLcDNA)	longestORF	-
Os08g0475400	Alpha/beta hydrolase fold-3 domain containing protein.	AK060706,AK073975	NP_001150584.1	Alpha/beta hydrolase fold-3
Os08g0476400	Similar to phosphoglycerate mutase gpmB.	AK241011	NP_001150922.1	Phosphoglycerate/bisphosphoglycerate mutase, active site
Os08g0477900	Helix-loop-helix DNA-binding domain containing protein.	EU968808	NP_001159035.1	Helix-loop-helix DNA-binding
Os08g0478566	Similar to Maturase K 2.	AY964640	C6ES29	-
Os08g0478800	Phosphoglucose isomerase (PGI) family protein.	AK107494	A3BU52	Phosphoglucose isomerase
Os08g0479400	Similar to Hydroxyproline-rich glycoprotein DZ-HRGP precursor.	AK109528	B6TE62	Homeobox domain, ZF-HD class
Os08g0480800	Similar to TaWIN2.	AK101599	Q84J55	14-3-3 protein
Os08g0480901	Non-protein coding transcript.	BT017754	NONE	-
Os08g0481000	Hypothetical conserved gene.	AK111069	Q6Z235	Pentatricopeptide repeat
Os08g0481100	Similar to SKIP interacting protein 3 (Fragment).	AK240733	B8Q889	-
Os08g0482025	Hypothetical protein.	BT009228	longestORF	-
Os08g0482300	Pistil-specific extensin-like protein domain containing protein.	AK107940	longestORF	-
Os08g0482600	Cupredoxin domain containing protein.	AK063639	A2YWC7	Blue
Os08g0482700	Cupredoxin domain containing protein.	AK060044	A2YWC8	Plastocyanin-like
Os08g0483100	Conserved hypothetical protein.	AK099821	A3BU75	-
Os08g0483600	Conserved hypothetical protein.	AK099543	B9G1G7	-
Os08g0483800	Hypothetical gene.	AK100866	longestORF	-
Os08g0483900	Helix-loop-helix DNA-binding domain containing protein.	AK107626	NP_001151793.1	Helix-loop-helix DNA-binding
Os08g0484100	Similar to predicted protein.	AK242582	A3BU81	Glycoside hydrolase, subgroup, catalytic domain
Os08g0484200	Zinc finger, RING/FYVE/PHD-type domain containing protein.	AK287991,AK241976	NP_001062080.1	Zinc finger, RING-type
Os08g0484300	Conserved hypothetical protein.	BT019110	C0P2Z7	-
Os08g0484450	Hypothetical protein.	tplb0055k03 (Wheat FLcDNA)	longestORF	-
Os08g0484500	Similar to Phospho-2-dehydro-3-deoxyheptonate aldolase 2.	AK106196	B6UAK5	DAHP synthetase, class II
Os08g0484533	Hypothetical protein.	tplb0022g15 (Wheat FLcDNA)	longestORF	-
Os08g0484633	Hypothetical protein.	tplb0031c07 (Wheat FLcDNA)	longestORF	-
Os08g0484800	Hypothetical conserved gene.	FP092392	Q6YTS7	-
Os08g0485000	Similar to PHI-1.	AK073011	NP_001150825.1	Phosphate-induced protein 1
Os08g0485500	Conserved hypothetical protein.	AK241136	A3BU93	-
Os08g0485600	Hypothetical conserved gene.	AK103243	B8BBU4	Zinc finger, C2H2
Os08g0485800	Barwin-related endoglucanase domain containing protein.	AK242586	NP_001148968.1	Expansin/pollen allergen, DPBB domain
Os08g0485900	Haloacid dehalogenase-like hydrolase domain containing protein.	AK110716	NP_568077.1	Haloacid dehalogenase-like hydrolase
Os08g0486100	Similar to Potential copper-transporting ATPase PAA1 (EC 3.6.3.4).	AK059217	XP_002304082.1	ATPase, P-type, H+ transporting proton pump
Os08g0486200	Similar to Splicing factor SC35.	AK103676	B6UG76	RNA recognition motif domain
Os08g0486233	Similar to EIL transcription factor.	CT835917	Q8W3L9	Ethylene insensitive 3-like protein, DNA-binding domain
Os08g0486266	Conserved hypothetical protein.	AK288793	Q53LF9	-
Os08g0486300	Similar to P-type R2R3 Myb protein (Fragment).	BT033636	Q8S416	SANT domain, DNA binding
Os08g0486400	Hypothetical protein.	EU971393	longestORF	-
Os08g0486700	Conserved hypothetical protein.	AK241195	NP_001062097.1	-
Os08g0486750	Non-protein coding transcript.	AK363293	NONE	-
Os08g0486801	Non-protein coding transcript.	AK241869	NONE	-
Os08g0487100	Similar to BZIP transcription factor BZI-2.	AK107150	NP_001152649.1	Basic-leucine zipper
Os08g0487500	Zinc finger, RING/FYVE/PHD-type domain containing protein.	AK066326	B6U108	Zinc finger, RING-type
Os08g0489100	Hypothetical conserved gene.	ab initio prediction	Q6ZBR2	UDP-glucuronosyl/UDP-glucosyltransferase
Os08g0489300	Methyladenine glycosylase domain containing protein.	AK104597,AK059000	B4FL85	Methyladenine glycosylase
Os08g0490000	Helix-loop-helix DNA-binding domain containing protein.	AK100183	B6SVP6	Helix-loop-helix DNA-binding
Os08g0490100	Similar to PBF protein.	AK241364	Q1HFQ1	Zinc finger, Dof-type
Os08g0490300	RNA recognition motif, glycine rich protein domain containing protein.	AK066895	A2YWH8	RNA recognition motif domain
Os08g0490400	Hypothetical gene.	AK334302	GeneMark	-
Os08g0490600	FAS1 domain domain containing protein.	AK108305	Q06IA2	FAS1 domain
Os08g0490800	Similar to Histone H2B.	FP097117	B9G1J0	Histone H2B
Os08g0490900	Similar to Histone H2B.2.	FP098904	C5XPC6	Histone H2B
Os08g0491000	Hypothetical conserved gene.	AK073673	B9G1J1	-
Os08g0491100	Conserved hypothetical protein.	AK060215	B8BC14	-
Os08g0491200	Serine/threonine protein kinase domain containing protein.	AK103656	B7EQ74	Protein kinase, catalytic domain
Os08g0491400	Interferon-related developmental regulator domain containing protein.	AK109684	A2YWI6	Interferon-related developmental regulator, N-terminal
Os08g0491700	Putative zinc finger CCCH domain-containing protein 57.	A3BUD2	A3BUD2	Zinc finger, CCCH-type
Os08g0492400	C2 calcium-dependent membrane targeting domain containing protein.	AK241274	B9G1J5	C2 calcium-dependent membrane targeting
Os08g0494000	Harpin-induced 1 domain containing protein.	AK110788	NP_001148671.1	Late embryogenesis abundant protein, LEA-14
Os08g0494100	Transcription factor, MADS-box domain containing protein.	ab initio prediction	Q6Z5F8	Transcription factor, MADS-box
Os08g0494300	Copine domain containing protein.	AK066150	NP_001147447.1	Zinc finger, RING-type
Os08g0494350	Pentatricopeptide repeat domain containing protein.	ab initio prediction	B8BC26	Pentatricopeptide repeat
Os08g0495300	Similar to cDNA clone:J013000K10, full insert sequence.	AK287721	B9G1K9	Protein of unknown function DUF3615
Os08g0496100	Hypothetical conserved gene.	EU952779	Q6Z5C4	-
Os08g0496600	Hypothetical conserved gene.	FP095819	Q7F8V1	-
Os08g0497300	Similar to phosphatidylinositol transporter/ transporter.	AK059604	B6U127	Cellular retinaldehyde-binding/triple function, C-terminal
Os08g0498100	Similar to Caffeoyl-CoA O-methyltransferase 2 (EC 2.1.1.104) (Trans-caffeoyl-CoA 3-O-methyltrans	AK071482,AK104326,AK104801	B4G080	O-methyltransferase, family 3
Os08g0498400	Similar to Caffeoyl-CoA O-methyltransferase (Fragment).	AK065515	A5A5Y9	O-methyltransferase, family 3
Os08g0499000	Conserved hypothetical protein.	AK107539	A2YWN4	-
Os08g0499100	ATPase, AAA+ type, core domain containing protein.	AK243638	XP_002864442.1	NB-ARC
Os08g0499250	Hypothetical conserved gene.	EU971673	B6U358	-
Os08g0499300	WRKY transcription factor 30.	AK065518	B8BC43	DNA-binding WRKY
Os08g0499400	Conserved hypothetical protein.	AK241142	NP_001175641.1	-
Os08g0500400	Hypothetical protein.	AK111024	longestORF	-
Os08g0500900	Similar to Phosphoribosylglycinamide formyltransferase, chloroplast precursor (EC 2.1.2.2) (GART)	AK102314	XP_002334132.1	Phosphoribosylglycinamide formyltransferase, active site
Os08g0501600	Similar to Wall associated kinase.	DQ177499	Q2L3D5	Protein kinase, catalytic domain
Os08g0501800	Non-protein coding transcript.	EU955321	NONE	-
Os08g0501900	Non-protein coding transcript.	AK063130	NONE	-
Os08g0502101	Conserved hypothetical protein.	AK241735	NP_001175644.1	-
Os08g0502600	EF hand domain containing protein.	AK107730	A2YWR5	Calcium-binding EF-hand
Os08g0503600	Hypothetical protein.	AK119300	longestORF	-
Os08g0503700	Sodium/sulphate symporter family protein.	AK072183	NP_001148320.1	Sodium/sulphate symporter
Os08g0504000	Similar to WIP5 protein.	AK287566	A6N8R9	Zinc finger, C2H2
Os08g0504200	Similar to H0315A08.1 protein.	FP099421	Q01I63	Ribonuclease H1, N-terminal
Os08g0504400	Similar to uncharacterized enzyme involved in pigment biosynthesis.	AK072089	B6T7U3	Pseudouridine-5&apos%3B-phosphate glycosidase
Os08g0504500	Mog1/PsbP/DUF1795, alpha/beta/alpha sandwich domain containing protein.	AK062330	NP_001148434.1	Photosystem II PsbP, oxygen evolving complex
Os08g0504600	Nucleotide-binding, alpha-beta plait domain containing protein.	AK064868	B9G1P6	RNA recognition motif domain
Os08g0504700	Similar to Multiple stress-responsive zinc-finger protein ISAP1 (Stress- associated protein 1) (OsISA	AK101969	Q84PD8	Zinc finger, AN1-type
Os08g0505200	tRNA-binding arm domain containing protein.	AK067190	NP_181761.1	-
Os08g0505250	Similar to Zinc finger, C3HC4 type family protein.	CT836004	Q2R253	-
Os08g0505300	Similar to 60S ribosomal protein L31.	D29722	B6SJB0	Ribosomal protein L31e
Os08g0505900	Similar to DNA-damage-repair/toleration protein DRT100 precursor.	AK060670,AK120866,AK120908	NP_001150670.1	Leucine-rich repeat
Os08g0506350	Conserved hypothetical protein.	AK241499	NP_001175650.1	-
Os08g0506850	Hypothetical gene.	CT836002	longestORF	-
Os08g0507100	Cytochrome P450 family protein.	AF488521	Q8S3F6	Cytochrome P450
Os08g0507200	Hypothetical conserved gene.	EU958332	A3BUN9	-
Os08g0507701	Hypothetical protein.	AK242603	longestORF	-
Os08g0507702	Cytochrome P450 family protein.	AK099637	Q8S3F6	Cytochrome P450
Os08g0508700	Similar to EIL3.	AK072462	E3VXB5	Ethylene insensitive 3
Os08g0509400	Non-protein coding transcript.	AK060593	NONE	-
Os08g0510400	Short-chain dehydrogenase/reductase SDR domain containing protein.	AK069393	B6UEX0	Short-chain dehydrogenase/reductase SDR
Os08g0510601	Non-protein coding transcript.	X02683	NONE	-
Os08g0510700	Hypothetical conserved gene.	AK111116	Q7EXY8	SANT domain, DNA binding
Os08g0510750	Hypothetical gene.	EU975368	longestORF	-
Os08g0511800	Conserved hypothetical protein.	AK063264	A2YWY7	-
Os08g0511900	Similar to aminoacylase-1.	AK240689	NP_001148589.1	Peptidase M20

Os08g0512100	Conserved hypothetical protein.	AK359537	Q6Z8P1	-
Os08g0512200	Similar to heavy metal-associated domain containing protein.	EU952883	NP_001147000.1	Heavy metal-associated domain, HMA
Os08g0512300	Similar to Rab geranylgeranyl transferase beta subunit (Fragment).	AK102273	XP_002871518.1	Prenyltransferase/squalene oxidase
Os08g0512400	Protein of unknown function DUF296 domain containing protein.	AK067561	NP_001149717.1	Domain of unknown function DUF296
Os08g0512600	Protein cdc2 kinase.	AK059682	Q0J4I1	Protein kinase, catalytic domain
Os08g0519501	Hypothetical gene.	AK109201	longestORF	-
Os08g0519600	Similar to predicted protein.	AK072374	B9G1U1	Ribosomal protein L34Ae
Os08g0519700	Protein of unknown function DUF630 domain containing protein.	AK064559	A2YX44	Domain of unknown function DUF630
Os08g0519750	Hypothetical conserved gene.	EU950331	A2YX44	Domain of unknown function DUF632
Os08g0519800	Similar to Maternal pumilio protein. Splice isoform E.	AK121539	Q6YZW8	Pumilio RNA-binding repeat
Os08g0519900	Conserved hypothetical protein.	ab initio prediction	NP_001062263.1	-
Os08g0520000	Mitochondrial substrate carrier family protein.	AK063556	NP_001151333.1	Mitochondrial carrier protein
Os08g0520200	Similar to predicted protein.	AK288100	Q287W6	Tetratricopeptide-like helical
Os08g0520300	Similar to Oligouridylate binding protein.	AK069912	NP_001148487.1	RNA recognition motif domain
Os08g0520400	Conserved hypothetical protein.	AK119371,AK064309,AK102428	A2YX50	-
Os08g0520500	Similar to Isoform 2 of Auxin response factor 21.	AK059543	Q6YZW0-2	-
Os08g0524600	Similar to 60S ribosomal protein L32.	P51421	P51421	Ribosomal protein L32e
Os08g0525000	Ras GTPase family protein.	AK103220	NP_001152371.1	Small GTPase superfamily
Os08g0525050	Non-protein coding transcript.	BT084300	NONE	-
Os08g0533300	Similar to ACR5.	AK287480	B6SYQ4	Amino acid-binding ACT
Os08g0533450	Hypothetical protein.	tplb0061b06 (Wheat FLcDNA)	longestORF	-
Os08g0535000	Similar to cation cation antiporter.	AK066324	NP_001148426.1	Vesicle transport protein, Use1
Os08g0536333	Non-protein coding transcript.	CT835951	NONE	-
Os08g0557500	Similar to predicted protein.	AK111941	XP_002877347.1	Peptidyl-prolyl cis-trans isomerase, cyclophilin-type
Os08g0557600	Similar to Monodehydroascorbate reductase (EC 1.6.5.4) (MDAR) (Ascorbate free radical reductase	AK102459	Q9SXX0	Pyridine nucleotide-disulphide oxidoreductase, NAD-binding domain
Os08g0557700	Similar to Histidine-containing phosphotransfer protein.	AK061111	Q6PT60	Signal transduction histidine kinase, phosphotransfer
Os08g0558800	Similar to Ribosomal protein.	AK122095	B8B9K6	Ribosomal protein L1
Os08g0559501	Conserved hypothetical protein.	CT837577	NP_001175701.1	-
Os08g0564300	Hypothetical conserved gene.	AK106260	Q8GU78	ABC transporter, transmembrane domain
Os09g0114500	Hypothetical protein.	AK059015	longestORF	-
Os09g0114566	Non-protein coding transcript.	X06284	NONE	-
Os09g0115900	Non-protein coding transcript.	AK060240	NONE	-
Os09g0116800	Similar to DNA binding protein.	EU975895	B6TCD9	Transcription factor, MADS-box
Os09g0119600	UDP-glucuronosyl/UDP-glucosyltransferase family protein.	AK059022	C4MF39	UDP-glucuronosyl/UDP-glucosyltransferase
Os09g0122200	Hypothetical conserved gene.	AK106587	Q60DP5	Myb/SANT-like domain
Os09g0123400	Hypothetical conserved gene.	EU941192	Q6K269	-
Os09g0125400	Hypothetical conserved gene.	ab initio prediction	Q6K385	-
Os09g0127800	Similar to cDNA clone:J023075G08, full insert sequence.	AK058402	B7EQ85	Coatomer, WD associated region
Os09g0129301	Non-protein coding transcript.	AK058758	NONE	-
Os09g0129800	Conserved hypothetical protein.	ab initio prediction	Q6ERA9	-
Os09g0133800	Conserved hypothetical protein.	AK069372	A2YYQ1	-
Os09g0134900	Hypothetical gene.	AK063225	longestORF	-
Os09g0240500	Similar to Sulfate transporter 4.1.	AK287664	B6SXI4	Sulphate transporter/antisigma-factor antagonist STAS
Os09g0241200	Hypothetical protein.	AK120155	longestORF	-
Os09g0242000	Similar to F-box domain containing protein, expressed.	ab initio prediction	D8L9K3	-
Os09g0247700	Similar to Zinc finger, ZZ-type%3B Zinc finger, C2H2-type.	AK059400	A2Q2P2	-
Os09g0251400	Non-protein coding transcript.	AK059379	NONE	-
Os09g0253775	Hypothetical protein.	AK372466	longestORF	-
Os09g0264011	Conserved hypothetical protein.	AK372466	Q6K2H9	-
Os09g0273600	Hypothetical gene.	CT835836	longestORF	-
Os09g0311600	Similar to NBS-LRR type resistance protein (Fragment).	AK102321	Q69KE4	Disease resistance protein
Os09g0325140	Hypothetical conserved gene.	ab initio prediction	D7UPN3	-
Os09g0334600	Similar to Cytochrome b5.	AK335015	B6UG94	Cytochrome b5
Os09g0343600	Non-protein coding transcript.	AK061057	NONE	-
Os09g0365450	Similar to DNA binding protein.	AK367679	NP_001146992.1	-
Os09g0376900	Similar to Potassium transporter 13 (AtPOT13) (AtKT5).	AK070831	Q6H4R6	K+ potassium transporter
Os09g0380400	Conserved hypothetical protein.	AK073073,AK063909	B8BF06	-
Os09g0386600	Conserved hypothetical protein.	AK064522	B9G3D2	-
Os09g0391501	Hypothetical gene.	CT835895	longestORF	-
Os09g0392400	Similar to Pleiotropic drug resistance protein 13.	AK072152	Q8S628	-
Os09g0395300	GARP DNA-binding protien, Leaf polarity modeling	FJ268748	Q0J235	-
Os09g0396900	Protein of unknown function DUF125, transmembrane family protein.	AK071589	NP_001150466.1	Domain of unknown function DUF125, transmembrane
Os09g0397200	Conserved hypothetical protein.	AK241559	NP_001063103.2	-
Os09g0397400	Protein of unknown function DUF231, plant domain containing protein.	AK063997	Q6ERD8	Domain of unknown function DUF231, plant
Os09g0397700	Glutathione-dependent formaldehyde-activating, GFA family protein.	AK111009	B6T550	Glutathione-dependent formaldehyde-activating family, GFA
Os09g0397800	Hypothetical gene.	AK062361	longestORF	-
Os09g0400200	Similar to Cinnamyl alcohol dehydrogenase (EC 1.1.1.195).	AK072000	longestORF	-
Os09g0400300	Similar to Cinnamyl alcohol dehydrogenase (EC 1.1.1.195).	AK067085	Q2HZE4	Alcohol dehydrogenase superfamily, zinc-type
Os09g0400350	Conserved hypothetical protein.	EU947353	NP_001173247.1	-
Os09g0406000	Similar to Uncharacterized protein OsI_031781.	AK120862	A2Z5D8	-
Os09g0406100	Similar to Uncharacterized protein OsI_030282.	AK288278	A2Z139	-
Os09g0406300	Similar to Uncharacterized protein OsI_031781.	AK288790	A2Z5D8	-
Os09g0406500	Similar to Uncharacterized protein OsI_030282.	AK288278	A2Z139	-
Os09g0426050	Hypothetical gene.	BT084142	longestORF	-
Os09g0445700	ATPase, AAA+ type, core domain containing protein.	ab initio prediction	B8BCH8	ATPase, AAA+ type, core
Os09g0447000	Homeodomain-related domain containing protein.	AY559046	Q67UE2	Homeobox
Os09g0449000	Hypothetical conserved gene.	AK288120	NP_197618.1	Zinc finger, PHD-type
Os09g0451800	Protein of unknown function DUF1644 family protein.	AK109302	XP_002462476.1	Protein of unknown function DUF1644
Os09g0452900	Glycosyl transferase, family 31 protein.	AK068378	B6TMZ4	Glycosyl transferase, family 31
Os09g0473050	Hypothetical protein.	AK372466	longestORF	-
Os09g0473400	Peptidase C12, ubiquitin carboxyl-terminal hydrolase 1 domain containing protein.	EU976366	NP_192633.2	Plant organelle RNA recognition domain
Os09g0473502	Hypothetical gene.	CT835876	longestORF	-
Os09g0473600	Hypothetical conserved gene.	EU973630	B9G446	-
Os09g0474000	bZIP transcription factor, bZIP-1 domain containing protein.	AK108319	B8BCQ0	Basic-leucine zipper
Os09g0480532	Conserved hypothetical protein.	ab initio prediction	Q69QS1	-
Os09g0480600	Conserved hypothetical protein.	AK107853	A3BZZ7	-
Os09g0499201	Hypothetical gene.	EU947570	longestORF	-
Os09g0526700	Similar to UDP-glucose 4-epimerase (EC 5.1.3.2) (Galactowaldenase) (UDP-galactose 4-epimerase)	AK073610	A6MD13	NAD-dependent epimerase/dehydratase
Os09g0526800	NC domain containing protein.	AK105121,AK060048	A6MD14	NC
Os09g0527500	Similar to MEG5.	AK110546	B7EM90	RNA recognition motif domain
Os09g0527600	Similar to GTP-binding protein (Fragment).	AK111543	A6MD16	Small GTPase superfamily
Os09g0527650	Hypothetical protein.	BT084300	longestORF	-
Os09g0527700	Similar to Auxin-induced protein IAA4.	AK111128	Q652A1	AUX/IAA protein
Os09g0528000	Hypothetical conserved gene.	AK060588	B9G4P1	Protein of unknown function DUF3490
Os09g0528200	Similar to Homeobox-leucine zipper protein HOX6.	AK103160	Q651Z5	Helix-turn-helix motif, lambda-like repressor
Os09g0545901	Non-protein coding transcript.	FP098421	NONE	-
Os09g0545999	Hypothetical protein.	FP098421	longestORF	-
Os09g0546100	Auxin responsive SAUR protein family protein.	AK240906	NP_001150183.1	Auxin responsive SAUR protein
Os09g0546200	Similar to auxin induced protein.	CT837633	NP_001105462.1	Auxin responsive SAUR protein
Os09g0546400	Similar to Auxin induced protein.	AK072378	longestORF	-
Os09g0546700	Similar to SAUR55 - auxin-responsive SAUR family member.	EU975305	B6TXI9	Auxin responsive SAUR protein
Os09g0546900	Similar to Auxin induced protein.	AK062365	A2Z3Q4	Auxin responsive SAUR protein
Os09g0546950	Non-protein coding transcript.	FP098421	NONE	-
Os09g0547000	Similar to Auxin induced protein.	AK062728	GeneMark	Auxin responsive SAUR protein
Os09g0547033	Similar to SAUR44 - auxin-responsive SAUR family member.	AK287456	NP_001149574.1	Auxin responsive SAUR protein
Os09g0547067	Conserved hypothetical protein.	FP098421	A3C166	-
Os09g0548083	Non-protein coding transcript.	tplb0058a02 (Wheat FLcDNA)	NONE	-
Os09g0548200	Peptidoglycan-binding Lysin subgroup domain containing protein.	AK060664	Q651P3	Peptidoglycan-binding Lysin subgroup
Os09g0549425	Conserved hypothetical protein.	AK374610	F2EEN4	-
Os09g0549500	Nucleotide-binding, alpha-beta plait domain containing protein.	FP096667	NP_001150292.1	RNA recognition motif domain
Os09g0551100	Hypothetical protein.	AK107540	longestORF	-
Os09g0551251	Similar to H0105C05.10 protein.	ab initio prediction	Q25A21	Protein kinase, catalytic domain
Os09g0552500	RmlC-like jelly roll fold domain containing protein.	AK108790	B9G4Y6	Cupin 1
Os09g0552800	No apical meristem (NAM) protein domain containing protein.	AK361030	B9G4Y8	No apical meristem
Os09g0552900	Similar to NAC domain protein, IPR003441.	EU942489	XP_002318332.1	No apical meristem

Os09g0553100	Similar to Histone H4.	CU406630	B6T0P4	Histone H4
Os09g0553300	NUDIX hydrolase domain containing protein.	AK109549	NP_565965.1	NUDIX hydrolase domain
Os09g0553700	Similar to PRLI-interacting factor A (Fragment).	AK070189	B6TGN5	-
Os09g0553750	Hypothetical gene.	EU970031	longestORF	-
Os09g0555400	Pentatricopeptide repeat domain containing protein.	AK073402	A2Z3X5	Pentatricopeptide repeat
Os09g0555450	Conserved hypothetical protein.	EU950356	F2EDP0	-
Os09g0555700	Zinc finger, C2H2-type domain containing protein.	AK061264	Q5UDB7	Zinc finger, C2H2
Os09g0564400	Hypothetical conserved gene.	Z97023	B9G543	Peptidase, cysteine peptidase active site
Os09g0567300	Similar to Monodehydroascorbate reductase (EC 1.6.5.4) (MDAR) (Ascorbate free radical reductase	AK071558	Q9XFZ3	Pyridine nucleotide-disulphide oxidoreductase, NAD-binding domain
Os09g0568000	SUZ domain domain containing protein.	AK120601	Q652Q6	SUZ domain
Os09g0568100	Conserved hypothetical protein.	FP092518	B9G564	-
Os09g0568400	Similar to 60S ribosomal protein L40 (CEP52).	AK059386	Q7XYD4	Ribosomal protein L40e
Os09g0568500	Germin family protein.	AK108987	Q84XR7	Germin
Os09g0568700	RmlC-like jelly roll fold domain containing protein.	AK109010	Q84XR7	Germin
Os09g0568900	Similar to predicted protein.	AK059442	XP_002879140.1	-
Os09g0569000	Hypothetical gene.	AK108627	longestORF	-
Os09g0569100	Haloacid dehalogenase-like hydrolase domain containing protein.	AK071860	B4FI81	Haloacid dehalogenase-like hydrolase
Os09g0569200	Similar to Beta-amylase (EC 3.2.1.2) (1,4-alpha-D-glucan maltohydrolase).	AK070300	B6SVZ0	Glycoside hydrolase, family 14B, plant
Os09g0569300	Similar to calmodulin-binding heat-shock protein.	AK069587	NP_001149987.1	Lipase, class 3
Os09g0569400	Beta-lactamase-like domain containing protein.	AK070608,AK063384	B6U1S6	Beta-lactamase-like
Os09g0569450	Conserved hypothetical protein.	AK242543	NP_001175993.1	-
Os09g0569700	Conserved hypothetical protein.	AK108778	NP_001063979.1	-
Os09g0569780	Conserved hypothetical protein.	AK241041	NP_001175963.1	-
Os09g0569800	Serine/threonine protein kinase-related domain containing protein.	ab initio prediction	NP_001063980.1	Protein kinase, catalytic domain
Os09g0570100	Protein kinase, catalytic domain domain containing protein.	AK061135	B9G572	Protein kinase, catalytic domain
Os09g0570150	Hypothetical conserved gene.	EU953102	B6SK37	-
Os09g0570300	Similar to Short-chain dehydrogenase Tic32.	AK061532	B4FKX6	Short-chain dehydrogenase/reductase SDR
Os09g0570400	Non-protein coding transcript.	AK061710	NONE	-
Os09g0570500	Zinc finger, RING-type domain containing protein.	AK069259	B9G575	Zinc finger, RING-type
Os09g0570600	Non-protein coding transcript.	AK105456	NONE	-
Os09g0570850	Similar to Histone H2B.	ab initio prediction	B6SGC3	Histone H2B
Os09g0571000	Protein of unknown function DUF966 family protein.	ab initio prediction	Q651B3	Protein of unknown function DUF966
Os09g0571033	Conserved hypothetical protein.	AK241104	NP_001175997.1	-
Os09g0571100	Pectin lyase fold/virulence factor domain containing protein.	AK106869	C5X6M1	Pectinesterase, catalytic
Os09g0571200	C2 domain containing protein.	AK062604	B6TT83	C2 calcium-dependent membrane targeting
Os09g0571500	Lipase, class 3 family protein.	AK071951	NP_181773.2	Lipase, class 3
Os09g0572000	Pathogenesis-related transcriptional factor and ERF domain containing protein.	AK241281	XP_002306842.1	Pathogenesis-related transcriptional factor/ERF, DNA-binding
Os09g0572550	Hypothetical gene.	AK243102	longestORF	-
Os09g0572600	Similar to Receptor protein kinase-like protein.	AK070226	NP_188689.1	Protein kinase, catalytic domain
Os09g0572950	Non-protein coding transcript.	tplb0034e16 (Wheat FLcDNA)	NONE	-
Os09g0573000	Pentatricopeptide repeat domain containing protein.	AK073399	B9G584	Pentatricopeptide repeat
Os09g0573100	Ubiquitin domain containing protein.	AK121232	XP_002881393.1	Uncharacterised domain SAYSvFN
Os09g0573150	AIG2-like domain containing protein.	tplb0010b04 (Wheat FLcDNA)	Q5Z481	AIG2-like
Os10g0116650	Hypothetical protein.	tplb0029l08 (Wheat FLcDNA)	longestORF	-
Os10g0116701	Hypothetical conserved gene.	ab initio prediction	Q7XH75	EGF-type aspartate/asparagine hydroxylation site
Os10g0122100	Hypothetical protein.	AK372466	longestORF	-
Os10g0141300	Similar to Protein kinase domain containing protein.	ab initio prediction	Q7XGY5	Protein kinase, catalytic domain
Os10g0141400	Similar to 26S proteasome non-ATPase regulatory subunit 4.	BT063362	NP_001150010.1	von Willebrand factor, type A
Os10g0141900	Queuine/other tRNA-ribosyltransferase family protein.	AK106467	Q948C4	Queuine/other tRNA-ribosyltransferase
Os10g0142100	Similar to Zinc finger, C3HC4 type family protein, expressed.	AK058523	Q10A50	Zinc finger, RING-type
Os10g0142301	Non-protein coding transcript.	FP100510	NONE	-
Os10g0142500	Cyclin-like F-box domain containing protein.	AK072788	Q948C2	F-box domain, cyclin-like
Os10g0144000	Conserved hypothetical protein.	EU945087	NP_001064152.1	-
Os10g0192301	Non-protein coding transcript.	FP100159	NONE	-
Os10g0208900	Hypothetical conserved gene.	AK109751	Q53NH3	-
Os10g0320201	Hypothetical protein.	EU942896	longestORF	-
Os10g0339650	Non-protein coding transcript.	AK109165	NONE	-
Os10g0340950	Non-protein coding transcript.	EU941912	NONE	-
Os10g0351414	Endonuclease/exonuclease/phosphatase domain containing protein.	tplb0014n19 (Wheat FLcDNA)	NP_001057661.2	Endonuclease/exonuclease/phosphatase
Os10g0355800	Similar to ATP synthase CF1 beta subunit.	ab initio prediction	NP_039390.1	ATPase, F1/V1/A1 complex, alpha/beta subunit, nucleotide-binding domain
Os10g0356000	Similar to ribulose-1,5-bisphosphate carboxylase/oxygenase large subunit.	ab initio prediction	NP_039391.1	Ribulose bisphosphate carboxylase, large subunit, C-terminal
Os10g0391100	Conserved hypothetical protein.	AK063378	Q7XEZ9	-
Os10g0391200	Cyclin-like F-box domain containing protein.	EU962621	NP_001064509.1	F-box domain, cyclin-like
Os10g0391300	DNA-binding SAP domain containing protein.	AK374966	B8BGM9	DNA-binding SAP
Os10g0391400	Tify domain containing protein.	AK107854	B6U6B4	Tify
Os10g0411500	IQ calmodulin-binding region domain containing protein.	AK072572	B8BGS6	-
Os10g0411600	Hypothetical protein.	AK108669	longestORF	-
Os10g0411650	Non-protein coding transcript.	EU947431	NONE	-
Os10g0411700	Similar to S28 ribosomal protein (Fragment).	AK058209,AK121099	Q7X9K4	-
Os10g0412100	Similar to Endonuclease/Exonuclease/phosphatase family protein, expressed.	AK102038	Q10R98	Endonuclease/exonuclease/phosphatase
Os10g0413400	Similar to Glycerol-3-phosphate acyltransferase 6 (EC 2.3.1.15) (AtGPAT6).	BT039538	NP_001176155.1	Phospholipid/glycerol acyltransferase
Os10g0429600	Similar to BTB/POZ domain containing protein.	AK369602	Q7XEA4	BTB/POZ-like
Os10g0430800	Conserved hypothetical protein.	AK111261	A3C4Z6	-
Os10g0431000	Similar to DNA-binding protein-like.	AK106568	NP_199554.2	Zinc finger, RING-type
Os10g0432001	Hypothetical gene.	EU943065	longestORF	-
Os10g0432200	Conserved hypothetical protein.	AK106572	Q337X2	-
Os10g0432300	Non-protein coding transcript.	AK111369	NONE	-
Os10g0434200	Similar to BTB/POZ domain containing protein.	AK369602	Q337W6	BTB/POZ-like
Os10g0438000	Similar to Potassium channel tetramerisation domain-containing protein 9.	ab initio prediction	B6TKK7	BTB/POZ-like
Os10g0438100	Similar to Fimbriata-associated protein (Fragment).	tplb0006i07 (Wheat FLcDNA)	NP_001064680.1	SKP1 component
Os10g0438800	Similar to RING-H2 finger protein ATL2E.	AK063228	NP_001151998.1	Zinc finger, RING-type
Os10g0439333	Similar to BTB/POZ domain containing protein.	AK376276	Q7XE33	BTB/POZ-like
Os10g0468250	Putative harbinger transposase-derived nuclease domain containing protein.	AK335074	Q8S819	Putative harbinger transposase-derived nuclease
Os10g0468301	Hypothetical conserved gene.	AK106587	Q60DP5	Myb/SANT-like domain
Os10g0472501	Hypothetical conserved gene.	tplb0015l09 (Wheat FLcDNA)	Q9FW60	-
Os10g0478350	Hypothetical protein.	EU971393	longestORF	-
Os10g0479800	Protein of unknown function DUF1336 domain containing protein.	AK107572	NP_180399.2	Domain of unknown function DUF1336
Os10g0488400	Protein of unknown function DUF761, plant domain containing protein.	AK108465	Q8W5I9	Protein of unknown function DUF761, plant
Os10g0492000	Conserved hypothetical protein.	AK064354	B9G6E3	-
Os10g0492101	Hypothetical conserved gene.	CT841749	Q9FWV2	-
Os10g0492200	Glycosyltransferase AER61, uncharacterized domain containing protein.	AK109476	NP_001105165.1	Glycosyltransferase AER61, uncharacterised
Os10g0493600	Similar to Alpha-galactosidase (Fragment).	AK058373	O04944	Glycosyl hydrolase, family 13, all-beta
Os10g0493800	Small nuclear RNA activating complex (SNAPc), subunit SNAP43 domain containing protein.	AK108599	F2D1V4	Small nuclear RNA activating complex
Os10g0493850	Hypothetical protein.	tplb0029b01 (Wheat FLcDNA)	longestORF	-
Os10g0493900	Protein of unknown function DUF6, transmembrane domain containing protein.	AK071193	XP_002882247.1	Drug/metabolite transporter
Os10g0494000	Protein of unknown function DUF789 family protein.	AK106067,AK071373	A2Z8W3	Protein of unknown function DUF789
Os10g0495050	Non-protein coding transcript.	EU946286	NONE	-
Os10g0497500	K Homology, type 1, subgroup domain containing protein.	AK107213	A3C631	K Homology
Os10g0497550	Non-protein coding transcript.	EU946286	NONE	-
Os10g0503550	Non-protein coding transcript.	CT835611	NONE	-
Os10g0504650	Similar to nonspecific lipid-transfer protein.	ab initio prediction	Q7G2C5	Plant lipid transfer protein/seed storage/trypsin-alpha amylase inhibitor
Os10g0516551	Hypothetical protein.	BT088333	longestORF	-
Os10g0518150	Non-protein coding transcript.	BT016918	NONE	-
Os10g0524300	Peptidoglycan-binding lysin domain domain containing protein.	AK063244	NP_001150468.1	Peptidoglycan-binding lysin domain
Os10g0524400	Similar to Phospholipase D beta 2.	AK121075	B9G6P8	C2 calcium-dependent membrane targeting
Os10g0524766	Hypothetical gene.	EU941347	longestORF	-
Os10g0525000	Similar to Cytochrome P450 family protein, expressed.	AK105884	Q109D2	Cytochrome P450
Os10g0525101	Hypothetical gene.	BT065154	longestORF	-
Os10g0525301	Hypothetical gene.	BT065154	longestORF	-
Os10g0527601	Glutathione S-transferase, C-terminal-like domain containing protein.	ab initio prediction	Q6WSC2	Glutathione S-transferase, N-terminal
Os10g0528900	Similar to Tau class GST protein 3.	AK059726	Q6WSC2	Glutathione S-transferase, N-terminal
Os10g0529400	Similar to Glutathione S-transferase GSTU6.	AK102196	B6U486	Glutathione S-transferase, N-terminal
Os10g0529450	Hypothetical protein.	AK364077	longestORF	-
Os10g0530300	Similar to Glutathione S-transferase GST 42 (Fragment).	ab initio prediction	Q9FQ97	Glutathione S-transferase, C-terminal
Os10g0530800	Hypothetical protein.	AK111429	longestORF	-

Os10g0530900	Similar to Glutathione S-transferase GST 30 (EC 2.5.1.18).	AK103699,AK059760	Q8RW01	Glutathione S-transferase, N-terminal
Os10g0532100	Similar to Amine oxidase, flavin-containing.	AK065403	Q336Y0	Amine oxidase
Os10g0532150	Similar to Amine oxidase, flavin-containing family protein, expressed.	AK372474	Q336Y0	-
Os10g0532600	Protein of unknown function DUF623, plant domain containing protein.	AK060309	NP_001146915.1	Domain of unknown function DUF623
Os10g0533200	NAD(P)-binding domain containing protein.	AK106036	NP_001149828.1	Ornithine cyclodeaminase/mu-crystallin
Os10g0534500	Similar to Resistance protein candidate (Fragment).	AK070172	Q8LN27	Protein kinase, catalytic domain
Os10g0534600	Hypothetical protein.	AK103845	longestORF	-
Os10g0539900	Similar to Hexose transporter.	AK059705	B6U6U2	General substrate transporter
Os10g0540100	Pentatricopeptide repeat domain containing protein.	AK074016	Q9FRJ8	Pentatricopeptide repeat
Os10g0541200	Similar to Protein phosphatase 2C containing protein, expressed.	AK063496	Q336W3	Protein phosphatase 2C, manganese/magnesium aspartate binding site
Os10g0542800	Similar to ATP binding protein.	AK121234	NP_001151339.1	Tetratricopeptide-like helical
Os10g0543800	Similar to Glutathione S-transferase GST 11 (EC 2.5.1.18).	CT836577	NP_001104983.1	Glutathione S-transferase, N-terminal
Os10g0544933	Non-protein coding transcript.	EU943254	NONE	-
Os10g0544966	Hypothetical protein.	tplb0043b08 (Wheat FLcDNA)	longestORF	-
Os10g0545000	Similar to magnesium transporter CorA-like family protein.	AK069472	NP_567076.1	Fibronectin-attachment
Os10g0545100	Similar to predicted protein.	AK287840	B9G1Z7	Uncharacterised conserved protein UCP014543
Os10g0545300	Zinc finger, CCHC retroviral-type domain containing protein.	AK065156,AK121559	NP_001149324.1	Zinc finger, CCHC-type
Os10g0545700	Similar to Rhodanese-like protein.	AK073024	Q66T88	Rhodanese-like
Os10g0545800	Nucleic acid-binding, OB-fold domain containing protein.	AK103222	NP_001152237.1	CcmE/CycJ protein
Os10g0546400	Conserved hypothetical protein.	AK069919	B9G6V0	-
Os10g0546600	Similar to Chloroplast carotenoid epsilon-ring hydroxylase.	AK065689	NP_190881.2	Cytochrome P450
Os10g0547200	Harpin-induced 1 domain containing protein.	AK060129	A2Z9Y3	Late embryogenesis abundant protein, LEA-14
Os10g0548400	Similar to F25C20.9.	EU973528	Q9SAA1	-
Os10g0549500	Similar to Branched-chain-amino-acid aminotransferase.	AK330986	A2ZA03	Aminotransferase, class IV
Os10g0549650	Hypothetical protein.	EU951562	longestORF	-
Os10g0550066	Hypothetical conserved gene.	BT067999	XP_002466024.1	Mediator complex, subunit Med12
Os10g0550100	Conserved hypothetical protein.	AK106583	Q94LW0	-
Os10g0551100	Conserved hypothetical protein.	AK106659	Q9FWN4	-
Os10g0551700	Plant lipid transfer protein and hydrophobic protein, helical domain containing protein.	AK107851	A2ZA19	Plant lipid transfer protein/seed storage/trypsin-alpha amylase inhibitor
Os10g0551800	Root-specific protein (RCc2 protein).	L27209	A2ZA20	Plant lipid transfer protein/seed storage/trypsin-alpha amylase inhibitor
Os10g0553900	Hypothetical conserved gene.	AK060446	A2ZA32	Protein of unknown function DUF863, plant
Os10g0554050	Non-protein coding transcript.	tplb0022l14 (Wheat FLcDNA)	NONE	-
Os10g0554100	Similar to Oxysterol-binding protein.	AK071048	C3SAC1	Oxysterol-binding protein
Os10g0556100	Similar to beta-expansin EXPB4.	AK101357	Q94LR4	Major pollen allergen Lol pI
Os10g0556600	Similar to predicted protein.	EU971180	NP_171710.3	CCR4-Not complex, Not1 subunit, domain of unknown function DUF3819
Os10g0557133	Similar to H0315A08.1 protein.	FP099421	Q01I63	Ribonuclease H1, N-terminal
Os10g0557366	Conserved hypothetical protein.	FP100733	A2XC28	-
Os10g0557600	Similar to GATA transcription factor 9.	CT837819	NP_001147669.1	Zinc finger, GATA-type
Os10g0557700	Conserved hypothetical protein.	AK241699	NP_001176270.1	-
Os10g0557750	Hypothetical genes.	ab initio prediction	NONE	-
Os10g0557800	Conserved hypothetical protein.	AK073483	B9G6Y4	-
Os10g0557900	Peptidase M10A and M12B, matrixin and adamalysin family protein.	CT835768	Q8LET7	Peptidase M10, metallopeptidase
Os10g0558200	2OG-Fe(II) oxygenase domain containing protein.	ab initio prediction	Q7XC87	Oxoglutarate/iron-dependent oxygenase
Os10g0558301	Hypothetical gene.	EU953848	longestORF	-
Os10g0558650	Non-protein coding transcript.	EU946768	NONE	-
Os10g0558700	2OG-Fe(II) oxygenase domain containing protein.	AK067086	Q336T3	Oxoglutarate/iron-dependent oxygenase
Os10g0558725	Hypothetical protein.	EU953848	longestORF	-
Os10g0558850	Hypothetical protein.	tplb0043f10 (Wheat FLcDNA)	longestORF	-
Os10g0559200	Similar to Oxidoreductase, 2OG-Fe oxygenase family protein, expressed.	AK066405	Q336T1	-
Os10g0559300	Hypothetical protein.	AK063546	longestORF	-
Os10g0559400	Similar to cDNA clone:J023138C05, full insert sequence.	AK064149	Q7XC75	-
Os10g0559700	Similar to Agenet domain containing protein, expressed.	AK070955	Q7XC74	-
Os10g0559866	Hypothetical protein.	ab initio prediction	NONE	-
Os10g0559900	Ribosomal protein L18/L5 domain containing protein.	AK070091	NP_849617.1	Ribosomal protein L18/L5
Os10g0560000	Protein of unknown function DUF828, plant family protein.	AK066334	Q8SB99	Domain of unknown function DUF828
Os10g0560400	Similar to CONSTANS-like protein CO9 (Fragment).	AK109732	Q8SBA3	CCT domain
Os10g0560500	Conserved hypothetical protein.	AK100157	Q8SBA5	-
Os10g0560850	Hypothetical gene.	EU940765	longestORF	-
Os10g0561000	Hypothetical conserved gene.	BT064708	Q9AUT7	-
Os10g0561200	Similar to CigA protein.	AK066634	Q9AUT5	-
Os10g0561250	Hypothetical protein.	tplb0041o18 (Wheat FLcDNA)	longestORF	-
Os10g0561300	Similar to Monosaccharid transporter.	AK068343	NP_001149551.1	Sugar/inositol transporter
Os10g0562000	Similar to glycoprotein.	AK106294	NP_001151220.1	-
Os10g0566800	Haem peroxidase, plant/fungal/bacterial family protein.	AK063299	Q5U1H3	Plant peroxidase
Os10g0567000	Camphor resistance CrcB protein family protein.	AK103373	NP_001147074.1	Camphor resistance CrcB protein
Os10g0567100	Similar to Chlorophyll b synthase (Fragment).	AK063367	Q8S7E1	Pheophorbide a oxygenase
Os10g0567200	Hypothetical protein.	AK100517	longestORF	-
Os10g0567300	SH2 motif domain containing protein.	AK070829	Q336Q9	SH2 motif
Os10g0567400	Similar to Rieske.	AK065124,AK071899	Q8S7E1	Pheophorbide a oxygenase
Os10g0567500	RNA-directed DNA polymerase (reverse transcriptase) domain containing protein.	AK072336	B6SWF1	Reverse transcriptase
Os10g0567600	Uncharacterised protein family UPF0503 domain containing protein.	AK105119	Q8S7E3	Uncharacterised protein family UPF0503
Os10g0567800	Similar to predicted protein.	AK061703	NP_567094.1	Dynamin central domain
Os10g0567900	Similar to F-box protein interaction domain containing protein, expressed.	AK064083	Q108X5	F-box associated interaction domain
Os10g0571250	Non-protein coding transcript.	EU950639	NONE	-
Os10g0571600	No apical meristem (NAM) protein domain containing protein.	AK110684	XP_002329572.1	No apical meristem
Os10g0572100	Uncharacterised protein family UPF0005 domain containing protein.	AK073002,AK105742	O49696	Malate transporter, aliminium toerance
Os10g0572400	Conserved hypothetical protein.	AK062550	Q8S7M6	-
Os10g0572500	Zinc finger, RING/FYVE/PHD-type domain containing protein.	AK067954	Q108W5	Zinc finger, FYVE/PHD-type
Os10g0575401	VQ domain containing protein.	BT042864	NP_188465.1	VQ
Os10g0575650	Hypothetical gene.	BT086509	longestORF	-
Os10g0575950	Similar to F5D14.6 protein.	AK241904	Q9LQM9	-
Os10g0577000	Conserved hypothetical protein.	AK059043	Q8W3G9	-
Os10g0578500	Hypothetical conserved gene.	ab initio prediction	Q7XBT9	Pentatricopeptide repeat
Os10g0578900	Similar to CTP:phosphorylcholine cytidylyltransferase (EC 2.7.7.15).	AK073939	NP_001150315.1	Cytidylyltransferase
Os10g0579150	Hypothetical gene.	EU951391	longestORF	-
Os10g0579400	Conserved hypothetical protein.	AK108346	Q9FRP5	-
Os10g0579800	TGF-beta receptor, type I/II extracellular region family protein.	AK121847	NP_001148010.1	Oligopeptide transporter
Os10g0579850	Hypothetical protein.	tplb0038k08 (Wheat FLcDNA)	longestORF	-
Os10g0580300	Serine/threonine protein kinase domain containing protein.	AK066824,AK104880	Q336M2	Protein kinase, catalytic domain
Os10g0580900	Non-protein coding transcript.	AK062821	NONE	-
Os11g0100600	Alpha/beta hydrolase family protein.	AK060331	B4FK75	Alpha/beta hydrolase fold-1
Os11g0100800	Similar to Exo70 exocyst complex subunit family protein.	AK287644	Q2RBS0	Exo70 exocyst complex subunit
Os11g0102100	WUS-type homenodomain protein, Leaf development	AM490243	A8Y7W4	-
Os11g0104900	Similar to Clathrin heavy chain.	ab initio prediction	Q39834	Clathrin, heavy chain/VPS, 7-fold repeat
Os11g0104933	Non-protein coding transcript.	AK242528	NONE	-
Os11g0111101	Similar to cDNA clone:J033057N11, full insert sequence.	AK243555	B7ES31	-
Os11g0111200	Conserved hypothetical protein.	AK109277	Q2RBH4	-
Os11g0127800	Similar to FHA domain containing protein, expressed.	AK059969,AK104179	Q2QYC9	Forkhead-associated
Os11g0127900	Similar to 40S ribosomal protein S16.	AK059550	Q0IQF7	Ribosomal protein S9
Os11g0127951	Hypothetical protein.	ab initio prediction	NONE	-
Os11g0128500	Similar to Myb factor.	AK241549	O04109	SANT domain, DNA binding
Os11g0128600	Conserved hypothetical protein.	ab initio prediction	NP_001065637.1	-
Os11g0128700	Mad3/BUB1 homology region 1 domain containing protein.	AK102502	Q2RB24	Mad3/BUB1 homology region 1
Os11g0128800	Similar to Homoserine dehydrogenase-like protein.	AK071434	B8AES5	Homoserine dehydrogenase, catalytic
Os11g0128932	Hypothetical gene.	BT054648	longestORF	-
Os11g0129500	Similar to GDSL-like Lipase/Acylhydrolase family protein, expressed.	AK107889	Q2RB18	-
Os11g0130100	Conserved hypothetical protein.	AK063914	Q2RB13	-
Os11g0130400	Harpin-induced 1 domain containing protein.	AK243509	NP_001065651.2	Late embryogenesis abundant protein, LEA-14
Os11g0130500	Cyclin-like F-box domain containing protein.	AK241516	Q2RB09	F-box domain, cyclin-like
Os11g0130600	Conserved hypothetical protein.	AK066342	NP_001066054.1	-
Os11g0130700	Conserved hypothetical protein.	ab initio prediction	B9G976	-
Os11g0131100	VQ domain containing protein.	CT835703	Q2RB04	VQ
Os11g0131400	Hypothetical conserved gene.	AK109466	Q2RB01	Pentatricopeptide repeat
Os11g0131500	Hypothetical protein.	BT085496	longestORF	-
Os11g0131600	Similar to YDG/SRA domain containing protein.	BT055690	Q2RB00	SET domain

Os11g0131700	Zinc finger, Tim10/DDP-type domain containing protein.	AK243327	B6SLJ2	Mitochondrial inner membrane translocase complex, Tim8/9/10/13-zinc finger-like
Os11g0132400	Uncharacterised protein family UPF0414 domain containing protein.	AK059074	NP_001176772.1	Protein of unknown function DUF872, transmembrane
Os11g0133700	Similar to Glucosamine-fructose-6-phosphate aminotransferase, isomerizing family protein, expresse	AK058784	Q2RAY0	-
Os11g0133850	Hypothetical gene.	EU946073	longestORF	-
Os11g0134400	Calmodulin-like protein 3.	Q0IQB6	Q0IQB6	Calcium-binding EF-hand
Os11g0137050	Hypothetical protein.	tplb0036f07 (Wheat FLcDNA)	longestORF	-
Os11g0137250	Hypothetical gene.	AK242555	longestORF	-
Os11g0144950	Hypothetical protein.	FP101342	longestORF	-
Os11g0147500	Heavy metal transport/detoxification protein domain containing protein.	AK105759	Q2QXS5	Heavy metal-associated domain, HMA
Os11g0166800	Transcription elongation factor, TFIIS/CRSP70, N-terminal domain containing protein.	AK070928	Q53PV2	Transcription factor IIS, N-terminal
Os11g0166866	Non-protein coding transcript.	BT065504	NONE	-
Os11g0166932	Non-protein coding transcript.	EU949846	NONE	-
Os11g0167500	Similar to Serine esterase family protein, expressed.	BT083721	NP_564732.1	Domain of unknown function DUF676, lipase-like
Os11g0168000	Protein of unknown function DUF1278 family protein.	AK062599	Q9SES8	Protein of unknown function DUF784
Os11g0168200	60S ribosomal protein L3.	AK073392,AK101469	A5D934	Ribosomal protein L3
Os11g0168250	Hypothetical gene.	BT016883	longestORF	-
Os11g0168300	Alba, DNA/RNA-binding protein family protein.	AK061362,AK104405	B6T7F3	DNA/RNA-binding protein Alba-like
Os11g0168600	Protein kinase, core domain containing protein.	AK063805	Q53JG3	Protein kinase, catalytic domain
Os11g0180000	Plant disease resistance response protein family protein.	AK060077	NP_001150515.1	Plant disease resistance response protein
Os11g0180100	Conserved hypothetical protein.	AK375448	Q53NQ8	-
Os11g0180900	Myb transcription factor domain containing protein.	BT069656	Q6SL45	SANT domain, DNA binding
Os11g0181000	Hypothetical protein.	EU971393	longestORF	-
Os11g0181200	Hypothetical protein.	AK063747	longestORF	-
Os11g0182100	Protein of unknown function DUF688 domain containing protein.	AK107888	Q53MM1	Protein of unknown function DUF688
Os11g0182400	WD40/YVTN repeat-like domain containing protein.	AK112089	B6TCZ0	WD40 repeat
Os11g0193300	Hypothetical conserved gene.	AK109751	Q53NH3	-
Os11g0201175	Similar to H0315A08.1 protein.	FP099421	Q01I63	-
Os11g0203400	Hypothetical conserved gene.	AK105579	Q6ZI94	-
Os11g0222800	Similar to LGC1.	EU947580	Q53P07	-
Os11g0272900	Conserved hypothetical protein.	AK068971	Q53MK6	-
Os11g0300501	Non-protein coding transcript.	AK242366	NONE	-
Os11g0440200	Similar to GDA1/CD39 family protein, expressed.	ab initio prediction	Q53KM8	Nucleoside phosphatase GDA1/CD39
Os11g0460851	Non-protein coding transcript.	tplb0037b22 (Wheat FLcDNA)	NONE	-
Os11g0574725	Hypothetical conserved gene.	ab initio prediction	Q2R289	F-box domain, cyclin-like
Os11g0596000	Hypothetical conserved gene.	AK288199	Q2R1R7	-
Os11g0623650	Non-protein coding transcript.	BT065526	NONE	-
Os11g0629900	Conserved hypothetical protein.	AK288199	Q2R0W5	-
Os11g0656500	Integrase, catalytic core domain containing protein.	AK072370	Q01M13	Integrase, catalytic core
Os11g0671600	Conserved hypothetical protein.	AK288199	Q2QZU2	-
Os11g0697350	Conserved hypothetical protein.	AK241561	NP_001176733.1	-
Os11g0697600	Hypothetical gene.	CT841618	longestORF	-
Os11g0699532	Hypothetical gene.	CT841618	longestORF	-
Os12g0100200	Conserved hypothetical protein.	AK369990	NP_001176293.1	-
Os12g0100700	Similar to Exo70 exocyst complex subunit family protein.	AK287644	Q2RBS0	Exo70 exocyst complex subunit
Os12g0101000	Similar to CCT motif family protein.	AK100957	Q2QYZ4	CCT domain
Os12g0101600	WUS-type homenodomain protein, Leaf development	AM490243	A8Y7W4	-
Os12g0101901	Hypothetical gene.	EU949907	longestORF	-
Os12g0102100	Alcohol dehydrogenase superfamily, zinc-containing protein.	AK068187	C5MRG3	Alcohol dehydrogenase superfamily, zinc-type
Os12g0102300	Similar to WRKY transcription factor 57.	AK242855	Q6IEM4	-
Os12g0106801	Hypothetical genes.	ab initio prediction	NONE	-
Os12g0110150	Hypothetical gene.	EU949682	longestORF	-
Os12g0118400	Similar to Inwardly rectifying potassium channel subunit.	AK062407	Q6ZXS4	Potassium channel, voltage-dependent, EAG/ELK/ERG
Os12g0124200	Similar to 40S ribosomal protein S16.	AK121503,AK058697	Q0IQF7	Ribosomal protein S9
Os12g0124700	CDC45-like protein family protein.	AK073156	NP_189146.1	CDC45 family
Os12g0124900	Non-protein coding transcript.	AK106719	NONE	-
Os12g0125000	Myb factor.	AK061437	O04109	SANT domain, DNA binding
Os12g0125300	Mitotic checkpoint serine/threonine protein kinase, Bub1 domain containing protein.	AK102502	A2ZHN6	Mad3/BUB1 homology region 1
Os12g0125400	Similar to Homoserine dehydrogenase-like protein.	AK062006	B8BLT4	Homoserine dehydrogenase, catalytic
Os12g0125550	Hypothetical gene.	BT054648	longestORF	-
Os12g0125850	Hypothetical gene.	BT054648	longestORF	-
Os12g0126100	Similar to GDSL-like Lipase/Acylhydrolase family protein, expressed.	BT033892	Q2RB18	Lipase, GDSL
Os12g0126200	Engulfment and cell motility, ELM domain containing protein.	AK099983	NP_566211.1	Engulfment/cell motility, ELMO
Os12g0127100	Similar to Nse1 non-SMC component of SMC5-6 complex family protein.	AK101224	Q2QYA0	Nse1 non-SMC component of SMC5-6 complex
Os12g0127200	Harpin-induced 1 domain containing protein.	AK122005	A2ZHQ7	Late embryogenesis abundant protein, LEA-14
Os12g0127500	Conserved hypothetical protein.	AK064595	A2ZHQ9	-
Os12g0127600	Similar to Ulp1 protease family, C-terminal catalytic domain containing protein, expressed.	AK368982	Q2QY95	Peptidase C48, SUMO/Sentrin/Ubl1
Os12g0127650	Similar to Ulp1 protease family, C-terminal catalytic domain containing protein, expressed.	AK242802	Q2QY95	-
Os12g0127800	Similar to VQ motif family protein.	AK242367	Q2QY94	VQ
Os12g0132200	Similar to CBL-interacting protein kinase 33.	AK111953	Q2RAX3	Protein kinase, catalytic domain
Os12g0132300	Similar to Calmodulin (CaM).	AK111834,AK119956	Q0IQB6	-
Os12g0132400	Similar to Grwd-pending-prov protein.	AK111507	Q1HFN6	WD40 repeat
Os12g0132500	Similar to Major facilitator superfamily antiporter.	CT833630	Q8H6D5	-
Os12g0132650	Non-protein coding transcript.	tplb0023l07 (Wheat FLcDNA)	NONE	-
Os12g0133025	Non-protein coding transcript.	tplb0023l07 (Wheat FLcDNA)	NONE	-
Os12g0133050	Similar to 60S acidic ribosomal protein P0.	AK361198	NP_001105482.1	Ribosomal protein L10/acidic P0
Os12g0133350	Non-protein coding transcript.	tplb0023l07 (Wheat FLcDNA)	NONE	-
Os12g0133700	Similar to Dual specificity protein phosphatase 19 (EC 3.1.3.48) (EC 3.1.3.16) (Stress-activated prot	CU405769	Q2HSZ6	Dual specificity phosphatase, catalytic domain
Os12g0133750	Hypothetical protein.	ab initio prediction	NONE	-
Os12g0133850	Hypothetical protein.	tplb0036f07 (Wheat FLcDNA)	longestORF	-
Os12g0134000	Similar to hydroxymethylglutaryl-CoA lyase.	AK061875	B6TKJ5	Pyruvate carboxyltransferase
Os12g0134500	Similar to Zinc finger, C3HC4 type family protein.	AK358384	Q2RAT8	Zinc finger, RING-type
Os12g0135100	Similar to Phosphoglycerate mutase family protein.	BT062522	Q2QY22	Phosphoglycerate/bisphosphoglycerate mutase, active site
Os12g0140300	FBD domain containing protein.	AK102092	B9G816	-
Os12g0140550	Hypothetical gene.	EU945989	longestORF	-
Os12g0149300	Similar to galactosyl transferase GMA12/MNN10 family protein.	AK110842	XP_002887538.1	Galactosyl transferase
Os12g0149800	Similar to G10.	AK103299	NP_001148940.1	G10 protein
Os12g0150200	Similar to Cytochrome P450 CYP94C20.	AK064287	B6STM6	Cytochrome P450
Os12g0150901	Conserved hypothetical protein.	tplb0051a13 (Wheat FLcDNA)	Q2QXM5	-
Os12g0151600	Hypothetical conserved gene.	BT061672	NP_190845.1	-
Os12g0152000	Similar to Terminal flower 1-like protein.	AF159883	Q9XGS5	Phosphatidylethanolamine-binding, conserved site
Os12g0152100	Alpha/beta hydrolase fold-1 domain containing protein.	AK070021	A6N1T8	-
Os12g0152200	Cyclin-like F-box domain containing protein.	ab initio prediction	NP_001066174.1	F-box domain, cyclin-like
Os12g0152500	Hypothetical conserved gene.	BT039089	A2ZI44	-
Os12g0152600	Pentatricopeptide repeat domain containing protein.	AK288612	NP_001066176.1	Pentatricopeptide repeat
Os12g0152700	Amino acid-binding ACT domain containing protein.	AK099473	NP_001151043.1	Amino acid-binding ACT
Os12g0152800	Rhodanese-like domain containing protein.	AK073401	NP_001152230.1	Dual specificity phosphatase, catalytic domain
Os12g0152900	bZIP transcription factor, bZIP-1 domain containing protein.	AK101620	D3U2G1	Basic-leucine zipper
Os12g0153500	Carbonic anhydrase, CAH1-like domain containing protein.	AK070276	A2ZI48	Carbonic anhydrase, alpha-class, catalytic domain
Os12g0154000	Protein of unknown function DUF250 domain containing protein.	AK105118	NP_001150661.1	Domain of unknown function DUF250
Os12g0154900	Germin-like protein 12-3, Disease resistance	Inferred from literature	Q2QXJ1	Germin
Os12g0155000	Germin-like protein 12-4, Disease resistance	CT837600	Q2QXJ0	-
Os12g0155100	Protein of unknown function DUF3049 domain containing protein.	AK060647	A2ZI76	Protein of unknown function DUF3049
Os12g0155200	Similar to Rac GTPase activating protein.	AK067300	B6SWP2	PAK-box/P21-Rho-binding
Os12g0155250	Hypothetical protein.	tplb0049d09 (Wheat FLcDNA)	longestORF	-
Os12g0155300	Conserved hypothetical protein.	AK060480	Q2QXI6	-
Os12g0155400	Similar to Leucine Rich Repeat family protein, expressed.	X79130	Q8S5D5	-
Os12g0155600	Non-protein coding transcript.	X06283	NONE	-
Os12g0156400	Dcp1-like decapping family protein.	AK072086,AK099278	NP_001149166.1	Dcp1-like decapping
Os12g0156500	Cyclin-like F-box domain containing protein.	EU969867	NP_001151212.1	F-box domain, cyclin-like
Os12g0156866	Similar to Amino acid permease family protein, expressed.	AK332141	Q2QXH2	Amino acid/polyamine transporter I
Os12g0156883	Hypothetical protein.	tplb0044b08 (Wheat FLcDNA)	longestORF	-
Os12g0156900	Pentatricopeptide repeat domain containing protein.	BT018623	Q2QXH1	Pentatricopeptide repeat
Os12g0157000	DNA-binding pseudobarrel domain domain containing protein.	AK110593	B8BNA0	DNA-binding pseudobarrel domain
Os12g0157066	Hypothetical protein.	AK375277	longestORF	-
Os12g0163000	Similar to CNGC18 (CYCLIC NUCLEOTIDE-GATED CHANNEL 18)%3B calmodulin binding / c	ab initio prediction	NP_196991.1	Cyclic nucleotide-binding domain

Os12g0169300	Conserved hypothetical protein.	AK287997	NP_001176998.1	-
Os12g0177500	Peptidase A1 domain containing protein.	AK064771	Q2QWY6	Peptidase A1
Os12g0177800	Similar to D-mannose binding lectin family protein, expressed.	AK105397	Q2QWY4	S-locus glycoprotein
Os12g0177900	Similar to Aldehyde dehydrogenase.	ab initio prediction	Q2QWY3	Aldehyde dehydrogenase, N-terminal
Os12g0178250	Similar to dehydration-responsive protein-related.	ab initio prediction	NP_001031109.1	Protein of unknown function DUF248, methyltransferase putative
Os12g0178300	Similar to ankyrin-like protein.	ab initio prediction	NP_001151565.1	Protein of unknown function DUF248, methyltransferase putative
Os12g0178600	Similar to dehydration-responsive protein-related.	ab initio prediction	NP_190676.1	Protein of unknown function DUF248, methyltransferase putative
Os12g0178633	WD40 repeat domain containing protein.	AK369075	B8BNG0	WD40 repeat
Os12g0178800	Hypothetical protein.	AK063709	longestORF	-
Os12g0179100	Hypothetical gene.	AK106975	longestORF	-
Os12g0179700	Hypothetical conserved gene.	AK068305	Q2QWX0	-
Os12g0190875	Similar to H0315A08.1 protein.	FP099421	Q01I63	Ribonuclease H1, N-terminal
Os12g0191300	Similar to Phage head-tail adaptor family protein, expressed.	BT067332	Q2QWM4	Aminotransferase, class V/Cysteine desulfurase
Os12g0191400	Conserved hypothetical protein.	BT085962	NP_001183161.1	-
Os12g0191500	Similar to Peroxidase 43 precursor (EC 1.11.1.7) (Atperox P43).	AK061040	B9V0M3	Plant peroxidase
Os12g0191901	Hypothetical gene.	CU406111	longestORF	-
Os12g0195100	Similar to amino acid permease.	EU962989	O82044	Amino acid transporter, transmembrane
Os12g0195183	Hypothetical protein.	tplb0036o08 (Wheat FLcDNA)	longestORF	-
Os12g0198500	Conserved hypothetical protein.	AK242191	Q2QWE0	-
Os12g0202700	Similar to N-methyltransferase.	CU406772	Q96565	O-methyltransferase, family 2
Os12g0203000	Similar to Cyclin-dependent protein kinase-like protein.	AK100360	Q2QW88	Protein kinase, catalytic domain
Os12g0203300	Similar to amino acid permease 1.	EU949945	NP_001149517.1	Amino acid transporter, transmembrane
Os12g0203900	Hypothetical gene.	AK072494	longestORF	-
Os12g0207500	Similar to ATP synthase subunit beta.	ab initio prediction	Q2QW50	ATPase, F1/V1/A1 complex, alpha/beta subunit, nucleotide-binding domain
Os12g0207600	Similar to ribulose-1,5-bisphosphate carboxylase/oxygenase large subunit.	ab initio prediction	YP_654221.1	Ribulose bisphosphate carboxylase, large subunit, C-terminal
Os12g0220800	Hypothetical protein.	AK108124	longestORF	-
Os12g0222800	Leucine-rich repeat, N-terminal domain containing protein.	ab initio prediction	Q2QVP4	Leucine-rich repeat
Os12g0222900	Leucine-rich repeat, N-terminal domain containing protein.	AK111528	Q2QVP4	Leucine-rich repeat
Os12g0233366	Non-protein coding transcript.	CT835611	NONE	-
Os12g0299650	Myb/SANT-like domain domain containing protein.	BT063151	NP_001176912.1	Myb/SANT-like domain
Os12g0424180	Similar to ATP synthase F0 subunit 6.	GU075810	YP_514657.1	ATPase, F0 complex, subunit A
Os12g0424300	Similar to rp13.	ab initio prediction	YP_398395.1	Ribosomal protein S13
Os12g0510766	Non-protein coding transcript.	EU942847	NONE	-
Os12g0510900	Similar to LIM domain protein WLIM-1.	AK058220	Q2QQ10	Zinc finger, LIM-type
Os12g0511750	Nucleic acid-binding, OB-fold domain containing protein.	AK287637	NP_001175163.1	Nucleic acid-binding, OB-fold
Os12g0520200	Similar to phenylalanine ammonia-lyase.	AK240729	Q2QPR0	Phenylalanine/histidine ammonia-lyase
Os12g0521101	Hypothetical protein.	tplb0048a09 (Wheat FLcDNA)	longestORF	-
Os12g0521300	Similar to HAT family dimerisation domain containing protein.	EU942994	Q2QPQ7	HAT dimerisation
Os12g0522516	Similar to ATRL5 (ARABIDOPSIS RAD-LIKE 5)%3B DNA binding / transcription factor.	tplb0003e07 (Wheat FLcDNA)	NP_564087.2	SANT domain, DNA binding
Os12g0524750	Similar to ribosomal protein S7.	ab initio prediction	YP_514635.1	Ribosomal protein S7
Os12g0538066	Similar to HAT family dimerisation domain containing protein.	AK122139	Q2QPA8	Zinc finger, BED-type predicted
Os12g0572400	Similar to SC35-like splicing factor SCL30, 30 kD.	AK065531	Q7XXQ1	RNA recognition motif domain
Os12g0572601	Hypothetical gene.	AK287811	longestORF	-
Os12g0572700	Exostosin-like domain containing protein.	ab initio prediction	NP_001177020.1	Exostosin-like
Os12g0576100	Conserved hypothetical protein.	CU406037	NP_001067107.1	-
Os12g0587116	Non-protein coding transcript.	AK358137	NONE	-
Os12g0590550	Hypothetical gene.	AK062999	longestORF	-
Os12g0592832	Hypothetical protein.	ab initio prediction	NONE	-
Os12g0594000	WD40 repeat-like domain containing protein.	AK354406	Q1EPE0	WD40 repeat
Os12g0594200	Hypothetical conserved gene.	EU942864	NP_001067177.2	-
Os12g0594201	Hypothetical gene.	CU406459	longestORF	-
Os12g0594700	Similar to F-box domain containing protein.	ab initio prediction	Q2QMR2	F-box domain, Skp2-like
Os12g0594950	Hypothetical conserved gene.	AK287432	NP_001177039.1	-
Os12g0595300	Conserved hypothetical protein.	AK106840	A3CJ25	-
Os12g0595600	Hypothetical conserved gene.	ab initio prediction	B9GE25	SKP1 component
Os12g0596401	Non-protein coding transcript.	X06283	NONE	-
Os12g0596501	Hypothetical protein.	tplb0045o13 (Wheat FLcDNA)	longestORF	-
Os12g0596600	Conserved hypothetical protein.	AK064196	B9GE28	-
Os12g0596800	Zinc finger, LIM-type domain containing protein.	AK065760	B6SPK3	Zinc finger, LIM-type
Os12g0596850	Non-protein coding transcript.	tplb0034a09 (Wheat FLcDNA)	NONE	-
Os12g0597200	Similar to MATH domain containing protein, expressed.	AK059486	Q2QMP1	-
Os12g0598000	Non-protein coding transcript.	AK242434	NONE	-
Os12g0599400	Helix-loop-helix DNA-binding domain containing protein.	ab initio prediction	Q2QML8	Helix-loop-helix DNA-binding
Os12g0599550	Similar to Helix-loop-helix DNA-binding domain containing protein.	ab initio prediction	Q2QML8	-
Os12g0599800	Pectinesterase inhibitor domain containing protein.	AK111139	A2ZME3	Pectinesterase inhibitor
Os12g0600000	Conserved hypothetical protein.	AK109945	NP_001067202.1	-
Os12g0600150	Hypothetical gene.	AK241829	longestORF	-
Os12g0600400	Similar to Pyridoxal kinase.	AK066514	Q69G22	Pyridoxal phosphate
Os12g0600550	Non-protein coding transcript.	AK288257	NONE	-
Os12g0600701	Conserved hypothetical protein.	CU406704	NP_001177048.1	-
Os12g0601000	Similar to Leucine Rich Repeat family protein, expressed.	EU973703	Q2QMK5	F-box domain, Skp2-like
Os12g0601200	Uridine kinase family protein.	AK059928	B6TCV6	Phosphoribulokinase/uridine kinase
Os12g0603300	Similar to AP2 domain containing protein, expressed.	AK242027	Q2QMI3	Pathogenesis-related transcriptional factor/ERF, DNA-binding
Os12g0603700	Similar to CBL-interacting protein kinase 4.	Q2QMI0	Q2QMI0	Protein kinase, catalytic domain
Os12g0606900	Similar to Ulp1 protease family, C-terminal catalytic domain containing protein, expressed.	AK072659	Q2QME9	-
Os12g0607000	Similar to homocysteine S-methyltransferase-2.	AK103293	NP_001105012.1	Homocysteine S-methyltransferase
Os12g0607100	Similar to Eukaryotic translation initiation factor 2 gamma subunit.	AK061482	B6TNU0	Protein synthesis factor, GTP-binding
Os12g0611500	Similar to H0315A08.1 protein.	FP099421	Q01I63	Ribonuclease H1, N-terminal
Os12g0613000	Similar to NF-YA subunit.	AK065163	B6TY54	CCAAT-binding transcription factor, subunit B
Os12g0613200	Hypothetical conserved gene.	AK069599	B9GE94	SET domain
Os12g0613850	Similar to Galactosyltransferase family.	ab initio prediction	B6SVW0	Galectin, carbohydrate recognition domain
Os12g0614100	Similar to Lipase family protein.	BT042072	Q2QM82	Lipase, class 3
Os12g0614200	Similar to Lipase family protein.	BT042072	Q2QM81	Lipase, class 3
Os12g0614201	Conserved hypothetical protein.	AK243381	NP_001177060.1	-
Os12g0614600	Serine/threonine protein kinase domain containing protein.	AK106290	NP_001106051.1	Protein kinase, catalytic domain
Os12g0617100	Similar to Translation initiation factor eIF-2B delta subunit (eIF-2B GDP-GTP exchange factor) (Gu	AK103148	NP_001159071.1	Initiation factor 2B-related
Os12g0617175	Non-protein coding transcript.	tplb0051c11 (Wheat FLcDNA)	NONE	-
Os12g0621766	Hypothetical protein.	EU947100	GeneMark	-

Pedigree-based genome re-sequencing reveals genetic variation patterns of elite backbone varieties during modern rice improvement

Xingfei Zheng, Lanzhi Li, Fan Liang, Changjun Tan, Shuzhu Tang, Sibin Yu, Ying Diao, Shuangcheng Li,and Zhongli Hu

Supplementary Table S15 Putative genes with strong selective pressure that specially exist in the SH527 pedigree

Chromosome	Gene name	Start sites	End site	Special genes for SH527 pedigree			Annotatioin
				Low diversity	Zero-diversity	SH527 sweeps	
chr01	Os01g0929800	40799614	40800553	√	√	×	Hypothetical gene.
chr01	Os01g0869750	37695077	37696475	√	√	×	Conserved hypothetical protein.
chr01	Os01g0868400	37625827	37634176	√	√	×	Hypothetical protein.
chr01	Os01g0193250	5014697	5015197	√	√	×	Hypothetical gene.
chr01	Os01g0170900	3665042	3666044	√	√	×	Conserved hypothetical protein.
chr02	Os02g0167366	3619823	3622457	√	√	×	Hypothetical protein.
chr02	Os02g0131600	1650800	1651201	√	√	×	Similar to Mitochondrial import receptor subunit TOM22 homolog
chr02	Os02g0131500	1644301	1648677	√	√	×	Hypothetical protein.
chr02	Os02g0131400	1644223	1648808	√	√	×	Beta-D-glucan exohydrolase 2C isoenzyme ExoII
chr02	Os02g0131200	1632049	1636772	√	√	×	Similar to RNA-binding region RNP-1
chr03	Os03g0808400	33796789	33797866	√	√	×	Ubiquitin domain containing protein.
chr03	Os03g0803700	33530088	33534423	√	√	×	Hypothetical conserved gene.
chr04	Os04g0602900	30428193	30431681	√	√	×	Similar to cDNA clone:J023087M06 2C full insert sequence.
chr04	Os04g0602800	30416463	30424148	√	√	×	Similar to Yarrowia lipolytica chromosome D of strain CLIB99 of Yarrowia lipolytica.
chr04	Os04g0602700	30413771	30415745	√	√	×	RNA recognition motif 2C RNP-1 domain containing protein.
chr04	Os04g0602600	30410105	30413160	√	√	×	Pentatricopeptide repeat domain containing protein.
chr04	Os04g0602500	30405474	30409351	√	√	×	Similar to Pectin acetylesterase.
chr04	Os04g0602250	30390323	30390419	√	√	×	Non-protein coding transcript.
chr04	Os04g0602200	30388420	30390045	√	√	×	Bacterial extracellular solute-binding protein 2C family 1 protein.
chr04	Os04g0528300	26395912	26401438	√	√	×	Similar to ABC transporter.
chr05	Os05g0295700	13088568	13092914	√	√	×	Mitochondrial matrix Mmp37 domain containing protein.
chr06	Os06g0181400	4019847	4022584	√	√	×	Similar to oxidoreductase.
chr06	Os06g0181200	4010785	4013536	√	√	×	Serine/threonine protein kinase domain containing protein.
chr06	Os06g0180533	3992690	3993053	√	√	√	Similar to predicted protein.
chr06	Os06g0180100	3972493	3972960	√	√	√	Hypothetical conserved gene.
chr06	Os06g0180050	3967253	3969392	√	√	√	Hypothetical protein.
chr06	Os06g0180000	3966107	3969423	√	√	√	Similar to Root determined nodulation 1.
chr06	Os06g0179800	3956913	3961586	√	√	√	Leucine-rich repeat 2C N-terminal domain containing protein.
chr06	Os06g0179500	3941687	3943447	√	√	√	Protein of unknown function DUF247 2C plant family protein.
chr06	Os06g0179400	3941560	3943507	√	√	√	Hypothetical protein.
chr06	Os06g0179200	3934027	3937229	√	√	√	Similar to Nodulin-like protein.
chr06	Os06g0179100	3932020	3933032	√	√	√	Similar to H0306F03.13 protein.
chr06	Os06g0179050	3926026	3927613	√	√	√	Hypothetical gene.
chr07	Os07g0146700	2389707	2390872	√	√	×	Hypothetical protein.
chr08	Os08g0520500	25870837	25871444	√	√	×	Similar to Isoform 2 of Auxin response factor 21.
chr08	Os08g0520400	25867086	25870277	√	√	×	Conserved hypothetical protein.
chr08	Os08g0520300	25859053	25864844	√	√	×	Similar to Oligouridylate binding protein.
chr08	Os08g0520200	25856980	25858374	√	√	×	Similar to predicted protein.
chr08	Os08g0520000	25848686	25852963	√	√	×	Mitochondrial substrate carrier family protein.
chr08	Os08g0519900	25847203	25847991	√	√	×	Conserved hypothetical protein.
chr08	Os08g0519800	25836766	25844536	√	√	×	Similar to Maternal pumilio protein. Splice isoform E.
chr08	Os08g0519750	25833627	25834331	√	√	×	Hypothetical conserved gene.
chr08	Os08g0519700	25829944	25834691	√	√	×	Protein of unknown function DUF630 domain containing protein.
chr08	Os08g0519600	25817761	25823466	√	√	×	Protein of unknown function DUF1666 family protein.
chr10	Os10g0560400	22069784	22072449	√	√	√	Similar to CONSTANS-like protein CO9
chr10	Os10g0560000	22055014	22056950	√	√	√	Protein of unknown function DUF828 2C plant family protein.
chr10	Os10g0559900	22046536	22048449	√	√	√	Ribosomal protein L18/L5 domain containing protein.
chr10	Os10g0559866	22046461	22047003	√	√	√	Hypothetical protein.
chr10	Os10g0559700	22030634	22036252	√	√	√	Similar to Agenet domain containing protein 2C expressed.
chr10	Os10g0559400	22014949	22019101	√	√	√	Similar to cDNA clone:J023138C05 2C full insert sequence.
chr10	Os10g0559300	22013124	22017335	√	√	√	Hypothetical protein.
chr10	Os10g0559200	22012573	22014287	√	√	√	Similar to Oxidoreductase 2C 2OG-Fe oxygenase family protein 2C expressed.
chr10	Os10g0558725	21989349	21990589	√	√	√	Hypothetical protein.
chr10	Os10g0558700	21988641	21991288	√	√	√	Similar to Oxidoreductase 2C 2OG-Fe oxygenase family protein 2C expressed.
chr10	Os10g0558650	21983981	21984346	√	√	√	Non-protein coding transcript.
chr10	Os10g0558301	21969310	21970499	√	√	√	Hypothetical gene.
chr10	Os10g0558200	21968925	21970501	√	√	√	2OG-Fe
chr10	Os10g0557900	21955492	21957095	√	√	√	Peptidase M10A and M12B 2C matrixin and adamalysin family protein.
chr10	Os10g0557800	21953519	21955536	√	√	√	Conserved hypothetical protein.
chr10	Os10g0557750	21952440	21953021	√	√	√	Hypothetical genes.
chr10	Os10g0557700	21950650	21953040	√	√	√	Conserved hypothetical protein.
chr10	Os10g0557600	21944314	21945183	√	√	√	Similar to GATA transcription factor 9.
chr10	Os10g0557366	21944163	21944875	√	√	√	Conserved hypothetical protein.
chr10	Os10g0556600	21893919	21895681	√	√	√	Similar to predicted protein.
chr12	Os12g0600701	25282668	25284327	√	√	×	Conserved hypothetical protein.
chr12	Os12g0177900	3947161	3947412	√	√	×	Similar to Aldehyde dehydrogenase.
chr12	Os12g0177500	3932583	3935899	√	√	×	Peptidase A1 domain containing protein.

Note: " √ " means that genes are specific to SH527 pedigree; "×" means that genes are not specific to SH527 pedigree

Pedigree-based genome re-sequencing reveals genetic variation patterns of elite backbone varieties during modern rice improvement

Xingfei Zheng, Lanzhi Li, Fan Liang, Changjun Tan, Shuzhu Tang, Sibin Yu, Ying Diao, Shuangcheng Li,and Zhongli Hu

Supplementary Table S16 The distribution of signature SNPs during conventional rice pedigree breeding

Pedigree	Inter-genic	5'UTR	3'UTR	Intron	CDS	No-coding-exon	Total	Genes with all improved SNPs	Genes with improved CDS-SNPs
GC2H	111,458	1,967	3,414	13,875	7,497	318	138,545	3,925	2,176
HHZ	46,134	1,326	1,713	5,937	3,391	214	58,715	2,412	1,236
Common	15,125	287	420	1,536	876	56	18,300	839	474
Total	143,398	3,227	4,619	19,049	10,173	582	181,048	5,489	2,938

Pedigree-based genome re-sequencing reveals genetic variation patterns of elite backbone varieties during modern rice improvement
Xingfei Zheng, Lanzhi Li, Fan Liang, Changjun Tan, Shuzhu Tang, Sibin Yu, Ying Diao, Shuangcheng Li,and Zhongli Hu

Supplementary Table S17 Genes with signature CDS SNPs during GC2H Pedigree breeding

Chromosome	Gene ID	Signature SNPs number	Start positon	End position	Function
chr01	Os01g0101600	9	72816	78349	Immunoglobulin-like fold domain containing protein.
chr01	Os01g0101800	10	85337	88844	Conserved hypothetical protein.
chr01	Os01g0102000	3	89763	91465	Phosphoesterase family protein.
chr01	Os01g0102400	3	139826	141555	Histone-fold domain containing protein.
chr01	Os01g0102500	3	141959	144554	Conserved hypothetical protein.
chr01	Os01g0102600	2	145603	147847	Shikimate kinase domain containing protein.
chr01	Os01g0102800	1	152853	156449	Similar to chromatin remodeling complex subunit.
chr01	Os01g0102850	5	164577	168921	Similar to nitrilase 2.
chr01	Os01g0103100	1	178607	180575	TGF-beta receptor 2C type I/II extracellular region family protein.
chr01	Os01g0103800	2	197647	200803	Conserved hypothetical protein.
chr01	Os01g0106700	1	335809	370652	Hypothetical conserved gene.
chr01	Os01g0108600	13	478384	481165	Basic helix-loop-helix dimerisation region bHLH domain containing protein.
chr01	Os01g0109366	1	508687	509191	Hypothetical conserved gene.
chr01	Os01g0109432	5	509226	509803	Hypothetical gene.
chr01	Os01g0110050	4	536973	538644	Pi homeostasis
chr01	Os01g0110100	13	537710	543354	Phosphate
chr01	Os01g0110200	3	543425	544510	Conserved hypothetical protein.
chr01	Os01g0110500	8	554025	558015	Protein kinase 2C core domain containing protein.
chr01	Os01g0110700	15	562088	572092	Similar to Phosphoenolpyruvate carboxylase
chr01	Os01g0110800	8	576495	579569	Leucine-rich repeat 2C plant specific containing protein.
chr01	Os01g0111000	7	585876	586445	Pectinesterase inhibitor domain containing protein.
chr01	Os01g0111100	5	586445	587696	Cyclophilin-like domain containing protein.
chr01	Os01g0111200	2	588917	593714	Like-Sm ribonucleoprotein
chr01	Os01g0111300	1	597009	597804	Conserved hypothetical protein.
chr01	Os01g0111600	47	612564	615631	Similar to MOTHER of FT and TF1 protein.
chr01	Os01g0112000	2	632139	632790	Conserved hypothetical protein.
chr01	Os01g0112100	80	635325	639015	Conserved hypothetical protein.
chr01	Os01g0112500	30	648288	651869	Similar to Plakoglobin/armadillo/beta-catenin-like protein
chr01	Os01g0113300	3	723693	726099	Similar to ARK protein
chr01	Os01g0113500	1	734071	737556	Similar to Receptor-like kinase.
chr01	Os01g0113600	5	738283	741797	Hypothetical protein.
chr01	Os01g0113700	9	742860	745177	Similar to Receptor-like kinase.
chr01	Os01g0113800	60	745452	748945	Protein kinase 2C core domain containing protein.
chr01	Os01g0114700	5	789714	792435	Similar to Receptor-like kinase.
chr01	Os01g0114900	1	794499	795413	Similar to LRK14.
chr01	Os01g0115600	8	843530	847399	Similar to LRK14.
chr01	Os01g0115750	8	857897	860583	Similar to Receptor-like protein kinase.
chr01	Os01g0115900	41	865938	869298	Similar to Receptor-like protein kinase.
chr01	Os01g0116000	2	872458	874817	Similar to Receptor-like protein kinase.
chr01	Os01g0116200	3	911735	914246	Protein kinase 2C core domain containing protein.
chr01	Os01g0116300	17	915605	917636	Similar to Receptor-like kinase.
chr01	Os01g0116400	1	921078	923630	Protein kinase 2C core domain containing protein.
chr01	Os01g0116600	2	929884	934458	Protein synthesis factor 2C GTP-binding domain containing protein.
chr01	Os01g0116800	4	935574	936704	Similar to Receptor-like kinase.
chr01	Os01g0117200	1	969340	973327	Similar to ARK protein
chr01	Os01g0117600	2	994712	997262	Protein kinase 2C catalytic domain domain containing protein.
chr01	Os01g0117700	1	997918	1000997	Similar to LRK14.
chr01	Os01g0118600	1	1066678	1069673	Similar to Hga5 protein.
chr01	Os01g0118700	1	1071962	1074829	Similar to HGA4.
chr01	Os01g0119000	4	1078565	1081597	Glycosyltransferase AER61 2C uncharacterized domain containing protein.
chr01	Os01g0119500	2	1103782	1106890	Protein of unknown function DUF1618 domain containing protein.
chr01	Os01g0120500	1	1160198	1164619	Similar to PAP fibrillin domain containing protein 2Cexpressed.
chr01	Os01g0120600	12	1165737	1168396	Similar to Oxidoreductase NAD-binding domain containing protein 2C expressed.
chr01	Os01g0120700	13	1168737	1171126	Similar to sarcoplasmic reticulum histidine-rich calcium-binding protein.
chr01	Os01g0120800	33	1171902	1179370	Similar to Eukaryotic translation initiation factor 3 subunit 10
chr01	Os01g0121000	5	1181317	1182147	Conserved hypothetical protein.
chr01	Os01g0121100	2	1183466	1186699	Similar to SLL2.
chr01	Os01g0125550	8	1401419	1403419	Conserved hypothetical protein.
chr01	Os01g0125600	6	1404816	1406889	Heavy metal transport/detoxification protein domain containing protein.
chr01	Os01g0125800	18	1413839	1418982	Actin-interacting 2C WD40-repeat protein 2C Actin turnover
chr01	Os01g0128000	2	1549241	1551011	Myb transcription factor domain containing protein.
chr01	Os01g0129500	3	1641272	1644151	Protein of unknown function DUF1666 family protein.
chr01	Os01g0129600	3	1657306	1658388	Similar to LBD40
chr01	Os01g0144000	3	2378825	2382584	Conserved hypothetical protein.
chr01	Os01g0144300	3	2388605	2390483	Conserved hypothetical protein.
chr01	Os01g0144500	7	2400945	2402336	Ubiquitin fusion degradation protein UFD1 domain containing protein.
chr01	Os01g0144600	114	2403276	2405947	Conserved hypothetical protein.
chr01	Os01g0144700	62	2406475	2408051	Conserved hypothetical protein.
chr01	Os01g0145000	13	2422347	2422682	Hypothetical conserved gene.
chr01	Os01g0147200	15	2560897	2565321	IWS1 2C C-terminal family protein.
chr01	Os01g0147400	42	2570072	2571010	Uncharacterised domain XH domain containing protein.
chr01	Os01g0147600	56	2573049	2577415	Conserved hypothetical protein.
chr01	Os01g0147700	6	2579565	2583575	Region of unknown function XH domain containing protein.
chr01	Os01g0147900	4	2589835	2593568	Triosephosphate isomerase 2C cytosolic
chr01	Os01g0148000	4	2599732	2603593	Similar to predicted protein.
chr01	Os01g0148050	10	2605614	2612731	Hypothetical conserved gene.
chr01	Os01g0148100	13	2613672	2623190	Conserved hypothetical protein.
chr01	Os01g0148700	3	2652648	2655564	Protein of unknown function DUF1677 2C Oryza sativa family protein.
chr01	Os01g0149350	1	2669251	2674068	NB-ARC domain containing protein.
chr01	Os01g0149400	2	2674414	2674683	Hypothetical protein.
chr01	Os01g0149900	10	2699128	2703491	Conserved hypothetical protein.
chr01	Os01g0150000	6	2707624	2711422	Similar to Synaptic glycoprotein SC2.
chr01	Os01g0150500	7	2727751	2736707	Similar to 3-hydroxyacyl-CoA dehydratase PASTICCINO 2A.
chr01	Os01g0150900	4	2752144	2761554	Conserved hypothetical protein.
chr01	Os01g0151100	6	2765779	2768602	HSP20-like chaperone domain containing protein.
chr01	Os01g0151500	18	2783189	2790377	Similar to GGT4
chr01	Os01g0152600	3	2830325	2833623	Protein kinase 2C catalytic domain domain containing protein.
chr01	Os01g0152951	8	2849160	2851689	Hypothetical conserved gene.
chr01	Os01g0165200	3	3362001	3367658	Kelch-type beta propeller domain containing protein.
chr01	Os01g0168400	4	3501094	3504775	Zinc finger 2C RING/FYVE/PHD-type domain containing protein.
chr01	Os01g0168500	17	3505759	3510343	WD40 repeat-like domain containing protein.
chr01	Os01g0169000	2	3520944	3521309	Hypothetical protein.
chr01	Os01g0169400	3	3551051	3553146	Similar to LOB domain protein 25.
chr01	Os01g0171200	7	3681798	3687409	Conserved hypothetical protein.

chr01	Os01g0171800	10	3696155	3699718	Conserved hypothetical protein.
chr01	Os01g0173600	1	3779634	3782002	Glyoxalase/bleomycin resistance protein/dioxygenase domain containing protein.
chr01	Os01g0174300	3	3820177	3824512	Flavoprotein pyridine nucleotide cytochrome reductase domain containing protein.
chr01	Os01g0174400	9	3825669	3829404	Hypothetical gene.
chr01	Os01g0174500	1	3827045	3830326	Metridin-like ShK toxin domain containing protein.
chr01	Os01g0179800	8	4167514	4173240	TMS membrane protein/tumour differentially expressed protein family protein.
chr01	Os01g0185900	3	4566304	4568589	Similar to WRKY 1
chr01	Os01g0193400	2	5016279	5020150	TNFR/CD27/30/40/95 cysteine-rich region domain containing protein.
chr01	Os01g0193900	9	5030157	5038482	Homeodomain-like domain containing protein.
chr01	Os01g0195000	5	5099555	5102080	Zinc finger 2C C2H2-type domain containing protein.
chr01	Os01g0195200	2	5109949	5112457	Similar to Serine/threonine-protein kinase PBS1
chr01	Os01g0196300	2	5201862	5203996	Helix-loop-helix DNA-binding domain containing protein.
chr01	Os01g0197350	1	5256634	5257053	Hypothetical genes.
chr01	Os01g0197400	1	5258298	5260964	Conserved hypothetical protein.
chr01	Os01g0197450	4	5261189	5263102	Pentatricopeptide repeat domain containing protein.
chr01	Os01g0199700	10	5398321	5402018	Protein of unknown function DUF1677 2C plant domain containing protein.
chr01	Os01g0200200	2	5424059	5424581	Protein of unknown function DUF1677 2C plant domain containing protein.
chr01	Os01g0200300	8	5426582	5429390	Similar to Homeobox-leucine zipper protein HOX29.
chr01	Os01g0200500	9	5442866	5446246	MPPN family protein.
chr01	Os01g0201100	1	5507567	5509494	Similar to xylosyltransferase oxt.
chr01	Os01g0201200	3	5510522	5514454	Similar to Protein kinase.
chr01	Os01g0201250	2	5529876	5531014	Similar to DNA-binding WRKY.
chr01	Os01g0201600	7	5549651	5551003	Virulence factor 2C pectin lyase fold family protein.
chr01	Os01g0202800	4	5663306	5665832	Similar to NOD26-like membrane integral protein ZmNIP1-1.
chr01	Os01g0203800	13	5699652	5707756	Protein of unknown function DUF641 2C plant domain containing protein.
chr01	Os01g0205200	1	5764652	5766659	Pentatricopeptide repeat domain containing protein.
chr01	Os01g0205600	1	5773300	5774176	Crotonase 2C core domain containing protein.
chr01	Os01g0208000	1	5862946	5863577	Protein of unknown function DUF6 2C transmembrane domain containing protein.
chr01	Os01g0209000	5	5911506	5916779	Alg9-like mannosyltransferase family protein.
chr01	Os01g0209500	18	5945633	5954315	Zinc finger 2C RING-type domain containing protein.
chr01	Os01g0209700	13	5968819	5972489	Similar to GA 2-oxidase 5.
chr01	Os01g0210400	10	6005040	6007163	Protein of unknown function DUF616 domain containing protein.
chr01	Os01g0210600	6	6010862	6011818	Protein of unknown function DUF538 family protein.
chr01	Os01g0212100	3	6112811	6132696	DEAD-like helicase 2C N-terminal domain containing protein.
chr01	Os01g0216400	1	6361526	6363457	Lipase 2C GDSL domain containing protein.
chr01	Os01g0216500	4	6365063	6367057	Lipase 2C GDSL domain containing protein.
chr01	Os01g0216900	19	6374207	6381837	Lipase 2C GDSL domain containing protein.
chr01	Os01g0217000	15	6387691	6391467	Protein of unknown function DUF231 2C plant domain containing protein.
chr01	Os01g0217050	1	6388780	6390920	Hypothetical protein.
chr01	Os01g0217100	3	6394270	6395665	Conserved hypothetical protein.
chr01	Os01g0217500	23	6412786	6419867	ThiJ/PfpI domain containing protein.
chr01	Os01g0217800	8	6432336	6435651	ThiJ/PfpI domain containing protein.
chr01	Os01g0220400	1	6587146	6587940	Hypothetical protein.
chr01	Os01g0221300	6	6642665	6645159	Conserved hypothetical protein.
chr01	Os01g0221500	3	6653148	6655290	Protein of unknown function DUF2358 domain containing protein.
chr01	Os01g0221600	2	6659237	6662320	Malate transporter 2C aliminium toerance domain containing protein.
chr01	Os01g0221700	6	6663533	6666725	Similar to GDT1-like protein 1 2C chloroplastic.
chr01	Os01g0221900	9	6667724	6670479	Similar to peptidase C45 2C acyl-coenzyme A/6-aminopenicillanic acid acyl-transferase.
chr01	Os01g0222300	1	6694030	6696795	Conserved hypothetical protein.
chr01	Os01g0222700	13	6705859	6710552	Protein of unknown function DUF659 domain containing protein.
chr01	Os01g0222800	22	6713909	6714986	Similar to OSIGBa0145C12.7 protein.
chr01	Os01g0222900	32	6715521	6716305	Curculin-like
chr01	Os01g0223000	23	6718349	6719944	Lipase 2C GDSL domain containing protein.
chr01	Os01g0223200	16	6724946	6728538	Lipase 2C GDSL domain containing protein.
chr01	Os01g0223600	4	6766511	6769519	Similar to Pto kinase interactor 1-like protein.
chr01	Os01g0223700	5	6774202	6776856	Bulb-type lectin domain domain containing protein.
chr01	Os01g0223800	3	6781919	6786415	Hypothetical conserved gene.
chr01	Os01g0223900	1	6787094	6789730	Serine/threonine protein kinase-related domain containing protein.
chr01	Os01g0227100	25	7024297	7030591	Chlorophyll b reductase 2C Leaf senescence
chr01	Os01g0227200	5	7038103	7042925	Similar to Somatic embryogenesis receptor kinase-like protein.
chr01	Os01g0227350	4	7048457	7050422	Hypothetical protein.
chr01	Os01g0227800	2	7071481	7073788	Similar to cDNA 2C clone: J075083L18 2C full insert sequence.
chr01	Os01g0228300	8	7085873	7089172	Mpv17/PMP22 family protein.
chr01	Os01g0228400	13	7092034	7095315	Pentatricopeptide repeat domain containing protein.
chr01	Os01g0228500	3	7095864	7097381	Harpin-induced 1 domain containing protein.
chr01	Os01g0228600	19	7100070	7103266	Similar to 2-hydroxyacid dehydrogenase
chr01	Os01g0228800	47	7104701	7109095	Conserved hypothetical protein.
chr01	Os01g0269000	8	9268690	9272912	Similar to Hydroxymethylglutaryl-CoA lyase.
chr01	Os01g0301500	35	11078206	11083482	Pentatricopeptide repeat domain containing protein.
chr01	Os01g0310100	13	11625271	11637356	Similar to PLDP1
chr01	Os01g0311100	1	11686442	11687191	Similar to RING-H2 finger protein ATL2L.
chr01	Os01g0311300	2	11688361	11689046	Similar to Sorbitol transporter.
chr01	Os01g0311400	1	11690379	11690975	Zinc finger 2C RING/FYVE/PHD-type domain containing protein.
chr01	Os01g0311600	4	11703247	11704653	Sulfotransferase family protein.
chr01	Os01g0311700	2	11713650	11714674	Pectinesterase inhibitor domain containing protein.
chr01	Os01g0313300	4	11783910	11784899	Similar to EREBP-3 protein
chr01	Os01g0314800	1	11863682	11864412	Late embryogenesis abundant protein 3 family protein.
chr01	Os01g0318000	1	12074526	12075088	Similar to esterase/lipase/thioesterase family protein.
chr01	Os01g0338150	11	13291338	13294921	Hypothetical gene.
chr01	Os01g0338100	4	13291658	13294473	Similar to Transcription initiation factor IID 2C 18kD subunit family protein.
chr01	Os01g0352100	1	14101994	14108083	2OG-Fe
chr01	Os01g0353400	19	14167662	14168570	Similar to Glutathione S-transferase GST 8
chr01	Os01g0354700	57	14252929	14256623	Pre-mRNA cleavage complex II Clp1 domain containing protein.
chr01	Os01g0357200	2	14453690	14454549	Sterol-binding domain containing protein.
chr01	Os01g0357500	5	14462311	14462787	Similar to Ferredoxin-nitrite reductase.
chr01	Os01g0357800	15	14471243	14476738	Pentatricopeptide repeat domain containing protein.
chr01	Os01g0361000	5	14702436	14705998	Conserved hypothetical protein.
chr01	Os01g0362400	1	14769271	14769881	Conserved hypothetical protein.
chr01	Os01g0364100	11	14825133	14828267	Similar to OSIGBa0145M07.8 protein.
chr01	Os01g0364450	1	14879651	14880271	Hypothetical gene.
chr01	Os01g0364800	29	14892931	14898978	Protein kinase 2C catalytic domain domain containing protein.
chr01	Os01g0365300	8	14924924	14927978	Hypothetical conserved gene.
chr01	Os01g0367300	2	15007028	15013275	Nucleotide-binding 2C alpha-beta plait domain containing protein.
chr01	Os01g0367900	4	15075863	15083305	Similar to Possible global transcription activator SNF2L1
chr01	Os01g0368600	3	15113035	15113731	Conserved hypothetical protein.
chr01	Os01g0371400	2	15289214	15291315	Similar to Glutathione s-transferase gstf2.
chr01	Os01g0371500	9	15291669	15294409	Similar to Glutathione-S-transferase 19E50.
chr01	Os01g0374200	8	15418911	15421118	Pentatricopeptide repeat domain containing protein.
chr01	Os01g0374501	1	15440625	15441473	Similar to mTERF family protein.
chr01	Os01g0382000	2	15934495	15935317	Similar to Pathogenesis-related protein PRB1-2 precursor.

chr01	Os01g0389200	2	16415043	16416009	Protein of unknown function DUF679 family protein.
chr01	Os01g0500500	7	17266185	17274780	Hypothetical conserved gene.
chr01	Os01g0508000	4	17756115	17764819	Similar to Beta-glucosidase.
chr01	Os01g0510800	9	17960990	17968544	Histone-fold domain containing protein.
chr01	Os01g0510901	16	17967782	17973329	Conserved hypothetical protein.
chr01	Os01g0511000	1	17975866	17977327	Similar to LOB domain protein 40.
chr01	Os01g0512800	1	18077464	18078185	CS-like domain domain containing protein.
chr01	Os01g0518651	1	18405728	18408073	Leucine-rich repeat domain containing protein.
chr01	Os01g0520600	28	18531902	18540738	NB-ARC domain containing protein.
chr01	Os01g0521500	11	18601026	18611536	Heat shock protein DnaJ family protein.
chr01	Os01g0522100	4	18626121	18627135	Conserved hypothetical protein.
chr01	Os01g0522400	3	18639233	18641762	Armadillo-like helical domain containing protein.
chr01	Os01g0523000	36	18668266	18669004	Hypothetical gene.
chr01	Os01g0523100	1	18671229	18674473	Protein kinase 2C catalytic domain domain containing protein.
chr01	Os01g0524251	13	18720539	18721863	Conserved hypothetical protein.
chr01	Os01g0524400	7	18749285	18750480	Conserved hypothetical protein.
chr01	Os01g0524700	34	18763270	18774476	Armadillo-like helical domain containing protein.
chr01	Os01g0525100	2	18780600	18781392	Conserved hypothetical protein.
chr01	Os01g0526100	1	18847970	18851815	Similar to splicing factor 45.
chr01	Os01g0527600	5	18940389	18943033	RNA-dependent RNA polymerase 2C eukaryotic-type domain containing protein.
chr01	Os01g0530500	3	19095631	19097491	Protein of unknown function DUF295 domain containing protein.
chr01	Os01g0531200	3	19115283	19122409	Phospholipase C 2C phosphatidylinositol-specific 2C Y domain domain containing protein.
chr01	Os01g0531400	3	19129984	19131278	Hypothetical conserved gene.
chr01	Os01g0531500	3	19135843	19139893	Dienelactone hydrolase domain containing protein.
chr01	Os01g0533800	13	19317853	19325937	Conserved hypothetical protein.
chr01	Os01g0535100	1	19434294	19435079	Zinc finger 2C RING-type domain containing protein.
chr01	Os01g0536400	14	19505311	19508410	Isopenicillin N synthase family protein.
chr01	Os01g0536466	26	19509834	19510988	Conserved hypothetical protein.
chr01	Os01g0536501	5	19516756	19518873	Disease resistance protein domain containing protein.
chr01	Os01g0536950	16	19539370	19540140	Similar to Rust resistance protein rp3-1.
chr01	Os01g0537250	13	19549155	19550345	Protein of unknown function DUF3778 domain containing protein.
chr01	Os01g0541600	10	19911224	19914280	RNAse P 2C Rpr2/Rpp21 subunit domain containing protein.
chr01	Os01g0542100	4	19973271	19977472	Conserved hypothetical protein.
chr01	Os01g0542700	1	20034162	20035006	bZIP transcription factor 2C bZIP-1 domain containing protein.
chr01	Os01g0579600	8	22427835	22430779	Similar to Calcineurin B-like protein 9.
chr01	Os01g0603700	4	23776275	23780692	Hypothetical gene.
chr01	Os01g0604500	2	23810484	23811557	EF-Hand type domain containing protein.
chr01	Os01g0605700	54	23864681	23867205	MtN3 and saliva related transmembrane protein family protein.
chr01	Os01g0606000	30	23877540	23880467	MtN3 and saliva related transmembrane protein family protein.
chr01	Os01g0606133	3	23886851	23888222	Hypothetical conserved gene.
chr01	Os01g0607000	1	23914150	23914856	Glutelin family protein.
chr01	Os01g0607100	4	23919214	23921302	Bifunctional inhibitor/plant lipid transfer protein/seed storage domain containing protein.
chr01	Os01g0607400	24	23938395	23949885	Similar to STYLOSA protein.
chr01	Os01g0607600	8	23947829	23962251	WD40 repeat domain containing protein.
chr01	Os01g0607900	17	23976021	23981901	Serine/threonine protein kinase-related domain containing protein.
chr01	Os01g0608200	1	24018683	24019797	Hypothetical protein.
chr01	Os01g0608300	1	24018634	24020111	Similar to aspartic proteinase nepenthesin-2.
chr01	Os01g0609200	21	24065207	24073621	Similar to Pleiotropic drug resistance protein 3.
chr01	Os01g0609700	2	24102742	24104421	Conserved hypothetical protein.
chr01	Os01g0610400	3	24145891	24148846	Acyl-CoA N-acyltransferase domain containing protein.
chr01	Os01g0610500	9	24151040	24154089	Yip1 domain containing protein.
chr01	Os01g0610600	4	24155649	24162572	Endonuclease/exonuclease/phosphatase domain containing protein.
chr01	Os01g0621300	3	24763107	24768254	Hypothetical conserved gene.
chr01	Os01g0621500	3	24774161	24774574	Conserved hypothetical protein.
chr01	Os01g0621600	8	24784609	24788153	Serine/threonine protein kinase-related domain containing protein.
chr01	Os01g0621900	5	24808516	24813386	Similar to OSIGBa0097P08.1 protein.
chr01	Os01g0622300	2	24823998	24828139	Similar to Plastid uroporphyrinogen decarboxylase
chr01	Os01g0622800	4	24850443	24851215	Hypothetical protein.
chr01	Os01g0623000	2	24855937	24857421	Hypothetical conserved gene.
chr01	Os01g0623200	7	24861697	24864291	Similar to C4-dicarboxylate transporter/malic acid transport protein.
chr01	Os01g0626400	4	25009514	25012233	DNA-binding protein WRKY2-like
chr01	Os01g0630101	2	25187267	25189478	Hypothetical protein.
chr01	Os01g0631400	4	25257001	25263141	Six-hairpin glycosidase domain containing protein.
chr01	Os01g0632000	5	25300987	25302926	Conserved hypothetical protein.
chr01	Os01g0634500	3	25439082	25440898	Similar to Laccase-2.
chr01	Os01g0677500	1	27860530	27862131	Similar to ubiquitin-conjugating enzyme E2.
chr01	Os01g0689900	11	28482950	28496176	Hypothetical conserved gene.
chr01	Os01g0691200	3	28538115	28539701	Hypothetical protein.
chr01	Os01g0692200	1	28572037	28572921	Conserved hypothetical protein.
chr01	Os01g0738500	1	30828776	30832255	Phospholipase C 2C phosphatidylinositol-specific 2C Y domain domain containing protein.
chr01	Os01g0739400	11	30879000	30885267	Similar to predicted protein.
chr01	Os01g0739500	2	30885172	30886338	Conserved hypothetical protein.
chr01	Os01g0740000	1	30905585	30906259	Hypothetical protein.
chr01	Os01g0755100	5	31678312	31689878	Conserved hypothetical protein.
chr01	Os01g0770100	2	32484736	32489971	O-acyltransferase 2C WSD1 2C N-terminal domain containing protein.
chr01	Os01g0775100	3	32759157	32764753	Plus-3 domain containing protein.
chr01	Os01g0775300	8	32773565	32783571	MATH domain containing protein.
chr01	Os01g0775500	2	32812249	32814794	Mannose-binding lectin domain containing protein.
chr01	Os01g0776800	1	32860954	32863510	Domain of unknown function DUF1618 domain containing protein.
chr01	Os01g0777800	1	32929073	32932792	Hypothetical conserved gene.
chr01	Os01g0778100	1	32940137	32942493	Hypothetical conserved gene.
chr01	Os01g0778400	3	32946648	32946942	Hypothetical protein.
chr01	Os01g0779100	16	32985048	32994371	Peptidase M16 2C zinc-binding site domain containing protein.
chr01	Os01g0779300	2	32996762	32999176	Concanavalin A-like lectin/glucanase 2C subgroup domain containing protein.
chr01	Os01g0779400	27	33000078	33010221	Zinc finger 2C RING/FYVE/PHD-type domain containing protein.
chr01	Os01g0780400	6	33050684	33058490	Hypothetical gene.
chr01	Os01g0780600	1	33064726	33067834	Similar to Transposon protein Mutator sub-class.
chr01	Os01g0781200	6	33098082	33103904	Similar to Rust resistance protein.
chr01	Os01g0784600	7	33244777	33248099	Similar to nodulation protein-related.
chr01	Os01g0784900	2	33272134	33274419	Conserved hypothetical protein.
chr01	Os01g0785300	4	33302721	33303613	Hypothetical protein.
chr01	Os01g0785400	1	33308448	33310933	GH3 auxin-responsive promoter family protein.
chr01	Os01g0787400	2	33392410	33395692	Conserved hypothetical protein.
chr01	Os01g0787600	24	33409655	33415562	Similar to Salicylic acid-binding protein 2.
chr01	Os01g0788001	1	33448517	33449043	Hypothetical gene.
chr01	Os01g0789100	4	33489285	33492959	CDP-alcohol phosphatidyltransferase family protein.
chr01	Os01g0794100	3	33629559	33630287	Conserved hypothetical protein.
chr01	Os01g0795000	2	33668016	33672415	Subtilase.
chr01	Os01g0795100	5	33675136	33679717	Similar to Subtilase.
chr01	Os01g0795832	2	33708780	33709258	Conserved hypothetical protein.

chr01	Os01g0796000	1	33713552	33714121	Conserved hypothetical protein.
chr01	Os01g0798500	3	33803155	33805386	Similar to predicted protein.
chr01	Os01g0799100	5	33821312	33828770	NB-ARC domain containing protein.
chr01	Os01g0799500	1	33837388	33840967	Methyladenine glycosylase domain containing protein.
chr01	Os01g0800266	1	33884788	33887413	Similar to Phosphoglycerate kinase.
chr01	Os01g0800250	2	33884974	33887391	Hypothetical gene.
chr01	Os01g0800300	2	33890098	33891727	Immunoglobulin/major histocompatibility complex 2C conserved site domain containing protein.
chr01	Os01g0803200	3	34033159	34034924	Cysteine proteinase inhibitor-I
chr01	Os01g0804900	3	34131886	34133448	Similar to Cytochrome P450.
chr01	Os01g0805200	1	34138399	34139709	Protein of unknown function DUF3007 domain containing protein.
chr01	Os01g0805300	5	34139768	34141211	Mog1/PsbP 2C alpha/beta/alpha sandwich domain containing protein.
chr01	Os01g0805400	16	34141778	34145201	UDP-glucuronosyl/UDP-glucosyltransferase family protein.
chr01	Os01g0805500	14	34146163	34149144	Similar to indole-3-acetate beta-glucosyltransferase.
chr01	Os01g0805600	1	34149458	34151206	Similar to Cyclin-B1-1.
chr01	Os01g0806600	5	34217036	34218756	Conserved hypothetical protein.
chr01	Os01g0810600	3	34438388	34444340	Similar to cDNA 2C clone: J100063K14 2C full insert sequence.
chr01	Os01g0810800	36	34445750	34454439	Hypothetical conserved gene.
chr01	Os01g0813800	10	34605594	34611055	Similar to Beta-glucosidase 3.
chr01	Os01g0816400	1	34705486	34722561	Similar to microtubule organization protein.
chr01	Os01g0816500	5	34731151	34734804	Similar to microtubule organization protein.
chr01	Os01g0817100	8	34776154	34780863	TRAM 2C LAG1 and CLN8 homology domain containing protein.
chr01	Os01g0817400	2	34788192	34791588	Similar to proton antiporter/ sodium:hydrogen antiporter.
chr01	Os01g0823400	2	35156414	35159205	Hypothetical conserved gene.
chr01	Os01g0831200	2	35582280	35585575	Ubiquitin domain containing protein.
chr01	Os01g0849950	4	36527939	36529713	Hypothetical protein.
chr01	Os01g0850800	1	36634331	36636717	Laccase-8.
chr01	Os01g0854500	1	36813736	36815023	Homeobox domain containing protein.
chr01	Os01g0871400	7	37789052	37790984	Hypothetical protein.
chr01	Os01g0871500	5	37790484	37792882	TGF-beta receptor 2C type I/II extracellular region family protein.
chr01	Os01g0871700	5	37800956	37802110	Oligopeptide transporter domain containing protein.
chr01	Os01g0871900	1	37810770	37812129	Similar to POT family protein.
chr01	Os01g0872000	2	37814673	37816775	TGF-beta receptor 2C type I/II extracellular region family protein.
chr01	Os01g0872100	18	37821284	37826818	TGF-beta receptor 2C type I/II extracellular region family protein.
chr01	Os01g0872300	5	37828417	37829987	Similar to POT family protein.
chr01	Os01g0872350	5	37829568	37832278	Hypothetical protein.
chr01	Os01g0872500	3	37834199	37836889	Oligopeptide transporter domain containing protein.
chr01	Os01g0872700	3	37844994	37852790	Zinc finger 2C CCHC-type domain containing protein.
chr01	Os01g0872800	3	37853413	37857509	Similar to 3-phosphoinositide-dependent protein kinase-1
chr01	Os01g0873200	1	37866333	37868311	Similar to Amidophosphoribosyltransferase 2C chloroplast precursor
chr01	Os01g0874800	3	37955091	37959001	Similar to DNA polymerase I.
chr01	Os01g0880100	2	38191821	38192525	Conserved hypothetical protein.
chr01	Os01g0880800	1	38224446	38225878	Similar to Acyl-[acyl-carrier-protein] desaturase 2C chloroplast precursor
chr01	Os01g0880900	2	38226425	38228440	Similar to Pentatricopeptide repeat protein PPR868-14.
chr01	Os01g0881000	9	38228812	38237228	SNF2-related domain containing protein.
chr01	Os01g0881100	1	38238479	38240305	Epsin-like 2C N-terminal domain containing protein.
chr01	Os01g0881400	5	38257939	38261435	DNA repair metallo-beta-lactamase domain containing protein.
chr01	Os01g0881500	3	38261856	38265489	Similar to Scarecrow-like 1
chr01	Os01g0881800	1	38273297	38274800	Conserved hypothetical protein.
chr01	Os01g0882100	1	38290773	38290970	Hypothetical conserved gene.
chr01	Os01g0883100	1	38321342	38323892	Similar to PISTILLATA-like MADS box protein.
chr01	Os01g0888700	1	38631901	38635571	RIO-like kinase domain containing protein.
chr01	Os01g0891700	13	38770126	38771794	Leucine-rich repeat 2C N-terminal domain containing protein.
chr01	Os01g0898300	1	39052287	39058529	Armadillo-type fold domain containing protein.
chr01	Os01g0899100	1	39101061	39101498	Hypothetical conserved gene.
chr01	Os01g0899200	1	39102277	39103501	Similar to dehydration-responsive family protein.
chr01	Os01g0899275	1	39110685	39110934	Similar to ATP synthase F0 subunit 9.
chr01	Os01g0901600	3	39257838	39261362	Similar to 4-coumarate--CoA ligase-like 6.
chr01	Os01g0901900	6	39270573	39274992	S1 2C RNA binding domain containing protein.
chr01	Os01g0902100	1	39276426	39281147	Similar to MRP-like ABC transporter.
chr01	Os01g0902200	9	39289364	39296595	Peptidase M3A and M3B 2C thimet/oligopeptidase F domain containing protein.
chr01	Os01g0905200	2	39408313	39410361	Exo70 exocyst complex subunit family protein.
chr01	Os01g0905300	1	39411052	39413189	Similar to Leucine zipper protein-like.
chr01	Os01g0907400	2	39493173	39499938	Histone lysine demethylase 2C Stress-responsive H3K27me3
chr01	Os01g0907900	6	39523638	39527194	Similar to Terminal ear1.
chr01	Os01g0913100	1	39801475	39802517	Protein of unknown function DUF538 family protein.
chr01	Os01g0914600	1	39849645	39851426	Pentatricopeptide repeat domain containing protein.
chr01	Os01g0917500	1	40021069	40023811	Hypothetical conserved gene.
chr01	Os01g0937100	24	41142013	41143416	Similar to Xylanase inhibitor precursor
chr01	Os01g0937300	1	41149956	41154666	NB-ARC domain containing protein.
chr01	Os01g0937400	40	41155827	41163821	NB-ARC domain containing protein.
chr02	Os02g0106600	1	385998	386946	Zinc finger 2C RING/FYVE/PHD-type domain containing protein.
chr02	Os02g0106700	6	391819	395616	Peptidase C78 2C ubiquitin fold modifier-specific peptidase 1/ 2 domain containing protein.
chr02	Os02g0106900	10	404250	406672	Protein kinase 2C core domain containing protein.
chr02	Os02g0106966	3	410645	411491	Similar to emb1507
chr02	Os02g0107000	2	416973	418387	Sec63 domain domain containing protein.
chr02	Os02g0110101	1	554511	556079	Hypothetical gene.
chr02	Os02g0111600	4	627685	630534	Serine/threonine protein kinase-related domain containing protein.
chr02	Os02g0111800	2	637013	640452	Protein kinase 2C core domain containing protein.
chr02	Os02g0144200	1	2444421	2445497	Protein of unknown function DUF1677 2C Oryza sativa family protein.
chr02	Os02g0158500	2	3187973	3190043	Uncharacterized plant-specific domain 01627 containing protein.
chr02	Os02g0158701	1	3196192	3196414	Similar to predicted protein.
chr02	Os02g0182100	1	4573831	4578257	B-type response regulator 2C Cytokinin signaling
chr02	Os02g0182300	3	4594375	4595386	Hypothetical conserved gene.
chr02	Os02g0183550	1	4650762	4653969	Hypothetical protein.
chr02	Os02g0186200	9	4796037	4797937	Similar to Cytochrome P450 CYP71K14.
chr02	Os02g0186800	1	4824246	4825855	Cytochrome P450 family protein.
chr02	Os02g0186900	3	4826405	4828206	Cytochrome P450 family protein.
chr02	Os02g0187400	14	4847753	4852502	PLC-like phosphodiesterase 2C TIM beta/alpha-barrel domain domain containing protein.
chr02	Os02g0202600	1	5744792	5748139	Tetratricopeptide-like helical domain containing protein.
chr02	Os02g0202800	1	5750577	5753676	FAR1 domain containing protein.
chr02	Os02g0204400	1	5835504	5838694	Similar to
chr02	Os02g0205300	9	5918931	5923654	Similar to TAT-binding protein homolog
chr02	Os02g0205500	10	5930298	5933077	Similar to Fatty acid elongase 1.
chr02	Os02g0208100	2	6057100	6060267	Similar to plastidic ATP/ADP-transporter.
chr02	Os02g0211800	3	6247083	6250359	Hypothetical conserved gene.
chr02	Os02g0214500	1	6404019	6405861	No apical meristem
chr02	Os02g0215000	8	6433242	6441693	Conserved hypothetical protein.
chr02	Os02g0215050	2	6442988	6443886	Hypothetical gene.
chr02	Os02g0215700	14	6476685	6480097	Protein kinase 2C catalytic domain domain containing protein.
chr02	Os02g0217400	5	6559488	6562188	Cytochrome P450 family protein.

chr02	Os02g0218600	2	6621779	6623047	Similar to Protein kinase.
chr02	Os02g0225900	14	7062837	7064444	Conserved hypothetical protein.
chr02	Os02g0226000	8	7066391	7069494	Similar to Peroxisomal membrane protein PMP22
chr02	Os02g0234300	4	7613261	7615346	Armadillo-like helical domain containing protein.
chr02	Os02g0234450	3	7618738	7621248	Hypothetical conserved gene.
chr02	Os02g0234500	8	7621905	7627260	NAD
chr02	Os02g0234700	6	7627395	7628246	Conserved hypothetical protein.
chr02	Os02g0245000	1	8197017	8199726	Ubiquitin domain containing protein.
chr02	Os02g0248440	2	8397656	8398297	Zinc finger 2C RING-type domain containing protein.
chr02	Os02g0254600	1	8714222	8715050	Conserved hypothetical protein.
chr02	Os02g0255066	1	8760061	8760449	Hypothetical protein.
chr02	Os02g0256100	27	8834369	8837492	Pectin lyase fold/virulence factor domain containing protein.
chr02	Os02g0257001	147	8856566	8860576	Hypothetical conserved gene.
chr02	Os02g0257100	15	8866815	8867667	Cupredoxin domain containing protein.
chr02	Os02g0258300	6	8938133	8943482	Zinc finger 2C FYVE/PHD-type domain containing protein.
chr02	Os02g0261000	4	9106619	9107664	Conserved hypothetical protein.
chr02	Os02g0261100	9	9116165	9119456	Ubiquitin-conjugating enzyme OsUBC5b.
chr02	Os02g0265100	2	9446493	9448541	Hypothetical protein.
chr02	Os02g0265200	1	9446779	9448835	WRKY transcription factor 39.
chr02	Os02g0314925	1	12491942	12493673	Similar to Integral membrane protein.
chr02	Os02g0461300	1	15452946	15453705	Conserved hypothetical protein.
chr02	Os02g0478450	1	16425253	16425567	Conserved hypothetical protein.
chr02	Os02g0532800	4	19614070	19615072	7TM GPCR 2C rhodopsin-like domain containing protein.
chr02	Os02g0532900	20	19619243	19623214	Glycoside hydrolase 2C family 17 protein.
chr02	Os02g0533000	10	19623488	19628801	FeS cluster biogenesis domain containing protein.
chr02	Os02g0541150	1	20109198	20110897	Hypothetical protein.
chr02	Os02g0542100	10	20134804	20135382	Similar to Histone H3
chr02	Os02g0542500	22	20152453	20154121	Similar to NF-180.
chr02	Os02g0543100	12	20169587	20172428	Similar to predicted protein.
chr02	Os02g0543900	27	20227026	20235116	Similar to OSIGBa0159H11-OSIGBa0137A07.4 protein.
chr02	Os02g0550600	1	20717898	20721731	Plant neutral invertase family protein.
chr02	Os02g0551600	1	20776247	20777084	Conserved hypothetical protein.
chr02	Os02g0551700	6	20782334	20785252	Protein of unknown function DUF668 domain containing protein.
chr02	Os02g0553500	2	20880412	20882550	Tetratricopeptide-like helical domain containing protein.
chr02	Os02g0554100	12	20913876	20919054	Similar to UVB-resistance protein UVR8.
chr02	Os02g0554800	2	20955271	20956117	Conserved hypothetical protein.
chr02	Os02g0554900	7	20957207	20961970	Similar to Protein disulfide isomerase
chr02	Os02g0575800	2	22102488	22105007	Aldose-1-epimerase domain containing protein.
chr02	Os02g0587000	1	22626274	22627180	Similar to Glycine rich protein
chr02	Os02g0595200	4	23097995	23098588	Conserved hypothetical protein.
chr02	Os02g0595300	2	23101849	23102815	Conserved hypothetical protein.
chr02	Os02g0595500	6	23107011	23110311	Similar to NAD-dependent isocitrate dehydrogenase precursor
chr02	Os02g0596900	2	23177585	23182048	Actin/actin-like family protein.
chr02	Os02g0597000	2	23183131	23186475	Regulator of chromosome condensation 2C RCC1 domain containing protein.
chr02	Os02g0597700	3	23228826	23230766	Ubiquitin domain containing protein.
chr02	Os02g0597800	1	23237192	23241415	Nuclear hormone receptor 2C ligand-binding domain containing protein.
chr02	Os02g0599200	11	23402560	23404718	Protein phosphatase 2C-like domain containing protein.
chr02	Os02g0600400	17	23480321	23486812	Similar to Glucose-6-phosphate dehydrogenase.
chr02	Os02g0601000	2	23513097	23513707	Conserved hypothetical protein.
chr02	Os02g0601100	1	23514645	23515236	Similar to Nucleotide-binding protein.
chr02	Os02g0601700	4	23544805	23549077	Ankyrin repeat containing protein.
chr02	Os02g0602000	4	23559972	23561665	Remorin 2C C-terminal region domain containing protein.
chr02	Os02g0602500	3	23578399	23580839	Similar to Pre-mRNA-splicing factor ISY1.
chr02	Os02g0606300	11	23760964	23766121	Similar to Met-10+ like family protein.
chr02	Os02g0608100	2	23849739	23853728	FAR1 domain containing protein.
chr02	Os02g0608200	4	23855880	23858516	Conserved hypothetical protein.
chr02	Os02g0608801	1	23893258	23894469	Lipase 2C GDSL domain containing protein.
chr02	Os02g0609800	3	23943201	23946456	Hypothetical conserved gene.
chr02	Os02g0610500	1	23989803	23991271	CO-like protein containing two B-box zinc finger domains and one CCT domain 2C Constitutive flowering repressor
chr02	Os02g0610700	5	23998075	24000801	Chlorophyll a/b binding protein domain domain containing protein.
chr02	Os02g0611200	11	24045244	24048294	S-adenosylmethionine decarboxylase 2C Polyamine biosynthesis 2C Abiotic stress
chr02	Os02g0611300	14	24048829	24058147	Zinc finger 2C PHD-type domain containing protein.
chr02	Os02g0611500	9	24068339	24074731	Similar to Eukaryotic initiation factor-like protein.
chr02	Os02g0611800	23	24080704	24084615	Similar to Hydroxyanthranilate hydroxycinnamoyltransferase 3.
chr02	Os02g0612200	4	24098151	24099882	NB-ARC domain containing protein.
chr02	Os02g0613200	6	24166843	24174245	Hypothetical conserved gene.
chr02	Os02g0613400	3	24178420	24181293	Similar to CGS1 mRNA stability 1.
chr02	Os02g0613600	3	24196224	24201250	Similar to Trafficking protein particle complex protein 2
chr02	Os02g0613900	12	24223305	24228759	Similar to Adenine phosphoribosyltransferase 2
chr02	Os02g0615400	1	24323918	24324493	Leucine-rich repeat domain containing protein.
chr02	Os02g0615500	9	24327114	24331291	Protein kinase 2C core domain containing protein.
chr02	Os02g0616199	11	24380848	24382673	Conserved hypothetical protein.
chr02	Os02g0616450	4	24391737	24395742	Hypothetical gene.
chr02	Os02g0617075	2	24466435	24467715	Ribosome-inactivating protein domain containing protein.
chr02	Os02g0617200	5	24482920	24487358	Conserved hypothetical protein.
chr02	Os02g0617300	1	24488770	24501970	Similar to OSIGBa0157A06.7 protein.
chr02	Os02g0617700	5	24531302	24533644	Nucleic acid-binding 2C OB-fold domain containing protein.
chr02	Os02g0620100	7	24667251	24670792	Mis12 family protein.
chr02	Os02g0620800	1	24695219	24698501	Conserved hypothetical protein.
chr02	Os02g0621300	8	24718047	24724116	Fatty acid hydroxylase domain containing protein.
chr02	Os02g0621500	14	24728393	24733113	Zinc finger 2C RING/FYVE/PHD-type domain containing protein.
chr02	Os02g0622100	5	24766809	24772750	Casein kinase I 2C Low temperature tolerance 2C Root development 2C Hormone sensitivity
chr02	Os02g0622200	1	24773729	24775040	Phosphatidylinositol N-acetylglucosaminyltransferase family protein.
chr02	Os02g0623300	2	24819271	24820066	Conserved hypothetical protein.
chr02	Os02g0625900	9	24953147	24960082	Conserved hypothetical protein.
chr02	Os02g0626100	6	24973448	24977287	Similar to Phenylalanine ammonia-lyase.
chr02	Os02g0627100	14	25006495	25009018	Similar to Phenylalanine ammonia-lyase
chr02	Os02g0628200	5	25119080	25122933	Protein of unknown function DUF250 domain containing protein.
chr02	Os02g0629900	6	25191799	25193194	Hypothetical protein.
chr02	Os02g0646500	56	26034336	26041422	Aldehyde dehydrogenase domain containing protein.
chr02	Os02g0648300	1	26099326	26101023	SANT domain 2C DNA binding domain containing protein.
chr02	Os02g0654700	3	26422187	26423485	AP2/ERF family protein 2C Abiotic stress response
chr02	Os02g0695050	21	28555557	28556687	Leucine-rich repeat domain containing protein.
chr02	Os02g0694900	27	28552733	28557616	Protein kinase 2C catalytic domain domain containing protein.
chr02	Os02g0699824	4	28791379	28792577	Conserved hypothetical protein.
chr02	Os02g0699850	1	28793941	28795051	Hypothetical gene.
chr02	Os02g0700000	9	28795583	28800683	EF-HAND 1 domain containing protein.
chr02	Os02g0700100	5	28802911	28805716	Similar to WD-repeat protein.
chr02	Os02g0700300	3	28808920	28811960	Similar to Phosphate starvation response regulator-like protein.
chr02	Os02g0701300	12	28863274	28866997	Conserved hypothetical protein.

chr02	Os02g0710700	6	29438189	29440763	Hypothetical conserved gene.
chr02	Os02g0710800	7	29447864	29451429	Similar to ATP binding / ATP-dependent DNA helicase/ ATP-dependent helicase/ helicase/ nucleic acid binding.
chr02	Os02g0710900	7	29451939	29457739	Heat shock protein Hsp70 family protein.
chr02	Os02g0711000	13	29458151	29461117	Conserved hypothetical protein.
chr02	Os02g0712700	15	29519619	29523253	Concanavalin A-like lectin/glucanase domain containing protein.
chr02	Os02g0712800	45	29523354	29527951	Conserved hypothetical protein.
chr02	Os02g0724500	12	30105720	30109792	Similar to transporter-related.
chr02	Os02g0724600	4	30110288	30115478	Similar to predicted protein.
chr02	Os02g0730775	3	30444816	30447866	Hypothetical protein.
chr02	Os02g0730900	1	30448000	30449510	Pentatricopeptide repeat domain containing protein.
chr02	Os02g0780500	1	33054175	33066629	Conserved hypothetical protein.
chr02	Os02g0807000	2	34468754	34470169	Similar to Phosphoenolpyruvate carboxylase kinase 1.
chr02	Os02g0816000	1	34958012	34961607	C2 domain containing protein.
chr02	Os02g0816300	12	34982175	34989869	Armadillo-type fold domain containing protein.
chr02	Os02g0816900	2	35028569	35040463	Myosin XI
chr02	Os02g0817900	1	35091247	35099291	Cytochrome P450 family protein.
chr02	Os02g0819300	8	35162641	35168331	Hypothetical conserved gene.
chr02	Os02g0823700	15	35376562	35378825	Similar to Xyloglucan endotransglucosylase/hydrolase 9.
chr02	Os02g0823800	2	35379378	35385150	Smg8/Smg9 domain containing protein.
chr02	Os02g0824000	2	35400295	35403096	Hypothetical conserved gene.
chr03	Os03g0115200	6	857832	859757	Similar to Partner of Nob1 3B Pno1p 3B Yor145cp like KH domain containing protein 2C transcripts identified by EST.
chr03	Os03g0115300	5	861320	862938	Pentatricopeptide repeat domain containing protein.
chr03	Os03g0115800	5	887193	888623	Conserved hypothetical protein.
chr03	Os03g0129800	6	1668201	1673176	Similar to Putataive InsB from Escherichia coli.
chr03	Os03g0130200	16	1706399	1711755	Conserved hypothetical protein.
chr03	Os03g0131000	1	1774911	1775816	Similar to H0409D10.5 protein.
chr03	Os03g0131100	1	1776356	1780897	Octicosapeptide/Phox/Bem1p domain containing protein.
chr03	Os03g0135800	8	1990495	1995980	Conserved hypothetical protein.
chr03	Os03g0136200	4	2003246	2004830	24-methylenesterol C-methyltransferase 2
chr03	Os03g0138600	1	2125787	2129505	Hypothetical conserved gene.
chr03	Os03g0138900	1	2139916	2141093	Conserved hypothetical protein.
chr03	Os03g0144100	2	2459016	2459570	Hypothetical genes.
chr03	Os03g0145775	1	2542773	2543266	Conserved hypothetical protein.
chr03	Os03g0161400	6	3310338	3312548	Similar to IQ calmodulin-binding motif family protein 2C expressed.
chr03	Os03g0170200	2	3760520	3762362	Similar to MADS-box transcription factor 30.
chr03	Os03g0188400	1	4628935	4630514	Helix-loop-helix DNA-binding domain containing protein.
chr03	Os03g0193600	1	4886388	4887435	Transcription factor TFIIB domain containing protein.
chr03	Os03g0222500	1	6405347	6406081	Hypothetical conserved gene.
chr03	Os03g0228800	1	6804619	6808346	Similar to LRK1 protein.
chr03	Os03g0229000	3	6814731	6817574	Conserved hypothetical protein.
chr03	Os03g0229100	31	6826703	6832274	Similar to Helix-loop-helix DNA-binding domain containing protein.
chr03	Os03g0233800	1	7088357	7092494	Protein of unknown function DUF547 domain containing protein.
chr03	Os03g0240900	12	7453731	7457759	Tubulin/FtsZ 2C GTPase domain containing protein.
chr03	Os03g0241900	6	7500164	7503768	Similar to Senescence-associated protein 12.
chr03	Os03g0242100	6	7505884	7513338	Conserved hypothetical protein.
chr03	Os03g0242200	7	7514225	7517543	Ras GTPase family protein.
chr03	Os03g0251000	1	7965176	7966095	Plant lipid transfer protein/seed storage/trypsin-alpha amylase inhibitor domain containing protein.
chr03	Os03g0251700	1	7998159	8000400	Similar to Protein kinase domain containing protein 2C expressed.
chr03	Os03g0264075	1	8691493	8692335	Conserved hypothetical protein.
chr03	Os03g0264700	3	8718148	8719815	WD40 repeat-like domain containing protein.
chr03	Os03g0264800	9	8720259	8728315	Similar to ATPase 2C AAA family protein 2C expressed.
chr03	Os03g0267000	1	8834869	8835576	Low molecular mass heat shock protein Oshsp18.0.
chr03	Os03g0267300	1	8841311	8843028	Similar to Fructose-1 2C6-bisphosphatase 2C chloroplast precursor
chr03	Os03g0267500	1	8851332	8854426	Protein of unknown function DUF620 family protein.
chr03	Os03g0267600	2	8854646	8859005	Protein of unknown function DUF3527 domain containing protein.
chr03	Os03g0278700	6	9480447	9488909	Hypothetical conserved gene.
chr03	Os03g0288700	3	9990513	9993307	Phosphatidic acid phosphatase type 2/haloperoxidase domain containing protein.
chr03	Os03g0288900	3	9999638	10002736	Hypothetical protein.
chr03	Os03g0341600	8	12739354	12740552	Pollen Ole e 1 allergen/extensin domain containing protein.
chr03	Os03g0346200	2	12930461	12930788	Conserved hypothetical protein.
chr03	Os03g0351700	6	13189478	13190315	Conserved hypothetical protein.
chr03	Os03g0353600	2	13301955	13305637	Similar to Aberrant pollen transmission 1.
chr03	Os03g0356526	1	13679030	13679794	Hypothetical conserved gene.
chr03	Os03g0374900	3	14784720	14785712	Similar to cationic amino acid transporter.
chr03	Os03g0375200	7	14788216	14789968	Hypothetical conserved gene.
chr03	Os03g0375300	33	14790176	14798032	Similar to cationic amino acid transporter.
chr03	Os03g0389000	9	15563374	15564927	Similar to LOL3.
chr03	Os03g0441101	1	18648052	18648490	Similar to H0323C08.17 protein.
chr03	Os03g0576900	17	21091940	21096981	Amino acid/polyamine transporter I family protein.
chr03	Os03g0581100	2	21334814	21337846	Hypothetical conserved gene.
chr03	Os03g0584050	1	21520109	21522077	Conserved hypothetical protein.
chr03	Os03g0584300	1	21541982	21544545	Conserved hypothetical protein.
chr03	Os03g0585900	6	21607446	21608537	Conserved hypothetical protein.
chr03	Os03g0691050	9	27641720	27644999	Hypothetical gene.
chr03	Os03g0691400	17	27644644	27647511	Thioesterase superfamily domain containing protein.
chr03	Os03g0691500	4	27652240	27655767	Hypothetical conserved gene.
chr03	Os03g0692100	2	27691815	27693150	Hypothetical conserved gene.
chr03	Os03g0693000	12	27736390	27741374	Similar to predicted protein.
chr03	Os03g0693800	1	27783892	27784581	Similar to Oxalate oxidase 1
chr03	Os03g0695500	2	27854761	27858498	Similar to predicted protein.
chr03	Os03g0695600	4	27858693	27861038	Proteasome subunit beta type 2
chr03	Os03g0697833	2	27983768	27986675	Conserved hypothetical protein.
chr03	Os03g0699950	1	28058425	28058695	Conserved hypothetical protein.
chr03	Os03g0700700	14	28106903	28113300	Similar to Lipoxygenase
chr03	Os03g0700800	4	28116620	28124217	Similar to EIN2.
chr03	Os03g0701000	8	28128716	28137109	Similar to HEAT repeat family protein 2C expressed.
chr03	Os03g0701700	7	28169837	28174078	Similar to Ethylene receptor.
chr03	Os03g0701900	5	28186203	28190115	Similar to Pinin/SDK/memA/ protein conserved region containing protein.
chr03	Os03g0703000	3	28240368	28245227	Similar to Beta-glucosidase.
chr03	Os03g0720300	59	29200210	29208674	Similar to Glutamate decarboxylase isozyme 1
chr03	Os03g0720400	3	29209969	29212204	Cyclin-like F-box domain containing protein.
chr03	Os03g0720500	5	29212637	29215913	Hypothetical conserved gene.
chr03	Os03g0720800	8	29236187	29238382	Similar to MYB transcription factor R2R3 type.
chr03	Os03g0722700	27	29323875	29326305	Conserved hypothetical protein.
chr03	Os03g0722800	8	29329004	29336560	Similar to F-box domain containing protein.
chr03	Os03g0723600	6	29397649	29401365	Conserved hypothetical protein.
chr03	Os03g0723700	3	29401966	29403115	Conserved hypothetical protein.
chr03	Os03g0726700	1	29584235	29585030	Conserved hypothetical protein.
chr03	Os03g0726900	5	29592966	29595799	Similar to Ubiquitin ligase protein FANCL.
chr03	Os03g0727000	18	29596367	29606861	Similar to Homeobox protein.

chr03	Os03g0728800	10	29766132	29774115	Similar to RNA helicase
chr03	Os03g0735601	1	30149905	30150520	Hypothetical gene.
chr03	Os03g0735900	1	30151747	30153756	Phospholipid/glycerol acyltransferase domain containing protein.
chr03	Os03g0740600	1	30381680	30385974	Similar to Deoxyhypusine synthase
chr03	Os03g0740833	5	30397995	30401277	Conserved hypothetical protein.
chr03	Os03g0741000	1	30401970	30402872	Similar to Acyl-[acyl-carrier protein] desaturase.
chr03	Os03g0742400	3	30453833	30456585	Similar to MRS2-7.
chr03	Os03g0743650	2	30519650	30521125	Conserved hypothetical protein.
chr03	Os03g0743800	7	30523073	30531098	Similar to predicted protein.
chr03	Os03g0783725	1	32515404	32515721	Hypothetical conserved gene.
chr03	Os03g0783800	1	32523609	32524843	Protein of unknown function DUF295 family protein.
chr03	Os03g0784300	1	32550906	32554338	Similar to predicted protein.
chr03	Os03g0784700	2	32566499	32569324	Ferredoxin--NADP reductase 2C root isozyme 2C chloroplast precursor
chr03	Os03g0784800	17	32570073	32576114	Homo sapiens protein involved in sexual development 2C complete cds.
chr03	Os03g0786500	18	32656085	32660637	Similar to Glycine-rich protein 2.
chr03	Os03g0786700	5	32665994	32671079	N2 2CN2-dimethylguanosine tRNA methyltransferase family protein.
chr03	Os03g0786900	14	32679834	32691068	Tetratricopeptide-like helical domain containing protein.
chr03	Os03g0794800	4	33049423	33060417	Similar to XRN3.
chr03	Os03g0794900	1	33061357	33064912	Frigida-like family protein.
chr03	Os03g0795600	12	33099408	33101484	Uncharacterised protein family UPF0005 domain containing protein.
chr03	Os03g0795800	5	33103037	33105394	Uncharacterised protein family UPF0005 domain containing protein.
chr03	Os03g0795900	10	33105843	33109034	Similar to Heat shock transcription factor 31
chr03	Os03g0796000	9	33110648	33118198	Hypothetical conserved gene.
chr03	Os03g0796301	2	33137576	33138066	Similar to WW domain-binding protein 11.
chr03	Os03g0796501	8	33143530	33146079	Similar to 60S ribosomal protein L4
chr03	Os03g0796700	1	33180922	33181636	Conserved hypothetical protein.
chr03	Os03g0796800	4	33184009	33187019	Similar to guanylate-binding family protein.
chr03	Os03g0796900	12	33187429	33191788	BZIP protein.
chr03	Os03g0797000	10	33194541	33198077	Similar to Indole synthase.
chr03	Os03g0798400	3	33281634	33282656	Prenylated rab acceptor PRA1 family protein.
chr03	Os03g0798500	1	33286651	33288551	Similar to WRKY DNA binding domain containing protein 2C expressed.
chr03	Os03g0802300	2	33474343	33480206	Hypothetical conserved gene.
chr03	Os03g0802700	4	33489601	33493527	RH27 helicase
chr03	Os03g0804400	5	33579493	33585133	Vps51/Vps67 domain containing protein.
chr03	Os03g0816150	3	34207176	34207668	Conserved hypothetical protein.
chr03	Os03g0830200	3	34870676	34871354	Protein of unknown function Cys-rich family protein.
chr03	Os03g0830800	2	34895427	34896125	Pectinesterase inhibitor domain containing protein.
chr03	Os03g0830900	50	34896339	34900928	Lipase 2C class 3 family protein.
chr03	Os03g0839000	11	35255869	35258649	Zinc finger 2C RING/FYVE/PHD-type domain containing protein.
chr03	Os03g0849800	11	35753369	35757214	Nucleotide-diphospho-sugar transferase domain containing protein.
chr03	Os03g0849900	1	35766964	35768997	Nucleotide-diphospho-sugar transferase domain containing protein.
chr03	Os03g0850100	4	35772701	35777460	NLI interacting factor domain containing protein.
chr03	Os03g0850200	2	35777902	35778650	Similar to Cytochrome P450.
chr03	Os03g0850400	80	35790044	35795425	Similar to Aspartokinase.
chr03	Os03g0852400	4	35895803	35896803	Conserved hypothetical protein.
chr03	Os03g0852600	4	35899615	35901119	Protein of unknown function DUF565 family protein.
chr03	Os03g0852700	5	35900827	35903066	Pentatricopeptide repeat domain containing protein.
chr03	Os03g0852800	2	35907129	35908400	Similar to NPC4
chr03	Os03g0852900	6	35908721	35911694	Similar to Cleavage and polyadenylation specificity factor 2C 73 kDa subunit
chr03	Os03g0853200	8	35922773	35923848	Tetraspanin domain containing protein.
chr03	Os03g0853600	7	35955549	35960595	Conserved hypothetical protein.
chr03	Os03g0854800	12	36001958	36007478	Similar to Poly polymerase catalytic domain containing protein 2C expressed.
chr03	Os03g0855100	16	36039164	36043821	Similar to WRKY transcription factor 12.
chr03	Os03g0855500	1	36073172	36074304	Similar to F-box domain containing protein 2C expressed.
chr03	Os03g0855600	14	36074928	36079713	Similar to DANA2.
chr03	Os03g0855700	33	36082364	36096479	Nucleic acid-binding 2C OB-fold domain containing protein.
chr04	Os04g0108600	1	521286	521708	Similar to Sesquiterpene synthase
chr04	Os04g0121100	8	1248450	1260282	Similar to OSIGBa0115D20.4 protein.
chr04	Os04g0127200	2	1705451	1710275	Hypothetical conserved gene.
chr04	Os04g0162600	2	4309095	4310448	Protein of unknown function DUF295 family protein.
chr04	Os04g0162701	2	4314795	4316249	Hypothetical gene.
chr04	Os04g0162800	17	4319058	4321316	Protein of unknown function DUF594 family protein.
chr04	Os04g0163800	18	4353559	4355594	Protein of unknown function DUF594 family protein.
chr04	Os04g0164300	2	4382052	4385552	Ribosomal protein S12/S23 domain containing protein.
chr04	Os04g0164900	80	4408357	4418889	Similar to Starch debranching enzyme.
chr04	Os04g0165000	8	4419427	4421142	Similar to thaumatin-like protein 1a.
chr04	Os04g0165300	8	4436866	4441624	Conserved hypothetical protein.
chr04	Os04g0167800	12	4638237	4640929	Similar to Chalcone reductase homologue
chr04	Os04g0168000	14	4651469	4652572	Conserved hypothetical protein.
chr04	Os04g0169100	5	4738375	4742148	Similar to Ethylene receptor-like protein 1.
chr04	Os04g0178300	24	5318070	5326425	Similar to Isoform 3 of Syn-copalyl diphosphate synthase.
chr04	Os04g0179700	11	5428347	5434023	Syn-pimara-7 2C15-diene synthase.
chr04	Os04g0180300	3	5483444	5484258	Similar to OSIGBa0105N24.3 protein.
chr04	Os04g0181901	2	5560524	5564754	Hypothetical conserved gene.
chr04	Os04g0184100	5	5728101	5731927	Similar to OSIGBa0140C02.7 protein.
chr04	Os04g0184900	11	5778855	5782412	Hypothetical protein.
chr04	Os04g0188500	2	5989525	5990234	Conserved hypothetical protein.
chr04	Os04g0190000	9	6097184	6100998	Similar to OSIGBa0111E13.1 protein.
chr04	Os04g0193200	4	6378760	6380789	Methyl-CpG DNA binding domain containing protein.
chr04	Os04g0193300	7	6384637	6388945	Cyclin-like F-box domain containing protein.
chr04	Os04g0194000	3	6454698	6456042	Cyclin-like F-box domain containing protein.
chr04	Os04g0194433	21	6470608	6474509	Hypothetical gene.
chr04	Os04g0194800	8	6499189	6502384	Hypothetical conserved gene.
chr04	Os04g0196600	5	6593198	6595756	Glycosyltransferase AER61 2C uncharacterized domain containing protein.
chr04	Os04g0197100	1	6630053	6630961	Similar to OSIGBa0146N20.4 protein.
chr04	Os04g0201200	2	6902893	6909851	Hypothetical conserved gene.
chr04	Os04g0201800	18	6912953	6925360	Similar to H0512B01.7 protein.
chr04	Os04g0208400	1	7254765	7256866	Cyclin-like F-box domain containing protein.
chr04	Os04g0221300	11	8083780	8090712	Pentatricopeptide repeat domain containing protein.
chr04	Os04g0223500	3	8243749	8249931	Dimethylaniline monooxygenase 2C N-oxide-forming domain containing protein.
chr04	Os04g0274100	1	11498702	11499639	Conserved hypothetical protein.
chr04	Os04g0275100	2	11569339	11574645	Serine/threonine protein kinase-related domain containing protein.
chr04	Os04g0275950	5	11638394	11642909	Conserved hypothetical protein.
chr04	Os04g0278100	5	11747336	11750502	Hypothetical protein.
chr04	Os04g0291100	5	12669831	12673930	Similar to Short-chain dehydrogenase Tic32.
chr04	Os04g0295500	9	12997753	12999822	Conserved hypothetical protein.
chr04	Os04g0296034	2	13056659	13057081	Zinc finger 2C PMZ-type domain containing protein.
chr04	Os04g0297800	7	13154099	13155441	Similar to OSIGBa0105O19.3 protein.
chr04	Os04g0298700	8	13220130	13225103	Clp 2C N-terminal domain containing protein.
chr04	Os04g0301500	1	13466455	13468869	Helix-loop-helix DNA-binding domain containing protein.

chr04	Os04g0303100	13	13552826	13555333	Similar to H0215E01.10 protein.
chr04	Os04g0304200	18	13641661	13654849	Similar to Nonphototropic hypocotyl protein 1
chr04	Os04g0305700	1	13771472	13773274	Similar to OSIGBa0096F13.5 protein.
chr04	Os04g0306400	1	13802278	13803456	Ribose 5-phosphate isomerase family protein.
chr04	Os04g0307900	1	13913869	13915062	Similar to H0211A12.1 protein.
chr04	Os04g0308100	14	13923888	13925115	Similar to Wall-associated kinase 4.
chr04	Os04g0308400	2	13934207	13937518	Similar to H0211A12.13 protein.
chr04	Os04g0308600	1	13949076	13950288	Similar to H0211A12.11 protein.
chr04	Os04g0310800	1	14072641	14073478	Similar to H0211A12.17 protein.
chr04	Os04g0310900	1	14072868	14074667	Hypothetical protein.
chr04	Os04g0315350	2	14352976	14354418	Similar to cytokinin-O-glucosyltransferase 2.
chr04	Os04g0348300	77	16579948	16587179	Similar to OSIGBa0130K07.8 protein.
chr04	Os04g0348400	24	16590486	16593924	Similar to OSIGBa0130K07.9 protein.
chr04	Os04g0348600	1	16602478	16605137	Hypothetical conserved gene.
chr04	Os04g0353000	1	16843350	16844876	Similar to Cation-transporting ATPase.
chr04	Os04g0365100	97	17584989	17589580	Similar to OSIGBa0135K14.8 protein.
chr04	Os04g0366200	1	17723870	17724941	Hypothetical protein.
chr04	Os04g0367000	10	17761013	17766782	Hypothetical conserved gene.
chr04	Os04g0368000	2	17874313	17877280	Serine/threonine protein kinase-related domain containing protein.
chr04	Os04g0378575	1	18490353	18494159	Similar to NBS-LRR protein
chr04	Os04g0383100	4	18801134	18801475	Conserved hypothetical protein.
chr04	Os04g0383700	1	18823823	18824451	Conserved hypothetical protein.
chr04	Os04g0391000	4	19240745	19245988	Similar to OSIGBa0132D06.6 protein.
chr04	Os04g0439200	11	21853482	21856316	T-complex 11 family protein.
chr04	Os04g0463500	10	23139191	23142817	Similar to Anthranilate synthase beta chain.
chr04	Os04g0463700	13	23148230	23151738	Similar to ADL064Wp.
chr04	Os04g0463800	2	23153453	23158544	Pentatricopeptide repeat domain containing protein.
chr04	Os04g0466700	20	23318895	23326781	Armadillo-type fold domain containing protein.
chr04	Os04g0467100	33	23331134	23336945	Similar to OSIGBa0115M15.9 protein.
chr04	Os04g0470300	5	23500031	23502611	Uncharacterised protein family UPF0136 2C Transmembrane domain containing protein.
chr04	Os04g0470700	9	23512858	23522971	Amino acid transporter 2C transmembrane family protein.
chr04	Os04g0471000	1	23530482	23532361	Similar to H0418A01.4 protein.
chr04	Os04g0471300	4	23544095	23546859	Similar to H0418A01.6 protein.
chr04	Os04g0471400	5	23553857	23560611	Similar to H0418A01.7 protein.
chr04	Os04g0471700	31	23579872	23587188	Similar to WRKY10
chr04	Os04g0477000	3	23876327	23878830	BTB/POZ fold domain containing protein.
chr04	Os04g0477200	3	23882661	23885227	Pentatricopeptide repeat domain containing protein.
chr04	Os04g0479000	12	23955481	23959974	Similar to OSIGBa0116M22.9 protein.
chr04	Os04g0479100	44	23960070	23963329	Similar to OSIGBa0116M22.10 protein.
chr04	Os04g0479200	20	23964000	23967781	Similar to NAD-dependent isocitrate dehydrogenase c 3B1.
chr04	Os04g0479300	29	23968221	23972158	Similar to OSIGBa0116M22.12 protein.
chr04	Os04g0479700	1	23983802	23987394	Similar to Phosphomethylpyrimidine kinase/thiamin-phosphate pyrophosphorylase.
chr04	Os04g0479800	15	23998827	24002236	Cyclin-like F-box domain containing protein.
chr04	Os04g0480050	4	24007292	24007969	Conserved hypothetical protein.
chr04	Os04g0480100	8	24013077	24016828	Eukaryotic translation initiation factor 4B.
chr04	Os04g0480200	3	24018298	24019456	Transporter 2C high affinity nitrate 2C Nar2 domain containing protein.
chr04	Os04g0480300	12	24021491	24025994	Myb 2C DNA-binding domain containing protein.
chr04	Os04g0480400	9	24026635	24029050	Similar to OSIGBa0158F13.7 protein.
chr04	Os04g0480500	26	24032526	24035930	Leucine-rich repeat 2C N-terminal domain containing protein.
chr04	Os04g0480600	14	24044380	24045225	Similar to Cytochrome P450 71A1
chr04	Os04g0480650	7	24046426	24047084	Similar to OSIGBa0158F13.10 protein.
chr04	Os04g0480700	12	24049070	24051142	Cytochrome P450 family protein.
chr04	Os04g0480802	1	24054117	24054476	Hypothetical protein.
chr04	Os04g0480900	34	24054885	24058958	Glycoside hydrolase 2C family 5 protein.
chr04	Os04g0481000	18	24060045	24062872	Similar to H0510A06.1 protein.
chr04	Os04g0481500	2	24078142	24079931	Similar to H0510A06.6 protein.
chr04	Os04g0481700	2	24096146	24098246	Similar to MDR-like ABC transporter.
chr04	Os04g0481800	2	24098311	24099631	Membrane bound O-acyl transferase 2C MBOAT family protein.
chr04	Os04g0481900	2	24101427	24102538	Similar to H0510A06.12 protein.
chr04	Os04g0482800	3	24134006	24138398	Similar to Topoisomerase-like protein.
chr04	Os04g0483200	3	24156588	24161025	Steroid nuclear receptor 2C ligand-binding domain containing protein.
chr04	Os04g0483400	1	24161435	24165181	Similar to H0805A05.2 protein.
chr04	Os04g0483450	10	24168477	24170948	Hypothetical conserved gene.
chr04	Os04g0483600	11	24180805	24187260	Kelch-type beta propeller domain containing protein.
chr04	Os04g0484800	20	24229248	24236905	Cullin family protein.
chr04	Os04g0484900	9	24237179	24240711	Similar to Kluyveromyces lactis strain NRRL Y-1140 chromosome E of strain NRRL Y- 1140 of Kluyveromyces lactis.
chr04	Os04g0485000	1	24240921	24243702	Similar to 26S proteasome subunit RPN7.
chr04	Os04g0485100	7	24243991	24247747	Similar to Protein phosphatase 2A B' regulatory subunit.
chr04	Os04g0485200	5	24253332	24257461	UBX domain containing protein.
chr04	Os04g0485300	13	24261434	24267801	Glucose-6-phosphate dehydrogenase.
chr04	Os04g0485400	4	24267885	24270169	Similar to Nucleotide-binding protein.
chr04	Os04g0486300	2	24294058	24295721	Protein of unknown function DUF295 family protein.
chr04	Os04g0486800	8	24312618	24319681	Similar to predicted protein.
chr04	Os04g0486900	2	24321375	24325557	Acid phosphatase/vanadium-dependent haloperoxidase related family protein.
chr04	Os04g0487200	3	24348107	24350845	Serine/threonine protein kinase domain containing protein.
chr04	Os04g0487400	2	24356759	24357616	Zinc finger 2C RING-type domain containing protein.
chr04	Os04g0532700	1	26637816	26640529	Hypothetical protein.
chr04	Os04g0538000	1	26913320	26919727	Tetratricopeptide TPR-1 domain containing protein.
chr04	Os04g0543700	1	27222677	27226323	Similar to Serine proteinase
chr04	Os04g0545200	5	27318763	27323515	Similar to OSIGBa0101C23.4 protein.
chr04	Os04g0545700	1	27336576	27337862	Transferase family protein.
chr04	Os04g0546100	4	27345290	27347008	Similar to OSIGBa0101C23.10 protein.
chr04	Os04g0546300	2	27353820	27357710	Similar to GMPK2 3DPROTEIN kinase.
chr04	Os04g0546500	3	27362091	27362951	Similar to Oleosin.
chr04	Os04g0548100	3	27431357	27434622	Similar to OSIGBa0106P14.3 protein.
chr04	Os04g0549600	5	27505498	27509994	Heat shock protein DnaJ family protein.
chr04	Os04g0549700	1	27517205	27518340	Similar to Dehydration responsive element binding protein 1F
chr04	Os04g0550400	3	27551423	27559580	Helix-turn-helix 2C AraC type 2C subdomain 2 domain containing protein.
chr04	Os04g0550500	2	27563230	27566440	Similar to N-acetyl glutamate kinase 2.
chr04	Os04g0550600	2	27567824	27570449	Carotenoid oxygenase family protein.
chr04	Os04g0551500	2	27614776	27618001	SBP domain containing protein.
chr04	Os04g0551600	3	27627478	27628262	Zinc finger 2C FYVE/PHD-type domain containing protein.
chr04	Os04g0551700	11	27633216	27637876	PAP fibrillin family protein.
chr04	Os04g0551800	6	27638063	27643857	Similar to T-complex protein 1 2C alpha subunit
chr04	Os04g0554900	1	27759193	27759736	Hypothetical conserved gene.
chr04	Os04g0555300	1	27783578	27787138	Similar to glycerol 3-phosphate permease.
chr04	Os04g0555400	3	27791895	27795858	Similar to Serologically defined breast cancer antigen NY-BR-99.
chr04	Os04g0555500	1	27795857	27798763	WD40 repeat-like domain containing protein.
chr04	Os04g0555600	8	27806814	27815440	Similar to WD-40 repeat family protein / beige-related.
chr04	Os04g0555800	4	27820669	27823138	Similar to Actin depolymerizing factor.

chr04	Os04g0555900	3	27823815	27826495	Pyridoxal phosphate-dependent enzyme 2C beta subunit domain containing protein.
chr04	Os04g0556000	3	27829841	27835533	Heavy metal P-type ATPase 2C Xylem loading of copper
chr04	Os04g0600200	6	30287197	30289860	Homodimeric diiron-carboxylate protein 2C Cyanide-resistant respiration in mitochondria 2C Reactive oxygen species
chr04	Os04g0600800	10	30311574	30316221	Similar to B0403H10-OSIGBa0105A11.8 protein.
chr04	Os04g0600900	9	30317735	30321610	Similar to B0403H10-OSIGBa0105A11.9 protein.
chr04	Os04g0605500	10	30572141	30584308	Similar to Calcium-transporting ATPase 8 2C plasma membrane-type
chr04	Os04g0606000	1	30608872	30610396	Transferase family protein.
chr04	Os04g0614300	5	31161634	31163557	Hypothetical conserved gene.
chr04	Os04g0614500	24	31169012	31174731	Pyridoxal phosphate-dependent transferase 2C major region 2C subdomain 1 domain containing protein.
chr04	Os04g0615700	4	31239185	31243430	Argonaute and Dicer protein 2C PAZ domain containing protein.
chr04	Os04g0615800	1	31247863	31249612	Similar to H0525G02.5 protein.
chr04	Os04g0616200	1	31263110	31264993	Similar to H0525G02.9 protein.
chr04	Os04g0616300	29	31274513	31282357	Similar to H0525G02.10 protein.
chr04	Os04g0616500	4	31299841	31307729	Similar to H0525G02.11 protein.
chr04	Os04g0617050	1	31349656	31350385	Similar to H0313F03.1 protein.
chr04	Os04g0617200	3	31353779	31354403	Conserved hypothetical protein.
chr04	Os04g0617350	1	31357541	31357847	Similar to H0313F03.3 protein.
chr04	Os04g0617600	6	31364111	31369399	Similar to Cdc48 cell division control protein 48 2C AAA family.
chr04	Os04g0617800	13	31389665	31393330	Similar to Imidazoleglycerol-phosphate dehydratase 1
chr04	Os04g0617900	6	31393340	31395182	Similar to germin-like protein subfamily 1 member 11.
chr04	Os04g0618050	20	31398698	31400185	Pentatricopeptide repeat containing protein.
chr04	Os04g0618100	3	31400914	31401532	Hypothetical gene.
chr04	Os04g0618400	1	31410510	31410971	Similar to H0313F03.13 protein.
chr04	Os04g0618700	2	31420801	31425092	Protein kinase 2C core domain containing protein.
chr04	Os04g0619300	2	31458951	31462013	Similar to H0114G12.4 protein.
chr04	Os04g0619400	7	31463105	31466291	Protein kinase 2C core domain containing protein.
chr04	Os04g0619700	5	31485367	31488482	Similar to H0114G12.8 protein.
chr04	Os04g0620000	1	31502967	31505045	Similar to Canalicular multispecific organic anion transporter 2
chr04	Os04g0620700	11	31544124	31551203	Nucleotide-binding 2C alpha-beta plait domain containing protein.
chr04	Os04g0620950	8	31554480	31558637	Hypothetical conserved gene.
chr04	Os04g0622501	11	31635735	31639598	Hypothetical gene.
chr04	Os04g0623066	1	31660819	31662691	Similar to XA1.
chr04	Os04g0623100	2	31663355	31665410	Bromodomain containing protein.
chr04	Os04g0623300	2	31669220	31674595	Similar to H0215F08.3 protein.
chr04	Os04g0623400	1	31678912	31683890	Exonuclease domain containing protein.
chr04	Os04g0624100	21	31721661	31726923	Hypothetical conserved gene.
chr04	Os04g0624400	2	31727770	31728411	Similar to H0215F08.12 protein.
chr04	Os04g0624500	9	31749141	31751604	Similar to Polyphenol oxidase
chr04	Os04g0624600	14	31751600	31759420	Similar to Starch synthase DULL1
chr04	Os04g0624900	1	31767073	31767689	Conserved hypothetical protein.
chr04	Os04g0625000	4	31769056	31773154	Conserved hypothetical protein.
chr04	Os04g0625500	8	31809708	31810835	Similar to OSIGBa0148A10.5 protein.
chr04	Os04g0625800	2	31819464	31823467	Similar to RNA Binding Protein 45.
chr04	Os04g0625900	10	31824102	31828850	Similar to OSIGBa0148A10.9 protein.
chr04	Os04g0626450	3	31855518	31858459	NB-ARC domain containing protein.
chr04	Os04g0626500	17	31858607	31867385	Similar to Disease resistance protein RGA2
chr04	Os04g0626900	3	31895293	31900168	Similar to OSIGBa0117N13.4 protein.
chr04	Os04g0627000	6	31900217	31907077	Similar to Homeobox-leucine zipper protein ROC2.
chr04	Os04g0627300	2	31932864	31933709	Similar to H0303G06.2 protein.
chr04	Os04g0627900	4	31957711	31958630	Translation initiation factor SUI1 domain containing protein.
chr04	Os04g0628000	3	31959627	31961695	Protein of unknown function DUF794 2C plant family protein.
chr04	Os04g0628100	2	31962541	31965069	Similar to Polyubiquitin.
chr04	Os04g0628200	4	31965780	31973005	Similar to H0303G06.9 protein.
chr04	Os04g0628900	1	31995412	31998252	Cyclin-P1-1.
chr04	Os04g0629200	2	32008449	32009263	Cupredoxin domain containing protein.
chr04	Os04g0629300	4	32009536	32016407	Similar to H0303G06.18 protein.
chr04	Os04g0629400	1	32017656	32018383	Similar to H0303G06.19 protein.
chr04	Os04g0629500	14	32019411	32022508	Similar to Thioredoxin h.
chr04	Os04g0630000	6	32044764	32048497	SDA1 domain containing protein.
chr04	Os04g0630100	5	32049063	32051694	NAD
chr04	Os04g0630400	5	32063756	32065731	NAD
chr04	Os04g0630500	3	32075395	32076284	Similar to H0323C08.15 protein.
chr04	Os04g0630600	10	32078504	32081665	Similar to H0323C08.16 protein.
chr04	Os04g0630700	27	32083281	32083982	Similar to H0323C08.17 protein.
chr04	Os04g0630900	10	32090880	32093039	Similar to H0105C05.3 protein.
chr04	Os04g0631800	4	32173571	32177352	Similar to H0105C05.10 protein.
chr04	Os04g0632100	2	32178831	32182153	S-locus receptor-like kinase RLK13.
chr04	Os04g0632200	2	32183396	32184034	Hypothetical protein.
chr04	Os04g0633300	2	32229267	32232359	Similar to H0613A10.4 protein.
chr04	Os04g0633600	3	32239057	32242134	Similar to H0613A10.6 protein.
chr04	Os04g0633800	5	32244877	32248172	Protein kinase 2C catalytic domain domain containing protein.
chr04	Os04g0634500	4	32278194	32281408	Similar to H0315F07.6 protein.
chr04	Os04g0634700	11	32286661	32291433	Similar to Diacylglycerol kinase.
chr04	Os04g0635000	2	32297571	32298126	Similar to H0315F07.10 protein.
chr04	Os04g0635900	8	32342580	32348393	DNA repair exonuclease family protein.
chr04	Os04g0636000	4	32348410	32351851	Similar to H0410G08.12 protein.
chr04	Os04g0636600	15	32370472	32378265	Hypothetical conserved gene.
chr04	Os04g0636700	9	32378707	32382044	Similar to 117M18_7.
chr04	Os04g0637400	8	32419840	32423901	Similar to PolI-like DNA polymerase.
chr04	Os04g0653000	3	33306668	33310084	Tify domain containing protein.
chr04	Os04g0653100	2	33311330	33313304	Uncharacterised protein family UPF0136 2C Transmembrane domain containing protein.
chr04	Os04g0653200	19	33314700	33319036	Similar to Low affinity calcium transporter CAX2
chr04	Os04g0654400	1	33374335	33375212	Eggshell protein family protein.
chr04	Os04g0654500	6	33377245	33378457	Conserved hypothetical protein.
chr04	Os04g0654550	7	33383015	33383987	General substrate transporter domain containing protein.
chr04	Os04g0654600	7	33386622	33391644	Protein kinase 2C core domain containing protein.
chr04	Os04g0654800	15	33399564	33402242	Similar to OSIGBa0147J19.7 protein.
chr04	Os04g0655000	5	33407358	33409844	Curculin-like
chr04	Os04g0655300	54	33418637	33421199	Protein kinase 2C core domain containing protein.
chr04	Os04g0655600	10	33429129	33436181	Similar to OSIGBa0147J19.13 protein.
chr04	Os04g0655700	1	33437188	33437749	Similar to OSIGBa0147J19.14 protein.
chr04	Os04g0656100	20	33457745	33464852	Similar to Plasma membrane H+-ATPase-like protein
chr04	Os04g0663100	17	33840011	33845901	Similar to OSIGBa0099L20.2 protein.
chr04	Os04g0663200	1	33848655	33850175	Zinc finger 2C CCCH-type domain containing protein.
chr04	Os04g0663300	4	33854490	33856500	Similar to PHD finger-like domain protein 5A
chr04	Os04g0663500	10	33856586	33859127	Similar to OSIGBa0099L20.5 protein.
chr04	Os04g0663600	1	33860374	33861424	Similar to WUSCHEL-related homeobox 1A.
chr04	Os04g0663700	8	33869085	33872898	Similar to OSIGBa0099L20.7 protein.
chr05	Os05g0141700	1	2400650	2401930	Phosphoglycerate mutase domain containing protein.
chr05	Os05g0142400	1	2464329	2465034	Conserved hypothetical protein.

chr05	Os05g0158600	10	3459576	3465989	Similar to OsGA2ox1.
chr05	Os05g0189300	7	5472720	5474678	Vegetative storage protein/acid phosphatase domain containing protein.
chr05	Os05g0192700	15	5673470	5674010	Conserved hypothetical protein.
chr05	Os05g0193900	16	5756504	5760603	Hypothetical conserved gene.
chr05	Os05g0198100	14	6057365	6060278	Protein of unknown function DUF247 2C plant domain containing protein.
chr05	Os05g0198200	4	6085887	6087017	Thioredoxin fold domain containing protein.
chr05	Os05g0198400	11	6090801	6094068	Similar to Zinc transporter 4 2C chloroplast precursor
chr05	Os05g0202200	2	6344746	6345363	Hypothetical protein.
chr05	Os05g0202800	1	6393899	6396069	Similar to Metallothionein-like protein 3B.
chr05	Os05g0203500	17	6419463	6422962	Myb/SANT-like domain domain containing protein.
chr05	Os05g0205100	2	6546758	6547978	NLI interacting factor domain containing protein.
chr05	Os05g0208550	12	6721224	6727270	Similar to gibberellin 2-beta-dioxygenase.
chr05	Os05g0208500	20	6721756	6723481	Hypothetical protein.
chr05	Os05g0213500	12	7034322	7036548	Similar to CAPIP1.
chr05	Os05g0215033	2	7136907	7137963	Hypothetical gene.
chr05	Os05g0215183	1	7149662	7150094	Conserved hypothetical protein.
chr05	Os05g0215300	3	7152334	7153941	UDP-glucuronosyl/UDP-glucosyltransferase family protein.
chr05	Os05g0217700	3	7249980	7251356	BURP domain containing protein.
chr05	Os05g0218100	3	7271691	7272578	Carbohydrate/puine kinase 2C PfkB 2C conserved site domain containing protein.
chr05	Os05g0218400	8	7279432	7284634	Similar to Somatic embryogenesis receptor kinase-like protein.
chr05	Os05g0232500	9	8034111	8039392	Ubiquitin-conjugating enzyme/RWD-like domain containing protein.
chr05	Os05g0235600	17	8301019	8303268	Similar to Nucleosome assembly protein 1-like protein 2.
chr05	Os05g0235701	6	8305741	8306159	Similar to Geranylgeranyl diphosphate synthase
chr05	Os05g0236600	26	8345447	8347374	Protein of unknown function DUF594 domain containing protein.
chr05	Os05g0237400	7	8378906	8382300	Similar to Viroid symptom modulation protein.
chr05	Os05g0241000	9	8584562	8589468	Prefoldin domain containing protein.
chr05	Os05g0243100	26	8689448	8691503	Similar to 50S ribosomal protein L13.
chr05	Os05g0246200	3	8869613	8869900	Similar to UDP-glycosyltransferase UGT710F3.
chr05	Os05g0246300	3	8870363	8871941	Barwin-related endoglucanase domain containing protein.
chr05	Os05g0270800	1	10914088	10916007	Similar to glycinamide ribonucleotide transformylase.
chr05	Os05g0310500	6	14161390	14167285	Similar to Cathepsin B-like cysteine protease.
chr05	Os05g0318100	15	14749809	14753963	Similar to Protein kinase-related protein.
chr05	Os05g0320100	8	14866515	14869847	Cyclin-like F-box domain containing protein.
chr05	Os05g0320500	1	14893395	14894078	Conserved hypothetical protein.
chr05	Os05g0320700	4	14899987	14904546	Similar to Cytochrome P450.
chr05	Os05g0320800	7	14906587	14907252	Hypothetical protein.
chr05	Os05g0321900	11	14948062	14953148	DNA-binding WRKY domain containing protein.
chr05	Os05g0322900	2	14991579	14993673	Similar to WRKY transcription factor 45.
chr05	Os05g0323800	3	15041971	15044759	Similar to ubiquinone biosynthesis protein ubiB.
chr05	Os05g0324700	1	15077683	15078347	Conserved hypothetical protein.
chr05	Os05g0325000	11	15081429	15083245	Similar to OSIGBa0140J09.3 protein.
chr05	Os05g0325200	6	15084916	15086140	Cyclin-related 2 domain containing protein.
chr05	Os05g0326625	1	15132717	15134330	Hypothetical conserved gene.
chr05	Os05g0330700	2	15471101	15471859	Hypothetical gene.
chr05	Os05g0332250	2	15553257	15554363	Conserved hypothetical protein.
chr05	Os05g0332600	2	15574253	15575640	Xanthine/uracil/vitamin C permease family protein.
chr05	Os05g0335800	3	15709038	15710367	TGF-beta receptor 2C type I/II extracellular region family protein.
chr05	Os05g0336000	28	15709282	15728922	Hypothetical protein.
chr05	Os05g0346400	4	16306509	16310464	Protein of unknown function DUF538 family protein.
chr05	Os05g0346500	1	16312809	16319379	Glycoside hydrolase 2C family 85 domain containing protein.
chr05	Os05g0347400	1	16362771	16364006	Protein of unknown function DUF640 domain containing protein.
chr05	Os05g0349000	4	16468040	16472834	Protein of unknown function DUF3355 domain containing protein.
chr05	Os05g0350150	4	16548225	16548915	Similar to HAT family dimerisation domain containing protein.
chr05	Os05g0350500	3	16552223	16555649	Peptidase M24 2C structural domain domain containing protein.
chr05	Os05g0350600	5	16559837	16562519	Similar to Atranbp1b protein.
chr05	Os05g0350700	25	16566541	16577995	Conserved hypothetical protein.
chr05	Os05g0351200	2	16600698	16601813	Similar to AP2 domain transcription factor.
chr05	Os05g0352750	5	16669918	16671903	Zinc finger 2C RING-type domain containing protein.
chr05	Os05g0354400	10	16781887	16786652	Similar to ESK1
chr05	Os05g0357200	8	16928160	16932116	Adenine nucleotide translocator 1 domain containing protein.
chr05	Os05g0357600	14	16966090	16972086	Similar to Developmentally regulated GTP binding protein.
chr05	Os05g0357700	6	16972974	16975853	Similar to CAAX prenyl protease 2.
chr05	Os05g0357800	11	16977838	16982184	Phospholipid/glycerol acyltransferase domain containing protein.
chr05	Os05g0358500	6	17017745	17021890	Protein phosphatase 2C domain containing protein.
chr05	Os05g0360800	12	17197743	17198577	Hypothetical conserved gene.
chr05	Os05g0360900	1	17199779	17200375	Pectinesterase inhibitor domain containing protein.
chr05	Os05g0361200	6	17209599	17216204	Similar to Ferrochelatase.
chr05	Os05g0382900	1	18500678	18503010	Annexin family protein.
chr05	Os05g0383100	1	18508442	18512694	Clathrin adaptor 2C mu subunit domain containing protein.
chr05	Os05g0384600	8	18575668	18578864	ABC transporter-like domain containing protein.
chr05	Os05g0384800	10	18586998	18595443	Similar to Chromosome condensation regulator protein
chr05	Os05g0386000	9	18680766	18685444	Similar to Root determined nodulation 1.
chr05	Os05g0386900	10	18728817	18734916	Reticulon family protein.
chr05	Os05g0387200	4	18738656	18741959	Similar to UDP-sulfoquinovose synthase 2C chloroplast precursor
chr05	Os05g0387750	10	18763585	18768243	Hypothetical gene.
chr05	Os05g0388025	6	18772849	18773587	Similar to DNA binding protein.
chr05	Os05g0388151	19	18774154	18779300	Hypothetical protein.
chr05	Os05g0388600	20	18796477	18801851	Protein of unknown function DUF3411 domain containing protein.
chr05	Os05g0389000	3	18813590	18817685	Similar to AP2-1 protein
chr05	Os05g0389600	14	18862715	18867283	Zinc finger 2C RING/FYVE/PHD-type domain containing protein.
chr05	Os05g0389800	1	18872363	18875789	Similar to ATP-dependent helicase DHX8
chr05	Os05g0389900	5	18877770	18878429	Hypothetical gene.
chr05	Os05g0390000	5	18880611	18887896	Similar to predicted protein.
chr05	Os05g0390100	5	18892000	18900658	Dynamin family protein.
chr05	Os05g0390300	1	18919135	18920528	Conserved hypothetical protein.
chr05	Os05g0390500	5	18928206	18933263	BRCT domain containing protein.
chr05	Os05g0390550	8	18941226	18942889	Conserved hypothetical protein.
chr05	Os05g0390932	4	18969257	18970726	Hypothetical protein.
chr05	Os05g0391600	24	19021698	19030826	Glycosyltransferase AER61 2C uncharacterized domain containing protein.
chr05	Os05g0392050	1	19066737	19067981	Hypothetical conserved gene.
chr05	Os05g0392400	2	19097132	19099197	SNF2-related domain containing protein.
chr05	Os05g0392700	7	19108696	19112785	Mitochondrial substrate carrier family protein.
chr05	Os05g0392900	35	19114517	19115838	Conserved hypothetical protein.
chr05	Os05g0393800	6	19160140	19166101	Protein of unknown function DUF221 domain containing protein.
chr05	Os05g0393900	3	19166560	19168329	Pentatricopeptide repeat domain containing protein.
chr05	Os05g0394301	1	19178614	19181214	Hypothetical gene.
chr05	Os05g0394200	8	19178379	19183276	Hypothetical conserved gene.
chr05	Os05g0395116	1	19226611	19231602	Similar to dehydration-responsive protein-related.
chr05	Os05g0395600	10	19255491	19260019	Hypothetical conserved gene.
chr05	Os05g0396700	1	19306887	19307493	Conserved hypothetical protein.

chr05	Os05g0397100	2	19325595	19326042	Hypothetical protein.
chr05	Os05g0397700	2	19346175	19353385	SecY protein family protein.
chr05	Os05g0402300	4	19588424	19590720	Uncharacterised protein family UPF0005 domain containing protein.
chr05	Os05g0402700	2	19606771	19608914	Similar to Fructose-bisphosphate aldolase 2C cytoplasmic isozyme
chr05	Os05g0402800	15	19609681	19619904	Similar to p-type ATPase transporter.
chr05	Os05g0402900	1	19631965	19633439	Similar to EDGP.
chr05	Os05g0403100	5	19638874	19640194	Peptidase A1 domain containing protein.
chr05	Os05g0403200	1	19639074	19640220	Hypothetical protein.
chr05	Os05g0403300	2	19645657	19647395	Peptidase A1 domain containing protein.
chr05	Os05g0403400	1	19649766	19651093	Mitochodrial transcription termination factor-related family protein.
chr05	Os05g0404000	2	19678186	19680083	Mitochodrial transcription termination factor-related family protein.
chr05	Os05g0405000	17	19718538	19737605	Orthophosphate dikinase precursor
chr05	Os05g0405900	6	19768783	19775018	WD40 repeat-like domain containing protein.
chr05	Os05g0406150	2	19780573	19785694	Hypothetical protein.
chr05	Os05g0407100	3	19837789	19838522	Four F5 protein family protein.
chr05	Os05g0407200	19	19838548	19846592	WD repeat protein 23 domain containing protein.
chr05	Os05g0429500	3	21078436	21079882	Similar to dienelactone hydrolase family protein.
chr05	Os05g0429700	8	21081086	21084058	C2 calcium-dependent membrane targeting domain containing protein.
chr05	Os05g0429802	33	21091737	21093414	Conserved hypothetical protein.
chr05	Os05g0429900	2	21099467	21100588	Similar to MybHv5
chr05	Os05g0430400	8	21126879	21129643	GTP1/OBG domain containing protein.
chr05	Os05g0430800	7	21141378	21143453	Similar to Amidophosphoribosyltransferase 2C chloroplast precursor
chr05	Os05g0430900	10	21144334	21148363	TGF-beta receptor 2C type I/II extracellular region family protein.
chr05	Os05g0431700	25	21184600	21191740	Similar to peptide transporter PTR2-B.
chr05	Os05g0432200	1	21198105	21198987	Similar to Gibberellin-regulated protein 2 precursor.
chr05	Os05g0432400	7	21201969	21204725	Hypothetical conserved gene.
chr05	Os05g0432600	7	21207922	21214430	Alpha/beta hydrolase fold-1 domain containing protein.
chr05	Os05g0433500	5	21247664	21248941	Conserved hypothetical protein.
chr05	Os05g0433900	2	21266342	21267625	Conserved hypothetical protein.
chr05	Os05g0435300	2	21311523	21312499	Protein of unknown function DUF1218 domain containing protein.
chr05	Os05g0435700	1	21322823	21325134	Similar to
chr05	Os05g0435800	4	21325749	21328295	Similar to Subtilisin-like protease.
chr05	Os05g0436100	5	21343174	21345468	Similar to predicted protein.
chr05	Os05g0436400	24	21370156	21376618	Similar to signal peptide peptidase family protein.
chr05	Os05g0436700	13	21385137	21386114	Conserved hypothetical protein.
chr05	Os05g0436900	6	21388790	21392518	Zinc finger 2C DHHC-type domain containing protein.
chr05	Os05g0437700	2	21432457	21435612	Similar to G-box-binding factor 4.
chr05	Os05g0438500	3	21491943	21493609	Similar to Soluble inorganic pyrophosphatase
chr05	Os05g0438700	2	21499449	21500179	Similar to Histone H3.
chr05	Os05g0438800	5	21500616	21504228	Similar to Actin 1.
chr05	Os05g0439750	1	21529083	21529742	Conserved hypothetical protein.
chr05	Os05g0455200	6	22354150	22358564	Similar to Homeodomain protein JUBEL2.
chr05	Os05g0455500	8	22374029	22380820	Similar to Delta-1-pyrroline-5-carboxylate synthetase.
chr05	Os05g0455600	5	22384138	22387806	Prenylated rab acceptor PRA1 family protein.
chr05	Os05g0455700	7	22387970	22392458	Phosphoribosyltransferase domain containing protein.
chr05	Os05g0478800	1	23550611	23551282	Similar to WRKY transcription factor 48.
chr05	Os05g0480800	2	23636020	23637614	Hypothetical conserved gene.
chr05	Os05g0556000	6	27643114	27647653	Oxoglutarate/iron-dependent oxygenase domain containing protein.
chr05	Os05g0556100	8	27648092	27653659	Similar to SDL5A.
chr05	Os05g0556300	5	27660691	27664963	Uncharacterised protein family UPF0089 domain containing protein.
chr05	Os05g0559600	5	27860628	27865020	Glycosyl transferase 2C family 43 protein.
chr05	Os05g0561600	11	27944014	27947970	Protein of unknown function DUF1421 domain containing protein.
chr05	Os05g0561800	7	27950781	27955317	Hypothetical conserved gene.
chr05	Os05g0562400	8	27975677	27983968	DDT domain containing protein.
chr05	Os05g0563400	4	28034380	28039748	Similar to Auxin response factor 5.
chr05	Os05g0563500	3	28040221	28042695	Conserved hypothetical protein.
chr05	Os05g0564500	2	28100584	28100778	Similar to WUSCHEL-related homeobox 12.
chr05	Os05g0564601	1	28107309	28107839	Similar to predicted protein.
chr05	Os05g0569700	2	28351859	28356807	Similar to Glutamine amidotransferase 2C class I 2C active site.
chr06	Os06g0154800	6	2827985	2830314	Hypothetical conserved gene.
chr06	Os06g0180300	1	3981988	3982486	Conserved hypothetical protein.
chr06	Os06g0212100	2	5712154	5714608	Similar to Galactoside 2-alpha-L-fucosyltransferase
chr06	Os06g0213400	8	5784199	5788998	Protein of unknown function DUF829 2C eukaryotic family protein.
chr06	Os06g0218500	60	6085459	6097660	MCM family protein.
chr06	Os06g0218800	2	6101061	6102004	Uncharacterised protein family UPF0503 domain containing protein.
chr06	Os06g0219600	7	6155940	6159841	Similar to Poly
chr06	Os06g0219700	37	6160836	6163651	Similar to TA2 protein
chr06	Os06g0219900	1	6177896	6179163	Similar to Phi-1 protein.
chr06	Os06g0220300	1	6199518	6200510	Similar to Phi-1 protein.
chr06	Os06g0221100	3	6243743	6247717	TLDc domain containing protein.
chr06	Os06g0222100	4	6279737	6281730	Similar to Ramosa 3
chr06	Os06g0222900	13	6349120	6352936	Similar to Type I inositol-1 2C4 2C5-trisphosphate 5-phosphatase 2
chr06	Os06g0223300	23	6372353	6376374	MADS box transcription factor 2C Pollen maturation
chr06	Os06g0223800	1	6401517	6406389	C2 calcium-dependent membrane targeting domain containing protein.
chr06	Os06g0224000	1	6419602	6420378	Protein of unknown function DUF3049 domain containing protein.
chr06	Os06g0224400	3	6452927	6454454	Mitochodrial transcription termination factor-related family protein.
chr06	Os06g0224500	1	6461189	6462628	Mitochodrial transcription termination factor-related family protein.
chr06	Os06g0224650	9	6463494	6465270	Mitochodrial transcription termination factor-related family protein.
chr06	Os06g0225100	9	6479283	6480980	Mitochodrial transcription termination factor-related family protein.
chr06	Os06g0225200	27	6482378	6483751	Mitochodrial transcription termination factor-related family protein.
chr06	Os06g0225300	33	6484167	6488553	Similar to SERK1
chr06	Os06g0225400	2	6489414	6491807	Conserved hypothetical protein.
chr06	Os06g0225600	6	6492817	6493820	Conserved hypothetical protein.
chr06	Os06g0225800	3	6495002	6497904	Shikimate kinase domain containing protein.
chr06	Os06g0229300	9	6725343	6734351	Similar to Hox2b protein.
chr06	Os06g0229400	2	6736085	6737686	Conserved hypothetical protein.
chr06	Os06g0229800	2	6748398	6753302	Similar to Starch synthase IIA.
chr06	Os06g0231600	2	6809574	6811873	Similar to RING-H2 finger protein ATL1Q.
chr06	Os06g0232000	8	6816406	6819804	Similar to Pro-resilin.
chr06	Os06g0232100	1	6823036	6832862	Hypothetical conserved gene.
chr06	Os06g0232400	4	6878753	6879545	Hypothetical conserved gene.
chr06	Os06g0232700	6	6895777	6901079	Similar to SWIM zinc finger family protein.
chr06	Os06g0233000	2	6915702	6916605	Conserved hypothetical protein.
chr06	Os06g0233800	4	6953695	6956081	Conserved hypothetical protein.
chr06	Os06g0234300	9	6995343	6996671	Hypothetical conserved gene.
chr06	Os06g0234400	4	7000436	7001602	Hypothetical conserved gene.
chr06	Os06g0234600	6	7005872	7008330	Domain of unknown function DUF231 2C plant domain containing protein.
chr06	Os06g0234633	16	7013989	7018602	Similar to Inositol phosphate kinase.
chr06	Os06g0235000	3	7031413	7032804	Domain of unknown function DUF231 2C plant domain containing protein.
chr06	Os06g0235100	4	7037047	7038406	Hypothetical conserved gene.

chr06	Os06g0235200	1	7043614	7045626	Protein of unknown function DUF231 domain containing protein.
chr06	Os06g0235300	23	7047260	7052737	ATPase 2C F1/V1/A1 complex 2C alpha/beta subunit 2C nucleotide-binding domain containing protein.
chr06	Os06g0235400	4	7055731	7056204	Hypothetical conserved gene.
chr06	Os06g0236266	3	7086668	7088509	Conserved hypothetical protein.
chr06	Os06g0236300	1	7093925	7096087	Conserved hypothetical protein.
chr06	Os06g0245900	17	7584780	7590605	E2F Family domain containing protein.
chr06	Os06g0250000	2	7785904	7786731	Conserved hypothetical protein.
chr06	Os06g0250300	2	7789570	7789983	Similar to Histone H2A.
chr06	Os06g0251100	8	7835332	7840950	Membrane attack complex component/perforin
chr06	Os06g0251200	9	7841699	7844795	Kelch related domain containing protein.
chr06	Os06g0270982	44	9100687	9102101	Hypothetical protein.
chr06	Os06g0271000	1	9101145	9102125	UDP-glucuronosyl/UDP-glucosyltransferase domain containing protein.
chr06	Os06g0284500	4	10101308	10102349	Zinc finger 2C Dof-type family protein.
chr06	Os06g0286700	3	10387793	10390465	Similar to Piz-t.
chr06	Os06g0291800	2	10711028	10714423	Conserved hypothetical protein.
chr06	Os06g0296900	1	10973966	10976836	Conserved hypothetical protein.
chr06	Os06g0298000	8	11058542	11065385	Conserved hypothetical protein.
chr06	Os06g0298400	9	11085836	11094202	WW/Rsp5/WWP domain containing protein.
chr06	Os06g0298700	5	11103008	11105189	Short-chain dehydrogenase/reductase SDR domain containing protein.
chr06	Os06g0299200	8	11121902	11123663	Similar to H0207B04.1 protein.
chr06	Os06g0300000	49	11170411	11174887	Short-chain dehydrogenase/reductase SDR domain containing protein.
chr06	Os06g0300200	10	11179549	11181358	Similar to H0207B04.1 protein.
chr06	Os06g0312950	1	11964978	11965357	Hypothetical protein.
chr06	Os06g0548500	2	20760706	20761602	Conserved hypothetical protein.
chr06	Os06g0553550	1	21040598	21041308	Conserved hypothetical protein.
chr06	Os06g0554600	1	21090109	21091328	Conserved hypothetical protein.
chr06	Os06g0585982	4	22951386	22955144	Protein kinase 2C catalytic domain domain containing protein.
chr06	Os06g0587100	4	23000435	23001408	Conserved hypothetical protein.
chr06	Os06g0587200	18	23005337	23008846	Protein kinase 2C catalytic domain domain containing protein.
chr06	Os06g0587300	103	23017969	23021462	Hypothetical protein.
chr06	Os06g0589300	8	23105298	23113054	ABC transporter-like domain containing protein.
chr06	Os06g0589700	3	23136424	23139448	Similar to Poly
chr06	Os06g0589800	1	23144729	23148845	Protein kinase 2C core domain containing protein.
chr06	Os06g0590700	4	23196356	23198035	UDP-glucuronosyl/UDP-glucosyltransferase family protein.
chr06	Os06g0591200	14	23227138	23228388	Conserved hypothetical protein.
chr06	Os06g0593200	3	23310215	23311945	UDP-glucuronosyl/UDP-glucosyltransferase family protein.
chr06	Os06g0594100	9	23343928	23350815	Crotonase 2C core domain containing protein.
chr06	Os06g0594200	14	23357768	23360733	Disease resistance protein domain containing protein.
chr06	Os06g0594300	4	23367126	23368775	Hypothetical protein.
chr06	Os06g0595800	3	23438800	23440545	Transferase family protein.
chr06	Os06g0595900	13	23442875	23449453	Conserved hypothetical protein.
chr06	Os06g0596300	6	23467234	23471674	Similar to Acyl-ACP thioesterase
chr06	Os06g0597800	3	23556493	23559215	FAR1 domain containing protein.
chr05	Os05g0480400	2	23605439	23610991	Serine/threonine protein kinase domain containing protein.
chr06	Os06g0598800	10	23608738	23611096	Similar to Fatty acid elongase 1-like protein.
chr06	Os06g0598900	24	23613188	23621153	Similar to Serine-threonine kinase receptor-associated protein
chr06	Os06g0600100	7	23685179	23688096	Similar to TAT-binding protein homolog
chr06	Os06g0600700	7	23721777	23726845	Agenet domain containing protein.
chr06	Os06g0601000	1	23732555	23734397	Homeodomain-like containing protein.
chr06	Os06g0601600	4	23777893	23780396	Cellulose synthase domain containing protein.
chr06	Os06g0602400	14	23806008	23811036	Similar to DEAD-box protein 3 2C X-chromosomal
chr06	Os06g0602900	12	23847493	23851042	Similar to NADP-dependent oxidoreductase P2.
chr06	Os06g0603600	1	23875437	23879965	SPX domain-containing protein 2C Phosphate
chr06	Os06g0604000	1	23898257	23899819	Similar to Ethylene response factor 1.
chr06	Os06g0620200	14	24907230	24909987	Protein kinase 2C catalytic domain domain containing protein.
chr06	Os06g0620550	6	24916372	24916777	Hypothetical protein.
chr06	Os06g0620600	19	24917274	24920283	Hrf1 family protein.
chr06	Os06g0621900	7	24982296	24986652	Similar to Alpha-expansin OsEXPA16
chr06	Os06g0622000	7	24986845	24997890	Zinc finger 2C CW-type domain containing protein.
chr06	Os06g0622500	1	25025002	25028105	Similar to predicted protein.
chr06	Os06g0623200	3	25066390	25071028	Similar to Cinnamoyl-CoA reductase
chr06	Os06g0623300	4	25073667	25076452	NAD
chr06	Os06g0624100	6	25122722	25126173	Similar to predicted protein.
chr06	Os06g0624700	7	25145594	25148015	Protein of unknown function DUF23 family protein.
chr06	Os06g0625200	5	25182993	25184931	Protein kinase-like domain containing protein.
chr06	Os06g0625700	3	25231214	25235848	Glycosyl transferase 2C family 2 domain containing protein.
chr06	Os06g0646400	6	26399276	26405213	Tyrosine protein kinase domain containing protein.
chr06	Os06g0646600	4	26411491	26414775	Similar to Homeobox protein knotted-1-like 11.
chr06	Os06g0646801	23	26433253	26434294	Hypothetical conserved gene.
chr06	Os06g0646900	13	26435460	26439235	Similar to Homogentisic acid geranylgeranyl transferase.
chr06	Os06g0647150	11	26450752	26454632	Hypothetical protein.
chr06	Os06g0647200	2	26450761	26455305	Similar to Response regulator.
chr07	Os07g0102100	6	122255	127934	Similar to Potassium transporter 22.
chr07	Os07g0102200	3	128713	131371	Conserved hypothetical protein.
chr07	Os07g0102300	50	134173	143578	Putative glycosylphosphatidylinositol-anchored protein 2C Regulation of leaf rolling
chr07	Os07g0102500	22	154906	158601	Lupus La protein family protein.
chr07	Os07g0103000	2	182515	183243	Similar to GRF zinc finger family protein 2C expressed.
chr07	Os07g0104500	20	259712	262742	Haem peroxidase 2C plant/fungal/bacterial family protein.
chr07	Os07g0105100	5	292708	294492	Similar to DNA-directed RNA polymerases I 2C II 2C and III 7.3 kDa polypeptide.
chr07	Os07g0105600	1	306054	306968	Photosystem II oxygen evolving complex protein PsbQ family protein.
chr07	Os07g0106100	6	343935	349227	Leucine-rich repeat 2C SDS22 containing protein.
chr07	Os07g0106200	13	349201	353686	Similar to Hexose transporter.
chr07	Os07g0106700	20	373811	383052	Similar to disease resistance response protein 206.
chr07	Os07g0107583	1	412918	413595	Conserved hypothetical protein.
chr07	Os07g0109100	2	496582	498981	Protein of unknown function DUF594 family protein.
chr07	Os07g0111700	8	664618	667101	Flavin monooxygenase FMO domain containing protein.
chr07	Os07g0112000	1	677468	678907	Dimethylaniline monooxygenase-like protein.
chr07	Os07g0112100	8	685467	687222	Similar to J023132I08 2C full insert sequence.
chr07	Os07g0112400	8	693416	694906	3&apos 3B-5&apos 3B exonuclease domain containing protein.
chr07	Os07g0112600	10	697413	698927	Conserved hypothetical protein.
chr07	Os07g0113100	2	706967	719112	Similar to X1
chr07	Os07g0113000	37	708843	718771	Domain of unknown function DUF223 domain containing protein.
chr07	Os07g0113300	1	726627	727559	Hypothetical conserved gene.
chr07	Os07g0113500	2	742920	744853	Pentatricopeptide repeat domain containing protein.
chr07	Os07g0113600	9	749501	753179	Hypothetical conserved gene.
chr07	Os07g0113700	16	753673	763372	Tetratricopeptide-like helical domain containing protein.
chr07	Os07g0114400	6	799617	805177	Casein kinase II alpha subunit.
chr07	Os07g0115200	11	834313	835728	Conserved hypothetical protein.
chr07	Os07g0115300	4	838070	841318	Similar to Peroxidase2 precursor
chr07	Os07g0115700	1	857727	859369	Hypothetical conserved gene.

chr07	Os07g0115720	1	859413	859895	Conserved hypothetical protein.
chr07	Os07g0117350	1	948072	949493	Hypothetical conserved gene.
chr07	Os07g0117400	10	950862	953818	NB-ARC domain containing protein.
chr07	Os07g0119400	4	1055475	1058127	Similar to Pectinesterase like protein.
chr07	Os07g0120650	2	1095077	1095776	Protein of unknown function DUF538 family protein.
chr07	Os07g0120700	7	1096381	1101697	Conserved hypothetical protein.
chr07	Os07g0120800	7	1101854	1105389	Protein of unknown function DUF1719 2C Oryza sativa family protein.
chr07	Os07g0121100	2	1113969	1116298	Protein of unknown function DUF1719 2C Oryza sativa family protein.
chr07	Os07g0122350	1	1164617	1164862	Hypothetical genes.
chr07	Os07g0123100	4	1194474	1195091	Conserved hypothetical protein.
chr07	Os07g0137600	2	1985492	1987785	Conserved hypothetical protein.
chr07	Os07g0137800	5	1992626	1994724	Protein kinase 2C core domain containing protein.
chr07	Os07g0162450	12	3339394	3340629	Protein of unknown function DUF3778 domain containing protein.
chr07	Os07g0162500	10	3342219	3343596	Alpha/beta hydrolase fold-3 domain containing protein.
chr07	Os07g0162600	21	3344780	3346551	Alpha/beta hydrolase fold-3 domain containing protein.
chr07	Os07g0162700	4	3351308	3352672	Alpha/beta hydrolase fold-3 domain containing protein.
chr07	Os07g0162900	2	3360001	3361559	Lipase 2C GDXG 2C active site domain containing protein.
chr07	Os07g0163200	7	3372597	3374464	FBD domain containing protein.
chr07	Os07g0163900	6	3426467	3428467	Pentatricopeptide repeat domain containing protein.
chr07	Os07g0164000	17	3429482	3434732	Methyltransferase type 12 domain containing protein.
chr07	Os07g0164100	9	3436026	3444708	Histone deacetylase superfamily protein.
chr07	Os07g0164900	4	3471834	3476921	Similar to ABA aldehyde oxidase.
chr07	Os07g0165300	28	3505469	3508474	Hypothetical conserved gene.
chr07	Os07g0165700	14	3539543	3542189	Conserved hypothetical protein.
chr07	Os07g0165900	5	3547492	3548592	Plastocyanin-like domain containing protein.
chr07	Os07g0166300	15	3566767	3570883	Hypothetical protein.
chr07	Os07g0167100	12	3602446	3610749	Similar to Yarrowia lipolytica chromosome A of strain CLIB99 of Yarrowia lipolytica.
chr07	Os07g0167500	48	3620456	3630778	Conserved hypothetical protein.
chr07	Os07g0225300	12	6968003	6969254	OsNAC3 protein.
chr07	Os07g0226200	3	7053727	7054324	Hypothetical protein.
chr07	Os07g0227300	2	7110702	7112739	Protein kinase 2C catalytic domain domain containing protein.
chr07	Os07g0227700	1	7145549	7149736	EGF-like region 2C conserved site domain containing protein.
chr07	Os07g0227750	21	7152849	7155428	Hypothetical gene.
chr07	Os07g0227800	14	7152964	7158005	Similar to ubiquinone biosynthesis protein ubiB.
chr07	Os07g0228400	31	7165550	7167725	Cyclin-like F-box domain containing protein.
chr07	Os07g0229200	5	7206630	7209537	Cyclin-like F-box domain containing protein.
chr07	Os07g0240200	2	7786781	7788818	Similar to Beta-1 2C3 glucanase precursor
chr07	Os07g0241500	1	7900573	7902415	UDP-glucuronosyl/UDP-glucosyltransferase family protein.
chr07	Os07g0241700	5	7905050	7909408	Similar to Cytokinin-N-glucosyltransferase 1.
chr07	Os07g0242700	2	7950627	7952162	F-box domain 2C cyclin-like domain containing protein.
chr07	Os07g0242900	1	7966373	7967638	F-box domain 2C cyclin-like domain containing protein.
chr07	Os07g0243200	5	7983125	7989283	Similar to ADP-glucose pyrophosphorylase large subunit 2
chr07	Os07g0244400	1	8043562	8045191	Pentatricopeptide repeat domain containing protein.
chr07	Os07g0244900	3	8064978	8066298	Bifunctional inhibitor/plant lipid transfer protein/seed storage domain containing protein.
chr07	Os07g0246001	17	8129280	8134841	Hypothetical conserved gene.
chr07	Os07g0247000	3	8191035	8192577	Pectinesterase inhibitor domain containing protein.
chr07	Os07g0267200	8	9538087	9541917	Conserved hypothetical protein.
chr07	Os07g0271500	9	10018726	10020733	Similar to Chalcone synthase.
chr07	Os07g0271600	55	10031346	10034702	Similar to MAK16 protein-related.
chr01	Os01g0294500	1	10720396	10721623	Similar to Class III peroxidase 9.
chr07	Os07g0283600	6	10841308	10845737	Conserved hypothetical protein.
chr07	Os07g0285900	16	10988939	10994274	F-box domain 2C cyclin-like domain containing protein.
chr07	Os07g0286000	4	10991914	10992578	Hypothetical conserved gene.
chr07	Os07g0288700	8	11170896	11182966	Similar to white-brown-complex ABC transporter family.
chr07	Os07g0291100	11	11280349	11285318	Transport protein particle
chr07	Os07g0291200	49	11288040	11292046	B-cell receptor-associated 31-like domain containing protein.
chr07	Os07g0293000	37	11383084	11388213	Hypothetical conserved gene.
chr07	Os07g0294100	2	11432125	11435900	Similar to NB-ARC domain containing protein.
chr07	Os07g0406300	3	12544349	12550057	Similar to Glucose-6-phosphate 1-dehydrogenase.
chr07	Os07g0414200	72	13057843	13068285	Methyltransferase 2C METTL2 2C predicted domain containing protein.
chr07	Os07g0414700	10	13072251	13073480	Protein of unknown function DUF707 domain containing protein.
chr07	Os07g0418200	23	13313597	13316522	Conserved hypothetical protein.
chr07	Os07g0418500	34	13323931	13326242	Similar to Cytochrome P450.
chr07	Os07g0418600	5	13354235	13355291	Proline-rich glycoprotein 2C ABA-dependent inhibition of root growth
chr07	Os07g0419000	6	13382831	13383316	Similar to Cytochrome P450.
chr07	Os07g0419100	9	13383377	13383987	Cytochrome P450 family protein.
chr07	Os07g0419500	66	13399139	13411985	Similar to UDP-glucose:sterol glucosyltransferase.
chr07	Os07g0419800	31	13436748	13440194	Hypothetical conserved gene.
chr07	Os07g0421300	110	13534771	13548698	Similar to Alpha glucosidase-like protein.
chr07	Os07g0422700	3	13640924	13642840	Glucoamylase 2C putatuve domain containing protein.
chr07	Os07g0423000	3	13654341	13655841	Mitochodrial transcription termination factor-related family protein.
chr07	Os07g0425000	7	13776950	13778155	Biopterin transport-related protein BT1 family protein.
chr07	Os07g0434500	8	14506007	14510037	Similar to chromatin remodeling complex subunit.
chr07	Os07g0436100	4	14574609	14580312	Similar to predicted protein.
chr07	Os07g0438500	7	14723111	14727159	Octicosapeptide/Phox/Bem1p domain containing protein.
chr07	Os07g0438800	3	14753753	14759288	MYB-CC family protein 2C Pi-starvation signaling
chr07	Os07g0439750	1	14817883	14818361	Hypothetical conserved gene.
chr07	Os07g0440100	1	14824356	14825681	Conserved hypothetical protein.
chr07	Os07g0443500	6	15033384	15037334	Molecular chaperone 2C heat shock protein 2C Hsp40 2C DnaJ domain containing protein.
chr07	Os07g0447000	1	15297784	15300052	Protein of unknown function DUF594 domain containing protein.
chr07	Os07g0448400	2	15376916	15378289	Similar to Aquaporin PIP2-5.
chr07	Os07g0452400	4	15595751	15600607	3'-5' exonuclease domain containing protein.
chr07	Os07g0453132	3	15649283	15652158	Conserved hypothetical protein.
chr07	Os07g0454400	8	15732952	15745599	Conserved hypothetical protein.
chr07	Os07g0454700	3	15753902	15755787	Similar to Trafficking protein particle complex subunit 4
chr07	Os07g0455100	5	15769883	15787386	Hypothetical conserved gene.
chr07	Os07g0456700	1	15884357	15890031	Similar to
chr07	Os07g0456900	5	15899431	15902146	No apical meristem
chr07	Os07g0463400	2	16303025	16307421	Similar to RES protein.
chr07	Os07g0463800	28	16323142	16326992	Similar to Bowman-Birk type bran trypsin inhibitor.
chr07	Os07g0464001	6	16329354	16331080	Hypothetical gene.
chr07	Os07g0464200	12	16346965	16349480	Similar to O-methyltransferase ZRP4.
chr07	Os07g0464600	26	16375059	16387446	ABC transporter-like domain containing protein.
chr07	Os07g0466000	6	16503119	16504714	Conserved hypothetical protein.
chr07	Os07g0467200	8	16600103	16601979	Similar to Clone ZZD536 mRNA sequence.
chr07	Os07g0468100	6	16674227	16675326	Similar to Glutathione S-transferase GST 19
chr07	Os07g0471200	3	16893333	16895018	Hypothetical protein.
chr07	Os07g0480900	2	17496502	17498002	Similar to Cysteine protease
chr07	Os07g0484200	9	17740777	17742290	Similar to Actin-depolymerizing factor 1
chr07	Os07g0484300	9	17744219	17755312	Zinc finger 2C RING/FYVE/PHD-type domain containing protein.

chr07	Os07g0485366	4	17832460	17833743	Similar to OSIGBa0136O08-OSIGBa0153H12.1 protein.
chr07	Os07g0485500	12	17845825	17848717	Protein of unknown function DUF6 2C transmembrane domain containing protein.
chr07	Os07g0486000	13	17870561	17880263	Similar to MSH4.
chr07	Os07g0491900	6	18331928	18333468	Similar to CTF2A
chr07	Os07g0492100	17	18338217	18351636	DNA helicase 2C UvrD/REP type domain containing protein.
chr07	Os07g0628900	2	26059428	26062993	Similar to KI domain interacting kinase 1.
chr07	Os07g0649100	34	27132411	27137858	Armadillo-like helical domain containing protein.
chr07	Os07g0649600	122	27155068	27157912	Similar to OSIGBa0093K19.10 protein.
chr07	Os07g0650600	1	27197715	27201395	Similar to BLE2 protein.
chr07	Os07g0654700	6	27501837	27507177	Similar to BLE2 protein.
chr07	Os07g0654800	16	27504846	27508206	Hypothetical gene.
chr07	Os07g0655600	8	27569369	27571494	Pectin lyase fold/virulence factor domain containing protein.
chr07	Os07g0659800	3	27807792	27811531	Similar to Ubiquitin ligase SINAT5
chr07	Os07g0660200	2	27825945	27831979	Similar to chromatin remodeling complex subunit.
chr07	Os07g0664400	20	28053814	28056606	Short-chain dehydrogenase/reductase SDR domain containing protein.
chr07	Os07g0664600	2	28061378	28063141	Glucose/ribitol dehydrogenase family protein.
chr07	Os07g0668900	2	28250047	28253655	Similar to Serine/threonine-protein kinase PBS1
chr07	Os07g0678600	1	28726817	28729527	Similar to Serine/threonine protein kinase.
chr07	Os07g0679100	1	28752679	28753308	Conserved hypothetical protein.
chr07	Os07g0679400	3	28770335	28772979	Conserved hypothetical protein.
chr07	Os07g0680600	1	28848874	28852236	Protein of unknown function DUF3133 domain containing protein.
chr07	Os07g0681100	6	28863557	28868627	Similar to Receptor-like protein kinase.
chr07	Os07g0681400	10	28889240	28892030	Similar to Calcium-binding protein CAST.
chr07	Os07g0681500	8	28893100	28900962	Ankyrin repeat containing protein.
chr07	Os07g0681600	9	28901514	28908685	DNA helicase 2C ATP-dependent 2C RecQ type domain containing protein.
chr07	Os07g0681700	6	28909026	28914027	Glycosyl transferase 2C family 8 protein.
chr07	Os07g0682250	11	28922317	28923550	Hypothetical protein.
chr07	Os07g0682400	4	28928736	28932799	Nucleotide-binding 2C alpha-beta plait domain containing protein.
chr08	Os08g0102400	2	138407	139410	Hypothetical conserved gene.
chr08	Os08g0104900	8	268733	272077	Protein of unknown function DUF6 2C transmembrane domain containing protein.
chr08	Os08g0105000	5	272854	276978	Zinc finger 2C RING/FYVE/PHD-type domain containing protein.
chr08	Os08g0105100	3	278146	281347	Cyclin-like F-box domain containing protein.
chr08	Os08g0107400	8	361169	366561	Similar to GDP-mannose transporter.
chr08	Os08g0107600	5	371457	373854	DNA repair metallo-beta-lactamase domain containing protein.
chr08	Os08g0108200	19	395230	400100	G-protein 2C beta subunit family protein.
chr08	Os08g0108300	21	400135	401424	Pistil-specific extensin-like protein family protein.
chr08	Os08g0110200	3	536368	539032	Similar to Fertility restorer.
chr08	Os08g0111300	4	594453	598038	Transferase family protein.
chr08	Os08g0112300	1	655771	657391	Transferase domain containing protein.
chr08	Os08g0112400	4	658548	661608	Pentatricopeptide repeat domain containing protein.
chr08	Os08g0112500	5	662169	667531	Similar to Cyclin-dependent protein kinase-like protein.
chr08	Os08g0112950	2	697270	698157	Hypothetical protein.
chr08	Os08g0113200	6	704559	713864	RNA-processing protein 2C HAT helix domain containing protein.
chr08	Os08g0113800	9	740821	745479	Hypothetical conserved gene.
chr08	Os08g0113900	1	746499	747134	Conserved hypothetical protein.
chr08	Os08g0117700	1	974289	978910	Serine/threonine protein kinase domain containing protein.
chr08	Os08g0120000	4	1091154	1094690	Succinate dehydrogenase iron-protein subunit
chr08	Os08g0148566	4	2782926	2785760	Peptidase 2C trypsin-like serine and cysteine domain containing protein.
chr08	Os08g0148600	15	2786227	2792182	Similar to Endoribonuclease Dicer homolog 3a.
chr08	Os08g0150200	23	2889065	2892003	F-box domain 2C cyclin-like domain containing protein.
chr08	Os08g0150600	30	2909637	2912538	F-box domain 2C cyclin-like domain containing protein.
chr08	Os08g0150800	35	2924246	2928513	Similar to Tyrosyl-tRNA synthetase
chr08	Os08g0151000	42	2934798	2940214	Similar to Myb-like DNA-binding domain 2C SHAQKYF class family protein.
chr08	Os08g0152000	1	2989650	2991016	Aquaporin NIP3-2.
chr08	Os08g0152100	15	2997601	2998471	Similar to Aquaporin NIP3-3.
chr08	Os08g0152366	17	3007271	3008757	Hypothetical protein.
chr08	Os08g0153000	5	3054487	3056073	Similar to MDR-like ABC transporter.
chr08	Os08g0154000	2	3102013	3105084	O-methyltransferase 2C family 3 protein.
chr08	Os08g0154200	4	3113598	3115221	Similar to DEAD-box ATP-dependent RNA helicase 45.
chr08	Os08g0154225	3	3115509	3117296	DEAD-box ATP-dependent RNA helicase 42.
chr08	Os08g0154900	12	3158796	3165175	Pleckstrin homology-type domain containing protein.
chr08	Os08g0155400	59	3182341	3190117	Similar to Nitrate/chlorate transporter.
chr08	Os08g0156066	12	3247638	3248588	Hypothetical gene.
chr08	Os08g0248300	10	9049938	9051200	Hypothetical protein.
chr08	Os08g0263000	6	9958741	9960475	Similar to Cytochrome P450-like protein
chr08	Os08g0393600	8	18676001	18678475	Protein-tyrosine phosphatase-like 2C PTPLA domain containing protein.
chr08	Os08g0393700	3	18680131	18681463	Similar to predicted protein.
chr08	Os08g0425300	1	20429460	20431143	Similar to Endoglucanase 21.
chr08	Os08g0425800	9	20461754	20465184	Similar to DRP2 protein
chr08	Os08g0426900	6	20522449	20525910	Conserved hypothetical protein.
chr08	Os08g0519300	5	25778245	25779466	Glycoside hydrolase 2C family 18 2C catalytic domain domain containing protein.
chr08	Os08g0523100	1	26013703	26014560	BTB/POZ-like domain containing protein.
chr08	Os08g0531100	3	26448418	26452961	MAP65/ASE1 family protein.
chr08	Os08g0531200	8	26452131	26462511	WD40 repeat domain containing protein.
chr08	Os08g0536100	11	26785706	26791061	Hypothetical conserved gene.
chr08	Os08g0537200	20	26861859	26862428	Hypothetical conserved gene.
chr08	Os08g0538600	2	26918197	26921534	Stress up-regulated Nod 19 family protein.
chr08	Os08g0538700	1	26922222	26928300	Retinoblastoma-related protein.
chr08	Os08g0539300	7	26948601	26953317	Zinc finger 2C RING/FYVE/PHD-type domain containing protein.
chr08	Os08g0539400	4	26968546	26978236	Similar to Resistance protein T10rga2-1A.
chr08	Os08g0539600	18	26982655	26986691	Similar to Ankyrin repeat domain-containing protein 2.
chr08	Os08g0539700	34	26989664	26997637	NB-ARC domain containing protein.
chr08	Os08g0539900	55	27016951	27027229	NB-ARC domain containing protein.
chr08	Os08g0540300	55	27047369	27054956	Zinc finger 2C C6HC-type domain containing protein.
chr08	Os08g0540400	7	27055091	27059334	Similar to Calcium-dependent protein kinase.
chr08	Os08g0540500	111	27064824	27068182	Bicoid-interacting 3 domain containing protein.
chr08	Os08g0540900	10	27072163	27072652	Conserved hypothetical protein.
chr08	Os08g0541300	2	27086410	27089863	Leucine-rich repeat 2C N-terminal domain containing protein.
chr08	Os08g0541500	8	27103316	27109475	Protein of unknown function DUF936 2C plant family protein.
chr08	Os08g0541800	1	27118803	27119345	Hypothetical conserved gene.
chr08	Os08g0542000	4	27123639	27127540	Similar to Methionine aminopeptidase.
chr08	Os08g0542100	1	27127762	27129490	Similar to 60S ribosomal protein L7.
chr08	Os08g0544500	6	27277738	27290452	Similar to ARP2/3 regulatory protein subunit NAPP.
chr08	Os08g0544700	2	27295561	27296206	Hypothetical protein.
chr08	Os08g0547000	6	27397948	27401077	RNA recognition motif 2C RNP-1 domain containing protein.
chr08	Os08g0547100	5	27403444	27406438	Similar to 6-phosphogluconolactonase.
chr08	Os08g0547800	8	27473747	27475042	Alpha/beta hydrolase fold-3 domain containing protein.
chr08	Os08g0547900	5	27479062	27481059	Similar to Flavonoid 3-monooxygenase.
chr08	Os08g0548500	1	27511196	27513554	Armadillo-like helical domain containing protein.
chr08	Os08g0548700	3	27520994	27521450	Hypothetical conserved gene.

chr08	Os08g0550200	23	27592835	27598698	Multi antimicrobial extrusion protein MatE family protein.
chr08	Os08g0550400	1	27602301	27603934	Similar to RING-H2 finger protein ATL5P.
chr08	Os08g0550600	1	27617790	27621251	Hypothetical conserved gene.
chr08	Os08g0551200	12	27634982	27638852	Hypothetical conserved gene.
chr08	Os08g0551300	1	27643356	27644156	Protein of unknown function DUF241 2C plant family protein.
chr08	Os08g0551366	1	27645208	27645921	Protein of unknown function DUF241 2C plant domain containing protein.
chr08	Os08g0552400	4	27665567	27667468	Hypothetical conserved gene.
chr08	Os08g0552666	1	27672019	27672526	Conserved hypothetical protein.
chr08	Os08g0552800	4	27673598	27674323	Hypothetical conserved gene.
chr08	Os08g0552900	1	27675520	27676439	Protein of unknown function DUF241 2C plant domain containing protein.
chr08	Os08g0553050	2	27677447	27678390	Hypothetical conserved gene.
chr08	Os08g0553100	8	27679902	27682169	Hypothetical conserved gene.
chr08	Os08g0553300	4	27684127	27685646	Hypothetical conserved gene.
chr08	Os08g0553400	4	27685838	27687265	Hypothetical conserved gene.
chr08	Os08g0553500	2	27690054	27690770	Protein of unknown function DUF241 2C plant family protein.
chr08	Os08g0553800	4	27695184	27700281	NAD
chr08	Os08g0554699	5	27756270	27756946	Similar to electron transporter.
chr08	Os08g0555200	1	27790207	27794126	Nonaspanin
chr08	Os08g0555600	14	27807895	27810686	Hypothetical conserved gene.
chr08	Os08g0556550	1	27855280	27856246	Hypothetical conserved gene.
chr08	Os08g0556700	1	27859063	27868317	Similar to phosphatase/ protein tyrosine phosphatase.
chr08	Os08g0561000	1	28084607	28086381	Hypothetical conserved gene.
chr08	Os08g0561600	6	28123306	28126259	Protein of unknown function DUF966 family protein.
chr08	Os08g0562500	4	28162972	28165305	Transferase family protein.
chr09	Os09g0240350	1	3062376	3063000	Hypothetical gene.
chr09	Os09g0284300	3	6243180	6245883	Conserved hypothetical protein.
chr09	Os09g0284400	3	6246356	6249737	GrpE nucleotide exchange factor domain containing protein.
chr09	Os09g0284800	4	6266117	6266662	Hypothetical conserved gene.
chr09	Os09g0285800	17	6323254	6323796	Hypothetical protein.
chr09	Os09g0286300	1	6368337	6371361	Hypothetical conserved gene.
chr09	Os09g0286400	21	6374327	6383568	Sodium/hydrogen exchanger subfamily protein.
chr09	Os09g0286600	6	6387891	6389789	Pathogenesis-related transcriptional factor and ERF domain containing protein.
chr09	Os09g0295000	2	7077214	7078851	No apical meristem
chr09	Os09g0295200	24	7083652	7086647	Conserved hypothetical protein.
chr09	Os09g0295300	5	7087266	7087829	Similar to cDNA clone:002-138-B03 2C full insert sequence.
chr09	Os09g0305900	3	7843134	7844185	Putative zinc finger CCCH domain-containing protein 58.
chr09	Os09g0306700	9	7914083	7925334	Phytochrome and flowering time 1 protein.
chr09	Os09g0306800	7	7925628	7938161	Zinc finger 2C C2H2-type domain containing protein.
chr09	Os09g0307400	4	7995705	7997271	Similar to zinc finger 2C C2H2 type family protein.
chr09	Os09g0307800	11	8039179	8046886	SET domain-containing histone methyltransferase 2C H3 lysine 36
chr09	Os09g0309600	1	8198943	8199729	Conserved hypothetical protein.
chr09	Os09g0313500	1	8510839	8516543	NB-ARC domain containing protein.
chr09	Os09g0313550	27	8533820	8542200	Hypothetical gene.
chr09	Os09g0314400	16	8603014	8609484	Protein phosphatase 2C-like domain containing protein.
chr09	Os09g0314500	10	8613403	8618697	Hypothetical conserved gene.
chr09	Os09g0315800	8	8710149	8716892	Phosphotransferase system 2C HPr serine phosphorylation site domain containing protein.
chr09	Os09g0323400	15	9459302	9460255	F-box domain 2C cyclin-like domain containing protein.
chr09	Os09g0323700	3	9486037	9487139	Haem peroxidase family protein.
chr09	Os09g0324000	4	9496346	9497016	Similar to Oleosin.
chr09	Os09g0324200	7	9498904	9500493	Cyclin-like F-box domain containing protein.
chr09	Os09g0324400	2	9505694	9507077	Cyclin-like F-box domain containing protein.
chr09	Os09g0325366	2	9549220	9550410	Hypothetical conserved gene.
chr09	Os09g0326100	17	9577710	9581099	Protein kinase 2C core domain containing protein.
chr09	Os09g0326800	16	9620376	9628563	Similar to PSMD2 subunit
chr09	Os09g0326900	10	9634476	9638003	Similar to Eukaryotic translation initiation factor 5
chr09	Os09g0327200	35	9647717	9650347	Pentatricopeptide repeat domain containing protein.
chr09	Os09g0327300	7	9652209	9654956	Similar to Plastid 5 2C10-methylene-tetrahydrofolate dehydrogenase
chr09	Os09g0327400	14	9655475	9659099	Glycoside hydrolase-type carbohydrate-binding 2C subgroup domain containing protein.
chr09	Os09g0327500	6	9661773	9663035	Conserved hypothetical protein.
chr09	Os09g0327550	36	9663570	9665770	Similar to predicted protein.
chr09	Os09g0327575	70	9667216	9670256	Similar to Pi5-1.
chr09	Os09g0327600	56	9672226	9674673	Similar to Pi5-1.
chr09	Os09g0327800	91	9681913	9684808	Similar to Pi5-2.
chr09	Os09g0328600	50	9743301	9744363	Hypothetical conserved gene.
chr09	Os09g0329000	33	9781580	9783652	BURP domain-containing protein 14.
chr09	Os09g0330000	35	9825477	9832295	UDP-glucuronosyl/UDP-glucosyltransferase family protein.
chr09	Os09g0329800	16	9826420	9827121	Hypothetical protein.
chr09	Os09g0330700	4	9857202	9860810	Ankyrin repeat domain containing protein.
chr09	Os09g0331600	38	9910746	9917711	Ankyrin repeat domain containing protein.
chr09	Os09g0331801	5	9918657	9919062	Similar to Uncharacterized protein ycf73.
chr09	Os09g0331900	147	9921540	9926715	Hypothetical conserved gene.
chr09	Os09g0332100	5	9933451	9934162	Conserved hypothetical protein.
chr09	Os09g0332300	9	9944988	9949327	Similar to PDR20.
chr09	Os09g0332700	2	9970574	9979350	Similar to PDR-type ABC transporter 2
chr09	Os09g0333000	4	10008771	10014439	ABC transporter-like domain containing protein.
chr09	Os09g0334900	4	10158597	10160954	Ankyrin repeat domain containing protein.
chr09	Os09g0344700	2	10751518	10752279	Conserved hypothetical protein.
chr09	Os09g0357400	24	11540699	11556180	Disease resistance protein domain containing protein.
chr09	Os09g0360900	28	11779735	11785476	Conserved hypothetical protein.
chr09	Os09g0361366	4	11805638	11808912	Hypothetical gene.
chr09	Os09g0361500	67	11810070	11815321	Isochorismate synthase domain containing protein.
chr09	Os09g0365000	20	11990015	11993428	Disease resistance protein domain containing protein.
chr09	Os09g0365300	25	11998384	12001136	NB-ARC domain containing protein.
chr09	Os09g0368500	1	12166727	12176968	Terminal catabolism-type polyamine oxidase 2C Anther development
chr09	Os09g0376000	1	12585718	12590898	Conserved hypothetical protein.
chr09	Os09g0440200	2	16325947	16329922	BRCT domain containing protein.
chr10	Os10g0104300	7	345785	347108	Helix-loop-helix DNA-binding domain containing protein.
chr10	Os10g0105700	6	427319	430563	Similar to predicted protein.
chr10	Os10g0105966	3	444530	444696	Hypothetical conserved gene.
chr10	Os10g0106000	4	445128	445571	Hypothetical protein.
chr10	Os10g0108700	5	583024	584657	Transferase family protein.
chr10	Os10g0108750	4	591332	592370	Hypothetical protein.
chr10	Os10g0109066	6	631995	633265	Hypothetical protein.
chr10	Os10g0109600	1	677177	678880	Peroxidase
chr10	Os10g0109700	18	680099	683038	Similar to POT family protein.
chr10	Os10g0109900	4	689474	691847	Major facilitator superfamily 2C general substrate transporter domain containing protein.
chr10	Os10g0111300	16	787911	791157	Similar to Nitrate transporter
chr10	Os10g0111400	30	794387	799383	Similar to Protein kinase domain containing protein 2C expressed.
chr10	Os10g0111700	17	806037	809751	Similar to POT family protein 2C expressed.
chr10	Os10g0113000	1	866391	868564	Similar to Chalcone reductase homologue

chr10	Os10g0113900	9	911557	916911	Similar to NAD
chr10	Os10g0117800	11	1126728	1131484	Guanine nucleotide binding protein
chr10	Os10g0118800	2	1189829	1190703	Hypothetical conserved gene.
chr10	Os10g0118900	1	1193347	1194794	Similar to tRNA-splicing endonuclease positive effector-related.
chr10	Os10g0124500	1	1556778	1559803	Cyclin-like F-box domain containing protein.
chr10	Os10g0135700	1	2211559	2212224	Hypothetical gene.
chr10	Os10g0159800	12	3793940	3803819	Alcohol dehydrogenase superfamily 2C zinc-containing protein.
chr10	Os10g0162834	9	4034799	4038082	Thioredoxin-like fold domain containing protein.
chr10	Os10g0162836	11	4050820	4051634	Similar to Protein kinase domain containing protein.
chr10	Os10g0162838	25	4051304	4055581	Hypothetical protein.
chr10	Os10g0163190	2	4316084	4316670	Hypothetical gene.
chr10	Os10g0163290	6	4326615	4329302	Similar to ABC-2 type transporter family protein.
chr10	Os10g0165300	16	4497746	4503667	Cytochrome P450 family protein.
chr10	Os10g0166600	3	4599943	4602849	Cytochrome P450 family protein.
chr10	Os10g0167200	2	4629337	4630248	Similar to Cytochrome P450 family protein.
chr10	Os10g0167900	3	4680861	4682894	Similar to Chalcone and stilbene synthases 2C N-terminal domain containing protein.
chr10	Os10g0168751	27	4743725	4744277	Similar to Osr40g3 protein.
chr10	Os10g0168900	5	4744350	4747440	Conserved hypothetical protein.
chr10	Os10g0170501	6	4848139	4849125	Conserved hypothetical protein.
chr10	Os10g0174548	7	5157636	5164933	Similar to Calcium binding EGF domain containing protein.
chr10	Os10g0177200	13	5325563	5329971	EF-HAND 2 domain containing protein.
chr10	Os10g0343100	10	10170579	10174444	Syntaxin 6 2C N-terminal domain containing protein.
chr10	Os10g0343200	14	10174526	10177244	Uncharacterised protein family UPF0497 2C trans-membrane plant domain containing protein.
chr10	Os10g0343400	23	10180422	10187639	Cellulose synthase family protein.
chr10	Os10g0345900	2	10357811	10358530	Conserved hypothetical protein.
chr10	Os10g0362300	12	11191933	11203599	Similar to Histidine kinase 3.
chr10	Os10g0363500	1	11289218	11290800	Pentatricopeptide repeat containing protein.
chr09	Os09g0355400	1	11387149	11396236	Protein kinase 2C core domain containing protein.
chr10	Os10g0365001	15	11395165	11396073	Conserved hypothetical protein.
chr10	Os10g0365800	3	11414311	11417030	Protein of unknown function DUF3615 domain containing protein.
chr10	Os10g0368100	42	11537637	11538948	Similar to Glutathione S-transferase GSTU35
chr10	Os10g0368400	97	11543109	11546028	Similar to Adapter-related protein complex 4 sigma 1 subunit
chr10	Os10g0369950	1	11627361	11629055	Hypothetical gene.
chr10	Os10g0370000	13	11629686	11634486	Protein phosphatase 2C-related domain containing protein.
chr10	Os10g0370400	40	11662601	11666585	Similar to NB-ARC domain containing protein.
chr10	Os10g0371100	2	11731475	11732720	Similar to AP2 domain containing protein 2C expressed.
chr10	Os10g0371200	1	11737407	11739004	Conserved hypothetical protein.
chr10	Os10g0372100	8	11784282	11788237	Conserved hypothetical protein.
chr10	Os10g0372800	6	11807697	11811899	Conserved hypothetical protein.
chr10	Os10g0373400	1	11825931	11826899	Hypothetical protein.
chr10	Os10g0374450	7	11874009	11877350	Similar to Barren inflorescence2-like serine/threonine protein kinase
chr10	Os10g0374600	1	11880133	11883412	Hypothetical conserved gene.
chr10	Os10g0375400	3	11918620	11919360	Leucine-rich repeat domain containing protein.
chr10	Os10g0376200	18	11981020	11986066	Leucine-rich repeat 2C typical subtype containing protein.
chr10	Os10g0376900	2	12015873	12017663	Helix-loop-helix DNA-binding domain containing protein.
chr10	Os10g0379100	36	12181085	12183127	Transferase family protein.
chr10	Os10g0379233	2	12181406	12183056	Hypothetical protein.
chr10	Os10g0382100	5	12321053	12321838	Ribosome-inactivating protein domain containing protein.
chr10	Os10g0382300	71	12327444	12336589	Similar to Signal peptide containing large protein with proline stretches.
chr10	Os10g0384600	132	12480539	12506202	Cyclin-like F-box domain containing protein.
chr10	Os10g0387201	1	12784279	12784930	Hypothetical protein.
chr10	Os10g0388100	9	12807152	12812141	Conserved hypothetical protein.
chr10	Os10g0388900	1	12867952	12869919	Conserved hypothetical protein.
chr10	Os10g0389200	2	12886331	12888289	Similar to Red chlorophyll catabolite reductase [Oryza sativa
chr10	Os10g0389800	2	12923153	12928312	Protein kinase 2C core domain containing protein.
chr10	Os10g0390600	77	12977443	12982476	Similar to Alanine aminotransferase 2
chr10	Os10g0390701	12	12983013	12983507	Conserved hypothetical protein.
chr10	Os10g0391400	3	13031180	13032006	Tify domain containing protein.
chr10	Os10g0392700	11	13091133	13096752	Similar to Initiation factor 2 subunit family protein 2C expressed.
chr10	Os10g0392900	6	13107087	13108437	Similar to esterase.
chr10	Os10g0393600	9	13123214	13125751	Similar to esterase.
chr10	Os10g0393700	6	13126198	13127542	Similar to esterase.
chr10	Os10g0393800	9	13131433	13133327	Lipase 2C GDSL domain containing protein.
chr10	Os10g0394200	1	13161347	13163933	Similar to subtilase family protein.
chr10	Os10g0395000	6	13219950	13226081	Protein kinase 2C core domain containing protein.
chr10	Os10g0395500	6	13265745	13269460	Proteasome assembly chaperone 3 domain containing protein.
chr10	Os10g0396300	11	13289659	13292217	Similar to MOB1.
chr10	Os10g0397400	1	13359632	13361009	Similar to Cell elongation protein DIMINUTO
chr10	Os10g0397800	1	13387600	13388592	Mitochondrial carrier protein family protein.
chr10	Os10g0399100	10	13438132	13444187	Similar to Cys/Met metabolism PLP-dependent enzyme family protein.
chr10	Os10g0399901	2	13478227	13482415	Conserved hypothetical protein.
chr10	Os10g0400250	1	13501851	13503806	Tetratricopeptide-like helical domain containing protein.
chr10	Os10g0400500	4	13524417	13525925	Similar to Pyridoxal-dependent decarboxylase conserved domain containing protein 2C expressed.
chr10	Os10g0400900	3	13543442	13552221	Similar to Colon cancer-associated protein Mic1-like containing protein 2C expressed.
chr10	Os10g0403400	13	13690098	13691656	Hypothetical protein.
chr10	Os10g0403700	10	13708218	13713302	Similar to oxidoreductase.
chr10	Os10g0452700	1	16507486	16508277	Eggshell protein family protein.
chr10	Os10g0452800	3	16509089	16512835	Similar to Rev interacting protein mis3-like.
chr10	Os10g0464000	1	17117290	17119815	Similar to Hypersensitive-induced response protein.
chr10	Os10g0464500	1	17140936	17145821	Zinc finger 2C RING-type domain containing protein.
chr10	Os10g0495200	3	18832249	18836049	Similar to Fertility restorer.
chr10	Os10g0516701	1	19956384	19957356	Similar to fringe-related protein.
chr10	Os10g0516800	9	19962773	19971271	Diacylglycerol kinase 2C catalytic region domain containing protein.
chr10	Os10g0519300	6	20082041	20087739	Similar to ATP binding protein.
chr10	Os10g0519800	1	20098367	20100033	Protein of unknown function DUF295 family protein.
chr10	Os10g0521400	9	20175634	20179154	Alpha/beta hydrolase fold-1 domain containing protein.
chr10	Os10g0521700	8	20196610	20201299	Similar to Shaggy-related protein kinase NtK-1
chr10	Os10g0522000	13	20210994	20214302	Similar to Methyltransferase family protein 2C expressed.
chr10	Os10g0561400	1	22128463	22136769	Similar to Transcription factor MYBS3.
chr11	Os11g0108700	4	395576	396910	Similar to Laccase-23.
chr11	Os11g0108800	6	397403	401779	Similar to Protein kinase domain containing protein 2C expressed.
chr11	Os11g0108932	2	417575	421210	Similar to Leucine Rich Repeat family protein 2C expressed.
chr11	Os11g0126400	9	1202740	1206308	Similar to GDA1/CD39 family protein.
chr11	Os11g0126800	2	1225190	1226553	Nucleoside phosphatase GDA1/CD39 family protein.
chr11	Os11g0127000	8	1240063	1245394	Similar to NAC domain-containing protein 77.
chr11	Os11g0136400	5	1681204	1686789	Major facilitator superfamily domain containing protein.
chr11	Os11g0136500	1	1690003	1691592	Similar to 4'-phosphopantetheinyl transferase superfamily protein.
chr11	Os11g0138300	1	1775407	1777333	Conserved hypothetical protein.
chr11	Os11g0138350	1	1777330	1778832	Hypothetical conserved gene.
chr11	Os11g0140400	8	1859333	1865302	Similar to autoinhibited calcium ATPase.

chr11	Os11g0140700	7	1879339	1883768	Similar to Saccharopine dehydrogenase family protein 2C expressed.
chr11	Os11g0141000	7	1894738	1898886	Similar to PP2A regulatory subunit-like protein.
chr11	Os11g0141100	3	1901250	1902469	Hypothetical conserved gene.
chr11	Os11g0141700	1	1942620	1944140	Similar to GRAS family transcription factor containing protein.
chr11	Os11g0141800	2	1946099	1946770	Conserved hypothetical protein.
chr11	Os11g0142800	4	1998790	1999485	Similar to Zinc finger 2C C3HC4 type family protein.
chr11	Os11g0145000	2	2059411	2063380	Similar to phosphatidylinositol-4-phosphate 5-kinase family protein.
chr11	Os11g0147150	5	2148786	2151562	Hypothetical gene.
chr11	Os11g0147200	1	2154400	2158091	Hypothetical conserved gene.
chr11	Os11g0147500	1	2168334	2171145	Heavy metal transport/detoxification protein domain containing protein.
chr11	Os11g0147600	1	2177141	2177719	Conserved hypothetical protein.
chr11	Os11g0150200	12	2335086	2341117	Conserved hypothetical protein.
chr11	Os11g0151400	2	2390496	2392366	Cytochrome P450 family protein.
chr11	Os11g0151800	17	2432926	2437010	Similar to ATPAP18/PAP18.
chr11	Os11g0155500	1	2598116	2602703	Similar to Amino acid permease family protein 2C expressed.
chr11	Os11g0155900	2	2625671	2626264	Histone H3.
chr11	Os11g0156000	2	2631709	2633563	Similar to AP2 domain containing protein RAP2.8
chr11	Os11g0156200	23	2640635	2646300	Peptidase S28 family protein.
chr11	Os11g0156750	9	2696749	2699523	Hypothetical protein.
chr11	Os11g0158400	7	2817601	2822362	Similar to Digalactosyldiacylglycerol synthase 1.
chr11	Os11g0160100	7	2909410	2918095	Zinc finger 2C RING/FYVE/PHD-type domain containing protein.
chr11	Os11g0161200	1	2987096	2987410	Similar to ATOZI1.
chr11	Os11g0161900	1	3026703	3027395	Similar to Integral membrane protein.
chr11	Os11g0162000	1	3027714	3031891	Autophagy-related protein 13 domain containing protein.
chr11	Os11g0165700	1	3173825	3175505	Mannose-binding lectin domain containing protein.
chr11	Os11g0166100	2	3178940	3181626	Cyclin-like F-box domain containing protein.
chr11	Os11g0166201	1	3183224	3183778	Similar to BZIP transcription factor family protein 2C expressed.
chr11	Os11g0177400	1	3853571	3856790	Similar to ATPase 2C coupled to transmembrane movement of substances.
chr11	Os11g0184800	1	4295162	4297455	Similar to Aspartic proteinase Asp1 precursor
chr11	Os11g0184900	13	4299277	4301784	Similar to OsNAC5 protein [imported]-rice.
chr11	Os11g0186050	1	4355110	4355880	Hypothetical protein.
chr11	Os11g0208100	2	5530077	5534187	Cyclin-like F-box domain containing protein.
chr11	Os11g0209050	5	5609834	5610544	Similar to nucleic acid binding protein.
chr11	Os11g0209100	5	5611296	5615069	Cyclin-like F-box domain containing protein.
chr11	Os11g0209200	1	5615461	5618397	Similar to Ribosomal RNA apurinic site specific lyase.
chr11	Os11g0211300	3	5777015	5787839	Similar to NBS-LRR disease resistance protein homologue
chr11	Os11g0211650	13	5799558	5802020	Similar to Leucine Rich Repeat family protein.
chr11	Os11g0218560	6	6187696	6188042	Similar to Protein kinase domain containing protein 2C expressed.
chr11	Os11g0218619	2	6188378	6188807	Similar to Protein kinase domain containing protein 2C expressed.
chr11	Os11g0224900	1	6532813	6535926	Similar to NBS-LRR protein
chr11	Os11g0226800	2	6631719	6636377	Similar to NB-ARC domain containing protein 2C expressed.
chr11	Os11g0226900	1	6633737	6635869	Hypothetical protein.
chr11	Os11g0226933	1	6638306	6640336	NB-ARC domain containing protein.
chr11	Os11g0226966	22	6646291	6647404	Hypothetical conserved gene.
chr11	Os11g0227000	9	6649202	6650239	Conserved hypothetical protein.
chr11	Os11g0229333	13	6871951	6878271	Hypothetical gene.
chr11	Os11g0229300	3	6872016	6877424	Similar to NBS-LRR-like protein.
chr11	Os11g0229400	59	6878726	6884768	Similar to NBS-LRR protein
chr11	Os11g0229700	4	6897656	6898840	Similar to NBS-LRR-like protein.
chr11	Os11g0230400	16	6925637	6927149	Protease inhibitor I4 2C serpin domain containing protein.
chr11	Os11g0231400	31	6968635	6970984	Protein of unknown function DUF295 family protein.
chr11	Os11g0231800	8	6980647	6981198	Similar to Serpin
chr11	Os11g0232000	1	7005794	7007235	Putative serpin-Z5.
chr11	Os11g0233000	18	7056514	7058106	Similar to Leucine Rich Repeat family protein.
chr11	Os11g0234200	22	7125124	7128857	Zinc finger 2C RING/FYVE/PHD-type domain containing protein.
chr11	Os11g0235200	6	7193230	7198486	Peptide transporter
chr11	Os11g0235300	2	7202765	7205523	Hypothetical gene.
chr11	Os11g0235400	4	7212407	7214557	Hypothetical genes.
chr11	Os11g0235700	11	7221675	7228567	Similar to FHY1.
chr11	Os11g0236200	3	7261709	7262674	Similar to Sucrose-phosphate synthase.
chr11	Os11g0237600	2	7342094	7345975	Hypothetical conserved gene.
chr11	Os11g0237700	9	7346265	7348818	Similar to URF 4-related.
chr11	Os11g0237900	1	7356166	7359604	Similar to NB-ARC domain containing protein.
chr11	Os11g0239000	6	7419636	7421020	Serpin-Z2A.
chr11	Os11g0239200	2	7428300	7429611	Serpin-Z2B.
chr11	Os11g0239400	1	7434637	7435428	Similar to Serpin
chr11	Os11g0240900	27	7511885	7528278	Protein of unknown function DUF2363 domain containing protein.
chr11	Os11g0241000	6	7543920	7544969	Conserved hypothetical protein.
chr11	Os11g0241100	1	7544235	7544754	Hypothetical protein.
chr11	Os11g0241200	3	7551980	7552671	Protein of unknown function DUF538 family protein.
chr11	Os11g0243600	2	7708790	7709483	Hypothetical protein.
chr11	Os11g0243750	2	7716589	7717070	Similar to NB-ARC domain containing protein 2C expressed.
chr11	Os11g0246600	6	7909213	7916588	Similar to Peptidase S1 and S6 2C chymotrypsin/Hap.
chr11	Os11g0249000	31	8072537	8078086	NB-ARC domain containing protein.
chr11	Os11g0249300	2	8082926	8084825	Conserved hypothetical protein.
chr11	Os11g0249400	1	8085126	8086522	Similar to Polygalacturonase family protein 2C expressed.
chr11	Os11g0258500	2	8568422	8572176	Disease resistance protein domain containing protein.
chr11	Os11g0258400	11	8567230	8572839	Hypothetical protein.
chr11	Os11g0258700	11	8583891	8587507	Similar to Helix-loop-helix DNA-binding domain containing protein.
chr11	Os11g0260200	127	8702363	8704999	Similar to Sulfotransferase domain containing protein 2C expressed.
chr11	Os11g0261500	2	8784187	8786064	Hypothetical conserved gene.
chr11	Os11g0262500	26	8860655	8862772	Similar to F-box domain containing protein.
chr11	Os11g0262600	8	8864479	8866289	Conserved hypothetical protein.
chr11	Os11g0264300	105	8970147	8977440	Similar to RWP-RK domain containing protein 2C expressed.
chr11	Os11g0265500	3	9079181	9085404	Similar to ProFAR isomerase associated family protein 2C expressed.
chr11	Os11g0266800	46	9156607	9173269	Tetratricopeptide-like helical domain containing protein.
chr11	Os11g0267000	1	9177329	9178565	GUN4-like domain containing protein.
chr11	Os11g0267100	6	9178505	9184295	Uncharacterised protein family UPF0454 domain containing protein.
chr11	Os11g0267400	7	9193137	9198640	Similar to ClpC.
chr11	Os11g0268300	42	9244401	9248300	Similar to transferase family protein.
chr11	Os11g0270500	6	9423621	9432504	NB-ARC domain containing protein.
chr11	Os11g0271000	22	9463861	9466447	Hypothetical protein.
chr11	Os11g0271100	4	9471589	9479354	Similar to Blast and wounding induced mitogen-activated protein kinase.
chr11	Os11g0274700	3	9724410	9728188	Hypothetical conserved gene.
chr11	Os11g0276000	42	9794920	9807643	Similar to Roothairless 1.
chr11	Os11g0279750	8	9957024	9957355	Similar to Inhibitor I family protein.
chr11	Os11g0282700	11	10081836	10086180	Homeodomain-like containing protein.
chr11	Os11g0283500	11	10133473	10143842	TGF-beta receptor 2C type I/II extracellular region family protein.
chr11	Os11g0285000	61	10235899	10267375	Similar to Beta-amyrin synthase.
chr11	Os11g0286500	62	10329661	10339334	Conserved hypothetical protein.

chr11	Os11g0286800	42	10352224	10376610	Squalene cyclase domain containing protein.
chr11	Os11g0287000	2	10371608	10372177	Conserved hypothetical protein.
chr11	Os11g0291000	28	10575509	10582899	Similar to Beta-D-xylosidase.
chr11	Os11g0291475	14	10610057	10615844	Hypothetical protein.
chr11	Os11g0291800	1	10637691	10638122	Hypothetical protein.
chr11	Os11g0293900	12	10738489	10747538	UBX domain containing protein.
chr11	Os11g0295000	12	10825629	10831894	Similar to histidine triad family protein / HIT family protein.
chr11	Os11g0295900	9	10863409	10868622	Similar to ASGR-BBM-like1.
chr11	Os11g0296800	6	10911131	10915851	Chromatin SPT2 family protein.
chr11	Os11g0296900	1	10918543	10919959	Similar to Cyclopropane-fatty-acyl-phospholipid synthase family protein 2C expressed.
chr11	Os11g0297300	15	10935559	10939478	Similar to Beta-D-xylosidase.
chr11	Os11g0297650	1	10972643	10973314	Conserved hypothetical protein.
chr11	Os11g0297800	15	10972674	10978662	Similar to Beta-D-xylosidase.
chr11	Os11g0298866	4	11058795	11061528	Conserved hypothetical protein.
chr11	Os11g0428700	4	13719439	13720479	Similar to predicted protein.
chr11	Os11g0429433	4	13763875	13764561	Hypothetical gene.
chr11	Os11g0430000	32	13770580	13785516	Similar to serine carboxypeptidase 1.
chr11	Os11g0433600	2	14028186	14029896	Tetratricopeptide-like helical domain containing protein.
chr11	Os11g0433800	6	14038904	14042499	Protein of unknown function DUF635 family protein.
chr11	Os11g0434850	2	14085190	14086514	Ankyrin repeat domain containing protein.
chr11	Os11g0436450	7	14189093	14201379	Conserved hypothetical protein.
chr11	Os11g0436500	3	14203011	14204045	Protein of unknown function DUF295 domain containing protein.
chr11	Os11g0437800	17	14286671	14287154	2OG-Fe
chr11	Os11g0442380	2	14568688	14571605	Hypothetical gene.
chr11	Os11g0442900	33	14605584	14607289	Zinc finger 2C C2H2-type domain containing protein.
chr11	Os11g0444400	3	14687711	14688398	Conserved hypothetical protein.
chr11	Os11g0445475	1	14744466	14744944	Similar to harpin-induced protein.
chr11	Os11g0449600	1	15075242	15075772	Conserved hypothetical protein.
chr11	Os11g0451700	1	15223376	15224746	Similar to Dehydrin DHN1
chr11	Os11g0454800	23	15403907	15411736	Similar to ATPase 2C histidine kinase 2C DNA gyrase B- 2C and HSP90-like domain containing protein.
chr11	Os11g0456100	10	15447285	15450034	Protein of unknown function DUF760 domain containing protein.
chr11	Os11g0457700	2	15512128	15515505	Hypothetical conserved gene.
chr11	Os11g0458100	2	15531495	15532837	Conserved hypothetical protein.
chr11	Os11g0458500	12	15560842	15563274	Hypothetical conserved gene.
chr11	Os11g0458600	9	15564389	15565050	Similar to MATH domain containing protein.
chr11	Os11g0459200	5	15598634	15600299	Hypothetical protein.
chr11	Os11g0459400	29	15600678	15601310	Hypothetical protein.
chr11	Os11g0460800	10	15683051	15694779	Peptidase S10 2C serine carboxypeptidase family protein.
chr11	Os11g0460900	5	15700955	15701662	Protein of unknown function DUF1118 domain containing protein.
chr11	Os11g0461000	108	15706845	15738054	Peptidase S10 2C serine carboxypeptidase family protein.
chr11	Os11g0461200	1	15747042	15748173	UDP-glucuronosyl/UDP-glucosyltransferase domain containing protein.
chr11	Os11g0462100	1	15764680	15766318	Glycoside hydrolase 2C family 18 protein.
chr11	Os11g0463600	49	15840376	15844396	Similar to FHA domain containing protein 2C expressed.
chr11	Os11g0464600	5	15904809	15905270	Similar to dirigent-like protein pDIR9.
chr11	Os11g0469900	17	16129241	16132519	Similar to Bx2-like protein.
chr11	Os11g0470500	55	16144914	16161474	Similar to Protein kinase domain containing protein 2C expressed.
chr11	Os11g0470600	3	16163244	16164242	Conserved hypothetical protein.
chr11	Os11g0470966	4	16195482	16198355	Hypothetical conserved gene.
chr11	Os11g0471200	63	16205716	16216386	Conserved hypothetical protein.
chr11	Os11g0471300	10	16205860	16214048	Similar to OSIGBa0145N07.3 protein.
chr11	Os11g0474566	3	16394809	16395386	Hypothetical conserved gene.
chr11	Os11g0481150	137	16860235	16864216	NB-ARC domain containing protein.
chr11	Os11g0481200	19	16866331	16872004	Protein of unknown function DUF246 2C plant family protein.
chr11	Os11g0482100	85	16929500	16937094	Similar to Protein transport protein Sec24-like CEF.
chr11	Os11g0482400	9	16943497	16946613	Pentatricopeptide repeat domain containing protein.
chr11	Os11g0482500	4	16948517	16953836	Zinc finger 2C RING/FYVE/PHD-type domain containing protein.
chr11	Os11g0483600	3	17010749	17016159	DNA-binding SAP domain containing protein.
chr11	Os11g0485200	7	17108744	17114089	ATPase 2C P-type 2C K/Mg/Cd/Cu/Zn/Na/Ca/Na/H-transporter family protein.
chr11	Os11g0490200	43	17290977	17294288	Protein kinase 2C catalytic domain domain containing protein.
chr11	Os11g0490300	8	17298741	17299603	Hypothetical conserved gene.
chr11	Os11g0490600	49	17318918	17324441	Conserved hypothetical protein.
chr11	Os11g0490900	13	17352249	17355819	Similar to WRKY transcription factor 72.
chr11	Os11g0491500	1	17383893	17384607	Similar to Plastocyanin-like domain containing protein.
chr11	Os11g0495950	12	17599930	17601552	FAD-linked oxidase 2C FAD-binding 2C subdomain 2 domain containing protein.
chr11	Os11g0496400	4	17616598	17618036	Octicosapeptide/Phox/Bem1p domain containing protein.
chr11	Os11g0496600	7	17631574	17640991	Hypothetical conserved gene.
chr11	Os11g0501000	4	17829546	17831391	Conserved hypothetical protein.
chr11	Os11g0501500	27	17856680	17858107	Conserved hypothetical protein.
chr11	Os11g0519500	8	18690522	18702124	Conserved hypothetical protein.
chr11	Os11g0522000	14	18728970	18733435	XY4 protein
chr11	Os11g0521000	2	18770070	18772676	Lipase 2C GDSL domain containing protein.
chr11	Os11g0520500	24	18780521	18788731	Conserved hypothetical protein.
chr11	Os11g0522900	4	18831750	18835479	Peptidase S10 2C serine carboxypeptidase family protein.
chr11	Os11g0523700	15	18922046	18925254	Similar to Transcription factor ICE1
chr11	Os11g0523800	8	18935399	18941686	Similar to Isoform 3 of Auxin response factor 23.
chr11	Os11g0524051	8	18981927	18987762	Hypothetical gene.
chr11	Os11g0524601	91	18994678	19000728	Similar to NB-ARC domain containing protein 2C expressed.
chr11	Os11g0524900	22	19022642	19026620	Similar to Jacalin-like lectin domain containing protein 2C expressed.
chr11	Os11g0525200	17	19048566	19052876	Similar to Cytochrome P450 51
chr11	Os11g0526800	4	19112659	19116593	Conserved hypothetical protein.
chr11	Os11g0527500	3	19147963	19148639	Hypothetical protein.
chr11	Os11g0528700	7	19184337	19190125	Indole-3-acetic acid
chr11	Os11g0529100	14	19208418	19211842	Similar to Chalcone and stilbene synthases 2C N-terminal domain containing protein.
chr11	Os11g0529500	2	19226962	19228479	Polyketide synthase 2C type III domain containing protein.
chr11	Os11g0533000	8	19414910	19420228	Hypothetical protein.
chr11	Os11g0537900	6	19615010	19615672	Conserved hypothetical protein.
chr11	Os11g0543300	6	19949921	19953420	Protein of unknown function DUF247 2C plant domain containing protein.
chr11	Os11g0543500	3	19956772	19963299	Similar to H0502B11.6 protein.
chr11	Os11g0543800	2	19971044	19972011	Similar to importin-related.
chr11	Os11g0544100	16	19988741	19991058	Conserved hypothetical protein.
chr11	Os11g0544300	5	20002692	20005365	Peptidase C12 2C ubiquitin carboxyl-terminal hydrolase 1 domain containing protein.
chr11	Os11g0544500	16	20008560	20013355	Uncharacterised protein family UPF0016 domain containing protein.
chr11	Os11g0544600	21	20018260	20024765	Histone chaperone domain CHZ domain containing protein.
chr11	Os11g0544800	26	20030056	20034630	Similar to GATB
chr11	Os11g0544866	15	20035464	20040371	Hypothetical gene.
chr11	Os11g0545600	2	20098066	20106820	Similar to chromatin modification-related protein EAF3.
chr11	Os11g0545650	1	20107052	20107816	Conserved hypothetical protein.
chr11	Os11g0545800	8	20119120	20125235	Similar to regulator of chromosome condensation
chr11	Os11g0546000	13	20129027	20134231	Similar to RNase L inhibitor-like protein.
chr11	Os11g0546100	1	20136228	20140598	Transmembrane receptor 2C eukaryota domain containing protein.

chr11	Os11g0546500	3	20152474	20153947	Galactosyl transferase family protein.
chr11	Os11g0546600	14	20174656	20176341	Similar to phospholipase A2.
chr11	Os11g0547000	2	20182477	20187327	Similar to FKF1.
chr11	Os11g0549605	4	20321331	20326035	Domain of unknown function DUF629 domain containing protein.
chr11	Os11g0549615	9	20336764	20340697	Metallophosphoesterase domain containing protein.
chr11	Os11g0549635	22	20359909	20368828	Similar to ABC1 family protein.
chr11	Os11g0549700	6	20426260	20432203	Ankyrin domain containing protein.
chr11	Os11g0549900	15	20433314	20437020	Similar to tubulin alpha-6 chain.
chr11	Os11g0552000	2	20525553	20530015	Similar to Tyrosine/nicotianamine aminotransferases family protein.
chr11	Os11g0552100	1	20533132	20538142	Protein of unknown function DUF724 family protein.
chr11	Os11g0554200	12	20617095	20618386	Conserved hypothetical protein.
chr11	Os11g0555300	6	20643384	20647412	Similar to Rp1-like protein.
chr11	Os11g0555600	9	20651030	20654076	Protein kinase 2C core domain containing protein.
chr11	Os11g0556400	5	20672585	20674513	Serine/threonine protein kinase-related domain containing protein.
chr11	Os11g0556733	7	20683795	20685811	Hypothetical protein.
chr11	Os11g0557500	4	20716456	20718510	Protein kinase 2C core domain containing protein.
chr11	Os11g0557700	10	20720724	20723628	Similar to S-adenosylmethionine-dependent methyltransferase/ catalytic.
chr11	Os11g0557800	3	20723964	20724619	Conserved hypothetical protein.
chr11	Os11g0558200	4	20735117	20737678	Similar to P-type R2R3 Myb protein
chr11	Os11g0558300	4	20743741	20748358	Similar to Acyl CoA synthetase
chr11	Os11g0559200	1	20802978	20806262	Protein kinase 2C core domain containing protein.
chr11	Os11g0562266	4	20947646	20948320	Conserved hypothetical protein.
chr11	Os11g0565920	2	21079173	21082347	Leucine-rich repeat domain containing protein.
chr11	Os11g0566800	2	21102794	21104582	Similar to Bibenzyl synthase
chr11	Os11g0567900	7	21161254	21162387	Hypothetical protein.
chr11	Os11g0568666	3	21193026	21194597	Similar to OSIGBa0130K07.4 protein.
chr11	Os11g0568633	1	21195134	21195969	Conserved hypothetical protein.
chr11	Os11g0568700	10	21201721	21207327	Conserved hypothetical protein.
chr11	Os11g0569300	1	21243403	21246824	Serine/threonine protein kinase domain containing protein.
chr11	Os11g0569500	9	21250919	21254683	Similar to Receptor kinase-like protein.
chr11	Os11g0569600	3	21261123	21265839	Similar to Receptor kinase-like protein.
chr11	Os11g0569800	3	21284892	21289519	Protein kinase 2C catalytic domain domain containing protein.
chr11	Os11g0570825	1	21337280	21337942	Hypothetical protein.
chr11	Os11g0571900	9	21413596	21417634	Targeting for Xklp2 family protein.
chr11	Os11g0572100	18	21443052	21452910	Similar to DNA ligase
chr11	Os11g0572800	3	21490564	21493265	Cyclin-like F-box domain containing protein.
chr11	Os11g0573000	10	21500549	21514325	Peptidase C19 2C ubiquitin carboxyl-terminal hydrolase 2 family protein.
chr11	Os11g0576900	5	21730871	21732981	Similar to HAT family dimerisation domain containing protein 2C expressed.
chr11	Os11g0579600	1	21881646	21882938	Protein of unknown function DUF295 family protein.
chr11	Os11g0580000	1	21906342	21908484	Similar to predicted protein.
chr11	Os11g0582801	7	22030012	22031935	Hypothetical gene.
chr11	Os11g0586900	2	22215791	22219119	Similar to ADP-ribosylation factor-like protein 5.
chr11	Os11g0587000	6	22221628	22231042	Iron-containing protein 2C Strigolactones biosynthesis
chr11	Os11g0597400	1	22763793	22767680	Similar to Leucine Rich Repeat family protein 2C expressed.
chr11	Os11g0597500	1	22768985	22771759	Conserved hypothetical protein.
chr11	Os11g0602800	8	23194895	23197316	Galactose oxidase/kelch 2C beta-propeller domain containing protein.
chr11	Os11g0603200	4	23230112	23234075	Similar to ABCF-type protein.
chr11	Os11g0605100	4	23333715	23337436	NB-ARC domain containing protein.
chr11	Os11g0606300	1	23398772	23401453	Similar to eukaryotic translation initiation factor 3 subunit 2.
chr11	Os11g0606400	47	23403454	23407183	Similar to NB-ARC domain containing protein 2C expressed.
chr11	Os11g0606800	2	23415625	23418653	Similar to NB-ARC domain containing protein.
chr11	Os11g0614500	11	23852063	23860876	Similar to Patatin-like protein.
chr11	Os11g0615700	4	23938240	23942543	Proteasome subunit alpha type 5
chr11	Os11g0620300	9	24180911	24181379	Similar to Nonspecific lipid-transfer protein 2
chr11	Os11g0620500	3	24192139	24195237	Tyrosine protein kinase domain containing protein.
chr11	Os11g0621300	3	24219144	24224045	Protein of unknown function DUF1399 family protein.
chr11	Os11g0630501	2	24733779	24734420	Similar to BTB/POZ domain containing protein.
chr11	Os11g0630700	13	24735670	24737370	Similar to BTB/POZ domain containing protein.
chr11	Os11g0631400	1	24797306	24797659	Hypothetical conserved gene.
chr11	Os11g0632200	28	24847426	24853969	Myb/SANT-like domain domain containing protein.
chr11	Os11g0637100	5	25160019	25161761	Similar to Sugar transporter family protein.
chr11	Os11g0637700	14	25193013	25197347	Nucleotide-binding 2C alpha-beta plait domain containing protein.
chr11	Os11g0638000	3	25213451	25216758	Similar to GTP-binding protein engA.
chr11	Os11g0641800	15	25432425	25434791	Similar to Laccase-20.
chr11	Os11g0643100	7	25501586	25503169	Transferase family protein.
chr11	Os11g0643400	28	25509587	25515285	Similar to Serine carboxypeptidase family protein.
chr11	Os11g0643600	1	25522647	25523204	Hypothetical conserved gene.
chr11	Os11g0644000	10	25542846	25546129	Similar to Protein kinase domain containing protein 2C expressed.
chr11	Os11g0644100	3	25552080	25553309	Leucine-rich repeat 2C N-terminal domain containing protein.
chr11	Os11g0644500	4	25583803	25585451	Similar to Transferase family protein 2C expressed.
chr11	Os11g0644600	4	25589890	25591645	Conserved hypothetical protein.
chr11	Os11g0644800	58	25596663	25608463	Similar to Tyrosine/nicotianamine aminotransferases family protein 2C expressed.
chr11	Os11g0647866	4	25760411	25763215	Similar to NB-ARC domain containing protein.
chr11	Os11g0648000	99	25768487	25772558	Similar to Na+/H+ antiporter.
chr11	Os11g0648700	49	25827938	25833625	Protein of unknown function DUF3615 domain containing protein.
chr11	Os11g0649000	2	25840593	25846990	Conserved hypothetical protein.
chr11	Os11g0654800	1	26195130	26198011	Conserved hypothetical protein.
chr11	Os11g0655700	24	26259796	26261416	Similar to Leucine Rich Repeat family protein.
chr11	Os11g0658800	3	26420114	26423933	Pectin lyase fold/virulence factor domain containing protein.
chr11	Os11g0660300	2	26501646	26504211	WD40 repeat-like domain containing protein.
chr11	Os11g0660700	1	26517361	26518059	Conserved hypothetical protein.
chr11	Os11g0661600	26	26572255	26574116	Similar to Peroxidase
chr11	Os11g0661900	10	26579230	26580721	Conserved hypothetical protein.
chr11	Os11g0664400	1	26774246	26775075	Hypothetical conserved gene.
chr11	Os11g0664500	8	26777816	26783973	Hypothetical conserved gene.
chr11	Os11g0666200	6	26878083	26880047	Similar to Protein kinase domain containing protein.
chr11	Os11g0668300	6	26967537	26977525	Hypothetical conserved gene.
chr11	Os11g0669100	11	27015358	27021340	Calmodulin binding protein-like family protein.
chr11	Os11g0669200	2	27024430	27027589	Similar to Protein kinase domain containing protein 2C expressed.
chr11	Os11g0670100	2	27077916	27080652	Serine-threonine/tyrosine-protein kinase domain containing protein.
chr11	Os11g0670700	1	27090664	27107667	Similar to EAP30 family protein.
chr11	Os11g0670900	3	27111943	27112811	Conserved hypothetical protein.
chr11	Os11g0671650	5	27142195	27145889	Hypothetical gene.
chr11	Os11g0672600	13	27175081	27177923	Reverse transcriptase domain containing protein.
chr11	Os11g0672700	94	27179323	27184026	RecQ DNA helicase 2C RNA silencing
chr11	Os11g0672800	2	27191023	27195490	Hypothetical protein.
chr11	Os11g0673200	22	27203693	27210574	Similar to Auxin-induced beta-glucosidase.
chr11	Os11g0677400	4	27366801	27368303	Pectinesterase inhibitor domain containing protein.
chr11	Os11g0679000	1	27451209	27452951	von Willebrand factor 2C type A domain containing protein.
chr11	Os11g0679700	19	27486903	27490269	Phospholipid/glycerol acyltransferase domain containing protein.

chr11	Os11g0679900	62	27492119	27495458	Conserved hypothetical protein.
chr11	Os11g0680200	43	27495679	27498080	Pentatricopeptide repeat domain containing protein.
chr11	Os11g0682000	15	27583624	27589186	Hypothetical conserved gene.
chr11	Os11g0684000	29	27670321	27673334	Similar to Transcription factor MYB21
chr11	Os11g0684100	11	27683640	27695070	NB-ARC domain containing protein.
chr11	Os11g0684700	4	27703769	27707310	Similar to NB-ARC domain containing protein 2C expressed.
chr11	Os11g0685600	5	27735451	27737645	Conserved hypothetical protein.
chr11	Os11g0686100	3	27758164	27761632	Conserved hypothetical protein.
chr11	Os11g0686400	5	27788716	27792722	Similar to WRKY transcription factor 41.
chr11	Os11g0686500	1	27793778	27797821	NB-ARC domain containing protein.
chr11	Os11g0686900	25	27809097	27818431	Similar to NB-ARC domain containing protein 2C expressed.
chr11	Os11g0690332	2	28075457	28076938	Similar to Protein kinase domain containing protein.
chr11	Os11g0695900	6	28462112	28469811	Armadillo-like helical domain containing protein.
chr11	Os11g0696400	7	28483633	28486502	Glycoside hydrolase 2C family 3 2C N-terminal domain containing protein.
chr11	Os11g0700900	22	28722579	28724062	Glycoside hydrolase 2C subgroup 2C catalytic core domain containing protein.
chr11	Os11g0701000	10	28727508	28728821	Class III chitinase homologue
chr11	Os11g0704600	3	28838859	28842804	Similar to Beta-1 2C3 glucanase precursor
chr12	Os12g0102350	3	103029	103978	Similar to WRKY transcription factor 57.
chr12	Os12g0102400	15	105922	112629	Zinc finger 2C RING/FYVE/PHD-type domain containing protein.
chr12	Os12g0102500	7	112744	120226	Serine/threonine protein kinase-related domain containing protein.
chr12	Os12g0103150	11	150863	152571	Conserved hypothetical protein.
chr12	Os12g0104200	1	205970	207000	Conserved hypothetical protein.
chr12	Os12g0104300	6	220457	225793	Clp 2C N-terminal domain containing protein.
chr12	Os12g0104400	5	226239	230184	Fatty acid desaturase 2C type 1 domain containing protein.
chr12	Os12g0111800	1	614314	616031	Similar to Class III peroxidase 136.
chr12	Os12g0111900	1	617081	617614	GCN5-like 1 domain containing protein.
chr12	Os12g0114100	14	698969	705122	Similar to MAP kinase-like protein.
chr12	Os12g0114200	8	707630	712835	Hypothetical conserved gene.
chr12	Os12g0114400	3	718564	722185	Pentatricopeptide repeat domain containing protein.
chr12	Os12g0121600	7	1018201	1021176	Cation/H+ exchanger domain containing protein.
chr12	Os12g0122000	5	1043608	1047116	SCARECROW.
chr12	Os12g0122100	2	1052828	1053873	Protein of unknown function DUF607 family protein.
chr12	Os12g0135200	1	1732197	1733659	Kelch-type beta propeller domain containing protein.
chr12	Os12g0160200	4	3007925	3009773	Conserved hypothetical protein.
chr12	Os12g0160500	6	3029600	3034635	Similar to Associated with HOX family protein 2C expressed.
chr12	Os12g0161700	27	3100851	3110416	Conserved hypothetical protein.
chr12	Os12g0161900	17	3118079	3124707	Hypothetical conserved gene.
chr12	Os12g0171200	5	3619385	3626426	Similar to tRNA adenylyltransferase-like protein.
chr12	Os12g0171500	8	3634846	3635935	Conserved hypothetical protein.
chr12	Os12g0171600	7	3636454	3637403	Conserved hypothetical protein.
chr12	Os12g0171700	9	3639258	3643421	Conserved hypothetical protein.
chr12	Os12g0171900	2	3650086	3650580	Hypothetical gene.
chr12	Os12g0175600	6	3853810	3854330	Conserved hypothetical protein.
chr12	Os12g0175700	1	3855819	3860795	Nonaspanin
chr12	Os12g0175900	2	3861470	3862853	Similar to RNase P subunit p30 family protein.
chr12	Os12g0175800	4	3857591	3863903	Hypothetical gene.
chr12	Os12g0176100	5	3864215	3864698	Hypothetical gene.
chr12	Os12g0180500	4	4056054	4058982	Similar to Protein kinase domain containing protein.
chr12	Os12g0208900	6	5674410	5676438	Similar to ZF-HD protein dimerisation region containing protein 2C expressed.
chr12	Os12g0209000	5	5685138	5689991	Similar to sporulation protein-related.
chr12	Os12g0209700	6	5736998	5739519	Hypothetical conserved gene.
chr12	Os12g0209800	60	5740543	5747593	Similar to NB-ARC domain containing protein 2C expressed.
chr12	Os12g0209901	8	5749555	5750951	Similar to NB-ARC domain containing protein 2C expressed.
chr12	Os12g0210000	13	5752504	5754821	Similar to NB-ARC domain containing protein 2C expressed.
chr12	Os12g0210200	4	5756479	5760022	Similar to Glutathione S-transferase GST 18
chr12	Os12g0210400	30	5766036	5773224	Protein kinase 2C core domain containing protein.
chr12	Os12g0211400	4	5849738	5852392	A/G-specific adenine glycosylase MutY 2C bacterial form domain containing protein.
chr12	Os12g0211500	13	5856248	5859276	Leucine-rich repeat 2C N-terminal domain containing protein.
chr12	Os12g0216500	8	6168271	6169490	Hypothetical gene.
chr12	Os12g0218100	5	6301536	6302135	Similar to cDNA 2C clone: J065028F04 2C full insert sequence.
chr12	Os12g0218500	2	6343046	6344082	Hypothetical conserved gene.
chr12	Os12g0221700	1	6585435	6586455	Hypothetical conserved gene.
chr12	Os12g0223700	3	6715421	6717177	Conserved hypothetical protein.
chr12	Os12g0224400	1	6754961	6756761	Similar to Exostosin family protein 2C expressed.
chr12	Os12g0225100	5	6799607	6802817	Nucleotide-binding 2C alpha-beta plait domain containing protein.
chr12	Os12g0226400	30	6889996	6896735	Similar to 2-alkenal reductase.
chr12	Os12g0226900	4	6916185	6918431	Similar to Allyl alcohol dehydrogenase.
chr12	Os12g0228400	10	6976909	6982100	Similar to cDNA clone:J033003J10 2C full insert sequence.
chr12	Os12g0228800	1	7014180	7014828	Similar to Calmodulin-like protein.
chr12	Os12g0230100	4	7095860	7101608	Similar to ATP-dependent Clp protease ATP-binding subunit clpA homolog 2C chloroplast precursor
chr12	Os12g0230600	2	7130738	7132096	Protein of unknown function DUF1685 family protein.
chr12	Os12g0232300	9	7235000	7237115	Similar to ZCN20.
chr12	Os12g0232700	4	7243314	7244392	Hypothetical protein.
chr12	Os12g0233100	1	7286400	7287545	WW/Rsp5/WWP domain containing protein.
chr12	Os12g0233200	2	7289748	7291739	Hypothetical conserved gene.
chr12	Os12g0234800	7	7383443	7384747	Hypothetical conserved gene.
chr12	Os12g0236700	24	7520744	7535092	Chaperonin Cpn60/TCP-1 family protein.
chr12	Os12g0236800	6	7536249	7538021	Conserved hypothetical protein.
chr12	Os12g0242800	1	7919575	7920541	TRAM 2C LAG1 and CLN8 homology domain containing protein.
chr12	Os12g0245100	32	8031420	8036687	Conserved hypothetical protein.
chr12	Os12g0245700	1	8076291	8076808	Hypothetical conserved gene.
chr12	Os12g0247700	6	8239432	8241147	Similar to Jasmonate-induced protein.
chr12	Os12g0257500	7	8859355	8860654	Conserved hypothetical protein.
chr12	Os12g0257600	25	8869547	8875025	Similar to Laccase-25.
chr12	Os12g0258200	95	8906428	8914969	Similar to ADL257Cp.
chr12	Os12g0258700	81	8960777	8964016	Cupredoxin domain containing protein.
chr12	Os12g0259800	4	9073742	9077143	Similar to Laccase-25.
chr12	Os12g0261250	5	9164566	9168540	Conserved hypothetical protein.
chr12	Os12g0262100	4	9209610	9211645	HAT dimerisation domain containing protein.
chr12	Os12g0263000	2	9263755	9266580	Similar to Glutathione synthase.
chr12	Os12g0263600	5	9286790	9291494	Similar to Isoflavone reductase-like protein 1.
chr12	Os12g0264500	40	9350914	9365572	Similar to CoA-thioester hydrolase CHY1
chr12	Os12g0264800	3	9374938	9375975	Conserved hypothetical protein.
chr12	Os12g0265201	3	9429281	9433003	Conserved hypothetical protein.
chr12	Os12g0266200	2	9476374	9478760	Similar to Fibrillin-2 precursor.
chr12	Os12g0267200	5	9531874	9540793	Cyclopropane-fatty-acyl-phospholipid synthase domain containing protein.
chr12	Os12g0267400	28	9546322	9547871	Hypothetical protein.
chr12	Os12g0268000	2	9579773	9581772	Similar to Cytochrome P450 71A1
chr12	Os12g0270200	2	9777776	9782458	Pentatricopeptide repeat-DYW protein 2C RNA editing in mitochondria
chr12	Os12g0270900	2	9821581	9823929	Sulfotransferase family protein.

chr12	Os12g0273800	53	10053411	10056454	Similar to OSIGBa0147O06.5 protein.
chr12	Os12g0273900	2	10058775	10061686	Leucine-rich repeat domain containing protein.
chr12	Os12g0274200	3	10070074	10072136	Lipase 2C GDSL domain containing protein.
chr12	Os12g0277000	3	10254122	10257442	Zinc finger 2C RING/FYVE/PHD-type domain containing protein.
chr12	Os12g0277200	2	10258835	10259284	Conserved hypothetical protein.
chr12	Os12g0279100	2	10468382	10478397	Transcription factor jumonji domain containing protein.
chr12	Os12g0279702	3	10522532	10525270	Conserved hypothetical protein.
chr12	Os12g0280050	3	10530403	10530895	Similar to Thioredoxin family protein.
chr12	Os12g0281600	5	10624037	10635373	NB-ARC domain containing protein.
chr12	Os12g0282766	6	10703640	10705631	Conserved hypothetical protein.
chr12	Os12g0283100	1	10711964	10717815	Nucleotidyltransferase domain containing protein.
chr12	Os12g0284525	2	10790598	10791269	Conserved hypothetical protein.
chr12	Os12g0285100	9	10822535	10833618	Hypothetical conserved gene.
chr12	Os12g0285500	13	10848222	10856270	Hypothetical conserved gene.
chr12	Os12g0285600	11	10862295	10872221	Pleckstrin homology-type domain containing protein.
chr12	Os12g0288000	12	10999138	11004664	Similar to cDNA clone:J023146F15 2C full insert sequence.
chr12	Os12g0288400	8	11045896	11057114	Similar to Ubiquitin ligase protein mib
chr12	Os12g0288600	3	11060788	11073485	Conserved hypothetical protein.
chr12	Os12g0290150	1	11194132	11205015	Similar to QS
chr12	Os12g0290200	1	11201823	11202284	Hypothetical protein.
chr12	Os12g0291033	2	11260276	11260721	Hypothetical conserved gene.
chr12	Os12g0291200	7	11265647	11267889	Similar to Ribulose bisphosphate carboxylase small chain.
chr12	Os12g0291400	1	11277247	11278306	Similar to Petunia ribulose 1 2C5-bisphosphate carboxylase small subunit mRNA
chr12	Os12g0297400	5	11716268	11724611	Hypothetical protein.
chr12	Os12g0297500	11	11727437	11744407	Pyruvate phosphate dikinase 2C PEP/pyruvate-binding domain containing protein.
chr12	Os12g0299700	3	11915468	11918594	NOP5 2C N-terminal domain containing protein.
chr12	Os12g0406000	4	12258469	12262067	Conserved hypothetical protein.
chr12	Os12g0406333	15	12276607	12282863	Conserved hypothetical protein.
chr12	Os12g0407000	1	12303450	12304062	Similar to SRF-type transcription factor family protein 2C expressed.
chr12	Os12g0407300	1	12315850	12318391	Similar to intron maturase 2C type II family protein.
chr12	Os12g0409600	3	12428805	12432766	Conserved hypothetical protein.
chr12	Os12g0409700	5	12433463	12439186	Hypothetical conserved gene.
chr12	Os12g0409800	9	12439519	12442244	Conserved hypothetical protein.
chr12	Os12g0410000	1	12446119	12446734	Conserved hypothetical protein.
chr12	Os12g0411700	12	12568930	12587004	ABC transporter-like domain containing protein.
chr12	Os12g0412400	1	12581812	12582469	Hypothetical gene.
chr12	Os12g0415800	2	12807810	12808492	Similar to Histone H3.
chr12	Os12g0419000	3	13025972	13028599	Conserved hypothetical protein.
chr12	Os12g0419700	7	13055949	13060893	Hypothetical gene.
chr12	Os12g0420200	1	13103675	13106278	NAD
chr12	Os12g0427000	2	13599841	13601184	Protein kinase 2C catalytic domain domain containing protein.
chr12	Os12g0428000	1	13657101	13658736	Similar to senescence-associated protein DIN1.
chr12	Os12g0428200	8	13664455	13675555	Autophagy-related protein 1010 domain containing protein.
chr12	Os12g0428600	1	13697949	13702555	Similar to E3 ubiquitin protein ligase UPL2
chr12	Os12g0428700	1	13703310	13703839	Hypothetical protein.
chr12	Os12g0431700	1	13913818	13915949	Hypothetical conserved gene.
chr12	Os12g0432600	1	13982073	13982902	Zinc finger 2C RING/FYVE/PHD-type domain containing protein.
chr12	Os12g0432750	1	13990533	13990808	Hypothetical conserved gene.
chr12	Os12g0437800	7	14411366	14412019	Similar to MPI.
chr12	Os12g0437932	4	14419117	14419367	Hypothetical conserved gene.
chr12	Os12g0438400	2	14453237	14456471	Hypothetical conserved gene.
chr12	Os12g0439700	1	14597050	14597355	Similar to RER1A protein.
chr12	Os12g0440400	1	14652534	14652969	Hypothetical protein.
chr12	Os12g0441700	23	14754353	14757981	Conserved hypothetical protein.
chr12	Os12g0443200	3	14883197	14884310	Hypothetical conserved gene.
chr12	Os12g0443600	2	14893170	14896224	Similar to UDP-glucose 6-dehydrogenase
chr12	Os12g0443800	16	14907829	14916625	Hypothetical conserved gene.
chr12	Os12g0444800	6	14974988	14977605	Similar to O-methyltransferase ZRP4.
chr12	Os12g0447466	10	15262097	15262959	Hypothetical conserved gene.
chr12	Os12g0447932	40	15262567	15268325	Conserved hypothetical protein.
chr12	Os12g0448400	11	15284375	15284934	Hypothetical protein.
chr12	Os12g0448650	8	15316001	15316708	Similar to AIR12.
chr12	Os12g0465700	4	16500219	16504887	Conserved hypothetical protein.
chr12	Os12g0466800	18	16565027	16565942	Hypothetical protein.
chr12	Os12g0467200	13	16582692	16585353	Protein of unknown function DUF1677 2C Oryza sativa family protein.
chr12	Os12g0468500	32	16702529	16707209	Similar to predicted protein.
chr12	Os12g0468600	175	16706568	16712920	Amidohydrolase 1 domain containing protein.
chr12	Os12g0470700	5	16872793	16874580	ATPase 2C AAA-type 2C core domain containing protein.
chr12	Os12g0472800	40	17009383	17011944	B repeat unit of collagen binding surface protein
chr12	Os12g0473900	20	17070764	17071549	Similar to Protease inhibitor/seed storage/LTP family protein.
chr12	Os12g0477400	3	17391342	17392627	No apical meristem
chr12	Os12g0479400	16	17571844	17577848	Similar to Auxin response factor 1.
chr12	Os12g0481100	6	17684802	17694915	Similar to cDNA clone:J033010C22 2C full insert sequence.
chr12	Os12g0482500	1	17779264	17782698	Protein of unknown function DUF593 domain containing protein.
chr12	Os12g0482700	4	17788701	17791111	Similar to L-galactose dehydrogenase.
chr12	Os12g0486600	2	18098260	18101695	Similar to J013164K19 2C full insert sequence
chr12	Os12g0486800	33	18111165	18113735	Hypothetical protein.
chr12	Os12g0486900	9	18116036	18120679	Similar to Protein kinase domain containing protein 2C expressed.
chr12	Os12g0487250	1	18172066	18173580	Arbuscular mycorrhizal specific marker 26.
chr12	Os12g0487832	14	18272198	18273207	Hypothetical conserved gene.
chr12	Os12g0488300	10	18287172	18287652	Similar to ribosomal protein L22.
chr12	Os12g0488900	7	18315197	18319057	Armadillo-like helical domain containing protein.
chr12	Os12g0489800	8	18369705	18373748	Similar to NBS-LRR protein
chr12	Os12g0490000	2	18383645	18385277	Hypothetical conserved gene.
chr12	Os12g0490100	6	18388482	18391056	Pentatricopeptide repeat domain containing protein.
chr12	Os12g0490566	2	18412383	18413445	Similar to embryo-sac basal-endosperm-layer embryo-surrounding-region2.
chr12	Os12g0493900	3	18618482	18621147	Armadillo-like helical domain containing protein.
chr12	Os12g0503101	1	19194860	19199795	Hypothetical conserved gene.
chr12	Os12g0503200	1	19199289	19205182	Similar to TRANSLATION INITIATION FACTOR IF-2P.
chr12	Os12g0504900	1	19332572	19333201	Glucoamylase 2C putatuve domain containing protein.
chr12	Os12g0506700	1	19434492	19438009	Carbamoyl phosphate synthetase 2C large subunit 2C ATP-binding domain containing protein.
chr12	Os12g0507000	8	19452186	19456650	Similar to Molybdopterin biosynthesis CNX2 protein
chr12	Os12g0507600	1	19493189	19494900	Conserved hypothetical protein.
chr12	Os12g0516200	11	20058131	20059545	Conserved hypothetical protein.
chr12	Os12g0516300	3	20061564	20066924	NB-ARC domain containing protein.
chr12	Os12g0516900	2	20084212	20088112	Cyclin-like F-box domain containing protein.
chr12	Os12g0546800	1	22089434	22091864	Alpha expansin 26.
chr12	Os12g0547500	121	22126680	22133291	Kinesin 2C motor region domain containing protein.
chr12	Os12g0547600	45	22133336	22136891	Calmodulin binding protein-like domain containing protein.
chr12	Os12g0548300	3	22182885	22189908	Similar to Nucleoside diphosphate kinase II 2C chloroplast precursor

chr12	Os12g0548501	6	22197957	22198462	Similar to CI2C.
chr12	Os12g0548800	8	22213958	22218978	Armadillo-type fold domain containing protein.
chr12	Os12g0551200	9	22328237	22334839	Armadillo-like helical domain containing protein.
chr12	Os12g0552575	2	22447830	22449052	Hypothetical protein.
chr12	Os12g0552600	1	22447769	22449195	Similar to MATE efflux family protein.
chr12	Os12g0552800	1	22466362	22469274	Conserved hypothetical protein.
chr12	Os12g0554400	1	22518982	22529230	Tetratricopeptide-like helical domain containing protein.
chr12	Os12g0555200	3	22591681	22593505	Similar to Probenazole-inducible protein PBZ1.
chr12	Os12g0555300	9	22595531	22596804	Similar to Pathogenesis-related protein PR-10a.
chr12	Os12g0555400	1	22597827	22598994	Similar to Raffinose synthase protein.
chr12	Os12g0555500	3	22600049	22601182	Probenazole-inducible protein PBZ1.
chr12	Os12g0555600	4	22602934	22607307	Cellulose synthase-like protein 2C Cell wall biosynthesis
chr12	Os12g0556200	27	22628910	22632952	Calmodulin binding protein-like family protein.
chr12	Os12g0556300	21	22635834	22640197	Calmodulin binding protein-like family protein.
chr12	Os12g0556500	12	22648120	22656258	Calmodulin binding protein-like family protein.
chr12	Os12g0556600	7	22656374	22662598	CTP synthase family protein.
chr12	Os12g0557400	7	22710855	22713349	Conserved hypothetical protein.
chr12	Os12g0557900	1	22731572	22732467	Similar to F-box domain containing protein.
chr12	Os12g0558366	7	22773516	22774906	Similar to F-box domain containing protein.
chr12	Os12g0559000	2	22825636	22827555	Hypothetical conserved gene.
chr12	Os12g0559100	2	22848617	22850629	Conserved hypothetical protein.
chr12	Os12g0559200	21	22854591	22860198	Lipoxygenase
chr12	Os12g0559400	4	22872891	22875304	Similar to Leucine Rich Repeat family protein.
chr12	Os12g0559600	3	22875416	22876184	Similar to Resistance protein RGA2.
chr12	Os12g0560500	1	22948747	22951075	Pseudouridine synthase domain containing protein.
chr12	Os12g0560700	3	22954078	22959404	Similar to PROLIFERA protein.
chr12	Os12g0561200	2	22972925	22976141	Similar to PAPA-1-like conserved region family protein 2C expressed.
chr12	Os12g0561500	1	23000258	23000963	Pectinesterase inhibitor domain containing protein.
chr12	Os12g0561750	16	23009303	23012933	Similar to ferroportin protein family.
chr12	Os12g0561900	10	23015929	23017657	Similar to UDP-glucoronosyl and UDP-glucosyl transferase family protein 2C expressed.
chr12	Os12g0562100	6	23030950	23035532	Major facilitator superfamily 2C general substrate transporter domain containing protein.
chr12	Os12g0563000	6	23091540	23094141	Phospholipid/glycerol acyltransferase domain containing protein.
chr12	Os12g0563200	3	23097021	23097600	Similar to Lozenge protein.
chr12	Os12g0563300	9	23099865	23100742	Hypothetical conserved gene.
chr12	Os12g0563400	9	23102049	23105182	Rhodanese-like domain containing protein.
chr12	Os12g0563700	29	23118859	23122699	Pectin lyase fold domain containing protein.
chr12	Os12g0564100	10	23135061	23137352	Similar to Myb-like DNA-binding domain containing protein 2C expressed.
chr12	Os12g0566000	8	23248824	23253304	Similar to Boron transporter.
chr12	Os12g0566200	6	23271514	23272915	Conserved hypothetical protein.
chr12	Os12g0566300	4	23275214	23279087	Similar to ATP citrate lyase beta
chr12	Os12g0566500	5	23290875	23292674	Conserved hypothetical protein.
chr12	Os12g0566800	5	23302307	23306305	Ion channel regulatory protein 2C UNC-93 domain containing protein.
chr12	Os12g0566900	14	23307980	23315465	Protein of unknown function DUF895 2C eukaryotic domain containing protein.
chr12	Os12g0567500	32	23338183	23344857	Protein kinase 2C catalytic domain domain containing protein.
chr12	Os12g0567700	1	23349996	23351824	Similar to 60S ribosomal protein L2
chr12	Os12g0568100	27	23376363	23378129	Similar to Zinc knuckle family protein 2C expressed.
chr12	Os12g0573900	7	23674545	23677352	Conserved hypothetical protein.
chr12	Os12g0574000	8	23687585	23691107	Amino acid transporter 2C transmembrane domain containing protein.
chr12	Os12g0574400	36	23703442	23712905	Similar to BPM.
chr12	Os12g0574800	13	23725985	23729782	Conserved hypothetical protein.
chr12	Os12g0574900	6	23729946	23734874	Similar to Asparagine synthase family protein.
chr12	Os12g0576300	1	23794337	23797952	Conserved hypothetical protein.
chr12	Os12g0577100	8	23842441	23845218	Nucleotide-binding 2C alpha-beta plait domain containing protein.
chr12	Os12g0577200	2	23850905	23852346	Conserved hypothetical protein.
chr12	Os12g0577700	7	23885845	23888835	Similar to Protein kinase domain containing protein 2C expressed.
chr12	Os12g0577800	3	23892674	23898184	Protein of unknown function DUF863 2C plant family protein.
chr12	Os12g0582666	5	24180594	24182306	Cytochrome P450 family protein.
chr12	Os12g0586600	4	24485299	24490983	Similar to Plasma membrane Ca2+-ATPase.
chr12	Os12g0624700	8	26684734	26685828	Protein of unknown function DUF1677 2C Oryza sativa domain containing protein.
chr12	Os12g0626100	5	26759757	26762339	Similar to HEAT-like.
chr12	Os12g0626150	5	26763811	26764359	Conserved hypothetical protein.
chr12	Os12g0626300	7	26770732	26779064	Similar to MRNA 2C complete cds 2C clone: RAFL24-07-B09.
chr12	Os12g0626500	1	26793127	26793851	Similar to late embryogenesis abundant protein D-34.
chr12	Os12g0628100	17	26888810	26890166	Similar to Actin-depolymerizing factor 6
chr12	Os12g0628300	15	26895057	26896260	Conserved hypothetical protein.
chr12	Os12g0628600	2	26913087	26913851	Similar to Thaumatin-like pathogenesis-related protein 3 precursor.
chr12	Os12g0629700	4	26946904	26947698	Similar to Thaumatin-like protein precursor.
chr12	Os12g0630200	1	26960487	26961383	Thaumatin 2C pathogenesis-related family protein.
chr12	Os12g0631100	3	27022013	27025203	Small GTPase 2C Storage protein trafficking
chr12	Os12g0631200	8	27025315	27029985	Zinc finger 2C C6HC-type domain containing protein.
chr12	Os12g0631600	6	27046194	27049741	Conserved hypothetical protein.
chr12	Os12g0631800	7	27051976	27056972	Similar to predicted protein.
chr12	Os12g0632700	93	27094649	27099336	Malate dehydrogenase 2C glyoxysomal precursor
chr12	Os12g0632800	48	27109127	27112150	Protein kinase 2C catalytic domain domain containing protein.
chr12	Os12g0633200	1	27132446	27134705	Erg28-like family protein.
chr12	Os12g0633400	6	27145970	27147649	Similar to Pathogenesis-related protein PR-1 precursor.
chr12	Os12g0633501	1	27151981	27152307	Similar to MYB36
chr12	Os12g0633700	2	27165898	27167702	Similar to GDP dissociation inhibitor protein OsGDI1.
chr12	Os12g0633800	2	27168720	27169628	Similar to Oxidoreductase 2C short chain dehydrogenase/reductase family protein 2C expressed.
chr12	Os12g0634600	1	27205497	27206108	Conserved hypothetical protein.
chr12	Os12g0634700	2	27209219	27209842	Similar to glycine-rich protein.
chr12	Os12g0635400	4	27238568	27239382	VQ domain containing protein.

Pedigree-based genome re-sequencing reveals genetic variation patterns of elite backbone varieties during modern rice improvement
Xingfei Zheng, Lanzhi Li, Fan Liang, Changjun Tan, Shuzhu Tang, Sibin Yu, Ying Diao, Shuangcheng Li,and Zhongli Hu

Supplementary Table S18 Genes with signature CDS SNPs during HHZ pedigree breeding

Chromosome	Gene ID	Signature SNPs number	Start positon	End position	Function
chr01	Os01g0110500	13	554025	558015	Protein kinase 2C core domain containing protein.
chr01	Os01g0110600	10	554334	557257	Hypothetical protein.
chr01	Os01g0115600	11	843530	847399	Similar to LRK14.
chr01	Os01g0117200	2	969340	973327	Similar to ARK protein
chr01	Os01g0117600	4	994712	997262	Protein kinase 2C catalytic domain domain containing protein.
chr01	Os01g0130900	2	1712137	1712947	Conserved hypothetical protein.
chr01	Os01g0132800	2	1813011	1817601	Peptidyl-tRNA hydrolase family protein.
chr01	Os01g0139200	4	2084490	2087203	Octicosapeptide/Phox/Bem1p domain containing protein.
chr01	Os01g0143300	7	2357871	2360437	Similar to Glycine-rich protein
chr01	Os01g0143400	22	2361799	2363814	Protein of unknown function DUF594 domain containing protein.
chr01	Os01g0143700	4	2372323	2374066	Similar to Mitochondrial glycoprotein.
chr01	Os01g0143800	20	2374541	2376761	Mitochondrial glycoprotein family protein.
chr01	Os01g0144000	29	2378825	2382584	Conserved hypothetical protein.
chr01	Os01g0144340	26	2391464	2397295	Similar to Actin-related protein 5.
chr01	Os01g0144380	32	2393483	2398292	Hypothetical gene.
chr01	Os01g0144500	16	2400945	2402336	Ubiquitin fusion degradation protein UFD1 domain containing protein.
chr01	Os01g0144600	11	2403276	2405947	Conserved hypothetical protein.
chr01	Os01g0144900	66	2416643	2418825	Hypothetical protein.
chr01	Os01g0145000	18	2422347	2422682	Hypothetical conserved gene.
chr01	Os01g0145200	48	2434793	2438772	Protein of unknown function DUF3681 domain containing protein.
chr01	Os01g0145600	63	2453742	2456495	Conserved hypothetical protein.
chr01	Os01g0146600	2	2530890	2531390	Conserved hypothetical protein.
chr01	Os01g0146801	2	2541950	2542484	Conserved hypothetical protein.
chr01	Os01g0147400	2	2570072	2571010	Uncharacterised domain XH domain containing protein.
chr01	Os01g0147600	5	2573049	2577415	Conserved hypothetical protein.
chr01	Os01g0147700	13	2579565	2583575	Region of unknown function XH domain containing protein.
chr01	Os01g0147800	22	2584986	2587547	Domain of unknown function DUF547 domain containing protein.
chr01	Os01g0147850	6	2585294	2585668	Hypothetical protein.
chr01	Os01g0147900	80	2589835	2593568	Triosephosphate isomerase 2C cytosolic
chr01	Os01g0148000	5	2599732	2603593	Similar to predicted protein.
chr01	Os01g0148050	6	2605614	2612731	Hypothetical conserved gene.
chr01	Os01g0148400	12	2632618	2637829	Brix domain containing protein.
chr01	Os01g0148500	22	2641000	2644665	Protein of unknown function DUF1677 2C Oryza sativa family protein.
chr01	Os01g0149350	10	2669251	2674068	NB-ARC domain containing protein.
chr01	Os01g0149400	4	2674414	2674683	Hypothetical protein.
chr01	Os01g0149500	8	2683222	2686364	Similar to Resistance gene analog PIC23
chr01	Os01g0149700	80	2688460	2692024	Hypothetical conserved gene.
chr01	Os01g0149750	76	2688638	2691961	Hypothetical protein.
chr01	Os01g0149900	19	2699128	2703491	Conserved hypothetical protein.
chr01	Os01g0150000	173	2707624	2711422	Similar to Synaptic glycoprotein SC2.
chr01	Os01g0150100	129	2713300	2717425	Similar to Geranylgeranyltransferase type I beta subunit.
chr01	Os01g0150200	24	2717836	2721203	Protein-tyrosine phosphatase-like 2C PTPLA domain containing protein.
chr01	Os01g0150400	28	2722605	2726163	Similar to 3-hydroxyacyl-CoA dehydratase PASTICCINO 2A.
chr01	Os01g0150500	15	2727751	2736707	Similar to 3-hydroxyacyl-CoA dehydratase PASTICCINO 2A.
chr01	Os01g0150800	17	2742507	2747439	Protein-tyrosine phosphatase-like 2C PTPLA domain containing protein.
chr01	Os01g0151500	2	2783189	2790377	Similar to GGT4
chr01	Os01g0151700	3	2800516	2803701	Similar to Short-chain dehydrogenase Tic32.
chr01	Os01g0151750	2	2801825	2802125	Hypothetical conserved gene.
chr01	Os01g0152000	96	2810313	2815017	Protein kinase 2C core domain containing protein.
chr01	Os01g0152050	134	2811498	2819553	Hypothetical protein.
chr01	Os01g0152100	8	2815148	2816554	F-box domain 2C Skp2-like domain containing protein.
chr01	Os01g0152200	24	2817650	2818897	F-box domain 2C Skp2-like domain containing protein.
chr01	Os01g0152600	66	2830325	2833623	Protein kinase 2C catalytic domain domain containing protein.
chr01	Os01g0152687	39	2835281	2836233	Serine-threonine/tyrosine-protein kinase domain containing protein.
chr01	Os01g0152700	4	2837892	2838171	Similar to Histone H2B
chr01	Os01g0152800	80	2839934	2843512	Protein kinase 2C core domain containing protein.
chr01	Os01g0152850	74	2840037	2843293	Hypothetical protein.
chr01	Os01g0152900	16	2844503	2845212	Similar to Histone H2B.1.
chr01	Os01g0152950	14	2847554	2848849	Cyclin-like F-box domain containing protein.
chr01	Os01g0152951	24	2849160	2851689	Hypothetical conserved gene.
chr01	Os01g0153000	20	2851954	2855225	Protein kinase 2C catalytic domain domain containing protein.
chr01	Os01g0153050	20	2851988	2855214	Hypothetical protein.
chr01	Os01g0153150	104	2859098	2864539	Hypothetical conserved gene.
chr01	Os01g0153250	24	2864276	2867341	Hypothetical conserved gene.
chr01	Os01g0153275	22	2864726	2867411	Hypothetical protein.
chr01	Os01g0153500	12	2885392	2886301	Protein of unknown function DUF674 domain containing protein.
chr01	Os01g0153550	10	2885449	2886073	Hypothetical protein.
chr01	Os01g0153700	14	2892920	2894707	Conserved hypothetical protein.
chr01	Os01g0154100	4	2907071	2907926	Protein of unknown function DUF674 domain containing protein.
chr01	Os01g0154500	2	2925935	2928765	Protein of unknown function DUF674 domain containing protein.
chr01	Os01g0155200	2	2962422	2964972	Similar to H0525E10.8 protein.
chr01	Os01g0168800	15	3516766	3519077	Alpha/beta hydrolase fold-1 domain containing protein.
chr01	Os01g0168850	8	3518372	3519175	Hypothetical gene.
chr01	Os01g0169800	11	3577473	3581859	Tryptophan aminotransferase 2C Indole-3-acetic acid
chr01	Os01g0170500	32	3633290	3639159	Protein of unknown function DUF239 2C plant domain containing protein.
chr01	Os01g0170600	14	3645508	3650691	Hypothetical conserved gene.
chr01	Os01g0170700	21	3652273	3656479	Thiol-activated cytolysin family protein.
chr01	Os01g0171200	26	3681798	3687409	Conserved hypothetical protein.
chr01	Os01g0171800	10	3696155	3699718	Conserved hypothetical protein.
chr01	Os01g0173600	2	3779634	3782002	Glyoxalase/bleomycin resistance protein/dioxygenase domain containing protein.
chr01	Os01g0174400	8	3825669	3829404	Hypothetical gene.
chr01	Os01g0174500	6	3827045	3830326	Metridin-like ShK toxin domain containing protein.
chr01	Os01g0182100	2	4306924	4309933	Protein of unknown function DUF1618 domain containing protein.
chr01	Os01g0182600	4	4329362	4338486	GIGANTEA protein.
chr01	Os01g0196300	4	5201862	5203996	Helix-loop-helix DNA-binding domain containing protein.
chr01	Os01g0196500	15	5213979	5216701	Prenylated rab acceptor PRA1 family protein.
chr01	Os01g0196600	2	5218573	5220335	Similar to 260 kDa major acidic fibroblast growth factor-stimulated phosphoprotein

chr01	Os01g0196800 24	5227044	5229966	Hypothetical protein.
chr01	Os01g0197450 10	5261189	5263102	Pentatricopeptide repeat domain containing protein.
chr01	Os01g0200500 17	5442866	5446246	MPPN family protein.
chr01	Os01g0201250 2	5529876	5531014	Similar to DNA-binding WRKY.
chr01	Os01g0201800 7	5579959	5585078	Hypothetical conserved gene.
chr01	Os01g0202800 6	5663306	5665832	Similar to NOD26-like membrane integral protein ZmNIP1-1.
chr01	Os01g0203300 8	5675669	5679548	Conserved hypothetical protein.
chr01	Os01g0205200 2	5764652	5766659	Pentatricopeptide repeat domain containing protein.
chr01	Os01g0205600 2	5773300	5774176	Crotonase 2C core domain containing protein.
chr01	Os01g0208000 2	5862946	5863577	Protein of unknown function DUF6 2C transmembrane domain containing protein.
chr01	Os01g0209400 2	5934586	5939516	Nucleotide-binding 2C alpha-beta plait domain containing protein.
chr01	Os01g0209700 28	5968819	5972489	Similar to GA 2-oxidase 5.
chr01	Os01g0209750 24	5969790	5972419	Hypothetical protein.
chr01	Os01g0210400 13	6005040	6007163	Protein of unknown function DUF616 domain containing protein.
chr01	Os01g0210600 10	6010862	6011818	Protein of unknown function DUF538 family protein.
chr01	Os01g0212100 4	6112811	6132696	DEAD-like helicase 2C N-terminal domain containing protein.
chr01	Os01g0217400 4	6402871	6405410	Conserved hypothetical protein.
chr01	Os01g0218032 5	6452871	6455988	Similar to Repressor of silencing 1.
chr01	Os01g0221300 7	6642665	6645159	Conserved hypothetical protein.
chr01	Os01g0223500 2	6765268	6765888	Similar to Esterase.
chr01	Os01g0223600 7	6766511	6769519	Similar to Pto kinase interactor 1-like protein.
chr01	Os01g0223700 10	6774202	6776856	Bulb-type lectin domain domain containing protein.
chr01	Os01g0223800 13	6781919	6786415	Hypothetical conserved gene.
chr01	Os01g0223900 6	6787094	6789730	Serine/threonine protein kinase-related domain containing protein.
chr01	Os01g0224000 2	6790903	6793320	Serine/threonine protein kinase-related domain containing protein.
chr01	Os01g0227100 27	7024297	7030591	Chlorophyll b reductase 2C Leaf senescence
chr01	Os01g0227350 7	7048457	7050422	Hypothetical protein.
chr01	Os01g0227500 3	7053100	7056373	Cytochrome P450 family protein.
chr01	Os01g0228300 8	7085873	7089172	Mpv17/PMP22 family protein.
chr01	Os01g0228400 13	7092034	7095315	Pentatricopeptide repeat domain containing protein.
chr01	Os01g0228450 10	7092967	7095091	Hypothetical protein.
chr01	Os01g0228500 8	7095864	7097381	Harpin-induced 1 domain containing protein.
chr01	Os01g0228800 91	7104701	7109095	Conserved hypothetical protein.
chr01	Os01g0229000 31	7121191	7123857	Similar to Transcription factor myb.
chr01	Os01g0229100 191	7125406	7128899	Similar to Eukaryotic translation initiation factor 3 subunit 6-interacting protein.
chr01	Os01g0229200 32	7129236	7137327	VHS domain containing protein.
chr01	Os01g0269000 15	9268690	9272912	Similar to Hydroxymethylglutaryl-CoA lyase.
chr01	Os01g0294500 2	10720396	10721623	Similar to Class III peroxidase 9.
chr01	Os01g0329300 5	12701744	12704532	Pectin lyase fold domain containing protein.
chr01	Os01g0329350 3	12702583	12704246	Hypothetical protein.
chr01	Os01g0350900 2	14012517	14019436	Similar to VIP2 protein.
chr01	Os01g0355500 16	14292115	14303191	Hypothetical conserved gene.
chr01	Os01g0356800 2	14414670	14435665	Domain of unknown function DUF3406 2C chloroplast translocase domain containing protein.
chr01	Os01g0361000 8	14702436	14705998	Conserved hypothetical protein.
chr01	Os01g0363000 12	14789737	14790461	Hypothetical protein.
chr01	Os01g0364450 2	14879651	14880271	Hypothetical gene.
chr01	Os01g0384300 2	16086670	16088900	Similar to RKF3
chr01	Os01g0390400 6	16480398	16494334	Similar to Met-10+ like family protein.
chr01	Os01g0513400 4	18124934	18128862	Protein of unknown function DUF789 family protein.
chr01	Os01g0525100 6	18780600	18781392	Conserved hypothetical protein.
chr01	Os01g0530500 4	19095631	19097491	Protein of unknown function DUF295 domain containing protein.
chr01	Os01g0531200 4	19115283	19122409	Phospholipase C 2C phosphatidylinositol-specific 2C Y domain domain containing protein.
chr01	Os01g0531400 4	19129984	19131278	Hypothetical conserved gene.
chr01	Os01g0531500 8	19135843	19139893	Dienelactone hydrolase domain containing protein.
chr01	Os01g0535650 6	19454577	19456091	Hypothetical protein.
chr01	Os01g0535400 36	19454154	19458225	Protein kinase 2C catalytic domain domain containing protein.
chr01	Os01g0542000 2	19972083	19973030	Similar to DNA-damage-repair/toleration protein DRT102.
chr01	Os01g0542100 48	19973271	19977472	Conserved hypothetical protein.
chr01	Os01g0562400 4	21381884	21389642	Peptidyl-prolyl cis-trans isomerase 2C FKBP-type 2C domain domain containing protein.
chr01	Os01g0564300 15	21525509	21549819	Peptidyl-prolyl cis-trans isomerase 2C FKBP-type domain containing protein.
chr01	Os01g0568950 44	21805322	21810098	Similar to barren inflorescence2.
chr01	Os01g0569200 141	21814796	21817900	Domain of unknown function DUF1618 domain containing protein.
chr01	Os01g0602800 9	23727763	23733053	Serine/threonine protein kinase-related domain containing protein.
chr01	Os01g0603300 4	23760347	23762387	Similar to MCB2 protein.
chr01	Os01g0604500 2	23810484	23811557	EF-Hand type domain containing protein.
chr01	Os01g0605500 3	23850922	23854484	Kinesin 2C motor region domain containing protein.
chr01	Os01g0606133 4	23886851	23888222	Hypothetical conserved gene.
chr01	Os01g0606166 4	23886901	23888225	Conserved hypothetical protein.
chr01	Os01g0607000 2	23914150	23914856	Glutelin family protein.
chr01	Os01g0607600 3	23947829	23962251	WD40 repeat domain containing protein.
chr01	Os01g0607900 8	23976021	23981901	Serine/threonine protein kinase-related domain containing protein.
chr01	Os01g0609200 17	24065207	24073621	Similar to Pleiotropic drug resistance protein 3.
chr01	Os01g0609700 4	24102742	24104421	Conserved hypothetical protein.
chr01	Os01g0609900 22	24107086	24115287	Similar to Pleiotropic drug resistance protein 4.
chr01	Os01g0610400 5	24145891	24148846	Acyl-CoA N-acyltransferase domain containing protein.
chr01	Os01g0610500 14	24151040	24154089	Yip1 domain containing protein.
chr01	Os01g0610600 5	24155649	24162572	Endonuclease/exonuclease/phosphatase domain containing protein.
chr01	Os01g0630101 4	25187267	25189478	Hypothetical protein.
chr01	Os01g0630200 4	25187250	25189517	Similar to oligopeptide transporter OPT family.
chr01	Os01g0634500 6	25439082	25440898	Similar to Laccase-2.
chr01	Os01g0646700 18	26060892	26064410	Similar to OSIGBa0105P02.4 protein.
chr01	Os01g0677500 2	27860530	27862131	Similar to ubiquitin-conjugating enzyme E2.
chr01	Os01g0680900 8	28028749	28031687	Similar to Dopamine beta-monooxygenase.
chr01	Os01g0680950 4	28030724	28032032	Hypothetical gene.
chr01	Os01g0689900 52	28482950	28496176	Hypothetical conserved gene.
chr01	Os01g0690800 15	28524105	28534387	Protein kinase 2C core domain containing protein.
chr01	Os01g0691000 56	28534821	28536497	Similar to Acidic endochitinase SE2 precursor
chr01	Os01g0691050 20	28536737	28537615	Protein kinase 2C catalytic domain domain containing protein.
chr01	Os01g0691200 6	28538115	28539701	Hypothetical protein.
chr01	Os01g0691300 4	28538881	28539369	Plant lipid transfer protein and hydrophobic protein 2C helical domain containing protein.
chr01	Os01g0692200 2	28572037	28572921	Conserved hypothetical protein.
chr01	Os01g0781200 2	33098082	33103904	Similar to Rust resistance protein.

chr01	Os01g0802900 2	34017557	34019231	Hypothetical protein.
chr01	Os01g0813700 7	34588835	34602432	Similar to Non-cyanogenic beta-glucosidase.
chr01	Os01g0833400 6	35686409	35687796	Conserved hypothetical protein.
chr01	Os01g0833700 4	35700437	35707701	Clathrin adaptor 2C mu subunit 2C C-terminal domain containing protein.
chr01	Os01g0833800 8	35709533	35713436	IQ calmodulin-binding region domain containing protein.
chr01	Os01g0834200 41	35732665	35743682	Similar to T-cell immune regulator 1 transcript variant 3
chr01	Os01g0834500 3	35760071	35761608	Similar to 40S ribosomal protein S23
chr01	Os01g0835000 5	35784466	35793928	Similar to predicted protein.
chr01	Os01g0846450 12	36353072	36358534	Hypothetical conserved gene.
chr01	Os01g0847600 6	36403442	36407379	Aldo/keto reductase family protein.
chr01	Os01g0848200 28	36430978	36440843	Similar to Delta 1-pyrroline-5-carboxylate synthetase
chr01	Os01g0848400 4	36445456	36449951	BEL1-type homeobox family 2C Seed shattering
chr01	Os01g0850100 19	36540164	36545196	Similar to Phosphatidic acid phosphatase-like protein.
chr01	Os01g0850550 12	36617104	36621116	Similar to Laccase-6.
chr01	Os01g0850700 34	36622829	36627662	Similar to Laccase-7.
chr01	Os01g0850800 3	36634331	36636717	Laccase-8.
chr01	Os01g0851100 4	36643577	36648018	GDP-fucose protein O-fucosyltransferase domain containing protein.
chr01	Os01g0851600 2	36666965	36668075	3-oxo-5-alpha-steroid 4-dehydrogenase 2C C-terminal domain containing protein.
chr01	Os01g0852900 20	36732836	36737403	Pentatricopeptide repeat domain containing protein.
chr01	Os01g0854651 4	36838524	36839070	Similar to Adenylate cyclase.
chr01	Os01g0855000 16	36863177	36871094	Similar to Glycerol-3-phosphate acyltransferase 6
chr01	Os01g0855200 10	36894607	36897206	Tetratricopeptide-like helical domain containing protein.
chr01	Os01g0871400 6	37789052	37790984	Hypothetical protein.
chr01	Os01g0871500 10	37790484	37792882	TGF-beta receptor 2C type I/II extracellular region family protein.
chr01	Os01g0872100 22	37821284	37826818	TGF-beta receptor 2C type I/II extracellular region family protein.
chr01	Os01g0872300 6	37828417	37829987	Similar to POT family protein.
chr01	Os01g0872350 30	37829568	37832278	Hypothetical protein.
chr01	Os01g0872400 38	37830130	37832381	Similar to POT family protein.
chr01	Os01g0872450 6	37832970	37833242	Similar to POT family protein.
chr01	Os01g0872500 26	37834199	37836889	Oligopeptide transporter domain containing protein.
chr01	Os01g0872533 13	37834234	37836571	Hypothetical protein.
chr01	Os01g0872700 26	37844994	37852790	Zinc finger 2C CCHC-type domain containing protein.
chr01	Os01g0872800 10	37853413	37857509	Similar to 3-phosphoinositide-dependent protein kinase-1
chr01	Os01g0873200 8	37866333	37868311	Similar to Amidophosphoribosyltransferase 2C chloroplast precursor
chr01	Os01g0873300 14	37869529	37870325	Conserved hypothetical protein.
chr01	Os01g0874100 4	37914774	37918962	Hypothetical conserved gene.
chr01	Os01g0874800 8	37955091	37959001	Similar to DNA polymerase I.
chr01	Os01g0875300 2	37982651	37985429	Similar to Universal stress protein family protein.
chr01	Os01g0875800 4	38012799	38014299	Protein of unknown function DUF266 2C plant family protein.
chr01	Os01g0878900 2	38144793	38146141	Similar to 4 2C5-DOPA dioxygenase extradiol-like protein.
chr01	Os01g0879100 14	38147082	38148018	Hypothetical protein.
chr01	Os01g0879000 10	38147023	38147798	Hypothetical protein.
chr01	Os01g0879400 3	38154147	38156987	Glycoside hydrolase 2C family 43 protein.
chr01	Os01g0881000 4	38228812	38237228	SNF2-related domain containing protein.
chr01	Os01g0881100 2	38238479	38240305	Epsin-like 2C N-terminal domain containing protein.
chr01	Os01g0881400 4	38257939	38261435	DNA repair metallo-beta-lactamase domain containing protein.
chr01	Os01g0881800 4	38273297	38274800	Conserved hypothetical protein.
chr01	Os01g0883400 4	38344272	38344937	Similar to PHD finger protein-like protein
chr01	Os01g0898400 9	39063700	39065530	Conserved hypothetical protein.
chr01	Os01g0898500 12	39066177	39068629	Similar to Patatin
chr01	Os01g0899200 2	39102277	39103501	Similar to dehydration-responsive family protein.
chr01	Os01g0899350 2	39114759	39115037	Similar to ATP synthase F0 subunit 9.
chr01	Os01g0899425 2	39121870	39122187	Similar to ribulose 1 2C5-bisphosphate carboxylase/oxygenase large subunit.
chr01	Os01g0901600 11	39257838	39261362	Similar to 4-coumarate--CoA ligase-like 6.
chr01	Os01g0901700 8	39262881	39266565	Similar to emb2421
chr01	Os01g0901800 12	39266781	39269329	Similar to acid phosphatase/vanadium-dependent haloperoxidase related.
chr01	Os01g0901900 8	39270573	39274992	S1 2C RNA binding domain containing protein.
chr01	Os01g0902100 6	39276426	39281147	Similar to MRP-like ABC transporter.
chr01	Os01g0902200 12	39289364	39296595	Peptidase M3A and M3B 2C thimet/oligopeptidase F domain containing protein.
chr01	Os01g0902800 6	39312496	39315691	Similar to cDNA 2C clone: J090081K18 2C full insert sequence.
chr01	Os01g0905200 8	39408313	39410361	Exo70 exocyst complex subunit family protein.
chr01	Os01g0905300 2	39411052	39413189	Similar to Leucine zipper protein-like.
chr01	Os01g0906425 2	39465264	39465617	Hypothetical conserved gene.
chr01	Os01g0907400 5	39493173	39499938	Histone lysine demethylase 2C Stress-responsive H3K27me3
chr01	Os01g0907600 2	39501378	39502910	Cysteine endopeptidase.
chr01	Os01g0907900 4	39523638	39527194	Similar to Terminal ear1.
chr01	Os01g0914600 2	39849645	39851426	Pentatricopeptide repeat domain containing protein.
chr01	Os01g0917500 2	40021069	40023811	Hypothetical conserved gene.
chr01	Os01g0976900 63	43175447	43178814	Similar to kinase.
chr01	Os01g0976950 26	43177784	43178961	Hypothetical gene.
chr02	Os02g0127300 2	1423979	1424390	Hypothetical protein.
chr02	Os02g0158500 6	3187973	3190043	Uncharacterized plant-specific domain 01627 containing protein.
chr02	Os02g0158550 4	3188143	3189588	Hypothetical protein.
chr02	Os02g0158701 2	3196192	3196414	Similar to predicted protein.
chr02	Os02g0185766 2	4785252	4787028	Hypothetical protein.
chr02	Os02g0185900 2	4785324	4787163	Hypothetical conserved gene.
chr02	Os02g0188500 6	4908958	4911334	Ubiquitin domain containing protein.
chr02	Os02g0247600 10	8350331	8351592	Conserved hypothetical protein.
chr02	Os02g0257001 91	8856566	8860576	Hypothetical conserved gene.
chr02	Os02g0258300 8	8938133	8943482	Zinc finger 2C FYVE/PHD-type domain containing protein.
chr02	Os02g0286200 3	10758967	10762460	Lipase 2C class 3 family protein.
chr02	Os02g0287900 2	10857586	10859012	Cyclin-like F-box domain containing protein.
chr02	Os02g0288400 2	10880647	10886393	Similar to MRP-like ABC transporter.
chr02	Os02g0290000 14	10960662	10968059	Pentatricopeptide repeat domain containing protein.
chr02	Os02g0290600 11	10999890	11004650	Similar to prolyl oligopeptidase family protein.
chr02	Os02g0292600 2	11116177	11120334	Lipase 2C GDSL domain containing protein.
chr02	Os02g0292800 13	11126248	11127515	Protein of unknown function DUF599 family protein.
chr02	Os02g0293050 10	11126629	11127361	Hypothetical protein.
chr02	Os02g0293300 6	11151750	11156506	Similar to Gene X-like protein
chr02	Os02g0478450 2	16425253	16425567	Conserved hypothetical protein.
chr02	Os02g0503100 2	17805404	17808857	Similar to flavonoid 3-monooxygenase.
chr02	Os02g0510600 16	18252570	18256034	Heavy metal transport/detoxification protein domain containing protein.

chr02	Os02g0532800 8	19614070	19615072	7TM GPCR 2C rhodopsin-like domain containing protein.
chr02	Os02g0532900 42	19619243	19623214	Glycoside hydrolase 2C family 17 protein.
chr02	Os02g0533000 15	19623488	19628801	FeS cluster biogenesis domain containing protein.
chr02	Os02g0538700 4	19954579	19955654	Harpin-induced 1 domain containing protein.
chr02	Os02g0541150 2	20109198	20110897	Hypothetical protein.
chr02	Os02g0542100 18	20134804	20135382	Similar to Histone H3
chr02	Os02g0542500 44	20152453	20154121	Similar to NF-180.
chr02	Os02g0542600 40	20152543	20154023	Hypothetical protein.
chr02	Os02g0543100 21	20169587	20172428	Similar to predicted protein.
chr02	Os02g0543900 23	20227026	20235116	Similar to OSIGBa0159H11-OSIGBa0137A07.4 protein.
chr02	Os02g0596900 2	23177585	23182048	Actin/actin-like family protein.
chr02	Os02g0599151 2	23386353	23391753	Probable protein phosphatase 2C 17.
chr02	Os02g0600200 6	23465969	23468038	IQ calmodulin-binding region domain containing protein.
chr02	Os02g0603800 3	23653493	23659174	Similar to Isoprenoid biosynthesis-like protein
chr02	Os02g0608801 2	23893258	23894469	Lipase 2C GDSL domain containing protein.
chr02	Os02g0610000 4	23966075	23967661	Leucine-rich repeat domain containing protein.
chr02	Os02g0610700 4	23998075	24000801	Chlorophyll a/b binding protein domain domain containing protein.
chr02	Os02g0611200 15	24045244	24048294	S-adenosylmethionine decarboxylase 2C Polyamine biosynthesis 2C Abiotic stress
chr02	Os02g0611800 16	24080704	24084615	Similar to Hydroxyanthranilate hydroxycinnamoyltransferase 3.
chr02	Os02g0612200 2	24098151	24099882	NB-ARC domain containing protein.
chr02	Os02g0612600 2	24132135	24132736	Hypothetical protein.
chr02	Os02g0612800 12	24139771	24146705	AT hook 2C DNA-binding 2C conserved site domain containing protein.
chr02	Os02g0614700 2	24283189	24283680	B30.2/SPRY domain domain containing protein.
chr02	Os02g0614732 4	24283088	24285260	Hypothetical gene.
chr02	Os02g0615500 113	24327114	24331291	Protein kinase 2C core domain containing protein.
chr02	Os02g0615800 4	24350730	24354486	Protein kinase 2C core domain containing protein.
chr02	Os02g0617200 13	24482920	24487358	Conserved hypothetical protein.
chr02	Os02g0617300 6	24488770	24501970	Similar to OSIGBa0157A06.7 protein.
chr02	Os02g0618100 2	24566332	24568207	Similar to Glutaredoxin-C4 2C chloroplastic.
chr02	Os02g0618200 4	24569489	24572391	Signal transduction response regulator 2C receiver region domain containing protein.
chr02	Os02g0621100 4	24708074	24717284	Similar to Su
chr02	Os02g0695050 50	28555557	28556687	Leucine-rich repeat domain containing protein.
chr02	Os02g0694900 52	28552733	28557616	Protein kinase 2C catalytic domain domain containing protein.
chr02	Os02g0699824 8	28791379	28792577	Conserved hypothetical protein.
chr02	Os02g0699850 2	28793941	28795051	Hypothetical gene.
chr02	Os02g0699900 2	28794171	28795262	Bromodomain transcription factor containing protein.
chr02	Os02g0700000 11	28795583	28800683	EF-HAND 1 domain containing protein.
chr02	Os02g0700100 7	28802911	28805716	Similar to WD-repeat protein.
chr02	Os02g0700300 5	28808920	28811960	Similar to Phosphate starvation response regulator-like protein.
chr02	Os02g0701300 16	28863274	28866997	Conserved hypothetical protein.
chr02	Os02g0702400 2	28935436	28938889	Pectinacetylesterase family protein.
chr02	Os02g0706500 8	29199147	29204307	ATPase 2C AAA-type 2C core domain containing protein.
chr02	Os02g0706600 5	29206676	29208686	Similar to zinc finger homeodomain protein 1.
chr02	Os02g0706900 5	29224218	29226240	Alpha/beta hydrolase family protein.
chr02	Os02g0707200 2	29238147	29239269	Similar to Dof-like protein 34.
chr02	Os02g0709400 8	29341254	29357890	Similar to Dek1-calpain-like protein.
chr02	Os02g0711400 2	29471779	29472570	Conserved hypothetical protein.
chr02	Os02g0712000 14	29483433	29495702	Similar to DegP7
chr02	Os02g0712800 2	29523354	29527951	Conserved hypothetical protein.
chr02	Os02g0713500 8	29555642	29557498	F-box domain 2C cyclin-like domain containing protein.
chr02	Os02g0713900 19	29587196	29591875	Similar to 3-hydroxy-3-methylglutaryl-coenzyme A reductase 1.
chr02	Os02g0714801 2	29659062	29659686	Conserved hypothetical protein.
chr02	Os02g0720600 2	29892488	29894313	Peptidase A1 domain containing protein.
chr02	Os02g0721800 2	29950718	29956811	Similar to Phosphatidylinositol transfer-like protein IV.
chr02	Os02g0721901 2	29953414	29956021	Hypothetical protein.
chr02	Os02g0722800 5	30015998	30025935	WD40 repeat-like domain containing protein.
chr02	Os02g0724500 13	30105720	30109792	Similar to transporter-related.
chr02	Os02g0724600 5	30110288	30115478	Similar to predicted protein.
chr02	Os02g0729300 16	30352710	30356478	Zinc finger 2C CCHC retroviral-type domain containing protein.
chr02	Os02g0729350 10	30357071	30359162	Hypothetical conserved gene.
chr02	Os02g0729700 10	30381303	30383486	Similar to HAHB-7
chr02	Os02g0729801 6	30381327	30383436	Hypothetical protein.
chr02	Os02g0730000 14	30392547	30396729	Similar to Mitochondrial aldehyde dehydrogenase.
chr02	Os02g0730050 4	30392816	30395045	Zinc finger 2C RING-type domain containing protein.
chr02	Os02g0730100 6	30398551	30399887	Similar to Pre-mRNA processing ribonucleoprotein 2C binding region 3B NOSIC.
chr02	Os02g0730300 9	30418498	30423139	Similar to Potassium transporter HAK3p
chr02	Os02g0730800 5	30444949	30448020	Nucleotide-binding 2C alpha-beta plait domain containing protein.
chr02	Os02g0730775 5	30444816	30447866	Hypothetical protein.
chr02	Os02g0730900 2	30448000	30449510	Pentatricopeptide repeat domain containing protein.
chr02	Os02g0736100 6	30720856	30726454	Myosin II heavy chain-like family protein.
chr02	Os02g0739400 4	30899582	30901300	Similar to Avr9/Cf-9 rapidly elicited protein 231 precursor.
chr02	Os02g0739650 3	30918850	30920070	Hypothetical gene.
chr02	Os02g0739900 10	30930149	30934255	Similar to microtubule-associated protein TORTIFOLIA1.
chr02	Os02g0740300 13	30958609	30965222	ATPase 2C AAA-type 2C core domain containing protein.
chr02	Os02g0740600 4	30974228	30974781	Conserved hypothetical protein.
chr02	Os02g0740700 6	30980995	30982317	Peptidase M10A and M12B 2C matrixin and adamalysin family protein.
chr02	Os02g0740800 8	30986861	30988272	Hypothetical protein.
chr02	Os02g0740900 6	30987024	30988403	WD-40 repeat containing protein.
chr02	Os02g0741000 12	30989383	30989940	Glutaredoxin domain containing protein.
chr02	Os02g0741300 12	31003974	31008671	Glycoside hydrolase 2C family 47 protein.
chr02	Os02g0741400 9	31009240	31011527	Conserved hypothetical protein.
chr02	Os02g0741700 10	31038552	31040540	Conserved hypothetical protein.
chr02	Os02g0742000 5	31064064	31071218	Transcription elongation factor 2C TFIIS/CRSP70 2C N-terminal domain containing protein.
chr02	Os02g0742100 14	31080741	31083917	Conserved hypothetical protein.
chr02	Os02g0742700 5	31111841	31114590	Conserved hypothetical protein.
chr02	Os02g0742800 8	31120649	31124019	Conserved hypothetical protein.
chr02	Os02g0743200 2	31145280	31146137	Conserved hypothetical protein.
chr02	Os02g0743400 6	31158979	31161998	Auxin transport protein REH1.
chr02	Os02g0805800 4	34372417	34378347	Aminoglycoside phosphotransferase domain containing protein.
chr03	Os03g0103600 2	254279	255121	Hypothetical conserved gene.
chr03	Os03g0130200 22	1706399	1711755	Conserved hypothetical protein.
chr03	Os03g0135800 9	1990495	1995980	Conserved hypothetical protein.

chr03	Os03g0143700 5	2415606	2419564	Protein of unknown function DUF3741 domain containing protein.
chr03	Os03g0144100 2	2459016	2459570	Hypothetical genes.
chr03	Os03g0170300 3	3763827	3767153	3-dehydroquinate synthase family protein.
chr03	Os03g0170701 2	3786235	3787068	Hypothetical gene.
chr03	Os03g0204100 4	5458099	5462322	Actin-binding FH2 domain containing protein.
chr03	Os03g0204300 18	5466785	5468445	Conserved hypothetical protein.
chr03	Os03g0204366 8	5467138	5467740	Conserved hypothetical protein.
chr03	Os03g0204600 4	5476440	5477287	Conserved hypothetical protein.
chr03	Os03g0205400 9	5508753	5516754	Similar to XPG I-region family protein 2C expressed.
chr03	Os03g0225100 4	6545153	6546478	Serine/threonine protein kinase domain containing protein.
chr03	Os03g0225150 2	6545960	6546361	Hypothetical protein.
chr03	Os03g0233200 3	7066551	7067714	Similar to Thaumatin-like protein 3B 23251-22305.
chr03	Os03g0233251 2	7066564	7067291	Hypothetical protein.
chr03	Os03g0251700 2	7998159	8000400	Similar to Protein kinase domain containing protein 2C expressed.
chr03	Os03g0269900 2	9011629	9016463	Protein of unknown function DUF604 family protein.
chr03	Os03g0273200 4	9167619	9170326	Similar to Laccase
chr03	Os03g0275300 2	9296962	9300002	Hypothetical conserved gene.
chr03	Os03g0356526 4	13679030	13679794	Hypothetical conserved gene.
chr03	Os03g0405500 18	16590192	16595911	Similar to PDI-like protein.
chr03	Os03g0430500 2	18053010	18059557	Peptidase S8/S53 2C subtilisin/kexin/sedolisin domain containing protein.
chr03	Os03g0430575 2	18053185	18055328	Hypothetical protein.
chr03	Os03g0432666 4	18204733	18205753	Hypothetical gene.
chr03	Os03g0441500 2	18686899	18689372	ABC transporter-like domain containing protein.
chr03	Os03g0576200 8	21060638	21064968	Potassium transporter 21.
chr03	Os03g0577500 14	21117886	21123379	Similar to Avr9 elicitor response-like protein.
chr03	Os03g0582900 12	21457124	21458020	Hypothetical gene.
chr03	Os03g0586300 4	21622958	21628739	Reticulon family protein.
chr03	Os03g0586800 34	21665280	21673555	Similar to Lysyl-tRNA synthetase
chr03	Os03g0587200 4	21687370	21693162	Kinesin 2C motor region domain containing protein.
chr03	Os03g0588200 18	21733006	21737915	Frigida-like family protein.
chr03	Os03g0588400 6	21745957	21747104	Similar to HT1 protein kinase.
chr03	Os03g0588600 14	21748596	21751686	Frigida-like family protein.
chr03	Os03g0589100 2	21782331	21783161	Conserved hypothetical protein.
chr03	Os03g0589300 36	21784848	21791170	Ovarian tumour 2C otubain domain containing protein.
chr03	Os03g0589400 4	21792860	21793702	Hypothetical conserved gene.
chr03	Os03g0590700 17	21881938	21885330	NADH dehydrogenase [ubiquinone] 1 alpha subcomplex assembly factor 3 domain containing protein.
chr03	Os03g0591300 7	21910439	21913786	Helix-loop-helix DNA-binding domain containing protein.
chr03	Os03g0591801 11	21960011	21963975	Hypothetical protein.
chr03	Os03g0594100 2	22083215	22086165	Cytochrome P450 family protein.
chr03	Os03g0594250 2	22083544	22085792	Hypothetical protein.
chr03	Os03g0594700 4	22110421	22116473	Conserved hypothetical protein.
chr03	Os03g0594900 4	22123330	22125585	Cytochrome P450 family protein.
chr03	Os03g0596900 9	22219267	22227186	GTP-binding signal recognition particle SRP54 2C G-domain containing protein.
chr03	Os03g0598002 24	22271544	22272752	Hypothetical gene.
chr03	Os03g0653900 2	25463483	25464304	Hypothetical conserved gene.
chr03	Os03g0667300 4	26276422	26277614	Similar to Metal transport protein.
chr03	Os03g0687200 15	27431846	27436591	Conserved hypothetical protein.
chr03	Os03g0690500 4	27580909	27582211	Similar to Oxidoreductase 2C 2OG-Fe oxygenase family protein 2C expressed.
chr03	Os03g0693000 2	27736390	27741374	Similar to predicted protein.
chr03	Os03g0694600 4	27814133	27815913	Hypothetical protein.
chr03	Os03g0695400 14	27846706	27847581	Ankyrin repeat containing protein.
chr03	Os03g0697200 2	27943411	27945040	Similar to carboxy-lyase.
chr03	Os03g0697833 4	27983768	27986675	Conserved hypothetical protein.
chr03	Os03g0700700 52	28106903	28113300	Similar to Lipoxygenase
chr03	Os03g0700750 2	28112258	28113049	Hypothetical gene.
chr03	Os03g0701400 30	28151940	28161762	Similar to Pentatricopeptide repeat protein PPR868-14.
chr03	Os03g0701600 7	28164813	28167428	Conserved hypothetical protein.
chr03	Os03g0701700 10	28169837	28174078	Similar to Ethylene receptor.
chr03	Os03g0701800 41	28181543	28186115	Similar to Phosphatidylinositol-4-phosphate 5-kinase 1.
chr03	Os03g0701900 15	28186203	28190115	Similar to Pinin/SDK/memA/ protein conserved region containing protein.
chr03	Os03g0702900 2	28239396	28240049	Conserved hypothetical protein.
chr03	Os03g0708600 4	28568347	28571308	DEAD-like helicase 2C N-terminal domain containing protein.
chr03	Os03g0710000 12	28631644	28633255	Protein of unknown function DUF292 2C eukaryotic domain containing protein.
chr03	Os03g0710100 22	28633286	28643842	Similar to Protein kinase domain containing protein 2C expressed.
chr03	Os03g0710900 3	28671127	28678954	Similar to CCAAT displacement protein-related / CDP-related.
chr03	Os03g0712000 2	28766711	28767454	Similar to Lipase family protein.
chr03	Os03g0712400 12	28795423	28798180	Similar to atypical receptor-like kinase MARK.
chr03	Os03g0712500 2	28798419	28798833	Hypothetical protein.
chr03	Os03g0719850 8	29180902	29183157	Similar to Protein kinase 2.
chr03	Os03g0719900 22	29185055	29190361	Similar to Peptide transporter 1.
chr03	Os03g0720300 43	29200210	29208674	Similar to Glutamate decarboxylase isozyme 1
chr03	Os03g0721200 22	29260530	29272230	Armadillo-like helical domain containing protein.
chr03	Os03g0721300 12	29272374	29274638	Protein of unknown function DUF946 2C plant family protein.
chr03	Os03g0722000 8	29292555	29294090	Hyccin domain containing protein.
chr03	Os03g0722100 6	29299399	29302083	SANT 2C eukarya domain containing protein.
chr03	Os03g0722200 2	29302852	29303463	Hypothetical protein.
chr03	Os03g0728500 2	29741931	29745253	Forkhead-associated domain containing protein.
chr03	Os03g0728600 2	29745719	29747861	Conserved hypothetical protein.
chr03	Os03g0728800 6	29766132	29774115	Similar to RNA helicase
chr03	Os03g0735601 2	30149905	30150520	Hypothetical gene.
chr03	Os03g0735900 2	30151747	30153756	Phospholipid/glycerol acyltransferase domain containing protein.
chr03	Os03g0770900 8	31942581	31945141	Conserved hypothetical protein.
chr03	Os03g0786900 15	32679834	32691068	Tetratricopeptide-like helical domain containing protein.
chr03	Os03g0788600 4	32757036	32760505	FAS1 domain domain containing protein.
chr03	Os03g0796301 2	33137576	33138066	Similar to WW domain-binding protein 11.
chr03	Os03g0796501 4	33143530	33146079	Similar to 60S ribosomal protein L4
chr03	Os03g0811550 2	33994325	33995305	Protein of unknown function DUF617 2C plant domain containing protein.
chr03	Os03g0811600 4	33995633	33998631	Similar to Peptidyl prolyl isomerase H.
chr03	Os03g0811900 8	34006999	34009631	Similar to ADP-ribosylation factor.
chr03	Os03g0812000 10	34010918	34015642	DNA topoisomerase 2C type IIA 2C subunit A or C-terminal domain containing protein.
chr03	Os03g0812200 42	34031452	34034030	Zinc finger 2C RING/FYVE/PHD-type domain containing protein.
chr03	Os03g0812300 6	34034393	34035101	Hypothetical gene.

chr03	Os03g0812400 54	34036246	34037092	EF-Hand type domain containing protein.
chr03	Os03g0813200 7	34071601	34072369	Similar to prohibitin1.
chr03	Os03g0816150 6	34207176	34207668	Conserved hypothetical protein.
chr03	Os03g0823100 4	34554649	34556794	Major facilitator superfamily protein.
chr03	Os03g0823301 2	34560598	34560851	Hypothetical conserved gene.
chr03	Os03g0824300 6	34622463	34625204	Hypothetical conserved gene.
chr03	Os03g0828800 12	34810077	34813240	Serine/threonine protein kinase-related domain containing protein.
chr03	Os03g0836400 10	35145634	35146689	Harpin-induced 1 domain containing protein.
chr03	Os03g0836500 10	35147311	35149802	Conserved hypothetical protein.
chr03	Os03g0836800 19	35161307	35163174	Similar to IAA-amino acid hydrolase 1
chr03	Os03g0839100 4	35259526	35264535	Ubiquinone biosynthesis hydroxylase 2C UbiH/UbiF/VisC/COQ6 domain containing protein.
chr03	Os03g0839700 6	35303778	35305155	Hypothetical conserved gene.
chr03	Os03g0839800 14	35309856	35310752	Protein of unknown function DUF688 domain containing protein.
chr03	Os03g0839900 32	35312453	35317359	Serine/threonine protein kinase-related domain containing protein.
chr03	Os03g0849600 102	35736085	35741607	COG complex component 2C COG2 family protein.
chr03	Os03g0849900 22	35766964	35768997	Nucleotide-diphospho-sugar transferase domain containing protein.
chr03	Os03g0850900 2	35820760	35821517	Similar to Chemocyanin precursor
chr03	Os03g0851100 13	35826423	35830202	Similar to Translational elongation factor EF-TuM.
chr03	Os03g0851900 34	35868698	35875491	ATPase 2C AFG1-like domain containing protein.
chr03	Os03g0852000 18	35877545	35882905	Similar to Pentatricopeptide
chr03	Os03g0852300 10	35890202	35894172	Histone-fold domain containing protein.
chr03	Os03g0852400 6	35895803	35896803	Conserved hypothetical protein.
chr03	Os03g0852600 3	35899615	35901119	Protein of unknown function DUF565 family protein.
chr03	Os03g0852800 4	35907129	35908400	Similar to NPC4
chr03	Os03g0852900 11	35908721	35911694	Similar to Cleavage and polyadenylation specificity factor 2C 73 kDa subunit
chr03	Os03g0853700 8	35965090	35968402	GC-rich sequence DNA-binding factor-like family protein.
chr03	Os03g0854500 14	35989130	35992565	Similar to Heat shock transcription factor 31
chr03	Os03g0854600 16	35994445	35995919	Concanavalin A-like lectin/glucanase domain containing protein.
chr04	Os04g0196300 4	6567884	6568626	Hypothetical protein.
chr04	Os04g0208400 2	7254765	7256866	Cyclin-like F-box domain containing protein.
chr04	Os04g0208425 2	7254929	7256881	Hypothetical protein.
chr04	Os04g0295500 17	12997753	12999822	Conserved hypothetical protein.
chr04	Os04g0297800 42	13154099	13155441	Similar to OSIGBa0105O19.3 protein.
chr04	Os04g0315350 4	14352976	14354418	Similar to cytokinin-O-glucosyltransferase 2.
chr04	Os04g0508200 2	25389649	25395401	Isocitrate/isopropylmalate dehydrogenase domain containing protein.
chr04	Os04g0545700 2	27336576	27337862	Transferase family protein.
chr04	Os04g0546100 10	27345290	27347008	Similar to OSIGBa0101C23.10 protein.
chr04	Os04g0546300 2	27353820	27357710	Similar to GMPK2 3DPROTEIN kinase.
chr04	Os04g0550400 6	27551423	27559580	Helix-turn-helix 2C AraC type 2C subdomain 2 domain containing protein.
chr04	Os04g0550500 8	27563230	27566440	Similar to N-acetyl glutamate kinase 2.
chr04	Os04g0550600 2	27567824	27570449	Carotenoid oxygenase family protein.
chr04	Os04g0550800 6	27575661	27576994	Major intrinsic protein family protein.
chr04	Os04g0550833 2	27576034	27576647	Hypothetical protein.
chr04	Os04g0551500 4	27614776	27618001	SBP domain containing protein.
chr04	Os04g0607400 4	30716170	30722227	Conserved hypothetical protein.
chr04	Os04g0613900 14	31128314	31133415	Similar to Potassium transporter 18.
chr04	Os04g0613950 4	31128547	31129579	Hypothetical protein.
chr04	Os04g0614300 4	31161634	31163557	Hypothetical conserved gene.
chr04	Os04g0614500 38	31169012	31174731	Pyridoxal phosphate-dependent transferase 2C major region 2C subdomain 1 domain containing protein.
chr04	Os04g0614600 24	31176650	31182430	Similar to Viroid RNA-binding protein
chr04	Os04g0616500 8	31299841	31307729	Similar to H0525G02.11 protein.
chr04	Os04g0618400 4	31410510	31410971	Similar to H0313F03.13 protein.
chr04	Os04g0618700 35	31420801	31425092	Protein kinase 2C core domain containing protein.
chr04	Os04g0618800 28	31422225	31425278	Hypothetical protein.
chr04	Os04g0618950 4	31434880	31435978	Similar to H0114G12.1 protein.
chr04	Os04g0619000 26	31439595	31443397	Similar to L1332.9 protein.
chr04	Os04g0619300 7	31458951	31462013	Similar to H0114G12.4 protein.
chr04	Os04g0619400 11	31463105	31466291	Protein kinase 2C core domain containing protein.
chr04	Os04g0619500 2	31474399	31477315	Ovarian tumour 2C otubain domain containing protein.
chr04	Os04g0619600 27	31478250	31481997	Similar to Resistance protein candidate
chr04	Os04g0619700 15	31485367	31488482	Similar to H0114G12.8 protein.
chr04	Os04g0620066 2	31505399	31506769	Similar to MRP-like ABC transporter
chr04	Os04g0620950 15	31554480	31558637	Hypothetical conserved gene.
chr04	Os04g0621500 27	31577517	31582927	Disease resistance protein domain containing protein.
chr04	Os04g0621650 32	31597884	31602430	Hypothetical gene.
chr04	Os04g0622501 26	31635735	31639598	Hypothetical gene.
chr04	Os04g0623066 2	31660819	31662691	Similar to XA1.
chr04	Os04g0623100 4	31663355	31665410	Bromodomain containing protein.
chr04	Os04g0624100 26	31721661	31726923	Hypothetical conserved gene.
chr04	Os04g0624400 4	31727770	31728411	Similar to H0215F08.12 protein.
chr04	Os04g0624450 2	31740856	31741449	Similar to H0215F08.12 protein.
chr04	Os04g0624900 2	31767073	31767689	Conserved hypothetical protein.
chr04	Os04g0625000 6	31769056	31773154	Conserved hypothetical protein.
chr04	Os04g0625200 6	31786589	31788246	Phospholipid/glycerol acyltransferase family protein.
chr04	Os04g0625300 6	31788464	31790490	Similar to OSIGBa0148A10.3 protein.
chr04	Os04g0625350 34	31800144	31804447	Similar to OSIGBa0148A10.3 protein.
chr04	Os04g0625400 14	31801895	31803232	Similar to OSIGBa0148A10.4 protein.
chr04	Os04g0625800 22	31819464	31823467	Similar to RNA Binding Protein 45.
chr04	Os04g0625850 10	31820440	31822528	Hypothetical protein.
chr04	Os04g0625900 21	31824102	31828850	Similar to OSIGBa0148A10.9 protein.
chr04	Os04g0626000 5	31831245	31836454	Similar to OSIGBa0148A10.10 protein.
chr04	Os04g0626100 16	31838166	31842050	HMG-I and HMG-Y 2C DNA-binding domain containing protein.
chr04	Os04g0626450 8	31855518	31858459	NB-ARC domain containing protein.
chr04	Os04g0626500 21	31858607	31867385	Similar to Disease resistance protein RGA2
chr04	Os04g0627000 4	31900217	31907077	Similar to Homeobox-leucine zipper protein ROC2.
chr04	Os04g0627300 3	31932864	31933709	Similar to H0303G06.2 protein.
chr04	Os04g0627900 8	31957711	31958630	Translation initiation factor SUI1 domain containing protein.
chr04	Os04g0628100 4	31962541	31965069	Similar to Polyubiquitin.
chr04	Os04g0629000 38	31999368	32002716	Similar to H0303G06.15 protein.
chr04	Os04g0629100 18	32002722	32007327	Similar to H0303G06.16 protein.
chr04	Os04g0629200 2	32008449	32009263	Cupredoxin domain containing protein.
chr04	Os04g0629300 25	32009536	32016407	Similar to H0303G06.18 protein.

chr04	Os04g0629400 4	32017656	32018383	Similar to H0303G06.19 protein.
chr04	Os04g0629500 144	32019411	32022508	Similar to Thioredoxin h.
chr04	Os04g0629700 16	32033904	32039009	Similar to Kinesin-1
chr04	Os04g0630000 10	32044764	32048497	SDA1 domain containing protein.
chr04	Os04g0630100 10	32049063	32051694	NAD
chr04	Os04g0630500 4	32075395	32076284	Similar to H0323C08.15 protein.
chr04	Os04g0630600 7	32078504	32081665	Similar to H0323C08.16 protein.
chr04	Os04g0630800 31	32085612	32088272	Similar to Anthocyanidin reductase.
chr04	Os04g0631000 11	32131925	32135388	Similar to H0105C05.4 protein.
chr04	Os04g0632901 5	32199986	32202721	Similar to H0613A10.1 protein.
chr04	Os04g0633800 9	32244877	32248172	Protein kinase 2C catalytic domain domain containing protein.
chr04	Os04g0633900 8	32248949	32253028	Similar to H0315F07.1 protein.
chr04	Os04g0634000 16	32254122	32258262	Serine/threonine protein kinase-related domain containing protein.
chr04	Os04g0634400 2	32273357	32276841	Similar to H0315F07.5 protein.
chr04	Os04g0636900 4	32390377	32392487	RNA-binding region RNP-1
chr04	Os04g0637000 56	32403357	32412059	Similar to BZIP transcription factor bZIP52.
chr04	Os04g0637400 7	32419840	32423901	Similar to PolI-like DNA polymerase.
chr05	Os05g0122400 30	1222843	1230989	Hypothetical conserved gene.
chr05	Os05g0122500 10	1231013	1232703	Similar to PWWP domain containing protein 2C expressed.
chr05	Os05g0135700 24	2089037	2091950	S-adenosylmethionine synthetase 1
chr05	Os05g0135750 10	2090474	2091669	Hypothetical protein.
chr05	Os05g0141700 2	2400650	2401930	Phosphoglycerate mutase domain containing protein.
chr05	Os05g0141800 2	2417277	2418356	Similar to S-adenosylmethionine decarboxylase proenzyme.
chr05	Os05g0142400 2	2464329	2465034	Conserved hypothetical protein.
chr05	Os05g0169300 75	4152905	4155880	Tryptophan aminotransferase 2C Indole-3-acetic acid
chr05	Os05g0181300 2	4895311	4895815	Conserved hypothetical protein.
chr05	Os05g0196200 3	5914193	5916931	Similar to Pollen-specific protein SF21.
chr05	Os05g0197900 14	6046098	6048503	Protein of unknown function DUF247 2C plant family protein.
chr05	Os05g0198000 4	6050142	6050885	Conserved hypothetical protein.
chr05	Os05g0198200 4	6085887	6087017	Thioredoxin fold domain containing protein.
chr05	Os05g0213500 17	7034322	7036548	Similar to CAPIP1.
chr05	Os05g0241000 2	8584562	8589468	Prefoldin domain containing protein.
chr05	Os05g0322900 4	14991579	14993673	Similar to WRKY transcription factor 45.
chr05	Os05g0324600 2	15069432	15070481	Protein of unknown function DUF623 2C plant domain containing protein.
chr05	Os05g0328333 4	15266307	15266924	Similar to Prolamin.
chr05	Os05g0330700 4	15471101	15471859	Hypothetical gene.
chr05	Os05g0332250 4	15553257	15554363	Conserved hypothetical protein.
chr05	Os05g0337200 2	15753065	15754783	Helix-loop-helix DNA-binding domain containing protein.
chr05	Os05g0357525 9	16959114	16959822	Similar to Universal stress protein.
chr05	Os05g0357600 36	16966090	16972086	Similar to Developmentally regulated GTP binding protein.
chr05	Os05g0357700 12	16972974	16975853	Similar to CAAX prenyl protease 2.
chr05	Os05g0357850 16	16984532	16987132	Conserved hypothetical protein.
chr05	Os05g0358400 13	17014645	17018325	Butirosin biosynthesis 2C BtrG-like domain containing protein.
chr05	Os05g0358500 24	17017745	17021890	Protein phosphatase 2C domain containing protein.
chr05	Os05g0360800 18	17197743	17198577	Hypothetical conserved gene.
chr05	Os05g0360900 4	17199779	17200375	Pectinesterase inhibitor domain containing protein.
chr05	Os05g0361200 19	17209599	17216204	Similar to Ferrochelatase.
chr05	Os05g0384600 15	18575668	18578864	ABC transporter-like domain containing protein.
chr05	Os05g0384800 7	18586998	18595443	Similar to Chromosome condensation regulator protein
chr05	Os05g0386400 14	18713908	18714603	Conserved hypothetical protein.
chr05	Os05g0386900 32	18728817	18734916	Reticulon family protein.
chr05	Os05g0387700 4	18757319	18760229	Protein kinase 2C core domain containing protein.
chr05	Os05g0388500 16	18789640	18793642	Similar to 50S ribosomal protein L1.
chr05	Os05g0388600 31	18796477	18801851	Protein of unknown function DUF3411 domain containing protein.
chr05	Os05g0389000 4	18813590	18817685	Similar to AP2-1 protein
chr05	Os05g0389600 7	18862715	18867283	Zinc finger 2C RING/FYVE/PHD-type domain containing protein.
chr05	Os05g0389900 2	18877770	18878429	Hypothetical gene.
chr05	Os05g0390000 10	18880611	18887896	Similar to predicted protein.
chr05	Os05g0390100 11	18892000	18900658	Dynamin family protein.
chr05	Os05g0390932 12	18969257	18970726	Hypothetical protein.
chr05	Os05g0393100 8	19118103	19123031	Leucine-rich repeat 2C N-terminal domain containing protein.
chr05	Os05g0393200 11	19124235	19128438	Similar to structural constituent of ribosome.
chr05	Os05g0393400 60	19130327	19135940	Similar to PAC
chr05	Os05g0393700 10	19155053	19157429	Similar to Isoamylase-type starch debranching enzyme ISO2.
chr05	Os05g0393800 10	19160140	19166101	Protein of unknown function DUF221 domain containing protein.
chr05	Os05g0393900 6	19166560	19168329	Pentatricopeptide repeat domain containing protein.
chr05	Os05g0394301 8	19178614	19181214	Hypothetical gene.
chr05	Os05g0394200 15	19178379	19183276	Hypothetical conserved gene.
chr05	Os05g0395400 4	19240768	19241359	Hypothetical protein.
chr05	Os05g0395850 14	19281578	19283505	Hypothetical protein.
chr05	Os05g0396000 12	19281673	19283577	Similar to Hexose carrier.
chr05	Os05g0396900 2	19311394	19313597	Conserved hypothetical protein.
chr05	Os05g0397700 6	19346175	19353385	SecY protein family protein.
chr05	Os05g0397900 15	19359212	19363466	Kinesin 2C motor region domain containing protein.
chr05	Os05g0398800 9	19398909	19403514	Similar to Serine/threonine-protein kinase PBS1
chr05	Os05g0399100 4	19409584	19413589	Similar to Endo-1 2C3 3B1 2C4-beta-D-glucanase.
chr05	Os05g0399400 2	19435401	19436495	Chitinase 9.
chr05	Os05g0400100 5	19465133	19467637	Dienelactone hydrolase domain containing protein.
chr05	Os05g0401100 8	19507539	19508996	Protein of unknown function DUF477 family protein.
chr05	Os05g0402300 2	19588424	19590720	Uncharacterised protein family UPF0005 domain containing protein.
chr05	Os05g0403100 10	19638874	19640194	Peptidase A1 domain containing protein.
chr05	Os05g0403200 8	19639074	19640220	Hypothetical protein.
chr05	Os05g0403300 4	19645657	19647395	Peptidase A1 domain containing protein.
chr05	Os05g0404000 2	19678186	19680083	Mitochodrial transcription termination factor-related family protein.
chr05	Os05g0404500 9	19700612	19704404	Hypothetical protein.
chr05	Os05g0404700 12	19705359	19707432	Similar to MBD1.
chr05	Os05g0407100 7	19837789	19838522	Four F5 protein family protein.
chr05	Os05g0407200 46	19838548	19846592	WD repeat protein 23 domain containing protein.
chr05	Os05g0480200 33	23600961	23605047	Thioredoxin domain 2 containing protein.
chr05	Os05g0480400 27	23605439	23610991	Serine/threonine protein kinase domain containing protein.
chr05	Os05g0514800 2	25530831	25531070	Hypothetical conserved gene.
chr05	Os05g0534400 2	26535935	26537873	Similar to Calcineurin B-like protein 4

chr05	Os05g0549850 2	27311815	27312465	Conserved hypothetical protein.
chr05	Os05g0550700 20	27346433	27356977	Protein kinase 2C catalytic domain domain containing protein.
chr05	Os05g0551000 4	27377539	27382166	Zinc finger 2C RING-type domain containing protein.
chr05	Os05g0556800 2	27690004	27691439	Similar to Triose-phosphate Transporter family domain containing protein.
chr05	Os05g0562000 4	27958358	27960858	Conserved hypothetical protein.
chr05	Os05g0562400 37	27975677	27983968	DDT domain containing protein.
chr05	Os05g0563400 3	28034380	28039748	Similar to Auxin response factor 5.
chr05	Os05g0564800 4	28112936	28114907	Similar to 60S ribosomal protein L18A.
chr05	Os05g0573800 3	28585627	28588880	Hypothetical conserved gene.
chr05	Os05g0573900 26	28589103	28592602	Similar to predicted protein.
chr05	Os05g0574100 6	28598933	28603266	Lipase 2C class 3 family protein.
chr05	Os05g0575500 4	28667173	28668847	Conserved hypothetical protein.
chr05	Os05g0580550 6	28897619	28898513	Similar to 10A19I.15.
chr05	Os05g0580600 10	28900488	28903104	Conserved hypothetical protein.
chr05	Os05g0580800 7	28901257	28906665	Similar to GTP binding protein.
chr05	Os05g0582600 22	29000212	29002044	Peptidase S10 2C serine carboxypeptidase family protein.
chr05	Os05g0582800 4	29004127	29005828	Peptidase S10 2C serine carboxypeptidase family protein.
chr05	Os05g0584400 4	29077046	29078529	Conserved hypothetical protein.
chr05	Os05g0585500 7	29140518	29144459	Similar to Calcium-dependent protein kinase.
chr05	Os05g0585601 2	29143724	29144285	Hypothetical protein.
chr05	Os05g0586200 2	29200200	29205715	Gretchen hagen 3
chr05	Os05g0586300 8	29208375	29210085	Similar to FMA
chr05	Os05g0586350 2	29208809	29209334	Hypothetical protein.
chr05	Os05g0586600 4	29225038	29227592	Similar to SIGE
chr05	Os05g0586700 6	29228254	29229250	Conserved hypothetical protein.
chr05	Os05g0586800 4	29228293	29229233	Hypothetical protein.
chr05	Os05g0586900 10	29236686	29240068	Hypothetical conserved gene.
chr05	Os05g0587400 9	29271262	29274798	Prefoldin domain containing protein.
chr05	Os05g0587700 5	29284596	29286850	Conserved hypothetical protein.
chr05	Os05g0590100 6	29382087	29384471	Ubiquitin domain containing protein.
chr05	Os05g0591400 2	29455559	29457153	Heat shock protein Hsp70 family protein.
chr05	Os05g0591550 12	29466185	29466905	Conserved hypothetical protein.
chr05	Os05g0591800 25	29482551	29485096	Protein kinase 2C catalytic domain domain containing protein.
chr05	Os05g0591900 50	29486800	29492176	Hypothetical gene.
chr05	Os05g0592100 7	29495935	29499457	Peptidase S14 2C ClpP family protein.
chr05	Os05g0592300 35	29507007	29509972	Protein of unknown function DUF1637 family protein.
chr05	Os05g0592400 32	29511892	29520052	UV-damaged DNA binding protein.
chr05	Os05g0592500 13	29520232	29527462	SIT4 phosphatase-associated protein family protein.
chr05	Os05g0592600 26	29528780	29534681	Translation initiation factor 2 related domain containing protein.
chr05	Os05g0592800 67	29538446	29542019	Similar to Protein phosphatase 2C.
chr05	Os05g0593000 6	29545102	29546639	Phospholipase A2 2C active site domain containing protein.
chr05	Os05g0593150 5	29547868	29550160	Hypothetical gene.
chr05	Os05g0593200 2	29552673	29554019	Conserved hypothetical protein.
chr05	Os05g0593300 29	29554792	29559818	Conserved hypothetical protein.
chr05	Os05g0593550 7	29563935	29566473	Hypothetical conserved gene.
chr05	Os05g0593800 15	29571146	29575990	Similar to Dual specificity kinase 1.
chr05	Os05g0593900 6	29579051	29580572	Similar to C13 endopeptidase NP1 precursor.
chr05	Os05g0594100 8	29584835	29586141	Similar to GDA2 protein.
chr05	Os05g0594500 4	29595609	29601356	Invasin/intimin cell-adhesion domain containing protein.
chr05	Os05g0595800 3	29677769	29681781	Serine-threonine/tyrosine-protein kinase domain containing protein.
chr06	Os06g0102100 4	163259	165320	Cytochrome P450 93G2 protein
chr06	Os06g0102200 2	164238	165038	Similar to H0102C09.4 protein.
chr06	Os06g0124300 2	1289047	1291619	Protein of unknown function DUF594 family protein.
chr06	Os06g0154800 3	2827985	2830314	Hypothetical conserved gene.
chr06	Os06g0179500 4	3941687	3943447	Protein of unknown function DUF247 2C plant family protein.
chr06	Os06g0180300 7	3981988	3982486	Conserved hypothetical protein.
chr06	Os06g0181800 4	4051268	4052441	Protein of unknown function DUF868 2C plant domain containing protein.
chr06	Os06g0183900 6	4189161	4190563	Protein of unknown function DUF602 family protein.
chr06	Os06g0185300 2	4286708	4287890	Transferase family protein.
chr06	Os06g0186600 16	4371000	4376383	Conserved hypothetical protein.
chr06	Os06g0186700 5	4379813	4383418	Biopterin transport-related protein BT1 family protein.
chr06	Os06g0196200 2	4885942	4886693	Conserved hypothetical protein.
chr06	Os06g0220100 2	6180408	6181596	Hypothetical protein.
chr06	Os06g0220200 22	6191490	6192496	Similar to Phi-1-like phosphate-induced protein.
chr06	Os06g0221000 4	6239975	6241172	Similar to P-type R2R3 Myb protein
chr06	Os06g0221100 8	6243743	6247717	TLDc domain containing protein.
chr06	Os06g0222100 6	6279737	6281730	Similar to Ramosa 3
chr06	Os06g0222400 6	6304589	6305923	Similar to DRE-binding protein 2.
chr06	Os06g0222900 32	6349120	6352936	Similar to Type I inositol-1 2C4 2C5-trisphosphate 5-phosphatase 2
chr06	Os06g0223300 12	6372353	6376374	MADS box transcription factor 2C Pollen maturation
chr06	Os06g0229300 9	6725343	6734351	Similar to Hox2b protein.
chr06	Os06g0231600 2	6809574	6811873	Similar to RING-H2 finger protein ATL1Q.
chr06	Os06g0232000 42	6816406	6819804	Similar to Pro-resilin.
chr06	Os06g0232100 4	6823036	6832862	Hypothetical conserved gene.
chr06	Os06g0232400 6	6878753	6879545	Hypothetical conserved gene.
chr06	Os06g0232700 6	6895777	6901079	Similar to SWIM zinc finger family protein.
chr06	Os06g0233000 4	6915702	6916605	Conserved hypothetical protein.
chr06	Os06g0234400 2	7000436	7001602	Hypothetical conserved gene.
chr06	Os06g0234600 19	7005872	7008330	Domain of unknown function DUF231 2C plant domain containing protein.
chr06	Os06g0235100 8	7037047	7038406	Hypothetical conserved gene.
chr06	Os06g0235200 2	7043614	7045626	Protein of unknown function DUF231 domain containing protein.
chr06	Os06g0245700 6	7565169	7568366	Similar to predicted protein.
chr06	Os06g0245800 13	7574953	7581463	Similar to Alanyl-tRNA synthetase.
chr06	Os06g0271000 6	9101145	9102125	UDP-glucuronosyl/UDP-glucosyltransferase domain containing protein.
chr06	Os06g0271350 31	9116926	9124196	Hypothetical gene.
chr06	Os06g0271300 30	9116877	9124244	Similar to OSIGBa0140C02.5 protein.
chr06	Os06g0284500 8	10101308	10102349	Zinc finger 2C Dof-type family protein.
chr06	Os06g0284800 8	10120998	10124400	Spectrin repeat containing protein.
chr06	Os06g0284900 6	10126921	10127396	Hypothetical protein.
chr06	Os06g0285200 2	10137823	10138746	Similar to Leafy cotyledon 1-like L1L protein.
chr06	Os06g0286700 8	10387793	10390465	Similar to Piz-t.
chr06	Os06g0291600 2	10690711	10694167	Similar to Protein kinase G11A

chr06	Os06g0291800 4	10711028	10714423	Conserved hypothetical protein.
chr06	Os06g0314300 2	12062651	12065269	Conserved hypothetical protein.
chr06	Os06g0548500 4	20760706	20761602	Conserved hypothetical protein.
chr06	Os06g0554600 4	21090109	21091328	Conserved hypothetical protein.
chr06	Os06g0568200 4	21963208	21968733	Vacuolar ATPase B subunit.
chr06	Os06g0587100 14	23000435	23001408	Conserved hypothetical protein.
chr06	Os06g0587200 24	23005337	23008846	Protein kinase 2C catalytic domain domain containing protein.
chr06	Os06g0587300 12	23017969	23021462	Hypothetical protein.
chr06	Os06g0588800 4	23093973	23097578	Protein kinase 2C catalytic domain domain containing protein.
chr06	Os06g0589300 16	23105298	23113054	ABC transporter-like domain containing protein.
chr06	Os06g0589500 14	23119059	23122162	Conserved hypothetical protein.
chr06	Os06g0589800 2	23144729	23148845	Protein kinase 2C core domain containing protein.
chr06	Os06g0590301 8	23181263	23184271	Similar to short-chain dehydrogenase/reductase
chr06	Os06g0590600 4	23187194	23188967	Similar to Beta-1 2C3-glucanase-like protein.
chr06	Os06g0590700 8	23196356	23198035	UDP-glucuronosyl/UDP-glucosyltransferase family protein.
chr06	Os06g0591200 17	23227138	23228388	Conserved hypothetical protein.
chr06	Os06g0591450 8	23234119	23234772	Conserved hypothetical protein.
chr06	Os06g0592951 15	23298136	23299239	Conserved hypothetical protein.
chr06	Os06g0593200 2	23310215	23311945	UDP-glucuronosyl/UDP-glucosyltransferase family protein.
chr06	Os06g0593800 8	23333368	23335030	UDP-glucuronosyl/UDP-glucosyltransferase family protein.
chr06	Os06g0594100 42	23343928	23350815	Crotonase 2C core domain containing protein.
chr06	Os06g0594200 6	23357768	23360733	Disease resistance protein domain containing protein.
chr06	Os06g0594300 4	23367126	23368775	Hypothetical protein.
chr06	Os06g0594400 2	23367376	23368829	Kelch-type beta propeller domain containing protein.
chr06	Os06g0595900 10	23442875	23449453	Conserved hypothetical protein.
chr06	Os06g0596300 5	23467234	23471674	Similar to Acyl-ACP thioesterase
chr06	Os06g0597000 4	23501973	23503491	Similar to Auxin responsive protein IAA-Re.
chr06	Os06g0597200 19	23505601	23510147	Similar to DNA-binding protein phosphatase 2C.
chr06	Os06g0597800 4	23556493	23559215	FAR1 domain containing protein.
chr06	Os06g0598800 12	23608738	23611096	Similar to Fatty acid elongase 1-like protein.
chr06	Os06g0598850 10	23609159	23610725	Hypothetical protein.
chr06	Os06g0598900 30	23613188	23621153	Similar to Serine-threonine kinase receptor-associated protein
chr06	Os06g0599700 4	23656248	23668376	Conserved hypothetical protein.
chr06	Os06g0604600 2	23949819	23950657	Conserved hypothetical protein.
chr06	Os06g0620200 2	24907230	24909987	Protein kinase 2C catalytic domain domain containing protein.
chr06	Os06g0620700 30	24920808	24921218	Conserved hypothetical protein.
chr06	Os06g0623200 11	25066390	25071028	Similar to Cinnamoyl-CoA reductase
chr06	Os06g0623700 4	25093242	25094294	Similar to strictosidine synthase 1.
chr06	Os06g0624700 5	25145594	25148015	Protein of unknown function DUF23 family protein.
chr06	Os06g0625200 6	25182993	25184931	Protein kinase-like domain containing protein.
chr06	Os06g0625400 35	25200331	25210590	Similar to Metalloendopeptidase.
chr06	Os06g0625500 18	25214052	25215245	Similar to Thioredoxin peroxidase.
chr06	Os06g0641600 12	26097154	26098278	Similar to Cytochrome P450 family protein.
chr06	Os06g0641575 14	26097136	26098831	Hypothetical protein.
chr06	Os06g0641800 8	26105139	26105547	Cytochrome P450 family protein.
chr06	Os06g0641866 24	26105242	26107212	Hypothetical protein.
chr06	Os06g0651300 2	26661060	26664595	Zinc finger 2C U1-type domain containing protein.
chr06	Os06g0653800 16	26779067	26781799	Similar to WD-repeat protein 57
chr06	Os06g0654600 2	26834016	26836134	Similar to BRASSINOSTEROID INSENSITIVE 1-associated receptor kinase 1.
chr07	Os07g0100200 6	11648	12983	Similar to PDX1-like protein 4.
chr07	Os07g0100500 34	36140	42289	Conserved hypothetical protein.
chr07	Os07g0100600 2	42657	44528	Similar to Peptide transporter.
chr07	Os07g0100900 6	56872	58217	Legume lectin 2C beta domain containing protein.
chr07	Os07g0101000 8	58808	60650	Cupredoxin domain containing protein.
chr07	Os07g0101200 6	72958	75621	Pentatricopeptide repeat domain containing protein.
chr07	Os07g0101800 4	109286	110520	Hypothetical conserved gene.
chr07	Os07g0102100 42	122255	127934	Similar to Potassium transporter 22.
chr07	Os07g0102150 4	126296	127477	Hypothetical protein.
chr07	Os07g0102200 6	128713	131371	Conserved hypothetical protein.
chr07	Os07g0102300 19	134173	143578	Putative glycosylphosphatidylinositol-anchored protein 2C Regulation of leaf rolling
chr07	Os07g0102400 7	147148	150184	Protein of unknown function DUF1084 family protein.
chr07	Os07g0103000 4	182515	183243	Similar to GRF zinc finger family protein 2C expressed.
chr07	Os07g0103100 28	191053	196429	Similar to Glutamate receptor 3.4 precursor
chr07	Os07g0103200 9	196515	199802	Similar to DDRGK domain-containing protein 1.
chr07	Os07g0104233 4	240466	240945	Similar to peroxidase 1.
chr07	Os07g0104366 2	252925	253553	Hypothetical protein.
chr07	Os07g0105100 4	292708	294492	Similar to DNA-directed RNA polymerases I 2C II 2C and III 7.3 kDa polypeptide.
chr07	Os07g0105200 2	299704	301425	Conserved hypothetical protein.
chr07	Os07g0105600 2	306054	306968	Photosystem II oxygen evolving complex protein PsbQ family protein.
chr07	Os07g0105800 2	326737	329181	Protein of unknown function DUF594 domain containing protein.
chr07	Os07g0105900 4	333276	334779	NB-ARC domain containing protein.
chr07	Os07g0106700 18	373811	383052	Similar to disease resistance response protein 206.
chr07	Os07g0107366 4	404708	405708	Hypothetical gene.
chr07	Os07g0107583 12	412918	413595	Conserved hypothetical protein.
chr07	Os07g0107800 20	424972	428523	Similar to Phytosulfokine receptor precursor
chr07	Os07g0107900 14	428798	431607	Conserved hypothetical protein.
chr07	Os07g0108000 12	431999	437549	Hypothetical conserved gene.
chr07	Os07g0108100 11	438289	439843	Similar to OSIGBa0147O06.5 protein.
chr07	Os07g0108200 7	440518	444836	Similar to transparent testa 12 protein.
chr07	Os07g0108400 10	451750	453710	Similar to Pollen-specific C2 domain containing protein.
chr07	Os07g0108500 16	457738	460218	Similar to Pollen-specific C2 domain containing protein.
chr07	Os07g0108800 14	467286	468753	Similar to Calcium-activated outward-rectifying potassium channel 1
chr07	Os07g0108900 29	471234	477655	Similar to MADS-box transcription factor 15.
chr07	Os07g0109100 2	496582	498981	Protein of unknown function DUF594 family protein.
chr07	Os07g0109800 13	538041	539687	Conserved hypothetical protein.
chr07	Os07g0110000 27	548481	554208	Conserved hypothetical protein.
chr07	Os07g0110650 42	597035	598203	Hypothetical gene.
chr07	Os07g0110800 4	604586	607425	Conserved hypothetical protein.
chr07	Os07g0111600 5	658777	663168	Similar to Purple acid phosphatase.
chr07	Os07g0111700 31	664618	667101	Flavin monooxygenase FMO domain containing protein.
chr07	Os07g0112000 2	677468	678907	Dimethylaniline monooxygenase-like protein.
chr07	Os07g0112300 4	689982	690619	3'-5' exonuclease domain containing protein.

chr07	Os07g0112351 4	690060	690567	Hypothetical protein.
chr07	Os07g0112400 4	693416	694906	3&apos 3B-5&apos 3B exonuclease domain containing protein.
chr07	Os07g0112600 24	697413	698927	Conserved hypothetical protein.
chr07	Os07g0113101 2	719584	720210	Conserved hypothetical protein.
chr07	Os07g0114500 3	809697	812515	Conserved hypothetical protein.
chr07	Os07g0114550 8	813276	813966	Conserved hypothetical protein.
chr07	Os07g0115300 2	838070	841318	Similar to Peroxidase2 precursor
chr07	Os07g0115350 2	838279	840844	Hypothetical protein.
chr07	Os07g0115600 6	852331	855022	Protein of unknown function DUF566 domain containing protein.
chr07	Os07g0119300 5	1046046	1047972	Homeodomain-related domain containing protein.
chr07	Os07g0119400 4	1055475	1058127	Similar to Pectinesterase like protein.
chr07	Os07g0119475 3	1055540	1057811	Hypothetical protein.
chr07	Os07g0120700 3	1096381	1101697	Conserved hypothetical protein.
chr07	Os07g0120800 6	1101854	1105389	Protein of unknown function DUF1719 2C Oryza sativa family protein.
chr07	Os07g0121750 223	1140062	1142002	Conserved hypothetical protein.
chr07	Os07g0122200 4	1156619	1159170	Protein of unknown function DUF1719 2C Oryza sativa family protein.
chr07	Os07g0122300 12	1159806	1163369	Protein of unknown function DUF1719 2C Oryza sativa family protein.
chr07	Os07g0124750 3	1305328	1308048	Similar to cDNA clone:J033097L05 2C full insert sequence.
chr07	Os07g0124800 2	1313767	1316444	Similar to Chaperone protein dnaJ.
chr07	Os07g0129300 2	1523654	1524512	Similar to PR1a protein.
chr07	Os07g0133500 4	1770644	1772559	Protein of unknown function DUF1005 family protein.
chr07	Os07g0133601 4	1770882	1772490	Hypothetical protein.
chr07	Os07g0162450 10	3339394	3340629	Protein of unknown function DUF3778 domain containing protein.
chr07	Os07g0162500 20	3342219	3343596	Alpha/beta hydrolase fold-3 domain containing protein.
chr07	Os07g0162600 32	3344780	3346551	Alpha/beta hydrolase fold-3 domain containing protein.
chr07	Os07g0162900 4	3360001	3361559	Lipase 2C GDXG 2C active site domain containing protein.
chr07	Os07g0167300 2	3616170	3618158	Conserved hypothetical protein.
chr07	Os07g0244400 2	8043562	8045191	Pentatricopeptide repeat domain containing protein.
chr07	Os07g0422700 6	13640924	13642840	Glucoamylase 2C putatuve domain containing protein.
chr07	Os07g0425000 10	13776950	13778155	Biopterin transport-related protein BT1 family protein.
chr07	Os07g0434300 2	14502054	14502650	Similar to Myb-like DNA-binding domain containing protein.
chr07	Os07g0434700 4	14520218	14524575	Peptidase M24 2C methionine aminopeptidase family protein.
chr07	Os07g0436100 3	14574609	14580312	Similar to predicted protein.
chr07	Os07g0439100 6	14774623	14779028	KH 2C type 1 domain containing protein.
chr07	Os07g0439750 10	14817883	14818361	Hypothetical conserved gene.
chr07	Os07g0440100 2	14824356	14825681	Conserved hypothetical protein.
chr07	Os07g0443500 7	15033384	15037334	Molecular chaperone 2C heat shock protein 2C Hsp40 2C DnaJ domain containing protein.
chr07	Os07g0446600 2	15280459	15282788	Similar to OSIGBa0102I15.5 protein.
chr07	Os07g0452400 5	15595751	15600607	3'-5' exonuclease domain containing protein.
chr07	Os07g0452600 6	15614582	15616817	Hypothetical conserved gene.
chr07	Os07g0454400 7	15732952	15745599	Conserved hypothetical protein.
chr07	Os07g0455100 12	15769883	15787386	Hypothetical conserved gene.
chr07	Os07g0456033 6	15852356	15854513	Hypothetical gene.
chr07	Os07g0456500 8	15875835	15881340	Hypothetical conserved gene.
chr07	Os07g0456900 10	15899431	15902146	No apical meristem
chr07	Os07g0457400 17	15918969	15922849	Hypothetical conserved gene.
chr07	Os07g0458500 35	15982511	15983945	Hypothetical conserved gene.
chr07	Os07g0458700 23	15990914	15993925	Transmembrane protein 97 2C predicted domain containing protein.
chr07	Os07g0458800 18	15998528	16003262	Similar to Serine/threonine-protein kinase 19.
chr07	Os07g0459000 4	16009452	16010073	Hypothetical protein.
chr07	Os07g0468100 36	16674227	16675326	Similar to Glutathione S-transferase GST 19
chr08	Os08g0104900 14	268733	272077	Protein of unknown function DUF6 2C transmembrane domain containing protein.
chr08	Os08g0113100 4	701252	703998	Similar to Fructokinase
chr08	Os08g0113200 12	704559	713864	RNA-processing protein 2C HAT helix domain containing protein.
chr08	Os08g0113400 4	720518	721865	Methyltransferase type 11 domain containing protein.
chr08	Os08g0113800 14	740821	745479	Hypothetical conserved gene.
chr08	Os08g0113900 2	746499	747134	Conserved hypothetical protein.
chr08	Os08g0114100 7	751421	753191	Conserved hypothetical protein.
chr08	Os08g0152700 4	3017359	3021986	Diacylglycerol kinase 2C catalytic region domain containing protein.
chr08	Os08g0152800 8	3022668	3026892	Similar to predicted protein.
chr08	Os08g0152900 11	3031282	3038465	Armadillo-like helical domain containing protein.
chr08	Os08g0153900 6	3093804	3094878	Protein of unknown function DUF599 family protein.
chr08	Os08g0154000 8	3102013	3105084	O-methyltransferase 2C family 3 protein.
chr08	Os08g0189900 2	5260239	5261296	Germin-like protein 8-10 2C Disease resistance
chr08	Os08g0190200 11	5268917	5273165	Similar to cDNA clone:J033051E20 2C full insert sequence.
chr08	Os08g0191000 2	5322769	5325114	Auxin efflux carrier domain containing protein.
chr08	Os08g0192800 6	5418272	5420390	Bromodomain containing protein.
chr08	Os08g0192900 6	5422274	5426674	Nucleotide-binding 2C alpha-beta plait domain containing protein.
chr08	Os08g0193700 2	5466374	5469315	Similar to NBS-LRR disease resistance protein homologue
chr08	Os08g0194000 2	5479726	5481493	F-box domain 2C Skp2-like domain containing protein.
chr08	Os08g0197100 102	5618541	5621553	Cyclin-like F-box domain containing protein.
chr08	Os08g0237800 7	8384563	8385805	Similar to Xyloglucan endotransglycosylase
chr08	Os08g0243866 4	8789627	8792452	Similar to chromatin remodeling complex subunit.
chr08	Os08g0245200 2	8874179	8878862	Similar to 4-coumarate--CoA ligase 1
chr08	Os08g0246300 8	8939500	8953088	NB-ARC domain containing protein.
chr08	Os08g0248300 20	9049938	9051200	Hypothetical protein.
chr08	Os08g0249200 4	9111086	9111844	Similar to GDU1.
chr08	Os08g0249300 4	9123551	9126818	Hypothetical protein.
chr08	Os08g0249400 2	9126454	9127450	Hypothetical conserved gene.
chr08	Os08g0250100 18	9174654	9178792	Hypothetical conserved gene.
chr08	Os08g0250300 4	9181114	9181517	Similar to predicted protein.
chr08	Os08g0250900 21	9224375	9227905	Conserved hypothetical protein.
chr08	Os08g0254300 15	9399521	9402233	Similar to OSIGBa0112M24.2 protein.
chr08	Os08g0254500 514	9405688	9414700	Similar to Preprotein translocase secY subunit 2C chloroplast precursor
chr08	Os08g0254600 211	9411335	9416401	Similar to cyclic nucleotide-gated ion channel 14.
chr08	Os08g0254900 3	9424760	9428473	Similar to Glutamyl-tRNA reductase binding protein.
chr08	Os08g0263000 2	9958741	9960475	Similar to Cytochrome P450-like protein
chr08	Os08g0425300 2	20429460	20431143	Similar to Endoglucanase 21.
chr08	Os08g0523100 2	26013703	26014560	BTB/POZ-like domain containing protein.
chr08	Os08g0535800 6	26766816	26770335	No apical meristem
chr08	Os08g0542900 10	27163603	27169027	Lupus La protein family protein.
chr08	Os08g0548500 2	27511196	27513554	Armadillo-like helical domain containing protein.

chr08	Os08g0558000 8	27929363	27931717	Similar to Ribosomal protein.
chr08	Os08g0558100 6	27931885	27934321	EF hand domain containing protein.
chr09	Os09g0295000 8	7077214	7078851	No apical meristem
chr09	Os09g0305300 8	7797868	7800107	Protein of unknown function DUF247 2C plant family protein.
chr09	Os09g0306700 2	7914083	7925334	Phytochrome and flowering time 1 protein.
chr09	Os09g0315700 2	8692296	8697399	Phosphoenolpyruvate carboxylase family protein.
chr09	Os09g0318600 9	8969316	8973855	Homeodomain-like containing protein.
chr09	Os09g0330000 9	9825477	9832295	UDP-glucuronosyl/UDP-glucosyltransferase family protein.
chr09	Os09g0344700 2	10751518	10752279	Conserved hypothetical protein.
chr09	Os09g0348400 36	10936863	10947944	Protein kinase 2C catalytic domain domain containing protein.
chr09	Os09g0348600 4	10956083	10965501	Protein kinase 2C catalytic domain domain containing protein.
chr09	Os09g0350900 17	11123332	11132792	Protein kinase 2C core domain containing protein.
chr09	Os09g0355400 41	11387149	11396236	Protein kinase 2C core domain containing protein.
chr09	Os09g0357400 2	11540699	11556180	Disease resistance protein domain containing protein.
chr09	Os09g0360900 8	11779735	11785476	Conserved hypothetical protein.
chr09	Os09g0361100 2	11790541	11793359	Similar to Protein kinase.
chr09	Os09g0361400 14	11805613	11809021	Outer mitochondrial membrane protein porin
chr09	Os09g0364500 2	11958028	11960980	GTP-binding protein 2C HSR1-related domain containing protein.
chr09	Os09g0415300 19	14843939	14847953	Hypothetical conserved gene.
chr09	Os09g0415700 2	14867389	14870846	Protein of unknown function DUF248 2C methyltransferase putative family protein.
chr09	Os09g0415750 2	14868118	14870584	Hypothetical protein.
chr09	Os09g0441700 2	16403807	16407995	Similar to flavonoid 3-monooxygenase.
chr09	Os09g0442100 2	16422290	16425218	Protein kinase 2C core domain containing protein.
chr09	Os09g0557500 2	22156928	22159600	Similar to ATPUP5.
chr09	Os09g0558300 4	22192750	22197181	Similar to Cyclic nucleotide-gated channel A
chr09	Os09g0558850 2	22207975	22208505	Similar to ZFP1
chr09	Os09g0560150 2	22259974	22260398	Hypothetical genes.
chr09	Os09g0560200 2	22260384	22262115	Similar to 26S protease regulatory subunit 6B
chr09	Os09g0560800 9	22281491	22284167	Molybdenum cofactor sulfurase 2C C-terminal domain containing protein.
chr10	Os10g0104300 3	345785	347108	Helix-loop-helix DNA-binding domain containing protein.
chr10	Os10g0108700 12	583024	584657	Transferase family protein.
chr10	Os10g0124500 2	1556778	1559803	Cyclin-like F-box domain containing protein.
chr10	Os10g0135700 2	2211559	2212224	Hypothetical gene.
chr10	Os10g0151100 6	3060912	3065547	Similar to Protein kinase domain containing protein 2C expressed.
chr10	Os10g0151800 4	3113841	3118873	Similar to predicted protein.
chr10	Os10g0152000 2	3122019	3127181	Serine/threonine protein kinase domain containing protein.
chr10	Os10g0152400 2	3122025	3127180	Hypothetical protein.
chr10	Os10g0174548 6	5157636	5164933	Similar to Calcium binding EGF domain containing protein.
chr10	Os10g0372800 11	11807697	11811899	Conserved hypothetical protein.
chr10	Os10g0374200 8	11863947	11865368	Paired amphipathic helix domain containing protein.
chr10	Os10g0389200 4	12886331	12888289	Similar to Red chlorophyll catabolite reductase [Oryza sativa
chr10	Os10g0434000 2	15462806	15463918	Similar to BTB/POZ domain containing protein.
chr10	Os10g0502900 14	19190130	19194448	Cyclin-like F-box domain containing protein.
chr10	Os10g0514600 20	19840596	19842443	Similar to Clone ZZD584 mRNA sequence.
chr10	Os10g0521100 6	20160992	20162434	Similar to Actin-depolymerizing factor 6
chr10	Os10g0522900 4	20281431	20285006	Isopenicillin N synthase family protein.
chr10	Os10g0545500 2	21320574	21322000	Similar to xyloglucan endotransglucosylase/hydrolase protein 32.
chr10	Os10g0547500 2	21417294	21419832	Divalent ion symporter domain containing protein.
chr10	Os10g0547650 2	21417314	21419289	Hypothetical protein.
chr10	Os10g0555200 4	21802742	21804315	Resolvase 2C holliday junction-type 2C YqgF-like domain containing protein.
chr10	Os10g0555300 12	21809273	21810954	Zinc finger 2C C2H2-like domain containing protein.
chr10	Os10g0557366 2	21944163	21944875	Conserved hypothetical protein.
chr10	Os10g0557600 2	21944314	21945183	Similar to GATA transcription factor 9.
chr10	Os10g0558400 2	21979109	21981026	Similar to Oxidoreductase 2C 2OG-Fe oxygenase family protein.
chr10	Os10g0558750 2	21992040	21993690	Similar to Oxidoreductase 2C 2OG-Fe oxygenase family protein 2C expressed.
chr11	Os11g0141700 2	1942620	1944140	Similar to GRAS family transcription factor containing protein.
chr11	Os11g0141800 4	1946099	1946770	Conserved hypothetical protein.
chr11	Os11g0142800 2	1998790	1999485	Similar to Zinc finger 2C C3HC4 type family protein.
chr11	Os11g0143500 10	2019766	2024207	Similar to L-Galactono-1 2C4-lactone dehydrogenase.
chr11	Os11g0146000 2	2087123	2087685	Conserved hypothetical protein.
chr11	Os11g0146700 18	2112283	2118775	Similar to predicted protein.
chr11	Os11g0146750 4	2119120	2119608	Hypothetical conserved gene.
chr11	Os11g0147150 2	2148786	2151562	Hypothetical gene.
chr11	Os11g0150200 11	2335086	2341117	Conserved hypothetical protein.
chr11	Os11g0150700 2	2356476	2360843	Serine/threonine protein kinase domain containing protein.
chr11	Os11g0150801 2	2359925	2360755	Hypothetical protein.
chr11	Os11g0151400 2	2390496	2392366	Cytochrome P450 family protein.
chr11	Os11g0158832 2	2842577	2844384	Similar to Associated with HOX family protein 2C expressed.
chr11	Os11g0163600 2	3098464	3102645	Similar to Tubby-like F-box protein 13.
chr11	Os11g0163800 2	3110576	3112429	Protein of unknown function DUF793 family protein.
chr11	Os11g0165700 2	3173825	3175505	Mannose-binding lectin domain containing protein.
chr11	Os11g0166201 2	3183224	3183778	Similar to BZIP transcription factor family protein 2C expressed.
chr11	Os11g0184800 8	4295162	4297455	Similar to Aspartic proteinase Asp1 precursor
chr11	Os11g0187500 72	4443167	4446247	Similar to Heat shock protein 70.
chr11	Os11g0208100 5	5530077	5534187	Cyclin-like F-box domain containing protein.
chr11	Os11g0208300 4	5546196	5554530	Hypothetical conserved gene.
chr11	Os11g0208400 12	5557380	5560163	Cyclin-like F-box domain containing protein.
chr11	Os11g0208500 6	5558242	5560096	Hypothetical protein.
chr11	Os11g0209502 8	5630932	5634827	F-box domain 2C cyclin-like domain containing protein.
chr11	Os11g0209550 4	5633618	5634870	Hypothetical protein.
chr11	Os11g0211300 2	5777015	5787839	Similar to NBS-LRR disease resistance protein homologue
chr11	Os11g0212000 32	5820879	5826729	Similar to Leucine Rich Repeat family protein 2C expressed.
chr11	Os11g0212801 17	5852691	5853472	Conserved hypothetical protein.
chr11	Os11g0234200 18	7125124	7128857	Zinc finger 2C RING/FYVE/PHD-type domain containing protein.
chr11	Os11g0235200 4	7193230	7198486	Peptide transporter
chr11	Os11g0235250 2	7196789	7197296	Hypothetical gene.
chr11	Os11g0235300 2	7202765	7205523	Hypothetical gene.
chr11	Os11g0239400 2	7434637	7435428	Similar to Serpin
chr11	Os11g0246600 7	7909213	7916588	Similar to Peptidase S1 and S6 2C chymotrypsin/Hap.
chr11	Os11g0249000 25	8072537	8078086	NB-ARC domain containing protein.
chr11	Os11g0249300 4	8082926	8084825	Conserved hypothetical protein.
chr11	Os11g0249400 2	8085126	8086522	Similar to Polygalacturonase family protein 2C expressed.

chr11	Os11g0260200 158	8702363	8704999	Similar to Sulfotransferase domain containing protein 2C expressed.
chr11	Os11g0262600 12	8864479	8866289	Conserved hypothetical protein.
chr11	Os11g0287100 2	10373993	10374618	Similar to disulfide oxidoreductase/ monooxygenase/ oxidoreductase.
chr11	Os11g0474900 2	16441481	16442206	Similar to Stemar-13-ene synthase.
chr11	Os11g0493700 4	17388238	17390587	Similar to NBS-LRR type resistance protein
chr11	Os11g0492300 2	17434258	17436861	Similar to NB-ARC domain containing protein.
chr11	Os11g0494100 4	17562956	17563942	Similar to NBS-LRR type resistance protein
chr11	Os11g0495400 4	17596500	17596895	Similar to PHF5-like protein.
chr11	Os11g0495950 6	17599930	17601552	FAD-linked oxidase 2C FAD-binding 2C subdomain 2 domain containing protein.
chr11	Os11g0503900 7	17943546	17945362	Sulfotransferase family protein.
chr11	Os11g0506700 18	18069286	18071596	Similar to ATPase 2C coupled to transmembrane movement of substances.
chr11	Os11g0506800 10	18070313	18071749	Similar to BAG domain containing protein.
chr11	Os11g0513700 2	18372832	18378851	Protein kinase-like domain containing protein.
chr11	Os11g0518900 92	18636476	18650782	Conserved hypothetical protein.
chr11	Os11g0522900 6	18831750	18835479	Peptidase S10 2C serine carboxypeptidase family protein.
chr11	Os11g0523700 8	18922046	18925254	Similar to Transcription factor ICE1
chr11	Os11g0523800 11	18935399	18941686	Similar to Isoform 3 of Auxin response factor 23.
chr11	Os11g0524601 11	18994678	19000728	Similar to NB-ARC domain containing protein 2C expressed.
chr11	Os11g0525200 34	19048566	19052876	Similar to Cytochrome P450 51
chr11	Os11g0525600 8	19058420	19067600	Similar to Alpha-mannosidase.
chr11	Os11g0528200 4	19170791	19172631	Similar to NEF1
chr11	Os11g0528400 8	19175188	19179728	Similar to aminopeptidase.
chr11	Os11g0543500 8	19956772	19963299	Similar to H0502B11.6 protein.
chr11	Os11g0543900 10	19985515	19987297	Hypothetical protein.
chr11	Os11g0544000 8	19985660	19989024	Hypothetical conserved gene.
chr11	Os11g0544100 2	19988741	19991058	Conserved hypothetical protein.
chr11	Os11g0545000 10	20045013	20048974	NOP5 2C N-terminal domain containing protein.
chr11	Os11g0545600 17	20098066	20106820	Similar to chromatin modification-related protein EAF3.
chr11	Os11g0545650 2	20107052	20107816	Conserved hypothetical protein.
chr11	Os11g0545700 4	20109599	20113395	Similar to Centromere/kinetochore protein zw10 homolog.
chr11	Os11g0546000 24	20129027	20134231	Similar to RNase L inhibitor-like protein.
chr11	Os11g0546600 6	20174656	20176341	Similar to phospholipase A2.
chr11	Os11g0550900 31	20482685	20488052	Conserved hypothetical protein.
chr11	Os11g0552500 2	20566514	20567788	Conserved hypothetical protein.
chr11	Os11g0556733 22	20683795	20685811	Hypothetical protein.
chr11	Os11g0557300 4	20709538	20714159	BSD domain containing protein.
chr11	Os11g0557700 18	20720724	20723628	Similar to S-adenosylmethionine-dependent methyltransferase/ catalytic.
chr11	Os11g0557800 6	20723964	20724619	Conserved hypothetical protein.
chr11	Os11g0558300 3	20743741	20748358	Similar to Acyl CoA synthetase
chr11	Os11g0558400 4	20748838	20751918	Leucine-rich repeat 2C N-terminal domain containing protein.
chr11	Os11g0558566 12	20759465	20760108	Hypothetical conserved gene.
chr11	Os11g0562600 2	20964403	20966323	Conserved hypothetical protein.
chr11	Os11g0567800 2	21156776	21159062	Leucine-rich repeat-containing N-terminal 2C type 2 domain containing protein.
chr11	Os11g0568800 2	21213554	21216313	Leucine-rich repeat 2C typical subtype domain containing protein.
chr11	Os11g0576100 2	21705480	21708029	Toll/interleukin-1 receptor homology
chr11	Os11g0583200 12	22040372	22044971	Hypothetical conserved gene.
chr11	Os11g0599200 4	22926178	22928035	UDP-glucuronosyl/UDP-glucosyltransferase family protein.
chr11	Os11g0600500 2	23060525	23061100	Calcium-binding EF-hand domain containing protein.
chr11	Os11g0600900 5	23079578	23081682	Similar to Mannose-6-phosphate isomerase
chr11	Os11g0601500 5	23119941	23123201	Similar to Protein kinase domain containing protein.
chr11	Os11g0602300 2	23171943	23176340	Similar to HVA22-like protein a
chr11	Os11g0602750 21	23187999	23193264	Conserved hypothetical protein.
chr11	Os11g0602800 8	23194895	23197316	Galactose oxidase/kelch 2C beta-propeller domain containing protein.
chr11	Os11g0614800 3	23866045	23867666	Similar to Cyclin-dependent kinase inhibitor 7.
chr11	Os11g0615700 524	23938240	23942543	Proteasome subunit alpha type 5
chr11	Os11g0615750 238	23938499	23942192	Hypothetical gene.
chr11	Os11g0619500 14	24120143	24122947	Peptidase C19 2C ubiquitin carboxyl-terminal hydrolase 2 domain containing protein.
chr11	Os11g0620300 18	24180911	24181379	Similar to Nonspecific lipid-transfer protein 2
chr11	Os11g0620500 5	24192139	24195237	Tyrosine protein kinase domain containing protein.
chr11	Os11g0621300 2	24219144	24224045	Protein of unknown function DUF1399 family protein.
chr11	Os11g0623700 22	24382955	24384066	Conserved hypothetical protein.
chr11	Os11g0628000 3	24616115	24619613	Protein kinase 2C catalytic domain domain containing protein.
chr11	Os11g0634800 2	24989004	24993250	Similar to Adenylyl-sulfate kinase.
chr11	Os11g0639600 152	25289129	25293951	NB-ARC domain containing protein.
chr11	Os11g0640000 12	25316103	25318049	NB-ARC domain containing protein.
chr11	Os11g0640300 4	25321323	25325479	NB-ARC domain containing protein.
chr11	Os11g0652100 4	26027842	26032480	NB-ARC domain containing protein.
chr11	Os11g0659500 7	26468486	26473248	Similar to Acyl-ACP thioesterase
chr11	Os11g0661000 125	26524929	26531006	Pentatricopeptide repeat domain containing protein.
chr11	Os11g0661600 41	26572255	26574116	Similar to Peroxidase
chr11	Os11g0661700 9	26572401	26573275	Hypothetical protein.
chr11	Os11g0661900 18	26579230	26580721	Conserved hypothetical protein.
chr11	Os11g0664800 8	26794437	26799952	Hypothetical conserved gene.
chr11	Os11g0668300 7	26967537	26977525	Hypothetical conserved gene.
chr11	Os11g0670100 3	27077916	27080652	Serine-threonine/tyrosine-protein kinase domain containing protein.
chr11	Os11g0670900 6	27111943	27112811	Conserved hypothetical protein.
chr11	Os11g0671650 10	27142195	27145889	Hypothetical gene.
chr11	Os11g0672600 24	27175081	27177923	Reverse transcriptase domain containing protein.
chr11	Os11g0672700 117	27179323	27184026	RecQ DNA helicase 2C RNA silencing
chr11	Os11g0677400 6	27366801	27368303	Pectinesterase inhibitor domain containing protein.
chr11	Os11g0678400 2	27428398	27430560	Similar to NB-ARC domain containing protein 2C expressed.
chr11	Os11g0679000 6	27451209	27452951	von Willebrand factor 2C type A domain containing protein.
chr11	Os11g0679900 78	27492119	27495458	Conserved hypothetical protein.
chr11	Os11g0680200 40	27495679	27498080	Pentatricopeptide repeat domain containing protein.
chr11	Os11g0681400 3	27571044	27574081	Similar to protein kinase.
chr11	Os11g0683700 8	27656251	27658409	Pectin lyase fold domain containing protein.
chr11	Os11g0683800 12	27660470	27662654	Pectin lyase fold/virulence factor domain containing protein.
chr11	Os11g0684000 61	27670321	27673334	Similar to Transcription factor MYB21
chr11	Os11g0684100 34	27683640	27695070	NB-ARC domain containing protein.
chr11	Os11g0684400 5	27686517	27689972	Hypothetical protein.
chr11	Os11g0684700 4	27703769	27707310	Similar to NB-ARC domain containing protein 2C expressed.
chr11	Os11g0685600 6	27735451	27737645	Conserved hypothetical protein.

chr11	Os11g0686100 14	27758164	27761632	Conserved hypothetical protein.
chr11	Os11g0686400 17	27788716	27792722	Similar to WRKY transcription factor 41.
chr11	Os11g0686500 2	27793778	27797821	NB-ARC domain containing protein.
chr11	Os11g0688832 2	27978644	27979861	Similar to Leucine Rich Repeat family protein.
chr11	Os11g0689500 6	28005496	28008609	Conserved hypothetical protein.
chr11	Os11g0689650 2	28009048	28011861	Conserved hypothetical protein.
chr11	Os11g0690332 2	28075457	28076938	Similar to Protein kinase domain containing protein.
chr11	Os11g0691280 4	28142192	28148337	EGF-type aspartate/asparagine hydroxylation site domain containing protein.
chr11	Os11g0696400 10	28483633	28486502	Glycoside hydrolase 2C family 3 2C N-terminal domain containing protein.
chr11	Os11g0696501 10	28483678	28486409	Hypothetical protein.
chr11	Os11g0700100 2	28653511	28655917	Similar to LZ-NBS-LRR class RGA.
chr11	Os11g0701200 4	28733145	28734249	Glycoside hydrolase 2C family 18 protein.
chr12	Os12g0109600 2	515426	517055	Protein of unknown function DUF1677 2C Oryza sativa family protein.
chr12	Os12g0135200 2	1732197	1733659	Kelch-type beta propeller domain containing protein.
chr12	Os12g0160300 7	3013268	3015766	Conserved hypothetical protein.
chr12	Os12g0205633 20	5535012	5535540	Conserved hypothetical protein.
chr12	Os12g0206700 9	5568838	5573431	Hypothetical protein.
chr12	Os12g0207000 6	5584743	5590285	Transcription factor.
chr12	Os12g0208900 8	5674410	5676438	Similar to ZF-HD protein dimerisation region containing protein 2C expressed.
chr12	Os12g0209000 3	5685138	5689991	Similar to sporulation protein-related.
chr12	Os12g0209100 14	5692925	5696271	Similar to predicted protein.
chr12	Os12g0209700 54	5736998	5739519	Hypothetical conserved gene.
chr12	Os12g0209800 98	5740543	5747593	Similar to NB-ARC domain containing protein 2C expressed.
chr12	Os12g0209901 6	5749555	5750951	Similar to NB-ARC domain containing protein 2C expressed.
chr12	Os12g0210450 2	5772511	5773053	Hypothetical gene.
chr12	Os12g0211400 2	5849738	5852392	A/G-specific adenine glycosylase MutY 2C bacterial form domain containing protein.
chr12	Os12g0211500 4	5856248	5859276	Leucine-rich repeat 2C N-terminal domain containing protein.
chr12	Os12g0217400 4	6224187	6227225	Similar to Leucine Rich Repeat family protein 2C expressed.
chr12	Os12g0225900 2	6869987	6872291	Allyl alcohol dehydrogenase.
chr12	Os12g0226700 5	6911276	6913929	Similar to Allyl alcohol dehydrogenase.
chr12	Os12g0231100 3	7171810	7176574	Conserved hypothetical protein.
chr12	Os12g0232300 7	7235000	7237115	Similar to ZCN20.
chr12	Os12g0232700 16	7243314	7244392	Hypothetical protein.
chr12	Os12g0233200 2	7289748	7291739	Hypothetical conserved gene.
chr12	Os12g0233900 2	7330373	7331104	FAS1 domain domain containing protein.
chr12	Os12g0264500 25	9350914	9365572	Similar to CoA-thioester hydrolase CHY1
chr12	Os12g0265201 8	9429281	9433003	Conserved hypothetical protein.
chr12	Os12g0266200 5	9476374	9478760	Similar to Fibrillin-2 precursor.
chr12	Os12g0267400 14	9546322	9547871	Hypothetical protein.
chr12	Os12g0268000 4	9579773	9581772	Similar to Cytochrome P450 71A1
chr12	Os12g0268100 4	9580088	9582014	Hypothetical protein.
chr12	Os12g0273900 24	10058775	10061686	Leucine-rich repeat domain containing protein.
chr12	Os12g0273980 12	10062718	10066028	Similar to Leucine Rich Repeat family protein 2C expressed.
chr12	Os12g0276300 2	10211510	10212437	Similar to Ubiquitin family protein 2C expressed.
chr12	Os12g0277000 8	10254122	10257442	Zinc finger 2C RING/FYVE/PHD-type domain containing protein.
chr12	Os12g0277500 16	10293602	10297903	Similar to RuBisCO subunit binding-protein alpha subunit 2C chloroplast precursor
chr12	Os12g0277900 9	10320410	10321997	Hypothetical conserved gene.
chr12	Os12g0278101 9	10320410	10321941	Hypothetical gene.
chr12	Os12g0283100 2	10711964	10717815	Nucleotidyltransferase domain containing protein.
chr12	Os12g0283300 2	10724195	10725087	Conserved hypothetical protein.
chr12	Os12g0283900 4	10766538	10768960	Heat shock protein DnaJ 2C conserved site domain containing protein.
chr12	Os12g0284000 9	10771046	10775430	Regulator of chromosome condensation 2C RCC1 domain containing protein.
chr12	Os12g0284525 4	10790598	10791269	Conserved hypothetical protein.
chr12	Os12g0285100 32	10822535	10833618	Hypothetical conserved gene.
chr12	Os12g0285600 3	10862295	10872221	Pleckstrin homology-type domain containing protein.
chr12	Os12g0285650 3	10862429	10872065	Hypothetical protein.
chr12	Os12g0285700 9	10874698	10877400	Hypothetical conserved gene.
chr12	Os12g0290150 6	11194132	11205015	Similar to QS
chr12	Os12g0290200 2	11201823	11202284	Hypothetical protein.
chr12	Os12g0291033 2	11260276	11260721	Hypothetical conserved gene.
chr12	Os12g0297400 4	11716268	11724611	Hypothetical protein.
chr12	Os12g0407900 2	12349459	12350966	Pentatricopeptide repeat domain containing protein.
chr12	Os12g0408000 134	12354378	12356834	Protein of unknown function DUF594 domain containing protein.
chr12	Os12g0408100 118	12354567	12356767	Hypothetical protein.
chr12	Os12g0409700 8	12433463	12439186	Hypothetical conserved gene.
chr12	Os12g0412400 6	12581812	12582469	Hypothetical gene.
chr12	Os12g0415800 4	12807810	12808492	Similar to Histone H3.
chr12	Os12g0417100 4	12892992	12893423	Hypothetical conserved gene.
chr12	Os12g0418900 13	13021779	13025304	Hypothetical conserved gene.
chr12	Os12g0419000 5	13025972	13028599	Conserved hypothetical protein.
chr12	Os12g0420200 2	13103675	13106278	NAD
chr12	Os12g0427100 2	13602957	13604220	Similar to Protein kinase domain containing protein 2C expressed.
chr12	Os12g0427450 2	13610317	13611468	Similar to Protein kinase domain containing protein.
chr12	Os12g0428600 3	13697949	13702555	Similar to E3 ubiquitin protein ligase UPL2
chr12	Os12g0428700 2	13703310	13703839	Hypothetical protein.
chr12	Os12g0431300 4	13885480	13888224	Six-bladed beta-propeller 2C TolB-like domain containing protein.
chr12	Os12g0437800 12	14411366	14412019	Similar to MPI.
chr12	Os12g0437932 6	14419117	14419367	Hypothetical conserved gene.
chr12	Os12g0438000 9	14423865	14424890	Similar to Histone H2A.
chr12	Os12g0439700 10	14597050	14597355	Similar to RER1A protein.
chr12	Os12g0441600 2	14750544	14752232	O-methyltransferase 2C family 2 domain containing protein.
chr12	Os12g0441700 64	14754353	14757981	Conserved hypothetical protein.
chr12	Os12g0444800 13	14974988	14977605	Similar to O-methyltransferase ZRP4.
chr12	Os12g0447466 14	15262097	15262959	Hypothetical conserved gene.
chr12	Os12g0447932 50	15262567	15268325	Conserved hypothetical protein.
chr12	Os12g0448400 34	15284375	15284934	Hypothetical protein.
chr12	Os12g0448650 10	15316001	15316708	Similar to AIR12.
chr12	Os12g0448900 25	15330237	15340502	Fatty acid alpha-dioxygenase family 2C Enzyme that oxygenates fatty acids into 2R-hydroperoxides
chr12	Os12g0464400 2	16398248	16400772	Similar to Oxidoreductase 2C short chain dehydrogenase/reductase family protein 2C expressed.
chr12	Os12g0465700 2	16500219	16504887	Conserved hypothetical protein.
chr12	Os12g0466800 34	16565027	16565942	Hypothetical protein.
chr12	Os12g0468600 4	16706568	16712920	Amidohydrolase 1 domain containing protein.

chr12	Os12g0470700	10	16872793	16874580	ATPase 2C AAA-type 2C core domain containing protein.
chr12	Os12g0480200	11	17628181	17632392	Similar to H0321H01.14 protein.
chr12	Os12g0480250	2	17628428	17629204	Hypothetical protein.
chr12	Os12g0482700	10	17788701	17791111	Similar to L-galactose dehydrogenase.
chr12	Os12g0486800	66	18111165	18113735	Hypothetical protein.
chr12	Os12g0487832	28	18272198	18273207	Hypothetical conserved gene.
chr12	Os12g0488300	2	18287172	18287652	Similar to ribosomal protein L22.
chr12	Os12g0490566	3	18412383	18413445	Similar to embryo-sac basal-endosperm-layer embryo-surrounding-region2.
chr12	Os12g0503101	2	19194860	19199795	Hypothetical conserved gene.
chr12	Os12g0503200	14	19199289	19205182	Similar to TRANSLATION INITIATION FACTOR IF-2P.
chr12	Os12g0504900	2	19332572	19333201	Glucoamylase 2C putatuve domain containing protein.
chr12	Os12g0516300	12	20061564	20066924	NB-ARC domain containing protein.
chr12	Os12g0552600	2	22447769	22449195	Similar to MATE efflux family protein.
chr12	Os12g0552800	10	22466362	22469274	Conserved hypothetical protein.
chr12	Os12g0554400	4	22518982	22529230	Tetratricopeptide-like helical domain containing protein.
chr12	Os12g0555200	5	22591681	22593505	Similar to Probenazole-inducible protein PBZ1.
chr12	Os12g0556300	11	22635834	22640197	Calmodulin binding protein-like family protein.
chr12	Os12g0557800	6	22723146	22726841	Pentatricopeptide repeat domain containing protein.
chr12	Os12g0559400	2	22872891	22875304	Similar to Leucine Rich Repeat family protein.
chr12	Os12g0559600	6	22875416	22876184	Similar to Resistance protein RGA2.
chr12	Os12g0578800	6	23952363	23956048	Zinc finger 2C C2H2 domain containing protein.
chr12	Os12g0582666	8	24180594	24182306	Cytochrome P450 family protein.
chr12	Os12g0583900	48	24246429	24252312	Similar to PDE135
chr12	Os12g0586600	11	24485299	24490983	Similar to Plasma membrane Ca2+-ATPase.
chr12	Os12g0603300	4	25414541	25418738	Similar to AP2 domain containing protein 2C expressed.
chr12	Os12g0605900	8	25583204	25586395	Similar to Kinase like protein.
chr12	Os12g0606100	2	25605994	25611594	Hypothetical conserved gene.
chr12	Os12g0608500	4	25699763	25701967	Serine/threonine protein kinase-related domain containing protein.
chr12	Os12g0608600	4	25702077	25706956	Similar to Thiosulfate sulfurtransferase
chr12	Os12g0608900	4	25718546	25720672	Protein of unknown function DUF26 domain containing protein.
chr12	Os12g0613100	2	25960810	25964140	Similar to Amino acid permease family protein.
chr12	Os12g0613150	2	25960847	25964139	Hypothetical protein.
chr12	Os12g0615400	2	26101180	26103543	Similar to 37 kDa inner envelope membrane protein 2C chloroplast precursor
chr12	Os12g0618800	4	26363271	26365704	Protein of unknown function DUF266 2C plant family protein.
chr12	Os12g0619000	30	26369246	26374651	IQ calmodulin-binding region domain containing protein.
chr12	Os12g0619100	8	26377806	26379849	Heat shock protein DnaJ 2C cysteine-rich domain domain containing protein.
chr12	Os12g0619700	6	26381679	26400289	Conserved hypothetical protein.
chr12	Os12g0620400	22	26425353	26430083	Methyl-CpG DNA binding domain containing protein.
chr12	Os12g0620600	3	26433354	26437997	Conserved hypothetical protein.
chr12	Os12g0621900	57	26548466	26555878	Similar to Mov34/MPN/PAD-1 family protein 2C expressed.
chr12	Os12g0631800	27	27051976	27056972	Similar to predicted protein.
chr12	Os12g0632100	6	27061650	27063765	Similar to ARL2 G-protein.
chr12	Os12g0632800	78	27109127	27112150	Protein kinase 2C catalytic domain domain containing protein.
chr12	Os12g0632900	9	27122488	27126252	Serine/threonine protein kinase-related domain containing protein.
chr12	Os12g0633000	2	27128175	27131959	Xylulose kinase.

Pedigree-based genome re-sequencing reveals genetic variation patterns of elite backbone varieties during modern rice improvement
Xingfei Zheng, Lanzhi Li, Fan Liang, Changjun Tan, Shuzhu Tang, Sibin Yu, Ying Diao, Shuangcheng Li, and Zhongli Hu
Supplementary Table 19 Gene ontology (GO) enrichment analysis for selective genes in pedigree breeding.

GO ID	GO Term	GO Class	*P* value	Adjusted *P* value	Number of selective genes	Enrich Direction	GO level
GO enrichment analysis for genes with signature SNPs in GC2H pedigree							
GO:0030554	adenyl nucleotide binding	MF	2.20E-14	2.17E-11	271	Over	6
GO:0032559	adenyl ribonucleotide binding	MF	3.37E-14	2.17E-11	270	Over	6
GO:0017076	purine nucleotide binding	MF	1.67E-12	7.16E-10	281	Over	5
GO:0001883	purine nucleoside binding	MF	3.61E-12	7.76E-10	279	Over	5
GO:0032550	purine ribonucleoside binding	MF	3.61E-12	7.76E-10	279	Over	6
GO:0032555	purine ribonucleotide binding	MF	3.61E-12	7.76E-10	279	Over	5
GO:0032549	ribonucleoside binding	MF	4.82E-12	8.14E-10	279	Over	5
GO:0001882	nucleoside binding	MF	5.05E-12	8.14E-10	279	Over	4
GO:0032553	ribonucleotide binding	MF	1.71E-11	2.45E-09	279	Over	4
GO:0097367	carbohydrate derivative binding	MF	4.88E-11	6.29E-09	279	Over	3
GO:0000166	nucleotide binding	MF	1.53E-09	1.64E-07	339	Over	4
GO:1901265	nucleoside phosphate binding	MF	1.53E-09	1.64E-07	339	Over	4
GO:0036094	small molecule binding	MF	3.95E-09	3.92E-07	342	Over	3
GO:0043168	anion binding	MF	4.47E-09	4.12E-07	298	Over	4
GO:0005524	ATP binding	MF	1.25E-08	1.08E-06	218	Over	6
GO:0043531	ADP binding	MF	3.50E-08	2.82E-06	52	Over	5
GO:0005623	cell	CC	8.69E-08	6.23E-06	129	Under	2
GO:0044464	cell part	CC	8.69E-08	6.23E-06	129	Under	2
GO:0004672	protein kinase activity	MF	1.31E-07	8.90E-06	139	Over	6
GO:0004674	protein serine/threonine kinase activity	MF	1.83E-07	1.18E-05	122	Over	7
GO:0016301	kinase activity	MF	3.23E-07	1.98E-05	152	Over	5
GO:0035639	purine ribonucleoside triphosphate binding	MF	4.19E-07	2.45E-05	227	Over	5
GO:0006468	protein phosphorylation	BP	4.45E-07	2.49E-05	140	Over	7
GO:0005622	intracellular	CC	6.33E-07	3.27E-05	123	Under	3
GO:0016310	phosphorylation	BP	6.35E-07	3.27E-05	143	Over	6
GO:0044424	intracellular part	CC	1.16E-06	5.74E-05	93	Under	3
GO:0032991	macromolecular complex	CC	1.44E-06	6.47E-05	34	Under	2
GO:0006857	oligopeptide transport	BP	1.51E-06	6.47E-05	18	Over	6
GO:0015833	peptide transport	BP	1.51E-06	6.47E-05	18	Over	5
GO:0042886	amide transport	BP	1.51E-06	6.47E-05	18	Over	4
GO:0006952	defense response	BP	4.18E-06	0.00017389	42	Over	4
GO:0016773	phosphotransferase activity	MF	5.80E-06	0.000233501	145	Over	5
GO:0016772	transferase activity,	MF	6.30E-06	0.000246002	166	Over	4
GO:0005737	cytoplasm	CC	7.59E-06	0.000286809	38	Under	4
GO:0071705	nitrogen compound transport	BP	7.79E-06	0.000286809	22	Over	5
GO:0006796	phosphate-containing compound metabolic process	BP	9.30E-06	0.000332925	165	Over	5
GO enrichment analysis for genes with signature SNPs in HHZ pedigree							
GO:0032559	adenyl ribonucleotide binding	MF	6.87E-11	4.05E-08	156	Over	6
GO:0030554	adenyl nucleotide binding	MF	7.92E-11	4.05E-08	156	Over	6
GO:0001883	purine nucleoside binding	MF	7.47E-10	1.16E-07	162	Over	5
GO:0032550	purine ribonucleoside binding	MF	7.47E-10	1.16E-07	162	Over	6
GO:0032555	purine ribonucleotide binding	MF	7.47E-10	1.16E-07	162	Over	5
GO:0032549	ribonucleoside binding	MF	9.05E-10	1.16E-07	162	Over	5
GO:0001882	nucleoside binding	MF	9.34E-10	1.16E-07	162	Over	4
GO:0017076	purine nucleotide binding	MF	9.65E-10	1.16E-07	162	Over	5
GO:0032553	ribonucleotide binding	MF	1.08E-09	1.16E-07	163	Over	4
GO:0097367	carbohydrate derivative binding	MF	1.14E-09	1.16E-07	164	Over	3
GO:0004672	protein kinase activity	MF	5.73E-09	5.33E-07	89	Over	6
GO:0043168	anion binding	MF	1.28E-08	1.09E-06	176	Over	4
GO:0016773	phosphotransferase activity	MF	2.39E-08	1.88E-06	95	Over	5
GO:0006468	protein phosphorylation	BP	2.79E-08	2.04E-06	89	Over	7
GO:0004674	protein serine/threonine kinase activity	MF	1.04E-07	7.06E-06	76	Over	7
GO:0016301	kinase activity	MF	1.14E-07	7.32E-06	94	Over	5
GO:0016310	phosphorylation	BP	1.61E-07	9.70E-06	89	Over	6
GO:0000166	nucleotide binding	MF	2.89E-07	1.55E-05	192	Over	4
GO:1901265	nucleoside phosphate binding	MF	2.89E-07	1.55E-05	192	Over	4
GO:0036094	small molecule binding	MF	7.33E-07	3.75E-05	193	Over	3
GO:0005524	ATP binding	MF	1.00E-06	4.89E-05	125	Over	6
GO:0016772	transferase activity	MF	1.42E-06	6.61E-05	102	Over	4
GO:0043531	ADP binding	MF	2.55E-06	0.00011355	31	Over	5
GO:0006464	cellular protein modification process	BP	4.35E-06	0.00017814	97	Over	6
GO:0036211	protein modification process	BP	4.35E-06	0.00017814	97	Over	5
GO:0006796	phosphate-containing compound metabolic process	BP	4.91E-06	0.0001933	100	Over	5
GO:0006793	phosphorus metabolic process	BP	5.22E-06	0.00019767	100	Over	4
GO:0035639	purine ribonucleoside triphosphate binding	MF	5.79E-06	0.0002117	131	Over	5
GO:0004713	protein tyrosine kinase activity	MF	7.82E-06	0.00027583	45	Over	7
GO enrichment analysis for selective genes in SH527pedigree							
GO:0016706	oxidoreductase activity	MF	3.88E-09	3.11E-06	15	Over	5
GO:0051213	dioxygenase activity	MF	1.70E-07	6.78E-05	15	Over	4

Pedigree-based genome re-sequencing reveals genetic variation patterns of elite backbone varieties during modern rice improvement

Xingfei Zheng, Lanzhi Li, Fan Liang, Changjun Tan, Shuzhu Tang, Sibin Yu, Ying Diao, Shuangcheng Li,and Zhongli Hu

Supplementary Table S20 Well-known genes with signature SNPs during conventional rice pedigree breeding

Phenotype classification	Chromosome	Gene name	Start position	End position	Gene symbol[a]	Pedigree	Improvement SNPs number	Detailed regulated traits	Annotation
Grain quality	chr04	Os04g0624600	31751600	31759420	*OsSSIIIb. SSIII-1*	GC2H	14	Prediction grain quality	Soluble starch synthase 3
	chr04	Os04g0164900	4408357	4418889	*OsPUL, SDBE, OsPUL3*	GC2H	80	Prediction grain quality	Starch debranching enzyme
	chr05	Os05g0405000	19718538	19737605	*flo4; OsPPDKB*	GC2H	17	grain quality	PEP-utilising enzyme
	chr05	Os05g0393700	19155053	19157429	*OsISA2*	HHZ	5	Prediction grain quality	Isoamylase-type starch debranching enzyme
	chr06	Os06g0229800	6748398	6753302	*ALK; SSIIa;*	GC2H	2	Gelatinization temperature	Soluble starch synthase II
	chr06	Os06g0222100	6279737	6281730	TPP8	GC2H, HH:	4; 2	Prediction grain quality	Ramosa 3
	chr07	Os07g0243200	7983125	7989283	*OsAPL4*	GC2H	5	Seed development	ADP-glucose pyrophosphorylase
	chr07	Os07g0421300	13534771	13548698	unknow	GC2H	110	Prediction grain quality	Alpha glucosidase-like protein
	chr07	Os07g0681700	28909026	28914027	Hexosyltransferase	GC2H	6	Prediction grain quality	Glycosyl transferase 2C family 8 protein
	chr08	Os08g0113100	701252	703998	*FRK2*	HHZ	1	grain quality	PfkB family
	chr12	Os12g0443600	14893170	14896224	*UGD5*	GC2H	2	grain quality	UDP-glucose 6-dehydrogenase
	chr12	Os12g0563700	23118859	23122699	Pectinesterase	GC2H	29	Prediction grain quality	Pectin lyase
	chr12	Os12g0631100	27022013	27025203	*OsRab5a; gpa1; glup4*	GC2H	3	Storage protein transport	Small GTPase
Plant shape	chr04	Os04g0550400	27551423	27559580	*MIP1; OsHUB1*	GC2H, HH:	3,6	Plant height and tiller number	Ubiquitin ligase activity
	chr04	Os04g0663600	33860374	33861424	*MOC3;WOX1*	GC2H	1	tiller number	Transcription factor
	chr11	Os11g0490600	17318918	17324441	*LA1; LAZY1*	GC2H	49	Tiller angle	unknow
	chr11	Os11g0587000	22221628	22231042	*d-27*	GC2H	6	Tillering and plant height	Iron-containing protein
Leaf shape	chr07	Os07g0102300	134173	143578	*SRL1*	GC2H, HH:	50, 19	Leaf roll	GPI-anchored protein
	chr12	Os12g0555600	22602934	22607307	*OsCSLD4; NRL1; ND1; sle1*	GC2H	4	Leaf width and cell wall	Cellulose synthase-like protein
Grain shape	chr05	Os05g0135700	2089037	2091950	*OsSAMS1*	HHZ	24	Grain size and grain number pe	S-Adenosyl-L-Methionine Synthetase
	chr06	Os06g0623700	25093242	25094294	*TGW6*	HHZ	4	Grain weight	IAA-glucose hydrolase
Root morphology	chr11	Os11g0184900	4299277	4301784	*OsNAC5*	GC2H	13	Root diameter, yield and deseas	NAC domain transcription factor
	chr01	Os01g0898300	39052287	39058529	*Osspr1*	GC2H	1	Root length and Iron homeostas	unknow
Heading date	chr01	Os01g0881500	38261856	38265489	*OsPH1*	GC2H	3	Flowering and plant heigth	GA signal transduction
	chr09	Os09g0307800	8039179	8046886	*SDG724; lvp1*	GC2H	11	Flowering	Histone methyltransferase
Spike shape	chr11	Os11g0235200	7193230	7198486	*SP1; OsNPF4.1*	GC2H, HH:	6, 4	Panicle development	PTR family transporter
Disease resistance	chr01	Os01g0527600	18940389	18943033	*OsRDR6; SHL2*	GC2H	5	Stripe virus defense/Spikelet de	RNA-dependent RNA polymerase
	chr01	Os01g0149500	2683222	2686364	*Pit*	HHZ	8	Blast disease defense	leucine-rich repeat-containing (NLR) family proteins
	chr06	Os06g0286700	10387793	10390465	*Pi9*	GC2H, HH:	3,8	Blast disease defense	leucine-rich repeat-containing (NLR) family proteins
	chr08	Os08g0539400	26968546	26978236	*Pib*	GC2H	4	Blast disease defense	leucine-rich repeat-containing (NLR) family proteins
	chr09	Os09g0327600	9672226	9674673	*Pi5; Pi5-1*	GC2H	56	Blast disease defense	leucine-rich repeat-containing (NLR) family proteins
	chr11	Os11g0249000	8072537	8078086	*NLS1*	GC2H, HH:	31, 25	Bacteria disease resistance	NB-LRR-type protein
	chr11	Os11g0688832	27978644	27979861	*PIK-1*	HHZ	2	Blast disease defense	NB-LRR-type protein
	chr11	Os11g0559200	20802978	20806262	*Xa21; Xa-21*	GC2H	1	bacterial blight defence	Serine / threonine protein kinase activity
Hormone response	chr04	Os04g0169100	4738375	4742148	*ETR2*	GC2H	5	Ethylene signaling	Ethylene receptor
	chr11	Os11g0523800	18935399	18941686	*OsARF23; OsARF1*	GC2H, HH:	8, 8	auxin signaling	auxin response factor
	chr12	Os12g0479400	17571844	17577848	*OsARF24*	GC2H	16	auxin signaling	auxin response factor
Pollen development	chr04	Os04g0479000	23955481	23959974	*CRC1*	GC2H	12	Meiosis	Synaptonemal complex-related
	chr06	Os06g0223300	6372353	6376374	MADS63	GC2H, HH:	23, 12	Pollen development	Transcription factor
	chr07	Os07g0108900	471234	477655	*OsMADS15; DEP*	HHZ	29	Pseudovivipary	Transcription factor
	chr08	Os08g0110200	536368	539032	*Rf6*	GC2H	3	Fertility restorer	Pentatricopeptide-repeat family protein
	chr10	Os10g0495200	18832249	18836049	*Rf4*	GC2H	3	Fertility restorer	pentatricopeptide repeat protein
Fertilizers application	chr06	Os06g0603600	23875437	23879965	*OsSPX1*	GC2H	1	Phosphate starvation response	Transcription regulator
	chr07	Os07g0438800	14753753	14759288	*OsPHR2*	GC2H	3	Phosphate starvation response	Transcription factor
	chr04	Os04g0624500	31749141	31751604	*PHR1*	GC2H	9	Phosphate starvation response	Transcription factor
Domestication	chr01	Os01g0848400	36445456	36449951	*qSH1*	HHZ	4	Seed shattering	BEL1-type homeobox gene

Note: (a) genes marked in red represent that they are not yet been cloned,but have already been reported at transcript level.

Pedigree-based genome re-sequencing reveals genetic variation patterns of elite backbone varieties during modern rice improvement

Xingfei Zheng, Lanzhi Li, Fan Liang, Changjun Tan, Shuzhu Tang, Sibin Yu, Ying Diao, Shuangcheng Li, and Zhongli Hu

Supplementary Table S21 Selective sweeps identified from the restorer (SH527) pedigree

Chromosome	Position start	Position end	Regions (kb)	100kb/10kb sliding windows			
				Max $\log_2\pi$	Min $\log_2\pi$	Max ZF_{ST}	Min ZF_{ST}
chr01	3440001	3540000	100.0	2.16	2.16	2.03	2.03
chr01	8940001	9050000	110.0	2.21	1.89	2.55	2.13
chr01	9100001	9200000	100.0	1.77	1.77	2.13	2.13
chr03	10960001	11170000	210.0	3.40	2.71	2.13	1.96
chr03	16920001	17020000	100.0	2.41	2.41	2.02	2.02
chr03	24750001	24930000	180.0	1.76	1.73	2.07	1.96
chr03	26350001	26470000	120.0	2.41	2.28	2.08	2.01
chr03	33950001	34050000	100.0	2.88	2.88	1.98	1.98
chr04	20830001	21010000	180.0	2.35	1.87	2.84	2.42
chr05	5310001	5490000	180.0	3.06	2.31	2.43	2.07
chr06	800001	970000	170.0	2.51	1.88	3.04	1.99
chr06	1100001	1230000	130.0	1.97	1.73	2.01	1.96
chr06	1470001	1880000	410.0	2.42	1.75	3.76	2.08
chr06	2820001	2940000	120.0	1.98	1.85	2.51	2.06
chr06	2950001	3050000	100.0	1.79	1.79	2.07	2.07
chr06	3240001	3480000	240.0	2.62	1.74	3.57	2.92
chr06	3580001	4010000	430.0	4.14	1.77	3.00	1.99
chr06	6480001	6580000	100.0	1.83	1.83	1.96	1.96
chr06	11070001	11280000	210.0	2.34	1.82	2.92	2.18
chr07	1270001	1410000	140.0	2.20	1.84	2.39	2.28
chr07	1510001	1640000	130.0	2.08	1.92	2.25	1.99
chr07	1790001	1890000	100.0	1.86	1.86	2.20	2.20
chr07	2050001	2280000	230.0	1.79	1.73	2.46	2.22
chr07	8800001	8980000	180.0	2.04	1.78	3.67	3.44
chr07	9050001	9310000	260.0	2.32	1.76	2.87	2.10
chr07	13680001	13780000	100.0	2.00	2.00	2.05	2.05
chr07	14550001	14700000	150.0	3.30	2.96	2.20	1.96
chr07	15980001	16080000	100.0	1.81	1.81	2.36	2.36
chr07	16240001	16350000	110.0	1.84	1.83	2.13	2.09
chr07	16560001	16700000	140.0	2.80	2.03	2.60	2.29
chr08	830001	930000	100.0	2.43	2.43	1.96	1.96
chr09	12030001	12180000	150.0	2.68	2.04	2.18	2.00
chr10	21800001	22120000	320.0	5.88	3.04	2.13	1.96
chr11	2510001	2690000	180.0	1.99	1.79	2.08	1.98
chr11	3780001	3880000	100.0	1.73	1.73	2.17	2.17

Pedigree-based genome re-sequencing reveals genetic variation patterns of elite backbone varieties during modern rice improvement
Xingfei Zheng, Lanzhi Li, Fan Liang, Changjun Tan, Shuzhu Tang, Sibin Yu, Ying Diao, Shuangcheng Li, and Zhongli Hu

Supplementary Table 22 Putative artificial selective genes in the restorer (SH527) pedigree (*wx*,*OsNRAMP5*,*d-6*,*OsSIK1* and *GHD7* genes are marked in blue)

Gene name	Annotation	Transcript_evidence	Orf evidence	InterPro annotaition
Os01g0167400	Similar to Protein synthesis inhibitor II (EC 3.2.2.22) (Ribosome-inactivating protein II) (rRNA N-glycosidase).	AK120073	Q7GC66	Ribosome-inactivating protein
Os01g0167500	Protein of unknown function DUF250 domain containing protein.	AK062253 ,AK119227	B4G114	Domain of unknown function DUF250
Os01g0167600	Hypothetical conserved gene.	EU970199	Q5VQG2	-
Os01g0167700	Conserved hypothetical protein.	AK069523	B9ET17	-
Os01g0167750	Pentatricopeptide repeat domain containing protein.	BT085700	NP_001172197.1 (RefSeq)	Pentatricopeptide repeat
Os01g0167800	Conserved hypothetical protein.	AK101983	A2ZPP2	-
Os01g0167900	KIP1-like domain containing protein.	AK072705 ,AK060540	A2WL42	KIP1-like
Os01g0168000	Hypothetical protein.	AK120808	longestORF	-
Os01g0168100	KIP1-like domain containing protein.	AK121992	NP_001146907.1 (RefSeq)	KIP1-like
Os01g0168200	Similar to Thioredoxin-like protein.	AK103756	Q9SWG6	Thioredoxin
Os01g0168300	Conserved hypothetical protein.	AK063678	F2EKM9	-
Os01g0168350	Non-protein coding transcript.	EU949302	NONE	-
Os01g0168400	Zinc finger, RING/FYVE/PHD-type domain containing protein.	AK120658	NP_001149464.1 (RefSeq)	Zinc finger, RING-type
Os01g0168450	Hypothetical protein.	tplb0030g10 (Wheat FLcDNA)	longestORF	-
Os01g0168500	WD40 repeat-like domain containing protein.	AK072657	NP_191203.2 (RefSeq)	WD40 repeat
Os01g0168600	Similar to predicted protein.	AK288853	Q9LMF6	GDP-fucose protein O-fucosyltransferase
Os01g0168800	Alpha/beta hydrolase fold-1 domain containing protein.	AK289212	NP_001152296.1 (RefSeq)	Alpha/beta hydrolase fold-1
Os01g0168850	Hypothetical gene.	AK287979	longestORF	-
Os01g0169000	Hypothetical protein.	EU971393	GeneMark	-
Os01g0263600	U5 snRNP-associated 102 kDa protein (U5-102 kDa protein).	AK101040	A2WN54	RNA-processing protein, HAT helix
Os01g0263900	Hypothetical protein.	EU949860	GeneMark	-
Os01g0264000	Zinc finger, Dof-type domain containing protein.	AK063318	B9EV34	Zinc finger, Dof-type
Os01g0264100	Similar to UDP-glucose pyrophosphorylase.	tplb0010b10 (Wheat FLcDNA)	Q6Y643	UTP--glucose-1-phosphate uridylyltransferase
Os01g0264125	Non-protein coding transcript.	BT016879	NONE	-
Os01g0264400	Conserved hypothetical protein.	AK107988	A2WN59	-
Os01g0264500	Similar to T11I18.15 protein.	AK062518	Q9SQQ8	-
Os01g0264600	Conserved hypothetical protein.	BT086466	NP_001172271.1 (RefSeq)	-
Os01g0264700	Cellular retinaldehyde binding/alpha-tocopherol transport family protein.	AK071712	XP_002893215.1 (RefSeq)	Cellular retinaldehyde binding/alpha-tocopherol transport
Os01g0264900	Non-protein coding transcript.	AK059509	NONE	-
Os01g0265000	Aromatic-ring hydroxylase-like domain containing protein.	AK366103	Q4U3Z4	Aromatic-ring hydroxylase-like
Os01g0265100	Similar to ADP-ribosylation factor.	AK067217	D7REK4	Small GTP-binding protein domain
Os01g0265200	Mitochondrial carrier protein domain containing protein.	AK121045	B6TN88	Mitochondrial carrier protein
Os01g0265400	Conserved hypothetical protein.	AK120531	Q10DI9	-
Os01g0266400	Conserved hypothetical protein.	AK121188	A2WN82	-
Os01g0266500	Phenazine biosynthesis PhzC/PhzF protein family protein.	AK101084	B6U0C2	Phenazine biosynthesis PhzC/PhzF protein
Os01g0266600	Thioredoxin fold domain containing protein.	AK069749	B7E2T3	Thioredoxin-like fold
Os01g0266800	Cystinosin/ERS1p repeat containing protein.	AK069474	B6UIB9	Cystinosin/ERS1p repeat
Os01g0267050	Non-protein coding transcript.	AK105155	NONE	-
Os01g0267100	MT-A70 family protein.	AK065023	Q10RS1	DNA methylase, N-6 adenine-specific, conserved site
Os01g0267200	Similar to 26S proteasome non-ATPase regulatory subunit 14 (26S proteasome regulatory subunit rpn11).	AK099455	B4FLQ8	Mov34/MPN/PAD-1
Os01g0267300	Hypothetical conserved gene.	AK359881	NP_001042684.2 (RefSeq)	Protease inhibitor I4, serpin
Os01g0267400	Similar to cell number regulator 5.	AK242388	NP_001132085.1 (RefSeq)	Uncharacterised protein family Cys-rich
Os01g0267600	Sad1/UNC-like, C-terminal domain containing protein.	AK287530	B6TRH2	Sad1/UNC-like, C-terminal
Os01g0267800	Serine/threonine protein kinase domain containing protein.	AK336038	NP_001042687.2 (RefSeq)	Protein kinase, catalytic domain
Os01g0267900	Similar to Calmodulin NtCaM3 (Calmodulin NtCaM4) (Calmodulin NtCaM5) (Calmodulin NtCaM6) (Calmodulin NtCaM7) (C	AK242346	NP_001060691.1 (RefSeq)	Calcium-binding EF-hand
Os03g0307900	Conserved hypothetical protein.	AK072469	NP_001057005.1 (RefSeq)	-
Os03g0308000	Ubiquitin-conjugating enzyme/RWD-like domain containing protein.	AK100355	B6TT84	Ubiquitin-conjugating enzyme, E2
Os03g0308100	Peptidase S14, ClpP family protein.	AK105977	B9F7Y8	Peptidase S14, ClpP
Os03g0308200	Armadillo-type fold domain containing protein.	AK072741	A3AH82	Armadillo-type fold
Os03g0308500	DEAD-like helicase, N-terminal domain containing protein.	AK103891	NP_001147596.1 (RefSeq)	RNA helicase, ATP-dependent, DEAD-box, conserved site
Os03g0308600	Conserved hypothetical protein.	AK070495	B8AMH7	-
Os03g0308700	Conserved hypothetical protein.	AK361640	B9F828	-
Os03g0308800	Tetratricopeptide-like helical domain containing protein.	AK068534	NP_188329.1 (RefSeq)	RNA-processing protein, HAT helix
Os03g0308850	Similar to Fip1 motif family protein, expressed.	EU948240	Q10MH2	-
Os03g0308900	Similar to Fip1 motif family protein, expressed.	AK064183	Q10MH2	Pre-mRNA polyadenylation factor Fip1
Os03g0309000	Virulence factor, pectin lyase fold family protein.	AK070071	B8AMH9	Cytochrome c-552/DMSO reductase-like, haem-binding domain
Os03g0309200	Similar to Phytochrome B.	AK071993	B9F830	PAS
Os03g0309250	Hypothetical gene.	AK251801	longestORF	-
Os03g0309300	Hypothetical protein.	AK360624	longestORF	-
Os03g0309400	Pectin lyase fold domain containing protein.	AK100728	C6TJY2	Pectinesterase, catalytic
Os03g0309800	Similar to PPR2.	AK106141 ,AK060922 ,AK098942	Q7Y1W6	Pentatricopeptide repeat
Os03g0310000	Lipase, GDSL domain containing protein.	AK060625	Q10MF9	Lipase, GDSL
Os03g0310400	Crotonase, core domain containing protein.	AK068241	NP_001149527.1 (RefSeq)	Crotonase, core
Os03g0310500	CAP, conserved site domain containing protein.	AK068123	Q10MF7	-
Os03g0310600	Protein of unknown function DUF247, plant family protein.	AK109731	A2XFX4	Protein of unknown function DUF247, plant
Os03g0310633	Non-protein coding transcript.	BT085296	NONE	-
Os03g0310666	Hypothetical protein.	CT837847	longestORF"	-
Os03g0310701	Conserved hypothetical protein.	CT835221	NP_001173393.1 (RefSeq)"	-
Os03g0310800	Similar to Hypersensitive reaction associated Ca2+-binding protein.	AK099476 ,AK058663	NP_001147406.1 (RefSeq)	Calcium-binding EF-hand
Os03g0310900	Conserved hypothetical protein.	AK108200	B8AMI9	-
Os03g0311000	Hypothetical protein.	AK064126	longestORF	-
Os03g0311300	Similar to HAD-superfamily hydrolase, subfamily IA, variant 3 containing protein, expressed.	AK073167 ,AK103974 ,AK109395	Q10ME8	Haloacid dehalogenase/epoxide hydrolase
Os03g0311401	Conserved hypothetical protein.	AK242599	NP_001173394.1 (RefSeq)	-
Os03g0311600	Helix-loop-helix DNA-binding domain containing protein.	AK103709	A2XFX7	-
Os03g0312100	Hypothetical conserved gene.	ab initio prediction	Q10ME2	-
Os03g0312300	Protein of unknown function DUF26 domain containing protein.	AK111364	B8AN03	Gnk2-homologous domain
Os03g0312350	Hypothetical gene.	L14835	longestORF	-
Os03g0312500	Similar to SKIP interacting protein 23 (Fragment).	AK106657	B8Q8A9	-
Os03g0410300	Protein of unknown function DUF1313 family protein.	AK071843	C5WWN3	Protein of unknown function DUF1313
Os03g0410700	Similar to binding / catalytic.	AK240833	NP_001031269.1 (RefSeq)	NAD
Os03g0410900	Like-Sm ribonucleoprotein, core family protein.	AK243125	NP_001148720.1 (RefSeq)	Like-Sm ribonucleoprotein
Os03g0411000	Apoptosis inhibitory 5 family protein.	AK120207 ,AK072079	A3AJ11	-
Os03g0411100	Similar to Nuclear Y/CCAAT-box binding factor A subunit NF-YA.	AK071595	A6BLV2	CCAAT-binding transcription factor, subunit B
Os03g0411300	EF-Hand type domain containing protein.	AK241952	B6TEB1	Calcium-binding EF-hand
Os03g0411500	Peptidase S14, ClpP family protein.	AK058696 ,AK071057	B9F8Z4	Peptidase S14, ClpP
Os03g0411600	Similar to predicted protein.	AK374266	NP_001050350.1 (RefSeq)	Protein of unknown function DUF1195
Os03g0411800	Similar to Metal transport protein.	AK121551	Q852F6	Zinc/iron permease
Os03g0642450	Hypothetical conserved gene.	CT835137	NP_001055980.1 (RefSeq)	-
Os03g0642600	Similar to Protein kinase domain containing protein, expressed.	AK366256	Q2QM75	Protein kinase, catalytic domain
Os03g0642900	Similar to Hydrolase-like protein.	BT055664	B4FSG2	-
Os03g0643000	Similar to Hydrolase-like protein.	AK366873	B4FSG2	-
Os03g0643050	Transposase, IS4-like domain containing protein.	EU953241	NP_001176912.1 (RefSeq)	Transposase, IS4-like
Os03g0643100	Homeodomain-like containing protein.	AK106587	Q60DP5	Myb/SANT-like domain
Os03g0643250	Similar to Hydrolase-like protein.	EU969825	B4FSG2	-
Os03g0643300	Similar to Ornithine-oxo-acid transaminase.	AK108010	A7Y209	Aminotransferase class-III
Os03g0643433	Similar to J013022N21, full insert sequence.	EU948995	B7EAX3	Reverse transcriptase
Os03g0643522	Conserved hypothetical protein.	ab initio prediction	Q10G55	-
Os03g0643611	Hypothetical protein.	ab initio prediction	NONE	-
Os03g0643700	Similar to GST6 protein (EC 2.5.1.18).	AK110550	NP_001105714.1 (RefSeq)	Glutathione S-transferase, N-terminal
Os03g0643800	Similar to hydroquinone glucosyltransferase.	FP097203	NP_001148283.1 (RefSeq)	-
Os03g0643900	Conserved hypothetical protein.	AK069402	B9F0M2	-
Os03g0644000	Autophagy protein 6 domain containing protein.	AK099664 ,AK066359	Q4A194	Autophagy-related protein 6
Os03g0644200	Pentatricopeptide repeat domain containing protein.	AK073773	B6SWY6	Pentatricopeptide repeat
Os03g0644400	Amino acid permease.	AK067118	Q9LUH7	Amino acid transporter, transmembrane
Os03g0644600	Protein of unknown function DUF761, plant domain containing protein.	AK376083	Q60DN1	Protein of unknown function DUF761, plant
Os03g0644700	Conserved hypothetical protein.	AK072883	F2E977	-
Os03g0644850	Conserved hypothetical protein.	BT085651	NP_001169271.1 (RefSeq)	-
Os03g0645000	Similar to Expansin-B12.	CT835183	A2XK27	Major pollen allergen Lol pI
Os03g0645100	Similar to pyruvate dehydrogenase E1 component subunit beta.	AK103256	NP_001150473.1 (RefSeq)	Transketolase-like, pyrimidine-binding domain
Os03g0645200	Similar to Translation initiation factor eIF-2B delta subunit (eIF-2B GDP-GTP exchange factor).	AK060478	NP_001159071.1 (RefSeq)	Initiation factor 2B-related
Os03g0645250	Hypothetical protein.	tplb0051c11 (Wheat FLcDNA)	longestORF	-
Os03g0668400	Peptidase C12, ubiquitin carboxyl-terminal hydrolase 1 domain containing protein.	AK119454	NP_178033.1 (RefSeq)	Plant organelle RNA recognition domain
Os03g0668500	Conserved hypothetical protein.	AK064871	Q7Y198	-
Os03g0668900	Similar to acidic leucine-rich nuclear phosphoprotein 32 family member A.	AK108369	NP_001152645.1 (RefSeq)	Leucine-rich repeat
Os03g0669000	Similar to RNA helicase (Fragment).	AK067769	B6T5H9	RNA helicase, ATP-dependent, DEAD-box, conserved site
Os03g0669100	Similar to predicted protein.	AK063742	Q10FF9	DeoxyUTP pyrophosphatase
Os03g0669200	Similar to GTP binding protein2.	AK242811	E0AEC9	G-protein, beta subunit
Os03g0669300	Beta-1,3-glucanase-like protein.	AK120903	NP_001148381.1 (RefSeq)	Glycoside hydrolase, family 17
Os03g0669450	Hypothetical protein.	BT019229	longestORF	-
Os03g0669600	Conserved hypothetical protein.	AK073152	B8AP32	-
Os03g0669700	Non-protein coding transcript.	EU949000	NONE	-
Os03g0669800	Zinc finger, FYVE-type domain containing protein.	AK121639	Q7Y185	Zinc finger, FYVE-type
Os03g0669900	Similar to F-box domain containing protein.	tplb0002n20 (Wheat FLcDNA)	Q10L74	F-box domain, cyclin-like
Os03g0670000	Domain of unknown function DUF1713, mitochondria domain containing protein.	AK064735	Q6ASX3	Domain of unknown function DUF1713, mitochondria
Os03g0670100	Similar to ATP-binding protein of ABC transporter.	AK071650	Q10FF0	ABC transporter-like
Os03g0670200	Similar to YJR013Wp (Fragment).	AK069553	B6T639	Mannosyltransferase, DXD

Os03g0670366	Hypothetical protein.	tplb0049a10 (Wheat FLcDNA)	longestORF	-
Os03g0670700	Similar to Glycine-rich RNA-binding, abscisic acid-inducible protein.	AK059164	Q8VXC4	RNA recognition motif domain
Os03g0670800	Conserved hypothetical protein.	tplb0009g14 (Wheat FLcDNA)	Q6ASX8	-
Os03g0671000	Similar to Helix-loop-helix DNA-binding domain containing protein.	BT039963	Q10FE3	-
Os03g0811100	Similar to Magnesium-chelatase subunit chlD, chloroplast precursor (EC 6.6.1.1) (Mg-protoporphyrin IX chelatase) (Mg-chelatas	AK072463	O22437	Magnesium chelatase, ChlI subunit
Os03g0811200	BRCT domain containing protein.	AK069532	B8AMB9	BRCT
Os03g0811300	Similar to AP-1 complex subunit sigma-2.	AK071871	B6U542	Clathrin adaptor complex, small chain
Os03g0811400	Similar to DNA binding protein.	EU970714	NP_001151425.1 (RefSeq)	Helix-loop-helix DNA-binding
Os03g0811500	Zinc finger, HIT-type domain containing protein.	AK103214	B6SV90	PAPA-1-like conserved region
Os03g0811550	Protein of unknown function DUF617, plant domain containing protein.	AK332854	Q7XZH1	Protein of unknown function DUF617, plant
Os03g0811600	Similar to Peptidyl prolyl isomerase H.	AK071076	Q1KL26	Peptidyl-prolyl cis-trans isomerase, cyclophilin-type
Os03g0811700	Similar to Pre-mRNA branch site protein p14 (SF3B 14 kDa subunit).	AK121661 ,AK059288	B6T0W0	RNA recognition motif domain
Os03g0811800	Ribosomal protein L36 family protein.	AK063320	B8AM87	Ribosomal protein L36
Os03g0811900	Similar to ADP-ribosylation factor.	AK122007	Q6S4R7	Small GTP-binding protein domain
Os03g0812000	DNA topoisomerase, type IIA, subunit A or C-terminal domain containing protein.	AK059281	Q7XZF7	DNA gyrase/topoisomerase IV, subunit A, C-terminal beta-pinwheel
Os03g0812200	Zinc finger, RING/FYVE/PHD-type domain containing protein.	AK067585	B6T559	Zinc finger, RING-type
Os03g0812300	Hypothetical gene.	AK108409	longestORF	-
Os03g0812400	EF-Hand type domain containing protein.	AK119951	A2XNA2	Calcium-binding EF-hand
Os03g0812500	Non-protein coding transcript.	AK102548	NONE	-
Os04g0421700	FAR1 domain containing protein.	AK072998	Q01JZ9	FAR1 DNA binding domain
Os04g0421800	EF-Hand type domain containing protein.	AK068951	Q01JZ8	EF-hand-like domain
Os04g0421850	Hypothetical protein.	EU949573	longestORF	-
Os04g0421900	Similar to H0525E10.11 protein.	AK066958	Q01JZ7	Cullin repeat-like-containing domain
Os04g0422000	PAP fibrillin family protein.	AK070660	Q01JZ6	Plastid lipid-associated protein/fibrillin conserved domain
Os04g0422200	Similar to H0525E10.13 protein.	EU975418	Q01JZ5	Plastocyanin-like
Os04g0422300	Protein of unknown function DUF6, transmembrane domain containing protein.	AK107131	Q01JZ4	Drug/metabolite transporter
Os04g0422500	Similar to H0525E10.15 protein.	AK108092	Q01JZ3	-
Os04g0422600	Similar to H0525E10.16 protein.	AK099964	Q01JZ2	Drug/metabolite transporter
Os04g0422700	Similar to H0525E10.17 protein.	AK111485	Q01K08	-
Os04g0422900	Hypothetical conserved gene.	AK111398 (DDBJ, Secondary hit)	NP_001176912.1 (RefSeq)	-
Os04g0422950	Myb/SANT-like domain domain containing protein.	BT066123	NP_001176912.1 (RefSeq)	Myb/SANT-like domain
Os04g0423100	Monooxygenase, FAD-binding domain containing protein.	AK360863	B8ATR1	Monooxygenase, FAD-binding
Os04g0423200	Conserved hypothetical protein.	AK373784	NP_001173935.1 (RefSeq)	-
Os04g0423400	Similar to OSIGBa0076I14.3 protein.	AK104594 ,AK061494 ,AK103997 ,A	Q01L89	ABA/WDS induced protein
Os04g0423600	SET domain containing protein.	AK065331	Q01L87	SET domain
Os04g0423700	Similar to predicted protein.	AK288427	Q7X8A2	-
Os04g0423800	Peroxidase (EC 1.11.1.7).	AK108989	B6E1W9	Plant peroxidase
Os04g0424200	Similar to OSIGBa0076I14.10 protein.	AK362665	Q01L82	-
Os04g0425100	Zinc finger, RING/FYVE/PHD-type domain containing protein.	AK111894	Q01L80	Zinc finger, PHD-type
Os04g0425800	Hypothetical protein.	tplb0045k11 (Wheat FLcDNA)	longestORF	-
Os05g0186900	Similar to Auxin-responsive protein IAA16.	AK111403	P0C127	-
Os05g0187000	Beta 2 subunit of 20S proteasome (20S proteasome beta subunit).	AK103126	A2Y157	Peptidase T1A, proteasome beta-subunit
Os05g0187100	Similar to Hexokinase.	AK067988	Q1WM16	Hexokinase
Os05g0187500	IQ calmodulin-binding region domain containing protein.	tplb0011k15 (Wheat FLcDNA)	NP_001054836.1 (RefSeq)	IQ motif, EF-hand binding site
Os05g0187800	Similar to Derlin-1.	AK243296	Q06397	Derlin
Os05g0188100	Conserved hypothetical protein.	AK242429	NP_001054839.1 (RefSeq)	-
Os05g0188300	Uncharacterised protein family UPF0546 domain containing protein.	AK070957	B8AYW4	Uncharacterised protein family UPF0546
Os05g0188500	Similar to SCC3.	AK100219	Q6UB39	Armadillo-like helical
Os05g0188600	Conserved hypothetical protein.	AK111527	B9FMW0	-
Os05g0188700	Leucine-rich repeat domain containing protein.	AK243190	NP_001174255.1 (RefSeq)	Leucine-rich repeat
Os05g0188750	Hypothetical protein.	BT068200	longestORF	-
Os05g0188800	Similar to H0403D02.13 protein.	ab initio prediction	Q259S1	Coenzyme Q biosynthesis Coq4
Os05g0189300	Vegetative storage protein/acid phosphatase domain containing protein.	AK067327 ,AK060874 ,AK104624 ,A	B6TV55	Acid phosphatase
Os05g0189425	Conserved hypothetical protein.	CU406507	Q5Z550 "	-
Os06g0113900	Major facilitator superfamily, general substrate transporter domain containing protein.	AK120464	B7ETL1	Major facilitator superfamily domain, general substrate transporter
Os06g0113950	Hypothetical conserved gene.	AK065021	B9FR62	-
Os06g0114000	Hypothetical gene.	AK063734	longestORF	-
Os06g0114066	Hypothetical protein.	tplb0058a16 (Wheat FLcDNA)	longestORF	-
Os06g0114132	Non-protein coding transcript.	X06284	NONE	-
Os06g0114200	Similar to ATARFB1B%3B GTP binding.	ab initio prediction	NP_197208.1 (RefSeq)	Small GTPase superfamily, Rab type
Os06g0114300	Cyclin-like F-box domain containing protein.	AK242261	NP_001174567.1 (RefSeq)	-
Os06g0114366	Pentatricopeptide repeat domain containing protein.	AK360182	NP_001174568.1 (RefSeq)	Pentatricopeptide repeat
Os06g0114400	Conserved hypothetical protein.	AK335198	Q9LWT3	-
Os06g0114500	Similar to ATOZI1 protein (Stress-induced protein OZI1) (AT0ZI1 protein).	AK066771	NP_001151357.1 (RefSeq)	Protein of unknown function DUF1138
Os06g0114600	Conserved hypothetical protein.	AK067972	B9FR65	-
Os06g0114650	Similar to predicted protein.	AK240881	NP_565393.1 (RefSeq)	Shikimate kinase
Os06g0114651	Hypothetical gene.	EU970221	longestORF	-
Os06g0114700	Protein of unknown function DUF1218 family protein.	AK061552	B8B1L7	Protein of unknown function DUF1218
Os06g0114900	Conserved hypothetical protein.	AK071346	Q6ZDM2	-
Os06g0115000	Conserved hypothetical protein.	AK108096	A2Y8J6	-
Os06g0115100	Similar to ATOZI1 protein (Stress-induced protein OZI1) (AT0ZI1 protein).	AK059160	NP_001151357.1 (RefSeq)	Protein of unknown function DUF1138
Os06g0115200	Quinonprotein alcohol dehydrogenase-like domain containing protein.	AK243259	NP_001056610.2 (RefSeq)	-
Os06g0115300	Similar to Acyl-CoA-binding protein 2 (ACBP 2) (Fragment).	AK058833	B6SRG5	Acyl-CoA-binding protein, ACBP
Os06g0115400	Similar to Superoxide dismutase [Fe], chloroplast (EC 1.15.1.1) (Fragment).	AK111656	B8B1M4	Manganese/iron superoxide dismutase
Os06g0115500	Similar to 60S ribosomal protein L13 (BBC1 protein homolog).	AK070995	A2Y8K1	Ribosomal protein L13e
Os06g0115550	Hypothetical gene.	EU734565	longestORF	-
Os06g0115600	Plant-specific protein with a short C-terminal coiled-coil domain and a functional NLS, Common symbiosis signaling (SYM) patl	AK243034 ,EF569223	A9XMT5	-
Os06g0115700	Conserved hypothetical protein.	AK060414	B8B1M6	-
Os06g0115800	Hypothetical conserved gene.	AK069560	Q5VRL0	-
Os06g0116100	Hypothetical protein.	AK069526	GeneMark	-
Os06g0116300	Similar to Syntaxin.	AK243105	B4FBQ6	Target SNARE coiled-coil domain
Os06g0116400	Nickel/cobalt transporter, high-affinity domain containing protein.	AK105979	NP_565394.1 (RefSeq)	Nickel/cobalt transporter, high-affinity
Os06g0116500	Pleckstrin homology-type domain containing protein.	AK068442	Q5K2M9	Pleckstrin homology domain
Os06g0116600	Proteinase inhibitor, propeptide domain containing protein.	AK103500	NP_001150208.1 (RefSeq)	-
Os06g0116800	Similar to GFA2.	AK058985	B6TA99	Heat shock protein DnaJ, cysteine-rich domain
Os06g0116900	Conserved hypothetical protein.	AK063112	A2Y8L3	-
Os06g0116933	Conserved hypothetical protein.	EU964353	Q8H652	-
Os06g0117101	Hypothetical gene.	AK070750	longestORF	-
Os06g0117500	Hypothetical conserved gene.	AK067271 (DDBJ, Secondary hit)	B9FR86	-
Os06g0121000	Conserved hypothetical protein.	ab initio prediction	NP_001174573.1 (RefSeq)	-
Os06g0121200	Protein of unknown function DUF594 family protein.	tplb0058f06 (Wheat FLcDNA)	NP_001174574.1 (RefSeq)	Protein of unknown function DUF594
Os06g0121300	Conserved hypothetical protein.	AK064715	A2Y8P2	-
Os06g0121400	Conserved hypothetical protein.	FP093145	Q5VQ88	-
Os06g0121500	Peptidase A1 domain containing protein.	AK058425	Q5VQ87	Peptidase A1
Os06g0121600	Conserved hypothetical protein.	EU960106	NP_001056684.2 (RefSeq)	-
Os06g0121650	Conserved hypothetical protein.	AK064715	A2Y8P2	-
Os06g0121700	Conserved hypothetical protein.	AK252656	Q5VQ84	-
Os06g0121800	Hypothetical conserved gene.	AK372301	B8B1W8	Peptidase A1
Os06g0122001	Hypothetical protein.	AK242323	longestORF	-
Os06g0122200	Protein of unknown function DUF594 domain containing protein.	AK109712	A2Y8P7	Protein of unknown function DUF594
Os06g0122500	Conserved hypothetical protein.	AK108322	B8B1X0	-
Os06g0122600	Peptidase C48, SUMO/Sentrin/Ubl1 domain containing protein.	ab initio prediction	NP_001174577.1 (RefSeq)	Peptidase C48, SUMO/Sentrin/Ubl1
Os06g0122800	Hypothetical conserved gene.	AK287864	NP_001041816.1 (RefSeq)	Peptidase C48, SUMO/Sentrin/Ubl1
Os06g0123000	Conserved hypothetical protein.	AK108643	A2Y8Q3	-
Os06g0127900	Ribonucleotide reductase, Chloroplast biogenesis	EU602347 (Genbank),AK073063 (Gen	Q5VRJ6	-
Os06g0128000	Similar to formiminotransferase-like.	EU976079	NP_001148236.1 (RefSeq)	Formiminotransferase, N-terminal subdomain
Os06g0128100	Protein of unknown function DUF248, methyltransferase putative domain containing protein.	AK241973	Q8H636	Protein of unknown function DUF248, methyltransferase putative
Os06g0128150	Hypothetical protein.	tplb0038j22 (Wheat FLcDNA)	longestORF	-
Os06g0128200	LMBR1-like conserved region domain containing protein.	AK068886	NP_195766.1 (RefSeq)	LMBR1-like membrane protein
Os06g0128300	Similar to Mitochondrial half-ABC transporter.	AK072699	NP_200635.1 (RefSeq)	ABC transporter, transmembrane domain
Os06g0128400	WD40 repeat-like domain containing protein.	AK121983 ,AK064390	NP_001152334.1 (RefSeq)	WD40 repeat
Os06g0128450	Hypothetical gene.	BT084810	longestORF	-
Os06g0128500	Ribosomal protein L47, mitochondrial family protein.	AK122132 ,AK058563	NP_001147665.1 (RefSeq)	Ribosomal protein L29
Os06g0128600	Ankyrin repeat containing protein.	AK119921	NP_196344.2 (RefSeq)	-
Os06g0128700	Ankyrin repeat domain containing protein.	AK108566	A3B7Z9	Ankyrin repeat
Os06g0128800	C2 calcium/lipid-binding domain, CaLB domain containing protein.	AK110551	A2Y8U0	C2 calcium/lipid-binding domain, CaLB
Os06g0129000	Hypothetical conserved gene.	AK287686	Q658H9	-
Os06g0129100	Short-chain dehydrogenase/reductase SDR family protein.	AK243431	NP_001152242.1 (RefSeq)	Short-chain dehydrogenase/reductase SDR
Os06g0129200	BTB/POZ fold domain containing protein.	AK102435	A2Y8U3	BTB/POZ fold
Os06g0129300	Armadillo-like helical domain containing protein.	AK072613	Q5TJC3	Armadillo-like helical
Os06g0129400	Similar to cDNA clone:J013056J05, full insert sequence.	AK243620	A2Y8U6	SPX, N-terminal
Os06g0129600	Similar to Alpha-L-fucosidase 2.	AK375707	B6STS8	-
Os06g0129650	Hypothetical protein.	tplb0048c08 (Wheat FLcDNA)	longestORF	-
Os06g0129700	NUDIX hydrolase domain containing protein.	AK288101	NP_567384.1 (RefSeq)	NUDIX hydrolase domain
Os06g0129800	Hypothetical conserved gene.	AK334231	Q658H0	-
Os06g0129900	Similar to Cytochrome P450 CYPD.	AK066075	XP_002320071.1 (RefSeq)	Cytochrome P450
Os06g0130000	Similar to Tobacco mosaic virus helicase domain-binding protein (Fragment).	AK070084	NP_182074.4 (RefSeq)	ATPase, AAA+ type, core
Os06g0130100	Receptor-like kinase (RLK), Drought and salt stress tolerance	GQ423058	D0U6M6	-
Os06g0130200	Cell cycle regulated microtubule associated protein domain containing protein.	AK101132	B9FRB4	Cell cycle regulated microtubule associated protein
Os06g0130400	Similar to 1-aminocyclopropane-1-carboxylate synthase (EC 4.4.1.14) (Fragment).	AK065212	Q43201	1-aminocyclopropane-1-carboxylate synthase
Os06g0130500	Similar to Cyclophilin-like protein PPIL3b.	AK105360	A2Y8V7	Peptidyl-prolyl cis-trans isomerase, cyclophilin-type
Os06g0130600	GAGA binding-like family protein.	AK070616	B4FYC0	GAGA binding-like

Os06g0130800	Winged helix repressor DNA-binding domain containing protein.	AK069863	O24227	Histone H1/H5
Os06g0130900	Similar to Histone H3 (Fragment).	CT841989	D7SZ96	Histone H3
Os06g0131001	Conserved hypothetical protein.	BT016934	F2DKH6	-
Os06g0131100	Similar to guanine nucleotide-binding protein beta subunit-like protein 1.	AK101808	B8B2C2	WD40 repeat
Os06g0131300	Similar to Arginine decarboxylase (EC 4.1.1.19) (Fragment).	Q9SNN0	Q9SNN0	Ornithine/DAP/Arg decarboxylase
Os06g0131350	Hypothetical gene.	AK335026	longestORF	-
Os06g0131400	Non-protein coding transcript.	AK062712	NONE	-
Os06g0131500	Glycoside hydrolase, family 17 protein.	AK105303	B6T478	Glycoside hydrolase, family 17
Os06g0131700	Similar to NAM protein.	AK119784	Q9SNM6	No apical meristem
Os06g0131800	Hypothetical protein.	FP093663	longestORF	-
Os06g0131900	Non-protein coding transcript.	EU941912	NONE	-
Os06g0132000	Similar to H0315A08.1 protein.	FP099421	Q01I63	Ribonuclease H1, N-terminal
Os06g0132100	Transmembrane receptor, eukaryota domain containing protein.	AK072859 ,AK100206	B6TBV2	Transmembrane receptor, eukaryota
Os06g0132200	Non-protein coding transcript.	AK062713	NONE	-
Os06g0132300	Conserved hypothetical protein.	AK107873	A2Y8W8	-
Os06g0132400	Similar to Magnesium-protoporphyrin O-methyltransferase (EC 2.1.1.11) (Magnesium- protoporphyrin IX methyltransferase).	AK059151	E5GBL6	Magnesium-protoporphyrin IX methyltransferase, C-terminal
Os06g0132450	Non-protein coding transcript.	CT836189	NONE"	-
Os06g0132500	Similar to hydrolase, alpha/beta fold family protein.	ab initio prediction	NP_194145.1 (RefSeq)	-
Os06g0132550	Similar to hydrolase, alpha/beta fold family protein.	ab initio prediction	NP_194145.1 (RefSeq)"	-
Os06g0132600	Repair protein Rad1/Rec1 domain containing protein.	AK061226	NP_001149448.1 (RefSeq)	Repair protein Rad1/Rec1
Os06g0132801	Hypothetical conserved gene.	AK289264	Q5VRQ1	-
Os06g0133000	Granule-bound starch synthase I, chloroplast precursor (EC 2.4.1.21).	AK070431	D3U2H9	Glycosyl transferase, family 1
Os06g0133100	Hypothetical protein.	AK109227	longestORF	-
Os06g0133200	Conserved hypothetical protein.	AK108349	B6T051	-
Os06g0133300	Hypothetical conserved gene.	ab initio prediction	B8B285	-
Os06g0133400	Conserved hypothetical protein.	AK240870	Q5VRP7	-
Os06g0133500	Conserved hypothetical protein.	EU723765	NP_001056709.1 (RefSeq)	-
Os06g0133600	Phosphate-induced protein 1 conserved region family protein.	AK105690	NP_199968.1 (RefSeq)	Phosphate-induced protein 1
Os06g0133800	Similar to Transferase.	AK067452	Q7SIC9	Transketolase, N-terminal
Os06g0133900	Similar to 3-phosphoshikimate 1-carboxyvinyltransferase.	AK242404	Q93VK6	Enolpyruvate transferase domain
Os06g0134000	Similar to 40S ribosomal protein S20.	AK062184 ,AK120319	P35686	Ribosomal protein S10
Os06g0134050	Similar to EMB1067 (EMBRYO DEFECTIVE 1067)%3B tRNA 2'-phosphotransferase.	AK102796	NP_182058.2 (RefSeq)	Phosphotransferase KptA/Tpt1
Os06g0134200	Hypothetical conserved gene.	AK111432	B9FRC9	-
Os06g0134300	Protein of unknown function DUF3615 domain containing protein.	AK071534	B8B291	Protein of unknown function DUF3615
Os06g0134400	Protein of unknown function DUF3615 domain containing protein.	AK068098	B8B292	Protein of unknown function DUF3615
Os06g0134433	Non-protein coding transcript.	AK108212	NONE	-
Os06g0134466	Hypothetical conserved gene.	AK241422	Q5VNV4	Protein of unknown function DUF3615
Os06g0134500	Protein of unknown function DUF3615 domain containing protein.	AK063440	B8B293	Protein of unknown function DUF3615
Os06g0134700	Protein kinase, core domain containing protein.	AK103589	A3B850	Protein kinase, catalytic domain
Os06g0134750	Hypothetical gene.	BT065932	longestORF	-
Os06g0134800	Folate-binding, YgfZ domain containing protein.	AK063692	NP_001147280.1 (RefSeq)	Glycine cleavage T-protein, N-terminal
Os06g0134900	Conserved hypothetical protein.	AK103205	B8B295	-
Os06g0154800	Hypothetical conserved gene.	AK072563	B9GCU9	-
Os06g0154900	Similar to RING-type E3 ubiquitin ligase.	AK101097	B8YPX6	-
Os06g0155050	Hypothetical protein.	EU947580	longestORF	-
Os06g0155300	IQ calmodulin-binding region domain containing protein.	AK099462	B6TIB2	IQ motif, EF-hand binding site
Os06g0155400	Conserved hypothetical protein.	AK361186	B9FRK4	-
Os06g0155500	Transferase family protein.	AK363829	NP_001056851.1 (RefSeq)	Transferase
Os06g0155600	ATPase, AAA-type, core domain containing protein.	AK101138	NP_177460.2 (RefSeq)	ATPase, AAA+ type, core
Os06g0155750	Hypothetical conserved gene.	AB332077	B9FRK5	-
Os06g0155900	Conserved hypothetical protein.	AK060403	Q5VMB1	-
Os06g0156400	Similar to carboxylic ester hydrolase/ hydrolase, acting on ester bonds.	AK334535	NP_001148234.1 (RefSeq)	Lipase, GDSL
Os06g0156501	Conserved hypothetical protein.	CT836282	Q6I543 "	-
Os06g0156600	Lipase, GDSL domain containing protein.	AK121780 ,AK061497 ,AK104394 ,A	NP_001142390.1 (RefSeq)	Lipase, GDSL
Os06g0156650	Hypothetical protein.	tplb0048c08 (Wheat FLcDNA)	longestORF	-
Os06g0156700	Lipase, GDSL domain containing protein.	AK107226	B6STS8	Lipase, GDSL
Os06g0156801	Hypothetical protein.	tplb0048c08 (Wheat FLcDNA)	longestORF	-
Os06g0156900	Glycosyl transferase, family 31 protein.	AK110688	NP_001152267.1 (RefSeq)	Glycosyl transferase, family 31
Os06g0157000	Esterase, SGNH hydrolase-type domain containing protein.	AK066113	B6TLP8	Lipase, GDSL
Os06g0157125	Hypothetical protein.	EU950608	longestORF	-
Os06g0157500	Similar to CiFT protein.	AB062676	E5RQ92	Phosphatidylethanolamine-binding, conserved site
Os06g0158000	Similar to Acyl-CoA synthetase (EC 6.2.1.3).	AK063337	NP_001147847.1 (RefSeq)	AMP-dependent synthetase/ligase
Os06g0158050	Hypothetical protein.	BT024042	longestORF	-
Os06g0158100	Similar to WRKY transcription factor 63.	AY676932	A1C0L7	DNA-binding WRKY
Os06g0158200	Sulfotransferase domain containing protein.	AK121981	B9FRL5	Sulfotransferase
Os06g0158300	NB-ARC domain containing protein.	AK073612	C0LHH5	Disease resistance protein
Os06g0158400	Hypothetical conserved gene.	AK287627	NP_001174610.1 (RefSeq)	NB-ARC
Os06g0158500	Conserved hypothetical protein.	AK101823	B8B319	-
Os06g0158600	Protein of unknown function DUF652 family protein.	AK059986 ,AK101378	B4FS24	Nucleotide binding protein, PINc
Os06g0158700	Glyoxalase/bleomycin resistance protein/dioxygenase domain containing protein.	AK103202	NP_001147921.1 (RefSeq)	-
Os06g0158800	Protein of unknown function DUF1191 family protein.	AK070453	A2Y9L3	Protein of unknown function DUF1191
Os06g0158850	Non-protein coding transcript.	AK241360	NONE	-
Os06g0158900	Similar to Multidrug-resistance associated protein 3.	AK368184	Q6J0P5	ABC transporter, transmembrane domain
Os06g0159400	Tetratricopeptide-like helical domain containing protein.	AK101286	A2Y9L5	U box domain
Os06g0159450	Similar to Histone H3.	FP099648	B9FRM3	-
Os06g0159600	Tetratricopeptide-like helical domain containing protein.	AK068486	B9FRM1	U box domain
Os06g0159700	Similar to histone H3.	BT018930	XP_002299242.1 (RefSeq)	-
Os06g0159725	Similar to histone H3.	A2XHJ3	XP_002299242.1 (RefSeq)	Histone H3
Os06g0159750	Hypothetical conserved gene.	EU964122	Q01I89	-
Os06g0163900	Conserved hypothetical protein.	AK102733	B9FRN9	-
Os06g0164000	Zinc finger, MIZ-type domain containing protein.	AK069464	B8B381	Zinc finger, MIZ-type
Os06g0164100	Similar to Serine/threonine protein phosphatase PP1 (EC 3.1.3.16).	AK064345 ,AK102278	B8B382	-
Os06g0164300	Similar to H0502G05.11 protein.	CT836200	Q01JF5	-
Os06g0164400	Similar to predicted protein.	AK243447	Q5VRS4	Helix-loop-helix DNA-binding
Os06g0164500	Conserved hypothetical protein.	AK355771	NP_001056903.1 (RefSeq)	-
Os06g0164600	Hypothetical gene.	AK102554	longestORF	-
Os06g0164800	Hypothetical conserved gene.	AK108018	B9FRP3	-
Os06g0164900	Similar to OSIGBa0147J19.7 protein.	AK369457	Q01KM2	Protein kinase, catalytic domain
Os06g0165000	Hypothetical protein.	AK064542	longestORF	-
Os06g0165100	Non-protein coding transcript.	AK109271	NONE	-
Os06g0165300	Transposase (putative), gypsy type domain containing protein.	tplb0014k20 (Wheat FLcDNA)	NP_001056907.1 (RefSeq)	Transposase
Os06g0165500	Similar to OSIGBa0147J19.7 protein.	AK365081	Q01KM2	Protein kinase, catalytic domain
Os06g0165600	CRT/DRE binding factor (Transcription factor RCBF4).	AF243384	Q6J1A5	Pathogenesis-related transcriptional factor/ERF, DNA-binding
Os06g0165800	Similar to Caffeoyl-CoA 3-O-methyltransferase (Fragment).	AK065744	A9X9L7	O-methyltransferase, family 3
Os06g0165900	Conserved hypothetical protein.	ab initio prediction	NP_001174617.1 (RefSeq)	-
Os06g0166000	Cyclin-like F-box domain containing protein.	AK060317	NP_001151406.1 (RefSeq)	F-box domain, cyclin-like
Os06g0166050	Hypothetical gene.	EU941382	longestORF	-
Os06g0166100	FAR1 domain containing protein.	AK066174	B8B386	FAR1 DNA binding domain
Os06g0166200	Zinc finger, C2H2-type domain containing protein.	AK106360	NP_001150644.1 (RefSeq)	Zinc finger, C2H2
Os06g0166400	Similar to TINY-like protein (AP2 domain containing protein RAP2.10) (Fragment).	AK107775	NP_001147519.1 (RefSeq)	Pathogenesis-related transcriptional factor/ERF, DNA-binding
Os06g0166500	Similar to Auxin-responsive protein IAA20 (Indoleacetic acid-induced protein 20).	AK102541	Q5VRR0	AUX/IAA protein
Os06g0166900	Protein kinase, core domain containing protein.	AK058497	B6T5E9	Protein kinase, catalytic domain
Os06g0167000	Similar to predicted protein.	AK065529	NP_178124.1 (RefSeq)	PROCN
Os06g0167100	Armadillo-like helical domain containing protein.	AK101686	B6SV75	Clathrin/coatomer adaptor, adaptin-like, N-terminal
Os06g0167125	Non-protein coding transcript.	BT016958	NONE	-
Os06g0167150	Hypothetical gene.	AK242224	NP_001174618.1 (RefSeq)	-
Os06g0167200	Similar to RING-H2 finger protein ATL1R (RING-H2 finger protein ATL8).	AK100240	A2Y9S1	Zinc finger, RING-type
Os06g0167400	Di-trans-poly-cis-decaprenylcistransferase family protein.	AK102123	B6TD36	Di-trans-poly-cis-decaprenylcistransferase-like
Os06g0167500	Leucine-rich repeat, plant specific containing protein.	AK069490	B8B391	Leucine-rich repeat
Os06g0167600	Similar to Proteasome subunit alpha-3 (Fragment).	AK067977	P0C8Y9	Proteasome, alpha-subunit, conserved site
Os06g0168000	Glutathione S-transferase, C-terminal-like domain containing protein.	AK059250 ,AK099356	Q5VRG1	Glutathione S-transferase, C-terminal-like
Os06g0168150	Hypothetical gene.	CU405671	longestORF	-
Os06g0168400	RNA polymerase Rpb7, N-terminal domain containing protein.	AK109865	NP_565115.1 (RefSeq)	RNA polymerase Rpb7, N-terminal
Os06g0168500	Similar to Syntaxin-like protein (Fragment).	AK100316	NP_568187.1 (RefSeq)	Target SNARE coiled-coil domain
Os06g0168600	Ribonucleotide reductase, Chloroplast biogenesis	EU602344 (Genbank),AK068858 (Gen	B2M117 ,Q5VRF5	-
Os06g0168700	Similar to Prolin rich protein.	AK103637 ,AK101305	NP_001149720.1 (RefSeq)	Plant lipid transfer protein/seed storage/trypsin-alpha amylase inhibitor
Os06g0168800	Similar to Protein kinase.	AK111753	C6ZRY2	Protein kinase, catalytic domain
Os06g0168901	Hypothetical conserved gene.	CT837860	Q5VRF0	X8
Os06g0169001	GOS9 protein.	AK243234	P0C5C6	Mannose-binding lectin
Os06g0171500	Conserved hypothetical protein.	AK073301	A2Y9U5	-
Os06g0171600	Membrane insertion protein, OxaA/YidC domain containing protein.	AK105530	B9FRR4	Membrane insertion protein, OxaA/YidC
Os06g0171700	Cdk-activating kinase assembly factor (MAT1) family protein.	AK103771	NP_001150873.1 (RefSeq)	Cdk-activating kinase assembly factor
Os06g0171800	VRR-NUC domain containing protein.	AK120233	NP_001117447.1 (RefSeq)	Zinc finger, Rad18-type putative
Os06g0171900	WD40 subfamily protein, Salt stress	AK100886 (Genbank)	Q5SND4	WD40 repeat
Os06g0172000	Pentatricopeptide repeat domain containing protein.	AK068738	B8B3E9	Pentatricopeptide repeat
Os06g0172101	Hypothetical gene.	EU951657	longestORF	-
Os06g0172200	EF-Hand type domain containing protein.	AK105920 ,AK070725	Q5SND2	Calcium-binding EF-hand
Os06g0172600	Similar to predicted protein.	CU405936	B6SLI1	Ribosomal protein S30
Os06g0172675	Hypothetical protein.	BT086967	longestORF	-
Os06g0172750	Non-protein coding transcript.	AK242296	NONE	-
Os06g0172800	Similar to alkaline alpha galactosidase 2.	BT061992	NP_001105794.1 (RefSeq)	Raffinose synthase
Os06g0172901	Hypothetical protein.	tplb0060c15 (Wheat FLcDNA)	longestORF	-

Os06g0173000	Armadillo-type fold domain containing protein.	ab initio prediction	A3B8V4	Armadillo-type fold
Os06g0173100	Similar to 26S protease regulatory subunit 6A homolog (TAT-binding protein homolog 1) (TBP-1) (Mg(2+)-dependent ATPase 1	AK065612	P46465	ATPase, AAA+ type, core
Os06g0173450	Hypothetical protein.	BT085228	longestORF	-
Os06g0173800	SANT domain, DNA binding domain containing protein.	EU951889	Q5SNB3	SANT domain, DNA binding
Os06g0174300	Similar to predicted protein.	AK242396	Q2MGR0	ParB-like nuclease
Os06g0174350	Hypothetical conserved gene.	AK240737	Q658G1	-
Os06g0174375	Conserved hypothetical protein.	AK242835	NP_001174628.1 (RefSeq)	-
Os06g0174400	Similar to Vesicle-associated membrane protein 712 (AtVAMP712).	AK065311	B6U726	Synaptobrevin
Os06g0174700	Hypothetical protein.	AK099635	longestORF	-
Os06g0174800	Conserved hypothetical protein.	tplb0014k20 (Wheat FLcDNA)	A2Y9X0	-
Os06g0174900	Conserved hypothetical protein.	tplb0014k20 (Wheat FLcDNA)	Q658F8	-
Os06g0175400	Hypothetical conserved gene.	tplb0015n10 (Wheat FLcDNA)	B4FNU7	BSD
Os06g0175500	Epsin-like, N-terminal domain containing protein.	AK100481	NP_198370.1 (RefSeq)	ENTH/VHS
Os06g0175800	Similar to Cystathionine beta-lyase, chloroplast precursor (EC 4.4.1.8) (CBL) (Beta-cystathionase) (Cysteine lyase).	AK119521	B4FU01	Cys/Met metabolism, pyridoxal phosphate-dependent enzyme
Os06g0175900	Similar to Cytochrome b-c1 complex subunit 8.	AK120341 ,AK119716 ,AK243475	D6BQP2	UcrQ
Os06g0176000	Proteasome subunit alpha type 4 (EC 3.4.25.1) (20S proteasome alpha subunit C) (20S proteasome subunit alpha-3).	AK059984	P0C1G8	Proteasome, alpha-subunit, conserved site
Os06g0176100	Similar to H0201G08.5 protein.	CT836215	Q25AD8	Exostosin-like
Os06g0176125	Hypothetical protein.	tplb0040n23 (Wheat FLcDNA)	longestORF	-
Os06g0176150	Similar to Ribosomal protein S15 containing protein.	ab initio prediction	B6TK08	Ribosomal protein S15
Os06g0176200	Glycosyl transferase, family 31 protein.	AK058356 ,AK102880	Q7FN01	Glycosyl transferase, family 31
Os06g0176300	2OG-Fe(II) oxygenase domain containing protein.	CT837739	NP_001152138.1 (RefSeq)	Oxoglutarate/iron-dependent oxygenase
Os06g0176500	Similar to leucoanthocyanidin dioxygenase.	ab initio prediction	NP_001152138.1 (RefSeq)	Oxoglutarate/iron-dependent oxygenase
Os06g0176600	Hypothetical conserved gene.	AK062794	Q658D9	-
Os06g0176650	Similar to Leucoanthocyanidin dioxygenase.	ab initio prediction	B6SSL8	Oxoglutarate/iron-dependent oxygenase
Os06g0176700	Isopenicillin N synthase family protein.	AK241948	NP_001152138.1 (RefSeq)	Isopenicillin N synthase
Os06g0176750	Hypothetical protein.	AK373427	longestORF	-
Os06g0176801	Hypothetical protein.	AK373427	longestORF	-
Os06g0176850	Similar to leucoanthocyanidin dioxygenase.	CU406252	B6SSL8	-
Os06g0176900	Similar to Cystathionine gamma-synthase.	ab initio prediction	B4FU01	Cys/Met metabolism, pyridoxal phosphate-dependent enzyme
Os06g0177000	Similar to Cytochrome b-c1 complex subunit 8.	AK120341 ,AK119716 ,AK243475	D6BQP2	UcrQ
Os06g0177100	Proteasome subunit alpha type 4 (EC 3.4.25.1) (20S proteasome alpha subunit C) (20S proteasome subunit alpha-3).	AK059984	P0C1G8	Proteasome, alpha-subunit, conserved site
Os06g0177200	Similar to H0201G08.5 protein.	CT836215	Q25AD8	Exostosin-like
Os06g0177225	Hypothetical protein.	tplb0040n23 (Wheat FLcDNA)	longestORF	-
Os06g0177250	Similar to Ribosomal protein S15 containing protein.	ab initio prediction	B6TK08	Ribosomal protein S15
Os06g0177300	Glycosyl transferase, family 31 protein.	AK058356 ,AK102880	Q7FN01	Glycosyl transferase, family 31
Os06g0177600	2OG-Fe(II) oxygenase domain containing protein.	CT837739	NP_001152138.1 (RefSeq)	Oxoglutarate/iron-dependent oxygenase
Os06g0177700	Similar to leucoanthocyanidin dioxygenase.	ab initio prediction	NP_001152138.1 (RefSeq)	Oxoglutarate/iron-dependent oxygenase
Os06g0178400	Hypothetical conserved gene.	AK062794	Q658D9	-
Os06g0178450	Similar to Leucoanthocyanidin dioxygenase.	ab initio prediction	B6SSL8	Oxoglutarate/iron-dependent oxygenase
Os06g0178500	Isopenicillin N synthase family protein.	AK241948	NP_001152138.1 (RefSeq)	Isopenicillin N synthase
Os06g0178550	Hypothetical protein.	AK373427	longestORF	-
Os06g0178601	Hypothetical protein.	AK373427	longestORF	-
Os06g0178650	Similar to leucoanthocyanidin dioxygenase.	CU406252	B6SSL8	-
Os06g0178700	Similar to Iron/ascorbate-dependent oxidoreductase.	AK102979	NP_001152138.1 (RefSeq)	Oxoglutarate/iron-dependent oxygenase
Os06g0178800	Hypothetical protein.	AK373427	longestORF	-
Os06g0178900	Vacuolar H+-pyrophosphatase (EC 3.6.1.1) (Ovp2).	AK066933	P93410	Pyrophosphate-energised proton pump
Os06g0178950	Hypothetical protein.	tplb0034o06 (Wheat FLcDNA)	longestORF	-
Os06g0179000	Glycoside hydrolase family 79, N-terminal protein.	AK065263	A2Y9Y7	Glycoside hydrolase, family 79
Os06g0179050	Hypothetical gene.	BT085373	longestORF	-
Os06g0179100	Similar to H0306F03.13 protein.	EU963950	Q25A62	Sterile alpha motif/pointed domain
Os06g0179200	Similar to Nodulin-like protein.	AK101156	Q8H6R3	Nodulin-like
Os06g0179400	Hypothetical protein.	AK103645	longestORF	-
Os06g0179500	Protein of unknown function DUF247, plant family protein.	ab initio prediction	NP_001056969.1 (RefSeq)	Protein of unknown function DUF247, plant
Os06g0179700	Similar to DNA-binding protein phosphatase 2C.	AK065602	C4JAG1	Protein phosphatase 2C, manganese/magnesium aspartate binding site
Os06g0179800	Leucine-rich repeat, N-terminal domain containing protein.	AK121252	A2Y9Z2	Leucine-rich repeat
Os06g0179900	Major facilitator superfamily protein.	AK287929	NP_001147465.1 (RefSeq)	Major facilitator superfamily
Os06g0179950	Hypothetical protein.	AK369010	longestORF	-
Os06g0180000	Similar to Root determined nodulation 1.	AK069615	E9KID2	-
Os06g0180050	Hypothetical protein.	tplb0025h12 (Wheat FLcDNA)	longestORF	-
Os06g0180100	Hypothetical conserved gene.	ab initio prediction	Q8H604	-
Os06g0180300	Conserved hypothetical protein.	ab initio prediction	Q5SMJ6	-
Os06g0180533	Similar to predicted protein.	EU943442	XP_002321824.1 (RefSeq)	Cysteine, histidine-dependent amidohydrolase/peptidase
Os06g0180666	Hypothetical gene.	BT055164	longestORF	-
Os06g0181100	Octicosapeptide/Phox/Bem1p domain containing protein.	AK073372	NP_001056976.1 (RefSeq)	Phox/Bem1p
Os06g0225100	Mitochondrial transcription termination factor-related family protein.	AK071845	B8B482	Mitochondrial transcription termination factor-related
Os06g0225200	Mitochondrial transcription termination factor-related family protein.	AK104692 ,AK067095	A2YAW2	Mitochondrial transcription termination factor-related
Os06g0225300	Similar to SERK1 (Fragment).	AK100258	E2J573	Protein kinase, catalytic domain
Os06g0225350	Hypothetical conserved gene.	EU942158	B6TW49	-
Os06g0225400	Conserved hypothetical protein.	AK108638	B8B484	-
Os06g0225600	Conserved hypothetical protein.	AK109260	B8B485	-
Os06g0225800	Shikimate kinase domain containing protein.	AK109730	Q5NTH4	Shikimate kinase
Os06g0225900	Similar to ATP binding / ATPase/ nucleoside-triphosphatase/ nucleotide binding.	AK058549	NP_188608.3 (RefSeq)	ATPase, AAA+ type, core
Os06g0226000	Similar to Transposase (Fragment).	AK121923	B9FSA9	Putative harbinger transposase-derived nuclease
Os06g0226050	Similar to Salt-stress induced protein (Salt protein).	ab initio prediction	A2WPN7	Mannose-binding lectin
Os06g0226083	Hypothetical protein.	EU974948	longestORF	-
Os06g0226100	Glutaredoxin domain containing protein.	ab initio prediction	NP_001057202.2 (RefSeq)	Glutaredoxin
Os06g0226500	Helix-loop-helix protein, Brassinosteroid signaling	AK071601 (Genbank)	Q0DDF6	Helix-loop-helix DNA-binding
Os06g0226600	TB2/DP1 and HVA22 related protein family protein.	AK102752	B6UCA0	TB2/DP1/HVA22-related protein
Os06g0226700	Similar to PCF1.	AK064240	Q9C9L2	Transcription factor, TCP
Os06g0298200	Similar to CCT motif family protein.	AK098865	F1DK44	Zinc finger, B-box
Os06g0298400	WW/Rsp5/WWP domain containing protein.	AK066952	B9FSV3	WW/Rsp5/WWP
Os06g0298500	SEC-C motif domain containing protein.	AK108252	B8B0V5	SEC-C motif
Os06g0298700	Short-chain dehydrogenase/reductase SDR domain containing protein.	FP096368	B8B0V6	Short-chain dehydrogenase/reductase SDR
Os06g0299100	Glucose/ribitol dehydrogenase family protein.	AK066533	Q01MW5	Short-chain dehydrogenase/reductase SDR
Os06g0299200	Similar to H0207B04.1 protein.	ab initio prediction	NP_001057446.2 (RefSeq)	Short-chain dehydrogenase/reductase SDR
Os06g0299300	Short-chain dehydrogenase/reductase SDR domain containing protein.	AK060501 ,AK067625	Q01MW5	-
Os06g0299400	Non-protein coding transcript.	BT086891	NONE	-
Os06g0299500	Similar to J023036E09, full insert sequence (Fragment).	AJ495773	B7EH87 "	-
Os06g0300000	Short-chain dehydrogenase/reductase SDR domain containing protein.	AK109790	Q01MW5	Short-chain dehydrogenase/reductase SDR
Os06g0300100	Hypothetical protein.	tplb0031o17 (Wheat FLcDNA)	longestORF	-
Os06g0300200	Similar to H0207B04.1 protein.	AK375094	Q01MW5	Short-chain dehydrogenase/reductase SDR
Os06g0300300	SFT2-like family protein.	AK068813 ,AK109425	NP_001149600.1 (RefSeq)	Vesicle transport protein, Got1/SFT2-like
Os06g0300333	Hypothetical gene.	BT009643	longestORF	-
Os06g0300366	Hypothetical gene.	AK288218	longestORF	-
Os06g0300500	Steroid nuclear receptor, ligand-binding domain containing protein.	AK111400	B9FSW0	-
Os06g0300600	Similar to Decoy.	AK060668	NP_172914.1 (RefSeq)	Ribosomal protein L46
Os06g0300700	Protein phosphatase 4 core regulatory subunit R2 domain containing protein.	AK071388	A2YC20	Protein phosphatase 4 core regulatory subunit R2
Os06g0300800	Hypothetical conserved gene.	AK111728	Q5ZA32	WD40 repeat
Os06g0301000	Zinc finger, RING/FYVE/PHD-type domain containing protein.	AK073960	XP_002882977.1 (RefSeq)	Zinc finger, RING-type
Os06g0301100	Similar to OSIGBa0124N08.6 protein.	AK242142	B8B0W5	Uncharacterised protein family UPF0136, Transmembrane
Os06g0301300	Similar to predicted protein.	CT836217	NP_187941.5 (RefSeq)	-
Os07g0124500	Similar to Eukaryotic translation initiation factor 3 subunit 8 (eIF3 p110) (eIF3c).	AK070529 ,AK101000	Q1SL20	Proteasome component
Os07g0124600	Nucleotide-binding, alpha-beta plait domain containing protein.	AK073437	A2YHR7	RNA recognition motif domain
Os07g0124650	Hypothetical gene.	EU942577	longestORF	-
Os07g0124700	Similar to PLETHORA1.	AK109848	Q84Z02	Pathogenesis-related transcriptional factor/ERF, DNA-binding
Os07g0124725	Hypothetical protein.	EU949353	longestORF	-
Os07g0124750	Similar to cDNA clone:J033097L05, full insert sequence.	AK358114	B7F7S0	Glycosyl transferase, family 2
Os07g0124800	Similar to Chaperone protein dnaJ.	AK068837	B6U0R7	Heat shock protein DnaJ, N-terminal
Os07g0124900	Allergen V5/Tpx-1 related family protein.	AK060057	D5KR57	Allergen V5/Tpx-1-related
Os07g0125000	Allergen V5/Tpx-1 related family protein.	AK060005 ,AK104140	Q8LLU7	-
Os07g0125201	Allergen V5/Tpx-1 related family protein.	DQ167191	O04000	Allergen V5/Tpx-1-related
Os07g0125500	Allergen V5/Tpx-1 related family protein.	AK060057	D5KR57	Allergen V5/Tpx-1-related
Os07g0125600	Allergen V5/Tpx-1 related family protein.	AK060005 ,AK104140	Q8LLU7	-
Os07g0126100	Allergen V5/Tpx-1 related family protein.	DQ167191	O04000	Allergen V5/Tpx-1-related
Os07g0126301	Allergen V5/Tpx-1 related family protein.	AK060057	D5KR57	Allergen V5/Tpx-1-related
Os07g0126401	Allergen V5/Tpx-1 related family protein.	AK060005 ,AK104140	Q8LLU7	-
Os07g0126500	Allergen V5/Tpx-1 related family protein.	DQ167191	O04000	Allergen V5/Tpx-1-related
Os07g0129200	Similar to Pathogenesis-related protein PR1a.	AK243664	A0N0C1	Allergen V5/Tpx-1-related
Os07g0129300	Similar to PR1a protein.	CT841995	Q8LLU7	Allergen V5/Tpx-1-related
Os07g0129700	OSH15 protein (Homeobox gene).	AK111877	B9FVB7	Homeobox
Os07g0129800	Similar to lectin-like receptor kinase 7.	AK360292	NP_001147473.1 (RefSeq)	Protein kinase, catalytic domain
Os07g0129900	Similar to lectin-like receptor kinase 7.	tplb0022b01 (Wheat FLcDNA)	NP_001147990.1 (RefSeq)	Protein kinase, catalytic domain
Os07g0130000	F-box domain, Skp2-like domain containing protein.	ab initio prediction	Q84ZI5	F-box domain, Skp2-like
Os07g0130100	Similar to Resistance protein candidate (Fragment).	AK069990	A2YHU5	Protein kinase, catalytic domain
Os07g0130200	Similar to Resistance protein candidate (Fragment).	AK068307	NP_001147990.1 (RefSeq)	Protein kinase, catalytic domain
Os07g0130400	Similar to Lectin-like receptor kinase 7%3B2.	AK069319	NP_001147990.1 (RefSeq)	Protein kinase, catalytic domain
Os07g0130450	Hypothetical protein.	EU956864	longestORF	-
Os07g0130500	Hypothetical protein.	AK066229	longestORF	-
Os07g0130800	Protein kinase, catalytic domain domain containing protein.	AK067638 ,AK066399	Q84ZH6	Protein kinase, catalytic domain
Os07g0131001	Hypothetical protein.	AK376570	longestORF	-
Os07g0131250	Similar to Hexose transporter HT2.	ab initio prediction	NP_001148202.1 (RefSeq)	Sugar/inositol transporter
Os07g0133100	Concanavalin A-like lectin/glucanase, subgroup domain containing protein.	AK243480	NP_001175043.1 (RefSeq)	Protein kinase, catalytic domain

Os07g0133500	Protein of unknown function DUF1005 family protein.	AK106244	A2YHW7	Protein of unknown function DUF1005
Os07g0133601	Hypothetical protein.	tplb0021a06 (Wheat FLcDNA)	longestORF	-
Os07g0133700	Similar to FK506 binding protein.	AK240803	B4FK23	Peptidyl-prolyl cis-trans isomerase, FKBP-type, domain
Os07g0134000	Similar to Amino acid permease-like protein (Fragment).	AK120257 ,AK106110	Q7Y076	-
Os07g0134101	Hypothetical protein.	tplb0036o08 (Wheat FLcDNA)	longestORF	-
Os07g0134200	Receptor-like protein kinase 3.	AK243601	Q9M6D8	Protein kinase, catalytic domain
Os07g0134400	Similar to bacterial transferase hexapeptide repeat-containing protein.	AK073204	XP_002867858.1 (RefSeq)	Bacterial transferase hexapeptide repeat
Os07g0134500	Similar to hydrolase/ protein serine/threonine phosphatase.	AK069557	NP_194031.5 (RefSeq)	Metallophosphoesterase domain
Os07g0134600	Serine/threonine protein kinase domain containing protein.	AK240859	B6SSJ5	Protein kinase, catalytic domain
Os07g0134650	Hypothetical protein.	tplb0046o04 (Wheat FLcDNA)	longestORF	-
Os07g0134700	Mitochodrial transcription termination factor-related family protein.	tplb0010e17 (Wheat FLcDNA)	NP_001152167.1 (RefSeq)	Mitochodrial transcription termination factor-related
Os07g0134800	Similar to Succinate dehydrogenase [ubiquinone] flavoprotein subunit, mitochondrial (EC 1.3.5.1) (FP) (Flavoprotein subunit of c	AK072048 ,AK065901 ,AK099460	Q6ZDY8	Fumarate reductase/succinate dehydrogenase, FAD-binding site
Os07g0135550	Non-protein coding transcript.	BT019039	NONE	-
Os07g0139000	Putative zinc finger CCCH domain-containing protein 48.	Q8GVZ8	Q8GVZ8	Zinc finger, CCCH-type
Os07g0139300	Similar to G1 protein.	AB512480	D1MYE4	Domain of unknown function DUF640
Os07g0139400	NAD(P)-binding domain containing protein.	AK065558	Q2LC83	NAD-dependent epimerase/dehydratase
Os07g0139500	Conserved hypothetical protein.	AK065452	A2YI13	-
Os07g0139600	Non-protein coding transcript.	AK103216	NONE	-
Os07g0139700	Similar to nucleosome/chromatin assembly factor 104.	EU976876	B4FZY9	Histone-binding protein RBBP4
Os07g0140232	Similar to Thaumatin-like protein 1.	BT086406	NP_177640.1 (RefSeq)	Thaumatin, pathogenesis-related
Os07g0140566	Similar to hydrolase/ protein serine/threonine phosphatase.	tplb0052b08 (Wheat FLcDNA)	NP_192917.2 (RefSeq)	-
Os07g0140633	Hypothetical gene.	CT836129	longestORF	-
Os07g0141100	Protein kinase, core domain containing protein.	AK106343	A2YI18	Protein kinase, catalytic domain
Os07g0141400	Similar to 23 kDa polypeptide of photosystem II.	AK065248	O65103	Photosystem II PsbP, oxygen evolving complex
Os07g0141450	Conserved hypothetical protein.	AK375155	B8B758	-
Os07g0141500	Zinc finger, SWIM-type domain containing protein.	AK068893	B8B758	Zinc finger, PMZ-type
Os07g0141600	Similar to Serologically defined breast cancer antigen NY-BR-99.	AK103156	A2Q2G1	Pre-mRNA processing ribonucleoprotein, snoRNA-binding domain
Os07g0141650	Non-protein coding transcript.	BT016977	NONE	-
Os07g0141700	NB-ARC domain containing protein.	AK369881	NP_001058866.1 (RefSeq)	NB-ARC
Os07g0141900	Similar to OSIGBa0148A10.13 protein.	tplb0054c18 (Wheat FLcDNA)	Q01K85	Disease resistance protein
Os07g0142000	Reticulon family protein.	AK099414 ,AK059877	B6T4A9	Reticulon
Os07g0142050	Non-protein coding transcript.	BT019310	NONE	-
Os07g0142100	Conserved hypothetical protein.	CT836140	Q8H4K5	-
Os07g0142200	Conserved hypothetical protein.	AK108897	A3BGH3	-
Os07g0142300	Similar to Mucin-2.	AK108823	B6SR31	-
Os07g0142500	Conserved hypothetical protein.	CU405778	NP_001058871.1 (RefSeq)	-
Os07g0142600	Similar to Vegetative cell wall protein gp1.	AK062842	B6SY74	-
Os07g0142700	Transferase family protein.	AK365069	NP_001058873.1 (RefSeq)	Transferase
Os07g0142802	Hypothetical protein.	tplb0022f07 (Wheat FLcDNA)	longestORF	-
Os07g0142900	Aldo/keto reductase family protein.	AK060737 ,AK064848	NP_001149144.1 (RefSeq)	Aldo/keto reductase
Os07g0143000	Aldo/keto reductase family protein.	AK067910	E9JVD8	Aldo/keto reductase
Os07g0143200	Similar to Helix-loop-helix DNA-binding domain containing protein, expressed.	ab initio prediction	NP_001058876.2 (RefSeq)	Helix-loop-helix DNA-binding
Os07g0143401	Hypothetical gene.	CT836171	longestORF	-
Os07g0143500	Non-protein coding transcript.	AK064714	NONE	-
Os07g0143600	Hypothetical conserved gene.	AK241402	Q8H5E1	-
Os07g0143700	Similar to DEAD-Box RNA helicase-like protein.	AK068871	NP_001147777.1 (RefSeq)	DNA/RNA helicase, DEAD/DEAH box type, N-terminal
Os07g0143800	Hypothetical protein.	BT086170	longestORF	-
Os07g0143900	Conserved hypothetical protein.	AK064739	A2YI41	-
Os07g0144500	Similar to Patatin-like phospholipase family protein, expressed.	AK367891	Q2QMB1	Acyl transferase/acyl hydrolase/lysophospholipase
Os07g0144732	Conserved hypothetical protein.	CT836058	B9FVG7 "	-
Os07g0256200	RNA recognition motif, RNP-1 domain containing protein.	AK072904	A3BIC6	RNA recognition motif domain
Os07g0256300	Conserved hypothetical protein.	EU961484	B8AES2	-
Os07g0256700	Protein of unknown function DUF231, plant domain containing protein.	AK063314	A2X6R8	Domain of unknown function DUF231, plant
Os07g0256866	Hypothetical gene.	CT836065	longestORF	-
Os07g0257200	Similar to OsNramp1 (Integral membrane protein).	AK070788	Q8H4H5	Natural resistance-associated macrophage protein
Os07g0257400	Hypothetical protein.	BT086902	longestORF	-
Os07g0257600	Similar to H0315A08.1 protein.	FP099421	Q01I63	-
Os07g0257800	Conserved hypothetical protein.	FP099421	Q7XWB3	-
Os07g0258000	Domain of unknown function DUF1618 domain containing protein.	AK109782	B9FWG8	Domain of unknown function DUF1618
Os07g0258100	Conserved hypothetical protein.	AK064037	B9FWG9	-
Os07g0258400	OsNramp1 (Integral membrane protein).	AK121534	Q0D7E4	Natural resistance-associated macrophage protein
Os07g0258501	Hypothetical protein.	BT086902	longestORF	-
Os07g0260000	Pentatricopeptide repeat domain containing protein.	AK372160	Q8LIP4	Pentatricopeptide repeat
Os07g0260300	Thioredoxin fold domain containing protein.	AK066852	B6U038	Alkyl hydroperoxide reductase subunit C/ Thiol specific antioxidant
Os07g0260400	Similar to Phospholipase D nu-2 (Fragment).	AK070203	B8B515	C2 calcium-dependent membrane targeting
Os07g0261200	CCT (CO, CO-LIKE and TIMING OF CAB1) domain protein, Heading date	EU286800 (Genbank)	B2KYD3	CCT domain protein
Os07g0261732	Similar to 10A19I.4.	BT034152	Q9XHW2	-
Os07g0262200	Similar to Prohibitin.	AK071615 ,AK104133	NP_001105553.1 (RefSeq)	-
Os07g0262300	Conserved hypothetical protein.	tplb0007p15 (Wheat FLcDNA)	NP_001063061.1 (RefSeq)	-
Os07g0262600	Legume lectin, beta chain domain containing protein.	AK361888	GeneMark	Legume lectin, beta chain
Os07g0262700	Hypothetical protein.	tplb0031l02 (Wheat FLcDNA)	longestORF	-
Os07g0262800	Similar to Resistance protein candidate (Fragment).	AK120208	B9FWI2	Protein kinase, catalytic domain
Os07g0263400	Similar to oxidoreductase family protein.	ab initio prediction	XP_002888861.1 (RefSeq)	D-isomer specific 2-hydroxyacid dehydrogenase, catalytic domain
Os07g0423700	Peptidase A1 domain containing protein.	AK374841	NP_001042193.1 (RefSeq)	Peptidase A1
Os07g0424400	Similar to Cellulose synthase-7.	AK073561	B7F6W2	Zinc finger, RING-type
Os07g0425000	Biopterin transport-related protein BT1 family protein.	AK108531	NP_001151276.1 (RefSeq)	Biopterin transport-related protein BT1
Os07g0435850	Non-protein coding transcript.	EU949846	NONE	-
Os07g0435900	SET domain containing protein.	AK067172	XP_002315593.1 (RefSeq)	SET domain
Os07g0436100	Similar to predicted protein.	AK243522	NP_563716.3 (RefSeq)	Ankyrin repeat
Os07g0436350	Hypothetical conserved gene.	AK241013	Q7XHM4	-
Os07g0436600	Pentatricopeptide repeat domain containing protein.	AK369439	NP_001059506.1 (RefSeq)	Pentatricopeptide repeat
Os07g0436700	Conserved hypothetical protein.	AK119377	A2YKR4	-
Os07g0437000	Flavin monooxygenase-like enzyme , Auxin biosynthesis	AP005783_BAC80117_41	Q7XHL8	-
Os07g0437250	Hypothetical protein.	EU972174	longestORF	-
Os07g0437500	Aromatic L-amino acid decarboxylase (AADC), Serotonin biosynthesis	AK103253 (Genbank)	Q0D6V0	Pyridoxal phosphate-dependent decarboxylase
Os07g0458500	Hypothetical conserved gene.	AK250315	Q84YQ5	Transmembrane protein 6/97
Os07g0458700	Transmembrane protein 97, predicted domain containing protein.	AK112061	A2YKZ2	Transmembrane protein 6/97
Os07g0458800	Similar to Serine/threonine-protein kinase 19.	AK111873	B6TW02	Serine-threonine protein kinase 19
Os07g0459000	Hypothetical protein.	AK066176	longestORF	-
Os07g0459200	Similar to Possible kinase.	AK102099	NP_001148089.1 (RefSeq)	ABC-1
Os07g0459400	Regulator of chromosome condensation, RCC1 domain containing protein.	AK101767	B9FX29	Regulator of chromosome condensation, RCC1
Os07g0459500	F-box domain, Skp2-like domain containing protein.	AK373184	Q84YP6	F-box domain, Skp2-like
Os07g0462500	C2 calcium-dependent membrane targeting domain containing protein.	ab initio prediction	B9FX35	C2 calcium-dependent membrane targeting
Os07g0462700	Thioesterase superfamily domain containing protein.	AK073834 ,AK104968	NP_001152568.1 (RefSeq)	Thioesterase superfamily
Os07g0462800	O-methyltransferase, family 2 domain containing protein.	AK251187	A2YL13	O-methyltransferase, family 2
Os07g0463100	RNA polymerase subunit, RPB6/omega domain containing protein.	AK121807	B6TJG4	RNA polymerase, subunit omega/K/RPABC2
Os07g0463400	Similar to RES protein.	AK067630	F1DJW2	Zinc finger, RING-type
Os07g0463500	Similar to thioesterase family protein.	AK354998	NP_001152568.1 (RefSeq)	Thioesterase superfamily
Os07g0463600	O-methyltransferase, family 2 domain containing protein.	AK330822	GeneMark	O-methyltransferase, family 2
Os07g0463800	Similar to Bowman-Birk type bran trypsin inhibitor.	AK249661	Q0JR25	-
Os07g0464001	Hypothetical gene.	AK289077	Q9FP73	-
Os07g0464200	Similar to O-methyltransferase ZRP4.	EU961745	NP_001148593.1 (RefSeq)	O-methyltransferase, family 2
Os07g0467200	Similar to Clone ZZD536 mRNA sequence.	AK070358	NP_001147357.1 (RefSeq)	Iron sulphur-containing domain, CDGSH-type
Os07g0467500	WRC domain containing protein.	AK066400	A5HEG9	WRC
Os07g0467600	Similar to Plus agglutinin.	AK065880	Q69RL4	SNARE associated Golgi protein
Os07g0467800	Similar to Transposon protein.	AK072236	B4FUP6	Zinc finger, DHHC-type, palmitoyltransferase
Os07g0467850	Hypothetical protein.	tplb0036o16 (Wheat FLcDNA)	longestORF	-
Os07g0467900	Similar to DNA primase.	AK073933	NP_001154775.1 (RefSeq)	DNA primase, UL52/UL70 type, Herpesviridae
Os07g0468100	Similar to Glutathione S-transferase GST 19 (EC 2.5.1.18).	AK069604	Q9SES7	Glutathione S-transferase, N-terminal
Os08g0115800	No apical meristem (NAM) protein domain containing protein.	AK109860	XP_002443765.1 (RefSeq)	No apical meristem
Os08g0116100	Hypothetical protein.	FP093663	longestORF	-
Os08g0116400	Similar to CID11.	AK103042	NP_001150079.1 (RefSeq)	RNA recognition motif domain
Os08g0116500	Similar to 60S acidic ribosomal protein P1 (L12).	AK058240	Q5I7K5	Ribosomal protein 60S
Os08g0116566	Hypothetical conserved gene.	BT069214	B9FYS0	-
Os08g0116632	Hypothetical gene.	EU946409	longestORF	-
Os08g0116700	Conserved hypothetical protein.	AK065511	B8BAF9	-
Os08g0116750	Hypothetical gene.	AK288619	longestORF	-
Os09g0365900	L-ascorbate oxidase precursor (EC 1.10.3.3) (Ascorbase) (ASO).	AK243471	Q8S8Z5	Multicopper oxidase, type 1
Os09g0366101	Hypothetical protein.	tplb0051b23 (Wheat FLcDNA)	longestORF	-
Os09g0366500	Conserved hypothetical protein.	AK101278	B9G377	-
Os09g0366800	Hypothetical conserved gene.	ab initio prediction	Q69QF5	-
Os09g0367700	Similar to GST6 protein (EC 2.5.1.18).	AK103316 ,AK120037	NP_001105112.1 (RefSeq)	Glutathione S-transferase, N-terminal
Os09g0367900	Conserved hypothetical protein.	AK063310	Q6H5T9	-
Os09g0368101	Non-protein coding transcript.	EU943375	NONE	-
Os09g0368200	Similar to polyamine oxidase precursor.	AK367335	NP_001105106.1 (RefSeq)	Flavin amine oxidase
Os09g0368250	Hypothetical protein.	tplb0036e02 (Wheat FLcDNA)	longestORF	-
Os09g0368400	Hypothetical protein.	tplb0036e02 (Wheat FLcDNA)	longestORF	-
Os09g0368500	Similar to Polyamine oxidase precursor (EC 1.5.3.11).	AK069392	NP_001105106.1 (RefSeq)	Flavin amine oxidase
Os10g0555100	Similar to DNA chromosome 4, ESSA I CONTIG fragment NO. 6 (Glucosyltransferase like protein).	AK120349	NP_001148114.1 (RefSeq)	Glycosyl transferase, family 8
Os10g0555200	Resolvase, holliday junction-type, YqgF-like domain containing protein.	AK068935	NP_001149481.1 (RefSeq)	Resolvase, holliday junction-type, YqgF-like
Os10g0555300	Zinc finger, C2H2-like domain containing protein.	AK106924	B6UD41	Zinc finger, C2H2
Os10g0555600	Beta-expansin precursor.	AK101728 ,AK061498 ,AK104197 ,A	B7E6G9	Major pollen allergen Lol pI

Os10g0555651	Conserved hypothetical protein.	EU947703	NP_001176265.1 (RefSeq)	-
Os10g0555700	Beta-expansin.	AK061068 ,AK058895 ,AK104128	O24230	-
Os10g0555900	Similar to Beta-expansin.	AK105318	B7ER55	Major pollen allergen Lol pI
Os10g0556000	Hypothetical protein.	EU947703	longestORF	-
Os10g0556100	Similar to beta-expansin EXPB4.	AK101357	Q94LR4	Major pollen allergen Lol pI
Os10g0556200	Hypothetical conserved gene.	AK287925	B8BI66	Helix-loop-helix DNA-binding
Os10g0556500	Hypothetical conserved gene.	AK241512	NP_001176267.1 (RefSeq)	-
Os10g0556600	Similar to predicted protein.	EU971180	NP_171710.3 (RefSeq)	CCR4-Not complex, Not1 subunit, domain of unknown function DUF3819
Os10g0556700	Hypothetical conserved gene.	BT068414	Q10T37	-
Os10g0556801	Non-protein coding transcript.	CU406112	NONE"	-
Os10g0556900	Conserved hypothetical protein.	AK318544	NP_001176269.1 (RefSeq)	-
Os10g0557133	Similar to H0315A08.1 protein.	FP099421	Q01I63	Ribonuclease H1, N-terminal
Os10g0557366	Conserved hypothetical protein.	FP100733	A2XC28	-
Os10g0557600	Similar to GATA transcription factor 9.	CT837819	NP_001147669.1 (RefSeq)	Zinc finger, GATA-type
Os10g0557700	Conserved hypothetical protein.	AK241699	NP_001176270.1 (RefSeq)	-
Os10g0557750	Hypothetical genes.	ab initio prediction	NONE	-
Os10g0557800	Conserved hypothetical protein.	AK073483	B9G6Y4	-
Os10g0557900	Peptidase M10A and M12B, matrixin and adamalysin family protein.	CT835768	Q8LET7	Peptidase M10, metallopeptidase
Os10g0558100	Similar to Matrixin family protein, expressed.	AK362972	Q108Z3	Peptidase M10, metallopeptidase
Os10g0558125	Similar to Matrixin family protein, expressed.	AK335065	Q108Z3	Peptidase M10, metallopeptidase
Os10g0558200	2OG-Fe(II) oxygenase domain containing protein.	ab initio prediction	Q7XC87	Oxoglutarate/iron-dependent oxygenase
Os10g0558301	Hypothetical gene.	EU953848	longestORF	-
Os10g0558400	Similar to Oxidoreductase, 2OG-Fe oxygenase family protein.	BT085094	Q7XC87	Oxoglutarate/iron-dependent oxygenase
Os10g0558466	Hypothetical protein.	EU953848	longestORF	-
Os10g0558600	Pentatricopeptide repeat domain containing protein.	ab initio prediction	Q94LP5	Pentatricopeptide repeat
Os10g0558650	Non-protein coding transcript.	EU946768	NONE	-
Os10g0558700	Similar to Oxidoreductase, 2OG-Fe oxygenase family protein, expressed.	AK060854	Q336T3	-
Os10g0558725	Hypothetical protein.	EU953848	longestORF	-
Os10g0558750	Similar to Oxidoreductase, 2OG-Fe oxygenase family protein, expressed.	AK066303	Q336T1	Oxoglutarate/iron-dependent oxygenase
Os10g0558801	Non-protein coding transcript.	EU953848	NONE	-
Os10g0558850	Hypothetical protein.	tplb0043f10 (Wheat FLcDNA)	longestORF	-
Os10g0558900	Similar to Oxidoreductase, 2OG-Fe oxygenase family protein, expressed.	AK068525	Q336T1	Oxoglutarate/iron-dependent oxygenase
Os10g0559050	Non-protein coding transcript.	EU953848	NONE	-
Os10g0559200	Similar to Oxidoreductase, 2OG-Fe oxygenase family protein, expressed.	AK066405	Q336T1	-
Os10g0559300	Hypothetical protein.	AK063546	longestORF	-
Os10g0559400	Similar to cDNA clone:J023138C05, full insert sequence.	AK064149	Q7XC75	-
Os10g0559450	Conserved hypothetical protein.	AK241769	NP_001176272.1 (RefSeq)	-
Os10g0559500	2OG-Fe(II) oxygenase domain containing protein.	AK072706	Q8SB95	Oxoglutarate/iron-dependent oxygenase
Os10g0559550	Conserved hypothetical protein.	ab initio prediction	B9G6Z3	-
Os10g0559600	Agenet domain containing protein.	AK066219	Q108Y7	Agenet
Os10g0559650	Hypothetical protein.	tplb0029f23 (Wheat FLcDNA)	longestORF	-
Os10g0559700	Similar to Agenet domain containing protein, expressed.	AK070955	Q7XC74	-
Os10g0559800	Protein of unknown function DUF547 domain containing protein.	AK105615	B9G6Z5	Domain of unknown function DUF547
Os10g0559833	Hypothetical protein.	tplb0038j02 (Wheat FLcDNA)	longestORF	-
Os10g0559866	Hypothetical protein.	ab initio prediction	NONE	-
Os10g0559900	Ribosomal protein L18/L5 domain containing protein.	AK070091	NP_849617.1 (RefSeq)	Ribosomal protein L18/L5
Os10g0560000	Protein of unknown function DUF828, plant family protein.	AK066334	Q8SB99	Domain of unknown function DUF828
Os10g0560200	Protein of unknown function Cys-rich family protein.	AK069913	B6SGC5	Uncharacterised protein family Cys-rich
Os10g0560400	Similar to CONSTANS-like protein CO9 (Fragment).	AK109732	Q8SBA3	CCT domain
Os10g0560450	Similar to Autophagy-related protein 3.	AK241567	B9G6Z8	Autophagy-related protein 3, N-terminal
Os10g0560451	Hypothetical gene.	EU940960	longestORF	-
Os10g0560500	Conserved hypothetical protein.	AK100157	Q8SBA5	-
Os10g0560600	Non-protein coding transcript.	AK064207	NONE	-
Os10g0560700	Conserved hypothetical protein.	CT837759	NP_001065380.1 (RefSeq)	-
Os10g0560850	Hypothetical gene.	EU940765	longestORF	-
Os10g0560900	Pyridoxal phosphate-dependent transferase, major region, subdomain 1 domain containing protein.	AK071526	B6TFA9	Aminotransferase, class I/classII
Os10g0561000	Hypothetical conserved gene.	BT064708	Q9AUT7	-
Os10g0561100	Similar to Quinone oxidoreductase.	AK110882	Q10T48	Alcohol dehydrogenase superfamily, zinc-type
Os11g0153600	Similar to Signal recognition particle 54 kDa subunit (Fragment).	AK065028	O82532	Signal recognition particle, SRP54 subunit, GTPase
Os11g0153700	Similar to Signal recognition particle 54 kDa protein, chloroplast precursor (SRP54) (54 chloroplast protein) (54CP) (FFC).	AK058576	NP_196014.1 (RefSeq)	Signal recognition particle, SRP54 subunit, GTPase
Os11g0153800	Similar to Ribosomal protein S25 (40S ribosomal 25S subunit).	AK058630	A6N085	Ribosomal protein S25
Os11g0153851	Hypothetical genes.	ab initio prediction	NONE	-
Os11g0153900	Ribonuclease HII/HIII family protein.	AK103273	A2ZBK3	Ribonuclease HII/HIII
Os11g0154000	Non-protein coding transcript.	X06283	NONE	-
Os11g0154300	Protein of unknown function DUF584 family protein.	AK072969	Q01IJ8	Protein of unknown function DUF584
Os11g0154500	No apical meristem (NAM) protein domain containing protein.	AK106277	Q9SXQ0	No apical meristem
Os11g0154700	Similar to CBL-interacting protein kinase 14.	EU970334	C4P7S4	NAF domain
Os11g0154900	Similar to DNA-binding factor of bZIP class (Fragment).	AK287905	Q53QH0	Basic-leucine zipper
Os11g0155000	Dcp1-like decapping family protein.	AK064254	NP_001149166.1 (RefSeq)	Dcp1-like decapping
Os11g0155100	Similar to F-box domain containing protein.	AK108413	NP_001151212.1 (RefSeq)	F-box domain, cyclin-like
Os11g0155350	Hypothetical protein.	AK287665 (DDBJ, Secondary hit)	longestORF"	-
Os11g0155425	Hypothetical protein.	EU957609	longestORF	-
Os11g0155500	Similar to Amino acid permease family protein, expressed.	AK287730	Q53QH5	Amino acid/polyamine transporter I
Os11g0155600	Conserved hypothetical protein.	AK065863	Q53QH6	-
Os11g0155900	Histone H3.	U77296	XP_002299242.1 (RefSeq)	Histone H3
Os11g0155950	Hypothetical gene.	BT016934	longestORF	-
Os11g0155975	Non-protein coding transcript.	EU944780	NONE	-
Os11g0156000	Similar to AP2 domain containing protein RAP2.8 (Fragment).	AK070487	NP_001149371.1 (RefSeq)	Transcriptional factor B3
Os11g0156050	Non-protein coding transcript.	AK375277	NONE	-
Os11g0156100	Non-protein coding transcript.	AK240982	NONE	-
Os11g0156200	Peptidase S28 family protein.	AK100124	B6TWH6	Peptidase S28
Os11g0156300	Protein of unknown function DUF623, plant domain containing protein.	AK107140	A2ZBM6	Domain of unknown function DUF623
Os11g0156401	Protein of unknown function DUF623, plant domain containing protein.	AK243385	NP_001151444.1 (RefSeq)	Domain of unknown function DUF623
Os11g0156600	Similar to H0622F05.7 protein.	ab initio prediction	Q01LE7	TB2/DP1/HVA22-related protein
Os11g0156700	Conserved hypothetical protein.	AK108381	A2ZBN1	-
Os11g0175900	Similar to TPLATE%3B binding.	AK068683	NP_186827.2 (RefSeq)	-
Os11g0176000	Similar to nucleotide binding protein.	AK105568	NP_001147030.1 (RefSeq)	WD40 repeat
Os11g0176100	Nucleotide-binding, alpha-beta plait domain containing protein.	AK067921	B8BJE1	RNA recognition motif domain
Os11g0176200	Similar to HUA enhancer 2.	AK121851 ,AK122056	XP_002883745.1 (RefSeq)	Helicase, C-terminal
Os11g0176250	Non-protein coding transcript.	BT019066	NONE	-
Os11g0176300	GDP-fucose protein O-fucosyltransferase domain containing protein.	AK288044	B8BJE3	GDP-fucose protein O-fucosyltransferase
Os11g0177200	Conserved hypothetical protein.	AK240948	NP_001176385.1 (RefSeq)	-
Os11g0177301	Conserved hypothetical protein.	EU969600	B9G9P3	-
Os11g0177400	Similar to ATPase, coupled to transmembrane movement of substances.	AK120669	NP_001147989.1 (RefSeq)	ABC transporter-like
Os11g0177750	Hypothetical gene.	BT084685	longestORF	-

Pedigree-based genome re-sequencing reveals genetic variation patterns of elite backbone varieties during modern rice improvement

Xingfei Zheng, Lanzhi Li, Fan Liang, Changjun Tan, Shuzhu Tang, Sibin Yu, Ying Diao, Shuangcheng Li and Zhongli Hu

a

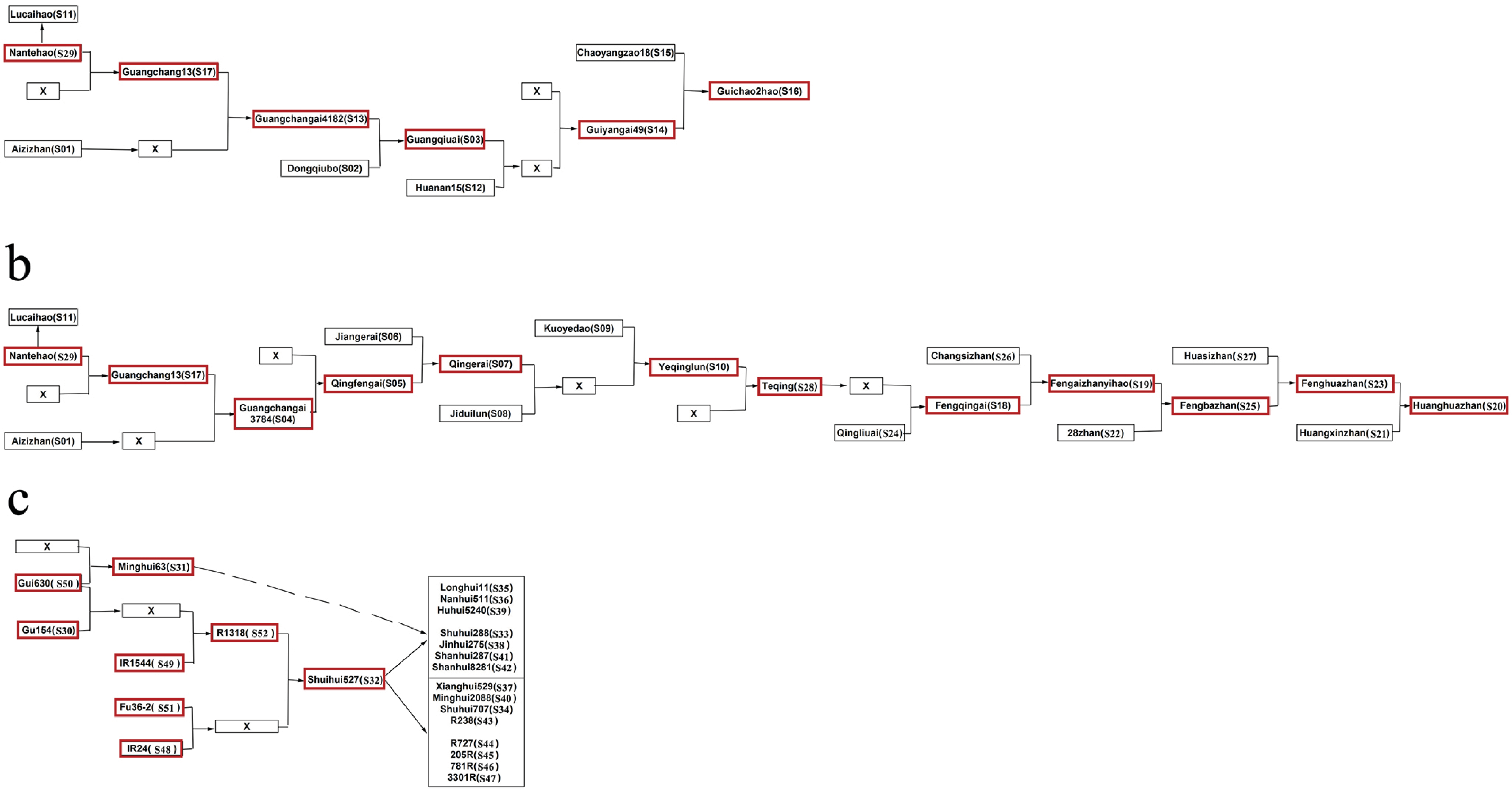

Supplementary Figure S1 Pedigree charts of 52 re-sequenced varieties. Red boxes represent core pedigree varieties (or immediate varietes) that were used to detect low diverisity genomic regions and 0-diveristiy genes; “X” represent missing varieties that were not re-sequenced. (a) Conventional rice: Guichao2hao (GC2H) pedigree; (b) Conventional rice : Huanghuazhan (HHZ) pedigree; (c) Restorer: Shuhui527 (SH527) pedigree.

Pedigree-based genome re-sequencing reveals genetic variation patterns of elite backbone varieties during modern rice improvement

Xingfei Zheng, Lanzhi Li, Fan Liang, Changjun Tan, Shuzhu Tang, Sibin Yu, Ying Diao, Shuangcheng Li, and Zhongli Hu

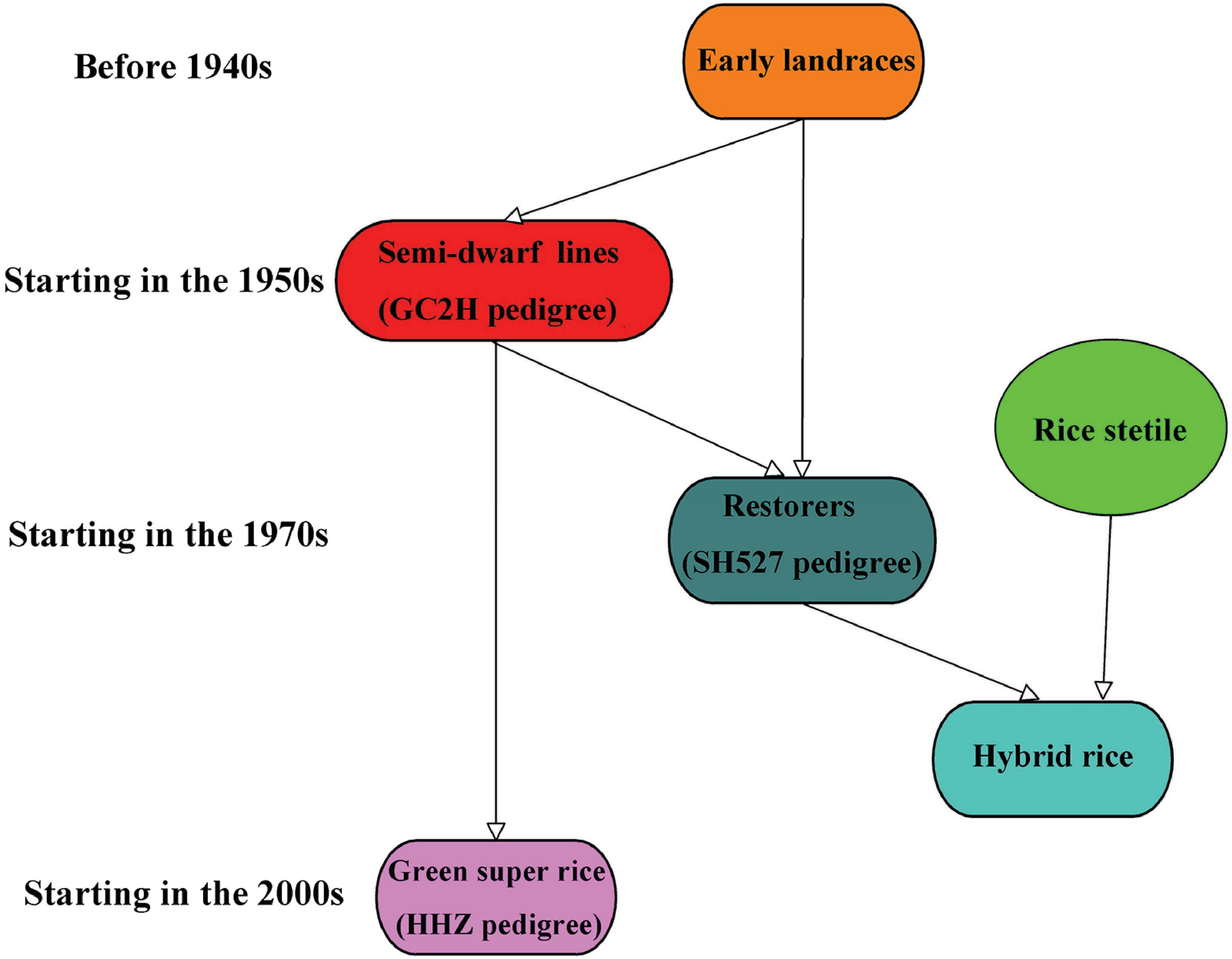

Supplementary Figure S2 The summary of rice breeding history. GC2H pedigree belongs to semi-dwarf rice from 1946 to 1976; SH527 pedigree belongs to hybrid rice from 1976 to 2011, and HHZ pedigree belongs to Green Super Rice (GSR) from 1946 to 2002.

Pedigree-based genome re-sequencing reveals genetic variation patterns of elite backbone varieties during modern rice improvement

Xingfei Zheng, Lanzhi Li, Fan Liang, Changjun Tan, Shuzhu Tang, Sibin Yu, Ying Diao
Shuangcheng Li, and Zhongli Hu

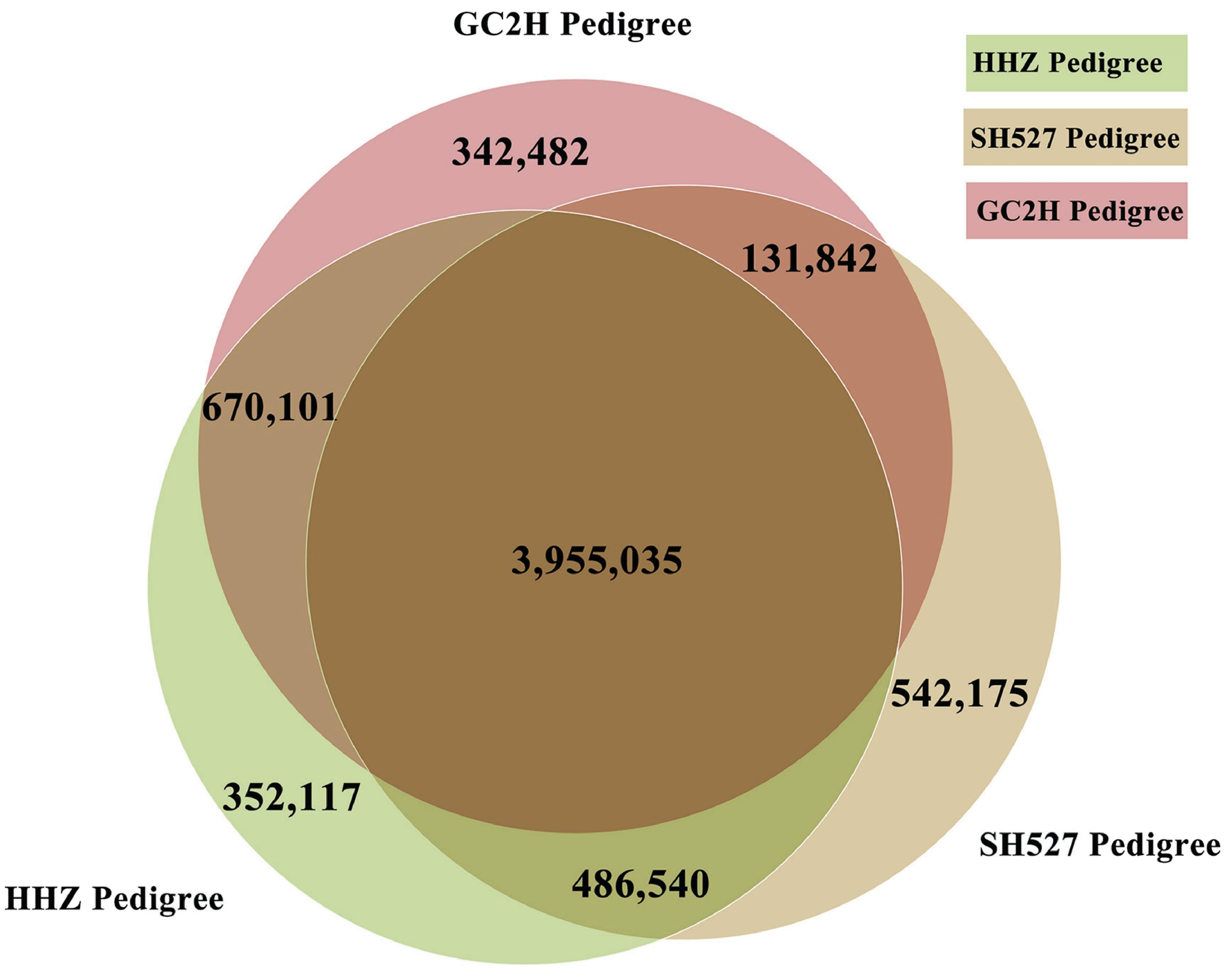

Supplementary Figure 3. Venn diagram of the private and common SNPs in three rice pedigrees

Pedigree-based genome re-sequencing reveals genetic variation patterns of elite backbone varieties during modern rice improvement

Xingfei Zheng, Lanzhi Li, Fan Liang, Changjun Tan, Shuzhu Tang, Sibin Yu, Ying Diao, Shuangcheng Li,and Zhongli Hu

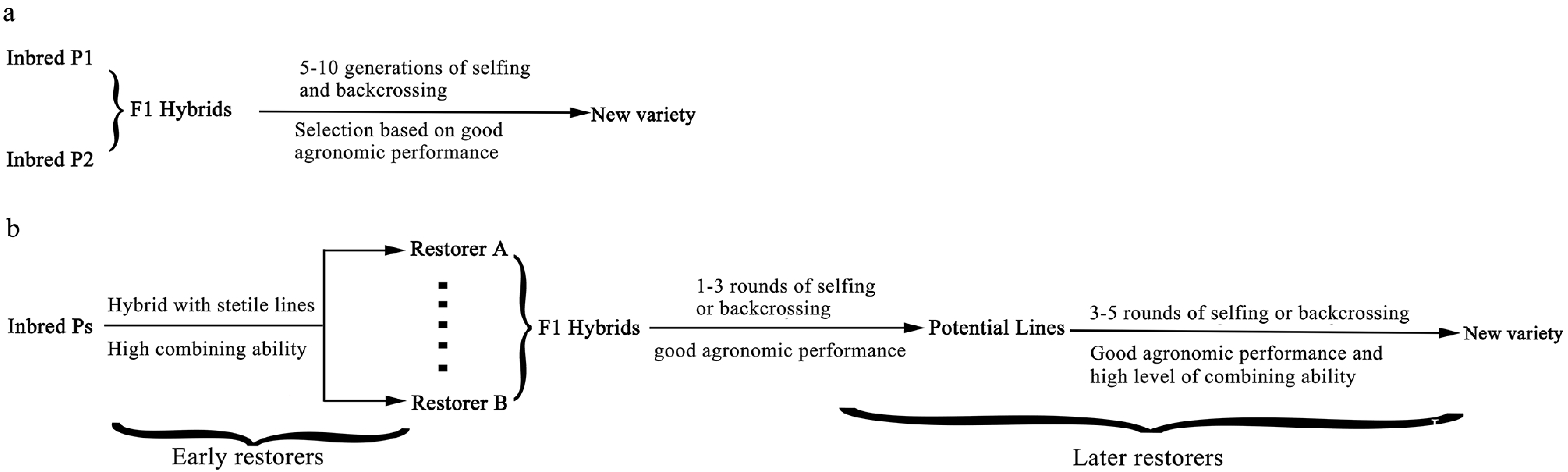

Supplementary Figure S4 Flow-charts of the rice breeding. (a) the Process of Conventional rice breeding; (b) the process of restorer breeding. Early restorer varieties were derectly selected from conventional rice inbred, and later restorers were created by crossing breeding method using restore lines as parents.

Pedigree-based genome re-sequencing reveals genetic variation patterns of elite backbone varieties during modern rice improvement

Xingfei Zheng, Lanzhi Li, Fan Liang, Changjun Tan, Shuzhu Tang, Sibin Yu, Ying Diao, Shuangcheng Li, and Zhongli Hu

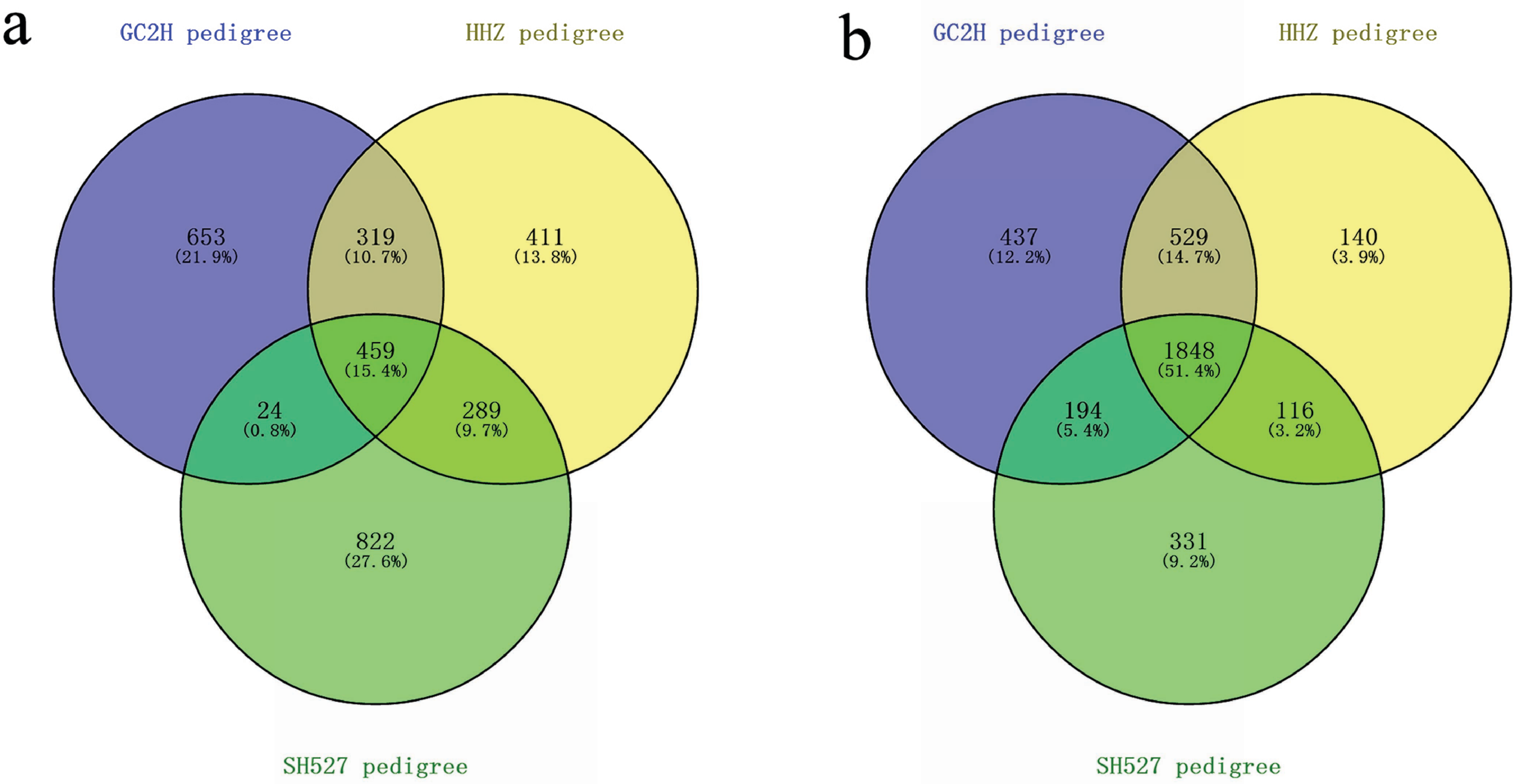

Supplementary Figure S5 Venn diagrams of genes identified from three pedigrees. (a) genes located on low diversity regions from three breeding pedigree; (b) Zero-diversity genes in rice pedigrees.
